# Correlations Between Prokaryotic Microbes and Stress-Resistant Algae in Different Corals Subjected to Environmental Stress in Hong Kong

**DOI:** 10.3389/fmicb.2020.00686

**Published:** 2020-04-23

**Authors:** Haoya Tong, Lin Cai, Guowei Zhou, Weipeng Zhang, Hui Huang, Pei-Yuan Qian

**Affiliations:** ^1^Department of Ocean Science, Division of Life Science and Hong Kong Branch of the Southern Marine Science and Engineering Guangdong Laboratory (Guangzhou), The Hong Kong University of Science and Technology, Hong Kong, China; ^2^Key Laboratory of Tropical Marine Bio-resources and Ecology, South China Sea Institute of Oceanology, Chinese Academy of Sciences, Guangzhou, China; ^3^CAS-HKUST Sanya Joint Laboratory of Marine Science Research, Hainan Key Laboratory of Tropical Marine Biotechnology, Tropical Marine Biological Research Station in Hainan, Chinese Academy of Sciences, Sanya, China

**Keywords:** climate change, symbiosis, South China Sea, reef-building coral, holobiont

## Abstract

Coral reefs are extremely vulnerable to global climate change, as evidenced by increasing bleaching events. Previous studies suggest that both algal and microbial partners benefit coral hosts, but the nature of interactions between Symbiodiniaceae and prokaryotic microbes and their effects on coral hosts remains unclear. In the present study, we examined correlations between Symbiodiniaceae and prokaryotic microbes in *Montipora* spp. and *Porites lutea* sampled from two sites in Hong Kong with contrasting environmental conditions in March and October 2014. The results showed that the prokaryotic microbial communities had adaptable structures in both *Montipora* spp. and *P. lutea*, and environmental conditions had greater effects on the algal/microbial communities in *Montipora* spp. than in *P. lutea*. Further network analysis revealed a greater number of prokaryotic microbes were significantly correlated with potentially stress-resistant Symbiodiniaceae in *P. lutea* than in *Montipora* spp. Stress-resistant Symbiodiniaceae played more important roles in the community and in the algal–microbial correlations in *P. lutea* than in *Montipora* spp. Since *P. lutea* is faring better in Hong Kong as the seawater temperature gradually increases, the results suggest that the correlations between stress-resistant algae and prokaryotic microbes could provide a compensation mechanism allowing coral hosts to adapt to higher temperatures, particularly as the prokaryotic microbes correlated with Symbiodiniaceae provide the ecological functions of photosynthesis and nitrogen fixation.

## Introduction

Coral reef ecosystems are threatened by various anthropogenic stressors, such as elevated sea surface temperature (SST), ocean acidification, eutrophication, and sedimentation (Hoegh-Guldberg et al., 2007; Bégin et al., 2016; Duprey et al., 2016). Thermal stress is a major threat to coral reefs and causes coral bleaching and shrinking reef areas. Anomalously high temperatures can trigger coral bleaching even if it is just 1°C above the summer mean SST. During El Niño in 2015–2016, coral bleaching was rampant globally (Ampou et al., 2017; Bainbridge, 2017). Coral thermal susceptibility and adaptation have drawn substantial attention to the importance of coral reef conservation.

Coral-associated algae and microbes and their extensive interactions are significant for reef health and function (Muscatine et al., 1984). Dinoflagellates living in corals, known as Symbiodiniaceae, provide corals with energy through algal photosynthesis. Loss of Symbiodiniaceae due to thermal stress can lead to coral death if symbiosis cannot be reestablished. Nine genera of Symbiodiniaceae with varying levels of photosynthetic efficiency and heat tolerance have been identified thus far (Baker, 2003; Pochon and Gates, 2010). Corals can acquire a greater number of beneficial Symbiodiniaceae genera through horizontal and/or vertical transmission under specific environmental conditions (Lajeunesse et al., 2004; Padilla-Gamino et al., 2012), but which coral species will perform better under future climate change conditions remains controversial.

Prokaryotic microbial communities in corals can change quickly in structure and are highly specific to the host species. They perform a wide range of metabolic functions, such as carbon and nitrogen fixation (Morrow et al., 2015); they can also protect coral hosts from potential pathogens through antibiotic production and can regulate coral surface structure (Kim, 2006). The structure of prokaryotic microbial communities in corals can change in response to environmental stressors, but it is debatable how the altered prokaryotic microbial communities affect coral reefs (Pantos et al., 2015). In some cases, disrupted prokaryotic microbial communities can lead to a bloom of pathogens and viruses, thus inducing pathogenicity (Thurber et al., 2008, 2009). In other cases, environmental stressors can increase the population of potentially beneficial prokaryotic microbes, thus improving the functional capacity of the host (Bourne et al., 2008). These findings suggest that the coral microbiome can establish new equilibria under gradually changing environmental conditions to benefit the health of the host. However, rapid changes in environmental stressors can tip the balance of the coral microbiome and impair reef function. In other holobionts, such as green algae, algae and microbes can be affected not only by environmental factors but also by each other (Lachnit et al., 2011; Kim et al., 2014). In corals, some prokaryotic microbes have been shown to be associated with algae and to work with Symbiodiniaceae in organomineralization processes to enhance the adaptation of coral reefs to ocean acidification (Frommlet et al., 2015). We anticipate that correlations between algae and prokaryotic microbes can reflect their potential interactions, which are crucial to coral microbiome alteration and balance and can ultimately help improve coral adaptation.

Located between 22°10′N and 22°30′N, Hong Kong is home to 84 scleractinian coral species despite its large fluctuations in temperature, salinity and coastline nutrient loading. Some sites in Hong Kong show high coral diversity and coverage (Yeung et al., 2014). Thus, Hong Kong corals are considered “tough” species with a large capacity to adapt to future climate change. Two dominant coral genera, *Montipora* and *Porites*, were selected for the study due to their different fates in Hong Kong in recent years (Duprey et al., 2016). Specifically, *Montipora* has declined rapidly in recent years in Hong Kong, while *Porites* has fared much better. We suspect that their different fates are attributed to different algal–microbial correlations. In the present study, Crescent Bay and Lamma Island were selected as sampling sites because they are representative coral sites in Hong Kong. Crescent Bay is located in the northeast of Hong Kong. The cove is hardly influenced by the Pearl River discharge and has a high coral cover. In contrast, Lamma Island is located in the southwest of Hong Kong. The area is greatly affected by the Pearl River discharge and has a low coral cover (Goodkin et al., 2011).

To assess the nature of the Symbiodiniaceae/prokaryotic microbial community in the two coral genera and how they contribute to coral adaptation, we collected 48 samples of coral tissue from the two coral genera, *Montipora* spp. and *Porites lutea*, from each site in March (wintertime) and October (summertime) 2014 and extracted the genomic DNA of the Symbiodiniaceae/prokaryotic microbial community from each sample. For analysis, we first characterized the Symbiodiniaceae and prokaryotic microbial communities and then assessed the environmental impacts on the Symbiodiniaceae and prokaryotic microbial communities. Furthermore, the algal–microbial correlations in the corals were calculated and visualized through network analysis (Bokulich et al., 2014; Woodhouse et al., 2016). Finally, we conducted PICRUSt functional prediction (Langille et al., 2013) to interpret the altered algal–microbial correlations in these corals under different environmental conditions.

## Materials and Methods

### Coral Sample Collection and Field Environmental Data

In total, 48 coral samples of *Montipora* spp. (*n* = 24 colonies) and *P. lutea* (*n* = 24 colonies) and 8 seawater samples were collected from the two sampling sites in Hong Kong in March and October 2014 (Table 1 and Supplementary Figure S1). Briefly, one small piece of coral (∼1 cm × 1 cm) was taken from each healthy colony using a hammer and chisel and wrapped underwater in a tagged bag. The coral fragments were washed with seawater filtered through 0.22-μm polycarbonate membranes as soon as they were brought to the surface and immediately fixed in 70% ethanol. The coral samples were then stored in a cool box with dry ice and quickly transported to the laboratory. Seawater samples for use as controls were also taken from the coral collection sites during each sampling. These seawater samples were immediately filtered through 0.22-μm polycarbonate membranes to collect free-living Symbiodiniaceae and prokaryotic microbes, and the membranes were then fixed in 50% ethanol. All fixed samples were stored at −30°C until DNA extraction.

**TABLE 1:** Sampling information.

**Sites**	**Coordinates**	**Sampling month**	**Samples**	**Species**
Crescent Bay (CB)	E114.314°, N22.531°	March-14	CB3_MO1-6, CB3_PO1-6, CB3_SW1-2	*Montipora peltiformis*,
		October-14	CB10_MO1-6, CB10_PO1-6, CB10_SW1-2	*Porites lutea*
Lamma Island (LI)	E114.135°, N22.187°	March-14	LI3_MO1-6, LI3_PO1-6, LI3_SW1-2	*Montipora venosa*
		October-14	LI10_MO1-6, LI10_PO1-6, LI10_SW1-2	*Porites lutea*

Environmental data from each field site were acquired from the Hong Kong Environmental Protection Department (HKEPD). For each sampling site, five nearby monitoring stations were selected as references, and data from these monitoring stations were collected, averaged and then used for the further analysis of the environmental impacts on Symbiodiniaceae/prokaryotic microbial communities in corals Supplementary Figure S1 and Supplementary Tables S1–S3).

### DNA Extraction, PCR, and High-Throughput Amplicon Sequencing

A small fragment (∼0.5 cm × 0.5 cm) was removed from each fixed coral sample, rinsed with 1x PBSE (137 mM NaCl, 2.7 mM KCl, 4.3 mM Na_2_HPO_4_⋅7H_2_O, 1.4 mM KH_2_PO_4_ and 10 mM EDTA) and crushed with a mortar and a pestle in 1x PBSE. The resulting coral homogenates were centrifuged at 12,000 × *g*, and the pellets were retained for DNA extraction. DNA was extracted from the pellets with the FastDNA^®^ Spin Kit for Soil (MP Biomedicals, France) following the manufacturer’s instructions. The extracted DNA samples were used as PCR templates after quality and purity examinations. To amplify the Symbiodiniaceae ITS2 region of the rRNA gene and the prokaryotic microbial V3 and V4 regions of the 16S rRNA gene, two primer sets (F: 5′-GAATTGCAGAACTCCGTG-3′; R: 5′-GGATCCATATGCTTAAGTTCAGCGGGT-3′ for ITS2. 341F: 5′-CCTAYGGGRBGCASCAG-3′; 802R: 5′-TACNVGGGTATCTAATCC-3′ for 16S) were selected for PCR amplification (Lajeunesse and Trench, 2000; Behrendt et al., 2011; Cai et al., 2013). Six-nucleotide unique barcodes were modified at the forward primer’s 5′ terminus for multiplex sequencing (Supplementary Table S4). The PCRs were conducted according to the following program: 5 min at 94°C, followed by 35 cycles of 30 s at 94°C, 30 s at 51°C, 30 s at 72°C, and another 5 min at 72°C for Symbiodiniaceae; 5 min at 94°C, followed by 30 cycles of 30 s at 94°C, 30 s at 50°C, 1 min at 72°C, and another 5 min at 72°C for prokaryotic microbes. Each amplification was performed with ∼50 ng of the DNA template, primers each at a concentration of 200 nM, 25 μL of 2 × Premix Ex Taq solution (TaKaRa, China), and ddH_2_O in a total volume of 50 μL. Three independent reactions were conducted for each sample to minimize potential PCR bias. The PCR products were purified using the PureLink^®^ PCR Purification Kit (Invitrogen, United States). The purified products were quantified by a Thermo NanoDrop 2000 UV-Vis Spectrophotometer and mixed at equal mass for amplicon sequencing. The quantified DNA samples were sequenced by Novogene (Beijing, China) on an Illumina MiSeq system based on the paired-end 300 bp × 2 strategy. The sequencing datasets are available from NCBI’s Sequence Read Archive. The accession numbers are SRP066283 for the ITS2 dataset and SRR2917918 under SRP066229 for the 16S dataset.

### Symbiodiniaceae and Prokaryotic Microbial Sequence Analysis

Adaptors, low-quality reads, reads containing poly-N and short reads were removed by the sequencing company. The PEAR (paired-end read merger) tool was used to merge overlapping paired-end reads and obtain full-length ITS2 and 16S fragments (Zhang et al., 2014). ITS2 and 16S tags were demultiplexed and identified by unique barcodes into all samples on the QIIME platform (Caporaso et al., 2010).

Since the previously published ITS2 database was found to contain duplicate sequences (Arif et al., 2014), the database was uploaded to the CD-HIT Suite website (Huang et al., 2010) to remove duplicates and merge the annotations. The sequences generated were used to build a non-redundant ITS2 database. BLASTN (Altschul et al., 1990) was then applied to align all the ITS2 sequences with the newly constructed ITS2 database using an e-value of 1e-50, and the outputs were filtered in Microsoft Excel at 97% similarity to identify Symbiodiniaceae annotations. Analyzed Symbiodiniaceae types were established as a phylogenetic tree by RAxML under the GTRGAMMA model (Stamatakis, 2014) to perform the phylogenetic relationships. A sequence similarity of 97% for ITS2 rDNA is reliable for Symbiodiniaceae identification (Thomas et al., 2014). The filtered results were kept for downstream analysis.

QIIME’s ChimeraSlayer command was used to detect and remove potential PCR chimeras in 16S tags (Haas et al., 2011). The clean 16S tags were retained for downstream analysis after strict filtration. Operational taxonomic units (OTUs) were clustered and annotated at 97% similarity using the QIIME pipeline. The structure of the prokaryotic microbial community was analyzed at the phylum to genus levels. The results of the genus-level analysis were kept for downstream use. Analysis code is available in Supplementary Data Sheet S2.

### Statistical Analysis

To profile the Symbiodiniaceae and prokaryotic microbial communities in the samples, a heat map was created in OriginPro 2019 (OriginLab, United States). To validate differences in Symbiodiniaceae/prokaryotic microbial community structures between sampling sites, seasons and hosts, a non-parametric multivariate analysis of similarity (ANOSIM) was conducted using PRIMER 7 software (Clarke and Gorley, 2015). To study the relationships between environmental factors and the structure of Symbiodiniaceae/prokaryotic microbial communities in different samples, stepwise distance-based redundancy analysis (dbRDA) using Bray–Curtis distance metrics was performed in Canoco 5 (Šmilauer and Lepš, 2014). Sequences belonging to each key prokaryotic microbe were separated based on OTU and representative sequence information. To predict the functions of prokaryotic microbial communities in different samples and key prokaryotic microbial genera, PICRUSt was applied to sequences for samples and key prokaryotic microbial genera based on the GreenGenes database (v13_5) (Langille et al., 2013). The functional profiles were assessed for variance using OriginPro 2015. The associations between Symbiodiniaceae types, prokaryotic microbial genera and environmental factors were assessed using Spearman’s correlation coefficient (*r*) in OriginPro 2019 as well as CoNet, combining Pearson’s correlation, Spearman’s correlation and Kendall rank correlation in Cytoscape 3.7.2. These associations were visualized in Cytoscape 3.7.2. Node, edge and topological metrics, including betweenness centrality and density, were measured by the Network Analysis plug-in (Assenov et al., 2008).

## Results

### Sequence Information

High-quality ITS2 and 16S sequences were retained for further analysis. After removal of the fusion primers, the median lengths of the sequences were ∼330 bp and ∼450 bp for Symbiodiniaceae and prokaryotic microbes, respectively. In total, six replicates were sampled twice for each coral species from both sites; thus, there were 56 samples, including six *Montipora* spp. and six *P. lutea* samples and two seawater samples from March (representing winter) and October (representing summer) 2014, from Crescent Bay and Lamma Island. The samples were labeled as follows: CB3_MO1-6 (CB3_MO6 was a technical replicate of CB3_MO5), CB10_MO1-6, LI3_MO1-6, LI10_MO1-6, CB3_PO1-6, CB10_PO1-6, LI3_PO1-6, LI10_PO1-6, CB3_SW1-2, CB10_SW1-2, LI3_SW1-2, and LI10_SW1-2. CB and LI represent Crescent Bay and Lamma Island; 3 and 10 represent the sampling months, March and October; and MO, PO, and SW represent *Montipora* spp., *P. lutea*, and seawater samples, respectively.

### Symbiodiniaceae Community Structure

**FIGURE 1:**
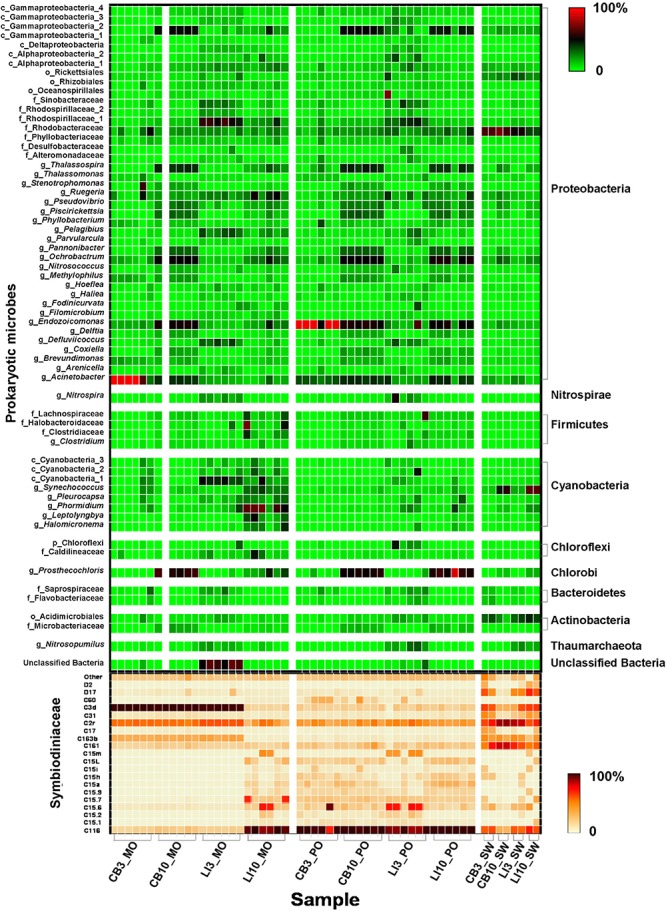
Microbial community structure in different samples. Relative abundance of key Symbiodiniaceae types and prokaryotic microbes in *Montipora* spp., *Porites lutea*, and seawater samples in Hong Kong. The color scale represents the percentage of a microbe in each sample.

**FIGURE 2:**
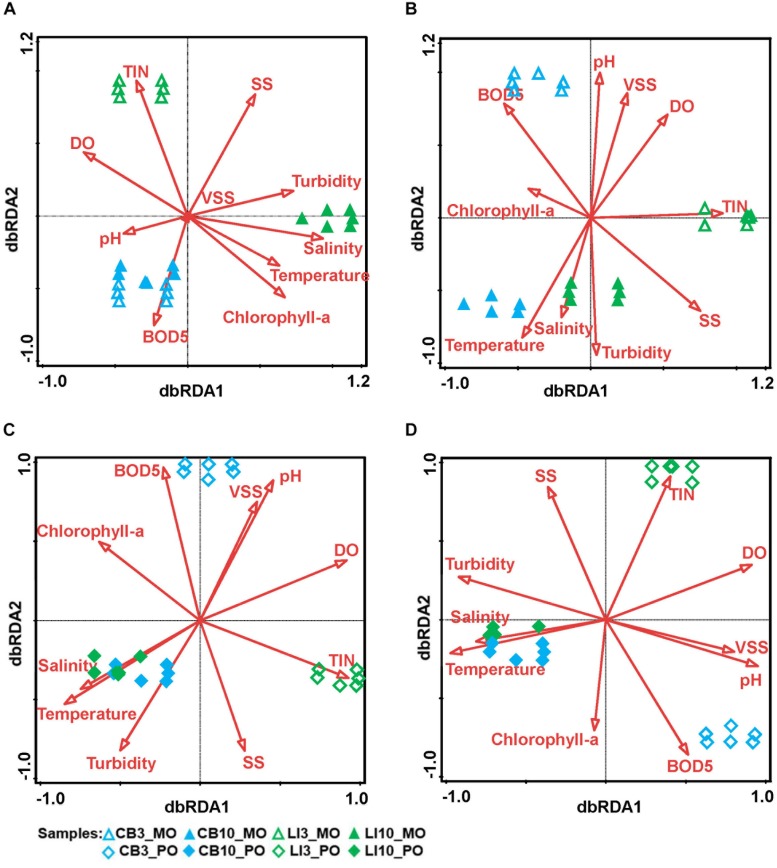
Relationships between environmental conditions and algal/microbial communities from different samples. dbRDA indicates the relationships between environmental factors and microbial communities from different samples. **(A)** dbRDA of Symbiodiniaceae communities in *Montipora* spp. The first axis explains 93.95% of the total variation and 99.77% of the fitted variation, while the second axis explains 0.19% of the total variation and 0.19% of the fitted variation. **(B)** dbRDA of prokaryotic microbial communities in *Montipora* spp. The first axis explains 37.22% of the total variation and 52.47% of the fitted variation, while the second axis explains 28.60% of the total variation and 40.32% of the fitted variation. **(C)** dbRDA of Symbiodiniaceae communities in *Porites lutea*. The first axis explains 27.94% of the total variation and 87.60% of the fitted variation, while the second axis explains 3.41% of the total variation and 10.69% of the fitted variation. **(D)** dbRDA of prokaryotic microbial communities in *Porites lutea*. The first axis explains 38.30% of the total variation and 58.26% of the fitted variation, while the second explains 25.98% of the total variation and 39.51% of the fitted variation.

In total, 159 Symbiodiniaceae types from five Symbiodiniaceae genera (*Symbiodinium*, *Breviolum*, *Cladocopium*, *Durusdinium*, and *Fugacium*) were detected in the samples. Twenty key Symbiodiniaceae types, including 2 types of *Durusdinium* and 18 types of *Cladocopium*, covering more than 95% of the sequences in each sample, were kept for further analysis based on their average relative abundance (Figure 1 and Supplementary Figure S2). Specific epithets of most Symbiodiniaceae have not been identified since systematic revision; thus, subclade names were kept for clarification in the present study (Lajeunesse et al., 2018). Some redundant sequences were given multiple ITS2 type designations in the previous database. These include C17 and C3d in the present study, which are identical to C17.2 and C21/3k, respectively. The phylogenetic analysis showed larger genetic distances between *Cladocopium* and *Durusdinium*, and several types of *Cladocopium* in the present study exhibited high similarity (Supplementary Figure S3). According to the heat map and Shannon’s diversity index, *P. lutea* maintained a more diverse Symbiodiniaceae community than did *Montipora* spp. at each site (Supplementary Table S5). Symbiodiniaceae in *Montipora* spp. were dominated by C3d, C2r, C163b, and C116, with few subdominant Symbiodiniaceae types, while *P. lutea* was mostly dominated by C116 and C2r and several subdominant Symbiodiniaceae types. The Symbiodiniaceae community in the LI10_MO samples was dominated by C116, C15.6, and C15.7, which were different from those in the other *Montipora* samples, possibly due to the low water quality near Lamma Island during summertime. The seawater samples had several dominant Symbiodiniaceae types, and all of the Symbiodiniaceae types found in the corals could be found in the surrounding seawater. ANOSIM indicated a spatial and temporal separation of *Montipora* spp. samples (Table 2): the Symbiodiniaceae communities in *Montipora* spp. differed significantly between sites/seasons, while they were similar between *Montipora* spp. and the surrounding seawater. In contrast, the Symbiodiniaceae communities in *P. lutea* from different sites/seasons were similar but significantly different from those in seawater. dbRDA identified temperature, salinity, turbidity, chlorophyll-a, pH, and DO as the major environmental factors affecting the Symbiodiniaceae community structures in *Montipora* spp., and the 10 measured environmental factors explained 94.17% of the observed variation (Figure 2A). For *P. lutea*, salinity, temperature, turbidity, chlorophyll-a, pH, DO, and TIN were the major environmental factors affecting the Symbiodiniaceae community structures, but the 10 measured environmental factors explained only 31.89% of the total variation (Figure 2C). dbRDA clarified that the environment similarly affected the Symbiodiniaceae community structures in *Montipora* spp. and *P. lutea*. However, the measured factors explained only 20.50% of the total variation (Supplementary Figure S5A) when combining all samples.

**TABLE 2:** ANOSIM of Symbiodiniaceae and prokaryotic microbial communities from different sites, months, and hosts.

	**Symbiodiniaceae**		**Prokaryotic microbes**	
	***R***	**Sig.**	***R***	**Sig.**
**Site**				
MO-MO	0.8640	0.0001	0.7990	0.0001
PO-PO	0.2840	0.0060	0.6030	0.0001
**Month**				
MO-MO	0.7110	0.0001	0.8980	0.0001
PO-PO	0.2930	0.0070	0.9840	0.0001
**Host**				
MO-SW	0.3020	0.0140	0.0400	0.2810
PO-SW	0.9950	0.0001	0.1220	0.0540
MO-PO	0.5840	0.0001	0.0990	0.0110

### Prokaryotic Microbial Community Compositions and Functions

Prokaryotic microbial community analysis identified OTUs at 97% similarity and annotated 1095 prokaryotic microbial genera in total. Sixty-three prokaryotic microbial genera with relative abundances greater than or equal to 0.01% in at least three samples were regarded as key prokaryotic microbial genera and kept for further analysis. Annotation of the OTUs revealed that *Montipora peltiformis* in Crescent Bay was dominated by Proteobacteria in March (winter) and by Chlorobi and Proteobacteria in October (summer). Additionally, *Montipora venosa* near Lamma Island was dominated by Proteobacteria and Cyanobacteria in March (winter) and by Proteobacteria, Cyanobacteria, and Chlorobi in October (summer). *P. lutea* at both sites was dominated by Proteobacteria in March (winter) and by Proteobacteria and Chlorobi in October (summer) (Figure 1 and Supplementary Figure S4). ANOSIM revealed a significant spatial and temporal variation in the prokaryotic microbial communities in both coral species: prokaryotic microbial communities differed significantly between sites and seasons; in the same coral species, the variation in prokaryotic microbial communities between seasons was greater than that between sites. Prokaryotic microbial communities in both coral species were similar to those from the surrounding seawater, and the prokaryotic microbial communities in *Montipora* spp. showed greater similarity to those in the surrounding seawater. dbRDA identified TIN, turbidity, pH, and temperature as the major environmental factors affecting the prokaryotic microbial community structure in both coral species, and the 10 environmental factors considered explained 70.94% and 65.74% of the observed variations in *Montipora* spp. and *P. lutea*, respectively (Figures 2B,D). dbRDA combining *Montipora* spp. and *P. lutea* suggested that the environment affected prokaryotic microbial communities similarly in the two coral genera, and the measured factors explained 46.38% of the total variations (Supplementary Figure S5B).

**FIGURE 3:**
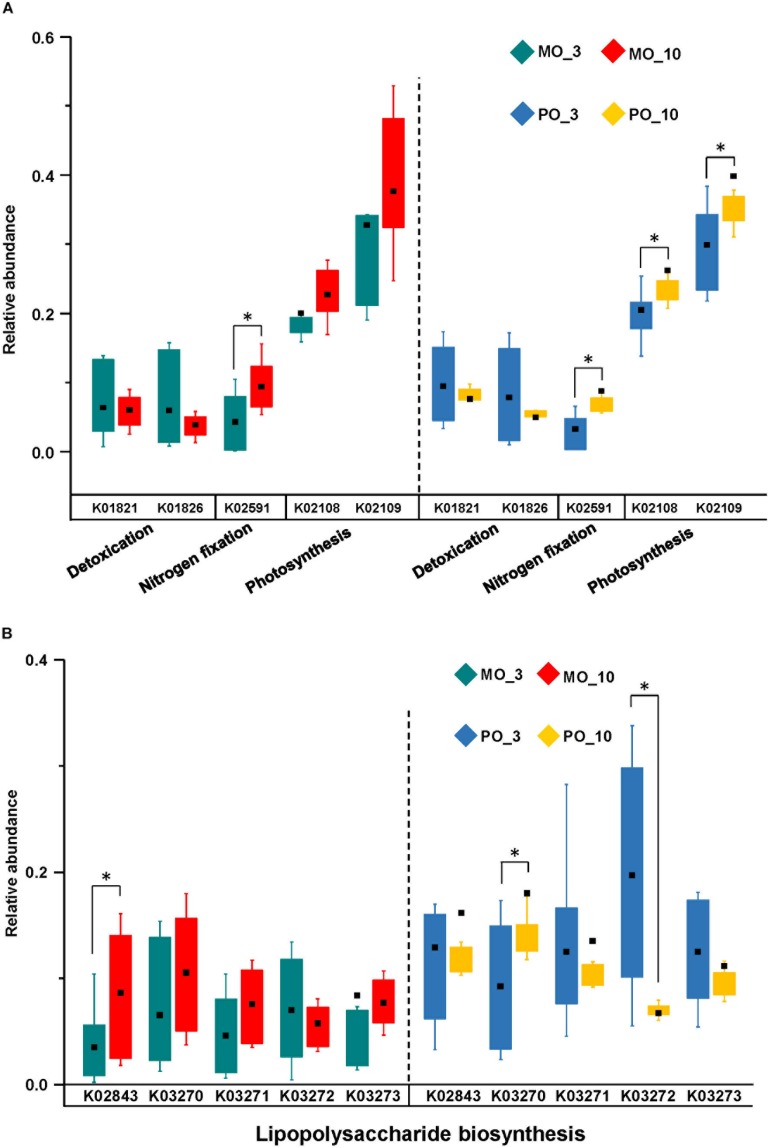
Functional predictions by PICRUSt of prokaryotic microbial communities from different corals in different months. In total, there were four main functions: detoxification, nitrogen fixation, photosynthesis **(A)**, and lipopolysaccharide biosynthesis **(B)**. The asterisk indicates significant difference at the 0.1 level.

PICRUSt predicted detoxification, nitrogen fixation, photosynthesis, and lipopolysaccharide biosynthesis to be the major prokaryotic microbial community functions in corals based on the average abundance of predicted function (≥1%) as well as whether the function could potentially affect coral adaptation (Figure 3 and Supplementary Table S6). Nitrogen fixation increased significantly in both *Montipora* spp. and *P. lutea* from March (winter) to October (summer), while photosynthesis increased significantly in only *P. lutea*. This result is consistent with the variations in the prokaryotic microbial community in each coral genus. In *Montipora* spp., Cyanobacteria differed between winter and summer. In *P. lutea*, although the abundance of Cyanobacteria did not diverge much between the two time points, the populations of other prokaryotic microbes predicted to maintain photosynthesis increased significantly from winter to summer (e.g., g_*Prosthecochloris*).

### Covariance Analysis of Symbiodiniaceae and Prokaryotic Microbes

**FIGURE 4:**
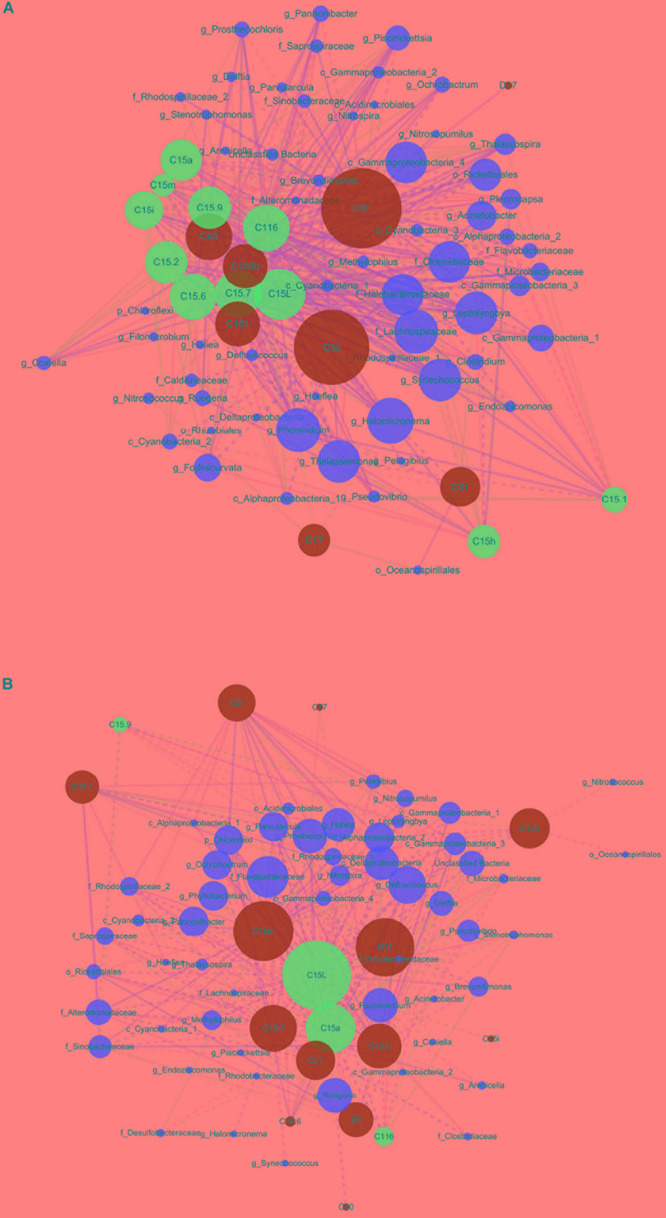
Organic correlation network visualizing significant pairwise correlations between Symbiodiniaceae and prokaryotic microbes in *Montipora* spp. **(A)** and *Porites lutea* **(B)** using Spearman’s correlation analysis. Green, blue, and red nodes represent non-stress-resistant Symbiodiniaceae, and potentially stress-resistant Symbiodiniaceae and microbes, respectively. Node size is reflective of the betweenness centrality of the variable. Line types (solid = positive and dashed = negative) are indicative of Spearman’s correlation coefficient. Line colors are indicative of Spearman’s correlation significance, a brighter color represents a more significant correlation.

The selected key Symbiodiniaceae types and prokaryotic microbial genera were used to form a single interconnected network to show their correlations. Spearman’s correlation analysis showed that of the 1,260 correlations tested, only 365 among 78 variables and 261 among 71 variables were considered significant (*p* ≤ 0.05) for *Montipora* spp. and *P. lutea*, respectively. Organic correlation visualization of the network revealed patterns of the algal–microbial correlations in *Montipora* spp. and *P. lutea* (Figure 4). Network density describes the percentage of the actual connections in a network out of potential connections of the network. The network of *P. lutea* exhibited lower network density than that of *Montipora* spp. (Supplementary Table S7). Betweenness centrality measures the degree to which certain variables are connected with each other, and a high value represents large control over the network. In both networks, most variables with high values of betweenness centrality belonged to Symbiodiniaceae. The network displays the correlations between the Symbiodiniaceae community and the prokaryotic microbial community, and more prokaryotic microbial variables were included than Symbiodiniaceae variables. Thus, the betweenness centrality of Symbiodiniaceae would be higher than that of prokaryotic microbes due to quantity effects. The main functions of key prokaryotic microbes were predicted to be photosynthesis, nitrogen fixation, denitrification, and carbon fixation (Supplementary Table S8). These results were somewhat different from the predicted major prokaryotic microbial functions. Because prokaryotic microbial community function prediction was performed with total sequences, predictions for key prokaryotic microbes were performed with annotated sequences for each prokaryotic microbial genus without those sequences unannotated or annotated as other prokaryotic microbes. However, *Montipora* spp. and *P. lutea* had different prokaryotic microbial genera with high betweenness centrality, and their main functions were similar, i.e., photosynthesis and nitrogen fixation. In *Montipora* spp., there were 21 variables with a betweenness centrality higher than 0.02 and 31 variables with a betweenness centrality higher than 0.01, accounting for 26.92% and 39.74% of the total network variability, respectively (Figure 4A). In *P. lutea*, there were 22 variables with a betweenness centrality higher than 0.02 and 36 variables with a betweenness centrality higher than 0.01, accounting for 30.98% and 50.70% of the total network variability, respectively (Figure 4B). Several dominant prokaryotic microbial genera, such as *Endozoicomonas* and *Acinetobacter*, did not display high levels of betweenness centrality in the algal–microbial correlation network, while some functional microbes, such as Cyanobacteria, had strong correlations with Symbiodiniaceae. Stress-resistant Symbiodiniaceae, i.e., C15 and *Durusdinium*, showed a higher betweenness centrality in *P. lutea* than in *Montipora* spp. (C15L_MO_ = 0.0078, C15L_PO_ = 0.23; D2_MO_ = 0, D2_PO_ = 0.027; D17_MO_ = 0, D17_PO_ = 0.17).

Concerning ongoing climate change and the features of different Symbiodiniaceae types, the Symbiodiniaceae positively correlated with temperature in each coral genus were identified as potentially stress-resistant Symbiodiniaceae and labeled in blue. C15.9, C15a, C15L, and C116 were found to be positively correlated with temperature in both coral genera. Stress-resistant Symbiodiniaceae maintained higher betweenness centrality in the network of *P. lutea*. Although not found to be positively correlated with temperature in this study, other stress-resistant Symbiodiniaceae, e.g., other C15 and *Durusdinium* (D2, D17), displayed more significant roles in the network of *P. lutea* than in the network of *Montipora* spp. Subnetworks involving significant correlations between stress-resistant Symbiodiniaceae and prokaryotic microbial variables for both coral genera were formed, demonstrating variability in the nature and number of correlations between different coral genera (Supplementary Figure S6). *Montipora* spp. still presented higher network density in the subnetwork than did *P. lutea*. More prokaryotic microbial variables with a higher proportion of significant correlations were detected to interact significantly with potentially stress-resistant Symbiodiniaceae in *P. lutea*. The two networks have different prokaryotic microbial variables with high betweenness centrality, but these prokaryotic microbial variables maintained similar functions, including photosynthesis, carbon fixation, and nitrogen fixation. Only one prokaryotic microbial variable was significantly correlated with *Durusdinium* in *Montipora* spp., while in the *P. lutea* network, there were 25 prokaryotic microbial variables. In *Montipora* spp., prokaryotic microbes positively correlated with *Durusdinium* performed denitrification functions, but no photosynthesis function was predicted. In *P. lutea*, most of the prokaryotic microbial variables positively correlated with *Durusdinium* performed photosynthesis, carbon fixation, or nitrogen fixation functions, while those prokaryotic microbes negatively correlated with *Durusdinium* mostly had denitrification functions (only g_*Prosthecochloris* was predicted to maintain photosynthesis and nitrogen fixation functions). The results of CoNet (Supplementary Figure S7) were somewhat similar to those of Spearman’s correlation analysis comparing the networks of *P. lutea* and *Montipora* spp., in that potentially stress-resistant Symbiodiniaceae exhibited high betweenness centrality in *P. lutea*. However, more bacterial genera maintaining photosynthesis and nitrogen fixation functions showed key roles in networks drawn from CoNet. As CoNet also combined Pearson’s correlation and Kendall rank correlation in its calculation and only correlations supported by all methods were kept; thus, significant correlations were minimized in the network. In this way, some prokaryotic microbial variables could have a few more correlations than did Symbiodiniaceae variables, which eventually displayed much higher betweenness centrality.

## Discussion

### Symbiodiniaceae Diversity and Community Composition

In ANOSIM and dbRDA, both *Montipora* spp. and *P. lutea* showed flexible coral–algal symbioses under different environmental conditions, while Symbiodiniaceae communities with *Montipora* spp. appeared to be more vulnerable to environmental changes in the environment, and those with *P. lutea* were more stable. The structures of Symbiodiniaceae communities with *Montipora* spp. were simpler, with very few subdominant Symbiodiniaceae types, while those with *P. lutea* maintained several subdominant Symbiodiniaceae types. Highly flexible coral–algal symbiosis may benefit the survival of coral hosts by assisting them in adapting to environmental changes (Grottoli et al., 2014). *P. lutea* in Hong Kong has fared well (Duprey et al., 2016), while *Montipora* spp. have declined rapidly in recent years. The simpler Symbiodiniaceae community structures in *Montipora* spp. than in *P. lutea* may be the cause, as high biodiversity can help prevent declines in ecosystem functions (Yachi and Loreau, 1999). As there were only twelve samples for *Montipora* spp., a limited number of environmental factors can be used for dbRDA (Supplementary Table S9). The Symbiodiniaceae community of *Montipora* spp. were more affected by environments than was *P. lutea*, and *M. venosa* on Lamma Island was much more affected than was *M. peltiformis*. ANOSIM for each *Montipora* species also supported the conclusion from dbRDA (Supplementary Table S10).

Temperature is an important environmental factor affecting coral–algal symbiosis (Cooper et al., 2011; Rodolfo-Metalpa et al., 2014; Silverstein et al., 2015), consistent with our past and present studies. Most factors considered have all been previously found to affect coral–algal symbiosis (Sawall et al., 2014; Duprey et al., 2016). Several studies have revealed that subdominant and rare Symbiodiniaceae types might maintain certain functions that are fundamental to corals (Baker, 2003; Ziegler et al., 2018). The same coral species had different subdominant/rare Symbiodiniaceae between Crescent Bay and Lamma Island in the same season, mainly due to the different nutrients. Thus, changes in nutrients could alter the structures of subdominant and rare Symbiodiniaceae communities and ultimately their functions. Several studies showed that the abundance of stress-resistant Symbiodiniaceae increased in corals under thermal stress, which was also consistent with the present study, as C15 was previously found to be stress-resistant (Jones et al., 2008; Fisher et al., 2012).

Some variability in Symbiodiniaceae communities cannot be explained well by environmental parameters in both coral species, especially in *P. lutea*. The environmental parameters were obtained from HKEPD and did not include Mg, K, light availability or other parameters that may be correlated with Symbiodiniaceae community variations. The large differences in the percentages of variation explained by environmental factors between *Montipora* spp. and *P. lutea* were due to biological and other factors that could not be quantified in the current study.

### Prokaryotic Microbial Diversity and Community Composition

The results of ANOSIM indicate that prokaryotic microbial communities in corals are largely affected by the surrounding seawater, suggesting that most coral-associated prokaryotic microbes may not be strictly symbiotic, consistent with many previous studies (Pantos et al., 2015; Jensen et al., 2019). Very rare microbial symbionts were detected, and these related microbial symbionts were usually less sensitive to the environment. Examples include *Ralstonia* spp. and *Actinobacter*, which are niche-specific (Ainsworth et al., 2015). Several common coral-associated bacteria were also detected in the present study, e.g., *Thalassospira* and *Endozoicomonas* (Bayer et al., 2013; Neave et al., 2017; Rubio-Portillo et al., 2018), and exhibited relatively high abundance in several coral samples. No holobiont-specific microbes have been detected thus far (Hester et al., 2016), and none were found in the present study, suggesting that, as with human and other mammalian microbiomes, a single-coral holobiont maintains a group of stable microbial symbionts that are likely fundamental to the stability of the whole community (Muegge et al., 2011).

Both ANOSIM and dbRDA results show that environmental conditions affect the algal and prokaryotic microbial communities in *Montipora* spp. similarly. However, environmental conditions have more impacts on the prokaryotic microbial communities than on the algal communities in *P. lutea*. Although most coral-associated prokaryotic microbes are not regarded as strictly symbiotic, they are not randomly selected. Some bacteria (e.g., f_Rhodospirillaceae_1) were present at high proportions in corals but at low proportions in seawater, while some bacteria (e.g., f_Rhodobacteraceae) were present at low proportions in corals but at high proportions in seawater. These observations suggest that corals can acquire certain prokaryotic microbes according to environmental conditions and that different coral species may prefer different prokaryotic microbes, which was also supported by previous studies (Roder et al., 2015; Ziegler et al., 2019). Prokaryotic microbial communities in *Montipora* spp. were more likely to be affected by the surrounding seawater than those in *P. lutea*, revealing more stable prokaryotic microbial communities in *P. lutea* than in *Montipora* spp. Studies have shown that microbial communities shift under stressful environments, which can affect coral reef functions (Webster et al., 2016; Ziegler et al., 2017). A stable microbial community provides corals with resistance under thermal stress (Grottoli et al., 2018), as demonstrated in the present study. The results of ANOSIM on the dissimilarities between seawater and coral samples would be affected by the limited number of seawater samples, but the analysis still showed that algal communities differed between corals and seawater as did prokaryotic microbial communities. Environmental factors had similar impacts on the algal and prokaryotic microbial communities in the same coral group, suggesting consistent changes in algal and prokaryotic microbial communities as well as in their potential interactions.

Studies have revealed that coral-associated microbes have ecological functions such as photosynthesis, nitrogen fixation, denitrification and carbon fixation that are potentially beneficial to coral holobionts (Rosenberg et al., 2007; Ainsworth et al., 2015; Yang et al., 2019). These functional prokaryotic microbes were all detected in the coral samples in the present study. PICRUSt revealed a small difference in the potential functions of prokaryotic microbial communities among different coral groups. This result suggests that although different coral species prefer different prokaryotic microbes, the prokaryotic microbes acquire similar functions to maintain coral holobiont fitness. Prokaryotic microbial communities in *P. lutea* might be able to acquire significantly increased photosynthetic capability in the summer season, thereby contributing to *P. lutea*’s greater survival in recent years. Although the structures of the prokaryotic microbial community did not show similar changes as those seen in previous studies due to different coral species and different environmental conditions, the functional profile changes of the prokaryotic microbial community were consistent (Ziegler et al., 2017). This result further supported that different corals could acquire different microbes to maintain similar essential functions.

### Covariance of Algae and Prokaryotic Microbes

Although the network of *Montipora* spp. included more variables, fewer variables with betweenness centrality values greater than 0.01 were detected, suggesting that fewer subdominant/rare Symbiodiniaceae/prokaryotic microbes play significant roles in *Montipora* spp., consistent with the findings from the heat map and the diversity index calculation. No *M. peltiformis* was found near Lamma Island, which is a more polluted area. *M. venosa* near Lamma Island should be more tolerant than *M. peltiformis*, which was found only in Crescent Bay. The network of *M. venosa* from Lamma Island displayed a more complicated structure than that of *M. peltiformis* from Crescent Bay (Supplementary Figure S8). Potentially stress-resistant Symbiodiniaceae also showed higher betweenness centrality in the *M. venosa* network. These factors can benefit *M. venosa*’s adaptation under stressful environments. Thus, different coral species in the same coral genus can have different adaptive powers and maintain diverse algal–microbial correlations. These findings reveal that a more diverse Symbiodiniaceae community, together with more correlations between stress-resistant algae and prokaryotic microbes, can help maintain coral health and strengthen the capacity of corals to adapt. Although network analysis could overestimate the true correlations between species (Friedman and Alm, 2012), this approach was effective at identifying the characteristic variables affecting algal–microbial correlations in corals.

Algal–microbial interactions may include all kinds of symbiotic relationships, such as mutualism, commensalism, and parasitism (Ramanan et al., 2016). However, studies of algal-bacterial interactions are limited, as it is difficult to separate the two partners. There has been direct proof that nanoplankton and cyanobacteria can exchange carbon and nitrogen (Thompson et al., 2012). Microbes can provide algae with vitamin B_12_ for fixed carbon in return (Croft et al., 2005). As an ecosystem that is highly sensitive to environmental change (Hoegh-Guldberg et al., 2007), corals are regarded as similar to the well-studied epiphytic lichens (Ramanan et al., 2016). Corals under bleaching conditions can alter prokaryotic microbial communities at the genus level, which might disrupt coral–algal symbiosis (Bourne et al., 2008; Tout et al., 2015). Disturbance of either algal or microbial communities could lead to coral mortality (Rosenberg et al., 2007; Barott et al., 2011).

Our findings that the key interacting prokaryotic microbes are mainly involved in photosynthesis, nitrogen fixation, carbon fixation, and denitrification are consistent with previous studies suggesting that Symbiodiniaceae can help prokaryotic microbes in coral tissue and that Cyanobacteria can also aid carbon fixation in Symbiodiniaceae (Rosenberg et al., 2007; Ainsworth et al., 2015). Some prokaryotic microbes can provide Symbiodiniaceae and corals with nitrogen (Lema et al., 2012). Symbiodiniaceae can also offer hosts and microbes high concentrations of oxygen to enhance their growth and prevent infection (Kühl et al., 1995). As more Symbiodiniaceae acquired high betweenness centrality in *P. lutea* than in *Montipora* spp., algal–microbial interactions in *P. lutea* should be more dependent on Symbiodiniaceae, and thus, an algal–microbial correlation network that is more reliant on Symbiodiniaceae could be beneficial to coral survival.

**FIGURE 5:**
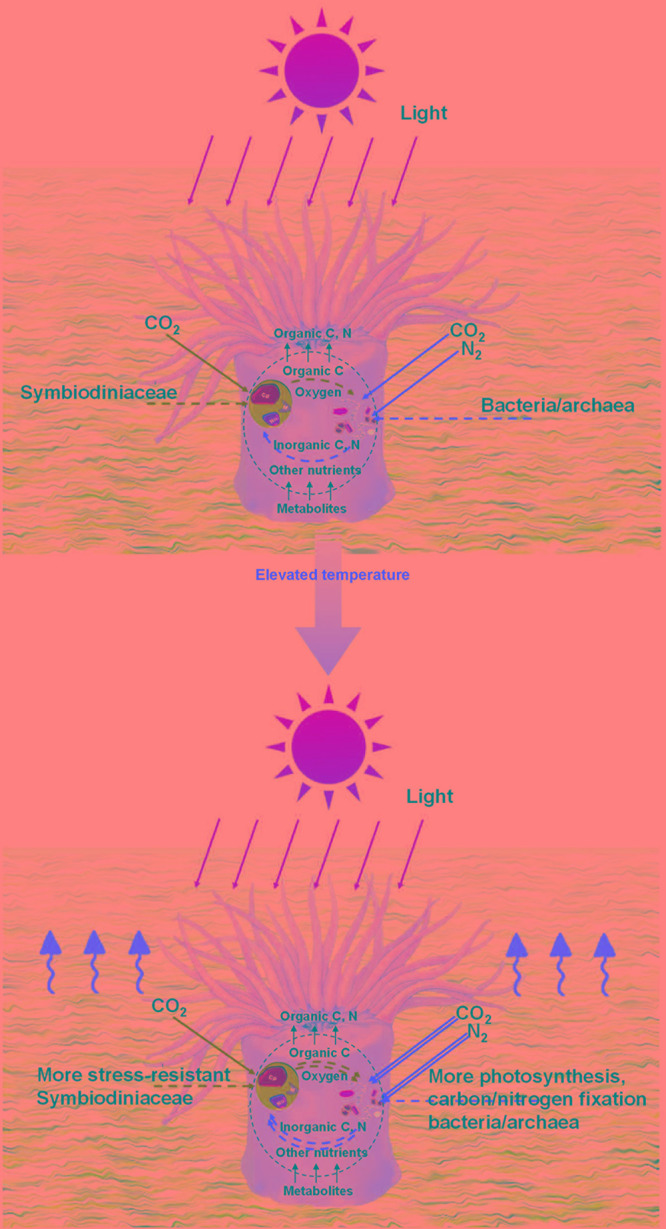
Potential mechanism for coral adaptation. Illustration of the algal–microbial partnership in coral holobionts at different temperatures. Symbiodiniaceae can provide microbes with oxygen and organic carbon for inorganic carbon and nitrogen in return. The coral microbiome as a whole provides corals with organic carbon, nitrogen, and oxygen. Double lines represent relative amplification in pathways compared with those seen in wintertime.

Different coral species are susceptible to different levels of environmental stress (Gardner et al., 2019). The characteristics of coral species and the features of coral–microbial associations can both contribute to coral stress resistance. Potentially stress-resistant Symbiodiniaceae, together with C15 and *Durusdinium* (known as stress-resistant Symbiodiniaceae) (Berkelmans and Van Oppen, 2006; Fisher et al., 2012; Jih-Terng Wang et al., 2012), which will be important for coral survival under future climate change, interacted with more prokaryotic microbial variables in *P. lutea*. Stress-resistant Symbiodiniaceae in *P. lutea* also possibly accounted for a larger proportion of the Symbiodiniaceae community. These factors can help establish stress-resistant algal–microbial partnerships as well as stress-resistant coral–algal symbiosis in *P. lutea*, which could partly explain why *P. lutea* in Hong Kong has fared better in recent years. Potentially stress-resistant Symbiodiniaceae, including C15, were subdominant in the Symbiodiniaceae community in *P. lutea* compared with C2r, but exhibited central roles in the algal–microbial networks. In *Montipora* spp., dominant but not stress-resistant Symbiodiniaceae, e.g., C3d and C2r, played key roles in the algal–microbial network. Meanwhile, in *M. venosa* samples from Lamma Island in summer, potentially stress-resistant Symbiodiniaceae C116 was found to become dominant for adaption to the high pollution in this area during the summer. We interpret this as evidence that stress-resistant algal–microbial partnerships are critical to coral adaptation. C15 was found to maintain better photosynthetic fitness *in-hospite* than C3 under elevated temperatures (Fisher et al., 2012). *Durusdinium* maintains low photosynthesis efficiency (Cunning et al., 2015), while the photosynthetic efficiency of *Durusdinium in-hospite* could be less of a disadvantage at higher temperatures. The finding that the photosynthetic capacity of prokaryotic microbial communities in *P. lutea* increased significantly in the summer season supports our conjecture that prokaryotic microbes maintaining photosynthesis and carbon fixation might assist energy production in stress-resistant Symbiodiniaceae as a potential adaptation mechanism for coral growth under elevated temperatures (Figure 5). At low temperatures, as in winter, corals mainly select various Symbiodiniaceae to maintain photosynthetic efficiency because it has no heat stress but needs sufficient production for the holobiont. When the temperature rises in summer, the coral may have to introduce more stress-resistant but less photosynthetically efficient Symbiodiniaceae. To maintain the holobiont function under elevated temperatures or to compensate for the low photosynthetic efficiency characteristic of some stress-resistant Symbiodiniaceae, such as *Durusdinium*, certain beneficial microorganisms (Peixoto et al., 2017), particularly those involved in the ecological functions of photosynthesis and nitrogen fixation, may actively interact with Symbiodiniaceae and the corals.

## Conclusion

We identified correlations between symbiotic algae and prokaryotic microbes in corals by means of high-throughput sequencing and network analysis. The results highlight the potential functions of algal–microbial correlations in coral hosts and propose a theoretic algal–microbial aid mechanism for coral meta-organisms at elevated temperatures. Further research is required to explore the nature of the effects that particular prokaryotic microbes have on Symbiodiniaceae or nutrient cycling. Long-term investigation of the dynamics of algal–microbial interactions is necessary to determine the critical partnerships that would benefit future coral reef conservation and management.

## Data Availability Statement

The datasets generated for this study can be found in the National Center for Biotechnology Information (NCBI) Sequence Read Archive under the accession numbers SRP066283 and SRR2917918.

## Author Contributions

HT, HH, and P-YQ contributed to the conception and design of the study. HT, LC, GZ, and WZ wrote the sections of the manuscript. All authors contributed to the manuscript revision, read and approved the submitted version.

## Conflict of Interest

The authors declare that the research was conducted in the absence of any commercial or financial relationships that could be construed as a potential conflict of interest.
