# Supplementary material for: Correlations Between Prokaryotic Microbes and Stress-Resistant Algae in Different Corals Subjected to Environmental Stress in Hong Kong
Source: Front Microbiol. 2020 Apr 23;11:686. doi: 10.3389/fmicb.2020.00686 (PMC7191007; doi:10.3389/fmicb.2020.00686)
Supplement: Supplementary file 12 [file Data_Sheet_1.pdf]

*__Supplementary Material__*

## Supplementary Figures

**Supplementary Figure 1.** Map of sampling sites ( △ ) and Hong Kong Environmental Protection Department (HKEPD) monitoring stations. https://www.map.gov.hk/gm/map/

**Supplementary Figure 2.** Symbiodinaceae community structures in different samples at genus level.

**Supplementary Figure 3.** Phylogenetic trees of analyzed Symbiodiniaceae types by RAxML under the GTRGAMMA model. (A) Topology of the phylogenetic tree. Bootstrapping values equal to/greater than 50 were shown. (B) Rooted phylogenetic tree displaying genetic distances among Symbiodiniaceae types.

**Supplementary Figure 4.** Microbial community structures in different samples at phylum level.

**Supplementary Figure 5.** Relationships between environmental conditions and algal/prokaryotic microbial communities from different samples. dbRDA indicates the relationships between environmental factors and microbial communities from different samples. (A) dbRDA of Symbiodiniaceae communities. The first axis explains 19.49% of the total variation and 95.05% of the fitted variation, while the second axis explains 0.86% of the total variation and 4.22% of the fitted variation. (B) dbRDA of microbial communities. The first axis explains 26.20% of the total variation and 56.48% of the fitted variation, while the second axis explains 16.21% of the total variation and 34.96% of the fitted variation.

**Supplementary Figure 6.** Organic correlation network visualizing significant pairwise correlations between potentially stress-resistant Symbiodiniaceae and prokaryotic microbes in *Montipora* spp. (A) and *Porites lutea* (B). Green/blue and red nodes represent potentially stress-resistant Symbiodiniaceae and microbes, respectively. Node size is reflective of the betweenness centrality of the variable. Line types (solid = positive and dashed = negative) are indicative of the Spearman correlation coefficient. Line colors are indicative of Spearman’s correlation significance, a brighter color represents a more significant correlation.

**Supplementary Figure 7.** Organic correlation network visualizing significant pairwise correlations between Symbiodiniaceae and microbes in *Montipora* (A) and *Porites* (B) using CoNet analysis. Green and red nodes represent Symbiodiniaceae and microbes, respectively. Node size is reflective of the betweenness centrality of the variable. Line types are indicative of edge weight. Line colors represent edge betweenness and dark colors represent low values.

**Supplementary Figure 8.** Organic correlation network visualizing significant pairwise correlations between Symbiodiniaceae and microbes in *Montipora* spp. at Crescent Bay (A) and Lamma Island (B). Green and red nodes represent Symbiodiniaceae and microbes, respectively. Node size is reflective of the betweenness centrality of the variable. Line types (solid = positive and dashed = negative) are indicative of the Spearman correlation coefficient. Line colors are indicative of Spearman’s correlation significance, a brighter color represents a more significant correlation.

## Supplementary Tables

**Supplementary Table S1.** Detailed information of the Hong Kong Environmental Protection Department (HKEPD) water quality monitoring stations.

**Supplementary Table S2.** Averaged Environmental data of the two sampling sites.

**Supplementary Table S3.** Total Environmental data of the two sampling sites.

**Supplementary Table S4.** Barcode primers for 16S and ITS2 samples.

**Supplementary Table S5.** Shannon diversity index and sequence information of Symbiodiniaceae/prokaryotic microbial community in different samples.

**Supplementary Table S6.** Total functional prediction for prokaryotic microbial community by PICRUSt.

**Supplementary Table S7.** Network parameters for *Montipora* spp. and *Porites lutea*.

**Supplementary Table S8.** Functional prediction for key microbes by PICRUSt.

**Supplementary Table S9.** dbRDA of environmental impacts on *Montipora* spp. in different sites.

**Supplementary Table S10.** ANOSIM of Symbiodiniaceae and microbial communities in *Montipora* spp. from different sites and months.

**Supplementary Table S11.** Library ID for each sample in the current study.

**Supplementary Data**

**Supplementary Data Sheet 2.** Analysis code for the present study.

**Supplementary Data Sheet 3.** OTU table of the analyzed sequences in the current study.
