# Supplementary material for: Correlations Between Prokaryotic Microbes and Stress-Resistant Algae in Different Corals Subjected to Environmental Stress in Hong Kong
Source: Front Microbiol. 2020 Apr 23;11:686. doi: 10.3389/fmicb.2020.00686 (PMC7191007; doi:10.3389/fmicb.2020.00686)
Supplement: Supplementary file 13 [file Data_Sheet_2.pdf]

Analysis code

```
# Demultiplex

split_libraries_fastq.py -i seqs.fastq.gz -b barcode.fastq.gz -o seqs/

#!/bin/bash
trap 'error' ERR
set -e

# interactive commands are commented out
#print_qiime_config.py

# Pick OTUs command
echo "pick otus"
pick_otus.py -i seqs.fna -o otus/uclust_picked_otus -z
cd otus/uclust_picked_otus
cp seqs_otus.txt seqs_otus_with_singleton.txt
find -name seqs_otus.txt | xargs perl -pi -e 's/denovo\d+\t\w+\n//g'
cd ..

# Pick representative set command
```

```
echo "pick rep seq"

mkdir rep_set

pick_rep_set.py -i uclust_picked_otus/seqs_otus.txt -f ../seqs.fna -l

rep_set/seqs_rep_set.log -m longest -o rep_set/seqs_rep_set.fasta

# Assign taxonomy command

assign_taxonomy.py -i rep_set/seqs_rep_set.fasta -o rdp_assigned_taxonomy -c

0.5 --rdp_max_memory 8000



# remove ekaryotes, chloroplasts and mitochondria

cd rdp_assigned_taxonomy

grep -i eukaryot seqs_rep_set_tax_assignments.txt | sed 's/\t/;/g' | cut -d ';' -f 1 >

removed.list

grep -i chloroplast seqs_rep_set_tax_assignments.txt | sed 's/\t/;/g' | cut -d ';' -f

1 >> removed.list

grep -i mitochond seqs_rep_set_tax_assignments.txt | sed 's/\t/;/g' | cut -d ';' -f

1 >> removed.list

cd ..

cd uclust_picked_otus

cp /home/tianrenmao/scripts/remove_from_otu_table.pl .
```

```
perl remove_from_otu_table.pl

seqs_otus.txt ../rdp_assigned_taxonomy/removed.list > 1

mv 1 seqs_otus.txt

cd ..

pick_rep_set.py -i uclust_picked_otus/seqs_otus.txt -f ../seqs.fna -l

rep_set/seqs_rep_set.log -m longest -o rep_set/seqs_rep_set.fasta




# Align sequences command

echo "align--pynast-silva"

align_seqs.py -i rep_set/seqs_rep_set.fasta -o pynast_aligned_seqs -e 100 -t

/share/apps/Silva/Silva_108/core_aligned/Silva_108_core_aligned_seqs.fasta


echo "align--pynast-core"

align_seqs.py -i rep_set/seqs_rep_set.fasta -o pynast_aligned_seqs_core -e 100


#echo "align--muscle"

#align_seqs.py -i rep_set/seqs_rep_set.fasta -m muscle -o muscle_aligned_seqs
```

```
#chimera

echo "identify chimeric seq"

identify_chimeric_seqs.py -m ChimeraSlayer -i

pynast_aligned_seqs_core/seqs_rep_set_aligned.fasta -a /share/apps/qiime-

1.7.0-deployed/core_set_aligned.fasta.imputed -o chimeric_seqs.txt


filter_alignment.py -i pynast_aligned_seqs_core/seqs_rep_set_aligned.fasta -o

pynast_aligned_seqs_core


filter_fasta.py -f pynast_aligned_seqs_core/seqs_rep_set_aligned_pfiltered.fasta -

o non_chimeric_rep_set_aligned.fasta -s chimeric_seqs.txt -n


# Build phylogenetic tree command

make_phylogeny.py -i non_chimeric_rep_set_aligned.fasta -o rep_set.tre


echo "make otu table"

# Make OTU table command

make_otu_table.py -i uclust_picked_otus/seqs_otus.txt -t

rdp_assigned_taxonomy/seqs_rep_set_tax_assignments.txt -o otu_table.txt -e
```

```
chimeric_seqs.txt

cd ..

#OTU Heatmap
echo "OTU Heatmap"
make_otu_heatmap_html.py -i otus/otu_table.txt -o otus/OTU_Heatmap/

#OTU Network
echo "OTU Network"
make_otu_network.py -m map_1d.txt -i otus/otu_table.txt -o otus/OTU_Network

#Make Taxa Summary Charts
echo "Summarize taxa"
rm -rf wf_taxa_summary ; summarize_taxa_through_plots.py -i otus/otu_table.txt -
o wf_taxa_summary -s -m map_1d.txt

echo "Alpha rarefaction"
#alpha_diversity.py -h
```

```
echo "alpha_diversity:metrics
shannon,PD_whole_tree,chao1,observed_species" > alpha_params.txt

rm -rf wf_arare ; alpha_rarefaction.py -i otus/otu_table.txt -m map_1d.txt -o
wf_arare/ -p alpha_params.txt -t otus/rep_set.tre -n 500 -e 30000
#improved: alpha_rarefaction.py -i otus/otu_table.txt -m map_1d.txt -o
wf_arare_improved/ -p ~/scripts/qiime_parameters.txt   -t otus/rep_set.tre -n 50 --
min_rare_depth 10 -e 3000 -a -O 32 &

echo "Beta diversity and plots"
rm -rf wf_bdiv ; beta_diversity_through_plots.py -i otus/otu_table.txt -m
map_1d.txt -o wf_bdiv/ -t otus/rep_set.tre -e 100 -p ~/scripts/qiime_parameters.txt

echo "Jackknifed beta diversity"
rm -rf wf_jack ; jackknifed_beta_diversity.py -i otus/otu_table.txt -t otus/rep_set.tre
-m map_1d.txt -o wf_jack -e 100 -p ~/scripts/qiime_parameters.txt

echo "Make Bootstrapped Tree"
make_bootstrapped_tree.py -m
wf_jack/unweighted_unifrac/upgma_cmp/master_tree.tre -s
```

wf_jack/unweighted_unifrac/upgma_cmp/jackknife_support.txt -o

wf_jack/unweighted_unifrac/upgma_cmp/jackknife_named_nodes.pdf

make_bootstrapped_tree.py -m

wf_jack/weighted_unifrac/upgma_cmp/master_tree.tre -s

wf_jack/weighted_unifrac/upgma_cmp/jackknife_support.txt -o

wf_jack/weighted_unifrac/upgma_cmp/jackknife_named_nodes.pdf

#Function predictions

normalize_by_copy_number.py -i otu_table.biom -o normalized_otus.biom

predict_metagenomes.py -i normalized_otus.biom -o

metagenome_predictions.biom
