# Supplementary figures and images for: Correlations Between Prokaryotic Microbes and Stress-Resistant Algae in Different Corals Subjected to Environmental Stress in Hong Kong
Source: Front Microbiol. 2020 Apr 23;11:686. doi: 10.3389/fmicb.2020.00686 (PMC7191007; doi:10.3389/fmicb.2020.00686)

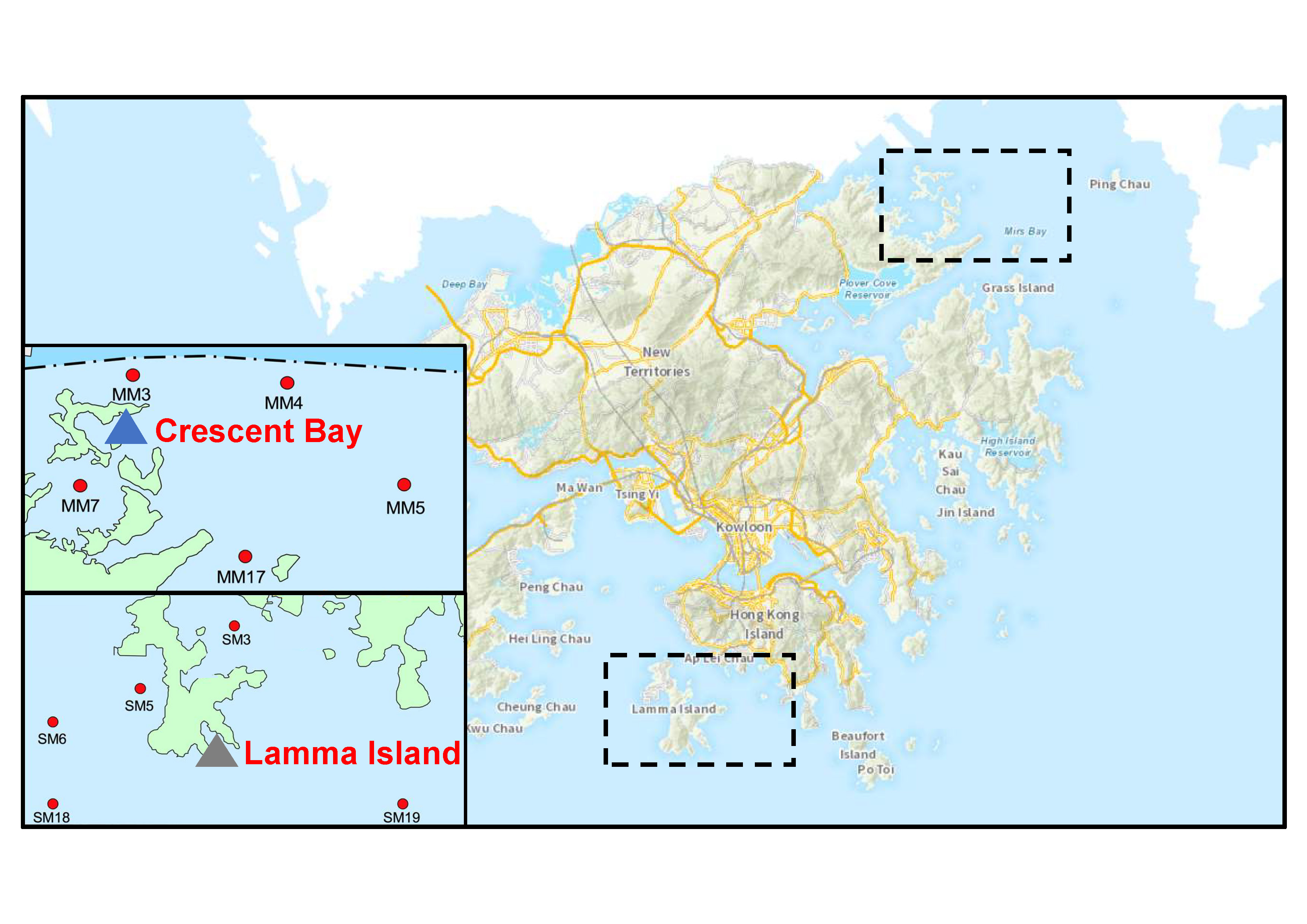

Supplement: Supplementary file 15 [file Image_1.JPEG]

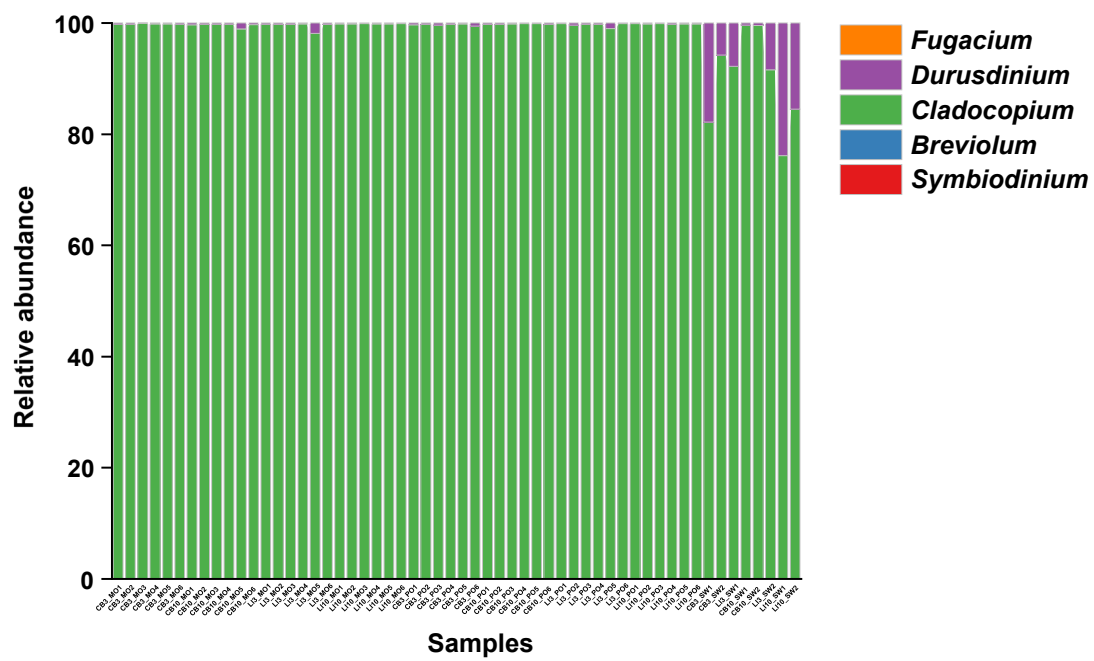

Supplement: Supplementary file 16 [file Image_2.PDF]

**A**

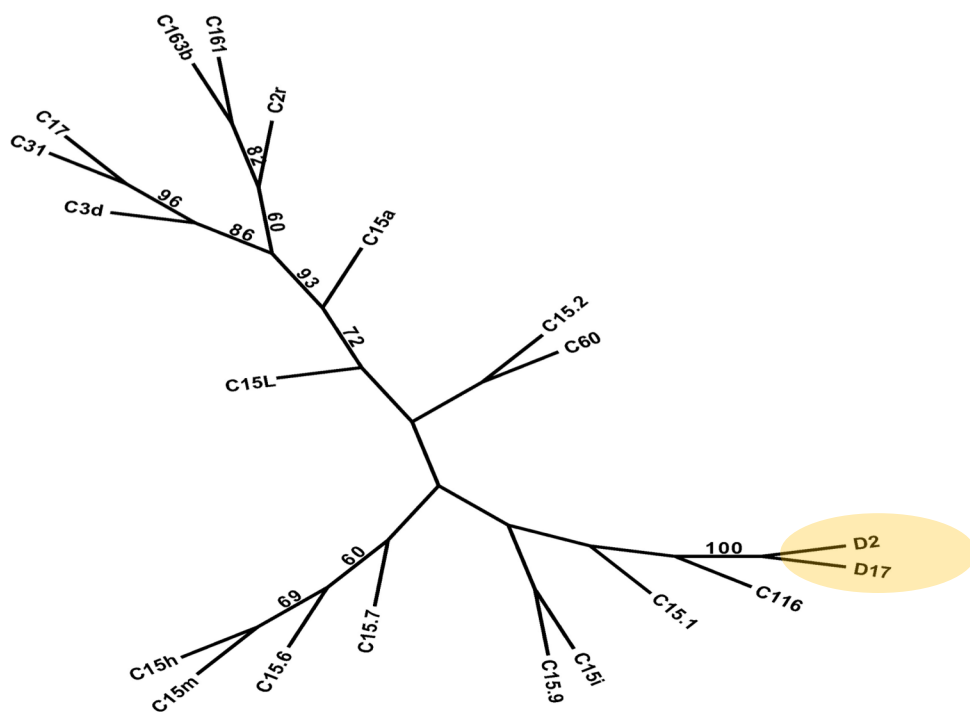

# B

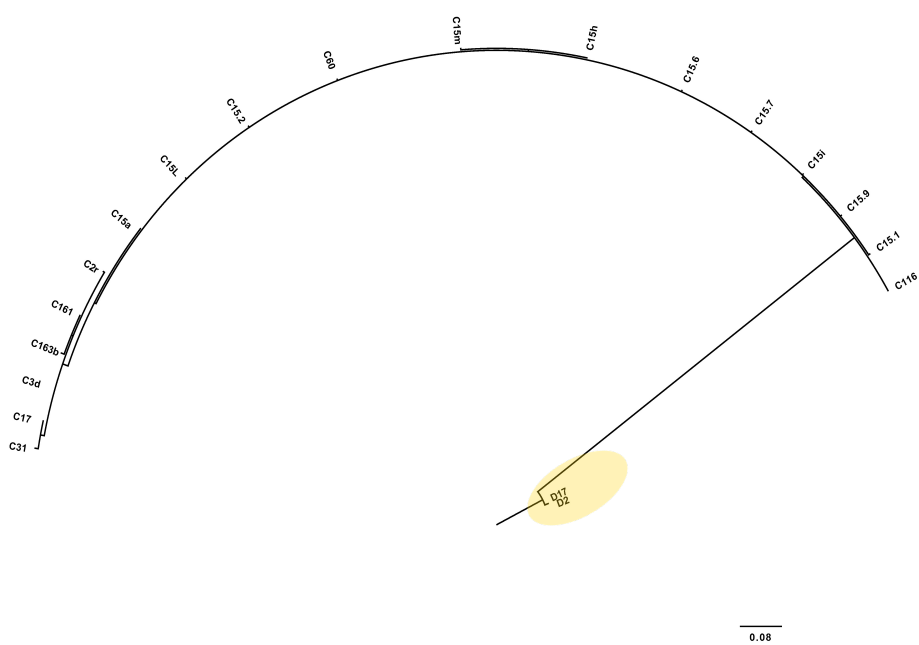

0.08

Supplement: Supplementary file 17 [file Image_3.PDF]

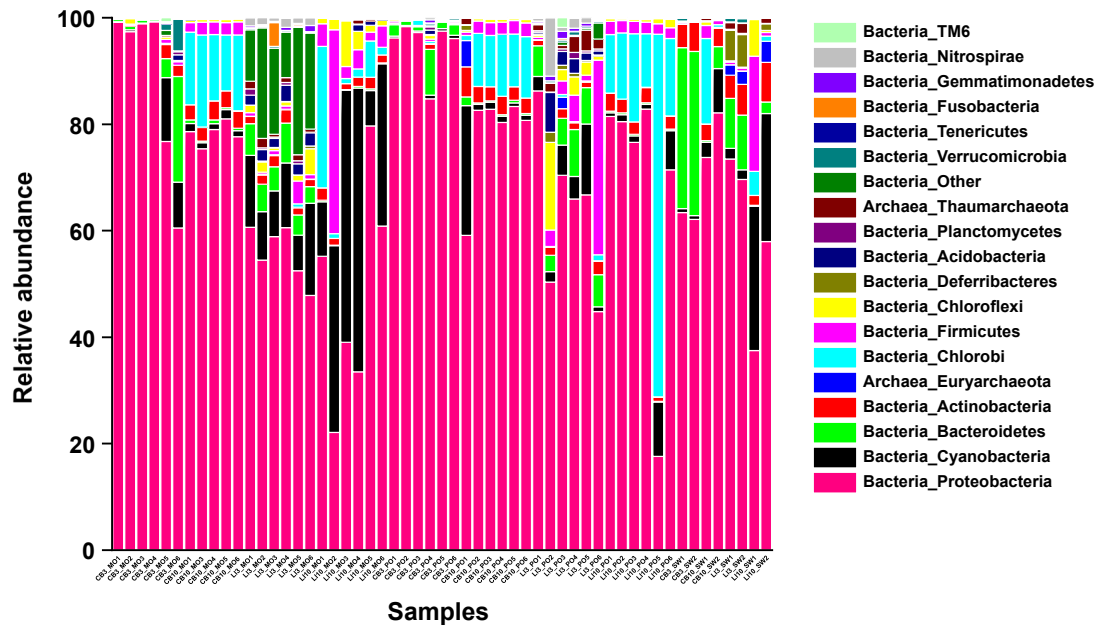

Supplement: Supplementary file 18 [file Image_4.PDF]

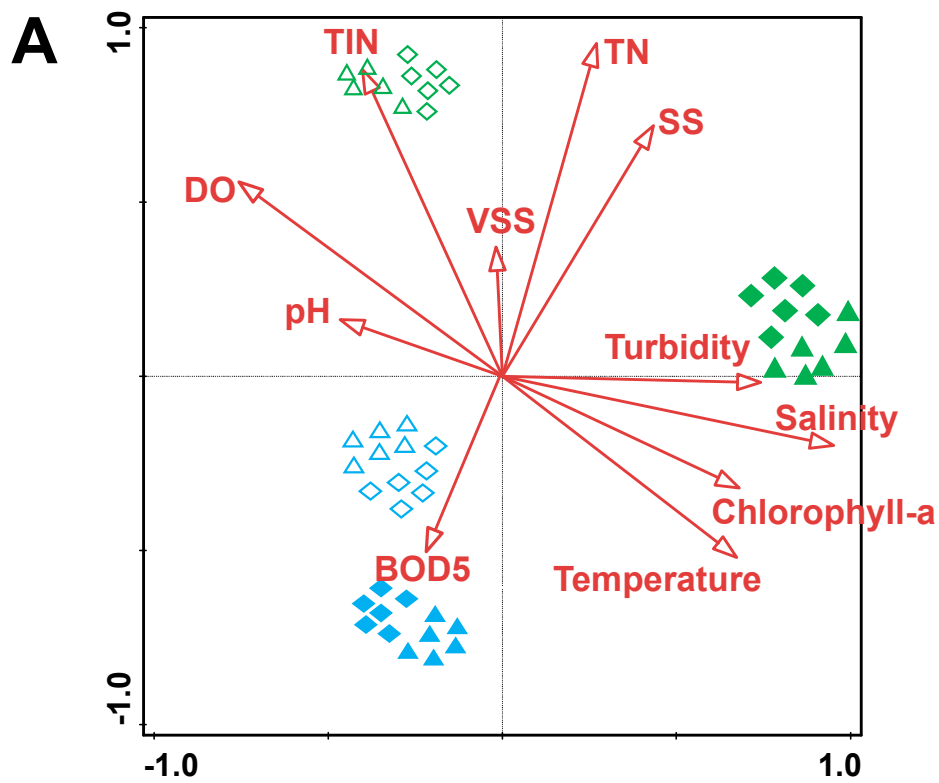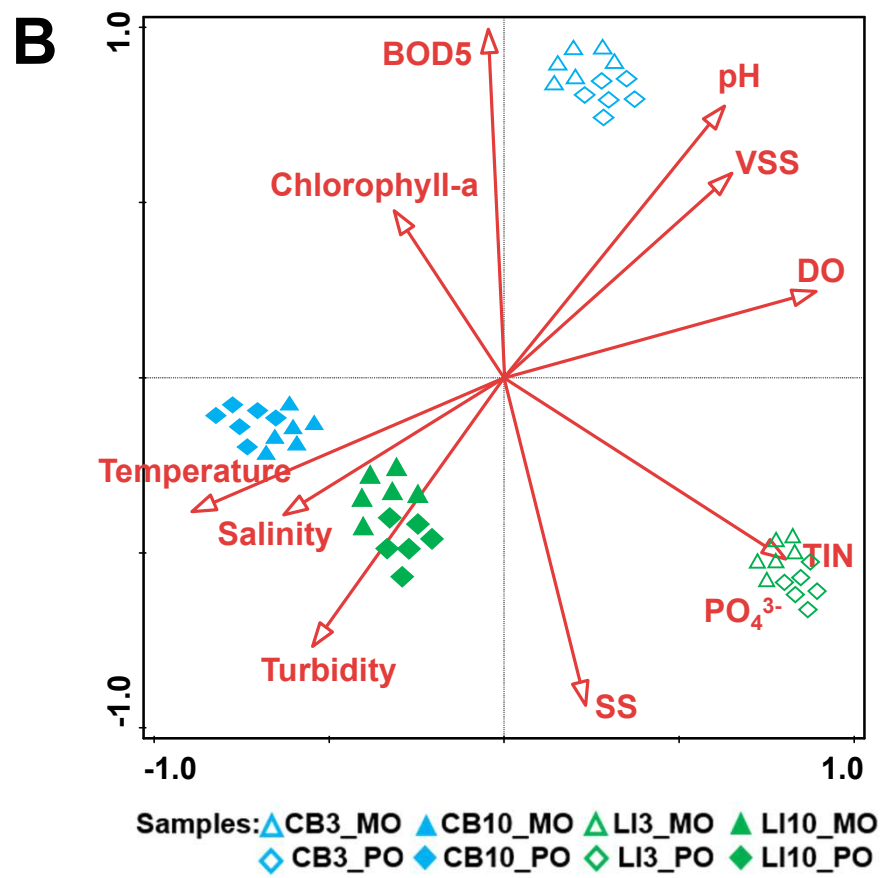

Supplement: Supplementary file 19 [file Image_5.PDF]

# A

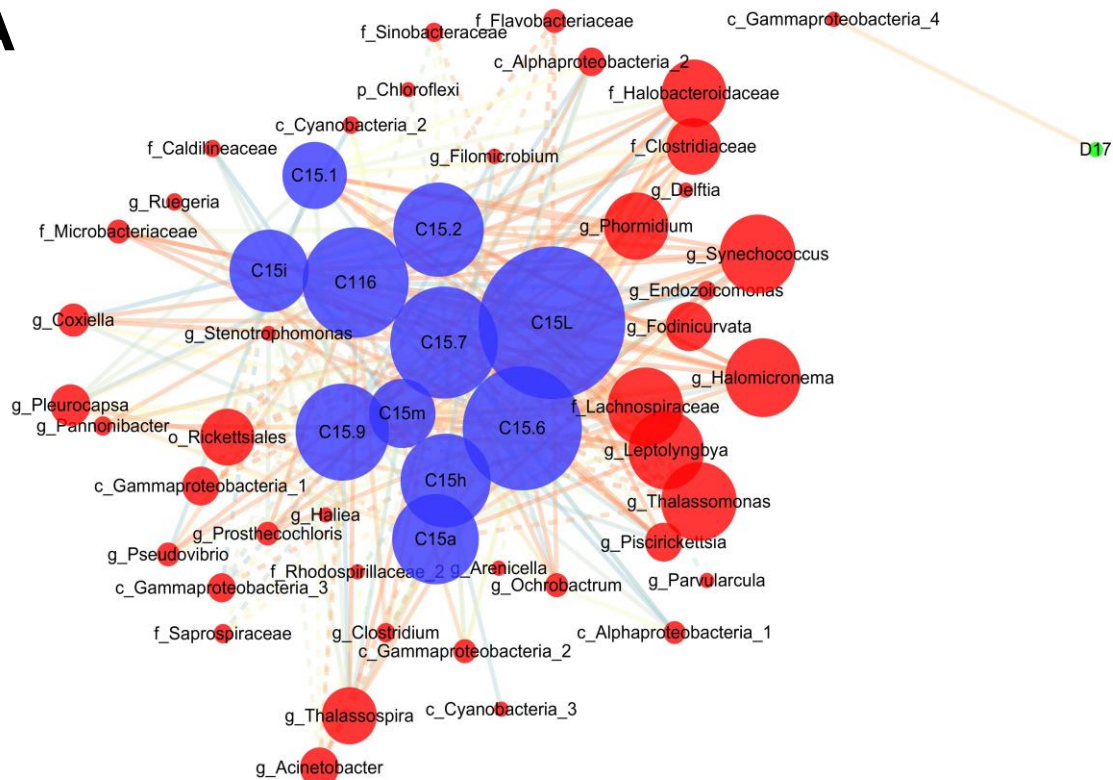

# B

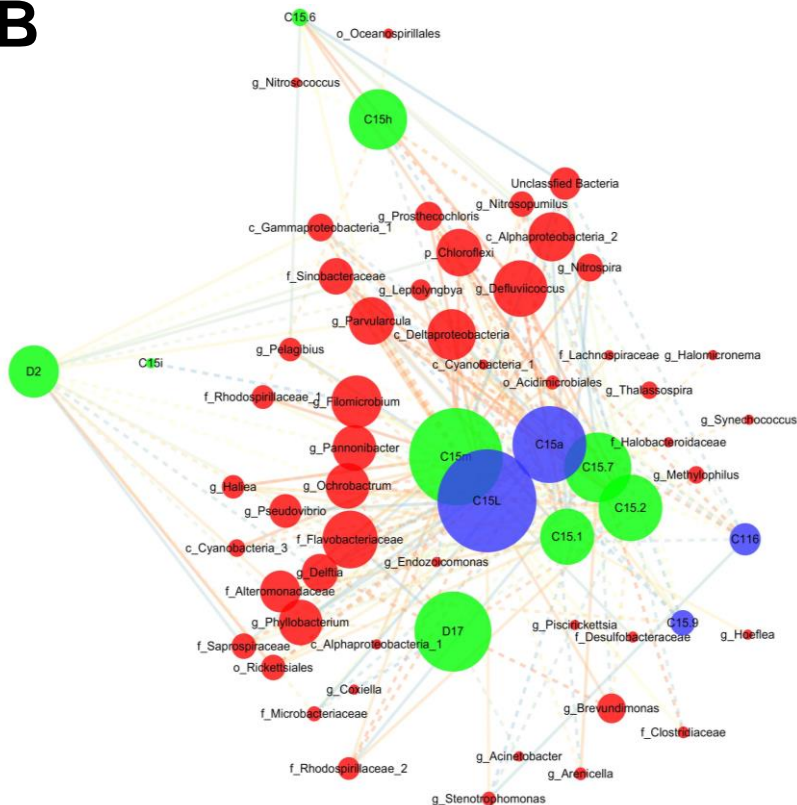

Supplement: Supplementary file 20 [file Image_6.PDF]

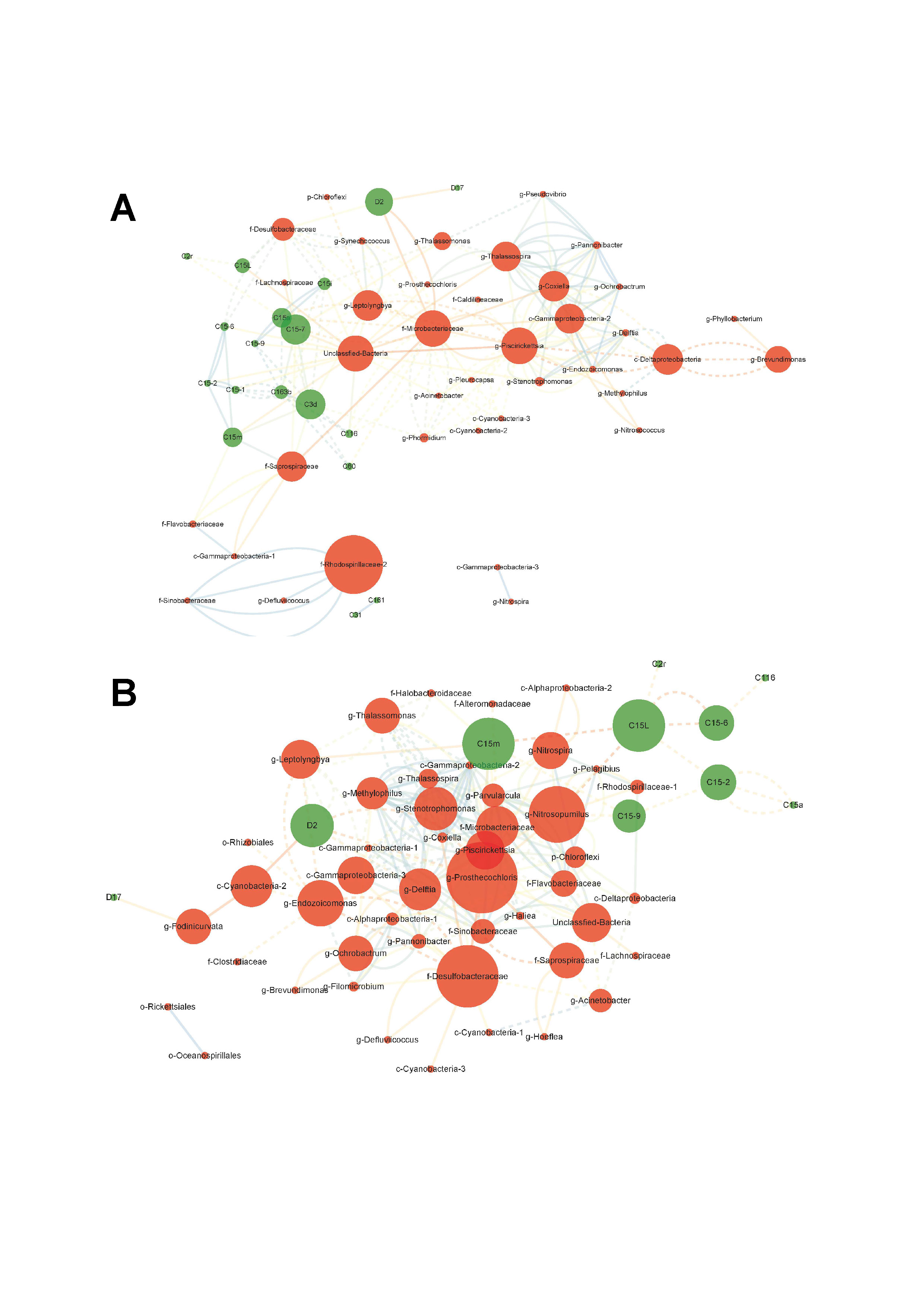

Supplement: Supplementary file 21 [file Image_7.JPEG]
