# Supplementary material for: Correlations Between Prokaryotic Microbes and Stress-Resistant Algae in Different Corals Subjected to Environmental Stress in Hong Kong
Source: Front Microbiol. 2020 Apr 23;11:686. doi: 10.3389/fmicb.2020.00686 (PMC7191007; doi:10.3389/fmicb.2020.00686)
Supplement: Supplementary file 14 [file Data_Sheet_3.pdf]

OTU table

{"id": "None","format": "Biological Observation Matrix 1.0.0","format_url": "http://biom-format.org","type": "OTU table","generated_by": "QIIME 1.7.0","date": "2015-08-27T06:04:31.600649","matrix_type": "sparse","matrix_element_type": "int","shape": [17828, 80],"data": [[0,0,11],[0,1,1],[1,2,3],[2,2,1],[2,3,1],[2,4,1],[2,5,1],[2,6,1],
[3,7,1],[3,8,1],[3,9,1],[3,10,2],[3,11,1],[3,12,2],[3,13,1],[3,14,1],
[3,15,1],[3,16,1],[3,17,2],[3,18,1],[3,19,1],[4,15,1],[4,20,1],
[5,21,1],[5,22,1],[6,2,1],[6,23,1],[7,24,1],[7,25,1],[7,26,1],
[7,27,1],[7,28,1],[8,2,1],[8,28,1],[9,0,2],[9,2,1],[9,3,2],[9,5,1],
[9,6,2],[9,8,1],[9,9,1],[9,10,1],[9,13,1],[9,20,3],[9,22,1],[9,23,6],
[9,24,4],[9,25,1],[9,27,1],[9,28,2],[9,29,1],[9,30,6],[9,31,1],
[9,32,3],[9,33,2],[9,34,1],[9,35,3],[9,36,1],[9,37,1],[9,38,1],
[10,9,1],[10,15,3],[10,20,1],[10,36,1],[10,39,1],[10,40,1],[11,9,1],
[11,11,1],[11,41,1],[12,12,1],[12,30,2],[13,21,1],[13,42,2],[14,3,1],
[14,24,2],[14,27,1],[14,43,1],[15,8,1],[15,20,1],[15,32,1],[16,3,1],
[16,5,4],[16,7,2],[16,8,1],[16,12,4],[16,20,6],[16,21,3],[16,25,5],
[16,27,2],[16,33,2],[16,37,1],[16,44,2],[16,45,1],[16,46,1],
[16,47,1],[16,48,1],[16,49,1],[16,50,1],[16,51,1],[16,52,1],
[17,23,1],[17,26,1],[17,39,1],[18,23,1],[18,25,1],[19,2,1],[19,5,1],
[19,11,1],[19,12,1],[19,20,2],[19,23,1],[19,29,1],[19,31,1],
[19,33,1],[19,53,1],[19,54,1],[19,55,1],[20,28,1],[20,56,1],
[21,35,1],[21,55,1],[22,1,1],[22,3,29],[22,5,7],[22,6,5],[22,10,3],
[22,11,1],[22,15,1],[22,16,1],[22,17,2],[22,18,1],[22,19,3],
[22,20,1],[22,21,3],[22,22,1],[22,23,7],
[22,24,18],[22,25,15],[22,26,6],[22,28,14],[22,30,2],[22,31,7],
[22,32,11],[22,33,1],[22,37,5],[22,38,3],[22,40,1],[22,41,2],
[22,42,2],[22,45,1],[22,46,3],[22,47,1],[22,48,12],[22,50,1],
[22,52,1],[22,53,1],[22,54,1],[22,56,38],[22,57,1],[22,58,1],
[22,59,4],[22,60,4],[22,61,1],[22,62,2],[22,63,1],[22,64,1],[22,65,1],
[22,66,1],[23,62,2],[24,2,8],[24,3,2],[24,4,1],[24,13,1],[24,20,1],
[24,22,1],[24,23,1],[24,26,1],[24,27,1],[24,28,2],[24,31,3],[24,32,4],
[24,39,3],[24,43,1],[24,62,1],[24,67,1],[25,35,3],[26,21,1],[26,28,2],
[26,31,1],[26,33,3],[26,34,1],[26,35,2],[27,6,1],[27,24,1],[28,28,1],
[28,35,2],[29,15,1],[29,55,1],[30,2,1],[30,24,1],[31,15,1],[31,19,1],
[31,28,2],[31,29,1],[31,33,2],[32,2,1],[32,31,1],[33,5,1],[33,22,1],
[33,27,2],[34,17,1],[34,28,1],[34,55,1],[35,28,11],[35,68,1],[36,2,2],
[36,31,4],[37,12,2],[37,25,3],[37,33,3],[38,25,1],[38,26,1],[38,43,1],
[39,4,1],[39,6,1],[39,28,1],[39,35,49],[39,43,1],[39,69,1],[40,23,1],
[40,26,1],[40,70,1],[41,3,2],[41,5,2],[41,25,1],[41,27,3],[41,32,1],
[41,39,2],[42,1,1],[42,3,3],[42,4,17],[42,5,1],[42,6,7],[42,9,1],
[42,10,1],[42,13,1],[42,15,7],[42,17,18],[42,21,1],[42,22,2],
[42,24,3],[42,25,2],[42,26,1],[42,27,4],[42,28,2],[42,30,1],[42,31,2],
[42,33,1],[42,34,1],[42,35,4],[42,38,6],[42,40,1],[42,42,2],[42,43,3],
[42,49,1],[42,50,12],[42,51,2],[42,53,19],[42,55,3],[42,56,2],
[42,65,2],[42,66,5],[42,67,1],[42,69,1],[42,70,2],[42,71,1],[42,72,2],
[42,73,1],[43,10,1],[43,19,1],[43,22,7],[43,24,1],[43,25,8],[43,27,6],
[43,28,4],[43,30,5],[43,32,9],[43,33,54],[43,35,33],[43,38,1],
[43,40,1],[43,47,1],[43,52,24],[43,58,1],[43,63,1],[43,65,1],
[44,28,4],[45,15,1],[45,17,1],[45,53,3],[46,2,1],[46,26,1],[46,55,1],
[47,17,2],[48,26,1],[48,31,1],[48,33,2],[48,51,1],[49,7,2],[50,22,1],

[50,66,1],[51,20,1],[51,28,1],[51,32,1],[52,28,4],[52,51,1],[52,66,1],
[53,3,1],[53,20,4],[53,21,1],[53,22,2],[53,26,2],[53,27,1],[53,31,3],
[53,33,1],[53,35,1],[53,36,1],[53,42,1],[53,74,1],[54,5,1],[54,6,1],
[54,25,1],[54,26,3],[54,28,1],[54,32,1],[54,33,3],[54,43,54],
[54,47,1],[54,50,1],[55,23,1],[55,27,1],[56,10,1],[56,15,2],[56,72,1],
[57,25,1],[57,26,1],[58,1,1],[58,5,1],[58,13,1],[58,17,2],[58,23,1],
[58,27,1],[58,28,1],[58,40,1],[58,50,4],[58,53,1],[58,70,1],[58,74,1],
[59,23,1],[59,24,4],[59,26,1],[59,27,1],[59,40,1],[59,48,1],[60,31,5],
[61,23,4],[61,24,1],[61,26,2],[61,28,1],[62,4,1],[62,21,1],[62,23,1],
[62,28,82],[62,35,1],[62,63,38],[62,69,2],[62,70,1],[62,74,31],
[62,75,21],[62,76,40],[63,3,1],[63,6,2],[63,22,2],[63,23,1],[63,26,1],
[63,28,1],[63,56,1],[63,66,1],[64,35,1],[64,48,1],[65,0,1],[65,54,1],
[66,2,2],[66,23,1],[67,5,1],[67,28,1],[68,6,2],[68,8,1],[68,20,1],
[68,35,2],[69,21,1],[69,33,1],[70,6,1],[70,42,1],[71,47,1],[71,68,1],
[72,0,16],[72,1,26],[72,2,15],[72,3,13],[72,4,1139],[72,5,87],
[72,6,4],[72,7,6],[72,8,8],[72,9,34],[72,10,62],[72,11,11],[72,12,16],
[72,13,17],[72,14,5],[72,15,811],[72,16,26],[72,17,2244],[72,18,8],
[72,19,70],[72,20,20],[72,21,15],[72,22,46],[72,23,17],[72,24,15],
[72,25,21],[72,26,13],[72,27,21],[72,28,36],[72,29,8],[72,30,8],
[72,31,13],[72,32,31],[72,33,23],[72,34,18],[72,35,21],[72,36,9],
[72,37,11],[72,38,208],[72,39,13],[72,40,5],[72,41,17],[72,42,11],
[72,43,10],[72,44,498],[72,45,22],[72,46,10],[72,47,13],[72,48,4],
[72,49,6],[72,50,1285],[72,51,1],[72,52,2],[72,53,2488],[72,54,14],
[72,55,2209],[72,56,14],[72,57,13],[72,58,15],[72,59,11],[72,60,7],
[72,61,2],[72,62,17],[72,63,11],[72,64,7],[72,65,22],[72,66,548],
[72,67,10],[72,68,4],[72,69,2],[72,70,7],[72,71,6],[72,72,2],
[72,73,8],[72,74,21],[72,75,14],[72,76,15],[72,77,12],[72,78,4],
[72,79,3],[73,16,1],[73,18,1],[74,1,1],[74,13,4],[74,39,1],[74,40,1],
[75,1,1],[75,2,1],[75,3,2],[75,12,3],[75,23,1],[75,37,1],[75,47,1],
[75,55,1],[75,60,2],[75,66,1],[75,77,1],[76,30,2],[76,32,1],[76,43,1],
[76,74,1],[77,27,4],[77,33,1],[77,35,1],[77,50,1],[78,23,2],[79,5,4],
[79,6,3],[79,9,1],[79,10,1],[79,18,1],[79,33,2],[79,42,3],[79,43,1],
[79,46,1],[79,47,4],[79,56,1],[79,72,1],[80,21,1],[80,31,1],[81,1,42],
[81,9,1],[81,29,27],[82,15,1],[82,17,1],[83,17,1],[83,31,1],[83,40,1],
[83,68,3],[83,70,1],[83,74,1],[84,28,1],[84,55,1],[84,56,14],[85,8,1],
[85,54,1],[85,57,1],[85,58,1],[86,19,1],[86,24,1],[86,27,1],[86,38,1],
[86,59,3],[87,20,3],[88,28,2],[88,42,1],[89,1,2],[89,7,2],[89,13,1],
[89,20,2],[89,28,1],[89,31,1],[89,62,1],[90,2,58],[90,4,1],[90,28,3],
[90,31,3],[90,35,1],[91,63,1],[91,74,1],[91,76,1],[92,0,3],[92,1,65],
[92,2,1],[92,8,4],[92,12,4],[92,13,1],[92,15,3],[92,17,2],[92,20,2],
[92,21,1],[92,25,1],[92,26,1],[92,28,1],[92,29,32],[92,30,4],
[92,31,9],[92,32,1],[92,34,4],[92,35,1],[92,36,6],[92,37,2],[92,39,3],
[92,42,1],[92,43,2],[92,45,1],[92,53,6],[92,55,2],[92,57,1],[92,60,1],
[92,73,1],[92,78,1],[93,2,8],[93,31,1],[94,28,2],[95,20,2],[95,23,1],
[95,27,2],[95,43,1],[95,59,1],[96,19,1],[96,65,1],[97,3,1],[97,21,1],
[98,25,5],[98,28,1],[98,30,4],[98,32,35],[98,33,9],[98,35,17],
[98,40,1],[98,52,9],[99,2,2],[99,17,1],[100,42,1],[100,62,1],
[101,25,1],[101,28,1],[101,33,4],[101,50,1],[101,62,1],[101,74,1],
[102,10,1],[102,16,1],[102,65,1],[103,32,6],[104,1,32],[104,5,1],
[104,12,2],[104,13,9],[104,19,1],[104,20,3],[104,23,13],[104,24,3],

[104,25,1],[104,26,1],[104,29,3],[104,32,12],[104,33,1],[104,34,1],
[104,36,1],[104,37,1],[104,42,15],[104,43,1],[104,44,1],[104,45,1],
[104,47,3],[104,50,1],[104,51,4],[104,53,1],[104,54,1],[104,59,1],
[104,63,1],[104,65,1],[104,66,2],[104,68,1],[104,69,1],[104,70,4],
[104,71,1],[104,74,3],[104,75,1],[104,76,1],[104,77,1],[105,16,1],
[105,22,5],[106,28,1],[106,50,1],[107,9,1],[107,13,1],[107,53,1],
[107,71,1],[108,5,2],[108,9,1],[108,24,2],[108,31,1],[108,52,1],
[108,66,1],[108,75,1],[109,41,1],[109,60,1],[109,65,1],[110,4,1],
[110,5,1],[110,22,1],[110,35,2],[110,69,1],[110,70,1],[111,39,1],
[111,62,2],[112,23,1],[112,62,1],[113,2,2],[114,28,2],[115,24,2],
[115,48,1],[116,2,1],[116,28,1],[117,1,3],[117,2,1],[117,3,2],
[117,5,1],[117,8,1],[117,13,18],[117,20,1],[117,21,2],[117,22,1],
[117,23,3],[117,24,2],[117,25,3],[117,26,2],[117,27,11],[117,28,1],
[117,31,2],[117,32,2],[117,34,2],[117,35,3],[117,36,1],[117,40,1],
[117,41,1],[117,42,1],[117,43,4],[117,54,1],[117,59,3],[117,62,8],
[117,70,1],[117,76,1],[118,2,6],[119,1,1],[119,20,1],[120,44,1],
[120,66,1],[121,27,1],[121,28,1],[122,7,11],[122,8,1],[122,10,1],
[123,2,1],[123,4,1],[123,9,1],[123,13,1],[123,15,3],[123,28,6],
[123,31,3],[123,38,11],[123,44,51],[123,47,1],[123,50,4],[123,55,1],
[123,56,1],[123,66,41],[123,70,1],[123,74,1],[124,27,2],[124,43,1],
[125,22,1],[125,28,2],[126,8,1],[126,13,1],[126,26,1],[126,42,1],
[126,43,1],[126,50,1],[126,60,1],[127,4,2],[127,31,1],[127,45,1],
[127,50,4],[128,9,3],[128,12,2],[128,20,1],[128,30,3],[128,33,1],
[128,47,1],[128,57,1],[128,78,1],[129,1,1],[129,9,1],[129,19,1],
[129,33,1],[129,38,2],[129,41,1],[129,42,1],[129,44,1],[129,47,1],
[129,50,3],[129,52,1],[129,55,1],[129,58,1],[129,59,1],[129,75,2],
[130,2,1],[130,3,1],[130,23,2],[130,62,1],[131,10,1],[131,20,1],
[132,2,1],[132,3,1],[132,5,1],[132,22,1],[132,23,1],[132,27,1],
[132,47,2],[133,2,12],[133,20,7],[133,21,2],[133,24,2],[133,27,1],
[133,28,1],[133,31,3],[133,32,1],[133,34,2],[133,37,2],[133,39,1],
[133,40,1],[133,42,6],[133,43,1],[133,46,1],[133,47,1],[133,57,1],
[133,78,1],[134,1,1],[134,2,12],[134,3,22],[134,4,1],[134,5,8],
[134,6,2],[134,7,1],[134,9,4],[134,10,2],[134,12,1],[134,13,1],
[134,16,2],[134,17,1],[134,20,15],[134,21,5],[134,22,5],[134,23,42],
[134,24,10],[134,25,18],[134,26,14],[134,27,43],[134,28,17],
[134,29,1],[134,30,6],[134,31,4],[134,32,5],[134,33,10],[134,34,2],
[134,35,10],[134,36,1],[134,37,3],[134,38,2],[134,39,5],[134,40,1],
[134,41,1],[134,42,26],[134,43,11],[134,44,1],[134,46,1],[134,47,22],
[134,48,2],[134,49,1],[134,54,1],[134,56,5],[134,59,1],[134,60,5],
[134,62,6],[134,64,1],[134,66,1],[134,70,1],[134,73,1],[134,74,1],
[134,75,1],[135,2,2],[135,31,1],[136,1,1],[136,9,3],[136,10,1],
[136,12,1],[136,15,11],[136,17,19],[136,19,1],[136,23,1],[136,24,1],
[136,26,2],[136,28,3],[136,29,1],[136,30,1],[136,33,1],[136,38,1],
[136,40,1],[136,41,1],[136,44,3],[136,45,1],[136,50,2],[136,53,24],
[136,55,2],[136,58,1],[136,64,1],[136,65,1],[136,66,3],[136,67,1],
[136,68,1],[136,74,1],[136,77,1],[137,26,2],[137,39,1],[138,15,2],
[138,17,1],[138,55,1],[138,65,1],[139,26,1],[139,52,2],[140,5,1],
[140,28,1],[141,38,2],[141,74,1],[142,32,11],[142,39,1],[143,5,3],
[143,26,4],[143,27,4],[143,28,1],[143,32,8],[143,35,1],[143,43,1],
[144,24,2],[144,55,1],[144,56,1],[144,62,1],[144,65,1],[145,2,1],

[145,23,1],[145,31,3],[145,42,1],[145,46,1],[145,47,1],[146,28,4],
[146,72,1],[147,4,1],[147,26,1],[147,47,1],[148,9,1],[148,15,1],
[148,16,1],[148,28,1],[148,46,1],[148,55,1],[148,58,1],[148,73,2],
[148,77,1],[149,0,44],[149,1,62],[149,2,14],[149,3,9],[149,4,19],
[149,5,19],[149,6,3],[149,7,18],[149,8,14],[149,9,55],[149,10,61],
[149,11,17],[149,12,55],[149,13,48],[149,14,21],[149,15,46],
[149,16,30],[149,17,31],[149,18,25],[149,19,35],[149,20,49],
[149,21,10],[149,22,13],[149,23,26],[149,24,23],[149,25,11],
[149,26,9],[149,27,32],[149,28,109],[149,29,15],[149,30,27],
[149,31,14],[149,32,16],[149,33,15],[149,34,25],[149,35,22],
[149,36,12],[149,37,18],[149,38,16],[149,39,19],[149,40,16],
[149,41,25],[149,42,9],[149,43,10],[149,44,27],[149,45,23],
[149,46,13],[149,47,9],[149,48,6],[149,49,9],[149,50,18],[149,51,8],
[149,52,5],[149,53,26],[149,54,12],[149,55,34],[149,56,9],[149,57,21],
[149,58,25],[149,59,20],[149,60,14],[149,61,3],[149,62,12],
[149,63,143],[149,64,7],[149,65,43],[149,66,22],[149,67,17],
[149,68,10],[149,69,4],[149,70,42],[149,71,9],[149,72,9],[149,73,21],
[149,74,200],[149,75,144],[149,76,219],[149,77,32],[149,78,2],
[150,3,1],[150,9,1],[150,19,1],[150,24,3],[150,36,2],[150,60,1],
[150,64,3],[151,20,4],[152,8,1],[152,43,2],[153,0,12],[153,1,77],
[153,2,5],[153,3,12],[153,4,9],[153,5,12],[153,6,6],[153,7,14],
[153,8,12],[153,9,45],[153,10,48],[153,11,19],[153,12,45],[153,13,41],
[153,14,17],[153,15,76],[153,16,26],[153,17,29],[153,18,29],
[153,19,29],[153,20,52],[153,21,7],[153,22,7],[153,23,17],[153,24,20],
[153,25,14],[153,26,15],[153,27,22],[153,28,95],[153,29,20],
[153,30,11],[153,31,14],[153,32,14],[153,33,11],[153,34,20],
[153,35,18],[153,36,9],[153,37,14],[153,38,12],[153,39,31],
[153,40,15],[153,41,23],[153,42,7],[153,43,7],[153,44,19],[153,45,24],
[153,46,10],[153,47,11],[153,48,7],[153,49,9],[153,50,24],[153,51,5],
[153,52,1],[153,53,41],[153,54,11],[153,55,60],[153,56,7],[153,57,14],
[153,58,17],[153,59,36],[153,60,5],[153,62,6],[153,63,39],[153,64,5],
[153,65,29],[153,66,18],[153,67,21],[153,68,13],[153,69,3],
[153,70,29],[153,71,7],[153,72,5],[153,73,14],[153,74,40],[153,75,17],
[153,76,51],[153,77,25],[153,78,5],[153,79,4],[154,7,1],[154,8,1],
[154,9,5],[154,10,6],[154,11,2],[154,14,2],[154,16,2],[154,18,2],
[154,19,2],[154,36,1],[154,37,3],[154,41,2],[154,45,3],[154,46,3],
[154,49,2],[154,50,1],[154,53,3],[154,54,1],[154,57,2],[154,58,2],
[154,64,1],[154,65,5],[154,67,1],[154,73,2],[154,77,2],[154,78,1],
[155,6,1],[155,33,2],[155,56,1],[156,4,1],[156,28,1],[157,3,4],
[157,4,2],[157,5,3],[157,6,5],[157,10,2],[157,13,5],[157,15,2],
[157,23,2],[157,24,5],[157,25,1],[157,26,2],[157,28,5],[157,31,1],
[157,32,1],[157,33,4],[157,37,3],[157,38,19],[157,39,1],[157,40,2],
[157,43,1],[157,44,53],[157,47,2],[157,48,2],[157,49,1],[157,50,5],
[157,51,2],[157,53,1],[157,56,12],[157,60,5],[157,62,3],[157,63,1],
[157,66,51],[157,67,1],[157,68,1],[157,70,2],[157,72,2],[157,74,1],
[157,75,4],[157,76,3],[158,20,2],[158,27,2],[158,32,2],[158,36,1],
[159,28,1],[159,44,1],[159,66,3],[160,3,1],[160,23,1],[160,26,1],
[160,35,3],[161,22,2],[162,48,12],[163,35,2],[164,0,2],[164,1,1],
[164,2,1],[164,3,1],[164,5,1],[164,20,3],[164,23,4],[164,27,1],
[164,28,2],[164,30,10],[164,31,1],[164,32,4],[164,34,2],[164,36,4],

[165,1,1],[165,4,1],[165,28,3],[166,1,2],[166,7,1],[166,9,2],
[166,10,4],[166,11,3],[166,12,3],[166,13,3],[166,14,2],[166,15,2],
[166,16,2],[166,17,1],[166,18,1],[166,19,2],[166,30,1],[166,34,1],
[166,37,1],[166,39,1],[166,41,1],[166,45,2],[166,49,1],[166,54,1],
[166,57,3],[166,58,1],[166,59,2],[166,60,1],[166,64,1],[166,65,6],
[166,67,2],[166,71,2],[166,73,2],[166,77,1],[167,1,3],[168,20,1],
[168,23,82],[168,25,1],[168,31,1],[169,27,3],[170,2,1],[170,3,1],
[170,4,1],[170,5,2],[170,6,15],[170,22,12],[170,23,6],[170,24,12],
[170,25,2],[170,26,2],[170,27,31],[170,28,7],[170,32,3],[170,33,2],
[170,35,20],[170,38,2],[170,40,1],[170,43,1],[170,44,2],[170,50,1],
[170,56,5],[170,62,3],[170,66,1],[170,68,1],[170,69,1],[170,70,1],
[170,72,1],[170,74,4],[170,75,2],[170,76,1],[171,8,1],[171,28,1],
[172,1,2],[172,2,4],[172,3,14],[172,4,447],[172,5,34],[172,6,2],
[172,13,1],[172,19,1],[172,21,11],[172,22,20],[172,23,20],[172,24,17],
[172,25,20],[172,26,14],[172,27,20],[172,28,100],[172,31,14],
[172,32,16],[172,33,12],[172,35,10],[172,38,361],[172,40,15],
[172,42,13],[172,43,6],[172,44,1450],[172,45,1],[172,47,13],
[172,48,5],[172,50,491],[172,51,6],[172,52,3],[172,55,1],[172,56,11],
[172,60,1],[172,61,3],[172,62,12],[172,63,15],[172,65,1],
[172,66,1465],[172,67,1],[172,68,8],[172,69,8],[172,70,23],[172,71,1],
[172,72,9],[172,74,25],[172,75,17],[172,76,21],[172,77,1],[172,79,1],
[173,2,1],[173,3,2],[173,5,3],[173,8,1],[173,21,1],[173,22,19],
[173,23,6],[173,24,6],[173,25,7],[173,26,14],[173,27,71],[173,28,22],
[173,31,3],[173,32,10],[173,33,3],[173,35,15],[173,42,2],[173,43,13],
[173,47,1],[173,48,1],[173,52,2],[173,59,1],[173,70,1],[173,74,1],
[174,12,2],[174,13,1],[174,19,1],[174,23,1],[174,37,1],[174,77,1],
[175,47,1],[175,60,1],[176,22,3],[176,35,2],[176,69,1],[177,7,1],
[177,60,1],[178,22,1],[178,33,3],[178,35,1],[178,52,7],[178,63,1],
[178,68,2],[178,75,1],[179,1,9],[179,39,1],[180,13,1],[180,22,1],
[180,25,1],[180,30,5],[180,33,1],[180,52,1],[180,56,1],[180,69,1],
[181,22,4],[181,24,1],[181,26,1],[181,28,1],[182,1,1],[182,22,1],
[182,33,1],[183,32,1],[183,69,1],[184,3,1],[184,28,1],[185,4,1],
[185,15,1],[185,24,1],[185,28,4],[185,31,1],[185,44,2],[185,48,1],
[185,50,1],[185,51,2],[185,52,1],[185,69,1],[186,5,2],[187,2,1],
[187,23,1],[187,28,2],[187,30,1],[187,51,1],[188,25,1],[188,35,1],
[189,28,1],[189,33,1],[190,35,1],[190,56,1],[190,68,3],[190,69,1],
[191,31,9],[191,33,1],[192,1,1],[192,4,12],[192,5,4],[192,17,3],
[192,19,3],[192,23,1],[192,24,1],[192,25,2],[192,26,1],[192,27,1],
[192,28,1],[192,32,1],[192,37,1],[192,38,18],[192,40,1],[192,44,36],
[192,50,6],[192,53,3],[192,56,1],[192,59,1],[192,62,1],[192,63,1],
[192,65,1],[192,66,32],[192,70,1],[192,74,2],[193,0,2],[194,3,21],
[195,2,2],[195,26,1],[195,27,1],[195,30,1],[195,42,1],[196,15,1],
[196,55,1],[197,5,1],[197,22,1],[197,24,4],[197,26,1],[197,27,1],
[198,28,1],[198,42,1],[199,2,3],[200,10,1],[200,15,1],[200,53,1],
[200,55,1],[201,35,2],[202,42,2],[203,37,3],[204,28,1],[204,47,1],
[205,27,2],[206,21,1],[206,31,1],[207,2,3],[207,5,1],[207,23,2],
[207,24,1],[207,26,1],[207,27,1],[207,28,1],[207,31,1],[207,32,1],
[207,39,1],[207,42,8],[207,43,1],[207,62,1],[207,72,1],[208,15,1],
[208,53,1],[209,22,1],[209,23,1],[209,27,1],[209,28,3],[209,29,1],
[209,31,8],[209,33,3],[209,43,1],[209,59,2],[210,20,2],[211,63,4],

[211,74,5],[211,75,3],[211,76,5],[212,7,1],[212,27,1],[213,26,1],
[213,33,1],[214,2,10],[215,1,1],[215,9,1],[216,21,2],[217,0,1],
[217,1,12],[217,7,2],[217,8,5],[217,9,7],[217,10,14],[217,11,6],
[217,12,16],[217,13,3],[217,14,6],[217,15,10],[217,16,9],[217,17,7],
[217,18,4],[217,19,12],[217,20,2],[217,22,1],[217,23,2],[217,28,4],
[217,29,8],[217,30,7],[217,31,1],[217,34,6],[217,36,4],[217,37,7],
[217,39,3],[217,40,1],[217,41,9],[217,45,10],[217,46,4],[217,49,2],
[217,51,2],[217,53,8],[217,54,6],[217,55,4],[217,57,4],[217,58,8],
[217,59,9],[217,60,1],[217,64,2],[217,65,10],[217,67,7],[217,71,1],
[217,73,6],[217,75,1],[217,77,7],[217,78,2],[218,74,2],[219,17,2],
[220,10,1],[220,16,1],[221,26,2],[221,27,2],[221,28,4],[221,32,1],
[221,39,3],[221,62,1],[222,56,3],[223,28,2],[224,24,1],[224,30,1],
[224,47,3],[225,2,2],[225,27,1],[225,28,1],[225,33,1],[225,35,1],
[225,42,1],[226,26,1],[226,27,6],[226,43,4],[227,23,2],[227,27,2],
[227,62,1],[228,5,1],[228,70,1],[229,8,1],[229,13,2],[229,22,2],
[229,25,8],[229,27,4],[229,28,2],[229,30,13],[229,33,39],[229,35,23],
[229,38,2],[229,39,1],[229,41,1],[229,52,4],[229,58,1],[229,62,1],
[229,78,1],[230,2,1],[230,4,1],[230,50,3],[231,0,1],[231,1,4],
[231,4,1],[231,9,1],[231,10,6],[231,12,3],[231,14,1],[231,15,1],
[231,16,2],[231,18,2],[231,19,3],[231,28,2],[231,30,3],[231,33,1],
[231,34,1],[231,37,1],[231,41,5],[231,45,1],[231,49,1],[231,53,3],
[231,54,1],[231,57,1],[231,58,2],[231,59,1],[231,63,1],[231,64,2],
[231,65,6],[231,67,2],[231,71,1],[231,73,4],[231,74,1],[231,77,2],
[232,9,1],[232,12,1],[232,16,1],[232,67,1],[233,13,1],[233,29,1],
[233,45,3],[234,60,1],[234,72,1],[235,39,2],[235,53,1],[235,55,2],
[236,3,4],[236,22,1],[236,23,3],[236,24,3],[236,27,1],[236,31,1],
[236,32,2],[236,33,1],[236,35,2],[236,40,2],[236,47,1],[236,51,6],
[236,56,6],[236,61,1],[236,66,1],[236,68,2],[236,70,2],[237,24,3],
[238,32,2],[239,28,4],[240,31,3],[241,2,1],[241,4,1],[241,5,4],
[241,27,1],[241,28,4],[241,61,1],[241,63,1],[242,22,1],[242,38,9],
[243,24,1],[243,50,1],[243,74,1],[243,75,1],[243,76,1],[244,28,1],
[244,44,1],[245,28,1],[245,35,5],[245,44,1],[246,6,1],[246,22,1],
[246,23,1],[246,24,1],[246,26,1],[246,27,2],[246,32,1],[246,35,1],
[246,42,1],[246,43,1],[247,1,4],[247,2,4],[247,3,1],[247,5,1],
[247,10,2],[247,16,1],[247,21,1],[247,27,2],[247,29,1],[247,30,2],
[247,31,1],[247,34,5],[247,35,1],[247,36,1],[247,39,1],[247,50,1],
[247,53,1],[247,62,1],[248,28,6],[248,35,2],[248,52,1],[248,59,1],
[249,9,1],[249,10,1],[249,18,1],[250,21,1],[250,25,2],[250,30,1],
[250,42,2],[250,43,1],[251,2,2],[252,25,3],[252,30,1],[252,31,1],
[253,3,1],[253,23,2],[253,24,1],[253,31,1],[253,32,1],[253,33,1],
[253,35,1],[253,40,1],[253,42,3],[253,43,1],[253,47,1],[253,51,1],
[253,55,1],[253,56,1],[254,27,1],[254,28,1],[254,33,1],[255,20,1],
[255,24,1],[255,28,1],[256,17,1],[256,27,1],[256,59,1],[257,1,1],
[257,3,1],[257,5,1],[257,6,2],[257,27,1],[257,68,2],[257,69,1],
[258,4,3],[258,15,1],[258,17,3],[258,27,2],[258,41,1],[258,50,2],
[258,53,7],[258,55,3],[259,2,1],[259,38,1],[259,47,1],[260,28,1],
[260,33,4],[261,13,1],[261,18,1],[262,17,1],[262,76,1],[263,19,1],
[263,29,1],[263,54,1],[263,55,1],[263,58,1],[263,65,1],[263,77,1],
[264,15,3],[264,49,1],[265,12,1],[265,37,1],[265,64,2],[266,4,1],
[266,23,3],[266,24,3],[266,26,8],[266,27,9],[266,31,1],[266,35,8],

[266,43,6],[266,50,2],[266,62,1],[266,63,1],[267,63,1],[267,74,2],
[267,76,1],[268,4,2],[268,15,7],[268,17,9],[268,19,1],[268,53,16],
[268,54,1],[268,55,4],[268,62,1],[268,66,1],[269,0,1],[269,1,13],
[269,3,4],[269,4,1],[269,7,1],[269,8,2],[269,12,1],[269,13,1],
[269,17,1],[269,19,2],[269,20,6],[269,21,1],[269,22,4],[269,23,13],
[269,24,22],[269,25,5],[269,26,1],[269,28,7],[269,29,2],[269,30,4],
[269,31,6],[269,32,5],[269,33,3],[269,34,5],[269,35,1],[269,36,3],
[269,37,1],[269,39,5],[269,42,1],[269,43,6],[269,47,1],[269,48,2],
[269,53,1],[269,73,1],[270,23,7],[270,24,1],[270,26,1],[270,31,2],
[271,26,1],[271,27,1],[272,28,1],[272,43,6],[273,1,3],[274,15,1],
[274,21,1],[274,31,1],[275,2,2],[276,0,1],[276,1,7],[276,2,11],
[276,3,51],[276,4,19],[276,5,13],[276,6,6],[276,7,1],[276,8,2],
[276,9,3],[276,12,1],[276,16,1],[276,17,46],[276,21,14],[276,22,82],
[276,23,111],[276,24,205],[276,25,102],[276,26,20],[276,27,115],
[276,28,71],[276,30,24],[276,31,27],[276,32,57],[276,33,8],[276,34,3],
[276,35,99],[276,38,22],[276,39,14],[276,42,6],[276,43,11],[276,44,1],
[276,45,2],[276,47,12],[276,48,31],[276,50,57],[276,51,1],[276,52,13],
[276,53,63],[276,54,1],[276,55,4],[276,56,11],[276,59,6],[276,62,19],
[276,70,1],[276,74,1],[276,79,1],[277,21,2],[277,26,1],[277,33,3],
[277,43,1],[278,20,2],[278,38,1],[278,53,1],[278,55,1],[278,58,1],
[279,27,1],[279,28,1],[280,22,2],[281,2,1],[281,4,1],[281,5,1],
[281,23,1],[281,26,1],[281,50,1],[281,51,2],[281,62,1],[281,68,2],
[281,72,1],[282,24,1],[282,28,1],[282,31,1],[283,1,6],[283,7,1],
[283,8,1],[283,12,2],[283,13,7],[283,20,1],[283,29,2],[283,39,4],
[284,2,1],[284,28,1],[284,35,1],[285,30,1],[285,35,4],[285,44,1],
[285,59,1],[286,22,1],[286,24,1],[287,3,1],[287,8,1],[287,31,1],
[287,64,1],[288,2,12],[288,23,1],[289,25,1],[289,27,1],[290,3,1],
[290,4,1],[290,15,1],[290,38,1],[290,42,1],[290,44,1],[290,53,1],
[290,55,1],[290,63,1],[290,66,3],[291,12,2],[292,1,1],[292,24,1],
[292,41,1],[293,2,1],[293,3,1],[293,24,2],[293,27,1],[293,31,2],
[293,32,3],[293,35,1],[293,42,1],[293,43,1],[294,28,1],[294,72,5],
[295,28,1],[295,56,1],[295,68,1],[296,23,8],[296,26,10],[296,33,1],
[296,43,1],[296,48,1],[296,66,1],[296,68,1],[297,5,1],[297,23,1],
[297,28,1],[298,26,5],[298,27,22],[298,47,2],[299,63,8],[299,74,5],
[299,75,3],[299,76,6],[300,12,1],[300,17,1],[300,34,2],[300,35,1],
[301,0,1],[301,1,1],[301,24,3],[301,28,1],[302,27,1],[302,28,1],
[303,7,1],[303,10,1],[303,12,2],[303,13,8],[303,29,1],[303,30,1],
[303,34,1],[303,65,1],[304,1,1],[304,23,1],[305,13,1],[305,25,1],
[305,35,1],[305,42,1],[306,28,1],[306,30,1],[307,27,2],[308,20,3],
[308,23,2],[308,24,2],[308,27,1],[308,31,2],[309,2,5],[309,22,2],
[309,23,3],[309,27,1],[309,30,1],[309,42,1],[309,47,1],[309,50,1],
[309,66,2],[309,68,1],[309,70,1],[310,5,1],[310,51,1],[311,40,1],
[311,50,1],[312,22,2],[313,12,1],[313,18,1],[313,30,6],[313,36,7],
[313,65,1],[314,2,5],[314,21,1],[315,9,1],[315,13,1],[315,30,1],
[315,39,10],[316,27,3],[317,15,1],[317,22,1],[317,24,1],[317,33,1],
[317,42,1],[318,22,1],[318,28,1],[318,30,1],[319,28,2],[320,46,1],
[320,78,1],[321,23,1],[321,26,1],[321,32,1],[322,20,2],[322,21,1],
[322,22,1],[322,23,1],[322,26,1],[322,27,1],[322,28,1],[322,30,1],
[322,35,1],[322,39,1],[322,42,1],[322,43,1],[322,52,1],[322,62,1],
[322,66,1],[323,2,3],[323,28,2],[324,44,1],[324,50,1],[324,66,2],

[325,49,1],[325,68,1],[326,1,3],[326,12,2],[326,13,6],[326,16,1],
[326,20,5],[326,23,5],[326,24,4],[326,26,1],[326,27,1],[326,28,2],
[326,31,1],[326,32,1],[326,34,2],[326,35,4],[326,37,1],[326,38,1],
[326,39,6],[326,46,1],[326,59,1],[326,62,6],[326,65,1],[326,70,2],
[326,73,1],[327,21,2],[327,27,1],[327,43,1],[328,3,1],[328,24,2],
[328,25,1],[328,26,1],[328,32,1],[328,33,2],[328,43,1],[328,48,1],
[328,56,1],[329,23,1],[329,24,2],[329,27,7],[329,31,2],[329,39,7],
[329,42,2],[329,47,1],[330,2,4],[330,10,1],[330,20,1],[330,31,1],
[330,73,1],[331,3,1],[331,5,2],[331,6,2],[331,22,2],[331,27,7],
[331,32,1],[331,33,2],[331,35,3],[331,42,1],[331,43,2],[331,44,2],
[331,56,3],[331,66,1],[331,68,1],[332,3,1],[332,25,1],[332,35,1],
[332,75,1],[333,31,43],[334,28,1],[334,38,1],[335,22,2],[335,35,1],
[336,1,8],[336,2,5],[336,3,9],[336,4,4],[336,5,1],[336,6,1],[336,8,3],
[336,12,1],[336,13,17],[336,15,1],[336,17,2],[336,20,4],[336,21,1],
[336,22,4],[336,23,2],[336,24,5],[336,25,4],[336,26,1],[336,27,57],
[336,28,18],[336,30,1],[336,31,4],[336,32,10],[336,33,7],[336,34,2],
[336,35,19],[336,36,3],[336,39,23],[336,42,16],[336,43,1],[336,44,1],
[336,45,1],[336,47,15],[336,48,8],[336,50,2],[336,51,6],[336,52,1],
[336,53,2],[336,56,1],[336,57,2],[336,59,9],[336,62,1],[336,63,1],
[336,66,1],[336,69,1],[336,78,1],[336,79,1],[337,56,2],[338,24,1],
[338,31,1],[338,34,1],[338,47,1],[339,9,1],[339,28,1],[339,47,3],
[340,4,1],[340,23,1],[340,24,1],[340,50,1],[341,21,1],[341,42,1],
[341,43,2],[341,48,1],[342,0,1],[342,1,1],[342,7,1],[342,9,1],
[342,10,1],[342,19,2],[342,20,1],[342,24,1],[342,31,1],[342,37,1],
[342,41,1],[342,53,1],[342,65,1],[343,26,1],[343,43,1],[343,60,1],
[344,9,3],[344,10,1],[344,12,2],[344,13,1],[344,15,1],[344,29,1],
[344,37,1],[344,41,1],[344,49,1],[344,65,1],[344,71,1],[345,8,1],
[345,19,1],[346,5,2],[346,10,1],[346,22,7],[346,24,1],[346,25,3],
[346,27,3],[346,28,12],[346,30,9],[346,32,10],[346,33,45],[346,35,40],
[346,38,4],[346,50,2],[346,52,54],[346,70,1],[346,76,1],[347,2,3],
[347,3,1],[347,9,2],[347,10,1],[347,13,2],[347,15,1],[347,20,4],
[347,23,8],[347,24,3],[347,27,5],[347,28,5],[347,31,6],[347,33,1],
[347,34,1],[347,35,1],[347,37,1],[347,39,3],[347,42,2],[347,43,1],
[347,45,1],[347,47,1],[347,59,2],[347,77,1],[348,27,2],[349,35,3],
[350,4,2],[350,53,1],[350,62,5],[351,28,1],[351,76,1],[352,21,1],
[352,60,1],[353,5,1],[353,21,1],[354,44,2],[355,2,6],[355,3,1],
[355,26,1],[355,28,1],[355,43,1],[356,8,1],[356,26,1],[356,34,1],
[356,59,1],[357,1,1],[357,2,5],[357,3,8],[357,4,2],[357,5,3],
[357,8,4],[357,9,2],[357,10,3],[357,12,1],[357,13,1],[357,16,1],
[357,19,1],[357,20,1],[357,21,2],[357,22,6],[357,23,9],[357,24,9],
[357,25,15],[357,26,10],[357,27,20],[357,28,23],[357,29,1],
[357,30,82],[357,31,17],[357,32,37],[357,33,76],[357,35,58],
[357,37,1],[357,38,5],[357,39,2],[357,40,5],[357,41,1],[357,42,6],
[357,43,8],[357,44,2],[357,45,1],[357,46,4],[357,47,2],[357,48,2],
[357,49,1],[357,50,1],[357,52,111],[357,53,2],[357,54,1],[357,55,2],
[357,56,2],[357,57,1],[357,58,2],[357,61,1],[357,62,4],[357,65,1],
[357,66,2],[357,67,1],[357,69,3],[357,70,4],[357,71,1],[357,72,1],
[357,74,2],[357,76,2],[357,77,2],[357,79,1],[358,2,2],[359,3,1],
[359,21,1],[359,23,1],[359,27,1],[359,32,10],[359,38,1],[359,44,1],
[359,47,1],[359,48,1],[359,66,1],[359,74,1],[360,26,1],[360,27,1],

[360,47,1],[360,60,1],[361,27,1],[361,37,1],[362,0,1],[362,7,1],
[362,8,1],[362,12,1],[362,13,1],[362,29,1],[362,31,1],[362,41,1],
[362,77,1],[363,66,2],[364,2,1],[364,3,1],[364,5,1],[364,23,1],
[365,9,1],[365,20,1],[365,22,1],[365,28,4],[365,33,1],[365,42,1],
[365,55,1],[365,59,1],[366,5,1],[366,32,1],[367,5,5],[367,43,1],
[367,52,1],[368,5,1],[368,10,1],[368,15,1],[368,43,1],[368,53,1],
[369,32,5],[370,3,1],[370,24,2],[370,34,1],[370,43,1],[371,2,1],
[371,23,7],[371,27,6],[371,35,1],[371,37,2],[371,43,1],[371,47,1],
[371,56,1],[371,70,1],[371,78,2],[372,2,3],[372,27,1],[373,24,1],
[373,28,1],[374,4,8],[374,5,1],[374,15,29],[374,17,3],[374,21,1],
[374,22,1],[374,25,1],[374,27,1],[374,28,2],[374,44,9],[374,47,1],
[374,50,9],[374,53,6],[374,55,14],[374,66,7],[374,74,1],[374,76,1],
[375,9,1],[375,37,1],[376,31,1],[376,44,1],[376,47,1],[377,28,2],
[377,76,1],[378,3,1],[378,5,1],[378,38,1],[378,66,1],[379,4,1],
[379,63,3],[379,74,1],[379,75,1],[379,76,3],[380,27,1],[380,28,1],
[381,28,1],[381,40,1],[382,1,2],[382,35,1],[382,71,1],[383,26,1],
[383,27,1],[383,31,2],[384,2,1],[384,3,3],[384,6,1],[384,20,1],
[384,21,1],[384,22,8],[384,23,5],[384,24,11],[384,25,1],[384,26,8],
[384,27,3],[384,28,8],[384,31,1],[384,32,10],[384,34,1],[384,35,1],
[384,37,3],[384,38,1],[384,39,1],[384,43,2],[384,44,1],[384,56,1],
[384,59,1],[384,62,1],[384,72,1],[385,21,2],[385,31,2],[385,42,7],
[386,28,1],[386,63,3],[386,74,2],[386,75,1],[386,76,6],[387,68,1],
[387,69,1],[388,51,3],[389,2,3],[390,2,1],[390,47,1],[391,2,1],
[391,22,4],[391,24,6],[391,33,3],[392,0,2],[392,1,4],[392,7,1],
[392,8,1],[392,9,8],[392,10,6],[392,11,2],[392,12,2],[392,13,2],
[392,14,1],[392,15,4],[392,16,7],[392,17,4],[392,18,2],[392,19,4],
[392,20,1],[392,29,1],[392,30,3],[392,34,1],[392,36,3],[392,37,1],
[392,41,5],[392,45,1],[392,46,3],[392,49,2],[392,53,3],[392,54,2],
[392,55,4],[392,57,1],[392,58,2],[392,59,1],[392,60,2],[392,64,1],
[392,65,4],[392,67,2],[392,73,1],[392,78,1],[393,23,1],[393,28,5],
[394,2,1],[394,59,2],[395,28,1],[395,66,1],[396,3,1],[396,6,1],
[396,24,1],[396,27,2],[396,28,6],[396,42,1],[396,68,1],[396,69,1],
[396,70,2],[396,75,1],[396,76,1],[397,0,1],[397,1,13],[397,7,6],
[397,8,7],[397,9,43],[397,10,52],[397,11,25],[397,12,9],[397,13,8],
[397,14,14],[397,15,7],[397,16,32],[397,17,7],[397,18,16],[397,19,40],
[397,20,14],[397,29,5],[397,30,6],[397,34,19],[397,36,13],[397,37,20],
[397,39,7],[397,41,30],[397,45,32],[397,46,16],[397,49,10],[397,53,3],
[397,54,10],[397,55,11],[397,57,24],[397,58,20],[397,59,20],
[397,60,5],[397,64,4],[397,65,37],[397,67,23],[397,71,12],[397,73,20],
[397,77,16],[397,78,3],[398,28,2],[398,41,1],[399,4,1],[399,16,1],
[399,17,1],[399,20,3],[399,22,2],[399,23,1],[399,25,1],[399,26,1],
[399,27,3],[399,28,1],[399,32,1],[399,33,1],[399,35,1],[399,51,1],
[399,65,1],[399,67,1],[399,74,1],[400,22,1],[400,23,2],[400,28,4],
[401,4,1],[401,28,1],[401,35,4],[401,44,1],[402,2,3],[402,3,1],
[402,23,2],[402,24,1],[402,26,3],[402,27,3],[402,28,3],[402,33,1],
[402,35,5],[402,43,1],[402,65,1],[402,75,1],[403,2,2],[404,1,43],
[404,2,44],[404,3,1],[404,5,2],[404,7,4],[404,8,3],[404,9,2],
[404,12,3],[404,13,29],[404,15,1],[404,17,2],[404,20,2],[404,21,9],
[404,25,3],[404,26,3],[404,27,6],[404,28,2],[404,29,4],[404,30,14],
[404,32,1],[404,33,4],[404,34,3],[404,35,3],[404,36,3],[404,39,1],

[404,42,1],[404,43,10],[404,44,1],[404,47,7],[404,48,10],[404,52,1],
[404,53,3],[404,55,1],[404,59,1],[404,66,1],[404,69,2],[404,70,1],
[404,76,1],[405,2,3],[405,21,2],[405,31,1],[405,37,1],[405,40,1],
[405,42,2],[405,77,1],[406,10,1],[406,67,1],[407,17,1],[407,28,7],
[407,44,1],[407,50,1],[407,55,1],[408,27,1],[408,70,1],[409,2,1],
[409,28,1],[410,50,1],[410,62,2],[411,27,1],[411,28,1],[412,21,8],
[412,26,3],[412,28,1],[412,31,6],[412,32,1],[412,33,1],[412,42,2],
[412,44,2],[412,51,1],[413,2,2],[414,28,3],[415,1,3],[415,9,1],
[415,10,2],[415,12,2],[415,17,1],[415,18,1],[415,29,1],[415,30,1],
[415,36,1],[415,41,1],[415,45,1],[415,59,1],[416,9,10],[416,28,4],
[416,53,1],[416,55,2],[416,66,1],[417,6,1],[417,32,1],[417,68,1],
[418,1,2],[418,13,2],[418,29,1],[418,39,1],[419,30,5],[420,23,1],
[420,28,1],[421,7,1],[421,18,3],[421,24,1],[421,25,3],[421,26,1],
[421,27,2],[421,32,7],[421,38,1],[421,43,5],[421,59,6],[421,62,4],
[421,70,2],[421,76,1],[422,26,1],[422,50,1],[423,24,3],[423,27,2],
[423,35,1],[424,9,1],[424,10,1],[424,15,10],[424,17,1],[424,19,1],
[424,22,1],[424,54,1],[424,55,5],[424,58,1],[424,59,1],[424,61,1],
[424,74,1],[425,5,1],[425,27,1],[426,3,2],[426,23,1],[426,62,1],
[427,24,1],[427,62,1],[428,25,1],[428,39,1],[429,3,4],[429,6,2],
[429,35,1],[429,39,1],[429,46,1],[429,64,1],[430,19,1],[430,37,1],
[430,46,1],[430,53,1],[430,57,1],[431,28,1],[431,32,2],[431,33,2],
[431,42,1],[431,69,1],[432,1,1],[432,3,1],[433,1,2],[434,50,2],
[435,26,1],[435,27,1],[435,48,1],[436,2,20],[436,21,1],[436,74,1],
[437,2,1],[437,3,6],[437,4,67],[437,5,8],[437,6,1],[437,15,1],
[437,21,1],[437,22,5],[437,23,4],[437,24,3],[437,25,5],[437,26,8],
[437,27,7],[437,28,62],[437,31,23],[437,32,2],[437,33,3],[437,35,5],
[437,38,123],[437,40,4],[437,42,2],[437,43,1],[437,44,929],[437,47,2],
[437,48,3],[437,50,92],[437,51,2],[437,52,1],[437,53,1],[437,56,4],
[437,61,3],[437,62,5],[437,63,2],[437,66,949],[437,68,3],[437,69,2],
[437,70,3],[437,72,1],[437,75,4],[437,76,10],[437,79,2],[438,74,1],
[438,75,1],[438,76,1],[439,21,1],[439,24,1],[440,23,1],[440,70,1],
[441,32,2],[442,30,1],[442,53,1],[443,13,2],[444,3,1],[444,24,1],
[445,41,1],[445,42,1],[445,56,7],[445,70,1],[445,76,1],[446,3,1],
[446,4,5],[446,5,1],[446,10,1],[446,15,6],[446,19,1],[446,22,1],
[446,28,4],[446,50,3],[446,55,3],[446,58,1],[446,65,1],[447,0,1],
[447,2,1],[447,3,1],[447,28,3],[447,38,1],[447,50,1],[448,28,2],
[448,42,22],[449,23,1],[449,28,1],[449,63,2],[450,71,2],[451,2,2],
[451,5,2],[451,37,1],[451,42,1],[452,22,1],[452,23,3],[452,28,1],
[452,35,1],[453,5,2],[453,13,1],[453,16,1],[453,17,18],[453,19,2],
[453,22,2],[453,23,6],[453,24,1],[453,25,5],[453,26,1],[453,27,2],
[453,28,28],[453,31,1],[453,33,2],[453,35,2],[453,40,8],[453,44,3],
[453,45,1],[453,47,2],[453,48,1],[453,50,3],[453,52,1],[453,53,24],
[453,55,1],[453,56,1],[453,63,4],[453,65,2],[453,66,4],[453,68,4],
[453,69,1],[453,70,4],[453,72,2],[453,74,3],[453,75,2],[453,76,7],
[454,35,1],[454,62,2],[455,13,1],[455,28,2],[455,70,1],[456,28,2],
[457,1,1],[457,8,2],[457,20,7],[457,24,1],[457,25,1],[457,27,6],
[457,28,1],[457,35,1],[457,66,1],[458,8,1],[458,9,3],[458,28,1],
[459,25,1],[459,31,1],[460,4,9],[460,5,2],[460,6,6],[460,7,1],
[460,8,1],[460,10,1],[460,12,1],[460,13,1],[460,15,11],[460,17,3],
[460,18,1],[460,20,1],[460,28,1],[460,32,1],[460,38,4],[460,39,1],

[460,44,7],[460,50,4],[460,53,1],[460,55,5],[460,58,1],[460,66,7],
[461,23,3],[461,52,1],[462,27,1],[462,32,1],[463,31,3],[464,1,1],
[464,3,1],[464,10,1],[464,37,1],[465,3,1],[465,26,1],[465,27,1],
[465,31,1],[466,30,3],[467,20,4],[468,3,1],[468,6,1],[468,26,2],
[468,28,1],[468,60,1],[469,22,1],[469,27,4],[469,32,1],[469,35,1],
[470,27,1],[470,38,1],[471,6,1],[471,42,1],[471,43,1],[472,27,1],
[472,31,1],[473,5,2],[473,21,1],[474,1,1],[474,2,5],[474,3,1],
[474,5,1],[474,6,1],[474,22,1],[474,23,2],[474,26,1],[474,27,6],
[474,30,1],[474,33,1],[474,42,1],[474,43,1],[474,46,1],[474,47,4],
[474,56,5],[474,59,1],[474,60,3],[474,62,1],[474,65,1],[474,78,1],
[475,2,3],[475,22,1],[475,26,1],[475,28,1],[475,31,6],[475,47,5],
[475,63,1],[476,2,3],[476,3,4],[476,5,2],[476,6,1],[476,22,2],
[476,23,1],[476,24,10],[476,25,2],[476,26,1],[476,27,1],[476,28,2],
[476,31,1],[476,32,1],[476,33,2],[476,35,1],[476,42,2],[476,43,3],
[476,56,1],[476,70,1],[477,27,1],[477,28,1],[477,56,1],[478,22,1],
[478,28,1],[478,52,1],[479,24,1],[479,27,2],[479,35,1],[479,40,2],
[480,27,1],[480,35,1],[481,28,1],[481,68,1],[482,28,1],[482,44,1],
[483,1,5],[483,2,20],[483,3,8],[483,5,4],[483,6,1],[483,8,4],
[483,9,2],[483,10,2],[483,13,29],[483,15,1],[483,17,1],[483,18,1],
[483,19,2],[483,20,29],[483,21,11],[483,22,7],[483,23,17],[483,24,6],
[483,25,9],[483,26,9],[483,27,14],[483,28,5],[483,29,1],[483,30,9],
[483,31,18],[483,32,6],[483,33,3],[483,34,1],[483,35,7],[483,37,2],
[483,38,1],[483,39,9],[483,41,1],[483,42,8],[483,43,8],[483,44,1],
[483,47,7],[483,48,5],[483,49,1],[483,56,2],[483,57,1],[483,58,1],
[483,59,8],[483,62,1],[483,66,1],[483,72,1],[483,78,2],[484,28,1],
[484,33,1],[485,2,2],[485,59,1],[486,28,1],[486,57,1],[487,28,1],
[487,60,1],[487,74,1],[488,2,2],[488,5,1],[488,23,2],[488,24,1],
[488,26,1],[488,31,1],[488,35,1],[488,42,2],[488,46,1],[488,47,1],
[489,3,1],[489,6,1],[489,24,2],[489,28,2],[489,56,1],[490,4,2],
[490,17,1],[490,24,6],[490,25,1],[490,26,2],[490,27,2],[490,28,2],
[490,31,2],[490,32,2],[490,35,2],[490,47,1],[490,48,1],[490,53,1],
[490,56,6],[490,62,1],[491,3,1],[491,12,1],[491,13,2],[491,20,1],
[491,23,2],[491,26,1],[491,27,1],[491,33,1],[491,42,1],[492,6,2],
[492,27,1],[492,32,1],[493,32,8],[494,33,2],[494,62,1],[495,63,2],
[496,25,2],[497,56,1],[497,62,4],[498,23,1],[498,24,1],[498,27,3],
[499,62,1],[499,69,1],[499,76,1],[500,28,2],[500,74,1],[501,2,1],
[501,23,1],[501,24,3],[501,32,11],[501,37,4],[501,62,1],[501,73,1],
[502,1,1],[502,8,1],[502,15,2],[502,17,1],[502,53,3],[502,55,1],
[502,59,1],[503,4,1],[503,15,1],[504,16,1],[504,65,1],[505,1,1],
[505,12,1],[505,15,2],[505,17,2],[505,45,1],[505,55,1],[506,5,3],
[506,23,1],[506,25,1],[506,26,1],[507,4,1],[507,38,1],[507,53,1],
[508,47,1],[508,51,2],[508,62,1],[509,5,1],[509,6,1],[509,8,1],
[509,22,4],[509,23,1],[509,26,1],[509,28,1],[509,32,2],[509,35,1],
[509,70,1],[510,0,3],[510,57,1],[511,54,1],[511,67,1],[512,9,1],
[512,22,1],[512,23,1],[512,24,2],[512,26,1],[512,27,10],[512,32,3],
[512,33,2],[512,34,1],[512,35,3],[512,62,1],[513,50,2],[513,69,1],
[514,43,2],[515,42,1],[515,50,1],[516,28,1],[516,75,1],[517,2,2],
[517,28,1],[518,28,3],[518,74,1],[519,24,1],[519,27,1],[520,10,1],
[520,22,2],[520,24,1],[520,25,5],[520,26,1],[520,28,13],[520,30,6],
[520,31,1],[520,32,7],[520,33,48],[520,35,44],[520,40,1],[520,42,1],

[520,44,1],[520,52,79],[520,53,1],[520,62,1],[520,63,1],[520,66,1],
[520,68,1],[521,21,1],[521,23,4],[521,24,2],[521,26,1],[521,35,4],
[522,1,2],[522,5,5],[522,6,1],[522,10,1],[522,21,1],[522,33,1],
[522,42,2],[522,46,1],[522,47,2],[522,78,1],[523,56,4],[524,2,15],
[524,9,1],[524,10,3],[524,13,6],[524,15,1],[524,16,1],[524,17,5],
[524,18,1],[524,20,8],[524,21,1],[524,23,1],[524,25,3],[524,26,1],
[524,29,2],[524,30,1],[524,31,1],[524,33,2],[524,34,38],[524,35,1],
[524,37,1],[524,39,9],[524,42,2],[524,43,1],[524,44,1],[524,53,1],
[524,54,1],[524,60,1],[524,62,4],[524,70,1],[524,76,1],[524,77,2],
[525,2,8],[525,3,3],[525,19,1],[525,20,3],[525,22,1],[525,23,2],
[525,24,2],[525,27,4],[525,28,1],[525,30,2],[525,31,2],[525,47,1],
[525,60,1],[525,67,1],[526,6,3],[526,21,1],[526,27,1],[526,35,15],
[526,66,1],[527,28,6],[528,23,2],[529,3,1],[529,40,1],[530,1,1],
[530,12,1],[531,6,1],[531,35,1],[532,5,1],[532,25,6],[533,28,1],
[533,40,1],[534,1,1],[534,11,1],[534,12,84],[534,41,1],[534,67,1],
[534,73,1],[535,0,46],[535,1,4384],[535,2,32],[535,3,2],[535,4,3],
[535,5,4],[535,6,5],[535,7,293],[535,8,165],[535,9,17],[535,10,24],
[535,11,7],[535,12,875],[535,13,971],[535,14,3],[535,15,73],
[535,16,26],[535,17,168],[535,18,4],[535,19,7],[535,20,124],
[535,21,3],[535,22,1],[535,23,11],[535,24,33],[535,25,45],[535,26,5],
[535,27,3],[535,28,1],[535,29,1927],[535,30,60],[535,31,10],
[535,32,2],[535,33,35],[535,34,191],[535,35,2],[535,36,90],
[535,37,35],[535,39,2004],[535,40,4],[535,41,3],[535,42,12],
[535,43,27],[535,45,11],[535,46,5],[535,47,9],[535,48,4],[535,49,3],
[535,50,3],[535,51,5],[535,52,1],[535,53,209],[535,54,1],[535,55,91],
[535,57,4],[535,58,2],[535,59,41],[535,60,1],[535,62,1],[535,63,1],
[535,64,2],[535,65,11],[535,66,1],[535,67,6],[535,70,6],[535,71,3],
[535,73,73],[535,75,1],[535,76,1],[535,77,25],[535,78,10],[536,9,2],
[536,12,1],[536,57,1],[537,2,1],[537,17,1],[538,10,1],[538,12,1],
[538,18,1],[538,20,1],[538,38,1],[538,48,1],[538,55,1],[538,57,1],
[538,65,1],[538,67,2],[538,71,1],[538,73,1],[538,74,1],[539,1,1],
[539,10,1],[539,21,1],[539,39,1],[540,27,3],[540,35,1],[540,66,1],
[541,12,1],[541,29,1],[541,30,1],[542,17,1],[542,53,2],[543,4,1],
[543,22,1],[543,28,1],[543,32,1],[543,47,1],[543,56,1],[543,68,2],
[544,18,1],[544,65,1],[544,76,1],[545,11,1],[545,59,1],[546,9,1],
[546,10,2],[546,14,1],[546,57,2],[546,65,1],[546,73,1],[547,20,1],
[547,23,1],[547,27,1],[547,37,1],[547,42,1],[548,7,1],[548,11,1],
[549,2,1],[549,5,1],[549,20,1],[549,23,7],[549,24,1],[549,25,1],
[549,26,8],[549,28,4],[549,33,2],[549,35,2],[549,38,1],[549,43,2],
[549,47,2],[549,56,79],[549,65,1],[549,70,1],[549,74,9],[549,75,1],
[549,77,2],[550,2,1],[550,30,1],[550,31,1],[551,2,4],[551,24,1],
[551,42,1],[552,8,1],[552,15,1],[553,3,1],[553,27,1],[554,63,6],
[554,74,4],[554,75,2],[554,76,7],[555,5,2],[555,25,23],[555,28,2],
[555,33,3],[556,4,1],[556,5,1],[556,15,1],[556,22,1],[556,34,1],
[556,42,1],[556,56,1],[557,2,2],[558,6,1],[558,24,1],[558,27,1],
[558,33,1],[558,35,1],[558,43,1],[558,50,1],[558,51,3],[558,66,2],
[559,3,1],[559,13,1],[559,20,1],[559,31,1],[559,37,7],[559,43,1],
[559,45,1],[559,60,1],[560,28,1],[560,31,1],[561,37,1],[561,53,2],
[562,2,3],[562,3,1],[562,21,14],[562,22,13],[562,23,11],[562,24,1],
[562,25,1],[562,26,13],[562,27,6],[562,28,2],[562,30,1],[562,31,9],

[562,33,4],[562,35,2],[562,42,9],[562,43,3],[562,47,1],[562,48,1],
[562,50,1],[562,72,1],[562,78,1],[563,25,3],[563,48,1],[564,55,2],
[565,53,2],[566,13,1],[566,15,1],[566,16,1],[566,34,1],[566,40,1],
[567,23,1],[567,27,2],[567,43,1],[567,62,1],[568,68,2],[568,70,4],
[569,1,2],[569,2,10],[569,3,4],[569,4,6],[569,5,1],[569,6,3],
[569,7,1],[569,8,3],[569,9,1],[569,11,2],[569,13,2],[569,14,1],
[569,15,10],[569,16,1],[569,17,2],[569,19,1],[569,20,8],[569,21,5],
[569,22,3],[569,23,2],[569,24,5],[569,25,1],[569,26,4],[569,27,2],
[569,28,10],[569,30,2],[569,31,2],[569,32,9],[569,33,4],[569,36,1],
[569,37,9],[569,38,3],[569,39,1],[569,40,3],[569,41,3],[569,42,3],
[569,43,3],[569,44,6],[569,45,1],[569,47,3],[569,48,2],[569,50,7],
[569,51,2],[569,53,3],[569,55,9],[569,56,2],[569,59,1],[569,61,1],
[569,62,3],[569,66,13],[569,68,2],[569,70,9],[569,72,1],[569,74,1],
[569,75,2],[569,77,1],[569,78,1],[570,24,1],[570,26,1],[570,27,1],
[570,31,1],[571,2,2],[571,3,1],[571,5,1],[571,9,3],[571,13,1],
[571,15,1],[571,21,2],[571,22,1],[571,23,111],[571,24,9],[571,26,2],
[571,28,1],[571,29,1],[571,30,1],[571,31,7],[571,32,1],[571,33,4],
[571,42,14],[571,43,13],[571,44,2],[571,47,3],[571,59,1],[571,63,1],
[571,65,1],[571,68,1],[571,74,1],[572,15,5],[573,0,1],[573,1,1],
[573,12,15],[574,5,1],[574,26,1],[575,27,2],[575,28,1],[576,15,1],
[576,33,1],[576,51,1],[576,56,1],[576,68,1],[576,69,1],[576,74,1],
[577,69,1],[577,72,1],[578,6,1],[578,42,1],[578,44,2],[578,47,4],
[578,50,2],[578,51,2],[578,56,4],[578,69,1],[578,72,1],[579,5,1],
[579,23,2],[579,25,2],[579,27,2],[579,38,1],[579,48,1],[580,2,3],
[580,5,1],[580,23,3],[580,24,3],[580,27,5],[580,28,1],[580,31,2],
[580,33,1],[580,35,2],[580,43,1],[580,62,3],[580,74,1],[581,22,1],
[581,28,1],[582,23,1],[582,31,1],[583,5,1],[583,21,1],[584,16,1],
[584,19,1],[585,24,3],[585,27,1],[585,28,1],[585,33,1],[586,3,1],
[586,5,1],[586,42,1],[587,5,1],[587,9,4],[587,10,1],[587,24,1],
[587,25,1],[587,28,265],[587,33,1],[587,38,1],[587,41,1],[587,48,1],
[587,63,41],[587,72,1],[587,74,20],[587,75,13],[587,76,42],[588,1,1],
[588,2,2],[588,3,2],[588,16,2],[588,21,1],[588,26,1],[588,27,1],
[588,30,1],[588,39,1],[588,47,2],[588,53,1],[589,3,3],[589,13,1],
[589,20,1],[589,24,1],[590,22,2],[590,23,1],[590,27,1],[590,45,1],
[591,22,1],[591,62,1],[592,2,2],[593,17,2],[593,19,1],[594,23,1],
[594,24,1],[594,25,3],[594,26,2],[594,32,4],[594,33,1],[594,42,1],
[595,63,2],[595,76,4],[596,26,1],[596,27,1],[597,7,1],[597,9,2],
[597,10,3],[597,12,1],[597,16,2],[597,17,1],[597,19,1],[597,39,1],
[597,41,1],[597,45,1],[597,46,1],[597,49,1],[597,55,1],[597,58,1],
[597,60,1],[598,26,10],[598,42,1],[598,56,1],[598,76,1],[599,3,1],
[599,5,1],[599,6,1],[599,21,1],[599,22,3],[599,23,1],[599,24,1],
[599,27,2],[599,39,2],[599,47,1],[599,62,1],[600,27,2],[600,28,1],
[601,10,1],[601,15,1],[601,17,2],[601,53,5],[601,57,1],[601,71,1],
[602,22,1],[602,43,1],[603,1,2],[603,12,1],[603,13,1],[603,17,1],
[603,21,2],[603,22,1],[603,23,2],[603,24,1],[603,27,17],[603,28,1],
[603,31,1],[603,32,2],[603,34,1],[603,39,2],[603,43,1],[603,53,1],
[604,77,2],[605,13,1],[605,22,3],[605,24,6],[605,27,17],[605,35,1],
[605,38,2],[605,70,1],[606,27,1],[606,28,1],[606,35,2],[607,19,1],
[607,43,1],[608,20,3],[609,2,2],[609,4,3],[609,5,1],[609,21,1],
[609,22,1],[609,23,2],[609,24,2],[609,25,1],[609,26,5],[609,27,5],

[609,28,11],[609,31,3],[609,32,1],[609,33,1],[609,35,5],[609,38,2],
[609,40,4],[609,42,1],[609,43,1],[609,44,2],[609,47,1],[609,50,4],
[609,51,1],[609,63,6],[609,66,2],[609,68,1],[609,69,3],[609,70,7],
[609,72,2],[609,74,5],[609,75,2],[609,76,6],[610,24,1],[610,27,2],
[610,35,2],[611,30,2],[611,33,1],[611,43,1],[611,50,1],[611,56,2],
[612,25,1],[612,40,1],[612,70,1],[612,74,1],[613,17,1],[613,37,1],
[613,44,1],[613,53,2],[614,3,1],[614,31,1],[614,42,1],[615,4,1],
[615,24,1],[616,27,2],[616,28,1],[616,32,2],[617,6,1],[617,56,1],
[618,15,1],[618,55,2],[619,28,5],[619,76,1],[620,4,1],[620,6,1],
[620,22,1],[620,27,2],[620,62,2],[621,1,5],[621,13,2],[621,73,1],
[622,24,2],[622,26,1],[622,27,1],[622,28,2],[623,9,2],[623,15,1],
[623,17,3],[623,28,1],[623,53,4],[623,55,4],[624,4,1],[624,9,1],
[624,15,1],[624,37,1],[624,53,2],[624,67,1],[625,12,1],[625,30,1],
[625,53,2],[626,22,1],[626,23,3],[626,24,6],[626,26,4],[626,27,6],
[626,28,8],[626,31,1],[626,32,3],[626,35,1],[626,56,1],[626,70,1],
[627,1,1],[627,28,1],[627,38,1],[628,3,1],[628,43,1],[628,75,1],
[629,5,25],[629,21,1],[629,24,1],[629,27,2],[629,32,1],[629,33,10],
[629,35,2],[629,44,1],[629,70,1],[630,28,2],[630,45,1],[630,70,1],
[631,63,1],[631,74,1],[631,75,1],[631,76,6],[632,2,1],[632,32,1],
[633,28,8],[633,31,1],[634,38,1],[634,46,1],[634,50,1],[634,53,1],
[635,38,2],[636,20,2],[637,28,1],[637,38,1],[638,18,1],[638,55,1],
[639,34,1],[639,47,1],[640,22,1],[640,27,2],[641,3,2],[641,5,1],
[641,22,4],[641,23,2],[641,24,1],[641,25,1],[641,26,2],[641,27,4],
[641,31,3],[641,32,4],[641,33,7],[641,35,4],[641,38,1],[641,43,1],
[641,47,1],[641,48,1],[641,50,1],[641,56,1],[641,63,1],[641,66,1],
[641,70,1],[642,27,3],[643,15,1],[643,53,1],[644,23,3],[645,9,1],
[645,12,1],[645,15,4],[645,16,1],[645,53,1],[645,55,1],[646,26,1],
[646,28,3],[646,32,11],[647,23,2],[648,26,1],[648,28,1],[648,62,8],
[649,23,9],[649,27,1],[649,28,1],[650,18,1],[650,54,1],[651,3,1],
[651,4,2],[651,10,1],[651,23,1],[651,25,2],[651,28,2],[651,35,1],
[651,38,1],[651,40,2],[651,42,1],[651,44,1],[651,68,1],[651,70,4],
[651,75,1],[652,24,1],[652,44,1],[652,47,1],[653,28,1],[653,32,10],
[654,1,1],[654,23,1],[654,45,1],[655,4,1],[655,14,1],[655,15,3],
[655,30,1],[655,44,3],[655,66,1],[656,28,3],[656,75,1],[657,0,1],
[657,2,6],[657,34,3],[657,36,1],[658,53,3],[659,26,2],[659,28,1],
[659,35,2],[659,43,1],[660,3,1],[660,21,1],[660,22,2],[660,43,1],
[661,3,1],[661,8,1],[661,22,1],[661,30,1],[661,34,1],[662,10,1],
[662,21,1],[662,25,4],[662,27,1],[662,28,2],[662,31,3],[662,33,6],
[662,36,1],[662,39,7],[662,42,4],[662,50,1],[662,72,1],[663,1,8],
[663,29,1],[664,25,1],[664,38,1],[665,5,1],[665,15,1],[665,20,4],
[665,32,1],[665,33,1],[665,38,1],[665,42,4],[665,44,1],[665,47,4],
[665,50,2],[665,51,4],[666,22,1],[666,25,1],[666,26,1],[666,27,2],
[666,62,1],[667,27,3],[668,20,1],[668,23,2],[668,26,1],[668,27,7],
[668,35,1],[668,42,1],[668,77,1],[669,39,2],[670,25,7],[670,74,1],
[671,28,1],[671,42,2],[672,30,1],[672,31,1],[672,43,2],[673,3,1],
[673,46,1],[674,2,2],[674,3,1],[674,21,1],[675,56,6],[676,2,1],
[676,30,1],[676,42,3],[677,3,1],[677,27,1],[677,66,1],[678,3,1],
[678,5,4],[678,6,5],[678,9,1],[678,22,2],[678,23,2],[678,24,5],
[678,25,12],[678,26,4],[678,27,6],[678,32,4],[678,35,5],[678,42,2],
[678,43,3],[678,48,1],[678,56,2],[679,38,2],[680,4,1],[680,28,1],

[681,28,19],[682,1,1],[682,28,2],[682,35,9],[683,3,2],[683,6,1],
[684,47,1],[684,50,2],[684,53,1],[685,2,2],[685,15,1],[685,17,1],
[686,2,4],[686,62,1],[687,28,1],[687,33,1],[688,2,9],[688,3,12],
[688,9,1],[688,20,1],[688,22,1],[688,24,1],[688,42,1],[688,47,5],
[688,60,1],[688,64,2],[688,68,1],[689,17,1],[689,24,1],[689,61,1],
[689,68,1],[689,69,2],[689,70,3],[689,74,1],[690,15,2],[691,1,2],
[691,13,1],[691,29,4],[691,48,1],[691,77,1],[692,1,1],[692,13,1],
[692,33,1],[693,20,1],[693,46,1],[694,0,13],[694,1,1],[694,2,23],
[694,3,1],[694,12,4],[694,13,1],[694,19,1],[694,20,2],[694,21,1],
[694,26,1],[694,27,4],[694,28,1],[694,31,30],[694,32,11],[694,39,2],
[694,42,4],[694,67,1],[694,74,1],[695,14,1],[695,57,1],[696,1,1],
[696,8,1],[696,27,2],[696,30,1],[696,32,2],[696,33,1],[697,11,1],
[697,61,2],[697,63,1],[698,4,1],[698,21,2],[698,28,1],[698,33,1],
[698,35,2],[698,44,1],[698,62,1],[698,69,1],[698,70,1],[699,74,1],
[699,76,1],[700,2,2],[701,24,5],[701,38,1],[702,47,6],[702,66,1],
[703,2,2],[704,28,1],[704,50,1],[705,16,1],[705,65,2],[706,1,1],
[706,4,1],[706,15,3],[706,19,1],[706,21,1],[706,26,1],[706,41,1],
[706,44,1],[706,47,1],[706,55,1],[706,59,2],[706,66,2],[707,2,1],
[707,3,3],[707,6,1],[707,19,1],[707,21,2],[707,22,4],[707,23,3],
[707,24,2],[707,25,1],[707,26,2],[707,27,4],[707,28,1],[707,31,5],
[707,32,1],[707,35,1],[707,37,1],[707,43,1],[707,47,1],[707,60,1],
[708,51,1],[708,55,1],[709,41,1],[709,55,1],[710,3,1],[710,26,3],
[710,27,1],[710,28,1],[711,5,1],[711,23,1],[711,27,1],[711,35,2],
[711,43,4],[711,47,1],[712,47,1],[712,68,1],[713,22,1],[713,23,1],
[713,24,1],[713,51,2],[713,70,1],[714,22,2],[715,5,1],[715,34,1],
[715,46,1],[715,62,1],[716,0,2],[716,1,468],[716,2,2],[716,3,1],
[716,5,2],[716,6,2],[716,8,9],[716,10,1],[716,12,17],[716,13,41],
[716,15,6],[716,16,2],[716,17,6],[716,20,5],[716,21,2],[716,24,3],
[716,26,1],[716,27,2],[716,29,4],[716,30,3],[716,31,1],[716,32,2],
[716,33,1],[716,34,10],[716,35,2],[716,36,6],[716,37,4],[716,39,19],
[716,40,1],[716,42,1],[716,45,2],[716,47,7],[716,50,2],[716,53,14],
[716,55,5],[716,57,1],[716,59,2],[716,69,1],[716,73,6],[716,78,1],
[717,42,2],[718,27,1],[718,28,2],[718,68,1],[719,30,3],[720,27,1],
[720,28,2],[720,30,3],[720,33,3],[720,35,1],[720,52,1],[720,74,1],
[721,21,3],[722,2,1],[722,28,1],[723,25,2],[724,23,2],[724,24,1],
[724,25,1],[724,27,1],[724,31,1],[724,32,7],[724,33,1],[725,47,2],
[725,62,2],[725,72,1],[726,22,3],[726,24,1],[727,38,2],[727,53,1],
[728,3,2],[728,47,2],[729,2,17],[729,28,3],[730,6,1],[730,13,1],
[730,20,2],[730,23,4],[730,24,3],[730,28,1],[730,31,1],[730,32,1],
[730,34,1],[730,38,1],[731,1,7],[731,2,1],[731,9,1],[731,11,1],
[731,13,14],[731,14,1],[731,20,4],[731,23,1],[731,28,6],[731,29,1],
[731,34,12],[731,41,3],[731,45,2],[731,52,1],[731,57,1],[731,59,5],
[731,61,1],[731,62,1],[731,65,2],[731,71,1],[731,76,1],[731,77,1],
[731,78,1],[732,3,1],[732,6,1],[732,20,2],[732,21,2],[732,22,1],
[732,23,9],[732,24,2],[732,26,9],[732,27,11],[732,28,3],[732,31,1],
[732,32,5],[732,33,6],[732,35,5],[732,38,1],[732,42,2],[732,43,6],
[732,59,3],[733,5,3],[733,44,1],[733,47,1],[733,51,1],[733,56,1],
[733,63,28],[733,66,1],[733,68,3],[733,70,1],[733,72,1],[733,74,20],
[733,75,14],[733,76,56],[734,15,1],[734,30,1],[734,50,2],[734,55,1],
[735,5,1],[735,6,1],[735,20,3],[735,21,2],[735,22,4],[735,23,12],

[735,24,5],[735,25,2],[735,26,9],[735,27,9],[735,28,5],[735,31,6],
[735,32,8],[735,35,2],[735,38,2],[735,42,5],[735,43,28],[735,59,4],
[735,63,2],[735,69,1],[735,70,2],[735,72,1],[736,6,1],[736,42,1],
[737,21,1],[737,61,3],[738,1,2],[738,5,1],[738,6,1],[738,21,1],
[738,32,1],[738,52,1],[739,1,1],[739,9,1],[739,45,1],[740,2,2],
[741,33,1],[741,51,2],[742,3,5],[742,5,1],[742,23,2],[742,24,1],
[742,27,2],[742,28,3],[742,32,1],[742,35,3],[742,37,1],[742,39,1],
[742,43,1],[742,47,1],[742,59,1],[742,62,1],[743,2,1],[743,31,1],
[744,22,1],[744,24,31],[744,28,3],[744,43,1],[744,47,1],[744,56,6],
[744,66,2],[745,28,1],[745,44,1],[746,26,2],[746,27,4],[746,34,11],
[747,12,1],[747,26,1],[747,28,1],[747,32,1],[748,32,1],[748,78,1],
[749,27,1],[749,32,1],[749,38,1],[749,42,1],[750,28,1],[750,33,3],
[751,2,1],[751,28,1],[752,23,4],[752,26,5],[752,27,1],[752,28,1],
[752,35,1],[752,62,2],[753,19,1],[753,54,1],[753,57,1],[754,4,3],
[754,17,3],[754,23,1],[754,30,1],[754,44,6],[754,50,1],[754,66,2],
[755,27,5],[755,28,1],[756,22,2],[756,23,1],[756,28,4],[756,35,1],
[756,56,1],[757,2,3],[758,23,3],[758,28,1],[759,2,1],[759,3,3],
[759,4,2],[759,12,1],[759,18,1],[759,19,1],[759,23,8],[759,24,2],
[759,25,3],[759,27,1],[759,28,9],[759,32,2],[759,35,2],[759,38,11],
[759,40,1],[759,42,2],[759,44,1],[759,47,2],[759,50,1],[759,55,1],
[759,57,1],[759,58,1],[759,62,1],[759,63,2],[759,66,1],[759,69,1],
[759,70,3],[759,76,1],[760,20,1],[760,74,1],[761,2,1],[761,20,1],
[761,28,1],[762,8,4],[762,23,1],[762,31,1],[762,38,1],[763,1,2],
[763,2,1],[763,3,1],[763,6,1],[763,10,3],[763,11,3],[763,13,14],
[763,15,1],[763,19,4],[763,20,12],[763,22,5],[763,23,13],[763,24,3],
[763,25,1],[763,26,13],[763,27,26],[763,28,6],[763,29,1],[763,30,2],
[763,31,7],[763,32,16],[763,34,1],[763,35,7],[763,36,3],[763,38,3],
[763,39,2],[763,42,3],[763,43,8],[763,45,3],[763,47,1],[763,48,3],
[763,55,3],[763,56,3],[763,57,1],[763,60,2],[763,62,3],[763,63,1],
[763,64,1],[763,70,1],[763,71,1],[763,73,1],[764,28,2],[764,31,2],
[765,4,5],[765,12,1],[765,15,2],[765,17,5],[765,28,16],[765,44,8],
[765,50,8],[765,53,2],[765,59,1],[765,66,2],[765,68,2],[766,3,1],
[766,25,1],[766,32,1],[766,52,1],[767,20,1],[767,32,1],[768,6,2],
[769,51,2],[770,3,2],[770,27,1],[771,44,2],[772,22,1],[772,68,4],
[773,22,1],[773,27,1],[774,2,5],[774,24,1],[774,30,1],[774,43,2],
[774,53,2],[775,9,1],[775,14,1],[775,15,8],[775,19,1],[775,24,1],
[775,28,1],[775,30,1],[775,35,1],[775,55,5],[775,56,1],[776,3,3],
[776,8,2],[776,13,2],[776,17,2],[776,19,1],[776,20,1],[776,22,1],
[776,24,2],[776,27,1],[776,39,1],[776,45,1],[776,56,1],[776,60,1],
[776,68,1],[776,69,1],[776,70,2],[776,71,1],[777,2,1],[777,5,2],
[777,8,1],[777,13,1],[777,15,1],[777,23,1],[777,24,3],[777,25,1],
[777,30,6],[777,35,1],[777,39,4],[777,42,1],[777,43,1],[777,45,1],
[777,47,2],[777,48,12],[777,55,1],[777,56,1],[777,62,1],[777,78,1],
[778,24,2],[779,1,2],[780,3,1],[780,20,1],[780,22,2],[780,23,1],
[780,27,14],[780,30,1],[780,31,1],[780,32,3],[780,35,13],[780,38,1],
[780,42,1],[780,62,1],[781,2,1],[781,8,3],[781,29,1],[781,37,4],
[782,28,1],[782,62,1],[783,4,1],[783,27,1],[783,32,1],[783,50,2],
[783,62,1],[784,52,20],[785,2,1],[785,3,1],[785,5,1],[785,10,1],
[785,13,1],[785,20,1],[785,21,1],[785,22,1],[785,23,5],[785,24,4],
[785,25,4],[785,27,3],[785,28,2],[785,31,2],[785,32,2],[785,35,4],

[785,42,1],[785,43,1],[785,47,1],[785,59,1],[785,62,1],[785,72,1],
[786,3,2],[787,28,1],[787,43,1],[788,32,28],[788,62,2],[789,6,6],
[789,22,1],[789,27,4],[789,35,14],[789,43,5],[789,44,1],[789,56,1],
[789,62,6],[789,68,1],[790,35,2],[791,1,12],[791,2,10],[791,3,4],
[791,5,4],[791,8,1],[791,10,1],[791,12,1],[791,13,6],[791,16,62],
[791,23,5],[791,25,28],[791,26,1],[791,28,27],[791,30,1],[791,32,61],
[791,37,359],[791,39,51],[791,47,3],[791,53,1],[791,59,42],[791,62,8],
[791,68,20],[791,70,1],[791,71,1],[792,31,2],[792,43,1],[793,3,1],
[793,23,1],[794,21,1],[794,28,2],[795,37,1],[795,46,1],[796,1,1],
[796,23,2],[796,34,1],[797,44,3],[798,55,1],[798,70,1],[799,13,1],
[799,15,1],[799,17,7],[799,53,3],[799,65,2],[800,2,1],[800,6,1],
[800,15,2],[800,23,1],[800,24,1],[800,27,1],[800,28,1],[800,31,2],
[800,35,3],[800,38,3],[800,40,1],[800,42,1],[800,43,1],[800,45,1],
[800,47,1],[800,50,1],[800,56,3],[800,61,1],[800,62,1],[800,63,1],
[800,68,1],[800,70,4],[800,72,1],[800,74,2],[800,75,1],[800,76,2],
[801,12,1],[801,29,1],[802,9,1],[802,40,1],[802,63,11],[802,74,7],
[802,75,8],[802,76,14],[803,3,1],[803,20,4],[803,26,2],[803,47,1],
[803,74,1],[803,76,1],[804,23,1],[804,29,1],[805,2,2],[806,5,1],
[806,20,1],[806,53,1],[807,72,2],[808,3,50],[808,10,1],[808,12,2],
[808,25,1],[808,28,1],[808,29,1],[808,30,1],[808,31,2],[808,39,2],
[808,43,1],[808,54,1],[808,66,1],[808,70,1],[809,2,7],[809,21,1],
[809,23,3],[809,24,7],[809,25,3],[809,27,3],[809,31,2],[809,32,11],
[809,35,6],[809,42,3],[809,43,5],[809,47,2],[809,50,1],[810,2,3],
[810,3,1],[810,5,1],[810,17,1],[810,23,2],[810,31,1],[810,45,1],
[811,16,1],[811,22,1],[811,27,1],[811,54,2],[812,11,1],[812,15,3],
[812,55,4],[813,19,1],[813,39,1],[813,56,1],[814,2,1],[814,4,1],
[814,5,1],[814,21,2],[814,25,1],[814,31,1],[814,43,1],[814,44,1],
[814,47,1],[815,68,1],[815,69,1],[816,0,1],[816,1,130],[816,2,24],
[816,3,437],[816,4,17],[816,5,29],[816,6,17],[816,7,4],[816,8,5],
[816,9,9],[816,10,20],[816,11,7],[816,12,14],[816,13,97],[816,14,2],
[816,15,14],[816,16,9],[816,17,5],[816,18,1],[816,19,19],[816,20,184],
[816,21,13],[816,22,62],[816,23,731],[816,24,136],[816,25,43],
[816,26,49],[816,27,307],[816,28,100],[816,29,4],[816,30,22],
[816,31,61],[816,32,32],[816,33,112],[816,34,122],[816,35,103],
[816,36,13],[816,37,44],[816,38,47],[816,39,14],[816,40,13],
[816,41,8],[816,42,135],[816,43,28],[816,44,29],[816,45,7],
[816,46,12],[816,47,95],[816,48,93],[816,49,3],[816,50,24],[816,51,5],
[816,52,3],[816,53,6],[816,54,1],[816,55,13],[816,56,50],[816,57,8],
[816,58,4],[816,59,13],[816,60,7],[816,61,5],[816,62,56],[816,63,10],
[816,64,3],[816,65,16],[816,66,19],[816,67,3],[816,68,7],[816,69,2],
[816,70,16],[816,71,5],[816,72,5],[816,73,11],[816,74,22],[816,75,11],
[816,76,12],[816,77,6],[816,78,17],[817,15,3],[817,17,2],[817,53,4],
[817,55,3],[818,17,3],[818,28,1],[818,35,1],[818,39,1],[818,53,4],
[819,10,1],[819,16,1],[820,20,2],[820,42,1],[820,48,1],[821,2,2],
[822,53,1],[822,60,1],[823,22,1],[823,44,1],[824,25,4],[825,17,1],
[825,50,1],[826,2,6],[826,3,11],[826,5,1],[826,6,2],[826,8,1],
[826,13,1],[826,22,3],[826,23,13],[826,24,2],[826,25,2],[826,26,3],
[826,27,11],[826,28,3],[826,30,1],[826,31,5],[826,32,1],[826,33,6],
[826,35,3],[826,37,1],[826,39,1],[826,42,5],[826,47,4],[826,48,1],
[826,56,1],[826,59,1],[826,62,2],[826,66,1],[826,71,1],[826,72,1],

[827,17,1],[827,67,1],[828,19,1],[828,67,1],[829,38,2],[829,48,1],
[829,51,1],[829,79,1],[830,8,2],[830,20,1],[830,25,1],[830,35,1],
[830,68,2],[831,5,1],[831,26,2],[831,28,4],[831,32,1],[831,43,1],
[832,43,2],[833,2,19],[833,9,1],[833,28,1],[833,37,3],[833,56,1],
[834,31,1],[834,42,1],[834,68,1],[835,21,1],[835,22,1],[835,23,1],
[835,24,2],[836,23,5],[836,27,1],[836,28,1],[836,42,1],[836,50,1],
[836,76,1],[837,2,1],[837,24,1],[837,32,1],[837,35,1],[837,47,1],
[838,25,2],[839,56,1],[839,75,1],[840,15,1],[840,55,1],[841,3,1],
[841,12,1],[841,19,1],[841,24,1],[841,28,1],[841,32,1],[841,37,2],
[841,52,1],[841,59,1],[841,62,1],[842,1,1],[842,10,1],[842,12,1],
[842,13,1],[842,18,2],[842,20,2],[842,53,2],[842,55,1],[842,59,1],
[843,5,1],[843,21,1],[843,23,4],[843,26,1],[843,27,1],[843,28,2],
[843,32,2],[843,35,2],[843,65,1],[844,1,1],[844,5,1],[844,9,1],
[844,11,1],[844,15,8],[844,17,2],[844,29,1],[844,44,2],[844,53,1],
[844,55,7],[844,61,1],[844,65,2],[844,77,1],[845,31,1],[845,32,2],
[846,3,1],[846,28,1],[847,12,1],[847,20,3],[847,24,1],[847,25,1],
[847,26,1],[847,43,1],[848,1,2],[848,9,1],[848,10,1],[848,12,2],
[848,13,2],[848,17,1],[848,19,1],[848,30,3],[848,36,1],[848,39,1],
[848,53,1],[848,73,1],[849,27,1],[849,28,2],[849,38,2],[849,43,1],
[849,68,1],[849,74,1],[850,20,1],[850,22,1],[850,27,4],[850,28,1],
[850,31,1],[850,35,2],[850,38,1],[850,42,5],[850,43,1],[850,75,1],
[851,68,1],[851,69,1],[851,70,4],[852,51,16],[853,5,1],[853,50,1],
[854,23,1],[854,27,1],[854,35,1],[855,4,4],[855,17,1],[855,50,1],
[855,53,3],[856,1,2],[856,9,1],[856,20,1],[856,53,1],[856,55,1],
[857,22,1],[857,23,1],[858,4,1],[858,12,1],[858,23,1],[858,38,1],
[859,4,3],[859,8,7],[859,9,1],[859,11,1],[859,16,1],[859,28,38],
[859,33,1],[859,35,1],[859,75,1],[859,77,1],[860,4,3],[860,15,1],
[860,28,1],[860,50,1],[861,22,1],[861,79,1],[862,2,1],[862,3,1],
[862,23,1],[862,26,1],[862,27,4],[862,37,1],[863,27,1],[863,32,2],
[864,23,1],[864,27,2],[864,32,1],[864,38,1],[864,40,1],[864,46,1],
[864,69,1],[865,5,1],[865,28,1],[865,62,1],[865,76,1],[866,3,1],
[866,4,1],[866,21,1],[866,22,1],[866,28,3],[866,38,2],[866,40,1],
[866,66,1],[866,74,1],[867,5,1],[867,7,1],[867,25,2],[867,26,1],
[867,27,4],[867,29,1],[867,30,1],[867,31,1],[867,32,1],[867,33,3],
[867,35,1],[867,38,1],[867,42,1],[867,45,1],[867,50,1],[867,56,1],
[867,62,2],[867,68,1],[868,16,1],[868,19,1],[868,39,1],[868,47,1],
[868,65,1],[869,1,2],[869,2,1],[869,3,3],[869,6,1],[869,15,1],
[869,20,3],[869,22,9],[869,23,2],[869,24,5],[869,26,1],[869,27,13],
[869,28,7],[869,30,1],[869,31,2],[869,32,14],[869,34,2],[869,35,8],
[869,37,18],[869,38,5],[869,40,1],[869,42,1],[869,43,1],[869,45,1],
[869,46,8],[869,48,3],[869,56,33],[869,64,1],[869,69,1],[869,70,1],
[869,76,1],[869,78,5],[870,23,2],[871,15,1],[871,30,1],[872,43,4],
[873,25,3],[873,32,2],[873,42,1],[873,47,1],[874,23,13],[874,24,1],
[874,26,2],[874,28,1],[875,6,20],[876,1,2],[877,6,1],[877,24,7],
[878,5,1],[878,27,2],[878,28,6],[878,42,1],[879,2,1],[879,27,3],
[879,31,2],[880,13,3],[880,20,1],[880,28,1],[880,62,1],[881,17,2],
[882,1,1],[882,2,1],[882,3,1],[882,4,3],[882,5,2],[882,6,6],[882,7,1],
[882,9,1],[882,10,2],[882,12,6],[882,13,1],[882,15,1],[882,20,1],
[882,22,10],[882,23,4],[882,24,9],[882,25,1],[882,26,3],[882,27,2],
[882,28,3],[882,29,2],[882,32,3],[882,33,1],[882,35,3],[882,37,1],

[882,38,4],[882,42,3],[882,44,4],[882,47,12],[882,50,6],[882,51,9],
[882,53,1],[882,55,1],[882,56,15],[882,61,2],[882,62,1],[882,66,3],
[882,68,5],[882,69,13],[882,70,17],[882,72,5],[882,73,1],[882,74,20],
[882,77,1],[883,6,1],[883,24,1],[883,25,1],[883,33,1],[884,28,1],
[884,31,2],[885,28,3],[885,47,1],[885,52,1],[886,2,1],[886,4,2],
[886,17,1],[886,20,1],[886,23,1],[886,24,1],[886,25,1],[886,26,1],
[886,70,1],[886,72,1],[886,75,1],[886,76,1],[887,28,2],[888,32,3],
[888,35,1],[889,3,1],[889,22,1],[889,25,1],[889,28,1],[889,31,1],
[889,35,3],[889,43,1],[890,2,2],[890,9,1],[890,21,6],[890,28,1],
[890,33,1],[890,48,4],[891,3,1],[891,23,1],[891,26,1],[891,39,1],
[891,70,1],[892,0,198],[892,1,88],[892,2,1],[892,4,2],[892,7,41],
[892,8,13],[892,9,22],[892,10,17],[892,11,1],[892,12,74],[892,13,19],
[892,14,3],[892,15,12],[892,16,26],[892,17,18],[892,18,6],[892,19,14],
[892,20,12],[892,22,3],[892,23,2],[892,24,6],[892,26,1],[892,27,2],
[892,28,7],[892,29,86],[892,30,3514],[892,31,3],[892,32,1],
[892,33,66],[892,34,7],[892,35,1],[892,36,3],[892,37,9],[892,38,10],
[892,39,8],[892,40,1],[892,41,4],[892,44,1],[892,45,11],[892,46,5],
[892,47,1],[892,52,201],[892,53,24],[892,54,4],[892,55,16],
[892,57,11],[892,58,5],[892,59,9],[892,60,5],[892,63,1],[892,64,1],
[892,65,12],[892,67,1],[892,71,4],[892,72,1],[892,73,19],[892,75,2],
[892,77,4],[892,78,4],[893,2,1],[893,3,1],[893,5,1],[893,26,3],
[893,35,3],[893,42,1],[893,48,1],[893,51,1],[893,62,1],[893,63,1],
[893,70,1],[893,74,2],[893,79,1],[894,44,1],[894,66,1],[895,9,1],
[895,13,1],[896,15,2],[896,55,1],[897,27,1],[897,33,7],[898,24,1],
[898,28,15],[898,34,1],[898,55,1],[898,74,1],[899,9,1],[899,12,1],
[900,5,4],[900,28,1],[900,32,2],[901,28,37],[901,40,1],[901,70,1],
[901,75,1],[902,31,1],[902,53,1],[903,7,2],[903,8,1],[903,12,10],
[903,14,1],[903,16,1],[903,28,2],[903,29,3],[903,44,1],[903,74,1],
[903,77,1],[904,1,20],[904,8,2],[904,12,1],[904,15,1],[904,20,1],
[904,29,1],[904,36,3],[904,39,1],[904,59,1],[905,27,1],[905,28,1],
[905,33,1],[905,35,2],[905,74,1],[906,20,2],[906,42,1],[907,28,1],
[907,50,1],[908,3,1],[908,28,1],[909,35,1],[909,42,1],[910,2,1],
[910,27,1],[910,28,1],[911,6,1],[911,28,1],[912,1,5],[912,2,2],
[912,3,2],[912,4,1],[912,5,3],[912,8,1],[912,9,1],[912,10,1],
[912,11,1],[912,12,4],[912,13,22],[912,15,3],[912,16,1],[912,19,3],
[912,20,30],[912,21,2],[912,23,6],[912,24,39],[912,25,1],[912,26,4],
[912,27,3],[912,28,1],[912,29,3],[912,30,3],[912,31,1],[912,32,11],
[912,33,2],[912,35,11],[912,36,1],[912,37,1],[912,40,1],[912,42,2],
[912,43,4],[912,44,2],[912,48,1],[912,50,1],[912,51,3],[912,53,1],
[912,56,11],[912,59,5],[912,62,1],[912,64,2],[912,65,2],[912,66,1],
[912,69,3],[912,70,2],[912,71,1],[912,73,2],[912,74,1],[912,76,3],
[913,8,1],[913,9,1],[913,20,5],[913,22,1],[913,23,14],[913,24,19],
[913,25,4],[913,27,7],[913,28,6],[913,31,2],[913,32,2],[913,33,1],
[913,35,3],[913,36,2],[913,37,2],[913,42,22],[913,43,1],[913,45,1],
[913,51,1],[913,52,1],[913,59,9],[913,62,21],[913,65,1],[913,67,2],
[913,68,1],[913,77,1],[914,6,1],[914,23,1],[914,66,1],[914,76,1],
[915,8,1],[915,20,2],[915,31,1],[915,34,1],[915,63,1],[916,2,7],
[916,3,3],[916,6,1],[916,23,1],[916,28,3],[916,40,3],[916,56,4],
[916,62,11],[916,63,1],[916,70,4],[917,0,1],[917,8,5],[917,20,1],
[917,22,5],[917,23,1],[917,26,2],[917,27,1],[917,33,1],[917,34,3],

[917,35,1],[917,37,2],[917,45,4],[917,54,1],[917,74,1],[918,63,6],
[918,74,1],[918,76,2],[919,62,2],[920,2,1],[920,39,1],[921,1,1],
[921,22,1],[921,49,1],[921,67,1],[922,32,1],[922,39,1],[922,47,1],
[923,24,8],[924,15,1],[924,28,1],[924,55,1],[925,23,1],[925,32,1],
[925,59,1],[926,3,1],[926,23,4],[926,25,2],[926,27,1],[926,31,1],
[927,27,2],[928,22,1],[928,28,1],[928,31,4],[929,0,5],[929,1,1],
[930,27,1],[930,32,1],[931,24,1],[931,27,3],[931,32,7],[931,43,1],
[931,56,1],[931,72,1],[932,21,3],[932,25,1],[932,26,1],[932,31,7],
[932,39,1],[932,43,3],[933,15,2],[934,19,1],[934,20,3],[934,45,1],
[935,2,2],[935,3,1],[935,9,1],[935,45,1],[936,2,1],[936,3,1],
[936,5,1],[936,10,1],[936,12,1],[936,20,2],[936,22,2],[936,23,1],
[936,24,1],[936,25,4],[936,26,3],[936,27,9],[936,28,3],[936,31,3],
[936,32,3],[936,35,2],[936,39,2],[936,42,1],[936,62,1],[936,71,1],
[936,74,1],[937,28,4],[937,31,4],[937,33,1],[938,23,1],[938,26,1],
[938,27,1],[938,35,1],[938,40,1],[939,2,15],[939,17,1],[939,20,2],
[939,25,1],[939,32,1],[939,35,1],[939,62,1],[940,35,2],[941,2,4],
[941,25,1],[942,0,31],[942,1,13],[942,2,12],[942,3,13],[942,4,1],
[942,5,1],[942,6,1],[942,7,5],[942,8,16],[942,9,11],[942,10,11],
[942,11,6],[942,12,21],[942,13,26],[942,14,3],[942,15,2],[942,16,7],
[942,17,6],[942,18,5],[942,19,9],[942,20,12],[942,21,4],[942,22,5],
[942,23,164],[942,24,64],[942,25,39],[942,26,28],[942,27,33],
[942,28,25],[942,29,12],[942,30,14],[942,31,47],[942,32,271],
[942,33,1],[942,34,13],[942,35,29],[942,36,3],[942,37,2],[942,38,2],
[942,39,60],[942,40,4],[942,41,8],[942,42,16],[942,43,4],[942,44,3],
[942,45,9],[942,47,2],[942,48,1],[942,49,6],[942,50,2],[942,53,3],
[942,54,3],[942,55,5],[942,56,1],[942,57,8],[942,58,8],[942,60,3],
[942,62,21],[942,63,2],[942,64,2],[942,65,10],[942,66,2],[942,67,5],
[942,68,1],[942,69,1],[942,70,3],[942,71,2],[942,73,5],[942,74,2],
[942,75,1],[942,76,1],[942,77,5],[942,79,1],[943,1,9],[943,5,1],
[943,15,1],[943,29,3],[944,27,1],[944,43,1],[945,1,3],[945,9,1],
[946,20,1],[946,38,1],[947,4,8],[947,50,2],[947,66,1],[948,2,27],
[948,3,28],[948,4,35],[948,5,38],[948,6,12],[948,9,1],[948,15,7],
[948,21,10],[948,22,23],[948,23,32],[948,24,51],[948,25,41],
[948,26,22],[948,27,45],[948,28,436],[948,31,26],[948,32,35],
[948,33,17],[948,35,33],[948,37,1],[948,38,27],[948,40,82],
[948,42,13],[948,43,22],[948,44,31],[948,45,1],[948,47,13],[948,48,9],
[948,50,36],[948,51,5],[948,52,9],[948,53,1],[948,55,11],[948,56,24],
[948,61,6],[948,62,44],[948,63,48],[948,66,54],[948,68,39],
[948,69,31],[948,70,149],[948,72,55],[948,74,91],[948,75,38],
[948,76,81],[948,77,1],[948,79,8],[949,23,1],[949,24,1],[949,25,1],
[949,27,1],[949,32,1],[949,40,1],[949,52,1],[949,66,1],[950,2,4],
[950,3,5],[950,4,4],[950,5,26],[950,6,1],[950,10,1],[950,21,4],
[950,22,3],[950,23,8],[950,24,11],[950,25,4],[950,26,6],[950,27,9],
[950,28,94],[950,31,9],[950,32,6],[950,33,6],[950,35,5],[950,40,11],
[950,42,3],[950,43,7],[950,44,7],[950,47,2],[950,48,2],[950,50,9],
[950,52,1],[950,56,2],[950,62,5],[950,63,184],[950,65,2],[950,66,6],
[950,68,11],[950,69,2],[950,70,17],[950,72,8],[950,74,255],
[950,75,182],[950,76,351],[950,79,3],[951,2,3],[952,4,2],[952,28,9],
[952,50,1],[952,55,1],[952,66,1],[953,5,2],[953,21,2],[954,4,7],
[954,17,1],[954,23,2],[954,42,1],[954,50,5],[954,53,2],[954,66,1],

[955,2,6],[955,28,1],[956,43,2],[957,3,3],[957,47,5],[957,74,1],
[958,2,1],[958,4,6],[958,5,1],[958,15,4],[958,23,1],[958,24,1],
[958,35,1],[958,39,1],[958,44,1],[958,50,2],[958,55,2],[958,62,1],
[958,76,1],[959,5,1],[959,23,1],[959,28,1],[959,32,4],[960,22,1],
[960,26,1],[960,38,3],[961,21,3],[961,23,8],[961,26,1],[961,35,1],
[962,62,2],[963,14,1],[963,17,1],[963,34,3],[963,35,1],[963,59,1],
[964,17,7],[964,53,5],[965,2,2],[966,5,1],[966,52,1],[967,5,1],
[967,21,1],[967,32,1],[967,43,1],[967,50,1],[967,66,1],[967,68,1],
[967,70,2],[967,75,1],[967,79,1],[968,15,1],[968,17,1],[968,38,1],
[968,41,1],[969,26,1],[969,43,1],[969,56,2],[970,63,11],[970,70,1],
[970,74,9],[970,75,13],[970,76,22],[971,42,3],[972,5,1],[972,32,1],
[972,72,2],[973,7,1],[973,12,4],[973,59,1],[973,77,1],[974,16,1],
[974,17,2],[974,27,1],[974,28,5],[974,38,1],[974,40,1],[974,42,1],
[974,53,1],[974,56,1],[974,63,3],[974,66,1],[974,70,1],[974,72,1],
[975,28,1],[975,76,4],[976,3,1],[976,27,1],[976,28,3],[976,33,1],
[976,35,1],[976,44,1],[976,51,1],[976,56,8],[977,12,1],[977,30,1],
[977,34,1],[977,43,1],[977,60,1],[978,2,1],[978,5,10],[978,12,1],
[978,15,1],[978,21,4],[978,24,1],[978,25,1],[978,33,2],[978,38,1],
[978,40,1],[978,42,1],[978,48,1],[978,51,1],[978,52,2],[978,65,1],
[978,66,2],[978,68,2],[978,72,2],[979,9,1],[979,10,1],[979,20,4],
[979,47,1],[979,58,1],[979,71,1],[979,73,1],[980,30,1],[980,53,1],
[981,4,1],[981,28,6],[982,6,1],[982,22,2],[982,27,1],[983,12,1],
[983,20,1],[983,23,1],[983,25,1],[983,27,6],[983,28,4],[983,42,1],
[983,43,1],[983,44,1],[983,47,1],[983,48,1],[983,50,1],[983,61,1],
[983,62,3],[983,68,2],[983,70,1],[983,74,1],[984,0,1],[984,1,1],
[984,9,1],[984,53,1],[985,63,1],[985,76,1],[986,2,2],[986,8,2],
[986,21,3],[986,23,57],[986,24,22],[986,26,3],[986,27,3],[986,28,3],
[986,31,1],[986,32,6],[986,35,1],[986,42,5],[987,2,1],[987,3,3],
[987,5,2],[987,6,1],[987,21,1],[987,22,7],[987,23,1],[987,24,2],
[987,27,1],[987,28,1],[987,32,1],[987,33,1],[987,39,1],[987,40,1],
[987,42,4],[987,56,1],[987,70,1],[988,17,2],[988,55,1],[989,25,1],
[989,28,2],[990,22,1],[990,62,1],[991,7,1],[991,10,1],[992,3,1],
[992,25,1],[992,28,2],[992,40,1],[992,62,1],[992,66,1],[992,70,5],
[993,30,1],[993,55,1],[994,5,2],[994,28,1],[994,62,1],[995,2,1],
[995,28,1],[996,6,3],[996,18,1],[996,23,4],[996,24,1],[996,28,2],
[996,35,1],[996,40,3],[996,42,2],[996,47,1],[996,56,2],[996,61,3],
[996,68,1],[996,69,17],[996,70,10],[996,74,4],[997,9,1],[997,13,1],
[997,28,2],[997,37,1],[997,56,1],[998,20,2],[998,34,1],[999,56,1],
[999,66,1],[999,69,1],[1000,10,1],[1000,15,1],[1000,17,3],[1000,53,2],
[1001,17,1],[1001,33,1],[1001,56,1],[1002,23,1],[1002,28,2],
[1002,31,1],[1003,27,1],[1003,31,1],[1004,15,1],[1004,18,1],
[1004,21,2],[1004,58,1],[1004,60,1],[1005,2,2],[1006,23,3],
[1006,27,3],[1006,28,2],[1006,47,2],[1007,24,1],[1007,27,2],
[1007,38,1],[1007,43,1],[1008,51,2],[1009,3,1],[1009,9,1],[1009,15,1],
[1009,20,2],[1009,22,1],[1009,36,2],[1009,46,1],[1010,0,1],[1010,2,1],
[1010,5,3],[1010,9,1],[1010,13,1],[1010,21,2],[1010,32,1],[1010,37,1],
[1011,5,1],[1011,23,6],[1011,27,4],[1011,50,1],[1011,70,1],
[1012,11,1],[1012,18,1],[1013,10,1],[1013,28,2],[1013,58,1],
[1013,63,3],[1013,74,2],[1013,75,1],[1013,76,4],[1014,26,1],
[1014,28,1],[1014,35,1],[1015,20,2],[1015,24,1],[1016,27,2],

[1017,42,2],[1018,37,1],[1018,53,1],[1018,64,1],[1019,1,1],[1019,9,1],
[1019,16,1],[1019,19,1],[1019,45,1],[1019,58,1],[1019,71,1],
[1020,42,6],[1021,27,1],[1021,59,1],[1022,5,1],[1022,15,1],
[1022,20,7],[1022,22,2],[1022,23,1],[1022,32,1],[1022,35,1],
[1022,36,1],[1022,42,1],[1023,8,2],[1023,9,3],[1023,20,1],[1023,36,1],
[1023,54,1],[1023,55,1],[1023,58,2],[1024,0,1],[1024,20,1],
[1025,51,5],[1025,69,2],[1025,70,2],[1026,34,2],[1027,22,1],
[1027,24,1],[1027,47,2],[1028,3,2],[1028,22,1],[1028,23,2],
[1028,35,1],[1028,42,1],[1029,21,1],[1029,23,1],[1030,75,1],
[1030,76,1],[1031,2,3],[1031,31,2],[1031,42,1],[1031,55,1],
[1031,59,5],[1031,75,1],[1032,1,1],[1032,19,1],[1032,20,1],
[1032,22,1],[1032,23,6],[1032,26,3],[1032,27,7],[1032,28,2],
[1032,32,9],[1032,35,4],[1032,43,3],[1032,50,1],[1032,59,1],
[1032,70,1],[1033,26,1],[1033,35,1],[1034,23,1],[1034,35,1],
[1035,53,6],[1036,2,1],[1036,28,1],[1037,2,1],[1037,13,1],[1037,27,1],
[1037,28,1],[1037,43,1],[1038,28,1],[1038,40,1],[1038,68,5],
[1038,69,2],[1038,70,2],[1038,72,1],[1039,13,1],[1039,34,1],
[1040,21,1],[1040,26,1],[1041,3,1],[1041,53,1],[1042,26,1],
[1042,27,1],[1043,15,2],[1043,17,1],[1043,20,2],[1043,23,2],
[1043,31,1],[1043,35,1],[1043,40,1],[1043,55,1],[1043,58,1],
[1044,3,2],[1045,2,1],[1045,21,3],[1045,31,2],[1045,43,2],[1045,52,1],
[1046,2,8],[1047,20,1],[1047,22,1],[1047,24,1],[1048,10,1],
[1048,11,2],[1048,12,1],[1048,15,1],[1048,19,1],[1048,34,2],
[1048,37,1],[1048,39,1],[1048,45,1],[1048,46,2],[1048,49,1],
[1048,53,1],[1048,55,1],[1048,65,1],[1048,77,1],[1049,27,3],
[1049,32,2],[1049,38,1],[1049,42,1],[1049,57,1],[1050,32,1],
[1050,47,1],[1051,2,1],[1051,28,1],[1052,27,2],[1052,33,1],
[1052,35,1],[1053,4,2],[1053,28,8],[1053,44,1],[1053,50,1],
[1053,55,1],[1053,59,1],[1053,66,3],[1054,27,1],[1054,47,1],
[1055,2,1],[1055,22,1],[1056,6,1],[1056,15,2],[1056,25,2],[1056,35,1],
[1056,45,1],[1056,55,1],[1056,56,2],[1056,64,1],[1057,3,3],[1057,9,1],
[1057,13,1],[1057,17,1],[1057,19,1],[1057,25,1],[1057,30,1],
[1057,42,2],[1057,47,1],[1057,54,1],[1057,73,1],[1058,25,1],
[1058,35,5],[1059,5,3],[1059,35,4],[1060,9,2],[1060,10,1],[1060,11,1],
[1060,13,1],[1060,15,3],[1060,16,1],[1060,37,2],[1060,53,1],
[1060,55,12],[1061,22,1],[1061,23,13],[1061,27,1],[1061,28,1],
[1061,33,1],[1061,35,2],[1061,62,1],[1062,3,1],[1062,5,1],[1062,9,1],
[1062,10,1],[1062,11,1],[1062,12,3],[1062,15,1],[1062,16,1],
[1062,19,1],[1062,25,2],[1062,27,1],[1062,30,1],[1062,31,1],
[1062,32,1],[1062,35,2],[1062,36,1],[1062,37,1],[1062,39,1],
[1062,47,1],[1062,50,1],[1062,53,3],[1062,55,2],[1062,58,1],
[1062,59,1],[1062,60,1],[1062,63,6],[1062,74,4],[1062,75,3],
[1062,76,4],[1063,6,1],[1063,33,1],[1064,4,1],[1064,44,1],[1065,32,3],
[1066,28,2],[1067,27,24],[1067,38,1],[1067,43,1],[1068,35,1],
[1068,59,1],[1069,23,1],[1069,32,1],[1069,72,1],[1070,36,2],
[1071,6,3],[1071,21,1],[1071,25,1],[1071,26,1],[1071,42,1],
[1071,56,1],[1071,70,1],[1072,2,2],[1072,8,1],[1072,20,1],[1072,27,1],
[1072,39,1],[1073,1,3],[1073,2,2],[1073,12,4],[1073,16,1],[1073,29,2],
[1073,36,1],[1074,5,2],[1074,20,1],[1074,21,1],[1074,22,1],
[1074,28,1],[1074,33,2],[1074,35,2],[1075,70,1],[1075,74,3],

[1076,15,2],[1077,22,3],[1077,28,1],[1077,32,1],[1077,43,2],
[1078,25,5],[1079,4,22],[1079,5,1],[1079,10,2],[1079,16,1],
[1079,19,2],[1079,27,2],[1079,32,2],[1079,34,1],[1079,38,2],
[1079,41,2],[1079,44,3],[1079,47,1],[1079,50,31],[1079,57,1],
[1079,58,1],[1079,66,9],[1080,20,1],[1080,23,1],[1080,41,1],
[1081,25,1],[1081,33,17],[1081,35,9],[1081,52,2],[1082,29,1],
[1082,55,1],[1083,42,6],[1084,2,10],[1084,30,1],[1084,31,2],
[1085,11,1],[1085,13,1],[1085,23,2],[1085,24,7],[1085,33,1],
[1085,51,1],[1085,56,2],[1085,74,1],[1086,24,1],[1086,28,1],
[1086,47,1],[1086,59,1],[1087,43,2],[1088,22,1],[1088,25,1],
[1088,27,1],[1089,28,2],[1089,67,1],[1090,1,3],[1090,9,1],[1090,10,3],
[1090,11,2],[1090,19,2],[1090,37,2],[1090,39,1],[1090,45,2],
[1090,49,2],[1090,53,1],[1090,64,1],[1090,67,2],[1090,77,1],
[1091,1,4],[1091,2,2],[1091,5,1],[1091,6,1],[1091,8,1],[1091,9,1],
[1091,10,1],[1091,12,1],[1091,13,7],[1091,17,1],[1091,20,12],
[1091,21,1],[1091,22,1],[1091,23,2],[1091,24,31],[1091,27,2],
[1091,29,1],[1091,30,2],[1091,32,6],[1091,33,6],[1091,35,1],
[1091,40,1],[1091,41,1],[1091,42,1],[1091,43,1],[1091,44,1],
[1091,47,1],[1091,48,1],[1091,51,1],[1091,53,1],[1091,55,1],
[1091,56,5],[1091,59,1],[1091,62,1],[1091,63,2],[1091,69,2],
[1091,70,1],[1091,71,1],[1091,74,3],[1091,75,1],[1091,76,1],
[1091,77,1],[1091,78,1],[1092,35,1],[1092,56,1],[1093,28,1],
[1093,35,6],[1093,50,1],[1094,16,1],[1094,55,1],[1095,3,1],
[1095,22,3],[1095,23,6],[1095,24,1],[1095,27,1],[1095,31,1],
[1095,50,1],[1096,0,1],[1096,6,1],[1097,26,1],[1097,28,1],[1097,40,1],
[1097,70,1],[1098,9,1],[1098,12,1],[1098,13,1],[1098,28,1],
[1099,28,1],[1099,43,1],[1100,3,1],[1100,24,2],[1101,2,1],[1101,3,1],
[1101,4,1],[1101,5,239],[1101,22,1],[1101,23,2],[1101,24,2],
[1101,25,5],[1101,26,3],[1101,27,1],[1101,28,25],[1101,31,2],
[1101,32,1],[1101,33,181],[1101,35,3],[1101,38,5],[1101,40,3],
[1101,42,2],[1101,43,1],[1101,44,4],[1101,47,1],[1101,48,6],
[1101,50,3],[1101,51,2],[1101,52,1],[1101,63,3],[1101,66,3],
[1101,68,1],[1101,69,2],[1101,70,3],[1101,72,2],[1101,74,1],
[1101,76,3],[1102,23,2],[1103,44,2],[1104,2,4],[1105,5,1],[1105,35,1],
[1106,5,1],[1106,51,1],[1107,9,1],[1107,30,13],[1107,33,4],
[1107,52,3],[1107,60,1],[1108,22,1],[1108,35,1],[1108,42,2],
[1109,2,1],[1109,5,15],[1109,6,2],[1109,16,1],[1109,17,1],[1109,21,1],
[1109,24,1],[1109,27,2],[1109,28,5],[1109,31,1],[1109,32,3],
[1109,33,1],[1109,35,38],[1109,43,1],[1109,47,1],[1109,50,1],
[1109,56,5],[1109,61,1],[1109,66,1],[1109,69,1],[1109,70,1],
[1109,74,1],[1109,75,1],[1109,76,1],[1110,1,3],[1110,2,2],[1110,4,2],
[1110,5,2],[1110,6,10],[1110,8,1],[1110,12,1],[1110,13,2],[1110,20,1],
[1110,21,6],[1110,23,7],[1110,25,2],[1110,26,4],[1110,27,2],
[1110,28,1],[1110,29,1],[1110,32,1],[1110,33,5],[1110,39,1],
[1110,40,1],[1110,42,6],[1110,43,2],[1110,47,6],[1110,48,7],
[1110,50,3],[1110,55,1],[1110,66,1],[1111,2,5],[1111,31,1],[1112,9,2],
[1112,15,2],[1112,17,1],[1112,19,1],[1112,32,1],[1112,53,1],
[1112,55,2],[1112,67,1],[1113,5,1],[1113,21,1],[1113,27,1],
[1113,28,1],[1114,26,1],[1114,43,1],[1115,56,1],[1115,66,1],
[1115,74,1],[1116,2,2],[1117,4,2],[1117,5,2],[1117,20,3],[1117,21,1],

[1117,23,3],[1117,25,1],[1117,27,15],[1117,35,1],[1117,40,1],
[1117,43,1],[1117,72,1],[1118,2,1],[1118,5,3],[1118,21,1],[1118,22,2],
[1118,25,52],[1118,28,3],[1118,31,1],[1118,32,1],[1118,52,1],
[1118,66,1],[1119,3,1],[1119,25,1],[1119,43,1],[1119,61,1],
[1120,22,1],[1120,27,1],[1120,28,1],[1120,43,1],[1120,54,1],
[1121,22,1],[1121,24,1],[1121,27,1],[1121,34,1],[1121,35,4],
[1122,23,3],[1122,31,2],[1123,1,7],[1123,2,3],[1123,3,24],[1123,5,2],
[1123,6,2],[1123,7,2],[1123,13,4],[1123,20,4],[1123,22,2],
[1123,23,30],[1123,24,25],[1123,25,1],[1123,26,17],[1123,27,16],
[1123,28,5],[1123,30,1],[1123,31,10],[1123,32,9],[1123,33,2],
[1123,35,4],[1123,37,4],[1123,42,1],[1123,43,24],[1123,45,1],
[1123,47,7],[1123,48,1],[1123,51,1],[1123,52,1],[1123,62,6],
[1124,2,45],[1124,3,4],[1124,4,1],[1124,5,6],[1124,6,1],[1124,10,1],
[1124,17,1],[1124,20,2],[1124,21,3],[1124,22,1],[1124,23,7],
[1124,24,3],[1124,25,2],[1124,26,7],[1124,27,1],[1124,28,2],
[1124,30,2],[1124,31,15],[1124,32,3],[1124,33,3],[1124,35,1],
[1124,39,1],[1124,42,4],[1124,43,3],[1124,47,3],[1124,50,1],
[1124,53,1],[1124,60,3],[1124,64,1],[1124,69,3],[1124,70,5],
[1124,71,1],[1124,73,1],[1124,74,1],[1124,75,1],[1124,76,1],
[1125,27,1],[1125,33,1],[1125,35,1],[1126,74,1],[1126,75,1],
[1126,79,1],[1127,2,1],[1127,22,1],[1127,23,1],[1127,24,1],
[1127,26,1],[1127,42,1],[1128,15,1],[1128,18,1],[1129,6,1],
[1129,35,1],[1129,48,1],[1130,5,1],[1130,24,1],[1131,5,1],[1131,43,1],
[1132,32,1],[1132,43,1],[1133,1,10],[1133,4,1],[1133,6,1],[1133,8,2],
[1133,13,3],[1133,16,1],[1133,22,1],[1133,30,24],[1133,31,3],
[1133,35,1],[1133,36,1],[1133,39,1],[1133,42,2],[1133,47,1],
[1133,55,1],[1133,70,1],[1133,74,3],[1134,6,2],[1134,21,1],
[1134,22,3],[1134,23,43],[1134,24,1],[1134,27,13],[1134,28,3],
[1134,31,1],[1134,38,1],[1134,42,2],[1134,47,1],[1134,51,3],
[1134,61,2],[1134,68,2],[1134,69,4],[1134,72,3],[1134,74,1],
[1134,76,1],[1135,23,3],[1135,30,1],[1135,32,1],[1135,33,1],
[1136,22,7],[1136,24,3],[1136,31,2],[1137,2,1],[1137,28,1],
[1137,31,2],[1137,32,1],[1138,2,20],[1138,4,1],[1138,21,1],
[1138,25,1],[1139,2,1],[1139,31,4],[1140,3,2],[1140,6,5],[1140,14,1],
[1140,22,2],[1140,24,11],[1140,25,2],[1140,26,1],[1140,27,10],
[1140,31,1],[1140,32,2],[1140,33,3],[1140,34,1],[1140,37,6],
[1140,38,1],[1140,39,1],[1140,44,1],[1140,47,4],[1140,48,5],
[1140,56,18],[1140,59,3],[1140,62,1],[1140,70,1],[1140,75,1],
[1141,4,1],[1141,28,4],[1142,28,3],[1143,15,2],[1144,5,1],[1144,21,1],
[1144,23,1],[1144,25,1],[1144,26,2],[1144,28,1],[1144,29,1],
[1144,32,4],[1144,35,1],[1144,48,1],[1145,3,1],[1145,4,2],[1145,15,3],
[1145,28,1],[1145,44,2],[1145,50,4],[1145,55,1],[1145,66,2],
[1146,2,1],[1146,5,1],[1147,21,1],[1147,27,1],[1147,28,1],[1147,40,1],
[1148,15,4],[1148,17,1],[1148,19,1],[1148,43,1],[1148,55,2],
[1149,0,1],[1149,1,1],[1149,24,1],[1149,27,5],[1149,29,1],[1149,57,1],
[1149,62,1],[1150,30,2],[1151,23,1],[1151,35,1],[1151,47,1],
[1152,2,2],[1152,28,1],[1153,22,1],[1153,26,1],[1153,35,1],
[1153,47,1],[1153,69,1],[1154,23,3],[1154,27,2],[1154,35,3],
[1154,66,3],[1155,17,1],[1155,64,1],[1156,28,1],[1156,35,1],
[1157,5,1],[1157,31,1],[1157,42,2],[1158,1,1],[1158,4,5],[1158,5,2],

[1158,13,1],[1158,15,16],[1158,17,1],[1158,20,2],[1158,23,2],
[1158,25,1],[1158,27,5],[1158,28,1],[1158,32,2],[1158,35,1],
[1158,38,3],[1158,40,2],[1158,44,4],[1158,45,2],[1158,47,1],
[1158,50,4],[1158,51,1],[1158,53,1],[1158,55,7],[1158,65,1],
[1158,66,2],[1158,68,1],[1158,69,1],[1158,70,1],[1158,74,1],
[1158,75,2],[1158,76,4],[1159,25,1],[1159,28,1],[1160,15,1],
[1160,55,1],[1161,40,1],[1161,70,1],[1162,10,1],[1162,11,1],
[1162,13,1],[1162,20,1],[1162,22,1],[1162,24,3],[1162,27,1],
[1162,28,2],[1162,35,1],[1162,68,1],[1162,69,1],[1162,70,1],
[1162,72,2],[1162,74,7],[1163,5,26],[1163,21,27],[1163,22,13],
[1163,23,9],[1163,24,2],[1163,25,7],[1163,26,9],[1163,27,23],
[1163,28,6],[1163,31,63],[1163,32,3],[1163,33,2],[1163,35,8],
[1163,43,4],[1163,44,1],[1163,62,1],[1163,70,2],[1163,76,1],
[1164,3,3],[1164,5,2],[1164,8,1],[1164,22,5],[1164,23,3],[1164,24,7],
[1164,25,5],[1164,26,3],[1164,27,17],[1164,28,3],[1164,31,2],
[1164,32,4],[1164,33,4],[1164,35,15],[1164,39,1],[1164,42,4],
[1164,43,2],[1164,48,1],[1164,62,2],[1164,63,1],[1165,19,1],
[1165,73,1],[1166,2,1],[1166,27,2],[1166,28,1],[1166,35,1],
[1166,52,1],[1167,6,1],[1167,48,1],[1168,2,1],[1168,22,1],[1169,27,1],
[1169,43,1],[1170,3,3],[1170,5,1],[1170,6,2],[1170,7,1],[1170,8,2],
[1170,15,2],[1170,20,1],[1170,23,1],[1170,24,7],[1170,26,2],
[1170,27,2],[1170,28,13],[1170,31,1],[1170,32,2],[1170,38,1],
[1170,44,1],[1171,21,1],[1171,31,2],[1171,42,3],[1171,43,1],
[1172,5,1],[1172,27,1],[1172,35,1],[1172,63,1],[1172,66,1],[1173,5,2],
[1173,6,1],[1173,21,1],[1173,24,7],[1173,26,1],[1173,35,1],
[1173,47,1],[1173,52,1],[1174,23,4],[1174,24,1],[1174,26,1],
[1174,28,5],[1174,31,2],[1174,34,1],[1174,43,1],[1175,2,3],
[1175,20,2],[1175,22,2],[1175,23,2],[1175,24,1],[1175,25,1],
[1175,27,1],[1175,28,1],[1175,32,2],[1175,39,1],[1175,61,1],
[1176,4,2],[1176,31,1],[1176,44,2],[1177,28,1],[1177,74,1],
[1178,18,1],[1178,27,1],[1178,32,1],[1178,33,2],[1178,35,1],
[1178,38,1],[1178,43,1],[1178,51,1],[1179,44,1],[1179,66,2],
[1180,1,1],[1180,13,1],[1181,20,1],[1181,28,1],[1181,62,1],[1182,0,1],
[1182,3,1],[1182,13,1],[1182,20,1],[1182,29,1],[1182,32,1],
[1183,20,1],[1183,50,1],[1184,2,1],[1184,3,1],[1184,28,2],[1184,46,1],
[1185,3,1],[1185,21,2],[1185,28,1],[1185,31,4],[1185,32,2],
[1185,39,1],[1186,1,1],[1186,29,1],[1186,32,1],[1187,32,2],[1188,5,2],
[1189,35,3],[1190,23,1],[1190,42,1],[1191,3,2],[1191,30,1],
[1191,68,1],[1192,6,1],[1192,22,9],[1192,27,1],[1192,35,1],[1193,3,3],
[1193,4,3],[1193,5,2],[1193,6,1],[1193,22,1],[1193,23,2],[1193,24,2],
[1193,25,4],[1193,26,1],[1193,27,2],[1193,28,10],[1193,31,1],
[1193,32,2],[1193,33,2],[1193,35,1],[1193,38,3],[1193,40,1],
[1193,42,2],[1193,43,1],[1193,44,3],[1193,48,1],[1193,50,1],
[1193,62,2],[1193,66,1],[1193,70,11],[1193,72,1],[1193,74,1],
[1193,75,3],[1193,76,4],[1194,16,1],[1194,34,1],[1195,22,1],
[1195,28,1],[1196,23,2],[1196,28,1],[1196,42,1],[1197,34,1],
[1197,37,1],[1198,1,2],[1198,28,1],[1199,23,1],[1199,31,1],
[1199,51,2],[1200,12,1],[1200,13,1],[1201,22,1],[1201,25,1],
[1201,28,1],[1201,62,1],[1202,40,1],[1202,56,1],[1202,68,1],
[1202,75,1],[1203,34,1],[1203,39,1],[1203,57,1],[1204,30,1],

[1204,39,1],[1205,15,1],[1205,53,1],[1206,15,1],[1206,28,3],
[1206,53,1],[1207,1,1],[1207,29,1],[1208,5,1],[1208,24,1],[1208,26,1],
[1208,37,1],[1209,22,1],[1209,27,3],[1209,33,1],[1210,12,2],
[1210,20,1],[1210,21,1],[1210,22,2],[1210,23,5],[1210,24,2],
[1210,25,1],[1210,26,1],[1210,27,3],[1210,32,1],[1210,33,1],
[1210,35,1],[1210,37,1],[1210,38,2],[1210,39,1],[1210,42,3],
[1210,43,3],[1210,71,1],[1211,2,39],[1211,19,1],[1211,23,1],
[1211,40,1],[1211,43,1],[1211,50,1],[1211,77,3],[1212,28,1],
[1212,44,1],[1213,25,3],[1213,32,1],[1213,38,1],[1214,41,4],
[1215,27,1],[1215,28,1],[1216,15,1],[1216,23,3],[1216,24,18],
[1216,27,1],[1216,28,2],[1216,42,1],[1216,47,1],[1216,70,1],
[1216,75,1],[1216,76,1],[1217,1,1],[1217,29,1],[1218,21,1],
[1218,35,2],[1218,40,1],[1218,56,1],[1219,74,2],[1220,31,1],
[1220,53,1],[1221,12,1],[1221,20,4],[1221,24,2],[1221,39,1],
[1222,2,4],[1222,3,1],[1222,5,1],[1222,19,1],[1222,20,1],[1222,25,1],
[1222,58,1],[1222,62,1],[1223,12,5],[1223,29,1],[1224,15,2],
[1225,31,2],[1226,40,1],[1226,42,1],[1227,2,1],[1227,3,51],[1227,5,1],
[1227,23,2],[1227,25,346],[1227,27,1],[1227,28,6],[1227,32,1],
[1227,37,1],[1227,40,1],[1227,44,2],[1227,48,1],[1227,50,1],
[1227,51,1],[1227,52,1],[1227,62,1],[1227,66,1],[1227,68,1],
[1227,74,1],[1227,76,1],[1228,2,1],[1228,13,1],[1228,39,3],
[1228,42,1],[1229,12,1],[1229,40,1],[1229,55,1],[1229,69,1],
[1229,70,1],[1230,0,1],[1230,9,1],[1230,37,1],[1230,41,1],[1230,45,1],
[1231,4,4],[1231,17,3],[1231,44,1],[1231,50,3],[1231,53,4],
[1231,55,1],[1231,58,1],[1231,75,1],[1232,10,1],[1232,30,1],
[1232,45,1],[1232,65,1],[1233,23,2],[1234,8,1],[1234,21,1],
[1234,28,1],[1235,28,1],[1235,47,1],[1236,0,1],[1236,2,2],
[1236,12,14],[1236,13,1],[1236,18,1],[1236,25,1],[1236,29,5],
[1236,57,1],[1236,62,1],[1236,64,1],[1236,65,1],[1237,1,1],
[1237,13,1],[1238,17,2],[1238,19,1],[1238,53,2],[1239,3,3],
[1239,24,3],[1239,26,3],[1239,28,7],[1240,2,14],[1240,20,1],
[1240,30,1],[1240,31,3],[1240,34,2],[1241,4,1],[1241,22,1],
[1241,24,1],[1241,25,5],[1241,27,3],[1241,28,4],[1241,32,40],
[1241,33,18],[1241,35,20],[1241,38,3],[1241,52,30],[1241,62,1],
[1242,5,1],[1242,32,1],[1242,63,1],[1243,24,1],[1243,47,2],[1244,2,1],
[1244,68,1],[1244,70,1],[1245,2,1],[1245,28,3],[1246,24,1],
[1246,26,1],[1246,27,1],[1246,31,1],[1246,32,1],[1246,35,1],
[1246,43,1],[1246,44,3],[1246,48,1],[1246,51,1],[1246,62,1],
[1246,66,3],[1246,70,1],[1246,74,2],[1247,5,1],[1247,26,1],
[1247,28,27],[1247,35,1],[1248,2,1],[1248,5,1],[1248,22,4],
[1248,23,1],[1248,31,1],[1248,56,1],[1249,28,2],[1249,33,1],
[1249,76,1],[1250,3,1],[1250,6,1],[1250,32,2],[1250,43,1],[1250,56,3],
[1251,0,13],[1251,1,43],[1251,2,98],[1251,3,86],[1251,4,132],
[1251,5,414],[1251,6,44],[1251,7,22],[1251,8,26],[1251,9,117],
[1251,10,212],[1251,11,95],[1251,12,71],[1251,13,30],[1251,14,49],
[1251,15,43],[1251,16,101],[1251,17,19],[1251,18,71],[1251,19,175],
[1251,20,18],[1251,21,66],[1251,22,99],[1251,23,264],[1251,24,165],
[1251,25,149],[1251,26,126],[1251,27,173],[1251,28,2052],[1251,29,6],
[1251,30,17],[1251,31,117],[1251,32,135],[1251,33,87],[1251,34,42],
[1251,35,154],[1251,36,44],[1251,37,60],[1251,38,100],[1251,39,25],

[1251,40,475],[1251,41,95],[1251,42,72],[1251,43,88],[1251,44,157],
[1251,45,112],[1251,46,62],[1251,47,63],[1251,48,33],[1251,49,33],
[1251,50,136],[1251,51,30],[1251,52,41],[1251,53,29],[1251,54,56],
[1251,55,38],[1251,56,71],[1251,57,85],[1251,58,66],[1251,59,79],
[1251,60,18],[1251,61,16],[1251,62,61],[1251,63,17414],[1251,64,20],
[1251,65,114],[1251,66,153],[1251,67,78],[1251,68,315],[1251,69,168],
[1251,70,680],[1251,71,49],[1251,72,300],[1251,73,65],[1251,74,15156],
[1251,75,13205],[1251,76,20590],[1251,77,75],[1251,78,21],
[1251,79,60],[1252,3,2],[1253,8,1],[1253,12,1],[1253,22,1],
[1253,27,2],[1253,29,1],[1253,32,1],[1253,35,1],[1253,42,1],
[1253,70,1],[1254,22,24],[1254,28,1],[1254,47,1],[1255,2,5],
[1255,23,1],[1255,24,1],[1255,27,1],[1255,34,1],[1255,35,1],
[1255,42,1],[1255,54,1],[1256,1,1],[1256,39,1],[1257,34,3],
[1257,35,1],[1258,2,1],[1258,21,1],[1258,23,4],[1258,24,5],
[1258,25,1],[1258,26,2],[1258,28,2],[1258,33,1],[1259,24,1],
[1259,43,1],[1260,22,1],[1260,24,1],[1260,46,3],[1261,3,1],
[1261,23,2],[1261,24,1],[1261,27,5],[1261,33,6],[1261,37,1],
[1261,40,1],[1261,42,1],[1261,43,1],[1261,47,1],[1261,48,1],
[1262,2,2],[1263,21,2],[1263,26,3],[1263,31,1],[1263,33,10],
[1263,43,6],[1263,51,1],[1264,23,2],[1265,3,1],[1265,10,1],
[1265,20,3],[1265,25,1],[1265,26,3],[1265,27,4],[1265,28,2],
[1265,30,1],[1265,32,6],[1265,33,1],[1265,35,3],[1265,42,1],
[1265,56,1],[1265,62,6],[1266,44,4],[1266,50,1],[1266,66,3],
[1267,5,2],[1268,47,1],[1268,50,1],[1269,40,2],[1270,48,1],
[1270,70,1],[1271,22,2],[1272,2,1],[1272,20,1],[1272,42,1],
[1272,43,1],[1273,0,1],[1273,1,3],[1273,2,8],[1273,3,11],[1273,4,1],
[1273,5,3],[1273,6,5],[1273,9,8],[1273,10,5],[1273,11,3],[1273,13,7],
[1273,14,2],[1273,15,2],[1273,16,4],[1273,17,1],[1273,19,6],
[1273,20,3],[1273,21,15],[1273,22,29],[1273,23,131],[1273,24,30],
[1273,25,18],[1273,26,37],[1273,27,21],[1273,28,25],[1273,29,8],
[1273,30,2],[1273,31,25],[1273,32,5],[1273,33,31],[1273,35,8],
[1273,36,2],[1273,38,15],[1273,40,1],[1273,41,2],[1273,43,26],
[1273,45,2],[1273,47,6],[1273,48,65],[1273,52,1],[1273,55,2],
[1273,56,9],[1273,57,3],[1273,58,2],[1273,59,7],[1273,60,2],
[1273,61,2],[1273,63,1],[1273,65,2],[1273,66,4],[1273,67,1],
[1273,68,1],[1273,69,1],[1273,70,2],[1273,71,2],[1273,72,1],
[1273,74,3],[1273,75,1],[1273,76,3],[1273,77,2],[1273,78,8],
[1274,23,1],[1274,28,4],[1274,35,1],[1275,0,1],[1275,1,1],[1275,3,1],
[1275,9,1],[1275,20,3],[1275,22,1],[1275,23,2],[1275,24,1],
[1275,26,1],[1275,28,1],[1275,31,1],[1275,32,1],[1275,35,1],
[1275,36,1],[1275,39,1],[1275,41,1],[1275,42,5],[1275,45,1],
[1275,65,1],[1275,71,1],[1276,3,1],[1276,43,1],[1276,48,1],
[1276,61,1],[1277,32,4],[1277,35,1],[1278,2,1],[1278,4,13],[1278,9,2],
[1278,15,4],[1278,17,162],[1278,20,1],[1278,28,1],[1278,45,1],
[1278,50,26],[1278,53,193],[1278,54,1],[1278,55,49],[1278,65,1],
[1279,3,2],[1279,20,6],[1279,26,5],[1279,28,1],[1279,35,2],
[1279,71,1],[1280,25,1],[1280,27,1],[1280,32,1],[1281,25,1],
[1281,33,2],[1281,76,1],[1282,22,1],[1282,28,1],[1283,10,1],
[1283,54,1],[1283,78,1],[1284,17,2],[1284,53,1],[1285,22,1],
[1285,23,3],[1285,24,1],[1285,25,1],[1285,27,1],[1285,42,1],

[1285,47,1],[1285,62,1],[1286,2,2],[1286,3,1],[1286,26,1],[1286,35,1],
[1286,44,1],[1286,62,1],[1286,70,1],[1287,25,2],[1287,33,2],
[1287,35,2],[1288,28,2],[1288,35,3],[1289,10,1],[1289,11,1],
[1289,17,1],[1290,6,1],[1290,19,1],[1290,20,3],[1290,21,3],
[1290,42,2],[1290,68,1],[1290,70,1],[1290,78,1],[1291,15,1],
[1291,17,3],[1292,1,1],[1292,9,1],[1292,10,1],[1292,14,1],[1292,15,4],
[1292,28,7],[1292,41,1],[1292,42,1],[1292,46,1],[1292,55,3],
[1292,58,1],[1292,59,1],[1293,3,1],[1293,37,2],[1294,21,1],
[1294,28,2],[1295,28,3],[1295,31,1],[1295,74,1],[1295,76,2],
[1296,16,1],[1296,36,1],[1297,19,1],[1297,35,1],[1298,3,1],
[1298,31,1],[1299,2,1],[1299,28,3],[1300,23,1],[1300,30,2],
[1301,15,1],[1301,24,1],[1301,56,1],[1302,5,1],[1302,44,1],
[1303,26,1],[1303,32,4],[1304,2,1],[1304,21,1],[1305,27,3],
[1305,40,1],[1306,1,1],[1306,45,1],[1307,1,2],[1307,3,1],[1307,7,1],
[1307,8,6],[1307,25,13],[1307,28,9],[1307,30,2],[1307,31,1],
[1307,32,11],[1307,35,36],[1307,38,1],[1307,56,1],[1307,62,1],
[1307,72,1],[1307,79,1],[1308,1,2],[1308,4,1],[1308,7,1],[1308,9,1],
[1308,12,2],[1308,15,1],[1308,17,114],[1308,29,1],[1308,45,1],
[1308,50,3],[1308,53,48],[1308,55,4],[1308,73,1],[1309,0,1],
[1309,1,1],[1309,9,2],[1309,10,2],[1309,15,6],[1309,28,1],[1309,36,1],
[1309,53,3],[1309,55,6],[1309,67,1],[1310,18,1],[1310,29,1],
[1310,36,1],[1310,39,8],[1311,3,1],[1311,5,2],[1311,23,3],[1311,24,1],
[1311,25,3],[1311,26,1],[1311,27,1],[1311,28,1],[1311,31,1],
[1311,35,6],[1311,62,1],[1312,3,2],[1312,14,1],[1312,23,1],
[1312,24,2],[1312,26,1],[1312,30,1],[1312,31,15],[1312,32,1],
[1312,33,3],[1312,38,1],[1312,67,1],[1313,38,1],[1313,48,8],
[1314,3,1],[1314,31,2],[1315,20,3],[1316,1,1],[1316,4,1],[1316,6,1],
[1316,9,1],[1316,10,1],[1316,12,2],[1316,13,1],[1316,15,1],
[1316,17,36],[1316,20,1],[1316,22,1],[1316,24,1],[1316,27,1],
[1316,28,7],[1316,30,1],[1316,32,3],[1316,33,2],[1316,38,1],
[1316,39,1],[1316,40,1],[1316,42,2],[1316,44,1],[1316,45,1],
[1316,50,2],[1316,53,21],[1316,66,2],[1316,68,2],[1316,70,3],
[1316,74,2],[1316,76,1],[1317,13,1],[1317,23,1],[1317,26,1],
[1317,32,1],[1317,33,2],[1317,35,1],[1317,37,1],[1317,43,1],
[1318,28,1],[1318,32,2],[1318,43,1],[1319,9,1],[1319,39,1],
[1320,40,2],[1321,25,2],[1322,1,2],[1322,8,1],[1322,9,2],[1322,12,6],
[1322,16,6],[1322,29,1],[1322,39,1],[1323,3,1],[1323,6,1],[1323,23,2],
[1323,24,27],[1323,28,1],[1323,38,1],[1323,56,9],[1323,68,1],
[1323,70,1],[1324,0,1],[1324,1,4],[1324,8,1],[1324,9,1],[1324,10,1],
[1324,12,2],[1324,13,23],[1324,16,2],[1324,48,4],[1324,49,1],
[1325,28,1],[1325,31,1],[1326,2,1],[1326,5,2],[1326,28,1],[1326,70,1],
[1327,25,1],[1327,28,1],[1327,56,1],[1328,0,3],[1328,1,1],[1328,3,1],
[1328,8,44],[1328,11,1],[1328,12,2],[1328,15,2],[1328,17,2],
[1328,19,1],[1328,20,2],[1328,22,50],[1328,23,30],[1328,24,34],
[1328,25,1],[1328,27,26],[1328,28,14],[1328,29,3],[1328,30,3],
[1328,34,19],[1328,35,12],[1328,36,6],[1328,37,1],[1328,54,1],
[1328,55,1],[1328,62,1],[1328,70,1],[1328,71,1],[1328,76,1],
[1328,79,1],[1329,1,2],[1329,4,18],[1329,23,2],[1329,27,1],
[1329,28,7],[1329,31,1],[1329,33,1],[1329,35,1],[1329,38,1],
[1329,40,2],[1329,44,48],[1329,47,1],[1329,50,17],[1329,56,1],

[1329,59,1],[1329,66,36],[1329,69,1],[1329,70,2],[1329,76,2],
[1330,6,2],[1330,26,1],[1330,32,8],[1330,33,4],[1330,35,11],
[1330,38,1],[1330,44,1],[1330,48,1],[1330,50,2],[1330,56,3],
[1331,39,2],[1332,25,1],[1332,26,1],[1332,27,1],[1332,28,2],
[1332,74,1],[1333,28,1],[1333,31,1],[1333,47,1],[1334,3,1],
[1334,25,1],[1334,27,1],[1334,62,3],[1335,0,29],[1335,1,109],
[1335,5,1],[1335,7,40],[1335,8,39],[1335,9,213],[1335,10,415],
[1335,11,151],[1335,12,153],[1335,13,86],[1335,14,115],[1335,15,107],
[1335,16,220],[1335,17,47],[1335,18,108],[1335,19,324],[1335,20,87],
[1335,25,59],[1335,29,27],[1335,30,24],[1335,34,88],[1335,36,61],
[1335,37,114],[1335,39,42],[1335,41,178],[1335,45,188],[1335,46,145],
[1335,49,103],[1335,53,19],[1335,54,88],[1335,55,75],[1335,57,175],
[1335,58,144],[1335,59,144],[1335,60,32],[1335,64,44],[1335,65,263],
[1335,67,126],[1335,68,1],[1335,71,101],[1335,73,96],[1335,77,157],
[1335,78,33],[1336,13,1],[1336,67,1],[1337,28,25],[1338,4,2],
[1338,11,1],[1338,13,1],[1338,28,5],[1338,32,1],[1338,44,1],
[1338,50,1],[1338,56,1],[1339,27,2],[1339,34,2],[1340,1,17],
[1340,7,1],[1340,12,12],[1340,17,1],[1340,39,1],[1341,12,1],
[1341,20,1],[1341,34,1],[1341,65,1],[1341,74,1],[1341,77,1],
[1342,2,5],[1342,13,1],[1342,20,2],[1343,2,6],[1344,2,1],[1344,21,2],
[1345,24,1],[1345,27,1],[1345,28,21],[1346,22,1],[1346,31,5],
[1347,2,1],[1347,3,1],[1348,2,2],[1349,1,1],[1349,2,7],[1349,3,8],
[1349,5,11],[1349,6,7],[1349,8,2],[1349,9,4],[1349,11,1],[1349,12,4],
[1349,13,26],[1349,15,3],[1349,17,1],[1349,19,1],[1349,20,143],
[1349,21,11],[1349,22,90],[1349,23,40],[1349,24,36],[1349,25,27],
[1349,26,24],[1349,27,75],[1349,28,23],[1349,29,2],[1349,30,5],
[1349,31,11],[1349,32,18],[1349,33,6],[1349,34,2],[1349,35,52],
[1349,36,2],[1349,37,1],[1349,38,7],[1349,39,2],[1349,40,1],
[1349,41,1],[1349,42,19],[1349,43,20],[1349,44,1],[1349,45,3],
[1349,47,9],[1349,48,14],[1349,50,1],[1349,52,4],[1349,53,1],
[1349,54,2],[1349,57,2],[1349,58,1],[1349,59,3],[1349,62,2],
[1349,64,1],[1349,65,3],[1349,66,3],[1349,67,2],[1349,70,7],
[1349,71,2],[1349,73,1],[1349,74,2],[1349,75,1],[1349,76,1],
[1349,78,1],[1349,79,1],[1350,2,1],[1350,8,1],[1350,30,1],[1351,51,5],
[1352,15,1],[1352,55,2],[1353,3,1],[1353,32,1],[1354,33,3],
[1355,15,1],[1355,30,1],[1355,37,1],[1355,57,1],[1355,64,1],
[1356,2,2],[1356,3,1],[1356,6,1],[1356,21,1],[1356,24,1],[1356,25,2],
[1356,26,1],[1356,27,5],[1356,28,2],[1356,32,5],[1356,35,1],
[1356,43,2],[1357,48,1],[1357,74,1],[1358,21,1],[1358,24,1],
[1358,26,1],[1358,31,2],[1359,9,2],[1359,10,1],[1360,6,2],[1361,26,1],
[1361,35,1],[1362,0,1],[1362,2,3],[1362,30,6],[1362,39,1],[1362,46,3],
[1362,65,1],[1363,7,2],[1363,68,1],[1364,9,1],[1364,17,1],[1365,9,1],
[1365,17,1],[1365,28,7],[1365,55,1],[1365,66,1],[1366,5,2],
[1366,26,1],[1366,49,1],[1366,62,1],[1367,2,1],[1367,47,1],[1368,5,1],
[1368,27,1],[1368,28,8],[1368,32,1],[1368,33,1],[1368,48,2],
[1369,5,2],[1370,43,2],[1371,1,1],[1371,4,1],[1371,21,1],[1371,26,1],
[1371,31,1],[1371,33,1],[1371,62,68],[1372,9,1],[1372,15,6],
[1372,25,1],[1372,55,14],[1373,21,1],[1373,24,1],[1373,25,1],
[1374,1,22],[1374,2,1],[1374,12,1],[1374,13,2],[1374,20,1],
[1374,28,1],[1374,29,2],[1374,47,1],[1375,2,4],[1375,20,1],

[1375,22,1],[1375,28,1],[1375,33,1],[1375,51,2],[1375,53,1],
[1375,65,1],[1376,62,2],[1377,0,10],[1377,2,4],[1377,12,1],
[1377,31,1],[1377,50,1],[1378,33,9],[1378,50,1],[1379,9,1],
[1379,17,1],[1380,6,1],[1380,24,2],[1380,27,1],[1380,35,1],
[1380,43,1],[1381,28,6],[1382,5,1],[1382,24,1],[1382,35,1],
[1382,47,1],[1382,56,1],[1382,72,1],[1383,24,1],[1383,28,1],
[1384,2,5],[1384,28,1],[1384,35,1],[1385,63,1],[1385,68,1],
[1386,22,1],[1386,31,1],[1386,42,1],[1386,44,1],[1386,50,1],
[1386,51,1],[1387,2,1],[1387,20,1],[1387,30,1],[1387,42,2],
[1387,47,14],[1388,21,2],[1389,28,25],[1389,68,1],[1390,20,5],
[1390,21,3],[1390,22,2],[1390,23,2],[1390,26,1],[1390,27,3],
[1390,31,2],[1390,36,1],[1390,39,1],[1390,42,1],[1391,17,1],
[1391,39,1],[1391,53,1],[1392,9,1],[1392,13,1],[1392,30,4],
[1392,67,1],[1392,78,1],[1393,22,1],[1393,23,1],[1393,25,9],
[1393,27,1],[1393,28,4],[1393,33,14],[1393,35,35],[1393,48,1],
[1393,52,1],[1394,8,1],[1394,9,1],[1394,37,1],[1394,65,1],[1395,2,1],
[1395,19,1],[1396,5,1],[1396,23,1],[1397,22,1],[1397,35,1],[1398,4,1],
[1398,5,1],[1398,9,1],[1398,11,2],[1398,15,2],[1398,17,20],
[1398,46,1],[1398,50,2],[1398,53,13],[1398,55,1],[1398,71,1],
[1399,4,3],[1399,38,2],[1399,44,20],[1399,50,1],[1399,51,1],
[1399,66,10],[1399,74,1],[1400,17,1],[1400,20,1],[1401,5,3],
[1401,9,1],[1401,50,1],[1402,2,10],[1402,3,1],[1402,4,1],[1402,5,2],
[1402,6,1],[1402,8,1],[1402,13,1],[1402,20,2],[1402,21,1],[1402,25,8],
[1402,26,3],[1402,27,5],[1402,31,2],[1402,33,3],[1402,34,2],
[1402,35,3],[1402,38,1],[1402,42,3],[1402,44,1],[1402,56,2],
[1402,72,1],[1403,20,3],[1403,21,8],[1403,22,5],[1403,23,2],
[1403,25,3],[1403,27,4],[1403,35,1],[1403,59,1],[1403,62,1],
[1403,73,1],[1404,4,1],[1404,70,1],[1405,45,1],[1405,49,1],
[1405,57,1],[1405,58,1],[1405,64,1],[1406,2,3],[1406,3,1],[1406,20,1],
[1406,23,1],[1406,51,1],[1406,53,1],[1406,60,1],[1407,7,1],
[1407,10,1],[1407,12,1],[1407,16,1],[1407,19,1],[1407,45,1],
[1407,46,1],[1407,54,1],[1407,58,1],[1407,71,2],[1408,24,1],
[1408,25,2],[1408,26,3],[1408,62,2],[1409,11,1],[1409,60,1],
[1410,31,3],[1411,4,6],[1411,5,2],[1411,10,1],[1411,17,3],[1411,19,1],
[1411,22,1],[1411,45,1],[1411,50,7],[1411,53,2],[1411,55,1],
[1411,65,1],[1411,73,1],[1411,74,1],[1411,76,1],[1412,2,19],
[1412,47,1],[1413,15,1],[1413,38,1],[1414,4,2],[1414,44,2],
[1414,51,1],[1414,66,4],[1414,68,1],[1415,12,2],[1416,23,1],
[1416,26,1],[1416,27,5],[1416,35,5],[1416,72,1],[1417,1,1],[1417,3,1],
[1417,13,2],[1417,28,3],[1417,48,3],[1418,3,1],[1418,27,3],
[1418,33,1],[1418,43,1],[1419,25,1],[1419,74,1],[1420,12,1],
[1420,40,1],[1420,63,1],[1420,76,4],[1421,2,1],[1421,28,1],
[1422,31,1],[1422,42,4],[1423,3,1],[1423,21,2],[1423,22,1],
[1423,23,1],[1423,28,1],[1423,35,1],[1424,27,2],[1425,32,2],
[1426,2,1],[1426,23,1],[1426,26,1],[1426,27,1],[1426,31,1],
[1426,35,1],[1426,40,1],[1426,54,1],[1426,62,2],[1427,1,1],[1427,7,1],
[1427,14,1],[1427,55,1],[1428,28,1],[1428,63,4],[1428,74,5],
[1428,75,5],[1428,76,12],[1429,3,1],[1429,24,6],[1429,63,1],
[1429,70,1],[1430,22,1],[1430,31,1],[1431,72,3],[1432,3,1],
[1432,15,2],[1432,20,1],[1432,26,1],[1432,27,1],[1432,32,1],

[1432,36,1],[1432,44,1],[1432,46,1],[1432,50,2],[1432,55,2],
[1432,56,2],[1432,67,1],[1432,69,1],[1432,70,2],[1432,77,1],
[1433,6,1],[1433,24,1],[1433,25,1],[1433,27,2],[1433,28,11],
[1433,33,3],[1433,35,1],[1433,37,1],[1433,46,1],[1433,48,1],
[1433,52,1],[1433,56,1],[1433,75,1],[1433,76,1],[1434,6,1],
[1434,26,3],[1434,27,4],[1434,38,1],[1434,43,3],[1435,24,2],
[1435,31,1],[1435,35,1],[1436,30,1],[1436,35,2],[1436,66,1],
[1437,22,1],[1437,23,1],[1437,24,1],[1437,31,1],[1437,55,1],
[1437,74,2],[1438,35,1],[1438,63,3],[1438,74,3],[1438,75,4],
[1438,76,7],[1439,13,1],[1439,15,2],[1439,20,6],[1439,21,1],
[1439,23,1],[1439,24,1],[1439,27,1],[1439,35,1],[1439,50,1],
[1439,55,3],[1439,70,1],[1439,72,1],[1440,32,1],[1440,44,1],
[1440,50,1],[1440,51,4],[1440,66,2],[1440,69,1],[1441,0,1],[1441,6,4],
[1441,23,1],[1441,32,1],[1441,45,1],[1441,47,1],[1441,62,1],
[1441,63,1],[1441,66,1],[1441,68,1],[1441,70,1],[1441,79,1],
[1442,0,1],[1442,1,1],[1442,8,1],[1442,9,1],[1443,23,1],[1443,24,1],
[1443,35,1],[1443,56,1],[1444,1,1],[1444,2,1],[1444,5,2],[1444,9,3],
[1444,28,21],[1444,30,1],[1444,43,1],[1444,50,1],[1444,61,1],
[1445,28,5],[1446,2,4],[1446,5,1],[1446,47,1],[1447,43,1],[1447,47,1],
[1447,64,1],[1447,69,1],[1448,3,2],[1448,28,2],[1448,37,1],
[1448,47,1],[1448,56,3],[1448,60,1],[1449,6,2],[1449,23,2],
[1449,26,2],[1449,27,1],[1449,28,4],[1449,42,1],[1449,47,5],
[1449,56,1],[1450,4,2],[1450,15,1],[1450,16,1],[1450,24,1],
[1450,26,1],[1450,31,1],[1450,34,1],[1450,37,1],[1450,38,1],
[1450,41,1],[1450,50,1],[1450,54,1],[1450,55,1],[1450,58,1],
[1450,59,1],[1450,60,1],[1450,67,1],[1450,70,1],[1450,75,1],
[1450,77,1],[1451,22,1],[1451,25,2],[1451,28,1],[1451,38,2],
[1451,52,18],[1452,5,1],[1452,28,1],[1452,32,1],[1452,35,1],
[1453,2,1],[1453,27,1],[1453,28,1],[1453,31,1],[1454,28,4],
[1455,23,1],[1455,51,1],[1455,72,1],[1456,2,1],[1456,21,1],
[1456,28,1],[1457,3,1],[1457,24,1],[1458,3,1],[1458,22,1],[1458,47,1],
[1459,9,2],[1459,20,2],[1459,27,3],[1459,28,3],[1460,28,4],
[1460,42,2],[1461,28,2],[1462,5,1],[1462,32,2],[1463,23,1],
[1463,24,16],[1463,31,3],[1463,76,1],[1464,31,4],[1464,33,1],
[1465,2,1],[1465,20,1],[1465,23,1],[1465,27,1],[1465,31,2],
[1465,47,1],[1465,56,2],[1465,62,1],[1465,68,1],[1466,22,2],
[1466,31,2],[1466,32,1],[1467,22,1],[1467,23,1],[1467,52,1],
[1467,75,1],[1467,76,3],[1468,20,1],[1468,24,1],[1468,35,1],
[1469,4,2],[1469,15,1],[1469,16,1],[1469,17,5],[1469,25,1],
[1469,50,1],[1469,53,8],[1469,55,1],[1470,63,1],[1470,73,1],
[1471,20,1],[1471,31,1],[1472,6,1],[1472,32,1],[1472,33,1],
[1472,56,2],[1473,4,10],[1473,28,2],[1473,32,1],[1473,50,9],
[1474,5,1],[1474,35,1],[1474,70,1],[1475,8,1],[1475,36,1],[1475,77,1],
[1476,4,1],[1476,5,1],[1476,21,1],[1476,22,1],[1476,24,2],[1476,25,2],
[1476,27,2],[1476,28,19],[1476,31,1],[1476,32,2],[1476,37,1],
[1476,38,2],[1476,40,2],[1476,42,1],[1476,44,1],[1476,50,2],
[1476,56,1],[1476,63,2],[1476,66,1],[1476,68,1],[1476,70,6],
[1476,72,1],[1476,74,2],[1476,75,1],[1476,76,5],[1477,10,1],
[1477,27,1],[1478,28,2],[1478,48,2],[1479,31,1],[1479,42,6],
[1480,2,1],[1480,3,1],[1480,24,2],[1480,25,3],[1480,31,1],[1480,33,1],

[1480,37,1],[1480,42,1],[1480,43,1],[1480,56,1],[1481,2,27],
[1481,19,1],[1481,31,23],[1481,39,11],[1481,42,1],[1481,47,1],
[1481,63,1],[1481,72,1],[1482,22,1],[1482,26,2],[1483,1,1],[1483,2,2],
[1483,3,2],[1483,8,1],[1483,12,1],[1483,13,3],[1483,19,1],[1483,20,1],
[1483,22,1],[1483,23,2],[1483,24,11],[1483,25,1],[1483,27,2],
[1483,29,1],[1483,32,2],[1483,44,1],[1483,55,1],[1483,56,2],
[1483,62,1],[1483,64,2],[1483,69,2],[1483,73,1],[1483,74,1],
[1484,37,4],[1485,33,2],[1486,2,5],[1486,28,1],[1486,35,1],
[1486,39,1],[1486,62,2],[1487,7,1],[1487,12,1],[1487,16,2],
[1487,27,1],[1487,42,2],[1487,51,2],[1487,70,5],[1488,15,1],
[1488,23,1],[1488,47,1],[1489,26,1],[1489,28,2],[1489,43,1],
[1489,44,1],[1489,50,1],[1490,10,1],[1490,15,4],[1490,41,1],
[1490,55,3],[1490,63,1],[1490,74,1],[1491,37,4],[1492,39,1],
[1492,59,1],[1493,47,2],[1494,30,1],[1494,32,1],[1494,66,1],
[1495,7,2],[1495,12,1],[1495,18,1],[1495,73,1],[1496,32,2],
[1496,43,1],[1497,24,1],[1497,28,1],[1498,9,1],[1498,20,1],[1499,1,1],
[1499,3,1],[1499,4,1],[1499,37,1],[1499,42,13],[1499,48,1],
[1500,32,27],[1500,42,1],[1501,27,2],[1501,35,1],[1502,3,1],
[1502,12,1],[1502,22,1],[1502,24,1],[1502,30,1],[1502,56,1],
[1502,72,1],[1502,75,1],[1503,20,1],[1503,26,2],[1503,35,2],
[1504,1,68],[1504,10,1],[1504,13,8],[1504,20,2],[1504,29,1],
[1504,41,1],[1504,55,1],[1504,58,1],[1504,73,1],[1505,64,2],
[1506,1,5],[1506,3,2],[1506,8,2],[1506,9,1],[1506,13,1],[1506,19,1],
[1506,22,3],[1506,23,5],[1506,24,9],[1506,26,1],[1506,27,2],
[1506,28,6],[1506,29,1],[1506,30,2],[1506,32,1],[1506,35,3],
[1506,43,5],[1506,44,1],[1506,45,1],[1506,46,1],[1506,59,4],
[1506,65,1],[1506,67,2],[1506,70,1],[1507,0,1],[1507,1,1],[1507,6,1],
[1507,9,2],[1507,10,2],[1507,15,9],[1507,19,2],[1507,20,1],
[1507,27,1],[1507,33,1],[1507,35,1],[1507,37,1],[1507,40,2],
[1507,44,1],[1507,47,1],[1507,50,2],[1507,55,8],[1507,59,1],
[1507,63,1],[1507,65,1],[1507,66,1],[1507,69,1],[1507,74,1],
[1507,75,2],[1507,76,1],[1508,28,1],[1508,33,1],[1509,1,1],
[1509,2,10],[1509,3,4],[1509,5,1],[1509,9,1],[1509,19,1],[1509,20,3],
[1509,21,1],[1509,23,2],[1509,24,4],[1509,27,5],[1509,28,2],
[1509,30,2],[1509,31,3],[1509,32,2],[1509,33,1],[1509,36,1],
[1509,39,4],[1509,43,1],[1509,47,1],[1509,53,1],[1510,2,1],
[1510,22,1],[1510,28,2],[1510,33,4],[1510,40,1],[1511,43,1],
[1511,70,1],[1512,61,1],[1512,72,1],[1513,36,1],[1513,63,1],
[1513,76,1],[1514,50,1],[1514,66,1],[1515,24,5],[1516,46,1],
[1516,59,1],[1517,21,1],[1517,23,3],[1518,23,2],[1518,33,2],
[1518,35,1],[1519,3,1],[1519,25,1],[1520,18,1],[1520,22,1],
[1520,39,1],[1520,43,2],[1521,63,1],[1521,75,1],[1521,76,1],
[1522,1,1],[1522,12,1],[1522,30,1],[1522,39,1],[1523,10,1],
[1523,11,1],[1523,12,1],[1523,17,1],[1523,20,1],[1523,36,1],
[1523,39,1],[1523,55,1],[1523,78,1],[1524,20,2],[1524,78,1],
[1525,5,1],[1525,68,1],[1526,15,5],[1526,23,1],[1526,31,1],
[1526,45,1],[1526,55,11],[1526,59,1],[1526,60,1],[1526,71,1],
[1527,5,1],[1527,22,3],[1527,23,3],[1527,27,6],[1528,28,4],[1529,0,3],
[1529,1,11],[1529,7,4],[1529,9,6],[1529,10,6],[1529,11,3],[1529,12,6],
[1529,13,4],[1529,14,2],[1529,15,2],[1529,16,3],[1529,17,5],

[1529,18,5],[1529,19,5],[1529,20,5],[1529,28,4],[1529,29,4],
[1529,30,6],[1529,31,1],[1529,32,1],[1529,36,2],[1529,37,2],
[1529,39,5],[1529,41,1],[1529,45,3],[1529,46,3],[1529,53,3],
[1529,54,3],[1529,55,5],[1529,57,2],[1529,58,5],[1529,59,4],
[1529,63,1],[1529,64,2],[1529,65,12],[1529,67,4],[1529,70,1],
[1529,71,4],[1529,73,3],[1529,74,2],[1529,76,1],[1529,77,3],
[1529,78,1],[1530,3,1],[1530,23,1],[1530,28,1],[1530,32,2],
[1530,47,1],[1531,7,6],[1532,28,18],[1532,76,2],[1533,2,1],[1533,3,2],
[1533,21,1],[1533,23,1],[1533,24,1],[1533,27,2],[1533,28,1],
[1533,40,1],[1533,44,2],[1533,47,1],[1533,56,1],[1533,62,1],
[1533,66,2],[1533,69,2],[1533,70,2],[1534,22,1],[1534,23,1],
[1534,27,8],[1534,28,1],[1534,38,3],[1534,44,1],[1534,63,2],
[1534,68,1],[1534,69,1],[1534,70,5],[1534,72,2],[1534,75,1],
[1535,2,2],[1536,7,1],[1536,9,1],[1537,2,2],[1537,62,3],[1538,2,1],
[1538,27,1],[1539,1,1],[1539,2,3],[1539,12,4],[1539,48,2],[1540,0,3],
[1540,2,1],[1541,9,1],[1541,49,1],[1542,2,3],[1542,28,1],[1543,28,16],
[1543,68,1],[1544,31,4],[1545,16,1],[1545,43,1],[1546,24,1],
[1546,28,4],[1546,60,1],[1547,23,1],[1547,28,1],[1547,33,1],
[1548,6,2],[1549,22,1],[1549,28,1],[1550,6,1],[1550,24,2],[1550,47,1],
[1550,75,1],[1551,28,4],[1551,35,1],[1551,75,1],[1552,47,1],
[1552,50,1],[1553,0,1],[1553,2,1],[1553,5,1],[1553,20,3],[1553,22,2],
[1553,23,3],[1553,24,3],[1553,26,2],[1553,27,5],[1553,28,3],
[1553,32,2],[1553,35,2],[1553,36,1],[1553,39,1],[1553,42,2],
[1553,43,1],[1553,47,1],[1553,48,3],[1553,76,1],[1554,1,1],
[1554,10,1],[1554,12,1],[1554,16,1],[1554,20,7],[1554,22,10],
[1554,23,1],[1554,25,1],[1554,27,1],[1554,32,2],[1554,33,1],
[1554,35,2],[1555,12,1],[1555,17,1],[1555,55,2],[1556,27,1],
[1556,28,1],[1557,28,8],[1557,70,1],[1558,23,1],[1558,47,1],
[1559,21,4],[1559,28,1],[1559,30,1],[1560,15,2],[1560,66,1],
[1561,24,2],[1561,62,3],[1562,55,2],[1563,15,1],[1563,53,1],
[1564,3,1],[1564,5,2],[1564,15,1],[1564,20,1],[1564,25,1],[1564,74,1],
[1565,5,1],[1565,28,2],[1565,32,1],[1566,20,1],[1566,23,5],
[1566,27,1],[1566,28,2],[1566,32,1],[1566,35,1],[1566,43,1],
[1567,15,2],[1567,22,2],[1567,23,5],[1567,24,7],[1567,27,1],
[1567,32,2],[1567,34,1],[1567,35,1],[1567,50,1],[1567,55,1],
[1567,59,1],[1567,65,1],[1567,66,1],[1567,68,1],[1567,73,1],
[1568,5,1],[1568,22,1],[1568,23,2],[1568,26,3],[1568,27,2],
[1568,33,3],[1568,35,4],[1568,38,1],[1569,13,1],[1569,59,1],
[1569,75,1],[1570,27,2],[1571,44,2],[1572,28,3],[1573,0,1],[1573,4,8],
[1573,15,10],[1573,17,13],[1573,23,1],[1573,28,1],[1573,31,2],
[1573,44,21],[1573,50,25],[1573,53,8],[1573,55,1],[1573,62,2],
[1573,63,1],[1573,66,14],[1573,70,1],[1573,74,1],[1573,79,1],
[1574,10,1],[1574,14,1],[1574,18,1],[1575,28,1],[1575,62,3],
[1576,21,2],[1576,23,1],[1576,26,4],[1576,27,1],[1576,31,4],
[1576,43,1],[1577,3,1],[1577,22,1],[1577,23,1],[1577,26,1],
[1577,27,2],[1577,34,1],[1577,35,1],[1577,37,1],[1577,47,1],
[1577,70,1],[1578,0,1],[1578,8,1],[1578,10,1],[1578,15,1],[1578,59,1],
[1579,5,1],[1579,42,1],[1580,27,1],[1580,28,2],[1581,12,1],
[1581,25,1],[1581,26,1],[1581,27,1],[1581,30,1],[1581,32,3],
[1581,44,1],[1581,45,1],[1581,66,1],[1581,76,1],[1581,79,1],

[1582,6,1],[1582,24,1],[1583,2,2],[1583,3,5],[1583,43,2],[1583,47,1],
[1583,59,1],[1584,10,1],[1584,56,3],[1585,1,1],[1585,33,1],
[1586,25,1],[1586,51,1],[1587,14,1],[1587,45,1],[1587,60,6],
[1588,4,1],[1588,23,1],[1588,24,1],[1588,27,1],[1588,50,1],
[1589,12,1],[1589,14,2],[1589,41,1],[1589,49,1],[1589,59,1],
[1590,27,1],[1590,28,1],[1591,45,3],[1592,2,2],[1592,5,1],[1592,22,1],
[1592,48,1],[1593,9,1],[1593,53,1],[1594,12,2],[1595,27,1],
[1595,28,1],[1596,4,1],[1596,27,2],[1597,1,2],[1597,9,1],[1597,12,1],
[1597,14,1],[1597,19,1],[1597,28,1],[1597,49,1],[1597,55,1],
[1597,58,1],[1597,65,1],[1598,10,1],[1598,39,1],[1598,77,2],
[1599,15,1],[1599,20,2],[1599,23,1],[1599,38,1],[1599,65,1],
[1599,68,1],[1600,11,1],[1600,17,1],[1600,20,1],[1601,33,3],
[1601,35,5],[1601,49,1],[1601,52,7],[1602,8,4],[1602,9,1],[1602,20,7],
[1602,23,4],[1602,24,1],[1602,27,2],[1602,31,1],[1602,32,2],
[1602,34,5],[1602,36,1],[1602,45,1],[1602,52,1],[1602,59,1],
[1603,20,1],[1603,67,1],[1604,15,4],[1604,45,1],[1604,55,9],
[1604,67,1],[1605,3,1],[1605,6,1],[1605,24,3],[1605,47,1],[1606,1,1],
[1606,5,3],[1606,9,2],[1606,10,1],[1606,17,1],[1606,22,1],[1606,43,1],
[1606,58,1],[1606,59,1],[1606,63,43],[1606,67,1],[1606,69,2],
[1606,70,3],[1606,74,26],[1606,75,25],[1606,76,55],[1607,2,6],
[1607,5,1],[1607,21,1],[1607,23,4],[1607,24,1],[1607,25,1],
[1607,31,1],[1607,32,5],[1607,47,4],[1607,62,1],[1608,46,2],
[1609,9,1],[1609,10,1],[1609,15,1],[1609,31,1],[1609,37,1],
[1610,32,2],[1611,31,2],[1612,0,2],[1612,1,3],[1612,3,2],[1612,4,1],
[1612,9,1],[1612,13,1],[1612,14,1],[1612,17,1],[1612,20,2],
[1612,21,1],[1612,22,9],[1612,23,3],[1612,24,4],[1612,26,1],
[1612,27,2],[1612,30,2],[1612,31,3],[1612,34,4],[1612,35,1],
[1612,36,1],[1612,38,1],[1612,43,2],[1612,59,2],[1613,12,2],
[1614,28,2],[1614,56,1],[1614,76,1],[1615,9,2],[1616,1,1],[1616,8,2],
[1616,9,1],[1616,10,1],[1616,12,2],[1616,15,1],[1616,16,1],
[1616,19,1],[1616,20,1],[1616,39,2],[1616,65,1],[1617,9,1],
[1617,12,1],[1617,19,1],[1617,20,1],[1617,36,1],[1618,44,2],
[1619,0,2],[1619,7,3],[1619,18,1],[1619,19,1],[1619,53,1],[1619,65,2],
[1620,22,1],[1620,28,1],[1621,38,10],[1621,40,1],[1622,4,1],
[1622,42,1],[1623,23,1],[1623,68,1],[1624,30,2],[1625,3,1],[1625,4,1],
[1625,5,1],[1625,6,2],[1625,22,1],[1625,24,1],[1625,27,1],[1625,28,2],
[1625,35,2],[1625,44,2],[1625,56,14],[1626,21,1],[1626,31,4],
[1627,24,1],[1627,69,1],[1628,23,1],[1628,32,1],[1629,62,2],
[1630,0,2],[1630,1,3],[1630,8,2],[1630,9,29],[1630,12,1],[1630,13,1],
[1630,17,1],[1630,26,1],[1630,35,1],[1630,39,4],[1630,47,15],
[1630,66,1],[1630,73,1],[1630,78,13],[1631,3,7],[1631,23,1],
[1631,24,22],[1631,26,3],[1631,28,7],[1631,47,1],[1632,20,3],
[1633,3,1],[1633,35,1],[1634,0,1],[1634,12,1],[1634,13,1],[1634,14,1],
[1634,49,1],[1634,58,1],[1634,77,2],[1634,78,1],[1635,0,5],[1635,1,3],
[1635,7,3],[1635,9,1],[1635,10,2],[1635,12,1],[1635,13,1],[1635,15,2],
[1635,16,1],[1635,20,2],[1635,22,1],[1635,23,21],[1635,24,1],
[1635,25,7],[1635,27,3],[1635,31,1],[1635,34,4],[1635,36,1],
[1635,37,1],[1635,39,7],[1635,54,1],[1635,57,1],[1635,59,1],
[1635,62,1],[1635,67,1],[1635,77,1],[1636,48,3],[1637,4,1],
[1637,15,1],[1637,44,1],[1637,55,1],[1637,66,1],[1638,59,2],

[1639,4,1],[1639,17,1],[1639,22,1],[1639,44,1],[1639,49,1],
[1639,50,2],[1639,63,1],[1639,66,1],[1640,5,1],[1640,8,1],[1641,0,1],
[1641,1,8],[1641,2,1],[1641,3,3],[1641,4,1],[1641,5,1],[1641,6,2],
[1641,8,4],[1641,9,15],[1641,10,6],[1641,11,3],[1641,12,8],
[1641,13,9],[1641,14,3],[1641,15,12],[1641,16,5],[1641,17,1],
[1641,19,3],[1641,20,13],[1641,24,4],[1641,25,2],[1641,26,4],
[1641,27,2],[1641,28,75],[1641,29,7],[1641,30,1],[1641,31,3],
[1641,32,1],[1641,33,1],[1641,34,1],[1641,35,2],[1641,37,4],
[1641,39,4],[1641,40,2],[1641,41,4],[1641,43,2],[1641,44,9],
[1641,45,5],[1641,46,1],[1641,47,1],[1641,49,4],[1641,50,1],
[1641,53,2],[1641,55,8],[1641,56,1],[1641,57,4],[1641,59,12],
[1641,63,5],[1641,65,4],[1641,66,4],[1641,67,3],[1641,68,3],
[1641,69,1],[1641,70,6],[1641,72,4],[1641,73,1],[1641,75,4],
[1641,76,3],[1641,77,4],[1641,78,1],[1642,6,1],[1642,24,2],
[1642,68,1],[1643,50,2],[1643,55,1],[1644,23,2],[1644,27,3],
[1644,28,1],[1645,2,7],[1645,3,6],[1645,4,12],[1645,5,2576],
[1645,6,4],[1645,13,1],[1645,21,2],[1645,22,80],[1645,23,14],
[1645,24,23],[1645,25,20],[1645,26,16],[1645,27,15],[1645,28,607],
[1645,31,9],[1645,32,75],[1645,33,24],[1645,35,149],[1645,36,1],
[1645,38,15],[1645,40,43],[1645,42,6],[1645,43,39],[1645,44,13],
[1645,47,6],[1645,48,7],[1645,50,19],[1645,51,1],[1645,52,8],
[1645,56,6],[1645,59,4],[1645,61,1],[1645,62,7],[1645,63,17],
[1645,66,9],[1645,68,23],[1645,69,10],[1645,70,104],[1645,72,41],
[1645,74,13],[1645,75,14],[1645,76,22],[1646,22,1],[1646,24,2],
[1646,26,1],[1646,35,1],[1646,56,2],[1646,70,1],[1647,10,3],
[1647,18,1],[1648,23,5],[1648,33,2],[1648,35,1],[1649,25,1],
[1649,27,1],[1649,56,1],[1650,28,1],[1650,33,13],[1650,35,12],
[1651,5,1],[1651,15,1],[1651,24,1],[1651,26,1],[1651,27,1],
[1651,33,1],[1651,36,1],[1651,43,1],[1651,53,1],[1652,7,1],
[1652,10,1],[1652,11,3],[1653,0,1],[1653,19,1],[1653,30,1],
[1653,55,1],[1654,27,2],[1654,62,1],[1655,3,1],[1655,22,1],
[1655,23,3],[1655,26,2],[1655,33,1],[1655,35,2],[1656,2,1],
[1656,10,2],[1656,20,1],[1656,24,1],[1656,27,1],[1656,28,1],
[1656,31,1],[1657,28,1],[1657,42,1],[1657,43,2],[1657,76,1],
[1658,10,1],[1658,24,1],[1658,32,1],[1658,39,1],[1658,42,4],
[1659,5,2],[1659,24,1],[1659,26,1],[1659,27,2],[1659,28,2],
[1659,31,1],[1659,32,1],[1659,35,1],[1659,40,3],[1659,44,1],
[1659,47,1],[1659,63,49],[1659,66,2],[1659,68,1],[1659,70,1],
[1659,74,29],[1659,75,21],[1659,76,58],[1660,15,1],[1660,17,1],
[1660,53,1],[1660,55,1],[1661,7,2],[1661,12,1],[1661,14,1],
[1661,19,1],[1661,34,1],[1662,3,1],[1662,5,1],[1662,23,2],[1662,56,1],
[1663,28,1],[1663,55,1],[1664,3,9],[1664,5,2],[1664,25,2],[1664,26,1],
[1664,28,1],[1664,32,1],[1664,33,1],[1664,44,1],[1664,47,1],
[1664,48,8],[1665,1,1],[1665,9,3],[1665,10,4],[1665,11,1],[1665,12,1],
[1665,16,4],[1665,17,2],[1665,19,2],[1665,39,2],[1665,41,1],
[1665,45,1],[1665,49,1],[1665,54,1],[1665,55,1],[1665,57,1],
[1665,58,1],[1665,59,2],[1665,64,1],[1665,65,3],[1665,67,1],
[1665,77,1],[1666,3,1],[1666,5,1],[1666,32,1],[1666,43,1],[1666,76,1],
[1667,18,1],[1667,23,2],[1667,24,1],[1667,27,2],[1667,30,1],
[1668,9,1],[1668,25,3],[1669,22,2],[1670,27,1],[1670,31,1],

[1670,47,2],[1671,31,1],[1671,63,1],[1672,25,3],[1673,2,1],
[1673,42,3],[1673,47,1],[1674,23,2],[1674,35,1],[1674,56,1],
[1675,15,1],[1675,17,1],[1676,18,3],[1676,19,1],[1676,59,2],
[1677,2,1],[1677,28,1],[1677,31,1],[1678,1,13],[1678,4,1],[1678,5,2],
[1678,9,1],[1678,10,1],[1678,13,3],[1678,15,1],[1678,20,3],
[1678,21,2],[1678,23,1],[1678,24,1],[1678,29,1],[1678,30,2],
[1678,32,1],[1678,34,1],[1678,35,1],[1678,36,1],[1678,37,1],
[1678,38,1],[1678,39,5],[1678,41,1],[1678,43,2],[1678,47,1],
[1678,51,1],[1678,56,1],[1678,74,1],[1679,2,4],[1680,18,1],
[1680,45,1],[1681,22,1],[1681,40,1],[1681,56,1],[1682,15,3],
[1683,8,1],[1683,22,1],[1683,28,2],[1684,17,1],[1684,53,1],[1685,9,1],
[1685,45,1],[1686,12,2],[1687,27,5],[1687,35,2],[1687,42,1],
[1687,44,1],[1688,1,1],[1688,15,5],[1688,16,1],[1688,17,1],
[1688,19,1],[1688,20,1],[1688,30,1],[1688,41,3],[1688,49,1],
[1688,53,1],[1688,55,1],[1688,57,1],[1688,58,1],[1689,15,1],
[1689,55,1],[1690,15,3],[1690,50,1],[1690,53,1],[1690,70,1],
[1691,1,1],[1691,16,1],[1691,20,1],[1691,35,1],[1691,46,1],
[1692,35,2],[1692,62,1],[1692,68,1],[1693,28,20],[1693,63,1],
[1693,68,1],[1693,70,2],[1694,10,1],[1694,28,1],[1694,31,17],
[1694,33,47],[1694,59,14],[1695,2,1],[1695,3,1],[1696,33,4],
[1697,10,1],[1697,23,1],[1697,24,13],[1697,27,3],[1697,35,4],
[1697,59,1],[1698,22,31],[1698,24,7],[1698,27,3],[1698,35,1],
[1699,2,1],[1699,15,1],[1699,43,1],[1700,24,1],[1700,28,1],
[1701,1,19],[1701,2,62],[1701,7,1],[1701,8,6],[1701,9,1],[1701,12,10],
[1701,13,109],[1701,15,3],[1701,17,5],[1701,20,1],[1701,23,1],
[1701,27,1],[1701,28,1],[1701,29,6],[1701,33,5],[1701,34,2],
[1701,35,1],[1701,36,2],[1701,39,6],[1701,51,1],[1701,53,5],
[1701,55,1],[1701,59,1],[1701,68,1],[1701,71,1],[1701,72,1],
[1701,73,2],[1702,26,2],[1703,1,7],[1703,7,1],[1703,8,4],[1703,9,22],
[1703,10,29],[1703,11,10],[1703,12,15],[1703,13,7],[1703,14,10],
[1703,15,9],[1703,16,17],[1703,17,3],[1703,18,7],[1703,19,30],
[1703,20,10],[1703,29,1],[1703,30,2],[1703,34,8],[1703,36,6],
[1703,37,7],[1703,39,3],[1703,41,7],[1703,45,9],[1703,46,12],
[1703,49,8],[1703,53,5],[1703,54,7],[1703,55,6],[1703,57,12],
[1703,58,9],[1703,59,6],[1703,60,2],[1703,63,1],[1703,64,1],
[1703,65,23],[1703,67,9],[1703,71,5],[1703,73,5],[1703,77,14],
[1703,78,2],[1704,20,1],[1704,22,1],[1705,25,3],[1705,27,1],
[1705,28,1],[1706,28,2],[1707,23,1],[1707,42,1],[1708,19,1],
[1708,31,1],[1708,32,1],[1708,35,1],[1708,36,1],[1709,14,1],
[1709,15,4],[1709,17,2],[1709,53,2],[1709,55,2],[1709,57,1],
[1709,77,1],[1710,3,3],[1711,2,1],[1711,3,9],[1711,5,2],[1711,20,1],
[1711,22,1],[1711,23,35],[1711,24,16],[1711,26,9],[1711,28,3],
[1711,31,6],[1711,32,4],[1711,36,1],[1711,39,1],[1711,43,8],
[1711,44,1],[1711,47,3],[1711,50,1],[1711,68,1],[1711,70,2],
[1712,2,2],[1712,28,1],[1713,9,1],[1713,30,1],[1714,15,1],[1714,55,1],
[1715,4,1],[1715,5,1],[1715,23,1],[1715,38,4],[1715,44,9],[1715,48,1],
[1715,55,1],[1715,66,6],[1715,68,1],[1716,22,1],[1716,27,1],
[1716,35,1],[1717,23,1],[1717,28,2],[1718,3,1],[1718,35,1],[1719,0,1],
[1719,10,1],[1719,12,19],[1719,31,1],[1720,23,1],[1720,25,1],
[1720,30,1],[1721,56,1],[1721,62,1],[1722,25,1],[1722,48,1],

[1722,56,1],[1723,22,1],[1723,76,1],[1724,0,2],[1724,7,4],[1724,19,1],
[1724,59,1],[1725,24,1],[1725,46,1],[1726,21,1],[1726,26,6],
[1726,42,1],[1726,63,1],[1727,10,1],[1727,12,1],[1727,16,1],
[1728,3,7],[1728,4,1],[1728,8,2],[1728,10,1],[1728,12,1],[1728,15,1],
[1728,20,17],[1728,22,1],[1728,23,20],[1728,24,32],[1728,25,2],
[1728,26,7],[1728,27,79],[1728,28,2],[1728,29,1],[1728,31,1],
[1728,32,3],[1728,34,3],[1728,35,2],[1728,39,3],[1728,42,4],
[1728,43,1],[1728,47,2],[1728,68,2],[1728,70,1],[1728,76,1],
[1729,8,1],[1729,15,1],[1729,25,1],[1729,30,24],[1729,53,1],
[1729,55,1],[1730,62,7],[1731,32,4],[1731,33,3],[1732,5,1],
[1732,28,1],[1732,42,3],[1732,44,1],[1732,70,1],[1733,2,6],[1733,3,3],
[1733,20,1],[1733,27,1],[1733,31,5],[1733,42,3],[1733,70,1],
[1734,5,1],[1734,23,2],[1734,27,3],[1734,56,1],[1734,70,1],[1735,0,1],
[1735,1,6],[1735,9,3],[1735,10,3],[1735,11,1],[1735,12,3],[1735,13,3],
[1735,15,1],[1735,16,2],[1735,17,2],[1735,19,5],[1735,29,1],
[1735,31,1],[1735,36,4],[1735,37,2],[1735,39,3],[1735,41,2],
[1735,45,5],[1735,46,2],[1735,55,1],[1735,57,4],[1735,59,3],
[1735,60,1],[1735,64,1],[1735,65,5],[1735,67,3],[1735,71,1],
[1735,73,1],[1735,77,3],[1736,9,1],[1736,30,1],[1736,36,1],
[1736,37,3],[1736,39,1],[1736,71,1],[1737,20,3],[1738,11,1],
[1738,15,3],[1738,17,3],[1738,30,1],[1738,37,1],[1738,53,3],
[1738,55,2],[1738,58,1],[1738,59,2],[1739,3,1],[1739,33,1],
[1740,13,1],[1740,47,1],[1741,5,1],[1741,24,1],[1741,59,1],
[1741,77,1],[1742,26,2],[1742,28,1],[1742,43,6],[1742,66,1],
[1743,23,1],[1743,50,1],[1743,76,1],[1744,5,1],[1744,10,1],
[1744,13,1],[1744,20,1],[1744,30,1],[1744,36,1],[1744,38,1],
[1744,40,1],[1744,44,1],[1744,56,21],[1745,24,1],[1745,62,1],
[1746,1,2],[1746,13,1],[1746,14,2],[1746,46,1],[1746,57,1],
[1746,65,1],[1746,67,1],[1746,76,1],[1746,77,1],[1747,5,1],
[1747,40,1],[1748,0,15],[1748,1,35],[1748,2,7],[1748,3,2],[1748,4,3],
[1748,5,3],[1748,6,2],[1748,7,14],[1748,8,11],[1748,9,45],
[1748,10,48],[1748,11,25],[1748,12,40],[1748,13,27],[1748,14,15],
[1748,15,33],[1748,16,27],[1748,17,19],[1748,18,18],[1748,19,40],
[1748,20,43],[1748,21,2],[1748,22,4],[1748,23,7],[1748,24,7],
[1748,25,4],[1748,26,4],[1748,27,10],[1748,28,53],[1748,29,11],
[1748,30,18],[1748,31,3],[1748,32,4],[1748,33,6],[1748,34,16],
[1748,35,3],[1748,36,18],[1748,37,12],[1748,38,1],[1748,39,28],
[1748,40,4],[1748,41,27],[1748,42,8],[1748,43,2],[1748,44,8],
[1748,45,27],[1748,46,26],[1748,47,4],[1748,48,1],[1748,49,11],
[1748,50,6],[1748,51,1],[1748,52,1],[1748,53,15],[1748,54,6],
[1748,55,25],[1748,56,3],[1748,57,16],[1748,58,22],[1748,59,30],
[1748,60,7],[1748,61,1],[1748,62,8],[1748,63,42],[1748,64,7],
[1748,65,43],[1748,66,5],[1748,67,13],[1748,68,1],[1748,70,6],
[1748,71,14],[1748,72,4],[1748,73,21],[1748,74,132],[1748,75,53],
[1748,76,42],[1748,77,27],[1748,78,6],[1748,79,4],[1749,0,1],
[1749,1,2],[1749,7,1],[1749,9,1],[1749,10,3],[1749,11,1],[1749,12,5],
[1749,16,1],[1749,17,3],[1749,18,1],[1749,19,4],[1749,20,2],
[1749,28,1],[1749,29,2],[1749,30,1],[1749,39,2],[1749,53,3],
[1749,55,1],[1749,59,1],[1749,73,1],[1749,77,1],[1750,28,1],
[1750,32,1],[1751,23,1],[1751,24,1],[1751,25,1],[1752,2,1],

[1752,11,1],[1752,12,1],[1752,13,2],[1752,14,1],[1752,20,2],
[1752,22,1],[1752,23,3],[1752,25,1],[1752,27,1],[1752,31,1],
[1752,32,1],[1752,43,2],[1752,62,1],[1752,77,1],[1753,1,1],[1753,2,8],
[1753,3,8],[1753,4,3],[1753,5,30],[1753,6,3],[1753,8,2],[1753,10,1],
[1753,12,1],[1753,13,7],[1753,15,2],[1753,16,1],[1753,18,1],
[1753,20,8],[1753,21,4],[1753,22,9],[1753,23,7],[1753,24,15],
[1753,25,1],[1753,26,3],[1753,27,12],[1753,28,6],[1753,29,1],
[1753,30,6],[1753,31,1],[1753,32,2],[1753,33,10],[1753,34,7],
[1753,35,10],[1753,37,3],[1753,38,5],[1753,40,1],[1753,42,5],
[1753,43,1],[1753,46,2],[1753,47,5],[1753,48,1],[1753,50,2],
[1753,51,3],[1753,53,1],[1753,54,1],[1753,56,5],[1753,58,1],
[1753,59,2],[1753,60,3],[1753,61,1],[1753,68,1],[1753,69,1],
[1753,76,2],[1753,77,1],[1754,32,1],[1754,52,1],[1755,27,1],
[1755,40,1],[1755,68,53],[1755,76,1],[1756,22,1],[1756,25,5],
[1756,28,1],[1756,30,3],[1756,33,26],[1756,35,18],[1756,38,2],
[1756,52,3],[1757,2,1],[1757,20,1],[1757,21,1],[1757,26,2],
[1757,27,1],[1757,32,1],[1757,39,2],[1757,47,3],[1757,48,8],
[1758,41,1],[1758,49,1],[1759,28,1],[1759,43,1],[1760,0,1],[1760,7,1],
[1760,11,1],[1760,13,1],[1760,14,1],[1760,19,1],[1760,34,2],
[1760,37,1],[1760,49,1],[1760,65,1],[1760,71,2],[1761,15,1],
[1761,17,2],[1762,22,1],[1762,27,3],[1763,20,2],[1764,13,1],
[1764,23,1],[1764,25,12],[1764,56,5],[1764,59,1],[1764,63,1],
[1764,78,1],[1765,28,7],[1766,28,3],[1767,0,1],[1767,1,5],[1767,2,2],
[1767,3,4],[1767,4,103],[1767,5,11],[1767,8,2],[1767,9,4],[1767,10,7],
[1767,11,1],[1767,12,4],[1767,13,7],[1767,14,2],[1767,15,24],
[1767,16,4],[1767,17,114],[1767,18,1],[1767,19,6],[1767,20,8],
[1767,21,2],[1767,22,8],[1767,23,4],[1767,24,2],[1767,25,5],
[1767,26,1],[1767,27,1],[1767,28,9],[1767,29,1],[1767,30,1],
[1767,31,3],[1767,32,7],[1767,33,4],[1767,34,1],[1767,35,3],
[1767,36,1],[1767,37,2],[1767,38,36],[1767,39,1],[1767,40,1],
[1767,41,1],[1767,42,2],[1767,43,2],[1767,44,63],[1767,45,3],
[1767,46,3],[1767,49,1],[1767,50,114],[1767,53,156],[1767,54,1],
[1767,55,23],[1767,56,6],[1767,57,1],[1767,58,1],[1767,59,5],
[1767,60,1],[1767,62,2],[1767,63,4],[1767,64,1],[1767,65,5],
[1767,66,73],[1767,67,1],[1767,68,2],[1767,70,3],[1767,72,2],
[1767,74,7],[1767,75,2],[1767,76,8],[1768,15,1],[1768,55,2],
[1769,5,1],[1769,24,3],[1769,32,1],[1769,43,1],[1770,22,1],
[1770,24,2],[1770,27,3],[1770,28,18],[1770,35,5],[1771,2,1],
[1771,4,1],[1771,27,1],[1771,38,3],[1771,44,6],[1771,66,6],
[1772,28,2],[1772,33,1],[1772,61,1],[1773,26,1],[1773,27,1],
[1773,30,1],[1774,35,2],[1775,43,2],[1776,17,6],[1776,53,1],
[1776,55,1],[1777,14,1],[1777,15,1],[1778,2,2],[1778,56,1],
[1778,69,1],[1779,5,1],[1779,35,1],[1780,40,1],[1780,63,8],
[1780,74,6],[1780,75,4],[1780,76,5],[1781,10,1],[1781,11,1],
[1781,14,3],[1781,16,1],[1781,45,2],[1781,60,1],[1781,67,1],
[1782,1,1],[1782,2,1],[1782,3,8],[1782,5,2],[1782,6,1],[1782,10,2],
[1782,12,2],[1782,13,1],[1782,15,1],[1782,19,2],[1782,20,1],
[1782,22,4],[1782,24,2],[1782,25,1],[1782,26,3],[1782,29,1],
[1782,32,2],[1782,36,2],[1782,37,1],[1782,38,140],[1782,41,1],
[1782,45,1],[1782,47,1],[1782,50,1],[1782,53,1],[1782,55,1],

[1782,57,1],[1782,59,1],[1782,62,5],[1782,70,2],[1782,74,1],
[1782,76,1],[1782,78,1],[1783,3,2],[1783,47,2],[1784,15,2],
[1784,17,1],[1784,53,3],[1784,71,1],[1785,28,6],[1785,56,1],
[1786,27,1],[1786,28,1],[1787,9,1],[1787,54,1],[1787,59,1],[1788,3,3],
[1788,28,1],[1789,12,1],[1789,23,1],[1789,27,2],[1789,47,1],
[1790,35,1],[1790,37,1],[1791,6,1],[1791,20,1],[1791,32,1],
[1791,35,2],[1791,42,2],[1791,44,1],[1791,56,6],[1792,19,1],
[1792,41,1],[1793,9,1],[1793,31,1],[1794,1,2],[1794,2,2],[1794,10,1],
[1794,13,1],[1794,20,1],[1794,23,5],[1794,24,1],[1794,31,1],
[1794,33,3],[1794,42,9],[1794,43,5],[1794,47,1],[1795,32,13],
[1795,66,1],[1795,68,1],[1796,25,6],[1796,28,2],[1796,30,13],
[1796,33,21],[1796,35,12],[1796,52,8],[1796,56,1],[1796,62,1],
[1796,63,1],[1797,28,1],[1797,43,2],[1798,6,1],[1798,24,1],
[1798,27,1],[1798,35,4],[1798,66,1],[1799,4,3],[1799,18,1],
[1799,32,1],[1799,38,4],[1799,50,2],[1799,51,2],[1799,65,1],
[1799,66,5],[1800,17,1],[1800,29,1],[1801,33,1],[1801,62,1],
[1802,6,1],[1802,22,1],[1802,27,2],[1802,35,4],[1802,38,1],
[1802,61,1],[1802,75,1],[1803,0,41],[1803,1,2],[1803,2,2],[1803,3,11],
[1803,4,4],[1803,5,10],[1803,6,6],[1803,8,1],[1803,10,2],[1803,12,2],
[1803,15,3],[1803,19,2],[1803,20,2],[1803,21,4],[1803,22,10],
[1803,23,10],[1803,24,13],[1803,25,4],[1803,26,20],[1803,27,907],
[1803,28,63],[1803,31,1],[1803,32,5],[1803,33,7],[1803,35,335],
[1803,37,7],[1803,38,18],[1803,39,2],[1803,40,5],[1803,42,65],
[1803,43,3],[1803,44,8],[1803,46,3],[1803,47,34],[1803,48,148],
[1803,50,1],[1803,51,1],[1803,56,4],[1803,61,4],[1803,62,4],
[1803,63,7],[1803,66,9],[1803,67,1],[1803,68,1],[1803,69,1],
[1803,70,11],[1803,72,1],[1803,74,10],[1803,75,4],[1803,76,5],
[1804,22,1],[1804,26,2],[1804,31,1],[1805,4,1],[1805,31,1],
[1805,66,1],[1805,70,1],[1806,26,1],[1806,35,1],[1806,42,7],
[1807,21,2],[1807,24,1],[1807,33,1],[1807,42,1],[1807,44,1],
[1807,47,1],[1807,74,2],[1808,16,2],[1809,23,1],[1809,32,1],
[1809,63,1],[1809,72,1],[1810,0,1],[1810,1,3],[1810,2,2],[1810,4,5],
[1810,5,4],[1810,6,1],[1810,9,1],[1810,10,2],[1810,12,1],[1810,13,4],
[1810,15,29],[1810,20,10],[1810,22,1],[1810,23,2],[1810,24,1],
[1810,26,1],[1810,27,2],[1810,29,1],[1810,30,1],[1810,31,3],
[1810,32,2],[1810,33,1],[1810,35,2],[1810,37,1],[1810,42,5],
[1810,43,2],[1810,44,3],[1810,45,4],[1810,46,2],[1810,47,1],
[1810,50,7],[1810,53,2],[1810,54,1],[1810,55,19],[1810,56,1],
[1810,58,1],[1810,59,4],[1810,60,1],[1810,61,1],[1810,62,2],
[1810,65,1],[1810,66,2],[1810,67,1],[1810,69,3],[1810,70,1],
[1810,71,1],[1810,72,4],[1810,73,1],[1810,75,2],[1810,76,1],
[1810,77,1],[1811,28,3],[1811,63,2],[1811,74,2],[1811,76,4],
[1812,28,7],[1812,31,1],[1812,33,1],[1812,35,1],[1812,52,1],
[1813,17,2],[1814,40,1],[1814,68,1],[1815,1,1],[1815,2,1],[1815,3,2],
[1815,20,1],[1815,30,1],[1815,37,1],[1815,46,1],[1815,47,3],
[1815,60,4],[1816,21,1],[1816,31,1],[1816,42,1],[1817,1,1],
[1817,73,1],[1818,25,1],[1818,31,1],[1818,38,1],[1818,44,14],
[1818,50,1],[1818,66,9],[1818,74,1],[1819,0,2],[1819,28,2],[1820,2,1],
[1820,8,1],[1820,21,1],[1820,23,3],[1820,68,1],[1821,17,1],
[1821,55,1],[1822,2,1],[1822,3,1],[1822,23,1],[1822,25,1],[1822,28,2],

[1822,35,1],[1822,43,1],[1823,24,27],[1823,26,1],[1823,30,1],
[1823,31,7],[1823,32,22],[1823,33,1],[1823,63,1],[1824,3,1],
[1824,4,1],[1824,20,1],[1824,24,1],[1824,25,1],[1824,26,1],
[1824,27,10],[1824,28,3],[1824,32,3],[1824,35,3],[1824,42,1],
[1825,19,1],[1825,21,1],[1825,28,5],[1825,33,1],[1825,35,1],
[1825,40,1],[1825,63,1],[1825,71,1],[1825,72,1],[1825,75,1],
[1826,9,2],[1826,67,1],[1827,34,3],[1827,73,1],[1828,23,1],
[1828,74,1],[1829,1,1],[1829,8,1],[1829,9,10],[1829,11,1],[1829,54,1],
[1829,59,1],[1830,28,3],[1830,32,1],[1830,37,1],[1831,1,4],[1831,2,2],
[1831,3,1],[1831,7,1],[1831,8,1],[1831,10,2],[1831,11,1],[1831,13,13],
[1831,19,2],[1831,21,1],[1831,22,1],[1831,24,1],[1831,25,1],
[1831,26,2],[1831,27,2],[1831,29,1],[1831,30,1],[1831,33,3],
[1831,34,2],[1831,35,4],[1831,37,1],[1831,39,3],[1831,40,1],
[1831,42,2],[1831,44,1],[1831,46,1],[1831,47,5],[1831,51,1],
[1831,56,3],[1831,66,2],[1831,68,1],[1831,73,1],[1831,74,1],
[1831,76,1],[1832,14,1],[1832,19,1],[1832,25,1],[1832,27,1],
[1832,47,1],[1832,54,1],[1833,0,10],[1833,1,1],[1833,8,13],[1833,9,2],
[1833,10,7],[1833,13,1],[1833,17,1],[1833,18,2],[1833,19,1],
[1833,20,1],[1833,23,11],[1833,27,3],[1833,28,1],[1833,35,1],
[1833,36,2],[1833,45,3],[1833,46,1],[1833,49,2],[1833,55,2],
[1833,57,4],[1833,58,1],[1833,59,2],[1833,67,1],[1833,71,2],
[1833,73,1],[1833,77,6],[1834,4,1],[1834,12,3],[1834,27,1],
[1834,40,1],[1834,68,2],[1834,69,1],[1834,70,4],[1834,72,1],
[1834,74,1],[1834,76,1],[1835,3,1],[1835,6,1],[1835,33,2],[1835,35,1],
[1835,38,1],[1835,40,1],[1835,52,1],[1835,56,5],[1835,63,1],
[1835,68,3],[1835,70,2],[1835,72,2],[1835,74,3],[1836,27,1],
[1836,59,1],[1837,55,1],[1837,78,1],[1838,22,1],[1838,23,1],
[1838,70,1],[1839,9,1],[1839,28,1],[1839,53,1],[1839,76,1],
[1840,26,1],[1840,68,1],[1841,0,1],[1841,19,2],[1841,31,6],
[1841,34,2],[1841,71,1],[1842,0,1],[1842,21,20],[1842,22,10],
[1842,23,19],[1842,24,4],[1842,25,8],[1842,26,3],[1842,27,1],
[1842,28,32],[1842,33,124],[1842,35,1],[1842,42,191],[1842,43,14],
[1842,45,4],[1842,51,6],[1842,54,5],[1842,58,1],[1842,62,1],
[1842,74,17],[1842,75,1],[1843,2,4],[1843,21,3],[1844,25,1],
[1844,28,2],[1844,39,2],[1844,43,1],[1845,2,4],[1845,47,1],[1846,0,1],
[1846,60,1],[1847,2,8],[1847,74,1],[1848,4,1],[1848,11,1],[1848,20,2],
[1849,1,3],[1849,27,1],[1849,31,1],[1850,23,11],[1850,26,1],
[1850,31,9],[1850,35,1],[1850,42,3],[1850,56,1],[1850,62,1],
[1851,28,1],[1851,31,1],[1852,24,2],[1852,73,1],[1853,1,1],[1853,2,2],
[1853,3,3],[1853,4,2],[1853,5,1],[1853,9,3],[1853,10,2],[1853,15,2],
[1853,16,8],[1853,19,3],[1853,20,2],[1853,21,3],[1853,22,1],
[1853,23,3],[1853,24,3],[1853,25,4],[1853,26,1],[1853,27,3],
[1853,28,1],[1853,29,1],[1853,30,72],[1853,31,3],[1853,32,8],
[1853,33,33],[1853,35,4],[1853,38,2],[1853,40,1],[1853,42,1],
[1853,43,1],[1853,44,3],[1853,45,1],[1853,46,1],[1853,47,1],
[1853,50,1],[1853,51,1],[1853,52,7],[1853,56,3],[1853,57,1],
[1853,58,2],[1853,62,2],[1853,63,2],[1853,64,1],[1853,66,4],
[1853,67,2],[1853,68,4],[1853,70,3],[1853,72,1],[1853,74,4],
[1853,76,1],[1854,25,1],[1854,27,1],[1854,28,1],[1854,35,1],
[1855,22,11],[1855,27,3],[1855,28,1],[1855,35,10],[1856,2,1],

[1856,27,1],[1856,30,1],[1856,32,1],[1856,43,1],[1856,50,1],
[1856,56,1],[1857,28,24],[1858,2,2],[1858,3,1],[1858,22,1],
[1858,23,1],[1858,28,2],[1858,42,1],[1859,13,1],[1859,57,1],
[1860,28,2],[1860,35,2],[1861,28,1],[1861,69,1],[1862,4,40],
[1862,5,2],[1862,22,1],[1862,24,1],[1862,25,1],[1862,28,3],
[1862,31,1],[1862,32,1],[1862,38,1],[1862,44,20],[1862,50,54],
[1862,51,7],[1862,66,57],[1862,68,2],[1862,70,1],[1862,76,1],
[1863,6,1],[1863,32,1],[1864,2,2],[1864,3,1],[1864,28,4],[1864,32,1],
[1864,70,1],[1865,10,1],[1865,24,1],[1865,48,1],[1865,60,1],
[1865,62,1],[1866,23,1],[1866,28,17],[1866,32,1],[1866,33,1],
[1866,35,2],[1866,74,1],[1867,2,1],[1867,23,3],[1867,24,1],
[1867,25,1],[1867,28,7],[1867,31,1],[1867,32,1],[1867,33,1],
[1867,38,1],[1867,44,1],[1867,70,1],[1868,2,2],[1868,17,1],
[1868,20,1],[1868,22,1],[1868,25,1],[1868,26,1],[1868,27,1],
[1868,37,2],[1868,43,2],[1868,62,1],[1868,75,1],[1869,6,1],
[1869,22,1],[1869,24,1],[1869,32,1],[1870,28,1],[1870,66,1],
[1871,0,6],[1871,9,61],[1871,10,1],[1871,12,1],[1871,37,1],
[1871,39,1],[1871,77,1],[1872,28,2],[1873,6,1],[1873,32,1],
[1873,47,4],[1873,63,1],[1874,2,1],[1874,30,2],[1874,47,2],
[1874,53,1],[1875,27,1],[1875,28,1],[1876,17,1],[1876,53,1],
[1877,2,2],[1877,3,1],[1877,26,1],[1877,27,3],[1877,42,1],[1877,47,4],
[1877,59,2],[1877,60,2],[1877,70,1],[1878,21,1],[1878,31,1],
[1879,27,1],[1879,28,1],[1880,4,1],[1880,12,1],[1880,18,1],
[1880,38,1],[1880,41,1],[1880,65,1],[1881,23,10],[1882,31,3],
[1883,3,1],[1883,28,1],[1883,48,2],[1884,1,1],[1884,5,1],[1884,28,2],
[1885,50,1],[1885,66,1],[1886,15,1],[1886,17,1],[1886,53,3],
[1887,3,1],[1887,22,1],[1887,24,1],[1888,21,1],[1888,31,2],
[1888,33,3],[1889,13,1],[1889,57,1],[1889,71,1],[1890,2,2],[1891,1,1],
[1891,2,1],[1891,5,3],[1891,6,1],[1891,8,3],[1891,9,2],[1891,21,1],
[1891,23,1],[1891,24,1],[1891,25,3],[1891,27,3],[1891,28,2],
[1891,31,1],[1891,33,4],[1891,34,3],[1891,35,1],[1891,42,1],
[1891,43,1],[1891,59,1],[1891,70,1],[1892,1,1],[1892,26,1],
[1893,22,2],[1893,23,1],[1894,22,1],[1894,24,3],[1895,44,2],
[1895,66,3],[1896,19,1],[1896,22,1],[1896,26,1],[1896,35,1],
[1896,53,1],[1897,43,2],[1898,3,1],[1898,13,1],[1898,30,1],[1899,1,1],
[1899,53,1],[1900,2,3],[1900,3,2],[1900,4,1],[1900,28,1],[1900,72,1],
[1900,73,1],[1901,21,3],[1901,31,5],[1901,33,4],[1901,42,1],
[1901,43,2],[1902,28,33],[1902,75,1],[1903,2,22],[1903,3,1],
[1903,4,1],[1903,6,1],[1903,12,2],[1903,13,1],[1903,19,1],[1903,24,1],
[1903,27,1],[1903,29,7],[1903,31,18],[1903,46,1],[1903,47,1],
[1903,53,1],[1903,62,1],[1903,65,1],[1903,70,1],[1903,73,1],
[1903,74,1],[1903,76,1],[1903,77,3],[1904,51,4],[1905,6,2],
[1905,22,3],[1905,23,1],[1905,24,2],[1905,27,3],[1905,35,3],
[1905,43,1],[1905,56,1],[1905,70,1],[1906,22,1],[1906,23,1],
[1906,27,1],[1906,60,1],[1906,65,1],[1906,70,1],[1907,1,1],
[1907,10,1],[1908,2,1],[1908,27,1],[1908,31,2],[1909,2,3],[1909,23,1],
[1909,26,1],[1909,30,2],[1909,31,6],[1909,39,1],[1909,42,1],
[1909,43,1],[1909,53,1],[1909,59,2],[1909,60,1],[1909,64,1],
[1909,73,1],[1910,23,1],[1910,25,1],[1910,28,2],[1911,1,6],[1911,2,4],
[1911,3,6],[1911,5,2],[1911,6,1],[1911,9,1],[1911,10,2],[1911,12,1],

[1911,13,1],[1911,14,1],[1911,17,1],[1911,20,12],[1911,21,4],
[1911,22,3],[1911,23,21],[1911,24,34],[1911,26,1],[1911,27,20],
[1911,28,14],[1911,30,2],[1911,31,22],[1911,32,5],[1911,33,3],
[1911,34,3],[1911,35,28],[1911,36,1],[1911,39,2],[1911,41,1],
[1911,42,15],[1911,43,11],[1911,44,1],[1911,45,1],[1911,47,2],
[1911,48,1],[1911,49,1],[1911,58,1],[1911,63,1],[1911,64,1],
[1911,66,1],[1911,70,1],[1911,76,1],[1911,77,3],[1912,28,3],
[1912,70,1],[1912,75,1],[1913,20,1],[1913,21,1],[1913,23,2],
[1913,24,1],[1913,62,1],[1914,2,3],[1914,23,1],[1914,28,1],
[1914,32,1],[1914,33,1],[1914,52,1],[1915,3,2],[1916,36,1],
[1916,37,5],[1917,5,1],[1917,44,1],[1917,66,1],[1918,0,1],[1918,41,1],
[1918,71,1],[1919,9,1],[1919,10,3],[1919,14,2],[1919,15,44],
[1919,16,1],[1919,17,2],[1919,19,3],[1919,20,1],[1919,26,1],
[1919,27,2],[1919,32,1],[1919,33,1],[1919,35,2],[1919,36,2],
[1919,37,2],[1919,38,1],[1919,40,1],[1919,41,3],[1919,43,1],
[1919,45,1],[1919,49,2],[1919,50,3],[1919,53,4],[1919,55,24],
[1919,58,2],[1919,59,5],[1919,61,1],[1919,63,2],[1919,65,3],
[1919,69,1],[1919,70,2],[1919,71,1],[1919,72,2],[1919,73,2],
[1919,74,1],[1919,76,1],[1919,77,2],[1920,2,4],[1920,12,1],
[1920,17,1],[1920,20,12],[1920,23,1],[1920,28,1],[1921,28,2],
[1922,0,2],[1922,10,1],[1922,12,4],[1922,45,3],[1922,57,1],[1923,2,8],
[1924,24,1],[1924,28,1],[1925,3,8],[1925,5,3],[1925,6,3],[1925,22,1],
[1925,23,14],[1925,24,8],[1925,25,1],[1925,26,3],[1925,27,11],
[1925,28,3],[1925,31,3],[1925,32,9],[1925,33,1],[1925,35,7],
[1925,38,2],[1925,48,4],[1925,56,6],[1925,65,1],[1925,77,1],
[1926,3,1],[1926,28,1],[1926,47,1],[1926,51,1],[1926,56,2],
[1926,68,1],[1926,76,2],[1927,20,3],[1927,22,1],[1927,24,1],
[1927,28,1],[1927,32,1],[1927,33,2],[1927,38,2],[1927,59,1],
[1928,2,1],[1928,21,1],[1928,22,1],[1928,23,1],[1928,32,1],[1929,5,1],
[1929,22,1],[1929,26,1],[1929,28,1],[1929,70,1],[1929,72,1],
[1930,15,2],[1930,27,1],[1930,65,1],[1931,28,1],[1931,35,1],
[1932,3,1],[1932,24,1],[1932,28,1],[1933,22,3],[1934,0,1],[1934,1,2],
[1934,2,1],[1934,5,6],[1934,9,1],[1934,10,3],[1934,11,1],[1934,12,13],
[1934,13,2],[1934,15,3],[1934,16,2],[1934,18,1],[1934,19,2],
[1934,20,2],[1934,21,13],[1934,22,1],[1934,23,3],[1934,24,4],
[1934,25,13],[1934,27,1],[1934,28,5],[1934,29,62],[1934,30,1],
[1934,31,87],[1934,32,1],[1934,33,27],[1934,34,2],[1934,35,5],
[1934,38,1],[1934,39,4],[1934,40,3],[1934,42,2],[1934,43,4],
[1934,44,1],[1934,47,1],[1934,49,1],[1934,50,2],[1934,54,1],
[1934,57,1],[1934,58,2],[1934,59,1],[1934,60,1],[1934,65,1],
[1934,68,3],[1934,69,2],[1934,70,10],[1934,74,2],[1934,75,1],
[1934,76,2],[1934,77,1],[1935,2,1],[1935,8,1],[1935,23,1],[1935,60,1],
[1936,43,1],[1936,68,1],[1937,0,3],[1937,1,3],[1937,15,1],[1937,17,8],
[1937,25,1],[1937,28,3],[1937,30,1],[1937,31,1],[1937,35,2],
[1937,39,1],[1937,48,1],[1937,50,4],[1937,53,15],[1937,59,1],
[1937,65,1],[1937,67,1],[1937,73,1],[1938,1,91],[1938,2,3],[1938,4,1],
[1938,8,1],[1938,12,3],[1938,13,29],[1938,15,1],[1938,17,6],
[1938,18,1],[1938,29,7],[1938,31,2],[1938,34,4],[1938,36,1],
[1938,39,6],[1938,51,5],[1938,53,2],[1938,55,4],[1938,59,1],
[1938,73,1],[1938,77,1],[1939,24,2],[1939,56,3],[1939,62,1],

[1940,16,1],[1940,17,1],[1940,49,1],[1940,71,1],[1941,3,1],
[1941,27,1],[1941,31,2],[1941,32,1],[1941,35,1],[1941,42,1],
[1941,51,1],[1941,62,2],[1941,70,4],[1942,25,1],[1942,32,3],
[1942,48,2],[1942,69,1],[1943,15,1],[1943,55,1],[1944,19,1],
[1944,34,1],[1945,1,2],[1945,2,7],[1945,3,18],[1945,4,1],[1945,5,10],
[1945,6,2],[1945,9,3],[1945,10,1],[1945,12,4],[1945,13,5],[1945,14,2],
[1945,15,2],[1945,16,9],[1945,19,1],[1945,20,130],[1945,21,23],
[1945,22,17],[1945,23,64],[1945,24,40],[1945,25,39],[1945,26,65],
[1945,27,26],[1945,28,29],[1945,29,1],[1945,30,5],[1945,31,91],
[1945,32,28],[1945,33,23],[1945,34,2],[1945,35,25],[1945,36,3],
[1945,38,3],[1945,39,2],[1945,40,1],[1945,41,1],[1945,42,29],
[1945,43,66],[1945,44,1],[1945,45,1],[1945,46,2],[1945,47,10],
[1945,48,21],[1945,49,1],[1945,50,1],[1945,52,3],[1945,53,1],
[1945,54,1],[1945,55,4],[1945,57,1],[1945,59,5],[1945,60,1],
[1945,61,1],[1945,62,2],[1945,63,1],[1945,65,3],[1945,66,1],
[1945,67,1],[1945,68,2],[1945,69,1],[1945,72,1],[1945,73,1],
[1945,74,2],[1945,75,4],[1945,76,4],[1945,77,1],[1945,78,2],
[1946,3,2],[1946,23,4],[1946,24,1],[1947,2,1],[1947,24,2],[1947,27,1],
[1948,22,1],[1948,24,1],[1948,27,1],[1948,28,2],[1948,35,1],
[1949,4,3],[1949,6,1],[1949,25,1],[1949,44,1],[1949,66,1],[1950,24,1],
[1950,28,1],[1951,25,1],[1951,28,1],[1951,32,4],[1951,33,1],
[1951,52,1],[1951,62,1],[1952,24,1],[1952,28,10],[1952,38,9],
[1952,45,1],[1952,75,1],[1952,77,1],[1953,10,2],[1953,23,1],
[1953,28,1],[1953,33,1],[1953,43,1],[1954,62,4],[1955,6,1],
[1955,79,1],[1956,28,2],[1957,14,1],[1957,16,1],[1957,19,1],
[1957,38,1],[1957,55,1],[1957,66,1],[1958,27,1],[1958,37,1],
[1959,41,1],[1959,45,1],[1959,53,1],[1960,19,1],[1960,20,1],
[1960,27,12],[1960,30,1],[1960,32,1],[1960,35,4],[1961,5,1],
[1961,23,1],[1961,63,11],[1961,70,1],[1961,72,1],[1961,74,2],
[1961,75,4],[1961,76,13],[1962,28,3],[1962,50,1],[1963,2,1],
[1963,5,1],[1963,22,1],[1963,23,1],[1963,26,1],[1963,28,5],
[1963,32,1],[1963,35,1],[1963,44,1],[1963,70,1],[1963,76,1],
[1964,27,1],[1964,28,1],[1965,5,1],[1965,28,1],[1966,28,1],
[1966,32,3],[1967,0,1],[1967,13,1],[1967,17,1],[1967,20,1],
[1967,53,1],[1968,2,4],[1968,4,1],[1968,7,1],[1968,17,1],[1968,28,3],
[1968,30,1],[1968,35,1],[1968,37,1],[1969,30,1],[1969,43,1],
[1969,56,1],[1970,4,1],[1970,5,1],[1970,13,1],[1970,23,1],[1970,24,1],
[1970,26,1],[1970,31,3],[1970,35,1],[1970,41,1],[1970,42,3],
[1970,43,1],[1971,1,1],[1971,10,1],[1971,17,1],[1971,23,5],
[1971,27,3],[1971,63,1],[1972,9,3],[1972,28,4],[1972,50,2],
[1972,66,1],[1973,23,1],[1973,27,4],[1973,28,2],[1973,35,2],
[1974,27,1],[1974,28,1],[1975,28,1],[1975,55,1],[1975,62,1],
[1975,76,1],[1976,17,1],[1976,53,1],[1977,9,1],[1977,12,1],
[1977,15,1],[1977,19,1],[1977,53,1],[1978,3,1],[1978,9,1],[1978,19,1],
[1978,24,2],[1978,33,1],[1979,31,1],[1979,68,1],[1979,74,1],
[1980,55,1],[1980,65,1],[1981,5,2],[1981,24,2],[1981,28,1],
[1981,32,1],[1981,43,1],[1982,22,1],[1982,25,1],[1982,32,2],
[1982,33,1],[1982,56,3],[1983,9,1],[1983,28,1],[1984,26,1],
[1984,55,1],[1984,60,2],[1984,77,1],[1985,1,101],[1985,2,53],
[1985,3,11],[1985,4,1],[1985,5,5],[1985,6,10],[1985,7,1],[1985,8,4],

[1985,9,6],[1985,10,6],[1985,11,4],[1985,12,3],[1985,13,46],
[1985,14,1],[1985,15,7],[1985,16,1],[1985,17,4],[1985,18,1],
[1985,19,5],[1985,20,134],[1985,21,7],[1985,22,15],[1985,23,15],
[1985,24,27],[1985,25,2],[1985,26,21],[1985,27,14],[1985,28,10],
[1985,29,3],[1985,30,7],[1985,31,21],[1985,32,14],[1985,33,23],
[1985,34,13],[1985,35,6],[1985,36,5],[1985,37,5],[1985,38,2],
[1985,39,8],[1985,40,1],[1985,41,1],[1985,42,40],[1985,43,33],
[1985,44,1],[1985,45,2],[1985,47,2],[1985,48,21],[1985,50,3],
[1985,52,1],[1985,54,2],[1985,55,3],[1985,56,2],[1985,57,5],
[1985,58,3],[1985,59,9],[1985,60,3],[1985,64,2],[1985,65,8],
[1985,66,3],[1985,67,4],[1985,68,2],[1985,69,1],[1985,70,2],
[1985,71,3],[1985,73,2],[1985,74,3],[1985,77,1],[1985,78,5],
[1986,28,1],[1986,39,1],[1986,42,1],[1986,74,2],[1987,3,1],
[1987,62,2],[1988,24,1],[1988,28,1],[1988,31,1],[1989,28,2],
[1990,3,1],[1990,47,1],[1990,48,2],[1991,2,1],[1991,3,1],[1991,6,3],
[1991,7,1],[1991,10,1],[1991,22,1],[1991,23,1],[1991,26,2],
[1991,27,6],[1991,28,1],[1991,30,1],[1991,31,1],[1991,32,1],
[1991,35,3],[1991,56,1],[1991,70,1],[1991,73,1],[1992,2,8],[1992,3,4],
[1992,23,1],[1992,24,1],[1992,25,2],[1992,28,1],[1992,31,1],
[1992,33,1],[1992,42,1],[1992,47,2],[1993,5,1],[1993,58,1],
[1994,24,1],[1994,27,1],[1994,31,1],[1994,39,1],[1995,37,2],
[1996,2,16],[1996,62,2],[1997,56,3],[1998,6,4],[1999,2,6],[1999,23,2],
[1999,25,4],[1999,27,3],[1999,28,2],[1999,37,1],[1999,42,1],
[1999,62,1],[2000,21,3],[2001,5,2],[2001,27,1],[2002,24,1],
[2002,43,1],[2003,13,2],[2003,31,1],[2004,10,2],[2004,15,2],
[2004,16,1],[2004,19,1],[2004,36,1],[2004,45,2],[2004,55,5],
[2004,57,2],[2004,58,1],[2004,65,1],[2004,71,2],[2004,73,2],
[2005,12,1],[2005,32,2],[2005,42,1],[2006,25,2],[2006,28,1],
[2006,33,2],[2006,35,1],[2006,52,1],[2007,12,1],[2007,21,1],
[2007,27,1],[2008,2,2],[2009,28,1],[2009,37,3],[2009,60,1],
[2010,27,1],[2010,28,2],[2011,7,3],[2012,23,1],[2012,60,1],
[2013,28,1],[2013,50,1],[2014,2,2],[2014,3,2],[2014,4,1],[2014,5,6],
[2014,22,5],[2014,23,2],[2014,24,5],[2014,26,1],[2014,27,1],
[2014,31,3],[2014,32,1],[2014,35,1],[2014,42,1],[2014,47,1],
[2014,56,5],[2014,62,3],[2015,2,1],[2015,38,1],[2016,33,2],
[2016,42,1],[2017,42,2],[2018,13,1],[2018,20,1],[2018,48,1],
[2019,2,1],[2019,6,2],[2019,20,1],[2020,22,2],[2020,23,1],[2020,24,2],
[2020,26,1],[2020,27,1],[2020,32,1],[2020,35,2],[2020,39,1],
[2020,42,1],[2020,43,1],[2020,47,1],[2020,48,1],[2020,49,1],
[2020,62,1],[2021,26,1],[2021,27,1],[2021,38,5],[2021,56,1],
[2021,68,1],[2021,69,1],[2022,25,1],[2022,27,1],[2022,76,1],
[2023,0,1],[2023,1,1],[2024,28,2],[2024,42,1],[2025,23,1],[2025,31,7],
[2026,10,1],[2026,19,1],[2027,46,1],[2027,70,1],[2028,25,3],
[2029,13,2],[2030,2,2],[2030,21,2],[2030,23,1],[2030,28,1],
[2030,31,5],[2031,6,1],[2031,23,1],[2031,24,3],[2031,26,1],
[2031,27,1],[2031,28,1],[2031,38,2],[2031,66,1],[2032,3,1],[2032,7,1],
[2032,21,4],[2032,23,3],[2032,24,1],[2032,26,2],[2032,27,10],
[2032,28,1],[2032,31,1],[2032,32,1],[2032,33,2],[2032,34,2],
[2032,35,1],[2032,40,1],[2032,42,2],[2032,43,22],[2032,52,1],
[2032,74,2],[2032,77,1],[2033,23,1],[2033,27,1],[2034,6,2],

[2034,21,1],[2034,26,2],[2035,15,1],[2035,41,1],[2036,0,1],[2036,1,4],
[2036,7,1],[2036,8,1],[2036,9,1],[2036,10,3],[2036,11,2],[2036,12,1],
[2036,15,2],[2036,17,1],[2036,19,2],[2036,20,1],[2036,29,2],
[2036,30,1],[2036,34,1],[2036,46,1],[2036,53,2],[2036,55,1],
[2036,59,1],[2036,65,5],[2036,67,1],[2037,4,1],[2037,5,1],[2037,23,1],
[2037,35,1],[2037,40,1],[2037,44,1],[2037,66,1],[2037,68,1],
[2037,69,1],[2037,75,1],[2037,76,1],[2037,79,1],[2038,28,6],
[2039,7,6],[2039,12,1],[2040,22,2],[2040,27,3],[2040,35,2],
[2041,47,2],[2041,68,2],[2042,5,1],[2042,25,1],[2042,48,1],
[2043,26,3],[2043,42,1],[2043,43,3],[2044,5,1],[2044,23,2],
[2044,24,1],[2044,25,1],[2044,28,2],[2044,35,5],[2044,42,1],
[2044,43,1],[2044,47,1],[2044,50,1],[2044,62,1],[2045,3,1],
[2045,21,1],[2045,22,3],[2045,23,1],[2045,26,1],[2045,27,1],
[2045,32,2],[2045,42,1],[2045,56,1],[2046,37,2],[2046,46,1],
[2047,28,2],[2048,13,1],[2048,20,1],[2048,22,1],[2048,28,1],
[2048,59,1],[2049,2,7],[2049,28,3],[2049,31,3],[2049,39,1],
[2049,46,1],[2049,65,1],[2050,1,1],[2050,13,1],[2051,24,1],
[2051,25,1],[2051,27,1],[2052,22,1],[2052,27,1],[2053,9,1],
[2053,28,2],[2054,38,1],[2054,66,1],[2055,22,1],[2055,62,1],
[2056,28,1],[2056,44,1],[2057,27,1],[2057,43,4],[2058,5,1],
[2058,13,1],[2058,20,2],[2058,21,3],[2058,23,4],[2058,25,1],
[2058,27,3],[2058,28,1],[2058,31,1],[2058,35,33],[2058,44,1],
[2059,1,3],[2059,4,1],[2059,9,2],[2059,10,1],[2059,13,11],[2059,15,2],
[2059,19,1],[2059,20,3],[2059,23,4],[2059,25,1],[2059,27,6],
[2059,28,5],[2059,29,1],[2059,31,2],[2059,32,7],[2059,34,1],
[2059,35,1],[2059,39,1],[2059,42,43],[2059,43,1],[2059,50,1],
[2059,53,1],[2059,62,1],[2059,73,1],[2060,21,1],[2060,24,1],
[2060,26,1],[2060,28,2],[2060,31,1],[2060,32,1],[2060,76,1],
[2061,3,1],[2061,22,3],[2061,28,1],[2061,38,1],[2061,67,1],[2062,5,1],
[2062,17,1],[2062,32,1],[2062,38,1],[2062,60,1],[2063,2,3],[2063,9,1],
[2063,12,3],[2063,20,1],[2063,27,1],[2063,62,2],[2064,10,1],
[2064,13,1],[2064,28,1],[2064,37,1],[2065,5,2],[2065,24,2],
[2065,50,2],[2066,23,1],[2066,28,1],[2067,18,1],[2067,28,1],
[2067,67,1],[2067,72,1],[2068,28,2],[2069,33,3],[2070,25,1],
[2070,35,1],[2070,56,1],[2070,74,1],[2070,79,1],[2071,28,1],
[2071,31,3],[2072,32,5],[2073,3,1],[2073,28,2],[2074,13,2],
[2074,29,1],[2074,39,1],[2075,2,1],[2075,31,2],[2075,44,1],
[2076,28,2],[2077,26,1],[2077,40,1],[2078,28,2],[2078,33,1],
[2079,23,2],[2080,2,4],[2080,3,3],[2080,5,2],[2080,20,1],[2080,21,37],
[2080,22,10],[2080,23,5],[2080,25,9],[2080,26,39],[2080,28,2],
[2080,31,42],[2080,33,11],[2080,35,2],[2080,38,1],[2080,42,7],
[2080,43,22],[2080,47,2],[2080,48,3],[2080,50,1],[2080,52,3],
[2080,72,1],[2080,76,1],[2081,2,4],[2081,17,1],[2081,23,1],
[2081,32,1],[2081,34,1],[2081,55,1],[2082,2,2],[2082,3,6],[2082,22,1],
[2082,23,1],[2082,24,3],[2082,26,1],[2082,35,1],[2082,40,1],
[2082,42,1],[2082,44,1],[2082,47,1],[2082,56,4],[2082,61,1],
[2082,68,1],[2082,70,1],[2082,72,1],[2083,43,2],[2083,48,2],
[2084,2,1],[2084,28,1],[2085,0,1],[2085,28,2],[2085,31,18],
[2085,33,1],[2085,39,1],[2085,42,1],[2085,43,4],[2086,2,2],[2086,8,2],
[2086,22,1],[2086,23,1],[2086,24,1],[2086,31,8],[2086,32,6],

[2086,33,3],[2086,42,2],[2086,43,2],[2086,56,1],[2087,25,3],
[2087,27,1],[2087,32,4],[2087,38,1],[2087,42,5],[2087,62,1],
[2087,69,1],[2088,35,2],[2088,38,1],[2089,27,2],[2089,42,2],
[2090,9,1],[2090,12,1],[2090,49,1],[2090,73,1],[2091,2,15],
[2091,10,1],[2091,21,2],[2091,25,1],[2091,30,1],[2091,31,1],
[2092,3,1],[2092,22,1],[2092,48,1],[2093,2,1],[2093,3,2],[2093,8,1],
[2093,22,2],[2093,23,1],[2093,24,4],[2093,25,3],[2093,26,1],
[2093,27,16],[2093,28,2],[2093,30,1],[2093,31,3],[2093,32,6],
[2093,33,12],[2093,34,1],[2093,35,4],[2093,42,14],[2093,47,4],
[2093,48,3],[2093,52,2],[2094,17,1],[2094,25,1],[2095,5,1],
[2095,15,1],[2095,17,3],[2095,31,1],[2095,35,1],[2095,44,3],
[2095,55,2],[2095,66,1],[2096,1,2],[2096,9,2],[2096,13,3],[2096,54,1],
[2097,44,2],[2098,53,1],[2098,58,1],[2099,28,3],[2100,35,2],
[2100,70,1],[2101,6,1],[2101,27,1],[2101,31,1],[2101,50,1],
[2101,74,1],[2102,40,4],[2102,68,2],[2102,69,2],[2102,70,6],
[2102,72,2],[2103,3,1],[2103,5,1],[2103,10,1],[2103,13,2],[2103,22,1],
[2103,23,2],[2103,25,1],[2103,26,1],[2103,28,2],[2103,47,1],
[2103,62,1],[2103,65,1],[2104,2,9],[2105,3,1],[2105,26,1],[2106,24,1],
[2106,26,1],[2106,42,1],[2106,48,3],[2107,28,2],[2107,44,1],
[2107,50,1],[2107,66,3],[2108,5,1],[2108,23,1],[2108,26,1],
[2108,43,1],[2108,44,1],[2108,63,20],[2108,68,1],[2108,74,8],
[2108,75,9],[2108,76,33],[2109,1,1],[2109,4,1],[2109,9,1],[2109,12,1],
[2109,26,1],[2109,34,1],[2109,38,12],[2109,53,1],[2109,55,1],
[2109,65,1],[2109,68,1],[2109,78,1],[2110,5,1],[2110,22,1],
[2110,24,1],[2110,25,1],[2110,28,1],[2110,50,1],[2110,69,1],
[2110,74,1],[2110,75,1],[2111,1,1],[2111,3,1],[2111,8,3],[2111,16,1],
[2111,22,1],[2111,23,5],[2111,24,1],[2111,26,1],[2111,27,1],
[2111,28,1],[2111,30,1],[2111,31,1],[2111,34,9],[2111,35,1],
[2111,37,1],[2111,38,1],[2111,41,1],[2111,42,1],[2111,43,3],
[2111,57,1],[2112,5,1],[2112,27,3],[2112,42,2],[2113,3,1],[2113,5,1],
[2113,22,1],[2113,23,5],[2113,24,6],[2113,25,1],[2113,26,2],
[2113,27,4],[2113,28,2],[2113,31,7],[2113,32,3],[2113,40,1],
[2113,42,1],[2113,47,1],[2113,62,1],[2113,68,1],[2113,70,1],
[2114,1,1],[2114,66,1],[2115,28,1],[2115,75,1],[2116,3,1],[2116,6,2],
[2116,27,1],[2117,11,1],[2117,27,1],[2117,28,1],[2117,34,1],
[2118,2,9],[2118,3,4],[2118,4,1],[2118,5,10],[2118,8,4],[2118,20,1],
[2118,21,3],[2118,22,13],[2118,23,13],[2118,24,3],[2118,25,18],
[2118,26,11],[2118,27,13],[2118,28,1],[2118,31,12],[2118,32,18],
[2118,33,1],[2118,35,9],[2118,37,1],[2118,40,3],[2118,42,4],
[2118,43,3],[2118,47,1],[2118,48,3],[2118,50,1],[2118,53,1],
[2118,59,2],[2118,60,1],[2118,61,1],[2118,62,5],[2118,66,2],
[2118,68,2],[2118,69,1],[2118,70,3],[2118,72,1],[2118,74,1],
[2118,75,1],[2118,79,1],[2119,3,1],[2119,9,1],[2119,12,3],[2119,13,6],
[2119,15,1],[2119,21,1],[2119,23,1],[2119,24,1],[2119,27,1],
[2119,28,1],[2119,30,1],[2119,31,2],[2119,36,1],[2119,42,1],
[2119,50,1],[2119,61,1],[2120,32,1],[2120,35,1],[2120,50,1],
[2120,66,1],[2121,22,1],[2121,50,3],[2121,63,1],[2122,2,2],[2122,4,1],
[2122,21,1],[2122,28,1],[2123,1,3],[2123,3,1],[2123,12,1],[2123,28,1],
[2123,59,1],[2124,17,1],[2124,55,1],[2125,26,1],[2125,66,3],
[2126,4,3],[2126,20,1],[2126,38,4],[2126,50,5],[2126,66,4],

[2127,20,2],[2128,1,4],[2128,21,2],[2128,23,1],[2128,26,3],
[2128,62,11],[2129,9,1],[2129,12,1],[2129,15,5],[2129,16,1],
[2129,28,5],[2129,55,1],[2130,2,1],[2130,7,1],[2130,35,1],[2130,56,1],
[2131,38,2],[2132,62,1],[2132,68,1],[2132,70,1],[2133,27,1],
[2133,28,1],[2134,5,3],[2134,28,1],[2134,35,1],[2135,27,1],
[2135,28,2],[2135,30,1],[2136,23,1],[2136,24,1],[2136,27,1],
[2137,2,1],[2137,28,1],[2138,25,2],[2138,35,2],[2139,15,5],
[2139,17,1],[2139,20,1],[2139,36,1],[2139,46,1],[2139,55,3],
[2139,58,1],[2140,12,1],[2140,29,5],[2141,28,1],[2141,32,3],
[2142,28,1],[2142,62,1],[2143,23,1],[2143,31,2],[2143,61,1],
[2143,66,1],[2144,24,1],[2144,56,1],[2145,44,2],[2145,47,2],
[2145,66,2],[2146,2,2],[2146,39,1],[2146,74,1],[2147,13,3],
[2147,15,1],[2147,20,1],[2147,58,1],[2148,68,1],[2148,70,1],
[2149,22,1],[2149,23,1],[2150,14,1],[2150,54,1],[2151,6,1],
[2151,28,1],[2151,56,1],[2152,4,1],[2152,23,1],[2153,23,2],
[2153,40,1],[2153,42,1],[2153,74,1],[2154,48,3],[2155,25,1],
[2155,28,5],[2155,31,85],[2155,40,2],[2155,43,1],[2155,56,4],
[2155,72,1],[2156,1,1],[2156,9,1],[2156,10,2],[2156,20,1],[2157,28,1],
[2157,74,1],[2158,35,4],[2159,28,12],[2159,66,1],[2160,6,1],
[2160,19,1],[2160,23,1],[2160,28,1],[2160,33,1],[2160,34,2],
[2160,35,1],[2161,3,1],[2161,6,1],[2161,9,1],[2161,10,1],[2161,11,1],
[2161,20,1],[2161,21,3],[2161,22,3],[2161,23,4],[2161,24,19],
[2161,25,10],[2161,26,1],[2161,27,29],[2161,28,6],[2161,30,1],
[2161,31,9],[2161,32,5],[2161,33,1],[2161,35,27],[2161,38,2],
[2161,39,3],[2161,42,5],[2161,43,7],[2161,50,1],[2161,52,1],
[2161,54,1],[2161,59,2],[2161,61,3],[2161,66,1],[2161,68,1],
[2161,73,1],[2161,76,1],[2162,2,1],[2162,47,1],[2163,4,1],[2163,28,1],
[2163,69,1],[2164,1,10],[2164,2,1],[2164,5,4],[2164,12,2],[2164,21,1],
[2164,27,1],[2164,45,1],[2164,55,1],[2164,65,1],[2164,66,1],
[2165,25,2],[2165,43,1],[2166,2,1],[2166,5,3],[2166,6,1],[2166,21,1],
[2166,22,8],[2166,23,3],[2166,25,1],[2166,26,2],[2166,27,20],
[2166,28,2],[2166,31,2],[2166,33,5],[2166,35,15],[2166,38,1],
[2166,48,1],[2166,56,1],[2167,15,1],[2167,17,1],[2167,53,1],
[2168,25,2],[2168,27,1],[2168,35,16],[2169,23,1],[2169,28,2],
[2169,35,1],[2169,62,1],[2169,66,2],[2170,15,1],[2170,17,1],
[2171,6,2],[2172,7,1],[2172,10,1],[2172,19,1],[2172,20,1],[2172,28,1],
[2172,67,2],[2172,71,2],[2173,23,1],[2173,28,4],[2173,47,1],
[2173,51,1],[2173,66,1],[2173,72,1],[2174,26,1],[2174,38,1],
[2175,2,6],[2175,3,2],[2175,5,2],[2175,6,1],[2175,22,1],[2175,23,2],
[2175,24,1],[2175,25,1],[2175,26,2],[2175,27,3],[2175,28,1],
[2175,40,1],[2175,56,1],[2175,72,1],[2175,77,1],[2176,0,2],
[2177,28,2],[2178,15,1],[2178,22,1],[2178,23,1],[2178,24,1],
[2178,43,1],[2178,50,1],[2179,20,3],[2179,22,1],[2179,35,1],
[2179,37,2],[2180,26,1],[2180,27,1],[2180,31,1],[2181,30,2],
[2182,22,1],[2182,42,1],[2182,44,1],[2183,24,1],[2183,33,3],
[2183,42,4],[2183,43,1],[2184,8,1],[2184,28,1],[2184,59,1],
[2185,15,1],[2185,17,2],[2185,53,5],[2185,55,1],[2185,73,1],
[2186,32,1],[2186,51,1],[2186,68,1],[2186,75,1],[2187,2,3],[2188,8,1],
[2188,20,1],[2188,26,1],[2188,27,3],[2189,23,1],[2189,31,2],
[2190,22,3],[2190,35,1],[2191,6,1],[2191,22,1],[2191,25,1],

[2191,27,1],[2191,28,4],[2191,32,287],[2191,33,2],[2191,40,1],
[2191,43,1],[2191,50,1],[2191,69,1],[2191,70,4],[2191,74,1],
[2191,75,1],[2192,2,4],[2192,21,2],[2192,22,1],[2192,23,2],
[2192,24,1],[2192,28,1],[2192,31,8],[2192,33,1],[2192,43,6],
[2192,50,1],[2193,61,2],[2194,3,1],[2194,27,2],[2194,63,1],[2195,6,2],
[2195,23,1],[2196,2,1],[2196,26,1],[2197,32,5],[2198,0,1],[2198,1,4],
[2198,2,2],[2198,3,1],[2198,7,1],[2198,12,1],[2198,13,1],[2198,14,1],
[2198,19,1],[2198,20,1],[2198,23,4],[2198,24,4],[2198,26,1],
[2198,28,1],[2198,30,1],[2198,31,2],[2198,32,2],[2198,41,2],
[2198,42,5],[2198,43,1],[2198,49,1],[2198,50,1],[2198,55,2],
[2198,59,1],[2198,65,2],[2198,69,1],[2199,43,1],[2199,54,2],
[2199,60,1],[2199,64,1],[2200,2,2],[2200,16,1],[2200,28,1],
[2201,26,1],[2201,42,1],[2202,23,7],[2202,27,2],[2202,35,1],
[2203,25,2],[2204,22,2],[2205,3,2],[2205,4,1],[2205,5,1],[2205,13,1],
[2205,22,4],[2205,23,3],[2205,24,1],[2205,26,1],[2205,27,5],
[2205,31,3],[2205,32,1],[2205,33,1],[2205,35,2],[2205,38,1],
[2205,43,2],[2205,44,1],[2205,70,2],[2205,72,1],[2205,75,1],
[2205,76,1],[2206,5,1],[2206,21,3],[2206,22,12],[2206,23,6],
[2206,24,1],[2206,26,3],[2206,27,6],[2206,28,4],[2206,32,1],
[2206,35,2],[2206,38,3],[2206,43,10],[2206,52,1],[2206,59,2],
[2207,28,1],[2207,33,1],[2208,15,2],[2208,37,4],[2208,41,1],
[2208,54,1],[2208,62,2],[2209,44,4],[2210,28,5],[2211,1,2],[2211,9,1],
[2211,12,1],[2211,14,1],[2211,65,1],[2212,1,1],[2212,21,15],
[2212,27,1],[2212,30,6],[2212,43,5],[2212,47,4],[2213,1,1],
[2213,39,1],[2214,4,1],[2214,50,1],[2214,70,1],[2215,3,1],[2215,5,1],
[2215,24,5],[2215,26,1],[2215,28,1],[2215,31,3],[2215,43,1],
[2216,2,1],[2216,22,1],[2217,5,1],[2217,50,1],[2217,63,11],
[2217,69,1],[2217,70,1],[2217,74,13],[2217,75,5],[2217,76,13],
[2218,28,3],[2219,26,1],[2219,44,1],[2219,68,1],[2219,70,1],
[2220,8,1],[2220,9,1],[2221,17,2],[2222,4,1],[2222,5,1],[2222,21,1],
[2222,50,3],[2223,2,1],[2223,5,1],[2223,8,1],[2223,22,6],[2223,23,4],
[2223,24,4],[2223,26,2],[2223,27,1],[2223,32,1],[2223,33,2],
[2223,35,4],[2223,37,1],[2223,42,1],[2223,43,1],[2223,51,1],
[2223,56,2],[2223,68,1],[2223,79,1],[2224,6,2],[2225,3,1],[2225,5,1],
[2225,23,1],[2225,26,1],[2226,2,1],[2226,23,1],[2226,27,2],
[2226,33,2],[2227,36,7],[2227,45,1],[2227,58,1],[2227,71,1],
[2228,3,1],[2228,7,1],[2228,24,1],[2229,2,1],[2229,4,28],[2229,5,2],
[2229,15,2],[2229,17,3],[2229,21,1],[2229,23,1],[2229,24,1],
[2229,27,2],[2229,33,1],[2229,35,1],[2229,42,1],[2229,44,1],
[2229,50,34],[2229,53,4],[2229,59,1],[2229,70,1],[2230,2,1],
[2230,31,1],[2231,12,2],[2231,23,1],[2231,26,1],[2231,27,3],
[2231,32,1],[2231,46,1],[2231,51,1],[2231,52,1],[2231,60,1],
[2231,65,1],[2232,2,3],[2233,6,1],[2233,15,1],[2233,20,1],[2233,24,4],
[2233,28,1],[2234,1,1],[2234,2,4],[2234,50,1],[2234,74,1],[2235,0,1],
[2235,8,4],[2235,43,1],[2235,53,1],[2236,3,1],[2236,26,2],[2237,12,1],
[2237,15,2],[2237,24,1],[2237,43,1],[2237,50,1],[2237,55,1],
[2238,14,1],[2238,16,1],[2238,46,1],[2238,53,1],[2239,15,1],
[2239,53,1],[2239,55,1],[2240,29,2],[2241,2,4],[2241,3,1],[2241,9,2],
[2241,10,2],[2241,12,5],[2241,15,1],[2241,18,1],[2241,19,2],
[2241,20,2],[2241,21,3],[2241,22,4],[2241,24,2],[2241,25,12],

[2241,26,4],[2241,28,1],[2241,29,1],[2241,31,14],[2241,33,2],
[2241,35,2],[2241,39,25],[2241,41,1],[2241,42,3],[2241,43,4],
[2241,47,1],[2241,62,8],[2241,73,1],[2241,78,1],[2242,3,1],[2242,5,2],
[2242,24,2],[2242,27,2],[2242,28,2],[2242,31,2],[2242,32,3],
[2242,70,1],[2243,28,1],[2243,75,1],[2244,21,1],[2244,40,1],
[2244,72,1],[2245,0,2],[2245,1,14],[2245,5,1],[2245,7,7],[2245,8,6],
[2245,9,30],[2245,10,45],[2245,11,23],[2245,12,20],[2245,13,10],
[2245,14,11],[2245,15,12],[2245,16,17],[2245,17,11],[2245,18,9],
[2245,19,27],[2245,20,15],[2245,24,1],[2245,25,2],[2245,26,1],
[2245,27,1],[2245,28,11],[2245,29,6],[2245,30,2],[2245,31,1],
[2245,33,2],[2245,34,12],[2245,35,2],[2245,36,7],[2245,37,8],
[2245,39,9],[2245,40,1],[2245,41,16],[2245,43,2],[2245,44,1],
[2245,45,19],[2245,46,9],[2245,48,1],[2245,49,9],[2245,51,1],
[2245,53,12],[2245,54,7],[2245,55,14],[2245,57,13],[2245,58,19],
[2245,59,13],[2245,60,4],[2245,62,1],[2245,63,1],[2245,64,2],
[2245,65,29],[2245,67,17],[2245,70,2],[2245,71,5],[2245,73,18],
[2245,75,1],[2245,76,8],[2245,77,13],[2245,78,3],[2246,0,1],
[2246,1,269],[2246,7,3],[2246,8,5],[2246,10,1],[2246,12,8],
[2246,13,23],[2246,15,2],[2246,17,4],[2246,20,4],[2246,29,5],
[2246,30,2],[2246,31,3],[2246,34,1],[2246,36,1],[2246,39,4],
[2246,41,1],[2246,45,1],[2246,53,7],[2246,55,3],[2246,59,3],
[2246,65,2],[2246,73,1],[2246,78,1],[2247,3,1],[2247,30,1],
[2247,37,1],[2248,2,3],[2248,8,2],[2248,23,1],[2248,25,2],[2248,27,1],
[2248,37,1],[2248,52,1],[2248,62,3],[2249,28,1],[2249,63,1],
[2250,15,2],[2251,49,1],[2251,54,1],[2252,22,1],[2252,55,2],
[2253,17,1],[2253,23,4],[2253,33,1],[2253,47,1],[2254,28,2],
[2255,55,2],[2256,7,3],[2257,20,2],[2257,31,3],[2258,27,3],
[2259,10,2],[2259,13,1],[2259,19,1],[2259,34,1],[2259,39,1],
[2259,45,1],[2259,53,1],[2259,57,1],[2259,59,1],[2259,65,1],
[2259,67,2],[2260,3,27],[2260,5,1],[2260,6,4],[2260,10,1],[2260,19,1],
[2260,20,1],[2260,24,33],[2260,25,1],[2260,26,7],[2260,27,10],
[2260,28,9],[2260,32,46],[2260,33,2],[2260,34,1],[2260,38,2],
[2260,39,4],[2260,40,1],[2260,42,6],[2260,43,5],[2260,47,6],
[2260,50,1],[2260,56,203],[2260,60,1],[2260,62,3],[2260,63,1],
[2260,66,1],[2260,73,1],[2260,74,1],[2260,75,1],[2260,77,2],
[2261,44,2],[2261,50,1],[2261,66,1],[2262,26,1],[2262,27,1],
[2262,42,1],[2263,23,5],[2264,44,1],[2264,66,1],[2265,15,1],
[2265,22,1],[2265,26,1],[2265,47,1],[2265,74,1],[2266,10,1],
[2266,12,2],[2266,20,13],[2266,26,1],[2266,28,1],[2266,39,4],
[2266,42,2],[2266,59,1],[2266,72,1],[2266,77,1],[2267,62,2],
[2268,24,2],[2268,26,5],[2268,43,1],[2269,2,2],[2269,3,1],[2269,23,1],
[2269,24,1],[2269,48,1],[2269,52,1],[2269,70,1],[2270,2,1],[2270,5,1],
[2271,5,1],[2271,20,10],[2271,22,1],[2271,23,22],[2271,26,8],
[2271,27,1],[2271,31,3],[2271,32,9],[2271,35,3],[2271,43,6],
[2272,4,1],[2272,10,1],[2272,17,1],[2272,50,1],[2273,1,4],[2273,12,1],
[2273,13,2],[2273,39,2],[2273,55,1],[2274,44,1],[2274,66,1],
[2275,0,1],[2275,1,1],[2275,9,1],[2275,10,1],[2275,12,1],[2275,17,2],
[2275,19,2],[2275,30,2],[2275,34,1],[2275,36,1],[2275,37,2],
[2275,44,1],[2275,45,1],[2275,50,1],[2275,55,1],[2275,57,1],
[2275,58,1],[2275,59,1],[2275,65,2],[2275,66,3],[2276,6,1],

[2276,22,3],[2276,24,2],[2276,27,3],[2276,35,3],[2276,56,3],
[2277,2,1],[2277,3,1],[2277,28,1],[2277,47,1],[2278,5,2],[2279,7,1],
[2279,23,2],[2279,28,6],[2280,26,1],[2280,28,1],[2281,24,1],
[2281,26,1],[2281,28,1],[2281,43,1],[2282,2,1],[2282,23,1],
[2282,27,2],[2282,31,1],[2282,35,1],[2283,25,2],[2284,21,1],
[2284,24,1],[2285,27,6],[2285,28,1],[2286,20,1],[2286,57,1],
[2287,34,3],[2288,3,1],[2288,5,1],[2288,21,1],[2288,23,1],[2288,26,1],
[2288,27,1],[2288,31,1],[2288,32,2],[2288,40,1],[2288,43,1],
[2288,64,1],[2289,2,1],[2289,3,7],[2289,4,2],[2289,5,1],[2289,6,3],
[2289,14,1],[2289,21,8],[2289,22,9],[2289,23,8],[2289,24,9],
[2289,25,2],[2289,26,6],[2289,27,5],[2289,28,2],[2289,31,1],
[2289,32,5],[2289,33,1],[2289,35,4],[2289,38,1],[2289,43,3],
[2289,44,1],[2289,45,1],[2289,48,1],[2289,56,2],[2289,63,1],
[2289,75,1],[2289,78,1],[2290,28,1],[2290,50,1],[2291,20,1],
[2291,28,1],[2292,28,2],[2293,3,6],[2293,5,1],[2293,6,2],[2293,15,1],
[2293,20,1],[2293,22,1],[2293,23,9],[2293,24,137],[2293,25,1],
[2293,26,2],[2293,27,3],[2293,28,9],[2293,32,1],[2293,33,3],
[2293,35,1],[2293,38,1],[2293,40,1],[2293,42,2],[2293,43,4],
[2293,47,3],[2293,55,1],[2293,56,15],[2293,61,2],[2293,62,2],
[2293,71,1],[2293,72,1],[2293,74,1],[2293,76,1],[2294,2,12],
[2294,28,1],[2295,6,1],[2295,27,1],[2295,32,13],[2295,33,1],
[2295,48,1],[2296,26,2],[2297,21,1],[2297,27,2],[2298,23,1],
[2298,26,1],[2299,47,4],[2299,52,1],[2300,2,1],[2300,28,1],
[2301,23,1],[2301,26,1],[2301,48,1],[2302,21,1],[2302,28,3],
[2303,2,1],[2303,35,1],[2303,43,1],[2303,46,1],[2304,5,2],[2304,23,2],
[2304,27,2],[2304,28,1],[2304,43,2],[2304,65,1],[2304,73,1],
[2305,20,1],[2305,35,3],[2305,38,1],[2305,58,1],[2305,63,1],
[2306,3,4],[2306,21,1],[2306,23,1],[2306,28,2],[2306,31,1],[2307,2,1],
[2307,14,2],[2307,23,1],[2307,32,2],[2307,37,3],[2308,21,1],
[2308,23,1],[2308,26,1],[2308,31,2],[2308,38,1],[2308,43,1],
[2308,59,3],[2308,76,1],[2309,4,1],[2309,17,1],[2309,50,4],
[2309,53,1],[2310,5,1],[2310,24,1],[2310,32,1],[2310,52,1],
[2310,79,1],[2311,28,1],[2311,43,1],[2312,2,3],[2313,8,1],[2313,28,2],
[2314,31,8],[2314,42,2],[2314,56,1],[2314,76,1],[2315,15,4],
[2315,17,1],[2315,35,1],[2315,55,1],[2315,68,1],[2315,69,1],
[2316,6,1],[2316,23,1],[2316,28,1],[2316,33,18],[2316,51,1],
[2317,22,1],[2317,27,2],[2318,1,1],[2318,13,1],[2318,20,2],
[2319,20,1],[2319,23,1],[2319,56,1],[2320,28,1],[2320,41,1],
[2321,33,3],[2322,2,6],[2323,23,1],[2323,28,2],[2323,31,1],[2324,2,3],
[2325,28,1],[2325,57,1],[2326,2,2],[2327,19,1],[2327,27,2],
[2328,31,5],[2329,27,1],[2329,28,1],[2330,3,4],[2330,5,1],[2330,22,1],
[2330,24,2],[2330,27,3],[2330,43,4],[2331,1,1],[2331,6,2],[2331,17,1],
[2332,2,1],[2332,3,1],[2332,8,1],[2332,30,3],[2332,31,3],[2332,64,1],
[2332,66,1],[2333,24,2],[2333,25,1],[2333,26,1],[2333,52,1],
[2334,3,4],[2334,30,1],[2334,74,1],[2335,38,2],[2336,28,1],
[2336,70,1],[2337,26,1],[2337,31,1],[2337,32,4],[2337,39,1],
[2338,3,2],[2338,22,1],[2338,23,3],[2338,24,4],[2338,26,7],
[2338,27,6],[2338,31,3],[2338,32,3],[2338,35,4],[2338,42,1],
[2338,43,1],[2338,50,1],[2338,56,1],[2339,5,1],[2339,68,1],
[2340,16,2],[2340,41,2],[2340,46,1],[2340,49,1],[2340,53,2],

[2340,54,1],[2341,5,1],[2341,21,3],[2341,25,3],[2341,27,1],
[2341,33,1],[2341,39,2],[2341,43,1],[2342,25,3],[2343,6,1],
[2343,28,2],[2343,35,1],[2344,23,1],[2344,26,1],[2344,43,4],
[2345,3,1],[2345,4,2],[2345,21,1],[2345,23,1],[2345,24,4],[2345,26,1],
[2345,27,1],[2345,32,2],[2345,33,1],[2345,35,1],[2345,62,1],
[2346,4,1],[2346,15,1],[2346,17,3],[2346,29,1],[2346,31,1],
[2346,53,3],[2346,55,2],[2347,2,9],[2347,28,1],[2347,64,1],
[2347,74,1],[2348,0,1],[2348,1,95],[2348,2,8],[2348,3,2],[2348,7,12],
[2348,8,3],[2348,9,19],[2348,10,13],[2348,11,5],[2348,12,6],
[2348,13,480],[2348,14,2],[2348,15,12],[2348,16,7],[2348,17,4],
[2348,18,3],[2348,19,8],[2348,20,10],[2348,21,6],[2348,22,1],
[2348,23,23],[2348,24,1],[2348,25,36],[2348,26,25],[2348,28,6],
[2348,29,1],[2348,30,2],[2348,31,16],[2348,32,1],[2348,33,88],
[2348,34,4],[2348,36,3],[2348,37,5],[2348,38,2],[2348,39,7],
[2348,41,4],[2348,42,30],[2348,43,55],[2348,45,3],[2348,46,5],
[2348,47,10],[2348,48,5],[2348,49,4],[2348,53,1],[2348,54,3],
[2348,55,4],[2348,57,9],[2348,58,9],[2348,59,7],[2348,60,2],
[2348,64,2],[2348,65,17],[2348,66,1],[2348,67,6],[2348,71,5],
[2348,73,6],[2348,77,4],[2348,78,4],[2349,33,14],[2350,25,2],
[2350,27,1],[2350,32,2],[2350,37,2],[2351,22,1],[2351,23,1],
[2351,32,1],[2351,34,1],[2352,1,1],[2352,2,1],[2353,32,1],[2353,43,5],
[2354,15,2],[2354,50,1],[2355,4,1],[2355,5,3],[2355,21,1],[2355,24,1],
[2355,25,4],[2355,26,1],[2355,27,1],[2355,35,1],[2355,53,1],
[2355,55,1],[2355,62,1],[2355,74,1],[2356,28,1],[2356,32,1],
[2357,4,1],[2357,50,2],[2358,2,3],[2359,6,1],[2359,20,1],[2359,28,5],
[2359,47,1],[2359,56,1],[2359,66,1],[2360,2,1],[2360,42,3],
[2361,23,1],[2361,74,1],[2362,8,1],[2362,9,1],[2362,25,2],[2362,27,1],
[2362,28,3],[2362,30,10],[2362,32,1],[2362,33,8],[2362,35,6],
[2362,42,2],[2362,49,1],[2362,52,13],[2362,76,1],[2363,3,1],
[2363,6,1],[2363,27,2],[2363,43,1],[2363,44,1],[2363,48,1],[2364,3,2],
[2364,24,1],[2364,28,2],[2364,35,2],[2365,29,1],[2365,40,1],
[2366,28,1],[2366,31,1],[2367,9,1],[2367,21,2],[2367,23,4],
[2367,26,1],[2367,27,1],[2367,28,3],[2367,31,3],[2367,32,2],
[2367,35,2],[2367,50,1],[2368,4,2],[2368,5,2],[2368,17,1],[2368,25,1],
[2368,28,1],[2368,50,2],[2368,53,1],[2368,61,1],[2368,66,1],
[2369,2,1],[2369,17,1],[2370,28,5],[2370,31,1],[2370,42,1],
[2370,69,1],[2371,5,1],[2371,22,1],[2371,23,1],[2371,26,1],
[2371,35,2],[2371,47,1],[2371,56,2],[2371,62,1],[2372,35,1],
[2372,70,1],[2373,6,1],[2373,22,1],[2373,27,1],[2373,34,1],
[2373,35,1],[2373,43,1],[2373,51,1],[2373,69,1],[2373,75,1],
[2374,25,1],[2374,28,4],[2375,4,1],[2375,28,2],[2376,28,2],
[2376,31,9],[2376,37,1],[2377,20,4],[2377,31,1],[2378,2,5],
[2378,66,1],[2379,20,1],[2379,23,1],[2380,7,3],[2380,8,1],[2381,12,3],
[2381,29,2],[2382,4,1],[2382,32,2],[2382,44,1],[2382,66,1],[2383,4,1],
[2383,10,1],[2383,11,1],[2383,15,1],[2383,17,1],[2383,53,1],
[2383,55,2],[2383,71,1],[2384,5,1],[2384,66,1],[2384,72,1],[2385,5,3],
[2385,26,1],[2385,29,1],[2385,71,1],[2386,28,1],[2386,43,1],
[2387,28,2],[2387,35,4],[2388,3,1],[2388,75,1],[2389,23,1],
[2389,28,1],[2390,14,1],[2390,36,1],[2391,15,2],[2391,19,1],
[2392,2,1],[2392,43,1],[2393,12,2],[2393,13,2],[2393,16,1],

[2393,28,1],[2393,29,1],[2393,39,1],[2393,44,1],[2393,53,1],
[2393,65,1],[2394,2,3],[2395,20,1],[2395,37,1],[2396,26,2],[2397,8,2],
[2397,30,1],[2398,0,12],[2398,1,157],[2398,2,17],[2398,3,42],
[2398,4,582],[2398,5,58],[2398,6,12],[2398,7,17],[2398,8,17],
[2398,9,122],[2398,10,102],[2398,11,46],[2398,12,83],[2398,13,102],
[2398,14,27],[2398,15,4467],[2398,16,58],[2398,17,273],[2398,18,24],
[2398,19,66],[2398,20,145],[2398,21,15],[2398,22,30],[2398,23,69],
[2398,24,56],[2398,25,37],[2398,26,47],[2398,27,56],[2398,28,389],
[2398,29,27],[2398,30,21],[2398,31,36],[2398,32,35],[2398,33,39],
[2398,34,32],[2398,35,58],[2398,36,16],[2398,37,38],[2398,38,184],
[2398,39,41],[2398,40,20],[2398,41,60],[2398,42,29],[2398,43,23],
[2398,44,2490],[2398,45,34],[2398,46,30],[2398,47,20],[2398,48,17],
[2398,49,16],[2398,50,755],[2398,51,16],[2398,52,9],[2398,53,259],
[2398,54,23],[2398,55,2786],[2398,56,34],[2398,57,31],[2398,58,25],
[2398,59,52],[2398,60,15],[2398,61,2],[2398,62,20],[2398,63,17],
[2398,64,11],[2398,65,67],[2398,66,2114],[2398,67,32],[2398,68,11],
[2398,69,16],[2398,70,52],[2398,71,27],[2398,72,21],[2398,73,21],
[2398,74,52],[2398,75,27],[2398,76,37],[2398,77,44],[2398,78,8],
[2398,79,2],[2399,9,1],[2399,20,1],[2399,25,1],[2400,5,1],[2400,21,1],
[2400,23,5],[2400,25,1],[2400,27,1],[2400,32,3],[2400,34,2],
[2400,35,2],[2400,38,1],[2400,39,1],[2400,53,1],[2400,56,1],
[2401,5,1],[2401,23,1],[2402,5,1],[2402,26,1],[2402,28,1],[2402,32,1],
[2402,56,1],[2403,0,1],[2403,2,1],[2403,3,1],[2403,5,1],[2403,6,1],
[2403,7,1],[2403,10,1],[2403,13,18],[2403,19,2],[2403,20,5],
[2403,23,9],[2403,24,1],[2403,25,3],[2403,26,5],[2403,27,8],
[2403,28,3],[2403,29,1],[2403,30,3],[2403,31,8],[2403,32,5],
[2403,33,1],[2403,35,4],[2403,36,1],[2403,37,1],[2403,39,7],
[2403,41,1],[2403,42,4],[2403,52,1],[2403,54,2],[2404,42,1],
[2404,62,1],[2405,3,1],[2405,28,1],[2406,3,1],[2406,23,3],[2406,35,1],
[2406,56,2],[2407,27,3],[2407,30,1],[2408,13,1],[2408,52,1],
[2409,10,1],[2409,19,1],[2409,37,1],[2409,40,1],[2409,46,1],
[2409,64,1],[2409,68,1],[2410,28,1],[2410,40,1],[2410,63,1],
[2411,2,1],[2411,28,1],[2412,20,1],[2412,45,1],[2413,9,2],[2413,13,1],
[2413,29,1],[2414,20,1],[2414,25,1],[2415,2,2],[2415,17,1],
[2415,31,1],[2415,50,1],[2416,17,1],[2416,53,3],[2417,28,1],
[2417,31,1],[2417,43,2],[2417,70,2],[2418,9,2],[2419,26,2],
[2419,27,1],[2419,43,2],[2419,48,5],[2420,27,2],[2420,32,1],
[2420,51,1],[2421,22,1],[2421,26,1],[2422,2,3],[2422,31,2],
[2422,35,1],[2423,24,1],[2423,30,2],[2423,35,1],[2424,8,2],
[2424,34,2],[2424,39,1],[2424,62,1],[2425,2,2],[2426,50,2],
[2427,28,1],[2427,42,1],[2427,68,1],[2428,2,3],[2428,3,33],[2428,4,1],
[2428,5,5],[2428,6,5],[2428,9,1],[2428,10,3],[2428,11,1],[2428,12,1],
[2428,19,2],[2428,21,211],[2428,22,2],[2428,23,30],[2428,24,95],
[2428,25,117],[2428,26,69],[2428,27,6],[2428,28,37],[2428,30,11],
[2428,31,140],[2428,32,54],[2428,33,105],[2428,34,2],[2428,35,4],
[2428,36,1],[2428,37,4],[2428,38,9],[2428,39,6],[2428,40,5],
[2428,42,16],[2428,43,71],[2428,44,3],[2428,45,1],[2428,46,1],
[2428,47,15],[2428,48,107],[2428,50,2],[2428,52,2],[2428,56,46],
[2428,59,15],[2428,60,3],[2428,61,6],[2428,62,6],[2428,63,4],
[2428,64,1],[2428,66,2],[2428,67,1],[2428,68,4],[2428,70,4],

[2428,72,2],[2428,74,3],[2428,75,2],[2428,76,3],[2428,78,7],
[2429,1,5],[2429,10,1],[2429,12,1],[2429,57,1],[2430,2,1],[2430,23,1],
[2431,3,2],[2432,6,2],[2432,24,2],[2432,26,1],[2432,27,2],[2432,42,1],
[2433,1,1],[2433,58,1],[2433,67,1],[2433,77,9],[2434,14,1],
[2434,65,1],[2435,25,1],[2435,76,1],[2436,46,1],[2436,54,1],
[2437,2,19],[2437,24,1],[2437,26,1],[2437,27,1],[2437,31,2],
[2437,37,1],[2437,56,1],[2438,3,3],[2438,19,1],[2438,20,1],
[2438,21,3],[2438,22,2],[2438,23,8],[2438,24,1],[2438,25,1],
[2438,26,3],[2438,27,9],[2438,28,3],[2438,33,1],[2438,35,1],
[2438,36,1],[2438,43,4],[2438,45,2],[2438,65,1],[2438,77,1],
[2439,47,1],[2439,60,1],[2440,1,1],[2440,12,1],[2441,5,1],[2441,21,5],
[2441,22,18],[2441,23,3],[2441,24,5],[2441,25,1],[2441,26,5],
[2441,28,1],[2441,32,1],[2441,35,10],[2441,42,2],[2441,43,5],
[2441,48,1],[2441,63,1],[2441,72,1],[2442,15,1],[2442,32,2],
[2443,58,1],[2443,73,8],[2443,77,1],[2444,12,2],[2445,63,1],
[2445,76,1],[2446,12,1],[2446,15,2],[2446,39,1],[2446,67,1],
[2447,17,1],[2447,53,1],[2448,38,1],[2448,44,17],[2448,66,12],
[2448,72,1],[2449,2,1],[2449,3,1],[2449,15,1],[2449,22,2],[2449,23,1],
[2449,24,1],[2449,26,1],[2449,28,1],[2449,30,1],[2449,31,1],
[2449,32,1],[2449,38,1],[2449,42,1],[2449,44,1],[2449,50,1],
[2449,51,3],[2449,56,1],[2449,66,3],[2449,69,5],[2449,70,6],
[2449,74,5],[2449,79,1],[2450,3,2],[2450,19,1],[2450,22,1],
[2450,26,1],[2450,27,3],[2450,33,1],[2450,47,2],[2450,56,1],
[2450,75,1],[2451,2,2],[2452,2,5],[2452,48,1],[2452,66,1],[2453,1,2],
[2453,2,2],[2453,3,1],[2453,23,4],[2453,24,1],[2453,25,2],[2453,26,1],
[2453,27,4],[2453,28,1],[2453,30,1],[2453,31,1],[2453,32,3],
[2453,37,2],[2453,39,1],[2453,56,1],[2453,62,1],[2453,70,2],
[2453,72,1],[2454,28,4],[2454,31,1],[2455,31,2],[2455,44,1],
[2456,9,1],[2456,20,2],[2456,59,1],[2457,26,2],[2457,40,1],
[2457,43,2],[2458,4,1],[2458,59,1],[2458,65,1],[2459,0,1],[2459,1,2],
[2459,9,1],[2459,10,4],[2459,11,2],[2459,12,1],[2459,13,1],
[2459,14,1],[2459,15,1],[2459,16,2],[2459,17,1],[2459,18,1],
[2459,19,2],[2459,23,1],[2459,27,1],[2459,28,22],[2459,35,1],
[2459,36,1],[2459,37,1],[2459,38,1],[2459,41,1],[2459,44,2],
[2459,46,1],[2459,48,1],[2459,49,1],[2459,50,1],[2459,54,2],
[2459,55,1],[2459,58,2],[2459,63,1],[2459,65,1],[2459,68,1],
[2459,74,1],[2459,77,2],[2460,28,2],[2460,79,1],[2461,22,1],
[2461,23,7],[2461,24,2],[2462,28,4],[2463,22,1],[2463,23,1],
[2464,41,1],[2464,62,1],[2465,9,1],[2465,24,1],[2465,42,1],[2466,5,1],
[2466,50,1],[2467,43,1],[2467,47,1],[2468,2,2],[2468,5,1],[2468,20,2],
[2468,24,1],[2469,24,14],[2469,32,3],[2469,38,1],[2469,56,1],
[2470,28,6],[2470,50,1],[2471,2,1],[2471,17,1],[2472,18,1],
[2472,20,21],[2472,26,10],[2472,35,31],[2472,58,1],[2472,64,1],
[2472,73,1],[2473,28,3],[2473,38,1],[2474,1,1],[2474,13,2],
[2474,17,1],[2474,46,1],[2475,4,1],[2475,17,1],[2475,43,1],
[2475,78,1],[2476,25,2],[2477,14,1],[2477,40,1],[2477,43,1],
[2477,62,1],[2478,24,1],[2478,31,1],[2478,35,2],[2479,2,8],
[2480,15,2],[2480,55,1],[2481,23,1],[2481,24,2],[2481,40,1],
[2481,56,1],[2482,3,1],[2482,22,1],[2482,23,8],[2482,31,2],
[2482,33,7],[2482,46,2],[2482,62,1],[2482,77,1],[2483,49,1],

[2483,53,1],[2484,2,1],[2484,8,1],[2484,28,1],[2485,30,1],[2485,44,1],
[2485,53,1],[2486,1,2],[2486,5,1],[2486,32,1],[2487,3,2],[2487,24,3],
[2487,28,2],[2487,57,1],[2488,28,1],[2488,56,1],[2489,27,1],
[2489,28,15],[2489,35,2],[2490,9,1],[2490,10,1],[2490,59,1],
[2491,28,1],[2491,63,1],[2492,9,1],[2492,22,1],[2492,30,1],
[2493,28,1],[2493,78,1],[2494,3,2],[2494,6,3],[2494,24,3],[2494,28,1],
[2494,55,1],[2494,57,1],[2495,22,1],[2495,23,2],[2495,28,1],
[2495,32,1],[2496,4,4],[2496,9,4],[2496,15,3],[2496,17,5],
[2496,28,10],[2496,31,1],[2496,38,1],[2496,53,9],[2496,55,5],
[2496,66,1],[2497,9,1],[2497,20,1],[2497,31,2],[2498,22,1],
[2498,24,1],[2498,47,6],[2498,51,12],[2498,56,1],[2498,66,1],
[2498,68,8],[2498,72,1],[2499,2,1],[2499,5,2],[2499,27,1],[2499,32,2],
[2499,56,1],[2500,2,1],[2500,28,1],[2501,28,2],[2501,43,1],
[2502,22,2],[2503,2,2],[2503,21,2],[2503,22,1],[2503,27,2],
[2503,76,1],[2504,22,1],[2504,26,5],[2504,27,1],[2504,28,2],
[2504,33,1],[2504,47,1],[2504,56,1],[2504,61,1],[2504,62,3],
[2504,69,13],[2504,70,1],[2504,72,3],[2505,15,2],[2505,55,1],
[2506,1,2],[2506,5,1],[2506,7,1],[2506,12,2],[2506,15,2],[2506,17,1],
[2506,19,1],[2506,20,1],[2506,35,1],[2506,36,2],[2506,39,1],
[2506,45,1],[2506,49,1],[2506,55,1],[2506,58,2],[2506,59,1],
[2506,64,1],[2506,68,1],[2507,0,1],[2507,1,7],[2507,2,22],[2507,3,3],
[2507,5,1],[2507,6,4],[2507,9,5],[2507,10,1],[2507,11,2],[2507,13,21],
[2507,15,2],[2507,17,2],[2507,19,2],[2507,20,21],[2507,21,5],
[2507,22,5],[2507,23,37],[2507,24,16],[2507,25,1],[2507,26,3],
[2507,27,9],[2507,28,8],[2507,30,6],[2507,31,3],[2507,32,2],
[2507,33,4],[2507,34,3],[2507,35,4],[2507,37,5],[2507,38,1],
[2507,39,1],[2507,41,3],[2507,42,3],[2507,45,2],[2507,46,1],
[2507,47,1],[2507,48,5],[2507,50,4],[2507,55,2],[2507,56,4],
[2507,57,1],[2507,58,2],[2507,59,6],[2507,60,2],[2507,62,3],
[2507,64,1],[2507,65,1],[2507,67,1],[2507,68,1],[2507,70,4],
[2507,72,1],[2507,74,3],[2507,75,1],[2507,76,12],[2507,77,2],
[2507,78,5],[2508,26,1],[2508,30,1],[2509,23,1],[2509,51,1],
[2509,68,2],[2510,4,13],[2510,8,1],[2510,15,3],[2510,17,15],
[2510,34,1],[2510,44,16],[2510,48,1],[2510,50,25],[2510,53,8],
[2510,55,3],[2510,65,1],[2510,66,6],[2511,51,1],[2511,56,1],
[2511,61,1],[2512,0,18],[2513,2,1],[2513,6,1],[2513,13,2],[2513,20,1],
[2513,22,3],[2513,23,1],[2513,24,5],[2513,27,1],[2513,28,1],
[2513,29,1],[2513,33,2],[2513,34,2],[2513,35,4],[2513,36,1],
[2513,42,5],[2513,45,1],[2513,48,3],[2513,56,1],[2513,59,1],
[2513,67,1],[2514,25,4],[2514,32,1],[2515,28,4],[2516,13,1],
[2516,20,1],[2517,1,1],[2517,22,2],[2517,25,2],[2517,26,1],
[2517,33,17],[2517,35,6],[2517,52,10],[2517,61,1],[2518,17,1],
[2518,47,1],[2519,3,2],[2519,13,1],[2519,26,1],[2519,28,2],
[2519,33,4],[2519,43,1],[2520,2,1],[2520,3,1],[2520,5,1],[2520,22,1],
[2520,25,3],[2520,27,1],[2520,35,1],[2520,52,1],[2520,66,1],
[2521,2,3],[2521,3,1],[2521,26,1],[2521,42,3],[2521,44,1],[2521,47,2],
[2521,66,1],[2522,2,4],[2522,8,6],[2522,14,1],[2522,21,1],[2522,31,9],
[2522,32,1],[2522,42,2],[2522,45,1],[2522,62,1],[2522,68,4],
[2522,70,1],[2523,26,1],[2523,43,1],[2524,3,1],[2524,8,1],[2524,28,1],
[2524,35,1],[2524,43,2],[2524,47,1],[2524,62,1],[2525,1,3],

[2525,2,17],[2525,3,5],[2525,5,5],[2525,7,1],[2525,10,1],[2525,13,3],
[2525,15,1],[2525,19,5],[2525,20,7],[2525,21,2],[2525,22,3],
[2525,23,13],[2525,24,49],[2525,25,6],[2525,26,7],[2525,27,6],
[2525,28,14],[2525,31,5],[2525,32,8],[2525,33,16],[2525,35,9],
[2525,40,1],[2525,42,18],[2525,43,8],[2525,44,1],[2525,45,1],
[2525,46,1],[2525,47,1],[2525,48,1],[2525,53,1],[2525,54,1],
[2525,55,1],[2525,56,4],[2525,57,2],[2525,59,1],[2525,60,1],
[2525,63,1],[2525,66,1],[2525,68,1],[2525,70,1],[2525,74,1],
[2525,76,1],[2526,2,2],[2526,30,1],[2527,4,1],[2527,25,2],[2527,32,1],
[2527,56,1],[2528,4,1],[2528,25,2],[2528,26,1],[2529,25,1],
[2529,28,1],[2529,62,1],[2530,32,2],[2530,43,1],[2531,2,1],[2531,3,2],
[2531,5,4],[2531,22,1],[2531,23,3],[2531,24,5],[2531,26,2],
[2531,28,2],[2531,32,2],[2531,35,1],[2531,43,1],[2531,47,3],
[2532,20,1],[2532,25,11],[2532,31,1],[2532,33,1],[2532,35,1],
[2532,43,1],[2533,1,1],[2533,9,1],[2533,10,2],[2533,13,1],[2533,15,3],
[2533,16,1],[2533,19,1],[2533,20,3],[2533,34,2],[2533,41,1],
[2533,53,1],[2533,55,3],[2533,59,3],[2533,60,1],[2533,65,1],
[2533,77,1],[2534,10,1],[2534,13,1],[2534,41,1],[2535,24,1],
[2535,37,1],[2535,75,1],[2536,23,2],[2536,24,1],[2536,27,1],
[2536,32,1],[2536,42,1],[2537,45,2],[2538,0,21],[2538,1,100],
[2538,2,1],[2538,7,8],[2538,8,31],[2538,9,6],[2538,10,5],[2538,11,3],
[2538,12,45],[2538,13,21],[2538,15,109],[2538,16,3],[2538,17,355],
[2538,18,2],[2538,19,2],[2538,20,16],[2538,23,2],[2538,26,1],
[2538,27,2],[2538,29,19],[2538,30,13],[2538,32,2],[2538,34,24],
[2538,35,1],[2538,36,21],[2538,37,3],[2538,39,45],[2538,44,1],
[2538,45,4],[2538,46,1],[2538,49,1],[2538,53,368],[2538,55,251],
[2538,59,17],[2538,64,2],[2538,65,1],[2538,66,1],[2538,67,1],
[2538,73,9],[2538,77,1],[2538,78,2],[2539,15,1],[2539,41,1],
[2539,53,1],[2539,55,2],[2540,4,3],[2540,10,1],[2540,18,1],
[2540,28,1],[2540,33,1],[2540,35,1],[2540,38,9],[2540,39,1],
[2540,44,15],[2540,50,3],[2540,56,1],[2540,63,6],[2540,65,2],
[2540,66,14],[2541,5,1],[2541,21,4],[2541,22,22],[2541,24,2],
[2541,25,2],[2541,26,4],[2541,27,6],[2541,28,3],[2541,31,8],
[2541,32,2],[2541,33,1],[2541,35,4],[2541,38,1],[2541,42,3],
[2541,43,6],[2541,52,1],[2542,5,3],[2542,11,1],[2542,15,1],
[2542,46,1],[2542,55,1],[2542,58,1],[2543,6,1],[2543,22,1],
[2543,27,4],[2543,42,2],[2543,56,1],[2544,11,1],[2544,27,1],
[2544,32,1],[2544,61,1],[2545,15,2],[2545,44,1],[2546,1,2],
[2547,24,1],[2547,56,1],[2548,21,1],[2548,24,1],[2548,28,1],
[2548,46,1],[2548,48,1],[2548,71,1],[2548,74,1],[2549,3,2],[2550,2,1],
[2550,23,3],[2550,31,1],[2551,44,3],[2551,66,2],[2552,3,1],[2552,5,1],
[2552,23,1],[2552,26,1],[2552,28,1],[2552,33,2],[2552,42,1],
[2552,44,1],[2552,47,1],[2552,48,2],[2552,70,1],[2553,37,3],
[2554,28,1],[2554,30,1],[2555,1,2],[2555,15,1],[2555,17,4],
[2555,20,1],[2555,45,1],[2555,53,4],[2555,55,3],[2555,73,1],
[2556,13,1],[2556,59,1],[2557,20,1],[2557,23,1],[2557,28,1],
[2558,2,5],[2558,3,5],[2558,5,4],[2558,6,1],[2558,22,1],[2558,24,5],
[2558,25,1],[2558,26,1],[2558,27,4],[2558,28,1],[2558,31,1],
[2558,33,1],[2558,42,1],[2558,63,1],[2558,76,1],[2559,3,1],
[2559,25,1],[2559,32,1],[2559,42,1],[2559,50,1],[2559,57,1],

[2560,3,1],[2560,19,1],[2560,20,1],[2560,30,1],[2560,51,1],
[2560,54,1],[2560,60,2],[2560,72,1],[2560,74,1],[2561,2,6],[2561,5,1],
[2561,8,1],[2561,14,1],[2561,20,2],[2561,22,2],[2561,23,1],
[2561,35,1],[2561,70,1],[2562,2,2],[2562,12,1],[2562,27,2],
[2563,21,1],[2563,62,1],[2564,20,1],[2564,23,1],[2564,24,1],
[2564,59,1],[2565,15,1],[2565,25,1],[2565,26,2],[2565,56,1],
[2566,3,1],[2566,20,1],[2566,31,21],[2566,33,2],[2566,56,1],
[2566,70,1],[2567,5,1],[2567,25,1],[2567,26,2],[2567,35,1],
[2567,44,1],[2567,74,2],[2568,3,1],[2568,20,2],[2568,23,3],
[2568,24,3],[2568,25,2],[2568,26,1],[2568,27,1],[2568,28,1],
[2568,32,1],[2568,33,4],[2568,42,1],[2568,47,2],[2569,28,1],
[2569,31,1],[2570,2,4],[2571,21,1],[2571,26,2],[2571,27,1],
[2571,31,2],[2571,33,1],[2571,43,3],[2571,70,1],[2572,3,1],
[2572,22,1],[2572,23,17],[2572,24,3],[2572,25,3],[2572,26,1],
[2572,27,2],[2572,28,13],[2572,32,119],[2572,43,1],[2572,47,19],
[2572,48,2],[2572,56,4],[2572,70,3],[2572,74,1],[2573,3,1],
[2573,20,4],[2573,22,1],[2573,23,4],[2573,24,1],[2573,26,1],
[2573,27,1],[2573,28,2],[2573,31,1],[2573,33,3],[2573,35,1],
[2573,42,1],[2573,51,2],[2573,56,1],[2573,68,1],[2573,70,2],
[2574,53,1],[2574,55,1],[2575,10,1],[2575,36,1],[2576,11,2],
[2577,30,1],[2577,36,1],[2577,46,1],[2578,28,3],[2579,2,42],
[2579,24,1],[2579,70,1],[2579,74,1],[2580,5,1],[2580,27,4],
[2580,30,1],[2580,42,1],[2580,43,1],[2580,46,1],[2581,74,1],
[2581,75,2],[2582,29,1],[2582,39,2],[2583,0,24],[2583,1,232],
[2583,2,3],[2583,3,6],[2583,4,1],[2583,6,1],[2583,7,11],[2583,8,2],
[2583,9,16],[2583,10,21],[2583,11,8],[2583,12,69],[2583,13,180],
[2583,14,7],[2583,15,7],[2583,16,14],[2583,17,8],[2583,18,3],
[2583,19,12],[2583,20,23],[2583,23,19],[2583,24,23],[2583,25,3],
[2583,26,5],[2583,27,38],[2583,28,7],[2583,29,526],[2583,30,12],
[2583,31,14],[2583,32,49],[2583,33,2],[2583,34,8],[2583,35,12],
[2583,36,5],[2583,37,5],[2583,38,1],[2583,39,11],[2583,40,1],
[2583,41,6],[2583,42,3],[2583,43,4],[2583,44,1],[2583,45,9],
[2583,46,5],[2583,48,1],[2583,49,3],[2583,50,1],[2583,52,1],
[2583,53,3],[2583,54,1],[2583,55,8],[2583,57,8],[2583,58,9],
[2583,59,14],[2583,60,3],[2583,62,54],[2583,63,2],[2583,64,2],
[2583,65,12],[2583,67,4],[2583,68,1],[2583,70,1],[2583,71,2],
[2583,72,1],[2583,73,3],[2583,74,1],[2583,77,42],[2583,78,2],
[2584,6,1],[2584,22,1],[2585,6,1],[2585,15,1],[2585,23,1],[2585,28,1],
[2585,31,1],[2586,1,1],[2586,20,1],[2587,1,2],[2587,2,1],[2587,6,1],
[2587,8,3],[2587,13,4],[2587,15,2],[2587,17,2],[2587,21,2],
[2587,23,1],[2587,24,3],[2587,26,1],[2587,27,5],[2587,28,1],
[2587,30,1],[2587,32,3],[2587,34,1],[2587,35,2],[2587,42,3],
[2587,47,5],[2587,50,1],[2587,52,1],[2587,53,1],[2587,55,2],
[2587,62,1],[2587,74,1],[2588,20,1],[2588,28,4],[2588,47,1],
[2589,4,1],[2589,32,4],[2589,69,2],[2590,15,1],[2590,32,1],
[2590,41,1],[2590,46,1],[2590,65,1],[2590,67,1],[2590,76,1],
[2591,4,1],[2591,37,1],[2591,56,1],[2591,74,1],[2592,1,1],[2592,3,3],
[2592,13,3],[2592,19,1],[2592,20,1],[2592,23,9],[2592,24,24],
[2592,62,1],[2593,26,2],[2594,28,2],[2594,43,1],[2595,24,1],
[2595,34,1],[2596,26,1],[2596,38,1],[2596,44,8],[2596,66,3],

[2597,6,2],[2597,24,4],[2597,26,1],[2597,42,1],[2598,30,1],
[2598,60,1],[2599,31,1],[2599,32,1],[2599,47,1],[2600,2,3],
[2600,3,16],[2600,5,2],[2600,6,6],[2600,8,2],[2600,13,1],[2600,20,12],
[2600,21,15],[2600,22,20],[2600,23,18],[2600,24,7],[2600,25,17],
[2600,26,16],[2600,27,91],[2600,28,25],[2600,31,17],[2600,32,59],
[2600,33,15],[2600,35,33],[2600,37,5],[2600,38,4],[2600,39,2],
[2600,42,16],[2600,43,19],[2600,44,1],[2600,47,5],[2600,48,5],
[2600,52,2],[2600,56,3],[2600,61,1],[2600,62,1],[2600,65,2],
[2600,66,1],[2600,70,1],[2600,75,2],[2601,4,2],[2601,50,1],
[2602,13,1],[2602,33,1],[2603,0,1],[2603,9,5],[2603,10,2],[2603,12,1],
[2603,13,1],[2603,16,1],[2603,19,3],[2603,25,1],[2603,28,6],
[2603,29,2],[2603,30,2],[2603,34,1],[2603,37,1],[2603,46,1],
[2603,53,3],[2603,55,1],[2603,65,2],[2603,71,1],[2604,1,1],
[2604,20,1],[2604,21,2],[2604,22,2],[2604,23,2],[2604,24,1],
[2604,27,1],[2604,31,1],[2604,32,2],[2604,35,2],[2604,43,3],
[2604,48,1],[2605,23,2],[2605,43,1],[2606,21,1],[2606,23,1],
[2606,26,1],[2607,19,1],[2607,30,1],[2608,25,1],[2608,26,2],
[2608,28,3],[2608,35,1],[2609,43,1],[2609,69,1],[2610,1,2],[2610,5,1],
[2610,8,2],[2610,10,2],[2610,19,1],[2610,25,1],[2610,28,7],
[2610,29,1],[2610,30,237],[2610,33,27],[2610,35,7],[2610,36,1],
[2610,37,1],[2610,39,1],[2610,43,1],[2610,52,9],[2610,53,1],
[2610,65,1],[2610,74,1],[2610,78,1],[2611,9,1],[2611,10,1],
[2612,10,1],[2612,19,1],[2613,27,1],[2613,30,1],[2614,3,3],
[2614,22,2],[2614,24,1],[2614,28,1],[2614,31,1],[2614,32,2],
[2614,34,1],[2614,47,1],[2614,62,2],[2615,9,1],[2615,10,1],
[2615,12,2],[2615,15,1],[2615,19,1],[2615,20,1],[2615,46,1],
[2615,53,2],[2615,58,1],[2615,65,1],[2615,71,1],[2616,1,2],
[2617,62,4],[2618,2,1],[2618,3,1],[2618,23,2],[2618,24,3],[2618,26,1],
[2618,28,1],[2618,31,1],[2618,42,2],[2619,45,1],[2619,70,1],
[2620,5,4],[2620,51,1],[2620,68,1],[2621,27,3],[2621,40,1],
[2622,45,1],[2622,77,1],[2623,3,1],[2623,22,1],[2623,30,1],[2624,7,1],
[2624,39,1],[2625,30,2],[2625,52,12],[2626,3,2],[2626,32,1],
[2626,42,1],[2626,56,2],[2626,66,1],[2626,68,3],[2627,15,1],
[2627,38,1],[2627,68,1],[2627,70,3],[2627,76,1],[2628,33,1],
[2628,35,1],[2628,47,1],[2629,40,1],[2629,68,2],[2629,70,4],
[2630,2,2],[2630,26,2],[2630,47,3],[2631,1,1],[2631,55,1],
[2632,28,27],[2632,35,1],[2632,42,1],[2632,62,1],[2633,70,1],
[2633,75,1],[2634,15,1],[2634,74,1],[2635,24,2],[2635,56,2],
[2636,0,3],[2636,26,1],[2637,6,1],[2637,20,2],[2637,21,2],[2637,27,1],
[2637,33,2],[2637,35,1],[2637,39,1],[2637,42,4],[2637,44,2],
[2638,10,1],[2638,19,1],[2639,9,3],[2639,10,3],[2639,11,1],
[2639,16,1],[2639,19,3],[2639,20,1],[2639,41,3],[2639,45,5],
[2639,49,1],[2639,54,1],[2639,57,3],[2639,58,2],[2639,65,4],
[2639,67,2],[2639,71,1],[2639,72,1],[2639,73,1],[2640,2,1],[2640,3,1],
[2640,28,1],[2641,62,2],[2642,2,1],[2642,62,15],[2643,23,1],
[2643,26,1],[2643,30,1],[2644,52,5],[2645,15,1],[2645,28,1],
[2645,55,1],[2646,5,1],[2646,9,1],[2646,11,1],[2646,12,1],[2646,15,1],
[2646,18,5],[2646,19,1],[2646,23,1],[2646,24,2],[2646,25,1],
[2646,27,29],[2646,29,1],[2646,31,3],[2646,32,8],[2646,35,1],
[2646,37,1],[2646,38,4],[2646,39,1],[2646,40,2],[2646,42,11],

[2646,47,4],[2646,48,2],[2646,50,2],[2646,53,1],[2646,56,38],
[2646,61,16],[2646,70,15],[2646,71,1],[2646,72,1],[2646,75,1],
[2646,78,2],[2647,4,1],[2647,15,1],[2647,17,3],[2647,28,1],
[2647,50,1],[2647,53,5],[2647,76,1],[2648,22,2],[2648,27,2],
[2648,28,2],[2648,35,1],[2648,50,1],[2649,20,2],[2649,25,1],
[2649,26,1],[2649,35,1],[2650,8,1],[2650,34,1],[2651,2,1],[2651,3,2],
[2651,21,1],[2651,22,1],[2651,23,1],[2651,25,1],[2651,27,1],
[2651,28,1],[2651,30,1],[2651,33,1],[2651,43,1],[2652,9,1],
[2652,28,1],[2653,13,3],[2653,23,1],[2653,24,1],[2653,25,2],
[2653,43,1],[2654,48,2],[2655,19,1],[2655,31,1],[2656,26,1],
[2656,27,1],[2656,43,1],[2657,17,1],[2657,50,1],[2657,53,1],
[2658,1,1],[2658,7,1],[2658,8,1],[2658,9,9],[2658,10,12],[2658,11,4],
[2658,12,4],[2658,14,1],[2658,15,94],[2658,16,6],[2658,17,4],
[2658,18,3],[2658,19,10],[2658,20,1],[2658,23,2],[2658,25,1],
[2658,27,1],[2658,28,7],[2658,32,1],[2658,33,1],[2658,34,8],
[2658,36,2],[2658,37,5],[2658,39,1],[2658,40,1],[2658,41,9],
[2658,42,1],[2658,45,11],[2658,46,5],[2658,48,1],[2658,49,3],
[2658,50,1],[2658,53,4],[2658,54,5],[2658,55,420],[2658,56,3],
[2658,57,6],[2658,58,2],[2658,59,2],[2658,60,4],[2658,63,1],
[2658,64,4],[2658,65,8],[2658,66,1],[2658,67,3],[2658,68,1],
[2658,69,1],[2658,70,1],[2658,71,2],[2658,73,4],[2658,77,6],
[2658,78,1],[2659,26,1],[2659,32,1],[2660,8,4],[2660,10,1],
[2661,22,1],[2661,23,1],[2661,24,2],[2661,36,2],[2662,4,1],[2662,5,1],
[2662,20,1],[2662,21,1],[2662,23,1],[2662,27,1],[2662,32,1],
[2662,35,2],[2662,48,1],[2662,49,1],[2662,62,1],[2663,15,1],
[2663,24,1],[2663,47,2],[2663,66,1],[2664,16,1],[2664,29,1],
[2664,30,1],[2664,76,2],[2665,20,1],[2665,36,1],[2665,37,1],
[2666,28,1],[2666,47,31],[2667,22,1],[2667,25,1],[2667,32,2],
[2667,57,1],[2668,22,1],[2668,28,1],[2668,62,2],[2669,32,2],
[2670,2,2],[2670,15,1],[2671,8,1],[2671,22,1],[2672,4,1],[2672,5,2],
[2672,6,1],[2672,13,1],[2672,15,5],[2672,19,1],[2672,20,2],
[2672,23,2],[2672,32,1],[2672,34,1],[2672,38,1],[2672,40,1],
[2672,41,1],[2672,44,1],[2672,50,1],[2672,51,1],[2672,55,2],
[2672,56,3],[2672,59,2],[2672,68,1],[2673,24,2],[2674,1,1],[2674,2,3],
[2674,12,1],[2674,23,5],[2674,25,2],[2674,27,11],[2674,28,1],
[2674,34,3],[2674,35,1],[2674,42,1],[2674,52,1],[2674,62,3],
[2675,66,2],[2676,5,1],[2676,28,2],[2676,38,1],[2677,4,1],[2677,9,3],
[2678,21,1],[2678,24,2],[2678,43,2],[2678,60,1],[2679,5,1],
[2679,22,1],[2679,24,1],[2680,6,1],[2680,23,2],[2681,2,1],[2681,28,1],
[2682,5,1],[2682,22,1],[2682,35,5],[2682,38,1],[2682,39,1],
[2682,59,4],[2682,70,1],[2683,22,1],[2683,35,1],[2684,25,3],
[2685,3,1],[2685,24,1],[2685,28,1],[2685,32,1],[2685,42,1],
[2685,74,2],[2686,6,1],[2686,28,1],[2687,2,117],[2687,23,2],
[2687,27,2],[2687,28,8],[2687,42,1],[2687,46,1],[2687,55,1],
[2687,56,1],[2687,64,1],[2688,15,5],[2688,55,1],[2689,19,1],
[2689,45,1],[2690,22,1],[2690,28,1],[2691,20,1],[2691,22,3],
[2691,26,2],[2691,27,4],[2691,28,1],[2691,30,1],[2691,32,1],
[2691,33,4],[2691,35,1],[2691,47,1],[2691,50,2],[2691,69,1],
[2692,2,1],[2692,6,1],[2692,22,1],[2692,24,1],[2692,27,1],[2692,33,1],
[2692,35,2],[2692,47,2],[2692,56,2],[2692,74,1],[2693,10,1],

[2693,20,1],[2694,21,1],[2694,42,1],[2695,24,1],[2695,26,1],
[2695,27,1],[2696,21,1],[2696,31,1],[2696,35,1],[2696,43,1],
[2697,28,1],[2697,32,5],[2697,47,1],[2698,38,2],[2699,2,1],
[2699,32,1],[2699,47,9],[2700,11,1],[2700,28,3],[2700,34,1],
[2700,63,1],[2701,2,2],[2701,3,2],[2701,21,2],[2701,23,4],[2701,27,5],
[2701,31,1],[2701,41,1],[2701,47,2],[2701,53,1],[2701,56,1],
[2701,72,1],[2701,74,1],[2702,33,8],[2703,40,3],[2704,50,1],
[2704,69,2],[2704,70,7],[2704,72,4],[2704,74,1],[2705,3,1],
[2705,22,1],[2705,24,1],[2705,27,1],[2705,35,1],[2705,38,1],
[2705,39,1],[2705,42,1],[2705,43,4],[2705,52,2],[2706,24,1],
[2706,27,1],[2707,1,1],[2707,67,1],[2708,4,1],[2708,23,1],[2708,38,1],
[2708,43,1],[2708,55,1],[2708,59,1],[2708,68,1],[2708,69,4],
[2708,70,6],[2708,74,11],[2709,8,1],[2709,27,1],[2709,28,2],
[2709,35,1],[2710,12,2],[2710,29,1],[2711,1,4],[2711,12,3],
[2711,29,3],[2712,1,20],[2712,2,2],[2712,12,1],[2712,13,5],
[2712,16,1],[2712,23,1],[2712,31,1],[2712,46,1],[2712,65,1],
[2712,68,1],[2712,71,1],[2712,78,1],[2713,2,2],[2714,1,3],[2714,4,5],
[2714,6,1],[2714,9,1],[2714,11,1],[2714,12,3],[2714,13,3],[2714,15,4],
[2714,16,2],[2714,17,8],[2714,19,2],[2714,20,1],[2714,23,1],
[2714,24,3],[2714,29,1],[2714,30,1],[2714,32,1],[2714,33,1],
[2714,36,1],[2714,40,2],[2714,43,1],[2714,45,4],[2714,48,1],
[2714,50,5],[2714,51,1],[2714,53,21],[2714,54,2],[2714,55,8],
[2714,56,1],[2714,57,1],[2714,59,3],[2714,62,1],[2714,63,1],
[2714,67,1],[2714,72,2],[2714,73,1],[2714,76,1],[2714,78,1],
[2715,4,1],[2715,25,1],[2715,31,1],[2715,42,1],[2716,28,1],
[2716,43,1],[2717,77,2],[2718,28,8],[2719,9,1],[2719,24,1],
[2719,25,1],[2719,26,1],[2719,27,1],[2719,28,7],[2719,32,1],
[2719,42,1],[2719,53,1],[2720,1,5],[2720,2,2],[2720,3,1],[2720,5,1],
[2720,6,1],[2720,10,1],[2720,12,17],[2720,13,9],[2720,15,3],
[2720,16,1],[2720,20,3],[2720,21,2],[2720,22,1],[2720,23,1],
[2720,24,1],[2720,26,1],[2720,31,1],[2720,43,1],[2720,47,1],
[2720,50,1],[2720,51,1],[2720,55,2],[2720,56,1],[2720,57,1],
[2720,59,1],[2720,60,1],[2720,63,1],[2720,66,2],[2720,67,1],
[2720,72,1],[2720,77,1],[2721,27,1],[2721,32,2],[2722,3,5],
[2722,33,1],[2723,15,1],[2723,20,1],[2723,27,16],[2723,34,1],
[2724,4,1],[2724,22,1],[2724,55,1],[2725,2,2],[2726,2,1],[2726,13,1],
[2726,24,2],[2726,28,1],[2726,31,1],[2726,39,1],[2726,42,1],
[2726,43,1],[2727,22,3],[2727,24,1],[2727,27,1],[2727,51,1],
[2728,3,5],[2728,5,1],[2728,22,1],[2728,23,3],[2728,24,30],
[2728,28,4],[2728,31,2],[2728,33,7],[2728,43,1],[2728,47,1],
[2728,56,4],[2728,76,3],[2729,3,1],[2729,4,3],[2729,5,2],[2729,9,1],
[2729,10,1],[2729,20,1],[2729,22,3],[2729,26,1],[2729,28,1],
[2729,34,1],[2729,38,3],[2729,44,6],[2729,50,4],[2729,66,10],
[2729,74,1],[2729,76,1],[2730,3,1],[2730,74,1],[2731,22,1],
[2731,24,2],[2731,26,1],[2731,32,6],[2732,33,3],[2733,2,14],
[2733,31,2],[2734,23,1],[2734,35,1],[2735,32,3],[2736,12,2],
[2736,13,1],[2737,25,1],[2737,26,1],[2737,44,1],[2737,61,1],
[2737,62,1],[2737,74,5],[2738,9,1],[2738,17,1],[2739,2,1],[2739,63,1],
[2739,74,1],[2740,5,1],[2740,25,1],[2740,31,1],[2741,22,1],
[2741,26,2],[2742,1,1],[2742,2,2],[2742,52,1],[2743,5,1],[2743,10,1],

[2743,15,1],[2744,31,5],[2744,42,1],[2744,43,1],[2744,75,1],
[2745,2,8],[2745,3,1],[2745,23,2],[2745,24,2],[2746,2,14],[2746,38,1],
[2746,47,1],[2747,22,1],[2747,28,1],[2747,50,1],[2747,70,1],
[2748,0,1],[2748,1,14],[2748,2,1],[2748,4,1],[2748,7,4],[2748,8,6],
[2748,9,50],[2748,10,56],[2748,11,45],[2748,12,20],[2748,13,11],
[2748,14,24],[2748,15,18],[2748,16,31],[2748,17,10],[2748,18,19],
[2748,19,45],[2748,20,12],[2748,21,1],[2748,22,1],[2748,24,1],
[2748,27,1],[2748,28,17],[2748,29,4],[2748,30,3],[2748,33,1],
[2748,34,17],[2748,36,9],[2748,37,13],[2748,39,9],[2748,40,3],
[2748,41,34],[2748,43,1],[2748,44,2],[2748,45,28],[2748,46,15],
[2748,49,9],[2748,53,8],[2748,54,11],[2748,55,11],[2748,57,30],
[2748,58,23],[2748,59,20],[2748,60,6],[2748,63,23],[2748,64,4],
[2748,65,44],[2748,67,21],[2748,68,1],[2748,70,1],[2748,71,16],
[2748,72,1],[2748,73,24],[2748,74,19],[2748,75,9],[2748,76,41],
[2748,77,26],[2748,78,1],[2748,79,4],[2749,44,1],[2749,50,1],
[2749,66,1],[2750,53,3],[2751,27,2],[2752,2,1],[2752,21,1],
[2752,24,1],[2752,35,1],[2753,29,1],[2753,71,1],[2754,30,2],
[2755,3,1],[2755,5,1],[2755,27,1],[2756,6,1],[2756,22,1],[2756,25,7],
[2756,28,1],[2756,43,1],[2756,56,2],[2757,3,2],[2757,23,2],
[2757,35,1],[2757,42,1],[2757,47,1],[2757,56,1],[2757,62,1],
[2757,68,1],[2757,74,1],[2758,3,1],[2758,21,3],[2758,43,1],
[2758,47,1],[2758,74,1],[2759,62,2],[2760,27,1],[2760,32,5],
[2760,35,2],[2760,63,1],[2761,2,2],[2761,20,1],[2761,72,1],
[2762,28,1],[2762,50,1],[2763,27,1],[2763,78,1],[2764,1,1],[2764,3,1],
[2764,15,1],[2764,20,2],[2764,21,1],[2764,22,1],[2764,24,2],
[2764,25,3],[2764,28,3],[2764,31,2],[2764,33,1],[2764,34,1],
[2764,35,2],[2764,39,2],[2764,43,1],[2764,59,3],[2764,62,1],
[2764,65,1],[2765,4,2],[2765,6,1],[2765,24,1],[2765,51,1],[2765,56,1],
[2765,69,1],[2765,74,4],[2766,10,1],[2766,11,1],[2766,16,1],
[2766,19,1],[2766,20,2],[2766,26,1],[2766,46,1],[2766,53,1],
[2766,65,1],[2766,76,1],[2766,77,1],[2767,16,1],[2767,23,1],
[2768,1,1],[2768,2,1],[2768,3,11],[2768,5,4],[2768,6,3],[2768,11,1],
[2768,20,2],[2768,21,10],[2768,22,35],[2768,23,53],[2768,24,18],
[2768,25,7],[2768,26,27],[2768,27,53],[2768,28,26],[2768,30,1],
[2768,31,14],[2768,32,22],[2768,33,13],[2768,35,42],[2768,36,1],
[2768,38,2],[2768,42,6],[2768,43,18],[2768,47,2],[2768,60,1],
[2768,62,2],[2769,12,1],[2769,13,3],[2769,20,7],[2769,34,1],
[2769,41,1],[2769,45,1],[2770,2,1],[2770,5,1],[2770,24,2],[2770,28,8],
[2770,31,1],[2770,45,1],[2770,48,1],[2770,52,1],[2770,66,1],
[2770,68,1],[2771,24,2],[2771,26,1],[2771,30,1],[2771,48,1],
[2772,40,12],[2772,68,6],[2772,69,10],[2772,70,18],[2772,72,9],
[2773,1,1],[2773,12,1],[2773,43,1],[2773,62,7],[2774,62,6],[2775,6,1],
[2775,28,1],[2775,32,2],[2775,38,2],[2776,20,1],[2776,57,1],
[2776,59,1],[2777,2,10],[2777,50,1],[2778,28,5],[2778,66,1],
[2779,2,1],[2779,20,1],[2779,39,1],[2780,24,1],[2780,25,1],
[2780,42,1],[2781,25,1],[2781,62,1],[2782,16,1],[2782,23,6],
[2782,24,6],[2782,26,2],[2782,37,1],[2782,43,2],[2782,48,1],
[2782,67,1],[2783,25,1],[2783,26,4],[2783,31,1],[2784,1,9],[2784,8,1],
[2784,13,1],[2784,24,5],[2784,27,1],[2784,29,1],[2784,35,2],
[2784,42,1],[2784,44,1],[2784,45,1],[2784,55,1],[2784,69,1],

[2784,73,1],[2785,33,1],[2785,35,1],[2786,8,17],[2786,13,1],
[2786,20,1],[2786,31,1],[2786,37,1],[2786,39,1],[2787,23,1],
[2787,24,1],[2788,35,1],[2788,43,1],[2789,2,2],[2789,3,1],[2789,6,1],
[2789,23,2],[2789,24,1],[2789,25,10],[2789,26,1],[2789,27,1],
[2789,28,1],[2789,31,4],[2789,35,4],[2789,56,2],[2790,2,3],
[2790,20,1],[2790,30,9],[2790,31,1],[2790,39,2],[2791,30,3],
[2792,45,1],[2792,73,1],[2793,24,1],[2793,70,1],[2794,26,1],
[2794,27,1],[2795,42,1],[2795,47,1],[2796,1,1],[2796,11,1],
[2796,29,1],[2796,59,1],[2797,31,1],[2797,59,1],[2798,13,1],
[2798,67,1],[2799,0,15],[2799,7,2],[2799,10,1],[2799,13,1],
[2799,14,1],[2799,19,1],[2799,34,1],[2799,37,1],[2799,57,1],
[2799,65,1],[2799,67,1],[2800,3,1],[2800,6,1],[2800,25,1],[2800,74,1],
[2801,26,1],[2801,70,1],[2802,0,3],[2802,7,2],[2803,2,4],[2803,6,1],
[2803,20,1],[2803,21,2],[2803,24,2],[2803,25,1],[2803,27,4],
[2803,31,2],[2803,33,3],[2803,35,10],[2803,42,1],[2803,45,3],
[2803,47,2],[2803,48,1],[2804,13,1],[2804,23,1],[2805,3,4],
[2805,22,1],[2805,25,1],[2805,32,1],[2805,48,10],[2805,62,1],
[2806,23,1],[2806,48,3],[2807,6,1],[2807,24,1],[2807,27,1],
[2808,56,2],[2809,5,1],[2809,27,1],[2809,32,2],[2809,33,1],[2810,2,2],
[2811,26,1],[2811,35,1],[2811,43,1],[2811,71,1],[2812,22,1],
[2812,25,1],[2813,11,1],[2813,18,1],[2813,59,1],[2814,63,3],
[2814,74,1],[2814,75,3],[2814,76,1],[2815,23,1],[2815,24,1],
[2815,31,4],[2815,32,3],[2816,1,4],[2816,36,1],[2817,23,1],
[2817,28,1],[2817,33,1],[2818,15,1],[2818,17,5],[2818,19,1],
[2818,29,1],[2818,45,1],[2818,50,1],[2818,53,5],[2818,73,1],
[2819,2,1],[2819,3,2],[2819,5,11],[2819,25,6],[2819,26,3],[2819,31,1],
[2819,32,3],[2819,35,38],[2819,40,3],[2819,42,2],[2819,62,19],
[2819,66,1],[2820,2,1],[2820,7,1],[2820,23,2],[2820,25,1],[2820,27,1],
[2820,28,4],[2820,35,4],[2821,0,15],[2821,1,96],[2821,7,22],
[2821,8,35],[2821,9,129],[2821,10,206],[2821,11,77],[2821,12,78],
[2821,13,70],[2821,14,48],[2821,15,89],[2821,16,109],[2821,17,37],
[2821,18,50],[2821,19,167],[2821,20,78],[2821,29,16],[2821,30,16],
[2821,34,45],[2821,36,30],[2821,37,67],[2821,39,26],[2821,41,98],
[2821,45,115],[2821,46,63],[2821,49,39],[2821,53,49],[2821,54,43],
[2821,55,67],[2821,57,85],[2821,58,56],[2821,59,61],[2821,60,23],
[2821,64,18],[2821,65,137],[2821,67,44],[2821,71,53],[2821,73,41],
[2821,77,63],[2821,78,22],[2822,1,1],[2822,5,2],[2822,7,1],[2822,8,1],
[2822,9,2],[2822,10,13],[2822,11,6],[2822,12,5],[2822,13,1],
[2822,14,6],[2822,15,15],[2822,16,2],[2822,17,4],[2822,18,4],
[2822,19,9],[2822,20,4],[2822,22,1],[2822,23,3],[2822,24,2],
[2822,28,5],[2822,29,1],[2822,33,1],[2822,36,4],[2822,39,1],
[2822,41,4],[2822,45,3],[2822,46,4],[2822,47,1],[2822,49,4],
[2822,50,1],[2822,53,8],[2822,54,4],[2822,55,17],[2822,57,7],
[2822,58,1],[2822,59,1],[2822,60,1],[2822,61,2],[2822,62,3],
[2822,64,1],[2822,65,13],[2822,66,2],[2822,68,1],[2822,70,2],
[2822,71,3],[2822,73,2],[2822,76,2],[2822,77,3],[2822,78,3],
[2823,2,4],[2823,4,1],[2823,23,1],[2823,31,1],[2824,1,3],[2824,2,1],
[2824,3,2],[2824,10,1],[2824,12,1],[2824,13,16],[2824,15,1],
[2824,16,1],[2824,20,25],[2824,21,2],[2824,22,1],[2824,23,6],
[2824,24,2],[2824,25,6],[2824,26,1],[2824,27,6],[2824,28,3],

[2824,31,1],[2824,32,9],[2824,33,2],[2824,34,3],[2824,35,2],
[2824,36,1],[2824,39,3],[2824,42,2],[2824,43,1],[2824,47,1],
[2824,55,1],[2824,59,3],[2824,67,1],[2824,71,1],[2825,8,4],
[2825,13,1],[2825,23,2],[2825,26,1],[2825,27,1],[2826,2,1],
[2826,34,1],[2827,11,1],[2827,13,6],[2827,45,1],[2827,47,1],
[2828,2,1],[2828,21,1],[2829,6,2],[2829,56,4],[2830,28,2],[2831,1,2],
[2831,5,1],[2831,7,1],[2831,9,10],[2831,10,6],[2831,11,1],[2831,12,1],
[2831,13,3],[2831,15,1],[2831,16,4],[2831,17,2],[2831,19,3],
[2831,20,7],[2831,34,3],[2831,36,1],[2831,37,1],[2831,39,4],
[2831,41,2],[2831,45,4],[2831,46,2],[2831,49,1],[2831,55,5],
[2831,57,6],[2831,58,1],[2831,59,3],[2831,60,3],[2831,65,8],
[2831,67,1],[2831,73,1],[2832,23,2],[2832,26,1],[2833,35,1],
[2833,38,1],[2834,43,2],[2835,23,1],[2835,24,2],[2835,32,1],
[2835,33,2],[2835,35,2],[2835,50,1],[2836,37,2],[2836,42,1],
[2836,48,1],[2836,62,1],[2837,31,4],[2838,25,1],[2838,27,1],
[2839,38,1],[2839,48,1],[2840,14,1],[2840,15,4],[2840,17,1],
[2840,55,3],[2840,75,1],[2841,33,2],[2841,69,1],[2842,5,1],
[2842,28,1],[2843,1,1],[2843,5,2],[2843,19,1],[2843,20,1],[2843,21,1],
[2843,24,1],[2843,27,2],[2843,31,1],[2843,32,2],[2843,33,1],
[2843,34,1],[2843,49,1],[2843,53,1],[2843,57,1],[2843,62,6],
[2844,6,2],[2844,47,1],[2844,48,2],[2845,2,2],[2846,31,3],[2847,12,1],
[2847,20,2],[2847,49,1],[2847,59,1],[2847,60,1],[2847,65,1],
[2848,31,2],[2849,1,1],[2849,7,1],[2850,3,1],[2850,6,3],[2850,21,2],
[2850,44,1],[2850,51,1],[2850,68,1],[2850,70,4],[2850,74,7],
[2851,22,1],[2851,24,2],[2851,28,3],[2851,56,3],[2851,79,1],
[2852,23,2],[2852,40,1],[2852,42,1],[2852,68,1],[2852,70,1],
[2853,34,1],[2853,55,1],[2854,23,1],[2854,28,1],[2855,22,1],
[2855,28,2],[2855,32,1],[2855,42,1],[2855,72,1],[2856,2,1],[2856,3,1],
[2857,24,1],[2857,56,1],[2858,2,30],[2858,27,1],[2858,28,1],
[2858,31,1],[2858,47,3],[2859,23,2],[2860,0,8],[2860,1,19],[2860,3,2],
[2860,7,8],[2860,8,13],[2860,9,61],[2860,10,82],[2860,11,31],
[2860,12,36],[2860,13,25],[2860,14,23],[2860,15,33],[2860,16,45],
[2860,17,5],[2860,18,22],[2860,19,63],[2860,20,18],[2860,29,9],
[2860,30,9],[2860,34,20],[2860,36,12],[2860,37,27],[2860,39,12],
[2860,40,2],[2860,41,46],[2860,45,47],[2860,46,23],[2860,49,15],
[2860,53,9],[2860,54,18],[2860,55,15],[2860,57,47],[2860,58,39],
[2860,59,30],[2860,60,8],[2860,64,8],[2860,65,68],[2860,67,32],
[2860,71,28],[2860,73,29],[2860,75,1],[2860,77,34],[2860,78,5],
[2861,28,1],[2861,47,1],[2861,70,1],[2862,1,1],[2862,7,1],[2862,8,1],
[2862,9,2],[2862,10,4],[2862,11,1],[2862,12,2],[2862,13,1],
[2862,14,1],[2862,15,1],[2862,16,2],[2862,17,1],[2862,18,1],
[2862,19,2],[2862,20,2],[2862,36,1],[2862,37,2],[2862,41,2],
[2862,49,2],[2862,53,1],[2862,57,2],[2862,58,2],[2862,59,2],
[2862,62,1],[2862,65,2],[2862,70,1],[2862,71,1],[2862,73,1],
[2862,77,4],[2863,20,1],[2863,22,1],[2863,24,2],[2863,26,2],
[2863,28,1],[2863,35,1],[2863,36,1],[2864,2,1],[2864,24,1],
[2864,27,1],[2864,31,12],[2864,32,4],[2864,37,1],[2864,42,1],
[2864,56,1],[2865,1,1],[2865,8,2],[2865,9,14],[2865,10,11],
[2865,11,10],[2865,12,3],[2865,13,1],[2865,14,1],[2865,15,5],
[2865,16,5],[2865,17,1],[2865,18,2],[2865,19,17],[2865,20,6],

[2865,29,2],[2865,30,1],[2865,34,3],[2865,36,3],[2865,37,3],
[2865,39,2],[2865,41,9],[2865,45,7],[2865,46,2],[2865,49,1],
[2865,53,1],[2865,54,5],[2865,55,2],[2865,57,5],[2865,58,4],
[2865,59,7],[2865,60,1],[2865,64,2],[2865,65,7],[2865,67,2],
[2865,71,3],[2865,73,4],[2865,77,4],[2865,78,1],[2866,26,1],
[2866,31,1],[2867,9,1],[2867,12,1],[2867,17,1],[2867,59,1],[2868,1,4],
[2868,12,5],[2868,15,1],[2868,25,1],[2868,28,2],[2868,29,1],
[2869,20,1],[2869,22,13],[2869,23,3],[2869,27,1],[2870,30,1],
[2870,33,1],[2870,58,2],[2871,15,2],[2872,4,1],[2872,28,2],
[2872,55,2],[2872,74,1],[2873,4,2],[2873,28,1],[2874,1,3],[2874,8,3],
[2874,12,1],[2874,13,2],[2874,15,1],[2874,17,2],[2874,18,1],
[2874,20,2],[2874,23,20],[2874,28,1],[2874,29,1],[2874,31,1],
[2874,34,4],[2874,36,1],[2874,45,44],[2874,48,1],[2874,76,1],
[2875,2,1],[2875,28,1],[2876,22,1],[2876,51,7],[2876,61,1],
[2876,68,1],[2876,70,1],[2877,25,6],[2877,28,1],[2878,1,1],
[2878,25,1],[2878,51,3],[2878,56,2],[2878,68,1],[2878,69,2],
[2879,5,2],[2879,22,1],[2879,25,2],[2879,35,2],[2880,10,1],
[2880,11,1],[2880,29,1],[2880,34,1],[2880,36,2],[2880,37,2],
[2880,46,1],[2880,54,1],[2880,57,1],[2880,58,1],[2880,65,1],
[2881,31,1],[2881,66,1],[2882,3,1],[2882,32,2],[2882,50,1],[2883,5,1],
[2883,28,1],[2884,1,1],[2884,20,1],[2884,45,1],[2884,60,1],
[2885,21,2],[2886,1,12],[2886,8,1],[2886,9,2],[2886,10,4],[2886,12,4],
[2886,13,2],[2886,15,1],[2886,16,2],[2886,17,3],[2886,19,2],
[2886,20,4],[2886,29,1],[2886,30,2],[2886,34,1],[2886,37,1],
[2886,39,2],[2886,41,2],[2886,45,2],[2886,46,2],[2886,49,1],
[2886,53,1],[2886,54,1],[2886,55,1],[2886,57,1],[2886,58,1],
[2886,59,1],[2886,65,1],[2886,73,1],[2886,77,2],[2887,1,1],[2887,8,1],
[2887,13,6],[2887,20,1],[2887,28,1],[2887,31,10],[2887,32,1],
[2887,34,1],[2887,39,1],[2888,22,1],[2888,27,1],[2888,40,1],
[2889,34,1],[2889,62,1],[2890,27,5],[2891,0,17],[2891,1,1890],
[2891,4,1],[2891,7,33],[2891,8,53],[2891,9,7],[2891,10,8],[2891,11,3],
[2891,12,294],[2891,13,61],[2891,14,1],[2891,15,19],[2891,16,7],
[2891,17,52],[2891,19,1],[2891,20,37],[2891,23,1],[2891,25,1],
[2891,27,2],[2891,29,242],[2891,30,11],[2891,31,1],[2891,34,60],
[2891,35,1],[2891,36,33],[2891,37,7],[2891,39,95],[2891,45,3],
[2891,46,3],[2891,53,51],[2891,54,1],[2891,55,31],[2891,57,2],
[2891,58,2],[2891,59,13],[2891,60,1],[2891,72,1],[2891,73,15],
[2891,77,9],[2891,78,5],[2892,28,3],[2893,4,1],[2893,22,1],
[2893,28,1],[2894,3,2],[2894,5,1],[2894,6,3],[2894,23,1],
[2894,24,128],[2894,25,1],[2894,27,1],[2894,28,2],[2894,32,1],
[2894,50,1],[2894,56,40],[2894,66,1],[2894,68,1],[2894,69,1],
[2894,74,1],[2894,76,1],[2895,28,15],[2896,62,4],[2897,3,1],
[2897,42,1],[2897,65,1],[2898,28,10],[2899,4,3],[2899,5,1],
[2899,12,1],[2899,26,1],[2899,28,1],[2899,32,4],[2899,50,5],
[2900,8,2],[2900,20,1],[2900,24,1],[2900,34,5],[2900,36,3],
[2901,28,1],[2901,32,1],[2901,42,1],[2902,28,1],[2902,50,3],
[2902,66,2],[2903,28,1],[2903,66,1],[2904,27,5],[2904,48,1],
[2905,9,1],[2905,19,1],[2905,33,1],[2906,0,5],[2906,1,9],[2906,3,2],
[2906,6,1],[2906,7,1],[2906,8,1],[2906,10,1],[2906,12,7],[2906,13,4],
[2906,15,2],[2906,17,6],[2906,19,1],[2906,20,6],[2906,22,2],

[2906,24,3],[2906,25,1],[2906,26,1],[2906,27,1],[2906,28,1],
[2906,29,2],[2906,30,2],[2906,31,1],[2906,32,1],[2906,33,2],
[2906,34,8],[2906,35,2],[2906,36,3],[2906,39,4],[2906,44,1],
[2906,45,2],[2906,50,1],[2906,52,1],[2906,53,3],[2906,54,2],
[2906,55,5],[2906,56,1],[2906,58,1],[2906,60,1],[2906,65,1],
[2906,73,1],[2906,77,1],[2907,20,2],[2908,2,2],[2908,3,9],[2908,23,1],
[2908,31,1],[2908,33,1],[2908,63,1],[2908,72,1],[2908,78,1],
[2909,2,1],[2909,5,1],[2909,20,1],[2909,22,3],[2909,23,1],[2909,25,1],
[2909,28,1],[2909,32,1],[2909,56,1],[2910,15,3],[2910,17,3],
[2910,55,1],[2910,62,1],[2910,70,1],[2910,74,1],[2911,11,1],
[2911,16,1],[2912,10,1],[2912,16,2],[2912,19,1],[2912,39,1],
[2912,55,2],[2912,59,1],[2913,44,1],[2913,62,1],[2914,31,7],
[2915,1,10],[2915,20,1],[2915,23,1],[2915,33,1],[2916,23,2],
[2916,26,1],[2917,30,31],[2918,26,3],[2918,28,1],[2918,35,1],
[2918,65,1],[2919,33,3],[2920,15,1],[2920,55,1],[2921,20,1],
[2921,32,1],[2921,45,1],[2922,53,1],[2922,59,1],[2923,2,1],[2923,4,3],
[2923,9,12],[2923,10,1],[2923,14,1],[2923,15,24],[2923,17,16],
[2923,19,3],[2923,25,1],[2923,28,9],[2923,34,2],[2923,45,2],
[2923,46,1],[2923,50,6],[2923,53,14],[2923,54,1],[2923,55,2],
[2923,57,1],[2923,59,2],[2923,66,2],[2923,67,3],[2923,77,1],
[2924,9,1],[2924,10,1],[2924,65,1],[2925,24,2],[2925,56,1],
[2926,12,1],[2926,31,2],[2927,27,2],[2927,28,2],[2928,2,2],[2928,8,1],
[2928,35,1],[2929,43,2],[2930,5,1],[2930,43,2],[2930,62,4],[2931,2,1],
[2931,16,1],[2931,21,1],[2931,22,1],[2931,26,1],[2931,28,1],
[2931,31,2],[2931,35,1],[2931,43,1],[2932,3,1],[2932,30,2],
[2932,67,1],[2933,1,1],[2933,18,1],[2933,19,1],[2933,45,2],[2934,2,3],
[2935,1,1],[2935,2,9],[2935,3,7],[2935,4,1],[2935,5,6],[2935,9,4],
[2935,10,1],[2935,11,2],[2935,12,2],[2935,13,1],[2935,15,2],
[2935,17,1],[2935,20,21],[2935,22,1],[2935,23,11],[2935,24,19],
[2935,26,5],[2935,27,11],[2935,28,5],[2935,30,3],[2935,31,18],
[2935,32,5],[2935,33,2],[2935,35,1],[2935,36,1],[2935,37,2],
[2935,39,1],[2935,42,5],[2935,43,7],[2935,44,1],[2935,47,6],
[2935,54,1],[2935,55,1],[2935,56,1],[2935,64,1],[2935,67,1],
[2935,68,1],[2935,74,2],[2935,77,1],[2936,31,2],[2936,32,2],
[2937,5,3],[2938,0,21],[2938,1,71],[2938,3,2],[2938,4,3],[2938,5,3],
[2938,6,3],[2938,7,6],[2938,8,20],[2938,9,32],[2938,10,39],
[2938,11,12],[2938,12,39],[2938,13,9],[2938,14,7],[2938,15,18],
[2938,16,11],[2938,17,10],[2938,18,7],[2938,19,13],[2938,20,33],
[2938,21,3],[2938,22,91],[2938,23,1],[2938,24,8],[2938,25,2],
[2938,26,1],[2938,27,62],[2938,28,7],[2938,29,32],[2938,30,36],
[2938,31,6],[2938,32,8],[2938,33,4],[2938,34,13],[2938,35,3],
[2938,36,4],[2938,37,9],[2938,38,8],[2938,39,51],[2938,41,15],
[2938,42,3],[2938,43,29],[2938,44,1],[2938,45,20],[2938,46,14],
[2938,47,1],[2938,48,4],[2938,49,6],[2938,52,4],[2938,53,11],
[2938,54,5],[2938,55,16],[2938,56,1],[2938,57,11],[2938,58,6],
[2938,59,16],[2938,60,5],[2938,62,3],[2938,64,6],[2938,65,24],
[2938,66,1],[2938,67,6],[2938,68,2],[2938,70,1],[2938,71,10],
[2938,73,23],[2938,76,1],[2938,77,11],[2938,78,2],[2939,3,2],
[2939,15,1],[2939,24,1],[2939,56,4],[2940,2,14],[2940,3,4],[2940,5,1],
[2940,6,1],[2940,26,1],[2940,30,1],[2940,35,1],[2940,41,1],

[2940,47,1],[2940,56,2],[2940,58,1],[2940,69,1],[2940,74,1],
[2940,76,1],[2941,3,3],[2941,23,1],[2941,24,1],[2941,25,1],
[2941,27,1],[2941,28,1],[2941,35,2],[2941,38,2],[2941,43,1],
[2941,47,1],[2942,6,1],[2942,27,1],[2942,32,4],[2942,40,1],
[2942,47,2],[2942,56,1],[2942,61,1],[2943,76,2],[2944,46,2],
[2945,31,1],[2945,32,1],[2946,20,1],[2946,25,1],[2946,31,1],
[2946,39,1],[2947,28,2],[2948,2,1],[2948,3,1],[2948,4,1],[2948,5,1],
[2948,9,1],[2948,12,1],[2948,15,1],[2948,19,1],[2948,21,1],
[2948,22,1],[2948,24,1],[2948,28,1],[2948,31,1],[2948,35,1],
[2948,38,1],[2948,40,1],[2948,41,1],[2948,47,2],[2948,50,1],
[2948,52,1],[2948,55,1],[2948,56,2],[2948,63,1],[2948,67,1],
[2948,68,1],[2948,69,2],[2948,70,3],[2948,74,1],[2948,77,3],
[2949,7,1],[2949,53,1],[2949,59,1],[2950,3,1],[2950,5,77],[2950,6,1],
[2950,21,19],[2950,22,22],[2950,23,1],[2950,25,2],[2950,27,2],
[2950,28,13],[2950,33,1],[2950,34,1],[2950,35,4],[2950,40,4],
[2950,43,1],[2950,52,2],[2950,56,1],[2950,59,1],[2950,61,1],
[2950,62,1],[2950,63,2],[2950,65,1],[2950,66,1],[2950,68,4],
[2950,69,3],[2950,70,7],[2950,72,7],[2950,74,2],[2950,75,1],
[2950,76,5],[2951,9,1],[2951,15,1],[2952,4,3],[2953,32,1],[2953,74,1],
[2954,27,1],[2954,42,1],[2955,0,14],[2955,1,1],[2955,15,1],
[2955,20,1],[2955,42,3],[2956,10,1],[2956,13,1],[2956,39,1],
[2956,57,1],[2957,13,1],[2957,27,1],[2957,69,1],[2957,70,2],
[2957,72,1],[2958,2,1],[2958,8,1],[2958,9,1],[2958,23,3],[2959,28,1],
[2959,30,1],[2959,47,1],[2959,50,1],[2959,51,2],[2959,56,1],
[2959,66,1],[2959,68,1],[2959,70,1],[2960,23,1],[2960,28,1],
[2960,43,1],[2961,2,2],[2961,5,1],[2961,6,2],[2961,27,1],[2961,50,1],
[2961,75,1],[2962,28,1],[2962,51,1],[2963,25,2],[2964,30,1],
[2964,34,1],[2965,35,1],[2965,40,2],[2965,47,1],[2965,68,1],
[2965,70,4],[2965,72,1],[2965,74,2],[2965,76,1],[2966,27,1],
[2966,42,1],[2966,70,1],[2967,24,1],[2967,35,2],[2968,31,1],
[2968,32,1],[2968,33,1],[2968,63,1],[2969,3,1],[2969,4,1],[2969,5,1],
[2969,24,1],[2969,27,1],[2969,28,41],[2969,32,2],[2969,35,1],
[2969,48,1],[2969,75,1],[2970,28,1],[2970,44,3],[2970,50,2],
[2970,66,2],[2971,2,26],[2971,28,1],[2971,76,1],[2972,3,1],[2972,4,7],
[2972,5,1],[2972,13,1],[2972,15,6],[2972,17,3],[2972,24,1],
[2972,27,1],[2972,28,3],[2972,44,6],[2972,50,12],[2972,53,4],
[2972,55,7],[2972,66,4],[2972,67,1],[2972,70,1],[2972,78,1],
[2973,33,2],[2974,23,1],[2974,35,1],[2975,4,2],[2975,17,1],
[2975,25,1],[2975,31,2],[2975,44,23],[2975,50,2],[2975,66,18],
[2975,70,1],[2976,56,4],[2977,4,1],[2977,28,52],[2977,63,16],
[2977,69,3],[2977,74,8],[2977,75,5],[2977,76,26],[2978,23,1],
[2978,50,3],[2978,53,4],[2978,55,3],[2978,56,1],[2978,62,1],
[2979,6,1],[2979,30,1],[2980,27,2],[2981,21,1],[2981,26,1],
[2981,27,1],[2981,35,1],[2981,47,1],[2981,63,1],[2981,66,1],
[2981,72,3],[2981,74,1],[2982,28,3],[2982,31,1],[2983,2,1],
[2983,28,1],[2984,21,1],[2984,43,1],[2985,23,1],[2985,31,1],
[2986,9,1],[2986,19,1],[2986,41,1],[2986,44,1],[2986,63,1],
[2986,68,1],[2987,23,1],[2987,27,1],[2987,56,1],[2988,26,1],
[2988,27,1],[2988,28,1],[2989,4,1],[2989,43,1],[2989,56,1],
[2990,27,3],[2991,11,1],[2991,45,1],[2991,49,3],[2991,59,1],

[2991,64,1],[2991,71,1],[2992,15,1],[2992,53,1],[2993,25,4],
[2993,28,2],[2994,28,2],[2995,4,1],[2995,17,1],[2995,44,1],
[2995,50,1],[2995,53,4],[2995,55,2],[2995,78,1],[2996,13,2],
[2996,23,1],[2996,24,3],[2996,25,1],[2996,39,3],[2996,62,1],
[2996,75,1],[2997,24,1],[2997,45,3],[2998,27,1],[2998,28,1],
[2999,5,2],[2999,9,1],[2999,35,2],[2999,37,3],[2999,47,2],[2999,60,1],
[2999,78,1],[3000,21,1],[3000,28,2],[3000,55,2],[3001,1,1],
[3001,14,1],[3002,0,18],[3002,1,1],[3002,7,5],[3002,8,1],[3002,10,1],
[3002,12,2],[3002,16,1],[3002,17,2],[3002,29,1],[3002,30,1],
[3002,36,4],[3002,39,1],[3002,46,1],[3002,57,1],[3002,59,1],
[3002,64,1],[3002,65,3],[3002,67,2],[3002,71,1],[3002,73,1],
[3003,2,2],[3003,23,2],[3003,24,3],[3003,26,1],[3003,28,2],
[3003,32,1],[3003,35,1],[3003,42,1],[3003,59,1],[3004,2,1],
[3004,24,1],[3004,43,1],[3005,2,1],[3005,47,1],[3006,1,1],[3006,3,1],
[3006,29,1],[3006,30,18],[3006,53,1],[3006,55,1],[3007,2,1],
[3007,4,1],[3007,20,2],[3007,23,2],[3007,25,2],[3007,26,2],
[3007,27,2],[3007,31,3],[3007,32,1],[3008,5,7],[3008,27,1],
[3008,32,1],[3008,35,1],[3008,44,1],[3008,47,1],[3008,68,1],
[3009,12,1],[3009,24,1],[3009,31,1],[3009,35,1],[3010,22,1],
[3010,23,1],[3011,2,3],[3011,21,1],[3012,23,1],[3012,26,1],
[3012,27,3],[3012,32,2],[3012,35,2],[3013,31,1],[3013,35,1],
[3013,57,1],[3013,63,3],[3013,70,1],[3013,74,4],[3013,75,5],
[3013,76,2],[3014,3,1],[3014,5,1],[3014,11,1],[3014,13,2],[3014,25,5],
[3014,28,1],[3014,39,1],[3014,59,2],[3014,62,1],[3014,70,1],
[3015,3,2],[3015,28,1],[3016,26,1],[3016,33,1],[3016,35,3],
[3016,70,1],[3017,2,2],[3018,1,1],[3018,10,1],[3018,12,1],[3018,14,1],
[3018,15,1],[3018,16,1],[3018,19,1],[3018,20,2],[3018,29,3],
[3018,41,2],[3018,53,1],[3018,55,1],[3018,59,1],[3018,78,1],
[3019,27,1],[3019,32,1],[3019,43,1],[3020,33,2],[3020,35,9],
[3021,12,1],[3021,17,1],[3021,29,1],[3021,53,2],[3021,55,1],
[3021,57,1],[3022,4,3],[3022,17,2],[3022,50,1],[3023,12,1],
[3023,20,7],[3023,28,1],[3023,29,1],[3023,50,1],[3023,55,1],
[3023,65,1],[3024,2,2],[3024,5,4],[3024,20,1],[3024,26,1],[3024,28,1],
[3024,43,1],[3024,47,1],[3024,50,1],[3025,7,3],[3025,18,1],
[3025,31,1],[3025,39,1],[3025,71,1],[3026,43,5],[3027,13,1],
[3027,20,1],[3027,67,1],[3028,1,3],[3028,2,36],[3028,9,1],[3028,10,1],
[3028,11,2],[3028,12,7],[3028,13,9],[3028,14,1],[3028,16,2],
[3028,19,4],[3028,20,13],[3028,23,1],[3028,28,2],[3028,30,4],
[3028,31,8],[3028,32,17],[3028,39,4],[3028,42,3],[3028,46,2],
[3028,47,1],[3028,57,1],[3028,58,1],[3028,60,1],[3029,47,1],
[3029,51,1],[3030,2,2],[3031,68,1],[3031,70,2],[3031,72,1],[3032,1,5],
[3032,27,1],[3032,32,1],[3032,43,1],[3033,4,1],[3033,5,10],
[3033,20,3],[3033,35,2],[3033,37,1],[3033,40,1],[3034,15,2],
[3035,31,2],[3036,21,1],[3036,22,8],[3036,27,2],[3036,28,1],
[3036,33,1],[3036,35,1],[3037,5,2],[3037,22,4],[3037,27,5],
[3037,43,1],[3038,26,1],[3038,46,1],[3039,3,1],[3039,6,1],[3039,13,1],
[3039,17,1],[3039,20,12],[3039,21,2],[3039,22,2],[3039,23,2],
[3039,24,1],[3039,25,1],[3039,27,20],[3039,32,2],[3039,33,1],
[3039,34,1],[3039,35,12],[3039,42,1],[3039,43,1],[3039,48,1],
[3039,68,2],[3039,72,3],[3040,2,3],[3040,3,1],[3040,23,1],[3040,24,3],

[3040,26,2],[3040,27,1],[3040,43,1],[3040,50,1],[3041,5,1],[3041,9,2],
[3041,12,1],[3041,13,2],[3041,15,59],[3041,16,1],[3041,19,1],
[3041,20,1],[3041,24,1],[3041,36,1],[3041,39,1],[3041,41,1],
[3041,46,2],[3041,53,4],[3041,55,37],[3041,57,1],[3041,61,1],
[3041,63,1],[3041,65,2],[3041,67,2],[3041,71,2],[3041,77,2],
[3042,28,1],[3042,32,1],[3042,42,1],[3043,28,1],[3043,33,1],
[3043,48,1],[3043,70,2],[3044,26,1],[3044,27,1],[3044,31,1],
[3044,33,3],[3045,19,1],[3045,41,1],[3046,68,1],[3046,70,1],
[3047,63,2],[3047,74,1],[3047,76,3],[3048,2,2],[3048,23,1],
[3048,28,7],[3048,50,1],[3048,62,163],[3048,68,1],[3048,70,1],
[3048,74,1],[3049,6,1],[3049,28,1],[3049,44,1],[3049,68,1],
[3049,72,1],[3049,74,2],[3050,2,15],[3050,3,13],[3050,6,4],
[3050,21,2],[3050,22,2],[3050,23,17],[3050,24,18],[3050,26,5],
[3050,27,12],[3050,28,12],[3050,31,17],[3050,32,4],[3050,33,1],
[3050,38,4],[3050,42,12],[3050,43,2],[3050,47,6],[3050,56,2],
[3050,60,1],[3050,70,1],[3050,78,1],[3051,4,1],[3051,10,1],
[3051,15,8],[3051,39,1],[3051,55,1],[3052,12,1],[3052,29,1],
[3052,55,1],[3053,24,1],[3053,27,1],[3054,5,1],[3054,20,1],
[3054,31,1],[3055,2,2],[3055,22,1],[3056,2,2],[3057,23,1],[3057,24,1],
[3057,27,1],[3057,28,1],[3057,42,3],[3058,21,2],[3059,23,1],
[3059,24,1],[3059,47,1],[3060,0,1],[3060,12,1],[3060,16,1],
[3060,18,1],[3060,33,1],[3060,34,1],[3060,55,1],[3060,58,1],
[3060,59,2],[3060,62,1],[3060,65,1],[3060,79,1],[3061,53,2],
[3062,2,1],[3062,78,1],[3063,22,2],[3063,23,4],[3063,25,3],
[3063,32,1],[3063,35,3],[3063,48,1],[3063,62,1],[3064,28,2],
[3065,0,1],[3065,2,2],[3065,3,31],[3065,4,1],[3065,5,1],[3065,23,2],
[3065,24,1],[3065,26,1],[3065,27,1],[3065,28,4],[3065,31,1],
[3065,40,1],[3065,41,1],[3065,51,1],[3065,62,1],[3065,68,1],
[3066,2,3],[3067,26,2],[3068,6,1],[3068,22,9],[3068,60,1],[3068,68,1],
[3068,70,1],[3069,28,1],[3069,37,1],[3070,23,2],[3070,26,1],
[3071,11,1],[3071,14,1],[3071,18,1],[3071,19,2],[3071,20,1],
[3071,34,1],[3071,37,2],[3071,41,1],[3071,45,1],[3071,55,1],
[3071,58,1],[3071,65,1],[3072,23,1],[3072,27,2],[3072,28,1],
[3072,35,2],[3073,0,1],[3073,1,6],[3073,3,1],[3073,7,1],[3073,9,1],
[3073,10,1],[3073,11,3],[3073,12,9],[3073,13,1],[3073,15,1],
[3073,16,1],[3073,17,2],[3073,19,2],[3073,22,55],[3073,28,3],
[3073,29,2],[3073,30,5],[3073,31,1],[3073,34,1],[3073,36,1],
[3073,38,1],[3073,40,1],[3073,42,1],[3073,43,1],[3073,45,1],
[3073,47,1],[3073,49,1],[3073,53,2],[3073,54,2],[3073,55,1],
[3073,65,2],[3073,68,1],[3073,75,1],[3073,77,1],[3074,2,17],
[3074,50,1],[3075,4,1],[3075,5,1],[3075,9,1],[3075,12,1],[3075,15,13],
[3075,17,12],[3075,19,1],[3075,32,1],[3075,50,1],[3075,53,14],
[3075,55,8],[3075,57,1],[3075,70,1],[3075,76,1],[3076,4,1],
[3076,50,2],[3076,74,1],[3077,23,1],[3077,25,1],[3078,3,1],[3078,9,1],
[3078,10,1],[3078,13,3],[3078,20,2],[3078,23,2],[3078,32,5],
[3079,21,4],[3079,42,1],[3079,43,1],[3079,44,1],[3079,66,1],
[3079,74,1],[3080,23,1],[3080,40,1],[3080,68,1],[3080,70,1],
[3080,72,1],[3080,74,1],[3081,17,1],[3081,53,3],[3081,55,1],
[3082,24,1],[3082,33,1],[3083,2,4],[3083,31,1],[3084,22,1],
[3084,26,1],[3085,17,1],[3085,53,1],[3086,1,1],[3086,3,1],[3086,5,1],

[3086,22,1],[3086,32,4],[3086,43,1],[3086,47,1],[3086,62,3],
[3086,66,1],[3087,21,1],[3087,40,1],[3087,68,1],[3087,70,2],
[3088,30,1],[3088,39,1],[3089,28,2],[3090,23,1],[3090,28,2],
[3090,32,1],[3091,27,2],[3092,26,1],[3092,28,2],[3093,69,1],
[3093,75,1],[3094,4,1],[3094,32,1],[3094,50,1],[3094,62,1],[3095,9,1],
[3095,22,1],[3095,59,1],[3096,23,1],[3096,28,1],[3097,2,2],[3098,2,1],
[3098,3,2],[3098,20,2],[3098,21,1],[3098,24,1],[3098,28,1],
[3098,30,1],[3098,69,1],[3098,70,1],[3099,28,1],[3099,40,1],
[3099,55,1],[3100,13,2],[3101,21,1],[3101,43,1],[3102,15,1],
[3102,20,2],[3102,22,1],[3102,24,1],[3102,35,1],[3102,38,2],
[3102,42,1],[3102,43,1],[3102,52,1],[3102,66,1],[3102,72,1],
[3103,21,1],[3103,23,1],[3104,2,2],[3104,3,2],[3104,6,1],[3104,12,10],
[3104,13,1],[3104,21,1],[3104,23,1],[3104,24,2],[3104,35,1],
[3104,42,1],[3104,43,1],[3104,46,1],[3104,47,3],[3104,60,1],
[3104,69,1],[3105,22,2],[3105,42,1],[3105,56,1],[3106,28,1],
[3106,31,1],[3106,47,1],[3107,1,1],[3107,16,1],[3108,23,1],
[3108,26,1],[3108,27,6],[3108,31,1],[3108,35,1],[3109,23,1],
[3109,24,1],[3109,47,1],[3110,27,1],[3110,35,1],[3111,39,1],
[3111,65,1],[3112,28,1],[3112,43,1],[3113,28,1],[3113,53,1],
[3113,55,1],[3114,25,1],[3114,42,1],[3115,23,3],[3115,26,1],
[3115,27,1],[3115,28,1],[3115,32,2],[3115,52,1],[3116,2,1],[3116,3,1],
[3116,21,1],[3116,28,1],[3116,32,4],[3116,43,1],[3116,50,1],
[3117,19,1],[3117,21,1],[3117,25,3],[3117,28,3],[3117,30,2],
[3117,35,2],[3117,40,1],[3117,52,4],[3117,70,1],[3117,75,1],
[3118,2,4],[3118,35,1],[3119,5,4],[3119,9,1],[3119,23,1],[3119,24,1],
[3119,26,1],[3119,27,7],[3119,28,1],[3119,29,1],[3119,31,1],
[3119,33,1],[3119,35,13],[3119,38,3],[3119,39,1],[3119,40,1],
[3119,43,12],[3119,52,1],[3119,61,1],[3119,65,1],[3119,77,1],
[3120,53,3],[3120,55,2],[3121,24,11],[3121,42,1],[3121,62,1],
[3121,76,1],[3122,20,3],[3122,35,1],[3122,56,1],[3122,65,1],
[3122,66,1],[3122,74,1],[3123,2,1],[3123,31,1],[3123,51,1],
[3123,63,1],[3124,22,2],[3124,43,1],[3124,62,5],[3125,1,1],[3125,2,5],
[3125,28,2],[3125,33,1],[3126,23,2],[3126,47,1],[3127,3,1],
[3127,32,2],[3128,56,2],[3129,15,2],[3130,28,2],[3131,28,1],
[3131,31,2],[3132,9,3],[3132,28,4],[3132,31,1],[3133,27,2],
[3133,40,1],[3133,42,1],[3134,4,1],[3134,28,1],[3135,1,1],[3135,7,2],
[3135,8,1],[3135,9,3],[3135,10,4],[3135,11,2],[3135,13,1],[3135,14,1],
[3135,16,2],[3135,18,4],[3135,19,7],[3135,20,1],[3135,36,3],
[3135,37,1],[3135,41,3],[3135,45,4],[3135,49,1],[3135,53,1],
[3135,54,1],[3135,59,1],[3135,63,1],[3135,64,1],[3135,65,2],
[3135,69,1],[3135,71,3],[3135,73,1],[3135,74,1],[3135,76,2],
[3135,77,5],[3136,9,1],[3136,20,1],[3136,28,13],[3136,38,1],
[3136,70,1],[3137,44,2],[3137,66,2],[3138,3,1],[3138,14,1],
[3138,19,1],[3138,24,1],[3138,33,1],[3138,34,2],[3138,41,1],
[3138,46,1],[3138,55,1],[3138,59,2],[3138,64,1],[3138,77,1],
[3139,0,2],[3139,10,1],[3139,23,1],[3139,46,1],[3140,38,1],
[3140,44,3],[3140,66,5],[3141,13,1],[3141,23,1],[3142,32,2],
[3143,2,4],[3143,28,1],[3144,9,1],[3144,42,1],[3145,26,1],[3145,77,1],
[3146,53,1],[3146,55,1],[3147,8,1],[3147,13,1],[3148,17,1],
[3148,50,1],[3148,53,5],[3148,55,1],[3149,2,2],[3149,50,1],

[3150,21,1],[3150,27,1],[3150,42,1],[3151,1,1],[3151,2,3],[3151,3,1],
[3151,4,1],[3151,5,32],[3151,7,1],[3151,10,3],[3151,12,2],[3151,13,3],
[3151,15,45],[3151,17,2],[3151,19,1],[3151,20,2],[3151,21,19],
[3151,22,1],[3151,23,2],[3151,24,4],[3151,25,3],[3151,26,2],
[3151,27,5],[3151,28,25],[3151,30,2],[3151,31,7],[3151,32,1],
[3151,33,7],[3151,35,3],[3151,37,1],[3151,38,3],[3151,39,2],
[3151,41,1],[3151,43,3],[3151,44,2],[3151,47,1],[3151,50,1],
[3151,51,1],[3151,52,3],[3151,55,32],[3151,56,6],[3151,58,1],
[3151,59,3],[3151,60,1],[3151,62,1],[3151,63,4],[3151,65,3],
[3151,66,8],[3151,68,1],[3151,69,6],[3151,70,9],[3151,71,1],
[3151,72,3],[3151,74,2],[3151,75,2],[3151,76,7],[3152,3,1],
[3152,28,1],[3152,50,1],[3152,53,1],[3153,23,2],[3153,24,2],
[3153,26,2],[3153,27,7],[3153,32,1],[3153,42,2],[3153,43,2],
[3154,24,1],[3154,28,1],[3154,50,1],[3154,63,1],[3155,1,2],[3155,8,1],
[3155,10,1],[3155,11,1],[3155,12,1],[3155,13,1],[3155,14,1],
[3155,16,1],[3155,17,1],[3155,20,1],[3155,29,2],[3155,41,1],
[3155,45,1],[3155,48,1],[3155,57,2],[3155,67,1],[3155,73,1],
[3155,77,1],[3156,2,1],[3156,3,2],[3156,4,1],[3156,5,1],[3156,6,4],
[3156,20,2],[3156,21,3],[3156,22,3],[3156,23,6],[3156,24,2],
[3156,26,1],[3156,27,13],[3156,28,2],[3156,32,3],[3156,33,6],
[3156,35,4],[3156,38,1],[3156,42,2],[3156,43,1],[3156,47,5],
[3156,48,1],[3157,1,1],[3157,3,4],[3157,9,1],[3157,20,3],[3157,22,1],
[3157,23,2],[3157,24,2],[3157,26,5],[3157,27,5],[3157,28,4],
[3157,32,4],[3157,33,1],[3157,35,1],[3157,38,1],[3157,39,1],
[3157,43,5],[3158,10,1],[3158,27,1],[3159,1,3],[3159,2,1],[3159,4,2],
[3159,6,2],[3159,17,1],[3159,19,1],[3159,20,1],[3159,22,2],
[3159,26,1],[3159,27,3],[3159,28,2],[3159,33,1],[3159,39,1],
[3159,50,1],[3159,56,1],[3159,66,1],[3159,68,1],[3159,69,2],
[3159,74,3],[3160,62,3],[3161,23,1],[3161,28,1],[3161,33,1],
[3161,44,1],[3161,74,13],[3162,28,1],[3162,41,1],[3163,23,1],
[3163,26,2],[3163,28,1],[3163,31,4],[3163,33,19],[3163,40,1],
[3164,0,1],[3164,1,1],[3164,2,1],[3164,3,1],[3164,8,1],[3164,10,3],
[3164,19,1],[3164,29,2],[3164,31,1],[3164,32,1],[3164,37,1],
[3164,39,1],[3164,44,8],[3164,50,2],[3164,53,1],[3164,60,1],
[3164,66,3],[3164,71,2],[3164,76,1],[3165,2,1],[3165,3,1],[3165,7,1],
[3165,20,1],[3165,25,2],[3165,29,1],[3165,38,1],[3165,66,1],
[3165,73,1],[3166,28,29],[3166,44,1],[3166,66,1],[3167,32,5],
[3168,2,4],[3168,9,1],[3168,20,1],[3168,30,2],[3168,34,5],[3168,37,1],
[3169,21,1],[3169,28,2],[3169,66,1],[3170,12,1],[3170,16,1],
[3170,34,1],[3170,45,1],[3171,2,1],[3171,5,1],[3171,6,2],[3171,23,1],
[3171,27,2],[3171,28,1],[3171,47,1],[3171,56,1],[3172,2,2],[3172,3,2],
[3172,4,2],[3172,5,16],[3172,6,6],[3172,10,1],[3172,20,4],
[3172,21,55],[3172,22,13],[3172,23,11],[3172,24,22],[3172,25,72],
[3172,26,44],[3172,27,23],[3172,28,39],[3172,30,1],[3172,31,86],
[3172,32,17],[3172,33,68],[3172,35,23],[3172,38,1],[3172,39,8],
[3172,40,1],[3172,41,1],[3172,42,2],[3172,43,52],[3172,44,2],
[3172,45,1],[3172,47,2],[3172,48,4],[3172,50,1],[3172,52,1],
[3172,56,9],[3172,59,2],[3172,68,1],[3172,70,2],[3172,76,1],
[3172,78,2],[3173,3,1],[3173,23,2],[3173,27,4],[3173,28,1],
[3174,27,2],[3175,2,2],[3175,3,1],[3175,4,2],[3175,22,1],[3175,26,1],

[3175,28,1],[3175,33,1],[3175,44,1],[3176,21,1],[3176,70,1],
[3177,26,1],[3177,32,1],[3178,45,2],[3178,47,2],[3178,55,1],
[3179,5,3],[3179,28,1],[3180,20,1],[3180,25,3],[3180,30,1],
[3180,31,11],[3180,35,1],[3180,43,3],[3180,48,1],[3180,50,1],
[3180,51,1],[3180,63,1],[3180,66,1],[3180,78,1],[3181,6,1],
[3181,24,1],[3181,28,3],[3181,62,1],[3181,70,1],[3182,1,1],[3182,2,1],
[3182,4,2],[3182,7,1],[3182,15,2],[3182,16,1],[3182,17,16],
[3182,20,1],[3182,50,1],[3182,53,20],[3182,55,13],[3182,59,1],
[3183,13,1],[3183,24,1],[3184,28,9],[3184,75,1],[3185,3,2],[3186,3,2],
[3186,13,1],[3186,23,1],[3186,26,2],[3186,27,1],[3186,28,3],
[3186,32,1],[3187,2,9],[3187,28,1],[3188,31,1],[3188,42,1],
[3189,32,1],[3189,35,1],[3189,44,1],[3190,23,78],[3190,26,2],
[3190,28,2],[3191,1,2],[3191,3,1],[3191,5,1],[3191,6,2],[3191,13,1],
[3191,21,1],[3191,22,1],[3191,23,2],[3191,24,42],[3191,25,1],
[3191,26,1],[3191,28,5],[3191,29,1],[3191,35,3],[3191,39,10],
[3191,42,1],[3191,62,1],[3191,63,1],[3191,70,1],[3191,74,1],
[3191,76,1],[3192,27,2],[3193,27,2],[3193,28,1],[3193,70,1],
[3194,2,1],[3194,32,1],[3194,40,1],[3194,75,6],[3195,3,1],[3195,63,3],
[3195,74,4],[3195,75,1],[3195,76,5],[3196,15,3],[3196,17,2],
[3196,53,2],[3196,55,4],[3197,1,1],[3197,3,1],[3197,13,1],[3197,20,1],
[3197,27,1],[3197,28,1],[3197,33,1],[3197,45,1],[3197,75,2],
[3198,5,3],[3198,15,1],[3198,21,1],[3198,28,2],[3199,26,1],
[3199,33,28],[3199,38,1],[3199,77,1],[3200,1,1],[3200,28,1],
[3201,63,1],[3201,75,2],[3201,76,2],[3202,24,2],[3203,52,1],
[3203,62,1],[3204,13,1],[3204,20,1],[3204,22,1],[3204,25,1],
[3204,39,1],[3205,10,1],[3205,16,1],[3205,19,1],[3206,5,1],
[3206,22,1],[3206,27,1],[3206,28,1],[3206,42,2],[3207,31,3],
[3207,35,5],[3208,51,1],[3208,56,2],[3209,2,1],[3209,24,1],
[3210,10,1],[3210,21,1],[3211,19,1],[3211,73,1],[3212,20,1],
[3212,46,1],[3213,40,3],[3213,63,1],[3214,22,1],[3214,23,2],
[3214,24,1],[3214,25,1],[3214,35,1],[3214,50,1],[3214,62,3],
[3214,70,1],[3215,26,1],[3215,28,1],[3216,2,4],[3217,23,4],
[3218,24,1],[3218,28,1],[3219,23,2],[3220,1,14],[3220,12,1],
[3220,13,1],[3220,17,2],[3220,36,1],[3220,39,1],[3221,11,1],
[3221,24,1],[3221,27,1],[3221,28,1],[3221,70,1],[3222,63,1],
[3222,76,2],[3223,21,1],[3223,42,4],[3224,8,1],[3224,73,1],[3225,5,1],
[3225,28,1],[3226,3,1],[3226,6,1],[3226,22,1],[3226,23,4],[3226,24,2],
[3226,26,5],[3226,30,1],[3226,32,2],[3226,35,1],[3226,43,10],
[3226,48,1],[3226,52,1],[3227,2,5],[3228,6,1],[3228,9,2],[3228,14,2],
[3228,21,1],[3228,23,1],[3228,37,1],[3228,41,1],[3228,43,1],
[3228,70,1],[3228,77,1],[3229,23,4],[3229,24,6],[3229,28,7],
[3229,42,1],[3230,16,1],[3230,57,1],[3230,77,1],[3231,3,2],
[3231,22,1],[3231,28,1],[3231,32,1],[3231,35,1],[3232,28,4],
[3233,4,1],[3233,32,14],[3233,38,1],[3233,62,1],[3234,20,2],
[3235,24,2],[3235,40,1],[3236,22,1],[3236,24,2],[3236,31,1],
[3237,2,2],[3238,0,14],[3238,9,1],[3238,19,1],[3238,37,1],[3238,41,2],
[3238,54,2],[3238,58,1],[3238,71,1],[3239,15,2],[3239,28,3],
[3239,33,1],[3239,40,1],[3239,50,1],[3239,68,1],[3239,69,1],
[3239,74,1],[3239,76,1],[3240,21,1],[3240,62,1],[3241,2,3],[3241,3,1],
[3241,4,1],[3241,13,2],[3241,17,1],[3241,20,1],[3241,21,46],

[3241,22,4],[3241,23,46],[3241,24,4],[3241,25,33],[3241,26,77],
[3241,27,1],[3241,28,9],[3241,31,56],[3241,33,76],[3241,35,2],
[3241,38,1],[3241,42,2],[3241,43,50],[3241,44,8],[3241,50,2],
[3241,52,3],[3241,56,1],[3241,69,1],[3241,72,1],[3241,74,1],
[3241,75,2],[3241,76,1],[3242,12,4],[3243,20,1],[3243,31,1],
[3244,5,2],[3244,6,1],[3244,22,1],[3244,23,2],[3244,24,1],
[3244,27,17],[3244,28,1],[3244,33,7],[3244,35,5],[3244,46,1],
[3245,5,1],[3245,20,1],[3245,22,1],[3245,27,1],[3245,28,1],
[3245,44,1],[3245,65,1],[3246,28,1],[3246,53,1],[3247,12,1],
[3247,20,4],[3247,23,10],[3247,42,8],[3248,35,1],[3248,50,1],
[3248,55,1],[3248,66,1],[3249,4,1],[3249,28,1],[3249,38,1],
[3249,70,1],[3250,2,1],[3250,26,1],[3250,39,2],[3250,42,1],
[3251,45,4],[3252,2,1],[3252,5,1],[3253,1,1],[3253,2,1],[3253,8,1],
[3253,29,1],[3253,39,19],[3254,2,1],[3254,9,1],[3254,12,2],
[3254,24,1],[3254,26,1],[3254,31,2],[3254,42,18],[3254,43,2],
[3254,47,1],[3254,48,3],[3254,66,1],[3254,70,1],[3254,76,1],
[3255,21,1],[3255,29,1],[3255,43,2],[3256,9,2],[3256,28,2],
[3256,55,2],[3257,15,2],[3258,22,2],[3258,24,1],[3258,26,1],
[3258,28,2],[3258,47,1],[3258,68,1],[3259,23,6],[3259,24,6],
[3259,26,1],[3259,28,6],[3259,32,1],[3259,33,4],[3259,75,1],
[3260,13,1],[3260,31,1],[3260,32,2],[3261,31,37],[3262,28,1],
[3262,50,1],[3263,1,1],[3263,26,1],[3263,62,1],[3264,24,1],
[3264,42,1],[3265,22,4],[3265,26,1],[3265,27,2],[3265,35,3],
[3266,19,1],[3266,45,1],[3266,53,1],[3266,55,2],[3267,31,1],
[3267,32,2],[3267,42,1],[3267,43,1],[3267,60,1],[3268,23,1],
[3268,27,2],[3268,28,3],[3269,8,1],[3269,10,1],[3269,13,1],
[3269,15,1],[3269,28,1],[3269,55,1],[3269,65,1],[3269,70,1],
[3270,17,1],[3270,53,1],[3271,2,2],[3271,15,1],[3271,18,5],
[3271,27,1],[3271,62,2],[3272,34,1],[3272,45,1],[3273,10,1],
[3273,20,1],[3273,33,1],[3274,21,1],[3274,22,1],[3274,27,3],
[3274,66,1],[3274,72,1],[3274,74,4],[3275,1,1],[3275,2,1],[3275,5,2],
[3275,7,1],[3275,15,1],[3275,23,1],[3275,24,2],[3275,25,24],
[3275,26,1],[3275,27,2],[3275,28,13],[3275,30,2],[3275,32,37],
[3275,33,56],[3275,35,103],[3275,38,4],[3275,52,69],[3275,66,1],
[3276,34,1],[3276,46,1],[3276,59,1],[3277,28,1],[3277,72,1],
[3278,27,1],[3278,42,1],[3279,20,6],[3280,6,1],[3280,28,1],
[3280,31,1],[3280,54,1],[3281,42,3],[3282,29,1],[3282,53,2],
[3282,54,1],[3282,77,1],[3283,2,1],[3283,9,1],[3283,10,1],[3283,19,2],
[3283,22,1],[3283,23,1],[3283,25,2],[3283,28,3],[3283,30,30],
[3283,32,18],[3283,33,30],[3283,35,1],[3283,38,1],[3283,44,1],
[3283,46,1],[3283,52,1],[3283,54,1],[3283,62,1],[3283,64,1],
[3283,67,2],[3283,70,1],[3283,71,1],[3283,75,1],[3283,76,1],
[3284,26,1],[3284,28,1],[3285,28,3],[3286,3,1],[3286,29,1],
[3286,56,2],[3287,26,2],[3288,35,3],[3289,9,12],[3289,10,1],
[3289,13,1],[3289,28,3],[3289,41,1],[3289,45,1],[3289,46,1],
[3289,49,1],[3289,55,1],[3289,57,1],[3289,58,3],[3289,67,1],
[3289,74,1],[3290,3,1],[3290,22,2],[3290,27,1],[3290,43,1],
[3290,48,1],[3291,15,1],[3291,64,1],[3292,28,1],[3292,37,1],
[3292,38,1],[3293,11,1],[3293,20,1],[3293,59,1],[3294,63,1],
[3294,75,1],[3294,76,4],[3295,2,5],[3295,3,5],[3295,4,8],[3295,5,6],

[3295,6,4],[3295,10,2],[3295,21,3],[3295,22,4],[3295,23,13],
[3295,24,8],[3295,25,24],[3295,26,14],[3295,27,15],[3295,28,83],
[3295,30,1],[3295,31,11],[3295,32,17],[3295,33,5],[3295,35,7],
[3295,38,8],[3295,40,57],[3295,42,4],[3295,43,6],[3295,44,3],
[3295,47,6],[3295,48,3],[3295,50,1],[3295,52,1],[3295,56,6],
[3295,61,4],[3295,62,14],[3295,63,16],[3295,65,1],[3295,66,3],
[3295,68,28],[3295,69,22],[3295,70,183],[3295,72,43],[3295,74,31],
[3295,75,12],[3295,76,29],[3295,77,1],[3296,16,1],[3296,23,1],
[3296,28,5],[3297,15,1],[3297,55,1],[3298,21,1],[3298,30,18],
[3299,25,1],[3299,28,2],[3300,28,2],[3300,35,1],[3301,2,9],
[3301,24,3],[3302,27,2],[3303,2,1],[3303,12,1],[3303,19,1],
[3303,21,1],[3303,23,3],[3303,27,1],[3303,28,4],[3303,30,2],
[3303,31,2],[3303,32,1],[3303,37,1],[3303,42,1],[3303,43,2],
[3303,47,3],[3303,57,1],[3304,9,1],[3304,15,1],[3304,19,1],
[3304,22,1],[3304,28,1],[3304,46,1],[3305,29,2],[3306,4,1],
[3306,44,1],[3306,50,1],[3306,56,1],[3307,33,2],[3308,8,1],[3308,9,4],
[3308,10,1],[3308,11,1],[3308,18,1],[3308,19,3],[3308,20,1],
[3308,36,1],[3308,37,1],[3308,39,1],[3308,45,3],[3308,49,1],
[3308,60,1],[3308,65,2],[3308,67,1],[3308,71,1],[3308,73,1],
[3308,77,1],[3309,2,2],[3310,28,1],[3310,35,1],[3311,23,2],
[3311,27,5],[3311,28,1],[3311,32,5],[3311,35,3],[3312,3,1],
[3312,23,8],[3312,78,2],[3313,20,3],[3313,29,1],[3314,39,2],
[3315,44,1],[3315,66,1],[3316,1,2],[3316,2,1],[3316,24,1],[3317,3,1],
[3317,5,2],[3317,9,1],[3317,11,1],[3317,17,1],[3317,18,1],[3317,24,1],
[3317,31,1],[3317,42,1],[3317,66,1],[3318,3,1],[3318,43,2],
[3318,48,2],[3318,61,1],[3319,32,10],[3320,22,2],[3321,30,2],
[3322,4,2],[3322,5,1],[3322,31,5],[3322,35,1],[3322,44,4],[3322,48,1],
[3322,66,1],[3323,5,1],[3323,21,6],[3323,24,1],[3323,27,2],
[3323,32,3],[3323,42,1],[3323,43,3],[3323,56,2],[3324,4,1],
[3324,38,1],[3324,44,1],[3324,66,1],[3325,70,1],[3325,75,1],
[3326,24,1],[3326,31,1],[3327,0,1],[3327,39,1],[3328,5,2],[3328,21,1],
[3328,52,1],[3329,23,12],[3329,28,1],[3330,2,1],[3330,31,2],
[3331,27,3],[3332,32,13],[3333,21,3],[3334,1,1],[3334,9,3],
[3334,15,1],[3334,22,1],[3335,2,2],[3336,22,1],[3336,38,1],
[3336,42,1],[3337,2,3],[3337,8,1],[3337,9,1],[3337,12,14],[3337,20,1],
[3337,21,3],[3337,31,1],[3337,45,1],[3337,49,1],[3338,22,1],
[3338,23,1],[3339,28,6],[3339,44,5],[3339,50,1],[3339,66,2],
[3340,29,3],[3341,5,1],[3341,44,1],[3341,68,1],[3341,70,1],
[3342,28,2],[3342,47,1],[3343,30,2],[3344,0,63],[3344,1,115],
[3344,2,144],[3344,7,30],[3344,8,37],[3344,9,28],[3344,10,16],
[3344,11,8],[3344,12,1335],[3344,13,70],[3344,14,2],[3344,15,20],
[3344,16,8],[3344,17,29],[3344,18,4],[3344,19,25],[3344,20,25],
[3344,22,1],[3344,23,1],[3344,25,3],[3344,26,1],[3344,27,1],
[3344,28,10],[3344,29,2661],[3344,30,77],[3344,31,472],[3344,32,2],
[3344,34,15],[3344,36,9],[3344,37,16],[3344,38,2],[3344,39,39],
[3344,41,4],[3344,42,2],[3344,45,26],[3344,46,3],[3344,49,4],
[3344,50,3],[3344,53,24],[3344,54,4],[3344,55,19],[3344,57,8],
[3344,58,10],[3344,59,8],[3344,60,4],[3344,63,2],[3344,65,16],
[3344,66,1],[3344,67,2],[3344,70,1],[3344,71,6],[3344,72,1],
[3344,73,14],[3344,77,9],[3344,78,2],[3345,40,1],[3345,56,1],

[3346,0,1],[3346,8,1],[3346,15,1],[3346,17,3],[3346,53,3],[3346,55,2],
[3346,59,1],[3346,65,1],[3347,5,1],[3347,11,1],[3347,20,1],
[3347,24,1],[3347,31,1],[3347,32,1],[3347,42,30],[3347,58,1],
[3347,66,1],[3347,68,1],[3347,69,1],[3347,70,2],[3347,74,1],
[3348,8,1],[3348,10,1],[3348,15,1],[3348,16,1],[3348,37,1],
[3349,52,6],[3350,5,4],[3350,21,2],[3350,23,1],[3350,24,1],
[3350,25,2],[3350,26,1],[3350,43,1],[3351,28,1],[3351,38,1],
[3352,31,3],[3353,23,1],[3353,27,2],[3354,2,2],[3355,33,1],
[3355,43,1],[3355,47,1],[3356,1,3],[3356,2,5],[3356,5,1],[3356,10,1],
[3356,19,1],[3356,20,1],[3356,22,2],[3356,24,1],[3356,26,2],
[3356,27,1],[3356,31,1],[3356,42,1],[3356,46,1],[3356,55,1],
[3356,56,1],[3356,59,2],[3356,73,2],[3356,74,1],[3356,76,1],
[3357,41,1],[3357,45,1],[3358,21,1],[3358,24,1],[3359,3,1],[3359,5,1],
[3359,22,1],[3359,32,1],[3360,1,1],[3360,12,1],[3361,13,1],
[3361,20,3],[3361,27,1],[3361,39,1],[3362,2,1],[3362,28,1],
[3362,44,1],[3362,66,1],[3362,74,1],[3363,56,2],[3364,31,2],
[3365,4,2],[3365,11,1],[3365,15,2],[3365,17,2],[3365,18,1],
[3365,19,1],[3365,24,1],[3365,38,1],[3365,45,1],[3365,55,2],
[3365,56,1],[3365,57,1],[3365,66,1],[3366,21,1],[3366,28,4],
[3367,26,1],[3367,31,1],[3368,25,3],[3368,32,1],[3369,5,2],[3369,6,1],
[3369,22,3],[3369,23,1],[3369,28,2],[3369,33,1],[3369,70,1],
[3370,25,1],[3370,28,1],[3370,38,1],[3370,66,1],[3370,70,1],
[3370,75,1],[3371,25,1],[3371,43,1],[3371,52,1],[3372,25,2],
[3373,1,4],[3373,3,1],[3373,5,1],[3373,8,2],[3373,13,3],[3373,14,1],
[3373,15,3],[3373,19,1],[3373,20,2],[3373,25,1],[3373,28,1],
[3373,35,1],[3373,40,1],[3373,45,2],[3373,46,1],[3373,53,4],
[3373,54,1],[3373,57,2],[3373,63,1],[3373,66,1],[3373,76,1],
[3374,38,1],[3374,66,1],[3375,15,1],[3375,55,3],[3376,5,1],
[3376,24,4],[3376,28,1],[3376,31,1],[3376,33,1],[3376,47,32],
[3376,48,3],[3376,50,1],[3376,51,20],[3376,56,3],[3376,74,5],
[3376,75,1],[3377,2,1],[3377,4,1],[3377,5,1],[3377,20,7],[3377,24,3],
[3377,26,1],[3377,28,9],[3377,47,1],[3377,49,1],[3377,53,1],
[3377,56,2],[3377,66,1],[3378,0,6],[3379,5,3],[3379,6,1],[3379,8,1],
[3379,12,1],[3379,21,2],[3379,23,1],[3379,25,1],[3379,28,1],
[3379,32,1],[3379,42,1],[3379,51,1],[3379,56,1],[3379,66,1],
[3379,69,2],[3379,70,3],[3379,74,1],[3380,25,3],[3380,27,2],
[3380,28,2],[3380,31,3],[3380,32,2],[3380,42,1],[3380,68,1],
[3381,30,2],[3382,9,2],[3382,12,1],[3382,13,1],[3382,39,1],
[3382,57,1],[3383,10,1],[3383,20,1],[3384,27,1],[3384,76,1],
[3385,15,1],[3385,53,1],[3386,28,26],[3386,74,1],[3387,2,11],
[3387,25,1],[3388,4,1],[3388,40,1],[3389,0,1],[3389,13,1],[3389,17,1],
[3390,27,1],[3390,28,1],[3391,27,4],[3391,62,1],[3392,5,1],
[3392,27,2],[3392,43,1],[3392,62,1],[3393,23,1],[3393,28,1],
[3394,2,1],[3394,22,1],[3394,30,1],[3394,61,1],[3394,68,1],[3395,2,4],
[3395,3,4],[3395,5,2],[3395,23,3],[3395,24,9],[3395,26,1],[3395,27,2],
[3395,28,5],[3395,31,4],[3395,32,2],[3395,43,1],[3396,28,1],
[3396,48,1],[3396,56,2],[3397,4,3],[3397,5,1],[3397,22,1],[3397,23,2],
[3397,24,1],[3397,26,1],[3397,28,2],[3397,32,1],[3397,35,1],
[3397,42,1],[3397,50,1],[3397,76,5],[3398,11,1],[3398,44,1],
[3399,0,2],[3399,8,3],[3399,34,3],[3399,36,1],[3400,23,18],

[3400,24,1],[3401,50,1],[3401,56,2],[3401,61,1],[3401,69,1],
[3402,4,1],[3402,17,1],[3402,25,1],[3402,28,44],[3402,38,1],
[3402,47,1],[3402,50,1],[3402,53,1],[3402,70,1],[3403,24,4],
[3403,25,2],[3403,28,3],[3403,43,1],[3403,70,1],[3404,3,1],
[3404,27,1],[3405,46,1],[3405,71,1],[3406,3,1],[3406,31,1],
[3406,35,3],[3407,27,2],[3408,23,2],[3408,28,2],[3409,27,1],
[3409,30,1],[3409,62,1],[3410,28,1],[3410,48,2],[3411,27,1],
[3411,31,2],[3411,32,1],[3411,35,1],[3411,62,1],[3412,28,5],
[3413,5,3],[3413,22,2],[3413,24,2],[3413,26,4],[3413,27,2],
[3413,53,1],[3414,2,1],[3414,3,2],[3414,6,1],[3414,22,2],[3414,23,1],
[3414,24,1],[3414,25,1],[3414,27,1],[3414,30,3],[3414,33,1],
[3414,35,1],[3414,56,1],[3414,75,1],[3415,20,1],[3415,27,1],
[3415,66,1],[3416,27,1],[3416,38,1],[3417,26,2],[3417,31,2],
[3418,25,2],[3418,28,1],[3418,32,9],[3418,42,1],[3418,43,2],
[3418,49,1],[3418,59,1],[3418,64,1],[3419,2,1],[3419,16,1],
[3419,21,4],[3419,23,3],[3419,26,40],[3419,31,2],[3419,33,1],
[3419,37,1],[3419,42,6],[3419,66,1],[3419,68,1],[3419,69,1],
[3419,70,1],[3419,74,1],[3420,3,3],[3420,26,1],[3420,43,1],
[3420,48,2],[3420,51,1],[3420,56,5],[3420,61,1],[3421,10,1],
[3421,15,3],[3421,66,1],[3422,5,1],[3422,26,2],[3422,31,1],
[3422,42,2],[3423,21,1],[3423,26,1],[3424,24,2],[3424,42,2],
[3425,3,1],[3425,13,1],[3425,29,1],[3425,47,1],[3426,56,2],
[3427,31,1],[3427,33,2],[3428,1,2],[3428,3,2],[3428,15,1],[3428,19,1],
[3428,20,1],[3428,22,1],[3428,23,9],[3428,24,5],[3428,25,1],
[3428,26,5],[3428,27,3],[3428,28,15],[3428,31,2],[3428,32,2],
[3428,35,2],[3428,38,1],[3428,39,1],[3429,24,1],[3429,34,1],
[3429,35,1],[3430,3,4],[3430,5,1],[3430,17,1],[3430,24,1],[3430,25,1],
[3430,32,2],[3430,33,1],[3430,35,3],[3430,38,1],[3430,42,1],
[3430,50,1],[3430,62,1],[3431,21,2],[3431,26,5],[3431,28,1],
[3431,31,7],[3431,43,1],[3432,27,4],[3432,28,2],[3432,35,1],
[3432,66,1],[3433,20,5],[3434,2,1],[3434,18,1],[3434,21,7],
[3434,22,1],[3434,23,1],[3434,25,15],[3434,26,4],[3434,27,19],
[3434,31,2],[3434,32,1],[3434,33,1],[3434,34,1],[3434,35,8],
[3434,42,2],[3434,43,13],[3434,47,1],[3434,56,1],[3434,62,2],
[3434,68,1],[3434,70,1],[3434,72,1],[3435,3,1],[3435,23,1],
[3435,25,4],[3436,5,1],[3436,28,1],[3437,2,1],[3437,3,2],[3437,20,3],
[3437,21,6],[3437,22,8],[3437,23,6],[3437,25,2],[3437,26,3],
[3437,27,5],[3437,28,2],[3437,29,1],[3437,31,1],[3437,34,1],
[3437,35,3],[3437,38,1],[3437,42,2],[3437,43,3],[3437,59,1],
[3437,75,1],[3438,27,3],[3439,51,2],[3440,20,1],[3440,28,1],
[3440,77,1],[3441,1,1],[3441,2,1],[3441,8,1],[3441,12,1],[3441,31,5],
[3442,2,2],[3442,23,2],[3442,25,2],[3442,28,13],[3442,35,1],
[3442,40,3],[3442,42,1],[3442,47,1],[3442,50,4],[3442,68,1],
[3442,76,1],[3443,3,1],[3443,6,1],[3443,21,1],[3443,22,1],[3443,28,1],
[3443,33,1],[3443,38,1],[3443,52,1],[3444,22,1],[3444,23,3],
[3444,27,1],[3444,33,1],[3444,35,2],[3444,39,1],[3445,9,1],
[3445,53,1],[3446,5,1],[3446,15,4],[3446,22,1],[3446,24,1],
[3446,27,1],[3446,29,1],[3446,31,1],[3446,32,1],[3446,33,2],
[3446,38,3],[3446,42,1],[3446,43,1],[3446,44,1],[3446,48,2],
[3446,52,1],[3446,55,2],[3446,62,1],[3446,63,1],[3446,66,2],

[3446,70,1],[3446,74,3],[3447,1,1],[3447,19,1],[3447,39,1],
[3447,64,1],[3448,1,1],[3448,3,5],[3448,24,10],[3448,28,3],
[3448,42,3],[3448,56,2],[3449,2,1],[3449,3,4],[3449,4,1],[3449,6,1],
[3449,22,1],[3449,23,2],[3449,26,1],[3449,27,2],[3449,28,1],
[3449,35,3],[3449,37,1],[3449,42,1],[3449,43,1],[3449,47,1],
[3449,48,1],[3449,50,1],[3449,51,16],[3449,56,7],[3449,68,2],
[3449,69,9],[3449,72,1],[3449,74,1],[3449,76,1],[3449,79,3],
[3450,31,3],[3450,35,1],[3450,42,1],[3451,1,1],[3451,3,17],[3451,5,4],
[3451,6,2],[3451,10,1],[3451,14,1],[3451,15,1],[3451,20,1],
[3451,21,2],[3451,22,3],[3451,23,2],[3451,24,11],[3451,25,1],
[3451,26,5],[3451,27,4],[3451,28,12],[3451,31,2],[3451,33,18],
[3451,34,3],[3451,35,6],[3451,42,9],[3451,43,8],[3451,44,1],
[3451,46,1],[3451,47,2],[3451,48,1],[3451,49,1],[3451,62,1],
[3451,63,1],[3451,72,1],[3452,62,3],[3453,62,5],[3454,22,2],
[3454,23,1],[3455,2,1],[3455,5,1],[3455,26,1],[3455,42,1],[3456,2,2],
[3457,22,1],[3457,25,1],[3457,28,2],[3457,42,1],[3457,43,2],
[3458,20,2],[3459,3,1],[3459,50,1],[3459,70,1],[3460,10,1],
[3460,59,2],[3461,22,2],[3461,43,2],[3461,74,1],[3462,43,1],
[3462,45,1],[3462,65,1],[3463,0,5],[3463,1,10],[3463,4,4],[3463,7,2],
[3463,8,2],[3463,9,3],[3463,10,4],[3463,11,1],[3463,12,10],
[3463,13,4],[3463,15,6],[3463,16,1],[3463,17,3],[3463,18,1],
[3463,19,4],[3463,20,4],[3463,22,1],[3463,25,1],[3463,27,2],
[3463,28,3],[3463,29,9],[3463,30,6],[3463,31,1],[3463,32,1],
[3463,35,1],[3463,36,1],[3463,37,4],[3463,38,1],[3463,39,6],
[3463,41,1],[3463,42,1],[3463,44,2],[3463,45,1],[3463,46,1],
[3463,50,10],[3463,53,5],[3463,54,1],[3463,55,4],[3463,58,1],
[3463,59,2],[3463,64,1],[3463,65,4],[3463,66,2],[3463,68,1],
[3463,73,1],[3463,74,1],[3463,76,1],[3463,77,1],[3464,23,1],
[3464,24,1],[3464,51,3],[3465,2,1],[3465,27,3],[3465,46,1],
[3466,28,3],[3466,32,1],[3467,27,1],[3467,28,1],[3467,31,1],
[3468,26,1],[3468,32,1],[3469,26,1],[3469,27,3],[3469,31,3],
[3469,33,1],[3469,42,1],[3469,43,2],[3470,0,1],[3470,1,19],[3470,2,6],
[3470,3,17],[3470,4,1],[3470,5,27],[3470,6,7],[3470,7,1],[3470,8,1],
[3470,9,2],[3470,11,2],[3470,12,19],[3470,13,4],[3470,15,6],
[3470,17,1],[3470,18,1],[3470,19,1],[3470,20,6],[3470,21,11],
[3470,22,10],[3470,23,12],[3470,24,16],[3470,25,2],[3470,26,7],
[3470,27,9],[3470,28,16],[3470,30,1],[3470,31,3],[3470,32,5],
[3470,33,44],[3470,35,5],[3470,36,1],[3470,37,2],[3470,38,3],
[3470,39,2],[3470,40,1],[3470,42,6],[3470,43,2],[3470,44,3],
[3470,45,1],[3470,47,15],[3470,50,3],[3470,51,1],[3470,52,1],
[3470,55,1],[3470,56,8],[3470,58,1],[3470,59,4],[3470,62,1],
[3470,65,2],[3470,66,3],[3470,67,4],[3470,70,3],[3470,74,4],
[3470,76,1],[3470,77,1],[3470,78,1],[3471,1,2],[3471,20,2],
[3471,25,1],[3471,26,3],[3471,39,2],[3471,43,2],[3471,62,1],
[3471,70,1],[3471,71,1],[3472,28,1],[3472,42,1],[3472,62,4],
[3473,41,1],[3473,60,1],[3474,27,1],[3474,37,1],[3474,53,1],
[3474,55,1],[3475,1,1],[3475,26,1],[3475,32,1],[3475,35,1],
[3475,62,3],[3476,5,1],[3476,35,1],[3476,74,1],[3477,28,1],
[3477,42,1],[3478,31,1],[3478,42,1],[3478,43,1],[3479,2,1],[3479,8,1],
[3479,9,1],[3479,11,1],[3479,15,1],[3479,20,16],[3479,23,1],

[3479,28,2],[3479,32,2],[3479,34,2],[3479,42,2],[3479,55,2],
[3479,60,1],[3479,70,1],[3480,28,2],[3481,28,1],[3481,46,1],
[3481,60,1],[3482,31,1],[3482,46,1],[3482,59,1],[3482,74,7],
[3483,21,1],[3483,33,1],[3484,1,1],[3484,9,2],[3484,10,5],[3484,12,2],
[3484,14,2],[3484,15,1],[3484,16,1],[3484,17,2],[3484,25,3],
[3484,27,1],[3484,29,1],[3484,32,1],[3484,41,2],[3484,45,4],
[3484,46,1],[3484,53,2],[3484,55,1],[3484,57,1],[3484,59,2],
[3484,64,1],[3484,65,1],[3484,77,1],[3485,2,1],[3485,3,1],[3485,25,1],
[3485,26,1],[3485,39,1],[3486,28,2],[3487,5,2],[3487,20,1],
[3487,27,2],[3488,2,1],[3488,22,1],[3488,42,1],[3488,43,1],
[3488,47,1],[3489,3,9],[3489,6,3],[3489,26,1],[3489,35,1],[3489,38,1],
[3489,56,2],[3490,2,3],[3490,3,5],[3490,5,20],[3490,8,1],[3490,13,2],
[3490,21,33],[3490,22,55],[3490,23,35],[3490,24,32],[3490,25,16],
[3490,26,40],[3490,27,126],[3490,28,16],[3490,31,60],[3490,32,51],
[3490,33,12],[3490,35,58],[3490,39,5],[3490,41,1],[3490,42,26],
[3490,43,43],[3490,44,1],[3490,47,4],[3490,52,1],[3490,59,1],
[3490,66,1],[3490,68,3],[3490,69,1],[3490,70,3],[3490,71,1],
[3490,72,2],[3490,74,1],[3490,75,2],[3490,76,1],[3491,10,1],
[3491,23,1],[3491,56,3],[3492,4,1],[3492,25,1],[3493,2,1],[3493,5,1],
[3493,20,1],[3494,15,2],[3494,53,1],[3495,1,18],[3495,2,3],[3495,3,2],
[3495,4,3],[3495,5,1],[3495,7,2],[3495,8,2],[3495,9,13],[3495,10,22],
[3495,11,3],[3495,12,11],[3495,13,10],[3495,14,11],[3495,15,13],
[3495,16,9],[3495,17,10],[3495,18,8],[3495,19,6],[3495,20,8],
[3495,21,1],[3495,22,2],[3495,23,3],[3495,24,2],[3495,25,1],
[3495,26,2],[3495,27,3],[3495,28,1],[3495,29,2],[3495,30,1],
[3495,34,6],[3495,35,2],[3495,37,8],[3495,38,2],[3495,39,8],
[3495,40,2],[3495,41,5],[3495,42,1],[3495,43,1],[3495,44,1],
[3495,45,12],[3495,46,6],[3495,47,3],[3495,50,1],[3495,53,8],
[3495,54,5],[3495,55,9],[3495,57,6],[3495,58,10],[3495,59,17],
[3495,60,3],[3495,63,4],[3495,64,4],[3495,65,16],[3495,66,3],
[3495,67,6],[3495,69,1],[3495,70,3],[3495,71,5],[3495,72,2],
[3495,73,7],[3495,74,10],[3495,75,9],[3495,76,21],[3495,77,8],
[3495,78,2],[3496,27,2],[3497,20,1],[3497,27,1],[3498,28,1],
[3498,42,1],[3499,3,1],[3499,23,4],[3499,32,13],[3499,42,1],
[3499,48,7],[3499,56,1],[3499,62,2],[3500,40,1],[3500,70,2],
[3500,72,1],[3501,1,3],[3501,2,1],[3501,10,1],[3501,11,1],
[3501,12,17],[3501,13,1],[3501,18,1],[3501,19,1],[3501,20,1],
[3501,29,12],[3501,30,2],[3501,51,1],[3501,59,1],[3501,64,1],
[3501,68,3],[3502,33,1],[3502,35,1],[3503,3,1],[3503,9,1],[3503,23,1],
[3503,47,1],[3503,59,1],[3504,2,1],[3504,74,1],[3505,3,1],[3505,30,1],
[3506,8,1],[3506,10,1],[3506,34,1],[3506,73,1],[3507,4,1],[3507,22,1],
[3507,43,2],[3508,27,1],[3508,30,1],[3509,29,1],[3509,55,1],
[3510,28,1],[3510,50,1],[3511,30,1],[3511,36,1],[3512,53,1],
[3512,55,2],[3513,4,2],[3513,9,14],[3513,13,1],[3513,15,9],
[3513,17,24],[3513,28,11],[3513,31,1],[3513,32,1],[3513,44,2],
[3513,50,1],[3513,53,35],[3513,55,22],[3513,65,1],[3513,66,1],
[3514,26,1],[3514,43,1],[3515,21,1],[3515,22,1],[3515,26,2],
[3515,43,3],[3515,44,1],[3516,18,1],[3516,39,2],[3517,28,2],
[3518,10,1],[3518,58,1],[3519,3,1],[3519,24,7],[3520,22,1],
[3520,28,1],[3521,1,1],[3521,4,71],[3521,5,9],[3521,9,1],[3521,11,1],

[3521,12,1],[3521,13,1],[3521,14,1],[3521,15,4],[3521,16,1],
[3521,21,3],[3521,22,8],[3521,23,6],[3521,25,2],[3521,27,1],
[3521,28,20],[3521,30,1],[3521,31,1],[3521,32,1],[3521,33,1],
[3521,35,3],[3521,36,1],[3521,38,11],[3521,40,1],[3521,43,2],
[3521,44,85],[3521,45,2],[3521,46,1],[3521,47,3],[3521,48,1],
[3521,50,64],[3521,54,1],[3521,55,1],[3521,56,2],[3521,57,1],
[3521,58,1],[3521,59,1],[3521,62,1],[3521,63,1],[3521,66,82],
[3521,68,2],[3521,69,1],[3521,70,7],[3521,71,1],[3521,73,3],
[3522,17,1],[3522,23,2],[3522,26,2],[3522,28,3],[3522,32,1],
[3522,43,1],[3522,44,1],[3522,59,1],[3523,16,1],[3523,19,2],
[3523,39,1],[3523,46,1],[3523,49,1],[3524,1,1],[3524,3,3],[3524,5,1],
[3524,20,1],[3524,23,2],[3524,32,4],[3524,33,1],[3524,38,1],
[3524,47,1],[3524,74,1],[3525,4,6],[3525,5,1],[3525,15,6],[3525,16,1],
[3525,17,22],[3525,21,2],[3525,38,1],[3525,50,10],[3525,53,13],
[3525,55,18],[3525,66,3],[3526,28,2],[3527,23,17],[3527,28,1],
[3527,32,1],[3527,43,1],[3528,15,1],[3528,19,1],[3528,24,1],
[3528,25,1],[3528,27,2],[3528,31,2],[3528,32,1],[3528,43,1],
[3528,57,2],[3528,73,1],[3529,3,1],[3529,15,3],[3529,22,2],
[3529,25,1],[3529,26,2],[3529,27,2],[3529,28,26],[3529,40,4],
[3529,42,1],[3529,44,1],[3529,61,1],[3529,63,2],[3529,69,1],
[3529,70,5],[3529,72,1],[3529,74,1],[3529,76,1],[3530,0,1],
[3530,20,1],[3530,36,1],[3531,44,1],[3531,66,1],[3532,21,1],
[3532,22,1],[3532,25,3],[3532,27,2],[3532,28,1],[3532,62,4],
[3533,70,1],[3533,72,1],[3534,4,1],[3534,32,1],[3535,7,8],[3535,8,2],
[3535,13,1],[3535,17,1],[3535,20,2],[3535,34,1],[3535,36,1],
[3535,39,13],[3535,45,1],[3535,55,1],[3535,64,1],[3536,37,2],
[3537,23,1],[3537,74,2],[3537,75,2],[3537,76,2],[3538,3,1],
[3538,22,2],[3538,28,1],[3538,47,5],[3539,0,111],[3539,1,807],
[3539,2,42],[3539,3,100],[3539,4,60],[3539,5,83],[3539,6,17],
[3539,7,171],[3539,8,169],[3539,9,848],[3539,10,943],[3539,11,467],
[3539,12,452],[3539,13,465],[3539,14,313],[3539,15,642],[3539,16,582],
[3539,17,291],[3539,18,419],[3539,19,814],[3539,20,659],[3539,21,33],
[3539,22,72],[3539,23,140],[3539,24,162],[3539,25,72],[3539,26,72],
[3539,27,152],[3539,28,894],[3539,29,165],[3539,30,133],[3539,31,100],
[3539,32,164],[3539,33,66],[3539,34,276],[3539,35,109],[3539,36,230],
[3539,37,353],[3539,38,99],[3539,39,242],[3539,40,94],[3539,41,522],
[3539,42,65],[3539,43,45],[3539,44,129],[3539,45,515],[3539,46,333],
[3539,47,59],[3539,48,56],[3539,49,193],[3539,50,81],[3539,51,28],
[3539,52,15],[3539,53,321],[3539,54,237],[3539,55,491],[3539,56,43],
[3539,57,359],[3539,58,321],[3539,59,444],[3539,60,115],[3539,61,21],
[3539,62,69],[3539,63,138],[3539,64,107],[3539,65,665],[3539,66,131],
[3539,67,314],[3539,68,62],[3539,69,43],[3539,70,194],[3539,71,226],
[3539,72,42],[3539,73,343],[3539,74,297],[3539,75,194],[3539,76,244],
[3539,77,614],[3539,78,64],[3539,79,1],[3540,1,1],[3540,2,1],
[3540,3,1],[3540,4,1],[3540,17,1],[3540,21,1],[3540,23,6],[3540,24,6],
[3540,26,1],[3540,27,1],[3540,28,1],[3540,31,1],[3540,33,2],
[3540,35,2],[3540,42,2],[3540,43,1],[3540,69,1],[3541,43,2],
[3542,21,1],[3542,22,1],[3542,23,1],[3542,27,2],[3542,28,1],
[3542,33,1],[3542,47,1],[3543,17,1],[3543,23,1],[3543,24,1],
[3543,28,2],[3544,4,1],[3544,42,2],[3544,52,1],[3544,68,1],[3545,7,1],

[3545,19,1],[3545,41,1],[3545,55,1],[3545,67,1],[3546,5,1],
[3546,63,6],[3546,75,1],[3546,76,2],[3547,15,2],[3547,42,1],
[3547,55,3],[3547,58,1],[3548,3,2],[3548,6,1],[3548,8,1],[3548,9,1],
[3548,10,1],[3548,20,1],[3548,21,2],[3548,22,9],[3548,23,2],
[3548,24,2],[3548,26,1],[3548,27,3],[3548,28,2],[3548,32,1],
[3548,35,3],[3548,40,1],[3548,43,1],[3548,44,1],[3548,62,1],
[3548,70,1],[3548,76,1],[3549,63,3],[3549,74,3],[3549,76,3],
[3550,25,1],[3550,27,2],[3550,28,1],[3550,33,1],[3550,35,2],
[3551,27,3],[3552,2,1],[3552,30,1],[3552,43,1],[3552,56,1],[3553,6,1],
[3553,28,1],[3554,2,1],[3554,20,1],[3554,22,2],[3554,24,1],
[3554,26,1],[3554,35,1],[3554,50,1],[3554,51,1],[3554,56,1],
[3555,0,1],[3555,1,1],[3555,10,4],[3555,15,6],[3555,16,2],[3555,18,1],
[3555,19,1],[3555,20,1],[3555,28,1],[3555,30,1],[3555,36,1],
[3555,37,2],[3555,39,1],[3555,43,1],[3555,45,1],[3555,49,2],
[3555,55,6],[3555,58,2],[3555,59,1],[3555,65,2],[3555,66,1],
[3555,67,1],[3555,70,2],[3555,71,2],[3555,73,2],[3556,3,1],
[3556,23,9],[3557,1,1],[3557,15,1],[3557,20,1],[3558,5,1],[3558,33,1],
[3559,12,2],[3559,29,1],[3559,45,1],[3559,71,1],[3560,3,1],
[3560,25,1],[3560,32,1],[3560,68,1],[3561,28,2],[3562,1,1],[3562,9,1],
[3562,11,1],[3563,2,1],[3563,3,1],[3563,8,1],[3563,21,2],[3563,22,2],
[3563,24,1],[3563,25,6],[3563,26,1],[3563,27,8],[3563,32,2],
[3563,35,1],[3563,37,1],[3563,38,1],[3563,40,1],[3563,44,1],
[3563,47,1],[3563,64,1],[3563,69,1],[3563,75,1],[3563,76,2],
[3564,13,1],[3564,34,1],[3565,17,1],[3565,70,1],[3566,2,3],[3566,5,1],
[3566,23,2],[3566,26,1],[3566,27,1],[3566,30,1],[3566,42,6],
[3566,62,1],[3567,17,2],[3567,53,3],[3567,76,1],[3568,3,4],
[3568,24,1],[3568,26,1],[3568,27,2],[3568,31,2],[3568,34,1],
[3568,40,1],[3568,51,1],[3568,56,1],[3568,60,1],[3568,62,1],
[3568,76,1],[3569,31,3],[3570,2,2],[3571,20,1],[3571,21,1],
[3571,22,3],[3571,27,4],[3571,30,1],[3571,31,1],[3571,33,1],
[3571,42,1],[3571,48,1],[3572,24,1],[3572,26,1],[3572,31,3],
[3573,2,1],[3573,68,1],[3574,15,5],[3574,55,1],[3575,5,1],[3575,33,5],
[3575,59,1],[3576,27,3],[3577,23,2],[3577,28,1],[3578,28,1],
[3578,70,1],[3579,28,4],[3580,4,1],[3580,27,3],[3580,32,6],
[3580,33,7],[3580,35,9],[3580,52,4],[3581,26,1],[3581,28,1],
[3581,43,1],[3582,24,2],[3582,28,1],[3583,14,1],[3583,15,1],
[3584,1,1],[3584,27,1],[3585,23,3],[3585,42,2],[3585,48,1],
[3586,42,1],[3586,56,1],[3586,72,1],[3587,2,3],[3587,3,1],[3587,22,1],
[3587,23,3],[3587,26,1],[3587,31,1],[3587,32,3],[3587,35,2],
[3587,39,1],[3587,40,1],[3587,59,2],[3587,76,2],[3588,32,4],
[3588,39,1],[3589,25,1],[3589,35,1],[3590,1,1],[3590,12,1],
[3590,19,1],[3590,37,1],[3590,58,1],[3591,55,2],[3592,0,1],[3592,1,2],
[3592,6,1],[3592,7,1],[3592,9,12],[3592,10,17],[3592,11,3],
[3592,12,3],[3592,13,4],[3592,14,1],[3592,15,70],[3592,16,6],
[3592,17,5],[3592,18,5],[3592,19,10],[3592,22,2],[3592,23,2],
[3592,26,1],[3592,27,1],[3592,28,5],[3592,31,1],[3592,32,1],
[3592,36,3],[3592,37,4],[3592,38,1],[3592,39,2],[3592,40,2],
[3592,41,3],[3592,42,1],[3592,44,1],[3592,45,2],[3592,46,2],
[3592,47,1],[3592,48,2],[3592,49,2],[3592,53,4],[3592,54,1],
[3592,55,309],[3592,57,7],[3592,58,1],[3592,59,2],[3592,63,1],

[3592,64,2],[3592,65,8],[3592,66,1],[3592,67,2],[3592,70,1],
[3592,71,5],[3592,72,1],[3592,73,2],[3592,77,5],[3592,78,2],
[3593,51,13],[3594,5,2],[3595,15,1],[3595,55,2],[3596,74,4],
[3597,2,1],[3597,27,1],[3597,34,1],[3597,65,1],[3597,68,1],[3598,3,2],
[3598,5,1],[3598,20,1],[3598,22,1],[3598,23,2],[3598,24,3],
[3598,25,2],[3598,27,2],[3598,32,2],[3598,35,2],[3598,38,1],
[3598,56,1],[3598,62,1],[3599,27,3],[3599,52,1],[3599,56,1],
[3600,0,2],[3600,1,39],[3600,2,8],[3600,3,3],[3600,4,150],[3600,5,17],
[3600,7,3],[3600,8,6],[3600,9,18],[3600,10,5],[3600,11,1],
[3600,12,13],[3600,13,21],[3600,14,1],[3600,15,262],[3600,16,1],
[3600,17,304],[3600,18,2],[3600,20,31],[3600,21,6],[3600,22,10],
[3600,23,11],[3600,24,10],[3600,25,11],[3600,26,4],[3600,27,15],
[3600,28,68],[3600,29,4],[3600,30,8],[3600,31,14],[3600,32,9],
[3600,33,4],[3600,34,5],[3600,35,7],[3600,36,1],[3600,37,1],
[3600,38,29],[3600,39,8],[3600,40,5],[3600,41,1],[3600,42,6],
[3600,43,3],[3600,44,104],[3600,45,3],[3600,46,3],[3600,47,5],
[3600,48,1],[3600,49,1],[3600,50,168],[3600,51,3],[3600,52,3],
[3600,53,337],[3600,55,516],[3600,56,7],[3600,57,2],[3600,58,2],
[3600,59,4],[3600,60,2],[3600,62,5],[3600,63,4],[3600,65,3],
[3600,66,112],[3600,67,1],[3600,68,1],[3600,70,16],[3600,71,1],
[3600,72,8],[3600,74,9],[3600,75,4],[3600,76,11],[3600,77,8],
[3600,78,2],[3600,79,1],[3601,4,1],[3601,5,2],[3601,21,1],[3601,23,2],
[3601,24,2],[3601,26,1],[3601,27,3],[3601,28,1],[3601,32,8],
[3601,35,1],[3601,43,1],[3602,23,2],[3602,24,2],[3602,27,2],
[3602,28,1],[3602,62,2],[3603,2,3],[3603,3,1],[3604,43,2],[3605,3,1],
[3605,33,1],[3605,66,1],[3606,56,1],[3606,62,1],[3607,24,1],
[3607,25,1],[3607,27,8],[3607,28,1],[3607,44,1],[3607,48,1],
[3608,10,1],[3608,13,1],[3608,22,1],[3608,27,1],[3608,35,1],
[3608,36,1],[3608,47,1],[3608,55,1],[3608,57,1],[3608,65,1],
[3609,7,1],[3609,21,1],[3609,27,1],[3609,35,1],[3610,3,3],[3610,5,1],
[3610,8,2],[3610,15,1],[3610,22,1],[3610,23,3],[3610,24,5],
[3610,26,1],[3610,28,1],[3610,47,1],[3610,50,1],[3610,56,3],
[3610,59,3],[3610,64,1],[3610,69,1],[3611,20,4],[3611,23,1],
[3611,26,1],[3611,32,1],[3611,46,1],[3611,59,2],[3612,5,1],
[3612,51,4],[3613,28,5],[3614,28,1],[3614,66,1],[3615,28,7],
[3616,23,5],[3616,31,1],[3616,39,1],[3616,42,3],[3616,47,1],
[3616,70,1],[3617,12,1],[3617,16,1],[3617,77,1],[3618,3,1],
[3618,31,1],[3619,2,1],[3619,13,1],[3619,27,1],[3620,1,1],[3620,12,2],
[3620,13,1],[3620,42,1],[3621,23,1],[3621,27,2],[3622,12,1],
[3622,23,1],[3622,54,1],[3622,55,1],[3622,60,1],[3623,23,4],
[3623,48,1],[3623,75,1],[3624,1,3],[3624,8,1],[3624,9,8],[3624,10,5],
[3624,11,4],[3624,12,2],[3624,13,3],[3624,14,2],[3624,15,4],
[3624,16,6],[3624,17,3],[3624,18,3],[3624,19,6],[3624,20,2],
[3624,29,3],[3624,30,2],[3624,34,4],[3624,37,4],[3624,39,2],
[3624,41,4],[3624,45,2],[3624,46,1],[3624,49,2],[3624,53,3],
[3624,54,4],[3624,55,2],[3624,59,1],[3624,60,1],[3624,64,1],
[3624,65,4],[3624,67,1],[3624,71,4],[3624,73,1],[3624,77,2],
[3625,78,2],[3626,4,3],[3626,28,2],[3626,47,2],[3626,50,3],
[3627,15,1],[3627,55,1],[3628,21,2],[3628,66,1],[3628,72,1],
[3628,76,1],[3629,1,4],[3630,31,3],[3631,1,2],[3632,1,1],[3632,9,1],

[3632,19,1],[3633,75,2],[3634,18,1],[3634,28,5],[3635,23,8],
[3635,27,1],[3635,42,1],[3635,51,1],[3636,21,5],[3636,23,1],
[3636,26,3],[3636,28,1],[3636,30,2],[3636,31,8],[3636,33,5],
[3637,23,1],[3637,24,1],[3637,28,1],[3637,32,1],[3637,75,1],
[3638,1,1],[3638,2,1],[3638,3,2],[3638,20,1],[3638,23,49],[3638,24,4],
[3638,26,1],[3638,27,103],[3638,28,4],[3638,32,1],[3638,33,1],
[3638,36,1],[3638,39,5],[3638,42,1],[3638,62,2],[3638,65,1],
[3638,70,1],[3639,5,1],[3639,28,1],[3640,5,1],[3640,50,1],[3641,23,1],
[3641,27,4],[3642,21,2],[3643,21,2],[3643,31,1],[3643,32,1],
[3643,42,1],[3644,3,3],[3645,4,2],[3645,5,1],[3645,50,1],[3646,2,35],
[3647,2,1],[3647,26,1],[3648,1,2],[3648,12,1],[3648,13,2],[3648,29,2],
[3648,39,2],[3649,1,1],[3649,2,1],[3649,3,6],[3649,5,1],[3649,6,5],
[3649,19,1],[3649,20,7],[3649,21,2],[3649,22,4],[3649,23,28],
[3649,24,13],[3649,25,4],[3649,26,11],[3649,27,23],[3649,28,19],
[3649,31,8],[3649,32,9],[3649,33,21],[3649,35,13],[3649,36,1],
[3649,37,1],[3649,38,3],[3649,39,8],[3649,40,1],[3649,43,3],
[3649,45,1],[3649,46,1],[3649,47,3],[3649,48,6],[3649,50,2],
[3649,53,1],[3649,56,1],[3649,65,1],[3649,68,1],[3649,69,1],
[3649,76,2],[3650,20,1],[3650,67,1],[3651,22,8],[3652,3,1],
[3652,13,2],[3652,32,1],[3652,62,3],[3653,1,3],[3653,9,1],[3653,28,1],
[3654,9,1],[3654,32,1],[3654,33,1],[3654,39,1],[3655,17,3],
[3655,53,2],[3656,27,1],[3656,35,1],[3656,55,1],[3656,75,1],
[3657,24,1],[3657,25,1],[3657,26,1],[3657,27,3],[3657,34,3],
[3657,39,1],[3657,43,1],[3658,8,4],[3658,27,1],[3658,46,1],
[3658,64,1],[3659,3,1],[3659,28,1],[3659,31,1],[3659,50,1],[3660,3,1],
[3660,56,1],[3660,76,1],[3661,0,1],[3661,5,3],[3661,6,3],[3661,12,1],
[3661,21,2],[3661,22,8],[3661,23,12],[3661,24,5],[3661,25,7],
[3661,26,1],[3661,27,28],[3661,28,6],[3661,31,6],[3661,32,20],
[3661,33,8],[3661,35,5],[3661,38,1],[3661,42,3],[3661,43,4],
[3661,47,1],[3661,56,5],[3661,61,2],[3661,65,1],[3662,10,1],
[3662,57,1],[3663,32,3],[3664,22,1],[3664,52,1],[3665,32,2],
[3665,33,1],[3665,75,1],[3666,3,4],[3666,23,12],[3666,24,23],
[3666,25,25],[3666,26,1],[3666,27,1],[3666,32,1],[3666,33,1],
[3666,34,1],[3666,42,1],[3666,44,1],[3666,59,1],[3666,62,1],
[3666,70,1],[3666,76,1],[3667,26,2],[3667,42,2],[3668,4,1],[3668,6,1],
[3668,25,1],[3668,26,3],[3668,27,1],[3668,28,9],[3668,33,1],
[3668,35,2],[3668,50,2],[3668,70,3],[3668,76,1],[3669,3,1],[3669,6,1],
[3669,20,1],[3669,23,4],[3669,24,11],[3669,25,1],[3669,28,6],
[3669,34,1],[3669,50,1],[3669,63,2],[3669,70,1],[3670,27,1],
[3670,28,2],[3670,32,1],[3670,42,2],[3670,56,1],[3671,9,1],
[3671,10,1],[3671,13,1],[3671,15,1],[3671,36,1],[3671,59,1],
[3671,65,1],[3672,10,2],[3672,16,1],[3672,17,1],[3672,19,2],
[3673,43,3],[3674,4,2],[3674,66,1],[3675,22,1],[3675,28,1],
[3675,54,1],[3676,15,1],[3676,28,1],[3677,2,2],[3677,3,1],[3677,10,2],
[3677,50,1],[3678,21,1],[3678,22,1],[3678,23,1],[3678,24,1],
[3678,51,1],[3678,56,1],[3679,23,1],[3679,28,1],[3679,32,1],
[3679,56,9],[3679,75,1],[3680,0,1],[3680,22,1],[3680,32,1],
[3680,59,1],[3681,10,1],[3681,41,1],[3682,4,1],[3682,23,1],
[3682,24,2],[3682,27,2],[3682,28,4],[3682,31,1],[3682,38,1],
[3682,43,1],[3682,52,1],[3682,63,1],[3682,70,1],[3683,14,1],

[3683,21,1],[3683,26,2],[3683,27,3],[3683,74,1],[3684,20,3],
[3685,17,1],[3685,24,1],[3685,33,1],[3685,34,1],[3685,50,1],
[3685,55,1],[3685,63,1],[3686,22,3],[3687,13,1],[3687,20,1],
[3687,21,1],[3688,2,1],[3688,23,2],[3688,25,6],[3688,27,1],
[3688,32,9],[3688,33,1],[3688,35,1],[3688,42,1],[3689,29,3],
[3690,1,1],[3690,2,2],[3690,3,4],[3690,5,1],[3690,7,1],[3690,8,1],
[3690,10,2],[3690,17,1],[3690,19,1],[3690,21,1],[3690,23,3],
[3690,24,3],[3690,25,3],[3690,27,4],[3690,28,1],[3690,29,1],
[3690,30,21],[3690,31,2],[3690,32,2],[3690,33,12],[3690,35,16],
[3690,42,1],[3690,43,1],[3690,44,1],[3690,46,1],[3690,47,1],
[3690,52,9],[3690,56,1],[3690,57,1],[3690,58,2],[3690,63,1],
[3690,65,1],[3690,70,4],[3690,72,1],[3690,73,1],[3690,74,3],
[3690,75,1],[3691,38,1],[3691,44,1],[3691,66,1],[3692,8,1],
[3692,13,1],[3692,20,1],[3693,27,1],[3693,28,1],[3693,32,52],
[3693,50,1],[3693,68,1],[3694,33,1],[3694,35,1],[3695,1,1],
[3695,11,1],[3695,12,2],[3695,20,1],[3695,29,2],[3695,39,1],
[3695,54,1],[3695,67,1],[3695,73,2],[3696,4,1],[3696,5,2],[3696,8,3],
[3696,9,1],[3696,13,2],[3696,16,2],[3696,20,1],[3696,22,4],
[3696,23,3],[3696,26,3],[3696,27,11],[3696,28,34],[3696,30,1],
[3696,32,1],[3696,33,1],[3696,35,24],[3696,43,1],[3696,50,1],
[3696,52,1],[3697,39,2],[3698,34,1],[3698,71,1],[3699,23,2],
[3699,32,1],[3699,56,1],[3700,22,1],[3700,28,2],[3700,31,1],
[3701,7,1],[3701,20,1],[3702,74,2],[3703,32,2],[3703,47,1],
[3704,23,1],[3704,24,2],[3704,28,6],[3704,40,1],[3705,10,1],
[3705,57,1],[3705,67,1],[3706,22,1],[3706,24,1],[3706,35,1],
[3707,2,3],[3708,5,1],[3708,28,1],[3708,31,1],[3708,33,67],
[3708,70,1],[3709,28,1],[3709,50,1],[3710,30,1],[3710,46,2],
[3711,25,1],[3711,47,1],[3711,48,1],[3712,4,2],[3713,12,1],
[3713,46,1],[3714,62,2],[3715,2,1],[3715,10,1],[3715,21,1],
[3715,23,1],[3715,26,1],[3715,31,1],[3715,33,1],[3715,35,1],
[3715,37,3],[3715,43,3],[3715,48,1],[3715,59,1],[3715,62,1],
[3716,28,1],[3716,38,1],[3717,17,1],[3717,49,1],[3717,57,1],
[3718,27,1],[3718,50,1],[3718,63,12],[3718,74,10],[3718,75,6],
[3718,76,6],[3719,27,3],[3719,48,2],[3720,1,1],[3720,4,1],[3720,15,3],
[3720,53,2],[3720,55,1],[3721,10,1],[3721,28,2],[3721,41,1],
[3722,0,5],[3722,1,2],[3722,3,1],[3722,7,1],[3722,10,5],[3722,12,8],
[3722,13,3],[3722,15,2],[3722,19,5],[3722,20,3],[3722,23,1],
[3722,27,2],[3722,29,9],[3722,30,2],[3722,31,18],[3722,32,1],
[3722,41,3],[3722,54,2],[3722,55,1],[3722,57,2],[3722,65,2],
[3722,71,2],[3722,77,8],[3723,2,1],[3723,24,1],[3723,27,1],
[3724,13,1],[3724,20,3],[3724,21,1],[3724,39,1],[3725,2,27],
[3725,3,5],[3725,4,1],[3725,15,1],[3725,17,1],[3725,20,1],[3725,21,1],
[3725,22,1],[3725,24,2],[3725,27,5],[3725,28,1],[3725,40,1],
[3725,42,2],[3725,47,7],[3725,53,1],[3726,0,1],[3726,2,4],[3726,9,8],
[3726,10,1],[3726,12,17],[3726,13,1],[3726,20,1],[3726,28,4],
[3726,29,9],[3726,30,1],[3726,44,1],[3726,46,1],[3726,54,1],
[3726,74,1],[3726,77,1],[3727,15,1],[3727,17,1],[3727,53,3],
[3727,55,3],[3728,21,1],[3728,23,1],[3728,24,1],[3728,26,1],
[3728,27,1],[3729,10,1],[3729,55,1],[3730,0,1],[3730,1,5],[3730,2,1],
[3730,4,1],[3730,5,129],[3730,6,2],[3730,8,1],[3730,9,5],[3730,10,4],

[3730,11,4],[3730,12,48],[3730,13,1],[3730,15,8],[3730,16,1],
[3730,17,3],[3730,18,1],[3730,19,3],[3730,20,4],[3730,23,3],
[3730,24,3],[3730,25,1],[3730,26,3],[3730,28,19],[3730,29,123],
[3730,30,2],[3730,31,1],[3730,32,2],[3730,34,3],[3730,35,1],
[3730,36,1],[3730,37,3],[3730,39,315],[3730,40,1],[3730,41,1],
[3730,42,3],[3730,43,1],[3730,44,5],[3730,45,1],[3730,46,3],
[3730,47,1],[3730,52,145],[3730,53,3],[3730,55,3],[3730,56,1],
[3730,57,1],[3730,58,3],[3730,59,15],[3730,60,1],[3730,65,5],
[3730,66,1],[3730,71,1],[3730,73,3],[3730,74,2],[3730,75,3],
[3730,76,3],[3730,77,1],[3731,24,1],[3731,28,2],[3731,43,1],
[3731,70,1],[3732,15,3],[3732,17,1],[3732,44,1],[3733,18,1],
[3733,41,1],[3734,23,1],[3734,62,1],[3735,15,1],[3735,17,1],
[3735,27,1],[3735,37,1],[3735,44,6],[3735,50,2],[3735,53,1],
[3735,55,6],[3735,56,1],[3735,66,4],[3736,19,1],[3736,43,1],
[3736,55,2],[3737,28,1],[3737,32,1],[3738,3,1],[3738,23,1],
[3738,24,3],[3738,25,1],[3738,35,1],[3738,56,1],[3738,66,1],
[3738,75,1],[3739,4,1],[3739,63,1],[3739,70,1],[3739,76,1],
[3740,72,2],[3741,28,1],[3741,40,1],[3742,32,2],[3742,53,1],
[3743,23,1],[3743,24,2],[3743,30,1],[3743,32,2],[3743,42,1],
[3744,8,1],[3744,12,3],[3744,17,2],[3744,20,1],[3744,27,1],
[3744,34,1],[3744,36,1],[3744,53,1],[3745,45,3],[3746,22,1],
[3746,27,1],[3746,31,1],[3747,1,1],[3747,57,1],[3748,2,2],[3749,22,1],
[3749,23,1],[3749,25,1],[3749,27,1],[3749,31,2],[3749,33,1],
[3749,35,5],[3749,38,3],[3749,40,4],[3749,41,1],[3749,43,2],
[3749,44,1],[3749,47,1],[3749,56,1],[3749,58,1],[3749,63,81],
[3749,66,1],[3749,68,2],[3749,69,1],[3749,70,4],[3749,72,2],
[3749,74,93],[3749,75,56],[3749,76,126],[3750,3,5],[3750,5,2],
[3750,13,4],[3750,24,2],[3750,27,3],[3750,33,1],[3750,35,2],
[3750,37,1],[3750,42,3],[3751,2,3],[3752,27,2],[3753,3,1],[3753,22,1],
[3754,9,1],[3754,20,1],[3755,14,1],[3755,33,1],[3756,2,1],[3756,25,1],
[3756,28,1],[3756,44,1],[3757,0,1],[3757,12,1],[3757,19,1],
[3757,29,1],[3757,31,3],[3757,34,2],[3757,58,1],[3757,62,1],
[3758,15,1],[3758,20,1],[3758,36,1],[3758,44,2],[3758,55,1],
[3759,24,7],[3760,39,1],[3760,73,1],[3761,2,1],[3761,63,1],
[3761,75,1],[3761,76,1],[3762,5,1],[3762,22,2],[3762,27,2],
[3762,28,1],[3763,52,1],[3763,70,1],[3764,25,2],[3765,15,1],
[3765,50,1],[3766,5,1],[3766,15,1],[3766,24,1],[3766,26,1],
[3766,31,3],[3766,38,1],[3766,44,30],[3766,50,2],[3766,61,1],
[3766,66,30],[3767,15,1],[3767,17,1],[3768,9,1],[3768,28,2],
[3769,3,2],[3769,6,1],[3769,20,1],[3769,34,3],[3769,48,1],[3769,56,1],
[3770,24,1],[3770,25,1],[3770,51,1],[3770,77,2],[3771,35,1],
[3771,52,1],[3772,22,1],[3772,27,1],[3773,31,1],[3773,43,1],
[3773,44,1],[3774,3,2],[3774,24,1],[3774,43,1],[3774,59,1],
[3775,15,1],[3775,17,2],[3775,53,4],[3775,55,1],[3776,5,1],[3776,8,1],
[3776,25,1],[3776,26,1],[3776,30,17],[3776,32,1],[3776,33,15],
[3776,37,1],[3776,53,1],[3777,1,2],[3777,4,1],[3777,6,1],[3777,7,2],
[3777,8,2],[3777,9,8],[3777,10,11],[3777,11,3],[3777,13,3],
[3777,14,1],[3777,15,5],[3777,16,5],[3777,18,2],[3777,19,7],
[3777,20,1],[3777,23,1],[3777,24,2],[3777,27,1],[3777,30,1],
[3777,33,1],[3777,34,2],[3777,35,1],[3777,36,1],[3777,37,2],

[3777,39,1],[3777,41,6],[3777,45,8],[3777,46,2],[3777,49,2],
[3777,50,1],[3777,53,1],[3777,54,4],[3777,55,2],[3777,57,6],
[3777,58,1],[3777,59,3],[3777,60,1],[3777,65,6],[3777,70,1],
[3777,76,1],[3777,77,1],[3777,78,1],[3778,20,4],[3778,39,2],
[3779,28,1],[3779,62,1],[3780,43,2],[3781,23,1],[3781,24,1],
[3781,26,1],[3781,31,2],[3781,35,1],[3781,47,2],[3781,51,1],
[3781,68,1],[3782,7,1],[3782,10,3],[3782,14,1],[3782,16,1],
[3782,18,3],[3782,19,3],[3782,20,1],[3782,34,1],[3782,41,4],
[3782,46,1],[3782,49,1],[3782,55,1],[3782,57,1],[3782,59,3],
[3782,65,4],[3782,67,3],[3782,73,3],[3782,77,4],[3783,2,5],[3783,3,1],
[3783,22,4],[3783,23,1],[3783,25,1],[3783,27,6],[3783,32,1],
[3783,33,1],[3783,35,3],[3783,52,1],[3783,63,1],[3783,75,1],
[3783,76,1],[3784,3,1],[3784,26,1],[3784,28,1],[3784,31,2],
[3784,47,1],[3785,2,2],[3785,4,1],[3785,5,2],[3785,6,1],[3785,10,1],
[3785,15,1],[3785,18,1],[3785,20,1],[3785,21,1],[3785,22,2],
[3785,23,2],[3785,27,4],[3785,28,1],[3785,30,4],[3785,31,1],
[3785,32,3],[3785,35,5],[3785,38,1],[3785,43,4],[3785,47,2],
[3785,50,1],[3785,51,1],[3785,59,1],[3785,62,1],[3785,69,1],
[3785,70,2],[3786,22,1],[3786,23,1],[3786,27,4],[3786,56,1],
[3787,2,1],[3787,28,1],[3788,27,2],[3789,2,2],[3790,8,1],[3790,25,5],
[3790,28,1],[3790,33,15],[3790,35,8],[3790,72,1],[3791,24,3],
[3792,4,1],[3792,50,1],[3792,53,3],[3793,1,4],[3793,2,1],[3793,4,32],
[3793,5,7],[3793,6,1],[3793,8,2],[3793,10,1],[3793,12,5],[3793,13,2],
[3793,14,1],[3793,15,46],[3793,17,10],[3793,19,1],[3793,20,1],
[3793,21,1],[3793,22,2],[3793,24,2],[3793,25,1],[3793,26,1],
[3793,28,5],[3793,29,2],[3793,32,1],[3793,34,1],[3793,35,1],
[3793,38,4],[3793,39,2],[3793,40,1],[3793,41,1],[3793,44,33],
[3793,45,1],[3793,46,1],[3793,48,1],[3793,50,44],[3793,52,1],
[3793,53,21],[3793,54,1],[3793,55,22],[3793,58,1],[3793,59,1],
[3793,60,1],[3793,62,1],[3793,64,1],[3793,65,3],[3793,66,25],
[3793,67,1],[3793,68,1],[3793,70,1],[3793,71,1],[3793,72,3],
[3793,73,1],[3793,74,1],[3793,76,1],[3794,24,14],[3794,25,43],
[3794,26,5],[3794,28,1],[3794,31,4],[3794,33,13],[3794,47,6],
[3795,3,1],[3795,4,1],[3795,5,1],[3795,6,2],[3795,23,2],[3795,25,1],
[3795,26,1],[3795,27,2],[3795,28,4],[3795,31,1],[3795,38,3],
[3795,44,2],[3795,47,6],[3795,51,3],[3795,56,3],[3795,61,2],
[3795,66,4],[3795,68,2],[3795,69,2],[3795,70,3],[3795,72,1],
[3795,74,29],[3795,76,1],[3796,2,4],[3796,28,2],[3797,6,1],
[3797,27,2],[3797,33,1],[3797,35,1],[3797,56,3],[3798,1,1],
[3798,13,1],[3798,59,1],[3799,31,2],[3800,1,5],[3800,2,11],[3800,3,4],
[3800,4,12],[3800,5,87],[3800,6,5],[3800,8,1],[3800,9,4],[3800,13,22],
[3800,16,1],[3800,20,2],[3800,21,62],[3800,22,5],[3800,23,8],
[3800,24,10],[3800,25,15],[3800,26,20],[3800,27,23],[3800,28,62],
[3800,29,1],[3800,31,7],[3800,32,66],[3800,33,83],[3800,34,2],
[3800,35,18],[3800,36,1],[3800,37,6],[3800,38,2],[3800,39,1],
[3800,40,2],[3800,42,2],[3800,43,5],[3800,47,215],[3800,48,95],
[3800,50,10],[3800,51,4],[3800,52,3],[3800,53,1],[3800,56,2],
[3800,59,1],[3800,61,2],[3800,62,3],[3800,63,1],[3800,66,3],
[3800,68,2],[3800,69,3],[3800,70,11],[3800,73,1],[3800,74,5],
[3800,75,2],[3800,76,2],[3800,78,1],[3800,79,5],[3801,2,1],[3801,3,1],

[3801,24,1],[3802,1,1],[3802,2,53],[3802,23,2],[3802,25,3],
[3802,42,1],[3802,56,1],[3802,68,1],[3803,20,2],[3804,6,2],
[3805,70,2],[3806,1,88],[3806,2,12],[3806,4,4],[3806,5,5],[3806,8,3],
[3806,9,3],[3806,10,1],[3806,11,46],[3806,12,154],[3806,13,8],
[3806,15,10],[3806,16,5],[3806,17,2],[3806,20,27],[3806,21,2],
[3806,22,3],[3806,23,2],[3806,24,5],[3806,26,2],[3806,27,1],
[3806,28,3],[3806,29,12],[3806,30,6],[3806,31,1],[3806,32,1],
[3806,33,1],[3806,34,2],[3806,35,1],[3806,36,1],[3806,37,1],
[3806,38,2],[3806,39,5],[3806,40,1],[3806,41,1],[3806,42,4],
[3806,43,3],[3806,44,4],[3806,45,2],[3806,46,1],[3806,47,1],
[3806,50,3],[3806,51,1],[3806,54,2],[3806,55,3],[3806,56,4],
[3806,57,1],[3806,58,1],[3806,59,3],[3806,60,2],[3806,61,1],
[3806,62,3],[3806,63,1],[3806,64,1],[3806,66,1],[3806,69,2],
[3806,70,1],[3806,73,1],[3806,74,2],[3806,75,1],[3806,77,2],
[3807,23,1],[3807,35,2],[3808,30,4],[3808,35,9],[3808,42,1],
[3809,22,1],[3809,28,1],[3810,18,1],[3810,40,1],[3810,44,1],
[3810,48,1],[3810,55,1],[3811,63,1],[3811,74,1],[3811,76,1],
[3812,14,1],[3812,15,1],[3812,57,1],[3812,77,1],[3813,4,1],
[3813,28,1],[3814,5,1],[3814,66,1],[3815,3,2],[3815,32,2],[3815,33,1],
[3815,46,1],[3816,25,7],[3817,2,2],[3818,22,1],[3818,23,2],
[3818,25,1],[3818,33,1],[3818,74,2],[3819,25,1],[3819,32,2],
[3820,2,44],[3820,3,2],[3820,5,3],[3820,22,1],[3820,24,2],[3820,27,2],
[3820,28,1],[3820,31,2],[3820,32,1],[3820,33,1],[3820,43,1],
[3820,44,1],[3820,47,2],[3820,62,1],[3820,71,1],[3821,2,1],[3821,4,1],
[3821,6,5],[3821,23,1],[3821,25,2],[3821,28,1],[3821,31,7],
[3821,43,4],[3821,47,4],[3821,48,4],[3822,29,2],[3823,1,2],
[3823,20,2],[3823,38,1],[3823,39,1],[3824,2,2],[3824,22,1],[3825,2,3],
[3826,22,1],[3826,23,2],[3826,27,3],[3826,29,1],[3826,32,1],
[3826,35,1],[3827,30,13],[3828,25,1],[3828,32,14],[3829,7,2],
[3830,4,2],[3830,7,1],[3830,24,1],[3830,57,1],[3831,26,1],[3831,28,1],
[3831,31,7],[3831,43,6],[3832,23,1],[3832,46,1],[3832,47,1],
[3833,20,1],[3833,27,1],[3834,1,1],[3834,17,1],[3834,42,8],
[3835,28,2],[3835,44,1],[3836,12,1],[3836,29,1],[3837,23,1],
[3837,24,1],[3837,35,1],[3838,2,5],[3838,47,1],[3839,26,1],
[3839,28,1],[3840,12,2],[3840,29,2],[3841,22,2],[3842,5,1],
[3842,23,1],[3842,26,1],[3842,31,1],[3842,32,1],[3842,33,3],
[3842,38,1],[3842,57,1],[3843,22,1],[3843,32,1],[3843,56,1],
[3844,29,1],[3844,55,1],[3845,0,1],[3845,3,1],[3845,28,1],[3846,24,2],
[3847,9,2],[3847,12,1],[3847,15,1],[3847,19,2],[3847,21,1],
[3847,23,1],[3847,25,1],[3847,27,1],[3847,28,6],[3847,45,1],
[3847,49,2],[3847,53,1],[3847,55,1],[3847,58,1],[3847,59,1],
[3847,62,2],[3847,66,1],[3847,68,2],[3847,70,1],[3847,74,3],
[3847,75,1],[3847,76,1],[3848,17,1],[3848,36,1],[3848,53,1],
[3848,55,1],[3849,2,2],[3850,20,2],[3851,39,3],[3852,27,2],[3853,4,1],
[3853,30,1],[3853,31,1],[3853,41,1],[3854,1,1],[3854,9,1],[3854,13,1],
[3854,19,1],[3854,30,1],[3854,57,1],[3855,2,1],[3855,3,6],[3855,22,2],
[3855,23,3],[3855,24,4],[3855,25,1],[3855,26,4],[3855,27,2],
[3855,28,3],[3855,31,2],[3855,42,1],[3855,43,1],[3855,72,1],
[3856,9,1],[3856,12,13],[3856,29,1],[3856,38,1],[3856,51,2],
[3856,69,1],[3856,73,1],[3857,18,1],[3857,20,1],[3858,2,7],[3858,3,2],

[3858,5,4],[3858,8,2],[3858,13,1],[3858,20,3],[3858,21,2],[3858,23,8],
[3858,24,9],[3858,25,2],[3858,26,7],[3858,27,5],[3858,28,4],
[3858,30,3],[3858,31,9],[3858,32,3],[3858,33,1],[3858,34,1],
[3858,39,2],[3858,42,2],[3858,43,3],[3858,47,1],[3858,52,1],
[3858,62,1],[3858,72,2],[3859,15,2],[3859,44,3],[3859,66,1],
[3860,28,1],[3860,63,1],[3860,75,1],[3861,12,1],[3861,28,9],
[3861,42,1],[3862,25,2],[3862,27,1],[3862,31,1],[3863,22,1],
[3863,47,2],[3864,27,2],[3865,11,1],[3865,31,1],[3866,25,5],
[3867,15,1],[3867,44,1],[3868,23,2],[3868,28,1],[3868,31,3],
[3868,43,1],[3869,5,1],[3869,25,1],[3870,15,1],[3870,55,1],
[3871,25,2],[3871,26,1],[3871,27,1],[3871,32,1],[3871,33,1],
[3871,42,1],[3871,43,2],[3872,3,4],[3872,4,1],[3872,23,7],[3872,24,1],
[3872,25,1],[3872,27,2],[3872,28,1],[3872,32,4],[3872,40,1],
[3872,48,3],[3872,56,3],[3872,62,1],[3873,2,1],[3873,4,1],[3873,5,1],
[3873,23,1],[3873,24,1],[3873,25,1],[3873,35,1],[3873,50,2],
[3873,63,53],[3873,66,1],[3873,67,1],[3873,68,2],[3873,70,2],
[3873,74,46],[3873,75,54],[3873,76,88],[3874,20,1],[3874,53,1],
[3875,0,4],[3875,1,19],[3875,7,3],[3875,8,4],[3875,9,18],[3875,10,15],
[3875,11,3],[3875,12,20],[3875,13,7],[3875,14,3],[3875,15,7],
[3875,16,9],[3875,17,7],[3875,18,3],[3875,19,13],[3875,20,10],
[3875,22,2],[3875,24,3],[3875,26,4],[3875,28,1],[3875,29,6],
[3875,30,1],[3875,31,2],[3875,32,3],[3875,34,2],[3875,35,32],
[3875,36,1],[3875,37,4],[3875,39,7],[3875,41,7],[3875,42,4],
[3875,43,1],[3875,45,6],[3875,46,6],[3875,49,3],[3875,53,8],
[3875,54,2],[3875,55,8],[3875,57,7],[3875,58,2],[3875,59,7],
[3875,60,1],[3875,62,2],[3875,64,1],[3875,65,18],[3875,67,4],
[3875,71,2],[3875,73,3],[3875,77,9],[3875,78,1],[3876,13,2],
[3876,32,1],[3877,3,2],[3877,25,2],[3877,42,1],[3878,15,3],
[3878,28,6],[3878,44,4],[3878,50,2],[3878,55,1],[3878,66,3],
[3878,74,1],[3879,3,6],[3879,4,4],[3879,5,1],[3879,12,3],[3879,15,1],
[3879,16,1],[3879,17,9],[3879,19,1],[3879,22,2],[3879,23,1],
[3879,24,5],[3879,27,2],[3879,28,1],[3879,31,6],[3879,32,9],
[3879,33,3],[3879,35,1],[3879,38,1],[3879,42,1],[3879,43,4],
[3879,47,1],[3879,50,4],[3879,52,1],[3879,53,12],[3879,55,1],
[3879,56,1],[3879,66,2],[3879,70,1],[3880,5,1],[3880,22,1],
[3880,28,3],[3880,50,3],[3880,55,3],[3880,62,1],[3880,66,3],
[3881,5,1],[3881,22,1],[3881,23,1],[3881,26,1],[3881,27,1],
[3881,35,1],[3881,62,1],[3882,63,2],[3882,74,1],[3882,75,1],
[3882,76,4],[3883,9,3],[3883,33,2],[3883,37,2],[3883,45,1],
[3883,59,2],[3884,2,1],[3884,35,1],[3885,52,5],[3886,2,1],[3886,6,1],
[3886,8,2],[3886,35,1],[3886,41,1],[3886,49,1],[3886,58,1],
[3886,77,1],[3887,15,1],[3887,59,1],[3888,1,4],[3888,13,1],[3889,3,1],
[3889,22,1],[3889,27,2],[3889,59,1],[3889,66,1],[3890,5,2],[3890,8,1],
[3890,21,1],[3890,23,1],[3890,27,1],[3890,32,2],[3890,33,1],
[3890,42,1],[3891,21,3],[3891,66,1],[3891,70,1],[3892,15,1],
[3892,50,1],[3892,55,1],[3893,3,2],[3893,12,1],[3893,16,1],
[3893,30,1],[3894,2,3],[3894,3,5],[3894,8,1],[3894,21,1],[3894,22,1],
[3894,23,4],[3894,24,5],[3894,25,1],[3894,26,4],[3894,27,3],
[3894,28,4],[3894,30,2],[3894,31,6],[3894,32,2],[3894,33,1],
[3894,35,1],[3894,42,2],[3894,43,3],[3894,47,3],[3894,56,3],

[3895,0,1],[3895,1,1],[3895,9,2],[3895,11,1],[3895,12,48],[3895,13,1],
[3895,20,1],[3895,30,2],[3895,55,1],[3896,28,60],[3896,74,1],
[3896,76,1],[3897,4,1],[3897,32,4],[3897,62,4],[3898,23,1],
[3898,48,2],[3898,56,1],[3899,4,2],[3899,23,1],[3899,35,1],
[3899,43,1],[3899,48,1],[3899,49,1],[3899,52,1],[3899,54,1],
[3899,66,1],[3899,70,3],[3899,72,1],[3899,74,1],[3899,75,2],
[3900,55,1],[3900,65,1],[3900,73,1],[3901,2,2],[3901,39,1],
[3902,39,2],[3903,1,1],[3903,15,1],[3903,16,1],[3903,18,1],[3904,6,5],
[3904,23,6],[3904,27,1],[3905,28,1],[3905,30,4],[3905,32,1],
[3905,52,6],[3906,24,2],[3907,23,1],[3907,35,1],[3907,40,1],
[3907,43,1],[3907,63,1],[3907,69,1],[3908,17,5],[3908,53,5],
[3909,2,10],[3909,28,1],[3909,42,1],[3910,44,1],[3910,50,1],
[3911,32,2],[3912,1,1],[3912,15,1],[3913,40,1],[3913,51,4],
[3913,68,1],[3913,74,2],[3913,75,2],[3913,76,4],[3914,28,1],
[3914,42,1],[3915,27,16],[3916,17,1],[3916,53,2],[3917,1,1],
[3917,45,3],[3917,57,3],[3918,22,8],[3918,24,2],[3918,27,1],
[3918,43,1],[3919,20,1],[3919,24,1],[3919,27,1],[3919,35,2],
[3920,3,1],[3920,10,1],[3920,65,1],[3921,2,1],[3921,31,1],[3922,27,1],
[3922,43,4],[3923,9,1],[3923,19,1],[3923,57,1],[3924,2,1],[3924,24,1],
[3924,28,4],[3925,28,3],[3925,38,2],[3926,0,1],[3926,6,1],[3926,9,1],
[3926,10,1],[3926,11,1],[3926,13,1],[3926,18,1],[3926,19,2],
[3926,20,2],[3926,23,30],[3926,28,1],[3926,36,2],[3926,42,1],
[3926,44,3],[3926,48,1],[3926,50,2],[3926,55,1],[3926,65,2],
[3926,66,1],[3926,74,1],[3926,77,1],[3927,4,1],[3927,28,1],[3928,5,3],
[3928,6,2],[3928,21,1],[3928,28,2],[3928,32,1],[3928,40,1],
[3928,43,1],[3928,44,1],[3928,47,83],[3928,62,2],[3929,2,1],
[3929,21,1],[3929,25,3],[3929,27,8],[3929,28,2],[3929,30,1],
[3929,33,1],[3929,34,1],[3929,35,3],[3929,38,1],[3929,42,1],
[3929,43,2],[3929,50,1],[3929,59,3],[3929,62,1],[3929,63,1],
[3929,74,1],[3930,2,1],[3930,3,1],[3930,5,2],[3930,21,1],[3930,22,1],
[3930,23,1],[3930,24,6],[3930,27,4],[3930,31,1],[3930,32,2],
[3930,33,1],[3930,43,1],[3930,46,1],[3931,21,1],[3931,23,1],
[3931,48,3],[3931,76,1],[3932,24,1],[3932,27,1],[3932,35,1],
[3933,53,2],[3934,9,1],[3934,17,1],[3935,63,1],[3935,74,1],[3936,2,1],
[3936,21,1],[3936,28,1],[3937,24,2],[3938,4,1],[3938,28,6],
[3939,52,1],[3939,70,1],[3940,20,1],[3940,57,1],[3941,53,2],
[3941,55,1],[3942,10,1],[3942,28,1],[3942,34,1],[3943,30,1],
[3943,53,1],[3943,54,1],[3943,77,1],[3944,17,1],[3944,22,1],
[3945,7,1],[3945,15,2],[3945,53,2],[3946,1,1],[3946,8,1],[3946,9,1],
[3946,16,1],[3946,19,1],[3946,30,1],[3946,31,1],[3946,34,1],
[3946,49,1],[3946,65,2],[3947,27,1],[3947,51,2],[3948,5,2],[3948,7,1],
[3948,21,2],[3948,24,1],[3948,26,1],[3948,27,1],[3948,28,1],
[3948,31,1],[3948,34,2],[3948,39,1],[3948,42,2],[3948,43,1],
[3949,13,2],[3950,6,1],[3950,27,2],[3951,17,1],[3951,53,3],
[3952,15,1],[3952,17,1],[3952,28,1],[3952,55,2],[3953,2,1],[3953,3,1],
[3953,16,1],[3953,21,5],[3953,22,1],[3953,23,5],[3953,26,5],
[3953,27,11],[3953,28,2],[3953,31,3],[3953,32,2],[3953,35,1],
[3953,38,1],[3953,40,2],[3953,42,4],[3953,43,5],[3953,47,1],
[3953,60,1],[3953,63,1],[3953,68,1],[3953,70,2],[3953,72,1],
[3954,9,1],[3954,10,1],[3954,12,1],[3954,13,1],[3954,19,3],

[3954,20,1],[3954,29,1],[3954,37,1],[3954,39,2],[3954,45,1],
[3954,57,1],[3954,58,1],[3954,65,1],[3954,73,1],[3955,2,2],
[3956,26,1],[3956,34,1],[3957,43,2],[3958,13,1],[3958,20,2],
[3958,57,1],[3959,56,1],[3959,72,1],[3960,25,4],[3961,2,1],[3961,3,1],
[3961,23,3],[3961,26,1],[3961,47,1],[3961,62,1],[3962,23,1],
[3962,25,6],[3962,32,4],[3962,51,2],[3962,78,1],[3963,28,1],
[3963,47,3],[3964,15,2],[3964,24,1],[3964,46,1],[3964,55,1],
[3965,17,5],[3965,19,1],[3965,53,4],[3966,50,1],[3966,66,1],
[3967,2,1],[3967,9,1],[3967,33,1],[3967,35,3],[3967,50,1],[3968,25,1],
[3968,43,1],[3969,0,1],[3969,9,1],[3969,10,5],[3969,11,3],[3969,12,1],
[3969,36,1],[3969,38,2],[3969,45,2],[3969,57,1],[3969,65,2],
[3969,67,1],[3969,73,1],[3969,77,1],[3970,2,1],[3970,35,1],[3971,0,1],
[3971,24,1],[3971,27,1],[3971,31,1],[3971,39,2],[3971,44,1],
[3972,6,1],[3972,28,1],[3972,31,1],[3972,33,1],[3973,5,1],[3973,20,1],
[3973,38,1],[3973,51,1],[3973,70,1],[3973,74,1],[3973,76,1],
[3974,12,1],[3974,34,1],[3975,2,15],[3975,10,1],[3975,20,1],
[3975,22,1],[3975,27,3],[3975,31,4],[3975,39,1],[3975,44,1],
[3975,69,1],[3976,39,1],[3976,48,3],[3977,4,1],[3977,15,1],
[3978,20,2],[3978,24,1],[3978,55,1],[3979,3,4],[3979,24,1],
[3979,25,1],[3979,31,1],[3979,32,2],[3979,43,1],[3979,44,1],
[3979,47,1],[3979,56,1],[3979,63,1],[3980,8,1],[3980,9,1],[3980,13,1],
[3980,16,1],[3980,19,1],[3980,41,1],[3980,54,1],[3980,55,1],
[3980,57,1],[3980,65,1],[3980,67,1],[3980,74,1],[3981,15,2],
[3981,55,1],[3981,56,1],[3982,4,1],[3982,22,1],[3982,23,7],
[3982,24,1],[3982,27,4],[3982,32,1],[3982,34,1],[3982,35,1],
[3982,44,1],[3982,50,1],[3982,66,1],[3983,26,1],[3983,27,1],
[3983,42,2],[3984,1,2],[3984,5,1],[3984,20,1],[3984,23,2],[3984,24,2],
[3984,34,2],[3984,43,1],[3984,56,1],[3985,28,1],[3985,56,1],
[3986,1,2],[3986,39,2],[3987,10,1],[3987,21,1],[3987,24,1],
[3987,27,1],[3987,33,1],[3987,43,1],[3988,12,1],[3988,26,1],
[3988,27,1],[3988,43,2],[3989,3,1],[3989,5,3],[3989,17,2],[3989,24,1],
[3989,25,2],[3989,26,2],[3989,28,9],[3989,30,1],[3989,33,2],
[3989,42,1],[3989,44,2],[3989,51,1],[3989,55,1],[3989,62,1],
[3989,74,1],[3989,75,1],[3990,28,1],[3990,50,1],[3991,21,1],
[3991,28,2],[3991,35,1],[3991,39,1],[3991,42,1],[3992,15,1],
[3992,32,1],[3992,55,1],[3993,1,1],[3993,2,1],[3993,3,3],[3993,4,220],
[3993,5,9],[3993,6,2],[3993,8,2],[3993,9,3],[3993,10,3],[3993,11,2],
[3993,12,3],[3993,13,2],[3993,14,1],[3993,15,2],[3993,16,2],
[3993,17,3],[3993,18,1],[3993,19,6],[3993,20,2],[3993,21,1],
[3993,22,6],[3993,23,1],[3993,24,3],[3993,25,6],[3993,26,2],
[3993,27,6],[3993,28,12],[3993,30,1],[3993,32,6],[3993,33,2],
[3993,34,1],[3993,37,1],[3993,38,85],[3993,39,1],[3993,40,3],
[3993,41,6],[3993,42,3],[3993,43,2],[3993,44,55],[3993,45,5],
[3993,46,4],[3993,47,1],[3993,48,1],[3993,49,3],[3993,50,221],
[3993,52,1],[3993,53,3],[3993,55,5],[3993,56,4],[3993,57,1],
[3993,58,2],[3993,59,4],[3993,60,1],[3993,62,1],[3993,63,2],
[3993,65,2],[3993,66,75],[3993,68,7],[3993,69,3],[3993,70,6],
[3993,71,1],[3993,72,5],[3993,73,1],[3993,74,3],[3993,75,6],
[3993,76,5],[3993,77,3],[3993,78,1],[3994,9,1],[3994,30,1],
[3995,25,1],[3995,27,2],[3995,31,2],[3995,32,1],[3995,35,1],

[3995,56,2],[3996,5,3],[3996,28,1],[3997,2,2],[3997,22,2],[3997,23,5],
[3997,24,1],[3997,26,3],[3997,27,4],[3997,30,2],[3997,31,7],
[3997,32,1],[3997,37,1],[3997,42,1],[3997,43,2],[3997,62,2],
[3998,30,1],[3998,57,1],[3999,13,1],[3999,23,1],[4000,4,1],
[4000,25,1],[4000,28,1],[4000,47,1],[4001,2,2],[4001,50,1],
[4002,25,1],[4002,56,1],[4002,60,1],[4003,27,1],[4003,28,2],
[4004,28,1],[4004,32,2],[4004,56,2],[4004,62,1],[4004,68,2],
[4004,69,1],[4004,70,1],[4004,72,1],[4005,2,2],[4005,3,2],[4005,5,3],
[4005,22,2],[4005,23,1],[4005,24,3],[4005,25,4],[4005,26,2],
[4005,27,5],[4005,28,5],[4005,31,5],[4005,32,5],[4005,33,6],
[4005,35,1],[4005,42,1],[4005,48,7],[4005,50,1],[4005,56,2],
[4005,61,1],[4005,62,5],[4005,63,1],[4005,68,1],[4005,69,4],
[4005,70,17],[4005,72,1],[4005,76,5],[4006,1,1],[4006,23,2],
[4006,28,1],[4006,51,3],[4006,69,3],[4006,70,5],[4007,15,1],
[4007,59,1],[4008,5,1],[4008,13,1],[4008,15,1],[4008,20,1],
[4008,24,1],[4008,30,6],[4008,35,1],[4008,40,3],[4008,47,1],
[4008,48,1],[4008,53,1],[4008,69,1],[4008,70,23],[4009,0,2],
[4009,1,3],[4009,7,2],[4009,8,2],[4009,9,15],[4009,10,20],[4009,11,3],
[4009,12,8],[4009,14,4],[4009,15,7],[4009,16,9],[4009,17,5],
[4009,18,6],[4009,19,19],[4009,20,4],[4009,29,1],[4009,30,5],
[4009,34,4],[4009,36,4],[4009,37,5],[4009,39,4],[4009,41,6],
[4009,45,10],[4009,46,5],[4009,49,6],[4009,53,3],[4009,54,4],
[4009,55,10],[4009,57,6],[4009,58,7],[4009,59,9],[4009,60,2],
[4009,64,2],[4009,65,16],[4009,67,8],[4009,71,6],[4009,73,5],
[4009,77,6],[4009,78,4],[4010,2,13],[4011,12,1],[4011,27,3],
[4012,22,1],[4012,39,1],[4012,43,1],[4012,56,1],[4013,15,1],
[4013,53,2],[4014,3,3],[4014,24,1],[4014,28,3],[4014,51,1],
[4014,56,2],[4015,20,10],[4015,28,1],[4015,30,1],[4015,42,1],
[4015,52,1],[4015,73,1],[4016,1,2],[4016,12,1],[4016,21,1],
[4016,30,1],[4016,34,1],[4016,39,1],[4017,2,4],[4018,38,1],
[4018,44,1],[4018,45,1],[4019,3,1],[4019,4,16],[4019,5,2],[4019,10,1],
[4019,12,1],[4019,22,2],[4019,23,2],[4019,27,2],[4019,28,1],
[4019,29,2],[4019,35,1],[4019,38,6],[4019,40,2],[4019,42,1],
[4019,43,1],[4019,44,4],[4019,50,20],[4019,54,3],[4019,56,2],
[4019,58,1],[4019,63,1],[4019,64,1],[4019,65,1],[4019,66,6],
[4019,68,2],[4019,70,2],[4019,76,1],[4020,8,1],[4020,30,1],
[4020,31,2],[4020,45,2],[4020,57,1],[4020,68,1],[4021,2,3],
[4021,23,1],[4021,24,1],[4021,32,1],[4021,35,2],[4022,2,1],
[4022,24,1],[4022,27,6],[4022,35,1],[4023,2,2],[4023,3,5],[4023,5,2],
[4023,6,3],[4023,9,1],[4023,15,2],[4023,16,1],[4023,17,20],
[4023,21,1],[4023,22,7],[4023,23,3],[4023,24,5],[4023,25,1],
[4023,26,3],[4023,27,4],[4023,32,3],[4023,33,1],[4023,34,1],
[4023,35,4],[4023,42,2],[4023,43,1],[4023,47,1],[4023,48,2],
[4023,53,20],[4023,55,1],[4023,56,1],[4023,58,2],[4023,67,1],
[4023,70,2],[4023,75,1],[4024,27,1],[4024,33,1],[4025,28,1],
[4025,48,1],[4026,2,1],[4026,3,1],[4026,5,2],[4026,27,1],[4027,15,2],
[4027,55,1],[4027,58,1],[4028,2,2],[4028,27,2],[4029,22,1],
[4029,74,1],[4030,23,2],[4030,27,3],[4030,33,1],[4031,0,1],[4031,1,4],
[4031,2,1],[4031,3,3],[4031,4,1],[4031,5,6],[4031,7,1],[4031,8,1],
[4031,9,9],[4031,10,13],[4031,11,2],[4031,12,438],[4031,13,3],

[4031,14,2],[4031,15,5],[4031,16,4],[4031,17,1],[4031,18,1],
[4031,19,15],[4031,20,7],[4031,21,2],[4031,22,1],[4031,23,2],
[4031,24,2],[4031,25,3],[4031,27,2],[4031,28,1],[4031,29,2],
[4031,30,5],[4031,32,2],[4031,33,1],[4031,35,1],[4031,36,2],
[4031,37,3],[4031,38,10],[4031,39,1],[4031,40,3],[4031,41,5],
[4031,42,2],[4031,44,1],[4031,45,5],[4031,46,4],[4031,47,2],
[4031,49,2],[4031,50,1],[4031,52,2],[4031,53,3],[4031,54,1],
[4031,55,2],[4031,57,7],[4031,58,4],[4031,59,3],[4031,62,6],
[4031,64,1],[4031,65,4],[4031,66,1],[4031,67,1],[4031,70,1],
[4031,71,1],[4031,73,1],[4031,74,6],[4031,75,1],[4031,76,1],
[4031,77,3],[4032,4,1],[4032,5,2],[4032,9,1],[4032,15,2],[4032,17,1],
[4032,23,2],[4032,31,1],[4032,32,1],[4032,35,1],[4032,38,1],
[4032,42,2],[4032,50,1],[4032,53,2],[4032,55,1],[4032,59,1],
[4032,65,1],[4032,69,1],[4032,70,1],[4032,76,1],[4032,77,1],
[4033,7,1],[4033,9,1],[4033,13,1],[4033,34,1],[4033,67,1],[4033,76,1],
[4034,23,1],[4034,27,1],[4035,23,3],[4035,26,1],[4035,42,1],
[4035,43,2],[4035,48,1],[4035,63,1],[4035,74,13],[4036,35,1],
[4036,70,1],[4037,62,1],[4037,68,1],[4038,2,1],[4038,31,1],
[4039,26,1],[4039,42,2],[4040,28,1],[4040,47,1],[4041,1,1],
[4041,27,1],[4042,33,1],[4042,44,1],[4042,70,1],[4043,1,2],[4043,9,2],
[4043,28,2],[4043,57,1],[4044,4,1],[4044,32,7],[4045,1,1],[4045,60,1],
[4046,28,2],[4047,1,2],[4047,5,1],[4047,7,1],[4047,8,1],[4047,17,1],
[4047,21,1],[4047,23,1],[4047,34,1],[4047,47,1],[4048,22,1],
[4048,26,2],[4048,63,1],[4049,32,15],[4050,21,1],[4050,32,2],
[4050,42,1],[4050,43,1],[4050,50,1],[4050,74,1],[4051,26,1],
[4051,31,1],[4052,28,1],[4052,50,1],[4053,4,1],[4053,28,1],[4054,1,1],
[4054,2,1],[4054,24,1],[4054,27,1],[4054,35,2],[4054,53,1],
[4055,12,1],[4055,15,2],[4055,53,1],[4055,55,3],[4056,1,1],[4056,2,3],
[4056,3,5],[4056,4,1],[4056,5,3],[4056,15,1],[4056,17,5],[4056,20,1],
[4056,21,2],[4056,22,8],[4056,23,17],[4056,24,31],[4056,25,13],
[4056,26,3],[4056,27,16],[4056,28,19],[4056,30,2],[4056,31,14],
[4056,32,8],[4056,33,1],[4056,35,4],[4056,38,1],[4056,39,6],
[4056,43,2],[4056,47,3],[4056,50,2],[4056,52,9],[4056,53,3],
[4056,56,1],[4056,62,11],[4057,32,2],[4058,2,3],[4058,3,1],[4058,4,1],
[4058,6,1],[4058,21,1],[4058,22,1],[4058,23,4],[4058,24,3],
[4058,25,1],[4058,27,6],[4058,28,11],[4058,31,1],[4058,32,1],
[4058,33,2],[4058,35,1],[4058,38,2],[4058,43,3],[4058,44,1],
[4058,50,2],[4058,56,1],[4058,62,1],[4058,63,2],[4058,68,2],
[4058,72,1],[4058,74,2],[4058,75,1],[4058,76,3],[4059,2,1],[4059,3,9],
[4059,5,4],[4059,6,6],[4059,21,3],[4059,22,10],[4059,23,7],
[4059,24,19],[4059,25,7],[4059,26,9],[4059,27,25],[4059,28,11],
[4059,31,5],[4059,32,9],[4059,33,8],[4059,35,13],[4059,40,1],
[4059,42,6],[4059,43,3],[4059,44,1],[4059,47,6],[4059,48,6],
[4059,52,1],[4059,56,1],[4059,61,4],[4059,66,1],[4059,75,1],
[4060,5,1],[4060,12,1],[4060,17,3],[4060,21,1],[4060,22,1],
[4060,23,1],[4060,24,1],[4060,27,1],[4060,31,2],[4060,32,2],
[4060,33,3],[4060,53,1],[4060,60,1],[4060,66,1],[4061,2,1],[4061,3,3],
[4061,4,42],[4061,5,13],[4061,17,1],[4061,22,5],[4061,23,2],
[4061,24,2],[4061,25,1],[4061,26,5],[4061,27,3],[4061,28,19],
[4061,31,2],[4061,32,3],[4061,33,3],[4061,35,6],[4061,38,34],

[4061,40,1],[4061,42,1],[4061,43,1],[4061,44,329],[4061,47,7],
[4061,48,1],[4061,50,66],[4061,52,1],[4061,56,3],[4061,62,1],
[4061,63,1],[4061,66,256],[4061,68,1],[4061,70,2],[4061,72,1],
[4061,74,3],[4061,75,3],[4061,76,1],[4061,79,1],[4062,43,1],
[4062,63,1],[4063,10,1],[4063,33,1],[4064,1,29],[4064,2,3],[4064,3,8],
[4064,5,17],[4064,6,1],[4064,9,1],[4064,13,20],[4064,15,5],
[4064,17,1],[4064,19,3],[4064,20,4],[4064,21,4],[4064,22,1],
[4064,23,34],[4064,24,5],[4064,25,2],[4064,27,2],[4064,28,3],
[4064,30,4],[4064,33,5],[4064,34,11],[4064,35,6],[4064,37,2],
[4064,39,7],[4064,40,1],[4064,41,3],[4064,42,5],[4064,43,2],
[4064,44,4],[4064,46,1],[4064,47,16],[4064,48,2],[4064,50,2],
[4064,52,1],[4064,54,1],[4064,56,14],[4064,57,1],[4064,58,2],
[4064,59,3],[4064,60,1],[4064,64,1],[4064,65,1],[4064,66,1],
[4064,67,2],[4064,70,5],[4064,71,1],[4064,72,3],[4064,75,1],
[4064,77,2],[4064,78,3],[4065,2,9],[4065,3,1],[4065,48,1],[4065,63,1],
[4065,74,1],[4066,5,1],[4066,31,1],[4066,38,2],[4066,40,1],
[4066,44,21],[4066,50,1],[4066,66,12],[4067,1,1],[4067,2,1],
[4067,33,1],[4068,11,2],[4068,16,1],[4068,23,1],[4068,35,1],
[4068,64,4],[4069,23,1],[4069,35,1],[4070,3,1],[4070,24,1],
[4070,43,2],[4071,25,1],[4071,52,1],[4071,68,1],[4071,75,3],
[4072,11,1],[4072,19,1],[4072,35,2],[4072,73,1],[4073,28,2],
[4074,2,1],[4074,22,1],[4075,3,1],[4075,5,1],[4075,21,1],[4075,22,3],
[4075,23,1],[4075,24,1],[4075,25,2],[4075,26,6],[4075,27,4],
[4075,28,2],[4075,30,1],[4075,31,5],[4075,35,2],[4075,39,1],
[4075,42,6],[4075,43,2],[4075,47,2],[4076,4,1],[4076,12,1],
[4076,51,1],[4076,53,1],[4077,4,1],[4077,38,1],[4078,2,2],[4079,13,1],
[4079,23,1],[4080,22,1],[4080,24,1],[4080,26,2],[4080,51,3],
[4080,62,1],[4080,68,3],[4080,69,1],[4081,6,1],[4081,28,1],
[4082,23,2],[4082,25,1],[4082,26,5],[4082,28,1],[4082,31,1],
[4082,35,6],[4082,42,1],[4082,43,1],[4083,24,2],[4083,25,1],
[4083,27,1],[4083,28,1],[4084,55,2],[4085,15,3],[4085,55,1],
[4086,44,1],[4086,50,1],[4086,62,1],[4087,26,1],[4087,33,3],
[4087,43,1],[4087,60,1],[4088,12,4],[4089,6,1],[4089,28,2],
[4089,38,1],[4089,56,1],[4090,5,1],[4090,24,1],[4091,3,1],[4091,23,1],
[4091,27,1],[4091,31,1],[4091,32,3],[4091,35,1],[4092,23,4],
[4092,42,9],[4092,43,2],[4093,28,1],[4093,43,1],[4094,27,1],
[4094,28,3],[4094,32,1],[4094,42,1],[4094,47,1],[4094,50,1],
[4095,23,21],[4095,24,1],[4095,28,1],[4095,33,1],[4095,40,1],
[4095,50,1],[4095,70,1],[4096,23,23],[4096,26,6],[4096,28,1],
[4096,30,3],[4096,32,1],[4096,40,1],[4096,42,1],[4096,46,1],
[4096,48,1],[4096,69,1],[4097,24,1],[4097,44,1],[4098,0,1],
[4098,45,1],[4099,27,2],[4100,44,1],[4100,66,1],[4101,7,1],
[4101,30,1],[4101,65,1],[4102,6,1],[4102,24,1],[4102,27,1],
[4102,28,1],[4103,0,1],[4103,1,6],[4103,5,2],[4103,6,1],[4103,8,1],
[4103,9,2],[4103,10,1],[4103,12,7],[4103,13,5],[4103,14,1],
[4103,15,53],[4103,17,3],[4103,20,6],[4103,25,1],[4103,28,2],
[4103,31,1],[4103,32,2],[4103,33,2],[4103,34,1],[4103,35,1],
[4103,37,8],[4103,38,1],[4103,39,3],[4103,41,2],[4103,42,3],
[4103,45,2],[4103,53,1],[4103,54,1],[4103,55,28],[4103,58,3],
[4103,59,2],[4103,66,1],[4103,70,1],[4103,78,1],[4104,1,3],[4104,8,1],

[4104,9,1],[4104,13,2],[4104,14,1],[4104,15,1],[4104,16,2],
[4104,17,1],[4104,18,1],[4104,19,2],[4104,20,1],[4104,34,85],
[4104,37,1],[4104,39,1],[4104,45,1],[4104,53,1],[4104,55,1],
[4104,58,1],[4104,59,1],[4104,65,2],[4104,68,1],[4104,71,1],
[4104,72,1],[4105,27,1],[4105,72,1],[4106,23,1],[4106,32,3],
[4107,26,1],[4107,27,1],[4107,32,1],[4107,35,1],[4107,72,1],
[4108,15,1],[4108,17,1],[4108,53,1],[4108,55,1],[4109,20,1],
[4109,27,1],[4110,2,1],[4110,6,2],[4110,28,2],[4110,32,1],[4110,42,1],
[4110,47,1],[4110,74,1],[4111,21,1],[4111,22,1],[4111,24,1],
[4111,25,1],[4111,26,2],[4111,28,3],[4111,32,1],[4111,37,1],
[4111,39,1],[4111,40,2],[4111,48,2],[4111,56,2],[4111,60,1],
[4111,64,1],[4111,70,2],[4111,78,2],[4112,17,1],[4112,39,2],
[4112,55,2],[4113,4,1],[4113,66,1],[4114,2,1],[4114,3,3],[4114,4,1],
[4114,5,3],[4114,9,1],[4114,21,1],[4114,23,7],[4114,24,2],[4114,26,3],
[4114,27,3],[4114,35,2],[4114,37,1],[4114,40,1],[4114,42,2],
[4114,46,1],[4114,51,2],[4114,56,1],[4114,66,2],[4114,68,1],
[4115,40,1],[4115,70,1],[4116,3,1],[4116,31,9],[4117,33,5],
[4118,25,2],[4119,10,1],[4119,19,1],[4119,25,1],[4119,38,1],
[4120,15,1],[4120,20,1],[4121,12,1],[4121,20,1],[4121,35,1],
[4122,15,1],[4122,46,1],[4122,64,3],[4122,78,1],[4123,31,9],
[4123,32,1],[4124,1,2],[4125,56,1],[4125,69,1],[4126,17,1],
[4126,21,2],[4126,42,1],[4127,4,1],[4127,44,1],[4128,15,5],
[4128,55,1],[4129,27,2],[4129,31,1],[4129,35,1],[4129,69,1],
[4130,6,1],[4130,74,1],[4131,1,1],[4131,2,2],[4131,3,3],[4131,5,1],
[4131,6,2],[4131,10,1],[4131,13,1],[4131,19,1],[4131,20,10],
[4131,21,2],[4131,22,1],[4131,23,6],[4131,24,2],[4131,25,2],
[4131,26,4],[4131,27,18],[4131,30,3],[4131,33,6],[4131,34,3],
[4131,35,8],[4131,39,1],[4131,42,3],[4131,43,2],[4131,44,1],
[4131,45,1],[4131,46,1],[4131,47,2],[4131,50,1],[4131,59,1],
[4131,62,1],[4131,66,2],[4131,67,1],[4131,68,1],[4131,70,5],
[4131,74,1],[4131,76,2],[4132,2,1],[4132,20,1],[4132,47,1],
[4133,15,2],[4133,16,1],[4133,55,1],[4133,78,1],[4134,55,1],
[4134,70,1],[4135,4,11],[4135,9,1],[4135,10,2],[4135,15,3],
[4135,25,2],[4135,28,36],[4135,38,1],[4135,44,24],[4135,45,1],
[4135,46,1],[4135,48,1],[4135,50,18],[4135,53,1],[4135,55,2],
[4135,65,1],[4135,66,22],[4135,73,1],[4135,75,1],[4135,76,1],
[4136,2,1],[4136,4,1],[4136,5,1],[4136,6,2],[4136,8,1],[4136,10,2],
[4136,14,1],[4136,15,2],[4136,16,1],[4136,17,5],[4136,19,2],
[4136,23,2],[4136,26,1],[4136,27,3],[4136,28,38],[4136,31,1],
[4136,32,1],[4136,34,1],[4136,36,1],[4136,40,4],[4136,43,1],
[4136,44,2],[4136,46,1],[4136,47,1],[4136,50,1],[4136,51,1],
[4136,52,1],[4136,53,4],[4136,55,3],[4136,56,1],[4136,57,1],
[4136,58,2],[4136,59,2],[4136,62,1],[4136,63,2],[4136,65,1],
[4136,68,3],[4136,69,2],[4136,70,10],[4136,71,1],[4136,72,1],
[4136,73,2],[4136,75,2],[4136,76,1],[4136,77,1],[4137,39,1],
[4137,47,1],[4138,27,2],[4139,23,1],[4139,26,1],[4139,27,1],
[4140,1,2],[4141,10,1],[4141,30,1],[4142,23,1],[4142,24,1],
[4142,25,9],[4142,27,2],[4142,32,2],[4142,33,1],[4142,62,1],
[4143,25,1],[4143,28,1],[4143,51,8],[4143,56,1],[4144,10,1],
[4144,21,4],[4144,22,16],[4144,23,9],[4144,26,1],[4144,27,1],

[4144,31,1],[4144,32,1],[4144,35,1],[4145,33,2],[4145,38,1],
[4145,50,1],[4146,32,1],[4146,56,1],[4147,0,1],[4147,2,2],[4147,8,18],
[4147,24,1],[4147,31,2],[4147,34,1],[4147,55,1],[4147,58,1],
[4147,59,1],[4147,73,1],[4148,20,1],[4148,73,1],[4149,8,1],
[4149,25,1],[4150,5,1],[4150,63,1],[4151,11,1],[4151,45,1],
[4151,64,2],[4152,21,1],[4152,28,1],[4152,56,1],[4153,27,1],
[4153,28,1],[4153,35,1],[4154,28,2],[4155,0,1],[4155,1,1],[4155,2,3],
[4155,12,2],[4155,13,1],[4155,19,1],[4155,20,2],[4155,23,2],
[4155,25,1],[4155,27,2],[4155,36,1],[4155,42,1],[4155,59,3],
[4155,60,1],[4155,62,1],[4155,69,1],[4156,22,1],[4156,35,2],
[4157,26,1],[4157,32,1],[4158,53,2],[4158,55,1],[4159,8,1],
[4159,19,1],[4159,39,1],[4159,45,1],[4159,65,1],[4160,1,1],
[4160,13,2],[4160,55,1],[4161,0,1],[4161,1,1],[4161,12,1],[4161,15,4],
[4161,17,1],[4161,19,1],[4161,20,1],[4161,29,1],[4161,45,1],
[4161,53,1],[4161,55,2],[4161,59,1],[4162,25,1],[4162,26,2],
[4162,31,1],[4162,42,1],[4162,43,3],[4163,20,1],[4163,27,2],
[4164,75,1],[4164,76,1],[4165,1,4],[4165,13,1],[4165,24,1],
[4165,28,1],[4165,40,1],[4165,55,1],[4165,56,2],[4165,62,1],
[4166,6,1],[4166,22,1],[4166,25,62],[4166,27,1],[4166,32,1],
[4166,33,1],[4166,35,8],[4166,44,1],[4166,48,1],[4166,63,1],
[4166,70,1],[4166,72,1],[4167,28,1],[4167,70,1],[4168,24,1],
[4168,26,1],[4168,28,1],[4169,28,2],[4169,29,1],[4169,70,1],
[4170,44,1],[4170,66,1],[4171,28,1],[4171,60,1],[4172,57,1],
[4172,65,1],[4173,19,1],[4173,58,1],[4174,1,3],[4174,2,3],[4174,3,2],
[4174,5,1],[4174,6,2],[4174,9,2],[4174,10,1],[4174,11,1],[4174,13,5],
[4174,15,1],[4174,17,1],[4174,19,1],[4174,20,20],[4174,21,7],
[4174,22,3],[4174,23,10],[4174,26,2],[4174,27,2],[4174,28,1],
[4174,32,2],[4174,33,3],[4174,34,1],[4174,35,14],[4174,39,2],
[4174,40,1],[4174,42,1],[4174,45,1],[4174,46,1],[4174,47,6],
[4174,55,1],[4174,56,1],[4174,59,4],[4174,65,1],[4174,71,1],
[4175,9,1],[4175,10,1],[4175,15,1],[4175,16,1],[4175,39,1],
[4175,55,1],[4176,1,1],[4176,16,1],[4176,49,1],[4176,55,1],
[4176,65,1],[4177,33,2],[4178,5,1],[4178,21,1],[4179,30,6],[4180,2,1],
[4180,3,1],[4180,35,1],[4180,37,2],[4181,35,2],[4182,74,1],
[4182,75,1],[4182,76,1],[4183,38,2],[4184,28,2],[4184,50,1],
[4184,56,1],[4185,1,2],[4185,32,1],[4186,4,1],[4186,23,1],[4186,28,5],
[4186,38,2],[4186,44,6],[4186,50,1],[4186,66,4],[4187,2,6],[4187,3,1],
[4187,5,3],[4187,10,1],[4187,20,5],[4187,22,1],[4187,23,2],
[4187,24,1],[4187,27,1],[4187,30,1],[4187,31,1],[4187,34,2],
[4187,35,1],[4187,42,1],[4187,53,1],[4187,60,2],[4187,64,2],
[4187,65,1],[4187,69,1],[4187,70,1],[4188,21,1],[4188,22,5],
[4188,26,2],[4188,27,3],[4188,35,1],[4188,62,1],[4188,66,1],
[4189,2,1],[4189,26,1],[4190,3,1],[4190,22,1],[4190,26,1],[4190,31,1],
[4190,38,2],[4190,42,1],[4190,46,1],[4190,47,2],[4190,48,6],
[4190,50,1],[4190,63,1],[4191,8,1],[4191,22,1],[4191,23,3],
[4191,24,1],[4191,26,1],[4191,33,1],[4191,35,1],[4191,38,1],
[4191,39,1],[4191,42,2],[4191,52,1],[4191,74,1],[4192,2,3],
[4192,26,1],[4192,35,1],[4193,24,1],[4193,28,1],[4194,7,1],
[4194,29,1],[4195,24,1],[4195,27,1],[4195,41,1],[4195,56,1],
[4196,2,4],[4197,2,1],[4197,21,1],[4198,23,2],[4198,24,1],[4198,25,1],

[4198,26,1],[4198,27,1],[4198,31,1],[4198,32,1],[4198,56,2],
[4199,27,1],[4199,35,1],[4200,2,2],[4200,6,1],[4200,24,1],[4200,32,1],
[4200,43,1],[4201,27,1],[4201,33,1],[4201,70,1],[4202,5,1],
[4202,10,1],[4202,21,1],[4202,28,1],[4202,47,2],[4203,4,4],[4203,5,1],
[4203,17,4],[4203,44,1],[4203,50,6],[4203,66,3],[4204,35,1],
[4204,76,1],[4205,9,1],[4205,13,2],[4205,20,2],[4205,24,3],
[4205,30,1],[4205,42,1],[4205,45,1],[4205,75,1],[4206,28,1],
[4206,40,1],[4206,63,1],[4206,76,1],[4207,25,1],[4207,27,2],
[4207,28,1],[4208,4,1],[4208,35,1],[4209,9,1],[4209,21,5],[4209,22,4],
[4209,26,1],[4209,27,1],[4209,28,1],[4209,47,1],[4210,21,5],
[4210,27,2],[4210,35,1],[4211,26,2],[4211,43,2],[4212,3,1],[4212,5,2],
[4212,8,2],[4212,20,1],[4212,21,2],[4212,22,3],[4212,23,7],
[4212,24,8],[4212,25,4],[4212,26,13],[4212,27,3],[4212,28,4],
[4212,30,1],[4212,31,3],[4212,32,3],[4212,33,3],[4212,35,5],
[4212,39,1],[4212,42,5],[4212,43,24],[4212,44,1],[4212,47,2],
[4212,58,1],[4212,74,1],[4213,2,2],[4213,28,1],[4214,0,1],[4214,1,1],
[4214,7,2],[4214,9,4],[4214,10,2],[4214,12,7],[4214,13,1],[4214,14,1],
[4214,15,1],[4214,17,1],[4214,18,2],[4214,20,3],[4214,24,1],
[4214,28,7],[4214,29,2],[4214,31,1],[4214,35,1],[4214,37,1],
[4214,39,1],[4214,42,1],[4214,44,1],[4214,45,2],[4214,49,1],
[4214,50,1],[4214,53,2],[4214,55,1],[4214,59,2],[4214,62,1],
[4214,63,1],[4214,64,1],[4214,65,1],[4214,70,1],[4214,73,3],
[4214,77,1],[4215,2,9],[4215,17,1],[4215,31,1],[4216,28,2],
[4217,23,1],[4217,32,1],[4217,38,1],[4218,28,3],[4219,12,2],
[4220,26,1],[4220,28,1],[4221,33,4],[4222,23,1],[4222,26,1],
[4222,47,2],[4223,5,1],[4223,16,1],[4223,20,1],[4223,24,2],
[4223,27,1],[4223,32,1],[4223,34,2],[4223,59,1],[4224,21,1],
[4224,24,1],[4224,25,6],[4224,26,3],[4224,31,2],[4224,52,1],
[4225,13,1],[4225,39,2],[4226,1,1],[4226,19,1],[4227,3,1],[4227,28,2],
[4227,31,1],[4227,32,1],[4227,33,1],[4227,43,4],[4228,12,1],
[4228,72,1],[4229,9,2],[4229,10,4],[4229,11,2],[4229,12,1],
[4229,13,2],[4229,15,1],[4229,17,1],[4229,19,1],[4229,27,5],
[4229,28,1],[4229,34,1],[4229,35,1],[4229,36,2],[4229,37,1],
[4229,40,1],[4229,43,1],[4229,45,2],[4229,49,1],[4229,54,1],
[4229,56,1],[4229,57,1],[4229,58,1],[4229,61,1],[4229,62,1],
[4229,65,2],[4229,67,1],[4229,73,2],[4230,1,2],[4230,3,2],[4230,7,4],
[4230,8,1],[4230,11,1],[4230,17,1],[4230,20,2],[4230,23,1],
[4230,25,1],[4230,37,1],[4230,42,2],[4230,47,2],[4230,50,1],
[4230,51,1],[4230,56,2],[4230,64,1],[4230,72,1],[4230,74,1],
[4230,76,1],[4231,28,2],[4231,63,1],[4232,6,3],[4232,66,1],
[4233,33,2],[4233,74,1],[4234,28,23],[4234,75,1],[4235,25,1],
[4235,30,1],[4236,9,1],[4236,23,1],[4236,39,1],[4236,62,1],[4237,2,4],
[4237,5,1],[4237,8,2],[4237,22,2],[4237,23,1],[4237,27,2],[4237,30,1],
[4237,31,1],[4237,33,1],[4237,37,1],[4237,39,1],[4237,42,1],
[4237,47,1],[4238,25,1],[4238,63,1],[4239,2,2],[4239,24,1],
[4239,35,1],[4239,47,1],[4239,50,1],[4239,70,1],[4239,72,1],
[4239,75,1],[4240,66,2],[4241,28,5],[4242,6,1],[4242,23,2],
[4242,25,8],[4242,26,1],[4242,27,1],[4242,31,1],[4242,32,1],
[4242,35,1],[4242,42,1],[4242,43,5],[4242,48,1],[4242,66,1],
[4242,77,1],[4242,79,1],[4243,59,1],[4243,64,1],[4244,1,5],

[4244,25,7],[4244,41,1],[4244,77,1],[4245,1,2],[4246,12,4],
[4246,54,1],[4247,8,2],[4247,22,1],[4247,24,1],[4247,25,1],
[4247,28,4],[4247,30,10],[4247,33,1],[4247,35,5],[4247,38,1],
[4247,52,3],[4248,1,5],[4248,3,21],[4248,5,2],[4248,8,4],[4248,9,1],
[4248,10,9],[4248,11,2],[4248,12,4],[4248,13,4],[4248,14,2],
[4248,15,10],[4248,16,2],[4248,17,1],[4248,18,1],[4248,19,3],
[4248,20,3],[4248,21,1],[4248,22,16],[4248,23,1],[4248,24,1],
[4248,27,1],[4248,28,2],[4248,29,3],[4248,30,1],[4248,31,1],
[4248,34,1],[4248,37,1],[4248,39,2],[4248,41,2],[4248,45,2],
[4248,46,2],[4248,49,2],[4248,52,12],[4248,53,5],[4248,55,6],
[4248,57,4],[4248,59,1],[4248,60,2],[4248,65,7],[4248,66,1],
[4248,67,1],[4248,71,1],[4248,73,2],[4248,75,1],[4248,76,3],
[4249,26,2],[4249,43,1],[4250,0,1],[4250,4,2],[4250,6,1],[4250,10,1],
[4250,12,1],[4250,23,2],[4250,24,8],[4250,25,2],[4250,26,1],
[4250,27,1],[4250,28,23],[4250,32,2],[4250,33,1],[4250,40,1],
[4250,43,1],[4250,44,3],[4250,47,1],[4250,50,1],[4250,56,1],
[4250,63,3],[4250,66,2],[4250,68,1],[4250,72,1],[4250,74,1],
[4251,17,1],[4251,53,2],[4252,24,3],[4252,28,1],[4253,1,1],[4253,3,2],
[4253,26,1],[4253,62,1],[4254,25,1],[4254,35,3],[4255,27,2],
[4255,33,1],[4255,35,1],[4256,4,1],[4256,28,1],[4257,1,1],[4257,13,1],
[4257,20,1],[4257,39,3],[4257,78,1],[4258,21,1],[4258,23,1],
[4258,24,3],[4258,28,1],[4258,56,1],[4259,1,1],[4259,6,1],[4259,9,1],
[4259,12,1],[4259,15,1],[4259,26,1],[4259,31,1],[4259,60,1],
[4259,65,1],[4259,67,1],[4259,70,1],[4260,23,1],[4260,24,1],
[4260,27,3],[4261,32,1],[4261,66,1],[4262,3,1],[4262,23,1],
[4262,27,1],[4262,35,1],[4262,51,1],[4263,23,2],[4263,31,1],
[4263,42,1],[4264,28,1],[4264,47,2],[4265,0,4],[4265,1,6],[4265,2,10],
[4265,3,14],[4265,5,10],[4265,6,4],[4265,7,3],[4265,8,4],[4265,9,10],
[4265,10,19],[4265,11,6],[4265,12,13],[4265,13,21],[4265,14,2],
[4265,15,1],[4265,16,9],[4265,17,4],[4265,18,11],[4265,19,8],
[4265,20,65],[4265,21,20],[4265,22,28],[4265,23,49],[4265,24,36],
[4265,25,11],[4265,26,22],[4265,27,78],[4265,28,30],[4265,29,4],
[4265,30,5],[4265,31,19],[4265,32,21],[4265,33,15],[4265,34,41],
[4265,35,35],[4265,36,7],[4265,37,5],[4265,38,7],[4265,39,14],
[4265,41,6],[4265,42,27],[4265,43,31],[4265,44,2],[4265,45,16],
[4265,46,2],[4265,47,3],[4265,49,2],[4265,50,1],[4265,51,1],
[4265,52,2],[4265,53,2],[4265,54,4],[4265,55,2],[4265,57,8],
[4265,58,5],[4265,59,13],[4265,60,2],[4265,62,3],[4265,64,1],
[4265,65,14],[4265,67,4],[4265,69,1],[4265,70,2],[4265,71,5],
[4265,73,6],[4265,74,2],[4265,77,6],[4265,78,4],[4266,1,1],[4266,5,2],
[4266,9,2],[4266,11,1],[4266,16,1],[4266,18,2],[4266,20,2],
[4266,23,1],[4266,26,1],[4266,28,18],[4266,31,1],[4266,33,1],
[4266,38,2],[4266,39,1],[4266,44,29],[4266,50,4],[4266,53,1],
[4266,55,4],[4266,57,1],[4266,65,1],[4266,66,16],[4266,72,2],
[4266,74,1],[4267,1,1],[4267,2,2],[4267,3,2],[4267,5,2],[4267,6,8],
[4267,8,4],[4267,9,2],[4267,10,2],[4267,12,1],[4267,13,4],[4267,15,1],
[4267,17,1],[4267,20,56],[4267,21,35],[4267,22,56],[4267,23,22],
[4267,24,11],[4267,25,7],[4267,26,11],[4267,27,20],[4267,28,5],
[4267,31,10],[4267,32,6],[4267,33,6],[4267,34,3],[4267,35,29],
[4267,36,3],[4267,38,3],[4267,39,7],[4267,41,2],[4267,42,4],

[4267,43,10],[4267,44,1],[4267,47,3],[4267,48,3],[4267,53,1],
[4267,57,1],[4267,63,1],[4267,65,1],[4267,69,1],[4267,70,1],
[4267,71,1],[4267,77,1],[4268,2,3],[4268,8,1],[4268,13,1],[4268,23,2],
[4268,27,1],[4268,30,11],[4268,31,2],[4268,32,1],[4268,39,3],
[4268,42,3],[4268,48,1],[4268,53,1],[4268,56,1],[4269,28,1],
[4269,33,5],[4270,32,5],[4270,56,3],[4271,2,1],[4271,3,1],[4271,10,2],
[4271,17,1],[4271,23,1],[4271,42,1],[4271,62,1],[4272,3,3],
[4272,50,2],[4273,11,1],[4273,18,1],[4273,19,1],[4273,34,1],
[4273,56,17],[4273,58,1],[4273,60,1],[4274,2,1],[4274,22,2],
[4274,28,3],[4275,20,3],[4276,23,6],[4276,27,2],[4276,31,5],
[4277,68,2],[4278,24,1],[4278,47,2],[4278,76,1],[4279,2,1],
[4279,23,1],[4279,29,1],[4279,42,1],[4280,45,1],[4280,65,1],
[4281,27,2],[4282,1,2],[4283,27,3],[4284,2,1],[4284,32,2],[4284,35,4],
[4285,2,2],[4285,20,1],[4285,24,2],[4285,26,1],[4285,28,1],
[4285,29,1],[4286,22,4],[4286,25,1],[4286,28,4],[4286,32,4],
[4286,33,3],[4286,35,2],[4286,43,3],[4286,67,1],[4287,63,1],
[4287,76,1],[4288,35,1],[4288,47,1],[4288,70,1],[4289,3,1],
[4289,28,3],[4289,66,1],[4290,5,1],[4290,27,2],[4291,28,4],
[4291,43,1],[4292,26,1],[4292,27,1],[4292,28,1],[4292,31,1],
[4292,35,2],[4293,10,1],[4293,11,1],[4294,2,18],[4294,4,1],
[4294,13,2],[4294,20,1],[4294,30,1],[4294,60,1],[4294,64,1],
[4294,71,1],[4295,1,1],[4295,53,2],[4296,25,1],[4296,28,14],
[4297,31,2],[4298,6,1],[4298,20,1],[4298,24,1],[4298,27,1],
[4299,32,1],[4299,35,2],[4299,52,1],[4300,2,1],[4300,31,1],
[4300,47,1],[4300,70,1],[4301,32,2],[4302,0,1],[4302,1,1],[4302,9,1],
[4302,10,2],[4302,12,1],[4302,15,2],[4302,17,1],[4302,18,1],
[4302,22,1],[4302,27,2],[4302,32,3],[4302,33,1],[4302,41,1],
[4302,46,1],[4302,55,1],[4302,56,1],[4302,60,1],[4302,63,1],
[4302,73,1],[4302,75,1],[4302,76,4],[4303,24,2],[4304,3,6],[4304,6,2],
[4304,23,2],[4304,24,1],[4304,25,3],[4304,26,6],[4304,30,1],
[4304,38,1],[4304,47,5],[4304,60,1],[4305,5,2],[4305,7,1],[4305,21,1],
[4305,23,1],[4305,63,1],[4305,68,1],[4305,69,1],[4305,75,1],
[4305,76,1],[4306,1,1],[4306,2,1],[4306,12,1],[4306,13,3],[4306,15,1],
[4306,20,2],[4306,24,1],[4306,26,1],[4306,46,1],[4306,62,1],
[4306,64,1],[4306,65,1],[4307,1,1],[4307,37,1],[4307,44,1],
[4308,22,3],[4308,23,1],[4308,27,1],[4309,26,1],[4309,28,2],
[4309,32,2],[4309,35,1],[4310,1,1],[4310,53,1],[4311,23,1],
[4311,27,1],[4311,51,1],[4312,3,1],[4312,22,5],[4312,24,2],
[4312,25,1],[4312,26,1],[4312,30,1],[4312,31,2],[4312,32,1],
[4312,35,1],[4312,43,1],[4312,47,1],[4312,59,1],[4312,66,1],
[4312,70,1],[4313,28,33],[4313,38,1],[4313,44,5],[4313,66,8],
[4313,75,1],[4314,0,2],[4314,1,33],[4314,2,1],[4314,3,18],[4314,4,4],
[4314,5,20],[4314,6,30],[4314,7,2],[4314,8,7],[4314,9,3],[4314,10,5],
[4314,12,9],[4314,13,16],[4314,14,1],[4314,15,8],[4314,16,3],
[4314,17,3],[4314,19,5],[4314,20,97],[4314,21,9],[4314,22,43],
[4314,23,57],[4314,24,56],[4314,25,11],[4314,26,7],[4314,27,44],
[4314,28,17],[4314,29,2],[4314,30,3],[4314,31,7],[4314,32,11],
[4314,33,33],[4314,34,4],[4314,35,40],[4314,36,1],[4314,37,3],
[4314,38,5],[4314,39,4],[4314,40,5],[4314,41,1],[4314,42,13],
[4314,43,5],[4314,44,8],[4314,45,2],[4314,46,4],[4314,47,13],

[4314,50,9],[4314,51,8],[4314,52,2],[4314,53,3],[4314,55,5],
[4314,56,41],[4314,58,1],[4314,59,17],[4314,60,4],[4314,61,1],
[4314,62,8],[4314,63,2],[4314,64,1],[4314,65,1],[4314,66,6],
[4314,68,4],[4314,70,8],[4314,71,2],[4314,72,3],[4314,73,1],
[4314,74,109],[4314,75,3],[4314,76,9],[4314,77,7],[4315,2,2],
[4315,25,2],[4315,27,1],[4315,35,1],[4315,72,1],[4316,2,10],
[4316,23,1],[4316,27,7],[4316,30,1],[4316,31,8],[4316,39,1],
[4316,42,1],[4316,47,2],[4317,0,3],[4317,1,7],[4317,5,1],[4317,7,1],
[4317,8,3],[4317,9,14],[4317,10,7],[4317,12,8],[4317,13,2],
[4317,14,1],[4317,15,1],[4317,16,1],[4317,17,4],[4317,18,7],
[4317,19,4],[4317,20,4],[4317,22,1],[4317,23,3],[4317,27,2],
[4317,28,2],[4317,29,4],[4317,33,1],[4317,34,1],[4317,35,1],
[4317,36,1],[4317,37,1],[4317,38,1],[4317,39,2],[4317,40,1],
[4317,41,4],[4317,44,2],[4317,45,1],[4317,46,1],[4317,53,3],
[4317,55,6],[4317,57,6],[4317,58,3],[4317,59,3],[4317,60,1],
[4317,63,5],[4317,65,1],[4317,66,1],[4317,67,2],[4317,69,1],
[4317,70,1],[4317,71,1],[4317,73,3],[4317,74,2],[4317,75,7],
[4317,76,2],[4317,77,3],[4317,78,1],[4318,5,2],[4318,28,14],
[4319,32,2],[4320,17,2],[4321,3,5],[4321,22,1],[4321,56,2],
[4322,0,34],[4322,1,127],[4322,7,55],[4322,8,64],[4322,9,467],
[4322,10,588],[4322,11,212],[4322,12,174],[4322,13,118],[4322,14,144],
[4322,15,147],[4322,16,322],[4322,17,58],[4322,18,166],[4322,19,508],
[4322,20,135],[4322,29,42],[4322,30,32],[4322,34,99],[4322,36,124],
[4322,37,185],[4322,39,74],[4322,41,259],[4322,45,326],[4322,46,169],
[4322,49,151],[4322,53,93],[4322,54,161],[4322,55,104],[4322,57,222],
[4322,58,255],[4322,59,220],[4322,60,50],[4322,64,43],[4322,65,459],
[4322,67,233],[4322,71,170],[4322,73,168],[4322,77,249],[4322,78,50],
[4323,23,4],[4324,21,1],[4324,28,1],[4325,4,1],[4325,62,1],
[4325,72,1],[4326,31,6],[4327,27,3],[4327,28,1],[4327,31,8],
[4327,35,1],[4328,18,1],[4328,19,1],[4328,20,1],[4328,45,1],
[4328,46,1],[4328,57,1],[4329,3,2],[4330,2,2],[4331,0,1],[4331,1,7],
[4331,2,16],[4331,3,5],[4331,4,1],[4331,5,4],[4331,9,5],[4331,10,1],
[4331,11,1],[4331,12,2],[4331,13,2],[4331,15,1],[4331,17,2],
[4331,20,6],[4331,22,6],[4331,24,8],[4331,27,4],[4331,28,1],
[4331,31,5],[4331,32,3],[4331,33,3],[4331,34,1],[4331,35,1],
[4331,36,1],[4331,39,2],[4331,42,2],[4331,43,4],[4331,47,2],
[4331,50,1],[4331,53,2],[4331,54,2],[4331,58,1],[4331,59,2],
[4331,60,2],[4331,62,3],[4331,65,1],[4331,69,1],[4332,28,3],
[4333,3,3],[4333,24,3],[4333,35,3],[4334,3,1],[4334,22,2],[4334,24,2],
[4334,56,2],[4335,13,1],[4335,51,1],[4336,1,2],[4336,13,1],
[4336,39,3],[4337,21,1],[4337,26,2],[4338,30,1],[4338,78,1],
[4339,42,5],[4339,56,1],[4340,0,1],[4340,26,2],[4340,48,1],
[4341,21,2],[4342,24,1],[4342,28,2],[4342,32,1],[4342,54,1],
[4343,31,1],[4343,33,1],[4344,2,1],[4344,28,1],[4345,75,3],
[4345,76,1],[4346,1,3],[4346,4,1],[4346,5,27],[4346,13,2],[4346,21,5],
[4346,22,3],[4346,23,1],[4346,24,2],[4346,26,1],[4346,27,1],
[4346,28,3],[4346,31,1],[4346,33,1],[4346,35,1],[4346,43,1],
[4346,45,2],[4346,47,8],[4346,51,1],[4346,52,1],[4346,53,2],
[4346,55,1],[4346,63,1],[4346,79,1],[4347,4,2],[4347,8,1],[4347,23,1],
[4348,2,1],[4348,3,1],[4348,23,1],[4348,32,1],[4348,48,1],[4349,3,1],

[4349,28,1],[4349,38,1],[4350,10,1],[4350,15,1],[4351,20,2],
[4351,24,1],[4351,27,1],[4351,35,1],[4352,3,3],[4352,5,1],[4352,23,1],
[4352,25,2],[4352,27,3],[4352,28,1],[4352,37,1],[4352,48,1],
[4352,56,1],[4353,12,3],[4353,49,1],[4354,23,1],[4354,28,2],
[4355,13,1],[4355,29,1],[4355,77,1],[4356,15,1],[4356,59,1],
[4357,38,1],[4357,56,4],[4358,6,1],[4358,50,1],[4359,2,1],[4359,27,1],
[4359,77,1],[4360,13,2],[4360,24,2],[4360,26,1],[4360,31,1],
[4360,42,1],[4360,62,1],[4361,16,1],[4361,18,1],[4361,19,1],
[4361,37,1],[4361,41,1],[4361,45,1],[4361,55,1],[4361,65,1],
[4361,71,1],[4362,22,1],[4362,23,1],[4362,24,1],[4362,28,2],
[4362,47,1],[4362,56,1],[4362,66,1],[4362,76,1],[4363,28,2],
[4363,35,1],[4363,42,2],[4364,0,1],[4364,1,1],[4364,15,1],[4364,17,2],
[4364,20,1],[4364,53,6],[4364,55,1],[4365,3,1],[4365,23,2],
[4365,25,12],[4365,28,1],[4365,31,3],[4365,40,1],[4365,43,1],
[4365,44,1],[4366,22,1],[4366,27,8],[4366,32,1],[4366,42,2],
[4366,56,3],[4367,3,1],[4367,5,4],[4367,23,1],[4367,24,1],[4367,25,1],
[4367,28,2],[4367,43,1],[4367,59,2],[4367,70,1],[4368,32,2],
[4369,40,1],[4369,47,1],[4369,66,1],[4369,70,1],[4369,72,1],
[4370,1,1],[4370,15,1],[4371,2,1],[4371,3,1],[4371,6,1],[4371,23,3],
[4371,25,1],[4371,27,2],[4371,32,2],[4371,35,1],[4371,43,1],
[4372,28,1],[4372,53,1],[4373,2,1],[4373,5,1],[4373,20,1],[4373,22,1],
[4373,27,1],[4373,28,7],[4373,30,1],[4373,47,1],[4374,12,1],
[4374,29,1],[4375,12,1],[4375,13,1],[4375,15,1],[4375,17,1],
[4375,20,1],[4375,26,1],[4375,27,2],[4375,47,1],[4375,51,1],
[4375,53,1],[4375,62,2],[4375,73,1],[4376,22,1],[4376,24,1],
[4377,23,1],[4377,41,1],[4378,12,1],[4378,30,1],[4379,2,4],
[4379,28,2],[4379,62,17],[4379,66,1],[4380,27,1],[4380,32,4],
[4380,34,2],[4380,40,1],[4380,48,6],[4380,56,2],[4381,16,3],
[4382,4,10],[4382,17,3],[4382,22,1],[4382,28,2],[4382,50,19],
[4382,53,2],[4382,55,2],[4382,66,1],[4383,15,2],[4384,23,6],
[4384,25,1],[4384,31,1],[4384,33,2],[4384,48,1],[4384,63,1],
[4384,73,1],[4385,8,1],[4385,10,1],[4385,19,1],[4385,46,2],
[4385,58,1],[4385,65,2],[4385,71,1],[4386,3,1],[4386,4,1],[4387,28,4],
[4387,62,1],[4387,70,1],[4388,1,6],[4388,2,1],[4388,7,1],[4388,30,1],
[4388,31,1],[4389,24,1],[4389,28,1],[4389,47,1],[4389,50,1],
[4389,62,1],[4389,66,1],[4389,74,1],[4390,2,2],[4391,20,3],[4392,2,2],
[4392,28,2],[4393,4,14],[4393,50,12],[4394,5,3],[4394,20,1],
[4394,21,1],[4394,22,1],[4394,23,3],[4394,26,4],[4394,28,1],
[4394,32,1],[4394,33,3],[4394,35,1],[4394,42,1],[4394,43,1],
[4394,44,1],[4394,47,1],[4394,48,1],[4394,78,2],[4395,20,2],
[4395,29,1],[4395,67,1],[4396,2,1],[4396,50,1],[4397,1,1],[4397,10,1],
[4397,13,1],[4397,29,2],[4397,45,1],[4397,53,1],[4397,58,1],
[4397,64,1],[4398,23,4],[4399,2,1],[4399,16,1],[4399,28,4],
[4399,70,1],[4399,77,1],[4400,30,1],[4400,65,1],[4401,10,1],
[4401,17,1],[4402,5,3],[4402,24,2],[4402,25,4],[4402,26,1],
[4402,27,4],[4402,28,2],[4402,33,1],[4402,35,1],[4403,2,3],[4404,5,4],
[4404,35,1],[4405,25,1],[4405,28,1],[4406,2,2],[4406,5,1],[4406,23,1],
[4406,26,1],[4406,27,22],[4406,28,2],[4406,31,1],[4406,35,2],
[4406,47,1],[4406,55,1],[4406,62,1],[4407,28,1],[4407,35,6],
[4408,2,8],[4408,28,2],[4408,53,1],[4409,9,1],[4409,17,3],[4409,45,1],

[4409,53,2],[4409,55,1],[4410,1,1],[4410,7,1],[4410,8,1],[4410,9,3],
[4410,10,3],[4410,11,2],[4410,13,2],[4410,15,2],[4410,16,1],
[4410,19,2],[4410,20,4],[4410,36,1],[4410,37,2],[4410,39,1],
[4410,41,2],[4410,45,5],[4410,46,1],[4410,49,1],[4410,55,1],
[4410,57,2],[4410,58,1],[4410,59,1],[4410,60,1],[4410,64,2],
[4410,65,3],[4410,67,1],[4410,73,2],[4410,77,2],[4411,3,1],[4411,8,2],
[4411,24,1],[4411,26,2],[4411,27,1],[4411,48,1],[4411,74,1],
[4412,4,1],[4412,28,2],[4412,35,1],[4412,42,1],[4412,55,1],
[4413,12,1],[4413,13,1],[4413,23,11],[4414,4,10],[4414,17,1],
[4414,26,1],[4414,32,1],[4414,44,8],[4414,50,4],[4414,66,3],
[4414,68,1],[4414,70,1],[4414,75,1],[4415,24,1],[4415,35,1],
[4416,17,1],[4416,53,3],[4417,24,1],[4417,26,1],[4417,27,1],
[4417,31,1],[4417,56,1],[4418,28,2],[4419,9,1],[4419,10,2],
[4419,20,1],[4419,28,1],[4419,36,1],[4419,59,1],[4420,10,1],
[4420,13,1],[4420,21,1],[4420,50,1],[4420,56,1],[4421,1,1],
[4421,15,1],[4421,30,1],[4421,59,1],[4422,24,1],[4422,28,1],
[4422,33,1],[4423,28,1],[4423,44,1],[4424,9,1],[4424,12,1],
[4424,26,1],[4424,29,1],[4424,65,1],[4424,77,1],[4425,3,2],
[4425,28,4],[4425,44,1],[4425,66,1],[4426,44,5],[4426,66,5],
[4427,16,1],[4427,17,1],[4427,30,1],[4427,55,1],[4428,23,1],
[4428,76,1],[4429,62,3],[4430,3,1],[4430,5,1],[4430,20,1],[4430,23,2],
[4430,24,3],[4430,27,1],[4430,38,1],[4430,50,2],[4430,59,1],
[4430,64,1],[4431,4,1],[4431,27,1],[4432,2,1],[4432,13,1],[4432,23,1],
[4432,26,1],[4432,38,4],[4433,5,1],[4433,43,1],[4434,4,1],[4434,50,1],
[4435,0,1],[4435,1,1],[4435,7,1],[4435,9,1],[4435,10,1],[4435,11,3],
[4435,12,3],[4435,13,2],[4435,15,3],[4435,17,1],[4435,20,1],
[4435,30,1],[4435,33,2],[4435,34,2],[4435,37,2],[4435,39,1],
[4435,46,1],[4435,55,8],[4435,57,1],[4435,58,1],[4435,59,1],
[4435,65,2],[4435,67,1],[4435,71,1],[4435,73,1],[4435,77,2],
[4436,9,1],[4436,10,1],[4436,11,1],[4436,19,1],[4436,57,1],
[4436,58,1],[4437,15,2],[4437,17,1],[4438,50,1],[4438,56,10],
[4438,61,1],[4439,0,12],[4439,1,5],[4439,7,4],[4439,8,5],[4439,9,7],
[4439,10,10],[4439,11,6],[4439,12,11],[4439,13,1],[4439,14,1],
[4439,15,3],[4439,16,10],[4439,17,2],[4439,18,3],[4439,19,11],
[4439,20,3],[4439,28,5],[4439,29,8],[4439,30,4],[4439,31,13],
[4439,34,11],[4439,36,2],[4439,37,18],[4439,39,1],[4439,41,4],
[4439,45,1],[4439,46,1],[4439,49,1],[4439,53,1],[4439,54,1],
[4439,55,3],[4439,57,6],[4439,58,2],[4439,59,4],[4439,60,1],
[4439,64,1],[4439,65,9],[4439,67,4],[4439,71,4],[4439,73,13],
[4439,77,11],[4439,78,2],[4440,22,3],[4440,26,1],[4440,27,3],
[4440,39,1],[4440,43,1],[4440,56,1],[4441,25,1],[4441,27,1],
[4441,37,1],[4442,5,5],[4442,21,1],[4442,24,1],[4442,50,1],
[4442,51,1],[4443,3,1],[4443,4,1],[4444,22,2],[4444,26,1],[4444,47,1],
[4445,44,5],[4445,47,1],[4445,66,4],[4446,35,1],[4446,42,1],
[4447,39,3],[4448,24,1],[4448,27,1],[4448,32,3],[4448,56,1],
[4449,25,2],[4449,33,1],[4449,35,2],[4449,62,1],[4450,25,1],
[4450,38,3],[4450,52,13],[4451,5,1],[4451,38,1],[4451,44,1],
[4451,63,15],[4451,74,13],[4451,75,12],[4451,76,22],[4452,74,3],
[4453,46,1],[4453,60,1],[4454,74,2],[4455,3,1],[4455,47,2],
[4455,75,1],[4456,2,8],[4456,4,2],[4456,22,1],[4456,26,1],[4456,34,1],

[4456,43,1],[4456,53,3],[4457,5,2],[4457,20,2],[4457,21,2],
[4457,22,1],[4457,23,16],[4457,24,4],[4457,26,4],[4457,27,4],
[4457,28,2],[4457,32,4],[4457,33,1],[4457,35,14],[4457,43,3],
[4457,76,1],[4458,22,1],[4458,27,2],[4458,79,1],[4459,27,1],
[4459,28,1],[4460,44,1],[4460,66,1],[4461,0,1],[4461,46,1],
[4462,15,3],[4462,17,1],[4462,19,1],[4462,53,2],[4462,55,2],
[4463,1,1],[4463,12,3],[4463,15,1],[4463,41,1],[4464,63,2],
[4464,74,3],[4464,75,5],[4464,76,15],[4465,31,2],[4466,24,1],
[4466,31,1],[4467,22,1],[4467,23,3],[4467,31,1],[4468,3,7],[4468,4,1],
[4468,5,8],[4468,6,1],[4468,13,1],[4468,14,1],[4468,17,1],[4468,22,6],
[4468,23,31],[4468,24,62],[4468,25,3],[4468,27,5],[4468,28,10],
[4468,31,1],[4468,32,28],[4468,33,4],[4468,35,5],[4468,37,4],
[4468,38,2],[4468,40,1],[4468,42,1],[4468,43,2],[4468,44,2],
[4468,47,8],[4468,52,6],[4468,55,1],[4468,56,15],[4468,60,1],
[4468,63,1],[4468,66,1],[4468,70,1],[4468,71,1],[4468,74,1],
[4468,75,1],[4468,76,1],[4468,78,1],[4469,2,1],[4469,9,1],[4469,20,2],
[4469,46,1],[4470,10,1],[4470,67,1],[4471,12,1],[4471,29,1],
[4472,1,1],[4472,11,1],[4473,2,1],[4473,24,1],[4474,3,1],[4474,21,1],
[4474,25,1],[4474,42,1],[4474,68,1],[4474,76,1],[4475,25,1],
[4475,28,1],[4476,12,3],[4476,24,1],[4476,29,1],[4476,39,1],
[4477,23,1],[4477,31,1],[4478,5,1],[4478,7,1],[4478,9,2],[4478,10,1],
[4478,13,1],[4478,16,2],[4478,18,1],[4478,20,8],[4478,22,8],
[4478,23,8],[4478,24,18],[4478,26,3],[4478,27,1],[4478,28,2],
[4478,29,5],[4478,30,1],[4478,31,1],[4478,33,1],[4478,34,1],
[4478,37,3],[4478,38,1],[4478,39,2],[4478,40,2],[4478,42,1],
[4478,46,1],[4478,47,1],[4478,53,1],[4478,66,1],[4478,70,1],
[4478,72,1],[4478,77,1],[4478,78,2],[4479,2,1],[4479,5,1],[4479,6,1],
[4479,24,1],[4479,27,1],[4479,42,1],[4479,72,1],[4480,28,3],
[4481,28,2],[4482,31,2],[4483,23,1],[4483,26,1],[4483,43,1],
[4483,46,1],[4484,2,72],[4484,28,2],[4484,31,2],[4484,46,1],
[4484,50,2],[4484,70,3],[4484,72,7],[4485,9,1],[4485,11,1],
[4485,23,2],[4485,24,1],[4485,27,1],[4485,32,1],[4485,46,1],
[4485,53,1],[4485,54,1],[4485,56,1],[4485,64,1],[4485,65,1],
[4485,74,2],[4486,2,1],[4486,6,1],[4486,22,1],[4486,24,1],[4486,27,2],
[4486,28,1],[4486,33,1],[4486,34,1],[4486,35,1],[4487,24,1],
[4487,28,1],[4488,32,6],[4488,38,1],[4489,30,1],[4489,74,3],
[4490,8,1],[4490,22,4],[4490,23,1],[4490,27,1],[4490,28,1],
[4490,30,1],[4490,32,1],[4490,33,6],[4490,35,1],[4490,36,1],
[4490,43,10],[4490,45,1],[4490,47,1],[4491,3,1],[4491,25,3],
[4492,26,1],[4492,70,1],[4493,3,1],[4493,13,1],[4493,30,1],
[4493,31,1],[4493,42,1],[4493,48,1],[4494,27,1],[4494,28,2],
[4494,35,1],[4495,2,2],[4495,28,3],[4495,43,1],[4495,77,1],
[4496,31,12],[4496,47,1],[4497,2,1],[4497,28,1],[4498,6,4],
[4498,28,1],[4498,47,2],[4498,56,40],[4499,24,2],[4500,1,1],
[4500,10,1],[4500,17,1],[4500,18,1],[4500,19,1],[4500,39,1],
[4500,57,1],[4501,31,4],[4502,28,2],[4502,40,7],[4502,68,1],
[4502,69,3],[4502,70,4],[4502,72,3],[4503,3,1],[4503,43,1],
[4503,47,1],[4504,3,1],[4504,26,1],[4504,35,1],[4504,55,1],
[4505,43,1],[4505,75,3],[4505,76,1],[4506,20,1],[4506,23,2],
[4507,2,6],[4507,3,1],[4507,4,1],[4507,5,1],[4507,23,1],[4507,24,3],

[4507,26,3],[4507,27,3],[4507,35,1],[4507,38,1],[4507,39,2],
[4507,42,2],[4507,47,4],[4507,63,1],[4507,69,1],[4507,76,1],
[4508,23,3],[4508,24,1],[4508,37,1],[4509,3,1],[4509,12,1],
[4509,21,2],[4509,22,1],[4509,24,2],[4509,43,1],[4510,28,1],
[4510,44,1],[4511,2,1],[4511,3,1],[4511,5,1],[4511,22,1],[4511,38,1],
[4511,56,1],[4511,66,1],[4511,68,1],[4511,74,1],[4512,2,5],
[4512,64,2],[4513,28,2],[4513,59,1],[4514,5,1],[4514,31,1],
[4514,43,1],[4515,1,1],[4515,13,1],[4515,19,1],[4515,45,1],
[4515,65,1],[4515,71,1],[4515,73,1],[4516,22,1],[4516,23,1],
[4516,48,1],[4517,3,1],[4517,28,2],[4517,31,1],[4517,62,3],[4518,2,1],
[4518,28,1],[4519,2,1],[4519,42,1],[4520,1,1],[4520,10,1],[4520,15,9],
[4520,16,2],[4520,17,1],[4520,18,1],[4520,55,11],[4520,57,1],
[4521,15,1],[4521,55,1],[4522,3,1],[4522,13,1],[4522,15,1],
[4522,20,1],[4522,38,1],[4522,42,1],[4522,62,13],[4523,3,1],
[4523,4,6],[4523,5,2],[4523,6,1],[4523,9,1],[4523,17,2],[4523,19,1],
[4523,22,5],[4523,23,14],[4523,24,16],[4523,25,5],[4523,26,6],
[4523,27,12],[4523,28,6],[4523,31,1],[4523,32,42],[4523,33,4],
[4523,35,3],[4523,38,2],[4523,42,2],[4523,43,2],[4523,44,6],
[4523,47,4],[4523,48,6],[4523,50,3],[4523,53,3],[4523,56,7],
[4523,61,3],[4523,62,2],[4523,63,1],[4523,65,2],[4523,66,3],
[4523,70,3],[4523,71,1],[4523,72,1],[4524,2,1],[4524,20,1],
[4524,21,1],[4524,23,1],[4524,24,1],[4524,27,1],[4524,28,1],
[4524,31,2],[4524,62,1],[4525,23,1],[4525,35,1],[4526,4,3],
[4526,28,1],[4526,50,3],[4527,2,1],[4527,20,2],[4527,27,4],[4528,5,1],
[4528,56,1],[4529,10,1],[4529,12,1],[4529,18,1],[4529,34,2],
[4529,41,1],[4529,46,1],[4529,58,1],[4529,65,2],[4529,71,1],
[4530,32,1],[4530,42,1],[4531,21,2],[4531,24,1],[4531,28,2],
[4531,35,3],[4531,43,1],[4531,56,1],[4531,68,1],[4531,70,2],
[4531,75,1],[4531,76,1],[4532,31,1],[4532,46,1],[4533,15,1],
[4533,17,1],[4533,53,1],[4533,57,1],[4534,2,1],[4534,3,1],[4534,22,1],
[4534,27,1],[4534,28,2],[4534,32,2],[4534,55,1],[4534,68,1],
[4534,77,1],[4535,11,1],[4535,14,1],[4535,17,1],[4535,65,1],
[4536,17,2],[4537,28,6],[4538,2,3],[4538,24,1],[4538,27,2],
[4538,42,1],[4539,7,1],[4539,39,1],[4540,5,1],[4540,28,6],[4541,5,1],
[4541,15,3],[4541,17,1],[4541,55,4],[4541,62,1],[4541,74,1],
[4542,28,34],[4543,23,1],[4543,35,1],[4544,10,1],[4544,12,1],
[4544,36,1],[4544,59,1],[4544,66,1],[4545,36,1],[4545,54,1],
[4545,57,2],[4545,77,1],[4546,26,1],[4546,32,4],[4546,35,1],
[4547,1,2],[4547,2,9],[4547,3,7],[4547,4,2],[4547,5,9],[4547,6,6],
[4547,9,1],[4547,12,1],[4547,13,1],[4547,16,1],[4547,17,2],
[4547,20,16],[4547,21,7],[4547,22,10],[4547,23,13],[4547,24,11],
[4547,25,5],[4547,26,13],[4547,27,22],[4547,28,7],[4547,30,4],
[4547,31,1],[4547,32,5],[4547,33,7],[4547,34,1],[4547,35,16],
[4547,36,2],[4547,37,2],[4547,38,3],[4547,39,1],[4547,42,16],
[4547,43,10],[4547,45,2],[4547,46,1],[4547,50,2],[4547,51,6],
[4547,53,1],[4547,56,1],[4547,57,1],[4547,60,1],[4547,62,6],
[4547,66,1],[4547,67,1],[4547,68,2],[4548,6,4],[4548,24,1],
[4548,27,5],[4548,28,1],[4548,31,2],[4548,74,1],[4549,3,1],
[4549,44,1],[4550,28,1],[4550,29,1],[4551,4,1],[4551,21,1],
[4551,24,3],[4551,31,1],[4551,40,1],[4551,56,1],[4551,68,3],

[4551,69,1],[4551,70,4],[4551,72,1],[4552,32,8],[4552,42,4],
[4553,28,1],[4553,74,11],[4554,2,2],[4554,3,3],[4554,21,1],
[4554,22,1],[4554,25,1],[4554,28,4],[4554,38,1],[4554,47,2],
[4554,56,1],[4554,76,1],[4555,12,1],[4555,31,1],[4555,46,1],
[4555,47,1],[4555,59,1],[4556,0,1],[4556,31,1],[4557,12,1],
[4557,13,1],[4558,9,4],[4559,19,1],[4559,57,1],[4560,23,1],
[4560,28,1],[4561,23,1],[4561,25,1],[4562,23,2],[4563,24,2],
[4563,27,3],[4563,31,1],[4563,47,1],[4564,1,1],[4564,12,1],
[4564,14,1],[4564,30,17],[4564,31,2],[4564,56,1],[4564,68,1],
[4565,17,1],[4565,21,1],[4565,28,1],[4565,29,1],[4565,39,1],
[4565,50,1],[4565,59,1],[4566,10,2],[4566,11,1],[4566,12,4],
[4566,14,1],[4566,16,2],[4566,17,1],[4566,18,2],[4566,19,3],
[4566,37,1],[4566,45,2],[4566,58,3],[4566,59,2],[4566,64,1],
[4566,67,3],[4566,71,1],[4567,10,1],[4567,18,1],[4567,36,1],
[4567,37,1],[4567,43,1],[4567,56,1],[4568,18,1],[4568,59,1],
[4569,0,3],[4569,37,1],[4569,45,1],[4569,65,1],[4569,73,1],
[4570,17,1],[4570,28,2],[4570,53,4],[4570,55,1],[4571,4,2],[4571,6,1],
[4571,23,6],[4571,25,1],[4571,26,2],[4571,27,1],[4571,28,9],
[4571,32,2],[4571,40,1],[4571,44,4],[4571,66,1],[4571,70,1],
[4571,74,1],[4571,76,3],[4572,28,1],[4572,31,1],[4572,43,1],
[4573,27,2],[4573,35,1],[4574,6,1],[4574,28,1],[4574,35,1],
[4575,23,1],[4575,56,1],[4576,15,1],[4576,28,1],[4576,43,1],
[4577,2,1],[4577,4,1],[4577,31,1],[4578,3,3],[4578,21,1],[4578,22,1],
[4578,23,6],[4578,24,1],[4578,25,1],[4578,26,1],[4578,31,1],
[4578,32,1],[4578,33,1],[4578,35,3],[4578,42,3],[4579,16,1],
[4579,33,1],[4580,24,2],[4581,10,1],[4581,17,1],[4581,57,1],
[4581,65,1],[4582,9,1],[4582,28,3],[4582,65,1],[4583,24,1],
[4583,32,1],[4584,10,1],[4584,13,1],[4584,24,1],[4584,26,1],
[4584,28,1],[4584,32,2],[4584,64,1],[4584,69,1],[4585,2,3],[4586,3,1],
[4586,21,1],[4587,14,1],[4587,16,1],[4587,19,1],[4587,37,1],
[4587,54,1],[4588,2,1],[4588,56,1],[4589,2,8],[4589,30,1],[4590,2,2],
[4591,2,3],[4591,28,2],[4591,62,1],[4592,22,1],[4592,23,2],
[4592,25,4],[4592,27,2],[4592,28,1],[4592,31,1],[4592,42,1],
[4592,43,2],[4593,3,1],[4593,4,1],[4593,11,1],[4593,15,1],[4593,20,1],
[4593,22,1],[4593,24,2],[4593,26,1],[4593,28,5],[4593,35,5],
[4593,42,2],[4593,45,2],[4593,60,1],[4593,62,1],[4593,73,1],
[4594,21,2],[4595,32,2],[4595,52,1],[4596,1,3],[4596,9,1],[4596,10,1],
[4596,13,2],[4596,20,1],[4596,59,1],[4597,0,2],[4597,12,1],
[4597,13,2],[4597,19,1],[4597,23,1],[4597,25,13],[4597,28,3],
[4597,39,8],[4597,41,1],[4597,49,1],[4597,72,1],[4598,3,2],
[4598,26,1],[4598,33,3],[4598,64,1],[4599,2,1],[4599,21,1],
[4599,23,1],[4600,5,1],[4600,19,1],[4600,28,1],[4600,60,1],
[4600,65,1],[4601,3,2],[4601,6,2],[4601,22,2],[4601,23,2],[4601,32,2],
[4601,33,1],[4601,40,1],[4601,44,1],[4601,47,1],[4601,56,6],
[4601,67,1],[4601,70,1],[4601,72,1],[4602,1,174],[4602,5,1],
[4602,7,8],[4602,8,4],[4602,12,48],[4602,13,9],[4602,15,1],
[4602,16,2],[4602,17,3],[4602,20,4],[4602,21,1],[4602,26,1],
[4602,29,9],[4602,30,3],[4602,34,5],[4602,36,1],[4602,37,1],
[4602,39,2],[4602,53,3],[4602,55,1],[4602,59,2],[4602,73,1],
[4603,2,1],[4603,28,1],[4603,38,1],[4604,74,2],[4605,23,1],

[4605,27,1],[4605,28,1],[4605,51,4],[4606,28,10],[4607,0,1],
[4607,1,47],[4607,2,3],[4607,3,6],[4607,6,2],[4607,8,1],[4607,9,1],
[4607,10,2],[4607,12,1],[4607,13,9],[4607,15,1],[4607,18,1],
[4607,19,1],[4607,20,6],[4607,23,2],[4607,24,2],[4607,25,2],
[4607,26,1],[4607,27,3],[4607,30,3],[4607,32,3],[4607,33,10],
[4607,38,1],[4607,39,11],[4607,42,6],[4607,43,4],[4607,45,1],
[4607,47,1],[4607,48,2],[4607,51,2],[4607,53,1],[4607,59,1],
[4607,64,1],[4607,65,2],[4607,66,2],[4607,67,1],[4607,71,1],
[4607,78,1],[4608,16,2],[4608,18,1],[4608,41,1],[4609,4,1],[4609,9,1],
[4609,10,1],[4609,15,18],[4609,16,1],[4609,17,12],[4609,20,1],
[4609,28,1],[4609,50,1],[4609,53,18],[4609,55,17],[4609,66,1],
[4610,15,1],[4610,32,1],[4610,44,1],[4611,3,1],[4611,25,2],
[4612,10,1],[4612,78,1],[4613,26,1],[4613,27,1],[4613,32,1],
[4613,66,1],[4614,47,1],[4614,53,1],[4615,13,1],[4615,20,1],
[4615,32,1],[4615,59,2],[4615,62,1],[4616,21,1],[4616,23,1],
[4616,28,1],[4616,42,1],[4617,3,1],[4617,24,1],[4617,26,1],[4618,3,1],
[4618,23,1],[4618,24,1],[4618,25,1],[4618,35,3],[4618,38,1],
[4619,12,1],[4619,26,1],[4620,2,11],[4620,21,1],[4620,24,1],
[4620,31,6],[4620,32,2],[4620,56,1],[4621,13,2],[4621,21,1],
[4621,23,2],[4621,25,2],[4621,33,5],[4621,43,1],[4622,4,1],
[4622,28,1],[4623,23,1],[4623,28,1],[4623,42,7],[4624,4,4],
[4624,13,1],[4624,15,1],[4624,17,1],[4624,22,1],[4624,28,6],
[4624,35,1],[4624,49,1],[4624,50,2],[4624,53,2],[4624,55,1],
[4624,59,2],[4625,24,1],[4625,25,1],[4625,28,2],[4625,32,1],
[4625,62,1],[4626,23,1],[4626,28,127],[4626,63,2],[4626,74,1],
[4627,13,1],[4627,24,5],[4627,55,1],[4627,59,1],[4628,28,2],
[4629,28,1],[4629,40,1],[4630,3,1],[4630,22,1],[4630,23,2],
[4630,28,1],[4631,25,1],[4631,42,1],[4632,23,3],[4632,25,1],
[4632,27,1],[4632,28,2],[4632,39,1],[4632,44,1],[4632,61,1],
[4632,74,1],[4633,4,1],[4633,15,1],[4633,55,1],[4633,66,1],
[4634,32,1],[4634,44,1],[4634,55,1],[4635,32,5],[4636,5,1],
[4636,30,1],[4636,31,1],[4637,23,1],[4637,26,1],[4637,35,1],
[4638,3,6],[4638,5,2],[4638,6,2],[4638,17,8],[4638,21,1],[4638,22,6],
[4638,23,5],[4638,24,31],[4638,26,5],[4638,27,3],[4638,28,11],
[4638,31,7],[4638,32,1],[4638,33,5],[4638,35,8],[4638,42,3],
[4638,43,2],[4638,47,3],[4638,52,1],[4638,53,2],[4638,56,2],
[4638,62,2],[4638,70,1],[4638,75,1],[4638,76,2],[4639,16,1],
[4639,65,1],[4640,23,2],[4641,9,1],[4641,23,1],[4641,30,1],[4642,2,5],
[4642,32,1],[4642,43,1],[4642,62,1],[4642,78,1],[4643,23,2],
[4643,26,1],[4643,32,4],[4643,58,1],[4643,59,1],[4644,31,2],
[4645,28,1],[4645,75,1],[4646,14,1],[4646,63,1],[4646,74,2],
[4646,75,1],[4646,76,1],[4647,6,3],[4647,51,1],[4648,22,1],
[4648,62,1],[4649,1,1],[4649,2,26],[4649,3,3],[4649,4,1],[4649,5,8],
[4649,8,2],[4649,9,2],[4649,10,5],[4649,11,3],[4649,13,5],[4649,15,2],
[4649,17,1],[4649,18,2],[4649,20,24],[4649,22,1],[4649,23,12],
[4649,24,7],[4649,25,1],[4649,26,4],[4649,27,3],[4649,28,10],
[4649,30,7],[4649,32,8],[4649,33,3],[4649,34,3],[4649,35,1],
[4649,37,2],[4649,39,4],[4649,40,1],[4649,41,1],[4649,42,5],
[4649,43,16],[4649,44,3],[4649,45,2],[4649,46,1],[4649,47,5],
[4649,48,1],[4649,49,1],[4649,50,2],[4649,52,1],[4649,54,1],

[4649,56,1],[4649,59,9],[4649,60,3],[4649,62,4],[4649,65,2],
[4649,66,1],[4649,67,1],[4649,68,1],[4649,69,1],[4649,70,2],
[4649,72,1],[4649,73,4],[4649,74,2],[4650,23,3],[4650,24,1],
[4650,34,6],[4650,38,1],[4650,69,1],[4650,71,1],[4651,0,2],[4651,1,1],
[4651,9,3],[4651,10,1],[4651,15,2],[4651,16,1],[4651,17,1],
[4651,19,2],[4651,20,3],[4651,34,2],[4651,53,1],[4651,55,1],
[4651,60,2],[4651,65,1],[4651,71,1],[4652,28,1],[4652,56,4],
[4653,23,1],[4653,26,1],[4653,27,2],[4653,28,1],[4653,31,1],
[4653,42,3],[4653,62,2],[4654,2,3],[4655,1,1],[4655,8,2],[4655,20,2],
[4655,34,8],[4655,36,2],[4655,39,1],[4655,53,1],[4655,73,1],
[4656,12,1],[4656,13,1],[4657,0,22],[4657,1,69],[4657,7,10],
[4657,8,12],[4657,9,66],[4657,10,100],[4657,11,44],[4657,12,69],
[4657,13,21],[4657,14,17],[4657,15,17],[4657,16,45],[4657,17,17],
[4657,18,23],[4657,19,69],[4657,20,22],[4657,22,2],[4657,24,99],
[4657,27,7],[4657,28,1],[4657,29,29],[4657,30,33],[4657,34,25],
[4657,36,19],[4657,37,35],[4657,39,34],[4657,41,61],[4657,44,1],
[4657,45,52],[4657,46,33],[4657,49,15],[4657,53,30],[4657,54,36],
[4657,55,16],[4657,57,41],[4657,58,31],[4657,59,25],[4657,60,7],
[4657,62,1],[4657,64,19],[4657,65,73],[4657,67,34],[4657,69,1],
[4657,71,22],[4657,73,33],[4657,77,35],[4657,78,16],[4658,24,4],
[4659,23,1],[4659,24,2],[4659,32,1],[4659,48,1],[4660,15,1],
[4660,44,1],[4661,28,1],[4661,61,1],[4662,2,1],[4662,23,1],
[4663,10,1],[4663,41,1],[4664,15,2],[4664,55,1],[4664,70,1],
[4665,20,2],[4665,22,3],[4665,24,1],[4665,28,1],[4666,0,3],[4666,9,1],
[4666,20,15],[4666,39,1],[4666,42,1],[4667,22,18],[4667,27,7],
[4667,28,1],[4667,35,4],[4667,43,1],[4667,70,1],[4668,28,1],
[4668,30,1],[4668,31,1],[4668,46,1],[4668,47,1],[4669,26,1],
[4669,28,1],[4669,32,2],[4670,6,2],[4670,24,1],[4670,28,1],
[4670,60,1],[4671,1,1],[4671,14,1],[4671,15,2],[4671,19,1],
[4671,20,1],[4671,23,1],[4671,38,22],[4671,39,2],[4671,41,1],
[4671,44,1],[4671,49,1],[4671,53,1],[4671,54,1],[4671,58,1],
[4671,62,1],[4671,65,1],[4671,67,1],[4671,76,1],[4672,2,1],
[4672,20,1],[4672,23,6],[4672,50,1],[4672,75,1],[4672,78,1],
[4673,2,1],[4673,4,1],[4673,5,1],[4673,15,1],[4673,23,1],[4673,24,4],
[4673,31,1],[4673,32,1],[4673,33,10],[4673,40,1],[4673,42,6],
[4673,43,1],[4673,47,2],[4673,48,2],[4673,51,1],[4673,69,2],
[4673,75,1],[4674,2,4],[4675,17,1],[4675,53,1],[4676,2,1],[4676,5,1],
[4676,6,2],[4676,20,1],[4676,22,1],[4676,26,1],[4676,27,1],
[4676,35,1],[4676,43,1],[4676,59,1],[4677,23,2],[4677,36,1],
[4677,59,1],[4678,32,1],[4678,56,1],[4679,23,2],[4679,35,1],
[4680,3,1],[4680,31,1],[4681,1,1],[4681,22,1],[4681,27,3],[4681,48,1],
[4682,1,1],[4682,3,6],[4682,6,2],[4682,16,1],[4682,20,6],[4682,21,1],
[4682,22,1],[4682,23,4],[4682,24,49],[4682,25,1],[4682,26,2],
[4682,27,6],[4682,28,1],[4682,29,2],[4682,32,5],[4682,33,5],
[4682,35,4],[4682,42,3],[4682,47,2],[4682,56,4],[4682,63,1],
[4682,66,2],[4682,69,1],[4682,74,1],[4682,76,1],[4683,15,1],
[4683,54,1],[4683,57,1],[4684,25,1],[4684,32,1],[4684,62,1],
[4684,70,2],[4684,76,1],[4685,10,2],[4685,11,1],[4685,14,1],
[4685,29,1],[4685,41,1],[4685,49,1],[4686,8,6],[4686,10,1],
[4686,12,1],[4687,5,1],[4687,8,1],[4687,20,4],[4687,21,1],[4687,22,4],

[4687,23,23],[4687,24,25],[4687,25,1],[4687,27,200],[4687,28,3],
[4687,31,1],[4687,33,2],[4687,34,2],[4687,35,5],[4687,40,2],
[4687,42,2],[4687,56,1],[4687,68,1],[4687,70,4],[4687,72,1],
[4687,76,4],[4688,3,1],[4688,24,3],[4688,27,1],[4688,28,1],
[4688,35,4],[4689,24,1],[4689,56,1],[4690,24,1],[4690,32,1],
[4691,15,1],[4691,53,2],[4691,55,2],[4692,5,2],[4692,28,2],[4693,0,7],
[4693,1,45],[4693,2,4],[4693,3,2],[4693,4,2],[4693,5,6],[4693,6,1],
[4693,7,16],[4693,8,17],[4693,9,94],[4693,10,124],[4693,11,47],
[4693,12,64],[4693,13,23],[4693,14,41],[4693,15,38],[4693,16,56],
[4693,17,12],[4693,18,40],[4693,19,98],[4693,20,30],[4693,21,2],
[4693,22,4],[4693,23,2],[4693,24,1],[4693,25,3],[4693,26,3],
[4693,27,3],[4693,28,73],[4693,29,13],[4693,30,16],[4693,31,3],
[4693,32,5],[4693,33,10],[4693,34,41],[4693,35,4],[4693,36,23],
[4693,37,35],[4693,38,3],[4693,39,18],[4693,40,5],[4693,41,55],
[4693,42,3],[4693,43,2],[4693,44,3],[4693,45,69],[4693,46,44],
[4693,47,2],[4693,48,2],[4693,49,21],[4693,50,5],[4693,51,4],
[4693,53,23],[4693,54,32],[4693,55,19],[4693,56,2],[4693,57,44],
[4693,58,35],[4693,59,42],[4693,60,13],[4693,61,3],[4693,62,3],
[4693,63,18],[4693,64,15],[4693,65,78],[4693,66,1],[4693,67,38],
[4693,68,4],[4693,69,1],[4693,70,21],[4693,71,31],[4693,73,34],
[4693,74,13],[4693,75,13],[4693,76,30],[4693,77,45],[4693,78,10],
[4693,79,1],[4694,31,2],[4694,32,1],[4695,10,1],[4695,26,1],
[4695,28,12],[4695,31,1],[4695,35,1],[4695,37,1],[4695,51,1],
[4695,56,2],[4695,59,1],[4695,61,1],[4695,70,2],[4695,74,1],
[4695,76,1],[4696,1,2],[4696,28,1],[4696,48,4],[4696,58,1],
[4696,65,1],[4697,21,1],[4697,28,1],[4698,50,2],[4698,57,1],
[4699,2,1],[4699,24,2],[4699,27,61],[4699,28,5],[4699,50,1],
[4700,20,1],[4700,28,1],[4700,33,1],[4701,28,1],[4701,66,1],
[4702,2,2],[4702,4,1],[4702,5,2],[4702,15,1],[4702,17,1],[4702,21,1],
[4702,26,5],[4702,27,3],[4702,32,4],[4702,35,2],[4702,37,1],
[4702,43,9],[4702,74,1],[4702,76,1],[4703,2,2],[4704,21,1],
[4704,39,1],[4705,3,1],[4705,8,1],[4705,9,1],[4705,13,1],[4705,15,1],
[4705,20,1],[4705,25,1],[4705,27,2],[4705,28,4],[4705,32,4],
[4705,35,3],[4705,45,1],[4705,65,1],[4705,70,1],[4706,28,1],
[4706,35,1],[4707,6,4],[4707,35,1],[4707,50,1],[4708,23,5],
[4708,32,2],[4709,26,2],[4710,28,1],[4710,63,1],[4710,75,1],
[4711,24,1],[4711,28,9],[4711,35,1],[4711,38,1],[4711,44,2],
[4711,50,1],[4711,66,2],[4712,26,1],[4712,27,1],[4712,31,1],
[4713,25,1],[4713,31,1],[4714,21,1],[4714,66,1],[4715,2,1],
[4715,19,1],[4715,28,4],[4715,44,2],[4715,51,1],[4715,62,1],
[4715,63,1],[4716,37,4],[4716,40,1],[4716,42,1],[4716,44,1],
[4716,56,1],[4717,1,1],[4717,12,1],[4718,2,1],[4718,27,1],[4718,28,1],
[4719,12,1],[4719,23,3],[4719,25,1],[4719,26,1],[4719,31,1],
[4719,33,23],[4719,38,1],[4719,39,2],[4719,50,1],[4719,65,1],
[4719,76,2],[4720,2,1],[4720,4,1],[4720,28,3],[4720,44,1],[4720,50,1],
[4720,51,1],[4720,55,2],[4720,66,1],[4721,11,1],[4721,15,2],
[4721,19,1],[4721,33,1],[4721,55,4],[4721,56,1],[4721,61,1],
[4721,65,1],[4722,37,8],[4723,2,3],[4723,3,1],[4723,28,2],[4724,24,1],
[4724,27,1],[4725,24,3],[4725,26,1],[4725,27,3],[4725,28,2],
[4725,32,8],[4725,35,3],[4725,42,2],[4726,5,1],[4726,21,1],

[4726,22,2],[4726,24,1],[4726,25,1],[4726,26,2],[4726,27,1],
[4726,31,2],[4726,33,1],[4726,35,2],[4726,38,1],[4726,40,1],
[4726,50,2],[4726,56,1],[4726,63,4],[4726,66,2],[4726,74,2],
[4726,76,1],[4727,13,2],[4727,19,1],[4727,59,1],[4728,9,1],
[4728,34,1],[4728,36,1],[4728,41,1],[4728,65,2],[4729,4,2],
[4729,50,1],[4730,1,2],[4730,8,1],[4730,13,1],[4730,17,1],[4730,39,1],
[4731,4,6],[4731,5,1],[4731,23,1],[4731,25,1],[4731,33,1],[4731,50,7],
[4732,0,1],[4732,1,6],[4732,4,17],[4732,5,1],[4732,7,2],[4732,8,3],
[4732,9,12],[4732,10,16],[4732,11,3],[4732,12,6],[4732,13,4],
[4732,15,282],[4732,16,9],[4732,17,136],[4732,18,3],[4732,19,8],
[4732,22,2],[4732,23,2],[4732,28,2],[4732,29,3],[4732,30,2],
[4732,34,2],[4732,36,1],[4732,37,4],[4732,38,1],[4732,41,13],
[4732,42,1],[4732,44,14],[4732,45,6],[4732,46,6],[4732,49,2],
[4732,50,20],[4732,53,102],[4732,54,5],[4732,55,174],[4732,56,1],
[4732,57,3],[4732,58,7],[4732,59,2],[4732,60,2],[4732,62,1],
[4732,64,1],[4732,65,8],[4732,66,16],[4732,67,5],[4732,70,1],
[4732,71,1],[4732,72,1],[4732,73,2],[4732,76,1],[4732,77,3],
[4733,13,1],[4733,24,1],[4733,26,1],[4733,58,1],[4733,67,1],
[4734,4,1],[4734,20,1],[4735,1,2],[4735,2,2],[4735,3,3],[4735,4,41],
[4735,5,2],[4735,9,1],[4735,12,1],[4735,15,2],[4735,16,1],[4735,17,7],
[4735,20,1],[4735,21,2],[4735,22,1],[4735,23,5],[4735,24,2],
[4735,25,4],[4735,26,4],[4735,27,6],[4735,28,4],[4735,32,1],
[4735,33,6],[4735,34,1],[4735,35,1],[4735,36,1],[4735,38,10],
[4735,39,1],[4735,42,4],[4735,43,3],[4735,44,341],[4735,47,4],
[4735,50,55],[4735,51,1],[4735,52,1],[4735,53,7],[4735,54,1],
[4735,62,9],[4735,63,2],[4735,66,263],[4735,68,1],[4735,69,2],
[4735,74,4],[4735,75,2],[4735,76,2],[4736,4,1],[4736,20,1],
[4736,53,1],[4736,55,6],[4737,4,2],[4737,5,1],[4737,6,1],[4737,21,1],
[4737,23,4],[4737,27,1],[4737,32,1],[4737,35,1],[4737,42,1],
[4737,47,1],[4737,50,2],[4737,56,4],[4737,62,1],[4738,24,1],
[4738,28,2],[4738,43,1],[4738,60,1],[4739,1,2],[4740,1,2],[4740,20,1],
[4741,2,4],[4741,5,1],[4741,17,1],[4741,21,1],[4741,22,2],[4741,26,1],
[4741,27,3],[4741,31,1],[4741,35,1],[4741,38,1],[4741,42,1],
[4741,53,1],[4741,63,1],[4741,68,1],[4741,75,1],[4742,3,2],
[4743,22,3],[4743,23,2],[4743,24,1],[4743,26,3],[4743,27,1],
[4743,28,4],[4743,33,1],[4743,35,1],[4743,43,1],[4743,47,1],
[4744,16,1],[4744,28,1],[4745,2,15],[4745,3,35],[4745,10,1],
[4745,39,1],[4745,64,1],[4745,70,1],[4745,74,1],[4746,5,1],
[4746,23,1],[4746,33,1],[4747,1,1],[4747,20,1],[4748,22,1],
[4748,28,1],[4748,63,1],[4749,28,2],[4749,70,1],[4750,1,2],[4750,2,9],
[4750,3,3],[4750,4,2],[4750,5,3],[4750,6,5],[4750,10,3],[4750,11,5],
[4750,12,4],[4750,13,7],[4750,17,1],[4750,20,18],[4750,21,4],
[4750,22,6],[4750,23,3],[4750,24,1],[4750,26,3],[4750,27,19],
[4750,28,4],[4750,31,1],[4750,32,6],[4750,33,4],[4750,34,3],
[4750,35,8],[4750,36,1],[4750,37,1],[4750,39,1],[4750,40,1],
[4750,41,1],[4750,42,1],[4750,43,1],[4750,44,1],[4750,45,2],
[4750,47,2],[4750,48,1],[4750,51,3],[4750,56,4],[4750,58,1],
[4750,59,1],[4750,62,1],[4750,65,1],[4750,66,1],[4750,68,1],
[4750,74,3],[4751,2,1],[4751,3,1],[4751,5,2],[4751,15,1],[4751,20,1],
[4751,23,3],[4751,24,1],[4751,25,1],[4751,28,2],[4751,30,1],

[4751,32,6],[4751,42,1],[4751,63,1],[4752,6,1],[4752,28,1],
[4753,16,1],[4753,53,1],[4754,2,1],[4754,23,1],[4754,24,1],
[4754,32,1],[4755,0,2],[4755,2,2],[4755,5,1],[4755,8,1],[4755,9,1],
[4755,10,2],[4755,12,3],[4755,15,1],[4755,17,1],[4755,18,1],
[4755,19,1],[4755,20,1],[4755,21,7],[4755,22,6],[4755,23,14],
[4755,24,3],[4755,25,15],[4755,26,7],[4755,27,7],[4755,28,6],
[4755,29,1],[4755,30,3],[4755,31,45],[4755,32,44],[4755,33,1],
[4755,34,1],[4755,35,1],[4755,40,2],[4755,41,1],[4755,42,41],
[4755,43,50],[4755,44,1],[4755,46,1],[4755,49,1],[4755,52,1],
[4755,55,1],[4755,57,1],[4755,59,1],[4755,60,2],[4755,62,1],
[4755,63,1],[4755,70,1],[4755,77,1],[4755,79,2],[4756,32,1],
[4756,35,1],[4756,40,1],[4756,44,1],[4757,3,2],[4757,28,1],
[4758,20,1],[4758,23,1],[4759,0,48],[4759,7,34],[4759,8,1],
[4759,10,1],[4759,11,4],[4759,12,5],[4759,14,2],[4759,16,3],
[4759,19,2],[4759,20,1],[4759,29,2],[4759,30,1],[4759,31,1],
[4759,34,2],[4759,36,2],[4759,41,2],[4759,45,1],[4759,53,1],
[4759,58,2],[4759,59,2],[4759,67,1],[4759,71,1],[4759,73,3],
[4759,77,2],[4759,78,1],[4760,62,2],[4761,2,2],[4761,26,1],[4762,3,1],
[4762,11,1],[4762,24,1],[4762,55,1],[4762,62,1],[4762,69,1],
[4762,72,1],[4763,2,10],[4764,22,1],[4764,38,1],[4764,42,1],
[4764,44,1],[4764,48,1],[4764,57,1],[4764,77,1],[4765,2,1],
[4765,24,1],[4766,9,1],[4766,10,1],[4766,20,2],[4766,21,1],
[4766,23,1],[4766,25,1],[4766,26,1],[4766,27,9],[4766,28,1],
[4766,31,6],[4766,33,10],[4766,34,3],[4766,35,6],[4766,39,1],
[4766,41,1],[4766,43,12],[4766,53,1],[4766,57,1],[4766,58,1],
[4767,22,8],[4767,25,14],[4767,27,2],[4767,35,6],[4767,56,1],
[4768,12,2],[4768,29,2],[4769,4,1],[4769,24,1],[4769,74,1],[4770,3,1],
[4770,31,1],[4771,15,2],[4772,13,1],[4772,25,1],[4772,30,1],
[4772,59,2],[4773,63,1],[4773,74,1],[4773,75,1],[4773,76,2],
[4774,22,1],[4774,31,1],[4775,1,2],[4775,12,1],[4775,28,2],
[4775,78,1],[4776,9,1],[4776,20,1],[4777,25,1],[4777,28,2],[4778,2,1],
[4778,3,1],[4778,20,1],[4778,22,1],[4778,23,1],[4778,30,1],
[4778,60,1],[4778,77,1],[4779,26,1],[4779,35,1],[4780,1,3],[4780,2,2],
[4780,3,1],[4780,5,1],[4780,8,1],[4780,10,2],[4780,12,11],[4780,13,6],
[4780,14,1],[4780,17,1],[4780,20,1],[4780,21,2],[4780,23,5],
[4780,24,9],[4780,25,1],[4780,26,1],[4780,27,7],[4780,28,8],
[4780,30,2],[4780,32,3],[4780,33,1],[4780,34,7],[4780,35,3],
[4780,36,1],[4780,37,1],[4780,39,4],[4780,41,1],[4780,42,2],
[4780,43,1],[4780,45,2],[4780,46,2],[4780,48,1],[4780,50,1],
[4780,54,1],[4780,55,1],[4780,61,2],[4780,62,13],[4780,64,1],
[4780,70,1],[4780,71,1],[4780,73,1],[4780,77,6],[4780,78,1],
[4781,15,1],[4781,37,3],[4781,71,1],[4782,3,2],[4782,17,1],
[4782,22,2],[4782,23,4],[4782,24,14],[4782,26,1],[4782,27,2],
[4782,33,9],[4782,38,1],[4782,42,1],[4782,43,1],[4782,47,2],
[4782,48,7],[4782,56,1],[4782,62,1],[4782,63,1],[4782,68,2],
[4782,70,1],[4782,74,1],[4782,76,1],[4783,24,12],[4783,47,3],
[4783,74,1],[4784,26,2],[4785,28,2],[4786,32,1],[4786,35,1],
[4786,47,1],[4786,59,3],[4786,61,1],[4786,65,1],[4786,68,3],
[4787,2,2],[4787,3,2],[4787,4,1],[4787,5,1],[4787,22,3],[4787,24,3],
[4787,26,1],[4787,27,6],[4787,28,28],[4787,31,2],[4787,32,2],

[4787,33,1],[4787,40,3],[4787,42,1],[4787,43,2],[4787,44,1],
[4787,50,2],[4787,52,2],[4787,63,2],[4787,68,1],[4787,70,1],
[4787,72,2],[4787,74,2],[4787,76,1],[4788,23,1],[4788,27,1],
[4789,26,1],[4789,31,1],[4789,33,3],[4790,21,1],[4790,31,2],
[4790,33,2],[4790,42,1],[4790,43,1],[4791,5,1],[4791,28,2],
[4791,31,2],[4792,2,6],[4792,72,2],[4793,10,1],[4793,20,1],[4794,3,1],
[4794,20,1],[4794,26,2],[4794,27,1],[4794,28,1],[4794,32,3],
[4794,35,1],[4795,2,3],[4795,3,2],[4795,26,1],[4795,27,4],[4795,33,5],
[4795,34,1],[4795,70,1],[4796,38,2],[4796,70,2],[4797,28,1],
[4797,76,2],[4798,27,1],[4798,38,1],[4799,5,1],[4799,33,1],
[4800,70,1],[4800,73,1],[4801,24,1],[4801,51,2],[4802,2,1],
[4802,30,1],[4802,32,2],[4802,51,2],[4802,56,1],[4802,61,1],
[4802,62,1],[4802,66,1],[4802,74,1],[4803,5,1],[4803,48,1],
[4803,72,1],[4804,2,1],[4804,28,2],[4805,33,1],[4805,35,1],
[4806,19,1],[4806,31,1],[4807,2,1],[4807,28,2],[4807,31,1],
[4808,27,6],[4808,28,1],[4809,0,1],[4809,5,1],[4809,9,3],[4809,10,1],
[4809,15,1],[4809,19,1],[4809,28,7],[4809,38,1],[4809,45,3],
[4809,46,1],[4809,54,1],[4809,56,1],[4809,59,1],[4810,24,1],
[4810,66,1],[4810,74,1],[4811,22,1],[4811,27,1],[4812,24,1],
[4812,28,1],[4812,44,14],[4812,50,1],[4812,66,7],[4813,23,1],
[4813,35,1],[4814,24,1],[4814,28,2],[4815,3,1],[4815,7,3],[4815,22,1],
[4815,30,1],[4816,5,1],[4816,56,1],[4817,31,2],[4818,25,2],
[4819,11,2],[4819,20,2],[4819,32,2],[4820,28,1],[4820,31,1],
[4820,42,1],[4820,43,2],[4821,6,1],[4821,23,1],[4821,24,1],
[4821,25,5],[4821,28,1],[4821,32,4],[4821,33,1],[4821,37,2],
[4821,72,1],[4822,23,3],[4822,28,1],[4822,31,1],[4823,37,2],
[4823,57,1],[4823,58,1],[4824,22,1],[4824,24,1],[4824,25,1],
[4824,27,1],[4825,0,2],[4825,1,13],[4825,7,1],[4825,8,6],[4825,9,22],
[4825,10,35],[4825,11,9],[4825,12,11],[4825,13,6],[4825,14,8],
[4825,15,11],[4825,16,18],[4825,17,4],[4825,18,8],[4825,19,28],
[4825,20,19],[4825,28,1],[4825,29,3],[4825,30,4],[4825,34,7],
[4825,36,7],[4825,37,7],[4825,39,8],[4825,41,19],[4825,45,17],
[4825,46,7],[4825,49,4],[4825,53,4],[4825,54,14],[4825,55,10],
[4825,57,19],[4825,58,18],[4825,59,9],[4825,60,4],[4825,64,2],
[4825,65,15],[4825,67,13],[4825,71,13],[4825,73,21],[4825,74,1],
[4825,77,7],[4825,78,2],[4825,79,32],[4826,2,4],[4826,28,1],
[4827,21,1],[4827,42,1],[4828,28,2],[4828,76,1],[4829,0,1],[4829,1,4],
[4829,2,2],[4829,4,10],[4829,5,3],[4829,8,1],[4829,9,5],[4829,10,11],
[4829,11,1],[4829,12,4],[4829,13,2],[4829,14,3],[4829,15,77],
[4829,16,2],[4829,17,26],[4829,18,1],[4829,19,7],[4829,20,2],
[4829,22,1],[4829,23,4],[4829,24,1],[4829,28,2],[4829,30,1],
[4829,32,1],[4829,33,1],[4829,34,1],[4829,36,1],[4829,37,3],
[4829,38,2],[4829,39,3],[4829,41,3],[4829,44,8],[4829,45,6],
[4829,46,1],[4829,49,3],[4829,50,20],[4829,53,39],[4829,54,1],
[4829,55,51],[4829,57,6],[4829,58,3],[4829,59,3],[4829,60,1],
[4829,61,1],[4829,62,1],[4829,64,1],[4829,65,7],[4829,66,6],
[4829,67,4],[4829,68,1],[4829,71,2],[4829,72,1],[4829,74,1],
[4829,77,2],[4829,78,1],[4830,53,1],[4830,55,2],[4831,3,1],[4831,6,2],
[4831,23,1],[4831,24,16],[4831,26,2],[4831,27,3],[4831,28,3],
[4831,33,5],[4831,42,2],[4831,43,1],[4831,47,2],[4831,48,3],

[4831,56,2],[4831,63,1],[4831,66,1],[4831,70,1],[4831,76,1],
[4832,0,1],[4832,5,1],[4832,8,1],[4832,9,1],[4832,11,1],[4832,17,15],
[4832,19,2],[4832,27,1],[4832,34,1],[4832,35,1],[4832,36,1],
[4832,38,1],[4832,50,1],[4832,53,16],[4832,54,1],[4832,55,5],
[4832,70,1],[4833,4,1],[4833,20,2],[4833,22,1],[4833,23,1],
[4833,24,1],[4833,33,2],[4833,48,1],[4833,50,1],[4833,62,1],
[4833,68,1],[4833,69,2],[4834,2,10],[4835,1,6],[4835,13,2],
[4835,20,1],[4835,59,1],[4836,1,4],[4836,5,6],[4836,7,1],[4836,8,1],
[4836,12,1],[4836,15,3],[4836,19,1],[4836,20,1],[4836,21,2],
[4836,38,2],[4836,39,2],[4836,44,2],[4836,45,1],[4836,47,1],
[4836,50,1],[4836,52,2],[4836,55,1],[4836,59,1],[4837,1,2],
[4837,12,4],[4837,13,1],[4837,16,1],[4837,17,1],[4838,26,1],
[4838,43,1],[4838,66,1],[4839,5,1],[4839,6,1],[4840,1,1],[4840,2,4],
[4840,20,9],[4840,23,8],[4840,24,6],[4840,27,11],[4840,39,1],
[4840,47,8],[4840,65,1],[4840,68,1],[4841,1,1],[4841,5,1],[4841,24,3],
[4841,25,9],[4841,26,3],[4841,27,1],[4841,28,1],[4841,31,6],
[4841,33,36],[4841,42,2],[4841,44,1],[4841,48,3],[4841,66,1],
[4841,70,3],[4841,74,2],[4842,26,1],[4842,27,1],[4843,28,1],
[4843,52,5],[4844,4,2],[4844,17,1],[4844,44,1],[4844,55,1],
[4845,33,1],[4845,43,1],[4846,9,1],[4846,53,1],[4847,23,3],
[4847,51,3],[4847,56,1],[4847,61,1],[4847,68,2],[4847,69,1],
[4848,0,9],[4848,1,13],[4848,2,1],[4848,3,1],[4848,4,3],[4848,5,2],
[4848,7,1],[4848,8,6],[4848,9,42],[4848,10,14],[4848,11,8],
[4848,12,12],[4848,13,6],[4848,14,5],[4848,15,54],[4848,16,6],
[4848,17,25],[4848,18,7],[4848,19,12],[4848,20,16],[4848,22,2],
[4848,23,1],[4848,25,2],[4848,26,1],[4848,28,16],[4848,29,4],
[4848,30,13],[4848,31,2],[4848,32,1],[4848,33,1],[4848,34,7],
[4848,35,1],[4848,36,2],[4848,37,14],[4848,38,2],[4848,39,8],
[4848,40,1],[4848,41,8],[4848,43,3],[4848,44,6],[4848,45,6],
[4848,46,6],[4848,48,21],[4848,49,7],[4848,50,3],[4848,52,1],
[4848,53,26],[4848,54,7],[4848,55,52],[4848,56,1],[4848,57,5],
[4848,58,8],[4848,59,4],[4848,60,6],[4848,62,3],[4848,64,5],
[4848,65,13],[4848,66,4],[4848,67,4],[4848,68,2],[4848,70,5],
[4848,71,3],[4848,72,1],[4848,73,8],[4848,74,4],[4848,75,1],
[4848,76,6],[4848,77,5],[4848,78,2],[4849,4,4],[4849,5,2],[4849,9,1],
[4849,22,1],[4849,27,1],[4849,28,1],[4849,34,1],[4849,38,3],
[4849,44,5],[4849,47,1],[4849,50,3],[4849,52,1],[4849,59,1],
[4849,66,6],[4849,70,1],[4849,75,1],[4850,50,1],[4850,66,1],
[4851,5,4],[4851,10,1],[4851,11,1],[4851,15,1],[4851,28,3],
[4851,42,1],[4852,4,1],[4852,5,2],[4852,17,4],[4852,24,1],[4852,27,1],
[4852,28,1],[4852,29,1],[4852,35,2],[4852,43,1],[4852,50,1],
[4852,53,6],[4852,55,1],[4852,56,1],[4852,64,1],[4852,68,1],
[4852,72,1],[4852,74,1],[4852,76,2],[4853,2,1],[4853,28,1],
[4853,43,2],[4853,47,12],[4854,28,1],[4854,42,1],[4855,47,12],
[4855,51,4],[4855,56,3],[4855,68,1],[4856,2,1],[4856,28,1],
[4857,77,2],[4858,1,1],[4858,12,1],[4859,28,2],[4859,30,1],
[4859,33,3],[4860,22,1],[4860,28,1],[4860,43,1],[4861,2,27],
[4861,24,1],[4861,28,2],[4861,32,1],[4861,38,1],[4861,72,1],
[4862,16,1],[4862,33,2],[4862,37,1],[4863,23,5],[4863,35,1],
[4864,28,1],[4864,31,1],[4865,22,1],[4865,33,1],[4866,25,1],

[4866,28,6],[4866,43,2],[4866,59,1],[4866,68,1],[4866,72,1],
[4866,75,1],[4866,76,1],[4867,4,1],[4867,10,1],[4868,17,1],
[4868,30,4],[4868,37,1],[4868,45,1],[4868,53,1],[4869,2,1],
[4869,12,1],[4870,47,1],[4870,53,1],[4871,20,1],[4871,28,1],
[4872,13,1],[4872,22,2],[4872,26,1],[4873,21,1],[4873,32,1],
[4873,42,2],[4874,30,2],[4874,31,2],[4875,22,1],[4875,23,1],
[4875,24,1],[4875,26,2],[4875,28,1],[4875,35,1],[4875,37,2],
[4875,38,2],[4875,42,5],[4875,47,1],[4875,48,7],[4875,50,1],
[4875,62,1],[4875,66,1],[4875,72,1],[4875,74,2],[4875,76,2],
[4876,3,1],[4876,12,1],[4876,13,1],[4876,21,1],[4876,23,1],
[4876,33,2],[4876,38,1],[4876,44,1],[4876,47,11],[4876,60,1],
[4876,67,1],[4876,69,1],[4876,78,1],[4877,19,1],[4877,25,1],
[4877,27,2],[4878,22,2],[4878,24,1],[4878,27,1],[4878,28,2],
[4878,39,1],[4879,70,1],[4879,72,1],[4880,2,4],[4880,3,1],[4880,4,1],
[4880,28,1],[4880,33,1],[4880,35,1],[4880,44,1],[4881,53,2],
[4882,0,5],[4882,1,20],[4882,2,1],[4882,3,1],[4882,5,2],[4882,7,9],
[4882,8,9],[4882,9,33],[4882,10,80],[4882,11,32],[4882,12,26],
[4882,13,28],[4882,14,24],[4882,15,22],[4882,16,41],[4882,17,9],
[4882,18,17],[4882,19,74],[4882,20,11],[4882,21,1],[4882,24,4],
[4882,25,3],[4882,26,2],[4882,27,1],[4882,28,11],[4882,29,7],
[4882,30,6],[4882,31,3],[4882,32,2],[4882,33,1],[4882,34,10],
[4882,35,4],[4882,36,16],[4882,37,20],[4882,38,1],[4882,39,6],
[4882,40,6],[4882,41,50],[4882,42,1],[4882,43,1],[4882,45,47],
[4882,46,22],[4882,48,1],[4882,49,17],[4882,53,9],[4882,54,16],
[4882,55,18],[4882,56,1],[4882,57,29],[4882,58,30],[4882,59,27],
[4882,60,6],[4882,63,4],[4882,64,7],[4882,65,60],[4882,66,2],
[4882,67,25],[4882,68,4],[4882,69,5],[4882,70,14],[4882,71,19],
[4882,72,9],[4882,73,33],[4882,74,2],[4882,75,3],[4882,76,3],
[4882,77,28],[4882,78,6],[4883,5,1],[4883,28,1],[4883,33,1],
[4883,40,1],[4883,42,1],[4883,47,2],[4883,50,1],[4883,56,1],
[4883,63,66],[4883,68,2],[4883,70,2],[4883,72,1],[4883,74,63],
[4883,75,54],[4883,76,103],[4884,1,2],[4884,12,1],[4885,24,2],
[4885,28,1],[4885,35,1],[4885,50,1],[4885,62,1],[4886,8,1],
[4886,28,2],[4886,30,1],[4886,31,1],[4886,41,1],[4886,46,1],
[4887,23,1],[4887,35,2],[4887,57,1],[4887,62,3],[4888,28,4],
[4888,75,1],[4888,76,1],[4889,13,1],[4889,20,1],[4889,27,1],
[4889,28,2],[4889,62,1],[4890,4,1],[4890,63,1],[4891,2,1],[4891,18,1],
[4891,45,1],[4892,1,1],[4892,3,1],[4892,5,2],[4892,15,1],[4892,22,2],
[4892,23,2],[4892,24,7],[4892,26,5],[4892,27,3],[4892,28,12],
[4892,31,2],[4892,32,1],[4892,33,2],[4892,35,3],[4892,38,363],
[4892,40,2],[4892,43,1],[4892,44,1],[4892,47,1],[4892,50,3],
[4892,53,1],[4892,54,1],[4892,55,1],[4892,56,1],[4892,58,1],
[4892,62,1],[4892,63,1],[4892,66,3],[4892,68,2],[4892,69,1],
[4892,70,3],[4892,72,1],[4892,74,1],[4892,75,5],[4892,76,2],
[4892,78,1],[4893,23,1],[4893,24,1],[4893,25,1],[4893,27,7],
[4893,28,4],[4893,35,6],[4893,38,1],[4893,43,1],[4893,56,2],
[4894,35,1],[4894,48,1],[4895,15,1],[4895,17,1],[4896,2,4],
[4896,13,1],[4897,2,2],[4898,23,2],[4898,68,1],[4898,74,1],
[4898,75,2],[4898,76,3],[4898,78,1],[4899,22,1],[4899,23,2],
[4899,24,2],[4899,32,3],[4899,34,1],[4899,68,1],[4899,76,1],

[4900,4,1],[4900,22,3],[4900,25,1],[4900,27,3],[4900,35,5],
[4900,38,2],[4901,5,1],[4901,53,1],[4902,28,1],[4902,68,1],
[4903,24,3],[4904,5,1],[4904,19,1],[4904,43,1],[4905,31,1],
[4905,35,1],[4906,9,1],[4906,13,1],[4907,52,3],[4908,2,18],
[4908,3,79],[4908,4,3],[4908,5,12],[4908,6,12],[4908,8,4],[4908,10,4],
[4908,12,4],[4908,13,11],[4908,15,1],[4908,16,3],[4908,20,17],
[4908,21,11],[4908,22,12],[4908,23,157],[4908,24,171],[4908,25,7],
[4908,26,89],[4908,27,68],[4908,28,71],[4908,30,10],[4908,31,34],
[4908,32,67],[4908,33,33],[4908,34,4],[4908,35,47],[4908,36,3],
[4908,37,2],[4908,38,4],[4908,39,7],[4908,40,2],[4908,42,62],
[4908,43,148],[4908,46,2],[4908,47,23],[4908,48,4],[4908,49,2],
[4908,50,5],[4908,52,1],[4908,53,1],[4908,54,2],[4908,56,10],
[4908,57,2],[4908,58,1],[4908,59,39],[4908,60,1],[4908,61,3],
[4908,62,4],[4908,63,1],[4908,64,3],[4908,66,1],[4908,70,1],
[4908,73,1],[4908,74,2],[4908,75,2],[4908,76,3],[4908,78,1],
[4909,1,1],[4909,21,1],[4909,28,1],[4910,2,1],[4910,20,3],[4910,23,1],
[4910,24,2],[4910,28,1],[4911,28,1],[4911,30,1],[4911,34,2],
[4912,1,1],[4912,11,1],[4912,14,1],[4912,15,1],[4912,16,3],
[4912,19,2],[4912,23,2],[4912,27,1],[4912,28,1],[4912,30,1],
[4912,32,1],[4912,34,6],[4912,35,1],[4912,38,1],[4912,46,1],
[4912,56,1],[4912,65,1],[4912,66,1],[4913,9,1],[4913,11,1],
[4913,20,1],[4913,36,1],[4913,54,1],[4913,58,1],[4913,59,1],
[4913,65,2],[4913,78,1],[4914,3,1],[4914,47,1],[4915,3,1],[4915,52,1],
[4916,2,1],[4916,30,1],[4916,53,1],[4917,47,4],[4918,14,1],
[4918,33,2],[4919,53,2],[4919,55,2],[4919,65,1],[4920,21,2],
[4920,27,1],[4920,28,1],[4921,2,3],[4921,8,1],[4922,1,1],[4922,15,2],
[4922,41,1],[4923,3,1],[4923,5,1],[4923,27,1],[4923,31,1],[4923,33,2],
[4923,47,2],[4924,2,3],[4924,10,1],[4924,12,1],[4924,15,1],
[4924,22,1],[4924,25,2],[4924,26,1],[4924,27,1],[4924,31,2],
[4924,42,1],[4924,47,1],[4924,54,1],[4925,20,2],[4926,61,2],
[4926,68,1],[4926,72,1],[4927,6,1],[4927,51,1],[4927,56,1],
[4927,70,1],[4928,1,1],[4928,13,1],[4928,14,1],[4928,46,1],[4929,3,1],
[4929,23,1],[4929,28,3],[4929,32,4],[4929,33,1],[4929,35,1],
[4929,43,2],[4930,60,2],[4931,62,2],[4932,21,1],[4932,23,1],
[4932,24,1],[4932,28,5],[4933,1,3],[4933,2,1],[4933,5,2],[4933,9,1],
[4933,13,2],[4933,20,3],[4933,21,1],[4933,23,1],[4933,28,1],
[4933,32,2],[4933,45,1],[4933,46,1],[4933,61,1],[4934,2,3],[4935,0,1],
[4935,1,1],[4935,2,2],[4935,3,7],[4935,4,5],[4935,5,7],[4935,6,1],
[4935,9,2],[4935,10,2],[4935,13,1],[4935,20,1],[4935,21,3],
[4935,22,2],[4935,23,6],[4935,24,12],[4935,25,12],[4935,26,3],
[4935,27,7],[4935,28,65],[4935,31,3],[4935,32,9],[4935,33,3],
[4935,35,9],[4935,37,1],[4935,38,7],[4935,40,15],[4935,42,3],
[4935,43,2],[4935,44,7],[4935,45,1],[4935,47,4],[4935,48,3],
[4935,49,1],[4935,50,5],[4935,51,1],[4935,56,3],[4935,59,2],
[4935,61,4],[4935,62,2],[4935,63,102],[4935,65,1],[4935,66,5],
[4935,68,17],[4935,69,6],[4935,70,22],[4935,72,10],[4935,73,1],
[4935,74,105],[4935,75,69],[4935,76,120],[4936,28,4],[4936,32,1],
[4936,63,1],[4937,4,1],[4937,28,1],[4938,3,1],[4938,21,1],[4939,23,1],
[4939,24,1],[4939,28,1],[4940,28,1],[4940,35,1],[4941,39,1],
[4941,41,1],[4941,62,1],[4942,0,1],[4942,1,3],[4942,2,2],[4942,3,15],

[4942,5,3],[4942,8,1],[4942,9,2],[4942,15,1],[4942,16,1],[4942,18,2],
[4942,19,1],[4942,20,1],[4942,21,3],[4942,22,1],[4942,23,9],
[4942,24,7],[4942,25,9],[4942,26,9],[4942,27,4],[4942,28,4],
[4942,31,2],[4942,32,98],[4942,33,2],[4942,35,3],[4942,37,1],
[4942,38,1],[4942,40,2],[4942,42,4],[4942,43,3],[4942,44,2],
[4942,46,1],[4942,47,8],[4942,51,2],[4942,52,1],[4942,55,1],
[4942,56,18],[4942,58,1],[4942,60,1],[4942,61,1],[4942,62,3],
[4942,63,3],[4942,64,2],[4942,66,1],[4942,72,1],[4942,74,6],
[4942,75,1],[4942,76,1],[4943,2,1],[4943,28,1],[4944,15,1],
[4944,17,1],[4944,53,2],[4945,22,1],[4945,27,1],[4945,35,2],
[4945,40,1],[4945,52,1],[4945,63,17],[4945,74,13],[4945,75,14],
[4945,76,25],[4946,21,1],[4946,23,1],[4946,24,1],[4946,27,1],
[4946,28,4],[4946,31,2],[4946,35,1],[4946,50,1],[4946,74,1],
[4947,1,5],[4947,2,7],[4947,3,11],[4947,4,1],[4947,5,3],[4947,6,3],
[4947,13,3],[4947,15,1],[4947,16,1],[4947,18,1],[4947,19,2],
[4947,21,1],[4947,22,1],[4947,23,15],[4947,24,24],[4947,25,138],
[4947,26,7],[4947,27,6],[4947,28,9],[4947,30,2],[4947,31,5],
[4947,32,3],[4947,33,10],[4947,35,7],[4947,37,3],[4947,40,3],
[4947,42,7],[4947,43,19],[4947,44,3],[4947,47,2],[4947,48,1],
[4947,50,2],[4947,52,1],[4947,53,2],[4947,55,2],[4947,56,3],
[4947,58,1],[4947,59,1],[4947,60,1],[4947,61,1],[4947,62,3],
[4947,63,3],[4947,65,3],[4947,66,1],[4947,67,1],[4947,68,3],
[4947,70,3],[4947,72,4],[4947,74,5],[4947,75,1],[4947,76,5],
[4947,77,1],[4948,25,1],[4948,27,1],[4949,2,6],[4949,3,1],[4949,5,3],
[4949,15,5],[4949,22,1],[4949,24,3],[4949,26,3],[4949,27,4],
[4949,28,1],[4949,31,1],[4949,32,2],[4949,37,4],[4949,38,3],
[4949,42,2],[4949,43,1],[4949,46,3],[4949,52,1],[4949,64,1],
[4949,68,1],[4949,72,1],[4950,1,7],[4950,2,1],[4950,5,7],[4950,6,2],
[4950,12,1],[4950,19,1],[4950,21,2],[4950,22,1],[4950,26,1],
[4950,27,1],[4950,33,4],[4950,39,2],[4950,52,1],[4950,77,1],
[4951,20,1],[4951,23,3],[4951,24,2],[4951,28,3],[4951,30,2],
[4951,33,1],[4951,35,1],[4951,43,1],[4952,6,1],[4952,56,8],[4953,0,2],
[4953,1,7],[4953,2,4],[4953,3,1],[4953,4,1],[4953,5,5],[4953,6,1],
[4953,7,2],[4953,8,2],[4953,9,5],[4953,10,3],[4953,12,5],[4953,13,3],
[4953,14,1],[4953,15,5],[4953,16,2],[4953,17,1],[4953,18,1],
[4953,19,4],[4953,20,4],[4953,21,2],[4953,22,3],[4953,23,6],
[4953,24,5],[4953,25,1],[4953,26,5],[4953,27,3],[4953,28,27],
[4953,29,2],[4953,30,2],[4953,31,3],[4953,32,7],[4953,33,2],
[4953,34,1],[4953,35,3],[4953,36,1],[4953,38,1],[4953,39,2],
[4953,40,1],[4953,41,1],[4953,42,7],[4953,43,1],[4953,44,4],
[4953,45,1],[4953,46,1],[4953,47,4],[4953,48,2],[4953,49,2],
[4953,50,5],[4953,52,1],[4953,53,3],[4953,54,1],[4953,55,3],
[4953,56,1],[4953,58,2],[4953,59,1],[4953,60,1],[4953,61,3],
[4953,62,5],[4953,63,2],[4953,65,3],[4953,66,5],[4953,70,8],
[4953,71,1],[4953,72,3],[4953,73,1],[4953,74,5],[4953,75,1],
[4953,76,6],[4953,77,2],[4953,78,1],[4954,5,1],[4954,28,3],
[4954,50,1],[4954,69,1],[4955,2,1],[4955,12,3],[4955,23,1],
[4956,51,4],[4956,68,2],[4957,8,1],[4957,27,2],[4957,35,2],[4958,5,1],
[4958,23,1],[4958,28,1],[4959,3,7],[4959,48,1],[4959,70,1],
[4960,24,1],[4960,25,1],[4961,15,1],[4961,35,1],[4962,2,2],

[4962,62,1],[4963,31,3],[4964,15,1],[4964,19,1],[4964,20,1],
[4964,23,1],[4964,38,1],[4964,45,2],[4964,46,1],[4964,49,1],
[4964,65,1],[4965,25,1],[4965,31,1],[4965,33,1],[4965,44,1],
[4965,50,1],[4965,63,1],[4965,70,2],[4965,74,1],[4966,24,1],
[4966,42,1],[4966,51,3],[4967,26,1],[4967,33,1],[4968,37,2],
[4969,9,1],[4969,64,1],[4970,3,1],[4970,28,2],[4971,3,1],[4971,4,1],
[4971,15,1],[4971,30,1],[4971,47,1],[4971,56,1],[4971,68,1],
[4972,68,2],[4972,70,1],[4973,50,2],[4974,2,2],[4975,1,2],[4975,62,3],
[4976,0,1],[4976,1,2],[4976,2,10],[4976,3,11],[4976,5,5],[4976,6,1],
[4976,7,1],[4976,8,3],[4976,9,2],[4976,10,7],[4976,11,2],[4976,12,3],
[4976,13,11],[4976,14,3],[4976,15,4],[4976,16,3],[4976,17,1],
[4976,19,6],[4976,20,9],[4976,21,9],[4976,22,12],[4976,23,58],
[4976,24,12],[4976,25,10],[4976,26,11],[4976,27,29],[4976,28,17],
[4976,30,10],[4976,31,19],[4976,32,22],[4976,33,5],[4976,34,4],
[4976,35,16],[4976,36,2],[4976,37,1],[4976,38,1],[4976,39,4],
[4976,40,2],[4976,41,3],[4976,42,6],[4976,43,23],[4976,45,2],
[4976,46,1],[4976,47,8],[4976,48,10],[4976,49,1],[4976,50,1],
[4976,51,1],[4976,53,2],[4976,55,4],[4976,59,10],[4976,60,1],
[4976,62,2],[4976,64,3],[4976,65,3],[4976,67,1],[4976,68,1],
[4976,70,1],[4976,72,3],[4976,74,1],[4976,76,1],[4976,77,2],
[4976,78,2],[4977,35,3],[4977,44,2],[4977,66,1],[4978,2,1],
[4978,30,1],[4978,33,1],[4979,5,1],[4979,28,3],[4980,28,1],
[4980,33,6],[4981,28,1],[4981,44,1],[4981,70,3],[4982,3,1],
[4982,47,3],[4982,48,3],[4983,15,1],[4983,55,2],[4984,28,1],
[4984,32,1],[4985,10,1],[4985,20,2],[4985,24,1],[4986,22,1],
[4986,45,3],[4987,4,2],[4987,27,1],[4987,50,2],[4987,56,1],
[4987,69,1],[4987,70,1],[4988,1,1],[4988,2,1],[4988,6,1],[4988,24,1],
[4988,28,4],[4988,30,1],[4988,33,1],[4988,35,1],[4989,23,1],
[4989,26,1],[4990,4,1],[4990,48,1],[4991,27,1],[4991,32,2],
[4991,38,2],[4991,48,1],[4991,50,2],[4992,2,1],[4992,3,1],[4993,5,2],
[4993,7,1],[4993,10,1],[4993,19,1],[4993,21,2],[4993,31,1],
[4993,38,2],[4993,42,1],[4993,50,1],[4993,63,1],[4993,72,1],
[4994,8,4],[4994,68,1],[4994,69,1],[4995,60,2],[4996,39,1],
[4996,56,1],[4997,0,1],[4997,1,1],[4997,8,8],[4997,30,4],[4997,39,2],
[4997,45,1],[4998,28,1],[4998,70,1],[4999,2,2],[4999,5,1],[4999,6,1],
[4999,21,1],[4999,22,1],[4999,23,3],[4999,24,4],[4999,26,1],
[4999,27,1],[4999,31,2],[4999,32,2],[4999,33,1],[5000,23,1],
[5000,26,2],[5000,28,1],[5001,2,9],[5001,31,2],[5002,1,4],[5002,7,4],
[5002,8,2],[5002,9,7],[5002,10,4],[5002,11,1],[5002,12,2],[5002,13,3],
[5002,14,1],[5002,15,8],[5002,16,4],[5002,17,3],[5002,18,1],
[5002,19,3],[5002,20,6],[5002,29,3],[5002,30,1],[5002,31,1],
[5002,32,1],[5002,34,2],[5002,45,1],[5002,46,1],[5002,49,1],
[5002,50,1],[5002,53,3],[5002,54,3],[5002,55,4],[5002,56,1],
[5002,57,1],[5002,58,1],[5002,59,1],[5002,65,1],[5002,67,3],
[5002,73,3],[5002,77,2],[5003,27,4],[5003,31,1],[5003,42,1],
[5004,2,1],[5004,3,1],[5004,27,2],[5004,31,1],[5004,70,1],[5005,28,1],
[5005,42,1],[5005,59,2],[5006,35,2],[5006,69,1],[5007,9,5],
[5007,28,1],[5008,40,1],[5008,63,9],[5008,66,1],[5008,72,1],
[5008,74,7],[5008,75,5],[5008,76,9],[5009,24,1],[5009,42,11],
[5009,44,1],[5010,28,1],[5010,44,1],[5010,47,1],[5010,50,2],

[5010,53,1],[5010,55,1],[5011,23,1],[5011,25,1],[5011,28,1],
[5011,42,17],[5012,16,1],[5012,20,4],[5012,31,1],[5012,53,1],
[5013,37,1],[5013,71,1],[5014,2,14],[5014,24,1],[5014,66,1],
[5014,70,1],[5014,74,1],[5015,39,2],[5016,10,1],[5016,67,1],
[5017,28,1],[5017,66,1],[5018,2,2],[5019,20,1],[5019,28,1],
[5020,26,1],[5020,28,1],[5021,3,2],[5022,2,1],[5022,3,1],[5022,13,2],
[5022,24,1],[5022,29,1],[5022,31,1],[5022,35,1],[5022,45,1],
[5023,63,1],[5023,75,1],[5024,25,1],[5024,26,1],[5024,27,1],
[5025,20,1],[5025,28,1],[5025,56,1],[5026,23,2],[5027,28,2],
[5027,37,1],[5028,46,1],[5028,60,1],[5029,47,1],[5029,56,1],
[5030,9,1],[5030,12,1],[5030,23,1],[5030,28,1],[5030,30,1],
[5030,42,2],[5031,3,1],[5031,22,2],[5031,23,2],[5031,25,1],
[5031,39,1],[5031,70,1],[5032,1,1],[5032,29,1],[5033,12,1],
[5033,18,1],[5033,19,1],[5033,53,1],[5034,22,2],[5034,26,1],
[5034,35,1],[5034,43,4],[5034,62,1],[5035,28,2],[5036,8,1],[5036,9,2],
[5036,13,1],[5036,15,1],[5036,16,1],[5036,22,1],[5036,23,1],
[5036,54,1],[5036,60,1],[5037,2,4],[5037,3,2],[5037,5,1],[5037,6,1],
[5037,23,1],[5037,27,1],[5037,30,1],[5037,31,1],[5037,42,1],
[5038,2,4],[5038,4,1],[5038,12,2],[5038,21,1],[5038,24,1],[5038,31,3],
[5038,32,1],[5038,39,1],[5038,47,1],[5039,3,1],[5039,5,4],[5039,22,1],
[5039,23,3],[5039,24,4],[5039,25,1],[5039,26,1],[5039,27,1],
[5039,32,2],[5039,35,1],[5039,47,1],[5040,33,4],[5041,2,3],[5041,3,2],
[5041,18,1],[5041,23,2],[5041,28,1],[5041,42,1],[5042,2,1],
[5042,21,1],[5042,24,2],[5042,26,2],[5042,31,4],[5042,32,3],
[5043,19,2],[5043,23,1],[5043,25,2],[5043,28,4],[5043,30,1],
[5043,35,18],[5043,43,1],[5043,52,80],[5044,3,2],[5044,13,1],
[5044,22,1],[5044,24,1],[5044,30,1],[5044,38,1],[5044,46,2],
[5044,56,1],[5044,59,1],[5044,60,1],[5044,72,1],[5045,28,4],
[5046,8,1],[5046,17,2],[5046,53,1],[5047,2,1],[5047,26,1],[5047,43,1],
[5048,31,4],[5049,2,2],[5050,25,2],[5050,27,1],[5050,29,1],
[5050,37,1],[5050,43,1],[5051,2,1],[5051,5,1],[5051,50,2],[5051,51,1],
[5051,74,14],[5052,23,1],[5052,26,1],[5053,28,2],[5054,31,2],
[5055,9,1],[5055,37,1],[5056,21,1],[5056,42,2],[5057,3,2],[5058,1,1],
[5058,3,1],[5058,5,1],[5058,10,2],[5058,16,1],[5058,27,2],[5058,28,1],
[5058,37,1],[5058,40,1],[5058,63,31],[5058,69,2],[5058,70,4],
[5058,74,31],[5058,75,19],[5058,76,56],[5059,0,1],[5059,1,26],
[5059,5,1],[5059,7,1],[5059,12,1],[5059,13,10],[5059,17,1],
[5059,19,3],[5059,20,2],[5059,29,2],[5059,36,1],[5059,39,2],
[5059,46,2],[5059,53,1],[5059,54,1],[5059,59,3],[5059,65,1],
[5059,77,3],[5060,3,2],[5061,3,1],[5061,26,1],[5061,27,1],[5062,28,1],
[5062,31,1],[5063,3,2],[5063,4,1],[5063,22,1],[5063,23,2],[5063,27,1],
[5063,31,3],[5063,35,1],[5063,38,10],[5063,44,48],[5063,47,1],
[5063,50,1],[5063,66,52],[5063,75,1],[5064,2,2],[5064,6,1],
[5064,21,1],[5064,22,3],[5064,23,7],[5064,24,3],[5064,26,3],
[5064,27,10],[5064,28,2],[5064,32,6],[5064,33,2],[5064,35,6],
[5064,38,2],[5064,43,1],[5064,48,1],[5064,52,1],[5064,56,1],
[5064,70,1],[5065,28,3],[5065,53,1],[5066,37,2],[5067,25,1],
[5067,51,3],[5068,23,1],[5068,26,1],[5068,28,1],[5068,31,1],
[5068,34,4],[5068,39,1],[5069,26,1],[5069,43,1],[5070,6,1],
[5070,32,2],[5070,76,1],[5071,1,1],[5071,4,1],[5071,8,1],[5071,10,1],

[5071,15,2],[5071,17,1],[5071,20,1],[5071,26,1],[5071,27,1],
[5071,53,3],[5071,55,2],[5071,63,1],[5071,66,1],[5072,24,1],
[5072,28,1],[5073,47,6],[5073,74,1],[5074,27,4],[5075,6,1],
[5075,27,1],[5075,35,2],[5076,20,1],[5076,28,1],[5076,33,1],
[5077,3,4],[5077,24,3],[5077,25,1],[5077,27,2],[5077,28,4],
[5077,35,1],[5077,76,1],[5078,42,1],[5078,45,1],[5079,20,1],
[5079,21,3],[5079,27,1],[5079,31,17],[5079,32,4],[5079,34,1],
[5079,36,1],[5079,39,5],[5079,40,1],[5079,42,2],[5080,2,1],[5080,5,1],
[5080,31,2],[5080,38,4],[5080,44,23],[5080,50,2],[5080,66,18],
[5080,75,2],[5081,31,2],[5082,4,3],[5082,15,2],[5082,16,1],
[5082,30,1],[5082,38,2],[5082,44,29],[5082,50,4],[5082,62,1],
[5082,66,25],[5082,74,1],[5083,22,1],[5083,27,3],[5084,1,2],
[5084,12,1],[5084,26,1],[5084,35,1],[5084,42,1],[5084,51,1],
[5084,56,1],[5084,69,2],[5084,70,1],[5085,2,4],[5085,3,1],[5085,4,1],
[5085,7,2],[5085,8,1],[5085,9,2],[5085,13,1],[5085,17,1],[5085,19,2],
[5085,20,1],[5085,21,1],[5085,22,1],[5085,23,1],[5085,24,2],
[5085,26,1],[5085,29,1],[5085,37,1],[5085,42,1],[5085,43,3],
[5085,50,1],[5085,53,2],[5086,1,9],[5086,2,1],[5086,3,1],[5086,5,1],
[5086,6,2],[5086,7,1],[5086,9,1],[5086,10,1],[5086,12,3],[5086,13,4],
[5086,15,1],[5086,20,5],[5086,22,3],[5086,26,2],[5086,27,10],
[5086,28,4],[5086,29,1],[5086,31,1],[5086,32,8],[5086,33,2],
[5086,35,6],[5086,42,5],[5086,43,4],[5086,44,1],[5086,59,6],
[5086,62,5],[5086,69,1],[5086,70,1],[5086,71,1],[5086,76,1],
[5086,77,1],[5086,78,1],[5087,1,16],[5087,2,2],[5087,8,1],[5087,9,1],
[5087,12,1],[5087,13,15],[5087,15,1],[5087,17,1],[5087,20,8],
[5087,21,2],[5087,23,2],[5087,25,5],[5087,26,2],[5087,27,1],
[5087,28,1],[5087,29,1],[5087,31,28],[5087,32,1],[5087,33,3],
[5087,34,2],[5087,35,1],[5087,39,5],[5087,40,1],[5087,42,1],
[5087,43,1],[5087,47,1],[5087,53,1],[5087,55,1],[5088,30,1],
[5088,38,1],[5088,71,1],[5089,24,2],[5089,48,1],[5090,1,4],[5090,9,1],
[5090,10,3],[5090,11,1],[5090,12,2],[5090,13,3],[5090,14,1],
[5090,15,1],[5090,17,1],[5090,18,1],[5090,20,1],[5090,37,1],
[5090,41,1],[5090,46,1],[5090,54,1],[5090,55,1],[5090,57,1],
[5090,60,1],[5090,64,1],[5090,65,2],[5090,77,1],[5091,1,1],
[5091,12,1],[5091,28,5],[5091,29,1],[5092,6,3],[5092,56,1],
[5092,68,2],[5092,69,1],[5092,70,1],[5093,1,2],[5093,12,1],
[5093,73,2],[5094,9,1],[5094,75,1],[5094,78,1],[5095,2,1],[5095,3,8],
[5095,6,5],[5095,35,1],[5095,48,2],[5095,54,1],[5095,64,1],
[5095,66,1],[5096,31,1],[5096,35,1],[5097,23,2],[5097,24,2],
[5097,28,3],[5097,32,3],[5097,42,1],[5098,5,3],[5098,24,1],
[5098,28,1],[5098,33,2],[5098,52,1],[5099,0,1],[5099,55,1],
[5100,28,11],[5100,40,1],[5100,74,1],[5101,27,1],[5101,28,1],
[5101,35,1],[5102,12,1],[5102,49,1],[5103,2,30],[5104,25,4],
[5105,34,1],[5105,45,1],[5106,24,13],[5107,3,1],[5107,31,1],
[5108,1,1],[5108,3,1],[5108,4,22],[5108,5,7],[5108,9,2],[5108,12,1],
[5108,13,1],[5108,17,3],[5108,19,1],[5108,21,1],[5108,22,5],
[5108,27,2],[5108,28,3],[5108,32,1],[5108,37,1],[5108,38,12],
[5108,40,1],[5108,44,26],[5108,50,13],[5108,55,1],[5108,56,1],
[5108,59,1],[5108,66,24],[5108,69,1],[5108,70,1],[5108,71,1],
[5108,73,1],[5108,76,2],[5108,77,1],[5109,3,1],[5109,31,1],

[5109,62,1],[5110,3,1],[5110,27,1],[5111,23,2],[5111,24,2],
[5111,28,1],[5111,32,1],[5111,35,1],[5111,47,1],[5112,9,1],
[5112,13,1],[5112,20,1],[5112,36,1],[5112,37,1],[5112,41,1],
[5112,45,2],[5112,71,1],[5113,21,1],[5113,23,1],[5113,24,1],
[5113,26,1],[5113,28,1],[5113,35,1],[5114,22,1],[5114,24,22],
[5114,31,1],[5114,40,1],[5114,41,1],[5114,47,1],[5114,56,13],
[5114,72,1],[5114,74,1],[5115,2,3],[5116,7,1],[5116,12,1],[5117,0,1],
[5117,1,1],[5117,8,4],[5117,16,1],[5117,18,1],[5117,20,1],
[5117,28,130],[5117,34,2],[5117,37,1],[5117,41,1],[5117,53,1],
[5117,57,1],[5117,64,1],[5117,70,1],[5117,72,2],[5118,9,1],
[5118,15,1],[5118,19,1],[5118,30,1],[5118,65,1],[5118,77,1],
[5119,30,1],[5119,34,1],[5119,64,1],[5120,6,1],[5120,22,2],
[5120,27,3],[5120,35,1],[5120,43,1],[5120,68,1],[5121,10,1],
[5121,15,1],[5121,17,1],[5121,19,1],[5121,34,1],[5121,41,1],
[5122,5,1],[5122,25,1],[5122,28,1],[5122,62,1],[5122,70,1],[5123,5,1],
[5123,21,1],[5123,23,1],[5123,32,2],[5123,35,1],[5123,56,1],
[5123,67,1],[5123,77,1],[5124,14,1],[5124,73,1],[5125,5,1],
[5125,69,1],[5126,37,4],[5127,28,3],[5127,38,1],[5128,28,22],
[5129,54,1],[5129,78,1],[5130,2,1],[5130,56,1],[5131,28,76],
[5132,27,3],[5132,31,2],[5133,2,2],[5133,61,1],[5134,1,1],[5134,25,1],
[5134,28,1],[5134,35,2],[5135,2,1],[5135,23,1],[5135,26,1],
[5136,15,1],[5136,53,1],[5137,1,1],[5137,5,2],[5137,17,5],[5137,24,2],
[5137,25,3],[5137,26,1],[5137,27,3],[5137,30,1],[5137,31,4],
[5137,32,1],[5137,35,2],[5137,42,1],[5137,48,1],[5137,50,1],
[5137,52,1],[5137,53,1],[5137,54,1],[5137,59,1],[5138,9,1],
[5138,53,1],[5139,4,1],[5139,50,1],[5139,53,1],[5140,23,1],
[5140,47,2],[5141,1,4],[5142,31,9],[5142,32,2],[5142,35,2],[5143,4,1],
[5143,6,3],[5143,22,8],[5143,23,11],[5143,24,3],[5143,26,7],
[5143,27,2],[5143,28,1],[5143,31,2],[5143,32,3],[5143,35,3],
[5143,39,1],[5143,42,1],[5143,43,9],[5143,56,1],[5143,63,1],
[5143,72,1],[5144,2,1],[5144,6,1],[5144,12,1],[5144,13,2],[5144,16,1],
[5144,20,4],[5144,23,3],[5144,27,4],[5144,28,5],[5144,30,3],
[5144,31,3],[5144,32,1],[5144,33,4],[5144,34,1],[5144,35,1],
[5144,38,1],[5144,39,1],[5144,40,1],[5144,42,5],[5144,47,1],
[5144,48,1],[5144,62,1],[5144,74,1],[5145,53,1],[5145,55,1],
[5146,62,1],[5146,70,1],[5147,1,2],[5147,2,1],[5147,44,1],[5148,2,2],
[5149,12,1],[5149,24,1],[5150,31,2],[5150,33,1],[5151,23,2],
[5151,24,10],[5151,43,1],[5151,47,1],[5152,1,2],[5153,56,3],
[5154,31,4],[5155,15,1],[5155,53,2],[5156,3,2],[5156,6,1],[5156,23,1],
[5157,9,1],[5157,36,1],[5158,2,2],[5158,8,8],[5158,12,2],[5158,15,1],
[5158,17,1],[5158,22,1],[5158,25,2],[5158,27,1],[5158,28,1],
[5158,31,2],[5158,32,4],[5158,34,1],[5158,35,1],[5158,43,1],
[5158,45,2],[5158,53,1],[5159,0,1],[5159,1,2],[5159,12,1],[5159,15,1],
[5159,39,1],[5160,32,2],[5160,35,2],[5160,43,2],[5160,68,1],
[5161,2,123],[5161,6,1],[5161,21,1],[5161,28,2],[5161,50,1],
[5162,2,1],[5162,18,1],[5163,28,1],[5163,32,1],[5163,75,1],
[5164,47,1],[5164,56,1],[5165,58,1],[5165,62,1],[5165,77,1],
[5166,17,1],[5166,19,1],[5166,25,1],[5166,28,1],[5166,50,1],
[5166,53,2],[5166,57,1],[5167,30,1],[5167,44,1],[5168,33,6],
[5169,0,1],[5169,1,1],[5169,8,1],[5169,9,4],[5169,10,7],[5169,11,5],

[5169,12,1],[5169,14,3],[5169,16,4],[5169,18,2],[5169,19,8],
[5169,20,2],[5169,24,1],[5169,27,1],[5169,31,1],[5169,32,1],
[5169,33,1],[5169,34,1],[5169,37,2],[5169,39,1],[5169,40,6],
[5169,41,2],[5169,44,1],[5169,45,3],[5169,46,3],[5169,53,2],
[5169,54,4],[5169,57,5],[5169,58,2],[5169,59,3],[5169,60,2],
[5169,64,5],[5169,65,2],[5169,67,2],[5169,70,6],[5169,71,1],
[5169,72,2],[5169,73,4],[5169,75,1],[5169,77,1],[5169,78,2],
[5170,28,1],[5170,44,1],[5171,23,2],[5171,27,1],[5171,31,2],
[5172,22,1],[5172,43,2],[5172,48,1],[5173,38,1],[5173,55,1],
[5174,1,1],[5174,12,1],[5174,39,1],[5175,21,2],[5175,22,1],
[5175,27,3],[5175,35,1],[5175,66,1],[5175,69,2],[5176,8,1],
[5176,10,3],[5176,14,1],[5176,15,1],[5176,16,1],[5176,20,1],
[5176,29,1],[5176,46,3],[5176,65,2],[5176,77,1],[5177,27,2],
[5178,8,1],[5178,12,1],[5178,16,1],[5178,53,1],[5178,55,1],
[5178,59,1],[5179,2,1],[5179,21,1],[5179,22,1],[5179,40,1],
[5179,42,3],[5179,70,1],[5180,1,3],[5180,2,169],[5180,3,7],[5180,8,1],
[5180,9,2],[5180,10,1],[5180,12,1],[5180,13,1],[5180,16,1],
[5180,19,2],[5180,20,4],[5180,21,1],[5180,24,1],[5180,28,7],
[5180,30,2],[5180,35,1],[5180,40,1],[5180,41,2],[5180,42,1],
[5180,45,1],[5180,47,1],[5180,55,1],[5180,60,1],[5180,62,1],
[5180,69,2],[5180,72,1],[5181,6,1],[5181,45,1],[5182,5,2],[5182,28,1],
[5183,40,2],[5183,72,1],[5184,1,2],[5184,2,14],[5184,3,30],[5184,4,1],
[5184,5,17],[5184,6,3],[5184,7,1],[5184,8,7],[5184,12,1],[5184,18,1],
[5184,19,1],[5184,20,8],[5184,21,44],[5184,22,126],[5184,23,71],
[5184,24,113],[5184,25,23],[5184,26,40],[5184,27,150],[5184,28,41],
[5184,30,6],[5184,31,39],[5184,32,28],[5184,33,41],[5184,34,5],
[5184,35,47],[5184,36,2],[5184,38,9],[5184,39,1],[5184,42,25],
[5184,43,55],[5184,47,12],[5184,48,3],[5184,56,5],[5184,59,1],
[5184,60,1],[5184,62,2],[5184,65,2],[5184,72,1],[5184,73,1],
[5185,5,1],[5185,28,2],[5185,76,7],[5186,13,1],[5186,15,3],
[5186,17,1],[5186,37,1],[5186,53,1],[5186,55,2],[5187,32,1],
[5187,66,1],[5188,28,9],[5188,53,1],[5189,23,2],[5189,28,2],
[5189,35,1],[5190,22,1],[5190,27,1],[5190,38,1],[5191,2,2],[5192,3,1],
[5192,17,2],[5192,22,1],[5192,28,1],[5192,53,3],[5193,24,1],
[5193,56,2],[5194,6,1],[5194,51,3],[5195,25,1],[5195,26,3],
[5195,33,2],[5196,28,1],[5196,63,1],[5196,75,1],[5197,74,2],
[5197,76,1],[5198,3,2],[5198,33,2],[5198,47,2],[5199,32,2],
[5199,48,1],[5200,5,3],[5201,25,3],[5201,45,1],[5202,3,1],[5202,10,1],
[5203,26,1],[5203,27,2],[5204,1,2],[5204,10,1],[5204,11,1],
[5204,15,29],[5204,16,1],[5204,17,3],[5204,20,1],[5204,34,1],
[5204,45,1],[5204,55,18],[5204,58,1],[5204,63,1],[5204,67,1],
[5204,71,2],[5204,77,1],[5204,79,1],[5205,22,1],[5205,27,1],
[5206,9,5],[5206,11,1],[5206,15,2],[5206,16,1],[5206,17,1],
[5206,19,1],[5206,28,4],[5206,55,4],[5206,60,1],[5207,24,1],
[5207,28,1],[5207,35,2],[5207,39,1],[5208,4,1],[5208,5,1],[5208,22,2],
[5208,47,1],[5209,22,1],[5209,24,1],[5209,32,2],[5209,52,1],
[5210,2,1],[5210,63,1],[5210,74,2],[5210,75,3],[5210,76,1],
[5211,25,3],[5212,15,2],[5212,53,2],[5213,13,2],[5214,4,1],
[5214,12,1],[5214,17,2],[5214,19,1],[5214,50,1],[5214,53,1],
[5214,55,1],[5214,79,1],[5215,13,2],[5215,26,1],[5215,31,1],

[5215,33,2],[5216,22,1],[5216,23,15],[5216,27,6],[5216,35,1],
[5216,44,1],[5216,51,1],[5216,62,1],[5216,66,1],[5216,69,1],
[5217,4,5],[5217,50,9],[5218,5,2],[5218,20,1],[5219,2,2],[5219,3,1],
[5219,31,1],[5220,38,3],[5221,22,6],[5221,27,18],[5221,28,5],
[5221,34,6],[5221,35,13],[5221,61,1],[5222,1,1],[5222,18,1],
[5222,19,1],[5222,46,1],[5222,67,2],[5223,28,3],[5224,44,4],
[5224,66,2],[5225,8,2],[5225,20,1],[5225,34,1],[5225,73,1],
[5226,16,1],[5226,28,1],[5226,32,1],[5226,59,5],[5227,0,14],
[5227,1,32],[5227,2,38],[5227,3,38],[5227,4,7],[5227,5,10],[5227,6,2],
[5227,7,27],[5227,8,6],[5227,9,12],[5227,10,16],[5227,11,3],
[5227,12,436],[5227,13,69],[5227,14,5],[5227,15,13],[5227,16,11],
[5227,17,5],[5227,18,7],[5227,19,17],[5227,20,26],[5227,21,2],
[5227,22,8],[5227,23,4],[5227,24,16],[5227,25,9],[5227,26,4],
[5227,27,134],[5227,28,40],[5227,29,362],[5227,30,38],[5227,31,3],
[5227,32,26],[5227,33,11],[5227,34,7],[5227,35,19],[5227,36,8],
[5227,37,7],[5227,38,3],[5227,39,25],[5227,40,2],[5227,41,19],
[5227,42,3],[5227,43,2],[5227,44,7],[5227,45,18],[5227,46,2],
[5227,47,4],[5227,48,1],[5227,49,3],[5227,50,11],[5227,51,2],
[5227,52,2],[5227,53,8],[5227,54,4],[5227,55,8],[5227,56,4],
[5227,57,8],[5227,58,11],[5227,59,3],[5227,60,1],[5227,62,1052],
[5227,63,2],[5227,64,1],[5227,65,11],[5227,66,10],[5227,67,10],
[5227,68,16],[5227,69,3],[5227,70,15],[5227,71,4],[5227,73,17],
[5227,74,9],[5227,75,3],[5227,76,6],[5227,77,8],[5227,78,1],
[5228,0,1],[5228,11,1],[5228,19,1],[5229,2,2],[5229,3,2],[5229,9,1],
[5229,12,1],[5229,19,1],[5229,20,1],[5229,23,1],[5229,24,1],
[5229,28,1],[5229,30,3],[5229,31,1],[5229,46,1],[5229,47,2],
[5230,28,1],[5230,35,1],[5231,1,2],[5231,10,1],[5231,11,1],
[5231,19,1],[5231,20,1],[5231,27,1],[5231,30,1],[5231,35,1],
[5231,39,2],[5231,49,1],[5231,55,1],[5231,66,1],[5232,9,1],
[5232,24,1],[5233,27,1],[5233,32,1],[5233,33,1],[5234,20,3],
[5235,10,1],[5235,45,1],[5235,65,1],[5236,2,10],[5236,3,3],[5236,4,1],
[5236,5,1],[5236,14,1],[5236,15,2],[5236,20,3],[5236,22,1],
[5236,23,2],[5236,24,2],[5236,26,2],[5236,28,2],[5236,31,1],
[5236,33,1],[5236,37,2],[5236,38,1],[5236,39,1],[5236,42,2],
[5236,43,1],[5236,46,1],[5236,47,2],[5236,53,2],[5236,56,1],
[5236,57,1],[5236,59,3],[5236,60,2],[5236,64,1],[5236,67,1],
[5236,69,1],[5237,20,1],[5237,22,1],[5238,23,1],[5238,35,1],
[5239,8,6],[5240,1,1],[5240,9,1],[5240,11,1],[5240,74,1],[5241,0,1],
[5241,23,2],[5242,42,6],[5242,55,1],[5243,44,1],[5243,66,1],
[5244,8,1],[5244,28,2],[5245,28,1],[5245,42,1],[5246,3,1],[5246,6,1],
[5246,8,1],[5246,27,2],[5246,35,1],[5246,50,1],[5246,56,2],
[5247,21,1],[5247,23,1],[5247,24,3],[5247,25,1],[5247,26,4],
[5247,27,2],[5247,32,1],[5247,43,1],[5248,24,2],[5248,40,1],
[5248,69,1],[5248,76,1],[5249,50,1],[5249,53,1],[5249,55,1],
[5250,2,1],[5250,3,1],[5250,4,2],[5250,5,1],[5250,6,1],[5250,8,1],
[5250,9,1],[5250,12,1],[5250,16,1],[5250,22,3],[5250,23,2],
[5250,24,1],[5250,25,1],[5250,27,9],[5250,28,35],[5250,30,45],
[5250,31,1],[5250,32,2],[5250,33,1],[5250,35,11],[5250,38,3],
[5250,40,3],[5250,42,1],[5250,43,1],[5250,50,3],[5250,52,10],
[5250,57,2],[5250,65,2],[5250,66,2],[5250,70,2],[5251,5,2],

[5251,28,2],[5251,35,1],[5251,51,1],[5251,70,2],[5252,20,1],
[5252,42,1],[5253,1,1],[5253,19,2],[5253,64,3],[5253,65,1],
[5254,22,1],[5254,28,1],[5255,28,1],[5255,32,1],[5256,2,4],
[5256,12,1],[5256,31,2],[5256,34,2],[5257,2,2],[5257,3,1],[5257,5,1],
[5257,17,1],[5257,22,1],[5257,74,1],[5258,23,1],[5258,24,1],
[5258,62,1],[5259,22,1],[5259,23,2],[5259,24,1],[5259,25,2],
[5259,27,2],[5259,28,1],[5259,33,3],[5259,35,1],[5259,43,1],
[5259,67,1],[5260,24,1],[5260,31,1],[5261,4,1],[5261,50,1],
[5261,56,1],[5262,2,12],[5262,3,2],[5262,4,2],[5262,8,2],[5262,10,1],
[5262,20,1],[5262,27,3],[5262,32,1],[5262,38,1],[5262,39,1],
[5262,60,1],[5262,62,4],[5263,10,1],[5263,60,1],[5264,32,1],
[5264,35,3],[5265,28,3],[5266,53,2],[5267,28,2],[5267,62,1],
[5268,2,1],[5268,33,1],[5268,34,4],[5269,2,1],[5269,20,1],[5270,17,3],
[5270,53,2],[5271,2,2],[5271,3,8],[5271,4,1],[5271,5,2],[5271,6,3],
[5271,15,1],[5271,17,1],[5271,21,5],[5271,22,3],[5271,23,40],
[5271,24,75],[5271,25,26],[5271,26,8],[5271,27,15],[5271,28,22],
[5271,30,1],[5271,31,2],[5271,32,24],[5271,33,4],[5271,34,1],
[5271,35,16],[5271,36,1],[5271,40,1],[5271,42,12],[5271,43,9],
[5271,45,1],[5271,46,1],[5271,47,1],[5271,48,1],[5271,51,1],
[5271,52,2],[5271,56,39],[5271,59,1],[5271,60,1],[5271,61,1],
[5271,62,3],[5271,64,1],[5271,66,1],[5271,68,1],[5271,69,1],
[5271,72,1],[5271,74,2],[5271,76,1],[5272,2,14],[5273,2,2],[5274,9,2],
[5275,26,4],[5276,2,1],[5276,4,75],[5276,5,2],[5276,17,4],[5276,22,1],
[5276,25,1],[5276,28,5],[5276,38,2],[5276,50,80],[5276,53,3],
[5276,66,2],[5277,53,1],[5277,55,1],[5278,3,1],[5278,28,1],[5279,2,1],
[5279,20,1],[5279,28,1],[5279,53,1],[5280,21,1],[5280,33,1],
[5281,28,1],[5281,38,1],[5282,2,4],[5282,6,1],[5282,27,1],[5282,28,1],
[5282,30,1],[5282,31,1],[5282,33,1],[5282,43,1],[5282,59,1],
[5283,2,1],[5283,13,1],[5283,26,1],[5283,31,2],[5284,25,1],
[5284,32,7],[5285,28,4],[5286,3,9],[5286,23,6],[5286,24,3],
[5286,27,1],[5286,28,2],[5286,31,5],[5286,47,1],[5286,48,1],
[5287,44,3],[5288,3,2],[5288,29,1],[5289,28,2],[5290,31,1],
[5290,32,1],[5290,42,1],[5290,43,1],[5291,0,10],[5291,1,1],[5291,2,3],
[5291,16,1],[5291,55,1],[5292,18,1],[5292,29,3],[5292,45,3],
[5292,46,1],[5292,53,1],[5292,58,1],[5292,65,1],[5292,67,1],
[5293,4,3],[5293,5,1],[5293,22,1],[5293,23,2],[5293,28,1],[5293,38,2],
[5293,44,1],[5293,47,2],[5294,4,1],[5294,17,1],[5294,27,3],
[5294,60,1],[5294,75,1],[5295,7,1],[5295,28,7],[5295,63,14],
[5295,74,16],[5295,75,1],[5295,76,1],[5296,2,1],[5296,27,1],
[5296,43,3],[5296,70,1],[5297,0,26],[5297,1,231],[5297,2,3],
[5297,3,11],[5297,4,4],[5297,5,10],[5297,6,5],[5297,7,35],[5297,8,56],
[5297,9,264],[5297,10,324],[5297,11,135],[5297,12,162],[5297,13,223],
[5297,14,83],[5297,15,215],[5297,16,227],[5297,17,75],[5297,18,102],
[5297,19,238],[5297,20,214],[5297,21,6],[5297,22,13],[5297,23,20],
[5297,24,18],[5297,25,11],[5297,26,11],[5297,27,86],[5297,28,172],
[5297,29,42],[5297,30,42],[5297,31,11],[5297,32,45],[5297,33,13],
[5297,34,88],[5297,35,39],[5297,36,48],[5297,37,129],[5297,38,12],
[5297,39,90],[5297,40,11],[5297,41,159],[5297,42,9],[5297,43,5],
[5297,44,13],[5297,45,158],[5297,46,72],[5297,47,4],[5297,48,20],
[5297,49,67],[5297,50,5],[5297,51,6],[5297,52,3],[5297,53,84],

[5297,54,76],[5297,55,139],[5297,56,6],[5297,57,116],[5297,58,99],
[5297,59,149],[5297,60,29],[5297,61,2],[5297,62,16],[5297,63,4],
[5297,64,37],[5297,65,217],[5297,66,10],[5297,67,109],[5297,68,9],
[5297,69,26],[5297,70,38],[5297,71,82],[5297,72,9],[5297,73,81],
[5297,74,16],[5297,75,7],[5297,76,18],[5297,77,197],[5297,78,31],
[5297,79,1],[5298,5,1],[5298,17,1],[5298,23,1],[5298,24,1],
[5298,26,1],[5298,31,1],[5299,2,1],[5299,8,1],[5299,21,1],[5299,43,1],
[5300,47,2],[5301,9,1],[5301,16,1],[5301,28,1],[5301,34,6],
[5301,57,1],[5301,65,1],[5302,66,2],[5303,27,2],[5303,62,1],
[5303,65,1],[5304,24,2],[5304,25,1],[5304,33,1],[5305,1,1],
[5305,2,19],[5305,3,19],[5305,4,32],[5305,5,16],[5305,6,9],[5305,9,1],
[5305,10,3],[5305,13,1],[5305,18,1],[5305,19,1],[5305,20,1],
[5305,21,8],[5305,22,18],[5305,23,36],[5305,24,32],[5305,25,16],
[5305,26,24],[5305,27,41],[5305,28,184],[5305,31,24],[5305,32,18],
[5305,33,10],[5305,35,31],[5305,36,3],[5305,37,13],[5305,38,31],
[5305,39,1],[5305,40,22],[5305,42,10],[5305,43,12],[5305,44,40],
[5305,47,12],[5305,48,2],[5305,50,38],[5305,51,6],[5305,52,6],
[5305,56,13],[5305,57,1],[5305,61,2],[5305,62,21],[5305,63,12],
[5305,66,37],[5305,68,19],[5305,69,6],[5305,70,38],[5305,72,18],
[5305,74,20],[5305,75,14],[5305,76,19],[5305,77,1],[5305,79,3],
[5306,22,1],[5306,28,1],[5306,43,1],[5307,9,1],[5307,12,1],
[5307,15,9],[5307,17,1],[5307,20,1],[5307,45,1],[5307,53,2],
[5307,55,6],[5308,20,1],[5308,21,1],[5308,22,1],[5308,24,1],
[5308,27,1],[5308,28,1],[5308,31,1],[5308,42,2],[5309,5,1],
[5309,14,1],[5309,15,1],[5309,20,12],[5309,44,1],[5309,55,1],
[5310,28,1],[5310,31,3],[5311,6,1],[5311,51,1],[5312,2,9],[5313,15,1],
[5313,20,1],[5314,1,357],[5314,7,3],[5314,8,1],[5314,9,4],[5314,10,3],
[5314,12,2],[5314,15,3],[5314,16,1],[5314,18,2],[5314,19,1],
[5314,20,3],[5314,34,2],[5314,36,1],[5314,39,1],[5314,41,1],
[5314,42,2],[5314,46,1],[5314,49,1],[5314,57,1],[5314,58,1],
[5314,59,1],[5314,60,1],[5314,65,4],[5314,67,1],[5314,77,1],
[5314,78,1],[5315,4,3],[5315,12,1],[5315,15,1],[5315,22,1],
[5315,39,1],[5315,50,4],[5315,53,4],[5315,55,2],[5316,55,1],
[5316,64,1],[5317,28,2],[5318,23,1],[5318,24,1],[5318,25,1],
[5318,27,3],[5318,28,1],[5318,35,1],[5318,42,2],[5318,43,1],
[5318,70,1],[5319,15,1],[5319,17,1],[5319,53,1],[5320,5,3],
[5320,24,2],[5320,26,1],[5320,27,1],[5320,28,1],[5320,31,2],
[5320,32,5],[5320,33,2],[5320,42,1],[5321,2,3],[5321,28,1],[5322,2,6],
[5322,3,3],[5322,5,3],[5322,6,20],[5322,11,1],[5322,22,9],[5322,23,4],
[5322,24,12],[5322,25,1],[5322,26,2],[5322,27,18],[5322,28,9],
[5322,30,9],[5322,31,4],[5322,32,6],[5322,33,5],[5322,35,16],
[5322,37,1],[5322,38,1],[5322,39,1],[5322,42,1],[5322,43,2],
[5322,45,1],[5322,46,1],[5322,47,8],[5322,48,1],[5322,49,1],
[5322,51,2],[5322,53,2],[5322,56,9],[5322,60,1],[5322,63,2],
[5322,65,1],[5322,68,4],[5322,69,2],[5322,70,5],[5322,73,1],
[5322,74,8],[5322,75,4],[5322,76,2],[5323,3,2],[5323,10,1],
[5323,21,1],[5323,23,2],[5323,26,1],[5323,35,1],[5323,41,1],
[5323,58,1],[5323,60,1],[5324,2,1],[5324,28,1],[5325,28,3],
[5326,24,4],[5327,8,1],[5327,21,1],[5327,23,1],[5327,25,2],
[5327,32,4],[5328,3,2],[5328,5,2],[5328,7,2],[5328,9,1],[5328,10,2],

[5328,17,1],[5328,32,4],[5328,33,1],[5328,38,1],[5328,42,1],
[5328,47,1],[5328,63,1],[5328,75,1],[5329,1,1],[5329,12,1],
[5330,22,1],[5330,23,2],[5330,25,1],[5330,42,2],[5331,1,7],[5331,7,2],
[5331,12,2],[5331,13,4],[5331,17,1],[5331,29,1],[5331,31,20],
[5331,39,4],[5331,50,1],[5332,15,3],[5332,55,2],[5333,15,1],
[5333,27,1],[5334,42,2],[5335,35,2],[5335,62,1],[5336,2,1],
[5336,26,2],[5336,27,1],[5336,30,1],[5336,31,1],[5336,43,1],
[5336,47,1],[5337,5,1],[5337,37,1],[5338,55,2],[5339,33,1],
[5339,47,1],[5340,21,8],[5340,42,1],[5341,3,1],[5341,56,1],
[5341,62,1],[5341,74,1],[5342,28,1],[5342,32,6],[5343,1,1],
[5343,2,10],[5343,20,1],[5343,28,1],[5343,31,20],[5343,42,1],
[5343,53,1],[5344,28,10],[5345,13,1],[5345,14,1],[5345,19,1],
[5345,20,1],[5345,27,1],[5345,55,2],[5345,65,1],[5346,2,2],[5346,3,1],
[5346,4,1],[5346,6,1],[5346,8,5],[5346,9,1],[5346,13,1],[5346,22,3],
[5346,23,1],[5346,24,3],[5346,28,2],[5346,32,5],[5346,47,2],
[5346,50,1],[5346,52,1],[5346,55,1],[5346,56,2],[5346,58,1],
[5346,60,3],[5346,61,1],[5346,64,1],[5346,66,1],[5346,68,9],
[5346,72,2],[5346,76,1],[5346,78,1],[5347,1,1],[5347,34,2],
[5347,36,1],[5347,53,1],[5348,15,1],[5348,55,2],[5349,3,1],[5349,9,1],
[5349,13,1],[5349,23,1],[5349,27,2],[5349,41,1],[5349,62,1],
[5349,70,1],[5350,6,1],[5350,22,1],[5350,23,1],[5350,24,1],
[5350,26,1],[5350,28,2],[5350,42,2],[5350,63,1],[5350,74,1],
[5350,75,1],[5351,25,11],[5351,28,4],[5351,43,1],[5351,56,1],
[5351,63,1],[5352,25,2],[5352,37,2],[5352,60,1],[5353,9,1],
[5353,20,4],[5353,26,1],[5353,28,3],[5353,30,1],[5353,42,13],
[5353,43,2],[5354,48,1],[5354,64,1],[5355,9,2],[5355,11,2],
[5355,12,1],[5355,34,1],[5356,24,1],[5356,28,11],[5356,32,1],
[5356,33,11],[5356,35,1],[5356,38,1],[5356,52,5],[5356,61,1],
[5357,2,2],[5357,3,1],[5357,28,1],[5357,43,1],[5357,60,2],[5358,5,1],
[5358,18,1],[5358,20,5],[5358,21,8],[5358,23,2],[5358,26,7],
[5358,27,1],[5358,31,23],[5358,33,2],[5358,34,1],[5358,42,3],
[5358,43,7],[5358,51,1],[5358,70,1],[5359,5,1],[5359,24,1],
[5359,28,1],[5360,18,1],[5360,58,1],[5361,5,1],[5361,17,1],
[5361,22,1],[5361,25,1],[5361,26,1],[5361,31,2],[5361,52,1],
[5361,53,1],[5361,55,1],[5361,62,1],[5361,69,1],[5361,70,1],
[5362,2,1],[5362,3,1],[5362,6,1],[5362,21,1],[5362,23,2],[5362,24,3],
[5362,26,2],[5362,35,2],[5363,43,2],[5363,56,1],[5363,60,1],
[5363,62,1],[5364,22,2],[5364,23,1],[5364,24,1],[5364,31,1],
[5364,42,2],[5364,52,1],[5365,4,1],[5365,17,1],[5365,20,1],
[5365,27,1],[5365,32,1],[5365,55,1],[5365,64,1],[5366,2,7],
[5366,42,1],[5367,9,1],[5367,19,1],[5368,2,1],[5368,3,1],[5369,2,1],
[5369,33,1],[5369,43,1],[5370,13,1],[5370,20,2],[5370,24,1],
[5371,3,2],[5371,4,1],[5371,24,1],[5371,56,1],[5371,66,1],[5372,23,1],
[5372,26,1],[5372,27,1],[5372,33,6],[5373,20,1],[5373,23,3],
[5373,24,6],[5373,27,1],[5373,28,3],[5373,32,10],[5373,34,1],
[5373,35,2],[5373,42,5],[5373,47,1],[5374,2,1],[5374,6,1],[5374,23,1],
[5374,24,1],[5374,27,1],[5374,48,1],[5374,51,1],[5374,53,1],
[5374,56,1],[5374,66,1],[5375,4,1],[5375,22,1],[5375,27,1],
[5375,28,6],[5375,31,1],[5375,33,1],[5375,35,1],[5375,44,1],
[5375,47,1],[5375,51,6],[5375,66,2],[5376,10,2],[5376,15,1],

[5376,20,1],[5376,29,1],[5376,37,2],[5376,39,1],[5376,49,1],
[5376,53,1],[5376,57,1],[5376,58,1],[5376,59,1],[5376,65,3],
[5376,77,1],[5376,78,1],[5377,4,1],[5377,27,1],[5377,35,1],
[5377,49,1],[5378,3,1],[5378,5,1],[5378,21,1],[5378,23,4],[5378,24,3],
[5378,26,1],[5378,28,3],[5378,31,1],[5378,32,10],[5378,35,2],
[5378,42,3],[5378,44,1],[5378,47,1],[5378,76,1],[5379,5,15],
[5379,21,1],[5379,25,1],[5379,27,23],[5379,31,4],[5379,33,1],
[5379,35,3],[5379,52,1],[5379,76,1],[5380,2,2],[5381,2,8],[5381,3,1],
[5381,5,1],[5381,18,1],[5381,20,1],[5381,22,1],[5381,24,5],
[5381,25,8],[5381,26,1],[5381,30,2],[5381,31,1],[5381,33,1],
[5381,43,1],[5381,47,2],[5381,48,2],[5381,56,4],[5381,76,1],
[5382,15,3],[5382,28,1],[5382,34,1],[5382,55,2],[5383,22,1],
[5383,23,2],[5384,14,1],[5384,27,1],[5385,2,3],[5386,28,1],
[5386,56,1],[5387,5,2],[5387,21,1],[5387,24,1],[5387,26,1],
[5387,31,1],[5387,40,1],[5387,43,1],[5388,24,2],[5388,75,1],
[5389,0,29],[5389,1,115],[5389,3,1],[5389,4,1],[5389,5,1],[5389,7,11],
[5389,8,40],[5389,9,9],[5389,10,6],[5389,12,49],[5389,13,38],
[5389,15,238],[5389,16,2],[5389,17,505],[5389,18,2],[5389,19,5],
[5389,20,20],[5389,22,2],[5389,23,4],[5389,25,2],[5389,26,1],
[5389,27,3],[5389,28,12],[5389,29,26],[5389,30,19],[5389,31,2],
[5389,32,2],[5389,33,3],[5389,34,37],[5389,35,1],[5389,36,25],
[5389,37,3],[5389,38,1],[5389,39,56],[5389,43,1],[5389,45,3],
[5389,46,2],[5389,47,2],[5389,48,2],[5389,50,3],[5389,51,1],
[5389,53,545],[5389,54,1],[5389,55,531],[5389,56,2],[5389,58,1],
[5389,59,29],[5389,60,2],[5389,61,1],[5389,62,2],[5389,63,2],
[5389,65,1],[5389,66,1],[5389,67,1],[5389,73,4],[5389,74,1],
[5389,76,2],[5389,77,3],[5389,78,3],[5390,2,1],[5390,3,1],[5390,4,1],
[5390,8,1],[5390,24,1],[5390,25,1],[5390,28,18],[5390,30,1],
[5390,31,1],[5390,32,7],[5390,33,17],[5390,35,16],[5390,38,2],
[5390,52,23],[5390,65,1],[5390,70,1],[5390,76,1],[5391,2,9],
[5391,3,19],[5391,4,1],[5391,5,8],[5391,6,11],[5391,13,1],[5391,15,2],
[5391,16,1],[5391,21,1],[5391,22,2],[5391,23,17],[5391,24,10],
[5391,25,30],[5391,26,6],[5391,27,22],[5391,28,8],[5391,30,1],
[5391,31,4],[5391,32,56],[5391,34,1],[5391,35,5],[5391,38,3],
[5391,39,1],[5391,42,6],[5391,43,3],[5391,44,5],[5391,46,1],
[5391,47,14],[5391,48,1],[5391,50,2],[5391,52,1],[5391,56,32],
[5391,59,1],[5391,60,9],[5391,61,2],[5391,62,16],[5391,63,2],
[5391,66,4],[5391,67,1],[5391,68,2],[5391,70,4],[5391,72,1],
[5391,73,1],[5391,74,3],[5391,75,2],[5391,78,1],[5392,1,1],[5392,9,1],
[5392,10,2],[5392,13,1],[5392,17,1],[5392,19,1],[5392,39,1],
[5392,64,7],[5392,77,1],[5393,25,4],[5393,31,1],[5393,32,1],
[5394,22,1],[5394,24,3],[5394,27,1],[5394,42,1],[5394,43,1],
[5394,72,1],[5395,8,1],[5395,14,3],[5395,21,1],[5395,24,1],
[5395,26,2],[5395,27,1],[5395,31,4],[5395,32,1],[5395,37,1],
[5395,42,1],[5395,43,2],[5395,48,1],[5395,50,1],[5396,8,1],
[5396,27,1],[5397,2,1],[5397,8,1],[5397,16,2],[5397,34,1],[5397,55,1],
[5397,71,1],[5398,51,1],[5398,70,1],[5399,28,31],[5400,17,3],
[5400,53,13],[5401,25,1],[5401,43,1],[5402,7,1],[5402,15,1],
[5402,70,1],[5403,10,3],[5403,12,2],[5403,14,2],[5403,16,1],
[5403,17,1],[5403,19,1],[5403,21,1],[5403,26,1],[5403,28,1],

[5403,31,2],[5403,36,1],[5403,40,1],[5403,44,1],[5403,46,1],
[5403,49,1],[5403,54,1],[5403,56,1],[5403,58,2],[5403,59,1],
[5403,61,1],[5403,65,2],[5403,66,1],[5403,67,1],[5403,70,1],
[5403,73,1],[5403,74,2],[5403,76,1],[5404,56,1],[5404,75,1],
[5405,44,1],[5405,66,1],[5406,2,2],[5406,12,2],[5406,31,2],
[5406,64,1],[5407,3,1],[5407,22,1],[5408,37,1],[5408,42,2],
[5408,43,1],[5408,47,4],[5408,48,7],[5408,61,1],[5409,50,1],
[5409,66,3],[5410,0,12],[5410,1,14],[5410,2,3],[5410,3,6],[5410,7,9],
[5410,8,1],[5410,9,5],[5410,10,4],[5410,11,3],[5410,12,191],
[5410,13,13],[5410,14,1],[5410,16,1],[5410,17,3],[5410,18,7],
[5410,19,5],[5410,20,4],[5410,22,2],[5410,23,4],[5410,25,1],
[5410,27,20],[5410,28,1],[5410,29,47],[5410,30,7],[5410,31,25],
[5410,32,20],[5410,33,1],[5410,34,4],[5410,35,5],[5410,36,1],
[5410,37,2],[5410,38,2],[5410,39,7],[5410,41,3],[5410,45,1],
[5410,46,1],[5410,47,1],[5410,49,2],[5410,53,2],[5410,56,1],
[5410,58,1],[5410,60,1],[5410,62,19],[5410,63,1],[5410,65,5],
[5410,66,1],[5410,67,1],[5410,69,2],[5410,70,2],[5410,71,1],
[5410,72,2],[5410,73,2],[5410,74,3],[5410,75,2],[5410,77,1],
[5410,78,1],[5411,33,15],[5411,75,1],[5412,32,1],[5412,56,1],
[5413,27,1],[5413,35,1],[5414,27,1],[5414,28,1],[5414,43,1],
[5414,70,1],[5415,23,2],[5415,26,1],[5415,32,1],[5415,78,1],
[5416,28,1],[5416,74,3],[5417,4,1],[5417,8,1],[5417,32,1],[5417,70,1],
[5418,55,1],[5418,66,1],[5419,3,3],[5419,31,1],[5419,39,1],
[5419,46,1],[5419,76,1],[5420,23,1],[5420,32,1],[5421,27,1],
[5421,31,1],[5422,3,1],[5422,20,1],[5422,21,1],[5422,22,1],
[5422,23,1],[5422,24,1],[5422,26,2],[5422,27,2],[5422,31,1],
[5422,35,2],[5422,47,2],[5422,74,1],[5423,2,26],[5423,4,1],
[5423,28,1],[5424,51,4],[5424,79,2],[5425,24,3],[5425,27,1],
[5425,32,1],[5426,43,2],[5427,4,1],[5427,6,1],[5427,32,19],
[5427,42,1],[5427,47,1],[5427,63,1],[5427,67,1],[5427,68,1],
[5428,24,3],[5428,28,4],[5428,32,1],[5428,62,1],[5428,66,1],
[5429,33,3],[5429,42,2],[5430,25,1],[5430,35,1],[5431,44,2],
[5431,66,3],[5431,79,1],[5432,27,1],[5432,31,1],[5432,42,1],
[5432,56,1],[5432,70,1],[5433,2,2],[5433,3,4],[5433,4,2],[5433,5,1],
[5433,6,2],[5433,15,1],[5433,17,1],[5433,20,1],[5433,21,1],
[5433,22,17],[5433,23,3],[5433,24,7],[5433,25,1],[5433,26,2],
[5433,27,2],[5433,28,3],[5433,31,1],[5433,32,3],[5433,35,4],
[5433,40,4],[5433,43,1],[5433,44,8],[5433,47,2],[5433,48,1],
[5433,50,2],[5433,51,3],[5433,56,11],[5433,62,2],[5433,66,2],
[5433,68,6],[5433,69,1],[5433,72,1],[5433,74,1],[5433,76,1],
[5434,28,3],[5435,23,1],[5435,24,1],[5436,2,6],[5436,21,8],
[5436,23,3],[5436,24,1],[5436,25,17],[5436,26,4],[5436,28,2],
[5436,31,22],[5436,42,6],[5436,43,13],[5436,44,1],[5436,47,1],
[5436,48,1],[5436,50,1],[5436,68,2],[5436,75,1],[5437,23,1],
[5437,33,7],[5438,22,2],[5438,27,1],[5438,28,1],[5438,32,3],
[5439,28,1],[5439,62,1],[5440,3,1],[5440,5,1],[5440,6,13],[5440,15,1],
[5440,24,30],[5440,25,3],[5440,28,2],[5440,32,2],[5440,33,2],
[5440,38,2],[5440,42,3],[5440,43,1],[5440,44,5],[5440,47,1],
[5440,50,1],[5440,51,4],[5440,56,1],[5440,62,3],[5440,66,1],
[5440,68,5],[5440,74,5],[5441,4,1],[5441,28,1],[5442,28,3],

[5442,56,2],[5443,12,1],[5443,64,1],[5444,20,1],[5444,27,1],
[5444,41,1],[5445,11,1],[5445,23,1],[5445,24,2],[5445,30,1],
[5445,34,1],[5446,28,1],[5446,38,1],[5447,27,1],[5447,33,7],
[5447,75,1],[5448,22,4],[5448,23,3],[5448,24,3],[5448,25,1],
[5448,26,1],[5448,27,1],[5448,28,1],[5448,32,2],[5448,45,1],
[5448,56,1],[5449,1,20],[5450,3,1],[5450,27,1],[5450,32,2],
[5451,13,1],[5451,57,1],[5452,12,1],[5452,18,1],[5452,41,2],
[5452,65,1],[5452,71,1],[5453,2,3],[5454,0,1],[5454,1,2],[5454,4,1],
[5454,7,2],[5454,8,2],[5454,15,2],[5454,17,1],[5454,28,21],
[5454,36,1],[5454,39,1],[5454,50,1],[5454,53,1],[5454,55,2],
[5454,73,1],[5455,0,1],[5455,1,100],[5455,8,9],[5455,10,1],
[5455,12,170],[5455,13,25],[5455,16,1],[5455,17,6],[5455,20,1],
[5455,29,69],[5455,30,1],[5455,34,5],[5455,36,4],[5455,39,13],
[5455,53,5],[5455,55,4],[5455,59,1],[5455,71,1],[5455,73,4],
[5455,77,6],[5456,3,1],[5456,5,1],[5456,24,1],[5456,33,1],[5457,0,1],
[5457,1,1],[5457,2,43],[5457,3,1],[5457,6,1],[5457,9,1],[5457,13,3],
[5457,18,1],[5457,20,16],[5457,21,2],[5457,23,1],[5457,24,1],
[5457,26,3],[5457,28,1],[5457,30,1],[5457,31,82],[5457,39,1],
[5457,40,2],[5457,42,8],[5457,43,18],[5457,44,1],[5457,47,21],
[5457,48,2],[5457,49,1],[5457,50,1],[5457,70,2],[5457,72,1],
[5457,73,1],[5458,9,1],[5458,60,1],[5459,2,11],[5460,28,3],
[5460,34,1],[5461,22,2],[5462,2,1],[5462,3,3],[5462,4,1],[5462,5,1],
[5462,9,5],[5462,10,1],[5462,13,1],[5462,16,1],[5462,19,1],
[5462,20,22],[5462,21,1],[5462,22,2],[5462,23,24],[5462,24,22],
[5462,26,1],[5462,27,46],[5462,28,10],[5462,30,3],[5462,31,2],
[5462,32,1],[5462,33,1],[5462,34,8],[5462,35,12],[5462,36,1],
[5462,44,1],[5462,47,1],[5462,48,2],[5462,53,1],[5462,54,1],
[5462,55,1],[5462,59,1],[5462,62,4],[5462,63,1],[5462,65,1],
[5462,66,1],[5462,70,1],[5462,74,2],[5462,78,1],[5463,2,1],
[5463,31,1],[5463,47,1],[5464,1,23],[5464,7,2],[5464,10,1],
[5464,12,25],[5464,13,4],[5464,14,1],[5464,17,1],[5464,21,1],
[5464,29,3],[5464,37,1],[5464,39,1],[5464,45,1],[5464,53,1],
[5464,57,2],[5464,62,2],[5464,77,2],[5465,8,1],[5465,10,5],
[5465,11,1],[5465,12,2],[5465,13,1],[5465,14,1],[5465,15,1],
[5465,16,1],[5465,18,7],[5465,19,4],[5465,36,1],[5465,37,2],
[5465,41,4],[5465,45,3],[5465,46,2],[5465,54,1],[5465,55,1],
[5465,57,4],[5465,58,2],[5465,59,2],[5465,65,3],[5465,67,2],
[5465,71,1],[5465,73,1],[5465,77,4],[5466,6,1],[5466,47,1],
[5466,51,1],[5466,56,1],[5466,68,3],[5466,69,1],[5466,70,2],
[5466,72,1],[5467,16,1],[5467,23,1],[5467,27,2],[5467,28,3],
[5467,32,1],[5467,38,2],[5467,40,1],[5467,47,45],[5467,51,1],
[5467,70,1],[5467,74,1],[5468,3,1],[5468,40,1],[5468,63,1],[5469,4,2],
[5469,6,1],[5469,23,1],[5469,31,1],[5469,38,1],[5469,44,1],
[5469,50,1],[5469,56,2],[5469,69,1],[5470,9,2],[5470,10,1],
[5470,16,1],[5470,23,1],[5470,24,1],[5470,25,3],[5470,26,1],
[5470,28,1],[5470,30,77],[5470,33,26],[5470,35,14],[5470,38,1],
[5470,39,1],[5470,52,37],[5470,54,1],[5470,58,1],[5470,64,1],
[5470,65,1],[5471,2,2],[5472,25,15],[5472,47,1],[5472,76,1],
[5473,3,1],[5473,6,1],[5473,22,1],[5473,24,1],[5473,25,1],[5473,28,1],
[5473,33,1],[5473,35,1],[5473,43,1],[5473,53,1],[5474,56,2],

[5475,1,1],[5475,9,1],[5476,1,1],[5476,12,1],[5477,9,1],[5477,17,1],
[5477,41,1],[5477,55,1],[5478,2,1],[5478,42,1],[5479,2,1],[5479,21,1],
[5479,28,1],[5480,0,1],[5480,7,1],[5480,16,1],[5480,17,2],[5480,48,1],
[5480,53,1],[5481,0,25],[5481,1,1],[5481,7,18],[5481,9,1],[5481,12,2],
[5481,17,1],[5481,18,1],[5481,30,1],[5481,31,1],[5481,34,1],
[5481,36,1],[5481,46,2],[5481,59,1],[5481,65,2],[5481,71,1],
[5481,73,4],[5482,23,2],[5482,24,2],[5482,30,3],[5482,47,1],
[5483,1,1],[5483,13,1],[5483,20,5],[5483,22,1],[5483,24,1],
[5483,27,2],[5483,36,1],[5483,53,2],[5483,78,1],[5484,28,4],
[5484,72,1],[5485,1,1],[5485,3,1],[5485,11,1],[5485,13,2],[5485,15,4],
[5485,16,3],[5485,19,4],[5485,20,63],[5485,23,3],[5485,24,6],
[5485,25,1],[5485,26,2],[5485,27,2],[5485,28,9],[5485,29,1],
[5485,30,1],[5485,32,85],[5485,33,1],[5485,38,2],[5485,39,8],
[5485,42,1],[5485,44,1],[5485,49,2],[5485,52,1],[5485,55,3],
[5485,57,1],[5485,59,3],[5485,60,1],[5485,62,1],[5485,63,1],
[5485,66,2],[5485,67,1],[5485,70,2],[5485,72,1],[5485,74,2],
[5485,76,1],[5485,77,1],[5486,27,1],[5486,35,1],[5487,23,1],
[5487,26,1],[5488,4,1],[5488,5,1],[5488,10,1],[5488,15,3],[5488,17,5],
[5488,36,1],[5488,41,1],[5488,53,2],[5488,55,6],[5488,59,1],
[5488,66,1],[5488,77,1],[5489,21,5],[5490,28,1],[5490,62,1],
[5491,4,5],[5491,50,4],[5492,23,3],[5492,78,1],[5493,20,2],
[5494,31,1],[5494,48,1],[5495,17,1],[5495,36,1],[5495,53,1],
[5496,2,1],[5496,23,1],[5497,2,1],[5497,32,3],[5498,27,2],[5499,2,8],
[5499,31,1],[5499,42,5],[5499,43,1],[5499,62,1],[5500,5,2],
[5500,23,1],[5500,43,1],[5501,2,1],[5501,27,1],[5501,75,1],[5502,0,2],
[5502,1,13],[5502,2,7],[5502,12,1],[5502,13,6],[5502,17,1],
[5502,20,16],[5502,26,7],[5502,29,2],[5502,31,12],[5502,34,1],
[5502,36,1],[5502,37,1],[5502,39,16],[5502,48,1],[5502,53,1],
[5502,73,1],[5502,77,1],[5502,78,1],[5503,28,2],[5503,32,1],
[5504,61,2],[5505,2,1],[5505,5,1],[5505,28,1],[5506,2,14],[5506,3,1],
[5506,28,1],[5506,38,1],[5507,38,3],[5508,3,1],[5508,21,6],
[5508,32,1],[5509,22,2],[5509,24,1],[5509,25,1],[5509,27,6],
[5509,31,1],[5509,32,2],[5509,35,2],[5509,50,1],[5510,24,1],
[5510,42,1],[5510,63,14],[5510,74,15],[5510,75,13],[5510,76,24],
[5511,6,1],[5511,16,1],[5511,22,1],[5511,24,2],[5511,32,1],
[5511,35,2],[5511,42,2],[5512,33,1],[5512,68,1],[5513,62,2],
[5514,13,1],[5514,17,1],[5514,53,3],[5514,55,1],[5515,2,1],[5515,3,3],
[5515,5,6],[5515,6,2],[5515,17,1],[5515,22,5],[5515,24,3],[5515,25,1],
[5515,26,1],[5515,27,1],[5515,28,1],[5515,33,2],[5515,35,1],
[5515,42,3],[5515,43,2],[5515,44,1],[5515,50,1],[5515,51,12],
[5515,56,1],[5515,68,1],[5515,69,1],[5515,74,1],[5516,15,3],
[5516,39,1],[5516,44,1],[5516,50,1],[5516,54,1],[5516,57,1],
[5516,66,2],[5517,6,1],[5517,27,1],[5518,21,1],[5518,22,1],
[5518,23,2],[5518,24,1],[5518,27,2],[5518,28,5],[5518,63,1],
[5519,4,1],[5519,5,1],[5519,17,6],[5519,21,1],[5519,42,1],[5519,50,1],
[5519,53,7],[5519,55,8],[5519,66,1],[5520,2,3],[5520,4,1],[5520,23,3],
[5520,25,1],[5520,26,1],[5520,31,2],[5520,32,2],[5520,35,1],
[5520,37,2],[5520,39,1],[5520,42,1],[5520,60,1],[5520,70,1],
[5520,78,1],[5521,5,1],[5521,44,1],[5521,50,1],[5521,68,1],
[5522,15,1],[5522,53,1],[5523,12,2],[5523,33,2],[5523,35,1],

[5523,43,2],[5524,2,1],[5524,22,1],[5524,31,1],[5524,50,1],
[5525,28,2],[5525,33,2],[5525,35,1],[5526,26,1],[5526,27,1],
[5527,26,2],[5528,2,1],[5528,28,1],[5529,35,16],[5530,5,1],
[5530,28,23],[5530,40,7],[5530,68,1],[5530,69,1],[5530,70,7],
[5530,72,5],[5531,6,1],[5531,22,1],[5531,28,1],[5531,32,2],
[5532,28,2],[5533,24,1],[5533,28,1],[5533,43,1],[5534,15,17],
[5534,16,1],[5534,23,1],[5534,26,1],[5534,33,7],[5534,35,1],
[5534,42,1],[5534,48,1],[5534,49,1],[5534,55,10],[5534,58,1],
[5534,65,1],[5534,71,1],[5534,72,1],[5534,74,1],[5535,5,1],
[5535,27,1],[5536,29,2],[5537,2,1],[5537,5,4],[5537,9,1],[5537,22,1],
[5537,24,1],[5537,28,4],[5537,32,1],[5537,44,1],[5538,35,1],
[5538,53,1],[5539,37,1],[5539,72,1],[5540,28,2],[5541,15,1],
[5541,25,2],[5541,48,1],[5541,53,2],[5542,28,2],[5542,31,15],
[5543,22,2],[5543,62,1],[5544,29,2],[5545,3,1],[5545,56,1],
[5546,26,1],[5546,27,1],[5547,16,2],[5547,28,1],[5547,55,1],
[5548,28,1],[5548,56,1],[5549,1,2],[5549,2,1],[5549,24,1],[5549,25,2],
[5549,28,3],[5549,30,3],[5549,35,1],[5549,52,3],[5550,4,1],[5550,9,1],
[5550,22,2],[5550,25,1],[5550,28,3],[5550,30,4],[5550,35,1],
[5550,52,13],[5550,57,1],[5550,63,1],[5550,72,1],[5550,74,1],
[5551,10,1],[5551,20,1],[5552,15,1],[5552,74,1],[5553,1,11],
[5553,10,1],[5553,12,5],[5553,13,7],[5553,19,1],[5554,20,1],
[5554,34,1],[5554,73,1],[5555,4,1],[5555,5,1],[5555,38,1],[5555,44,1],
[5555,50,3],[5556,1,2],[5556,5,2],[5556,6,1],[5556,21,1],[5556,24,3],
[5556,25,1],[5556,28,4],[5556,30,4],[5556,33,97],[5556,35,3],
[5556,38,1],[5556,42,2],[5556,43,1],[5556,44,4],[5556,47,2],
[5556,54,1],[5556,58,1],[5556,62,1],[5556,63,1],[5556,66,1],
[5556,67,1],[5556,70,2],[5556,74,1],[5557,22,2],[5558,0,1],
[5558,42,1],[5559,31,2],[5560,23,4],[5560,24,1],[5560,26,1],
[5560,27,2],[5560,32,9],[5560,33,1],[5560,42,1],[5560,43,2],
[5560,60,1],[5561,15,1],[5561,44,1],[5561,50,1],[5562,1,2],[5563,1,1],
[5563,25,1],[5564,36,1],[5564,39,1],[5564,43,1],[5565,23,3],
[5565,27,1],[5565,34,2],[5565,35,1],[5565,42,1],[5565,43,1],
[5566,1,1],[5566,33,1],[5566,42,3],[5566,47,1],[5567,2,2],[5568,3,2],
[5568,24,1],[5568,26,3],[5568,31,4],[5568,33,3],[5568,46,1],
[5569,6,1],[5569,28,1],[5570,22,1],[5570,24,1],[5570,25,1],
[5570,35,1],[5570,47,1],[5571,1,1],[5571,58,1],[5572,7,1],[5572,9,1],
[5572,13,1],[5572,18,1],[5572,20,1],[5572,41,1],[5572,65,1],
[5572,71,1],[5573,28,1],[5573,42,1],[5574,2,3],[5574,3,1],[5574,24,1],
[5574,35,1],[5574,56,1],[5575,10,1],[5575,11,1],[5575,14,1],
[5575,19,1],[5575,59,1],[5575,76,3],[5575,78,1],[5576,8,1],
[5576,20,1],[5576,23,1],[5576,27,1],[5576,68,1],[5577,10,2],
[5578,3,1],[5578,24,1],[5578,32,1],[5579,3,2],[5580,3,1],[5580,20,1],
[5581,4,1],[5581,26,1],[5581,33,1],[5581,76,1],[5582,2,1],[5582,3,1],
[5582,5,2],[5582,18,1],[5582,28,7],[5582,32,1],[5582,33,166],
[5582,35,2],[5582,38,1],[5582,40,3],[5582,43,1],[5582,44,2],
[5582,50,2],[5582,56,1],[5582,63,1],[5582,68,1],[5582,70,5],
[5583,46,1],[5583,57,1],[5584,1,1],[5584,10,1],[5584,60,1],
[5584,77,1],[5584,78,1],[5585,3,6],[5585,24,3],[5585,28,5],
[5585,42,2],[5586,2,4],[5586,17,2],[5586,20,2],[5586,25,1],
[5586,30,1],[5586,31,1],[5587,28,1],[5587,70,1],[5588,1,1],

[5588,17,1],[5588,20,1],[5589,2,1],[5589,23,1],[5589,26,4],
[5589,27,2],[5589,30,1],[5589,34,7],[5589,42,1],[5589,43,4],
[5589,76,1],[5590,28,2],[5591,19,1],[5591,59,1],[5592,22,1],
[5592,27,1],[5593,23,1],[5593,24,1],[5593,27,1],[5594,0,2],[5594,1,1],
[5594,2,1],[5594,3,2],[5594,8,1],[5594,9,1],[5594,10,1],[5594,13,4],
[5594,15,1],[5594,20,20],[5594,21,1],[5594,22,1],[5594,24,4],
[5594,26,2],[5594,28,1],[5594,29,1],[5594,31,1],[5594,32,1],
[5594,33,2],[5594,35,1],[5594,36,1],[5594,37,1],[5594,41,3],
[5594,44,1],[5594,55,1],[5594,60,2],[5594,65,1],[5595,2,1],
[5595,28,1],[5596,3,2],[5597,8,1],[5597,27,4],[5598,0,1],[5598,13,3],
[5598,20,3],[5598,42,3],[5598,51,4],[5599,1,1],[5599,8,1],[5599,16,1],
[5599,29,1],[5599,34,1],[5599,37,1],[5599,59,11],[5600,6,1],
[5600,22,1],[5600,26,1],[5600,38,1],[5600,42,1],[5601,2,1],
[5601,21,2],[5601,26,1],[5602,1,1],[5602,15,4],[5602,25,1],
[5602,55,5],[5603,2,1],[5603,3,1],[5603,4,1],[5603,25,1],[5603,28,1],
[5603,32,37],[5603,35,1],[5603,47,1],[5603,56,1],[5603,66,1],
[5603,70,2],[5603,72,1],[5603,74,1],[5604,5,1],[5604,17,1],
[5604,38,1],[5604,55,1],[5604,68,1],[5605,5,1],[5605,63,10],
[5605,75,5],[5605,76,13],[5606,1,1],[5606,39,2],[5607,12,1],
[5607,13,1],[5607,28,1],[5607,30,1],[5607,41,4],[5607,45,1],
[5608,3,1],[5608,25,3],[5608,26,1],[5608,43,3],[5608,44,2],
[5609,45,1],[5609,70,2],[5610,2,1],[5610,27,1],[5610,28,2],
[5610,56,1],[5610,66,1],[5610,70,1],[5611,3,1],[5611,25,1],
[5611,26,1],[5611,28,3],[5611,33,1],[5611,35,1],[5611,42,1],
[5611,43,1],[5612,28,4],[5612,38,1],[5612,69,1],[5612,76,1],
[5613,21,1],[5613,52,1],[5614,3,1],[5614,23,1],[5614,24,2],
[5614,47,1],[5615,22,2],[5616,20,1],[5616,21,1],[5616,23,1],
[5616,24,1],[5616,27,2],[5616,32,1],[5616,62,1],[5617,28,1],
[5617,31,1],[5618,27,1],[5618,28,3],[5618,51,1],[5618,66,2],
[5619,47,1],[5619,70,1],[5620,10,1],[5620,34,4],[5621,3,1],
[5621,65,1],[5621,70,2],[5622,33,1],[5622,44,1],[5622,69,1],
[5623,31,2],[5623,44,1],[5623,62,1],[5624,24,2],[5625,21,3],
[5625,24,1],[5625,27,5],[5625,28,3],[5625,35,1],[5626,8,1],
[5626,22,1],[5626,23,5],[5626,24,1],[5626,26,6],[5626,35,2],
[5626,42,4],[5626,43,7],[5626,47,2],[5627,25,3],[5627,28,8],
[5627,32,1],[5628,2,1],[5628,28,1],[5629,24,1],[5629,27,1],[5630,2,1],
[5630,4,6],[5630,5,3],[5630,15,1],[5630,22,1],[5630,24,1],[5630,29,1],
[5630,35,1],[5630,50,6],[5630,55,1],[5631,2,5],[5632,9,1],[5632,15,1],
[5632,17,1],[5632,19,2],[5632,20,1],[5632,29,1],[5632,37,1],
[5632,55,2],[5632,59,1],[5633,2,1],[5633,15,1],[5633,34,1],
[5633,57,1],[5633,65,1],[5634,2,1],[5634,17,1],[5634,25,4],
[5634,26,1],[5634,53,1],[5634,62,2],[5634,75,1],[5635,29,6],
[5636,28,2],[5636,30,2],[5636,33,11],[5636,52,2],[5636,69,1],
[5637,43,3],[5638,2,2],[5638,28,1],[5639,3,2],[5639,24,2],[5639,32,1],
[5639,44,1],[5639,48,1],[5640,8,1],[5640,20,1],[5641,28,10],
[5641,40,1],[5642,15,1],[5642,17,3],[5642,53,1],[5643,24,1],
[5643,55,3],[5644,42,2],[5645,2,1],[5645,3,1],[5645,22,1],[5645,27,1],
[5646,4,1],[5646,6,1],[5646,22,1],[5646,24,1],[5646,28,1],[5646,35,1],
[5646,37,2],[5646,42,1],[5646,48,1],[5646,68,1],[5646,77,1],
[5647,20,1],[5647,41,1],[5647,59,2],[5648,2,3],[5648,21,2],

[5648,23,30],[5648,25,1],[5648,31,9],[5648,33,2],[5648,42,1],
[5648,50,1],[5648,52,1],[5648,56,11],[5648,72,2],[5649,0,1],
[5649,1,1],[5649,2,1],[5649,5,3],[5649,8,1],[5649,9,1],[5649,13,2],
[5649,15,2],[5649,16,1],[5649,17,1],[5649,19,1],[5649,20,1],
[5649,30,2],[5649,31,8],[5649,32,1],[5649,33,1],[5649,35,2],
[5649,39,2],[5649,42,2],[5649,43,2],[5649,46,1],[5649,47,1],
[5649,51,1],[5649,55,1],[5649,60,1],[5649,68,1],[5649,74,1],
[5650,1,1],[5650,39,1],[5651,27,1],[5651,28,1],[5651,39,1],[5652,2,1],
[5652,28,1],[5652,31,1],[5652,55,1],[5653,26,3],[5653,28,1],
[5653,42,4],[5653,43,1],[5653,76,1],[5654,0,2],[5654,9,1],[5655,24,5],
[5655,27,2],[5655,33,3],[5655,35,6],[5655,42,1],[5655,66,1],
[5656,3,1],[5656,27,1],[5657,23,2],[5657,26,1],[5657,28,2],
[5657,31,1],[5657,32,6],[5657,43,3],[5658,12,2],[5658,20,4],
[5658,21,1],[5658,22,1],[5658,34,6],[5659,24,1],[5659,26,1],
[5659,32,1],[5659,34,2],[5660,27,3],[5660,28,1],[5660,33,1],
[5661,44,2],[5662,28,2],[5663,23,2],[5663,32,1],[5663,35,1],
[5663,59,1],[5664,12,6],[5664,29,8],[5664,67,1],[5665,28,7],
[5666,27,1],[5666,57,1],[5667,3,1],[5667,5,1],[5667,32,1],[5668,34,3],
[5669,24,3],[5670,27,1],[5670,28,1],[5670,68,2],[5670,70,1],
[5671,3,1],[5671,5,1],[5671,18,1],[5671,22,2],[5671,24,1],[5671,25,1],
[5671,33,1],[5671,40,2],[5671,48,1],[5671,55,1],[5671,58,1],
[5671,63,22],[5671,66,1],[5671,68,1],[5671,69,1],[5671,74,22],
[5671,75,31],[5671,76,50],[5672,1,1],[5672,5,1],[5672,23,1],
[5672,24,2],[5672,27,1],[5672,28,1],[5672,31,1],[5672,35,1],
[5672,40,1],[5672,44,1],[5672,61,1],[5672,63,1],[5672,68,4],
[5672,70,3],[5672,72,4],[5672,74,1],[5672,75,1],[5673,24,1],
[5673,32,3],[5674,50,3],[5675,20,1],[5675,35,1],[5676,0,8],
[5676,1,156],[5676,2,27],[5676,5,3],[5676,7,10],[5676,8,1],[5676,9,4],
[5676,10,2],[5676,12,32],[5676,13,32],[5676,15,6],[5676,16,1],
[5676,17,4],[5676,18,3],[5676,19,2],[5676,20,4],[5676,21,106],
[5676,22,2],[5676,23,1],[5676,25,2],[5676,26,95],[5676,28,7],
[5676,29,25],[5676,30,10],[5676,31,39],[5676,32,17],[5676,33,23],
[5676,34,1],[5676,35,3],[5676,36,1],[5676,37,5],[5676,39,93],
[5676,41,4],[5676,42,3],[5676,43,19],[5676,53,5],[5676,55,2],
[5676,57,1],[5676,58,1],[5676,59,3],[5676,64,2],[5676,65,3],
[5676,67,2],[5676,71,2],[5676,73,1],[5676,77,1],[5676,78,1],
[5677,2,9],[5677,31,1],[5677,32,1],[5677,43,1],[5677,62,1],[5678,4,1],
[5678,15,2],[5678,17,1],[5679,7,1],[5679,13,1],[5679,21,1],
[5679,23,1],[5679,24,1],[5679,26,1],[5679,27,1],[5679,30,3],
[5679,31,1],[5679,32,1],[5679,39,1],[5679,49,1],[5679,57,1],
[5680,10,1],[5680,18,1],[5681,30,1],[5681,31,1],[5682,5,1],
[5682,22,2],[5682,48,1],[5683,21,1],[5683,42,1],[5684,8,1],[5684,9,1],
[5684,12,1],[5684,15,1],[5684,16,1],[5684,20,1],[5684,41,1],
[5684,59,1],[5684,65,1],[5684,77,1],[5685,15,4],[5685,17,1],
[5686,3,1],[5686,24,1],[5686,26,1],[5687,9,1],[5687,12,2],[5687,15,1],
[5687,20,1],[5687,37,1],[5688,22,1],[5688,25,46],[5688,40,1],
[5689,59,1],[5689,77,1],[5690,1,1],[5690,3,1],[5690,8,1],[5690,9,1],
[5690,17,1],[5690,20,1],[5690,29,1],[5691,1,4],[5691,3,2],[5691,4,4],
[5691,5,2],[5691,6,2],[5691,12,1],[5691,13,1],[5691,15,3],[5691,17,1],
[5691,19,1],[5691,20,2],[5691,21,1],[5691,23,1],[5691,24,3],

[5691,25,1],[5691,26,2],[5691,27,9],[5691,30,1],[5691,31,1],
[5691,36,1],[5691,38,2],[5691,40,1],[5691,42,1],[5691,43,1],
[5691,44,3],[5691,46,1],[5691,47,9],[5691,50,3],[5691,51,4],
[5691,53,1],[5691,55,1],[5691,56,18],[5691,57,1],[5691,61,1],
[5691,62,1],[5691,63,2],[5691,66,2],[5691,69,3],[5691,70,2],
[5691,72,2],[5691,74,138],[5691,75,2],[5692,15,1],[5692,17,4],
[5692,23,1],[5692,28,2],[5692,35,1],[5692,38,1],[5692,40,1],
[5692,56,1],[5692,58,1],[5692,66,1],[5692,72,1],[5692,74,1],
[5693,6,1],[5693,25,1],[5693,27,6],[5693,28,1],[5693,32,1],
[5693,42,2],[5693,43,1],[5693,47,1],[5693,56,4],[5694,2,2],[5695,4,1],
[5695,28,1],[5695,43,1],[5695,44,5],[5695,45,1],[5695,50,1],
[5695,53,1],[5695,55,6],[5695,60,1],[5695,66,4],[5696,24,5],
[5697,9,1],[5697,10,2],[5697,11,1],[5697,12,3],[5697,13,1],
[5697,20,2],[5697,41,1],[5697,59,1],[5698,2,3],[5698,31,1],
[5698,40,1],[5699,3,1],[5699,30,1],[5699,68,1],[5700,27,3],
[5700,38,1],[5700,66,1],[5700,68,1],[5701,21,1],[5701,25,10],
[5701,27,2],[5702,2,1],[5702,6,1],[5702,24,1],[5702,27,1],[5702,35,2],
[5702,37,1],[5702,38,3],[5702,42,6],[5702,47,2],[5702,48,10],
[5702,52,1],[5702,63,1],[5703,1,1],[5703,12,1],[5703,17,2],
[5703,37,1],[5704,17,1],[5704,46,1],[5705,46,1],[5705,55,1],
[5706,22,1],[5706,24,1],[5706,62,1],[5707,25,1],[5707,27,1],
[5707,33,1],[5707,35,1],[5708,9,1],[5708,15,1],[5708,28,1],
[5708,37,1],[5709,22,2],[5709,23,1],[5709,31,1],[5709,37,1],
[5709,40,1],[5709,42,1],[5709,43,1],[5709,47,1],[5709,60,3],
[5709,74,1],[5710,5,1],[5710,31,1],[5710,42,1],[5711,28,1],
[5711,48,1],[5712,12,1],[5712,25,1],[5712,39,1],[5713,13,1],
[5713,23,4],[5713,24,1],[5713,52,1],[5714,31,1],[5714,56,3],
[5714,62,20],[5715,25,2],[5716,15,1],[5716,19,1],[5716,57,1],
[5716,64,1],[5717,28,1],[5717,66,1],[5718,2,6],[5718,5,1],[5718,20,1],
[5719,2,5],[5719,28,1],[5720,3,1],[5720,23,2],[5720,27,3],[5721,3,1],
[5721,28,1],[5721,42,1],[5721,43,1],[5722,24,1],[5722,28,2],
[5722,40,1],[5722,44,1],[5722,63,1],[5722,76,2],[5723,2,9],
[5724,26,2],[5725,28,1],[5725,56,1],[5726,10,1],[5726,17,1],
[5726,35,1],[5726,55,1],[5726,59,1],[5726,71,1],[5726,74,1],
[5727,21,2],[5728,5,1],[5728,28,1],[5729,4,1],[5729,7,2],[5729,12,1],
[5729,15,17],[5729,17,13],[5729,19,2],[5729,23,1],[5729,25,1],
[5729,27,1],[5729,28,10],[5729,29,1],[5729,35,1],[5729,44,1],
[5729,53,9],[5729,55,12],[5729,66,2],[5729,74,1],[5729,78,1],
[5730,13,2],[5730,29,2],[5731,1,7],[5731,13,1],[5731,31,11],
[5731,35,1],[5731,39,1],[5732,2,7],[5732,6,1],[5732,23,3],[5732,25,8],
[5732,27,3],[5732,35,1],[5732,43,1],[5732,74,1],[5733,28,1],
[5733,63,1],[5733,74,1],[5734,29,1],[5734,70,1],[5735,7,1],
[5735,50,1],[5736,28,1],[5736,74,1],[5737,56,2],[5738,21,2],
[5738,23,1],[5739,24,2],[5740,3,2],[5740,5,1],[5740,24,4],[5740,25,1],
[5740,26,1],[5740,27,1],[5740,28,2],[5740,32,1],[5740,56,1],
[5741,2,1],[5741,3,3],[5741,23,2],[5741,24,1],[5741,28,1],[5741,35,1],
[5741,37,1],[5741,66,4],[5742,33,1],[5742,43,1],[5743,2,1],
[5743,25,2],[5743,31,1],[5744,2,1],[5744,5,2],[5744,24,2],[5744,28,7],
[5744,38,2],[5744,40,1],[5744,50,1],[5744,63,1],[5744,66,2],
[5744,76,2],[5745,35,4],[5746,2,1],[5746,32,1],[5746,43,5],[5747,3,2],

[5747,66,1],[5748,28,3],[5749,38,2],[5750,5,1],[5750,7,1],[5750,9,1],
[5750,13,1],[5750,19,1],[5750,20,3],[5750,22,2],[5750,23,3],
[5750,24,3],[5750,26,3],[5750,27,3],[5750,33,1],[5750,34,6],
[5750,35,2],[5750,42,4],[5750,51,4],[5750,53,1],[5750,55,1],
[5750,60,1],[5750,68,2],[5750,69,2],[5750,70,1],[5750,72,1],
[5750,74,2],[5751,15,1],[5751,55,1],[5752,1,1],[5752,2,1],[5752,3,26],
[5752,4,5],[5752,5,11],[5752,6,7],[5752,7,1],[5752,10,1],[5752,12,2],
[5752,13,1],[5752,15,67],[5752,16,1],[5752,17,5],[5752,18,1],
[5752,19,2],[5752,20,12],[5752,21,7],[5752,22,4],[5752,23,62],
[5752,24,305],[5752,25,7],[5752,26,5],[5752,27,10],[5752,28,52],
[5752,29,1],[5752,31,1],[5752,32,3],[5752,33,1],[5752,34,1],
[5752,35,7],[5752,36,2],[5752,38,2],[5752,39,7],[5752,40,1],
[5752,42,6],[5752,43,6],[5752,44,5],[5752,45,1],[5752,46,1],
[5752,47,12],[5752,50,5],[5752,51,2],[5752,52,1],[5752,53,1],
[5752,55,35],[5752,56,40],[5752,57,1],[5752,58,1],[5752,59,1],
[5752,62,3],[5752,65,1],[5752,66,2],[5752,68,1],[5752,69,1],
[5752,70,5],[5752,71,1],[5752,72,2],[5752,73,1],[5752,74,6],
[5752,75,1],[5752,76,2],[5753,28,2],[5754,3,1],[5754,15,1],
[5754,39,1],[5754,50,1],[5755,28,8],[5756,22,4],[5756,43,1],
[5757,27,1],[5757,70,1],[5758,5,1],[5758,23,1],[5758,27,1],
[5758,32,32],[5758,38,1],[5758,42,1],[5758,47,1],[5758,56,1],
[5759,28,1],[5759,33,1],[5759,50,1],[5759,70,1],[5760,23,1],
[5760,24,1],[5760,32,1],[5761,10,1],[5761,13,2],[5761,16,1],
[5761,19,1],[5761,20,3],[5761,23,1],[5761,24,2],[5761,26,4],
[5761,27,10],[5761,30,1],[5761,34,7],[5761,42,4],[5761,52,1],
[5761,58,1],[5761,59,1],[5762,20,2],[5763,3,1],[5763,5,1],[5763,23,2],
[5763,26,1],[5763,31,3],[5763,33,1],[5763,42,4],[5763,47,1],
[5763,51,1],[5763,56,23],[5763,66,2],[5763,68,1],[5764,27,1],
[5764,28,1],[5765,2,1],[5765,47,1],[5766,2,1],[5766,20,1],[5767,43,1],
[5767,56,1],[5767,68,1],[5768,2,3],[5768,8,8],[5768,12,1],[5768,25,1],
[5768,38,1],[5768,62,1],[5768,65,1],[5769,6,1],[5769,8,1],[5769,15,1],
[5769,23,3],[5769,24,2],[5769,27,6],[5769,32,2],[5769,33,6],
[5769,40,1],[5769,42,1],[5769,43,1],[5770,26,1],[5770,32,8],
[5770,62,1],[5771,8,1],[5771,16,1],[5771,29,1],[5771,30,10],
[5771,33,5],[5771,35,5],[5771,39,1],[5771,52,12],[5772,22,1],
[5772,28,2],[5772,33,1],[5772,35,1],[5772,55,1],[5773,27,1],
[5773,28,1],[5774,1,1],[5774,2,2],[5774,5,1],[5774,9,2],[5774,14,1],
[5774,20,1],[5774,39,1],[5774,65,1],[5775,44,2],[5776,1,1],
[5776,24,1],[5776,42,2],[5777,20,6],[5777,31,1],[5777,55,1],
[5777,65,1],[5777,72,1],[5778,27,1],[5778,28,2],[5779,26,1],
[5779,42,1],[5780,27,1],[5780,28,1],[5780,30,1],[5780,33,4],
[5780,35,2],[5780,52,1],[5781,2,3],[5781,13,1],[5781,20,7],
[5781,21,1],[5781,24,1],[5781,26,3],[5781,27,1],[5781,42,17],
[5782,3,1],[5782,24,1],[5782,47,1],[5783,1,4],[5784,3,2],[5784,5,1],
[5784,12,1],[5784,22,1],[5784,23,1],[5784,26,4],[5784,27,3],
[5784,28,1],[5784,31,2],[5784,32,4],[5784,34,2],[5784,35,1],
[5784,39,1],[5784,43,1],[5785,28,1],[5785,76,1],[5786,32,2],
[5787,15,1],[5787,20,1],[5787,22,1],[5787,50,1],[5787,69,1],
[5788,3,7],[5788,24,1],[5788,26,1],[5788,27,1],[5788,28,3],[5789,3,1],
[5789,24,1],[5789,25,3],[5789,26,1],[5789,27,3],[5789,28,1],

[5789,29,1],[5789,32,1],[5789,33,6],[5789,35,1],[5789,40,1],
[5789,42,1],[5789,43,1],[5789,62,3],[5789,64,1],[5790,23,1],
[5790,24,1],[5790,31,19],[5790,33,5],[5791,22,2],[5791,23,1],
[5791,33,1],[5791,56,1],[5792,9,1],[5792,14,1],[5792,34,1],
[5792,58,1],[5793,25,1],[5793,26,2],[5793,27,2],[5793,28,1],
[5793,43,1],[5793,48,2],[5794,27,1],[5794,50,1],[5794,74,3],
[5795,17,1],[5795,53,1],[5796,2,2],[5796,3,1],[5796,9,1],[5796,10,1],
[5796,13,1],[5796,23,2],[5796,24,2],[5796,27,4],[5796,30,2],
[5796,31,2],[5796,37,1],[5796,48,1],[5797,3,1],[5797,23,2],
[5797,25,1],[5797,66,1],[5798,26,1],[5798,28,5],[5798,47,1],
[5798,52,1],[5799,3,1],[5799,6,1],[5799,27,2],[5799,35,1],[5800,3,2],
[5800,24,2],[5800,27,5],[5800,31,1],[5801,4,1],[5801,5,1],[5802,3,1],
[5802,28,1],[5803,26,1],[5803,28,1],[5804,5,12],[5804,26,1],
[5804,28,8],[5804,30,1],[5804,31,2],[5804,33,7],[5804,48,1],
[5804,66,2],[5805,1,1],[5805,13,1],[5805,23,1],[5805,77,1],[5806,2,3],
[5807,1,3],[5807,2,4],[5807,3,2],[5807,18,1],[5807,20,2],[5807,23,5],
[5807,28,1],[5807,38,1],[5807,48,1],[5807,59,2],[5808,5,1],
[5808,28,4],[5809,2,1],[5809,21,1],[5810,42,1],[5810,51,2],[5811,5,1],
[5811,10,1],[5811,13,1],[5811,30,1],[5811,78,1],[5812,28,2],
[5812,74,5],[5813,20,1],[5813,28,1],[5813,40,1],[5813,43,1],
[5813,48,1],[5814,1,1],[5814,13,2],[5814,20,2],[5815,4,3],[5815,5,2],
[5815,22,1],[5815,23,4],[5815,44,2],[5815,46,1],[5815,47,1],
[5815,50,5],[5815,62,1],[5815,66,2],[5816,17,1],[5816,53,1],
[5817,1,1],[5817,21,1],[5817,23,2],[5817,24,1],[5817,27,1],
[5817,28,3],[5817,31,5],[5818,22,1],[5818,27,1],[5818,28,3],
[5818,61,1],[5818,70,2],[5818,72,1],[5819,21,1],[5819,27,1],
[5819,31,1],[5819,62,1],[5820,5,3],[5820,13,2],[5820,14,1],
[5820,15,1],[5820,19,1],[5820,21,2],[5820,25,1],[5820,26,1],
[5820,28,3],[5820,32,2],[5820,35,2],[5820,37,3],[5820,39,2],
[5820,46,1],[5820,57,1],[5820,63,2],[5820,74,1],[5820,76,1],
[5821,25,2],[5822,1,1],[5822,8,2],[5822,25,4],[5822,31,6],[5822,34,1],
[5822,35,2],[5822,42,1],[5823,28,2],[5823,32,1],[5824,15,1],
[5824,39,1],[5825,25,31],[5825,28,1],[5825,44,1],[5825,66,1],
[5826,19,1],[5826,20,3],[5827,3,1],[5827,42,1],[5827,46,1],
[5828,15,1],[5828,20,2],[5828,21,1],[5828,23,29],[5828,25,1],
[5828,26,8],[5828,31,1],[5828,33,9],[5828,40,1],[5828,54,1],
[5828,59,1],[5828,63,1],[5828,72,1],[5828,74,1],[5829,47,2],
[5830,3,1],[5830,23,1],[5830,26,7],[5830,33,2],[5830,42,1],
[5830,43,11],[5830,50,1],[5831,3,1],[5831,20,2],[5831,24,4],
[5831,26,1],[5831,28,1],[5831,32,3],[5832,5,1],[5832,31,1],[5833,9,1],
[5833,13,1],[5833,28,1],[5833,35,1],[5834,43,1],[5834,56,1],
[5835,28,1],[5835,55,1],[5836,11,3],[5836,34,1],[5836,45,1],
[5836,63,1],[5836,65,1],[5837,6,1],[5837,21,1],[5837,22,1],
[5837,24,1],[5837,26,2],[5837,42,1],[5837,56,1],[5838,3,1],[5838,5,1],
[5838,6,3],[5838,22,1],[5838,23,1],[5838,24,1],[5838,27,8],
[5838,31,1],[5838,35,2],[5838,43,1],[5838,66,1],[5838,70,1],
[5839,2,1],[5839,5,3],[5839,26,1],[5839,43,2],[5839,55,1],[5840,2,7],
[5841,28,2],[5841,33,5],[5841,70,1],[5842,2,1],[5842,26,1],
[5842,33,1],[5842,35,1],[5842,40,1],[5842,47,1],[5842,50,1],
[5842,52,1],[5842,69,2],[5842,76,1],[5843,17,2],[5844,0,1],[5844,1,1],

[5844,4,1],[5844,9,1],[5844,10,1],[5844,15,18],[5844,18,1],
[5844,20,2],[5844,26,1],[5844,28,7],[5844,34,1],[5844,38,1],
[5844,43,1],[5844,55,16],[5844,65,1],[5844,67,2],[5844,77,1],
[5845,4,1],[5845,55,1],[5846,32,3],[5847,10,1],[5847,19,1],
[5847,34,1],[5848,5,1],[5848,32,1],[5849,0,1],[5849,2,1],[5849,16,1],
[5849,19,1],[5849,20,8],[5849,30,1],[5849,42,1],[5849,59,1],
[5850,21,2],[5851,15,1],[5851,28,2],[5852,63,6],[5852,74,4],
[5852,75,6],[5852,76,7],[5853,22,1],[5853,25,1],[5853,27,1],
[5853,35,1],[5853,40,1],[5853,66,1],[5853,74,3],[5854,13,1],
[5854,29,2],[5855,1,3],[5855,2,2],[5855,3,2],[5855,4,1],[5855,5,2],
[5855,7,1],[5855,8,6],[5855,9,1],[5855,10,1],[5855,11,2],[5855,12,1],
[5855,13,1],[5855,16,1],[5855,17,1],[5855,19,1],[5855,20,2],
[5855,21,1],[5855,23,4],[5855,24,4],[5855,25,2],[5855,26,1],
[5855,27,4],[5855,28,6],[5855,29,1],[5855,30,3],[5855,31,3],
[5855,32,1],[5855,34,3],[5855,35,2],[5855,39,5],[5855,40,1],
[5855,42,5],[5855,43,4],[5855,44,1],[5855,45,3],[5855,53,1],
[5855,54,2],[5855,56,1],[5855,62,1],[5855,65,3],[5855,71,1],
[5855,73,11],[5855,76,1],[5856,9,1],[5856,28,2],[5856,35,1],
[5856,43,1],[5857,51,3],[5858,22,1],[5858,26,1],[5859,5,4],
[5859,24,1],[5859,28,1],[5859,38,2],[5860,4,2],[5860,25,1],
[5860,28,1],[5860,50,2],[5861,2,1],[5861,23,1],[5861,28,1],
[5862,11,1],[5862,28,1],[5862,41,1],[5862,55,1],[5863,21,1],
[5863,23,3],[5864,1,2],[5864,3,2],[5864,4,1],[5864,9,5],[5864,10,7],
[5864,11,3],[5864,12,4],[5864,13,2],[5864,14,3],[5864,15,3],
[5864,17,38],[5864,18,1],[5864,19,1],[5864,20,3],[5864,22,1],
[5864,24,1],[5864,27,1],[5864,28,3],[5864,29,3],[5864,37,1],
[5864,39,1],[5864,40,1],[5864,43,1],[5864,44,2],[5864,45,4],
[5864,50,2],[5864,53,30],[5864,54,1],[5864,55,1],[5864,56,1],
[5864,57,1],[5864,58,1],[5864,59,4],[5864,63,1],[5864,65,3],
[5864,67,2],[5864,70,1],[5864,71,1],[5864,74,3],[5864,77,2],
[5865,28,2],[5866,15,5],[5866,20,1],[5866,55,3],[5867,4,1],
[5867,22,1],[5867,24,1],[5867,25,1],[5867,28,2],[5867,32,43],
[5868,28,3],[5868,35,1],[5869,33,1],[5869,35,1],[5869,70,1],
[5870,20,2],[5871,24,1],[5871,31,1],[5872,7,1],[5872,9,3],[5872,10,1],
[5872,11,1],[5872,12,1],[5872,16,1],[5872,28,2],[5872,53,1],
[5872,67,1],[5872,73,1],[5872,77,1],[5873,6,2],[5874,0,1],[5874,1,5],
[5874,2,1],[5874,3,1],[5874,4,1],[5874,5,1],[5874,7,1],[5874,9,1],
[5874,11,1],[5874,13,5],[5874,20,3],[5874,21,9],[5874,23,8],
[5874,24,1],[5874,25,34],[5874,26,1],[5874,27,2],[5874,29,1],
[5874,31,40],[5874,32,1],[5874,33,42],[5874,34,1],[5874,35,1],
[5874,39,2],[5874,42,5],[5874,43,4],[5874,44,1],[5874,47,1],
[5874,48,5],[5874,50,2],[5874,66,1],[5874,70,1],[5874,76,1],
[5874,78,4],[5875,5,4],[5875,9,1],[5875,10,2],[5875,16,2],[5875,19,1],
[5875,26,1],[5875,27,1],[5875,31,1],[5875,32,1],[5875,39,1],
[5875,40,3],[5875,42,1],[5875,50,1],[5875,54,1],[5875,57,1],
[5875,61,1],[5875,63,67],[5875,65,1],[5875,68,2],[5875,70,2],
[5875,72,2],[5875,74,62],[5875,75,51],[5875,76,112],[5875,79,1],
[5876,62,8],[5877,5,2],[5877,23,1],[5877,24,3],[5877,25,1],
[5877,27,4],[5877,28,1],[5877,32,2],[5877,35,4],[5877,43,1],
[5878,1,7],[5878,2,1],[5879,22,1],[5879,27,1],[5879,28,2],[5879,32,1],

[5880,20,1],[5880,22,1],[5881,24,2],[5882,20,1],[5882,27,1],
[5882,28,1],[5883,49,1],[5883,64,1],[5883,77,1],[5884,20,1],
[5884,21,1],[5884,24,2],[5884,26,1],[5884,27,3],[5884,35,2],
[5885,6,3],[5885,22,2],[5885,43,1],[5886,1,4],[5886,3,11],[5886,5,2],
[5886,6,1],[5886,12,2],[5886,13,2],[5886,17,1],[5886,18,1],
[5886,21,1],[5886,22,1],[5886,24,1],[5886,25,2],[5886,27,2],
[5886,38,1],[5886,44,1],[5886,46,1],[5886,47,1],[5886,56,7],
[5886,60,3],[5886,66,2],[5886,70,1],[5886,73,1],[5886,74,3],
[5886,76,1],[5887,7,1],[5887,12,1],[5887,73,18],[5888,21,2],
[5888,22,3],[5889,19,1],[5889,43,1],[5890,2,1],[5890,3,2],[5890,4,14],
[5890,5,3],[5890,7,1],[5890,15,14],[5890,22,3],[5890,23,3],
[5890,26,2],[5890,27,1],[5890,28,5],[5890,31,2],[5890,32,1],
[5890,33,2],[5890,38,6],[5890,43,1],[5890,44,115],[5890,50,13],
[5890,55,10],[5890,56,1],[5890,61,1],[5890,62,2],[5890,66,56],
[5890,72,1],[5891,33,2],[5892,28,2],[5892,44,1],[5893,4,1],[5893,6,1],
[5893,50,2],[5894,5,1],[5894,23,1],[5894,24,2],[5894,27,7],
[5894,28,3],[5894,31,2],[5894,42,2],[5894,43,1],[5894,50,1],
[5894,60,1],[5895,24,2],[5895,28,6],[5895,65,1],[5896,4,2],[5896,5,2],
[5896,6,1],[5896,15,6],[5896,22,2],[5896,27,1],[5896,32,1],
[5896,35,1],[5896,40,1],[5896,47,2],[5896,48,1],[5896,53,1],
[5896,55,3],[5896,56,3],[5896,63,1],[5897,2,7],[5897,9,1],[5897,28,1],
[5897,41,1],[5897,55,1],[5897,69,1],[5898,20,1],[5898,23,1],
[5898,33,1],[5899,27,1],[5899,32,1],[5899,35,1],[5900,3,1],
[5900,23,3],[5900,24,1],[5900,28,1],[5900,43,1],[5901,2,1],[5901,4,1],
[5901,17,1],[5901,24,1],[5901,27,1],[5901,28,1],[5901,38,1],
[5901,44,4],[5901,50,1],[5901,66,8],[5901,74,2],[5901,75,1],
[5902,2,2],[5903,2,1],[5903,9,1],[5903,35,1],[5903,37,1],[5904,25,1],
[5904,28,3],[5904,31,1],[5905,15,3],[5905,55,3],[5906,1,1],
[5906,12,1],[5906,39,1],[5907,21,1],[5907,23,3],[5907,26,2],
[5907,27,9],[5907,31,2],[5907,32,1],[5907,35,1],[5907,56,1],
[5908,17,1],[5908,53,1],[5909,22,1],[5909,23,2],[5909,24,1],
[5909,27,1],[5909,28,2],[5909,33,1],[5909,35,1],[5909,39,1],
[5910,24,1],[5910,26,1],[5910,50,1],[5911,9,1],[5911,13,4],
[5911,20,5],[5911,58,1],[5911,59,2],[5911,78,1],[5912,28,21],
[5912,50,1],[5912,75,1],[5913,1,1],[5913,2,1],[5913,24,1],[5913,27,1],
[5913,60,2],[5914,28,1],[5914,35,1],[5914,47,4],[5915,23,1],
[5915,69,1],[5916,33,2],[5917,63,1],[5917,74,1],[5917,76,1],
[5918,15,3],[5919,12,1],[5919,30,1],[5919,41,1],[5920,70,2],
[5921,0,90],[5921,1,3],[5921,7,8],[5921,8,1],[5921,9,1],[5921,10,5],
[5921,11,4],[5921,12,3],[5921,13,1],[5921,14,1],[5921,16,3],
[5921,18,1],[5921,19,8],[5921,30,1],[5921,34,1],[5921,36,27],
[5921,37,4],[5921,41,2],[5921,45,2],[5921,46,18],[5921,53,1],
[5921,54,2],[5921,56,2],[5921,57,2],[5921,58,2],[5921,59,1],
[5921,60,2],[5921,65,5],[5921,69,1],[5921,73,3],[5921,77,6],
[5922,28,1],[5922,31,2],[5923,1,1],[5923,20,2],[5924,3,2],[5924,21,1],
[5924,23,1],[5924,24,5],[5924,25,1],[5924,34,2],[5924,56,1],
[5925,5,1],[5925,21,1],[5925,28,1],[5926,2,1],[5926,21,3],[5926,26,1],
[5926,31,2],[5926,33,1],[5927,63,2],[5927,74,1],[5927,75,1],
[5928,13,1],[5928,32,1],[5928,47,1],[5929,4,2],[5929,5,1],[5929,23,1],
[5929,31,1],[5929,50,1],[5929,66,3],[5929,70,1],[5930,0,1],[5930,2,2],

[5930,3,1],[5930,13,1],[5930,20,4],[5930,21,2],[5930,22,1],
[5930,23,2],[5930,24,1],[5930,27,11],[5930,28,1],[5930,35,4],
[5930,36,1],[5930,43,1],[5930,44,2],[5931,5,1],[5931,22,1],
[5931,23,1],[5931,25,1],[5931,32,1],[5931,33,1],[5931,35,2],
[5931,40,1],[5931,79,1],[5932,24,1],[5932,28,7],[5932,33,2],
[5932,42,2],[5933,13,1],[5933,25,1],[5934,30,1],[5934,31,8],
[5935,23,2],[5935,42,1],[5935,47,1],[5936,2,1],[5936,28,1],[5937,3,1],
[5937,28,1],[5937,38,1],[5938,2,1],[5938,3,1],[5938,24,1],[5938,25,1],
[5938,28,2],[5939,5,2],[5939,6,1],[5939,15,3],[5939,17,1],[5939,22,1],
[5939,24,2],[5939,25,1],[5939,27,3],[5939,28,1],[5939,32,1],
[5939,42,2],[5939,46,1],[5939,50,1],[5939,55,2],[5939,66,2],
[5939,67,1],[5939,68,1],[5939,70,1],[5939,72,3],[5939,74,1],
[5939,75,1],[5940,3,2],[5940,28,1],[5941,1,1],[5941,10,1],[5941,16,1],
[5941,20,1],[5941,22,1],[5941,24,1],[5941,28,1],[5941,30,12],
[5941,33,11],[5941,35,8],[5941,38,1],[5941,48,1],[5941,52,2],
[5941,53,1],[5941,59,1],[5941,62,1],[5941,65,1],[5941,66,1],
[5941,70,2],[5941,78,1],[5942,10,1],[5942,13,1],[5942,16,2],
[5942,29,1],[5942,34,1],[5942,45,1],[5942,55,2],[5942,57,1],
[5943,9,1],[5943,15,1],[5943,55,1],[5944,13,1],[5944,20,1],
[5944,22,1],[5944,32,1],[5944,33,1],[5944,35,1],[5945,5,1],
[5945,23,3],[5945,25,3],[5945,31,1],[5946,44,2],[5947,3,1],[5947,9,1],
[5947,33,1],[5947,39,1],[5948,3,1],[5948,28,1],[5949,2,6],[5950,32,1],
[5950,43,1],[5951,3,1],[5951,20,1],[5951,26,1],[5951,27,3],
[5951,28,1],[5951,39,1],[5952,2,13],[5952,3,1],[5952,21,1],
[5952,23,2],[5952,24,3],[5952,26,1],[5952,27,1],[5952,35,1],
[5952,43,2],[5953,1,2],[5953,4,8],[5953,7,1],[5953,8,2],[5953,9,1],
[5953,10,2],[5953,12,5],[5953,13,2],[5953,15,57],[5953,16,1],
[5953,17,79],[5953,18,1],[5953,19,1],[5953,20,2],[5953,21,6],
[5953,25,1],[5953,26,3],[5953,28,13],[5953,29,1],[5953,30,1],
[5953,31,2],[5953,36,1],[5953,38,1],[5953,39,1],[5953,41,3],
[5953,43,1],[5953,44,1],[5953,45,2],[5953,46,1],[5953,50,10],
[5953,53,80],[5953,55,25],[5953,56,1],[5953,57,1],[5953,62,1],
[5953,64,1],[5953,65,1],[5953,71,1],[5953,74,1],[5954,26,2],
[5954,69,3],[5954,76,1],[5955,1,3],[5955,9,1],[5955,10,1],[5955,13,1],
[5955,15,4],[5955,20,1],[5955,29,1],[5955,30,1],[5955,37,1],
[5955,53,3],[5955,55,2],[5955,77,1],[5956,27,1],[5956,42,1],
[5957,2,4],[5957,17,1],[5958,1,50],[5958,2,21],[5958,3,16],[5958,4,6],
[5958,5,16],[5958,6,2],[5958,8,1],[5958,9,7],[5958,10,1],[5958,11,1],
[5958,12,13],[5958,13,5],[5958,15,1],[5958,16,2],[5958,17,3],
[5958,19,1],[5958,20,13],[5958,21,9],[5958,22,5],[5958,23,18],
[5958,24,8],[5958,25,1],[5958,26,5],[5958,27,30],[5958,28,9],
[5958,29,2],[5958,30,10],[5958,31,7],[5958,32,2],[5958,33,15],
[5958,34,5],[5958,35,17],[5958,37,1],[5958,38,7],[5958,39,1],
[5958,40,2],[5958,42,9],[5958,43,5],[5958,44,8],[5958,47,8],
[5958,48,1],[5958,50,4],[5958,52,1],[5958,53,3],[5958,55,4],
[5958,56,3],[5958,57,2],[5958,59,6],[5958,60,2],[5958,61,1],
[5958,62,5],[5958,65,1],[5958,66,5],[5958,67,1],[5958,68,2],
[5958,70,6],[5958,71,1],[5958,72,2],[5958,73,2],[5958,74,14],
[5958,75,5],[5958,76,4],[5958,77,1],[5959,4,1],[5959,6,1],[5959,14,1],
[5959,20,1],[5959,24,3],[5959,42,1],[5959,44,2],[5959,56,1],

[5959,66,1],[5959,68,1],[5959,70,3],[5959,72,2],[5959,74,1],
[5960,2,2],[5961,2,1],[5961,5,2],[5961,12,9],[5961,13,5],[5961,23,1],
[5961,25,1],[5961,28,1],[5961,33,1],[5961,48,1],[5961,52,1],
[5961,68,1],[5961,73,1],[5962,13,1],[5962,56,1],[5963,21,1],
[5963,23,1],[5963,63,1],[5963,74,1],[5964,27,3],[5964,28,1],
[5964,32,12],[5964,43,1],[5964,47,1],[5965,5,10],[5965,31,1],
[5965,35,1],[5965,38,1],[5965,52,1],[5965,76,1],[5966,8,1],
[5966,10,4],[5966,11,1],[5966,16,1],[5966,36,1],[5966,46,1],
[5966,49,1],[5966,65,1],[5966,67,1],[5966,73,1],[5966,77,1],
[5967,12,1],[5967,28,5],[5967,35,1],[5968,6,2],[5968,22,3],
[5968,28,1],[5968,31,4],[5968,35,2],[5968,38,1],[5968,66,1],
[5968,77,2],[5969,28,15],[5970,37,1],[5970,48,1],[5971,3,1],
[5971,4,15],[5971,9,8],[5971,15,13],[5971,17,34],[5971,20,1],
[5971,24,2],[5971,25,1],[5971,27,2],[5971,28,17],[5971,32,1],
[5971,35,1],[5971,37,1],[5971,44,17],[5971,45,1],[5971,50,35],
[5971,53,18],[5971,66,18],[5972,28,2],[5973,0,1],[5973,7,1],
[5974,28,1],[5974,35,2],[5975,19,1],[5975,30,1],[5976,20,3],
[5976,25,1],[5977,22,2],[5977,66,1],[5978,19,1],[5978,27,1],
[5978,28,1],[5978,77,1],[5979,25,1],[5979,63,1],[5980,35,3],
[5981,2,1],[5981,3,3],[5981,5,3],[5981,21,1],[5981,22,3],[5981,23,3],
[5981,25,3],[5981,26,3],[5981,27,8],[5981,28,33],[5981,31,4],
[5981,32,1],[5981,33,5],[5981,35,3],[5981,38,3],[5981,40,9],
[5981,42,4],[5981,43,1],[5981,44,3],[5981,48,2],[5981,50,8],
[5981,56,5],[5981,63,6],[5981,66,7],[5981,68,4],[5981,69,1],
[5981,70,11],[5981,72,9],[5981,74,6],[5981,75,2],[5981,76,6],
[5982,4,1],[5982,17,1],[5982,23,1],[5982,24,1],[5982,25,3],
[5982,28,10],[5982,35,1],[5982,40,1],[5982,47,1],[5982,50,1],
[5982,56,2],[5982,63,1],[5982,72,3],[5982,76,1],[5983,4,2],[5983,5,1],
[5983,15,3],[5983,17,3],[5983,22,1],[5983,33,1],[5983,43,1],
[5983,44,1],[5983,50,1],[5983,53,12],[5983,55,2],[5983,66,1],
[5984,23,1],[5984,27,2],[5984,62,1],[5985,62,1],[5985,70,1],
[5986,15,2],[5986,17,1],[5987,23,1],[5987,26,6],[5987,27,3],
[5987,31,1],[5987,33,3],[5987,35,2],[5987,42,1],[5987,43,1],
[5987,44,1],[5987,56,1],[5987,69,2],[5988,10,1],[5988,57,1],
[5989,17,1],[5989,71,1],[5990,30,2],[5991,23,1],[5991,25,1],
[5991,31,1],[5992,61,2],[5993,23,3],[5993,26,1],[5994,17,1],
[5994,53,1],[5995,9,1],[5995,13,1],[5996,27,4],[5996,47,1],[5997,5,1],
[5997,33,1],[5998,2,5],[5998,3,1],[5998,4,8],[5998,21,1],[5998,22,2],
[5998,23,10],[5998,24,2],[5998,25,4],[5998,26,2],[5998,27,4],
[5998,28,34],[5998,31,9],[5998,32,5],[5998,33,4],[5998,35,4],
[5998,38,2],[5998,42,1],[5998,43,1],[5998,44,29],[5998,47,4],
[5998,50,8],[5998,51,2],[5998,52,2],[5998,56,3],[5998,62,4],
[5998,63,1],[5998,66,30],[5998,68,1],[5998,69,2],[5998,70,8],
[5998,72,2],[5998,74,1],[5998,76,3],[5999,15,2],[6000,23,1],
[6000,28,1],[6001,20,1],[6001,71,1],[6002,3,1],[6002,15,2],
[6002,56,13],[6002,58,1],[6002,59,1],[6002,60,3],[6002,67,1],
[6002,78,1],[6003,1,1],[6003,2,1],[6003,3,1],[6003,24,1],[6003,27,2],
[6003,30,1],[6003,34,1],[6003,35,2],[6003,43,1],[6003,47,3],
[6003,63,1],[6004,2,3],[6004,3,2],[6004,5,2],[6004,22,2],[6004,27,1],
[6004,28,1],[6004,32,2],[6004,43,1],[6004,44,1],[6004,47,2],

[6005,13,2],[6005,20,3],[6005,23,2],[6005,24,3],[6005,26,2],
[6005,27,2],[6005,28,1],[6005,32,1],[6006,2,1],[6006,3,1],[6007,24,1],
[6007,26,1],[6008,0,1],[6008,23,1],[6008,25,1],[6008,31,1],
[6008,32,1],[6008,35,1],[6008,62,1],[6009,0,2],[6009,10,1],
[6009,18,1],[6009,60,1],[6010,9,2],[6010,15,3],[6011,22,1],
[6011,28,1],[6012,3,2],[6012,6,5],[6012,23,1],[6012,24,31],
[6012,28,4],[6012,32,1],[6012,33,2],[6012,38,1],[6012,40,1],
[6012,56,24],[6012,70,1],[6012,74,1],[6013,15,1],[6013,55,1],
[6014,2,2],[6014,28,1],[6015,10,1],[6015,27,1],[6016,27,1],
[6016,56,1],[6017,2,21],[6017,26,1],[6017,31,1],[6017,32,1],
[6018,21,1],[6018,28,1],[6019,27,10],[6019,60,1],[6019,77,1],
[6020,15,2],[6020,53,2],[6020,55,2],[6021,3,1],[6021,35,1],
[6021,38,1],[6021,47,6],[6022,17,1],[6022,37,1],[6022,49,1],
[6022,57,1],[6023,22,2],[6024,17,3],[6025,5,1],[6025,6,1],[6025,23,3],
[6025,26,1],[6025,27,1],[6025,35,1],[6025,43,1],[6026,1,1],[6026,8,3],
[6026,9,1],[6026,12,2],[6026,13,1],[6026,15,1],[6026,20,3],
[6026,27,17],[6026,29,1],[6026,30,1],[6026,32,2],[6026,50,1],
[6026,67,1],[6026,70,1],[6027,2,3],[6027,5,1],[6027,10,1],[6027,21,1],
[6027,22,1],[6027,23,6],[6027,24,2],[6027,26,2],[6027,27,3],
[6027,28,2],[6027,31,1],[6027,39,1],[6027,42,1],[6027,43,1],
[6027,46,2],[6027,47,1],[6027,59,2],[6027,60,1],[6027,68,1],
[6028,3,3],[6028,18,1],[6028,20,1],[6028,35,1],[6029,40,1],
[6029,51,1],[6030,31,14],[6030,35,1],[6031,2,3],[6031,27,3],
[6031,62,1],[6032,44,1],[6032,70,1],[6032,74,1],[6033,28,2],
[6033,70,1],[6033,75,2],[6034,2,17],[6034,28,2],[6034,35,1],
[6034,72,1],[6035,21,1],[6035,22,3],[6035,23,2],[6035,24,1],
[6035,26,1],[6035,35,1],[6035,51,2],[6036,13,1],[6036,23,1],
[6036,24,1],[6036,34,1],[6037,9,1],[6037,54,1],[6037,64,1],
[6038,0,13],[6039,2,1],[6039,5,1],[6039,21,1],[6039,23,2],[6039,24,1],
[6039,31,1],[6039,43,1],[6040,44,1],[6040,66,1],[6041,25,2],
[6042,5,1],[6042,30,1],[6042,31,1],[6043,25,1],[6043,32,1],
[6043,39,1],[6044,17,1],[6044,53,1],[6045,27,1],[6045,42,1],
[6046,3,1],[6046,21,1],[6046,23,3],[6046,26,2],[6046,27,1],
[6046,31,2],[6046,42,1],[6046,62,1],[6047,3,1],[6047,28,10],
[6048,26,1],[6048,27,1],[6048,28,2],[6049,0,3],[6049,8,1],[6049,9,3],
[6049,10,1],[6049,12,2],[6049,19,1],[6049,20,55],[6049,21,1],
[6049,22,2],[6049,23,132],[6049,24,11],[6049,27,11],[6049,28,1],
[6049,30,1],[6049,31,6],[6049,32,2],[6049,34,1],[6049,35,12],
[6049,36,1],[6049,42,2],[6049,47,1],[6049,54,1],[6049,55,1],
[6049,65,2],[6049,67,2],[6049,78,1],[6050,2,4],[6050,3,2],[6050,5,4],
[6050,8,1],[6050,12,1],[6050,20,2],[6050,21,2],[6050,22,3],
[6050,23,9],[6050,24,4],[6050,26,3],[6050,27,5],[6050,28,1],
[6050,31,8],[6050,33,2],[6050,34,1],[6050,35,4],[6050,36,3],
[6050,38,1],[6050,41,1],[6050,42,3],[6050,43,1],[6050,44,1],
[6050,47,1],[6050,62,4],[6050,69,1],[6050,70,1],[6051,55,2],
[6052,22,1],[6052,35,1],[6052,42,1],[6053,39,3],[6054,2,9],[6054,3,1],
[6054,5,1],[6054,12,2],[6054,13,1],[6054,20,2],[6054,23,1],
[6054,24,1],[6054,25,3],[6054,26,1],[6054,32,1],[6054,34,1],
[6054,39,1],[6054,43,1],[6054,53,2],[6055,32,13],[6055,44,1],
[6056,32,12],[6056,62,1],[6057,2,10],[6057,20,1],[6057,25,1],

[6057,27,1],[6057,28,1],[6057,30,1],[6057,31,237],[6057,33,1],
[6057,42,2],[6057,43,7],[6057,62,1],[6057,66,1],[6058,1,2],[6058,2,1],
[6058,3,1],[6058,5,1],[6058,6,2],[6058,13,2],[6058,20,1],[6058,21,2],
[6058,23,6],[6058,24,3],[6058,26,2],[6058,28,3],[6058,30,2],
[6058,33,2],[6058,35,5],[6058,38,3],[6058,40,4],[6058,42,2],
[6058,43,2],[6058,44,10],[6058,50,1],[6058,51,12],[6058,55,1],
[6058,61,2],[6058,62,1],[6058,66,5],[6058,68,3],[6058,69,3],
[6058,70,9],[6058,72,9],[6058,76,4],[6058,79,1],[6059,2,1],
[6059,28,3],[6059,29,1],[6059,31,7],[6059,43,3],[6060,9,1],
[6060,29,1],[6061,28,24],[6061,68,24],[6062,31,4],[6063,21,1],
[6063,23,3],[6063,24,2],[6063,26,1],[6063,28,1],[6063,31,2],
[6063,32,3],[6063,43,1],[6063,47,2],[6063,48,1],[6063,52,1],
[6063,56,1],[6064,8,1],[6064,65,1],[6065,40,5],[6065,62,42],
[6065,73,1],[6066,13,1],[6066,77,1],[6067,4,4],[6067,5,3],[6067,22,2],
[6067,23,1],[6067,25,2],[6067,32,1],[6067,41,1],[6067,43,1],
[6067,44,5],[6067,48,1],[6067,50,8],[6067,66,6],[6068,22,2],
[6068,23,1],[6068,24,1],[6068,26,2],[6068,27,2],[6068,29,1],
[6069,72,3],[6070,3,1],[6070,8,1],[6070,22,5],[6070,23,8],[6070,24,1],
[6070,26,1],[6070,27,13],[6070,28,1],[6070,32,1],[6070,33,2],
[6070,35,4],[6070,38,1],[6071,4,1],[6071,5,2],[6071,21,4],[6071,23,1],
[6071,72,1],[6072,0,1],[6072,3,5],[6072,22,1],[6072,56,1],[6073,4,1],
[6073,23,1],[6073,24,1],[6073,27,1],[6073,30,1],[6073,50,1],
[6074,3,1],[6074,21,1],[6074,27,1],[6074,28,1],[6075,4,1],[6075,20,1],
[6075,50,1],[6076,50,3],[6077,28,2],[6077,44,1],[6078,4,1],
[6078,15,1],[6078,17,1],[6078,28,3],[6078,66,1],[6079,2,20],
[6079,3,1],[6079,31,1],[6079,50,1],[6079,56,1],[6080,34,1],
[6080,37,1],[6081,8,1],[6081,9,1],[6081,28,1],[6082,22,1],[6082,23,1],
[6082,24,1],[6083,3,4],[6083,21,1],[6083,22,1],[6083,23,3],
[6083,24,4],[6083,25,1],[6083,26,1],[6083,27,2],[6083,28,1],
[6083,42,1],[6083,45,1],[6083,64,1],[6084,15,1],[6084,17,7],
[6084,53,2],[6084,55,4],[6084,59,1],[6085,2,2],[6085,6,2],[6085,27,1],
[6085,40,1],[6085,42,1],[6085,47,1],[6085,72,1],[6086,9,2],
[6086,67,1],[6087,27,6],[6087,35,2],[6088,21,2],[6088,25,1],
[6088,26,1],[6088,42,1],[6089,32,1],[6089,43,1],[6090,19,1],
[6090,23,3],[6091,0,1],[6091,1,2],[6091,2,1],[6091,3,1],[6091,13,1],
[6091,20,1],[6091,28,1],[6091,31,1],[6091,33,1],[6091,49,1],
[6091,71,1],[6092,28,1],[6092,47,1],[6093,26,1],[6093,42,1],
[6093,66,1],[6094,2,4],[6094,28,2],[6095,15,1],[6095,55,1],
[6096,25,1],[6096,28,1],[6096,38,1],[6097,12,1],[6097,46,1],
[6097,57,1],[6098,56,2],[6099,28,1],[6099,31,1],[6100,2,2],
[6100,31,3],[6101,3,2],[6101,4,1],[6101,22,4],[6101,23,9],[6101,24,1],
[6101,25,2],[6101,27,2],[6101,33,2],[6101,47,2],[6101,48,1],
[6101,62,1],[6101,63,1],[6102,27,1],[6102,28,5],[6102,40,8],
[6102,43,1],[6102,61,2],[6102,62,1],[6102,70,1],[6103,3,1],
[6103,28,1],[6104,4,1],[6104,28,8],[6104,44,1],[6104,66,1],
[6105,17,1],[6105,53,1],[6106,26,1],[6106,56,1],[6106,60,1],
[6107,23,2],[6107,27,1],[6108,1,2],[6108,11,1],[6108,12,2],
[6108,17,2],[6109,8,1],[6109,20,1],[6109,22,1],[6109,31,2],[6110,1,1],
[6110,3,1],[6110,6,4],[6110,19,1],[6110,22,1],[6110,23,2],[6110,24,6],
[6110,27,2],[6110,31,1],[6110,32,1],[6110,33,1],[6110,36,1],

[6110,44,1],[6110,45,1],[6110,47,1],[6110,50,1],[6110,51,1],
[6110,55,1],[6110,56,22],[6110,74,1],[6111,28,1],[6111,44,1],
[6112,10,1],[6112,11,1],[6113,2,1],[6113,48,3],[6114,6,1],[6114,44,1],
[6115,0,1],[6115,3,1],[6115,4,1],[6115,5,1],[6115,15,1],[6115,19,1],
[6115,23,2],[6115,25,4],[6115,27,2],[6115,28,2],[6115,32,5],
[6115,33,2],[6115,35,2],[6115,43,1],[6115,45,1],[6115,75,1],
[6116,23,5],[6116,38,1],[6116,42,2],[6117,3,3],[6117,5,4],[6117,6,2],
[6117,15,1],[6117,22,2],[6117,23,1],[6117,24,1],[6117,27,5],
[6117,28,1],[6117,32,2],[6117,35,1],[6117,42,1],[6117,56,1],
[6117,66,1],[6117,74,1],[6118,74,1],[6118,76,1],[6119,0,1],
[6119,2,27],[6119,31,1],[6119,62,1],[6120,5,1],[6120,22,1],
[6120,24,2],[6120,27,1],[6120,28,1],[6120,33,1],[6120,50,1],
[6120,62,1],[6120,63,11],[6120,68,1],[6120,70,3],[6120,72,1],
[6120,74,6],[6120,75,4],[6120,76,9],[6121,22,1],[6121,25,1],
[6122,22,1],[6122,28,1],[6122,31,1],[6123,28,2],[6124,0,2],[6125,9,7],
[6125,15,1],[6125,28,10],[6125,46,1],[6125,68,1],[6125,70,1],
[6125,78,1],[6126,1,2],[6126,2,6],[6126,6,1],[6126,13,1],[6126,69,1],
[6126,70,5],[6126,72,2],[6127,25,1],[6127,75,1],[6128,1,10],
[6128,2,3],[6128,3,3],[6128,4,85],[6128,5,11],[6128,6,2],[6128,7,1],
[6128,8,1],[6128,9,9],[6128,10,2],[6128,11,6],[6128,12,5],[6128,13,8],
[6128,14,2],[6128,15,157],[6128,16,10],[6128,17,283],[6128,18,1],
[6128,19,8],[6128,20,6],[6128,21,6],[6128,22,3],[6128,23,7],
[6128,24,8],[6128,25,5],[6128,26,6],[6128,27,8],[6128,28,47],
[6128,29,3],[6128,31,8],[6128,32,2],[6128,33,5],[6128,34,2],
[6128,35,5],[6128,36,3],[6128,37,10],[6128,38,10],[6128,39,5],
[6128,40,3],[6128,41,4],[6128,42,4],[6128,43,4],[6128,44,41],
[6128,45,7],[6128,46,4],[6128,47,2],[6128,48,3],[6128,50,81],
[6128,51,2],[6128,52,2],[6128,53,280],[6128,54,1],[6128,55,95],
[6128,56,3],[6128,57,5],[6128,58,2],[6128,59,4],[6128,60,2],
[6128,61,1],[6128,62,2],[6128,63,2],[6128,65,5],[6128,66,41],
[6128,67,3],[6128,68,1],[6128,69,1],[6128,70,5],[6128,71,3],
[6128,72,1],[6128,73,1],[6128,74,7],[6128,75,2],[6128,76,3],
[6128,77,4],[6129,28,1],[6129,47,4],[6129,50,1],[6129,61,1],
[6129,66,1],[6130,3,7],[6130,6,1],[6130,24,2],[6130,27,1],[6130,32,2],
[6130,42,1],[6130,56,6],[6131,0,1],[6131,1,1],[6131,3,1],[6131,28,7],
[6132,3,2],[6132,24,3],[6132,26,1],[6132,27,1],[6132,28,3],
[6132,32,2],[6132,76,1],[6133,2,2],[6133,33,3],[6133,47,1],
[6133,57,1],[6134,22,1],[6134,23,1],[6134,26,1],[6134,31,1],
[6135,5,1],[6135,27,2],[6135,31,2],[6136,3,1],[6136,24,1],[6137,23,1],
[6137,28,1],[6137,76,1],[6138,2,46],[6138,9,1],[6138,13,2],
[6138,21,1],[6138,26,4],[6138,27,1],[6138,31,24],[6138,42,6],
[6138,43,2],[6138,47,1],[6138,57,1],[6138,72,1],[6138,76,1],
[6139,15,4],[6139,17,5],[6139,19,1],[6139,41,1],[6139,53,8],
[6139,55,8],[6139,64,1],[6140,42,2],[6141,28,3],[6141,76,1],
[6142,32,4],[6142,35,1],[6143,24,3],[6144,22,1],[6144,62,1],
[6144,70,1],[6145,0,1],[6145,10,2],[6145,29,1],[6145,30,52],
[6145,53,1],[6145,57,1],[6145,71,1],[6146,1,1],[6146,10,1],
[6146,11,1],[6146,13,1],[6146,15,2],[6146,16,1],[6146,19,4],
[6146,29,1],[6146,54,1],[6146,58,1],[6146,65,1],[6146,71,1],
[6147,26,1],[6147,72,1],[6148,2,1],[6148,28,1],[6149,1,16],[6149,2,1],

[6149,3,2],[6149,4,1],[6149,8,2],[6149,9,1],[6149,12,3],[6149,20,3],
[6149,21,3],[6149,22,3],[6149,23,13],[6149,24,4],[6149,25,2],
[6149,26,15],[6149,27,10],[6149,28,12],[6149,29,1],[6149,30,9],
[6149,31,16],[6149,32,6],[6149,33,1],[6149,34,1],[6149,35,4],
[6149,36,3],[6149,37,5],[6149,39,11],[6149,42,3],[6149,43,17],
[6149,47,1],[6149,59,2],[6149,77,1],[6150,22,2],[6150,26,1],
[6150,33,1],[6151,5,2],[6152,22,2],[6153,28,6],[6154,25,1],
[6154,28,1],[6154,33,5],[6154,43,1],[6154,74,1],[6155,27,1],
[6155,43,1],[6156,69,1],[6156,72,1],[6157,13,1],[6157,63,1],
[6158,28,1],[6158,39,1],[6159,2,1],[6159,13,1],[6160,2,1],[6160,14,1],
[6160,27,2],[6161,30,1],[6161,45,1],[6162,13,3],[6163,0,1],
[6163,1,64],[6163,7,1],[6163,8,1],[6163,12,2],[6163,13,4],[6163,14,1],
[6163,15,1],[6163,17,1],[6163,19,1],[6163,20,1],[6163,26,1],
[6163,29,7],[6163,34,3],[6163,37,1],[6163,39,5],[6163,47,1],
[6163,48,2],[6163,53,2],[6163,55,1],[6163,59,1],[6163,67,1],
[6163,73,1],[6163,77,1],[6164,25,2],[6165,21,1],[6165,32,1],
[6166,5,1],[6166,7,1],[6166,9,1],[6166,11,1],[6166,15,12],
[6166,28,14],[6166,39,1],[6166,44,2],[6166,50,1],[6166,55,8],
[6166,74,1],[6167,35,3],[6168,39,2],[6169,21,1],[6169,23,1],
[6169,32,2],[6169,42,7],[6169,48,2],[6169,60,1],[6169,72,1],
[6170,30,1],[6170,64,1],[6171,9,1],[6171,21,1],[6172,3,1],[6172,22,1],
[6172,27,1],[6172,39,1],[6173,33,1],[6173,44,2],[6174,26,1],
[6174,55,1],[6175,2,1],[6175,6,1],[6175,27,2],[6175,70,1],[6176,6,1],
[6176,25,1],[6176,28,5],[6176,31,1],[6176,33,1],[6176,75,1],
[6176,76,2],[6177,23,1],[6177,24,1],[6177,26,1],[6177,28,7],
[6177,40,3],[6177,63,2],[6177,68,1],[6177,70,2],[6177,74,1],
[6177,76,1],[6178,15,2],[6179,3,1],[6179,21,2],[6179,28,1],
[6179,37,6],[6179,47,2],[6179,56,6],[6179,66,1],[6179,72,2],
[6180,42,9],[6181,27,3],[6182,28,2],[6182,51,1],[6182,70,1],
[6183,2,1],[6183,23,1],[6184,24,1],[6184,27,1],[6184,30,1],[6185,2,2],
[6185,20,1],[6185,26,1],[6185,27,1],[6185,73,1],[6186,9,1],
[6186,13,2],[6186,20,1],[6186,34,1],[6186,37,2],[6186,42,1],
[6187,26,2],[6187,28,1],[6187,32,1],[6188,0,1],[6188,2,1],[6188,3,9],
[6188,6,1],[6188,13,1],[6188,16,2],[6188,19,1],[6188,20,1],
[6188,21,1],[6188,22,2],[6188,23,1],[6188,26,1],[6188,27,34],
[6188,32,2],[6188,38,2],[6188,41,1],[6188,42,2],[6188,47,2],
[6188,54,1],[6188,62,22],[6188,69,1],[6188,70,1],[6188,71,1],
[6188,74,1],[6189,2,4],[6189,18,1],[6189,20,1],[6189,27,3],
[6189,34,2],[6189,45,1],[6189,57,1],[6190,58,1],[6190,73,1],
[6190,77,3],[6191,17,1],[6191,25,1],[6191,32,10],[6191,35,3],
[6191,48,3],[6191,55,1],[6191,72,1],[6192,37,1],[6192,56,4],
[6192,61,1],[6193,2,1],[6193,3,1],[6193,12,1],[6193,21,1],[6193,24,4],
[6193,26,1],[6193,27,1],[6193,31,5],[6193,42,2],[6193,58,1],
[6193,62,3],[6194,21,1],[6194,24,1],[6194,27,1],[6194,42,1],
[6194,51,2],[6194,60,1],[6194,72,1],[6194,74,15],[6195,1,1],
[6195,7,1],[6195,29,3],[6195,39,2],[6196,1,4],[6196,9,2],[6196,10,1],
[6196,12,3],[6196,13,1],[6196,16,2],[6196,19,1],[6196,20,1],
[6196,24,4],[6196,28,2],[6196,30,4],[6196,33,1],[6196,34,1],
[6196,35,1],[6196,40,2],[6196,41,1],[6196,43,1],[6196,47,1],
[6196,54,1],[6196,57,1],[6196,60,1],[6196,62,1],[6196,64,1],

[6196,66,1],[6196,77,38],[6197,23,1],[6197,30,1],[6197,43,1],
[6198,3,1],[6198,28,2],[6198,30,1],[6199,3,1],[6199,25,1],[6199,61,1],
[6199,68,1],[6200,4,1],[6200,23,1],[6200,27,13],[6200,28,1],
[6200,35,1],[6200,43,2],[6200,44,2],[6200,51,1],[6200,66,2],
[6200,70,2],[6200,74,1],[6201,12,1],[6201,39,1],[6202,27,2],
[6202,28,2],[6203,27,1],[6203,28,1],[6204,5,1],[6204,66,1],[6205,9,1],
[6205,28,2],[6205,37,1],[6206,28,2],[6207,25,1],[6207,69,1],
[6208,22,1],[6208,44,1],[6208,65,1],[6208,68,1],[6208,74,1],
[6209,1,8],[6209,3,2],[6209,6,1],[6209,21,1],[6209,22,1],[6209,27,2],
[6209,35,2],[6209,39,1],[6209,43,1],[6209,47,1],[6209,51,1],
[6209,59,2],[6209,76,1],[6210,4,4],[6210,12,1],[6210,17,3],
[6210,21,2],[6210,27,2],[6210,29,1],[6210,32,2],[6210,35,1],
[6210,37,1],[6210,42,1],[6210,50,11],[6210,62,1],[6211,13,1],
[6211,25,6],[6211,28,1],[6212,28,2],[6212,31,1],[6213,21,1],
[6213,28,1],[6213,31,1],[6214,22,4],[6214,27,1],[6214,35,2],
[6215,14,1],[6215,48,2],[6216,63,1],[6216,74,3],[6216,75,1],
[6216,76,2],[6217,39,2],[6218,12,1],[6218,41,1],[6219,2,3],[6220,2,1],
[6220,3,2],[6220,24,2],[6221,8,1],[6221,30,1],[6222,18,2],
[6223,25,11],[6223,28,1],[6223,62,1],[6223,63,1],[6224,33,2],
[6225,28,1],[6225,30,1],[6225,55,2],[6226,0,3],[6226,1,1],[6226,11,2],
[6226,41,1],[6227,25,1],[6227,39,1],[6227,62,2],[6228,30,2],
[6228,31,2],[6229,15,1],[6229,28,1],[6229,30,1],[6229,38,1],
[6230,20,1],[6230,72,1],[6231,9,2],[6231,10,1],[6231,12,1],
[6231,16,1],[6231,39,1],[6231,41,1],[6231,49,1],[6231,54,1],
[6231,56,1],[6231,58,1],[6231,60,1],[6232,5,1],[6232,13,1],
[6232,74,1],[6233,56,6],[6234,33,2],[6234,52,1],[6234,55,1],
[6234,77,1],[6235,15,3],[6235,53,2],[6236,20,1],[6236,24,2],
[6236,29,1],[6236,73,1],[6237,47,1],[6237,50,1],[6238,15,1],
[6238,55,1],[6239,3,1],[6239,20,2],[6239,23,1],[6239,27,12],
[6239,32,2],[6239,33,1],[6239,43,5],[6239,63,2],[6240,25,1],
[6240,28,3],[6240,48,2],[6241,38,2],[6241,70,2],[6242,40,1],
[6242,48,1],[6243,27,1],[6243,32,1],[6243,39,1],[6244,1,19],
[6244,7,1],[6244,12,1],[6244,13,1],[6244,34,1],[6244,36,1],
[6245,18,1],[6245,77,1],[6246,1,1],[6246,2,1],[6246,13,1],[6246,34,2],
[6246,45,1],[6246,62,1],[6246,71,2],[6247,13,1],[6247,39,1],
[6248,12,1],[6248,16,1],[6248,33,1],[6248,34,1],[6248,66,1],
[6249,8,1],[6249,34,1],[6250,2,67],[6250,13,1],[6250,27,1],
[6250,28,1],[6250,31,4],[6250,64,1],[6251,21,1],[6251,31,2],
[6252,4,1],[6252,24,1],[6252,35,1],[6252,38,1],[6252,44,2],
[6252,66,2],[6253,9,1],[6253,10,2],[6253,14,1],[6253,16,1],
[6253,19,1],[6253,20,1],[6253,23,1],[6253,30,17],[6253,37,1],
[6253,39,1],[6253,55,1],[6253,57,2],[6253,62,4],[6253,67,1],
[6253,78,1],[6254,28,2],[6254,41,1],[6255,25,2],[6255,37,1],
[6256,4,2],[6256,27,1],[6256,35,1],[6256,57,1],[6257,53,2],
[6258,25,1],[6258,62,1],[6259,2,1],[6259,23,1],[6260,2,11],
[6260,31,1],[6260,62,1],[6261,5,1],[6261,26,1],[6261,31,1],
[6261,35,1],[6262,2,1],[6262,3,1],[6262,6,1],[6262,47,2],[6263,13,2],
[6263,77,1],[6264,26,1],[6264,40,1],[6265,6,2],[6265,26,1],
[6266,56,2],[6267,22,2],[6267,27,1],[6267,28,2],[6267,31,16],
[6267,32,1],[6268,26,1],[6268,28,2],[6269,42,1],[6269,62,16],

[6270,21,1],[6270,56,1],[6271,3,1],[6271,15,1],[6271,28,1],
[6271,41,1],[6271,52,1],[6272,3,1],[6272,26,1],[6272,43,1],
[6272,63,1],[6273,22,3],[6273,27,2],[6274,12,1],[6274,15,1],
[6274,44,1],[6274,65,1],[6275,22,3],[6275,23,6],[6276,15,1],
[6276,30,1],[6276,56,1],[6277,3,1],[6277,27,1],[6277,34,1],
[6277,35,1],[6278,1,5],[6278,2,1],[6278,4,7],[6278,7,1],[6278,9,1],
[6278,10,5],[6278,11,1],[6278,13,2],[6278,15,2],[6278,16,1],
[6278,20,3],[6278,22,2],[6278,23,2],[6278,24,1],[6278,25,1],
[6278,26,2],[6278,27,1],[6278,28,7],[6278,32,1],[6278,33,1],
[6278,34,1],[6278,35,2],[6278,37,2],[6278,38,6],[6278,44,77],
[6278,45,3],[6278,46,1],[6278,47,2],[6278,48,1],[6278,50,6],
[6278,51,1],[6278,53,3],[6278,55,1],[6278,56,2],[6278,57,1],
[6278,58,1],[6278,59,1],[6278,60,1],[6278,63,1],[6278,65,1],
[6278,66,56],[6278,67,1],[6278,70,2],[6278,71,1],[6278,74,3],
[6278,76,1],[6278,77,3],[6279,4,1],[6279,17,1],[6279,19,1],
[6279,21,2],[6279,26,1],[6279,27,1],[6279,28,2],[6279,31,1],
[6279,35,2],[6279,37,1],[6279,39,1],[6279,43,1],[6279,45,1],
[6279,47,1],[6279,50,1],[6279,54,2],[6279,56,1],[6279,62,2],
[6279,70,1],[6279,74,1],[6280,62,2],[6281,22,1],[6281,24,1],
[6282,22,1],[6282,24,2],[6282,27,1],[6282,30,1],[6283,23,1],
[6283,33,1],[6283,48,1],[6283,51,2],[6284,27,1],[6284,35,1],
[6285,5,2],[6285,21,1],[6285,52,1],[6285,60,1],[6285,78,1],[6286,2,1],
[6286,47,1],[6287,10,1],[6287,37,1],[6287,77,1],[6288,1,1],
[6288,18,1],[6288,46,1],[6289,6,1],[6289,56,1],[6290,32,3],[6291,1,1],
[6291,20,1],[6291,32,21],[6291,56,1],[6292,31,1],[6292,33,1],
[6293,2,1],[6293,3,2],[6293,5,4],[6293,13,1],[6293,20,4],[6293,22,2],
[6293,24,1],[6293,26,2],[6293,27,1],[6293,28,1],[6293,35,1],
[6293,38,1],[6293,39,3],[6293,40,1],[6293,62,1],[6293,79,1],
[6294,22,1],[6294,24,2],[6294,27,2],[6295,22,1],[6295,38,1],
[6295,40,1],[6295,74,1],[6296,5,1],[6296,21,1],[6297,33,2],
[6298,22,2],[6298,33,3],[6299,22,1],[6299,27,1],[6300,25,2],
[6301,8,3],[6301,20,1],[6301,23,2],[6301,24,1],[6301,26,1],
[6301,27,3],[6301,32,1],[6301,39,1],[6301,42,1],[6301,43,1],
[6302,13,1],[6302,27,1],[6302,30,1],[6302,35,1],[6302,61,3],
[6303,35,1],[6303,39,1],[6304,12,2],[6305,9,1],[6305,20,6],
[6305,22,1],[6305,23,1],[6305,28,2],[6306,21,1],[6306,22,2],
[6306,23,4],[6306,24,3],[6306,25,1],[6306,27,1],[6306,28,1],
[6306,32,3],[6306,33,1],[6306,35,6],[6307,7,1],[6307,13,1],
[6307,65,3],[6308,8,2],[6308,28,2],[6309,1,4],[6309,2,2],[6309,3,4],
[6309,5,1],[6309,6,1],[6309,10,1],[6309,13,1],[6309,16,1],[6309,19,1],
[6309,20,7],[6309,22,2],[6309,23,5],[6309,24,6],[6309,25,1],
[6309,27,5],[6309,28,4],[6309,30,1],[6309,32,4],[6309,35,1],
[6309,37,1],[6309,38,2],[6309,39,1],[6309,48,2],[6309,55,1],
[6309,57,1],[6310,28,1],[6310,35,1],[6311,63,1],[6311,74,2],
[6311,76,1],[6312,4,1],[6312,5,1],[6312,28,1],[6312,50,4],[6313,22,1],
[6313,27,1],[6314,27,1],[6314,37,1],[6315,0,1],[6315,1,1],[6315,7,2],
[6315,9,5],[6315,10,11],[6315,11,5],[6315,12,2],[6315,13,4],
[6315,15,2],[6315,16,7],[6315,18,4],[6315,19,6],[6315,20,2],
[6315,29,1],[6315,30,2],[6315,34,3],[6315,36,1],[6315,37,3],
[6315,39,2],[6315,41,4],[6315,45,10],[6315,46,7],[6315,49,4],

[6315,53,1],[6315,54,5],[6315,57,3],[6315,58,2],[6315,59,2],
[6315,60,1],[6315,64,3],[6315,65,6],[6315,67,4],[6315,71,1],
[6315,77,3],[6315,78,2],[6316,40,2],[6316,63,12],[6316,74,6],
[6316,75,8],[6316,76,26],[6317,27,4],[6318,5,1],[6318,22,1],
[6318,27,2],[6318,35,1],[6318,44,1],[6318,50,1],[6318,56,1],
[6319,6,1],[6319,23,1],[6319,26,1],[6320,47,1],[6320,53,2],
[6320,55,1],[6321,4,1],[6321,28,3],[6321,44,1],[6321,50,1],
[6322,28,1],[6322,35,2],[6323,27,1],[6323,32,2],[6324,38,1],
[6324,75,1],[6325,20,3],[6326,26,1],[6326,42,1],[6326,56,1],
[6326,62,1],[6326,66,2],[6327,5,1],[6327,28,1],[6328,40,1],
[6328,70,1],[6329,32,7],[6329,35,1],[6330,23,1],[6330,28,4],
[6330,31,1],[6330,33,1],[6330,35,2],[6330,38,1],[6330,44,2],
[6330,51,1],[6330,63,1],[6330,68,1],[6331,27,1],[6331,68,1],
[6332,12,2],[6332,15,1],[6332,20,2],[6332,34,2],[6332,36,1],
[6332,55,2],[6333,3,1],[6333,25,1],[6333,27,1],[6333,48,9],
[6333,76,1],[6334,20,1],[6334,22,1],[6334,24,2],[6334,30,2],
[6334,35,1],[6334,39,1],[6334,62,2],[6335,3,1],[6335,24,2],
[6335,43,1],[6336,9,1],[6336,30,1],[6336,37,1],[6336,54,1],
[6336,73,1],[6337,1,1],[6337,3,2],[6337,10,1],[6337,11,2],[6337,13,1],
[6337,14,3],[6337,15,2],[6337,16,1],[6337,19,3],[6337,20,2],
[6337,30,1],[6337,32,1],[6337,34,3],[6337,39,1],[6337,41,3],
[6337,45,1],[6337,55,2],[6337,56,1],[6337,57,3],[6337,58,3],
[6337,59,1],[6337,60,1],[6337,70,2],[6337,71,3],[6337,73,1],
[6337,77,2],[6338,28,3],[6339,2,6],[6339,31,1],[6339,73,1],
[6340,16,1],[6340,55,1],[6341,42,3],[6341,58,1],[6341,73,2],
[6342,1,1],[6342,12,2],[6342,28,1],[6342,30,2],[6342,34,1],[6343,8,1],
[6343,33,1],[6343,42,1],[6344,10,1],[6344,20,3],[6344,32,1],
[6344,34,1],[6344,36,2],[6344,42,4],[6344,57,1],[6344,60,1],
[6344,65,1],[6344,77,1],[6345,53,1],[6345,73,1],[6346,74,6],
[6347,6,1],[6347,43,1],[6347,66,1],[6348,2,1],[6348,31,1],[6348,35,1],
[6348,55,1],[6348,68,2],[6349,61,3],[6350,9,41],[6350,10,2],
[6350,11,1],[6350,12,1],[6350,19,1],[6350,20,1],[6350,36,1],
[6350,41,1],[6350,46,1],[6350,57,1],[6350,59,1],[6350,60,1],
[6350,65,1],[6350,73,1],[6350,78,2],[6351,17,1],[6351,65,1],
[6352,6,1],[6352,77,1],[6353,12,1],[6353,37,1],[6353,54,1],
[6353,57,1],[6354,28,25],[6354,76,1],[6355,1,1],[6355,23,1],
[6355,24,1],[6355,27,2],[6356,2,1],[6356,3,2],[6356,17,1],[6356,30,1],
[6357,30,8],[6358,28,1],[6358,32,3],[6359,38,2],[6360,24,2],
[6361,1,16],[6361,33,2],[6361,47,1],[6361,54,1],[6361,69,1],
[6361,77,3],[6362,21,1],[6362,26,1],[6362,28,1],[6363,28,3],
[6364,17,1],[6364,53,1],[6365,3,1],[6365,5,3],[6365,21,1],[6365,26,1],
[6365,27,1],[6365,31,1],[6365,42,10],[6365,43,2],[6365,47,1],
[6365,51,1],[6365,52,1],[6365,66,1],[6365,70,1],[6365,74,1],
[6366,44,1],[6366,47,1],[6366,70,1],[6366,72,1],[6367,3,2],
[6367,26,1],[6368,2,15],[6368,28,1],[6368,31,2],[6369,9,1],
[6369,53,4],[6370,27,2],[6371,60,1],[6371,75,2],[6372,23,1],
[6372,30,8],[6372,31,1],[6372,32,2],[6372,37,2],[6372,39,1],
[6372,42,2],[6373,13,1],[6373,31,1],[6373,32,7],[6373,39,1],
[6373,56,1],[6373,62,2],[6374,2,6],[6374,13,1],[6374,46,1],[6375,0,1],
[6375,28,2],[6376,3,1],[6376,23,1],[6376,24,1],[6377,2,4],[6378,2,2],

[6379,10,2],[6379,15,1],[6379,25,1],[6379,27,1],[6379,28,2],
[6379,34,1],[6379,35,1],[6379,37,1],[6379,38,1],[6379,43,1],
[6379,44,1],[6379,57,1],[6379,66,1],[6379,68,2],[6379,74,1],
[6379,75,1],[6380,2,3],[6380,56,7],[6381,23,1],[6381,32,1],
[6381,72,2],[6381,78,1],[6382,2,1],[6382,17,1],[6383,5,1],[6383,17,1],
[6383,23,1],[6383,32,1],[6383,53,2],[6384,51,3],[6385,62,4],
[6386,48,2],[6387,4,1],[6387,5,2],[6387,23,1],[6387,72,1],[6387,76,1],
[6388,5,1],[6388,22,1],[6388,28,2],[6389,29,1],[6389,77,1],
[6390,28,1],[6390,56,1],[6391,35,2],[6392,24,1],[6392,27,1],
[6392,43,1],[6392,53,1],[6393,31,1],[6393,32,1],[6394,35,2],
[6395,3,1],[6395,42,4],[6396,17,1],[6396,20,1],[6396,29,1],
[6396,35,1],[6397,28,2],[6397,33,1],[6397,47,1],[6397,70,2],
[6397,75,1],[6398,33,1],[6398,42,4],[6399,2,1],[6399,5,1],[6399,13,1],
[6399,24,1],[6399,30,1],[6399,32,1],[6399,33,1],[6399,35,1],
[6399,38,1],[6399,51,1],[6399,70,1],[6399,72,1],[6400,10,1],
[6400,11,1],[6400,12,1],[6400,20,2],[6400,24,2],[6400,41,1],
[6400,59,1],[6401,9,1],[6401,13,1],[6401,15,1],[6401,17,1],
[6401,22,1],[6401,50,1],[6401,53,4],[6401,59,1],[6401,65,2],
[6402,2,1],[6402,3,1],[6402,5,1],[6402,22,1],[6402,23,4],[6402,25,1],
[6402,27,1],[6402,28,17],[6402,31,1],[6402,32,1],[6402,44,2],
[6402,47,1],[6402,48,3],[6402,52,1],[6402,61,1],[6402,62,2],
[6402,63,1],[6402,66,2],[6402,68,3],[6402,70,1],[6402,72,2],
[6402,75,1],[6402,76,3],[6403,14,1],[6403,17,1],[6403,53,2],
[6404,23,1],[6404,24,1],[6404,26,1],[6405,31,2],[6406,21,1],
[6406,31,2],[6407,9,1],[6407,10,2],[6407,11,1],[6407,12,2],
[6407,13,2],[6407,16,1],[6407,19,1],[6407,20,2],[6407,28,1],
[6407,30,1],[6407,34,1],[6407,36,1],[6407,41,1],[6407,45,1],
[6407,46,1],[6407,53,2],[6407,54,2],[6407,65,5],[6407,67,1],
[6407,71,4],[6407,73,1],[6407,77,1],[6408,2,26],[6408,3,1],[6408,4,2],
[6408,12,1],[6408,20,1],[6408,22,1],[6408,23,3],[6408,24,3],
[6408,25,2],[6408,26,1],[6408,27,4],[6408,28,2],[6408,31,9],
[6408,40,1],[6408,42,2],[6408,43,6],[6408,50,1],[6408,53,1],
[6409,23,3],[6410,19,1],[6410,39,1],[6410,73,1],[6411,21,1],
[6411,31,2],[6412,22,1],[6412,26,1],[6412,63,1],[6413,66,3],
[6414,28,4],[6415,4,1],[6415,5,1],[6415,12,2],[6415,18,1],[6415,22,1],
[6415,23,2],[6415,25,1],[6415,31,1],[6415,32,2],[6415,35,2],
[6415,43,1],[6415,47,3],[6415,50,1],[6415,56,2],[6415,66,2],
[6415,68,1],[6415,69,2],[6415,70,2],[6415,72,2],[6415,74,30],
[6415,76,2],[6416,9,2],[6416,28,2],[6416,33,1],[6417,27,2],
[6417,35,1],[6418,7,1],[6418,43,2],[6419,2,1],[6419,32,1],[6419,50,1],
[6420,8,2],[6420,12,1],[6421,31,1],[6421,44,1],[6421,70,1],
[6422,20,2],[6422,22,1],[6422,28,1],[6422,31,1],[6422,32,1],
[6422,48,2],[6422,70,1],[6423,22,1],[6423,26,2],[6423,27,2],
[6423,28,1],[6423,38,1],[6423,70,1],[6424,1,14],[6424,9,5],
[6424,10,8],[6424,11,6],[6424,12,5],[6424,13,5],[6424,14,2],
[6424,15,9],[6424,16,5],[6424,17,8],[6424,19,4],[6424,20,5],
[6424,28,4],[6424,29,3],[6424,30,1],[6424,31,1],[6424,34,2],
[6424,36,1],[6424,39,3],[6424,41,4],[6424,45,3],[6424,49,2],
[6424,50,1],[6424,53,2],[6424,54,1],[6424,55,4],[6424,57,4],
[6424,58,2],[6424,59,4],[6424,60,1],[6424,62,1],[6424,64,3],

[6424,65,2],[6424,73,3],[6424,74,1],[6424,76,1],[6424,77,5],
[6425,7,1],[6425,10,1],[6425,11,1],[6425,13,1],[6425,16,2],
[6425,19,3],[6425,36,1],[6425,58,2],[6425,59,1],[6425,65,1],
[6426,1,2],[6426,29,1],[6427,27,1],[6427,61,1],[6427,68,1],
[6427,72,1],[6428,1,1],[6428,2,2],[6428,3,1],[6428,9,1],[6428,20,1],
[6428,23,3],[6428,24,3],[6428,26,1],[6428,27,2],[6428,43,1],
[6429,0,1],[6429,7,1],[6429,9,3],[6429,10,3],[6429,13,1],[6429,15,2],
[6429,18,1],[6429,34,1],[6429,53,2],[6429,54,1],[6429,55,2],
[6429,65,1],[6429,71,1],[6430,23,1],[6430,26,1],[6431,2,4],
[6431,3,11],[6431,8,1],[6431,10,1],[6431,22,4],[6431,23,5],
[6431,24,10],[6431,25,1],[6431,26,2],[6431,27,5],[6431,28,2],
[6431,31,1],[6431,32,1],[6431,33,3],[6431,43,4],[6431,47,5],
[6431,48,1],[6432,22,1],[6432,23,1],[6432,27,6],[6432,28,1],
[6432,33,2],[6433,20,2],[6434,17,1],[6434,30,1],[6435,27,1],
[6435,28,1],[6435,36,1],[6435,42,1],[6435,43,1],[6436,4,1],[6436,5,1],
[6436,56,1],[6437,23,1],[6437,28,1],[6437,42,1],[6438,2,32],
[6438,30,1],[6439,14,1],[6439,15,1],[6439,18,1],[6439,41,3],
[6439,55,1],[6440,27,2],[6441,8,3],[6441,27,2],[6441,32,1],[6442,2,1],
[6442,28,1],[6442,40,1],[6443,24,1],[6443,26,1],[6443,28,3],
[6443,31,6],[6443,35,1],[6443,42,3],[6443,43,1],[6444,28,1],
[6444,44,1],[6445,2,3],[6446,1,1],[6446,12,1],[6446,15,1],[6446,23,1],
[6446,24,2],[6446,26,1],[6446,29,1],[6446,30,1],[6446,33,2],
[6446,55,1],[6446,73,1],[6447,9,2],[6447,15,3],[6447,16,1],
[6447,53,1],[6447,55,2],[6448,17,1],[6448,22,1],[6449,3,1],[6449,4,1],
[6450,40,1],[6450,69,1],[6451,1,4],[6451,12,1],[6451,13,2],
[6451,15,1],[6451,17,1],[6451,19,1],[6451,20,2],[6451,24,2],
[6451,29,3],[6451,30,1],[6451,31,1],[6451,34,1],[6451,39,2],
[6451,53,1],[6451,65,2],[6452,32,3],[6452,66,1],[6453,4,1],
[6453,50,1],[6454,35,2],[6455,20,2],[6455,23,1],[6456,3,2],
[6456,22,1],[6456,26,1],[6456,28,2],[6456,30,1],[6456,32,1],
[6456,35,1],[6456,39,1],[6456,43,1],[6456,53,1],[6456,70,1],
[6457,56,3],[6458,21,2],[6458,24,1],[6458,26,1],[6458,32,3],
[6458,33,1],[6458,35,1],[6458,38,1],[6458,42,2],[6458,50,2],
[6458,51,1],[6458,56,7],[6459,24,1],[6459,27,1],[6459,28,1],
[6460,16,1],[6460,24,4],[6460,46,1],[6460,47,1],[6460,53,1],
[6460,56,7],[6460,61,1],[6460,70,1],[6461,2,6],[6461,28,1],
[6462,50,3],[6462,55,2],[6463,5,1],[6463,8,1],[6463,28,5],[6464,2,35],
[6464,24,1],[6464,42,2],[6464,74,1],[6465,3,1],[6465,14,1],
[6465,22,1],[6465,23,1],[6465,26,66],[6465,27,96],[6465,28,6],
[6465,33,1],[6465,34,1],[6465,44,1],[6465,47,24],[6465,49,1],
[6465,60,1],[6465,63,1],[6465,65,1],[6465,66,1],[6465,67,1],
[6465,71,1],[6465,75,1],[6466,12,2],[6466,22,1],[6466,32,1],
[6466,38,1],[6466,40,1],[6466,63,1],[6467,0,1],[6467,2,25],[6467,3,8],
[6467,4,2],[6467,5,4],[6467,6,1],[6467,7,1],[6467,8,1],[6467,9,1],
[6467,11,2],[6467,12,1],[6467,13,3],[6467,14,1],[6467,15,1],
[6467,16,2],[6467,17,1],[6467,18,1],[6467,19,1],[6467,20,5],
[6467,21,3],[6467,23,13],[6467,24,8],[6467,25,6],[6467,26,6],
[6467,27,12],[6467,28,16],[6467,30,6],[6467,31,9],[6467,32,6],
[6467,34,2],[6467,35,2],[6467,39,2],[6467,42,2],[6467,43,6],
[6467,45,1],[6467,46,2],[6467,47,8],[6467,50,1],[6467,53,6],

[6467,57,1],[6467,58,1],[6467,59,1],[6467,60,1],[6467,62,5],
[6467,64,1],[6467,65,3],[6467,71,1],[6467,73,1],[6467,77,1],
[6468,1,1],[6468,2,3],[6468,3,1],[6468,4,3],[6468,5,16],[6468,6,3],
[6468,10,1],[6468,12,2],[6468,13,1],[6468,15,75],[6468,16,3],
[6468,17,3],[6468,19,1],[6468,20,3],[6468,21,1],[6468,22,1],
[6468,23,4],[6468,25,1],[6468,27,2],[6468,28,2],[6468,31,1],
[6468,32,3],[6468,33,4],[6468,35,1],[6468,36,1],[6468,40,1],
[6468,42,2],[6468,43,2],[6468,44,5],[6468,45,2],[6468,47,2],
[6468,50,4],[6468,52,2],[6468,53,6],[6468,54,1],[6468,55,46],
[6468,57,1],[6468,58,1],[6468,59,1],[6468,63,1],[6468,65,1],
[6468,68,1],[6468,70,2],[6468,71,2],[6468,74,1],[6468,76,1],
[6469,23,1],[6469,39,1],[6469,43,1],[6469,64,1],[6470,2,14],
[6470,19,1],[6470,28,2],[6470,31,5],[6470,34,1],[6471,2,4],[6472,1,3],
[6473,20,1],[6473,39,1],[6473,42,2],[6474,5,1],[6474,23,1],
[6474,28,1],[6474,43,1],[6475,4,1],[6475,16,1],[6475,27,1],
[6475,31,1],[6475,36,1],[6475,43,1],[6476,3,4],[6476,32,4],
[6476,33,2],[6476,38,1],[6476,44,2],[6477,5,1],[6477,6,1],[6477,22,1],
[6477,23,1],[6477,24,1],[6477,26,2],[6477,34,1],[6477,42,1],
[6477,43,2],[6477,44,1],[6477,62,1],[6477,70,1],[6478,28,1],
[6478,32,1],[6479,2,1],[6479,3,1],[6479,6,2],[6479,22,3],[6479,23,3],
[6479,24,2],[6479,26,1],[6479,27,4],[6479,28,1],[6479,30,1],
[6479,33,2],[6479,34,2],[6479,35,1],[6479,38,1],[6479,43,1],
[6479,47,2],[6479,70,2],[6480,2,2],[6480,3,1],[6480,15,1],[6480,22,1],
[6480,27,1],[6480,30,6],[6480,38,1],[6480,42,1],[6480,47,1],
[6480,59,2],[6480,60,3],[6480,68,1],[6480,71,1],[6481,24,1],
[6481,40,1],[6482,23,1],[6482,28,1],[6483,23,2],[6483,26,2],
[6484,9,1],[6484,10,1],[6484,11,1],[6484,20,1],[6484,34,1],
[6484,60,1],[6484,78,1],[6485,74,1],[6485,77,1],[6486,23,3],
[6486,24,1],[6486,28,3],[6487,4,1],[6487,10,1],[6487,23,1],
[6487,31,1],[6488,1,2],[6488,9,1],[6488,77,1],[6489,25,1],[6489,32,3],
[6490,1,4],[6490,2,1],[6490,3,3],[6490,4,1],[6490,6,1],[6490,9,1],
[6490,10,1],[6490,12,2],[6490,15,2],[6490,16,2],[6490,17,1],
[6490,18,1],[6490,20,4],[6490,21,3],[6490,22,4],[6490,23,1],
[6490,24,2],[6490,25,1],[6490,26,1],[6490,27,4],[6490,28,1],
[6490,30,1],[6490,31,2],[6490,32,1],[6490,33,3],[6490,35,5],
[6490,38,1],[6490,42,1],[6490,44,1],[6490,47,5],[6490,50,2],
[6490,51,3],[6490,56,3],[6490,58,2],[6490,61,1],[6490,63,3],
[6490,64,1],[6490,65,1],[6490,66,1],[6490,68,3],[6490,70,2],
[6490,74,1],[6490,76,1],[6491,2,2],[6491,20,6],[6491,23,1],
[6491,28,2],[6491,32,2],[6491,43,4],[6491,64,5],[6491,66,1],
[6492,0,67],[6492,1,2171],[6492,2,2],[6492,5,1],[6492,7,17],
[6492,8,11],[6492,9,17],[6492,10,18],[6492,11,6],[6492,12,55],
[6492,13,26],[6492,14,5],[6492,15,24],[6492,16,12],[6492,17,23],
[6492,18,2],[6492,19,19],[6492,20,14],[6492,23,1],[6492,24,1],
[6492,25,10],[6492,28,1],[6492,29,71],[6492,30,62],[6492,31,3],
[6492,32,8],[6492,33,24],[6492,34,6],[6492,36,4],[6492,37,9],
[6492,39,139],[6492,41,11],[6492,43,1],[6492,45,7],[6492,46,1],
[6492,49,3],[6492,53,17],[6492,54,1],[6492,55,14],[6492,57,7],
[6492,58,7],[6492,59,9],[6492,60,6],[6492,62,1],[6492,64,1],
[6492,65,10],[6492,67,4],[6492,70,1],[6492,71,3],[6492,73,5],

[6492,77,5],[6492,78,2],[6493,26,1],[6493,38,1],[6494,3,2],
[6494,28,1],[6495,24,1],[6495,25,1],[6495,40,1],[6495,48,1],
[6495,50,1],[6495,61,1],[6495,62,2],[6495,63,17],[6495,66,1],
[6495,68,1],[6495,69,1],[6495,70,2],[6495,72,2],[6495,74,13],
[6495,75,10],[6495,76,17],[6496,1,1],[6496,3,5],[6496,50,1],
[6497,1,1],[6497,17,1],[6498,4,4],[6498,15,1],[6498,16,1],
[6498,17,23],[6498,20,1],[6498,53,37],[6498,55,1],[6498,76,1],
[6498,78,1],[6499,22,3],[6499,33,5],[6499,35,6],[6499,38,1],
[6499,43,1],[6499,72,1],[6500,70,2],[6501,63,1],[6501,75,1],
[6501,76,1],[6502,60,1],[6502,74,1],[6503,8,1],[6503,14,1],
[6504,20,1],[6504,25,2],[6504,28,2],[6505,28,2],[6505,33,1],
[6505,50,1],[6505,53,1],[6506,23,3],[6506,31,2],[6506,63,1],
[6507,3,1],[6507,21,1],[6507,23,6],[6507,24,1],[6507,26,1],
[6507,27,1],[6507,28,3],[6507,32,6],[6507,35,3],[6507,43,1],
[6508,12,1],[6508,13,2],[6508,26,2],[6508,70,1],[6509,1,1],
[6509,20,1],[6510,3,1],[6510,6,1],[6510,24,1],[6510,25,1],[6510,28,1],
[6511,23,2],[6511,27,2],[6511,31,2],[6511,32,1],[6511,42,7],
[6511,43,1],[6511,52,1],[6512,2,3],[6512,3,2],[6512,5,1],[6512,17,1],
[6512,20,1],[6512,23,1],[6512,25,1],[6512,26,1],[6512,29,1],
[6512,30,3],[6512,33,1],[6512,34,1],[6512,35,1],[6512,39,2],
[6512,48,2],[6513,33,1],[6513,62,1],[6514,2,3],[6514,9,1],[6514,25,1],
[6514,36,1],[6514,46,5],[6514,59,1],[6514,64,2],[6514,71,1],
[6515,10,1],[6515,15,2],[6515,17,3],[6515,53,6],[6515,55,3],
[6515,77,1],[6516,2,1],[6516,3,1],[6517,0,1],[6517,1,1],[6517,3,4],
[6517,7,1],[6517,13,2],[6517,14,1],[6517,18,1],[6517,20,2],
[6517,22,2],[6517,29,1],[6517,32,1],[6517,36,1],[6517,55,1],
[6517,60,1],[6517,63,1],[6517,64,1],[6517,65,1],[6517,66,1],
[6517,78,1],[6518,9,3],[6518,10,6],[6518,13,1],[6518,14,1],
[6518,15,23],[6518,16,3],[6518,17,8],[6518,19,3],[6518,20,2],
[6518,34,2],[6518,36,4],[6518,37,1],[6518,39,2],[6518,41,5],
[6518,44,2],[6518,45,5],[6518,46,1],[6518,49,1],[6518,53,12],
[6518,54,3],[6518,55,25],[6518,59,1],[6518,60,1],[6518,64,1],
[6518,65,2],[6518,67,3],[6518,71,2],[6518,73,1],[6518,78,1],
[6519,2,2],[6519,30,1],[6520,0,2],[6520,1,67],[6520,2,4],[6520,3,4],
[6520,4,1],[6520,5,3],[6520,6,1],[6520,7,1],[6520,8,8],[6520,9,12],
[6520,10,1],[6520,12,30],[6520,13,83],[6520,14,1],[6520,15,3],
[6520,17,3],[6520,20,6],[6520,21,9],[6520,22,39],[6520,23,11],
[6520,24,3],[6520,25,115],[6520,26,73],[6520,27,16],[6520,28,15],
[6520,29,6],[6520,30,1],[6520,31,44],[6520,32,19],[6520,33,301],
[6520,34,5],[6520,35,13],[6520,36,6],[6520,37,13],[6520,39,58],
[6520,40,1],[6520,42,1],[6520,43,62],[6520,44,1],[6520,45,1],
[6520,47,3],[6520,48,24],[6520,50,6],[6520,51,4],[6520,52,1],
[6520,53,10],[6520,55,2],[6520,60,1],[6520,62,2],[6520,63,1],
[6520,68,1],[6520,69,2],[6520,70,2],[6520,72,1],[6520,73,4],
[6520,76,2],[6520,78,1],[6520,79,3],[6521,2,6],[6521,3,2],[6521,4,1],
[6521,5,1],[6521,6,1],[6521,20,1],[6521,22,2],[6521,23,1],[6521,24,3],
[6521,32,1],[6521,33,1],[6521,42,1],[6521,43,1],[6521,47,2],
[6521,51,1],[6521,56,1],[6521,70,1],[6522,3,1],[6522,70,1],[6523,1,1],
[6523,19,1],[6523,20,2],[6523,34,1],[6523,37,2],[6523,46,1],
[6523,49,1],[6524,2,2],[6525,28,2],[6525,38,1],[6525,55,1],

[6526,1,15],[6526,2,1],[6526,4,1],[6526,7,1],[6526,13,4],[6526,21,1],
[6526,22,1],[6526,23,11],[6526,24,2],[6526,26,1],[6526,30,1],
[6526,34,1],[6526,35,1],[6526,36,1],[6526,42,1],[6526,55,1],
[6526,73,1],[6527,21,1],[6527,27,1],[6527,44,1],[6527,79,1],
[6528,32,3],[6529,20,1],[6529,46,1],[6530,27,6],[6530,35,2],
[6530,59,1],[6530,74,1],[6531,34,1],[6531,73,1],[6532,1,1],
[6532,34,1],[6533,53,1],[6533,55,1],[6534,22,1],[6534,24,2],
[6534,53,1],[6534,59,1],[6535,63,3],[6535,70,1],[6535,74,7],
[6535,75,3],[6535,76,2],[6536,27,1],[6536,33,1],[6537,27,1],
[6537,38,1],[6538,23,2],[6539,19,1],[6539,62,1],[6539,70,1],
[6539,75,2],[6539,76,5],[6540,2,3],[6540,20,15],[6540,26,4],
[6540,27,1],[6540,31,7],[6540,42,7],[6541,26,1],[6541,28,1],
[6542,61,2],[6543,27,17],[6544,3,1],[6544,28,1],[6544,44,1],
[6545,28,3],[6545,61,1],[6546,22,1],[6546,27,1],[6547,56,2],
[6548,28,2],[6549,17,2],[6549,28,3],[6549,53,1],[6550,17,1],
[6550,20,1],[6550,28,2],[6551,5,1],[6551,63,3],[6551,72,1],
[6551,74,3],[6551,75,2],[6551,76,4],[6552,2,1],[6552,27,1],
[6553,31,5],[6554,1,14],[6554,2,1],[6554,4,10],[6554,7,3],[6554,8,1],
[6554,9,7],[6554,10,5],[6554,11,1],[6554,12,3],[6554,13,2],
[6554,15,7],[6554,16,2],[6554,18,1],[6554,19,5],[6554,20,15],
[6554,21,3],[6554,22,3],[6554,23,1],[6554,28,1],[6554,29,2],
[6554,30,2],[6554,31,1],[6554,32,2],[6554,33,2],[6554,34,1],
[6554,36,3],[6554,37,2],[6554,38,1],[6554,39,1],[6554,41,4],
[6554,44,1],[6554,45,6],[6554,46,2],[6554,49,2],[6554,50,14],
[6554,52,1],[6554,53,5],[6554,54,2],[6554,55,6],[6554,57,1],
[6554,58,3],[6554,60,4],[6554,64,1],[6554,65,2],[6554,67,3],
[6554,71,2],[6554,73,2],[6554,75,1],[6554,76,1],[6555,43,2],
[6556,2,44],[6556,3,10],[6556,4,1],[6556,5,23],[6556,6,4],[6556,21,4],
[6556,22,11],[6556,23,38],[6556,24,28],[6556,25,5],[6556,26,16],
[6556,27,14],[6556,28,15],[6556,30,17],[6556,31,7],[6556,32,14],
[6556,33,7],[6556,34,1],[6556,35,4],[6556,39,1],[6556,40,1],
[6556,42,4],[6556,43,13],[6556,44,2],[6556,47,10],[6556,50,1],
[6556,55,1],[6556,56,6],[6556,60,1],[6556,62,1],[6556,63,2],
[6556,66,2],[6556,68,3],[6556,70,1],[6556,74,2],[6556,75,1],
[6556,76,1],[6557,1,13],[6557,2,2],[6557,7,1],[6557,8,1],[6557,9,1],
[6557,10,1],[6557,12,7],[6557,13,2],[6557,17,4],[6557,20,1],
[6557,23,4],[6557,24,1],[6557,25,2],[6557,28,2],[6557,29,10],
[6557,30,18],[6557,34,2],[6557,39,1],[6557,54,1],[6557,55,4],
[6557,62,1],[6557,73,1],[6558,21,1],[6558,25,7],[6558,26,2],
[6558,33,2],[6559,2,2],[6559,26,2],[6559,33,1],[6559,56,1],[6560,0,5],
[6560,62,1],[6561,12,1],[6561,19,1],[6561,46,1],[6561,59,1],
[6561,77,1],[6562,9,1],[6562,13,1],[6562,28,1],[6562,57,1],
[6563,42,1],[6563,43,1],[6564,28,2],[6564,40,1],[6564,72,1],
[6565,7,1],[6565,41,1],[6566,3,1],[6566,25,1],[6567,3,1],[6567,28,1],
[6567,74,1],[6568,22,1],[6568,24,1],[6568,26,1],[6568,27,2],
[6568,33,1],[6568,50,1],[6569,23,1],[6569,28,6],[6570,41,1],
[6570,43,1],[6570,59,1],[6571,23,1],[6571,26,1],[6571,61,1],
[6572,22,1],[6572,33,1],[6572,35,1],[6573,28,9],[6574,30,2],
[6575,2,1],[6575,13,1],[6575,22,2],[6575,23,1],[6575,27,1],
[6575,33,1],[6575,35,1],[6575,39,1],[6575,42,2],[6575,43,1],

[6575,47,2],[6575,70,1],[6576,1,2],[6576,2,2],[6576,3,5],[6576,9,1],
[6576,18,1],[6576,23,2],[6576,25,2],[6576,28,1],[6576,31,1],
[6576,33,1],[6576,35,3],[6576,39,1],[6576,47,1],[6576,52,1],
[6576,55,2],[6576,61,1],[6576,72,2],[6576,76,1],[6576,78,1],
[6577,2,1],[6577,5,1],[6577,20,1],[6577,21,12],[6577,22,2],
[6577,23,17],[6577,24,2],[6577,26,5],[6577,27,7],[6577,29,1],
[6577,32,2],[6577,33,15],[6577,35,12],[6577,39,4],[6577,42,2],
[6577,43,1],[6577,57,1],[6578,2,4],[6578,23,1],[6578,31,2],
[6578,47,1],[6579,2,2],[6579,69,1],[6580,4,1],[6580,44,2],[6580,50,1],
[6580,66,7],[6581,28,2],[6582,15,2],[6583,31,4],[6584,6,5],
[6584,44,1],[6584,47,1],[6584,68,1],[6584,74,1],[6585,1,2],[6585,3,2],
[6585,11,1],[6585,13,1],[6585,20,4],[6585,23,1],[6585,24,2],
[6585,27,1],[6585,28,1],[6585,33,1],[6586,22,1],[6586,26,1],
[6586,43,1],[6586,66,1],[6587,4,1],[6587,38,4],[6587,44,3],
[6587,50,1],[6587,66,1],[6588,15,1],[6588,45,1],[6589,4,2],[6589,5,3],
[6589,22,4],[6589,23,2],[6589,26,5],[6589,28,2],[6589,31,1],
[6589,32,2],[6589,33,1],[6589,42,2],[6589,43,2],[6589,44,2],
[6589,51,12],[6589,68,3],[6589,79,1],[6590,2,6],[6590,24,1],
[6591,28,2],[6592,28,2],[6592,31,1],[6593,5,1],[6593,20,1],
[6593,24,2],[6593,26,1],[6593,32,4],[6593,34,2],[6594,47,1],
[6594,70,1],[6595,5,1],[6595,21,1],[6595,24,1],[6595,25,1],
[6595,26,1],[6596,10,1],[6596,28,1],[6597,25,1],[6597,28,1],
[6597,32,1],[6597,35,2],[6598,5,2],[6598,23,1],[6599,2,1],[6599,22,2],
[6599,27,1],[6599,30,1],[6599,31,2],[6599,47,1],[6599,60,1],
[6600,2,4],[6601,0,5],[6601,1,3],[6601,2,1],[6601,7,1],[6601,8,2],
[6601,9,2],[6601,10,2],[6601,11,3],[6601,12,8],[6601,13,4],
[6601,18,2],[6601,19,2],[6601,20,2],[6601,26,1],[6601,29,13],
[6601,30,2],[6601,31,28],[6601,32,12],[6601,35,1],[6601,36,1],
[6601,40,1],[6601,49,1],[6601,51,1],[6601,53,1],[6601,54,1],
[6601,62,4],[6601,73,3],[6601,77,5],[6602,4,1],[6602,27,1],
[6602,35,2],[6602,68,1],[6602,76,1],[6603,22,3],[6604,20,2],
[6604,21,1],[6604,22,2],[6604,23,1],[6604,24,2],[6604,25,1],
[6604,26,6],[6604,31,1],[6604,32,2],[6604,33,2],[6604,35,3],
[6604,41,1],[6604,42,1],[6604,43,3],[6604,45,1],[6604,55,1],
[6605,17,2],[6606,22,1],[6606,32,1],[6606,40,1],[6606,56,2],
[6607,37,2],[6607,46,1],[6608,22,1],[6608,26,1],[6608,43,2],
[6609,9,1],[6609,18,1],[6609,28,1],[6610,12,1],[6610,25,7],
[6611,11,1],[6611,16,1],[6611,33,1],[6611,53,1],[6611,63,2],
[6612,56,2],[6613,17,1],[6613,53,1],[6614,44,1],[6614,66,1],
[6615,23,4],[6615,27,1],[6616,26,2],[6616,58,1],[6617,39,1],
[6617,53,2],[6618,3,13],[6618,5,1],[6618,6,1],[6618,27,1],[6618,28,1],
[6618,31,1],[6618,35,1],[6618,42,1],[6618,43,1],[6618,47,3],
[6618,48,14],[6618,50,1],[6618,74,1],[6619,17,1],[6619,53,1],
[6620,32,2],[6621,2,2],[6621,6,1],[6621,27,1],[6621,28,2],[6621,32,1],
[6622,15,1],[6622,27,1],[6622,45,1],[6622,46,1],[6623,4,1],
[6623,66,1],[6624,4,4],[6624,17,1],[6624,50,1],[6624,53,1],
[6624,55,1],[6625,25,1],[6625,66,1],[6626,2,1],[6626,3,1],[6626,4,1],
[6626,27,2],[6626,30,1],[6626,35,1],[6626,44,1],[6626,47,1],
[6626,74,1],[6627,2,1],[6627,3,1],[6627,8,1],[6627,20,2],[6627,23,2],
[6627,24,2],[6627,27,3],[6627,28,10],[6627,31,2],[6627,33,1],

[6627,39,1],[6627,43,4],[6628,1,1],[6628,13,1],[6628,20,2],
[6629,23,1],[6629,33,1],[6629,56,1],[6630,42,1],[6630,50,1],
[6631,44,1],[6631,47,1],[6632,22,1],[6632,24,1],[6632,26,1],
[6632,32,1],[6632,50,1],[6633,15,10],[6633,17,1],[6633,55,2],
[6634,3,1],[6634,28,1],[6635,3,1],[6635,26,1],[6636,28,1],[6636,69,1],
[6637,28,1],[6637,30,1],[6637,43,1],[6638,4,7],[6638,5,1],[6638,17,3],
[6638,27,1],[6638,28,1],[6638,38,1],[6638,40,2],[6638,44,1],
[6638,50,7],[6638,60,1],[6638,66,2],[6638,70,2],[6639,5,1],
[6639,22,5],[6639,24,3],[6639,27,10],[6639,28,3],[6639,35,12],
[6639,42,2],[6639,74,1],[6639,76,1],[6640,2,1],[6640,3,1],[6640,5,1],
[6640,21,1],[6640,28,1],[6640,38,1],[6640,39,2],[6640,56,1],
[6641,6,1],[6641,27,13],[6641,70,1],[6642,3,1],[6642,13,2],
[6642,23,1],[6642,27,1],[6642,29,1],[6642,32,2],[6642,35,1],
[6642,38,1],[6642,50,1],[6642,56,1],[6642,62,1],[6643,3,2],[6643,5,1],
[6643,16,1],[6643,21,10],[6643,22,9],[6643,23,2],[6643,24,2],
[6643,25,1],[6643,26,9],[6643,27,6],[6643,28,8],[6643,31,21],
[6643,33,4],[6643,35,1],[6643,38,2],[6643,40,1],[6643,43,6],
[6643,66,1],[6643,70,1],[6643,74,1],[6644,59,2],[6645,4,1],[6645,6,1],
[6645,13,2],[6645,37,1],[6645,51,1],[6645,56,1],[6645,72,1],
[6645,74,2],[6646,24,1],[6646,28,1],[6647,25,2],[6647,31,1],
[6648,21,1],[6648,28,1],[6649,23,4],[6649,28,3],[6649,35,1],
[6649,56,1],[6650,32,2],[6650,43,1],[6651,9,1],[6651,15,1],
[6652,28,1],[6652,52,1],[6652,62,1],[6653,2,6],[6654,8,1],[6654,27,2],
[6654,28,1],[6654,31,2],[6654,39,1],[6655,24,1],[6655,26,1],
[6655,32,1],[6656,40,3],[6656,69,1],[6656,70,9],[6656,72,1],
[6657,2,3],[6657,5,2],[6657,13,1],[6658,4,1],[6658,50,1],[6659,5,1],
[6659,24,2],[6659,47,4],[6659,51,20],[6659,56,1],[6660,23,2],
[6661,5,1],[6661,23,1],[6661,27,1],[6661,28,1],[6661,35,1],
[6661,56,1],[6662,1,89],[6662,9,2],[6662,10,1],[6662,12,3],
[6662,13,1],[6662,15,1],[6662,19,3],[6662,37,1],[6662,49,1],
[6662,65,1],[6662,67,1],[6663,13,1],[6663,19,1],[6664,28,1],
[6664,33,1],[6664,42,1],[6664,64,2],[6665,4,1],[6665,22,1],
[6665,23,1],[6666,1,1],[6666,25,1],[6666,53,1],[6667,44,1],
[6667,56,1],[6668,2,1],[6668,28,1],[6669,9,2],[6669,12,1],[6669,13,1],
[6669,15,1],[6669,17,1],[6669,45,1],[6669,53,2],[6669,54,1],
[6669,55,4],[6669,65,2],[6670,1,1],[6670,29,1],[6671,21,1],
[6671,26,16],[6671,28,1],[6671,42,1],[6671,43,19],[6671,66,2],
[6671,76,1],[6672,27,6],[6672,35,3],[6672,56,2],[6672,76,1],
[6673,23,1],[6673,28,1],[6674,3,1],[6674,4,2],[6674,5,1],[6674,22,1],
[6674,24,1],[6674,28,6],[6674,35,1],[6674,38,1],[6674,42,1],
[6674,44,2],[6674,47,6],[6674,50,1],[6674,52,1],[6674,55,1],
[6674,69,1],[6674,70,3],[6674,72,1],[6675,12,1],[6675,23,2],
[6675,24,3],[6675,27,3],[6675,28,1],[6675,30,1],[6675,31,3],
[6675,32,1],[6675,56,1],[6675,58,1],[6676,3,2],[6676,10,1],
[6676,13,1],[6676,21,1],[6676,23,4],[6676,24,2],[6676,26,8],
[6676,27,3],[6676,28,6],[6676,32,2],[6676,35,1],[6676,42,3],
[6676,43,11],[6676,48,1],[6677,70,1],[6677,74,1],[6678,9,1],
[6678,49,1],[6679,13,1],[6679,23,1],[6680,15,1],[6680,17,1],
[6680,28,1],[6680,53,3],[6680,55,1],[6681,23,1],[6681,25,9],
[6682,30,1],[6682,47,1],[6682,48,1],[6683,25,1],[6683,30,1],

[6683,34,1],[6683,39,1],[6683,68,1],[6684,3,1],[6684,4,1],[6684,15,2],
[6684,24,1],[6684,44,3],[6684,47,1],[6684,50,1],[6684,53,2],
[6684,55,3],[6684,66,5],[6684,74,1],[6685,9,1],[6685,10,2],
[6685,19,2],[6685,45,1],[6685,57,1],[6685,67,2],[6686,26,1],
[6686,32,3],[6686,56,1],[6687,1,1],[6687,3,2],[6687,5,1],[6687,8,1],
[6687,10,1],[6687,11,1],[6687,12,1],[6687,19,1],[6687,20,3],
[6687,22,1],[6687,23,2],[6687,24,3],[6687,25,2],[6687,26,1],
[6687,27,4],[6687,28,12],[6687,32,1],[6687,33,3],[6687,35,2],
[6687,37,1],[6687,42,2],[6687,43,1],[6687,44,1],[6687,45,3],
[6687,47,1],[6687,50,1],[6687,51,1],[6687,53,2],[6687,56,2],
[6687,62,2],[6687,65,1],[6687,66,2],[6687,68,1],[6687,69,2],
[6687,70,4],[6687,72,1],[6687,74,1],[6687,76,1],[6687,77,1],
[6687,79,1],[6688,46,1],[6688,60,1],[6689,28,4],[6690,2,1],
[6690,22,2],[6690,25,20],[6690,28,1],[6690,35,2],[6690,61,1],
[6690,62,18],[6690,63,1],[6690,66,2],[6690,76,2],[6691,1,6],
[6691,4,1],[6691,6,1],[6691,8,1],[6691,13,18],[6691,16,2],[6691,19,2],
[6691,23,1],[6691,24,4],[6691,25,1],[6691,28,1],[6691,29,1],
[6691,30,1],[6691,32,2],[6691,33,1],[6691,34,2],[6691,35,2],
[6691,37,5],[6691,39,3],[6691,41,1],[6691,42,1],[6691,48,1],
[6691,50,1],[6691,51,1],[6691,55,1],[6691,56,12],[6691,59,10],
[6691,60,2],[6691,62,2],[6691,64,1],[6691,65,1],[6691,66,1],
[6691,68,1],[6691,70,2],[6691,72,1],[6691,76,1],[6691,77,1],
[6692,32,1],[6692,59,4],[6693,2,2],[6693,28,1],[6693,55,1],[6694,2,1],
[6694,28,1],[6695,28,19],[6696,0,2],[6696,2,1],[6697,11,1],
[6697,12,1],[6697,14,1],[6697,15,1],[6697,17,1],[6697,35,1],
[6697,39,1],[6697,46,1],[6697,57,1],[6698,12,1],[6698,29,2],
[6699,74,2],[6700,4,3],[6700,15,1],[6700,42,1],[6700,45,1],
[6700,50,3],[6700,55,1],[6701,5,1],[6701,24,2],[6702,33,1],
[6702,68,3],[6702,70,1],[6702,74,2],[6702,76,1],[6703,2,1],[6703,5,2],
[6703,6,1],[6703,23,4],[6703,24,41],[6703,25,3],[6703,28,8],
[6703,35,1],[6703,38,1],[6703,40,1],[6703,42,2],[6703,43,1],
[6703,50,1],[6703,56,1],[6703,62,1],[6703,70,1],[6703,72,1],
[6704,28,1],[6704,72,1],[6705,25,1],[6705,62,1],[6705,63,1],
[6705,70,1],[6705,75,1],[6706,3,1],[6706,5,1],[6706,22,5],[6706,23,5],
[6706,24,6],[6706,27,3],[6706,28,5],[6706,33,1],[6706,35,7],
[6706,50,1],[6707,7,1],[6707,12,1],[6707,16,1],[6707,19,2],
[6707,29,1],[6707,34,1],[6707,54,1],[6707,55,1],[6707,65,1],
[6708,28,7],[6709,25,4],[6709,28,1],[6710,2,1],[6710,31,1],[6711,6,1],
[6711,9,1],[6711,28,1],[6711,29,1],[6712,1,1],[6712,58,1],[6713,38,1],
[6713,56,37],[6714,22,2],[6714,27,2],[6715,5,1],[6715,28,2],
[6715,35,2],[6716,1,1],[6716,29,1],[6717,28,2],[6717,32,1],
[6718,21,7],[6718,23,2],[6718,24,1],[6718,28,2],[6718,33,3],
[6718,43,2],[6718,47,2],[6718,56,17],[6718,61,1],[6718,62,12],
[6718,66,1],[6719,17,7],[6719,50,1],[6719,53,3],[6720,2,1],[6720,9,1],
[6720,12,1],[6720,21,1],[6720,30,1],[6720,53,2],[6721,22,1],
[6721,26,2],[6721,32,5],[6721,70,1],[6722,2,2],[6722,3,7],[6722,5,1],
[6722,23,7],[6722,28,1],[6722,32,5],[6722,42,5],[6722,44,1],
[6722,48,9],[6722,56,2],[6722,62,7],[6723,2,7],[6724,22,1],
[6724,24,1],[6724,35,1],[6724,56,3],[6724,74,1],[6725,3,1],
[6725,24,2],[6725,28,1],[6725,32,3],[6726,0,1],[6726,1,1],[6726,20,1],

[6726,30,1],[6726,32,2],[6726,34,5],[6726,37,1],[6726,43,1],
[6726,44,1],[6726,70,1],[6726,77,1],[6727,22,1],[6727,27,1],
[6727,35,3],[6728,1,1],[6728,3,1],[6728,10,1],[6728,15,4],[6728,17,1],
[6728,18,1],[6728,19,1],[6728,20,2],[6728,31,1],[6728,32,3],
[6728,33,2],[6728,35,1],[6728,42,1],[6728,45,1],[6728,46,1],
[6728,49,1],[6728,55,3],[6728,64,3],[6728,70,1],[6729,20,2],
[6730,10,1],[6730,17,1],[6731,2,2],[6731,45,1],[6732,4,2],[6732,10,1],
[6732,18,1],[6732,56,1],[6732,57,1],[6732,66,1],[6733,1,1],
[6733,13,1],[6733,65,1],[6734,25,2],[6734,26,1],[6735,13,1],
[6735,20,1],[6736,28,1],[6736,44,2],[6736,50,1],[6737,31,10],
[6737,33,3],[6738,50,2],[6739,11,1],[6739,15,1],[6739,16,1],
[6739,17,2],[6739,64,1],[6740,22,1],[6740,24,1],[6740,27,1],
[6740,28,3],[6740,33,1],[6741,3,1],[6741,24,1],[6741,27,1],
[6741,33,1],[6741,37,1],[6741,42,1],[6741,56,1],[6741,60,1],
[6741,69,1],[6741,76,1],[6742,35,1],[6742,74,1],[6743,20,1],
[6743,24,1],[6744,9,2],[6744,24,1],[6744,26,1],[6744,27,1],
[6744,28,3],[6744,42,1],[6744,43,1],[6745,9,1],[6745,21,1],
[6745,28,2],[6745,33,1],[6745,42,1],[6746,28,33],[6746,74,1],
[6747,1,2],[6748,22,1],[6748,23,2],[6748,28,1],[6748,35,1],
[6748,70,1],[6749,17,1],[6749,53,1],[6750,1,3],[6750,3,1],[6750,13,1],
[6750,15,1],[6750,20,1],[6750,24,1],[6750,56,2],[6750,59,1],
[6751,11,1],[6751,20,2],[6752,15,1],[6752,21,1],[6752,23,1],
[6753,5,1],[6753,24,1],[6753,26,1],[6753,28,16],[6753,33,9],
[6754,26,1],[6754,43,1],[6755,10,1],[6755,11,1],[6755,19,2],
[6755,39,1],[6755,41,1],[6755,45,1],[6755,46,1],[6755,67,1],
[6756,1,1],[6756,2,12],[6756,3,3],[6756,15,2],[6756,20,1],[6756,22,1],
[6756,23,3],[6756,25,1],[6756,30,4],[6756,32,2],[6756,39,2],
[6756,50,1],[6756,55,1],[6757,27,1],[6757,28,1],[6758,62,2],
[6759,1,4],[6759,9,1],[6759,12,2],[6759,13,3],[6759,16,5],[6759,17,1],
[6759,18,1],[6759,20,2],[6759,29,1],[6759,34,1],[6759,39,1],
[6759,45,1],[6759,53,1],[6759,73,1],[6759,78,1],[6760,28,1],
[6760,50,1],[6761,3,1],[6761,5,1],[6761,8,1],[6761,22,1],[6761,23,1],
[6761,26,1],[6761,27,5],[6761,30,1],[6761,33,2],[6761,42,1],
[6761,59,1],[6762,7,2],[6763,31,9],[6764,30,2],[6764,33,1],
[6764,46,2],[6765,9,1],[6765,32,2],[6765,77,1],[6766,35,1],
[6766,46,1],[6767,1,7],[6767,2,2],[6767,3,1],[6767,5,1],[6767,8,4],
[6767,9,2],[6767,10,1],[6767,12,5],[6767,13,6],[6767,20,14],
[6767,23,4],[6767,24,2],[6767,25,3],[6767,26,1],[6767,27,34],
[6767,28,5],[6767,29,1],[6767,31,1],[6767,34,4],[6767,35,9],
[6767,36,1],[6767,39,1],[6767,43,3],[6767,46,2],[6767,47,1],
[6767,50,1],[6767,52,3],[6767,54,1],[6767,56,1],[6767,58,1],
[6767,59,3],[6767,61,2],[6767,62,62],[6767,65,1],[6767,66,1],
[6767,70,2],[6767,74,1],[6767,76,2],[6767,77,1],[6767,78,1],
[6768,1,1],[6768,3,1],[6768,28,2],[6769,21,1],[6769,26,1],[6770,21,1],
[6770,27,4],[6770,28,1],[6770,52,1],[6771,32,2],[6771,42,1],
[6772,1,1],[6772,10,1],[6773,1,1],[6773,29,1],[6774,5,1],[6774,27,1],
[6774,56,1],[6774,69,1],[6775,15,2],[6775,53,1],[6775,55,1],
[6776,2,1],[6776,27,1],[6776,28,1],[6777,22,1],[6777,72,1],[6778,3,1],
[6778,22,1],[6778,23,1],[6778,24,1],[6778,28,1],[6778,32,5],
[6778,35,1],[6778,42,2],[6779,1,1],[6779,8,1],[6779,9,2],[6779,10,1],

[6779,11,1],[6779,12,4],[6779,13,4],[6779,14,1],[6779,15,2],
[6779,16,1],[6779,17,2],[6779,18,1],[6779,20,1],[6779,29,2],
[6779,30,1],[6779,37,2],[6779,41,1],[6779,45,1],[6779,55,1],
[6779,59,1],[6779,60,1],[6779,65,1],[6779,73,1],[6779,77,2],
[6780,1,1],[6780,2,1],[6780,3,1],[6780,4,1],[6780,9,1],[6780,21,1],
[6780,28,1],[6780,30,1],[6780,31,5],[6780,42,1],[6780,43,1],
[6780,54,1],[6780,63,1],[6781,9,1],[6781,17,1],[6781,45,1],
[6782,22,5],[6782,28,1],[6783,20,1],[6783,30,1],[6783,39,3],
[6784,28,1],[6784,76,1],[6785,13,1],[6785,39,1],[6785,55,1],
[6786,0,1],[6786,2,1],[6786,3,3],[6786,5,2],[6786,6,5],[6786,7,1],
[6786,10,1],[6786,13,1],[6786,18,1],[6786,20,5],[6786,21,2],
[6786,22,5],[6786,23,2],[6786,24,8],[6786,25,1],[6786,26,1],
[6786,27,7],[6786,32,4],[6786,35,3],[6786,37,1],[6786,38,1],
[6786,42,9],[6786,47,2],[6786,50,1],[6786,51,1],[6786,53,1],
[6786,55,4],[6786,58,1],[6786,59,1],[6786,65,1],[6786,66,1],
[6786,68,2],[6786,69,2],[6786,70,1],[6786,74,3],[6787,15,1],
[6787,53,1],[6787,55,1],[6788,56,2],[6788,69,1],[6789,2,3],
[6790,26,2],[6791,23,2],[6791,28,1],[6792,17,1],[6792,55,1],
[6793,1,1],[6793,3,1],[6794,37,4],[6794,56,10],[6794,63,1],
[6795,32,1],[6795,72,1],[6796,28,3],[6796,47,1],[6796,56,3],
[6797,1,8],[6797,20,4],[6797,23,3],[6797,24,2],[6797,26,1],
[6797,30,1],[6797,35,3],[6797,42,1],[6798,24,1],[6798,47,1],
[6798,62,1],[6799,23,1],[6799,27,1],[6799,31,1],[6800,24,4],
[6800,35,1],[6800,40,1],[6801,1,2],[6801,9,2],[6801,10,2],[6801,12,1],
[6801,14,1],[6801,19,1],[6801,20,1],[6801,34,1],[6801,41,1],
[6801,59,1],[6801,67,1],[6801,78,1],[6802,13,1],[6802,27,3],
[6802,30,1],[6802,43,1],[6803,6,2],[6803,12,1],[6803,15,1],
[6803,22,1],[6803,24,18],[6803,27,1],[6803,40,1],[6803,42,1],
[6803,44,5],[6803,51,10],[6803,66,1],[6803,69,3],[6804,23,17],
[6804,35,1],[6805,28,2],[6806,29,1],[6806,54,1],[6807,2,1],
[6807,28,1],[6808,5,1],[6808,26,1],[6808,28,1],[6809,8,1],[6809,28,3],
[6809,35,3],[6810,1,2],[6811,2,1],[6811,27,24],[6811,28,4],
[6811,34,5],[6811,35,3],[6811,66,1],[6812,28,2],[6812,35,1],
[6813,0,526],[6813,1,5],[6813,7,481],[6813,8,16],[6813,9,184],
[6813,10,21],[6813,11,22],[6813,12,26],[6813,13,6],[6813,14,4],
[6813,15,5],[6813,16,37],[6813,17,2],[6813,18,19],[6813,19,21],
[6813,20,3],[6813,28,21],[6813,29,5],[6813,30,10],[6813,31,8],
[6813,34,4],[6813,36,8],[6813,37,12],[6813,39,6],[6813,41,13],
[6813,45,22],[6813,46,16],[6813,49,4],[6813,53,2],[6813,54,10],
[6813,55,2],[6813,56,1],[6813,57,9],[6813,58,8],[6813,59,8],
[6813,60,3],[6813,62,10],[6813,63,1],[6813,64,6],[6813,65,25],
[6813,67,15],[6813,71,2],[6813,73,53],[6813,77,30],[6813,78,1],
[6814,21,1],[6814,31,1],[6814,70,1],[6815,5,1],[6815,68,1],
[6816,13,2],[6816,31,1],[6817,3,4],[6817,6,1],[6817,32,1],[6817,38,1],
[6817,47,1],[6817,52,1],[6817,62,1],[6818,2,3],[6818,3,2],[6818,21,1],
[6818,22,1],[6818,23,2],[6818,24,2],[6818,31,1],[6818,33,1],
[6818,42,1],[6818,47,3],[6818,60,1],[6819,28,2],[6819,33,1],
[6820,2,3],[6820,20,1],[6820,27,1],[6820,31,1],[6820,33,2],
[6820,44,1],[6820,60,1],[6821,1,1],[6821,35,1],[6821,39,2],
[6822,21,1],[6822,31,2],[6822,33,2],[6822,35,3],[6823,3,1],[6823,4,1],

[6823,8,1],[6823,10,2],[6823,11,1],[6823,17,1],[6823,22,8],
[6823,23,1],[6823,24,1],[6823,25,3],[6823,27,7],[6823,28,2],
[6823,30,6],[6823,32,26],[6823,43,1],[6823,45,1],[6823,50,1],
[6823,52,13],[6823,58,1],[6823,65,2],[6823,68,1],[6824,10,1],
[6824,55,1],[6825,28,1],[6825,62,4],[6826,31,2],[6827,3,1],[6827,5,1],
[6827,24,1],[6827,27,1],[6828,2,1],[6828,4,1],[6828,28,2],[6828,50,1],
[6828,53,2],[6829,5,1],[6829,20,7],[6829,21,1],[6829,22,6],
[6829,23,1],[6829,24,1],[6829,26,1],[6829,27,4],[6829,35,6],
[6829,75,1],[6830,5,1],[6830,16,1],[6830,21,1],[6830,32,5],
[6830,61,1],[6831,10,2],[6831,57,1],[6832,2,2],[6832,3,3],[6832,47,1],
[6833,27,1],[6833,56,1],[6833,68,1],[6833,70,1],[6834,27,1],
[6834,28,1],[6834,35,4],[6835,21,1],[6835,28,2],[6835,50,2],
[6835,70,1],[6836,1,1],[6836,15,1],[6836,18,1],[6836,34,1],
[6836,53,1],[6836,57,1],[6836,65,1],[6836,74,1],[6837,28,2],
[6837,40,1],[6837,42,1],[6838,25,1],[6838,63,1],[6838,73,1],
[6838,75,2],[6839,24,2],[6839,31,1],[6839,35,4],[6839,40,1],
[6839,50,1],[6839,52,18],[6839,62,1],[6839,66,2],[6840,32,2],
[6840,68,1],[6841,1,1],[6841,9,2],[6841,17,1],[6841,77,1],[6842,28,1],
[6842,76,1],[6843,9,1],[6843,10,2],[6843,11,1],[6843,15,1],
[6843,16,1],[6843,17,1],[6843,19,1],[6843,23,1],[6843,36,1],
[6843,37,1],[6843,41,6],[6843,45,1],[6843,46,1],[6843,54,1],
[6843,64,1],[6843,67,2],[6843,71,1],[6844,1,53],[6844,2,6],[6844,3,1],
[6844,6,2],[6844,8,4],[6844,12,81],[6844,13,28],[6844,15,5],
[6844,16,29],[6844,17,7],[6844,20,1],[6844,21,1],[6844,23,3],
[6844,24,1],[6844,27,1],[6844,28,1],[6844,29,15],[6844,30,3],
[6844,33,1],[6844,34,3],[6844,36,3],[6844,37,2],[6844,39,4],
[6844,43,1],[6844,48,1],[6844,53,7],[6844,55,2],[6844,59,2],
[6844,60,1],[6844,70,1],[6844,73,2],[6845,24,4],[6845,26,2],
[6845,28,1],[6846,2,1],[6846,27,1],[6846,33,1],[6846,39,2],
[6847,53,1],[6847,73,1],[6848,63,1],[6848,76,1],[6849,21,1],
[6849,22,3],[6849,23,7],[6849,24,1],[6849,27,1],[6849,28,1],
[6849,62,1],[6850,28,1],[6850,35,1],[6851,23,1],[6851,27,1],
[6851,32,1],[6851,70,1],[6852,1,1],[6852,39,1],[6853,63,5],
[6853,74,4],[6853,75,3],[6853,76,5],[6854,2,2],[6854,5,1],[6854,10,1],
[6854,21,1],[6854,22,1],[6854,24,1],[6854,27,1],[6854,28,1],
[6854,31,1],[6854,35,1],[6854,50,1],[6854,59,1],[6855,1,4],[6855,4,4],
[6855,8,1],[6855,9,8],[6855,10,13],[6855,11,3],[6855,13,1],
[6855,14,3],[6855,15,25],[6855,16,5],[6855,17,68],[6855,18,5],
[6855,19,8],[6855,20,3],[6855,22,1],[6855,29,2],[6855,32,1],
[6855,33,3],[6855,34,1],[6855,37,4],[6855,41,6],[6855,44,2],
[6855,45,9],[6855,46,3],[6855,49,2],[6855,50,10],[6855,53,54],
[6855,54,2],[6855,55,19],[6855,57,3],[6855,58,4],[6855,59,1],
[6855,65,6],[6855,66,1],[6855,71,2],[6855,72,1],[6855,73,1],
[6855,77,4],[6855,78,2],[6856,10,1],[6856,14,1],[6856,39,6],
[6856,58,1],[6856,59,3],[6856,64,1],[6857,17,1],[6857,53,1],
[6858,21,1],[6858,28,57],[6858,31,14],[6858,33,2],[6858,42,1],
[6858,43,3],[6859,9,1],[6859,25,2],[6859,28,1],[6859,50,3],
[6859,70,1],[6860,3,1],[6860,6,1],[6860,28,1],[6861,12,1],[6861,29,5],
[6861,31,1],[6862,27,3],[6863,23,1],[6863,28,1],[6864,2,5],[6864,5,2],
[6864,62,2],[6864,74,1],[6865,15,4],[6865,53,4],[6865,55,6],

[6866,63,1],[6866,74,1],[6867,1,2],[6868,25,1],[6868,61,1],
[6868,76,1],[6869,24,1],[6869,39,1],[6869,70,1],[6870,4,1],[6870,5,2],
[6870,6,2],[6870,22,3],[6870,23,10],[6870,24,1],[6870,27,1],
[6870,32,3],[6870,35,1],[6870,38,1],[6870,44,8],[6870,47,1],
[6870,48,1],[6870,51,1],[6870,66,10],[6871,21,2],[6871,26,1],
[6871,43,1],[6871,47,1],[6871,62,1],[6872,14,1],[6872,15,1],
[6872,43,1],[6872,49,1],[6872,50,1],[6872,59,3],[6873,4,1],
[6873,27,3],[6873,32,1],[6873,33,1],[6873,47,1],[6873,56,2],
[6874,1,13],[6874,2,2],[6874,4,1],[6874,8,1],[6874,12,30],[6874,13,4],
[6874,16,16],[6874,17,2],[6874,20,2],[6874,21,1],[6874,29,5],
[6874,30,2],[6874,31,1],[6874,33,2],[6874,34,2],[6874,36,3],
[6874,39,1],[6874,53,2],[6874,56,1],[6874,59,2],[6875,2,1],
[6875,23,1],[6875,27,1],[6875,46,1],[6876,3,1],[6876,28,1],
[6876,56,1],[6877,31,1],[6877,68,1],[6878,40,1],[6878,70,2],
[6878,72,1],[6879,2,3],[6879,4,2],[6879,6,3],[6879,10,1],[6879,19,1],
[6879,20,5],[6879,22,2],[6879,23,2],[6879,24,1],[6879,26,2],
[6879,27,6],[6879,32,1],[6879,35,1],[6879,37,1],[6879,42,2],
[6879,44,1],[6879,51,1],[6879,56,2],[6879,59,4],[6879,60,1],
[6879,75,1],[6880,25,3],[6880,28,1],[6881,26,1],[6881,27,13],
[6881,28,2],[6881,35,1],[6881,52,1],[6882,35,2],[6883,22,1],
[6883,30,1],[6883,31,1],[6884,2,1],[6884,20,1],[6884,21,1],
[6884,23,1],[6884,27,2],[6884,35,1],[6884,43,1],[6885,22,1],
[6885,24,1],[6885,27,1],[6885,28,1],[6885,31,1],[6886,24,2],
[6886,27,15],[6886,65,1],[6887,8,1],[6887,14,1],[6888,27,13],
[6888,35,17],[6889,53,1],[6889,59,1],[6890,33,1],[6890,35,1],
[6891,37,1],[6891,56,4],[6892,30,1],[6892,33,2],[6892,52,1],
[6893,0,2],[6893,2,1],[6893,3,3],[6893,4,1],[6893,5,1],[6893,6,1],
[6893,7,1],[6893,8,1],[6893,9,5],[6893,10,10],[6893,11,1],[6893,12,1],
[6893,13,1],[6893,14,4],[6893,15,57],[6893,16,6],[6893,17,11],
[6893,18,1],[6893,19,8],[6893,20,9],[6893,21,1],[6893,23,3],
[6893,24,2],[6893,25,2],[6893,26,1],[6893,27,1],[6893,28,7],
[6893,29,1],[6893,31,5],[6893,32,1],[6893,34,1],[6893,36,1],
[6893,38,2],[6893,39,3],[6893,40,2],[6893,41,9],[6893,43,1],
[6893,44,1],[6893,45,5],[6893,46,6],[6893,47,2],[6893,48,1],
[6893,49,1],[6893,53,7],[6893,54,2],[6893,55,44],[6893,56,1],
[6893,57,5],[6893,58,2],[6893,59,6],[6893,60,5],[6893,62,1],
[6893,64,2],[6893,65,8],[6893,66,1],[6893,67,3],[6893,68,1],
[6893,70,4],[6893,71,2],[6893,72,2],[6893,73,8],[6893,74,2],
[6893,75,10],[6893,76,3],[6893,77,4],[6893,78,2],[6893,79,4],
[6894,53,2],[6894,55,2],[6895,22,1],[6895,31,1],[6896,45,1],
[6896,55,1],[6897,28,1],[6897,66,1],[6898,22,1],[6898,27,13],
[6898,28,1],[6898,33,2],[6898,35,10],[6899,1,1],[6899,13,1],
[6899,17,1],[6900,28,1],[6900,32,1],[6901,1,1],[6901,29,1],
[6902,28,1],[6902,35,1],[6903,3,1],[6903,12,1],[6903,28,1],
[6903,35,1],[6903,42,1],[6903,47,1],[6904,43,1],[6904,72,1],
[6905,3,1],[6905,6,1],[6905,8,1],[6905,21,1],[6905,24,6],[6905,26,1],
[6905,27,1],[6905,32,1],[6905,35,6],[6905,40,1],[6905,42,1],
[6905,56,1],[6905,70,1],[6906,1,5],[6907,4,1],[6907,5,2],[6907,7,1],
[6907,20,2],[6907,22,1],[6907,23,7],[6907,24,2],[6907,31,10],
[6907,33,13],[6907,38,1],[6907,42,4],[6907,43,1],[6907,49,1],

[6907,70,2],[6908,5,1],[6908,23,1],[6908,28,11],[6908,29,1],
[6908,30,1],[6908,33,61],[6908,35,3],[6908,52,2],[6908,66,1],
[6908,70,1],[6908,72,1],[6909,5,1],[6909,17,1],[6909,19,1],
[6909,23,2],[6909,27,1],[6909,28,2],[6909,32,1],[6910,13,1],
[6910,20,1],[6911,2,2],[6911,3,1],[6911,4,1],[6911,12,1],[6911,20,1],
[6911,23,1],[6911,31,1],[6911,34,2],[6911,42,2],[6911,43,2],
[6911,50,1],[6911,51,1],[6911,55,1],[6911,56,1],[6911,60,1],
[6911,72,1],[6911,74,1],[6912,2,1],[6912,12,1],[6912,13,1],
[6912,18,1],[6912,24,1],[6912,25,1],[6912,26,1],[6912,27,2],
[6912,28,1],[6912,31,1],[6912,40,1],[6912,42,1],[6912,53,1],
[6912,73,1],[6913,28,1],[6913,35,1],[6914,3,1],[6914,50,1],
[6914,66,4],[6915,27,7],[6915,35,9],[6915,70,1],[6916,20,2],
[6917,3,1],[6917,4,32],[6917,5,2],[6917,22,1],[6917,23,1],[6917,24,2],
[6917,25,1],[6917,26,4],[6917,27,5],[6917,28,18],[6917,31,6],
[6917,32,1],[6917,33,1],[6917,35,2],[6917,38,21],[6917,42,3],
[6917,43,1],[6917,44,262],[6917,47,4],[6917,50,33],[6917,52,1],
[6917,56,1],[6917,62,1],[6917,66,238],[6917,70,1],[6917,74,1],
[6917,75,1],[6917,76,1],[6918,1,1],[6918,9,1],[6918,12,1],[6918,14,1],
[6918,15,2],[6918,24,1],[6918,25,1],[6918,28,2],[6918,29,1],
[6918,30,9],[6918,33,2],[6918,35,9],[6918,52,22],[6918,59,1],
[6918,67,1],[6919,5,1],[6919,12,1],[6919,15,1],[6919,21,1],
[6919,22,1],[6919,23,1],[6919,27,2],[6919,32,1],[6920,15,1],
[6920,19,1],[6920,45,1],[6921,3,3],[6921,5,1],[6921,43,1],[6921,44,1],
[6921,48,1],[6921,52,1],[6921,56,2],[6921,68,2],[6921,69,7],
[6922,4,1],[6922,26,1],[6922,28,3],[6922,44,13],[6922,50,1],
[6922,66,6],[6923,3,1],[6923,24,1],[6923,28,1],[6924,28,5],
[6925,28,2],[6926,2,1],[6926,35,1],[6927,0,1],[6927,3,1],[6927,10,1],
[6927,13,1],[6927,27,1],[6927,30,8],[6927,31,1],[6927,45,1],
[6927,49,1],[6927,55,1],[6927,71,1],[6927,77,1],[6928,4,1],
[6928,28,1],[6928,50,1],[6929,32,4],[6929,63,1],[6930,25,2],
[6930,75,1],[6931,2,1],[6931,10,1],[6931,21,1],[6931,24,1],
[6931,30,1],[6931,31,1],[6931,35,1],[6931,37,1],[6931,47,1],
[6931,55,1],[6931,60,1],[6932,20,2],[6932,27,1],[6932,42,1],
[6933,47,1],[6933,68,2],[6934,25,2],[6934,27,5],[6934,59,1],
[6935,23,3],[6936,1,1],[6936,13,1],[6937,47,1],[6937,48,2],
[6937,70,1],[6938,4,1],[6938,15,3],[6938,17,5],[6938,53,1],
[6938,55,1],[6939,26,1],[6939,33,1],[6940,20,1],[6940,21,1],
[6940,22,1],[6940,26,1],[6940,28,1],[6941,27,1],[6941,33,1],
[6942,31,1],[6942,70,1],[6943,2,3],[6943,28,2],[6943,31,1],
[6944,28,1],[6944,75,1],[6945,4,1],[6945,15,1],[6945,20,1],
[6945,55,1],[6945,74,1],[6946,2,1],[6946,5,1],[6946,6,2],[6946,21,6],
[6946,22,3],[6946,26,1],[6946,27,1],[6946,28,4],[6946,33,1],
[6946,35,2],[6946,39,3],[6946,63,1],[6946,74,1],[6947,3,1],
[6947,23,1],[6947,24,2],[6947,25,18],[6947,26,1],[6947,27,5],
[6947,35,2],[6947,37,1],[6947,48,1],[6948,1,1],[6948,3,1],[6948,4,1],
[6948,15,2],[6948,17,8],[6948,20,1],[6948,24,1],[6948,25,1],
[6948,27,1],[6948,30,1],[6948,33,1],[6948,38,1],[6948,41,1],
[6948,44,1],[6948,50,1],[6948,53,8],[6948,55,3],[6948,63,2],
[6948,66,1],[6948,68,1],[6948,70,1],[6949,28,1],[6949,32,1],
[6949,35,1],[6950,2,1],[6950,3,3],[6950,4,2],[6950,5,1],[6950,22,4],

[6950,24,1],[6950,25,2],[6950,27,5],[6950,28,1],[6950,30,1],
[6950,31,5],[6950,33,1],[6950,34,1],[6950,35,1],[6950,43,2],
[6950,50,1],[6950,53,1],[6950,56,1],[6950,77,1],[6951,28,1],
[6951,31,1],[6952,22,1],[6952,32,1],[6952,33,2],[6953,3,1],
[6953,22,1],[6953,23,4],[6953,24,55],[6953,27,1],[6953,31,1],
[6953,32,7],[6953,38,1],[6953,42,1],[6953,43,1],[6953,56,1],
[6953,60,1],[6953,63,2],[6953,66,1],[6953,70,2],[6954,1,2],[6955,3,3],
[6955,27,4],[6955,28,2],[6955,30,1],[6956,27,4],[6956,33,1],
[6956,35,3],[6956,39,1],[6956,43,1],[6957,23,8],[6957,24,2],
[6957,28,1],[6957,63,1],[6958,28,2],[6959,22,3],[6959,27,1],
[6959,35,1],[6959,78,1],[6960,32,1],[6960,62,1],[6960,76,1],
[6961,21,1],[6961,27,3],[6961,32,3],[6961,35,2],[6961,42,1],
[6962,20,2],[6962,23,2],[6963,21,2],[6963,25,2],[6963,28,2],
[6963,38,2],[6963,43,1],[6964,1,1],[6964,4,1],[6964,22,1],[6964,38,3],
[6964,44,1],[6965,3,1],[6965,32,1],[6965,66,1],[6966,28,2],
[6967,28,9],[6968,26,1],[6968,27,1],[6968,28,1],[6969,5,1],
[6969,22,3],[6969,27,3],[6969,32,1],[6970,20,1],[6970,23,4],
[6970,24,1],[6970,25,1],[6970,27,6],[6970,35,5],[6970,37,14],
[6970,38,2],[6971,2,2],[6971,5,2],[6971,15,1],[6971,22,1],[6971,26,2],
[6971,28,2],[6971,69,1],[6971,72,1],[6971,76,4],[6972,22,1],
[6972,32,3],[6973,26,4],[6974,1,1],[6974,3,1],[6974,5,1],[6974,6,1],
[6974,7,1],[6974,19,2],[6974,20,3],[6974,22,1],[6974,23,1],
[6974,24,3],[6974,25,1],[6974,27,4],[6974,28,9],[6974,30,3],
[6974,31,1],[6974,32,4],[6974,33,5],[6974,35,2],[6974,37,1],
[6974,39,4],[6974,40,1],[6974,42,2],[6974,43,3],[6974,47,1],
[6974,48,2],[6974,60,6],[6974,64,2],[6974,70,2],[6975,1,1],
[6975,20,1],[6975,21,2],[6975,22,1],[6975,23,4],[6975,24,2],
[6975,26,5],[6975,27,2],[6975,34,1],[6975,35,3],[6975,38,2],
[6975,43,2],[6975,47,3],[6975,59,1],[6975,70,1],[6975,72,1],
[6976,43,3],[6977,3,1],[6977,23,1],[6977,33,1],[6978,1,73],[6978,2,3],
[6978,5,1],[6978,7,2],[6978,8,6],[6978,9,2],[6978,11,1],[6978,12,251],
[6978,13,20],[6978,15,3],[6978,16,4],[6978,17,9],[6978,20,6],
[6978,21,2],[6978,23,1],[6978,29,44],[6978,31,9],[6978,34,7],
[6978,36,4],[6978,39,23],[6978,41,1],[6978,45,2],[6978,52,1],
[6978,53,6],[6978,55,9],[6978,58,1],[6978,59,3],[6978,64,1],
[6978,70,1],[6978,73,2],[6978,78,1],[6979,3,1],[6979,24,1],
[6979,25,1],[6979,32,10],[6979,33,1],[6979,38,1],[6979,46,1],
[6979,62,3],[6980,24,5],[6980,27,3],[6981,2,33],[6981,3,4],[6981,4,1],
[6981,5,6],[6981,6,1],[6981,8,1],[6981,12,1],[6981,14,1],[6981,22,5],
[6981,23,5],[6981,24,4],[6981,25,1],[6981,26,4],[6981,27,6],
[6981,28,4],[6981,30,7],[6981,31,4],[6981,32,4],[6981,33,2],
[6981,34,1],[6981,35,5],[6981,42,3],[6981,43,3],[6981,47,4],
[6981,50,1],[6981,53,1],[6981,55,1],[6981,56,3],[6981,60,3],
[6981,61,1],[6981,62,1],[6981,68,1],[6981,70,1],[6981,72,1],
[6982,23,1],[6982,28,1],[6982,76,1],[6983,2,1],[6983,21,1],
[6983,31,2],[6983,32,1],[6983,48,1],[6984,1,1],[6984,2,1],[6984,5,1],
[6984,9,1],[6984,10,1],[6984,11,1],[6984,24,1],[6984,28,19],
[6984,31,1],[6984,34,1],[6984,42,1],[6984,43,1],[6984,59,1],
[6984,63,4],[6984,65,1],[6984,74,8],[6984,75,8],[6984,76,15],
[6985,2,1],[6985,3,2],[6985,20,1],[6985,42,1],[6986,25,1],[6986,46,1],

[6986,73,1],[6987,2,1],[6987,22,1],[6987,31,1],[6988,2,1],[6988,27,1],
[6989,13,1],[6989,59,1],[6990,32,2],[6991,0,3],[6991,3,1],[6991,20,7],
[6991,22,2],[6991,23,9],[6991,24,73],[6991,27,73],[6991,28,1],
[6991,31,1],[6991,32,4],[6991,33,1],[6991,35,1],[6991,39,1],
[6991,40,2],[6991,42,1],[6991,47,2],[6991,50,1],[6991,62,1],
[6991,66,1],[6991,70,5],[6991,74,1],[6991,76,3],[6992,29,1],
[6992,56,2],[6993,51,6],[6994,56,2],[6995,20,1],[6995,22,1],
[6996,1,2],[6996,6,4],[6996,13,7],[6996,20,1],[6996,23,2],[6996,34,1],
[6996,73,1],[6997,0,3],[6997,1,11],[6997,5,1],[6997,7,3],[6997,8,1],
[6997,9,30],[6997,10,31],[6997,11,9],[6997,12,9],[6997,13,7],
[6997,14,10],[6997,15,8],[6997,16,16],[6997,17,4],[6997,18,10],
[6997,19,26],[6997,20,7],[6997,23,2],[6997,28,2],[6997,29,6],
[6997,30,3],[6997,34,7],[6997,36,7],[6997,37,13],[6997,39,9],
[6997,40,1],[6997,41,17],[6997,44,1],[6997,45,30],[6997,46,9],
[6997,49,8],[6997,53,7],[6997,54,4],[6997,55,9],[6997,57,12],
[6997,58,7],[6997,59,14],[6997,60,3],[6997,63,8],[6997,64,10],
[6997,65,17],[6997,67,14],[6997,70,1],[6997,71,13],[6997,73,14],
[6997,74,1],[6997,75,4],[6997,76,8],[6997,77,5],[6997,78,6],
[6997,79,3],[6998,23,2],[6999,6,1],[6999,22,1],[6999,23,1],
[6999,28,1],[7000,10,1],[7000,77,1],[7001,2,3],[7001,3,2],[7001,8,1],
[7001,26,2],[7001,27,1],[7001,30,4],[7001,31,1],[7001,33,2],
[7001,56,2],[7001,60,1],[7001,68,1],[7002,53,1],[7002,55,1],
[7003,12,1],[7003,71,1],[7003,73,19],[7004,2,2],[7004,13,2],
[7004,20,3],[7004,21,4],[7004,22,1],[7004,23,3],[7004,26,3],
[7004,28,2],[7004,33,2],[7004,36,1],[7005,53,2],[7006,22,1],
[7006,32,1],[7006,74,1],[7007,2,1],[7007,3,1],[7007,4,10],[7007,5,1],
[7007,15,1],[7007,17,8],[7007,21,1],[7007,23,2],[7007,24,2],
[7007,27,3],[7007,28,2],[7007,31,1],[7007,32,1],[7007,35,1],
[7007,38,1],[7007,40,1],[7007,42,1],[7007,44,8],[7007,50,27],
[7007,53,4],[7007,66,11],[7007,70,2],[7007,74,2],[7007,75,1],
[7008,8,1],[7008,31,1],[7008,62,1],[7009,9,2],[7010,33,1],[7010,59,1],
[7011,23,1],[7011,28,1],[7012,50,1],[7012,62,1],[7013,1,2],[7013,5,1],
[7013,11,1],[7013,12,2],[7013,25,1],[7013,26,1],[7013,28,3],
[7013,29,1],[7013,30,30],[7013,32,2],[7013,33,30],[7013,38,1],
[7013,52,13],[7014,1,2],[7014,12,1],[7014,59,1],[7015,44,1],
[7015,66,2],[7016,28,3],[7017,23,1],[7017,28,1],[7017,31,8],
[7017,47,1],[7017,48,2],[7018,16,1],[7018,17,1],[7018,28,1],
[7018,42,1],[7018,62,15],[7018,72,1],[7018,76,1],[7019,5,1],
[7019,21,3],[7019,35,1],[7020,2,2],[7020,15,1],[7020,23,1],
[7020,24,1],[7020,26,1],[7020,30,1],[7020,31,2],[7020,42,1],
[7021,1,15],[7021,8,2],[7021,13,15],[7021,17,1],[7021,29,1],
[7021,34,1],[7021,39,1],[7021,53,1],[7021,59,1],[7022,28,1],
[7022,70,1],[7023,8,1],[7023,9,1],[7023,65,1],[7024,26,2],[7025,11,2],
[7025,55,1],[7026,1,1],[7026,64,1],[7026,78,1],[7027,5,1],[7027,23,1],
[7027,25,1],[7027,27,1],[7028,27,2],[7029,11,1],[7029,15,8],
[7029,53,2],[7029,55,2],[7030,3,1],[7030,5,1],[7030,21,1],[7030,22,1],
[7030,27,2],[7030,37,2],[7030,47,2],[7030,60,1],[7031,1,2],[7031,3,2],
[7031,4,3],[7031,5,3],[7031,6,1],[7031,7,1],[7031,9,3],[7031,11,1],
[7031,12,1],[7031,15,7],[7031,16,2],[7031,17,29],[7031,19,2],
[7031,20,2],[7031,22,1],[7031,28,1],[7031,30,1],[7031,33,1],

[7031,37,7],[7031,38,1],[7031,40,1],[7031,42,1],[7031,43,4],
[7031,50,2],[7031,52,1],[7031,53,40],[7031,55,5],[7031,56,1],
[7031,60,1],[7031,71,1],[7031,73,2],[7032,23,1],[7032,50,2],
[7032,74,2],[7033,8,1],[7033,31,1],[7034,4,1],[7034,15,1],[7034,39,1],
[7034,63,1],[7035,12,1],[7035,15,4],[7035,28,1],[7035,55,4],
[7036,3,1],[7036,31,2],[7036,35,1],[7036,61,1],[7037,4,1],[7037,5,1],
[7037,15,6],[7037,17,1],[7037,20,2],[7037,26,1],[7037,31,3],
[7037,35,1],[7037,44,2],[7037,45,1],[7037,50,1],[7037,55,3],
[7037,60,1],[7037,66,3],[7037,74,1],[7037,75,1],[7038,42,2],
[7039,0,1],[7039,2,1],[7039,9,1],[7039,28,1],[7039,32,1],[7039,39,2],
[7040,27,5],[7040,28,1],[7041,9,2],[7041,10,1],[7041,13,1],
[7041,15,1],[7041,16,1],[7041,18,1],[7041,30,1],[7041,36,1],
[7041,37,1],[7041,41,1],[7041,45,1],[7041,49,1],[7041,58,2],
[7041,59,1],[7041,65,1],[7041,77,1],[7042,28,1],[7042,31,1],
[7042,63,1],[7042,75,1],[7042,76,1],[7043,47,12],[7044,35,3],
[7045,21,1],[7045,33,1],[7046,2,4],[7047,8,1],[7047,9,2],[7047,11,1],
[7047,16,1],[7047,17,34],[7047,18,1],[7047,34,1],[7047,41,2],
[7047,45,2],[7047,46,1],[7047,50,1],[7047,53,14],[7047,54,1],
[7047,55,3],[7047,58,1],[7047,65,1],[7047,67,1],[7047,69,1],
[7048,15,1],[7048,18,1],[7049,1,1],[7049,4,10],[7049,5,4],[7049,8,1],
[7049,9,6],[7049,10,4],[7049,11,1],[7049,13,2],[7049,15,30],
[7049,16,3],[7049,17,15],[7049,19,4],[7049,20,1],[7049,26,1],
[7049,28,1],[7049,39,1],[7049,41,1],[7049,43,1],[7049,44,4],
[7049,49,1],[7049,50,11],[7049,53,18],[7049,54,1],[7049,55,33],
[7049,56,1],[7049,58,1],[7049,64,1],[7049,65,1],[7049,66,8],
[7049,71,1],[7049,73,1],[7049,74,2],[7049,76,1],[7050,28,1],
[7050,35,1],[7050,74,1],[7051,3,1],[7051,22,1],[7051,23,1],
[7051,27,1],[7051,66,1],[7052,10,1],[7052,13,1],[7052,17,1],
[7052,71,1],[7053,2,1],[7053,34,1],[7054,27,1],[7054,31,1],
[7055,11,1],[7055,13,2],[7055,54,2],[7056,2,4],[7057,10,1],
[7057,19,1],[7058,2,1],[7058,28,1],[7058,45,1],[7058,50,1],
[7058,73,1],[7059,15,1],[7059,28,1],[7060,40,1],[7060,68,1],
[7060,69,1],[7061,5,1],[7061,27,4],[7061,33,1],[7061,47,5],[7062,2,4],
[7062,3,1],[7062,5,4],[7062,6,2],[7062,21,5],[7062,22,3],[7062,23,1],
[7062,24,1],[7062,25,3],[7062,27,1],[7062,33,2],[7062,35,5],
[7062,42,2],[7062,43,1],[7062,47,3],[7062,60,1],[7062,61,1],
[7062,74,1],[7063,2,2],[7064,28,37],[7064,63,1],[7064,76,1],
[7065,12,1],[7065,30,1],[7065,34,1],[7065,36,1],[7066,2,10],
[7066,28,1],[7066,30,1],[7067,5,1],[7067,35,9],[7068,0,1],[7068,1,78],
[7068,6,1],[7068,7,1],[7068,8,3],[7068,9,1],[7068,12,109],[7068,13,8],
[7068,15,2],[7068,17,1],[7068,20,1],[7068,29,57],[7068,30,1],
[7068,34,7],[7068,36,3],[7068,37,3],[7068,39,3],[7068,53,4],
[7068,55,3],[7068,58,1],[7068,59,2],[7068,73,1],[7068,77,5],
[7069,24,1],[7069,25,1],[7069,26,2],[7069,28,2],[7069,32,1],
[7069,43,1],[7069,45,1],[7069,59,1],[7070,15,2],[7070,20,1],
[7070,55,1],[7071,3,1],[7071,6,1],[7071,20,1],[7071,58,1],[7072,2,2],
[7072,5,2],[7072,28,1],[7072,35,1],[7072,62,1],[7072,76,1],[7073,2,1],
[7073,3,1],[7073,22,1],[7073,23,1],[7073,24,4],[7073,25,1],
[7073,27,4],[7073,42,1],[7074,40,2],[7075,21,2],[7075,23,2],
[7075,27,2],[7075,28,1],[7075,33,1],[7075,35,1],[7075,39,1],

[7075,43,1],[7076,20,1],[7076,25,1],[7077,28,2],[7077,43,10],
[7078,30,1],[7078,34,1],[7078,46,1],[7078,59,1],[7079,0,16],
[7079,1,32],[7079,2,4],[7079,4,2],[7079,5,2],[7079,7,26],[7079,8,30],
[7079,9,168],[7079,10,205],[7079,11,118],[7079,12,78],[7079,13,48],
[7079,14,74],[7079,15,44],[7079,16,131],[7079,17,29],[7079,18,72],
[7079,19,175],[7079,20,48],[7079,21,2],[7079,22,1],[7079,23,2],
[7079,25,4],[7079,26,1],[7079,27,3],[7079,28,7],[7079,29,15],
[7079,30,15],[7079,31,3],[7079,32,1],[7079,33,3],[7079,34,45],
[7079,35,2],[7079,36,38],[7079,37,73],[7079,38,1],[7079,39,34],
[7079,40,4],[7079,41,121],[7079,42,1],[7079,43,1],[7079,44,2],
[7079,45,117],[7079,46,43],[7079,47,1],[7079,49,41],[7079,51,2],
[7079,53,36],[7079,54,38],[7079,55,63],[7079,56,1],[7079,57,87],
[7079,58,76],[7079,59,64],[7079,60,20],[7079,62,1],[7079,63,121],
[7079,64,22],[7079,65,138],[7079,66,1],[7079,67,60],[7079,68,1],
[7079,69,1],[7079,70,6],[7079,71,59],[7079,72,1],[7079,73,58],
[7079,74,68],[7079,75,80],[7079,76,164],[7079,77,107],[7079,78,13],
[7080,2,2],[7081,20,1],[7081,77,1],[7082,20,1],[7082,25,5],
[7082,33,1],[7082,35,1],[7082,67,1],[7083,27,1],[7083,32,1],
[7084,21,1],[7084,33,2],[7084,43,1],[7085,28,7],[7086,2,1],[7086,5,1],
[7086,66,1],[7087,63,1],[7087,74,2],[7087,76,1],[7088,2,4],
[7088,46,1],[7089,20,1],[7089,31,1],[7090,17,3],[7090,53,1],
[7091,19,1],[7091,27,1],[7091,32,1],[7091,42,1],[7091,47,1],
[7091,59,1],[7091,70,1],[7091,78,1],[7092,3,1],[7092,13,2],
[7092,20,1],[7092,23,1],[7092,24,1],[7092,25,2],[7092,28,4],
[7092,32,1],[7092,42,1],[7092,71,1],[7092,77,1],[7093,2,1],[7093,3,2],
[7094,25,1],[7094,28,11],[7094,32,1],[7094,35,1],[7094,62,1],
[7095,23,1],[7095,27,3],[7095,35,3],[7095,59,1],[7096,32,2],
[7096,43,1],[7096,61,1],[7097,23,2],[7098,23,1],[7098,25,2],
[7098,27,2],[7098,28,1],[7098,32,7],[7098,35,3],[7098,52,1],
[7099,3,2],[7099,24,1],[7099,46,1],[7099,47,1],[7100,24,1],
[7100,28,1],[7101,25,1],[7101,40,1],[7101,44,1],[7101,62,1],
[7101,63,1],[7101,72,1],[7101,74,2],[7101,75,1],[7102,15,1],
[7102,22,1],[7102,31,2],[7102,38,1],[7102,44,7],[7102,50,1],
[7102,55,1],[7102,66,5],[7102,74,1],[7103,0,1],[7103,7,1],[7103,9,3],
[7103,10,1],[7103,12,1],[7103,28,2],[7103,42,1],[7103,57,1],
[7103,65,1],[7104,2,1],[7104,28,1],[7104,47,1],[7104,50,1],
[7104,62,1],[7104,66,1],[7105,15,1],[7105,17,1],[7106,1,1],[7106,3,1],
[7106,20,1],[7106,21,1],[7106,23,3],[7106,24,1],[7106,25,1],
[7106,27,1],[7107,2,1],[7107,17,1],[7107,23,2],[7107,28,8],
[7108,28,2],[7109,23,1],[7109,24,1],[7109,74,1],[7110,28,2],
[7110,31,1],[7110,35,3],[7110,48,1],[7111,1,1],[7111,28,1],[7112,5,1],
[7112,6,1],[7112,28,3],[7113,15,1],[7113,53,2],[7114,0,1],[7114,3,1],
[7114,10,1],[7114,17,1],[7114,23,1],[7114,32,1],[7114,33,15],
[7114,35,3],[7114,42,1],[7114,45,1],[7114,51,1],[7114,52,1],
[7114,63,1],[7114,66,1],[7114,72,1],[7114,74,1],[7114,76,2],
[7115,3,1],[7115,47,1],[7116,6,1],[7116,23,1],[7116,32,3],[7116,35,2],
[7116,38,1],[7116,44,1],[7116,47,9],[7116,51,4],[7116,63,1],
[7116,68,1],[7116,70,1],[7116,73,1],[7116,74,3],[7117,24,2],
[7117,32,4],[7118,21,4],[7118,28,1],[7118,31,1],[7119,13,1],
[7119,22,2],[7119,39,1],[7119,57,1],[7120,1,5],[7120,5,4],[7120,9,1],

[7120,10,1],[7120,12,2],[7120,13,3],[7120,15,3],[7120,16,1],
[7120,17,1],[7120,21,1],[7120,23,2],[7120,24,1],[7120,31,1],
[7120,32,1],[7120,33,1],[7120,34,1],[7120,35,1],[7120,36,1],
[7120,37,1],[7120,38,1],[7120,39,1],[7120,41,2],[7120,44,1],
[7120,45,2],[7120,50,1],[7120,53,1],[7120,55,6],[7120,58,1],
[7120,59,2],[7120,60,1],[7120,68,1],[7120,70,1],[7120,77,1],
[7121,27,3],[7121,31,2],[7121,35,3],[7121,43,1],[7121,70,1],
[7121,77,1],[7122,13,1],[7122,18,1],[7122,60,1],[7122,77,1],
[7122,78,1],[7123,20,2],[7124,5,1],[7124,22,1],[7124,28,1],
[7124,66,1],[7124,68,3],[7125,31,2],[7126,13,1],[7126,21,1],
[7126,22,1],[7126,23,1],[7126,27,2],[7126,31,2],[7126,42,2],
[7127,3,2],[7128,10,1],[7128,13,1],[7128,22,1],[7128,23,1],
[7128,45,1],[7129,23,1],[7129,24,2],[7129,27,1],[7129,31,1],
[7129,44,1],[7129,56,14],[7129,74,1],[7130,8,1],[7130,25,1],
[7130,27,1],[7130,43,1],[7130,53,1],[7131,15,2],[7132,0,1],[7132,1,2],
[7132,2,3],[7132,4,85],[7132,5,22],[7132,9,5],[7132,10,3],[7132,11,2],
[7132,13,1],[7132,14,4],[7132,16,3],[7132,17,1],[7132,19,5],
[7132,22,4],[7132,24,1],[7132,25,2],[7132,27,4],[7132,28,13],
[7132,30,1],[7132,31,1],[7132,32,1],[7132,33,1],[7132,34,1],
[7132,37,1],[7132,38,10],[7132,40,1],[7132,43,1],[7132,44,22],
[7132,47,2],[7132,49,1],[7132,50,92],[7132,56,1],[7132,57,3],
[7132,58,2],[7132,59,1],[7132,62,1],[7132,63,3],[7132,65,4],
[7132,66,37],[7132,68,1],[7132,69,1],[7132,71,1],[7132,72,2],
[7132,73,1],[7132,74,1],[7132,76,2],[7133,20,1],[7133,39,1],
[7134,5,1],[7134,9,1],[7134,11,1],[7134,13,1],[7134,15,22],
[7134,17,23],[7134,19,1],[7134,20,1],[7134,22,1],[7134,24,1],
[7134,28,1],[7134,35,1],[7134,45,3],[7134,50,5],[7134,53,27],
[7134,55,24],[7134,60,1],[7134,65,1],[7134,71,1],[7134,73,1],
[7134,75,1],[7134,77,1],[7135,15,1],[7135,55,1],[7136,2,1],[7136,3,1],
[7136,23,8],[7136,24,5],[7136,25,5],[7136,26,1],[7136,27,7],
[7136,28,1],[7136,31,1],[7136,32,1],[7136,50,1],[7136,52,1],
[7136,62,1],[7136,65,1],[7136,75,1],[7137,1,2],[7137,3,2],[7137,5,1],
[7137,13,1],[7137,21,1],[7137,23,1],[7137,24,15],[7137,27,3],
[7137,28,5],[7137,30,1],[7137,31,2],[7137,32,8],[7137,33,2],
[7137,42,1],[7137,47,1],[7137,77,1],[7137,78,1],[7138,37,5],
[7139,20,2],[7139,31,1],[7139,39,1],[7140,22,1],[7140,62,1],
[7141,2,3],[7142,75,1],[7142,76,1],[7143,4,1],[7143,33,1],[7143,69,1],
[7144,22,2],[7144,23,2],[7144,25,1],[7144,31,2],[7144,42,1],
[7144,43,1],[7145,21,1],[7145,24,1],[7145,25,3],[7145,27,2],
[7145,32,4],[7145,33,1],[7145,44,1],[7145,76,1],[7146,3,1],
[7146,32,3],[7147,3,1],[7147,43,2],[7148,21,1],[7148,50,1],[7149,2,1],
[7149,22,1],[7149,28,1],[7149,69,1],[7150,0,1],[7150,2,12],[7150,7,1],
[7150,8,13],[7150,9,1],[7150,10,1],[7150,21,1],[7150,23,3],
[7150,24,1],[7150,28,1],[7150,29,1],[7150,30,2],[7150,31,15],
[7150,34,2],[7150,36,3],[7150,39,30],[7150,41,1],[7150,42,1],
[7150,43,3],[7150,53,1],[7150,55,1],[7150,56,1],[7150,72,1],
[7150,78,2],[7151,40,1],[7151,76,1],[7152,21,2],[7152,26,1],
[7152,31,2],[7152,33,1],[7152,43,1],[7153,28,1],[7153,31,2],
[7154,9,1],[7154,17,1],[7154,28,2],[7154,53,2],[7154,55,2],[7155,3,2],
[7155,16,1],[7155,17,1],[7155,20,1],[7155,23,1],[7155,24,1],

[7156,55,1],[7156,71,1],[7157,1,1],[7157,2,1],[7157,9,1],[7157,11,1],
[7157,20,2],[7157,24,3],[7157,31,1],[7157,34,1],[7157,39,1],
[7157,42,2],[7157,45,1],[7157,49,1],[7157,58,1],[7157,60,22],
[7157,66,1],[7157,78,2],[7158,1,4],[7158,3,1],[7158,5,1],[7158,6,1],
[7158,10,1],[7158,13,7],[7158,19,1],[7158,20,1],[7158,21,1],
[7158,23,1],[7158,24,2],[7158,28,1],[7158,30,3],[7158,33,1],
[7158,34,1],[7158,35,2],[7158,36,1],[7158,47,1],[7158,56,27],
[7158,58,1],[7158,66,1],[7158,71,1],[7158,72,1],[7158,75,1],
[7158,78,1],[7159,15,1],[7159,17,2],[7160,51,2],[7160,75,1],
[7161,3,1],[7161,5,1],[7161,31,1],[7162,23,2],[7162,28,1],[7163,2,7],
[7164,2,21],[7164,6,1],[7164,21,2],[7164,22,1],[7164,26,1],
[7164,28,1],[7164,31,11],[7164,32,3],[7164,35,2],[7164,42,4],
[7164,56,1],[7164,62,9],[7165,21,2],[7166,36,1],[7166,54,1],
[7167,28,6],[7168,9,1],[7168,39,8],[7168,64,1],[7169,30,2],[7170,2,1],
[7170,3,1],[7170,5,2],[7170,8,2],[7170,11,1],[7170,13,1],[7170,15,1],
[7170,17,2],[7170,19,1],[7170,20,11],[7170,21,13],[7170,22,27],
[7170,23,30],[7170,24,7],[7170,25,7],[7170,26,17],[7170,27,28],
[7170,28,5],[7170,29,1],[7170,31,32],[7170,32,2],[7170,33,3],
[7170,35,21],[7170,37,1],[7170,38,1],[7170,42,6],[7170,43,25],
[7170,48,1],[7170,51,1],[7171,2,1],[7171,3,2],[7171,24,1],[7171,25,1],
[7171,27,1],[7171,32,1],[7171,33,2],[7171,35,2],[7171,37,1],
[7171,44,2],[7171,62,1],[7171,66,1],[7171,76,1],[7172,48,1],
[7172,74,1],[7173,22,1],[7173,33,1],[7173,60,1],[7174,1,1],
[7174,15,2],[7174,74,1],[7175,22,2],[7175,24,1],[7175,31,1],
[7175,68,1],[7176,28,1],[7176,33,1],[7176,72,1],[7177,2,1],
[7177,25,1],[7177,28,1],[7178,4,1],[7178,28,1],[7178,38,1],
[7178,62,1],[7178,66,1],[7179,69,3],[7180,28,2],[7180,70,1],
[7181,22,1],[7181,35,1],[7182,2,1],[7182,27,2],[7182,35,1],
[7182,36,1],[7183,74,1],[7183,76,1],[7184,4,2],[7184,50,2],
[7185,21,2],[7185,31,3],[7185,43,1],[7186,3,4],[7186,4,1],[7186,5,2],
[7186,6,1],[7186,23,7],[7186,24,5],[7186,25,2226],[7186,27,3],
[7186,28,40],[7186,31,2],[7186,32,1],[7186,38,2],[7186,39,2],
[7186,40,17],[7186,42,1],[7186,43,1],[7186,44,6],[7186,47,2],
[7186,50,6],[7186,52,3],[7186,56,2],[7186,61,1],[7186,62,4],
[7186,63,9],[7186,66,5],[7186,68,7],[7186,69,3],[7186,70,17],
[7186,72,6],[7186,74,4],[7186,75,2],[7186,76,7],[7187,31,1],
[7187,43,1],[7188,17,1],[7188,29,3],[7188,67,1],[7189,4,1],
[7189,17,1],[7189,28,1],[7190,27,1],[7190,32,2],[7191,20,1],
[7191,27,1],[7191,28,3],[7191,31,1],[7192,28,1],[7192,56,4],
[7193,9,1],[7193,28,6],[7193,44,1],[7193,53,1],[7193,57,1],[7194,1,1],
[7194,31,5],[7194,59,1],[7195,6,3],[7195,21,3],[7195,26,7],
[7195,42,1],[7195,43,1],[7195,47,1],[7195,48,5],[7196,3,10],
[7196,20,2],[7196,23,2],[7196,26,3],[7196,27,1],[7196,28,2],
[7196,31,2],[7196,32,1],[7196,33,1],[7196,42,1],[7196,43,1],
[7197,32,3],[7197,35,1],[7197,48,2],[7197,56,3],[7197,70,1],
[7198,23,1],[7198,31,1],[7199,39,1],[7199,59,1],[7200,23,1],
[7200,78,1],[7201,5,6],[7201,21,1],[7202,25,2],[7202,26,1],
[7202,28,1],[7203,23,3],[7204,9,1],[7204,14,1],[7204,32,1],[7205,4,1],
[7205,12,1],[7205,13,1],[7205,20,1],[7205,27,1],[7205,35,2],
[7205,47,1],[7205,56,2],[7205,61,1],[7205,70,2],[7206,22,3],

[7206,23,1],[7206,24,5],[7206,26,1],[7206,27,3],[7206,32,3],
[7206,35,3],[7207,4,1],[7207,23,1],[7207,27,1],[7207,28,1],
[7207,31,1],[7207,38,4],[7207,44,21],[7207,50,2],[7207,66,29],
[7207,76,1],[7208,27,1],[7208,28,1],[7208,38,1],[7208,47,1],
[7209,27,1],[7209,28,47],[7209,32,1],[7209,63,17],[7209,68,1],
[7209,69,1],[7209,74,10],[7209,75,15],[7209,76,16],[7210,25,2],
[7211,23,2],[7211,29,1],[7211,35,1],[7211,79,1],[7212,1,1],
[7212,20,1],[7212,62,1],[7213,25,1],[7213,35,1],[7213,39,1],
[7214,32,3],[7215,3,2],[7215,28,1],[7215,51,1],[7216,20,1],
[7216,35,2],[7217,2,4],[7217,3,1],[7217,5,2],[7217,21,1],[7217,22,2],
[7217,23,5],[7217,24,6],[7217,25,2],[7217,26,5],[7217,27,6],
[7217,28,7],[7217,30,1],[7217,31,4],[7217,32,7],[7217,33,3],
[7217,35,1],[7217,42,1],[7217,43,3],[7217,46,2],[7217,47,2],
[7217,48,1],[7218,50,1],[7218,66,2],[7219,28,1],[7219,44,1],
[7220,16,1],[7220,67,1],[7220,77,1],[7221,28,1],[7221,44,1],
[7222,5,1],[7222,21,1],[7222,23,3],[7222,24,1],[7222,28,1],
[7222,40,1],[7222,50,1],[7222,56,1],[7222,62,1],[7222,68,1],
[7222,70,1],[7223,17,1],[7223,41,1],[7223,55,1],[7224,32,2],
[7224,33,1],[7224,35,1],[7224,43,1],[7225,3,1],[7225,24,1],
[7225,26,1],[7225,27,1],[7225,37,1],[7225,42,1],[7225,66,1],
[7226,27,1],[7226,70,1],[7227,53,3],[7228,6,2],[7228,15,1],
[7228,20,1],[7228,28,2],[7228,35,1],[7228,47,1],[7228,51,1],
[7228,56,2],[7228,63,1],[7228,66,1],[7228,70,1],[7228,74,3],
[7229,12,1],[7229,39,1],[7230,5,2],[7231,31,2],[7232,28,1],
[7232,32,1],[7232,42,1],[7232,63,1],[7233,27,2],[7233,35,2],
[7233,70,1],[7234,3,3],[7234,22,1],[7234,35,1],[7234,43,1],
[7234,56,1],[7235,0,1],[7235,1,1],[7235,9,2],[7235,10,1],[7235,12,1],
[7235,17,1],[7235,20,2],[7235,28,1],[7235,29,1],[7235,30,1],
[7235,39,1],[7235,41,1],[7235,49,1],[7235,54,1],[7235,58,1],
[7235,59,3],[7235,62,4],[7235,77,1],[7236,51,2],[7236,68,1],
[7237,1,1],[7237,4,2],[7237,10,2],[7237,17,3],[7237,53,1],[7237,55,5],
[7238,24,1],[7238,56,1],[7239,4,1],[7239,6,1],[7239,22,1],[7239,23,1],
[7239,27,3],[7239,31,1],[7239,43,1],[7239,72,1],[7239,79,1],
[7240,5,1],[7240,28,2],[7240,60,1],[7240,63,1],[7241,27,1],
[7241,28,1],[7241,59,1],[7242,2,1],[7242,31,6],[7243,12,2],
[7243,27,1],[7243,30,1],[7244,11,1],[7244,37,1],[7244,59,1],
[7244,60,1],[7245,0,2],[7245,1,1],[7245,2,1],[7245,4,1],[7245,7,2],
[7245,8,2],[7245,9,7],[7245,10,8],[7245,13,13],[7245,15,2],
[7245,16,4],[7245,17,2],[7245,19,5],[7245,20,4],[7245,22,1],
[7245,26,1],[7245,28,1],[7245,29,1],[7245,30,3],[7245,34,2],
[7245,36,1],[7245,37,1],[7245,38,2],[7245,39,1],[7245,41,2],
[7245,43,1],[7245,44,1],[7245,45,5],[7245,49,4],[7245,50,1],
[7245,53,1],[7245,55,2],[7245,57,2],[7245,58,2],[7245,59,3],
[7245,65,5],[7245,66,4],[7245,67,2],[7245,71,4],[7245,73,1],
[7245,77,3],[7245,78,1],[7246,15,2],[7247,8,1],[7247,9,1],[7247,33,1],
[7247,35,1],[7247,37,1],[7248,5,2],[7248,27,1],[7248,28,3],
[7248,33,2],[7248,63,1],[7249,2,9],[7250,23,1],[7250,24,1],
[7250,25,2],[7250,26,1],[7250,32,3],[7250,62,2],[7251,2,1],[7251,3,1],
[7251,28,3],[7251,30,2],[7251,33,1],[7251,41,1],[7251,78,1],
[7252,2,1],[7252,3,1],[7252,4,1],[7252,13,1],[7252,22,1],[7252,23,1],

[7252,24,2],[7252,27,1],[7252,33,2],[7252,38,1],[7252,60,1],
[7252,62,2],[7253,8,2],[7253,23,1],[7254,26,1],[7254,50,1],[7255,2,3],
[7256,22,1],[7256,23,1],[7256,24,1],[7256,35,2],[7256,62,3],
[7257,32,2],[7257,66,1],[7258,44,1],[7258,47,10],[7259,22,1],
[7259,26,1],[7259,28,1],[7260,47,1],[7260,61,1],[7261,1,4],
[7261,12,7],[7261,16,5],[7261,23,1],[7261,25,1],[7261,29,1],
[7261,36,1],[7261,39,1],[7261,55,1],[7262,74,7],[7262,75,3],
[7263,2,2],[7263,31,1],[7264,1,1],[7264,23,1],[7264,24,2],[7264,26,1],
[7264,32,3],[7264,35,1],[7264,38,1],[7264,44,1],[7264,47,4],
[7264,56,1],[7265,28,5],[7265,50,1],[7266,23,2],[7267,13,2],
[7268,62,2],[7269,6,1],[7269,8,1],[7269,26,3],[7269,27,2],[7269,33,2],
[7269,35,4],[7269,43,1],[7269,47,1],[7270,2,4],[7271,3,3],
[7271,24,12],[7271,28,3],[7272,51,1],[7272,68,1],[7273,4,6],
[7273,17,6],[7273,23,2],[7273,44,3],[7273,50,7],[7273,53,8],
[7273,55,9],[7273,65,1],[7274,22,1],[7274,26,1],[7274,27,3],
[7274,35,3],[7274,70,1],[7275,24,1],[7275,27,3],[7276,26,1],
[7276,42,1],[7277,9,1],[7277,20,1],[7278,6,1],[7278,22,4],[7278,26,3],
[7278,27,3],[7278,33,1],[7278,43,1],[7278,72,1],[7278,75,1],
[7279,12,1],[7279,13,8],[7279,15,1],[7279,28,3],[7279,34,1],
[7279,67,1],[7280,17,1],[7280,33,1],[7281,22,1],[7281,27,6],
[7281,28,6],[7281,35,4],[7282,2,2],[7282,24,2],[7282,26,1],
[7282,28,1],[7282,57,1],[7283,43,8],[7283,50,1],[7284,2,1],[7284,4,1],
[7284,5,1],[7284,14,1],[7284,20,3],[7284,21,1],[7284,23,3],
[7284,24,1],[7284,25,6],[7284,26,2],[7284,27,1],[7284,28,2],
[7284,31,13],[7284,32,1],[7284,33,1],[7284,34,1],[7284,35,2],
[7284,42,3],[7284,43,6],[7284,47,2],[7284,75,1],[7285,23,2],
[7285,24,1],[7285,36,1],[7285,47,2],[7285,48,2],[7285,56,2],
[7285,77,1],[7286,1,2],[7286,4,2],[7286,5,1],[7286,8,1],[7286,9,1],
[7286,10,2],[7286,13,1],[7286,15,2],[7286,16,2],[7286,19,1],
[7286,20,1],[7286,23,4],[7286,24,2],[7286,27,1],[7286,32,2],
[7286,36,1],[7286,38,1],[7286,44,1],[7286,50,3],[7286,53,3],
[7286,55,1],[7286,65,1],[7286,66,2],[7286,67,1],[7286,77,2],
[7287,3,1],[7287,22,1],[7287,28,5],[7287,43,2],[7288,28,2],
[7288,47,1],[7288,75,1],[7288,76,2],[7289,28,10],[7289,73,1],
[7290,3,1],[7290,20,1],[7291,53,1],[7291,55,1],[7292,7,1],[7292,9,1],
[7292,10,1],[7292,11,1],[7292,13,1],[7292,14,1],[7292,16,2],
[7292,18,1],[7292,19,2],[7292,21,1],[7292,24,1],[7292,26,1],
[7292,27,1],[7292,30,1],[7292,31,1],[7292,32,2],[7292,33,1],
[7292,37,1],[7292,39,2],[7292,41,1],[7292,43,1],[7292,44,1],
[7292,45,1],[7292,46,1],[7292,57,1],[7292,62,2],[7292,65,2],
[7292,70,1],[7292,71,1],[7292,72,1],[7292,74,1],[7292,76,2],
[7293,28,1],[7293,35,1],[7294,24,1],[7294,28,4],[7295,23,1],
[7295,28,1],[7295,33,1],[7296,44,2],[7297,32,2],[7297,35,15],
[7297,44,1],[7297,61,1],[7297,66,1],[7298,28,1],[7298,32,4],
[7299,2,2],[7299,31,1],[7300,17,2],[7300,53,4],[7301,5,1],[7301,15,1],
[7301,21,1],[7301,22,2],[7301,24,2],[7301,27,3],[7301,28,1],
[7301,33,1],[7301,35,1],[7301,46,1],[7301,56,1],[7301,57,1],
[7302,2,1],[7302,3,2],[7302,23,3],[7302,30,1],[7302,32,1],[7302,43,2],
[7302,72,1],[7303,32,1],[7303,35,1],[7304,53,1],[7304,55,1],
[7304,56,1],[7304,66,1],[7304,70,1],[7305,26,1],[7305,28,2],

[7306,27,3],[7307,1,2],[7307,7,1],[7307,15,1],[7307,36,1],[7307,51,1],
[7307,55,1],[7307,57,1],[7307,58,1],[7307,64,1],[7308,24,1],
[7308,27,1],[7309,3,2],[7310,20,2],[7310,78,1],[7311,2,2],[7311,3,1],
[7311,22,1],[7312,2,1],[7312,22,1],[7312,23,1],[7312,25,1],
[7312,27,1],[7312,44,1],[7312,66,1],[7312,68,1],[7312,70,5],
[7312,74,2],[7312,76,1],[7313,24,9],[7313,28,1],[7313,42,1],
[7313,75,1],[7314,27,1],[7314,35,2],[7315,1,1],[7315,7,5],[7315,9,1],
[7316,28,1],[7316,76,1],[7317,2,13],[7317,3,11],[7317,4,13],
[7317,5,15],[7317,6,8],[7317,21,4],[7317,22,5],[7317,23,35],
[7317,24,15],[7317,25,17],[7317,26,17],[7317,27,20],[7317,28,99],
[7317,31,13],[7317,32,5],[7317,33,9],[7317,35,23],[7317,38,17],
[7317,40,18],[7317,42,11],[7317,43,8],[7317,44,21],[7317,47,5],
[7317,48,5],[7317,50,20],[7317,51,1],[7317,52,3],[7317,56,8],
[7317,61,1],[7317,62,17],[7317,63,8],[7317,66,24],[7317,68,8],
[7317,69,5],[7317,70,40],[7317,72,13],[7317,74,15],[7317,75,11],
[7317,76,12],[7318,2,4],[7318,20,1],[7319,23,1],[7319,27,1],
[7319,28,1],[7320,32,1],[7320,35,1],[7321,1,1],[7321,15,1],
[7321,17,6],[7321,53,3],[7321,55,2],[7322,20,10],[7322,22,2],
[7322,23,26],[7322,24,2],[7322,25,1],[7322,27,1],[7322,35,5],
[7322,38,1],[7322,40,1],[7322,51,1],[7322,56,1],[7322,66,2],
[7322,79,1],[7323,23,1],[7323,70,1],[7324,20,1],[7324,30,1],
[7325,2,7],[7325,3,1],[7325,5,1],[7325,10,1],[7325,22,2],[7325,23,2],
[7325,26,3],[7325,33,2],[7325,35,1],[7325,36,1],[7325,46,1],
[7325,59,1],[7326,5,1],[7326,27,1],[7327,73,1],[7327,77,1],
[7328,11,1],[7328,13,1],[7328,20,10],[7328,35,1],[7328,59,1],
[7328,65,1],[7329,22,1],[7329,32,1],[7329,35,1],[7329,76,1],
[7330,25,2],[7330,30,5],[7330,33,1],[7330,35,10],[7330,38,2],
[7330,50,1],[7330,52,11],[7330,76,1],[7331,42,4],[7331,43,1],
[7332,27,1],[7332,42,1],[7333,3,1],[7333,38,1],[7334,31,1],
[7334,59,1],[7335,5,1],[7335,23,2],[7335,25,1],[7335,28,2],
[7335,50,1],[7335,52,1],[7335,62,1],[7336,12,1],[7336,25,1],
[7336,37,7],[7337,23,1],[7337,24,8],[7337,25,1],[7337,26,1],
[7337,27,2],[7337,28,1],[7337,32,1],[7337,35,1],[7337,48,1],
[7338,36,1],[7338,39,1],[7339,45,1],[7339,78,1],[7340,6,1],
[7340,22,1],[7340,35,2],[7340,50,1],[7340,66,1],[7341,2,1],
[7341,64,1],[7342,23,3],[7342,28,1],[7342,32,6],[7343,13,1],
[7343,29,1],[7343,43,1],[7344,0,1],[7344,10,1],[7344,57,1],
[7345,22,4],[7345,23,3],[7345,35,2],[7345,43,1],[7346,23,1],
[7346,55,1],[7346,66,1],[7347,28,15],[7348,3,1],[7348,15,1],
[7348,23,1],[7348,35,1],[7348,38,1],[7348,44,2],[7348,66,1],
[7348,68,1],[7348,70,2],[7349,9,2],[7349,59,1],[7350,21,1],
[7350,25,2],[7350,32,3],[7351,33,4],[7352,20,2],[7353,27,2],
[7353,35,1],[7354,70,1],[7354,74,1],[7355,28,2],[7355,33,1],
[7355,38,1],[7355,47,1],[7355,70,1],[7356,9,1],[7356,12,1],
[7356,15,1],[7356,37,1],[7357,27,1],[7357,33,1],[7358,2,4],[7359,0,1],
[7359,3,3],[7359,23,1],[7359,26,1],[7359,27,2],[7359,47,2],
[7359,51,1],[7359,61,1],[7359,62,3],[7359,75,1],[7359,77,1],
[7360,1,1],[7360,2,4],[7361,32,8],[7361,33,1],[7361,70,1],[7362,35,2],
[7363,1,2],[7364,20,1],[7364,21,1],[7364,27,2],[7365,1,3],[7365,2,1],
[7365,10,1],[7365,13,8],[7365,17,1],[7365,20,13],[7365,23,3],

[7365,26,1],[7365,27,1],[7365,34,10],[7365,35,1],[7365,42,2],
[7365,43,1],[7365,45,3],[7365,47,4],[7365,52,1],[7365,53,1],
[7365,55,1],[7365,62,3],[7365,65,1],[7365,78,1],[7366,32,1],
[7366,40,1],[7367,0,1],[7367,1,2],[7367,15,1],[7367,37,1],[7367,39,2],
[7367,55,1],[7367,73,1],[7368,22,1],[7368,47,1],[7369,43,2],
[7370,5,1],[7370,28,1],[7370,30,1],[7371,1,2],[7371,12,1],[7371,13,2],
[7371,26,1],[7371,36,1],[7372,1,68],[7372,3,1],[7372,10,3],
[7372,12,1],[7372,13,1],[7372,15,7],[7372,24,1],[7372,26,1],
[7372,35,1],[7372,36,1],[7372,40,1],[7372,41,1],[7372,44,1],
[7372,50,1],[7372,55,6],[7372,67,1],[7372,68,2],[7372,74,1],
[7372,76,1],[7373,17,2],[7374,23,1],[7374,24,2],[7374,28,1],
[7374,32,1],[7374,59,1],[7375,26,1],[7375,39,2],[7375,62,1],
[7376,3,1],[7376,5,1],[7376,43,2],[7376,47,1],[7376,53,1],[7377,1,2],
[7378,27,1],[7378,39,1],[7379,5,1],[7379,21,1],[7379,32,17],
[7379,35,3],[7379,42,1],[7379,47,1],[7379,48,1],[7380,3,2],[7381,7,5],
[7381,51,1],[7382,31,2],[7383,10,1],[7383,28,1],[7384,32,1],
[7384,63,3],[7384,70,1],[7384,71,1],[7384,74,5],[7384,75,2],
[7384,76,2],[7385,47,3],[7386,21,1],[7386,26,1],[7387,6,2],
[7387,34,1],[7387,39,2],[7387,48,1],[7387,59,2],[7388,2,5],[7389,1,1],
[7389,2,2],[7389,8,1],[7389,9,1],[7389,10,4],[7389,12,3],[7389,14,2],
[7389,16,3],[7389,17,2],[7389,18,3],[7389,20,1],[7389,22,2],
[7389,23,3],[7389,26,1],[7389,28,18],[7389,31,3],[7389,34,1],
[7389,35,1],[7389,36,1],[7389,37,2],[7389,38,1],[7389,40,2],
[7389,41,2],[7389,42,2],[7389,44,2],[7389,45,1],[7389,49,2],
[7389,50,3],[7389,53,3],[7389,54,3],[7389,62,1],[7389,63,20],
[7389,66,2],[7389,68,1],[7389,70,3],[7389,71,1],[7389,74,26],
[7389,75,19],[7389,76,33],[7390,2,1],[7390,26,1],[7390,28,3],
[7391,12,1],[7391,54,1],[7391,65,1],[7391,67,1],[7392,2,1],[7392,3,3],
[7392,5,7],[7392,6,4],[7392,8,2],[7392,10,1],[7392,12,1],[7392,13,1],
[7392,19,1],[7392,20,2],[7392,21,4],[7392,22,5],[7392,23,4],
[7392,24,3],[7392,25,1],[7392,26,3],[7392,27,30],[7392,28,4],
[7392,30,3],[7392,31,2],[7392,32,4],[7392,33,9],[7392,34,4],
[7392,35,10],[7392,36,1],[7392,38,1],[7392,39,4],[7392,42,2],
[7392,43,2],[7392,45,1],[7392,48,2],[7392,65,2],[7392,70,2],
[7393,28,5],[7393,50,1],[7394,1,2],[7394,40,1],[7395,40,4],
[7395,69,1],[7395,70,2],[7396,25,6],[7396,27,3],[7396,35,2],
[7396,62,5],[7397,5,2],[7397,22,1],[7397,26,2],[7397,38,1],
[7398,68,1],[7398,70,1],[7399,20,2],[7400,2,3],[7400,3,1],[7400,23,1],
[7400,25,1],[7400,31,4],[7400,33,1],[7400,42,1],[7400,59,1],
[7401,70,2],[7402,24,1],[7402,35,1],[7402,42,1],[7402,47,1],
[7403,2,1],[7403,3,1],[7403,22,4],[7403,25,1],[7403,27,5],[7403,28,2],
[7403,30,2],[7403,35,5],[7403,52,2],[7403,65,1],[7403,70,2],
[7404,5,1],[7404,15,1],[7404,23,1],[7404,24,2],[7404,27,2],
[7404,32,1],[7404,42,1],[7404,43,1],[7404,56,1],[7404,62,1],
[7404,66,1],[7405,31,3],[7405,35,1],[7405,42,1],[7406,2,26],
[7406,28,1],[7407,21,1],[7407,55,1],[7408,28,17],[7408,35,1],
[7409,2,1],[7409,3,10],[7409,5,2],[7409,6,2],[7409,23,7],[7409,24,2],
[7409,27,5],[7409,28,5],[7409,33,2],[7409,35,1],[7409,40,1],
[7409,44,1],[7409,47,1],[7409,56,3],[7409,72,1],[7410,2,3],[7410,7,1],
[7410,12,2],[7410,15,1],[7410,20,1],[7410,23,2],[7410,26,1],

[7410,28,1],[7410,29,1],[7410,30,2],[7410,35,1],[7410,39,5],
[7410,42,1],[7410,47,2],[7410,56,1],[7411,15,1],[7411,17,1],
[7412,25,2],[7412,32,2],[7413,0,22],[7413,1,309],[7413,2,1],
[7413,3,42],[7413,4,5],[7413,5,8],[7413,6,18],[7413,7,40],[7413,8,35],
[7413,9,259],[7413,10,285],[7413,11,82],[7413,12,148],[7413,13,193],
[7413,14,45],[7413,15,279],[7413,16,130],[7413,17,114],[7413,18,70],
[7413,19,155],[7413,20,248],[7413,21,5],[7413,22,649],[7413,23,5],
[7413,24,13],[7413,25,4],[7413,26,15],[7413,27,11],[7413,28,51],
[7413,29,87],[7413,30,60],[7413,31,6],[7413,32,8],[7413,33,13],
[7413,34,65],[7413,35,11],[7413,36,49],[7413,37,73],[7413,38,866],
[7413,39,64],[7413,40,13],[7413,41,101],[7413,42,4],[7413,43,14],
[7413,44,16],[7413,45,118],[7413,46,86],[7413,47,2],[7413,48,6],
[7413,49,36],[7413,50,11],[7413,51,3],[7413,52,12],[7413,53,119],
[7413,54,49],[7413,55,182],[7413,56,10],[7413,57,67],[7413,58,66],
[7413,59,114],[7413,60,27],[7413,62,119],[7413,63,3],[7413,64,25],
[7413,65,140],[7413,66,19],[7413,67,62],[7413,68,3],[7413,69,2],
[7413,70,8],[7413,71,55],[7413,72,4],[7413,73,47],[7413,74,5],
[7413,75,18],[7413,76,8],[7413,77,126],[7413,78,28],[7414,15,2],
[7415,22,1],[7415,27,2],[7415,28,1],[7416,10,1],[7416,24,1],
[7416,34,1],[7416,43,1],[7417,35,1],[7417,47,1],[7418,0,1],[7418,5,1],
[7418,20,1],[7418,22,2],[7418,28,6],[7418,35,1],[7418,38,1],
[7418,39,1],[7418,47,2],[7419,20,1],[7419,63,1],[7419,70,1],
[7420,22,1],[7420,28,1],[7420,31,1],[7420,35,1],[7421,2,1],
[7421,20,1],[7421,23,1],[7421,50,1],[7422,20,2],[7422,21,1],
[7422,23,1],[7422,25,2],[7422,27,2],[7422,31,1],[7422,33,1],
[7422,35,1],[7423,10,1],[7423,41,1],[7423,55,1],[7424,1,2],[7425,8,2],
[7426,31,3],[7426,35,2],[7426,38,1],[7427,28,1],[7427,70,1],
[7428,1,1],[7428,10,2],[7428,11,1],[7428,13,1],[7428,15,3],
[7428,16,2],[7428,17,1],[7428,18,1],[7428,19,1],[7428,22,1],
[7428,38,1],[7428,39,1],[7428,59,1],[7428,65,2],[7428,67,2],
[7429,28,4],[7430,5,1],[7430,8,1],[7430,20,1],[7430,25,12],
[7430,26,1],[7430,27,1],[7430,28,1],[7430,31,3],[7430,32,9],
[7430,42,24],[7430,43,5],[7430,52,2],[7430,59,1],[7431,22,2],
[7432,28,3],[7433,31,3],[7434,17,3],[7434,53,3],[7435,32,8],
[7435,33,1],[7436,15,1],[7436,31,1],[7436,41,1],[7436,55,3],
[7436,73,1],[7436,75,1],[7436,76,1],[7437,4,1],[7437,32,14],
[7437,33,1],[7437,35,2],[7437,38,2],[7437,50,1],[7437,76,1],
[7438,5,1],[7438,26,1],[7439,3,1],[7439,33,2],[7440,4,1],[7440,38,6],
[7440,44,16],[7440,66,18],[7441,0,1],[7441,12,1],[7442,5,1],
[7442,24,1],[7442,28,1],[7442,35,1],[7442,47,1],[7442,48,1],
[7442,56,3],[7442,68,2],[7442,69,2],[7443,17,2],[7443,53,1],
[7444,21,1],[7444,25,1],[7444,28,1],[7445,28,4],[7445,74,1],
[7446,2,1],[7446,28,1],[7446,33,1],[7446,43,1],[7446,47,1],[7447,1,1],
[7447,3,4],[7447,4,1],[7447,9,1],[7447,11,2],[7447,12,1],[7447,15,5],
[7447,20,8],[7447,21,2],[7447,23,7],[7447,27,1],[7447,33,1],
[7447,42,1],[7447,44,1],[7447,46,1],[7447,48,1],[7447,50,1],
[7447,51,8],[7447,52,3],[7447,53,2],[7447,55,1],[7447,58,1],
[7447,68,2],[7447,70,1],[7447,74,1],[7447,76,1],[7448,27,1],
[7448,28,3],[7448,77,1],[7449,24,1],[7449,33,2],[7450,16,1],
[7450,37,1],[7451,3,1],[7451,24,1],[7451,35,1],[7452,65,1],

[7452,76,1],[7453,48,1],[7453,51,4],[7454,35,1],[7454,42,8],
[7455,4,1],[7455,5,18],[7455,23,1],[7455,28,1],[7455,33,24],
[7455,40,1],[7455,42,1],[7455,48,1],[7455,50,1],[7455,52,1],
[7455,68,1],[7455,75,1],[7456,28,1],[7456,50,1],[7457,28,2],
[7458,2,1],[7458,3,1],[7459,0,40],[7459,1,210],[7459,2,7],[7459,3,6],
[7459,4,12],[7459,5,6],[7459,6,2],[7459,7,58],[7459,8,50],
[7459,9,414],[7459,10,546],[7459,11,181],[7459,12,190],[7459,13,108],
[7459,14,145],[7459,15,143],[7459,16,283],[7459,17,64],[7459,18,140],
[7459,19,513],[7459,20,128],[7459,21,2],[7459,22,5],[7459,23,5],
[7459,24,1],[7459,25,8],[7459,26,9],[7459,27,10],[7459,28,78],
[7459,29,49],[7459,30,35],[7459,31,10],[7459,32,8],[7459,33,6],
[7459,34,101],[7459,35,9],[7459,36,117],[7459,37,163],[7459,38,3],
[7459,39,59],[7459,40,47],[7459,41,277],[7459,42,4],[7459,43,5],
[7459,44,5],[7459,45,308],[7459,46,177],[7459,47,3],[7459,48,2],
[7459,49,129],[7459,50,12],[7459,51,2],[7459,52,1],[7459,53,71],
[7459,54,132],[7459,55,126],[7459,56,6],[7459,57,209],[7459,58,203],
[7459,59,199],[7459,60,50],[7459,62,4],[7459,63,14],[7459,64,59],
[7459,65,454],[7459,66,10],[7459,67,166],[7459,68,34],[7459,69,17],
[7459,70,67],[7459,71,170],[7459,72,27],[7459,73,165],[7459,74,8],
[7459,75,12],[7459,76,24],[7459,77,218],[7459,78,41],[7460,3,1],
[7460,5,1],[7460,42,1],[7460,43,1],[7461,72,2],[7462,23,1],
[7462,28,1],[7463,1,2],[7463,2,1],[7463,3,1],[7463,5,1],[7463,8,1],
[7463,9,1],[7463,12,1],[7463,13,3],[7463,20,3],[7463,22,4],
[7463,23,3],[7463,24,2],[7463,25,4],[7463,26,1],[7463,27,11],
[7463,28,4],[7463,30,1],[7463,31,1],[7463,32,1],[7463,33,2],
[7463,35,3],[7463,42,2],[7463,73,1],[7464,44,4],[7464,66,1],
[7465,2,1],[7465,20,1],[7466,28,2],[7467,33,1],[7467,75,1],
[7468,63,1],[7468,76,1],[7469,8,1],[7469,9,1],[7469,13,1],[7469,15,1],
[7469,20,2],[7469,21,1],[7469,27,1],[7469,28,1],[7469,30,1],
[7469,32,5],[7469,39,1],[7469,42,1],[7469,43,2],[7469,45,1],
[7469,50,1],[7469,56,1],[7469,62,1],[7470,16,1],[7470,57,1],
[7471,5,1],[7471,25,1],[7471,28,1],[7471,69,1],[7471,70,1],
[7471,74,1],[7471,76,1],[7472,5,1],[7472,22,1],[7472,31,1],
[7472,35,1],[7472,38,1],[7472,48,1],[7472,50,1],[7472,56,1],
[7472,63,1],[7472,70,2],[7473,2,2],[7473,28,1],[7474,59,2],[7475,3,1],
[7475,23,2],[7475,24,6],[7475,43,1],[7476,40,3],[7477,32,1],
[7477,71,1],[7478,22,1],[7478,27,1],[7478,31,1],[7478,35,1],
[7478,47,1],[7478,56,1],[7479,32,5],[7480,2,1],[7480,3,1],[7480,4,73],
[7480,5,4],[7480,9,2],[7480,15,124],[7480,16,1],[7480,17,17],
[7480,22,3],[7480,23,2],[7480,28,4],[7480,31,1],[7480,32,1],
[7480,33,1],[7480,38,4],[7480,40,1],[7480,43,1],[7480,44,13],
[7480,50,86],[7480,53,9],[7480,55,76],[7480,66,11],[7480,68,1],
[7480,75,5],[7481,2,5],[7481,3,1],[7481,4,1],[7481,50,1],[7482,23,3],
[7482,24,1],[7482,28,2],[7483,2,2],[7483,20,2],[7483,23,9],
[7483,31,1],[7483,32,1],[7483,39,1],[7483,42,3],[7483,43,1],
[7484,17,1],[7484,53,4],[7484,55,2],[7485,23,1],[7485,43,4],
[7485,59,1],[7486,13,1],[7486,45,2],[7486,65,1],[7486,67,1],
[7487,1,2],[7487,23,1],[7487,24,5],[7487,28,5],[7487,44,1],[7488,2,1],
[7488,28,1],[7488,31,3],[7488,32,2],[7489,22,2],[7489,26,1],
[7489,28,1],[7489,33,1],[7489,42,2],[7489,47,1],[7489,48,2],

[7490,3,1],[7490,32,1],[7491,3,2],[7491,22,2],[7491,27,2],[7491,43,4],
[7491,48,1],[7492,22,1],[7492,24,1],[7492,31,2],[7492,38,1],
[7492,39,1],[7492,47,1],[7493,53,2],[7493,55,1],[7494,28,1],
[7494,43,1],[7495,2,1],[7495,9,1],[7495,21,1],[7495,23,1],[7495,58,1],
[7496,28,1],[7496,38,1],[7496,47,1],[7496,48,1],[7497,0,1],[7497,1,1],
[7497,2,1],[7497,3,1],[7497,5,1],[7497,8,13],[7497,9,2],[7497,10,12],
[7497,12,3],[7497,13,5],[7497,14,1],[7497,15,4],[7497,16,1],
[7497,17,2],[7497,18,1],[7497,19,4],[7497,20,2],[7497,22,1],
[7497,23,2],[7497,25,2],[7497,27,2],[7497,29,1],[7497,30,33],
[7497,31,3],[7497,32,1],[7497,33,12],[7497,34,1],[7497,35,3],
[7497,36,1],[7497,37,4],[7497,38,3],[7497,41,6],[7497,42,1],
[7497,44,1],[7497,45,9],[7497,46,3],[7497,47,1],[7497,48,1],
[7497,49,1],[7497,50,1],[7497,52,5],[7497,53,2],[7497,54,1],
[7497,55,2],[7497,57,1],[7497,58,3],[7497,61,1],[7497,65,3],
[7497,66,2],[7497,71,1],[7497,73,2],[7497,74,2],[7497,76,1],
[7497,77,1],[7497,78,1],[7498,43,1],[7498,45,1],[7499,21,2],
[7499,31,1],[7499,40,1],[7500,2,1],[7500,3,1],[7500,6,5],[7500,8,1],
[7500,22,5],[7500,23,2],[7500,24,1],[7500,26,1],[7500,27,5],
[7500,28,3],[7500,33,1],[7500,35,6],[7500,43,2],[7500,47,1],
[7500,56,1],[7500,75,1],[7501,40,2],[7501,70,7],[7502,39,2],
[7503,2,1],[7503,4,2],[7503,9,2],[7503,10,1],[7503,11,1],[7503,13,1],
[7503,15,3],[7503,17,52],[7503,23,1],[7503,27,1],[7503,28,3],
[7503,33,1],[7503,34,1],[7503,37,1],[7503,39,1],[7503,40,2],
[7503,41,1],[7503,50,4],[7503,53,15],[7503,55,6],[7503,58,1],
[7503,59,1],[7503,71,1],[7503,74,1],[7504,47,1],[7504,56,2],
[7504,59,1],[7504,62,5],[7505,1,1],[7505,2,66],[7505,3,6],[7505,4,1],
[7505,6,2],[7505,8,1],[7505,9,1],[7505,15,1],[7505,17,1],[7505,20,2],
[7505,21,2],[7505,22,4],[7505,23,2],[7505,24,4],[7505,26,5],
[7505,27,8],[7505,28,4],[7505,30,4],[7505,31,23],[7505,32,8],
[7505,34,3],[7505,37,1],[7505,42,7],[7505,43,7],[7505,46,2],
[7505,47,6],[7505,48,7],[7505,50,1],[7505,56,1],[7505,58,1],
[7505,59,1],[7505,60,1],[7505,69,1],[7505,79,1],[7506,28,6],
[7506,63,1],[7507,24,1],[7507,28,2],[7507,33,1],[7507,74,1],
[7508,28,1],[7508,35,1],[7509,17,1],[7509,44,4],[7509,66,4],
[7510,1,1],[7510,3,1],[7510,4,1],[7510,5,2],[7510,9,1],[7510,11,1],
[7510,15,1],[7510,20,2],[7510,22,9],[7510,24,1],[7510,25,11],
[7510,27,8],[7510,28,9],[7510,30,12],[7510,31,1],[7510,32,31],
[7510,33,40],[7510,35,9],[7510,37,3],[7510,38,2],[7510,45,2],
[7510,46,3],[7510,52,40],[7510,53,1],[7510,54,1],[7510,58,1],
[7510,61,1],[7510,66,2],[7510,67,1],[7510,70,2],[7510,72,1],
[7510,74,1],[7510,76,1],[7511,21,1],[7511,23,1],[7511,68,1],
[7512,22,1],[7512,27,1],[7513,24,1],[7513,27,1],[7513,43,1],
[7513,50,1],[7513,77,1],[7514,20,2],[7515,30,2],[7516,22,1],
[7516,23,2],[7516,24,1],[7516,32,1],[7516,42,1],[7517,45,1],
[7517,58,1],[7518,28,2],[7519,20,2],[7520,6,4],[7520,28,3],
[7520,40,1],[7520,43,1],[7520,51,2],[7520,70,2],[7520,74,1],
[7521,28,1],[7521,56,1],[7522,0,1],[7522,1,3],[7522,9,3],[7522,10,2],
[7522,11,2],[7522,12,1],[7522,13,2],[7522,15,2],[7522,16,3],
[7522,17,1],[7522,19,2],[7522,20,3],[7522,34,1],[7522,37,2],
[7522,41,1],[7522,45,2],[7522,46,1],[7522,53,5],[7522,55,1],

[7522,57,1],[7522,58,1],[7522,59,2],[7522,64,3],[7522,65,3],
[7522,71,1],[7522,73,1],[7522,77,2],[7523,28,1],[7523,32,5],
[7524,2,2],[7524,5,5],[7524,21,3],[7524,22,1],[7524,23,7],[7524,24,2],
[7524,25,17],[7524,26,3],[7524,31,4],[7524,32,1],[7524,33,1],
[7524,35,1],[7524,40,1],[7524,43,3],[7524,62,2],[7524,70,1],
[7525,5,1],[7525,19,1],[7525,23,43],[7525,24,1],[7525,28,2],
[7525,42,45],[7525,54,1],[7525,71,1],[7526,63,1],[7526,76,1],
[7527,15,1],[7527,55,6],[7527,62,1],[7528,2,1],[7528,24,1],
[7528,42,1],[7529,28,1],[7529,50,1],[7530,4,1],[7530,24,1],
[7530,31,1],[7530,38,1],[7530,44,2],[7531,16,1],[7531,23,1],
[7531,26,1],[7531,31,1],[7531,32,1],[7531,34,3],[7531,45,1],
[7531,59,1],[7532,3,3],[7532,23,1],[7532,27,2],[7532,47,1],
[7532,77,1],[7533,25,2],[7534,35,2],[7535,9,1],[7535,20,1],
[7536,22,1],[7536,27,2],[7536,28,3],[7536,44,1],[7536,47,1],
[7536,53,1],[7536,55,1],[7536,56,1],[7536,72,1],[7537,2,4],[7537,3,1],
[7537,60,1],[7537,70,1],[7538,27,1],[7538,35,1],[7539,4,2],
[7539,14,1],[7540,10,1],[7540,15,2],[7540,28,1],[7540,30,5],
[7541,20,1],[7541,39,1],[7541,46,1],[7542,6,1],[7542,15,1],
[7543,28,2],[7543,32,1],[7544,25,1],[7544,32,1],[7545,1,1],[7545,3,2],
[7545,6,10],[7545,13,4],[7545,20,1],[7545,23,2],[7545,26,1],
[7545,28,2],[7545,35,2],[7545,42,1],[7545,47,4],[7545,51,1],
[7545,59,2],[7546,3,1],[7546,5,1],[7546,22,3],[7546,23,1],[7546,24,1],
[7546,27,1],[7546,28,1],[7547,5,1],[7547,52,1],[7548,5,1],
[7548,15,10],[7548,17,1],[7548,24,1],[7548,26,1],[7548,41,1],
[7548,53,2],[7548,55,6],[7549,3,1],[7549,5,1],[7550,2,1],[7550,51,1],
[7551,2,2],[7551,3,1],[7551,28,1],[7551,31,1],[7552,2,1],[7552,20,2],
[7552,31,1],[7553,16,1],[7553,20,1],[7554,28,1],[7554,40,1],
[7555,42,1],[7555,70,4],[7556,5,1],[7556,10,1],[7556,13,1],
[7556,16,1],[7556,20,1],[7556,22,1],[7556,23,1],[7556,24,2],
[7556,26,1],[7556,35,1],[7556,42,1],[7556,45,2],[7556,46,1],
[7556,47,1],[7556,60,1],[7556,65,1],[7557,50,2],[7557,66,1],
[7558,1,2],[7558,13,1],[7558,15,1],[7558,27,1],[7558,30,2],
[7558,45,1],[7559,27,6],[7560,23,2],[7560,27,1],[7560,28,4],
[7560,31,3],[7560,34,2],[7560,37,3],[7560,43,5],[7561,32,4],
[7562,23,2],[7563,2,2],[7564,4,1],[7564,6,1],[7564,21,2],[7564,23,1],
[7564,24,1],[7564,26,1],[7564,27,3],[7564,28,1],[7564,32,1],
[7564,35,2],[7564,44,1],[7564,50,1],[7564,68,1],[7564,69,1],
[7564,70,1],[7565,28,1],[7565,50,1],[7566,28,1],[7566,68,1],
[7567,2,6],[7567,21,1],[7567,22,2],[7567,23,6],[7567,24,4],
[7567,26,4],[7567,27,6],[7567,28,2],[7567,30,1],[7567,31,2],
[7567,32,3],[7567,33,1],[7567,35,2],[7567,39,1],[7567,42,2],
[7567,43,3],[7567,48,3],[7567,62,1],[7568,40,1],[7568,51,2],
[7568,66,1],[7569,27,2],[7569,35,1],[7570,25,2],[7570,33,3],
[7570,35,6],[7570,52,1],[7571,2,2],[7571,17,1],[7571,31,1],
[7571,35,1],[7571,44,1],[7572,63,2],[7572,74,2],[7572,75,1],
[7572,76,3],[7573,10,1],[7573,15,1],[7573,78,2],[7574,3,2],
[7574,28,1],[7574,35,2],[7575,2,9],[7575,20,1],[7575,24,1],
[7575,30,1],[7575,31,1],[7575,40,1],[7575,43,2],[7576,3,1],
[7576,28,2],[7577,1,2],[7577,4,1],[7577,15,2],[7577,16,2],[7577,20,3],
[7577,29,1],[7577,38,2],[7577,40,1],[7577,41,1],[7577,44,1],

[7577,46,1],[7577,59,1],[7577,70,1],[7577,79,1],[7578,15,1],
[7578,44,2],[7578,48,1],[7578,55,2],[7578,66,1],[7579,15,5],
[7579,17,1],[7579,30,1],[7579,53,2],[7579,55,3],[7579,60,1],
[7579,77,1],[7580,1,4],[7580,2,13],[7580,3,4],[7580,4,2],[7580,6,2],
[7580,7,1],[7580,8,2],[7580,10,7],[7580,11,1],[7580,12,1],[7580,13,4],
[7580,14,1],[7580,16,2],[7580,17,1],[7580,19,1],[7580,20,3],
[7580,21,2],[7580,22,3],[7580,23,7],[7580,24,4],[7580,25,2],
[7580,26,4],[7580,27,3],[7580,28,6],[7580,30,2],[7580,31,6],
[7580,32,3],[7580,33,3],[7580,35,3],[7580,36,1],[7580,37,1],
[7580,39,4],[7580,40,1],[7580,41,1],[7580,43,3],[7580,45,4],
[7580,46,1],[7580,48,2],[7580,50,1],[7580,53,1],[7580,54,1],
[7580,57,4],[7580,58,2],[7580,59,5],[7580,60,2],[7580,62,2],
[7580,64,2],[7580,66,2],[7580,68,1],[7580,70,1],[7580,77,1],
[7580,78,2],[7581,26,2],[7582,2,1],[7582,3,1],[7582,23,1],
[7582,28,23],[7582,30,15],[7582,33,1],[7582,35,2],[7582,38,1],
[7582,69,1],[7583,6,1],[7583,9,1],[7583,10,1],[7583,17,5],[7583,23,2],
[7583,27,1],[7583,28,2],[7583,47,1],[7583,53,2],[7583,54,1],
[7584,26,1],[7584,71,1],[7585,18,2],[7586,40,1],[7586,43,1],
[7586,51,4],[7586,61,1],[7586,68,1],[7586,74,1],[7587,15,4],
[7587,33,1],[7587,53,2],[7588,0,1],[7588,2,4],[7588,3,14],[7588,4,2],
[7588,5,2],[7588,6,8],[7588,8,1],[7588,10,1],[7588,20,1],[7588,21,1],
[7588,22,21],[7588,23,7],[7588,24,7],[7588,26,8],[7588,27,11],
[7588,28,2],[7588,30,2],[7588,31,4],[7588,32,4],[7588,33,4],
[7588,35,10],[7588,38,3],[7588,39,1],[7588,40,1],[7588,42,5],
[7588,43,5],[7588,47,6],[7588,50,2],[7588,51,3],[7588,52,1],
[7588,54,1],[7588,55,1],[7588,56,7],[7588,59,4],[7588,60,1],
[7588,62,1],[7588,66,1],[7588,68,1],[7588,70,2],[7588,75,1],
[7589,15,2],[7589,17,1],[7590,2,4],[7590,5,2],[7590,20,2],[7590,21,2],
[7590,23,4],[7590,25,2],[7590,27,1],[7590,30,1],[7590,31,1],
[7590,32,1],[7590,47,1],[7590,54,1],[7591,9,1],[7591,34,3],
[7592,27,2],[7592,28,1],[7592,74,1],[7592,76,1],[7593,44,1],
[7593,66,2],[7594,6,2],[7594,24,2],[7594,43,1],[7595,2,1],[7595,3,2],
[7595,23,1],[7595,28,10],[7595,31,4],[7595,33,1],[7595,40,1],
[7595,50,1],[7595,56,2],[7595,76,1],[7596,5,2],[7597,53,2],
[7598,22,1],[7598,23,1],[7598,35,1],[7598,42,1],[7599,22,1],
[7599,35,1],[7600,37,3],[7601,9,2],[7602,24,1],[7602,27,1],[7603,3,1],
[7603,6,2],[7603,23,1],[7603,47,1],[7603,56,3],[7604,2,2],[7604,9,1],
[7604,17,1],[7604,24,2],[7604,27,1],[7604,32,1],[7604,43,1],
[7605,5,1],[7605,25,1],[7605,31,1],[7605,66,1],[7606,20,1],
[7606,62,1],[7607,39,2],[7607,71,1],[7608,15,3],[7608,17,1],
[7608,53,2],[7608,55,5],[7609,5,1],[7609,6,1],[7609,30,1],[7609,70,1],
[7610,0,15],[7610,1,195],[7610,2,1],[7610,5,3],[7610,7,11],
[7610,8,39],[7610,9,22],[7610,10,28],[7610,11,13],[7610,12,88],
[7610,13,59],[7610,14,3],[7610,15,30],[7610,16,27],[7610,17,39],
[7610,18,5],[7610,19,21],[7610,20,55],[7610,21,1],[7610,22,1],
[7610,23,1],[7610,24,6],[7610,25,2],[7610,26,1],[7610,27,2],
[7610,28,3],[7610,29,63],[7610,30,47],[7610,31,4],[7610,32,4],
[7610,33,9],[7610,34,47],[7610,36,29],[7610,37,17],[7610,39,63],
[7610,41,14],[7610,42,2],[7610,43,2],[7610,45,19],[7610,46,11],
[7610,49,3],[7610,50,1],[7610,51,2],[7610,53,35],[7610,54,12],

[7610,55,26],[7610,56,1],[7610,57,15],[7610,58,10],[7610,59,22],
[7610,60,5],[7610,62,2],[7610,63,1],[7610,64,3],[7610,65,24],
[7610,67,13],[7610,70,4],[7610,71,16],[7610,73,25],[7610,77,9],
[7610,78,3],[7610,79,2],[7611,12,1],[7611,21,1],[7611,23,1],
[7611,26,1],[7611,42,2],[7611,50,1],[7611,56,6],[7612,28,118],
[7612,63,1],[7612,74,2],[7612,75,2],[7612,76,2],[7613,5,1],
[7613,22,1],[7613,24,1],[7613,27,2],[7613,35,1],[7613,42,1],
[7613,62,1],[7614,27,10],[7614,28,1],[7614,66,1],[7615,8,1],
[7615,9,5],[7615,10,1],[7615,14,1],[7615,18,1],[7615,37,2],
[7615,41,2],[7615,45,1],[7616,2,2],[7616,7,1],[7616,9,1],[7616,19,1],
[7616,20,2],[7616,21,1],[7616,25,1],[7616,45,1],[7616,53,1],
[7617,2,2],[7617,6,1],[7617,31,2],[7617,38,1],[7617,47,1],[7617,66,1],
[7617,74,1],[7618,15,6],[7618,53,1],[7618,55,6],[7619,2,2],
[7619,28,4],[7619,32,1],[7620,33,1],[7620,47,2],[7621,2,2],
[7622,66,1],[7622,70,1],[7623,40,1],[7623,61,1],[7623,70,1],
[7624,28,1],[7624,35,1],[7624,52,1],[7624,66,1],[7624,74,1],
[7625,40,3],[7625,44,1],[7625,50,1],[7625,61,1],[7625,62,2],
[7625,65,1],[7626,9,1],[7626,28,1],[7626,59,1],[7627,31,1],
[7627,32,12],[7627,35,2],[7627,43,1],[7627,56,1],[7628,5,1],
[7628,23,1],[7628,25,1],[7628,32,4],[7629,2,4],[7629,46,1],[7630,0,2],
[7630,1,6],[7630,3,1],[7630,4,33],[7630,9,8],[7630,10,3],[7630,11,2],
[7630,12,5],[7630,13,2],[7630,14,2],[7630,15,269],[7630,16,2],
[7630,17,593],[7630,18,2],[7630,19,6],[7630,20,5],[7630,21,2],
[7630,22,1],[7630,23,1],[7630,26,1],[7630,27,3],[7630,28,4],
[7630,29,1],[7630,30,2],[7630,32,3],[7630,33,1],[7630,34,1],
[7630,37,1],[7630,38,4],[7630,39,2],[7630,41,1],[7630,42,1],
[7630,44,6],[7630,45,2],[7630,47,1],[7630,49,1],[7630,50,48],
[7630,53,605],[7630,54,1],[7630,55,455],[7630,57,1],[7630,58,1],
[7630,62,1],[7630,64,1],[7630,65,2],[7630,66,9],[7630,67,1],
[7630,70,1],[7630,71,1],[7630,73,1],[7630,76,2],[7630,78,1],
[7630,79,1],[7631,25,31],[7632,27,1],[7632,28,1],[7632,43,1],
[7633,5,1],[7633,35,2],[7634,15,1],[7634,16,1],[7635,11,1],
[7635,46,1],[7636,0,4],[7636,1,6],[7636,10,2],[7636,11,1],[7636,13,1],
[7636,15,2],[7636,16,1],[7636,19,2],[7636,20,2],[7636,25,3],
[7636,30,279],[7636,31,1],[7636,32,14],[7636,33,1],[7636,34,1],
[7636,35,5],[7636,37,1],[7636,39,1],[7636,44,1],[7636,45,3],
[7636,46,1],[7636,52,10],[7636,55,1],[7636,57,1],[7636,60,1],
[7636,65,3],[7636,67,2],[7636,73,2],[7636,77,3],[7637,28,1],
[7637,69,1],[7638,23,1],[7638,26,1],[7638,31,1],[7638,33,1],
[7638,62,1],[7639,28,1],[7639,48,3],[7640,13,1],[7640,20,1],
[7641,31,1],[7641,53,1],[7642,1,1],[7642,15,1],[7642,20,3],
[7642,22,2],[7642,23,1],[7642,24,3],[7642,26,1],[7642,27,2],
[7642,32,1],[7642,33,1],[7642,35,1],[7642,42,1],[7642,43,2],
[7642,50,1],[7642,59,3],[7642,63,1],[7643,29,2],[7644,0,6],
[7644,1,11],[7644,5,1],[7644,8,5],[7644,10,3],[7644,11,1],[7644,12,3],
[7644,13,3],[7644,15,12],[7644,16,1],[7644,17,50],[7644,20,6],
[7644,22,1],[7644,29,11],[7644,30,3],[7644,32,1],[7644,34,4],
[7644,35,1],[7644,36,2],[7644,37,3],[7644,39,7],[7644,42,1],
[7644,43,1],[7644,44,5],[7644,45,1],[7644,53,54],[7644,55,21],
[7644,59,2],[7644,64,1],[7644,65,2],[7644,66,3],[7644,75,1],

[7644,76,2],[7645,25,1],[7645,27,1],[7645,28,1],[7645,32,3],
[7646,2,1],[7646,3,2],[7646,8,1],[7646,30,1],[7646,47,1],[7647,8,1],
[7647,28,1],[7647,31,8],[7647,32,1],[7648,2,1],[7648,8,2],[7648,19,1],
[7648,22,1],[7648,26,1],[7648,27,1],[7648,33,9],[7648,42,2],
[7648,68,1],[7648,76,1],[7649,28,7],[7649,70,1],[7650,39,2],
[7651,2,1],[7651,5,1],[7652,1,3],[7652,12,1],[7652,13,2],[7652,15,1],
[7652,29,2],[7652,30,1],[7652,36,32],[7652,53,2],[7652,65,1],
[7652,73,1],[7653,23,1],[7653,26,1],[7654,59,2],[7655,28,5],
[7656,27,2],[7656,62,1],[7657,13,1],[7657,21,3],[7657,25,2],
[7657,26,1],[7657,28,1],[7657,30,2],[7657,31,9],[7657,74,1],
[7658,16,1],[7658,53,1],[7659,27,1],[7659,33,1],[7660,15,1],
[7660,21,1],[7660,31,8],[7660,46,1],[7660,55,1],[7660,68,1],
[7661,9,1],[7661,19,1],[7661,41,1],[7661,59,1],[7661,65,1],
[7662,35,1],[7662,47,1],[7662,60,1],[7663,0,1],[7663,1,9],[7663,9,6],
[7663,10,5],[7663,11,2],[7663,12,10],[7663,13,3],[7663,14,1],
[7663,16,3],[7663,17,1],[7663,18,3],[7663,19,7],[7663,20,2],
[7663,24,3],[7663,28,1],[7663,29,3],[7663,30,3],[7663,31,1],
[7663,34,3],[7663,35,1],[7663,36,1],[7663,37,1],[7663,39,7],
[7663,41,8],[7663,43,1],[7663,45,8],[7663,46,2],[7663,49,1],
[7663,53,6],[7663,54,3],[7663,55,5],[7663,57,6],[7663,58,1],
[7663,59,1],[7663,62,1],[7663,64,1],[7663,65,6],[7663,67,1],
[7663,71,5],[7663,73,4],[7663,78,1],[7664,40,1],[7664,70,2],
[7665,12,1],[7665,33,1],[7666,2,4],[7666,31,2],[7666,53,1],
[7666,56,1],[7667,25,1],[7667,33,1],[7667,37,2],[7667,76,1],
[7668,23,10],[7668,27,1],[7668,42,5],[7669,9,1],[7669,15,1],
[7670,50,2],[7671,34,1],[7671,71,1],[7672,2,2],[7672,6,1],[7672,20,1],
[7672,31,4],[7672,32,2],[7673,21,2],[7673,22,1],[7673,23,1],
[7673,27,4],[7673,28,1],[7673,56,1],[7674,29,1],[7674,39,2],
[7675,2,3],[7676,21,1],[7676,42,1],[7677,2,1],[7677,24,1],[7677,26,1],
[7677,28,4],[7677,44,1],[7678,2,1],[7678,23,1],[7678,27,1],
[7678,62,1],[7679,36,1],[7679,50,1],[7679,55,1],[7679,61,2],
[7680,21,1],[7680,24,1],[7680,26,8],[7680,32,2],[7680,33,2],
[7680,35,1],[7680,47,1],[7680,70,1],[7681,2,1],[7681,3,1],[7681,23,1],
[7681,27,1],[7682,2,6],[7682,38,1],[7683,15,1],[7683,55,2],
[7684,28,2],[7685,4,1],[7685,9,2],[7685,17,2],[7685,28,2],[7685,50,1],
[7685,53,1],[7686,0,1],[7686,7,2],[7686,8,1],[7686,9,1],[7686,10,3],
[7686,11,2],[7686,12,4],[7686,14,1],[7686,15,32],[7686,16,4],
[7686,19,8],[7686,20,1],[7686,29,1],[7686,32,2],[7686,37,2],
[7686,41,4],[7686,42,1],[7686,44,2],[7686,45,1],[7686,46,2],
[7686,49,3],[7686,53,5],[7686,54,1],[7686,55,51],[7686,57,2],
[7686,59,2],[7686,60,2],[7686,64,1],[7686,65,3],[7686,66,2],
[7686,67,3],[7686,71,2],[7686,77,2],[7686,78,1],[7687,1,8],
[7687,2,11],[7687,3,5],[7687,4,4],[7687,5,46],[7687,6,11],[7687,7,2],
[7687,9,1],[7687,10,2],[7687,12,3],[7687,13,1],[7687,15,3],
[7687,16,1],[7687,18,1],[7687,19,1],[7687,20,2],[7687,21,15],
[7687,22,5],[7687,23,14],[7687,24,12],[7687,25,18],[7687,26,12],
[7687,27,24],[7687,28,19],[7687,29,4],[7687,30,2],[7687,31,9],
[7687,32,15],[7687,33,15],[7687,34,2],[7687,35,27],[7687,36,2],
[7687,37,3],[7687,38,7],[7687,39,1],[7687,40,3],[7687,42,14],
[7687,43,5],[7687,44,2],[7687,45,2],[7687,46,3],[7687,47,16],

[7687,48,4],[7687,50,7],[7687,51,8],[7687,52,2],[7687,54,1],
[7687,55,4],[7687,56,5],[7687,61,2],[7687,62,7],[7687,63,3],
[7687,65,1],[7687,66,10],[7687,68,5],[7687,69,10],[7687,70,15],
[7687,72,4],[7687,74,8],[7687,75,4],[7687,76,4],[7688,26,1],
[7688,40,1],[7689,2,1],[7689,5,1],[7690,1,2],[7690,4,1],[7690,5,3],
[7690,12,1],[7690,13,14],[7690,21,1],[7690,30,1],[7690,33,1],
[7690,34,1],[7691,21,1],[7691,28,1],[7692,8,1],[7692,20,1],[7693,1,1],
[7693,10,1],[7693,13,1],[7693,24,1],[7693,25,1],[7693,27,1],
[7693,43,1],[7693,45,1],[7693,59,1],[7693,65,1],[7694,26,1],
[7694,33,1],[7695,10,1],[7695,37,1],[7696,16,1],[7696,65,1],
[7696,78,1],[7697,4,1],[7697,15,1],[7697,17,2],[7697,25,1],
[7697,28,1],[7697,31,1],[7697,38,1],[7697,43,1],[7697,50,6],
[7697,53,1],[7697,55,2],[7698,10,1],[7698,35,1],[7699,3,1],
[7699,31,1],[7700,10,2],[7700,16,1],[7700,19,1],[7700,37,1],
[7700,41,2],[7700,46,1],[7700,49,1],[7700,59,2],[7700,65,1],
[7700,77,1],[7701,0,1],[7701,1,3],[7701,4,1],[7701,7,1],[7701,9,1],
[7701,10,2],[7701,11,1],[7701,12,1],[7701,13,2],[7701,14,2],
[7701,15,6],[7701,16,1],[7701,17,5],[7701,29,1],[7701,36,1],
[7701,39,3],[7701,41,1],[7701,45,1],[7701,46,1],[7701,49,1],
[7701,53,1],[7701,55,4],[7701,57,1],[7701,59,2],[7701,60,1],
[7701,63,1],[7701,65,1],[7701,71,1],[7701,77,2],[7701,78,1],
[7702,21,1],[7702,23,1],[7702,62,2],[7703,1,1],[7703,20,1],
[7703,34,1],[7703,41,1],[7703,60,1],[7703,78,1],[7704,20,1],
[7704,76,1],[7705,32,13],[7706,2,2],[7706,21,1],[7706,31,1],
[7706,42,1],[7707,6,1],[7707,8,1],[7707,11,1],[7707,19,1],[7707,20,3],
[7707,22,2],[7707,50,2],[7707,65,1],[7707,70,1],[7708,1,1],
[7708,22,1],[7708,29,1],[7708,33,4],[7708,47,1],[7708,72,1],
[7709,16,1],[7709,17,1],[7709,29,1],[7710,30,1],[7710,33,2],
[7711,8,2],[7711,22,6],[7711,24,1],[7711,26,1],[7711,31,1],
[7711,35,1],[7711,42,1],[7711,52,1],[7711,62,1],[7711,76,1],
[7712,20,3],[7712,41,3],[7713,1,1],[7713,13,1],[7714,5,1],[7714,23,1],
[7714,33,1],[7714,51,1],[7714,63,17],[7714,68,1],[7714,70,1],
[7714,74,18],[7714,75,18],[7714,76,27],[7715,1,1],[7715,4,1],
[7715,16,1],[7715,20,1],[7715,22,1],[7715,24,1],[7715,26,1],
[7715,41,1],[7715,55,1],[7715,56,1],[7715,57,2],[7715,77,1],
[7716,14,1],[7716,22,1],[7716,24,2],[7716,27,2],[7716,45,1],
[7716,65,1],[7717,74,1],[7717,75,1],[7718,28,1],[7718,32,14],
[7718,33,1],[7719,24,2],[7720,63,8],[7720,74,9],[7720,75,8],
[7720,76,4],[7721,17,1],[7721,21,1],[7722,9,1],[7722,20,1],
[7722,23,1],[7722,25,2],[7722,28,1],[7722,42,1],[7723,8,1],
[7723,20,1],[7723,24,1],[7723,26,1],[7723,27,2],[7723,28,6],
[7723,31,1],[7723,45,1],[7724,22,2],[7725,2,1],[7725,8,1],[7725,21,1],
[7725,34,1],[7725,42,1],[7726,13,1],[7726,29,1],[7726,71,1],
[7726,73,1],[7727,24,1],[7727,33,1],[7727,56,1],[7728,1,1],[7728,2,3],
[7728,3,1],[7728,4,56],[7728,5,2],[7728,7,2],[7728,8,3],[7728,9,13],
[7728,10,12],[7728,11,3],[7728,12,1],[7728,13,4],[7728,14,4],
[7728,15,15],[7728,16,5],[7728,17,54],[7728,18,3],[7728,19,8],
[7728,20,3],[7728,23,2],[7728,24,1],[7728,25,4],[7728,26,1],
[7728,27,4],[7728,28,17],[7728,30,2],[7728,31,4],[7728,33,2],
[7728,34,2],[7728,35,2],[7728,36,1],[7728,37,2],[7728,38,6],

[7728,39,3],[7728,40,3],[7728,41,4],[7728,44,49],[7728,45,8],
[7728,46,3],[7728,47,1],[7728,48,1],[7728,49,2],[7728,50,48],
[7728,53,58],[7728,54,4],[7728,55,23],[7728,56,2],[7728,57,5],
[7728,58,6],[7728,59,3],[7728,60,2],[7728,63,4],[7728,64,1],
[7728,65,8],[7728,66,66],[7728,67,6],[7728,69,1],[7728,70,7],
[7728,71,3],[7728,72,2],[7728,73,1],[7728,74,3],[7728,75,4],
[7728,76,4],[7728,77,6],[7728,78,2],[7728,79,1],[7729,1,4],
[7729,19,1],[7730,20,3],[7731,3,1],[7731,5,2],[7731,22,1],[7731,23,1],
[7731,24,1],[7731,26,1],[7731,27,1],[7731,32,2],[7731,33,1],
[7731,35,1],[7731,47,5],[7731,50,1],[7731,51,1],[7731,56,1],
[7731,68,1],[7732,2,2],[7732,3,13],[7732,5,2],[7732,6,12],[7732,11,1],
[7732,13,2],[7732,15,1],[7732,20,4],[7732,21,4],[7732,22,5],
[7732,23,16],[7732,24,24],[7732,25,4],[7732,26,5],[7732,27,11],
[7732,28,4],[7732,29,2],[7732,30,2],[7732,31,1],[7732,32,2],
[7732,33,16],[7732,34,3],[7732,35,14],[7732,37,5],[7732,38,1],
[7732,39,3],[7732,40,4],[7732,46,3],[7732,47,4],[7732,48,7],
[7732,50,1],[7732,52,1],[7732,62,5],[7732,65,1],[7732,66,1],
[7732,68,1],[7732,70,2],[7732,76,2],[7732,77,1],[7733,17,1],
[7733,19,1],[7733,20,1],[7733,23,1],[7733,34,1],[7733,42,1],
[7733,56,1],[7734,23,1],[7734,26,1],[7734,56,1],[7734,63,15],
[7734,74,6],[7734,75,11],[7734,76,20],[7735,25,1],[7735,70,1],
[7736,44,1],[7736,66,1],[7737,27,2],[7737,28,1],[7737,31,1],
[7737,62,1],[7738,27,3],[7738,28,1],[7738,31,1],[7739,2,1],[7739,5,1],
[7740,19,1],[7740,74,1],[7740,76,3],[7741,17,1],[7741,38,2],
[7741,55,1],[7742,24,1],[7742,31,2],[7742,33,1],[7742,35,1],
[7742,42,3],[7743,8,2],[7743,13,2],[7743,20,1],[7743,35,2],
[7744,21,1],[7744,59,1],[7745,32,2],[7745,40,1],[7746,23,1],
[7746,27,3],[7747,3,2],[7748,2,3],[7748,31,6],[7748,70,1],[7749,2,1],
[7749,5,1],[7749,8,3],[7749,11,2],[7749,12,1],[7749,17,1],[7749,19,1],
[7749,20,17],[7749,21,1],[7749,22,3],[7749,23,17],[7749,24,5],
[7749,26,1],[7749,27,28],[7749,28,1],[7749,30,1],[7749,31,2],
[7749,32,4],[7749,33,3],[7749,34,1],[7749,35,3],[7749,38,1],
[7749,39,2],[7749,42,2],[7749,44,1],[7749,47,1],[7749,48,2],
[7749,53,1],[7749,59,1],[7749,62,2],[7749,68,1],[7749,69,1],
[7749,70,1],[7749,74,1],[7749,76,1],[7750,3,1],[7750,6,1],[7750,8,1],
[7750,18,1],[7750,22,1],[7750,23,1],[7750,25,1],[7750,40,1],
[7750,61,1],[7750,63,19],[7750,74,17],[7750,75,8],[7750,76,18],
[7751,1,1],[7751,13,1],[7751,29,1],[7751,33,13],[7751,43,1],
[7752,20,2],[7752,27,4],[7752,35,2],[7753,1,182],[7753,2,2],
[7753,4,2],[7753,5,1],[7753,7,11],[7753,8,2],[7753,9,12],[7753,10,4],
[7753,11,3],[7753,12,136],[7753,13,108],[7753,15,5],[7753,16,3],
[7753,17,3],[7753,18,1],[7753,19,4],[7753,20,17],[7753,21,1],
[7753,22,1],[7753,23,22],[7753,24,1],[7753,25,575],[7753,26,29],
[7753,27,1],[7753,28,21],[7753,29,127],[7753,30,7],[7753,31,45],
[7753,32,2],[7753,33,253],[7753,34,1],[7753,36,1],[7753,37,1],
[7753,38,2],[7753,39,3],[7753,41,3],[7753,42,28],[7753,43,11],
[7753,44,1],[7753,45,4],[7753,46,2],[7753,48,1],[7753,49,2],
[7753,53,1],[7753,54,1],[7753,55,1],[7753,57,5],[7753,58,4],
[7753,59,2],[7753,60,1],[7753,62,18],[7753,63,1],[7753,65,5],
[7753,66,2],[7753,67,8],[7753,70,1],[7753,71,2],[7753,73,31],

[7753,77,4],[7753,78,3],[7754,0,31],[7754,1,3],[7754,7,34],[7754,9,1],
[7754,10,1],[7754,11,1],[7754,12,1],[7754,13,1],[7754,15,1],
[7754,16,2],[7754,18,4],[7754,34,1],[7754,37,1],[7754,41,1],
[7754,45,1],[7754,46,2],[7754,49,1],[7754,59,1],[7754,65,1],
[7754,67,2],[7754,73,5],[7754,77,8],[7755,32,8],[7756,22,1],
[7756,25,1],[7757,5,2],[7757,13,1],[7757,15,1],[7757,17,1],
[7757,21,1],[7757,22,2],[7757,23,3],[7757,24,1],[7757,26,1],
[7757,32,2],[7757,33,1],[7757,35,2],[7757,43,1],[7757,50,1],
[7757,69,1],[7758,3,3],[7758,24,1],[7758,26,1],[7758,27,1],
[7758,28,3],[7758,31,1],[7758,32,3],[7758,35,2],[7758,56,3],
[7759,2,4],[7759,5,3],[7759,6,1],[7759,10,2],[7759,15,1],[7759,22,1],
[7759,23,2],[7759,24,1],[7759,26,2],[7759,27,1],[7759,28,1],
[7759,31,2],[7759,32,1],[7759,35,3],[7759,56,1],[7759,66,1],
[7759,69,1],[7760,1,1],[7760,15,1],[7760,20,1],[7760,21,1],
[7760,22,1],[7760,30,3],[7760,31,1],[7760,32,1],[7760,34,1],
[7760,37,1],[7760,39,2],[7760,42,1],[7760,64,1],[7761,8,1],
[7761,45,1],[7762,0,2],[7762,1,1],[7762,13,1],[7762,14,1],[7762,28,1],
[7762,29,41],[7762,30,1],[7762,41,1],[7762,55,1],[7762,71,1],
[7763,9,1],[7763,18,1],[7764,22,2],[7765,40,1],[7765,47,1],
[7765,56,4],[7766,1,1],[7766,28,2],[7767,31,2],[7768,3,1],[7768,27,1],
[7768,42,1],[7769,6,1],[7769,40,2],[7769,50,1],[7769,62,1],
[7769,68,2],[7769,70,8],[7770,68,2],[7771,1,1],[7771,39,1],[7772,0,1],
[7772,9,1],[7772,30,4],[7773,1,1],[7773,4,1],[7773,15,1],[7773,17,1],
[7773,24,1],[7773,25,1],[7773,35,1],[7773,37,1],[7773,46,1],
[7773,50,2],[7773,53,1],[7773,55,1],[7773,70,1],[7774,23,15],
[7774,25,25],[7774,26,2],[7774,27,1],[7774,28,1],[7774,31,2],
[7774,32,2],[7774,35,1],[7774,42,10],[7774,43,2],[7774,78,1],
[7775,25,1],[7775,44,2],[7776,28,2],[7777,24,1],[7777,55,2],
[7778,0,1],[7778,2,5],[7778,3,1],[7778,5,1],[7778,7,1],[7778,13,5],
[7778,20,6],[7778,21,1],[7778,22,2],[7778,23,2],[7778,25,1],
[7778,26,1],[7778,27,1],[7778,28,4],[7778,30,1],[7778,33,1],
[7778,38,3],[7778,43,1],[7778,45,2],[7778,52,1],[7778,63,1],
[7778,65,1],[7778,71,1],[7779,1,7],[7779,16,1],[7779,25,1],
[7779,31,1],[7779,56,1],[7779,64,1],[7779,77,1],[7780,2,1],
[7780,47,2],[7781,1,8],[7781,13,1],[7781,17,2],[7781,29,6],
[7781,39,4],[7781,58,1],[7782,22,1],[7782,27,2],[7782,35,1],
[7782,51,1],[7782,62,1],[7783,24,1],[7783,27,2],[7784,62,22],
[7784,70,1],[7785,3,1],[7785,9,1],[7785,17,1],[7785,24,1],[7785,25,1],
[7785,27,1],[7785,30,1],[7785,31,4],[7785,46,1],[7785,64,1],
[7786,28,2],[7787,3,1],[7787,4,1],[7787,15,1],[7787,23,1],[7787,49,1],
[7788,25,2],[7789,3,2],[7789,5,3],[7789,6,1],[7789,22,5],[7789,23,5],
[7789,24,1],[7789,25,2],[7789,26,1],[7789,27,6],[7789,28,1],
[7789,31,2],[7789,32,1],[7789,35,5],[7789,42,2],[7789,43,1],
[7789,56,1],[7790,6,1],[7790,21,1],[7791,4,7],[7791,5,1],[7791,21,1],
[7791,23,1],[7791,28,2],[7791,31,2],[7791,33,1],[7791,35,1],
[7791,38,3],[7791,44,21],[7791,48,2],[7791,50,11],[7791,56,2],
[7791,66,21],[7792,5,2],[7793,2,3],[7794,39,1],[7794,42,4],
[7795,22,1],[7795,56,1],[7796,1,1],[7796,3,1],[7796,5,1],[7796,11,1],
[7796,12,1],[7796,13,1],[7796,15,1],[7796,20,2],[7796,22,1],
[7796,23,2],[7796,26,2],[7796,29,1],[7796,30,1],[7796,31,1],

[7796,41,1],[7796,42,7],[7796,43,4],[7796,51,2],[7796,60,1],
[7796,63,1],[7796,68,2],[7796,69,3],[7796,70,14],[7796,72,4],
[7797,6,1],[7797,23,1],[7798,21,5],[7798,24,1],[7798,42,1],
[7799,44,6],[7799,50,4],[7799,53,1],[7799,55,1],[7799,66,8],
[7800,23,3],[7801,21,1],[7801,74,5],[7802,4,8],[7802,5,1],[7802,8,1],
[7802,10,1],[7802,15,13],[7802,17,38],[7802,20,1],[7802,22,1],
[7802,26,1],[7802,37,1],[7802,38,2],[7802,40,1],[7802,44,3],
[7802,45,2],[7802,47,1],[7802,50,16],[7802,53,32],[7802,55,31],
[7802,58,1],[7802,65,1],[7802,66,5],[7802,67,1],[7802,73,1],
[7802,76,1],[7803,5,1],[7803,22,2],[7803,24,3],[7803,25,1],
[7803,27,4],[7803,31,1],[7803,35,1],[7803,42,1],[7803,43,3],
[7804,5,1],[7804,43,1],[7805,20,3],[7806,27,1],[7806,38,1],[7807,4,3],
[7807,15,1],[7807,17,8],[7807,48,1],[7807,50,2],[7807,53,5],
[7807,55,1],[7807,74,1],[7808,31,10],[7809,13,2],[7809,23,1],
[7809,53,1],[7810,2,2],[7810,3,1],[7810,23,2],[7810,26,4],[7810,31,1],
[7810,35,1],[7810,42,4],[7810,43,6],[7810,62,5],[7811,3,1],
[7811,22,5],[7811,27,1],[7811,35,1],[7812,1,3],[7812,29,1],[7813,1,1],
[7813,59,1],[7814,10,1],[7814,11,1],[7814,15,1],[7814,16,2],
[7814,18,3],[7814,19,3],[7814,20,1],[7814,37,3],[7814,39,1],
[7814,45,1],[7814,57,2],[7814,59,2],[7814,64,1],[7814,65,2],
[7814,71,1],[7814,73,1],[7815,23,1],[7815,24,1],[7816,1,8],[7816,3,1],
[7816,4,1],[7816,5,3],[7816,9,1],[7816,13,5],[7816,15,3],[7816,20,1],
[7816,21,2],[7816,22,4],[7816,24,8],[7816,27,1],[7816,28,3],
[7816,31,2],[7816,32,4],[7816,33,4],[7816,35,1],[7816,38,1],
[7816,39,1],[7816,40,2],[7816,42,4],[7816,43,3],[7816,44,1],
[7816,45,1],[7816,46,1],[7816,47,3],[7816,50,4],[7816,51,19],
[7816,56,19],[7816,61,1],[7816,64,1],[7816,65,1],[7816,66,1],
[7816,68,6],[7816,69,4],[7816,70,6],[7816,72,1],[7816,74,3],
[7816,75,2],[7816,77,7],[7817,24,1],[7817,28,2],[7818,28,1],
[7818,44,2],[7818,66,1],[7819,17,1],[7819,53,2],[7820,22,1],
[7820,28,1],[7820,56,1],[7821,1,2],[7821,4,2],[7821,5,1],[7821,6,2],
[7821,9,2],[7821,11,1],[7821,14,1],[7821,18,1],[7821,20,2],
[7821,23,2],[7821,27,4],[7821,31,1],[7821,44,1],[7821,49,1],
[7821,53,1],[7821,56,1],[7821,59,1],[7821,65,1],[7821,66,1],
[7821,78,1],[7822,22,4],[7822,27,4],[7822,28,1],[7823,8,1],
[7823,20,2],[7823,22,2],[7823,30,1],[7823,35,1],[7823,47,2],
[7823,59,1],[7823,61,1],[7823,63,1],[7823,68,1],[7824,20,1],
[7824,35,1],[7824,48,3],[7825,22,1],[7825,23,1],[7825,27,1],
[7825,28,1],[7825,32,2],[7825,33,1],[7825,35,1],[7825,62,4],
[7826,8,2],[7827,22,2],[7827,23,1],[7827,24,2],[7827,26,1],
[7827,27,3],[7827,28,1],[7827,35,1],[7827,62,1],[7828,21,4],
[7828,23,8],[7828,25,18],[7828,26,5],[7828,33,19],[7828,39,1],
[7828,43,3],[7828,56,2],[7829,9,1],[7829,59,1],[7830,15,2],
[7831,24,1],[7831,32,2],[7832,12,1],[7832,66,1],[7833,6,1],
[7833,28,1],[7834,23,2],[7834,33,1],[7834,47,3],[7834,48,1],
[7835,37,2],[7835,56,1],[7836,5,36],[7836,21,7],[7836,22,13],
[7836,23,14],[7836,26,19],[7836,27,40],[7836,31,3],[7836,32,14],
[7836,33,1],[7836,35,5],[7836,39,4],[7836,40,1],[7836,42,16],
[7836,43,12],[7836,44,2],[7836,56,1],[7836,59,1],[7836,62,1],
[7836,68,2],[7836,72,1],[7836,74,1],[7837,51,2],[7838,68,1],

[7838,69,1],[7839,5,1],[7839,26,1],[7840,25,1],[7840,28,1],[7841,0,1],
[7841,1,8],[7841,2,4],[7841,3,38],[7841,4,4],[7841,6,73],[7841,7,2],
[7841,8,5],[7841,12,3],[7841,13,5],[7841,17,2],[7841,20,7],
[7841,21,1],[7841,22,2],[7841,23,1],[7841,24,3],[7841,25,2],
[7841,26,6],[7841,27,7],[7841,28,6],[7841,29,2],[7841,30,12],
[7841,31,2],[7841,32,2],[7841,35,4],[7841,36,4],[7841,37,81],
[7841,38,1],[7841,39,3],[7841,40,1],[7841,42,1],[7841,43,4],
[7841,47,2],[7841,48,3],[7841,50,4],[7841,51,6],[7841,55,1],
[7841,60,7],[7841,64,1],[7841,65,1],[7841,67,1],[7841,68,2],
[7841,69,1],[7841,70,2],[7841,79,2],[7842,9,1],[7842,18,1],
[7842,21,1],[7842,24,2],[7842,25,1],[7842,28,4],[7842,31,1],
[7842,32,1],[7842,53,1],[7842,59,1],[7842,67,1],[7843,15,1],
[7843,55,1],[7844,28,2],[7844,32,1],[7845,22,1],[7845,35,1],
[7846,8,2],[7846,35,1],[7846,40,1],[7846,77,1],[7847,69,1],
[7847,70,1],[7848,4,2],[7848,19,1],[7848,41,1],[7848,50,4],
[7849,28,5],[7850,27,80],[7850,28,3],[7851,3,2],[7851,5,3],
[7851,21,1],[7851,25,5],[7851,27,25],[7851,28,5],[7851,32,1],
[7851,38,142],[7851,40,2],[7851,43,1],[7851,59,1],[7851,70,1],
[7851,72,2],[7851,74,3],[7852,27,2],[7853,24,1],[7853,25,1],
[7853,57,1],[7854,8,2],[7854,16,1],[7854,50,1],[7854,53,5],[7855,3,1],
[7855,21,8],[7855,27,32],[7855,28,1],[7856,1,1],[7856,5,2],
[7856,15,15],[7856,34,1],[7856,37,1],[7856,45,1],[7856,55,8],
[7856,57,3],[7856,66,1],[7856,71,1],[7857,2,4],[7857,5,1],[7857,21,3],
[7857,22,2],[7857,23,1],[7857,25,1],[7857,27,1],[7857,28,3],
[7857,30,2],[7857,38,2],[7857,43,1],[7857,48,1],[7857,57,1],
[7857,77,1],[7858,28,3],[7859,1,1],[7859,3,3],[7859,5,2],[7859,6,4],
[7859,17,1],[7859,21,1],[7859,22,6],[7859,23,6],[7859,24,8],
[7859,25,1],[7859,26,1],[7859,27,6],[7859,28,4],[7859,29,1],
[7859,32,4],[7859,33,4],[7859,35,5],[7859,38,1],[7859,42,2],
[7859,43,5],[7859,47,1],[7859,48,1],[7859,50,5],[7859,51,1],
[7859,56,7],[7859,62,3],[7859,63,1],[7859,70,1],[7860,0,1],[7860,4,1],
[7860,15,1],[7860,69,1],[7861,3,1],[7861,48,1],[7862,1,2],[7862,5,3],
[7862,8,2],[7862,13,1],[7862,15,1],[7862,21,2],[7862,28,1],
[7862,29,1],[7862,39,1],[7862,53,1],[7862,55,1],[7863,2,1],
[7863,23,1],[7863,33,1],[7863,43,1],[7864,1,2],[7864,2,1],[7864,20,1],
[7865,2,10],[7865,21,2],[7865,31,1],[7865,43,2],[7865,73,1],
[7866,2,1],[7866,40,1],[7866,42,1],[7866,63,1],[7866,70,1],
[7867,27,1],[7867,45,1],[7868,28,1],[7868,75,1],[7869,17,1],
[7869,59,1],[7870,31,2],[7871,2,21],[7872,24,2],[7872,26,1],
[7873,25,1],[7873,26,1],[7873,40,2],[7873,66,1],[7874,12,1],
[7874,29,1],[7874,57,1],[7874,59,1],[7875,22,1],[7875,23,5],
[7875,25,1],[7875,26,1],[7876,6,1],[7876,22,4],[7876,56,2],
[7877,28,2],[7878,6,1],[7878,21,1],[7878,24,1],[7878,25,1],
[7878,47,4],[7878,50,1],[7878,51,1],[7878,70,1],[7878,74,5],
[7878,76,2],[7879,26,1],[7879,32,1],[7879,42,7],[7880,6,1],
[7880,31,1],[7881,1,2],[7881,27,2],[7881,76,1],[7882,23,2],
[7882,25,2],[7882,27,1],[7882,31,26],[7882,33,1],[7882,39,3],
[7882,42,1],[7882,43,1],[7882,56,1],[7882,76,1],[7883,4,1],
[7883,15,27],[7883,19,1],[7883,50,1],[7883,65,1],[7884,22,1],
[7884,23,1],[7885,26,3],[7885,51,1],[7886,3,5],[7886,24,3],

[7886,26,2],[7886,27,1],[7886,33,1],[7886,62,2],[7887,3,2],
[7887,27,1],[7887,38,1],[7887,56,2],[7887,63,1],[7887,64,1],
[7888,70,2],[7889,28,3],[7889,31,2],[7890,1,1],[7890,2,4],[7890,3,4],
[7890,4,8],[7890,5,3],[7890,6,2],[7890,21,1435],[7890,22,2],
[7890,23,9],[7890,24,12],[7890,25,4],[7890,26,999],[7890,27,11],
[7890,28,187],[7890,31,4115],[7890,32,7],[7890,33,3050],[7890,35,6],
[7890,38,2],[7890,40,8],[7890,42,1655],[7890,43,815],[7890,44,5],
[7890,47,3],[7890,48,1],[7890,50,11],[7890,52,1],[7890,56,3],
[7890,61,1],[7890,62,94],[7890,66,6],[7890,68,4],[7890,69,3],
[7890,70,9],[7890,72,2],[7890,74,2],[7890,76,4],[7890,79,3],
[7891,0,6],[7891,1,22],[7891,2,1],[7891,3,1],[7891,5,2],[7891,7,10],
[7891,8,19],[7891,9,92],[7891,10,151],[7891,11,51],[7891,12,49],
[7891,13,23],[7891,14,36],[7891,15,22],[7891,16,75],[7891,17,8],
[7891,18,45],[7891,19,114],[7891,20,20],[7891,24,2],[7891,27,1],
[7891,28,18],[7891,29,11],[7891,30,4],[7891,32,1],[7891,34,29],
[7891,35,1],[7891,36,33],[7891,37,44],[7891,39,8],[7891,40,1],
[7891,41,73],[7891,42,1],[7891,43,1],[7891,45,86],[7891,46,47],
[7891,49,28],[7891,50,1],[7891,53,14],[7891,54,31],[7891,55,26],
[7891,56,1],[7891,57,57],[7891,58,61],[7891,59,57],[7891,60,25],
[7891,62,1],[7891,63,29],[7891,64,19],[7891,65,108],[7891,66,3],
[7891,67,48],[7891,70,4],[7891,71,38],[7891,73,37],[7891,74,23],
[7891,75,17],[7891,76,28],[7891,77,54],[7891,78,6],[7892,29,2],
[7893,26,1],[7893,27,4],[7894,1,2],[7894,3,1],[7894,6,1],[7894,13,5],
[7894,17,2],[7894,23,1],[7894,24,9],[7894,28,1],[7894,34,1],
[7894,40,1],[7894,73,1],[7895,3,1],[7895,5,4],[7895,22,1],[7896,23,1],
[7896,32,1],[7896,63,1],[7897,1,1],[7897,4,6],[7897,5,1],[7897,8,1],
[7897,10,1],[7897,11,2],[7897,12,1],[7897,15,1],[7897,16,1],
[7897,19,1],[7897,20,1],[7897,21,1],[7897,22,1],[7897,23,1],
[7897,24,1],[7897,26,1],[7897,27,2],[7897,30,1],[7897,33,1],
[7897,35,1],[7897,44,42],[7897,47,1],[7897,50,6],[7897,53,1],
[7897,54,1],[7897,55,4],[7897,56,1],[7897,57,1],[7897,60,1],
[7897,65,1],[7897,66,33],[7897,73,1],[7897,76,1],[7897,77,1],
[7898,0,3],[7898,1,11],[7898,2,1],[7898,3,2],[7898,4,1],[7898,5,3],
[7898,7,1],[7898,8,3],[7898,9,12],[7898,10,17],[7898,11,5],
[7898,12,9],[7898,13,8],[7898,14,4],[7898,15,8],[7898,16,8],
[7898,17,5],[7898,18,2],[7898,19,21],[7898,20,10],[7898,21,1],
[7898,22,1],[7898,23,2],[7898,24,3],[7898,26,1],[7898,27,1],
[7898,28,20],[7898,29,4],[7898,30,3],[7898,31,5],[7898,32,1],
[7898,33,2],[7898,35,2],[7898,36,5],[7898,37,7],[7898,38,3],
[7898,39,4],[7898,40,1],[7898,41,3],[7898,43,3],[7898,44,1],
[7898,45,1],[7898,46,5],[7898,47,1],[7898,48,2],[7898,49,2],
[7898,50,4],[7898,51,1],[7898,53,1],[7898,54,4],[7898,55,8],
[7898,56,2],[7898,57,5],[7898,58,6],[7898,59,3],[7898,63,4],
[7898,64,5],[7898,65,12],[7898,66,6],[7898,67,2],[7898,69,1],
[7898,70,3],[7898,71,3],[7898,72,1],[7898,73,3],[7898,74,1],
[7898,75,3],[7898,77,8],[7899,44,2],[7899,50,1],[7899,66,2],
[7900,2,4],[7901,2,23],[7901,3,1],[7901,32,1],[7901,42,1],[7901,43,2],
[7901,44,1],[7901,47,1],[7901,62,1],[7901,70,1],[7902,27,1],
[7902,28,1],[7903,9,2],[7903,20,2],[7904,5,2],[7904,21,1],[7904,52,1],
[7905,7,2],[7906,25,1],[7906,26,1],[7906,27,1],[7907,35,1],

[7907,42,1],[7907,43,1],[7907,60,1],[7908,22,2],[7908,23,1],
[7908,24,1],[7908,31,1],[7908,47,1],[7908,68,3],[7909,7,1],
[7909,13,1],[7909,24,1],[7909,28,1],[7910,9,3],[7910,10,1],
[7910,17,1],[7910,55,2],[7911,3,1],[7911,22,2],[7911,24,3],
[7911,62,1],[7912,15,3],[7912,17,3],[7912,53,3],[7912,55,2],
[7913,1,1],[7913,39,1],[7913,55,1],[7913,65,1],[7914,12,1],
[7914,55,1],[7915,22,6],[7915,23,3],[7915,24,1],[7915,27,3],
[7915,33,3],[7915,35,1],[7916,1,6],[7916,3,4],[7916,4,2],[7916,5,2],
[7916,9,1],[7916,10,1],[7916,20,2],[7916,21,1],[7916,23,6],
[7916,24,6],[7916,25,1],[7916,26,1],[7916,27,8],[7916,28,2],
[7916,30,4],[7916,31,4],[7916,32,2],[7916,36,1],[7916,37,1],
[7916,41,1],[7916,42,3],[7916,43,1],[7916,47,2],[7916,48,1],
[7916,54,1],[7916,57,1],[7916,65,1],[7917,9,9],[7917,28,45],
[7917,44,1],[7917,46,1],[7917,74,1],[7917,75,1],[7917,76,1],
[7918,30,1],[7918,34,1],[7919,29,1],[7919,30,1],[7920,23,1],
[7920,24,1],[7921,17,1],[7921,53,2],[7922,33,1],[7922,47,1],
[7923,47,1],[7923,68,1],[7924,0,1],[7924,2,1],[7924,5,1],[7924,18,1],
[7924,21,1],[7924,24,1],[7924,25,4],[7924,28,1],[7924,31,13],
[7924,32,6],[7924,33,1],[7924,34,1],[7924,39,1],[7924,42,1],
[7924,62,2],[7924,70,1],[7924,72,1],[7924,76,1],[7924,78,2],
[7925,41,1],[7925,78,1],[7926,20,3],[7927,7,1],[7927,55,1],[7928,3,1],
[7928,5,1],[7928,22,4],[7928,23,1],[7928,27,7],[7928,28,1],
[7928,35,2],[7928,76,1],[7929,1,5],[7929,8,3],[7929,12,1],[7929,13,1],
[7929,15,1],[7929,17,1],[7929,20,2],[7929,25,1],[7929,30,1],
[7929,35,1],[7929,36,1],[7929,39,2],[7929,53,2],[7929,55,3],
[7929,59,1],[7929,73,2],[7930,3,1],[7930,7,1],[7930,22,2],[7930,30,1],
[7930,34,1],[7930,35,1],[7930,43,1],[7931,1,1],[7931,2,1],[7931,28,1],
[7932,27,1],[7932,39,1],[7933,22,1],[7933,25,1],[7934,74,1],
[7934,76,1],[7935,22,1],[7935,28,1],[7936,2,15],[7936,3,2],[7936,4,1],
[7936,8,1],[7936,10,1],[7936,12,1],[7936,15,1],[7936,16,1],
[7936,17,1],[7936,18,1],[7936,20,5],[7936,22,1],[7936,23,1],
[7936,24,2],[7936,25,1],[7936,26,1],[7936,27,6],[7936,28,2],
[7936,30,2],[7936,31,6],[7936,37,1],[7936,39,1],[7936,41,1],
[7936,42,4],[7936,46,2],[7936,47,1],[7936,48,2],[7936,53,1],
[7936,55,1],[7936,60,1],[7936,62,2],[7936,63,1],[7936,70,1],
[7936,73,1],[7936,74,1],[7936,77,1],[7937,8,1],[7937,16,1],
[7937,30,1],[7937,34,1],[7938,23,2],[7938,25,1],[7938,62,1],
[7939,38,48],[7939,76,1],[7940,1,1],[7940,4,1],[7940,13,1],
[7940,20,1],[7940,26,1],[7940,27,1],[7940,38,1],[7940,47,1],
[7940,51,1],[7940,56,1],[7941,0,1],[7941,1,77],[7941,2,2],[7941,7,1],
[7941,8,8],[7941,12,12],[7941,13,57],[7941,15,2],[7941,16,24],
[7941,17,4],[7941,20,3],[7941,22,2],[7941,23,7],[7941,26,2],
[7941,27,8],[7941,28,2],[7941,29,14],[7941,34,3],[7941,35,17],
[7941,36,1],[7941,37,1],[7941,39,7],[7941,42,4],[7941,47,15],
[7941,48,10],[7941,53,6],[7941,55,3],[7941,57,1],[7941,66,1],
[7941,70,1],[7941,73,3],[7942,24,2],[7942,35,1],[7942,42,2],
[7943,23,1],[7943,24,2],[7943,25,5],[7943,28,2],[7943,31,2],
[7943,47,1],[7944,28,4],[7944,30,3],[7944,33,10],[7944,35,1],
[7945,31,1],[7945,72,1],[7945,76,1],[7946,9,1],[7946,55,1],
[7946,66,1],[7947,1,1],[7947,4,2],[7947,6,1],[7947,12,2],[7947,16,1],

[7947,19,1],[7947,22,2],[7947,24,1],[7947,25,3],[7947,28,1],
[7947,29,2],[7947,32,3],[7947,43,2],[7947,47,1],[7947,48,1],
[7947,49,1],[7947,56,3],[7947,62,3],[7947,66,1],[7947,69,4],
[7947,70,6],[7948,5,3],[7949,21,3],[7949,23,3],[7949,25,2],
[7949,26,4],[7949,28,3],[7949,31,8],[7949,33,2],[7949,43,1],
[7950,28,3],[7951,19,1],[7951,20,3],[7951,27,1],[7951,29,1],
[7951,34,1],[7951,36,3],[7952,28,1],[7952,56,1],[7953,0,1],[7953,9,1],
[7953,14,1],[7953,39,1],[7953,53,1],[7953,58,1],[7954,23,1],
[7954,27,1],[7954,32,3],[7954,35,1],[7955,59,1],[7955,69,1],
[7956,42,13],[7957,22,1],[7957,23,1],[7957,31,1],[7957,51,2],
[7957,62,2],[7957,66,1],[7957,69,1],[7957,70,3],[7958,3,1],[7958,5,1],
[7959,0,2],[7960,6,2],[7960,47,1],[7960,51,1],[7960,56,1],[7960,68,1],
[7960,69,3],[7960,70,1],[7961,23,1],[7961,26,1],[7961,27,1],
[7961,35,1],[7961,47,1],[7962,8,2],[7962,33,3],[7962,35,2],
[7962,52,1],[7963,2,2],[7964,1,6],[7964,10,1],[7964,13,3],[7964,20,1],
[7964,29,2],[7964,39,4],[7965,22,3],[7965,27,1],[7965,35,1],
[7965,39,1],[7965,47,1],[7965,59,7],[7966,3,1],[7966,5,1],[7966,27,4],
[7966,31,1],[7967,20,1],[7967,52,1],[7968,8,1],[7968,20,4],
[7968,26,2],[7969,3,1],[7969,24,6],[7969,26,1],[7969,28,1],
[7970,22,1],[7970,23,2],[7970,27,2],[7970,33,1],[7970,35,1],
[7970,55,1],[7970,68,4],[7970,69,1],[7971,14,1],[7971,34,1],
[7971,53,1],[7972,6,1],[7972,7,2],[7972,13,2],[7972,22,1],[7972,27,5],
[7972,36,1],[7972,38,1],[7972,41,1],[7972,74,1],[7973,28,1],
[7973,43,1],[7974,25,6],[7974,28,2],[7974,40,1],[7974,62,5],
[7974,72,1],[7975,1,2],[7976,8,1],[7976,15,5],[7976,55,3],[7976,73,1],
[7977,3,1],[7977,8,1],[7977,17,1],[7977,20,2],[7977,21,1],[7977,31,5],
[7977,53,1],[7977,57,1],[7978,28,1],[7978,44,1],[7979,31,1],
[7979,66,1],[7980,22,1],[7980,24,1],[7981,2,3],[7982,1,1],[7982,2,1],
[7982,4,1],[7982,9,1],[7982,11,18],[7982,17,1],[7982,20,1],
[7982,24,1],[7982,30,1],[7982,34,1],[7982,35,2],[7982,37,1],
[7982,38,1],[7982,43,1],[7982,44,2],[7982,50,1],[7982,66,1],
[7982,70,1],[7983,22,1],[7983,24,3],[7983,28,1],[7983,31,6],
[7984,25,7],[7984,27,1],[7984,32,1],[7985,2,2],[7986,1,2],[7986,9,5],
[7986,14,1],[7986,15,2],[7986,16,1],[7986,19,1],[7986,20,1],
[7986,30,12],[7986,38,4],[7986,39,3],[7986,41,1],[7986,43,1],
[7986,45,1],[7986,46,1],[7986,52,19],[7986,55,2],[7986,59,1],
[7986,67,1],[7986,73,1],[7987,42,3],[7988,27,4],[7988,54,1],
[7989,13,1],[7989,26,1],[7990,0,2],[7990,1,22],[7990,2,2],[7990,3,2],
[7990,4,27],[7990,5,31],[7990,6,3],[7990,7,1],[7990,8,6],[7990,9,6],
[7990,10,4],[7990,11,1],[7990,12,4],[7990,13,13],[7990,14,2],
[7990,15,43],[7990,16,7],[7990,17,8],[7990,18,2],[7990,19,4],
[7990,20,110],[7990,21,10],[7990,22,9],[7990,23,18],[7990,24,8],
[7990,25,2],[7990,26,5],[7990,27,10],[7990,28,8],[7990,29,2],
[7990,30,4],[7990,32,17],[7990,33,5],[7990,34,4],[7990,35,13],
[7990,38,5],[7990,39,2],[7990,40,3],[7990,41,4],[7990,42,6],
[7990,43,4],[7990,44,16],[7990,45,2],[7990,46,1],[7990,47,6],
[7990,49,1],[7990,50,35],[7990,51,3],[7990,53,8],[7990,55,17],
[7990,56,11],[7990,58,3],[7990,59,14],[7990,61,2],[7990,62,11],
[7990,63,1],[7990,64,1],[7990,65,6],[7990,66,15],[7990,67,1],
[7990,68,6],[7990,69,1],[7990,70,3],[7990,72,1],[7990,73,1],

[7990,74,4],[7990,76,1],[7990,77,1],[7991,2,1],[7991,28,1],
[7992,26,2],[7992,28,1],[7992,31,5],[7993,13,2],[7993,17,1],
[7993,20,1],[7993,45,1],[7993,65,1],[7993,77,1],[7994,28,1],
[7994,62,1],[7995,28,6],[7996,1,2],[7997,0,3],[7997,1,8],[7997,3,1],
[7997,4,1],[7997,7,1],[7997,9,3],[7997,10,5],[7997,11,2],[7997,12,4],
[7997,13,4],[7997,15,3],[7997,16,2],[7997,17,1],[7997,18,1],
[7997,19,1],[7997,20,3],[7997,28,2],[7997,30,3],[7997,34,1],
[7997,37,1],[7997,39,1],[7997,41,1],[7997,46,2],[7997,55,1],
[7997,58,1],[7997,59,1],[7997,60,2],[7997,67,3],[7997,73,1],
[7997,77,2],[7997,79,1],[7998,3,2],[7998,24,2],[7998,25,8],
[7998,27,1],[7998,35,1],[7998,43,1],[7998,47,1],[7998,53,2],
[7998,68,1],[7999,1,1],[7999,8,1],[7999,21,1],[7999,28,1],[7999,31,2],
[8000,24,1],[8000,27,1],[8000,35,2],[8001,2,3],[8001,3,1],[8001,4,2],
[8001,5,2],[8001,12,1],[8001,13,2],[8001,15,1],[8001,19,1],
[8001,22,1],[8001,23,23],[8001,24,12],[8001,25,1],[8001,26,1],
[8001,27,3],[8001,28,10],[8001,32,96],[8001,33,1],[8001,35,6],
[8001,38,1],[8001,40,1],[8001,44,1],[8001,47,1],[8001,48,4],
[8001,49,1],[8001,50,1],[8001,53,1],[8001,62,5],[8001,66,1],
[8001,68,1],[8001,70,1],[8001,74,4],[8002,17,2],[8003,23,11],
[8003,35,1],[8004,11,1],[8004,72,1],[8005,23,1],[8005,26,1],
[8005,66,1],[8006,15,1],[8006,27,1],[8007,2,1],[8007,25,1],
[8008,23,1],[8008,27,2],[8008,28,5],[8008,43,1],[8008,70,1],
[8009,6,1],[8009,26,1],[8009,32,1],[8009,40,1],[8009,50,1],
[8009,62,5],[8010,62,2],[8011,1,1],[8011,2,6],[8011,3,2],[8011,7,1],
[8011,12,3],[8011,20,4],[8011,21,4],[8011,22,1],[8011,23,2],
[8011,25,1],[8011,26,1],[8011,27,2],[8011,31,21],[8011,39,1],
[8011,42,5],[8011,47,8],[8011,53,2],[8011,70,1],[8012,5,1],
[8012,13,1],[8013,2,2],[8013,21,1],[8013,23,2],[8013,26,2],
[8013,27,3],[8013,31,2],[8013,33,1],[8013,35,3],[8013,42,1],
[8013,43,1],[8013,45,1],[8013,47,1],[8013,48,3],[8014,23,2],
[8014,24,2],[8014,26,1],[8014,27,2],[8014,31,1],[8014,39,1],
[8014,43,1],[8015,24,3],[8016,12,1],[8016,42,1],[8017,28,1],
[8017,42,1],[8017,63,1],[8017,74,1],[8017,76,2],[8018,3,1],[8018,6,1],
[8018,51,1],[8018,57,1],[8018,74,1],[8019,21,1],[8019,31,1],
[8019,33,1],[8019,42,1],[8019,51,5],[8020,3,1],[8020,27,1],
[8021,24,1],[8021,28,1],[8021,56,41],[8022,15,1],[8022,50,1],
[8022,55,1],[8023,4,2],[8023,15,3],[8023,22,1],[8023,38,1],
[8023,44,9],[8023,45,1],[8023,47,1],[8023,50,7],[8023,55,1],
[8023,66,9],[8023,76,1],[8023,77,1],[8024,8,2],[8024,39,1],
[8025,12,1],[8025,28,1],[8026,2,2],[8026,31,1],[8026,42,1],
[8026,65,1],[8027,15,1],[8027,35,1],[8028,27,1],[8028,47,1],
[8028,66,1],[8029,32,22],[8029,35,1],[8029,39,3],[8029,42,2],
[8029,43,3],[8030,2,9],[8030,3,2],[8030,13,1],[8030,20,1],[8030,22,2],
[8030,24,1],[8030,26,1],[8030,27,1],[8030,35,1],[8030,42,1],
[8030,56,3],[8030,60,1],[8031,2,1],[8031,8,9],[8031,28,2],[8031,31,8],
[8031,34,1],[8031,35,2],[8031,44,1],[8031,58,1],[8031,77,1],
[8032,4,1],[8032,6,1],[8032,23,1],[8032,28,1],[8033,8,1],[8033,24,1],
[8033,26,1],[8033,43,1],[8034,27,1],[8034,31,1],[8035,2,4],
[8035,31,5],[8036,27,1],[8036,56,3],[8037,2,1],[8037,28,1],
[8037,33,7],[8037,35,1],[8037,75,1],[8037,76,1],[8038,2,12],

[8038,3,11],[8038,4,20],[8038,5,33],[8038,6,2],[8038,7,1],[8038,9,10],
[8038,10,4],[8038,11,2],[8038,12,1],[8038,14,1],[8038,16,1],
[8038,18,1],[8038,19,2],[8038,20,1],[8038,21,4],[8038,22,10],
[8038,23,26],[8038,24,13],[8038,25,11],[8038,26,12],[8038,27,11],
[8038,28,3312],[8038,31,16],[8038,32,9],[8038,33,11],[8038,35,11],
[8038,38,9],[8038,40,40],[8038,41,2],[8038,42,7],[8038,43,5],
[8038,44,16],[8038,45,3],[8038,46,1],[8038,47,1],[8038,48,2],
[8038,49,1],[8038,50,11],[8038,51,3],[8038,52,3],[8038,53,1],
[8038,54,2],[8038,55,2],[8038,56,3],[8038,61,1],[8038,62,9],
[8038,63,1340],[8038,65,2],[8038,66,10],[8038,67,2],[8038,68,34],
[8038,69,17],[8038,70,61],[8038,71,2],[8038,72,30],[8038,73,1],
[8038,74,1058],[8038,75,982],[8038,76,1706],[8038,77,1],[8038,79,2],
[8039,28,5],[8039,35,1],[8039,55,1],[8039,66,2],[8040,27,1],
[8040,32,2],[8040,35,2],[8040,40,3],[8041,21,2],[8041,23,17],
[8041,24,1],[8041,26,2],[8041,27,16],[8041,28,2],[8041,30,1],
[8041,31,43],[8041,33,7],[8041,35,22],[8041,38,1],[8041,42,7],
[8041,43,2],[8042,2,2],[8042,24,1],[8042,28,1],[8042,32,1],
[8042,43,2],[8043,20,1],[8043,31,1],[8044,4,1],[8044,12,1],
[8044,44,2],[8044,66,4],[8045,2,1],[8045,12,1],[8046,6,1],[8046,22,1],
[8046,23,1],[8046,27,2],[8046,30,1],[8046,56,1],[8047,28,3],
[8047,47,1],[8047,74,1],[8048,24,1],[8048,26,1],[8049,4,1],[8049,9,1],
[8049,10,1],[8049,16,1],[8049,17,1],[8049,19,1],[8049,28,1],
[8049,38,1],[8049,41,1],[8049,44,17],[8049,50,3],[8049,55,1],
[8049,65,2],[8049,66,8],[8049,67,1],[8049,75,1],[8049,77,2],
[8050,27,1],[8050,62,1],[8051,39,1],[8051,66,1],[8052,16,1],
[8052,52,1],[8053,21,1],[8053,22,2],[8053,27,2],[8054,28,3],
[8055,4,1],[8055,20,1],[8055,22,1],[8055,23,1],[8055,26,1],
[8055,27,2],[8055,31,1],[8055,39,1],[8055,43,1],[8055,50,1],
[8055,62,1],[8056,10,1],[8056,15,1],[8056,16,1],[8056,19,2],
[8056,20,1],[8056,60,2],[8056,65,1],[8057,15,1],[8057,44,4],
[8057,66,1],[8058,25,8],[8059,6,1],[8059,21,1],[8059,23,4],
[8059,24,1],[8059,25,4],[8059,27,8],[8059,31,7],[8059,35,2],
[8059,42,1],[8059,52,1],[8060,40,7],[8060,68,7],[8060,69,3],
[8060,70,14],[8060,72,3],[8061,3,1],[8061,24,1],[8062,24,1],
[8062,26,1],[8063,24,3],[8063,32,1],[8064,22,2],[8065,1,2],[8065,2,5],
[8065,3,2],[8065,12,9],[8065,13,1],[8065,25,1],[8065,30,3],
[8065,31,4],[8065,42,1],[8065,47,4],[8065,69,1],[8065,75,1],
[8066,1,2],[8066,23,1],[8067,26,2],[8068,9,3],[8068,17,4],[8068,25,1],
[8068,28,4],[8068,31,1],[8068,33,1],[8068,39,1],[8068,43,1],
[8069,2,2],[8069,3,3],[8069,4,1],[8069,5,1],[8069,7,1],[8069,10,2],
[8069,11,1],[8069,12,2],[8069,18,7],[8069,19,1],[8069,23,3],
[8069,24,4],[8069,25,3],[8069,26,2],[8069,27,23],[8069,29,1],
[8069,31,6],[8069,32,18],[8069,33,1],[8069,35,5],[8069,38,1],
[8069,39,2],[8069,40,7],[8069,42,16],[8069,46,1],[8069,47,4],
[8069,48,4],[8069,50,1],[8069,56,49],[8069,61,18],[8069,70,29],
[8069,73,1],[8069,74,3],[8069,75,3],[8069,76,1],[8069,78,2],
[8070,26,1],[8070,43,2],[8071,23,2],[8071,27,1],[8071,28,2],
[8071,42,1],[8072,6,1],[8072,10,1],[8072,27,1],[8072,35,1],
[8073,28,1],[8073,30,1],[8073,37,1],[8073,43,1],[8074,3,1],[8074,5,1],
[8074,8,1],[8075,2,5],[8076,9,1],[8076,23,1],[8076,24,3],[8076,25,1],

[8076,56,1],[8077,9,1],[8077,15,1],[8077,16,1],[8077,46,1],
[8077,57,1],[8077,67,1],[8077,71,1],[8078,31,3],[8079,24,1],
[8079,33,1],[8079,44,1],[8080,2,1],[8080,33,2],[8081,5,3],[8081,8,1],
[8081,24,1],[8081,27,1],[8081,38,1],[8081,66,1],[8082,13,1],
[8082,17,1],[8082,20,3],[8082,22,3],[8082,24,1],[8082,27,3],
[8082,31,1],[8082,35,3],[8082,38,1],[8082,42,2],[8082,48,2],
[8083,37,1],[8083,48,1],[8084,17,1],[8084,55,2],[8085,21,1],
[8085,30,1],[8086,15,4],[8086,55,2],[8086,71,1],[8087,24,16],
[8087,62,1],[8087,63,1],[8087,76,1],[8088,1,1],[8088,2,1],[8088,5,1],
[8088,15,1],[8088,27,1],[8088,28,2],[8088,32,1],[8089,23,2],
[8090,3,1],[8090,31,1],[8090,48,1],[8091,23,1],[8091,43,1],
[8092,44,7],[8092,50,1],[8092,66,4],[8093,2,1],[8093,20,3],
[8093,26,1],[8093,28,1],[8093,30,1],[8093,47,1],[8093,63,1],
[8094,2,9],[8094,3,3],[8094,5,1],[8094,6,1],[8094,9,1],[8094,12,1],
[8094,13,4],[8094,16,1],[8094,20,8],[8094,21,1],[8094,22,2],
[8094,23,1],[8094,24,1],[8094,26,3],[8094,27,6],[8094,28,1],
[8094,31,1],[8094,33,1],[8094,35,7],[8094,38,1],[8094,39,1],
[8094,42,1],[8094,43,1],[8094,45,2],[8094,47,1],[8094,48,1],
[8094,52,1],[8094,53,1],[8094,55,1],[8094,59,2],[8094,60,1],
[8094,62,1],[8094,66,2],[8094,70,1],[8094,78,1],[8095,0,1],[8095,9,1],
[8095,10,2],[8095,13,1],[8095,16,1],[8095,17,1],[8095,34,1],
[8095,41,1],[8095,65,2],[8096,35,1],[8096,55,1],[8096,72,1],
[8097,1,11],[8097,7,1],[8097,15,1],[8097,29,1],[8097,39,1],
[8097,45,1],[8098,27,2],[8099,27,1],[8099,39,1],[8099,43,1],
[8100,63,6],[8101,2,1],[8101,25,1],[8101,27,1],[8101,35,1],
[8102,25,3],[8102,27,1],[8103,25,26],[8103,38,1],[8103,61,1],
[8103,68,1],[8103,70,1],[8104,15,1],[8104,53,1],[8105,0,1],[8105,1,1],
[8105,2,20],[8105,3,2],[8105,4,1],[8105,7,4],[8105,8,3],[8105,12,10],
[8105,13,1],[8105,16,3],[8105,17,3],[8105,19,1],[8105,20,8],
[8105,21,1],[8105,23,14],[8105,26,2],[8105,27,1],[8105,28,3],
[8105,30,1],[8105,31,9],[8105,34,1],[8105,37,1],[8105,39,1],
[8105,42,11],[8105,43,6],[8105,45,1],[8105,46,2],[8105,47,1],
[8105,49,1],[8105,53,1],[8105,57,1],[8105,68,1],[8105,77,1],
[8106,1,1],[8106,20,1],[8107,2,3],[8107,28,1],[8107,32,1],[8108,56,2],
[8109,26,1],[8109,28,3],[8110,43,1],[8110,51,1],[8110,56,1],
[8110,68,2],[8110,70,1],[8110,72,2],[8111,25,2],[8112,5,1],
[8112,32,1],[8113,56,2],[8114,4,1],[8114,28,1],[8114,66,1],
[8115,74,4],[8115,76,2],[8116,2,3],[8116,5,1],[8116,21,2],[8116,23,2],
[8116,26,4],[8116,42,6],[8116,43,2],[8116,50,1],[8116,53,1],
[8117,2,37],[8117,3,3],[8117,4,1],[8117,5,1],[8117,6,2],[8117,21,5],
[8117,22,6],[8117,23,30],[8117,24,19],[8117,26,10],[8117,27,13],
[8117,28,17],[8117,30,1],[8117,31,18],[8117,32,2],[8117,33,8],
[8117,34,1],[8117,35,7],[8117,37,8],[8117,39,1],[8117,42,11],
[8117,43,5],[8117,44,2],[8117,45,1],[8117,47,9],[8117,48,24],
[8117,56,10],[8117,61,2],[8117,63,1],[8117,64,1],[8117,66,1],
[8117,76,4],[8118,29,2],[8119,20,1],[8119,21,1],[8119,27,2],
[8119,28,5],[8119,33,1],[8119,35,1],[8119,43,1],[8120,34,1],
[8120,53,1],[8121,21,4],[8121,23,1],[8121,26,4],[8121,28,23],
[8121,31,5],[8121,33,3],[8121,39,2],[8121,42,1],[8122,2,1],
[8122,28,1],[8123,38,1],[8123,69,1],[8124,28,4],[8124,32,9],

[8124,69,1],[8125,31,3],[8126,16,1],[8126,28,1],[8126,44,1],
[8127,28,2],[8127,33,2],[8128,23,1],[8128,27,1],[8128,72,1],
[8129,76,3],[8130,24,1],[8130,28,1],[8130,33,3],[8131,28,2],
[8132,27,1],[8132,28,2],[8133,4,1],[8133,25,1],[8133,38,1],
[8133,66,1],[8134,2,2],[8134,5,1],[8134,7,1],[8134,8,1],[8134,13,2],
[8134,24,2],[8134,25,1],[8134,35,1],[8134,47,2],[8134,56,1],
[8135,10,1],[8135,16,1],[8135,46,1],[8135,55,1],[8135,67,1],
[8135,73,1],[8136,43,3],[8137,16,1],[8137,20,2],[8137,33,1],
[8138,12,1],[8138,15,1],[8138,46,1],[8139,2,1],[8139,33,1],
[8139,40,1],[8139,63,1],[8139,72,1],[8140,8,1],[8140,12,1],
[8140,33,1],[8141,24,1],[8141,56,1],[8142,10,1],[8142,47,1],
[8143,2,2],[8143,43,1],[8143,47,1],[8144,3,1],[8144,7,2],[8144,11,4],
[8144,17,1],[8144,18,1],[8144,21,1],[8144,24,1],[8144,26,1],
[8144,28,1],[8144,31,1],[8144,32,1],[8144,35,1],[8144,40,1],
[8144,43,1],[8144,56,6],[8144,70,1],[8145,2,4],[8146,5,1],[8146,28,1],
[8147,22,1],[8147,26,3],[8147,28,1],[8147,43,1],[8147,65,1],
[8148,4,5],[8148,5,1],[8148,9,1],[8148,15,12],[8148,17,12],
[8148,24,1],[8148,28,3],[8148,33,1],[8148,38,3],[8148,43,1],
[8148,44,24],[8148,47,2],[8148,50,6],[8148,53,3],[8148,55,12],
[8148,62,1],[8148,66,17],[8148,70,1],[8148,74,1],[8148,76,2],
[8149,5,3],[8149,28,1],[8150,3,1],[8150,22,1],[8150,24,1],[8150,26,1],
[8150,28,1],[8150,43,1],[8150,47,1],[8150,50,1],[8151,21,1],
[8151,28,1],[8152,23,3],[8152,25,1],[8152,28,3],[8153,23,2],
[8153,43,3],[8154,47,1],[8154,68,1],[8155,2,6],[8155,21,1],
[8156,22,1],[8156,23,2],[8156,24,4],[8156,25,2],[8156,26,1],
[8156,28,2],[8156,31,4],[8157,21,2],[8157,26,1],[8158,17,2],
[8158,25,1],[8158,53,1],[8159,2,1],[8159,9,1],[8159,22,1],[8159,24,2],
[8159,27,1],[8159,32,4],[8159,33,1],[8159,35,1],[8159,42,19],
[8159,50,1],[8159,51,1],[8159,55,1],[8159,56,1],[8159,66,1],
[8159,69,1],[8159,74,3],[8160,4,1],[8160,66,1],[8161,28,5],
[8161,70,1],[8162,9,1],[8162,12,2],[8162,20,1],[8162,23,5],
[8162,28,1],[8162,56,1],[8162,59,4],[8163,21,1],[8163,28,1],
[8163,33,1],[8163,52,2],[8164,1,1],[8164,11,1],[8164,12,1],
[8164,13,8],[8164,19,2],[8164,33,1],[8164,46,1],[8164,65,1],
[8165,19,1],[8165,65,1],[8166,31,3],[8167,8,1],[8167,10,1],
[8167,19,1],[8167,34,1],[8167,55,1],[8167,64,1],[8168,9,1],
[8168,17,1],[8168,53,1],[8169,0,3],[8170,20,1],[8170,68,1],[8171,2,1],
[8171,33,5],[8171,35,2],[8172,0,1],[8172,1,4],[8172,2,2],[8172,3,1],
[8172,4,125],[8172,5,8],[8172,6,1],[8172,8,1],[8172,9,8],[8172,10,5],
[8172,11,2],[8172,12,6],[8172,13,8],[8172,15,115],[8172,16,3],
[8172,17,107],[8172,18,1],[8172,19,7],[8172,20,12],[8172,22,7],
[8172,23,1],[8172,24,1],[8172,26,3],[8172,27,1],[8172,28,8],
[8172,29,2],[8172,30,4],[8172,32,3],[8172,33,3],[8172,34,1],
[8172,35,3],[8172,36,1],[8172,37,3],[8172,38,7],[8172,39,1],
[8172,40,1],[8172,41,2],[8172,42,2],[8172,43,3],[8172,44,47],
[8172,45,3],[8172,46,6],[8172,47,3],[8172,48,1],[8172,49,1],
[8172,50,137],[8172,51,2],[8172,52,1],[8172,53,112],[8172,54,1],
[8172,55,62],[8172,56,1],[8172,57,4],[8172,58,2],[8172,59,3],
[8172,62,1],[8172,63,5],[8172,64,1],[8172,65,5],[8172,66,27],
[8172,67,2],[8172,68,2],[8172,69,3],[8172,70,4],[8172,71,4],

[8172,72,3],[8172,73,4],[8172,74,3],[8172,75,2],[8172,77,2],
[8172,78,2],[8173,2,7],[8173,3,1],[8173,14,1],[8173,20,3],[8173,23,3],
[8173,27,2],[8173,28,1],[8173,32,2],[8173,33,1],[8173,35,3],
[8173,43,1],[8173,53,1],[8173,59,1],[8173,62,1],[8174,3,1],
[8174,25,1],[8174,26,1],[8174,27,1],[8174,28,1],[8174,31,1],
[8175,23,1],[8175,25,1],[8175,32,1],[8175,44,1],[8175,47,1],
[8175,66,1],[8175,70,1],[8176,21,2],[8176,34,5],[8177,22,2],
[8177,24,1],[8178,32,10],[8178,52,1],[8179,30,2],[8180,18,2],
[8181,0,4],[8181,73,1],[8182,42,1],[8182,74,1],[8183,1,1],[8183,26,1],
[8183,43,1],[8184,19,1],[8184,55,1],[8185,5,1],[8185,26,1],
[8185,35,1],[8186,8,1],[8186,13,1],[8186,50,1],[8186,55,1],
[8186,58,1],[8186,67,1],[8187,23,1],[8187,28,1],[8187,33,1],
[8188,6,1],[8188,39,3],[8189,3,1],[8189,73,1],[8190,12,1],[8190,18,1],
[8190,58,1],[8190,71,1],[8191,28,2],[8191,31,1],[8191,63,1],
[8191,72,1],[8192,2,1],[8192,27,1],[8193,5,1],[8193,56,1],[8194,9,1],
[8194,73,1],[8195,25,2],[8195,26,1],[8196,61,1],[8196,66,1],
[8196,68,2],[8197,17,1],[8197,19,1],[8197,65,1],[8198,17,1],
[8198,53,1],[8199,28,1],[8199,44,1],[8200,2,1],[8200,30,1],
[8200,35,1],[8201,14,1],[8201,21,1],[8201,22,1],[8201,23,1],
[8201,27,10],[8201,28,5],[8201,31,4],[8201,33,1],[8201,35,5],
[8201,43,1],[8202,8,1],[8202,27,4],[8202,70,1],[8203,1,42],
[8203,12,2],[8203,13,2],[8203,15,1],[8203,16,1],[8203,29,1],
[8203,34,1],[8203,39,1],[8203,55,1],[8203,67,1],[8203,73,1],
[8204,22,1],[8204,26,2],[8204,31,1],[8204,32,2],[8204,38,1],
[8204,47,2],[8204,66,1],[8205,3,2],[8205,42,1],[8205,47,1],
[8205,60,1],[8206,12,1],[8206,25,3],[8207,5,1],[8207,27,1],
[8207,31,6],[8207,42,1],[8207,72,1],[8208,6,1],[8208,23,3],
[8208,24,1],[8208,27,4],[8208,28,3],[8208,39,1],[8209,8,1],[8209,9,1],
[8209,16,1],[8209,20,1],[8209,29,59],[8209,58,1],[8209,65,3],
[8209,78,1],[8210,9,1],[8210,65,1],[8211,2,1],[8211,20,1],[8211,21,1],
[8211,23,1],[8211,25,1],[8211,27,1],[8211,32,1],[8211,39,1],
[8212,28,2],[8212,37,2],[8213,25,2],[8214,21,1],[8214,22,1],
[8214,23,1],[8214,33,1],[8215,40,1],[8215,70,1],[8216,28,1],
[8216,33,2],[8217,3,1],[8217,27,1],[8217,35,1],[8217,47,1],
[8217,74,1],[8218,28,1],[8218,68,1],[8219,1,5],[8219,13,3],
[8219,21,2],[8219,22,1],[8219,25,1],[8219,33,6],[8219,34,1],
[8219,39,1],[8219,52,1],[8220,24,1],[8220,28,1],[8220,35,1],
[8221,8,1],[8221,25,1],[8221,27,2],[8221,34,1],[8221,75,1],[8222,2,1],
[8222,3,1],[8222,21,1],[8222,22,3],[8222,23,2],[8222,25,1],
[8222,26,2],[8222,27,24],[8222,28,4],[8222,31,5],[8222,33,1],
[8222,35,2],[8222,47,6],[8222,50,1],[8222,63,1],[8222,69,1],
[8223,31,2],[8224,0,1],[8224,1,40],[8224,2,12],[8224,4,3],[8224,5,1],
[8224,6,4],[8224,7,24],[8224,8,1],[8224,9,5],[8224,10,3],[8224,11,35],
[8224,12,96],[8224,13,45],[8224,14,3],[8224,15,11],[8224,16,61],
[8224,17,1],[8224,18,2],[8224,19,3],[8224,20,17],[8224,21,1],
[8224,22,3],[8224,23,86],[8224,24,57],[8224,25,6],[8224,26,6],
[8224,27,7],[8224,28,9],[8224,29,29],[8224,30,3],[8224,31,26],
[8224,32,81],[8224,33,9],[8224,34,1],[8224,35,3],[8224,36,3],
[8224,37,2],[8224,38,1],[8224,39,6],[8224,40,4],[8224,42,2],
[8224,43,1],[8224,45,4],[8224,46,1],[8224,47,1],[8224,49,2],

[8224,50,3],[8224,51,8],[8224,53,3],[8224,54,1],[8224,55,3],
[8224,56,13],[8224,57,1],[8224,58,2],[8224,59,1],[8224,62,8],
[8224,63,1],[8224,64,1],[8224,65,5],[8224,66,2],[8224,68,1],
[8224,69,3],[8224,70,30],[8224,72,3],[8224,73,1],[8224,74,3],
[8224,75,1],[8224,76,1],[8224,77,47],[8225,26,1],[8225,27,6],
[8225,32,1],[8226,9,1],[8226,10,1],[8226,16,1],[8226,19,1],
[8226,36,1],[8226,37,1],[8226,77,1],[8227,1,1],[8227,22,1],
[8227,29,1],[8227,42,1],[8227,52,1],[8228,21,1],[8228,42,1],
[8229,0,1],[8229,8,1],[8229,9,5],[8229,10,1],[8229,12,1],[8229,16,2],
[8229,17,1],[8229,19,2],[8229,36,1],[8229,39,1],[8229,41,2],
[8229,45,2],[8229,46,1],[8229,53,1],[8229,55,2],[8229,58,1],
[8229,59,1],[8229,65,4],[8229,67,2],[8229,71,2],[8229,73,1],
[8229,77,1],[8230,1,1],[8230,3,1],[8230,4,13],[8230,5,4],[8230,8,1],
[8230,9,1],[8230,10,2],[8230,15,2],[8230,16,3],[8230,17,3],
[8230,19,2],[8230,20,1],[8230,21,1],[8230,22,1],[8230,23,1],
[8230,25,3],[8230,28,7],[8230,34,1],[8230,35,1],[8230,36,1],
[8230,37,1],[8230,38,19],[8230,40,1],[8230,41,1],[8230,44,65],
[8230,45,1],[8230,47,2],[8230,49,1],[8230,50,18],[8230,63,3],
[8230,65,2],[8230,66,51],[8230,67,1],[8230,68,2],[8230,70,1],
[8230,71,1],[8230,72,1],[8230,74,1],[8230,75,1],[8230,76,2],
[8231,15,1],[8231,17,1],[8232,28,1],[8232,62,1],[8233,23,2],
[8233,24,2],[8234,31,4],[8235,0,1],[8235,8,1],[8235,10,1],[8235,13,1],
[8235,16,1],[8235,29,2],[8235,45,1],[8235,66,1],[8235,71,1],
[8236,2,1],[8236,32,1],[8237,0,1],[8237,71,1],[8237,72,6],[8237,78,1],
[8238,3,1],[8238,27,1],[8238,38,1],[8239,24,1],[8239,31,1],
[8240,23,1],[8240,24,3],[8240,28,1],[8241,2,1],[8241,21,1],
[8241,24,1],[8241,25,1],[8241,42,1],[8242,30,1],[8242,52,1],
[8242,74,6],[8243,15,1],[8243,20,1],[8243,33,1],[8244,27,1],
[8244,52,1],[8245,21,1],[8245,25,1],[8246,28,2],[8247,4,1],[8247,5,1],
[8247,26,1],[8247,55,1],[8247,79,1],[8248,2,9],[8249,30,6],[8250,3,1],
[8250,4,1],[8250,23,1],[8250,30,1],[8250,33,3],[8250,38,1],
[8250,43,1],[8250,44,2],[8250,50,1],[8250,59,1],[8250,66,1],
[8251,4,1],[8251,31,1],[8251,50,1],[8252,22,1],[8252,24,4],
[8252,26,1],[8252,28,1],[8252,56,1],[8253,1,1],[8253,12,1],
[8253,13,1],[8253,29,1],[8254,3,1],[8254,26,1],[8254,27,1],
[8254,48,1],[8255,6,1],[8255,28,1],[8255,44,1],[8256,10,1],
[8256,20,1],[8257,31,3],[8257,33,1],[8258,4,8],[8258,5,2],
[8258,50,13],[8259,20,1],[8259,55,1],[8260,2,2],[8260,3,1],[8260,5,2],
[8260,9,1],[8260,12,4],[8260,13,1],[8260,18,1],[8260,20,2],
[8260,22,1],[8260,28,1],[8260,30,1],[8260,35,1],[8260,39,2],
[8260,42,1],[8260,48,1],[8260,50,1],[8260,56,1],[8260,73,1],
[8261,6,1],[8261,51,1],[8261,56,1],[8261,69,1],[8262,23,1],
[8262,24,26],[8262,27,23],[8262,28,1],[8262,32,6],[8262,35,6],
[8262,42,11],[8262,43,1],[8262,44,1],[8262,47,1],[8262,53,1],
[8262,59,3],[8263,9,1],[8263,24,1],[8263,28,1],[8263,33,1],
[8263,42,1],[8263,45,1],[8264,24,1],[8264,38,1],[8264,50,1],
[8265,15,1],[8265,55,1],[8266,13,1],[8266,26,2],[8266,39,1],
[8266,55,2],[8267,2,3],[8267,3,3],[8267,5,1],[8267,8,1],[8267,21,2],
[8267,22,1],[8267,23,2],[8267,24,1],[8267,26,1],[8267,27,1],
[8267,33,4],[8267,35,5],[8267,40,1],[8267,47,2],[8267,50,1],

[8268,11,1],[8268,53,1],[8269,22,1],[8269,26,1],[8269,50,1],
[8270,5,1],[8270,23,1],[8270,26,2],[8270,35,1],[8270,38,1],
[8270,47,1],[8270,48,1],[8270,56,5],[8270,68,1],[8270,76,1],
[8271,7,1],[8271,24,1],[8272,0,2],[8272,1,92],[8272,5,1],[8272,8,1],
[8272,12,4],[8272,13,5],[8272,15,2],[8272,17,2],[8272,20,1],
[8272,23,1],[8272,29,5],[8272,33,1],[8272,34,1],[8272,36,2],
[8272,39,5],[8272,53,1],[8272,55,1],[8272,57,1],[8272,59,1],
[8272,65,1],[8273,3,1],[8273,35,1],[8274,53,1],[8274,55,1],[8275,2,3],
[8276,21,1],[8276,22,1],[8276,26,1],[8276,31,2],[8276,47,1],
[8276,52,1],[8277,15,1],[8277,55,1],[8278,1,1],[8278,15,1],[8279,9,1],
[8279,10,1],[8280,4,1],[8280,24,1],[8280,31,1],[8280,33,1],
[8281,10,1],[8281,30,1],[8282,23,1],[8282,54,1],[8282,68,55],
[8282,70,1],[8282,72,3],[8282,76,1],[8283,32,3],[8284,28,1],
[8284,66,1],[8285,18,1],[8285,28,1],[8285,31,1],[8285,44,1],
[8285,72,1],[8285,76,1],[8286,1,1],[8286,8,1],[8286,23,1],[8286,39,1],
[8286,57,1],[8287,1,3],[8287,13,1],[8287,21,3],[8287,22,3],
[8287,23,6],[8287,24,6],[8287,25,2],[8287,27,1],[8287,28,1],
[8287,31,5],[8287,33,1],[8287,42,4],[8287,48,1],[8287,56,1],
[8287,59,1],[8287,73,1],[8288,26,1],[8288,33,1],[8289,22,4],
[8289,23,2],[8289,24,3],[8289,25,1],[8289,31,2],[8289,32,2],
[8289,42,1],[8290,28,1],[8290,44,1],[8291,2,1],[8291,40,1],
[8291,42,1],[8292,1,2],[8292,3,2],[8292,13,4],[8292,23,1],[8292,24,3],
[8292,26,1],[8292,28,4],[8292,32,1],[8292,33,1],[8292,34,1],
[8292,42,1],[8293,20,1],[8293,23,6],[8293,26,2],[8293,27,1],
[8293,28,2],[8293,30,2],[8293,31,2],[8293,32,1],[8293,33,1],
[8293,37,1],[8293,38,1],[8293,42,1],[8293,43,1],[8293,47,1],
[8293,60,1],[8294,15,3],[8294,55,4],[8295,3,17],[8295,25,1],
[8295,76,1],[8296,2,2],[8296,23,1],[8296,27,2],[8297,24,1],
[8297,28,2],[8297,51,1],[8297,70,2],[8297,76,1],[8298,30,2],
[8298,32,18],[8298,35,3],[8298,77,1],[8299,8,1],[8299,9,5],
[8299,28,4],[8299,74,2],[8300,25,2],[8300,33,1],[8301,53,2],
[8302,1,1],[8302,39,7],[8303,3,1],[8303,5,1],[8303,41,1],[8303,47,1],
[8304,3,9],[8304,10,1],[8304,22,2],[8304,23,1],[8304,24,6],
[8304,25,2],[8304,27,8],[8304,35,5],[8304,40,4],[8304,42,7],
[8304,56,1],[8305,2,1],[8305,23,1],[8305,25,1],[8305,31,3],
[8305,48,1],[8306,19,1],[8306,20,1],[8306,30,1],[8306,31,5],
[8306,32,1],[8306,36,1],[8306,42,2],[8307,2,1],[8307,24,1],
[8307,31,1],[8308,50,1],[8308,53,1],[8309,8,1],[8309,9,2],[8309,24,1],
[8310,27,1],[8310,60,1],[8311,28,1],[8311,68,2],[8312,2,17],
[8312,22,1],[8312,23,1],[8312,25,1],[8312,31,2],[8312,35,1],
[8312,47,1],[8312,65,1],[8312,68,2],[8312,74,1],[8313,17,1],
[8313,22,1],[8313,27,1],[8313,31,1],[8314,3,3],[8314,24,3],
[8314,32,1],[8314,43,1],[8315,1,7],[8315,7,1],[8315,8,1],[8315,9,9],
[8315,10,6],[8315,11,6],[8315,12,4],[8315,13,6],[8315,15,7],
[8315,16,4],[8315,17,2],[8315,18,1],[8315,19,8],[8315,20,4],
[8315,23,1],[8315,27,1],[8315,28,3],[8315,29,1],[8315,30,1],
[8315,31,3],[8315,34,4],[8315,37,3],[8315,39,2],[8315,41,9],
[8315,45,9],[8315,46,3],[8315,55,5],[8315,57,2],[8315,58,1],
[8315,59,4],[8315,62,1],[8315,65,3],[8315,66,1],[8315,67,1],
[8315,70,1],[8315,71,5],[8315,73,3],[8315,77,1],[8315,78,3],

[8316,1,54],[8316,3,1],[8316,4,2],[8316,5,1],[8316,7,12],[8316,8,4],
[8316,9,4],[8316,10,2],[8316,11,2],[8316,12,7],[8316,13,49],
[8316,15,10],[8316,16,1],[8316,17,4],[8316,19,3],[8316,20,11],
[8316,21,1],[8316,22,4],[8316,23,16],[8316,24,4],[8316,25,14],
[8316,26,11],[8316,27,7],[8316,28,11],[8316,29,10],[8316,30,1],
[8316,33,54],[8316,34,3],[8316,35,10],[8316,36,1],[8316,37,2],
[8316,39,1],[8316,42,18],[8316,43,10],[8316,44,3],[8316,45,2],
[8316,46,2],[8316,47,5],[8316,50,7],[8316,53,4],[8316,55,4],
[8316,56,2],[8316,57,2],[8316,59,2],[8316,60,2],[8316,61,1],
[8316,62,88],[8316,63,1],[8316,64,1],[8316,65,3],[8316,66,5],
[8316,67,1],[8316,68,3],[8316,69,3],[8316,74,2],[8316,75,3],
[8316,76,2],[8316,77,10],[8316,78,1],[8317,0,6],[8317,1,13],
[8317,7,2],[8317,9,11],[8317,10,9],[8317,11,3],[8317,12,8],
[8317,13,4],[8317,14,2],[8317,15,5],[8317,16,5],[8317,17,5],
[8317,18,5],[8317,19,6],[8317,20,11],[8317,21,1],[8317,24,2],
[8317,29,4],[8317,30,5],[8317,34,3],[8317,36,2],[8317,37,1],
[8317,39,4],[8317,41,3],[8317,45,7],[8317,46,1],[8317,49,1],
[8317,53,4],[8317,54,4],[8317,55,11],[8317,57,3],[8317,58,3],
[8317,59,3],[8317,60,1],[8317,64,2],[8317,65,7],[8317,67,3],
[8317,70,1],[8317,71,1],[8317,73,2],[8317,77,7],[8318,20,2],
[8318,47,1],[8318,59,1],[8319,15,5],[8319,55,2],[8320,22,1],
[8320,23,2],[8320,24,1],[8320,27,2],[8321,26,2],[8322,7,2],
[8323,27,1],[8323,33,1],[8324,28,1],[8324,66,1],[8325,22,1],
[8325,26,1],[8325,28,2],[8325,31,2],[8325,33,1],[8325,43,2],
[8326,32,2],[8327,2,2],[8327,34,1],[8327,47,1],[8328,26,1],
[8328,28,1],[8329,22,1],[8329,27,1],[8330,16,1],[8330,54,1],
[8331,2,11],[8331,35,1],[8331,70,1],[8331,74,1],[8332,1,1],
[8332,10,1],[8333,22,1],[8333,24,1],[8333,42,1],[8334,2,1],
[8334,28,1],[8335,3,1],[8335,9,1],[8335,20,1],[8335,21,2],[8335,22,3],
[8335,28,8],[8335,35,1],[8335,43,1],[8336,28,1],[8336,35,1],
[8337,37,1],[8337,41,1],[8338,25,1],[8338,56,1],[8339,27,2],
[8339,37,1],[8339,78,1],[8340,27,2],[8340,28,1],[8341,27,1],
[8341,35,2],[8341,59,1],[8342,8,1],[8342,19,1],[8342,22,2],
[8342,24,1],[8343,3,1],[8343,23,1],[8343,28,10],[8343,31,1],
[8343,42,1],[8343,43,1],[8344,0,1],[8344,30,6],[8345,2,2],[8345,3,1],
[8346,2,1],[8346,32,1],[8346,47,1],[8346,50,1],[8347,31,4],
[8347,63,1],[8348,3,1],[8348,23,1],[8348,27,2],[8348,31,1],
[8348,35,1],[8348,46,1],[8349,15,1],[8349,55,2],[8350,50,1],
[8350,57,1],[8351,1,2],[8351,3,1],[8351,23,1],[8351,24,1],[8351,32,1],
[8351,42,1],[8351,62,1],[8352,1,1],[8352,8,2],[8352,15,1],[8353,24,2],
[8354,2,1],[8354,31,2],[8355,2,1],[8355,39,1],[8356,69,1],[8356,70,1],
[8357,0,3],[8357,1,4],[8357,2,2],[8357,3,1],[8357,4,2],[8357,5,14],
[8357,6,6],[8357,8,4],[8357,9,1],[8357,10,4],[8357,11,1],[8357,12,3],
[8357,13,28],[8357,15,15],[8357,16,1],[8357,17,1],[8357,18,1],
[8357,19,1],[8357,20,20],[8357,21,7],[8357,22,11],[8357,23,7],
[8357,24,13],[8357,27,8],[8357,28,10],[8357,29,1],[8357,30,2],
[8357,31,3],[8357,32,4],[8357,33,16],[8357,35,9],[8357,36,2],
[8357,38,2],[8357,39,2],[8357,40,2],[8357,42,3],[8357,43,4],
[8357,44,2],[8357,47,6],[8357,48,2],[8357,49,1],[8357,51,1],
[8357,52,3],[8357,55,3],[8357,56,2],[8357,57,1],[8357,58,1],

[8357,59,4],[8357,61,1],[8357,65,2],[8357,66,2],[8357,68,2],
[8357,69,1],[8357,70,9],[8357,71,1],[8357,72,2],[8357,73,2],
[8357,74,11],[8357,75,2],[8357,76,2],[8357,77,15],[8357,79,1],
[8358,28,1],[8358,33,3],[8358,35,1],[8358,51,2],[8359,12,1],
[8359,23,1],[8359,30,1],[8360,22,1],[8360,24,1],[8360,26,1],
[8360,28,1],[8361,2,1],[8361,50,1],[8362,39,3],[8362,42,1],
[8363,17,1],[8363,45,1],[8364,15,1],[8364,28,5],[8365,23,1],
[8365,25,1],[8366,12,1],[8366,29,1],[8366,57,1],[8367,22,1],
[8367,35,1],[8368,2,1],[8368,28,1],[8369,44,1],[8369,50,1],
[8370,32,1],[8370,75,1],[8371,2,1],[8371,27,1],[8371,68,1],
[8371,70,1],[8372,22,1],[8372,24,5],[8372,25,1],[8372,27,1],
[8372,28,4],[8372,42,1],[8372,47,2],[8372,70,1],[8372,72,1],
[8373,32,5],[8374,4,2],[8374,50,1],[8374,55,1],[8375,5,1],[8375,24,1],
[8375,25,2],[8375,27,2],[8376,9,2],[8376,10,3],[8376,11,1],
[8376,17,1],[8376,18,1],[8376,30,1],[8376,37,1],[8376,65,3],
[8376,71,1],[8376,77,1],[8377,0,1],[8377,9,1],[8377,17,1],[8377,23,5],
[8377,27,5],[8377,41,1],[8377,42,1],[8378,40,1],[8378,42,1],
[8378,71,1],[8379,0,1],[8379,2,32],[8379,20,1],[8379,30,1],
[8379,31,2],[8379,33,1],[8380,4,10],[8380,15,1],[8380,17,10],
[8380,19,1],[8380,28,2],[8380,35,1],[8380,38,1],[8380,44,1],
[8380,45,1],[8380,50,6],[8380,53,5],[8380,55,1],[8380,62,1],
[8380,65,1],[8380,66,3],[8380,73,1],[8380,74,1],[8381,8,1],
[8381,17,1],[8382,20,1],[8382,28,1],[8383,2,1],[8383,5,1],[8383,23,1],
[8383,27,1],[8383,42,1],[8384,17,1],[8384,53,1],[8384,55,3],
[8384,64,1],[8385,25,2],[8386,21,1],[8386,22,1],[8386,27,1],
[8386,35,1],[8386,66,1],[8386,76,1],[8387,25,2],[8387,47,1],
[8388,23,2],[8389,2,1],[8389,13,1],[8389,51,1],[8390,21,2],
[8390,23,1],[8390,25,1],[8390,26,2],[8390,30,1],[8390,38,1],
[8390,39,1],[8390,48,2],[8391,2,1],[8391,28,2],[8392,2,1],[8392,3,1],
[8392,24,2],[8392,27,1],[8392,34,1],[8392,63,1],[8392,68,1],
[8393,15,2],[8394,9,1],[8394,28,4],[8394,74,1],[8394,76,1],[8395,2,2],
[8396,5,1],[8396,28,1],[8397,5,1],[8397,63,8],[8397,74,4],[8397,75,1],
[8397,76,8],[8398,28,1],[8398,35,1],[8399,27,3],[8399,42,1],
[8399,67,1],[8400,1,1],[8400,2,6],[8400,3,1],[8400,4,11],[8400,5,2],
[8400,6,3],[8400,15,5],[8400,22,5],[8400,23,1],[8400,24,1],
[8400,25,1],[8400,26,1],[8400,27,9],[8400,28,5],[8400,29,1],
[8400,30,3],[8400,31,1],[8400,32,1],[8400,35,10],[8400,39,2],
[8400,40,2],[8400,43,2],[8400,47,2],[8400,48,1],[8400,50,17],
[8400,51,3],[8400,55,2],[8400,56,8],[8400,62,1],[8400,66,1],
[8400,67,1],[8400,68,1],[8400,70,1],[8400,74,8],[8400,76,1],
[8401,2,1],[8401,3,1],[8401,4,8],[8401,5,1],[8401,15,24],[8401,17,4],
[8401,20,1],[8401,21,1],[8401,22,4],[8401,23,2],[8401,24,1],
[8401,25,3],[8401,26,2],[8401,27,1],[8401,28,33],[8401,31,3],
[8401,32,3],[8401,35,2],[8401,38,5],[8401,40,2],[8401,44,36],
[8401,47,2],[8401,48,1],[8401,50,14],[8401,51,1],[8401,53,5],
[8401,55,20],[8401,56,1],[8401,66,33],[8401,68,1],[8401,72,1],
[8401,76,3],[8402,28,1],[8402,40,1],[8402,44,1],[8402,62,1],
[8403,32,1],[8403,40,1],[8403,70,1],[8404,28,2],[8404,32,1],
[8404,33,1],[8404,38,2],[8404,50,1],[8404,51,1],[8404,52,1],
[8404,62,1],[8404,63,1],[8404,70,1],[8405,2,1],[8405,3,1],[8405,22,2],

[8405,23,11],[8405,24,6],[8405,25,2],[8405,26,3],[8405,27,1],
[8405,28,2],[8405,31,2],[8405,32,2],[8405,33,1],[8405,35,2],
[8405,39,3],[8405,43,1],[8405,52,1],[8405,53,1],[8405,62,2],
[8406,7,3],[8407,33,2],[8408,5,1],[8408,9,1],[8408,21,2],[8408,24,2],
[8408,25,6],[8408,26,4],[8408,28,16],[8408,29,1],[8408,31,1],
[8408,32,1],[8408,39,2],[8408,40,1],[8408,48,2],[8408,52,4],
[8408,59,1],[8408,60,1],[8408,68,1],[8409,3,1],[8409,26,2],[8410,6,1],
[8410,27,1],[8410,47,1],[8410,56,2],[8411,32,1],[8411,47,1],
[8412,15,2],[8412,16,1],[8412,55,1],[8413,1,2],[8413,20,1],
[8413,33,1],[8413,57,1],[8414,27,2],[8415,75,4],[8415,76,1],
[8416,3,1],[8416,24,1],[8416,33,2],[8416,56,1],[8417,39,1],
[8417,43,1],[8418,13,1],[8418,17,1],[8419,28,2],[8420,4,1],[8420,8,1],
[8420,12,1],[8420,21,1],[8420,23,3],[8420,24,1],[8420,25,10],
[8420,26,1],[8420,27,1],[8420,28,2],[8420,30,2],[8420,31,1],
[8420,32,4],[8420,39,1],[8420,43,2],[8420,61,1],[8420,73,3],
[8421,5,1],[8421,26,1],[8422,9,1],[8422,10,1],[8422,23,3],[8423,28,1],
[8423,47,1],[8423,66,1],[8424,28,2],[8425,4,1],[8425,23,1],
[8425,27,1],[8425,28,3],[8425,31,1],[8425,50,1],[8425,66,2],
[8425,72,1],[8425,76,1],[8426,32,1],[8426,53,2],[8426,55,1],
[8426,56,1],[8427,23,2],[8427,27,1],[8427,42,1],[8428,22,2],
[8428,24,1],[8428,28,2],[8429,2,1],[8429,20,1],[8429,23,1],
[8429,27,1],[8429,28,1],[8429,31,1],[8429,40,1],[8429,61,1],
[8430,27,3],[8430,28,1],[8430,43,2],[8430,69,1],[8431,4,1],
[8431,15,1],[8432,28,23],[8433,28,1],[8433,50,1],[8434,19,1],
[8434,72,1],[8435,2,2],[8435,3,1],[8435,30,2],[8435,33,1],[8436,30,1],
[8436,33,1],[8437,22,1],[8437,33,1],[8438,21,1],[8438,42,1],
[8438,43,1],[8438,62,1],[8439,2,1],[8439,3,5],[8439,9,1],[8439,11,1],
[8439,20,2],[8439,21,1],[8439,22,1],[8439,23,3],[8439,24,7],
[8439,25,1],[8439,26,2],[8439,27,5],[8439,28,3],[8439,29,1],
[8439,30,1],[8439,32,3],[8439,35,3],[8439,39,1],[8439,42,2],
[8439,43,3],[8439,47,2],[8439,55,1],[8439,62,1],[8439,69,1],
[8440,10,1],[8440,20,3],[8440,22,1],[8440,28,1],[8440,34,1],
[8440,35,1],[8440,38,2],[8441,1,4],[8441,2,1],[8441,5,1],[8441,13,2],
[8441,46,1],[8441,73,1],[8441,77,2],[8442,19,1],[8442,31,1],
[8442,33,1],[8442,40,1],[8442,43,1],[8442,48,4],[8442,63,1],
[8443,28,4],[8443,42,1],[8443,49,1],[8443,72,1],[8444,0,54],
[8444,1,3],[8444,7,25],[8444,9,1],[8444,10,1],[8444,11,1],[8444,12,4],
[8444,13,1],[8444,14,1],[8444,15,1],[8444,16,2],[8444,18,1],
[8444,19,2],[8444,31,4],[8444,37,3],[8444,39,1],[8444,45,1],
[8444,46,2],[8444,53,1],[8444,54,2],[8444,55,2],[8444,57,2],
[8444,65,2],[8444,67,1],[8444,71,1],[8444,73,7],[8444,77,2],
[8445,4,1],[8445,27,1],[8446,33,4],[8447,3,1],[8447,27,2],[8447,28,1],
[8447,33,2],[8447,47,1],[8447,56,3],[8447,62,1],[8447,74,1],
[8448,13,1],[8448,20,1],[8448,63,1],[8449,20,1],[8449,30,4],
[8449,52,5],[8450,22,1],[8450,28,1],[8451,4,1],[8451,23,1],
[8451,38,1],[8451,47,1],[8451,50,1],[8451,67,1],[8452,38,2],
[8453,33,1],[8453,38,1],[8454,5,2],[8455,16,1],[8455,44,1],
[8455,53,1],[8456,65,1],[8456,73,1],[8457,32,5],[8457,35,1],
[8458,6,2],[8459,1,1],[8459,12,1],[8459,31,3],[8459,39,1],[8459,51,1],
[8460,22,1],[8460,23,3],[8460,32,1],[8461,3,2],[8461,8,1],[8461,20,3],

[8461,23,2],[8461,27,3],[8461,28,3],[8461,31,5],[8461,33,3],
[8461,37,1],[8461,39,1],[8461,42,2],[8461,43,1],[8461,45,1],
[8461,47,1],[8461,54,1],[8461,63,1],[8462,9,2],[8462,10,1],
[8462,11,1],[8462,12,2],[8462,15,1],[8462,16,5],[8462,19,1],
[8462,20,1],[8462,28,2],[8462,29,1],[8462,34,3],[8462,37,1],
[8462,39,2],[8462,45,1],[8462,46,4],[8462,49,2],[8462,54,1],
[8462,57,1],[8462,58,1],[8462,59,1],[8462,60,1],[8462,64,3],
[8462,65,1],[8462,67,1],[8462,71,1],[8462,73,1],[8462,74,1],
[8462,76,1],[8462,77,1],[8462,78,1],[8463,2,186],[8463,28,6],
[8463,31,1],[8463,38,1],[8463,50,1],[8463,52,1],[8463,62,2],
[8463,70,1],[8463,74,1],[8464,19,1],[8464,28,1],[8464,30,12],
[8464,33,1],[8464,37,1],[8464,57,1],[8464,65,1],[8465,5,4],
[8465,20,1],[8465,24,15],[8465,27,1],[8465,28,1],[8465,32,1],
[8465,35,1],[8465,63,1],[8466,27,2],[8466,28,1],[8467,8,1],
[8467,19,1],[8467,37,1],[8467,54,1],[8467,57,1],[8467,65,3],
[8468,9,1],[8468,20,1],[8468,23,1],[8468,24,2],[8468,26,2],
[8468,28,2],[8468,35,2],[8468,42,1],[8468,43,1],[8469,9,1],
[8469,14,1],[8469,28,1],[8469,45,1],[8469,55,1],[8469,71,1],
[8470,1,1],[8470,13,1],[8470,14,1],[8470,16,1],[8470,17,1],
[8470,19,2],[8470,45,1],[8470,49,1],[8470,65,1],[8470,77,1],
[8471,6,2],[8471,21,2],[8471,24,1],[8471,26,1],[8471,28,1],
[8471,47,1],[8471,68,1],[8472,1,1],[8472,20,1],[8472,23,1],
[8472,27,9],[8472,32,2],[8472,34,2],[8472,65,1],[8473,27,3],
[8473,28,2],[8473,33,1],[8473,35,2],[8474,1,1],[8474,6,1],[8474,47,1],
[8475,1,1],[8475,3,1],[8475,7,1],[8475,12,1],[8475,15,1],[8475,23,2],
[8475,24,1],[8475,30,1],[8475,31,3],[8475,32,2],[8475,35,2],
[8475,49,1],[8475,69,1],[8476,2,2],[8477,1,1],[8477,3,1],[8477,17,1],
[8477,23,7],[8477,26,1],[8477,28,3],[8477,32,3],[8477,34,1],
[8477,35,3],[8477,38,1],[8477,39,1],[8477,43,1],[8478,3,1],
[8478,62,1],[8479,1,3],[8479,7,1],[8479,15,2],[8480,28,1],[8480,40,1],
[8480,47,1],[8480,69,1],[8481,2,2],[8481,3,2],[8481,8,1],[8481,20,1],
[8481,23,1],[8481,24,4],[8481,25,1],[8481,26,2],[8481,27,17],
[8481,28,4],[8481,31,4],[8481,32,1],[8481,42,1],[8481,78,1],
[8482,28,1],[8482,30,4],[8483,2,26],[8483,24,1],[8484,27,1],
[8484,31,1],[8485,28,1],[8485,43,1],[8486,33,4],[8486,42,1],
[8487,0,1],[8487,23,1],[8487,26,1],[8487,32,1],[8487,36,2],
[8487,43,1],[8488,1,1],[8488,9,1],[8488,10,2],[8488,12,1],[8488,13,1],
[8488,15,2],[8488,17,1],[8488,19,1],[8488,20,3],[8488,29,1],
[8488,30,1],[8488,37,1],[8488,45,1],[8488,46,1],[8488,53,2],
[8488,55,2],[8488,65,2],[8488,67,1],[8488,73,1],[8489,2,4],[8489,3,1],
[8489,5,4],[8489,7,1],[8489,12,1],[8489,13,1],[8489,14,1],[8489,15,5],
[8489,20,5],[8489,24,1],[8489,30,1],[8489,33,1],[8489,35,3],
[8489,37,1],[8489,42,1],[8489,44,1],[8489,55,2],[8489,56,2],
[8489,58,1],[8489,59,1],[8489,60,2],[8489,62,1],[8489,70,1],
[8489,74,2],[8490,5,1],[8490,21,1],[8491,28,1],[8491,42,4],
[8492,23,1],[8492,27,1],[8492,28,1],[8492,32,2],[8492,33,1],
[8492,67,1],[8493,10,4],[8493,16,1],[8493,18,1],[8493,20,1],
[8493,37,1],[8493,58,1],[8493,65,1],[8493,77,1],[8493,78,1],
[8494,3,1],[8494,5,1],[8494,21,2],[8494,23,3],[8494,25,1],[8494,26,1],
[8494,27,3],[8494,28,4],[8494,31,2],[8494,35,1],[8494,42,1],

[8494,43,5],[8494,48,1],[8495,44,2],[8496,25,1],[8496,38,2],
[8496,62,1],[8497,1,1],[8497,9,3],[8497,10,1],[8497,13,1],[8497,17,1],
[8497,19,1],[8497,41,1],[8498,8,1],[8498,20,1],[8498,34,2],
[8498,36,3],[8499,3,1],[8499,5,1],[8499,21,3],[8499,24,2],[8499,27,2],
[8499,32,1],[8499,35,1],[8499,38,1],[8499,40,1],[8499,42,1],
[8499,44,2],[8499,48,1],[8499,66,2],[8499,72,1],[8499,74,6],
[8499,76,1],[8500,39,5],[8500,45,1],[8501,25,2],[8501,31,1],
[8501,43,1],[8502,4,1],[8502,27,2],[8502,28,1],[8503,3,1],[8503,38,1],
[8503,56,1],[8504,17,3],[8504,25,1],[8504,53,1],[8505,12,1],
[8505,73,1],[8506,2,1],[8506,23,2],[8506,24,2],[8506,25,1],
[8506,42,1],[8506,56,4],[8506,68,1],[8507,27,1],[8507,28,1],
[8507,32,1],[8508,6,1],[8508,16,1],[8508,24,19],[8508,25,2],
[8508,27,1],[8508,28,1],[8508,35,1],[8508,41,2],[8508,42,1],
[8508,43,1],[8508,44,2],[8508,46,1],[8508,57,1],[8508,60,1],
[8508,63,1],[8508,65,1],[8508,67,1],[8508,75,1],[8509,21,1],
[8509,28,1],[8510,2,10],[8510,19,1],[8510,23,1],[8510,24,1],
[8510,28,1],[8510,30,2],[8510,31,1],[8510,43,2],[8510,58,1],
[8510,67,3],[8511,15,1],[8511,23,1],[8511,55,1],[8512,7,1],
[8512,54,1],[8513,12,1],[8513,13,1],[8513,18,1],[8513,19,3],
[8513,41,2],[8513,45,3],[8513,54,1],[8513,73,1],[8514,26,2],
[8515,27,1],[8515,72,1],[8516,3,2],[8516,20,1],[8516,28,2],
[8516,47,1],[8516,56,1],[8516,68,1],[8516,74,1],[8517,12,2],
[8517,29,1],[8518,2,1],[8518,17,1],[8518,44,1],[8519,13,2],[8520,3,1],
[8520,26,1],[8520,28,4],[8520,31,5],[8520,33,2],[8520,75,1],
[8521,2,9],[8521,31,1],[8522,37,1],[8522,50,1],[8522,66,1],[8523,2,1],
[8523,27,1],[8523,52,1],[8524,21,4],[8524,22,1],[8525,4,2],
[8525,55,2],[8526,56,3],[8527,27,1],[8527,53,1],[8528,0,2],
[8529,16,1],[8529,67,1],[8530,17,1],[8530,31,1],[8530,42,1],
[8530,44,10],[8530,50,2],[8530,55,1],[8530,66,12],[8531,2,1],
[8531,26,1],[8531,31,1],[8531,53,1],[8531,68,1],[8532,66,2],
[8533,23,1],[8533,31,1],[8533,44,1],[8534,32,10],[8535,21,1],
[8535,35,2],[8535,54,1],[8536,0,4],[8536,1,18],[8536,3,1],[8536,7,2],
[8536,8,3],[8536,9,29],[8536,10,27],[8536,11,13],[8536,12,14],
[8536,13,16],[8536,14,6],[8536,15,22],[8536,16,13],[8536,17,9],
[8536,18,11],[8536,19,23],[8536,20,8],[8536,24,2],[8536,29,9],
[8536,30,2],[8536,34,5],[8536,36,7],[8536,37,10],[8536,39,13],
[8536,41,23],[8536,45,11],[8536,46,13],[8536,49,12],[8536,53,8],
[8536,54,4],[8536,55,21],[8536,57,10],[8536,58,7],[8536,59,21],
[8536,60,3],[8536,64,2],[8536,65,21],[8536,67,13],[8536,71,5],
[8536,73,7],[8536,75,2],[8536,77,8],[8536,78,5],[8537,2,50],
[8537,21,1],[8537,28,26],[8537,52,1],[8537,72,1],[8538,4,3],
[8538,50,1],[8539,5,2],[8539,21,1],[8539,24,1],[8539,26,1],
[8540,35,1],[8540,44,1],[8541,2,5],[8541,28,2],[8542,4,1],[8542,50,1],
[8542,66,1],[8543,8,1],[8543,71,1],[8544,20,1],[8544,31,1],[8545,3,1],
[8545,4,11],[8545,5,1],[8545,9,1],[8545,11,1],[8545,13,2],[8545,15,2],
[8545,19,1],[8545,23,1],[8545,29,2],[8545,34,1],[8545,38,2],
[8545,39,1],[8545,43,1],[8545,44,17],[8545,46,1],[8545,47,1],
[8545,50,24],[8545,55,2],[8545,59,1],[8545,65,1],[8545,66,20],
[8545,68,1],[8545,70,1],[8545,74,1],[8545,78,1],[8546,15,3],
[8547,1,1],[8547,3,1],[8547,5,1],[8547,21,1],[8547,24,1],[8547,26,1],

[8547,28,1],[8547,44,1],[8547,56,1],[8547,70,2],[8547,74,2],
[8547,77,1],[8548,2,2],[8549,5,1],[8549,9,1],[8549,21,2],[8549,22,4],
[8549,23,6],[8549,26,1],[8549,27,3],[8549,31,2],[8549,35,3],
[8549,36,1],[8549,43,1],[8550,23,1],[8550,25,2],[8551,0,1],
[8551,17,1],[8551,29,1],[8551,53,2],[8551,55,1],[8551,62,1],
[8552,1,1],[8552,6,4],[8552,7,1],[8552,22,1],[8552,23,2],[8552,24,1],
[8552,33,1],[8552,34,5],[8552,47,1],[8552,66,1],[8552,68,1],
[8552,71,1],[8553,27,12],[8554,27,1],[8554,28,1],[8555,24,2],
[8555,42,1],[8556,53,1],[8556,55,1],[8557,21,7],[8557,23,1],
[8557,26,6],[8557,31,2],[8557,42,1],[8557,43,5],[8557,52,1],
[8558,33,4],[8559,2,1],[8559,11,1],[8559,20,2],[8559,24,2],
[8559,26,1],[8559,28,1],[8559,31,1],[8559,54,1],[8560,6,1],
[8560,27,1],[8560,28,4],[8560,35,1],[8560,56,1],[8560,74,1],
[8560,76,1],[8561,32,11],[8562,7,1],[8562,67,1],[8563,12,1],
[8563,15,1],[8563,73,1],[8564,63,2],[8564,76,1],[8565,2,3],[8566,0,2],
[8566,1,2],[8566,2,17],[8566,3,9],[8566,5,24],[8566,6,4],[8566,8,1],
[8566,9,8],[8566,10,9],[8566,11,1],[8566,12,196],[8566,13,10],
[8566,14,2],[8566,15,1],[8566,16,5],[8566,17,4],[8566,18,2],
[8566,19,1],[8566,20,16],[8566,21,6],[8566,22,16],[8566,23,27],
[8566,24,32],[8566,25,12],[8566,26,13],[8566,27,19],[8566,28,36],
[8566,29,4],[8566,30,2],[8566,31,5],[8566,32,113],[8566,33,11],
[8566,34,22],[8566,35,14],[8566,36,3],[8566,39,11],[8566,40,12],
[8566,42,18],[8566,43,6],[8566,44,1],[8566,45,3],[8566,46,2],
[8566,47,8],[8566,48,10],[8566,52,1],[8566,53,2],[8566,54,1],
[8566,55,1],[8566,57,1],[8566,58,2],[8566,59,58],[8566,60,2],
[8566,61,12],[8566,62,21],[8566,65,3],[8566,66,1],[8566,67,1],
[8566,68,12],[8566,69,4],[8566,70,6],[8566,71,1],[8566,72,2],
[8566,74,1],[8566,76,1],[8566,77,3],[8566,78,5],[8567,39,1],
[8567,78,1],[8568,3,1],[8568,20,2],[8568,24,1],[8568,28,1],
[8568,30,1],[8568,32,4],[8568,37,1],[8568,43,1],[8569,22,4],
[8569,24,3],[8569,27,1],[8569,28,1],[8569,56,1],[8570,21,1],
[8570,22,2],[8570,27,2],[8570,31,2],[8570,42,2],[8570,43,3],
[8571,20,1],[8571,27,22],[8571,28,3],[8571,31,4],[8571,34,1],
[8571,70,1],[8571,76,1],[8572,5,1],[8572,26,1],[8572,32,5],
[8573,27,2],[8574,1,2],[8574,12,1],[8574,23,2],[8574,25,20],
[8574,27,2],[8574,31,1],[8574,33,2],[8574,35,1],[8574,39,1],
[8574,43,2],[8574,44,1],[8574,45,1],[8574,50,1],[8575,15,1],
[8575,55,1],[8576,24,1],[8576,25,1],[8576,31,1],[8577,1,2],[8577,2,9],
[8577,3,2],[8577,5,4],[8577,6,2],[8577,13,10],[8577,23,3],[8577,24,2],
[8577,27,3],[8577,28,1],[8577,30,2],[8577,31,2],[8577,32,4],
[8577,33,2],[8577,34,1],[8577,35,3],[8577,40,1],[8577,42,5],
[8577,43,1],[8577,44,2],[8577,47,1],[8577,50,3],[8577,52,1],
[8577,53,1],[8577,59,1],[8577,62,1],[8577,63,2],[8577,66,3],
[8577,68,1],[8577,69,1],[8577,70,2],[8577,74,1],[8577,75,1],
[8578,1,15],[8578,7,1],[8578,9,1],[8578,12,5],[8578,13,1],
[8578,29,12],[8578,34,2],[8578,37,1],[8579,27,5],[8579,59,1],
[8580,27,1],[8580,42,1],[8581,3,1],[8581,22,2],[8581,23,4],
[8581,24,2],[8581,26,2],[8581,27,5],[8581,28,1],[8581,62,1],
[8582,0,1],[8582,4,1],[8582,7,1],[8582,9,4],[8582,12,1],[8582,14,2],
[8582,15,1],[8582,16,1],[8582,18,1],[8582,19,2],[8582,26,1],

[8582,30,1],[8582,38,2],[8582,39,1],[8582,44,1],[8582,49,2],
[8582,50,2],[8582,53,1],[8582,57,1],[8582,60,1],[8582,66,1],
[8582,70,1],[8582,71,2],[8582,75,1],[8583,20,2],[8583,23,2],
[8584,35,1],[8584,40,1],[8585,38,1],[8585,42,1],[8586,11,1],
[8586,17,1],[8586,41,1],[8586,60,1],[8587,20,1],[8587,50,1],
[8588,24,1],[8588,28,1],[8589,33,1],[8589,63,1],[8589,69,1],
[8590,4,1],[8590,33,1],[8590,44,1],[8590,50,1],[8590,61,1],
[8590,66,2],[8591,15,1],[8591,42,1],[8592,28,4],[8593,46,1],
[8593,59,1],[8594,41,1],[8594,55,2],[8595,28,1],[8595,33,1],
[8596,31,3],[8596,42,1],[8597,23,2],[8598,28,1],[8598,42,1],
[8599,53,1],[8599,57,1],[8600,3,1],[8600,12,1],[8600,16,1],
[8600,27,1],[8600,28,1],[8600,45,1],[8600,70,1],[8601,20,7],
[8601,22,2],[8601,73,1],[8602,15,1],[8602,55,1],[8603,12,1],
[8603,34,1],[8604,20,1],[8604,39,1],[8604,60,1],[8605,13,1],
[8605,20,1],[8606,21,1],[8606,23,2],[8606,26,1],[8606,28,167],
[8606,50,1],[8606,63,12],[8606,66,1],[8606,74,9],[8606,75,9],
[8606,76,18],[8607,27,4],[8608,4,10],[8608,15,3],[8608,17,8],
[8608,36,1],[8608,39,1],[8608,44,2],[8608,50,7],[8608,53,18],
[8608,55,9],[8608,66,4],[8608,68,1],[8609,15,1],[8609,17,1],
[8609,53,1],[8610,43,2],[8611,20,1],[8611,55,1],[8611,62,1],
[8612,30,2],[8613,9,1],[8613,65,1],[8614,23,1],[8614,38,1],
[8614,56,1],[8615,21,1],[8615,28,1],[8616,28,1],[8616,43,1],
[8616,53,1],[8617,2,1],[8617,28,1],[8618,21,2],[8618,26,3],
[8618,42,1],[8619,0,1],[8619,42,1],[8619,46,1],[8620,5,3],[8620,10,1],
[8620,21,2],[8620,23,2],[8620,25,5],[8620,32,5],[8620,35,1],
[8620,38,1],[8620,46,1],[8620,50,1],[8621,1,2],[8621,29,1],
[8622,40,3],[8623,2,1],[8623,33,1],[8623,47,1],[8624,3,2],[8624,5,1],
[8624,6,2],[8624,8,1],[8624,21,1],[8624,22,1],[8624,24,4],[8624,31,1],
[8624,33,4],[8624,38,1],[8624,40,1],[8624,43,9],[8624,51,1],
[8624,53,1],[8624,62,1],[8625,10,1],[8625,15,1],[8625,41,1],
[8625,53,1],[8625,74,1],[8626,27,2],[8627,30,1],[8627,56,1],
[8628,30,1],[8628,53,1],[8629,21,1],[8629,28,1],[8629,59,1],
[8629,62,1],[8630,5,1],[8630,23,1],[8630,24,1],[8630,27,3],
[8630,33,2],[8630,54,1],[8631,15,1],[8631,72,1],[8632,0,1],[8632,1,8],
[8632,2,1],[8632,3,1],[8632,9,1],[8632,12,2],[8632,13,1],[8632,15,1],
[8632,19,1],[8632,20,27],[8632,21,2],[8632,22,13],[8632,23,7],
[8632,24,5],[8632,25,1],[8632,26,3],[8632,27,4],[8632,30,1],
[8632,31,1],[8632,33,1],[8632,35,3],[8632,37,1],[8632,38,2],
[8632,43,2],[8632,45,1],[8632,46,1],[8632,47,1],[8632,48,3],
[8632,55,1],[8632,58,1],[8632,59,4],[8632,65,1],[8632,67,2],
[8633,3,2],[8633,13,2],[8633,20,2],[8633,62,2],[8634,2,4],[8635,2,1],
[8635,31,1],[8635,43,1],[8636,1,1],[8636,2,4],[8636,3,1],[8636,4,1],
[8636,10,1],[8636,12,1],[8636,17,2],[8636,19,1],[8636,20,3],
[8636,23,1],[8636,25,1],[8636,27,1],[8636,30,1],[8636,33,1],
[8636,34,1],[8636,38,1],[8636,43,1],[8636,47,1],[8636,51,1],
[8636,56,1],[8636,75,1],[8637,27,2],[8638,28,1],[8638,69,1],
[8638,76,1],[8639,16,1],[8639,28,1],[8639,39,1],[8639,76,2],
[8640,0,2],[8640,1,17],[8640,4,1],[8640,7,3],[8640,8,1],[8640,9,10],
[8640,10,18],[8640,11,1],[8640,12,13],[8640,13,5],[8640,14,4],
[8640,15,8],[8640,16,10],[8640,17,9],[8640,18,6],[8640,19,14],

[8640,20,2],[8640,29,5],[8640,30,4],[8640,34,7],[8640,37,5],
[8640,39,3],[8640,41,6],[8640,43,1],[8640,45,9],[8640,46,3],
[8640,49,6],[8640,53,9],[8640,54,7],[8640,55,10],[8640,57,7],
[8640,58,6],[8640,59,8],[8640,64,4],[8640,65,15],[8640,67,13],
[8640,73,1],[8640,77,7],[8640,78,3],[8641,22,1],[8641,25,1],
[8642,22,1],[8642,62,1],[8643,28,1],[8643,62,1],[8644,1,3],[8644,2,2],
[8644,28,1],[8644,77,1],[8645,24,1],[8645,26,1],[8645,68,1],
[8646,9,1],[8646,17,1],[8647,3,2],[8647,14,1],[8647,47,1],[8648,1,1],
[8648,6,1],[8648,10,1],[8648,12,1],[8648,20,4],[8648,23,1],
[8648,26,2],[8648,36,2],[8648,39,1],[8648,42,1],[8648,45,1],
[8648,51,6],[8648,53,1],[8649,20,1],[8649,22,1],[8649,31,2],
[8650,17,1],[8650,53,1],[8651,26,1],[8651,27,1],[8651,28,5],
[8651,38,1],[8651,42,1],[8651,43,1],[8651,53,1],[8652,66,2],
[8653,9,1],[8653,30,3],[8654,2,3],[8654,3,1],[8654,5,1],[8654,28,2],
[8654,34,1],[8654,46,1],[8655,22,1],[8655,28,1],[8655,73,1],
[8656,2,1],[8656,3,1],[8656,22,1],[8656,28,1],[8656,30,1],[8656,31,1],
[8656,35,1],[8656,39,2],[8656,43,1],[8656,56,1],[8657,43,3],
[8657,47,1],[8658,2,4],[8658,20,1],[8658,21,1],[8658,25,11],
[8658,27,3],[8658,28,4],[8658,31,4],[8658,32,6],[8658,35,2],
[8658,42,5],[8658,62,1],[8658,72,2],[8659,27,1],[8659,32,19],
[8659,42,4],[8659,43,2],[8660,23,2],[8661,2,2],[8661,28,1],
[8662,25,1],[8662,42,1],[8663,2,1],[8663,43,1],[8664,15,2],
[8664,17,2],[8664,53,3],[8664,55,2],[8665,3,1],[8665,4,1],[8665,22,3],
[8665,23,21],[8665,26,1],[8665,27,1],[8665,50,3],[8665,72,1],
[8666,28,1],[8666,75,1],[8667,51,3],[8668,32,2],[8669,8,2],
[8669,20,1],[8669,23,1],[8669,30,1],[8669,31,2],[8670,2,9],[8670,3,1],
[8670,35,1],[8671,33,2],[8672,20,3],[8673,6,1],[8673,22,1],
[8673,23,4],[8673,25,2],[8673,26,1],[8673,27,11],[8673,28,1],
[8673,31,4],[8673,35,2],[8673,43,2],[8674,31,1],[8674,44,2],
[8674,66,2],[8675,29,2],[8676,23,1],[8676,26,1],[8676,27,6],
[8676,28,1],[8677,23,2],[8677,26,1],[8677,59,2],[8678,13,1],
[8678,20,2],[8678,22,1],[8678,27,2],[8678,32,1],[8678,37,1],
[8678,42,1],[8678,43,1],[8678,48,1],[8679,9,1],[8679,10,1],
[8679,19,1],[8680,23,1],[8680,28,1],[8680,70,1],[8681,15,1],
[8681,27,1],[8682,20,1],[8682,21,7],[8682,22,1],[8682,24,1],
[8682,25,1],[8682,26,1],[8682,27,3],[8682,31,2],[8682,33,3],
[8682,35,1],[8682,42,1],[8682,44,1],[8682,47,1],[8682,74,1],
[8683,1,1],[8683,13,1],[8684,24,1],[8684,27,2],[8684,28,2],[8685,2,3],
[8686,28,3],[8687,12,1],[8687,20,1],[8688,0,1],[8688,9,1],[8688,39,1],
[8689,3,1],[8689,26,1],[8689,28,1],[8689,47,1],[8690,23,1],
[8690,59,1],[8691,24,1],[8691,70,1],[8692,17,1],[8692,22,1],
[8692,23,2],[8692,24,5],[8692,25,1],[8692,27,14],[8692,28,1],
[8692,30,1],[8692,31,1],[8692,32,3],[8692,38,1],[8692,43,1],
[8692,47,1],[8692,48,1],[8692,62,3],[8693,2,10],[8693,31,1],
[8694,28,4],[8694,42,1],[8695,27,1],[8695,42,1],[8695,60,1],
[8696,1,5],[8696,5,1],[8696,7,7],[8696,11,7],[8696,15,1],[8696,17,1],
[8696,20,1],[8696,25,1],[8696,27,1],[8696,28,9],[8696,29,1],
[8696,36,2],[8696,38,1],[8696,47,1],[8696,54,1],[8696,56,2],
[8696,63,1],[8696,74,8],[8696,76,3],[8696,78,1],[8697,1,34],
[8697,8,1],[8697,12,9],[8697,13,3],[8697,17,1],[8697,29,4],

[8697,36,1],[8697,39,3],[8697,55,1],[8698,13,1],[8698,32,1],
[8698,37,4],[8698,57,1],[8699,1,4],[8699,3,2],[8699,9,4],[8699,10,1],
[8699,13,1],[8699,14,1],[8699,16,1],[8699,19,1],[8699,24,1],
[8699,26,1],[8699,28,4],[8699,35,1],[8699,40,1],[8699,41,3],
[8699,45,1],[8699,50,1],[8699,57,1],[8699,58,1],[8699,63,2],
[8699,65,1],[8699,74,5],[8699,75,1],[8699,76,4],[8700,2,1],
[8700,20,1],[8701,3,1],[8701,5,1],[8701,25,1],[8701,26,1],[8701,28,2],
[8701,31,5],[8701,32,2],[8701,38,6],[8701,42,4],[8702,1,2],[8702,3,1],
[8702,8,1],[8702,23,1],[8702,27,1],[8702,28,2],[8702,32,4],
[8702,62,1],[8703,3,1],[8703,8,1],[8703,23,2],[8703,24,2],[8703,27,3],
[8703,31,2],[8703,32,1],[8703,35,2],[8703,43,2],[8704,32,1],
[8704,39,1],[8705,3,1],[8705,30,1],[8705,39,2],[8706,2,2],[8706,15,1],
[8706,20,3],[8706,22,1],[8706,30,1],[8706,34,1],[8706,37,1],
[8707,3,1],[8707,23,1],[8707,26,1],[8707,28,1],[8707,53,1],
[8707,57,1],[8707,72,1],[8708,8,1],[8708,13,1],[8708,15,7],
[8708,17,1],[8708,19,1],[8708,23,1],[8708,53,2],[8708,55,1],
[8708,57,1],[8708,77,1],[8709,4,1],[8709,44,3],[8710,1,3],[8710,67,1],
[8711,11,2],[8711,20,1],[8711,28,1],[8711,77,1],[8712,40,1],
[8712,69,1],[8712,70,2],[8712,72,1],[8713,2,1],[8713,23,1],
[8714,28,1],[8714,29,1],[8714,50,1],[8714,74,1],[8715,23,2],
[8716,2,1],[8716,22,2],[8716,33,1],[8716,35,1],[8716,43,1],
[8716,62,1],[8717,1,1],[8717,28,1],[8717,59,1],[8718,2,1],[8718,4,49],
[8718,5,4],[8718,28,6],[8718,32,2],[8718,42,1],[8718,44,2],
[8718,50,46],[8718,52,1],[8718,62,2],[8718,63,1],[8718,66,1],
[8718,68,1],[8718,70,1],[8719,30,1],[8719,65,1],[8719,70,1],
[8720,3,1],[8720,22,2],[8720,26,1],[8720,27,2],[8720,35,1],[8721,2,1],
[8721,21,1],[8721,55,1],[8721,63,1],[8721,69,1],[8722,22,1],
[8722,23,1],[8722,27,4],[8722,28,2],[8722,35,2],[8723,27,1],
[8723,28,13],[8724,4,1],[8724,22,1],[8724,28,1],[8725,25,1],
[8725,28,1],[8726,23,1],[8726,32,2],[8727,5,1],[8727,6,1],[8727,31,1],
[8727,32,1],[8727,40,1],[8727,50,1],[8727,72,2],[8727,74,3],
[8727,76,2],[8728,29,1],[8728,31,1],[8729,15,1],[8729,53,1],
[8730,15,1],[8730,55,1],[8731,11,1],[8731,28,1],[8731,70,1],
[8731,76,1],[8732,21,1],[8732,28,1],[8733,16,1],[8733,60,1],
[8733,65,1],[8733,67,1],[8734,23,1],[8734,27,2],[8734,43,1],
[8734,60,1],[8735,6,1],[8735,21,1],[8735,28,1],[8736,27,2],[8737,2,9],
[8737,47,1],[8737,72,1],[8738,22,1],[8738,23,1],[8738,24,1],
[8738,28,1],[8738,50,1],[8739,21,2],[8740,23,1],[8740,27,7],
[8741,8,1],[8741,15,1],[8742,3,1],[8742,9,1],[8742,26,1],[8742,28,1],
[8743,4,1],[8743,53,1],[8744,6,2],[8744,31,2],[8745,17,1],[8745,55,1],
[8745,57,1],[8746,24,1],[8746,33,1],[8747,1,1],[8747,5,1],[8747,6,1],
[8747,15,1],[8747,22,2],[8747,24,2],[8747,43,1],[8747,56,1],
[8748,24,1],[8748,32,1],[8748,48,1],[8749,2,3],[8749,28,1],[8750,3,6],
[8750,12,1],[8750,26,1],[8750,57,1],[8750,60,1],[8750,75,1],
[8751,27,1],[8751,35,1],[8751,38,1],[8751,40,1],[8751,58,1],
[8751,69,1],[8752,27,2],[8752,28,1],[8752,33,2],[8752,66,1],
[8753,2,9],[8753,22,1],[8753,25,1],[8753,29,1],[8753,43,1],
[8753,47,1],[8753,50,1],[8753,53,1],[8753,55,1],[8754,24,3],
[8755,31,4],[8756,20,1],[8756,38,1],[8756,74,1],[8756,79,1],
[8757,2,3],[8757,3,1],[8757,5,1],[8757,21,9],[8757,23,12],

[8757,25,30],[8757,26,44],[8757,27,1],[8757,28,3],[8757,31,2],
[8757,32,2],[8757,33,50],[8757,39,1],[8757,40,2],[8757,42,6],
[8757,43,31],[8757,44,1],[8757,47,2],[8757,51,1],[8757,63,1],
[8757,70,2],[8757,76,2],[8758,23,1],[8758,28,2],[8758,31,7],
[8759,3,5],[8759,6,1],[8759,21,1],[8759,22,4],[8759,23,2],[8759,24,3],
[8759,27,1],[8759,28,3],[8759,31,1],[8759,32,11],[8759,35,1],
[8759,42,6],[8759,51,1],[8759,66,1],[8760,1,1],[8760,8,1],[8760,9,4],
[8760,10,1],[8760,13,1],[8760,18,1],[8760,19,1],[8760,37,2],
[8760,41,1],[8760,46,1],[8760,58,1],[8760,64,1],[8760,65,3],
[8760,67,2],[8760,77,1],[8761,26,1],[8761,43,1],[8762,32,3],
[8763,2,2],[8763,3,1],[8763,27,1],[8763,47,1],[8763,70,1],
[8764,28,11],[8764,51,1],[8764,63,4],[8764,70,1],[8764,74,4],
[8764,75,1],[8764,76,5],[8765,15,1],[8765,20,1],[8765,38,1],
[8765,55,1],[8766,27,1],[8766,28,2],[8766,32,1],[8766,44,1],
[8767,25,1],[8767,52,1],[8767,75,1],[8768,24,1],[8768,28,1],
[8769,31,7],[8770,15,1],[8770,17,7],[8770,28,1],[8770,53,4],
[8770,55,1],[8770,76,1],[8771,8,1],[8771,15,1],[8771,19,1],
[8771,20,1],[8771,45,1],[8771,73,1],[8772,23,3],[8772,28,1],
[8773,21,1],[8773,28,1],[8773,32,1],[8773,35,1],[8774,47,1],
[8774,51,3],[8774,68,1],[8775,30,2],[8775,32,1],[8776,25,1],
[8776,26,1],[8776,28,1],[8776,31,1],[8776,44,1],[8777,15,2],
[8778,25,1],[8778,28,1],[8779,22,2],[8779,25,4],[8779,27,1],
[8780,30,2],[8781,0,16],[8781,7,3],[8781,10,1],[8781,12,3],
[8781,16,1],[8781,19,2],[8781,29,1],[8781,41,1],[8781,54,1],
[8781,65,1],[8781,73,2],[8781,77,2],[8782,28,2],[8783,30,3],
[8783,36,3],[8784,0,1],[8784,1,3],[8784,17,1],[8784,23,1],[8784,24,1],
[8784,25,268],[8784,28,3],[8784,29,2],[8784,30,1],[8784,34,1],
[8784,35,1],[8784,42,18],[8784,51,1],[8785,15,1],[8785,20,1],
[8785,29,2],[8785,41,1],[8785,55,1],[8785,58,1],[8785,65,1],
[8785,67,1],[8785,71,1],[8786,24,1],[8786,32,10],[8787,23,2],
[8788,8,2],[8788,22,1],[8789,28,13],[8790,27,5],[8791,3,4],
[8791,20,1],[8791,21,1],[8791,23,3],[8791,24,1],[8791,27,3],
[8791,31,4],[8791,33,1],[8791,41,1],[8791,43,2],[8791,47,1],
[8791,64,1],[8791,65,1],[8791,74,2],[8792,28,7],[8793,17,1],
[8793,53,2],[8794,47,1],[8794,61,1],[8795,4,1],[8795,28,1],
[8795,68,3],[8795,69,5],[8795,70,35],[8795,72,3],[8796,2,1],
[8796,28,1],[8796,47,1],[8797,3,1],[8797,24,1],[8797,63,1],[8798,5,4],
[8798,70,1],[8799,27,7],[8800,8,1],[8800,28,254],[8801,2,3],
[8801,45,1],[8802,5,1],[8802,13,1],[8802,70,1],[8803,5,1],[8803,6,1],
[8803,26,1],[8803,27,1],[8803,28,2],[8804,2,2],[8804,3,1],[8804,21,1],
[8804,24,1],[8804,39,1],[8804,56,1],[8805,6,1],[8805,24,2],
[8805,26,1],[8805,35,2],[8805,47,2],[8806,21,2],[8807,24,1],
[8807,44,1],[8807,50,1],[8808,28,1],[8808,62,1],[8809,41,5],
[8810,21,10],[8810,26,6],[8810,43,2],[8811,12,1],[8811,29,2],
[8811,34,1],[8811,36,3],[8812,28,1],[8812,47,1],[8813,74,1],
[8813,75,1],[8814,5,1],[8814,47,1],[8814,62,1],[8815,14,1],
[8815,15,2],[8815,18,1],[8815,54,1],[8816,0,1],[8816,1,1],[8816,10,1],
[8816,15,2],[8816,26,1],[8816,31,1],[8816,37,1],[8816,45,1],
[8816,57,1],[8816,58,1],[8816,67,1],[8816,76,1],[8817,27,3],
[8817,28,1],[8817,38,1],[8817,41,1],[8817,47,1],[8817,48,1],

[8818,0,1],[8818,1,60],[8818,2,15],[8818,3,1],[8818,4,1],[8818,7,1],
[8818,8,5],[8818,9,3],[8818,11,1],[8818,12,119],[8818,13,11],
[8818,15,4],[8818,16,16],[8818,17,5],[8818,20,10],[8818,23,4],
[8818,24,1],[8818,25,11],[8818,26,9],[8818,27,1],[8818,28,1],
[8818,29,22],[8818,30,15],[8818,32,2],[8818,33,2],[8818,34,5],
[8818,35,2],[8818,36,6],[8818,37,1],[8818,39,13],[8818,43,3],
[8818,45,1],[8818,47,15],[8818,48,15],[8818,51,1],[8818,53,4],
[8818,55,4],[8818,59,1],[8818,65,2],[8818,73,4],[8818,79,1],
[8819,3,2],[8819,8,2],[8819,24,1],[8819,26,1],[8820,5,1],[8820,23,5],
[8820,26,1],[8820,32,1],[8820,35,2],[8820,43,1],[8821,2,1],
[8821,26,1],[8822,25,1],[8822,33,2],[8822,34,1],[8822,35,1],
[8823,5,1],[8823,6,2],[8823,21,7],[8823,22,8],[8823,23,24],
[8823,24,21],[8823,26,5],[8823,27,30],[8823,28,6],[8823,31,1],
[8823,32,4],[8823,33,2],[8823,35,18],[8823,38,2],[8823,40,1],
[8823,42,2],[8823,43,11],[8823,44,2],[8823,47,3],[8823,48,1],
[8823,50,1],[8823,52,1],[8823,56,1],[8823,62,1],[8823,66,1],
[8823,70,1],[8823,74,1],[8824,18,1],[8824,20,1],[8825,20,1],
[8825,45,9],[8825,48,2],[8825,53,1],[8825,78,1],[8826,23,1],
[8826,27,1],[8826,28,1],[8826,43,2],[8827,28,4],[8828,28,1],
[8828,32,1],[8829,5,4],[8829,28,1],[8830,31,2],[8831,4,3],[8831,5,1],
[8831,8,1],[8831,15,5],[8831,16,1],[8831,20,1],[8831,22,2],
[8831,27,1],[8831,28,1],[8831,34,1],[8831,42,1],[8831,44,2],
[8831,49,1],[8831,50,5],[8831,53,1],[8831,54,1],[8831,55,2],
[8831,57,1],[8831,64,1],[8831,65,2],[8831,66,2],[8831,67,1],
[8831,71,1],[8831,72,2],[8832,20,1],[8832,25,2],[8833,9,1],
[8833,22,1],[8833,43,1],[8834,20,1],[8834,26,1],[8835,22,1],
[8835,27,1],[8835,31,3],[8836,3,1],[8836,22,3],[8836,25,2],
[8836,26,2],[8836,27,2],[8836,32,4],[8836,43,1],[8837,8,1],
[8837,28,2],[8837,32,1],[8837,47,1],[8837,52,1],[8838,28,2],
[8838,40,1],[8839,3,1],[8839,4,1],[8839,21,1],[8839,47,1],[8839,51,1],
[8839,74,1],[8839,78,1],[8840,29,1],[8840,39,7],[8840,59,4],
[8841,2,2],[8841,66,1],[8842,15,1],[8842,27,2],[8842,42,1],
[8843,12,1],[8843,20,1],[8843,24,6],[8843,27,4],[8843,32,7],
[8843,34,1],[8843,35,1],[8843,46,1],[8843,63,1],[8843,66,1],
[8843,68,1],[8843,73,1],[8843,76,1],[8844,23,1],[8844,24,1],
[8844,26,1],[8844,27,2],[8844,38,1],[8844,56,1],[8845,25,6],
[8845,28,1],[8845,32,3],[8845,35,1],[8846,27,2],[8847,2,2],[8847,3,1],
[8847,13,1],[8847,59,1],[8848,32,5],[8849,15,1],[8849,28,1],
[8849,35,1],[8849,55,1],[8849,70,1],[8850,28,4],[8850,63,1],
[8851,33,6],[8851,43,1],[8852,29,1],[8852,45,1],[8852,53,2],
[8852,55,1],[8853,10,1],[8853,11,1],[8853,65,1],[8854,25,1],
[8854,28,4],[8854,70,1],[8854,75,1],[8855,23,2],[8855,24,8],
[8855,31,3],[8855,52,1],[8855,66,1],[8856,24,1],[8856,27,1],
[8856,68,1],[8857,2,1],[8857,22,1],[8857,24,3],[8857,27,1],
[8857,32,2],[8857,51,1],[8857,70,1],[8857,72,1],[8857,74,1],
[8858,13,1],[8858,34,1],[8858,77,1],[8859,22,1],[8859,44,2],
[8859,66,1],[8860,27,1],[8860,56,1],[8861,27,1],[8861,28,1],
[8862,39,1],[8862,41,1],[8863,2,2],[8864,2,2],[8864,3,1],[8864,4,3],
[8864,22,1],[8864,23,3],[8864,24,5],[8864,25,2],[8864,27,1],
[8864,28,6],[8864,31,1],[8864,32,3],[8864,33,4],[8864,35,2],

[8864,38,1],[8864,43,1],[8864,44,6],[8864,47,1],[8864,50,3],
[8864,51,1],[8864,52,1],[8864,66,1],[8864,68,1],[8864,70,4],
[8864,72,1],[8864,74,3],[8864,76,2],[8865,22,9],[8865,25,2],
[8865,26,2],[8865,27,10],[8865,28,7],[8865,33,3],[8865,35,2],
[8865,62,1],[8865,70,1],[8866,20,4],[8866,57,1],[8867,2,16],
[8867,27,1],[8867,28,2],[8867,31,1],[8867,36,1],[8867,44,1],
[8868,33,2],[8869,1,1],[8869,23,2],[8869,27,3],[8869,31,3],
[8869,32,1],[8869,43,1],[8869,47,1],[8870,31,2],[8871,2,2],
[8872,28,1],[8872,50,1],[8873,3,1],[8873,22,3],[8873,27,1],
[8873,72,1],[8873,77,1],[8874,2,2],[8874,22,2],[8874,26,1],
[8874,31,1],[8874,39,1],[8874,63,1],[8875,2,1],[8875,22,1],
[8875,31,1],[8876,24,1],[8876,27,1],[8876,44,1],[8876,48,2],
[8876,70,1],[8877,24,1],[8877,27,1],[8877,48,1],[8878,4,1],
[8878,51,1],[8879,3,2],[8879,4,1],[8879,15,1],[8879,16,1],[8879,17,1],
[8879,21,1],[8879,24,2],[8879,25,2],[8879,26,1],[8879,27,2],
[8879,28,1],[8879,35,2],[8879,38,1],[8879,40,1],[8879,44,3],
[8879,50,3],[8879,55,1],[8879,63,1],[8879,66,1],[8879,68,1],
[8879,70,1],[8879,72,1],[8879,74,2],[8879,75,1],[8879,76,2],
[8880,1,1],[8880,9,1],[8880,12,1],[8880,14,1],[8880,17,1],[8880,19,1],
[8880,46,1],[8881,22,1],[8881,62,1],[8882,26,1],[8882,28,1],
[8883,20,2],[8884,2,4],[8885,5,1],[8885,6,2],[8885,47,3],[8885,56,3],
[8885,60,1],[8886,32,2],[8886,38,1],[8887,5,1],[8887,35,1],[8888,2,1],
[8888,3,2],[8888,5,1],[8888,24,1],[8888,25,4],[8888,26,1],[8888,27,1],
[8888,28,1],[8888,31,1],[8888,46,1],[8888,55,1],[8889,15,1],
[8889,17,1],[8889,55,3],[8890,51,6],[8891,33,2],[8892,4,1],
[8892,15,1],[8892,17,2],[8892,53,3],[8892,66,1],[8893,2,2],[8894,0,1],
[8894,1,3],[8894,2,5],[8894,5,2],[8894,10,1],[8894,12,37],[8894,13,2],
[8894,16,3],[8894,17,1],[8894,19,1],[8894,20,1],[8894,24,1],
[8894,25,1],[8894,27,1],[8894,29,16],[8894,30,1],[8894,32,1],
[8894,34,1],[8894,36,2],[8894,46,1],[8894,58,1],[8894,60,1],
[8894,73,1],[8895,2,3],[8895,3,1],[8895,13,2],[8895,30,1],[8895,49,1],
[8895,56,1],[8895,74,1],[8896,3,1],[8896,9,1],[8896,19,1],[8896,28,1],
[8896,37,1],[8896,41,1],[8896,49,1],[8897,2,1],[8897,47,1],[8898,2,2],
[8898,13,1],[8898,28,1],[8899,21,1],[8899,23,1],[8899,28,2],
[8899,33,1],[8899,35,1],[8900,17,2],[8900,53,3],[8900,66,1],
[8901,28,13],[8902,44,2],[8902,66,1],[8903,2,1],[8903,3,6],[8903,6,1],
[8903,22,3],[8903,24,1],[8903,33,3],[8903,35,12],[8903,40,1],
[8903,43,2],[8903,66,1],[8903,68,1],[8904,3,1],[8904,23,1],
[8904,25,1],[8904,32,1],[8904,62,2],[8905,2,2],[8906,2,2],[8906,22,1],
[8906,23,1],[8906,25,1],[8906,28,1],[8906,32,1],[8906,35,2],
[8906,47,1],[8906,62,2],[8907,28,2],[8908,6,1],[8908,42,1],
[8909,15,2],[8909,17,1],[8909,38,1],[8909,53,1],[8909,55,1],
[8910,0,1],[8910,3,1],[8910,5,1],[8910,14,1],[8910,22,2],[8910,23,11],
[8910,24,7],[8910,25,5],[8910,26,3],[8910,27,1],[8910,30,1],
[8910,31,1],[8910,32,5],[8910,33,1],[8910,42,1],[8910,43,3],
[8910,47,2],[8911,6,1],[8911,23,1],[8911,24,3],[8911,32,1],
[8911,40,1],[8911,51,2],[8911,61,2],[8911,69,1],[8911,70,1],
[8911,74,2],[8912,23,1],[8912,32,1],[8912,43,1],[8913,1,3],
[8914,24,1],[8914,27,1],[8914,28,1],[8915,25,3],[8916,28,1],
[8916,69,1],[8917,2,1],[8917,5,1],[8917,20,7],[8917,21,1],[8917,27,3],

[8917,35,2],[8917,39,1],[8917,59,1],[8917,76,1],[8918,15,1],
[8918,50,1],[8919,31,1],[8919,33,1],[8919,35,1],[8919,63,1],
[8919,76,1],[8920,22,1],[8920,27,1],[8920,32,1],[8920,35,2],
[8921,16,1],[8921,49,1],[8922,1,2],[8923,23,1],[8923,26,1],
[8923,27,2],[8924,17,1],[8924,20,1],[8924,27,1],[8924,35,2],
[8924,37,1],[8924,42,1],[8924,77,1],[8925,1,43],[8925,2,1],
[8925,10,2],[8925,19,1],[8925,77,1],[8926,22,1],[8926,27,1],
[8926,31,2],[8927,11,1],[8927,14,1],[8927,15,1],[8927,59,1],
[8927,65,1],[8927,67,1],[8927,77,1],[8928,22,2],[8928,23,1],
[8928,24,1],[8928,25,1],[8928,27,1],[8928,43,1],[8929,28,1],
[8929,35,1],[8929,53,1],[8930,17,1],[8930,55,1],[8931,4,1],
[8931,22,3],[8931,23,8],[8931,24,17],[8931,27,1],[8931,28,1],
[8931,31,1],[8931,32,15],[8931,35,2],[8931,40,25],[8931,61,4],
[8931,68,1],[8931,70,25],[8931,72,7],[8932,2,1],[8932,15,1],
[8932,30,1],[8932,55,2],[8932,56,1],[8933,21,1],[8933,23,1],
[8933,26,1],[8933,28,4],[8934,22,1],[8934,27,1],[8934,28,1],
[8934,33,1],[8934,56,1],[8935,4,7],[8935,7,1],[8935,8,1],[8935,9,6],
[8935,14,1],[8935,15,2],[8935,17,303],[8935,30,3],[8935,34,1],
[8935,36,1],[8935,37,1],[8935,39,1],[8935,41,1],[8935,45,1],
[8935,50,13],[8935,53,361],[8935,55,5],[8935,58,1],[8935,59,1],
[8935,60,1],[8935,64,1],[8936,5,1],[8936,22,1],[8936,27,1],
[8936,28,2],[8937,0,3],[8937,1,97],[8937,7,4],[8937,8,12],[8937,9,1],
[8937,11,1],[8937,12,9],[8937,13,9],[8937,14,1],[8937,15,5],
[8937,16,2],[8937,17,6],[8937,18,1],[8937,19,1],[8937,20,2],
[8937,24,1],[8937,26,8],[8937,28,1],[8937,29,7],[8937,30,3],
[8937,31,5],[8937,34,15],[8937,36,3],[8937,39,427],[8937,42,4],
[8937,43,4],[8937,45,1],[8937,46,3],[8937,48,4],[8937,53,10],
[8937,55,5],[8937,59,2],[8937,73,4],[8937,77,1],[8938,43,2],
[8939,3,9],[8939,22,1],[8939,23,6],[8939,24,8],[8939,26,2],
[8939,27,1],[8939,28,8],[8939,32,5],[8939,35,1],[8939,59,1],
[8939,70,1],[8939,76,1],[8940,12,1],[8940,13,1],[8940,17,1],
[8941,10,1],[8941,24,1],[8942,3,7],[8942,23,1],[8942,24,3],
[8942,56,1],[8943,3,2],[8943,5,1],[8943,13,1],[8943,14,1],[8943,20,1],
[8943,23,8],[8943,24,8],[8943,27,19],[8943,28,9],[8943,32,6],
[8943,33,2],[8943,35,4],[8943,37,1],[8943,38,2],[8943,40,1],
[8943,42,1],[8943,43,1],[8943,44,2],[8943,47,1],[8943,48,4],
[8943,59,1],[8943,62,9],[8943,66,1],[8943,69,1],[8943,70,2],
[8943,72,1],[8943,78,1],[8944,23,6],[8944,35,1],[8944,72,1],
[8945,45,2],[8945,53,1],[8946,4,1],[8946,27,1],[8946,28,1],
[8946,66,2],[8947,5,1],[8947,27,1],[8947,50,1],[8947,64,1],
[8948,10,1],[8948,17,1],[8948,34,7],[8948,49,1],[8948,65,1],
[8949,27,4],[8950,22,1],[8950,40,1],[8950,55,1],[8950,56,3],
[8950,64,1],[8950,68,1],[8950,70,1],[8950,76,1],[8951,4,2],[8951,5,1],
[8951,6,1],[8951,8,1],[8951,21,2],[8951,25,1],[8951,26,1],[8951,27,3],
[8951,28,69],[8951,32,1],[8951,33,18],[8951,34,1],[8951,35,1],
[8951,40,1],[8951,43,1],[8951,44,1],[8951,48,1],[8951,51,1],
[8951,53,2],[8951,62,4],[8951,63,1],[8951,66,3],[8951,70,3],
[8951,75,1],[8951,76,1],[8952,3,4],[8952,4,1],[8952,13,1],[8952,24,2],
[8952,28,2],[8952,54,1],[8953,4,1],[8953,28,1],[8954,72,2],
[8955,28,3],[8956,21,1],[8956,26,1],[8956,28,1],[8957,28,28],

[8957,44,1],[8957,76,1],[8958,5,1],[8958,10,1],[8958,52,1],
[8958,70,1],[8959,19,1],[8959,26,2],[8960,27,2],[8960,31,1],
[8961,24,1],[8961,43,1],[8961,44,1],[8962,1,2],[8963,3,3],[8963,15,1],
[8963,21,1],[8963,24,1],[8963,29,1],[8963,37,1],[8963,57,1],
[8963,67,1],[8964,2,1],[8964,25,1],[8965,5,1],[8965,24,1],[8965,27,1],
[8965,32,3],[8965,56,1],[8965,68,1],[8966,20,1],[8966,27,1],
[8967,56,3],[8968,27,1],[8968,55,1],[8968,76,1],[8969,3,5],[8969,4,3],
[8969,6,2],[8969,7,1],[8969,9,1],[8969,11,1],[8969,20,1],[8969,22,3],
[8969,23,1],[8969,24,1],[8969,26,2],[8969,27,4],[8969,28,2],
[8969,32,1],[8969,33,2],[8969,47,1],[8969,56,8],[8969,60,2],
[8969,74,1],[8969,76,1],[8970,38,1],[8970,70,1],[8971,47,1],
[8971,62,1],[8971,68,1],[8972,26,1],[8972,48,1],[8973,1,1],
[8973,67,1],[8974,3,2],[8974,10,1],[8974,17,1],[8974,53,1],
[8974,62,2],[8975,4,1],[8975,53,1],[8975,55,1],[8976,2,2],[8976,5,2],
[8976,27,2],[8976,35,1],[8977,28,1],[8977,31,1],[8977,32,1],
[8977,40,1],[8978,3,2],[8978,22,2],[8979,25,1],[8979,33,1],[8980,1,6],
[8980,12,1],[8980,13,3],[8980,29,2],[8980,39,2],[8981,2,1],
[8981,31,1],[8981,46,1],[8982,19,1],[8982,65,1],[8983,28,1],
[8983,35,1],[8984,2,1],[8984,39,1],[8984,42,1],[8984,52,2],
[8985,25,1],[8985,26,1],[8985,28,4],[8986,28,1],[8986,31,1],
[8987,0,1],[8987,34,1],[8988,26,1],[8988,42,1],[8989,44,2],
[8989,66,2],[8990,28,1],[8990,77,1],[8991,6,1],[8991,22,2],
[8991,25,1],[8991,27,4],[8991,51,1],[8991,56,1],[8991,68,1],
[8992,23,1],[8992,27,1],[8992,47,1],[8993,33,1],[8993,37,1],
[8993,42,1],[8994,2,1],[8994,26,1],[8994,27,1],[8994,28,1],
[8994,47,1],[8994,74,1],[8995,15,2],[8995,17,4],[8995,53,1],
[8995,55,2],[8996,1,2],[8996,12,1],[8996,27,1],[8997,2,1],[8997,52,1],
[8998,35,1],[8998,63,1],[8999,31,2],[9000,2,1],[9000,28,5],
[9000,31,1],[9001,30,8],[9001,34,1],[9002,39,2],[9002,45,1],
[9003,15,1],[9003,18,1],[9004,2,1],[9004,28,1],[9004,43,2],[9005,5,1],
[9005,47,4],[9005,51,1],[9005,69,1],[9005,70,2],[9006,2,3],
[9007,76,2],[9008,75,1],[9008,76,1],[9009,43,2],[9010,26,1],
[9010,40,1],[9010,57,1],[9011,2,1],[9011,15,1],[9011,21,1],
[9011,22,1],[9011,35,1],[9011,62,2],[9012,47,3],[9013,20,2],
[9013,39,1],[9013,62,3],[9014,27,2],[9014,46,1],[9014,70,1],
[9015,21,5],[9016,2,3],[9016,10,1],[9016,20,2],[9016,21,1],
[9016,22,1],[9016,31,2],[9016,34,1],[9016,37,1],[9016,43,1],
[9016,65,1],[9017,6,1],[9017,17,1],[9017,20,1],[9017,27,1],
[9017,43,1],[9018,3,1],[9018,21,1],[9018,26,2],[9018,27,1],
[9018,35,1],[9019,27,8],[9020,3,2],[9020,17,1],[9020,23,1],
[9020,37,5],[9020,50,1],[9021,6,1],[9021,27,1],[9021,33,2],
[9021,72,1],[9022,25,1],[9022,28,1],[9023,6,1],[9023,28,1],
[9023,35,1],[9024,2,1],[9024,17,1],[9024,20,3],[9024,34,10],
[9024,41,1],[9024,42,1],[9025,26,5],[9025,31,1],[9025,32,1],
[9025,42,1],[9025,43,1],[9025,51,4],[9026,27,1],[9026,31,1],
[9027,5,1],[9027,31,1],[9027,61,1],[9027,63,3],[9027,74,3],
[9027,75,3],[9027,76,1],[9028,28,3],[9029,24,2],[9029,28,19],
[9029,63,1],[9029,72,1],[9029,75,2],[9030,59,2],[9030,61,2],
[9031,5,1],[9031,6,1],[9031,22,1],[9031,27,1],[9031,32,20],
[9031,35,2],[9031,38,1],[9031,56,1],[9031,66,1],[9032,16,1],

[9032,29,1],[9032,43,1],[9032,53,2],[9032,55,2],[9032,62,1],
[9032,66,1],[9033,42,2],[9034,10,1],[9034,54,1],[9035,22,2],
[9035,33,1],[9036,10,1],[9036,37,2],[9037,0,1],[9037,4,8],[9037,8,4],
[9037,9,2],[9037,10,1],[9037,13,1],[9037,15,15],[9037,17,48],
[9037,19,3],[9037,20,1],[9037,28,1],[9037,30,1],[9037,39,1],
[9037,41,1],[9037,44,2],[9037,46,1],[9037,50,6],[9037,53,52],
[9037,55,52],[9037,57,1],[9037,59,1],[9037,65,1],[9037,66,3],
[9037,77,1],[9038,4,1],[9038,8,1],[9038,22,7],[9038,23,1],[9038,24,3],
[9038,26,2],[9038,28,1],[9038,47,1],[9038,52,1],[9039,21,1],
[9039,25,1],[9039,32,1],[9039,33,4],[9039,35,7],[9039,52,12],
[9040,53,2],[9041,28,1],[9041,61,1],[9042,26,1],[9042,28,1],
[9043,26,1],[9043,44,2],[9043,66,2],[9044,32,1],[9044,38,1],
[9044,40,1],[9044,47,3],[9044,70,1],[9044,76,1],[9045,23,1],
[9045,28,1],[9046,74,1],[9046,76,2],[9047,22,1],[9047,27,1],
[9048,24,1],[9048,28,21],[9048,42,1],[9048,59,1],[9049,2,2],
[9049,20,1],[9049,31,1],[9049,43,1],[9050,23,1],[9050,28,1],
[9051,24,2],[9052,24,1],[9052,27,1],[9052,43,1],[9053,31,2],
[9053,68,1],[9054,3,2],[9054,8,1],[9054,21,1],[9054,23,1],[9054,24,3],
[9054,25,1],[9054,27,1],[9054,35,3],[9054,43,1],[9054,56,1],
[9054,62,6],[9055,4,2],[9055,28,1],[9056,2,4],[9056,26,1],
[9056,33,10],[9057,2,5],[9057,41,1],[9058,28,1],[9058,32,3],
[9058,44,1],[9059,0,11],[9059,7,23],[9059,12,2],[9059,16,1],
[9059,30,1],[9059,37,2],[9059,45,2],[9059,46,1],[9059,54,1],
[9059,57,1],[9059,65,1],[9059,73,4],[9059,77,2],[9060,9,1],
[9060,30,3],[9060,64,1],[9060,65,1],[9061,22,1],[9061,28,2],
[9061,50,1],[9062,60,2],[9063,3,69],[9063,5,7],[9063,6,9],[9063,22,5],
[9063,23,80],[9063,24,109],[9063,25,1],[9063,26,8],[9063,27,2],
[9063,28,44],[9063,31,14],[9063,32,35],[9063,33,8],[9063,35,3],
[9063,38,2],[9063,42,9],[9063,43,2],[9063,44,1],[9063,47,31],
[9063,48,1],[9063,56,47],[9063,60,1],[9063,62,1],[9063,66,1],
[9063,70,1],[9063,74,1],[9063,75,1],[9063,76,1],[9064,4,1],
[9064,50,1],[9065,2,4],[9066,2,1],[9066,9,1],[9066,11,1],[9066,14,1],
[9066,17,1],[9066,20,8],[9066,21,1],[9066,23,9],[9066,24,1],
[9066,27,29],[9066,33,3],[9066,34,1],[9066,35,35],[9066,39,22],
[9066,40,14],[9066,42,3],[9066,46,1],[9066,52,4],[9066,56,1],
[9066,59,1],[9066,61,5],[9066,62,1],[9066,68,4],[9066,69,1],
[9066,70,16],[9066,73,1],[9066,74,1],[9066,76,6],[9066,77,1],
[9067,23,1],[9067,28,1],[9067,42,7],[9068,30,1],[9068,54,1],
[9069,28,3],[9069,70,1],[9070,10,2],[9070,24,1],[9071,23,2],
[9071,25,1],[9071,40,1],[9071,43,1],[9071,63,11],[9071,66,1],
[9071,70,2],[9071,74,12],[9071,75,8],[9071,76,11],[9072,1,1],
[9072,3,2],[9072,5,2],[9072,6,1],[9072,7,1],[9072,8,1],[9072,10,1],
[9072,11,1],[9072,12,3],[9072,13,5],[9072,18,1],[9072,19,2],
[9072,20,1],[9072,21,1],[9072,23,7],[9072,24,42],[9072,25,1],
[9072,27,1],[9072,28,5],[9072,29,1],[9072,53,1],[9072,56,8],
[9072,57,2],[9072,58,2],[9072,59,1],[9072,60,1],[9072,62,1],
[9072,63,1],[9072,65,2],[9072,66,1],[9072,73,1],[9073,28,2],
[9073,47,1],[9074,22,1],[9074,52,2],[9075,22,1],[9075,23,1],
[9075,40,1],[9075,74,1],[9075,76,3],[9076,2,35],[9076,3,3],
[9076,5,12],[9076,17,1],[9076,19,1],[9076,20,3],[9076,21,7],

[9076,22,6],[9076,23,8],[9076,24,4],[9076,25,1],[9076,26,3],
[9076,27,12],[9076,28,4],[9076,31,19],[9076,32,8],[9076,33,4],
[9076,34,1],[9076,37,1],[9076,42,3],[9076,43,6],[9076,44,1],
[9076,47,6],[9076,50,1],[9076,52,2],[9076,53,1],[9076,54,1],
[9076,56,3],[9076,60,2],[9076,61,1],[9076,62,2],[9076,68,1],
[9076,70,1],[9076,72,1],[9076,73,1],[9076,74,1],[9076,76,1],
[9076,78,1],[9077,2,13],[9077,4,1],[9077,21,1],[9077,22,1],
[9077,23,5],[9077,26,3],[9077,27,3],[9077,28,1],[9077,30,1],
[9077,31,6],[9077,33,3],[9077,39,1],[9077,41,1],[9077,42,2],
[9077,43,2],[9077,47,1],[9077,56,2],[9077,58,1],[9077,60,1],
[9077,62,10],[9077,65,1],[9077,69,1],[9077,74,1],[9078,5,3],
[9078,28,1],[9079,26,1],[9079,63,1],[9080,23,1],[9080,26,1],
[9080,27,2],[9080,32,1],[9080,38,2],[9080,52,1],[9081,23,1],
[9081,74,1],[9082,3,1],[9082,23,2],[9082,25,1],[9082,27,1],
[9082,30,1],[9082,70,1],[9082,77,1],[9083,13,1],[9083,18,1],
[9084,7,1],[9084,10,1],[9084,11,1],[9084,12,1],[9084,13,1],
[9084,14,1],[9084,15,2],[9084,16,1],[9084,17,2],[9084,18,1],
[9084,19,4],[9084,20,2],[9084,29,1],[9084,34,1],[9084,36,1],
[9084,37,1],[9084,41,1],[9084,45,4],[9084,49,2],[9084,53,6],
[9084,57,1],[9084,58,1],[9084,59,1],[9084,60,1],[9084,65,1],
[9084,67,2],[9084,70,2],[9084,77,1],[9085,5,2],[9085,28,1],
[9086,23,1],[9086,61,1],[9087,28,1],[9087,32,1],[9088,13,1],
[9088,44,2],[9088,50,4],[9088,53,2],[9088,62,1],[9088,66,3],
[9089,32,4],[9090,3,1],[9090,28,1],[9090,62,2],[9090,68,2],
[9090,70,1],[9091,13,2],[9092,5,1],[9092,15,1],[9092,23,1],
[9092,24,5],[9092,26,3],[9092,27,1],[9092,32,1],[9092,42,12],
[9092,48,1],[9092,49,1],[9092,55,2],[9092,59,2],[9092,62,1],
[9092,66,1],[9092,70,1],[9092,72,1],[9093,24,1],[9093,28,1],
[9093,39,1],[9094,3,1],[9094,26,1],[9095,7,2],[9096,38,1],[9096,42,1],
[9097,24,1],[9097,27,1],[9097,38,1],[9098,27,1],[9098,28,1],
[9099,2,5],[9099,25,1],[9099,32,1],[9099,70,1],[9100,28,1],
[9100,35,1],[9101,22,1],[9101,27,3],[9101,28,4],[9102,2,3],
[9102,28,1],[9102,56,1],[9103,47,1],[9103,69,1],[9104,2,1],
[9104,22,3],[9104,24,1],[9104,25,1],[9104,27,1],[9104,33,1],
[9105,3,2],[9105,5,1],[9105,9,19],[9105,10,1],[9105,11,1],[9105,12,1],
[9105,14,2],[9105,16,1],[9105,17,1],[9105,18,1],[9105,19,1],
[9105,20,1],[9105,28,165],[9105,31,1],[9105,38,1],[9105,44,1],
[9105,48,1],[9105,63,70],[9105,68,3],[9105,69,1],[9105,70,1],
[9105,72,2],[9105,74,52],[9105,75,34],[9105,76,81],[9106,15,1],
[9106,55,1],[9106,64,1],[9107,4,1],[9107,38,1],[9107,50,1],
[9107,66,3],[9107,75,1],[9108,28,1],[9108,32,1],[9109,9,1],
[9109,73,1],[9110,32,1],[9110,70,1],[9111,2,5],[9111,3,1],[9111,26,1],
[9111,27,1],[9111,32,1],[9111,43,1],[9112,22,1],[9112,24,1],
[9112,26,1],[9113,2,2],[9113,25,1],[9113,27,1],[9113,33,1],
[9113,43,1],[9114,8,2],[9114,25,6],[9114,46,1],[9115,23,1],
[9115,76,1],[9116,10,1],[9116,15,1],[9116,37,1],[9116,55,1],
[9116,73,1],[9117,2,1],[9117,3,11],[9117,6,2],[9117,18,1],[9117,20,2],
[9117,21,1],[9117,23,3],[9117,24,5],[9117,26,1],[9117,28,5],
[9117,35,1],[9117,39,1],[9117,60,1],[9118,0,226],[9118,1,754],
[9118,2,22],[9118,3,21],[9118,4,104],[9118,5,20],[9118,6,11],

[9118,7,90],[9118,8,243],[9118,9,58],[9118,10,55],[9118,11,13],
[9118,12,317],[9118,13,199],[9118,14,4],[9118,15,856],[9118,16,31],
[9118,17,2480],[9118,18,15],[9118,19,24],[9118,20,147],[9118,21,15],
[9118,22,14],[9118,23,44],[9118,24,46],[9118,25,44],[9118,26,33],
[9118,27,92],[9118,28,220],[9118,29,221],[9118,30,100],[9118,31,20],
[9118,32,35],[9118,33,30],[9118,34,267],[9118,35,59],[9118,36,138],
[9118,37,48],[9118,38,16],[9118,39,362],[9118,40,5],[9118,41,8],
[9118,42,20],[9118,43,15],[9118,44,97],[9118,45,22],[9118,46,12],
[9118,47,18],[9118,48,18],[9118,49,2],[9118,50,112],[9118,51,26],
[9118,52,4],[9118,53,2635],[9118,54,6],[9118,55,1615],[9118,56,4],
[9118,57,14],[9118,58,10],[9118,59,70],[9118,60,11],[9118,61,3],
[9118,62,18],[9118,63,8],[9118,64,1],[9118,65,25],[9118,66,70],
[9118,67,5],[9118,68,8],[9118,69,12],[9118,70,23],[9118,71,8],
[9118,72,9],[9118,73,126],[9118,74,13],[9118,75,4],[9118,76,13],
[9118,77,10],[9118,78,19],[9118,79,8],[9119,9,10],[9119,28,5],
[9120,28,2],[9120,44,1],[9121,3,1],[9121,32,1],[9121,35,1],
[9122,50,1],[9122,68,1],[9123,25,3],[9124,1,1],[9124,3,3],[9124,6,1],
[9124,14,1],[9124,16,1],[9124,18,1],[9124,20,6],[9124,22,1],
[9124,23,26],[9124,24,38],[9124,26,6],[9124,28,15],[9124,31,2],
[9124,33,5],[9124,34,2],[9124,38,1],[9124,42,1],[9124,43,1],
[9124,56,1],[9124,65,1],[9124,72,2],[9125,1,1],[9125,53,1],[9126,7,1],
[9126,13,1],[9126,20,1],[9126,30,1],[9126,34,1],[9126,60,1],
[9126,75,1],[9127,22,2],[9127,23,1],[9127,26,1],[9127,42,2],
[9128,22,1],[9128,23,1],[9129,23,1],[9129,28,1],[9129,35,1],
[9129,37,1],[9129,59,1],[9130,23,1],[9130,28,1],[9130,33,1],
[9130,35,1],[9131,6,1],[9131,10,1],[9131,13,1],[9131,39,3],
[9131,56,1],[9132,4,1],[9132,16,2],[9132,17,3],[9132,26,1],
[9132,53,2],[9133,4,3],[9133,25,4],[9133,26,1],[9133,27,2],
[9133,28,296],[9133,40,94],[9133,42,1],[9133,52,1],[9133,68,23],
[9133,69,26],[9133,70,118],[9133,72,51],[9134,15,2],[9135,0,1],
[9135,1,1],[9135,10,1],[9135,11,1],[9135,12,1],[9135,19,2],
[9135,49,1],[9135,73,1],[9135,77,1],[9136,33,1],[9136,48,1],
[9137,8,2],[9137,10,1],[9137,28,2],[9138,25,1],[9138,27,1],
[9139,21,8],[9139,30,2],[9140,28,28],[9140,63,1],[9141,53,2],
[9142,1,1],[9142,18,1],[9142,29,1],[9143,5,1],[9143,13,2],[9143,20,2],
[9143,23,2],[9143,35,1],[9143,43,3],[9143,45,1],[9143,59,1],
[9143,65,1],[9143,71,2],[9144,0,15],[9144,1,4],[9144,7,1],[9144,9,3],
[9144,10,1],[9144,11,2],[9144,12,3],[9144,15,3],[9144,17,1],
[9144,19,1],[9144,20,2],[9144,29,5],[9144,34,1],[9144,36,3],
[9144,37,1],[9144,45,1],[9144,49,2],[9144,57,1],[9144,65,1],
[9144,67,1],[9144,77,1],[9144,78,1],[9145,4,5],[9145,50,5],
[9146,17,6],[9146,53,1],[9147,12,1],[9147,13,2],[9147,14,1],
[9147,19,1],[9147,30,1],[9147,45,1],[9147,46,2],[9147,55,1],
[9147,65,1],[9147,73,1],[9148,15,1],[9148,30,1],[9148,46,1],
[9149,0,222],[9149,1,5],[9149,7,204],[9149,8,4],[9149,9,6],
[9149,10,6],[9149,11,2],[9149,12,21],[9149,14,5],[9149,15,2],
[9149,16,15],[9149,18,19],[9149,19,10],[9149,20,3],[9149,29,1],
[9149,30,2],[9149,31,7],[9149,34,3],[9149,35,1],[9149,36,5],
[9149,37,3],[9149,39,4],[9149,41,7],[9149,45,20],[9149,46,11],
[9149,50,1],[9149,53,4],[9149,54,2],[9149,55,2],[9149,57,7],

[9149,58,1],[9149,59,5],[9149,60,2],[9149,62,6],[9149,64,2],
[9149,65,12],[9149,67,1],[9149,71,4],[9149,73,19],[9149,77,15],
[9150,10,1],[9150,19,1],[9150,49,1],[9150,65,1],[9151,17,1],
[9151,53,1],[9152,2,4],[9153,1,2],[9153,2,1],[9153,3,1],[9153,5,1],
[9153,6,1],[9153,8,2],[9153,9,2],[9153,10,6],[9153,12,2],[9153,13,2],
[9153,15,3],[9153,16,2],[9153,19,3],[9153,20,1],[9153,23,3],
[9153,24,2],[9153,25,1],[9153,27,4],[9153,28,18],[9153,29,1],
[9153,30,22],[9153,31,4],[9153,33,1],[9153,34,1],[9153,35,3],
[9153,36,1],[9153,37,21],[9153,38,4],[9153,42,2],[9153,44,3],
[9153,46,1],[9153,48,1],[9153,50,3],[9153,52,57],[9153,53,2],
[9153,55,2],[9153,57,1],[9153,58,2],[9153,62,1],[9153,64,1],
[9153,65,3],[9153,66,4],[9153,67,1],[9153,69,1],[9153,71,1],
[9153,72,2],[9153,74,2],[9153,75,2],[9153,76,2],[9154,63,2],
[9154,74,3],[9154,75,3],[9154,76,2],[9155,34,3],[9155,42,2],
[9156,32,1],[9156,35,1],[9156,40,1],[9156,55,1],[9156,56,1],
[9156,68,1],[9156,70,1],[9157,33,2],[9157,47,1],[9158,15,2],
[9158,50,1],[9158,53,1],[9158,55,2],[9158,74,1],[9159,29,1],
[9159,73,2],[9160,20,1],[9160,31,1],[9160,39,1],[9161,8,4],
[9162,17,1],[9162,39,2],[9162,55,1],[9163,17,2],[9163,53,1],
[9164,2,1],[9164,5,3],[9164,24,2],[9164,25,3],[9164,38,1],[9164,42,2],
[9164,44,1],[9164,56,1],[9165,13,1],[9165,29,1],[9166,20,2],
[9166,23,1],[9167,1,1],[9167,12,2],[9167,48,1],[9167,55,1],
[9167,70,1],[9168,2,1],[9168,3,1],[9168,23,2],[9168,43,1],[9169,31,2],
[9169,48,1],[9169,73,1],[9170,22,1],[9170,24,1],[9170,25,1],
[9170,60,1],[9170,77,1],[9171,55,2],[9171,77,1],[9172,3,4],[9172,5,1],
[9172,23,20],[9172,26,1],[9172,32,4],[9172,33,1],[9172,38,2],
[9172,43,1],[9172,48,1],[9172,56,5],[9173,4,1],[9173,17,7],
[9173,50,1],[9173,53,4],[9173,59,1],[9174,15,2],[9175,6,2],
[9175,22,1],[9175,27,1],[9175,28,1],[9175,42,2],[9175,56,5],
[9175,72,1],[9176,21,1],[9176,33,1],[9177,0,1],[9177,2,1],[9177,3,3],
[9177,4,2],[9177,20,3],[9177,21,2],[9177,23,1],[9177,28,1],
[9177,30,1],[9177,35,1],[9177,37,2],[9177,38,2],[9177,43,1],
[9177,48,1],[9178,20,1],[9178,59,1],[9179,25,4],[9179,37,3],
[9180,2,1],[9180,31,1],[9181,9,1],[9181,19,1],[9182,2,62],[9182,24,1],
[9182,28,1],[9182,31,12],[9182,35,1],[9182,47,2],[9182,55,1],
[9182,70,1],[9182,75,1],[9183,23,5],[9183,26,1],[9183,39,1],
[9184,20,1],[9184,22,1],[9185,5,1],[9185,6,1],[9185,35,1],[9186,2,1],
[9186,43,1],[9187,13,1],[9187,19,1],[9187,23,1],[9187,24,3],
[9187,25,1],[9187,28,2],[9187,32,2],[9187,44,1],[9187,47,2],
[9187,51,1],[9187,56,1],[9187,77,1],[9188,44,1],[9188,76,1],
[9189,3,3],[9189,13,1],[9189,17,1],[9189,20,3],[9189,23,8],
[9189,24,3],[9189,25,2],[9189,26,1],[9189,27,2],[9189,28,3],
[9189,31,2],[9189,32,2],[9189,39,1],[9189,47,1],[9189,78,1],
[9190,2,2],[9190,3,3],[9190,23,3],[9190,24,1],[9190,27,6],[9190,28,1],
[9190,30,1],[9190,31,1],[9190,32,1],[9190,33,1],[9190,35,2],
[9190,47,2],[9190,70,1],[9190,72,1],[9190,73,1],[9191,2,1],[9191,3,1],
[9191,5,1],[9191,11,1],[9191,23,1],[9191,30,1],[9191,37,5],
[9191,47,1],[9191,50,1],[9191,62,3],[9191,70,1],[9192,1,1],
[9192,55,2],[9193,3,1],[9193,32,1],[9193,39,1],[9193,42,1],
[9194,23,1],[9194,27,1],[9194,28,1],[9195,24,1],[9195,38,3],

[9196,2,1],[9196,26,1],[9196,28,1],[9196,35,1],[9196,60,1],
[9197,37,3],[9198,63,1],[9198,75,2],[9198,76,2],[9199,23,1],
[9199,50,1],[9200,1,3],[9201,2,1],[9201,4,1],[9201,23,1],[9201,25,1],
[9201,28,5],[9201,33,14],[9201,35,2],[9201,40,1],[9201,52,15],
[9201,56,1],[9201,62,1],[9201,70,1],[9202,15,1],[9202,55,2],
[9203,9,1],[9203,20,1],[9203,27,1],[9203,32,10],[9203,40,1],
[9203,53,1],[9203,63,2],[9203,73,1],[9204,2,2],[9204,23,1],[9205,4,1],
[9205,17,1],[9206,10,2],[9206,19,2],[9206,30,7],[9206,37,11],
[9206,48,2],[9206,57,1],[9207,24,1],[9207,27,3],[9208,26,2],
[9208,28,1],[9209,2,2],[9209,17,1],[9209,19,2],[9209,20,6],
[9209,21,1],[9209,24,3],[9209,26,2],[9209,27,3],[9209,28,1],
[9209,32,1],[9209,35,1],[9209,36,1],[9209,39,1],[9209,43,1],
[9209,72,1],[9210,22,1],[9210,23,1],[9210,24,1],[9210,28,2],
[9210,58,1],[9211,70,3],[9212,12,1],[9212,15,1],[9212,57,1],
[9213,34,1],[9213,53,1],[9214,1,2],[9214,26,1],[9214,28,2],[9215,3,1],
[9215,28,2],[9215,30,1],[9215,31,1],[9215,67,1],[9215,77,1],
[9216,5,1],[9216,28,1],[9216,32,2],[9216,61,1],[9217,20,1],
[9217,25,2],[9217,28,1],[9218,0,2],[9218,27,1],[9218,30,2],
[9218,45,1],[9218,60,1],[9219,35,1],[9219,56,1],[9220,0,1],[9220,3,1],
[9220,5,1],[9220,8,1],[9220,11,1],[9220,19,2],[9220,31,1],[9220,47,1],
[9220,57,1],[9220,59,1],[9220,63,8],[9220,70,1],[9220,73,2],
[9220,74,6],[9220,75,7],[9220,76,13],[9221,2,3],[9221,5,1],
[9221,20,3],[9221,22,3],[9221,23,1],[9221,24,1],[9221,25,1],
[9221,26,1],[9221,27,12],[9221,32,1],[9221,35,2],[9221,40,1],
[9221,47,2],[9222,2,1],[9222,8,1],[9222,53,1],[9223,27,6],[9224,23,3],
[9224,42,1],[9225,1,1],[9225,20,1],[9225,26,2],[9225,41,1],
[9225,43,1],[9225,48,1],[9226,28,1],[9226,32,1],[9226,42,1],
[9226,52,1],[9227,26,1],[9227,27,1],[9227,28,1],[9228,6,1],
[9228,28,2],[9228,56,1],[9229,2,4],[9229,26,1],[9229,27,3],
[9229,28,1],[9229,43,2],[9230,24,1],[9230,26,1],[9230,31,1],
[9230,43,1],[9231,0,3],[9231,34,3],[9231,36,4],[9231,73,1],[9232,1,1],
[9232,12,1],[9233,23,1],[9233,24,1],[9233,27,4],[9233,41,1],
[9234,0,9],[9234,1,37],[9234,2,5],[9234,3,2],[9234,4,1],[9234,5,3],
[9234,6,1],[9234,7,5],[9234,8,28],[9234,9,38],[9234,10,29],
[9234,11,9],[9234,12,34],[9234,13,28],[9234,14,15],[9234,15,56],
[9234,16,17],[9234,17,24],[9234,18,11],[9234,19,31],[9234,20,74],
[9234,22,295],[9234,23,81],[9234,24,534],[9234,25,2],[9234,26,2],
[9234,27,56],[9234,28,32],[9234,29,13],[9234,30,15],[9234,31,2],
[9234,32,5],[9234,33,2],[9234,34,26],[9234,35,16],[9234,36,12],
[9234,37,15],[9234,38,3],[9234,39,10],[9234,40,7],[9234,41,19],
[9234,42,4],[9234,43,1],[9234,44,5],[9234,45,21],[9234,46,14],
[9234,47,3],[9234,48,1],[9234,49,10],[9234,50,1],[9234,52,1],
[9234,53,23],[9234,54,6],[9234,55,30],[9234,56,1],[9234,57,13],
[9234,58,18],[9234,59,14],[9234,60,7],[9234,61,6],[9234,62,2],
[9234,64,4],[9234,65,32],[9234,66,8],[9234,67,17],[9234,68,6],
[9234,69,2],[9234,70,81],[9234,71,9],[9234,72,56],[9234,73,7],
[9234,74,4],[9234,76,4],[9234,77,18],[9234,78,2],[9235,26,2],
[9236,28,1],[9236,32,5],[9236,56,1],[9237,2,1],[9237,3,1],[9237,5,1],
[9237,23,12],[9237,24,5],[9237,25,1],[9237,26,6],[9237,28,2],
[9237,31,9],[9237,32,4],[9237,33,1],[9237,38,1],[9237,43,6],

[9237,44,1],[9237,79,1],[9238,28,2],[9239,21,1],[9239,25,3],
[9239,32,1],[9239,34,1],[9239,53,1],[9239,62,8],[9240,10,1],
[9240,20,1],[9241,2,8],[9241,46,4],[9241,47,4],[9241,62,2],[9242,1,2],
[9243,21,3],[9244,13,1],[9244,71,1],[9245,28,1],[9245,44,1],
[9245,47,1],[9246,2,2],[9247,24,6],[9247,32,1],[9247,56,2],
[9248,17,1],[9248,45,1],[9248,78,1],[9249,9,1],[9249,37,1],
[9250,22,1],[9250,27,1],[9251,63,3],[9251,74,1],[9251,75,1],
[9252,1,1],[9252,20,1],[9253,17,1],[9253,27,1],[9253,28,2],
[9253,44,4],[9253,50,1],[9253,66,3],[9254,1,1],[9254,2,10],[9254,3,8],
[9254,4,2],[9254,5,4],[9254,6,5],[9254,9,5],[9254,10,3],[9254,11,1],
[9254,12,8],[9254,13,16],[9254,14,1],[9254,15,1],[9254,19,4],
[9254,20,4],[9254,21,3],[9254,22,1],[9254,23,8],[9254,24,8],
[9254,25,1],[9254,26,1],[9254,27,7],[9254,28,6],[9254,29,1],
[9254,30,7],[9254,33,5],[9254,35,1],[9254,37,1],[9254,38,2],
[9254,39,2],[9254,40,3],[9254,42,4],[9254,43,1],[9254,44,2],
[9254,45,3],[9254,46,3],[9254,47,1],[9254,48,1],[9254,49,1],
[9254,50,1],[9254,51,3],[9254,53,1],[9254,63,1],[9254,66,1],
[9254,68,1],[9254,69,1],[9254,70,15],[9254,72,2],[9254,73,2],
[9254,74,9],[9254,75,1],[9254,77,1],[9254,78,2],[9255,0,1],[9255,1,9],
[9255,2,1],[9255,3,1],[9255,4,14],[9255,5,1],[9255,9,6],[9255,10,2],
[9255,11,1],[9255,12,5],[9255,13,4],[9255,14,1],[9255,15,106],
[9255,16,2],[9255,17,89],[9255,18,1],[9255,19,4],[9255,20,5],
[9255,21,2],[9255,22,1],[9255,23,2],[9255,24,3],[9255,25,1],
[9255,26,3],[9255,27,1],[9255,28,6],[9255,29,5],[9255,30,1],
[9255,31,1],[9255,33,1],[9255,34,2],[9255,35,1],[9255,37,1],
[9255,38,3],[9255,39,2],[9255,40,3],[9255,41,1],[9255,42,3],
[9255,44,9],[9255,45,2],[9255,46,2],[9255,50,29],[9255,51,1],
[9255,52,1],[9255,53,102],[9255,54,2],[9255,55,59],[9255,56,2],
[9255,57,1],[9255,59,2],[9255,62,1],[9255,64,1],[9255,65,4],
[9255,66,19],[9255,70,1],[9255,72,1],[9255,73,1],[9255,77,1],
[9256,3,2],[9256,26,2],[9257,17,1],[9257,59,1],[9258,22,1],
[9258,24,1],[9258,27,2],[9258,28,4],[9258,35,1],[9259,2,1],
[9259,31,2],[9260,17,1],[9260,23,1],[9260,28,4],[9260,40,1],
[9260,50,1],[9260,55,1],[9261,2,1],[9261,15,1],[9261,61,1],
[9262,28,1],[9262,43,1],[9262,63,1],[9263,17,1],[9263,23,1],
[9263,26,1],[9263,27,1],[9263,28,11],[9263,39,1],[9263,42,1],
[9263,79,1],[9264,1,1],[9264,2,1],[9264,21,1],[9264,33,1],[9264,57,1],
[9264,70,1],[9265,2,2],[9265,43,1],[9266,42,2],[9267,1,2],[9267,2,2],
[9267,69,1],[9268,25,1],[9268,32,2],[9269,1,2],[9270,21,2],
[9270,23,1],[9270,24,2],[9270,26,2],[9270,28,1],[9270,32,1],
[9270,33,2],[9270,35,1],[9270,39,1],[9271,20,1],[9271,32,2],
[9271,69,1],[9272,15,1],[9272,45,1],[9273,9,1],[9273,42,1],
[9274,28,1],[9274,37,1],[9274,47,1],[9274,69,1],[9275,20,1],
[9275,33,1],[9275,35,2],[9275,70,1],[9276,30,1],[9276,55,1],
[9277,44,1],[9277,68,1],[9278,1,5],[9278,8,1],[9278,10,1],
[9278,12,20],[9278,16,7],[9278,17,1],[9278,36,1],[9278,39,1],
[9278,48,1],[9279,1,1],[9279,2,1],[9279,3,1],[9279,5,1],[9279,8,1],
[9279,13,1],[9279,20,5],[9279,24,2],[9279,27,2],[9279,35,1],
[9279,39,1],[9279,42,1],[9279,57,1],[9279,77,1],[9280,2,6],[9281,2,1],
[9281,3,1],[9281,5,5],[9281,12,1],[9281,13,1],[9281,15,2],[9281,20,2],

[9281,21,1],[9281,23,1],[9281,26,1],[9281,27,1],[9281,38,2],
[9281,55,1],[9281,56,1],[9281,72,1],[9281,76,1],[9282,9,1],
[9282,16,1],[9282,17,4],[9282,28,2],[9283,28,7],[9284,70,6],
[9285,2,2],[9285,3,2],[9285,4,1],[9285,6,1],[9285,21,1],[9285,22,1],
[9285,23,4],[9285,24,1],[9285,25,1],[9285,26,1],[9285,27,3],
[9285,28,74],[9285,31,1],[9285,32,3],[9285,33,2],[9285,35,5],
[9285,38,5],[9285,40,3],[9285,43,3],[9285,44,12],[9285,47,3],
[9285,48,2],[9285,50,5],[9285,51,1],[9285,56,3],[9285,61,1],
[9285,62,1],[9285,63,1],[9285,66,19],[9285,68,2],[9285,70,2],
[9285,72,1],[9285,75,2],[9285,76,1],[9286,10,1],[9286,34,3],
[9287,18,1],[9287,22,5],[9287,25,2],[9287,27,6],[9287,28,3],
[9287,30,5],[9287,31,1],[9287,33,19],[9287,35,11],[9287,50,1],
[9287,52,13],[9287,63,1],[9287,74,1],[9288,2,3],[9288,20,1],
[9288,22,1],[9288,29,1],[9288,50,1],[9289,33,1],[9289,70,1],
[9290,2,4],[9290,3,1],[9290,23,1],[9290,24,1],[9290,31,1],[9290,38,1],
[9290,53,1],[9291,11,1],[9291,67,1],[9292,22,1],[9292,24,1],
[9293,17,1],[9293,53,1],[9294,43,1],[9294,59,1],[9295,3,2],
[9296,12,1],[9296,50,1],[9296,73,1],[9297,4,9],[9297,22,1],
[9297,24,2],[9297,38,4],[9297,39,1],[9297,44,3],[9297,45,1],
[9297,50,4],[9297,66,7],[9297,76,1],[9298,23,1],[9298,27,1],
[9299,1,51],[9299,2,102],[9299,4,2],[9299,5,1],[9299,6,1],[9299,7,1],
[9299,9,1],[9299,11,1],[9299,12,1],[9299,13,12],[9299,14,1],
[9299,15,3],[9299,16,1],[9299,18,1],[9299,19,2],[9299,20,9],
[9299,21,33],[9299,22,2],[9299,23,231],[9299,24,232],[9299,25,6],
[9299,26,108],[9299,27,14],[9299,28,19],[9299,29,5],[9299,30,8],
[9299,31,269],[9299,32,3],[9299,33,152],[9299,34,1],[9299,35,7],
[9299,36,1],[9299,37,3],[9299,40,1],[9299,41,3],[9299,42,60],
[9299,43,162],[9299,44,1],[9299,46,1],[9299,47,12],[9299,48,4],
[9299,50,1],[9299,52,2],[9299,53,1],[9299,54,1],[9299,56,3],
[9299,57,2],[9299,58,1],[9299,59,6],[9299,60,1],[9299,61,1],
[9299,62,3],[9299,63,2],[9299,65,1],[9299,66,1],[9299,67,1],
[9299,68,4],[9299,70,10],[9299,72,1],[9299,73,1],[9299,74,2],
[9299,75,1],[9299,76,2],[9299,77,1],[9299,78,1],[9300,32,2],
[9301,24,2],[9301,27,1],[9301,35,1],[9301,47,3],[9301,51,2],
[9301,53,1],[9301,55,1],[9301,56,1],[9301,66,1],[9301,68,1],
[9302,2,1],[9302,21,1],[9302,27,1],[9302,28,3],[9302,31,1],
[9302,44,1],[9302,50,1],[9302,66,1],[9303,2,8],[9303,8,1],[9303,28,1],
[9303,31,1],[9303,53,1],[9304,3,1],[9304,24,1],[9304,27,1],
[9304,47,1],[9305,20,1],[9305,21,1],[9305,22,1],[9305,32,1],
[9305,43,4],[9305,68,1],[9306,13,1],[9306,58,1],[9307,28,1],
[9307,66,1],[9308,27,2],[9309,1,1],[9309,8,1],[9310,4,1],[9310,5,1],
[9310,11,1],[9310,22,1],[9310,27,1],[9310,28,1],[9310,42,1],
[9310,53,1],[9310,63,11],[9310,68,3],[9310,70,1],[9310,72,1],
[9310,74,10],[9310,75,5],[9310,76,34],[9311,20,2],[9312,15,1],
[9312,53,3],[9312,55,1],[9313,4,1],[9313,23,1],[9313,25,1],
[9313,26,1],[9313,28,1],[9313,35,1],[9313,38,2],[9313,40,1],
[9313,50,1],[9313,66,1],[9313,68,1],[9313,76,1],[9314,2,2],[9315,2,1],
[9315,23,1],[9316,7,1],[9316,15,1],[9316,19,1],[9316,48,1],
[9316,59,1],[9316,60,1],[9316,73,2],[9317,2,1],[9317,56,1],
[9317,62,1],[9318,31,1],[9318,42,1],[9318,43,1],[9319,23,2],

[9319,27,1],[9319,32,1],[9319,37,1],[9320,31,2],[9321,53,1],
[9321,54,1],[9322,1,1],[9322,2,4],[9322,3,4],[9322,17,1],[9322,19,1],
[9322,22,3],[9322,23,7],[9322,25,1],[9322,26,2],[9322,27,11],
[9322,31,4],[9322,39,2],[9322,47,2],[9322,53,1],[9322,59,1],
[9322,63,1],[9322,68,1],[9322,76,1],[9323,23,1],[9323,24,1],
[9323,26,1],[9324,23,1],[9324,26,1],[9324,28,1],[9324,32,1],
[9324,43,4],[9325,62,10],[9326,62,4],[9327,2,2],[9328,22,1],
[9328,23,1],[9329,40,3],[9329,70,1],[9330,3,2],[9331,0,1],[9331,10,4],
[9331,12,6],[9331,13,1],[9331,15,1],[9331,16,1],[9331,18,1],
[9331,19,2],[9331,20,2],[9331,23,1],[9331,36,1],[9331,39,2],
[9331,41,1],[9331,45,1],[9331,46,2],[9331,65,1],[9331,67,1],
[9331,73,3],[9332,23,1],[9332,28,1],[9332,47,1],[9333,1,1],[9333,3,1],
[9333,16,1],[9333,20,1],[9333,22,2],[9333,23,2],[9333,25,2],
[9333,27,2],[9333,35,4],[9333,56,2],[9333,62,1],[9334,21,2],
[9334,25,28],[9334,28,2],[9334,31,2],[9334,43,8],[9335,24,1],
[9335,28,2],[9335,32,1],[9336,23,2],[9336,31,3],[9337,3,1],
[9337,15,1],[9337,25,1],[9337,27,1],[9337,32,5],[9337,34,1],
[9337,38,8],[9337,53,1],[9337,56,7],[9337,63,1],[9337,70,1],
[9337,72,1],[9338,20,1],[9338,53,1],[9339,23,1],[9339,28,2],
[9339,75,1],[9339,76,1],[9340,44,2],[9341,22,2],[9342,30,1],
[9342,39,1],[9343,27,1],[9343,35,2],[9343,48,1],[9344,28,7],
[9344,74,1],[9345,2,1],[9345,10,1],[9345,29,1],[9345,30,1],
[9345,49,1],[9345,65,1],[9345,68,3],[9345,77,9],[9346,1,2],[9346,3,1],
[9346,9,2],[9346,10,1],[9346,12,1],[9346,13,1],[9346,41,1],
[9346,57,1],[9346,62,1],[9347,6,1],[9347,28,1],[9347,35,1],
[9347,69,1],[9348,4,2],[9348,17,2],[9348,25,1],[9348,28,1],
[9348,44,1],[9348,53,3],[9348,74,1],[9349,22,2],[9349,28,1],
[9350,21,1],[9350,66,1],[9351,44,2],[9351,66,1],[9352,2,1],
[9352,20,3],[9352,26,1],[9352,27,4],[9352,28,1],[9352,30,2],
[9352,39,1],[9352,42,1],[9352,43,1],[9353,0,3],[9353,1,6],[9353,2,2],
[9353,3,2],[9353,4,7],[9353,5,4],[9353,7,3],[9353,8,1],[9353,9,8],
[9353,10,17],[9353,11,2],[9353,12,5],[9353,13,3],[9353,15,73],
[9353,16,4],[9353,17,36],[9353,18,3],[9353,19,11],[9353,20,2],
[9353,22,1],[9353,23,3],[9353,27,1],[9353,28,14],[9353,30,2],
[9353,32,2],[9353,33,1],[9353,34,1],[9353,35,2],[9353,36,1],
[9353,37,5],[9353,38,8],[9353,39,2],[9353,41,6],[9353,43,2],
[9353,44,41],[9353,45,9],[9353,46,2],[9353,48,2],[9353,49,3],
[9353,50,15],[9353,51,1],[9353,53,30],[9353,54,2],[9353,55,271],
[9353,56,1],[9353,57,4],[9353,58,4],[9353,59,8],[9353,60,1],
[9353,63,1],[9353,64,2],[9353,65,3],[9353,66,38],[9353,67,3],
[9353,69,2],[9353,70,3],[9353,71,7],[9353,72,2],[9353,73,6],
[9353,74,1],[9353,75,1],[9353,76,2],[9353,77,7],[9353,78,1],
[9354,9,1],[9354,15,3],[9354,37,1],[9354,67,1],[9355,25,3],[9356,1,7],
[9356,2,1],[9356,3,6],[9356,4,1],[9356,5,3],[9356,6,1],[9356,7,1],
[9356,8,2],[9356,10,1],[9356,12,2],[9356,13,7],[9356,15,3],
[9356,16,1],[9356,18,2],[9356,19,2],[9356,20,21],[9356,22,4],
[9356,23,25],[9356,24,24],[9356,25,2],[9356,26,4],[9356,27,8],
[9356,28,14],[9356,30,4],[9356,31,4],[9356,32,11],[9356,33,3],
[9356,34,2],[9356,35,7],[9356,37,1],[9356,38,1],[9356,39,1],
[9356,40,1],[9356,42,4],[9356,43,4],[9356,44,2],[9356,45,1],

[9356,46,2],[9356,47,2],[9356,49,1],[9356,55,1],[9356,56,3],
[9356,58,2],[9356,59,2],[9356,61,2],[9356,62,5],[9356,65,2],
[9356,67,1],[9356,68,1],[9356,70,1],[9356,71,1],[9356,73,1],
[9356,77,1],[9356,78,1],[9357,6,1],[9357,42,1],[9357,56,1],
[9357,68,1],[9357,69,1],[9358,25,1],[9358,26,1],[9358,32,3],
[9358,35,1],[9359,32,1],[9359,42,1],[9359,56,4],[9360,0,1],
[9360,1,27],[9360,7,2],[9360,8,2],[9360,12,9],[9360,17,4],[9360,18,2],
[9360,20,1],[9360,29,16],[9360,34,2],[9360,36,1],[9360,39,2],
[9360,53,3],[9360,73,1],[9361,20,12],[9361,26,3],[9361,28,1],
[9361,43,17],[9361,51,1],[9361,56,1],[9361,61,1],[9361,65,1],
[9361,68,3],[9361,70,1],[9361,72,2],[9362,24,1],[9362,28,1],
[9363,12,1],[9363,28,1],[9364,3,1],[9364,43,1],[9364,48,1],[9365,2,3],
[9365,22,1],[9365,31,4],[9366,2,2],[9366,32,7],[9367,13,1],
[9367,50,1],[9368,17,1],[9368,55,1],[9369,2,1],[9369,28,1],
[9370,26,2],[9370,31,1],[9371,44,1],[9371,50,1],[9372,26,2],
[9372,28,1],[9373,27,1],[9373,28,1],[9373,32,3],[9374,0,1],
[9374,27,1],[9374,62,1],[9375,3,6],[9375,6,1],[9375,23,19],
[9375,24,24],[9375,26,1],[9375,28,2],[9375,48,1],[9375,56,1],
[9376,2,7],[9377,24,2],[9377,27,1],[9377,28,1],[9377,32,1],
[9377,33,5],[9377,60,1],[9377,63,1],[9378,11,1],[9378,34,1],
[9378,53,1],[9378,59,1],[9379,2,3],[9379,34,2],[9380,2,1],[9380,23,1],
[9380,26,1],[9380,27,71],[9380,28,2],[9380,31,1],[9380,32,2],
[9380,33,1],[9380,35,1],[9380,43,1],[9380,47,1],[9380,70,1],
[9381,53,2],[9381,55,1],[9382,20,1],[9382,34,1],[9382,53,1],
[9383,44,1],[9383,66,1],[9384,2,1],[9384,5,1],[9384,6,1],[9384,23,1],
[9384,35,1],[9385,24,1],[9385,27,3],[9385,28,3],[9385,32,4],
[9385,35,1],[9385,47,1],[9386,10,1],[9386,11,1],[9386,13,1],
[9386,16,1],[9386,18,1],[9386,20,1],[9386,30,1],[9386,46,1],
[9386,55,1],[9386,57,1],[9386,65,2],[9387,3,1],[9387,5,3],[9387,6,1],
[9387,14,1],[9387,20,1],[9387,24,1],[9387,27,1],[9387,34,1],
[9387,43,1],[9387,51,2],[9387,52,1],[9387,56,1],[9387,61,1],
[9387,62,1],[9387,68,1],[9387,70,1],[9387,74,2],[9388,15,1],
[9388,17,1],[9388,53,1],[9388,55,2],[9389,31,1],[9389,62,1],
[9390,28,35],[9390,63,1],[9391,27,2],[9391,49,1],[9391,72,1],
[9392,70,2],[9393,57,1],[9393,64,1],[9394,15,4],[9394,31,9],
[9394,33,3],[9394,72,1],[9395,35,1],[9395,62,1],[9396,33,6],
[9396,47,1],[9397,23,2],[9397,27,2],[9397,28,1],[9397,33,5],
[9397,56,1],[9398,1,2],[9398,10,1],[9398,14,2],[9398,15,1],
[9398,16,1],[9398,20,1],[9398,41,3],[9398,45,1],[9398,55,3],
[9398,60,1],[9398,66,1],[9398,78,1],[9399,17,1],[9399,27,1],
[9400,27,1],[9400,31,1],[9400,59,2],[9401,21,1],[9401,28,3],
[9401,70,1],[9402,28,1],[9402,43,2],[9403,27,2],[9404,1,1],
[9404,2,55],[9404,12,2],[9404,21,14],[9404,23,2],[9404,24,2],
[9404,26,3],[9404,27,7],[9404,28,5],[9404,31,39],[9404,32,2],
[9404,34,2],[9404,42,62],[9404,48,5],[9404,66,1],[9405,42,2],
[9406,4,1],[9406,65,1],[9407,2,1],[9407,43,1],[9408,10,1],[9408,23,2],
[9408,26,1],[9408,31,1],[9408,42,1],[9409,6,1],[9409,28,1],
[9409,42,2],[9410,39,2],[9411,28,10],[9412,2,407],[9412,10,1],
[9412,17,2],[9412,21,1],[9412,22,1],[9412,23,1],[9412,24,2],
[9412,26,1],[9412,27,4],[9412,28,11],[9412,29,1],[9412,30,2],

[9412,31,36],[9412,33,1],[9412,34,1],[9412,39,2],[9412,41,1],
[9412,42,1],[9412,47,1],[9412,56,2],[9412,69,1],[9412,71,1],
[9413,0,1],[9413,1,1],[9413,2,2],[9413,3,2],[9413,4,18],[9413,6,2],
[9413,7,1],[9413,9,3],[9413,10,3],[9413,11,1],[9413,12,6],[9413,13,5],
[9413,14,2],[9413,15,66],[9413,16,1],[9413,17,47],[9413,19,2],
[9413,20,6],[9413,21,3],[9413,22,2],[9413,23,6],[9413,24,5],
[9413,25,4],[9413,26,4],[9413,27,5],[9413,28,33],[9413,29,4],
[9413,30,2],[9413,31,6],[9413,32,1],[9413,33,2],[9413,34,1],
[9413,35,5],[9413,36,1],[9413,38,7],[9413,39,1],[9413,40,2],
[9413,41,2],[9413,42,1],[9413,44,6],[9413,45,1],[9413,46,1],
[9413,47,2],[9413,48,2],[9413,49,1],[9413,50,19],[9413,53,56],
[9413,55,64],[9413,57,1],[9413,58,2],[9413,61,1],[9413,62,1],
[9413,63,2],[9413,65,1],[9413,66,11],[9413,68,4],[9413,70,7],
[9413,71,1],[9413,72,3],[9413,74,4],[9413,75,1],[9413,76,2],
[9414,2,10],[9414,31,1],[9414,55,1],[9415,8,1],[9415,20,1],
[9415,22,1],[9415,26,1],[9415,31,1],[9415,42,1],[9415,55,1],
[9415,56,1],[9415,72,1],[9416,10,1],[9416,37,1],[9417,1,2],[9417,9,1],
[9417,10,2],[9417,11,1],[9417,16,1],[9417,20,1],[9417,29,1],
[9417,36,1],[9417,45,1],[9417,65,1],[9417,67,1],[9417,71,1],
[9418,2,1],[9418,13,1],[9418,77,1],[9419,4,1],[9419,5,1],[9419,25,1],
[9419,26,1],[9420,2,1],[9420,23,1],[9421,27,2],[9421,31,30],
[9421,50,1],[9422,27,4],[9422,31,1],[9422,62,1],[9423,5,2],[9424,2,6],
[9424,3,2],[9424,6,1],[9424,15,1],[9424,16,1],[9424,19,1],[9424,27,1],
[9424,30,2],[9424,31,1],[9424,43,1],[9424,47,1],[9424,60,1],
[9425,4,1],[9425,44,1],[9425,66,2],[9426,63,1],[9426,76,1],
[9427,44,2],[9427,66,1],[9428,28,1],[9428,31,2],[9429,9,1],
[9429,28,3],[9429,74,1],[9430,24,1],[9430,68,1],[9431,24,1],
[9431,27,1],[9432,3,2],[9432,12,1],[9432,15,1],[9432,30,1],
[9432,60,1],[9433,2,2],[9433,28,2],[9433,72,1],[9434,32,1],
[9434,39,1],[9435,2,3],[9436,25,11],[9437,39,1],[9437,55,1],
[9438,3,1],[9438,27,4],[9438,50,1],[9438,51,3],[9439,5,1],[9439,24,4],
[9439,26,2],[9440,8,1],[9440,31,1],[9441,4,1],[9441,26,2],[9441,27,9],
[9441,42,4],[9441,43,2],[9442,40,1],[9442,61,1],[9443,1,2],
[9443,13,1],[9443,15,1],[9443,20,3],[9443,55,1],[9443,65,1],
[9443,67,1],[9443,73,2],[9444,24,1],[9444,56,3],[9445,2,4],
[9446,48,3],[9447,1,1],[9447,7,2],[9447,9,2],[9447,10,5],[9447,11,2],
[9447,13,1],[9447,15,1],[9447,16,3],[9447,18,2],[9447,19,4],
[9447,20,1],[9447,37,1],[9447,41,2],[9447,45,3],[9447,46,2],
[9447,53,1],[9447,57,1],[9447,58,3],[9447,59,1],[9447,60,2],
[9447,65,3],[9447,67,1],[9447,71,3],[9447,73,1],[9447,77,1],
[9447,78,1],[9448,2,4],[9448,22,1],[9448,26,1],[9448,35,1],[9449,8,2],
[9450,23,1],[9450,24,1],[9450,25,2],[9450,28,3],[9450,35,1],
[9450,58,1],[9450,70,1],[9450,77,2],[9451,15,1],[9451,55,1],
[9452,34,6],[9452,75,1],[9453,20,1],[9453,21,1],[9453,24,2],
[9453,52,1],[9454,62,3],[9455,55,2],[9456,0,7],[9456,1,8],[9456,7,4],
[9456,8,13],[9456,9,4],[9456,10,1],[9456,12,11],[9456,13,7],
[9456,14,1],[9456,15,11],[9456,17,41],[9456,20,4],[9456,23,2],
[9456,24,1],[9456,25,1],[9456,28,7],[9456,29,3],[9456,33,1],
[9456,34,5],[9456,35,1],[9456,36,2],[9456,37,2],[9456,38,1],
[9456,39,5],[9456,44,3],[9456,45,1],[9456,53,37],[9456,55,26],

[9456,59,1],[9456,62,1],[9456,66,1],[9456,73,2],[9457,5,1],
[9457,22,1],[9457,24,1],[9457,28,1],[9457,35,1],[9457,43,1],
[9457,47,1],[9457,51,1],[9457,56,1],[9458,13,7],[9458,39,1],
[9458,57,1],[9459,36,2],[9460,3,1],[9460,51,6],[9461,13,1],
[9461,30,1],[9461,33,4],[9461,42,1],[9462,1,1],[9462,9,1],[9463,41,1],
[9463,59,1],[9464,2,1],[9464,24,1],[9464,28,1],[9464,43,1],
[9465,27,1],[9465,74,3],[9466,24,2],[9467,1,5],[9467,2,2],[9467,3,1],
[9467,5,10],[9467,6,1],[9467,9,2],[9467,12,3],[9467,13,8],[9467,14,1],
[9467,15,18],[9467,16,1],[9467,19,1],[9467,20,1],[9467,21,3],
[9467,22,2],[9467,23,2],[9467,24,7],[9467,26,6],[9467,27,1],
[9467,28,1],[9467,31,3],[9467,32,3],[9467,33,1],[9467,34,1],
[9467,35,3],[9467,37,1],[9467,39,1],[9467,41,1],[9467,42,2],
[9467,46,2],[9467,47,1],[9467,50,2],[9467,51,2],[9467,53,1],
[9467,55,11],[9467,56,1],[9467,57,1],[9467,62,2],[9467,63,2],
[9467,65,1],[9467,66,3],[9467,68,1],[9467,70,1],[9467,74,1],
[9467,76,2],[9468,74,1],[9468,76,2],[9469,27,2],[9470,39,1],
[9470,42,3],[9470,43,5],[9471,23,1],[9471,24,9],[9471,27,1],
[9472,5,4],[9472,21,1],[9472,22,2],[9472,23,1],[9473,0,1],[9473,1,2],
[9473,5,2],[9473,7,1],[9473,9,4],[9473,10,6],[9473,11,6],[9473,12,6],
[9473,15,1],[9473,16,4],[9473,17,1],[9473,18,5],[9473,19,16],
[9473,20,2],[9473,24,4],[9473,25,2],[9473,27,6],[9473,28,5],
[9473,29,2],[9473,30,4],[9473,32,3],[9473,34,10],[9473,35,4],
[9473,36,6],[9473,37,3],[9473,39,3],[9473,41,6],[9473,42,1],
[9473,45,7],[9473,49,1],[9473,53,1],[9473,54,1],[9473,55,1],
[9473,57,9],[9473,58,3],[9473,59,6],[9473,60,2],[9473,62,2],
[9473,65,9],[9473,66,1],[9473,67,5],[9473,71,4],[9473,73,3],
[9473,74,2],[9473,76,1],[9473,77,5],[9473,78,1],[9474,27,1],
[9474,56,1],[9475,31,3],[9476,1,1],[9476,10,1],[9476,14,1],
[9476,19,1],[9476,37,1],[9476,41,1],[9476,58,1],[9476,67,1],
[9476,77,1],[9477,4,1],[9477,18,1],[9477,23,1],[9477,28,1],
[9477,31,1],[9477,32,14],[9477,35,1],[9477,39,6],[9477,42,2],
[9477,52,1],[9477,65,1],[9478,3,1],[9478,23,1],[9479,28,1],
[9479,42,1],[9479,61,1],[9480,10,1],[9480,12,1],[9480,15,1],
[9480,18,1],[9480,31,3],[9480,41,1],[9480,46,1],[9480,60,1],
[9480,64,1],[9481,2,1],[9481,5,1],[9481,22,1],[9481,24,1],[9481,25,1],
[9481,26,1],[9481,27,1],[9481,31,3],[9481,33,1],[9481,38,1],
[9481,40,1],[9481,44,1],[9481,48,1],[9481,56,1],[9481,63,34],
[9481,66,1],[9481,69,2],[9481,70,1],[9481,74,54],[9481,75,29],
[9481,76,65],[9482,8,3],[9482,17,1],[9482,25,1],[9482,27,1],
[9482,28,7],[9482,33,59],[9482,35,26],[9482,38,4],[9482,52,41],
[9483,2,2],[9483,22,1],[9483,23,2],[9483,24,1],[9483,25,3],
[9483,26,1],[9483,27,4],[9483,31,1],[9483,35,1],[9483,76,1],
[9484,28,1],[9484,38,2],[9484,44,10],[9484,66,4],[9485,2,2],
[9486,24,2],[9486,27,4],[9486,32,1],[9486,33,1],[9486,49,1],
[9486,56,1],[9486,63,1],[9487,2,3],[9487,31,3],[9488,25,2],
[9488,32,1],[9489,2,1],[9489,62,1],[9490,3,1],[9490,7,1],[9490,22,1],
[9490,23,12],[9490,26,2],[9490,27,2],[9490,31,4],[9490,33,1],
[9490,43,3],[9490,74,25],[9490,76,1],[9491,2,1],[9491,20,2],
[9491,27,1],[9492,28,2],[9492,68,1],[9493,22,1],[9493,26,1],
[9493,28,1],[9494,4,1],[9494,15,6],[9494,17,4],[9494,53,2],

[9494,55,8],[9495,2,1],[9495,13,1],[9496,13,1],[9496,29,2],
[9497,45,1],[9497,73,1],[9498,23,1],[9498,28,1],[9498,32,4],
[9498,62,12],[9499,28,1],[9499,70,1],[9500,12,2],[9501,29,2],
[9502,22,1],[9502,28,1],[9502,74,1],[9502,75,1],[9503,2,2],[9503,5,1],
[9503,21,1],[9503,22,2],[9503,23,6],[9503,26,2],[9503,27,4],
[9503,28,3],[9503,31,2],[9503,32,3],[9503,35,1],[9503,43,3],
[9503,44,1],[9503,76,2],[9504,27,1],[9504,30,1],[9505,28,23],
[9505,31,1],[9506,23,1],[9506,26,1],[9506,33,1],[9506,47,5],
[9507,1,1],[9507,8,1],[9507,24,1],[9507,27,49],[9507,32,4],
[9507,42,3],[9507,47,1],[9507,56,1],[9507,62,2],[9508,17,1],
[9508,40,1],[9509,1,1],[9509,9,1],[9509,77,1],[9510,22,2],[9511,2,2],
[9511,22,1],[9511,23,6],[9511,31,1],[9511,35,1],[9511,43,3],
[9511,45,1],[9511,46,1],[9511,62,2],[9512,5,1],[9512,11,2],
[9512,13,1],[9512,17,1],[9512,20,2],[9512,22,1],[9512,28,1],
[9512,35,1],[9512,43,1],[9512,53,1],[9512,65,1],[9512,67,1],
[9513,38,1],[9513,50,1],[9513,62,1],[9514,15,2],[9514,55,1],
[9515,5,18],[9515,27,1],[9515,33,29],[9515,47,1],[9515,56,1],
[9515,70,2],[9516,3,1],[9516,5,1],[9516,22,1],[9516,23,3],[9516,24,1],
[9516,26,1],[9516,27,15],[9516,28,3],[9516,32,1],[9516,35,13],
[9516,62,1],[9516,74,1],[9517,1,3],[9517,2,1],[9517,3,1],[9517,4,1],
[9517,5,3],[9517,15,1],[9517,18,1],[9517,20,1],[9517,21,1],
[9517,23,1],[9517,24,1],[9517,27,1],[9517,28,7],[9517,31,1],
[9517,33,6],[9517,35,1],[9517,42,2],[9517,48,2],[9517,66,1],
[9517,69,1],[9517,74,1],[9518,7,1],[9518,12,1],[9518,54,1],
[9518,59,1],[9519,2,1],[9519,7,1],[9519,15,1],[9519,31,2],
[9520,28,21],[9520,66,1],[9521,1,1],[9521,13,1],[9521,20,3],
[9521,23,1],[9521,24,2],[9521,27,2],[9521,32,1],[9521,42,1],
[9521,56,2],[9521,59,1],[9521,65,1],[9521,69,2],[9522,1,2],[9522,3,2],
[9522,4,1],[9522,8,6],[9522,9,1],[9522,10,3],[9522,11,1],[9522,13,1],
[9522,14,2],[9522,15,1],[9522,19,2],[9522,20,7],[9522,22,2],
[9522,23,4],[9522,24,8],[9522,25,6],[9522,27,1],[9522,28,1],
[9522,32,2],[9522,34,2],[9522,37,9],[9522,39,1],[9522,42,2],
[9522,45,1],[9522,46,6],[9522,47,1],[9522,54,1],[9522,55,1],
[9522,59,9],[9522,62,1],[9522,63,1],[9522,65,1],[9522,69,1],
[9522,71,1],[9522,73,1],[9522,76,1],[9522,77,1],[9522,78,1],
[9523,1,1],[9523,3,2],[9523,5,2],[9523,27,1],[9523,33,4],[9523,35,1],
[9523,44,1],[9523,47,3],[9523,48,1],[9523,51,1],[9523,56,1],
[9523,62,1],[9523,66,2],[9523,70,1],[9524,28,2],[9524,32,1],
[9524,40,1],[9524,42,1],[9524,56,4],[9524,62,3],[9525,2,10],
[9526,13,1],[9526,51,1],[9527,3,1],[9527,11,1],[9527,30,1],
[9527,42,3],[9528,25,3],[9528,59,1],[9528,76,1],[9529,22,1],
[9529,26,1],[9529,44,2],[9530,8,1],[9530,22,1],[9531,27,2],
[9531,35,1],[9532,0,1],[9532,1,8],[9532,2,1],[9532,3,1],[9532,9,15],
[9532,12,1],[9532,13,3],[9532,20,3],[9532,24,4],[9532,28,27],
[9532,32,1],[9532,38,1],[9532,39,3],[9532,45,1],[9532,59,2],
[9532,64,1],[9532,74,1],[9532,77,1],[9533,19,1],[9533,21,1],
[9533,27,3],[9533,39,1],[9533,41,1],[9533,73,1],[9534,28,1],
[9534,44,1],[9535,3,2],[9535,6,1],[9535,35,1],[9535,42,1],[9535,46,1],
[9536,3,1],[9536,22,1],[9536,28,1],[9536,32,1],[9536,47,1],
[9536,60,1],[9536,68,1],[9537,1,1],[9537,3,1],[9537,21,1],[9537,23,6],

[9537,27,1],[9537,28,3],[9537,30,1],[9537,32,2],[9537,37,3],
[9538,3,2],[9538,4,1],[9538,6,1],[9538,23,2],[9538,26,1],[9538,28,2],
[9538,74,1],[9538,79,1],[9539,28,1],[9539,55,1],[9540,10,1],
[9540,19,1],[9540,20,13],[9540,22,6],[9540,23,4],[9540,24,4],
[9540,25,1],[9540,26,3],[9540,27,1],[9540,28,1],[9540,32,2],
[9540,35,2],[9540,39,4],[9540,42,1],[9540,43,1],[9540,48,1],
[9540,57,1],[9540,78,1],[9541,47,2],[9541,61,1],[9541,74,3],
[9542,4,1],[9542,50,1],[9543,24,2],[9544,3,1],[9544,7,1],[9544,20,6],
[9544,24,7],[9544,27,1],[9544,28,1],[9544,33,8],[9544,34,1],
[9544,35,1],[9544,56,1],[9544,65,1],[9545,1,1],[9545,13,2],
[9545,20,1],[9545,64,1],[9545,77,2],[9546,1,2],[9546,6,1],[9546,9,1],
[9546,10,1],[9546,13,2],[9546,15,2],[9546,18,1],[9546,20,1],
[9546,21,1],[9546,23,1],[9546,24,6],[9546,26,2],[9546,27,1],
[9546,28,3],[9546,32,1],[9546,33,1],[9546,34,1],[9546,39,1],
[9546,42,1],[9546,43,2],[9546,55,2],[9547,40,1],[9547,62,1],
[9548,16,4],[9548,20,1],[9548,23,1],[9548,26,6],[9548,31,1],
[9548,43,1],[9549,6,13],[9549,23,1],[9549,24,1],[9549,26,1],
[9549,27,1],[9549,28,1],[9549,43,3],[9549,47,1],[9549,52,1],
[9549,56,4],[9549,66,1],[9549,74,3],[9550,24,1],[9550,27,1],
[9551,23,1],[9551,33,2],[9552,27,1],[9552,42,1],[9553,25,1],
[9553,66,1],[9553,72,1],[9553,74,1],[9554,23,4],[9554,47,2],
[9554,48,1],[9554,68,1],[9555,27,1],[9555,28,1],[9555,40,1],
[9555,69,1],[9556,7,3],[9557,2,1],[9557,3,1],[9557,7,1],[9557,9,3],
[9557,10,2],[9557,12,6],[9557,13,4],[9557,15,1],[9557,16,3],
[9557,17,1],[9557,18,2],[9557,19,3],[9557,21,1],[9557,27,13],
[9557,28,1],[9557,31,1],[9557,33,2],[9557,37,1],[9557,38,12],
[9557,39,2],[9557,40,1],[9557,41,1],[9557,42,1],[9557,44,1],
[9557,45,1],[9557,54,2],[9557,55,1],[9557,56,1],[9557,57,1],
[9557,58,3],[9557,60,1],[9557,64,1],[9557,65,3],[9557,67,1],
[9557,68,1],[9557,70,1],[9557,73,1],[9557,75,1],[9557,77,1],
[9557,78,1],[9558,12,1],[9558,19,1],[9558,21,1],[9559,48,1],
[9559,69,1],[9559,77,2],[9560,43,4],[9561,22,3],[9562,10,1],
[9562,41,1],[9562,65,1],[9563,9,1],[9563,48,1],[9563,71,1],[9564,4,1],
[9564,28,1],[9564,44,1],[9565,40,2],[9566,23,1],[9566,27,1],
[9567,32,2],[9568,3,1],[9568,56,2],[9569,15,1],[9569,53,1],
[9569,69,1],[9570,26,2],[9570,27,1],[9570,28,1],[9570,35,2],
[9570,39,1],[9570,42,1],[9570,43,1],[9571,35,2],[9571,41,1],
[9572,23,1],[9572,26,1],[9572,27,1],[9572,31,1],[9573,6,3],
[9573,22,3],[9573,26,1],[9573,27,3],[9573,33,2],[9573,38,1],
[9573,42,1],[9573,47,1],[9573,51,1],[9573,56,1],[9573,66,1],
[9574,5,1],[9574,31,1],[9575,28,2],[9575,50,1],[9576,22,1],
[9576,26,7],[9576,38,1],[9576,43,13],[9576,70,1],[9577,28,1],
[9577,35,5],[9578,28,2],[9579,31,1],[9579,35,1],[9579,38,2],
[9579,44,1],[9579,47,1],[9579,51,2],[9579,66,1],[9579,68,1],
[9579,69,2],[9580,26,1],[9580,28,2],[9581,3,1],[9581,4,2],[9581,5,8],
[9581,6,1],[9581,22,2],[9581,23,4],[9581,24,1],[9581,25,4],
[9581,26,3],[9581,27,3],[9581,28,9],[9581,31,1],[9581,32,19],
[9581,35,1],[9581,38,1],[9581,40,1],[9581,41,1],[9581,42,1],
[9581,43,3],[9581,44,6],[9581,47,1],[9581,48,6],[9581,50,1],
[9581,51,1],[9581,52,1],[9581,56,10],[9581,61,220],[9581,62,1],

[9581,63,2],[9581,65,1],[9581,66,2],[9581,68,2],[9581,69,2],
[9581,70,5],[9581,73,1],[9581,74,2],[9581,76,2],[9582,22,1],
[9582,26,1],[9582,27,1],[9582,28,2],[9583,1,1],[9583,12,1],
[9583,16,1],[9583,17,1],[9583,19,2],[9583,29,1],[9583,30,1],
[9583,36,1],[9583,53,1],[9583,67,1],[9584,20,1],[9584,42,2],
[9584,56,1],[9585,44,1],[9585,50,1],[9585,66,1],[9586,2,2],
[9586,28,3],[9587,25,1],[9587,42,1],[9588,5,1],[9588,25,1],
[9588,35,1],[9589,19,2],[9590,3,1],[9590,51,2],[9590,74,7],
[9591,37,3],[9592,34,1],[9592,41,1],[9592,45,1],[9592,57,1],
[9592,65,2],[9593,1,1],[9593,4,1],[9593,10,2],[9593,11,1],[9593,15,5],
[9593,17,3],[9593,36,1],[9593,40,1],[9593,41,1],[9593,45,1],
[9593,53,5],[9593,55,5],[9593,59,1],[9593,65,2],[9593,71,3],
[9594,13,2],[9594,21,3],[9594,43,1],[9595,9,3],[9595,28,2],
[9596,22,3],[9596,28,1],[9596,35,5],[9597,32,2],[9597,48,1],
[9598,30,1],[9598,33,12],[9598,35,7],[9598,38,1],[9598,52,2],
[9599,24,1],[9599,26,1],[9600,27,1],[9600,28,1],[9601,28,2],
[9602,27,1],[9602,28,1],[9602,33,1],[9602,35,1],[9602,38,1],
[9602,56,7],[9602,69,1],[9603,2,1],[9603,13,1],[9603,20,2],
[9603,27,1],[9603,30,1],[9603,43,1],[9603,47,1],[9603,61,1],
[9604,39,1],[9604,74,1],[9605,2,1],[9605,8,1],[9605,9,1],[9605,15,1],
[9605,16,1],[9605,20,2],[9605,22,1],[9605,30,2],[9605,37,3],
[9605,43,1],[9605,46,3],[9605,57,1],[9605,58,1],[9605,60,1],
[9605,65,1],[9605,75,1],[9606,25,1],[9606,30,1],[9606,33,3],
[9606,52,4],[9607,1,1],[9607,21,1],[9607,28,1],[9607,33,1],
[9607,38,1],[9607,48,1],[9608,23,1],[9608,25,1],[9608,35,1],
[9608,52,1],[9608,74,1],[9609,12,1],[9609,19,1],[9609,59,1],
[9610,2,3],[9610,5,2],[9610,20,1],[9610,21,1],[9610,22,1],[9610,23,1],
[9610,24,2],[9610,26,1],[9610,28,1],[9610,30,2],[9610,31,4],
[9610,35,1],[9610,38,1],[9610,42,3],[9610,43,4],[9610,59,1],
[9610,60,1],[9611,6,1],[9611,38,1],[9611,44,1],[9611,54,1],
[9611,66,1],[9612,5,2],[9612,20,2],[9612,21,1],[9612,23,1],
[9612,27,3],[9612,28,1],[9612,33,3],[9612,40,1],[9612,42,2],
[9612,51,1],[9612,62,2],[9612,68,1],[9612,70,3],[9612,74,1],
[9613,3,1],[9613,18,1],[9613,19,1],[9613,21,1],[9613,23,2],
[9613,24,115],[9613,28,4],[9613,33,1],[9613,38,1],[9613,50,2],
[9613,56,1],[9613,62,1],[9613,68,2],[9613,70,3],[9613,72,3],
[9614,10,2],[9614,11,1],[9614,57,1],[9614,58,1],[9615,3,3],[9615,5,1],
[9615,23,1],[9615,26,1],[9615,27,1],[9615,31,3],[9615,43,1],
[9615,56,1],[9616,12,2],[9617,55,1],[9617,62,1],[9618,11,1],
[9618,13,1],[9618,29,1],[9618,39,1],[9618,59,1],[9618,77,1],
[9619,8,2],[9619,10,1],[9619,32,1],[9619,35,1],[9619,38,1],
[9619,59,1],[9620,3,1],[9620,9,1],[9620,10,1],[9620,15,1],[9620,28,2],
[9620,30,2],[9620,31,1],[9620,70,1],[9621,44,1],[9621,50,2],
[9622,2,1],[9622,4,1],[9622,9,1],[9622,23,1],[9623,34,2],[9623,36,1],
[9623,73,1],[9624,0,1],[9624,1,20],[9624,2,35],[9624,3,12],[9624,4,1],
[9624,5,3],[9624,8,10],[9624,9,5],[9624,10,4],[9624,11,1],
[9624,12,17],[9624,13,118],[9624,15,3],[9624,16,2],[9624,17,1],
[9624,18,1],[9624,19,4],[9624,20,67],[9624,21,47],[9624,22,2],
[9624,23,64],[9624,24,2],[9624,25,3],[9624,26,160],[9624,27,9],
[9624,28,31],[9624,29,7],[9624,30,30],[9624,31,492],[9624,32,58],

[9624,33,16],[9624,34,3],[9624,35,5],[9624,36,1],[9624,37,1],
[9624,38,4],[9624,39,5],[9624,40,1],[9624,42,340],[9624,43,77],
[9624,44,1],[9624,45,4],[9624,46,1],[9624,47,39],[9624,48,41],
[9624,49,1],[9624,50,3],[9624,51,2],[9624,52,2],[9624,53,3],
[9624,54,1],[9624,56,1],[9624,57,3],[9624,58,2],[9624,59,1],
[9624,60,3],[9624,61,1],[9624,62,4],[9624,63,1],[9624,64,3],
[9624,65,6],[9624,67,1],[9624,68,5],[9624,69,1],[9624,70,8],
[9624,71,2],[9624,72,3],[9624,73,2],[9624,75,1],[9624,76,2],
[9624,77,2],[9624,78,6],[9625,56,2],[9626,31,4],[9627,28,1],
[9627,50,2],[9628,5,1],[9628,35,1],[9629,28,2],[9630,2,5],[9630,31,1],
[9630,47,1],[9630,50,1],[9631,26,2],[9631,38,1],[9631,43,4],
[9631,44,1],[9632,24,1],[9632,28,1],[9633,28,3],[9634,15,1],
[9634,19,1],[9635,40,9],[9635,56,9],[9635,61,2],[9636,2,1],
[9636,60,1],[9637,27,3],[9637,34,1],[9638,2,2],[9638,28,2],
[9639,51,1],[9639,56,3],[9640,8,1],[9640,9,1],[9640,17,1],[9640,24,1],
[9640,27,1],[9640,28,4],[9640,31,36],[9640,36,5],[9640,47,1],
[9640,53,1],[9640,56,1],[9640,60,1],[9640,64,1],[9640,67,1],
[9640,78,3],[9641,28,1],[9641,46,1],[9642,4,1],[9642,31,2],
[9643,32,3],[9644,23,1],[9644,24,3],[9644,27,5],[9644,28,2],
[9644,35,2],[9644,43,3],[9645,22,1],[9645,35,1],[9646,31,2],
[9647,2,1],[9647,28,1],[9647,31,1],[9648,3,1],[9648,42,1],[9648,51,1],
[9648,69,1],[9649,27,1],[9649,42,1],[9650,5,1],[9650,10,3],
[9650,25,1],[9650,27,1],[9650,34,1],[9650,35,1],[9650,41,3],
[9650,59,1],[9650,66,1],[9650,68,1],[9650,69,1],[9650,76,3],
[9651,15,2],[9651,66,1],[9652,1,1],[9652,3,2],[9652,13,1],[9652,19,1],
[9652,21,1],[9652,23,1],[9652,26,2],[9652,27,3],[9652,28,2],
[9652,35,2],[9652,52,1],[9652,58,1],[9653,28,31],[9654,2,1],
[9654,22,1],[9654,27,2],[9654,35,2],[9655,10,1],[9655,37,1],
[9655,53,1],[9655,67,1],[9656,11,1],[9656,15,1],[9656,17,1],
[9656,38,1],[9657,7,1],[9657,17,1],[9657,25,1],[9657,28,3],
[9657,37,4],[9657,39,1],[9657,53,1],[9658,3,2],[9658,35,1],
[9659,22,1],[9659,25,1],[9659,26,2],[9660,2,4],[9660,9,1],[9660,31,2],
[9661,2,1],[9661,22,1],[9661,23,1],[9661,62,9],[9662,4,2],[9662,5,1],
[9662,15,2],[9662,17,1],[9662,22,1],[9662,33,1],[9662,44,12],
[9662,50,2],[9662,55,2],[9662,66,5],[9663,3,2],[9663,33,1],
[9663,47,1],[9664,34,1],[9664,71,1],[9665,9,1],[9665,16,1],
[9666,51,1],[9666,70,1],[9667,4,1],[9667,53,4],[9668,10,1],
[9668,13,1],[9668,16,1],[9668,18,1],[9668,20,3],[9668,22,1],
[9668,39,1],[9668,77,3],[9669,27,1],[9669,29,1],[9670,25,1],
[9670,33,3],[9670,56,1],[9671,33,1],[9671,50,1],[9672,26,1],
[9672,28,1],[9672,31,1],[9672,43,1],[9673,42,2],[9674,2,2],
[9674,5,13],[9674,22,5],[9674,23,1],[9674,27,4],[9674,32,3],
[9674,35,1],[9674,38,1],[9674,44,2],[9674,50,1],[9674,69,1],
[9674,72,1],[9675,5,1],[9675,28,1],[9675,33,12],[9675,35,1],
[9675,66,1],[9676,12,1],[9676,67,1],[9677,25,1],[9677,44,1],
[9677,50,1],[9677,66,3],[9678,10,1],[9678,19,1],[9678,29,1],
[9678,36,1],[9678,59,1],[9678,65,1],[9678,73,3],[9678,77,1],
[9679,8,1],[9679,15,1],[9679,17,1],[9680,3,1],[9680,28,2],[9680,31,1],
[9680,35,2],[9681,1,95],[9681,2,7],[9681,4,1],[9681,5,2],[9681,8,1],
[9681,9,1],[9681,10,5],[9681,11,4],[9681,12,17],[9681,13,53],

[9681,15,1],[9681,16,2],[9681,17,1],[9681,19,1],[9681,20,9],
[9681,21,57],[9681,22,1],[9681,23,2],[9681,24,5],[9681,25,50],
[9681,26,136],[9681,28,7],[9681,29,30],[9681,31,82],[9681,32,1],
[9681,33,147],[9681,35,4],[9681,36,2],[9681,40,1],[9681,42,1],
[9681,43,79],[9681,50,1],[9681,52,6],[9681,53,1],[9681,55,1],
[9681,59,1],[9681,65,1],[9681,67,1],[9681,70,1],[9681,72,1],
[9681,73,1],[9681,75,1],[9682,25,3],[9682,33,1],[9682,42,1],
[9682,68,1],[9683,15,2],[9683,28,1],[9683,55,1],[9684,28,4],
[9685,13,1],[9685,28,3],[9685,30,1],[9685,31,1],[9685,34,1],
[9685,43,1],[9685,74,1],[9686,41,1],[9686,55,1],[9687,2,4],[9687,8,3],
[9687,28,1],[9687,47,1],[9687,56,1],[9687,70,1],[9688,24,1],
[9688,28,1],[9688,33,1],[9689,5,1],[9689,18,1],[9689,39,2],
[9689,52,1],[9689,59,3],[9689,62,3],[9690,2,1],[9690,3,1],[9690,6,1],
[9690,31,1],[9690,32,1],[9691,6,1],[9691,24,1],[9691,28,1],[9692,2,4],
[9693,7,1],[9693,9,3],[9693,10,9],[9693,11,4],[9693,12,1],[9693,13,2],
[9693,15,1],[9693,16,5],[9693,18,1],[9693,19,4],[9693,20,2],
[9693,29,2],[9693,41,4],[9693,45,2],[9693,46,1],[9693,53,1],
[9693,55,4],[9693,57,5],[9693,58,2],[9693,59,1],[9693,64,1],
[9693,65,1],[9693,73,1],[9693,77,1],[9693,78,1],[9694,10,1],
[9694,28,5],[9695,61,1],[9695,62,1],[9696,25,2],[9696,30,2],
[9696,33,1],[9696,35,1],[9696,52,5],[9696,65,1],[9696,71,1],
[9697,15,1],[9697,53,1],[9698,1,1],[9698,2,1],[9698,3,1],[9698,9,1],
[9698,10,1],[9698,12,1],[9698,13,1],[9698,19,1],[9698,20,1],
[9698,21,1],[9698,23,6],[9698,25,8],[9698,26,1],[9698,28,37],
[9698,31,11],[9698,33,36],[9698,39,5],[9698,42,3],[9698,43,1],
[9698,48,4],[9698,51,1],[9698,55,1],[9698,65,1],[9698,70,1],
[9698,78,2],[9699,1,2],[9699,4,3],[9699,5,1],[9699,8,1],[9699,9,4],
[9699,10,3],[9699,12,3],[9699,13,3],[9699,14,1],[9699,16,2],
[9699,17,27],[9699,19,1],[9699,20,1],[9699,22,1],[9699,24,1],
[9699,28,1],[9699,29,1],[9699,34,1],[9699,38,2],[9699,44,2],
[9699,45,1],[9699,50,9],[9699,53,36],[9699,54,1],[9699,55,2],
[9699,56,1],[9699,57,1],[9699,59,1],[9699,63,1],[9699,70,1],
[9699,78,1],[9700,27,2],[9700,28,3],[9701,3,1],[9701,32,2],
[9701,35,1],[9701,36,3],[9702,26,1],[9702,28,1],[9703,5,1],
[9703,27,1],[9703,28,1],[9703,35,1],[9703,40,1],[9704,53,1],
[9704,55,2],[9705,27,1],[9705,28,1],[9706,18,1],[9706,24,1],
[9706,48,1],[9706,50,1],[9706,61,1],[9706,63,1],[9706,72,1],
[9707,1,2],[9707,7,1],[9707,8,3],[9707,9,5],[9707,10,11],[9707,11,6],
[9707,12,1],[9707,13,1],[9707,14,1],[9707,15,2],[9707,16,5],
[9707,17,1],[9707,18,3],[9707,19,10],[9707,20,1],[9707,29,1],
[9707,30,1],[9707,34,1],[9707,36,4],[9707,37,2],[9707,39,4],
[9707,41,2],[9707,45,3],[9707,54,3],[9707,57,5],[9707,59,2],
[9707,60,1],[9707,65,6],[9707,67,4],[9707,71,2],[9707,73,3],
[9707,77,2],[9707,78,1],[9708,3,1],[9708,28,1],[9709,24,1],
[9709,27,2],[9709,33,2],[9709,44,1],[9709,53,1],[9709,59,1],
[9709,76,1],[9710,26,3],[9710,32,1],[9710,43,5],[9711,8,1],
[9711,53,1],[9712,23,2],[9713,0,1],[9713,1,2],[9713,3,1],[9713,7,1],
[9713,9,1],[9713,10,2],[9713,11,2],[9713,13,5],[9713,14,2],
[9713,15,5],[9713,16,3],[9713,18,2],[9713,19,3],[9713,20,3],
[9713,29,1],[9713,30,1],[9713,34,3],[9713,36,2],[9713,42,1],

[9713,45,1],[9713,49,2],[9713,53,4],[9713,54,1],[9713,57,1],
[9713,58,1],[9713,64,1],[9713,65,3],[9713,67,1],[9713,71,1],
[9713,73,1],[9713,77,1],[9714,13,2],[9714,78,1],[9715,1,1],[9715,6,1],
[9716,27,1],[9716,28,3],[9717,5,1],[9717,21,1],[9717,28,2],
[9718,66,1],[9718,74,1],[9719,16,1],[9719,53,1],[9720,24,1],
[9720,28,1],[9721,4,4],[9721,14,1],[9721,22,2],[9721,40,1],
[9721,42,2],[9721,45,1],[9721,47,1],[9721,50,3],[9721,57,1],
[9721,65,2],[9722,3,2],[9722,20,1],[9722,22,5],[9722,24,2],
[9722,25,1],[9722,27,2],[9722,31,1],[9722,32,2],[9722,33,3],
[9722,35,1],[9722,40,1],[9722,43,1],[9722,47,1],[9722,56,1],
[9722,68,3],[9723,22,1],[9723,35,1],[9724,60,1],[9724,64,1],
[9725,35,2],[9726,50,1],[9726,66,5],[9727,28,1],[9727,32,1],
[9727,74,5],[9728,2,2],[9728,3,3],[9728,21,2],[9728,27,1],[9728,28,7],
[9728,31,2],[9728,32,1],[9728,38,2],[9728,44,1],[9728,50,1],
[9728,66,1],[9728,74,215],[9728,75,1],[9729,33,3],[9730,3,4],
[9730,4,1],[9730,5,8],[9730,22,2],[9730,23,1],[9730,35,1],[9730,40,1],
[9730,50,1],[9730,56,1],[9730,62,2],[9730,63,2],[9731,36,1],
[9731,43,6],[9732,1,1],[9732,41,1],[9733,28,2],[9734,28,2],
[9735,30,1],[9735,55,1],[9735,78,1],[9736,22,1],[9736,43,1],
[9736,59,1],[9737,23,1],[9737,28,1],[9737,32,5],[9738,34,2],
[9739,9,1],[9739,35,2],[9739,60,1],[9739,62,2],[9740,25,2],[9741,6,1],
[9741,28,1],[9742,4,2],[9742,9,2],[9742,15,4],[9742,17,113],
[9742,50,5],[9742,53,183],[9742,55,49],[9743,2,1],[9743,27,3],
[9743,43,1],[9743,52,1],[9743,62,1],[9744,25,4],[9744,28,1],
[9745,35,5],[9746,0,2],[9746,8,1],[9747,23,1],[9747,24,3],[9747,27,1],
[9747,56,1],[9747,68,1],[9748,5,1],[9748,28,1],[9748,32,1],[9749,3,2],
[9749,5,2],[9749,23,2],[9749,24,1],[9749,25,2],[9749,33,1],
[9749,35,1],[9749,38,1],[9749,42,2],[9749,48,1],[9749,66,1],
[9750,28,1],[9750,47,2],[9751,66,2],[9751,74,1],[9752,2,1],
[9752,22,1],[9752,35,2],[9753,24,1],[9753,25,1],[9754,0,1],[9754,1,2],
[9754,4,10],[9754,5,3],[9754,7,1],[9754,10,2],[9754,12,1],[9754,13,2],
[9754,14,2],[9754,15,5],[9754,16,1],[9754,17,5],[9754,19,3],
[9754,20,1],[9754,22,2],[9754,23,7],[9754,28,4],[9754,29,1],
[9754,30,1],[9754,32,3],[9754,35,3],[9754,36,1],[9754,37,1],
[9754,39,1],[9754,43,1],[9754,44,1],[9754,45,1],[9754,46,1],
[9754,49,1],[9754,50,14],[9754,53,3],[9754,54,2],[9754,55,7],
[9754,57,2],[9754,58,2],[9754,59,2],[9754,60,1],[9754,64,1],
[9754,65,1],[9754,66,1],[9754,73,1],[9754,78,1],[9755,0,2],[9755,1,2],
[9755,2,1],[9755,9,4],[9755,10,1],[9755,13,5],[9755,17,2],[9755,19,1],
[9755,20,1],[9755,24,1],[9755,29,1],[9755,39,1],[9755,45,1],
[9755,53,1],[9755,55,3],[9755,65,1],[9755,67,2],[9755,73,1],
[9755,77,1],[9756,32,1],[9756,40,1],[9756,76,4],[9757,25,1],
[9757,26,1],[9757,42,1],[9758,5,1],[9758,26,1],[9758,46,1],[9759,2,1],
[9759,3,2],[9759,10,1],[9759,24,2],[9759,25,3],[9759,26,2],
[9759,27,4],[9759,28,23],[9759,30,5],[9759,33,26],[9759,35,13],
[9759,38,4],[9759,52,46],[9759,66,2],[9759,72,1],[9759,74,2],
[9759,75,2],[9760,1,1],[9760,2,1],[9760,3,3],[9760,21,1],[9760,23,4],
[9760,24,1],[9760,26,1],[9760,27,4],[9760,31,6],[9760,32,2],
[9760,43,1],[9760,60,1],[9760,76,1],[9761,2,4],[9761,16,8],
[9761,19,3],[9761,30,9],[9761,49,1],[9761,58,1],[9762,10,1],

[9762,28,4],[9762,40,1],[9762,42,1],[9762,61,1],[9762,69,2],
[9763,3,1],[9763,53,1],[9764,2,13],[9764,3,3],[9764,5,2],[9764,13,2],
[9764,23,1],[9764,24,1],[9764,27,1],[9764,28,2],[9764,42,1],
[9764,43,1],[9764,48,1],[9764,68,1],[9765,6,1],[9765,23,1],
[9765,24,7],[9765,56,10],[9766,3,1],[9766,24,1],[9766,43,1],
[9767,1,1],[9767,30,2],[9767,33,2],[9767,52,1],[9768,2,1],[9768,24,2],
[9768,25,1],[9768,26,6],[9768,27,1],[9768,28,1],[9768,32,22],
[9768,39,2],[9768,43,2],[9769,26,1],[9769,35,4],[9769,44,5],
[9769,50,1],[9769,66,4],[9769,72,1],[9770,6,1],[9770,8,2],[9770,20,1],
[9770,21,2],[9770,25,1],[9770,33,2],[9770,43,2],[9770,47,1],
[9770,50,1],[9771,0,1],[9771,1,4],[9771,2,3],[9771,4,41],[9771,5,1],
[9771,6,1],[9771,10,1],[9771,13,1],[9771,15,1],[9771,16,1],
[9771,19,2],[9771,20,2],[9771,23,1],[9771,26,2],[9771,27,1],
[9771,29,1],[9771,30,1],[9771,32,2],[9771,33,1],[9771,38,3],
[9771,40,2],[9771,42,1],[9771,44,72],[9771,45,1],[9771,46,2],
[9771,50,37],[9771,52,1],[9771,53,1],[9771,56,1],[9771,57,1],
[9771,61,1],[9771,62,1],[9771,66,35],[9771,69,1],[9771,72,1],
[9771,76,1],[9771,77,2],[9771,78,1],[9772,2,2],[9773,23,1],
[9773,60,1],[9774,6,9],[9774,22,4],[9774,23,6],[9774,24,34],
[9774,28,1],[9774,32,1],[9774,35,36],[9774,66,1],[9774,70,2],
[9775,1,2],[9775,24,6],[9775,30,1],[9775,43,1],[9776,23,1],
[9776,24,1],[9777,3,1],[9777,21,1],[9778,1,3],[9778,7,2],[9778,12,1],
[9778,13,1],[9778,15,1],[9778,17,1],[9778,20,32],[9778,21,1],
[9778,22,2],[9778,26,1],[9778,28,1],[9778,29,2],[9778,34,2],
[9778,35,1],[9778,39,2],[9778,45,1],[9778,53,1],[9778,55,1],
[9778,59,1],[9778,78,12],[9779,4,1],[9779,25,1],[9780,23,1],
[9780,31,1],[9780,43,2],[9781,21,1],[9781,22,1],[9781,25,1],
[9781,31,4],[9781,32,1],[9781,35,1],[9781,43,1],[9781,44,1],
[9781,47,1],[9781,51,2],[9781,56,1],[9781,62,1],[9781,66,3],
[9781,68,1],[9781,79,1],[9782,28,3],[9782,75,1],[9783,22,1],
[9783,70,1],[9784,1,1],[9784,7,1],[9784,8,1],[9784,9,3],[9784,10,5],
[9784,11,2],[9784,12,4],[9784,13,2],[9784,14,3],[9784,15,3],
[9784,16,4],[9784,17,2],[9784,19,3],[9784,20,3],[9784,27,1],
[9784,29,1],[9784,34,2],[9784,37,4],[9784,39,3],[9784,41,1],
[9784,45,3],[9784,54,2],[9784,55,1],[9784,58,1],[9784,59,1],
[9784,60,2],[9784,62,1],[9784,63,1],[9784,65,4],[9784,67,1],
[9784,71,1],[9784,73,1],[9784,75,1],[9784,77,4],[9785,31,3],
[9785,62,1],[9786,0,1],[9786,1,1],[9786,8,1],[9786,10,1],[9786,14,1],
[9786,16,1],[9786,17,1],[9786,28,1],[9786,41,2],[9786,51,1],
[9786,57,1],[9786,58,1],[9786,71,1],[9786,73,1],[9786,76,1],
[9786,77,1],[9787,5,1],[9787,23,2],[9787,42,1],[9787,59,4],
[9788,28,2],[9788,51,1],[9789,2,3],[9789,3,1],[9790,7,1],[9790,15,2],
[9791,48,2],[9792,1,1],[9792,2,6],[9792,7,1],[9792,10,1],[9792,12,1],
[9792,13,12],[9792,18,1],[9792,20,1],[9792,22,1],[9792,23,1],
[9792,26,1],[9792,30,1],[9792,35,2],[9792,49,1],[9792,57,1],
[9792,65,1],[9792,70,1],[9792,77,1],[9793,32,1],[9793,62,1],
[9793,66,1],[9793,75,1],[9794,63,2],[9794,76,3],[9795,25,1],
[9795,38,5],[9796,25,2],[9796,27,18],[9796,28,15],[9796,35,23],
[9796,47,1],[9797,28,5],[9797,47,1],[9798,3,1],[9798,28,1],
[9798,31,1],[9798,32,7],[9799,24,1],[9799,33,8],[9799,62,1],

[9800,25,1],[9800,28,1],[9801,2,1],[9801,6,1],[9801,22,2],[9801,23,2],
[9801,26,1],[9801,27,3],[9801,31,1],[9801,32,1],[9801,33,1],
[9801,35,1],[9801,42,4],[9801,47,1],[9801,50,1],[9801,52,2],
[9801,56,3],[9801,63,1],[9801,70,1],[9801,72,1],[9801,74,1],
[9801,75,1],[9802,15,1],[9802,26,1],[9803,27,1],[9803,28,1],
[9803,32,2],[9803,43,1],[9804,3,1],[9804,23,1],[9804,48,1],
[9805,28,4],[9805,50,1],[9806,6,1],[9806,56,1],[9807,13,1],
[9807,15,1],[9807,16,1],[9807,17,1],[9807,27,1],[9807,31,1],
[9807,32,5],[9807,35,1],[9807,42,5],[9807,52,1],[9808,62,2],
[9809,28,1],[9809,68,1],[9810,4,3],[9810,5,1],[9810,22,1],[9810,25,2],
[9810,27,1],[9810,28,4],[9810,31,1],[9810,32,1],[9810,35,1],
[9810,38,4],[9810,40,2],[9810,44,29],[9810,47,1],[9810,50,2],
[9810,66,30],[9810,69,1],[9811,3,2],[9811,10,1],[9811,13,1],
[9811,15,1],[9811,37,15],[9812,2,1],[9812,56,1],[9813,2,2],
[9813,17,1],[9813,64,1],[9814,10,1],[9814,28,2],[9814,45,1],
[9814,50,1],[9815,0,1],[9815,2,2],[9815,10,1],[9815,13,2],[9815,14,1],
[9815,16,1],[9815,18,12],[9815,19,1],[9815,30,1],[9815,34,1],
[9815,36,1],[9815,39,1],[9815,59,2],[9815,62,1],[9815,64,1],
[9815,65,2],[9815,71,1],[9816,37,1],[9816,75,1],[9817,5,1],
[9817,28,1],[9817,31,1],[9818,23,1],[9818,28,3],[9818,32,2],
[9818,33,1],[9819,2,1],[9819,20,2],[9819,23,1],[9819,31,1],
[9819,46,1],[9820,37,2],[9821,22,3],[9821,24,1],[9821,27,1],
[9822,2,4],[9822,3,1],[9822,28,1],[9822,72,1],[9823,32,2],[9824,24,1],
[9824,28,1],[9825,2,1],[9825,3,6],[9825,6,3],[9825,23,1],[9825,24,4],
[9825,26,1],[9825,27,1],[9825,28,8],[9825,34,3],[9825,43,2],
[9825,47,1],[9825,68,2],[9825,70,1],[9825,72,1],[9826,26,3],
[9826,47,9],[9827,2,1],[9827,32,12],[9827,47,1],[9828,13,1],
[9828,20,1],[9828,58,1],[9829,24,1],[9829,51,3],[9829,61,1],
[9829,70,1],[9830,2,4],[9830,5,1],[9830,13,1],[9830,21,1],[9830,30,1],
[9830,35,1],[9830,39,3],[9830,51,1],[9830,53,1],[9830,58,1],
[9831,27,1],[9831,28,1],[9832,5,1],[9832,22,2],[9832,28,1],
[9832,56,1],[9833,27,2],[9833,55,1],[9834,28,1],[9834,33,1],
[9835,0,1],[9835,8,2],[9835,25,3],[9835,35,9],[9835,38,1],[9835,52,3],
[9835,72,1],[9836,6,3],[9836,8,2],[9836,22,1],[9836,35,1],[9836,38,2],
[9836,42,1],[9836,51,1],[9837,20,1],[9837,23,1],[9838,62,2],
[9839,23,1],[9839,24,1],[9839,27,2],[9839,31,1],[9839,32,3],
[9839,42,2],[9839,43,2],[9839,60,1],[9840,3,7],[9840,6,2],[9840,22,1],
[9840,24,2],[9840,32,3],[9840,35,2],[9840,47,1],[9840,56,20],
[9840,66,1],[9841,5,2],[9841,27,1],[9841,35,1],[9841,53,1],[9842,1,5],
[9842,3,81],[9842,5,1],[9842,8,2],[9842,9,27],[9842,10,7],[9842,11,4],
[9842,12,6],[9842,13,3],[9842,14,2],[9842,15,6],[9842,16,3],
[9842,17,3],[9842,18,3],[9842,19,8],[9842,20,13],[9842,21,1],
[9842,22,25],[9842,23,1],[9842,24,4],[9842,25,2],[9842,26,2],
[9842,28,16],[9842,29,2],[9842,30,2],[9842,31,2],[9842,34,3],
[9842,36,2],[9842,37,1],[9842,38,1],[9842,39,6],[9842,41,2],
[9842,43,1],[9842,45,5],[9842,46,5],[9842,49,1],[9842,52,19],
[9842,53,4],[9842,55,8],[9842,56,1],[9842,57,3],[9842,58,5],
[9842,59,7],[9842,60,1],[9842,62,2],[9842,65,5],[9842,67,2],
[9842,71,2],[9842,73,1],[9842,74,1],[9842,76,1],[9842,77,4],
[9842,78,1],[9843,56,4],[9843,72,1],[9844,2,7],[9844,20,3],

[9844,31,3],[9844,35,1],[9844,39,1],[9844,41,1],[9844,50,1],
[9844,59,1],[9844,70,1],[9844,77,1],[9845,75,1],[9845,76,2],
[9846,5,3],[9846,33,1],[9847,12,1],[9847,28,35],[9847,32,1],
[9847,38,1],[9847,44,16],[9847,50,2],[9847,55,1],[9847,66,15],
[9848,62,5],[9849,15,1],[9849,24,1],[9849,32,5],[9849,50,1],
[9849,62,1],[9849,70,1],[9850,12,1],[9850,27,1],[9851,26,1],
[9851,33,1],[9852,8,1],[9852,23,3],[9852,24,1],[9852,26,1],
[9852,36,1],[9853,31,1],[9853,43,1],[9853,44,1],[9853,47,3],
[9853,59,1],[9854,0,2],[9855,2,13],[9855,13,1],[9855,39,1],
[9855,47,1],[9856,2,1],[9856,21,2],[9856,31,1],[9857,26,1],
[9857,28,1],[9858,2,4],[9859,10,1],[9859,12,1],[9859,19,1],
[9859,20,1],[9859,24,1],[9859,29,1],[9859,32,1],[9859,37,2],
[9859,41,1],[9859,45,1],[9859,50,1],[9859,53,1],[9859,63,2],
[9859,65,1],[9859,70,1],[9859,71,1],[9859,74,3],[9859,75,1],
[9859,76,10],[9860,2,1],[9860,12,7],[9860,39,1],[9860,46,1],
[9861,4,1],[9861,28,2],[9862,23,1],[9862,25,1],[9862,32,1],
[9863,13,1],[9863,21,1],[9863,22,1],[9863,24,1],[9863,26,3],
[9864,65,2],[9865,66,2],[9866,5,1],[9866,6,1],[9866,23,1],[9867,2,3],
[9867,4,1],[9867,5,1],[9867,29,1],[9867,74,1],[9868,15,1],[9868,19,1],
[9868,53,1],[9869,28,1],[9869,69,1],[9870,3,1],[9870,38,1],[9871,9,2],
[9871,15,1],[9871,24,1],[9871,25,5],[9871,29,1],[9871,44,1],
[9872,15,1],[9872,53,1],[9873,1,11],[9873,3,1],[9873,5,1],[9873,11,1],
[9873,13,6],[9873,15,1],[9873,20,6],[9873,23,6],[9873,24,2],
[9873,25,1],[9873,26,6],[9873,27,8],[9873,28,1],[9873,30,1],
[9873,31,2],[9873,33,2],[9873,35,1],[9873,37,1],[9873,43,2],
[9873,56,5],[9873,57,1],[9873,59,1],[9873,65,1],[9873,76,1],
[9873,78,1],[9874,3,2],[9874,24,1],[9874,26,1],[9874,28,3],
[9874,31,5],[9874,32,1],[9874,39,2],[9874,70,1],[9875,3,2],[9875,6,2],
[9875,21,4],[9875,22,1],[9875,23,1],[9875,24,9],[9875,27,2],
[9875,31,1],[9875,32,1],[9875,34,1],[9875,38,1],[9875,39,24],
[9875,42,2],[9875,56,1],[9875,65,1],[9875,66,1],[9876,4,7],[9876,5,3],
[9876,6,1],[9876,9,3],[9876,10,2],[9876,11,2],[9876,12,1],[9876,13,1],
[9876,15,22],[9876,16,3],[9876,17,65],[9876,18,3],[9876,19,2],
[9876,22,1],[9876,23,1],[9876,25,1],[9876,26,1],[9876,27,1],
[9876,28,9],[9876,34,1],[9876,36,1],[9876,38,2],[9876,39,1],
[9876,40,1],[9876,44,1],[9876,45,3],[9876,47,2],[9876,49,2],
[9876,50,8],[9876,53,62],[9876,55,29],[9876,57,2],[9876,59,1],
[9876,65,1],[9876,68,1],[9876,70,1],[9876,74,2],[9876,75,2],
[9876,76,1],[9876,77,1],[9877,4,1],[9877,17,1],[9877,50,2],
[9877,53,1],[9877,62,1],[9878,23,1],[9878,24,2],[9878,28,1],
[9879,23,1],[9879,26,1],[9879,27,3],[9879,32,1],[9879,34,1],
[9879,39,1],[9880,60,1],[9880,77,1],[9881,3,1],[9881,28,1],
[9882,23,17],[9882,56,1],[9882,74,1],[9883,5,1],[9883,23,1],
[9883,24,1],[9883,25,4],[9883,28,10],[9883,31,1],[9884,10,1],
[9884,16,1],[9884,37,1],[9885,2,1],[9885,28,2],[9885,32,1],
[9885,42,1],[9886,21,1],[9886,26,1],[9886,31,29],[9886,33,8],
[9886,43,1],[9887,32,22],[9888,2,2],[9889,2,2],[9889,5,2],[9889,21,5],
[9889,24,2],[9889,26,7],[9889,31,1],[9889,32,1],[9889,33,3],
[9889,43,1],[9889,44,1],[9889,52,2],[9890,43,1],[9890,55,1],
[9891,5,2],[9891,9,1],[9891,21,5],[9891,23,1],[9891,34,1],[9891,40,1],

[9891,58,1],[9891,63,1],[9892,28,1],[9892,44,2],[9893,17,1],
[9893,53,1],[9893,55,3],[9894,0,1],[9894,1,111],[9894,2,14],
[9894,3,24],[9894,4,1],[9894,5,3],[9894,6,10],[9894,7,10],[9894,8,2],
[9894,9,21],[9894,10,26],[9894,11,16],[9894,12,35],[9894,13,50],
[9894,14,7],[9894,15,30],[9894,16,20],[9894,17,13],[9894,18,23],
[9894,19,23],[9894,20,28],[9894,21,1],[9894,22,3],[9894,23,2],
[9894,24,6],[9894,25,2],[9894,26,64],[9894,27,4],[9894,28,12],
[9894,29,18],[9894,30,9],[9894,31,15],[9894,32,8],[9894,33,2],
[9894,34,7],[9894,35,9],[9894,36,6],[9894,37,16],[9894,38,5],
[9894,39,13],[9894,40,6],[9894,41,9],[9894,42,8],[9894,43,15],
[9894,44,7],[9894,45,22],[9894,46,17],[9894,47,4],[9894,48,1],
[9894,49,7],[9894,50,5],[9894,51,3],[9894,52,2],[9894,53,10],
[9894,54,10],[9894,55,18],[9894,56,66],[9894,57,13],[9894,58,14],
[9894,59,26],[9894,60,18],[9894,62,5],[9894,63,2],[9894,64,7],
[9894,65,25],[9894,66,6],[9894,67,11],[9894,68,4],[9894,69,2],
[9894,70,2],[9894,71,13],[9894,72,3],[9894,73,24],[9894,74,37],
[9894,75,5],[9894,76,26],[9894,77,74],[9894,78,9],[9895,27,1],
[9895,28,1],[9895,32,1],[9895,35,4],[9895,74,1],[9896,24,8],
[9896,28,1],[9897,31,1],[9897,43,1],[9897,76,1],[9898,2,1],
[9898,47,1],[9898,61,1],[9898,70,1],[9899,12,1],[9899,15,1],
[9899,18,1],[9899,23,6],[9899,24,1],[9899,26,4],[9899,28,1],
[9899,33,5],[9899,42,5],[9899,47,1],[9899,52,1],[9899,65,1],
[9900,1,1],[9900,46,1],[9901,2,7],[9901,3,6],[9901,4,2],[9901,5,6],
[9901,20,4],[9901,22,1],[9901,23,4],[9901,24,2],[9901,25,1],
[9901,26,1],[9901,27,5],[9901,28,2],[9901,31,1],[9901,35,2],
[9901,36,2],[9901,39,1],[9901,40,1],[9901,43,5],[9901,47,1],
[9901,50,2],[9901,52,1],[9901,59,1],[9901,60,1],[9901,66,1],
[9901,68,1],[9901,70,1],[9901,75,1],[9902,0,1],[9902,7,1],[9902,33,1],
[9902,39,1],[9902,48,1],[9902,53,1],[9902,75,1],[9903,2,23],
[9903,3,5],[9903,5,2],[9903,6,1],[9903,10,1],[9903,21,4],[9903,22,14],
[9903,23,12],[9903,24,14],[9903,25,2],[9903,26,4],[9903,27,13],
[9903,28,20],[9903,30,1],[9903,31,9],[9903,32,16],[9903,33,6],
[9903,35,7],[9903,36,1],[9903,37,2],[9903,40,1],[9903,42,6],
[9903,43,11],[9903,47,7],[9903,48,1],[9903,56,2],[9903,62,3],
[9903,68,1],[9903,69,1],[9903,72,1],[9903,74,2],[9903,77,1],
[9904,35,1],[9904,47,3],[9904,51,3],[9905,9,1],[9905,19,1],
[9905,41,2],[9905,45,2],[9906,22,2],[9906,43,1],[9907,5,1],[9907,6,1],
[9907,25,13],[9907,28,1],[9907,32,2],[9907,40,1],[9908,15,1],
[9908,20,1],[9908,57,2],[9909,20,2],[9910,20,1],[9910,28,1],
[9910,33,1],[9910,35,1],[9911,2,1],[9911,9,1],[9911,11,1],[9911,27,1],
[9911,37,1],[9911,60,1],[9911,69,1],[9912,9,3],[9912,23,2],
[9912,27,1],[9912,28,5],[9912,32,3],[9912,42,1],[9913,5,1],
[9913,19,1],[9913,28,1],[9913,61,1],[9913,72,1],[9914,6,1],
[9914,11,1],[9914,15,1],[9914,23,2],[9914,26,1],[9914,47,3],
[9914,68,3],[9914,70,5],[9914,74,8],[9915,13,1],[9915,53,1],
[9916,26,3],[9916,27,14],[9916,75,2],[9917,23,2],[9917,28,5],
[9917,44,2],[9917,52,1],[9917,66,2],[9918,23,1],[9918,32,1],
[9918,42,1],[9919,0,1],[9919,1,5],[9919,2,1],[9919,7,1],[9919,9,5],
[9919,10,3],[9919,11,3],[9919,12,3],[9919,13,7],[9919,14,1],
[9919,15,8],[9919,16,1],[9919,17,2],[9919,18,1],[9919,19,2],

[9919,20,5],[9919,24,2],[9919,27,1],[9919,28,1],[9919,29,1],
[9919,30,2],[9919,32,1],[9919,34,3],[9919,35,1],[9919,36,1],
[9919,37,4],[9919,39,1],[9919,41,1],[9919,45,1],[9919,46,1],
[9919,53,4],[9919,54,2],[9919,55,6],[9919,57,1],[9919,59,3],
[9919,63,2],[9919,64,1],[9919,65,1],[9919,66,1],[9919,67,1],
[9919,71,1],[9919,74,6],[9919,77,4],[9920,2,2],[9920,3,1],[9920,6,1],
[9920,21,2],[9920,22,1],[9920,24,2],[9920,32,1],[9920,42,1],
[9920,43,1],[9921,24,1],[9921,28,1],[9922,2,1],[9922,23,1],
[9922,27,1],[9922,53,1],[9923,2,1],[9923,6,1],[9923,21,1],[9923,35,9],
[9924,28,1],[9924,31,1],[9924,42,1],[9924,43,2],[9925,2,161],
[9925,3,6],[9925,8,2],[9925,12,1],[9925,15,1],[9925,17,1],[9925,21,4],
[9925,22,3],[9925,24,4],[9925,25,1],[9925,26,1],[9925,27,5],
[9925,28,7],[9925,29,1],[9925,30,1],[9925,31,17],[9925,42,3],
[9925,47,7],[9925,55,1],[9925,56,1],[9925,57,1],[9925,60,1],
[9925,64,1],[9925,65,1],[9925,69,1],[9925,70,1],[9925,71,1],
[9926,24,3],[9927,1,17],[9927,2,3],[9927,3,2],[9927,4,1],[9927,5,2],
[9927,7,2],[9927,8,8],[9927,9,20],[9927,10,25],[9927,11,11],
[9927,12,13],[9927,13,11],[9927,14,3],[9927,15,20],[9927,16,12],
[9927,17,7],[9927,18,10],[9927,19,20],[9927,20,11],[9927,21,1],
[9927,22,1],[9927,23,1],[9927,24,2],[9927,25,2],[9927,26,4],
[9927,27,1],[9927,28,31],[9927,29,4],[9927,30,2],[9927,31,2],
[9927,32,1],[9927,33,2],[9927,34,7],[9927,35,2],[9927,36,5],
[9927,37,9],[9927,39,6],[9927,40,3],[9927,41,11],[9927,42,1],
[9927,44,1],[9927,45,10],[9927,46,9],[9927,47,1],[9927,48,2],
[9927,49,4],[9927,50,3],[9927,52,1],[9927,53,4],[9927,54,1],
[9927,55,10],[9927,56,3],[9927,57,10],[9927,58,2],[9927,59,6],
[9927,60,3],[9927,62,2],[9927,63,6],[9927,64,3],[9927,65,11],
[9927,66,1],[9927,67,6],[9927,69,1],[9927,70,3],[9927,71,3],
[9927,73,6],[9927,75,5],[9927,76,11],[9927,77,10],[9927,78,1],
[9927,79,1],[9928,1,2],[9928,2,3],[9928,3,2],[9928,4,102],[9928,5,15],
[9928,9,3],[9928,10,4],[9928,12,4],[9928,13,3],[9928,14,1],
[9928,15,27],[9928,16,1],[9928,17,38],[9928,18,2],[9928,19,4],
[9928,20,2],[9928,21,1],[9928,22,8],[9928,23,8],[9928,24,2],
[9928,25,5],[9928,26,1],[9928,27,1],[9928,28,19],[9928,29,3],
[9928,31,1],[9928,32,3],[9928,33,4],[9928,34,2],[9928,35,2],
[9928,37,1],[9928,38,31],[9928,40,1],[9928,41,1],[9928,42,1],
[9928,44,39],[9928,45,1],[9928,46,1],[9928,47,3],[9928,50,99],
[9928,53,40],[9928,54,1],[9928,55,25],[9928,56,1],[9928,58,2],
[9928,59,4],[9928,61,1],[9928,62,1],[9928,63,4],[9928,66,40],
[9928,68,1],[9928,69,2],[9928,70,7],[9928,71,1],[9928,72,5],
[9928,73,3],[9928,74,4],[9928,75,6],[9928,76,2],[9928,78,2],
[9929,2,1],[9929,25,1],[9929,32,1],[9929,73,1],[9930,2,9],[9930,8,1],
[9930,12,1],[9930,26,1],[9930,27,7],[9930,29,1],[9930,31,7],
[9930,32,1],[9930,39,2],[9930,47,1],[9930,56,1],[9930,62,1],
[9930,63,1],[9930,66,1],[9930,78,3],[9931,13,1],[9931,25,1],
[9931,28,4],[9931,31,1],[9931,62,11],[9932,3,4],[9932,5,1],[9932,6,5],
[9932,7,1],[9932,13,1],[9932,14,1],[9932,20,13],[9932,21,23],
[9932,22,5],[9932,23,13],[9932,24,31],[9932,25,35],[9932,26,46],
[9932,27,10],[9932,28,18],[9932,31,50],[9932,32,7],[9932,33,10],
[9932,34,4],[9932,35,17],[9932,37,2],[9932,38,5],[9932,42,8],

[9932,43,7],[9932,48,3],[9932,52,1],[9932,53,1],[9932,56,12],
[9932,57,1],[9932,59,2],[9932,60,2],[9932,62,14],[9932,63,1],
[9932,66,1],[9932,70,3],[9932,75,1],[9932,76,2],[9932,78,1],
[9933,0,3],[9933,2,1],[9933,9,1],[9933,14,1],[9933,17,1],[9933,18,2],
[9933,20,1],[9933,21,1],[9933,28,16],[9933,29,1],[9933,30,1],
[9933,31,1],[9933,33,2],[9933,38,1],[9933,42,1],[9933,44,1],
[9933,50,1],[9933,51,1],[9933,53,2],[9933,58,2],[9933,62,3],
[9933,65,1],[9933,66,1],[9933,70,3],[9933,71,1],[9933,73,1],
[9933,74,1],[9933,75,1],[9933,76,2],[9934,8,1],[9934,9,1],[9935,2,1],
[9935,20,1],[9936,24,1],[9936,32,1],[9937,4,2],[9937,50,1],
[9937,53,1],[9938,21,1],[9938,28,1],[9938,31,1],[9938,35,3],
[9938,36,2],[9939,15,1],[9939,53,2],[9940,10,1],[9940,19,1],
[9940,27,1],[9940,37,4],[9940,47,1],[9940,73,1],[9940,78,1],
[9941,75,2],[9942,27,10],[9942,62,1],[9942,72,1],[9943,9,1],
[9943,55,3],[9944,3,2],[9944,23,1],[9944,24,1],[9944,25,2],
[9944,27,1],[9944,31,1],[9944,59,3],[9944,62,2],[9945,28,1],
[9945,63,1],[9946,25,2],[9947,2,2],[9947,9,1],[9947,20,1],[9947,27,1],
[9947,28,1],[9947,31,1],[9947,35,1],[9947,44,1],[9948,3,2],[9948,5,1],
[9948,20,1],[9948,27,2],[9948,28,1],[9948,34,1],[9948,35,1],
[9948,38,1],[9948,42,1],[9948,46,1],[9949,7,1],[9949,24,1],
[9949,32,1],[9949,35,1],[9949,42,1],[9950,22,1],[9950,27,1],
[9951,22,1],[9951,35,1],[9952,13,1],[9952,14,1],[9952,20,1],
[9952,45,1],[9953,4,1],[9953,56,1],[9954,66,1],[9954,68,1],[9955,8,1],
[9955,36,1],[9956,28,2],[9957,4,1],[9957,9,1],[9957,15,4],[9957,28,2],
[9957,39,1],[9957,55,4],[9957,74,2],[9958,9,1],[9958,39,2],[9959,2,1],
[9959,5,1],[9959,9,1],[9959,10,1],[9959,13,1],[9959,17,1],[9959,19,2],
[9959,20,1],[9959,21,1],[9959,22,2],[9959,23,1],[9959,25,2],
[9959,27,1],[9959,28,29],[9959,30,23],[9959,33,7],[9959,35,4],
[9959,38,1],[9959,43,1],[9959,44,1],[9959,47,1],[9959,50,1],
[9959,51,1],[9959,52,2],[9959,53,1],[9959,58,1],[9959,63,1],
[9959,65,1],[9959,66,1],[9959,75,1],[9960,28,2],[9961,28,3],
[9962,4,1],[9962,13,1],[9962,23,7],[9962,25,1],[9962,28,2],
[9962,35,2],[9962,40,1],[9962,43,1],[9963,23,5],[9963,27,4],
[9964,21,1],[9964,22,1],[9965,0,1],[9965,2,1],[9965,3,1],[9965,24,1],
[9965,25,1],[9965,26,1],[9965,39,1],[9965,43,1],[9965,47,1],
[9965,61,1],[9966,0,1],[9966,1,2],[9966,2,3],[9966,3,2],[9966,5,2],
[9966,9,2],[9966,13,2],[9966,17,1],[9966,20,2],[9966,23,4],
[9966,24,1],[9966,27,1],[9966,28,2],[9966,30,1],[9966,31,3],
[9966,33,1],[9966,35,1],[9966,37,1],[9966,39,3],[9966,42,1],
[9966,43,1],[9966,51,1],[9966,72,1],[9967,0,2],[9967,1,6],[9967,2,1],
[9967,3,5],[9967,4,1],[9967,5,2],[9967,7,12],[9967,8,1],[9967,9,5],
[9967,10,3],[9967,12,6],[9967,13,2],[9967,15,1],[9967,16,2],
[9967,18,1],[9967,19,2],[9967,20,4],[9967,22,2],[9967,23,9],
[9967,24,14],[9967,25,1],[9967,27,17],[9967,28,17],[9967,29,27],
[9967,30,11],[9967,32,40],[9967,33,1],[9967,34,1],[9967,35,10],
[9967,36,1],[9967,37,3],[9967,38,1],[9967,41,1],[9967,42,3],
[9967,44,2],[9967,45,2],[9967,54,2],[9967,55,1],[9967,57,1],
[9967,62,68],[9967,63,1],[9967,65,3],[9967,67,1],[9967,70,1],
[9967,71,1],[9967,72,1],[9967,76,1],[9967,77,1],[9968,33,2],
[9969,2,17],[9969,25,2],[9969,30,1],[9970,24,2],[9970,29,1],

[9970,44,1],[9971,2,1],[9971,4,1],[9971,5,2],[9971,23,1],[9971,27,1],
[9971,28,7],[9971,31,1],[9971,52,1],[9971,60,1],[9971,68,2],
[9972,2,1],[9972,35,1],[9972,56,1],[9973,4,3],[9973,10,1],[9973,23,1],
[9973,44,8],[9973,50,3],[9973,56,1],[9973,66,3],[9974,28,2],
[9975,15,1],[9975,33,1],[9975,62,1],[9976,27,1],[9976,28,1],
[9976,61,1],[9976,68,1],[9976,70,1],[9977,2,1],[9977,3,1],[9977,23,2],
[9977,26,1],[9977,28,1],[9977,43,1],[9977,56,1],[9977,75,1],
[9978,3,1],[9978,74,1],[9979,2,5],[9979,28,1],[9979,42,1],[9979,62,1],
[9980,2,2],[9980,13,2],[9980,21,1],[9980,23,1],[9980,28,1],
[9980,34,1],[9980,62,3],[9981,2,1],[9981,5,1],[9981,28,2],[9982,1,2],
[9982,17,1],[9982,20,1],[9982,26,1],[9982,28,8],[9982,31,3],
[9982,33,1],[9982,48,1],[9982,63,2],[9982,76,1],[9982,77,1],
[9983,16,1],[9983,34,2],[9984,5,1],[9984,24,1],[9985,17,1],
[9985,30,1],[9986,2,1],[9986,19,1],[9986,27,1],[9986,31,1],
[9986,41,1],[9986,60,1],[9987,16,1],[9987,55,2],[9988,26,1],
[9988,27,1],[9989,9,5],[9989,64,1],[9990,10,2],[9990,32,1],
[9990,65,1],[9990,71,1],[9991,2,16],[9991,27,1],[9992,63,6],
[9992,75,1],[9992,76,5],[9993,6,1],[9993,28,1],[9994,1,1],[9994,3,1],
[9994,10,2],[9994,13,3],[9994,20,1],[9994,22,1],[9994,26,3],
[9994,30,4],[9994,31,3],[9994,32,3],[9994,33,1],[9994,34,1],
[9994,35,1],[9994,37,2],[9994,42,2],[9994,43,11],[9994,44,1],
[9994,55,1],[9994,59,1],[9994,60,1],[9994,61,1],[9994,70,1],
[9994,71,1],[9994,74,1],[9994,76,1],[9995,35,1],[9995,76,1],
[9996,21,1],[9996,25,1],[9996,26,1],[9996,28,2],[9996,33,1],
[9996,42,3],[9996,62,21],[9997,3,1],[9997,24,2],[9997,25,1],
[9997,34,1],[9997,47,1],[9998,28,2],[9998,33,9],[9998,79,1],
[9999,6,1],[9999,26,1],[9999,70,1],[10000,15,1],[10000,32,1],
[10000,40,1],[10000,74,1],[10001,27,3],[10001,35,1],[10001,42,10],
[10002,24,1],[10002,28,1],[10003,4,3],[10003,31,4],[10003,42,1],
[10003,43,1],[10003,44,5],[10003,50,3],[10003,66,11],[10003,70,1],
[10003,76,1],[10004,0,2],[10004,1,2],[10004,5,1],[10004,6,1],
[10004,12,1],[10004,15,1],[10004,20,3],[10004,22,3],[10004,25,1],
[10004,27,3],[10004,30,1],[10004,33,2],[10004,34,1],[10004,35,2],
[10004,36,1],[10004,43,1],[10004,50,1],[10004,56,2],[10004,58,1],
[10004,66,1],[10004,69,1],[10004,70,3],[10004,74,2],[10005,28,1],
[10005,40,1],[10006,23,1],[10006,32,12],[10006,52,1],[10006,62,5],
[10007,21,2],[10007,23,3],[10007,24,1],[10007,27,1],[10008,4,2],
[10008,28,1],[10008,32,1],[10008,50,4],[10009,15,1],[10009,31,1],
[10009,55,1],[10010,22,1],[10010,28,1],[10011,20,1],[10011,26,1],
[10012,33,1],[10012,35,2],[10012,39,1],[10013,28,1],[10013,32,1],
[10014,42,1],[10014,56,2],[10015,29,1],[10015,39,1],[10016,2,4],
[10017,27,1],[10017,43,2],[10018,21,2],[10018,22,1],[10018,23,1],
[10018,24,9],[10018,26,2],[10018,33,3],[10018,43,4],[10018,45,1],
[10019,2,10],[10019,31,4],[10019,43,1],[10020,28,2],[10021,17,2],
[10022,20,4],[10022,22,1],[10022,25,1],[10022,27,1],[10022,47,1],
[10023,6,1],[10023,22,1],[10023,23,1],[10023,24,1],[10023,27,2],
[10023,28,1],[10023,35,1],[10023,56,1],[10023,72,1],[10024,23,1],
[10024,28,2],[10024,31,1],[10024,50,1],[10025,2,1],[10025,3,1],
[10025,22,1],[10025,26,1],[10025,45,1],[10025,47,9],[10025,63,1],
[10025,78,1],[10026,21,2],[10026,27,1],[10026,35,1],[10027,2,1],

[10027,22,1],[10027,28,1],[10027,62,6],[10027,66,1],[10028,13,1],
[10028,28,2],[10029,23,1],[10029,24,1],[10029,32,1],[10029,71,1],
[10030,20,1],[10030,22,8],[10030,38,2],[10031,3,1],[10031,8,1],
[10031,9,1],[10031,22,1],[10031,24,1],[10031,25,2],[10031,37,4],
[10031,40,4],[10031,56,5],[10031,66,1],[10031,68,1],[10031,70,2],
[10031,75,1],[10032,53,2],[10033,35,2],[10034,2,2],[10035,30,2],
[10036,26,2],[10036,31,1],[10036,47,1],[10036,56,3],[10037,30,1],
[10037,51,2],[10038,20,1],[10038,29,1],[10039,0,7],[10039,1,11],
[10039,2,7],[10039,3,9],[10039,6,1],[10039,7,1],[10039,8,4],
[10039,9,5],[10039,10,1],[10039,11,3],[10039,12,13],[10039,13,33],
[10039,14,1],[10039,15,9],[10039,17,5],[10039,18,2],[10039,19,7],
[10039,20,11],[10039,22,3],[10039,23,79],[10039,24,59],[10039,25,5],
[10039,26,6],[10039,27,27],[10039,28,16],[10039,29,5],[10039,30,9],
[10039,32,71],[10039,33,1],[10039,34,44],[10039,35,15],[10039,36,1],
[10039,38,2],[10039,39,28],[10039,41,4],[10039,43,2],[10039,44,4],
[10039,45,2],[10039,46,7],[10039,49,3],[10039,50,1],[10039,53,1],
[10039,54,1],[10039,55,1],[10039,56,1],[10039,57,4],[10039,59,3],
[10039,62,42],[10039,65,2],[10039,66,1],[10039,67,2],[10039,69,1],
[10039,71,1],[10039,73,1],[10039,75,1],[10039,76,1],[10039,77,3],
[10040,33,2],[10041,6,1],[10041,21,1],[10041,24,1],[10041,25,3],
[10041,31,4],[10041,35,1],[10041,42,2],[10042,2,1],[10042,3,3],
[10042,5,2],[10042,9,1],[10042,10,1],[10042,11,2],[10042,15,1],
[10042,17,1],[10042,18,1],[10042,19,3],[10042,20,1],[10042,23,1],
[10042,24,2],[10042,25,1],[10042,26,1],[10042,27,2],[10042,28,7],
[10042,30,1],[10042,31,4],[10042,35,1],[10042,37,1],[10042,42,2],
[10042,46,1],[10042,55,2],[10042,59,1],[10042,65,2],[10042,70,1],
[10042,73,1],[10042,75,1],[10042,76,1],[10042,77,1],[10043,22,1],
[10043,24,1],[10043,34,1],[10043,55,1],[10044,14,1],[10044,19,1],
[10044,20,1],[10045,9,1],[10045,12,1],[10045,19,1],[10045,39,1],
[10045,41,1],[10045,54,1],[10045,58,1],[10045,71,1],[10046,2,5],
[10046,10,1],[10046,28,1],[10046,47,1],[10047,1,3],[10047,5,1],
[10047,12,1],[10047,13,1],[10047,27,1],[10047,30,2],[10047,36,1],
[10047,38,1],[10047,39,1],[10047,53,1],[10048,33,2],[10049,2,1],
[10049,13,1],[10050,2,1],[10050,33,2],[10050,60,1],[10051,2,2],
[10051,22,1],[10051,24,1],[10051,27,2],[10051,42,1],[10051,62,2],
[10052,21,1],[10052,35,1],[10053,28,4],[10054,15,2],[10055,2,1],
[10055,4,1],[10055,27,1],[10055,40,1],[10055,51,1],[10055,69,1],
[10056,26,1],[10056,28,4],[10056,38,1],[10056,48,1],[10056,66,1],
[10057,39,1],[10057,65,1],[10058,22,2],[10058,23,1],[10058,27,1],
[10058,42,2],[10058,51,2],[10058,56,1],[10058,61,1],[10059,17,1],
[10059,71,1],[10060,4,1],[10060,8,1],[10061,2,1],[10061,20,1],
[10061,21,2],[10061,31,2],[10061,42,1],[10061,43,2],[10062,20,2],
[10062,23,2],[10062,27,2],[10062,28,1],[10062,31,1],[10062,39,1],
[10062,43,1],[10062,47,1],[10062,65,1],[10063,20,1],[10063,36,1],
[10064,2,4],[10064,3,2],[10064,13,1],[10064,23,1],[10064,35,1],
[10064,42,1],[10064,47,1],[10064,52,1],[10064,61,1],[10064,69,1],
[10064,72,1],[10064,73,1],[10064,79,1],[10065,21,1],[10065,23,3],
[10065,24,1],[10065,26,1],[10065,32,1],[10065,35,1],[10065,42,1],
[10065,43,1],[10066,26,1],[10066,39,1],[10067,38,7],[10068,21,1],
[10068,47,1],[10069,32,8],[10069,68,1],[10070,23,1],[10070,39,1],

[10070,42,1],[10071,24,1],[10071,42,1],[10072,41,1],[10072,67,1],
[10073,28,1],[10073,44,1],[10074,25,1],[10074,28,1],[10074,33,1],
[10074,44,1],[10074,55,1],[10074,56,1],[10075,16,2],[10075,17,1],
[10075,19,1],[10075,20,1],[10075,24,1],[10075,35,1],[10075,40,1],
[10075,50,1],[10075,58,1],[10075,63,1],[10075,68,1],[10075,70,1],
[10075,72,2],[10075,74,3],[10075,77,1],[10076,18,1],[10076,30,1],
[10076,57,2],[10076,71,1],[10077,32,2],[10078,2,1],[10078,21,5],
[10078,23,1],[10078,24,2],[10078,26,1],[10078,33,10],[10078,35,1],
[10078,40,1],[10078,42,2],[10078,47,1],[10078,49,1],[10078,50,1],
[10078,52,1],[10078,58,1],[10078,61,1],[10078,64,1],[10078,70,2],
[10078,71,1],[10078,73,1],[10078,75,1],[10079,53,1],[10079,55,1],
[10080,2,16],[10081,52,4],[10082,2,1],[10082,22,1],[10082,30,1],
[10082,32,2],[10082,40,1],[10082,42,1],[10083,0,1],[10083,1,3],
[10083,5,1],[10083,6,1],[10083,7,5],[10083,8,4],[10083,9,29],
[10083,10,59],[10083,11,19],[10083,12,14],[10083,13,7],[10083,14,11],
[10083,15,19],[10083,16,30],[10083,17,4],[10083,18,14],[10083,19,36],
[10083,20,1],[10083,24,1],[10083,27,2],[10083,29,1],[10083,30,1],
[10083,31,2],[10083,32,1],[10083,34,12],[10083,36,11],[10083,37,13],
[10083,39,4],[10083,40,1],[10083,41,22],[10083,42,1],[10083,45,31],
[10083,46,16],[10083,49,9],[10083,53,2],[10083,54,11],[10083,55,15],
[10083,57,26],[10083,58,13],[10083,59,26],[10083,60,7],[10083,64,8],
[10083,65,35],[10083,67,20],[10083,69,1],[10083,70,1],[10083,71,13],
[10083,73,20],[10083,74,2],[10083,75,1],[10083,77,23],[10083,78,6],
[10084,2,11],[10085,1,6],[10085,8,1],[10085,13,2],[10085,39,2],
[10086,2,1],[10086,3,2],[10086,21,1],[10086,22,1],[10086,23,5],
[10086,24,7],[10086,26,1],[10086,27,5],[10086,40,1],[10086,43,4],
[10086,47,2],[10086,48,1],[10086,69,1],[10087,2,21],[10087,3,14],
[10087,4,4],[10087,5,12],[10087,6,2],[10087,8,2],[10087,9,1],
[10087,11,1],[10087,17,1],[10087,18,1],[10087,19,2],[10087,20,2],
[10087,21,3],[10087,22,18],[10087,23,19],[10087,24,12],[10087,25,4],
[10087,26,5],[10087,27,10],[10087,28,1],[10087,30,3],[10087,31,4],
[10087,32,2],[10087,33,2],[10087,34,1],[10087,35,9],[10087,37,4],
[10087,38,2],[10087,40,1],[10087,42,3],[10087,43,10],[10087,44,1],
[10087,45,1],[10087,47,7],[10087,50,1],[10087,51,8],[10087,53,2],
[10087,56,6],[10087,60,2],[10087,61,1],[10087,65,1],[10087,68,3],
[10087,70,2],[10087,72,2],[10087,74,2],[10087,75,2],[10087,76,2],
[10088,2,6],[10088,28,1],[10089,2,1],[10089,19,1],[10089,47,1],
[10090,30,3],[10090,32,10],[10090,53,1],[10091,37,2],[10091,65,1],
[10092,28,4],[10093,9,1],[10093,23,1],[10093,30,1],[10094,1,1],
[10094,27,1],[10094,66,1],[10095,0,1],[10095,1,2],[10095,2,29],
[10095,3,50],[10095,5,12],[10095,6,7],[10095,7,1],[10095,8,11],
[10095,9,3],[10095,10,1],[10095,11,1],[10095,13,16],[10095,14,1],
[10095,15,4],[10095,16,1],[10095,18,1],[10095,19,1],[10095,20,38],
[10095,21,25],[10095,22,20],[10095,23,110],[10095,24,65],
[10095,25,89],[10095,26,67],[10095,27,62],[10095,28,63],[10095,29,4],
[10095,30,16],[10095,31,86],[10095,32,30],[10095,33,50],[10095,34,13],
[10095,35,62],[10095,37,20],[10095,38,9],[10095,39,21],[10095,40,5],
[10095,41,2],[10095,42,25],[10095,43,74],[10095,44,2],[10095,45,3],
[10095,46,2],[10095,47,21],[10095,48,13],[10095,50,3],[10095,51,1],
[10095,52,2],[10095,53,1],[10095,54,2],[10095,56,30],[10095,57,1],

[10095,58,1],[10095,59,14],[10095,60,9],[10095,61,1],[10095,62,6],
[10095,63,1],[10095,64,5],[10095,65,1],[10095,66,3],[10095,67,1],
[10095,68,1],[10095,69,2],[10095,70,11],[10095,73,1],[10095,74,3],
[10095,75,1],[10095,76,2],[10095,77,1],[10095,78,4],[10096,31,2],
[10097,2,1],[10097,20,3],[10097,22,2],[10097,23,1],[10097,30,1],
[10098,6,1],[10098,39,1],[10098,43,4],[10099,2,3],[10099,18,1],
[10100,8,1],[10100,9,1],[10100,12,1],[10101,4,1],[10101,5,1],
[10101,23,1],[10101,25,1],[10101,27,3],[10101,32,1],[10101,35,3],
[10101,43,1],[10101,47,1],[10102,4,1],[10102,28,1],[10102,79,1],
[10103,4,1],[10103,16,1],[10103,23,2],[10103,27,2],[10103,31,1],
[10103,35,2],[10103,40,2],[10103,43,1],[10103,44,1],[10103,50,1],
[10103,56,1],[10103,63,46],[10103,66,3],[10103,68,2],[10103,69,1],
[10103,70,2],[10103,72,2],[10103,74,37],[10103,75,17],[10103,76,36],
[10104,28,1],[10104,32,1],[10105,31,2],[10106,22,1],[10106,25,5],
[10106,28,1],[10106,44,1],[10107,62,11],[10108,28,1],[10108,50,1],
[10109,0,1],[10109,53,2],[10109,55,1],[10110,22,1],[10110,23,2],
[10110,24,1],[10110,25,1],[10110,28,4],[10110,31,1],[10110,40,1],
[10110,44,2],[10110,61,1],[10110,63,1],[10110,70,1],[10110,72,1],
[10111,52,5],[10112,3,1],[10112,55,1],[10113,0,1],[10113,17,1],
[10113,30,3],[10113,34,4],[10113,35,1],[10114,34,1],[10114,73,1],
[10115,32,2],[10116,50,1],[10116,53,1],[10117,28,2],[10118,1,8],
[10118,2,3],[10118,3,13],[10118,4,46],[10118,5,21],[10118,6,4],
[10118,8,1],[10118,9,4],[10118,10,6],[10118,11,1],[10118,12,2],
[10118,13,3],[10118,14,1],[10118,15,17],[10118,16,4],[10118,17,2],
[10118,18,2],[10118,19,2],[10118,20,14],[10118,21,5],[10118,22,9],
[10118,23,8],[10118,24,19],[10118,25,6],[10118,26,2],[10118,27,6],
[10118,28,2],[10118,29,2],[10118,30,1],[10118,31,2],[10118,32,6],
[10118,33,10],[10118,34,3],[10118,35,5],[10118,36,1],[10118,37,4],
[10118,38,14],[10118,39,1],[10118,40,4],[10118,42,1],[10118,43,3],
[10118,44,72],[10118,45,1],[10118,46,2],[10118,47,50],[10118,48,2],
[10118,50,45],[10118,51,140],[10118,52,4],[10118,53,4],[10118,54,1],
[10118,55,13],[10118,56,8],[10118,57,1],[10118,58,1],[10118,59,1],
[10118,60,3],[10118,61,2],[10118,62,1],[10118,63,6],[10118,65,3],
[10118,66,61],[10118,68,11],[10118,69,16],[10118,70,11],[10118,71,1],
[10118,72,8],[10118,73,1],[10118,74,13],[10118,75,2],[10118,76,2],
[10118,77,1],[10119,1,10],[10119,12,15],[10119,13,10],[10119,16,1],
[10119,18,1],[10120,3,1],[10120,20,1],[10120,28,5],[10121,13,2],
[10122,13,1],[10122,37,1],[10123,41,1],[10123,59,1],[10124,22,1],
[10124,27,6],[10124,32,1],[10125,2,5],[10125,3,1],[10125,5,2],
[10125,13,1],[10125,20,1],[10125,22,2],[10125,23,5],[10125,24,1],
[10125,25,3],[10125,26,3],[10125,27,10],[10125,28,3],[10125,31,1],
[10125,32,4],[10125,33,1],[10125,35,2],[10125,43,2],[10125,47,1],
[10125,48,1],[10125,59,1],[10125,66,1],[10125,70,1],[10126,31,2],
[10127,8,8],[10127,20,2],[10127,34,4],[10127,36,1],[10128,22,1],
[10128,26,1],[10129,4,1],[10129,22,1],[10129,27,1],[10129,39,1],
[10129,44,2],[10129,50,1],[10129,63,1],[10129,68,1],[10130,27,2],
[10130,32,1],[10130,35,1],[10130,51,1],[10130,66,1],[10130,68,1],
[10130,70,1],[10131,5,1],[10131,10,1],[10131,23,2],[10131,38,1],
[10131,42,1],[10132,3,1],[10132,31,1],[10133,25,1],[10133,42,1],
[10134,11,1],[10134,16,1],[10134,19,1],[10135,31,3],[10136,12,4],

[10137,26,1],[10137,31,1],[10138,4,1],[10138,66,1],[10138,75,1],
[10139,17,1],[10139,21,1],[10139,22,2],[10139,23,4],[10139,25,2],
[10139,27,4],[10139,28,15],[10139,30,1],[10139,35,3],[10139,38,1],
[10139,39,2],[10139,53,4],[10140,9,1],[10140,17,2],[10140,55,1],
[10141,1,15],[10141,13,1],[10141,29,2],[10142,28,1],[10142,75,1],
[10143,2,1],[10143,10,1],[10143,11,2],[10143,13,1],[10143,22,2],
[10143,23,1],[10143,28,3],[10144,0,1],[10144,1,1],[10144,2,1],
[10144,8,2],[10144,10,1],[10144,12,2],[10144,13,4],[10144,15,1],
[10144,20,3],[10144,23,3],[10144,24,1],[10144,25,1],[10144,26,51],
[10144,27,1],[10144,29,1],[10144,30,3],[10144,31,2],[10144,33,12],
[10144,34,2],[10144,39,1],[10144,42,24],[10144,43,1],[10144,55,1],
[10144,70,1],[10144,79,1],[10145,15,3],[10145,41,1],[10145,55,1],
[10145,75,1],[10146,13,1],[10146,24,2],[10147,10,1],[10147,77,1],
[10148,26,2],[10148,28,3],[10148,43,10],[10148,59,1],[10149,26,1],
[10149,29,1],[10150,6,1],[10150,74,2],[10151,2,2],[10151,5,1],
[10151,27,1],[10152,17,1],[10152,53,4],[10153,16,1],[10153,18,1],
[10154,4,1],[10154,17,5],[10154,26,1],[10154,28,1],[10154,44,1],
[10154,50,2],[10154,53,4],[10154,55,1],[10154,70,1],[10154,71,1],
[10155,53,2],[10155,66,1],[10156,22,2],[10156,23,1],[10156,28,1],
[10157,3,1],[10157,5,1],[10157,28,1],[10157,62,1],[10157,76,1],
[10158,24,1],[10158,36,1],[10159,2,1],[10159,5,1],[10159,56,1],
[10160,34,4],[10160,35,1],[10161,17,1],[10161,22,2],[10161,23,1],
[10161,24,1],[10161,30,1],[10161,33,1],[10161,38,1],[10161,39,1],
[10161,45,1],[10161,51,1],[10162,21,1],[10162,27,4],[10163,1,1],
[10163,23,1],[10163,25,1],[10163,53,1],[10164,9,1],[10164,10,1],
[10164,18,1],[10164,37,1],[10164,41,1],[10164,45,1],[10164,59,1],
[10164,60,2],[10164,71,1],[10165,1,1],[10165,2,30],[10165,3,1],
[10165,5,1],[10165,8,1],[10165,10,1],[10165,13,1],[10165,15,1],
[10165,19,1],[10165,20,3],[10165,23,1],[10165,25,2],[10165,32,1],
[10165,42,1],[10165,57,1],[10165,68,1],[10165,70,1],[10165,73,2],
[10165,75,1],[10166,20,1],[10166,34,1],[10167,9,1],[10167,17,1],
[10167,19,1],[10167,20,1],[10167,21,1],[10167,23,1],[10168,32,1],
[10168,38,1],[10168,70,1],[10169,27,3],[10169,43,4],[10170,2,5],
[10170,27,1],[10171,53,3],[10172,26,1],[10172,28,1],[10172,35,1],
[10173,44,1],[10173,66,1],[10174,8,1],[10174,19,1],[10174,23,1],
[10174,33,1],[10174,62,9],[10175,23,3],[10175,24,1],[10175,27,1],
[10175,70,1],[10176,11,1],[10176,21,1],[10176,51,1],[10176,62,1],
[10177,12,2],[10178,8,1],[10178,10,1],[10178,24,1],[10178,39,1],
[10179,22,2],[10179,27,3],[10179,28,1],[10179,35,1],[10179,47,1],
[10180,0,2],[10180,2,3],[10180,7,1],[10180,28,2],[10180,31,1],
[10180,32,1],[10180,40,1],[10180,70,1],[10181,8,1],[10181,43,1],
[10182,0,5],[10182,1,1],[10182,2,32],[10182,4,1],[10182,5,1],
[10182,20,3],[10182,22,2],[10182,23,4],[10182,24,8],[10182,28,1],
[10182,31,4],[10182,32,14],[10182,34,10],[10182,35,2],[10182,36,1],
[10182,39,4],[10182,41,1],[10182,42,1],[10182,43,1],[10182,46,1],
[10182,47,3],[10182,59,1],[10182,62,8],[10182,63,1],[10182,65,1],
[10182,68,2],[10182,69,1],[10182,70,3],[10182,71,1],[10182,72,1],
[10182,78,2],[10183,68,20],[10183,70,1],[10183,75,1],[10184,20,1],
[10184,23,2],[10184,24,1],[10184,25,1],[10184,27,2],[10185,33,1],
[10185,57,1],[10186,2,9],[10186,3,3],[10186,5,3],[10186,6,1],

[10186,12,1],[10186,13,1],[10186,16,1],[10186,22,3],[10186,23,2],
[10186,27,3],[10186,28,1],[10186,29,1],[10186,30,3],[10186,37,1],
[10186,39,2],[10186,46,1],[10186,50,1],[10186,59,1],[10186,62,1],
[10186,66,1],[10186,72,1],[10187,2,1],[10187,21,2],[10187,23,2],
[10187,43,1],[10187,53,1],[10188,6,1],[10188,22,1],[10188,28,1],
[10188,31,1],[10188,39,1],[10189,22,3],[10189,23,1],[10190,24,1],
[10190,28,1],[10190,43,1],[10191,9,1],[10191,32,1],[10191,53,2],
[10192,44,1],[10192,66,1],[10193,24,1],[10193,27,1],[10193,28,1],
[10194,15,1],[10194,53,1],[10195,20,1],[10195,55,1],[10195,59,1],
[10196,42,2],[10197,3,1],[10197,6,3],[10197,24,3],[10198,3,2],
[10198,6,1],[10198,23,5],[10198,24,2],[10198,43,1],[10198,63,1],
[10199,0,1],[10199,1,9],[10199,5,17],[10199,6,1],[10199,7,2],
[10199,9,1],[10199,11,1],[10199,12,62],[10199,13,3],[10199,15,4],
[10199,16,1],[10199,19,1],[10199,20,2],[10199,21,3],[10199,22,2],
[10199,23,1],[10199,24,1],[10199,26,1],[10199,28,3],[10199,29,9],
[10199,33,19],[10199,35,1],[10199,38,1],[10199,39,1],[10199,40,1],
[10199,41,1],[10199,42,1],[10199,47,2],[10199,50,1],[10199,51,1],
[10199,53,1],[10199,55,2],[10199,56,1],[10199,58,1],[10199,60,1],
[10199,69,5],[10199,70,3],[10200,3,3],[10200,28,1],[10200,33,7],
[10201,27,3],[10202,28,2],[10202,31,2],[10203,28,1],[10203,35,16],
[10204,1,1],[10204,12,1],[10204,19,1],[10204,30,2],[10205,3,1],
[10205,28,1],[10206,1,1],[10206,42,3],[10206,48,4],[10206,72,1],
[10207,28,12],[10207,32,1],[10207,38,1],[10208,7,1],[10208,9,1],
[10208,13,1],[10208,20,4],[10208,23,3],[10208,24,7],[10208,26,1],
[10208,31,1],[10208,32,2],[10208,33,1],[10208,35,1],[10208,42,1],
[10208,43,1],[10208,44,1],[10208,45,1],[10208,64,1],[10208,69,2],
[10209,4,1],[10209,28,1],[10209,74,1],[10210,5,1],[10210,28,1],
[10211,1,1],[10211,2,1],[10211,12,2],[10211,13,2],[10211,15,1],
[10211,20,1],[10211,23,1],[10211,24,1],[10211,27,2],[10211,30,3],
[10211,34,1],[10211,35,1],[10211,44,1],[10211,47,1],[10211,51,2],
[10211,66,1],[10212,67,1],[10212,71,1],[10213,43,1],[10213,48,1],
[10214,3,1],[10214,27,2],[10214,33,1],[10214,40,1],[10214,56,1],
[10215,6,2],[10216,0,1],[10216,1,1],[10216,2,1],[10216,6,1],
[10216,10,1],[10216,13,1],[10216,15,2],[10216,20,5],[10216,21,2],
[10216,22,1],[10216,24,1],[10216,27,1],[10216,30,1],[10216,37,1],
[10216,52,1],[10216,55,1],[10216,57,1],[10216,74,1],[10217,0,1],
[10217,47,1],[10218,1,1],[10218,2,5],[10218,7,1],[10218,9,1],
[10218,15,2],[10218,20,15],[10218,37,1],[10218,42,1],[10218,43,1],
[10218,45,1],[10218,53,1],[10218,55,1],[10218,77,1],[10219,4,2],
[10219,25,1],[10219,50,2],[10219,63,1],[10220,0,1],[10220,1,1],
[10220,6,2],[10220,13,1],[10220,21,1],[10220,31,1],[10220,47,1],
[10221,9,1],[10221,15,1],[10221,26,2],[10222,24,1],[10222,40,1],
[10223,22,1],[10223,23,2],[10223,24,4],[10223,27,1],[10223,28,5],
[10223,35,2],[10223,42,1],[10223,56,16],[10224,17,1],[10224,53,1],
[10225,23,2],[10225,32,1],[10225,43,1],[10226,22,2],[10227,3,1],
[10227,16,1],[10227,22,1],[10227,56,1],[10227,68,2],[10228,43,2],
[10229,2,3],[10229,20,1],[10229,22,1],[10229,23,1],[10229,26,1],
[10229,27,1],[10229,31,1],[10229,32,1],[10229,35,2],[10229,65,1],
[10230,2,3],[10231,2,1],[10231,22,1],[10231,23,1],[10231,24,1],
[10231,26,1],[10231,35,1],[10231,60,1],[10232,10,1],[10232,15,2],

[10233,22,1],[10233,31,2],[10234,22,1],[10234,32,1],[10234,50,1],
[10234,63,1],[10235,35,1],[10235,56,1],[10236,21,1],[10236,23,1],
[10236,25,3],[10236,26,1],[10236,28,1],[10236,32,1],[10236,35,2],
[10236,62,1],[10237,23,1],[10237,24,1],[10237,26,1],[10237,35,1],
[10237,44,1],[10237,69,1],[10238,2,3],[10238,3,1],[10239,23,1],
[10239,28,1],[10239,47,4],[10240,26,1],[10240,28,1],[10241,47,1],
[10241,50,1],[10241,56,1],[10241,61,1],[10241,68,1],[10241,72,1],
[10242,27,2],[10242,28,7],[10242,31,1],[10242,32,250],[10242,35,2],
[10242,40,1],[10242,44,2],[10242,56,1],[10242,66,1],[10242,68,1],
[10243,62,2],[10244,24,2],[10244,28,1],[10245,23,1],[10245,25,1],
[10246,22,1],[10246,24,1],[10246,43,2],[10247,28,1],[10247,44,1],
[10247,70,1],[10248,2,1],[10248,22,2],[10249,31,4],[10249,42,8],
[10249,43,1],[10249,47,1],[10249,48,1],[10250,26,1],[10250,27,1],
[10250,59,1],[10250,74,1],[10251,4,1],[10251,10,1],[10251,13,2],
[10251,20,1],[10251,22,1],[10251,27,2],[10251,32,1],[10251,35,1],
[10251,37,1],[10251,50,4],[10252,32,1],[10252,43,2],[10253,28,1],
[10253,50,1],[10254,4,2],[10254,9,2],[10254,15,4],[10254,17,4],
[10254,28,3],[10254,50,1],[10254,53,4],[10254,55,1],[10254,66,1],
[10254,69,1],[10255,20,1],[10255,22,2],[10255,27,3],[10255,32,2],
[10255,33,2],[10255,52,1],[10256,2,2],[10256,3,13],[10256,4,1],
[10256,5,3],[10256,6,4],[10256,8,2],[10256,12,1],[10256,13,2],
[10256,21,3],[10256,22,7],[10256,23,14],[10256,24,10],[10256,25,3],
[10256,26,7],[10256,27,28],[10256,28,14],[10256,31,16],[10256,32,7],
[10256,35,9],[10256,37,1],[10256,38,2],[10256,40,1],[10256,42,4],
[10256,43,8],[10256,45,1],[10256,47,3],[10256,48,3],[10256,52,1],
[10256,56,9],[10256,60,1],[10256,61,1],[10256,62,3],[10256,66,1],
[10256,76,1],[10257,1,1],[10257,17,1],[10257,53,1],[10258,28,1],
[10258,42,1],[10258,44,1],[10258,70,1],[10259,20,1],[10259,21,1],
[10260,1,1],[10260,27,2],[10260,43,1],[10261,28,2],[10261,69,2],
[10262,22,2],[10263,28,1],[10263,30,1],[10263,34,1],[10264,18,1],
[10264,19,1],[10265,5,1],[10265,6,5],[10265,23,1],[10265,24,1],
[10265,26,1],[10265,27,3],[10265,28,1],[10265,31,1],[10265,35,4],
[10265,38,1],[10265,43,1],[10265,50,1],[10265,56,1],[10266,8,1],
[10266,33,1],[10266,35,1],[10267,37,2],[10268,5,2],[10268,53,2],
[10268,68,1],[10268,74,1],[10268,79,1],[10269,22,3],[10269,23,2],
[10269,24,2],[10269,62,2],[10269,74,1],[10270,1,1],[10270,8,2],
[10270,9,6],[10270,10,17],[10270,11,5],[10270,12,3],[10270,13,2],
[10270,14,1],[10270,15,3],[10270,16,7],[10270,18,3],[10270,19,9],
[10270,20,5],[10270,36,5],[10270,37,5],[10270,39,2],[10270,41,4],
[10270,43,1],[10270,45,6],[10270,46,2],[10270,49,4],[10270,54,2],
[10270,55,2],[10270,57,6],[10270,58,4],[10270,59,3],[10270,60,3],
[10270,65,7],[10270,67,2],[10270,70,1],[10270,71,2],[10270,73,6],
[10270,77,4],[10271,23,1],[10271,35,2],[10271,43,1],[10272,24,1],
[10272,27,1],[10273,44,1],[10273,66,1],[10274,3,1],[10274,21,6],
[10275,4,1],[10275,28,1],[10276,1,1],[10276,22,1],[10276,23,1],
[10277,2,2],[10278,15,1],[10278,56,1],[10279,12,1],[10279,67,1],
[10280,3,1],[10280,20,4],[10280,23,3],[10280,24,2],[10280,27,5],
[10280,28,1],[10280,35,1],[10281,32,5],[10281,39,1],[10282,1,2],
[10283,63,3],[10283,74,2],[10283,75,2],[10283,76,9],[10284,4,1],
[10284,22,5],[10284,32,1],[10284,35,1],[10284,41,1],[10284,47,1],

[10284,69,1],[10285,0,1],[10285,9,1],[10285,13,1],[10285,25,2],
[10285,30,2],[10285,33,1],[10285,53,1],[10285,65,1],[10286,56,2],
[10287,32,14],[10287,38,1],[10288,1,1],[10288,2,29],[10288,3,4],
[10288,4,1],[10288,5,1],[10288,6,1],[10288,21,3],[10288,22,2],
[10288,23,13],[10288,24,2],[10288,25,12],[10288,26,5],[10288,27,16],
[10288,28,5],[10288,29,1],[10288,31,2],[10288,32,7],[10288,33,3],
[10288,35,1],[10288,42,2],[10288,43,5],[10288,47,1],[10288,48,1],
[10288,50,1],[10288,52,1],[10288,56,1],[10288,62,5],[10289,1,2],
[10289,34,1],[10290,2,2],[10290,34,2],[10290,59,3],[10290,77,1],
[10291,22,2],[10292,22,1],[10292,50,1],[10293,27,3],[10294,22,1],
[10294,31,1],[10294,42,2],[10295,20,2],[10295,24,2],[10295,34,1],
[10295,55,2],[10296,28,1],[10296,35,1],[10296,62,1],[10297,1,1],
[10297,5,1],[10297,25,6],[10297,62,2],[10297,67,1],[10298,0,4],
[10298,15,1],[10298,23,1],[10298,30,1],[10298,34,6],[10298,39,1],
[10298,41,1],[10299,23,2],[10299,38,1],[10300,51,1],[10300,70,1],
[10301,3,2],[10301,4,1],[10301,6,1],[10301,20,1],[10301,22,1],
[10301,27,2],[10301,36,1],[10301,45,1],[10302,5,1],[10302,21,3],
[10302,28,1],[10302,31,3],[10303,26,1],[10303,28,1],[10303,48,1],
[10304,0,1],[10304,2,5],[10304,5,2],[10304,14,1],[10304,20,8],
[10304,31,1],[10304,42,2],[10304,53,2],[10304,67,1],[10304,71,1],
[10304,77,1],[10305,63,5],[10305,74,1],[10306,9,1],[10306,60,1],
[10307,28,3],[10307,48,1],[10308,23,29],[10309,31,6],[10309,35,1],
[10310,24,1],[10310,28,1],[10311,3,1],[10311,6,2],[10311,20,1],
[10311,21,1],[10311,22,1],[10311,26,3],[10311,27,1],[10311,30,3],
[10311,33,3],[10311,37,1],[10311,38,1],[10311,42,2],[10311,43,1],
[10311,53,1],[10312,23,1],[10312,26,1],[10313,2,1],[10313,47,3],
[10313,62,1],[10314,21,1],[10314,22,1],[10314,27,2],[10314,35,1],
[10314,38,1],[10315,52,4],[10316,20,2],[10316,35,1],[10316,74,1],
[10317,12,1],[10317,15,1],[10318,1,1],[10318,30,1],[10318,33,1],
[10318,35,1],[10318,43,1],[10318,55,1],[10319,27,1],[10319,43,2],
[10320,2,2],[10321,2,1],[10321,66,1],[10322,28,1],[10322,66,1],
[10322,74,1],[10323,2,1],[10323,31,1],[10324,22,2],[10325,28,1],
[10325,43,2],[10326,1,1],[10326,39,1],[10327,3,1],[10327,4,1],
[10327,22,2],[10327,23,2],[10327,24,1],[10327,26,1],[10327,28,12],
[10327,31,2],[10327,32,26],[10327,40,1],[10327,44,3],[10327,47,259],
[10327,50,1],[10327,52,1],[10327,61,11],[10327,62,2],[10327,63,1],
[10327,66,5],[10327,69,7],[10327,70,11],[10327,76,1],[10328,26,1],
[10328,27,1],[10328,68,1],[10328,69,1],[10329,25,1],[10329,27,2],
[10330,27,1],[10330,74,1],[10331,1,4],[10331,3,1],[10331,12,1],
[10331,13,8],[10331,20,1],[10331,22,1],[10331,23,1],[10331,24,2],
[10331,25,1],[10331,30,1],[10331,33,3],[10331,35,7],[10331,39,3],
[10331,41,1],[10331,45,1],[10331,46,1],[10331,50,2],[10331,52,1],
[10331,56,1],[10331,60,1],[10332,13,2],[10332,17,1],[10332,19,1],
[10332,20,6],[10332,21,1],[10332,22,4],[10332,24,1],[10332,25,2],
[10332,26,2],[10332,27,1],[10332,31,1],[10332,33,3],[10332,35,1],
[10332,38,1],[10332,39,2],[10332,42,2],[10332,43,2],[10332,47,2],
[10332,48,1],[10332,67,1],[10332,73,1],[10333,2,1],[10333,3,2],
[10333,5,1],[10333,6,1],[10333,22,5],[10333,23,2],[10333,24,2],
[10333,26,1],[10333,27,2],[10333,28,1],[10333,35,13],[10333,42,1],
[10333,43,1],[10333,47,1],[10334,15,1],[10334,17,1],[10334,53,1],

[10335,22,2],[10335,23,1],[10335,24,1],[10335,26,1],[10335,32,2],
[10335,35,1],[10336,3,1],[10336,5,1],[10336,23,2],[10336,24,1],
[10336,27,1],[10336,28,5],[10336,32,1],[10336,50,1],[10336,63,1],
[10336,74,1],[10336,75,1],[10337,1,1],[10337,2,2],[10337,3,4],
[10337,4,3],[10337,5,3],[10337,6,1],[10337,9,1],[10337,12,2],
[10337,13,1],[10337,15,3],[10337,20,5],[10337,21,1],[10337,22,3],
[10337,23,11],[10337,24,8],[10337,25,5],[10337,26,2],[10337,28,5],
[10337,32,15],[10337,35,2],[10337,40,1],[10337,42,12],[10337,43,1],
[10337,47,1],[10337,51,4],[10337,52,1],[10337,53,1],[10337,55,2],
[10337,56,1],[10337,59,1],[10337,66,2],[10337,68,1],[10337,70,3],
[10337,72,1],[10337,75,4],[10337,77,1],[10338,24,1],[10338,25,1],
[10338,26,1],[10339,65,1],[10339,78,1],[10340,2,1],[10340,4,2],
[10340,5,3],[10340,21,2],[10340,22,1],[10340,25,2],[10340,26,1],
[10340,28,1],[10340,33,1],[10340,40,1],[10340,41,1],[10340,42,1],
[10340,44,1],[10340,50,1],[10340,52,1],[10340,59,1],[10340,63,3],
[10340,66,1],[10340,74,1],[10341,53,1],[10341,65,1],[10342,0,2],
[10342,2,2],[10342,3,1],[10342,7,2],[10342,8,1],[10342,18,1],
[10342,24,7],[10342,27,7],[10342,29,6],[10342,32,15],[10342,34,1],
[10342,37,1],[10342,42,1],[10342,55,1],[10342,57,1],[10342,62,11],
[10342,66,1],[10342,76,1],[10343,15,1],[10343,17,3],[10343,28,3],
[10343,44,3],[10343,50,1],[10343,55,1],[10343,66,3],[10344,31,2],
[10345,3,1],[10345,5,1],[10346,15,1],[10346,33,12],[10346,35,1],
[10346,48,1],[10346,55,1],[10346,63,1],[10346,70,1],[10347,0,2],
[10347,1,14],[10347,7,4],[10347,8,5],[10347,9,49],[10347,10,62],
[10347,11,19],[10347,12,18],[10347,13,6],[10347,14,14],[10347,15,21],
[10347,16,31],[10347,17,11],[10347,18,16],[10347,19,62],[10347,20,17],
[10347,24,1],[10347,29,4],[10347,30,7],[10347,34,17],[10347,36,21],
[10347,37,28],[10347,39,4],[10347,41,31],[10347,45,36],[10347,46,24],
[10347,49,14],[10347,53,12],[10347,54,18],[10347,55,17],[10347,57,25],
[10347,58,17],[10347,59,23],[10347,60,6],[10347,64,5],[10347,65,46],
[10347,67,16],[10347,71,21],[10347,73,14],[10347,77,16],[10347,78,6],
[10348,9,1],[10348,53,1],[10349,5,1],[10349,69,1],[10350,9,1],
[10350,59,1],[10351,2,7],[10352,20,10],[10352,23,1],[10352,28,1],
[10353,6,1],[10353,27,2],[10354,2,1],[10354,31,1],[10354,32,2],
[10355,2,19],[10355,21,2],[10355,24,8],[10355,78,1],[10356,2,1],
[10356,23,1],[10357,1,1],[10357,4,1],[10357,15,6],[10357,17,12],
[10357,22,1],[10357,50,1],[10357,53,11],[10357,55,11],[10357,57,1],
[10357,70,1],[10358,19,2],[10358,55,1],[10358,59,1],[10359,63,2],
[10359,74,4],[10359,75,5],[10359,76,5],[10360,20,2],[10361,2,1],
[10361,3,3],[10361,28,2],[10361,37,1],[10361,39,1],[10361,46,1],
[10361,47,1],[10362,27,1],[10362,55,1],[10363,4,1],[10363,5,4],
[10363,13,2],[10363,21,2],[10363,26,2],[10363,27,3],[10363,32,2],
[10363,35,1],[10363,38,1],[10363,47,1],[10363,50,1],[10363,55,1],
[10363,56,2],[10363,59,1],[10363,72,1],[10363,74,1],[10364,43,7],
[10365,20,1],[10365,21,1],[10365,23,3],[10365,25,1],[10365,27,4],
[10365,28,1],[10365,35,1],[10365,62,1],[10366,6,2],[10367,26,1],
[10367,28,7],[10368,2,2],[10368,22,1],[10368,42,1],[10369,2,1],
[10369,21,12],[10369,24,1],[10369,26,8],[10369,31,5],[10369,33,2],
[10369,40,1],[10369,43,5],[10369,47,2],[10369,48,2],[10369,52,1],
[10370,47,2],[10371,2,3],[10371,3,1],[10371,21,1],[10371,42,1],

[10372,30,1],[10372,62,1],[10373,27,2],[10373,72,1],[10374,27,1],
[10374,33,1],[10375,0,5],[10375,2,5],[10375,12,1],[10375,27,1],
[10375,31,1],[10376,21,1],[10376,24,2],[10376,25,2],[10376,38,1],
[10376,48,1],[10376,66,1],[10376,68,1],[10377,23,1],[10377,31,1],
[10378,3,1],[10378,4,1],[10378,5,188],[10378,7,1],[10378,9,1],
[10378,11,1],[10378,12,1],[10378,13,2],[10378,15,2],[10378,21,1],
[10378,23,1],[10378,24,2],[10378,25,2],[10378,26,1],[10378,27,54],
[10378,28,304],[10378,30,1],[10378,31,2],[10378,32,2],[10378,33,15],
[10378,35,54],[10378,36,1],[10378,40,2],[10378,41,1],[10378,43,78],
[10378,44,2],[10378,45,1],[10378,46,2],[10378,48,4],[10378,49,1],
[10378,52,76],[10378,56,2],[10378,59,104],[10378,64,1],[10378,65,1],
[10378,68,2],[10378,69,1],[10378,70,1],[10378,72,1],[10378,75,2],
[10378,76,1],[10379,28,2],[10380,5,1],[10380,52,1],[10380,75,1],
[10381,8,1],[10381,25,1],[10381,27,1],[10382,10,1],[10382,22,1],
[10383,24,2],[10384,2,1],[10384,3,1],[10384,19,1],[10384,23,1],
[10384,26,2],[10384,27,2],[10384,28,1],[10384,30,1],[10384,31,1],
[10384,37,1],[10384,41,1],[10384,42,2],[10384,43,3],[10384,45,1],
[10384,64,1],[10384,70,1],[10385,48,1],[10385,70,1],[10386,10,1],
[10386,16,4],[10387,28,1],[10387,31,2],[10387,45,1],[10388,47,1],
[10388,48,2],[10389,25,1],[10389,28,4],[10389,30,1],[10389,55,1],
[10389,72,1],[10390,1,6],[10390,10,1],[10390,19,1],[10390,23,1],
[10390,24,3],[10390,27,2],[10390,28,1],[10390,30,1],[10390,32,2],
[10390,34,1],[10390,38,1],[10390,40,2],[10390,42,12],[10390,43,2],
[10390,50,1],[10390,59,6],[10390,62,4],[10390,63,1],[10390,66,1],
[10391,32,2],[10392,5,1],[10392,8,1],[10392,9,1],[10392,15,2],
[10392,23,1],[10392,28,3],[10392,38,15],[10392,40,1],[10392,42,1],
[10392,44,17],[10392,45,1],[10392,48,1],[10392,54,1],[10392,55,2],
[10392,59,1],[10392,66,16],[10392,70,2],[10392,72,1],[10393,3,1],
[10393,24,2],[10393,26,1],[10393,40,1],[10393,42,1],[10393,43,3],
[10393,47,1],[10393,55,2],[10393,56,1],[10393,70,1],[10394,2,4],
[10395,32,5],[10396,3,2],[10396,28,1],[10397,35,1],[10397,42,1],
[10398,1,2],[10398,27,1],[10398,34,1],[10398,39,1],[10398,56,1],
[10399,24,1],[10399,35,1],[10400,33,1],[10400,60,1],[10401,3,1],
[10401,4,2],[10401,17,3],[10401,25,1],[10401,28,1],[10401,32,1],
[10401,40,1],[10401,50,3],[10401,53,1],[10402,28,1],[10402,42,1],
[10403,8,1],[10403,46,1],[10403,55,1],[10404,4,1],[10404,15,1],
[10404,17,4],[10404,33,1],[10404,44,1],[10404,53,5],[10404,65,1],
[10404,75,1],[10404,76,1],[10405,27,1],[10405,28,1],[10405,43,1],
[10406,2,1],[10406,3,1],[10406,43,1],[10407,27,2],[10408,2,2],
[10409,2,1],[10409,23,1],[10409,24,1],[10409,28,3],[10409,32,117],
[10409,44,1],[10409,52,1],[10409,66,1],[10409,68,1],[10409,70,2],
[10409,74,1],[10409,76,1],[10410,1,1],[10410,4,1],[10410,5,1],
[10410,9,1],[10410,10,1],[10410,15,1],[10410,17,1],[10410,20,1],
[10410,30,1],[10410,37,1],[10410,38,1],[10410,44,4],[10410,45,1],
[10410,50,3],[10410,53,2],[10410,55,3],[10410,58,1],[10410,65,1],
[10410,77,1],[10411,4,1],[10411,44,4],[10411,50,3],[10411,66,1],
[10412,0,24],[10412,1,2],[10412,6,1],[10412,7,16],[10412,8,5],
[10412,9,3],[10412,10,3],[10412,11,7],[10412,12,3],[10412,14,1],
[10412,16,1],[10412,18,3],[10412,19,9],[10412,20,1],[10412,34,1],
[10412,36,1],[10412,39,2],[10412,41,4],[10412,45,2],[10412,46,3],

[10412,49,1],[10412,54,2],[10412,57,5],[10412,58,3],[10412,59,3],
[10412,62,1],[10412,65,3],[10412,71,2],[10412,73,9],[10412,77,1],
[10413,4,3],[10413,38,2],[10413,44,1],[10413,50,1],[10414,15,2],
[10414,17,1],[10414,30,2],[10414,54,1],[10414,65,1],[10414,70,2],
[10414,74,3],[10415,0,7],[10415,1,1],[10415,3,1],[10415,8,3],
[10415,10,1],[10415,17,2],[10415,19,1],[10415,22,2],[10415,28,5],
[10415,30,54],[10415,31,1],[10415,33,17],[10415,35,3],[10415,36,1],
[10415,44,1],[10415,49,1],[10415,52,11],[10415,55,1],[10415,59,1],
[10415,65,1],[10415,67,1],[10415,71,1],[10415,72,1],[10415,73,1],
[10416,1,1],[10416,3,9],[10416,9,1],[10416,10,1],[10416,11,1],
[10416,12,1],[10416,13,10],[10416,16,1],[10416,24,12],[10416,27,1],
[10416,28,10],[10416,32,4],[10416,39,1],[10416,43,3],[10416,65,1],
[10416,67,1],[10416,71,1],[10416,72,1],[10417,26,1],[10417,42,2],
[10418,3,1],[10418,6,1],[10418,24,1],[10418,25,4],[10418,28,1],
[10418,40,1],[10418,43,1],[10419,3,1],[10419,25,5],[10419,44,1],
[10420,21,8],[10420,23,3],[10420,32,16],[10420,33,1],[10421,15,1],
[10421,17,1],[10421,53,2],[10422,53,2],[10423,1,1],[10423,9,1],
[10424,49,1],[10424,57,1],[10424,65,1],[10425,23,6],[10425,24,2],
[10425,25,1],[10425,27,1],[10425,35,1],[10426,34,1],[10426,50,1],
[10427,61,2],[10427,62,1],[10428,17,1],[10428,35,1],[10428,70,1],
[10429,1,2],[10429,9,1],[10429,11,1],[10429,12,1],[10429,13,1],
[10429,14,2],[10429,15,5],[10429,16,1],[10429,17,1],[10429,18,1],
[10429,19,6],[10429,20,4],[10429,30,2],[10429,39,2],[10429,41,2],
[10429,45,4],[10429,53,4],[10429,54,1],[10429,55,1],[10429,57,3],
[10429,59,1],[10429,60,1],[10429,64,1],[10429,65,2],[10429,67,2],
[10429,77,2],[10430,9,1],[10430,37,1],[10431,2,4],[10432,76,2],
[10433,2,1],[10433,18,1],[10433,22,2],[10433,24,2],[10433,27,1],
[10433,31,1],[10433,32,1],[10433,47,1],[10433,51,1],[10433,56,1],
[10434,4,2],[10434,44,12],[10434,66,16],[10435,42,1],[10435,78,1],
[10436,5,2],[10436,25,1],[10436,27,2],[10436,28,1],[10436,30,1],
[10436,32,5],[10436,33,1],[10436,35,2],[10436,43,3],[10436,44,1],
[10436,50,1],[10437,2,1],[10437,20,1],[10437,28,1],[10438,5,3],
[10438,25,43],[10438,32,2],[10438,35,2],[10438,43,1],[10438,52,1],
[10439,5,1],[10439,28,1],[10439,44,1],[10440,6,3],[10440,61,1],
[10441,2,1],[10441,30,1],[10441,31,2],[10441,71,1],[10442,6,1],
[10442,24,1],[10442,32,2],[10442,47,1],[10443,1,25],[10443,2,2],
[10443,8,1],[10443,11,1],[10443,12,1],[10443,13,3],[10443,20,2],
[10443,23,11],[10443,27,1],[10443,28,1],[10443,29,3],[10443,34,1],
[10443,35,2],[10443,36,1],[10443,59,1],[10443,73,1],[10444,3,1],
[10444,14,1],[10444,23,2],[10444,24,4],[10444,32,1],[10444,33,4],
[10444,35,1],[10444,59,2],[10445,7,2],[10445,19,1],[10446,6,1],
[10446,22,1],[10446,24,1],[10446,26,1],[10446,27,1],[10446,33,1],
[10447,27,1],[10447,28,1],[10447,43,1],[10448,24,2],[10449,6,1],
[10449,26,4],[10449,28,8],[10449,32,4],[10449,43,2],[10449,68,1],
[10450,20,1],[10450,28,1],[10450,32,7],[10450,34,1],[10451,74,2],
[10452,21,2],[10453,5,1],[10453,24,1],[10453,56,1],[10454,37,1],
[10454,57,1],[10455,6,1],[10455,27,1],[10455,31,1],[10456,59,2],
[10457,56,2],[10458,2,1],[10458,8,1],[10458,28,3],[10458,31,1],
[10459,17,1],[10459,28,1],[10460,24,2],[10460,28,1],[10460,56,3],
[10461,3,1],[10461,5,1],[10461,21,1],[10461,27,2],[10462,0,3],

[10462,1,6],[10462,5,2],[10462,7,1],[10462,9,3],[10462,10,7],
[10462,11,7],[10462,12,2],[10462,13,4],[10462,14,6],[10462,15,4],
[10462,16,2],[10462,17,4],[10462,18,1],[10462,19,4],[10462,20,3],
[10462,22,1],[10462,23,1],[10462,24,2],[10462,27,1],[10462,28,7],
[10462,29,1],[10462,30,1],[10462,33,1],[10462,34,1],[10462,36,1],
[10462,37,2],[10462,39,4],[10462,41,4],[10462,44,1],[10462,45,5],
[10462,46,3],[10462,49,2],[10462,50,1],[10462,53,3],[10462,55,6],
[10462,57,1],[10462,58,1],[10462,59,5],[10462,60,1],[10462,62,1],
[10462,63,1],[10462,64,1],[10462,65,1],[10462,67,2],[10462,73,1],
[10462,74,1],[10462,77,5],[10462,78,2],[10463,0,2],[10463,1,65],
[10463,2,15],[10463,3,31],[10463,4,5],[10463,5,17],[10463,6,6],
[10463,7,19],[10463,8,13],[10463,9,59],[10463,10,63],[10463,11,36],
[10463,12,32],[10463,13,61],[10463,14,18],[10463,15,42],[10463,16,40],
[10463,17,23],[10463,18,36],[10463,19,51],[10463,20,57],[10463,21,10],
[10463,22,11],[10463,23,26],[10463,24,36],[10463,25,15],[10463,26,22],
[10463,27,46],[10463,28,119],[10463,29,9],[10463,30,10],[10463,31,17],
[10463,32,42],[10463,33,17],[10463,34,17],[10463,35,41],[10463,36,21],
[10463,37,21],[10463,38,18],[10463,39,19],[10463,40,19],[10463,41,39],
[10463,42,24],[10463,43,9],[10463,44,18],[10463,45,47],[10463,46,25],
[10463,47,12],[10463,48,17],[10463,49,6],[10463,50,21],[10463,51,4],
[10463,52,1],[10463,53,23],[10463,54,15],[10463,55,28],[10463,56,90],
[10463,57,26],[10463,58,27],[10463,59,47],[10463,60,5],[10463,61,43],
[10463,62,29],[10463,63,127],[10463,64,9],[10463,65,53],[10463,66,28],
[10463,67,17],[10463,68,6],[10463,69,5],[10463,70,86],[10463,71,22],
[10463,72,11],[10463,73,23],[10463,74,234],[10463,75,130],
[10463,76,175],[10463,77,52],[10463,78,11],[10464,2,20],[10465,17,1],
[10465,52,1],[10465,58,1],[10465,65,1],[10466,1,1],[10466,9,1],
[10466,16,1],[10466,30,1],[10466,57,1],[10467,19,1],[10467,22,1],
[10467,23,1],[10467,24,1],[10467,28,1],[10467,32,1],[10467,77,1],
[10468,28,2],[10468,39,1],[10469,32,2],[10470,14,1],[10470,23,2],
[10470,28,1],[10470,35,1],[10471,36,1],[10471,73,1],[10472,25,3],
[10473,22,2],[10473,24,1],[10473,43,1],[10474,2,4],[10474,3,1],
[10474,21,3],[10474,22,3],[10474,27,1],[10474,32,1],[10474,35,1],
[10474,40,2],[10474,47,1],[10474,65,1],[10474,74,2],[10475,26,1],
[10475,28,1],[10476,74,2],[10477,1,1],[10477,35,1],[10478,56,1],
[10478,62,1],[10479,1,3],[10479,2,5],[10479,3,3],[10479,4,52],
[10479,5,9],[10479,9,6],[10479,10,2],[10479,11,2],[10479,12,3],
[10479,15,2],[10479,16,1],[10479,17,31],[10479,18,1],[10479,19,2],
[10479,20,4],[10479,22,2],[10479,23,2],[10479,24,1],[10479,25,4],
[10479,27,6],[10479,28,16],[10479,29,3],[10479,31,1],[10479,32,2],
[10479,33,1],[10479,34,2],[10479,35,5],[10479,38,61],[10479,41,1],
[10479,42,2],[10479,44,112],[10479,46,2],[10479,47,1],[10479,48,2],
[10479,50,61],[10479,51,1],[10479,52,1],[10479,53,30],[10479,54,1],
[10479,55,5],[10479,56,5],[10479,58,1],[10479,59,3],[10479,61,2],
[10479,62,3],[10479,63,2],[10479,64,1],[10479,65,2],[10479,66,143],
[10479,70,6],[10479,71,1],[10479,72,2],[10479,73,1],[10479,74,2],
[10479,75,3],[10479,76,2],[10479,77,1],[10479,78,1],[10480,26,2],
[10480,27,1],[10481,4,1],[10481,32,1],[10482,12,1],[10482,29,1],
[10482,39,1],[10483,35,2],[10484,14,1],[10484,45,1],[10485,28,1],
[10485,38,1],[10486,3,4],[10486,5,1],[10486,6,1],[10486,18,1],

[10486,20,2],[10486,22,2],[10486,23,3],[10486,24,2],[10486,26,3],
[10486,27,1],[10486,30,7],[10486,32,3],[10486,33,1],[10486,35,1],
[10486,36,3],[10486,37,1],[10486,39,1],[10486,42,5],[10486,43,4],
[10486,48,1],[10486,50,1],[10486,56,1],[10486,59,1],[10486,62,1],
[10486,64,1],[10487,76,2],[10488,25,1],[10488,32,1],[10488,43,1],
[10488,56,1],[10489,42,2],[10490,3,2],[10490,5,1],[10490,6,1],
[10490,22,1],[10490,23,1],[10490,24,1],[10490,27,1],[10490,47,1],
[10490,56,1],[10490,66,1],[10491,37,1],[10491,59,1],[10492,2,1],
[10492,47,1],[10492,48,1],[10492,60,1],[10493,31,1],[10493,56,2],
[10494,9,1],[10494,10,3],[10494,18,1],[10494,36,1],[10494,41,1],
[10494,46,1],[10494,65,2],[10495,5,1],[10495,13,2],[10495,15,1],
[10495,19,1],[10495,43,1],[10496,22,1],[10496,23,1],[10497,24,1],
[10497,35,1],[10497,40,1],[10497,74,1],[10498,33,3],[10499,13,2],
[10499,20,2],[10499,38,1],[10499,60,1],[10500,3,1],[10500,28,1],
[10501,34,9],[10502,3,1],[10502,5,1],[10502,23,1],[10502,26,1],
[10502,27,1],[10502,33,1],[10502,50,1],[10502,63,13],[10502,69,1],
[10502,74,5],[10502,75,11],[10502,76,7],[10503,22,3],[10503,23,2],
[10503,27,2],[10503,28,1],[10503,31,2],[10503,35,2],[10504,2,2],
[10504,47,1],[10504,56,1],[10504,73,1],[10505,2,7],[10505,23,1],
[10505,30,1],[10505,35,1],[10505,48,1],[10506,4,2],[10506,17,1],
[10507,23,2],[10508,2,2],[10509,32,1],[10509,68,1],[10510,4,1],
[10510,66,1],[10511,3,1],[10511,24,1],[10511,27,1],[10512,15,1],
[10512,55,2],[10513,26,1],[10513,28,1],[10513,31,1],[10514,2,2],
[10515,5,1],[10515,11,1],[10515,15,1],[10515,24,1],[10515,35,1],
[10515,55,2],[10515,68,2],[10515,70,1],[10515,73,1],[10516,2,2],
[10516,3,1],[10516,8,4],[10516,21,1],[10516,22,1],[10516,28,1],
[10517,23,17],[10517,62,2],[10518,41,1],[10518,46,1],[10518,55,3],
[10519,9,1],[10519,28,1],[10519,77,1],[10520,19,2],[10521,22,1],
[10521,23,1],[10521,24,1],[10521,25,1],[10521,27,5],[10521,43,1],
[10521,52,1],[10522,30,2],[10522,35,1],[10523,21,2],[10523,34,1],
[10523,43,1],[10524,5,1],[10524,20,1],[10525,2,6],[10525,8,1],
[10525,20,2],[10525,46,1],[10525,66,1],[10525,75,1],[10526,32,2],
[10526,39,1],[10527,22,1],[10527,26,1],[10528,1,2],[10528,2,1],
[10528,13,4],[10528,17,1],[10528,26,1],[10528,28,8],[10528,29,1],
[10528,30,1],[10528,35,3],[10528,39,2],[10528,40,1],[10528,41,1],
[10528,55,2],[10529,20,1],[10529,40,1],[10529,55,1],[10530,22,1],
[10530,25,2],[10531,4,1],[10531,17,2],[10531,50,1],[10532,2,2],
[10533,31,1],[10533,33,1],[10534,5,2],[10534,26,1],[10534,42,2],
[10534,68,1],[10535,21,1],[10535,35,1],[10536,21,1],[10536,28,7],
[10537,5,1],[10537,28,1],[10538,31,2],[10539,15,1],[10539,28,3],
[10539,50,1],[10540,26,1],[10540,31,1],[10540,32,1],[10540,33,2],
[10540,43,1],[10541,31,2],[10542,9,1],[10542,30,1],[10543,2,4],
[10543,13,1],[10543,47,1],[10544,23,9],[10545,2,2],[10545,30,1],
[10545,42,1],[10545,47,1],[10546,22,1],[10546,31,1],[10547,0,43],
[10547,1,1],[10547,7,61],[10547,8,3],[10547,10,3],[10547,11,1],
[10547,12,4],[10547,14,1],[10547,16,2],[10547,18,3],[10547,20,1],
[10547,37,1],[10547,41,2],[10547,45,5],[10547,46,3],[10547,53,1],
[10547,54,4],[10547,55,2],[10547,59,2],[10547,65,6],[10547,73,3],
[10547,77,2],[10548,44,2],[10548,48,1],[10548,66,5],[10549,6,1],
[10549,26,5],[10549,43,4],[10550,3,2],[10550,5,2],[10550,20,1],

[10550,21,1],[10550,27,2],[10550,28,1],[10550,32,1],[10550,34,1],
[10550,38,1],[10550,42,1],[10550,43,1],[10550,71,1],[10550,72,1],
[10551,22,1],[10551,75,2],[10552,3,1],[10552,5,1],[10553,24,3],
[10553,28,2],[10553,34,1],[10554,12,1],[10554,20,4],[10555,2,3],
[10555,31,1],[10555,47,1],[10555,62,2],[10556,20,1],[10556,22,2],
[10557,26,3],[10558,15,1],[10558,17,1],[10559,23,1],[10559,27,3],
[10559,35,1],[10559,42,1],[10559,51,1],[10560,4,1],[10560,5,1],
[10560,26,1],[10560,40,1],[10560,56,1],[10560,66,1],[10560,70,2],
[10560,76,1],[10561,0,30],[10561,1,68],[10561,2,2],[10561,3,5],
[10561,4,107],[10561,5,13],[10561,6,3],[10561,7,3],[10561,8,10],
[10561,9,62],[10561,10,49],[10561,11,18],[10561,12,28],[10561,13,42],
[10561,14,5],[10561,15,668],[10561,16,21],[10561,17,1023],
[10561,18,11],[10561,19,66],[10561,20,41],[10561,21,4],[10561,22,10],
[10561,23,14],[10561,24,10],[10561,25,7],[10561,26,9],[10561,27,4],
[10561,28,29],[10561,29,16],[10561,30,8],[10561,31,9],[10561,32,6],
[10561,33,10],[10561,34,12],[10561,35,8],[10561,36,10],[10561,37,18],
[10561,38,20],[10561,39,17],[10561,40,9],[10561,41,18],[10561,42,4],
[10561,43,2],[10561,44,37],[10561,45,17],[10561,46,15],[10561,47,3],
[10561,48,3],[10561,49,10],[10561,50,136],[10561,51,4],[10561,52,1],
[10561,53,1070],[10561,54,10],[10561,55,1372],[10561,56,3],
[10561,57,8],[10561,58,16],[10561,59,21],[10561,60,8],[10561,61,3],
[10561,62,11],[10561,63,2],[10561,64,9],[10561,65,24],[10561,66,37],
[10561,67,13],[10561,69,1],[10561,70,14],[10561,71,10],[10561,72,2],
[10561,73,11],[10561,74,3],[10561,75,3],[10561,76,2],[10561,77,21],
[10561,78,5],[10562,38,1],[10562,44,7],[10562,66,5],[10563,31,2],
[10564,3,1],[10564,22,2],[10564,27,1],[10565,5,1],[10565,18,1],
[10565,37,1],[10566,1,1],[10566,5,1],[10566,8,1],[10566,19,2],
[10566,23,1],[10566,24,11],[10566,25,4],[10566,28,2],[10566,32,1],
[10566,36,1],[10566,37,1],[10566,42,32],[10566,50,1],[10566,56,1],
[10566,58,1],[10566,59,1],[10566,62,16],[10566,70,1],[10567,2,3],
[10567,28,1],[10568,2,4],[10568,5,1],[10568,8,2],[10568,23,2],
[10568,28,1],[10568,30,2],[10568,31,1],[10568,32,1],[10568,35,1],
[10569,27,1],[10569,35,1],[10569,51,1],[10569,63,1],[10570,2,1],
[10570,26,1],[10571,13,1],[10571,45,2],[10571,59,1],[10572,42,1],
[10572,48,2],[10573,37,1],[10573,59,1],[10574,28,4],[10575,3,1],
[10575,23,8],[10575,24,1],[10575,25,2],[10575,26,5],[10575,28,1],
[10575,32,1],[10575,35,1],[10575,43,2],[10575,59,2],[10576,20,1],
[10576,27,1],[10576,35,2],[10577,24,1],[10577,32,1],[10577,35,1],
[10578,22,1],[10578,27,5],[10579,2,1],[10579,31,1],[10580,3,2],
[10580,5,1],[10580,6,1],[10580,12,1],[10580,20,5],[10580,22,1],
[10580,23,1],[10580,24,1],[10580,26,4],[10580,27,6],[10580,32,1],
[10580,33,2],[10580,34,4],[10580,35,2],[10580,42,1],[10580,43,1],
[10580,63,1],[10580,76,1],[10581,1,2],[10581,12,1],[10581,20,3],
[10581,24,1],[10581,29,1],[10581,59,1],[10581,75,1],[10582,12,3],
[10582,25,1],[10582,30,9],[10582,33,7],[10582,35,1],[10583,28,1],
[10583,31,1],[10583,43,4],[10584,21,2],[10584,26,1],[10584,28,1],
[10585,1,1],[10585,2,3],[10586,2,1],[10586,3,6],[10586,5,2],
[10586,6,2],[10586,11,1],[10586,22,2],[10586,23,17],[10586,24,23],
[10586,25,21],[10586,26,2],[10586,27,8],[10586,28,4],[10586,31,10],
[10586,32,14],[10586,35,5],[10586,38,3],[10586,39,2],[10586,42,3],

[10586,43,2],[10586,47,1],[10586,48,1],[10586,52,1],[10586,56,20],
[10586,66,1],[10586,70,1],[10586,72,1],[10587,9,1],[10587,46,1],
[10588,3,2],[10588,6,2],[10588,22,1],[10588,24,4],[10588,26,1],
[10588,27,2],[10588,28,1],[10588,32,5],[10588,33,1],[10588,35,2],
[10588,40,1],[10588,43,1],[10588,47,2],[10588,52,1],[10588,66,1],
[10588,70,1],[10588,77,1],[10589,50,1],[10589,68,2],[10589,69,1],
[10589,70,6],[10589,74,2],[10590,5,1],[10590,14,1],[10590,15,24],
[10590,33,1],[10590,37,1],[10590,43,1],[10590,55,5],[10590,71,1],
[10591,27,5],[10591,32,1],[10591,38,2],[10591,42,2],[10591,44,2],
[10591,56,1],[10592,13,1],[10592,24,1],[10593,1,1],[10593,9,1],
[10593,12,7],[10593,13,2],[10593,25,1],[10593,72,1],[10593,74,1],
[10594,4,1],[10594,22,1],[10595,20,1],[10595,34,5],[10596,4,1],
[10596,27,1],[10596,30,2],[10596,33,5],[10596,37,1],[10596,52,2],
[10596,63,1],[10597,8,2],[10597,20,1],[10597,34,1],[10597,73,1],
[10598,27,3],[10598,35,21],[10599,0,2],[10599,1,7],[10599,8,4],
[10599,9,6],[10599,10,6],[10599,11,2],[10599,12,4],[10599,13,1],
[10599,14,2],[10599,15,1],[10599,16,2],[10599,17,4],[10599,18,3],
[10599,19,5],[10599,20,1],[10599,28,1],[10599,29,3],[10599,30,1],
[10599,36,1],[10599,37,3],[10599,39,1],[10599,40,1],[10599,41,6],
[10599,45,3],[10599,53,5],[10599,54,2],[10599,55,1],[10599,57,4],
[10599,59,3],[10599,60,1],[10599,64,1],[10599,65,5],[10599,67,2],
[10599,77,7],[10599,78,4],[10600,1,12],[10600,2,7],[10600,3,16],
[10600,4,2],[10600,5,1],[10600,6,3],[10600,9,2],[10600,10,4],
[10600,11,1],[10600,12,2],[10600,13,21],[10600,15,1],[10600,20,54],
[10600,21,4],[10600,23,68],[10600,24,66],[10600,25,10],[10600,26,17],
[10600,27,37],[10600,28,16],[10600,31,47],[10600,32,11],[10600,33,23],
[10600,34,7],[10600,35,24],[10600,36,1],[10600,37,5],[10600,38,3],
[10600,39,7],[10600,40,2],[10600,41,1],[10600,42,57],[10600,43,33],
[10600,45,2],[10600,46,1],[10600,47,7],[10600,48,5],[10600,50,1],
[10600,51,1],[10600,52,3],[10600,55,1],[10600,56,4],[10600,57,2],
[10600,59,4],[10600,62,3],[10600,65,2],[10600,66,1],[10600,67,1],
[10600,70,1],[10600,72,1],[10600,73,1],[10600,78,1],[10600,79,1],
[10601,22,2],[10602,13,1],[10602,15,2],[10602,21,1],[10602,42,3],
[10602,55,3],[10603,4,1],[10603,21,1],[10603,69,1],[10604,40,1],
[10604,51,1],[10604,56,1],[10604,76,1],[10605,3,1],[10605,5,1],
[10605,35,1],[10605,54,1],[10606,22,1],[10606,28,3],[10607,22,5],
[10607,24,4],[10607,28,1],[10608,22,1],[10608,27,13],[10608,31,1],
[10608,33,1],[10608,35,9],[10608,50,1],[10609,25,3],[10610,9,2],
[10610,28,2],[10611,22,2],[10611,32,2],[10611,48,1],[10611,56,2],
[10611,61,3],[10611,63,1],[10611,69,1],[10611,70,2],[10612,4,3],
[10612,14,1],[10612,15,1],[10612,17,7],[10612,31,1],[10612,50,1],
[10612,53,7],[10612,55,11],[10613,4,6],[10613,6,1],[10613,25,1],
[10613,27,1],[10613,31,1],[10613,44,5],[10613,50,3],[10613,66,7],
[10613,70,1],[10613,74,2],[10614,20,1],[10614,30,1],[10615,5,1],
[10615,26,1],[10615,28,1],[10615,35,1],[10616,4,1],[10616,5,2],
[10616,27,1],[10616,28,1],[10616,32,2],[10616,34,1],[10616,61,1],
[10616,62,1],[10617,2,2],[10617,3,4],[10617,4,1],[10617,21,1],
[10617,22,1],[10617,23,3],[10617,24,3],[10617,25,2],[10617,26,3],
[10617,27,2],[10617,28,2],[10617,31,1],[10617,32,1],[10617,33,1],
[10617,35,2],[10617,38,1],[10617,47,1],[10617,52,1],[10618,6,4],

[10618,35,1],[10618,48,1],[10619,61,2],[10620,28,1],[10620,62,2],
[10621,30,1],[10621,31,1],[10621,40,1],[10621,62,1],[10622,3,1],
[10622,56,1],[10622,62,1],[10623,4,2],[10623,32,1],[10623,56,1],
[10624,38,1],[10624,47,4],[10625,22,3],[10625,23,1],[10625,27,1],
[10625,28,1],[10625,32,3],[10625,35,1],[10625,56,2],[10626,27,2],
[10627,22,1],[10627,23,1],[10627,27,3],[10627,28,5],[10627,35,3],
[10627,42,1],[10627,47,1],[10627,56,1],[10628,37,1],[10628,55,1],
[10628,65,1],[10629,25,2],[10629,52,1],[10629,70,1],[10630,20,1],
[10630,38,1],[10630,47,1],[10631,2,1],[10631,19,1],[10631,20,2],
[10631,27,1],[10632,15,1],[10632,66,1],[10633,27,1],[10633,50,1],
[10634,1,3],[10634,29,1],[10634,39,1],[10635,23,1],[10635,42,1],
[10635,61,1],[10635,68,1],[10636,2,4],[10637,1,2],[10637,2,6],
[10637,3,5],[10637,8,1],[10637,10,1],[10637,11,1],[10637,13,1],
[10637,16,1],[10637,20,1],[10637,21,2],[10637,22,5],[10637,23,1],
[10637,25,2],[10637,26,2],[10637,27,5],[10637,31,1],[10637,33,1],
[10637,34,3],[10637,37,1],[10637,42,2],[10637,43,1],[10637,48,1],
[10637,55,1],[10637,59,2],[10638,3,1],[10638,28,1],[10638,30,1],
[10638,34,2],[10638,39,4],[10639,21,1],[10639,28,1],[10640,26,1],
[10640,35,1],[10640,38,1],[10641,1,1],[10641,7,1],[10641,8,1],
[10641,9,8],[10641,11,1],[10641,13,1],[10641,16,1],[10641,20,1],
[10641,26,3],[10641,28,6],[10641,36,1],[10641,38,4],[10641,41,1],
[10641,49,1],[10641,59,1],[10641,60,1],[10641,62,1],[10641,64,1],
[10641,67,1],[10641,73,1],[10642,6,1],[10642,32,1],[10642,42,1],
[10642,56,1],[10643,0,2],[10643,1,54],[10643,2,1],[10643,4,1],
[10643,7,3],[10643,8,4],[10643,10,1],[10643,12,41],[10643,13,8],
[10643,15,1],[10643,17,2],[10643,20,3],[10643,21,2],[10643,24,1],
[10643,25,6],[10643,27,2],[10643,28,3],[10643,29,5],[10643,30,4],
[10643,31,19],[10643,32,2],[10643,33,9],[10643,34,3],[10643,35,2],
[10643,36,2],[10643,37,1],[10643,39,4],[10643,47,2],[10643,48,1],
[10643,53,3],[10643,55,3],[10643,59,1],[10644,27,1],[10644,28,1],
[10645,6,1],[10645,28,4],[10645,51,1],[10645,56,3],[10645,74,1],
[10646,18,1],[10646,24,1],[10646,28,1],[10646,32,1],[10647,22,1],
[10647,23,1],[10647,26,5],[10647,43,6],[10648,5,1],[10648,33,5],
[10648,35,7],[10648,38,1],[10649,33,5],[10649,43,2],[10650,74,1],
[10650,75,1],[10650,76,1],[10651,9,1],[10651,28,1],[10652,31,1],
[10652,39,1],[10653,24,3],[10653,47,12],[10653,51,6],[10654,3,2],
[10654,17,1],[10654,23,1],[10654,24,1],[10654,25,2],[10654,26,1],
[10654,27,3],[10654,28,2],[10654,32,3],[10654,35,1],[10654,43,1],
[10654,56,2],[10654,59,1],[10655,33,2],[10656,16,1],[10656,20,1],
[10656,21,1],[10656,22,1],[10656,25,4],[10656,26,1],[10656,35,1],
[10657,0,2],[10657,2,1],[10657,3,1],[10657,6,4],[10657,8,1],
[10657,10,1],[10657,11,3],[10657,13,2],[10657,16,2],[10657,19,1],
[10657,20,21],[10657,21,2],[10657,22,16],[10657,23,12],[10657,24,9],
[10657,25,1],[10657,26,1],[10657,27,19],[10657,28,3],[10657,29,1],
[10657,30,1],[10657,32,1],[10657,33,1],[10657,34,1],[10657,35,12],
[10657,37,1],[10657,38,3],[10657,39,3],[10657,40,1],[10657,41,1],
[10657,42,4],[10657,45,1],[10657,47,1],[10657,50,2],[10657,52,1],
[10657,54,1],[10657,56,1],[10657,59,2],[10657,64,1],[10657,65,1],
[10657,66,1],[10657,74,1],[10657,75,1],[10657,76,5],[10658,38,2],
[10659,35,1],[10659,44,1],[10659,53,1],[10660,21,1],[10660,22,1],

[10660,30,1],[10660,47,1],[10660,48,3],[10660,61,2],[10660,69,1],
[10661,1,1],[10661,2,1],[10661,8,1],[10661,9,5],[10661,10,1],
[10661,11,1],[10661,15,2],[10661,19,1],[10661,20,1],[10661,22,2],
[10661,23,3],[10661,24,4],[10661,26,1],[10661,28,11],[10661,32,2],
[10661,35,1],[10661,37,1],[10661,39,1],[10661,43,1],[10661,44,2],
[10661,50,1],[10661,65,1],[10661,68,2],[10661,69,1],[10661,73,1],
[10661,74,1],[10662,22,5],[10662,42,1],[10663,51,3],[10664,2,4],
[10664,23,4],[10664,24,2],[10664,26,2],[10664,42,2],[10664,56,19],
[10664,61,1],[10665,4,1],[10665,5,1],[10665,15,5],[10665,17,1],
[10665,20,3],[10665,21,1],[10665,32,1],[10665,51,1],[10665,55,1],
[10665,72,1],[10666,1,3],[10666,9,1],[10666,10,1],[10666,11,1],
[10666,12,1],[10666,13,1],[10666,14,1],[10666,15,5],[10666,16,1],
[10666,19,1],[10666,20,1],[10666,29,1],[10666,36,1],[10666,37,3],
[10666,45,1],[10666,46,1],[10666,49,2],[10666,65,2],[10666,71,1],
[10666,77,1],[10667,0,5],[10667,1,28],[10667,7,6],[10667,8,12],
[10667,9,73],[10667,10,65],[10667,11,23],[10667,12,38],[10667,13,21],
[10667,14,14],[10667,15,51],[10667,16,38],[10667,17,31],[10667,18,19],
[10667,19,63],[10667,20,35],[10667,29,32],[10667,30,11],[10667,34,16],
[10667,36,14],[10667,37,26],[10667,39,10],[10667,41,25],[10667,45,31],
[10667,46,19],[10667,49,16],[10667,53,18],[10667,54,19],[10667,55,43],
[10667,57,36],[10667,58,29],[10667,59,32],[10667,60,14],[10667,64,7],
[10667,65,48],[10667,67,31],[10667,71,26],[10667,73,16],[10667,77,15],
[10667,78,4],[10668,13,1],[10668,26,2],[10668,27,1],[10668,28,2],
[10668,29,1],[10668,33,9],[10668,36,1],[10668,42,45],[10668,50,1],
[10668,52,1],[10669,1,1],[10669,7,1],[10669,25,1],[10669,60,1],
[10670,20,1],[10670,22,1],[10671,12,1],[10671,27,1],[10671,28,1],
[10671,42,1],[10672,22,1],[10672,44,1],[10673,2,2],[10673,39,1],
[10673,42,2],[10673,60,1],[10674,24,1],[10674,25,1],[10674,28,1],
[10675,20,1],[10675,24,1],[10676,11,1],[10676,15,3],[10676,17,1],
[10676,18,1],[10676,19,2],[10676,45,1],[10676,49,1],[10676,53,1],
[10676,55,2],[10676,58,1],[10676,71,1],[10677,22,3],[10678,3,1],
[10678,63,14],[10678,70,1],[10678,74,7],[10678,75,5],[10678,76,11],
[10679,22,1],[10679,26,1],[10680,3,2],[10681,0,1],[10681,2,4],
[10681,3,4],[10681,4,2],[10681,5,3],[10681,6,1],[10681,22,4],
[10681,23,2],[10681,24,6],[10681,25,1],[10681,26,2],[10681,33,2],
[10681,38,2],[10681,47,1],[10681,48,1],[10681,56,8],[10681,59,1],
[10681,68,3],[10681,69,1],[10682,29,1],[10682,57,1],[10683,20,1],
[10683,23,1],[10683,24,3],[10683,25,1],[10683,26,1],[10683,29,1],
[10683,47,8],[10684,24,1],[10684,56,1],[10685,0,3],[10685,1,165],
[10685,2,151],[10685,3,7],[10685,4,3],[10685,5,15],[10685,6,10],
[10685,7,4],[10685,8,25],[10685,9,11],[10685,10,1],[10685,11,2],
[10685,12,34],[10685,13,33],[10685,14,1],[10685,15,13],[10685,16,2],
[10685,17,25],[10685,18,1],[10685,19,1],[10685,20,38],[10685,21,384],
[10685,22,32],[10685,23,1227],[10685,24,21],[10685,25,39],
[10685,26,953],[10685,27,56],[10685,28,93],[10685,29,31],
[10685,30,44],[10685,31,637],[10685,32,53],[10685,33,720],
[10685,34,39],[10685,35,36],[10685,36,16],[10685,37,7],[10685,38,3],
[10685,39,884],[10685,40,1],[10685,42,558],[10685,43,758],
[10685,44,2],[10685,47,25],[10685,48,66],[10685,49,1],[10685,50,8],
[10685,51,10],[10685,52,40],[10685,53,16],[10685,55,11],[10685,56,4],

[10685,59,64],[10685,60,1],[10685,62,3],[10685,63,2],[10685,64,1],
[10685,66,2],[10685,67,2],[10685,68,1],[10685,69,3],[10685,70,5],
[10685,72,2],[10685,73,10],[10685,74,2],[10685,76,2],[10685,78,80],
[10685,79,4],[10686,12,1],[10686,53,1],[10687,2,1],[10687,6,1],
[10687,21,1],[10687,22,5],[10687,23,1],[10687,26,1],[10687,33,1],
[10687,38,1],[10687,61,1],[10687,66,1],[10688,1,1],[10688,2,1],
[10688,8,1],[10688,28,2],[10688,35,1],[10688,38,31],[10688,44,1],
[10689,2,3],[10690,5,1],[10690,24,1],[10690,26,1],[10690,27,1],
[10691,28,5],[10691,70,1],[10692,17,1],[10692,39,2],[10693,5,1],
[10693,6,1],[10693,8,1],[10693,24,3],[10694,25,5],[10694,27,1],
[10694,62,2],[10695,2,5],[10695,15,1],[10695,17,1],[10695,25,1],
[10695,30,1],[10695,33,2],[10695,37,1],[10695,42,1],[10695,43,1],
[10695,55,1],[10695,56,1],[10696,8,2],[10696,21,1],[10696,28,3],
[10697,20,1],[10697,35,1],[10698,6,2],[10699,6,1],[10699,72,3],
[10700,0,1],[10700,15,1],[10700,27,2],[10700,44,1],[10700,50,1],
[10700,55,2],[10700,66,1],[10700,76,1],[10701,3,2],[10701,70,1],
[10702,3,2],[10702,5,1],[10702,32,15],[10702,35,1],[10703,4,1],
[10703,12,1],[10703,15,3],[10703,17,1],[10703,28,2],[10703,50,1],
[10703,53,4],[10703,55,6],[10704,35,1],[10704,42,2],[10704,47,1],
[10704,48,1],[10704,51,1],[10704,68,1],[10705,12,1],[10705,25,2],
[10705,53,1],[10706,35,1],[10706,40,1],[10707,28,3],[10707,56,1],
[10708,10,1],[10708,13,1],[10708,18,2],[10708,31,1],[10708,34,1],
[10708,35,1],[10708,36,1],[10708,56,2],[10708,64,1],[10709,2,2],
[10710,0,5],[10710,1,73],[10710,2,12],[10710,3,32],[10710,4,6],
[10710,5,50],[10710,6,45],[10710,7,9],[10710,8,4],[10710,9,13],
[10710,10,23],[10710,11,10],[10710,12,19],[10710,13,145],[10710,14,4],
[10710,15,18],[10710,16,11],[10710,17,4],[10710,18,5],[10710,19,10],
[10710,20,60],[10710,21,26],[10710,22,37],[10710,23,89],[10710,24,97],
[10710,25,44],[10710,26,15],[10710,27,56],[10710,28,38],[10710,29,12],
[10710,30,13],[10710,31,16],[10710,32,28],[10710,33,35],[10710,34,10],
[10710,35,40],[10710,36,7],[10710,37,6],[10710,38,6],[10710,39,11],
[10710,40,8],[10710,41,7],[10710,42,32],[10710,43,16],[10710,44,11],
[10710,45,11],[10710,46,8],[10710,47,14],[10710,48,7],[10710,49,3],
[10710,50,3],[10710,51,14],[10710,52,4],[10710,53,4],[10710,54,3],
[10710,55,6],[10710,56,13],[10710,57,11],[10710,58,3],[10710,59,15],
[10710,60,1],[10710,62,8],[10710,63,10],[10710,64,4],[10710,65,13],
[10710,66,7],[10710,67,7],[10710,68,5],[10710,69,6],[10710,70,13],
[10710,71,2],[10710,72,3],[10710,73,5],[10710,74,77],[10710,75,5],
[10710,76,11],[10710,77,5],[10710,78,3],[10710,79,2],[10711,39,3],
[10711,43,1],[10711,62,1],[10712,12,1],[10712,15,1],[10713,24,1],
[10713,26,1],[10713,50,1],[10714,0,207],[10714,1,8],[10714,9,1],
[10714,10,2],[10714,11,1],[10714,12,5],[10714,13,1],[10714,14,1],
[10714,15,2],[10714,16,2],[10714,17,1],[10714,18,2],[10714,19,5],
[10714,20,8],[10714,28,1],[10714,29,7],[10714,30,2],[10714,34,25],
[10714,36,1],[10714,37,3],[10714,39,3],[10714,41,1],[10714,45,1],
[10714,46,1],[10714,53,1],[10714,55,1],[10714,57,2],[10714,58,2],
[10714,59,1],[10714,60,1],[10714,64,1],[10714,65,2],[10714,67,2],
[10714,71,1],[10714,73,3],[10714,77,2],[10714,78,1],[10715,19,1],
[10715,34,1],[10715,65,1],[10716,3,1],[10716,44,1],[10717,1,3],
[10717,8,1],[10717,11,1],[10717,15,1],[10717,20,1],[10717,55,1],

[10717,75,1],[10718,2,1],[10718,5,1],[10719,33,3],[10720,1,1],
[10720,2,2],[10720,3,1],[10720,4,1],[10720,16,1],[10720,27,1],
[10720,33,1],[10720,34,1],[10720,48,2],[10720,79,1],[10721,15,1],
[10721,56,1],[10722,9,1],[10722,10,1],[10722,71,1],[10723,55,1],
[10723,66,1],[10724,1,3],[10724,2,2],[10724,5,1],[10724,8,4],
[10724,10,2],[10724,13,2],[10724,21,3],[10724,22,2],[10724,28,1],
[10724,29,1],[10724,33,2],[10724,34,1],[10724,35,2],[10724,47,2],
[10724,60,1],[10724,66,1],[10724,67,1],[10725,8,3],[10725,20,1],
[10725,24,1],[10725,28,1],[10726,23,1],[10726,47,1],[10726,75,1],
[10727,2,1],[10727,3,7],[10727,5,1],[10727,20,1],[10727,22,10],
[10727,23,8],[10727,24,13],[10727,25,1],[10727,26,3],[10727,27,3],
[10727,30,1],[10727,31,1],[10727,32,5],[10727,33,1],[10727,35,3],
[10727,42,1],[10727,43,5],[10727,47,2],[10727,52,1],[10727,56,1],
[10728,6,1],[10728,21,1],[10728,26,1],[10728,32,1],[10728,33,2],
[10728,42,50],[10728,44,1],[10728,74,1],[10729,5,1],[10729,19,1],
[10729,20,1],[10729,25,1],[10729,27,1],[10729,49,1],[10729,55,1],
[10730,10,1],[10730,47,1],[10730,74,2],[10731,2,1],[10731,32,8],
[10731,43,1],[10731,48,2],[10731,62,3],[10731,70,1],[10732,22,4],
[10732,47,1],[10733,3,2],[10733,22,1],[10733,23,1],[10733,28,1],
[10733,38,1],[10733,44,1],[10733,56,5],[10733,62,1],[10734,51,3],
[10735,6,1],[10735,22,1],[10735,33,1],[10736,23,1],[10736,26,1],
[10736,33,1],[10736,43,1],[10737,64,1],[10737,65,1],[10738,4,1],
[10738,5,1],[10738,12,1],[10738,17,8],[10738,50,1],[10738,53,6],
[10739,1,1],[10739,2,10],[10739,11,2],[10739,20,1],[10739,39,1],
[10740,46,1],[10740,68,1],[10741,23,1],[10741,47,1],[10742,23,1],
[10742,27,2],[10742,28,1],[10742,31,1],[10742,32,1],[10742,33,1],
[10743,2,1],[10743,27,1],[10744,20,1],[10744,24,1],[10744,25,1],
[10744,26,1],[10744,47,1],[10745,3,1],[10745,24,1],[10745,26,1],
[10746,0,1],[10746,2,2],[10746,3,1],[10746,13,2],[10746,22,1],
[10746,25,1],[10746,27,2],[10746,28,1],[10746,31,1],[10746,35,2],
[10746,37,4],[10746,47,12],[10746,53,1],[10746,70,1],[10747,6,3],
[10747,24,1],[10747,27,1],[10747,33,1],[10747,47,1],[10748,2,1],
[10748,55,4],[10748,59,1],[10749,2,1],[10749,5,6],[10749,7,1],
[10749,19,1],[10749,22,3],[10749,23,3],[10749,32,28],[10749,40,2],
[10749,47,8],[10749,48,2],[10749,56,5],[10749,57,1],[10749,63,1],
[10749,70,1],[10750,23,3],[10750,28,1],[10750,60,1],[10750,62,1],
[10751,2,1],[10751,19,1],[10751,34,1],[10751,68,1],[10751,70,5],
[10751,72,22],[10752,2,2],[10752,5,1],[10752,26,1],[10752,35,1],
[10752,69,1],[10752,70,1],[10753,2,3],[10753,3,5],[10753,4,2],
[10753,6,2],[10753,8,1],[10753,22,4],[10753,23,3],[10753,24,5],
[10753,25,8],[10753,26,3],[10753,27,15],[10753,28,29],[10753,31,2],
[10753,33,40],[10753,35,25],[10753,38,4],[10753,40,2],[10753,42,1],
[10753,44,2],[10753,47,2],[10753,48,1],[10753,50,2],[10753,52,26],
[10753,56,2],[10753,62,2],[10753,63,3],[10753,66,1],[10753,70,2],
[10753,74,3],[10753,75,4],[10754,8,1],[10754,14,1],[10754,20,1],
[10754,21,1],[10754,24,1],[10754,27,1],[10754,28,1],[10754,39,1],
[10754,42,2],[10754,56,1],[10754,62,1],[10754,78,1],[10755,15,1],
[10755,17,1],[10755,53,1],[10756,2,1],[10756,5,2],[10756,6,1],
[10756,23,3],[10756,24,3],[10756,25,2],[10756,26,1],[10756,30,1],
[10756,32,1],[10756,35,1],[10756,50,1],[10757,27,1],[10757,43,1],

[10757,62,1],[10758,27,1],[10758,38,1],[10759,26,1],[10759,27,1],
[10759,31,1],[10759,47,1],[10760,20,1],[10760,35,1],[10761,51,1],
[10761,62,2],[10762,2,4],[10763,22,1],[10763,27,1],[10763,32,1],
[10764,3,1],[10764,6,1],[10764,23,1],[10764,26,1],[10764,27,1],
[10764,31,1],[10764,33,1],[10764,35,3],[10765,27,2],[10766,19,1],
[10766,30,1],[10766,52,1],[10766,62,1],[10767,17,1],[10767,19,1],
[10767,20,2],[10767,38,2],[10767,57,1],[10768,21,1],[10768,27,1],
[10768,31,1],[10769,3,1],[10769,24,2],[10769,75,1],[10770,2,2],
[10770,28,1],[10771,3,7],[10771,27,1],[10771,30,1],[10771,40,1],
[10771,47,2],[10771,51,1],[10771,56,2],[10772,25,2],[10772,33,3],
[10772,35,10],[10772,44,1],[10772,72,1],[10773,5,1],[10773,20,5],
[10773,22,1],[10773,23,1],[10773,26,1],[10773,27,11],[10774,63,14],
[10774,74,6],[10774,75,14],[10774,76,17],[10775,1,2],[10775,39,1],
[10776,9,1],[10776,28,7],[10777,3,2],[10777,37,4],[10777,39,1],
[10777,56,5],[10777,76,1],[10778,9,1],[10778,13,1],[10778,55,2],
[10779,2,2],[10780,3,1],[10780,4,5],[10780,5,2],[10780,15,7],
[10780,17,1],[10780,25,2],[10780,31,3],[10780,38,4],[10780,44,25],
[10780,47,1],[10780,50,9],[10780,53,2],[10780,55,4],[10780,56,1],
[10780,66,45],[10781,6,1],[10781,22,1],[10781,30,1],[10781,33,1],
[10781,55,1],[10781,56,2],[10781,60,3],[10781,68,1],[10782,28,1],
[10782,56,1],[10783,22,2],[10783,28,5],[10783,33,1],[10783,39,1],
[10784,33,1],[10784,43,1],[10785,24,2],[10786,9,1],[10786,12,1],
[10786,49,1],[10787,2,1],[10787,20,1],[10787,37,1],[10788,16,2],
[10789,1,1],[10789,39,1],[10790,28,174],[10791,2,1],[10791,35,1],
[10792,25,1],[10792,56,3],[10793,5,1],[10793,63,1],[10794,1,1],
[10794,3,1],[10794,5,1],[10794,9,1],[10794,12,1],[10794,15,1],
[10794,21,4],[10794,23,1],[10794,24,2],[10794,26,1],[10794,27,1],
[10794,28,2],[10794,33,8],[10794,40,2],[10794,42,1],[10794,43,1],
[10794,47,4],[10794,51,1],[10794,56,1],[10794,65,1],[10794,66,1],
[10794,68,1],[10794,70,18],[10794,72,7],[10794,74,2],[10794,77,1],
[10795,22,1],[10795,27,1],[10795,28,2],[10795,35,4],[10796,4,2],
[10796,15,1],[10796,25,1],[10796,43,1],[10796,50,5],[10796,66,2],
[10797,12,1],[10797,29,2],[10798,2,2],[10798,22,1],[10798,24,1],
[10798,26,3],[10798,27,1],[10798,31,1],[10798,32,2],[10798,35,2],
[10798,43,8],[10798,75,1],[10799,12,2],[10799,16,1],[10800,56,2],
[10801,0,1],[10801,3,1],[10801,6,1],[10801,12,1],[10801,16,1],
[10801,20,1],[10801,25,1],[10801,26,1],[10801,27,1],[10801,28,1],
[10801,32,1],[10801,33,2],[10801,35,1],[10801,40,2],[10801,44,1],
[10801,52,1],[10801,56,1],[10801,63,35],[10801,64,1],[10801,65,1],
[10801,70,3],[10801,74,24],[10801,75,19],[10801,76,45],[10802,21,1],
[10802,25,1],[10802,27,2],[10802,33,1],[10802,35,2],[10802,62,2],
[10803,15,1],[10803,17,2],[10803,23,1],[10803,53,2],[10803,55,1],
[10804,28,1],[10804,35,1],[10805,42,2],[10806,12,1],[10806,56,1],
[10806,59,1],[10807,2,1],[10807,5,2],[10807,6,1],[10807,8,1],
[10807,11,2],[10807,22,1],[10807,23,2],[10807,25,1],[10807,26,7],
[10807,27,2],[10807,28,5],[10807,32,33],[10807,35,1],[10807,43,10],
[10807,45,1],[10807,50,1],[10807,59,8],[10808,28,2],[10808,76,1],
[10809,3,1],[10809,23,2],[10809,24,1],[10809,26,3],[10809,31,1],
[10809,35,1],[10809,39,1],[10809,43,2],[10809,63,1],[10810,0,1],
[10810,9,1],[10810,13,1],[10810,29,1],[10810,31,1],[10810,32,1],

[10810,39,1],[10810,73,9],[10811,2,1],[10811,3,4],[10811,6,5],
[10811,16,1],[10811,23,4],[10811,24,16],[10811,25,1],[10811,26,1],
[10811,27,1],[10811,28,1],[10811,31,1],[10811,32,2],[10811,35,1],
[10811,38,2],[10811,40,1],[10811,43,4],[10811,47,2],[10811,50,1],
[10811,56,10],[10811,61,1],[10811,62,5],[10811,66,2],[10811,68,1],
[10811,70,2],[10811,76,2],[10812,6,1],[10812,8,1],[10812,15,1],
[10812,24,1],[10812,27,1],[10812,42,1],[10812,44,1],[10812,51,3],
[10812,54,1],[10812,55,3],[10813,2,2],[10813,5,4],[10813,21,8],
[10813,26,2],[10813,27,2],[10813,32,1],[10813,50,1],[10813,52,1],
[10813,62,4],[10813,66,1],[10813,74,1],[10814,2,1],[10814,51,1],
[10815,28,5],[10815,47,1],[10816,6,1],[10816,22,1],[10816,31,1],
[10816,35,2],[10816,47,1],[10817,22,1],[10817,27,1],[10817,28,1],
[10817,43,1],[10818,2,1],[10818,30,1],[10819,26,1],[10819,42,6],
[10820,20,1],[10820,39,1],[10821,15,1],[10821,22,1],[10821,47,1],
[10821,74,1],[10822,30,1],[10822,76,1],[10823,4,1],[10823,22,1],
[10824,1,2],[10824,7,1],[10824,10,1],[10824,11,1],[10824,12,3],
[10824,13,1],[10824,15,2],[10824,19,2],[10824,20,2],[10824,30,1],
[10824,37,1],[10824,45,2],[10824,55,2],[10824,58,1],[10824,59,1],
[10824,60,2],[10824,65,1],[10824,77,1],[10824,78,1],[10825,27,2],
[10825,28,3],[10825,50,2],[10826,31,2],[10827,1,3],[10827,4,1],
[10827,13,1],[10827,15,1],[10827,17,5],[10827,29,1],[10827,53,6],
[10827,55,1],[10828,1,1],[10828,29,1],[10828,39,1],[10829,0,1],
[10829,1,15],[10829,2,25],[10829,3,50],[10829,4,2],[10829,5,17],
[10829,6,21],[10829,7,2],[10829,8,2],[10829,9,2],[10829,10,6],
[10829,11,11],[10829,12,60],[10829,13,36],[10829,14,1],[10829,15,7],
[10829,16,13],[10829,17,1],[10829,18,4],[10829,19,5],[10829,20,45],
[10829,21,51],[10829,22,36],[10829,23,218],[10829,24,71],
[10829,25,201],[10829,26,219],[10829,27,122],[10829,28,65],
[10829,29,28],[10829,30,27],[10829,31,415],[10829,32,46],
[10829,33,258],[10829,34,4],[10829,35,63],[10829,36,3],[10829,37,3],
[10829,38,9],[10829,39,24],[10829,40,7],[10829,41,1],[10829,42,65],
[10829,43,93],[10829,44,3],[10829,45,8],[10829,46,1],[10829,47,25],
[10829,48,120],[10829,49,1],[10829,50,2],[10829,52,6],[10829,55,2],
[10829,56,2],[10829,57,2],[10829,58,2],[10829,59,33],[10829,60,1],
[10829,61,1],[10829,62,9],[10829,63,3],[10829,65,2],[10829,66,11],
[10829,67,3],[10829,68,1],[10829,69,2],[10829,70,8],[10829,71,2],
[10829,73,1],[10829,74,2],[10829,75,4],[10829,76,3],[10829,77,3],
[10829,78,10],[10830,43,2],[10831,13,2],[10831,45,1],[10832,23,2],
[10832,24,7],[10833,10,1],[10833,11,6],[10833,12,8],[10833,14,2],
[10833,29,1],[10833,40,1],[10833,51,3],[10833,60,1],[10833,61,1],
[10833,70,3],[10834,1,1],[10834,12,1],[10834,14,1],[10834,20,1],
[10834,21,1],[10834,23,1],[10834,24,1],[10834,26,3],[10834,27,1],
[10834,30,2],[10834,42,1],[10834,43,1],[10835,2,1],[10835,35,1],
[10836,23,1],[10836,42,1],[10837,20,5],[10837,53,1],[10837,58,1],
[10837,77,1],[10838,12,1],[10838,29,2],[10839,13,1],[10839,62,1],
[10840,2,1],[10840,23,2],[10840,31,1],[10841,10,1],[10841,16,1],
[10841,46,1],[10842,24,3],[10842,52,2],[10842,62,1],[10842,76,1],
[10843,50,1],[10843,72,1],[10843,75,1],[10843,76,1],[10844,61,4],
[10845,39,4],[10846,27,5],[10847,8,1],[10847,9,3],[10847,10,3],
[10847,11,2],[10847,12,1],[10847,13,1],[10847,18,3],[10847,19,8],

[10847,34,1],[10847,41,3],[10847,45,2],[10847,46,1],[10847,49,1],
[10847,58,1],[10847,59,2],[10847,60,1],[10847,64,1],[10847,65,2],
[10847,67,2],[10847,71,2],[10847,77,3],[10848,20,1],[10848,57,1],
[10849,2,1],[10849,13,1],[10849,62,1],[10850,28,1],[10850,33,1],
[10850,44,1],[10851,5,3],[10851,15,1],[10851,17,1],[10851,19,1],
[10851,22,1],[10851,55,2],[10851,74,4],[10852,26,1],[10852,32,3],
[10852,74,1],[10853,22,1],[10853,24,1],[10854,56,9],[10855,23,2],
[10855,24,2],[10856,22,1],[10856,32,1],[10857,3,1],[10857,5,1],
[10857,6,2],[10857,26,1],[10857,27,1],[10858,1,1],[10858,13,1],
[10858,15,4],[10858,17,1],[10858,19,1],[10858,55,4],[10858,58,1],
[10859,2,1],[10859,3,3],[10859,5,1],[10859,6,1],[10859,21,1],
[10859,22,1],[10859,23,5],[10859,24,8],[10859,25,2],[10859,26,2],
[10859,27,5],[10859,28,3],[10859,31,1],[10859,32,4],[10859,33,2],
[10859,38,1],[10859,42,5],[10859,43,1],[10859,44,1],[10859,47,1],
[10859,48,1],[10859,56,4],[10859,65,1],[10859,78,1],[10860,5,1],
[10860,28,1],[10861,28,1],[10861,60,1],[10862,1,1],[10862,2,1],
[10862,17,1],[10862,30,3],[10862,31,1],[10862,32,1],[10862,60,2],
[10863,23,1],[10863,26,2],[10863,31,1],[10863,33,1],[10863,56,2],
[10864,4,1],[10864,28,1],[10864,44,2],[10864,50,2],[10864,66,2],
[10865,2,3],[10865,70,1],[10866,2,5],[10866,3,1],[10866,4,1],
[10866,7,3],[10866,8,1],[10866,10,2],[10866,13,1],[10866,15,1],
[10866,17,1],[10866,20,5],[10866,23,1],[10866,27,2],[10866,30,1],
[10866,34,2],[10866,35,1],[10866,37,1],[10866,42,1],[10866,45,1],
[10866,46,1],[10866,53,1],[10866,59,1],[10866,65,1],[10866,67,1],
[10866,71,2],[10866,73,1],[10866,76,1],[10866,77,1],[10867,23,6],
[10867,27,1],[10867,47,1],[10868,68,2],[10869,5,2],[10869,13,1],
[10869,21,4],[10869,23,15],[10869,24,4],[10869,26,6],[10869,27,7],
[10869,28,21],[10869,30,2],[10869,31,3],[10869,33,656],[10869,35,4],
[10869,42,13],[10869,43,13],[10869,47,43],[10869,50,1],[10869,51,1],
[10869,62,2],[10869,66,1],[10869,69,1],[10869,70,1],[10869,76,2],
[10869,78,1],[10869,79,2],[10870,22,1],[10870,27,1],[10871,1,1],
[10871,39,1],[10871,43,1],[10871,78,1],[10872,11,1],[10872,15,1],
[10872,24,1],[10872,46,1],[10873,28,1],[10873,42,1],[10874,28,1],
[10874,33,3],[10874,47,1],[10874,64,1],[10875,16,1],[10875,17,1],
[10875,24,1],[10875,31,1],[10876,3,1],[10876,24,4],[10876,56,1],
[10877,2,1],[10877,3,1],[10877,4,1],[10877,5,6],[10877,6,3],
[10877,11,1],[10877,16,1],[10877,18,1],[10877,21,1],[10877,23,1],
[10877,24,4],[10877,25,2],[10877,26,1],[10877,27,3],[10877,28,1],
[10877,31,2],[10877,32,3],[10877,35,3],[10877,38,3],[10877,40,6],
[10877,42,2],[10877,43,1],[10877,44,2],[10877,45,1],[10877,47,1],
[10877,50,3],[10877,51,1],[10877,56,1],[10877,57,1],[10877,59,1],
[10877,61,1],[10877,62,1],[10877,63,123],[10877,67,1],[10877,68,1],
[10877,69,3],[10877,70,11],[10877,72,4],[10877,73,1],[10877,74,131],
[10877,75,134],[10877,76,198],[10878,28,1],[10878,31,3],[10879,4,2],
[10879,28,3],[10879,44,4],[10879,66,2],[10880,42,3],[10881,26,2],
[10882,28,1],[10882,32,1],[10883,1,1],[10883,10,1],[10883,20,1],
[10883,71,1],[10884,47,1],[10884,59,1],[10885,4,2],[10885,5,1],
[10885,22,1],[10885,24,2],[10885,25,1],[10885,26,1],[10885,27,3],
[10885,28,41],[10885,31,1],[10885,35,1],[10885,40,1],[10885,44,1],
[10885,50,1],[10885,51,1],[10885,52,1],[10885,62,1],[10885,63,7],

[10885,68,1],[10885,69,1],[10885,70,2],[10885,74,6],[10885,75,7],
[10885,76,13],[10886,5,2],[10886,23,3],[10886,27,1],[10886,28,1],
[10887,48,4],[10888,28,1],[10888,33,1],[10889,64,1],[10889,71,1],
[10890,1,1],[10890,45,1],[10891,33,3],[10892,9,1],[10892,23,1],
[10893,0,1],[10893,32,1],[10893,39,1],[10894,20,1],[10894,28,1],
[10894,30,1],[10894,32,1],[10894,42,1],[10895,1,1],[10895,7,1],
[10895,8,1],[10895,9,3],[10895,10,6],[10895,11,1],[10895,12,2],
[10895,14,1],[10895,16,2],[10895,17,2],[10895,19,1],[10895,20,1],
[10895,29,1],[10895,30,2],[10895,34,1],[10895,37,1],[10895,41,3],
[10895,45,5],[10895,49,1],[10895,55,2],[10895,57,2],[10895,58,2],
[10895,59,1],[10895,60,1],[10895,65,2],[10895,67,2],[10895,71,2],
[10895,73,2],[10896,12,1],[10896,13,1],[10897,2,2],[10897,23,2],
[10897,26,1],[10897,31,1],[10897,33,2],[10897,43,2],[10897,47,1],
[10897,67,3],[10898,27,1],[10898,28,1],[10898,43,1],[10899,59,1],
[10899,75,1],[10900,27,1],[10900,28,1],[10900,35,3],[10900,42,2],
[10900,66,1],[10901,24,2],[10902,40,2],[10902,72,2],[10903,37,1],
[10903,42,4],[10904,2,1],[10904,58,1],[10905,35,2],[10906,2,1],
[10906,23,1],[10906,25,1],[10906,32,1],[10906,40,1],[10906,68,1],
[10906,70,1],[10906,72,1],[10906,76,1],[10907,15,1],[10907,26,1],
[10907,44,1],[10907,47,1],[10907,50,1],[10907,74,1],[10907,76,1],
[10908,10,2],[10908,53,1],[10908,55,1],[10909,27,5],[10910,4,1],
[10910,5,1],[10910,20,2],[10910,22,1],[10910,26,2],[10910,27,4],
[10910,28,11],[10910,30,4],[10910,33,2],[10910,34,1],[10910,35,6],
[10910,38,1],[10910,40,1],[10910,45,1],[10910,52,2],[10910,57,1],
[10910,70,3],[10911,1,2],[10911,2,1],[10911,27,1],[10911,29,1],
[10911,32,1],[10911,78,1],[10912,28,2],[10913,6,1],[10913,22,1],
[10914,1,1],[10914,2,1],[10914,27,1],[10914,42,1],[10914,43,1],
[10914,53,1],[10915,26,1],[10915,35,1],[10916,8,2],[10916,46,1],
[10917,4,2],[10917,70,1],[10918,0,1],[10918,1,1],[10918,3,1],
[10918,5,1],[10918,12,1],[10918,13,1],[10918,21,1],[10918,25,1],
[10918,30,1],[10918,55,1],[10918,61,1],[10918,62,1],[10918,63,4],
[10918,65,2],[10918,72,1],[10918,74,12],[10918,75,5],[10918,76,10],
[10918,78,1],[10919,23,1],[10919,28,2],[10920,15,1],[10920,55,1],
[10921,28,1],[10921,32,1],[10921,38,1],[10921,44,3],[10921,66,2],
[10921,74,1],[10922,3,1],[10922,4,1],[10922,5,1],[10922,6,1],
[10922,22,1],[10922,23,3],[10922,24,6],[10922,26,1],[10922,27,3],
[10922,33,1],[10922,35,2],[10922,42,4],[10922,43,1],[10922,44,1],
[10922,47,2],[10922,66,2],[10922,68,1],[10922,70,2],[10923,28,12],
[10924,2,13],[10924,4,2],[10924,5,3],[10924,22,2],[10924,23,1],
[10924,24,4],[10924,26,1],[10924,27,1],[10924,28,41],[10924,33,1],
[10924,38,1],[10924,40,3],[10924,44,1],[10924,47,1],[10924,50,1],
[10924,66,1],[10924,70,1],[10924,74,1],[10925,22,1],[10925,28,2],
[10925,33,1],[10925,51,1],[10925,74,1],[10926,5,1],[10926,23,1],
[10926,25,1],[10926,37,2],[10926,42,1],[10926,43,3],[10926,48,1],
[10926,54,1],[10926,64,1],[10926,66,1],[10926,78,7],[10927,0,1],
[10927,16,1],[10927,19,1],[10927,25,2],[10927,28,2],[10927,30,263],
[10927,32,14],[10927,33,26],[10927,35,12],[10927,39,1],[10927,52,5],
[10927,67,1],[10928,2,1],[10928,27,8],[10929,2,2],[10929,15,1],
[10930,3,6],[10930,8,3],[10930,20,2],[10930,21,2],[10930,22,1],
[10930,23,18],[10930,24,13],[10930,25,1],[10930,26,12],[10930,27,5],

[10930,28,5],[10930,31,10],[10930,32,6],[10930,37,2],[10930,38,3],
[10930,42,2],[10930,43,11],[10930,48,1],[10930,56,1],[10930,59,1],
[10931,23,4],[10931,25,1],[10931,26,1],[10931,31,1],[10931,42,1],
[10932,57,1],[10932,77,1],[10933,19,1],[10933,22,1],[10933,65,1],
[10934,23,1],[10934,70,1],[10935,1,2],[10935,9,4],[10935,10,3],
[10935,11,4],[10935,12,2],[10935,13,2],[10935,14,1],[10935,15,1],
[10935,16,3],[10935,19,2],[10935,20,3],[10935,30,1],[10935,36,1],
[10935,37,1],[10935,41,3],[10935,45,6],[10935,46,2],[10935,49,2],
[10935,54,1],[10935,55,2],[10935,58,2],[10935,60,1],[10935,65,7],
[10935,67,5],[10935,71,1],[10935,77,2],[10936,0,1],[10936,27,1],
[10937,12,1],[10937,20,1],[10937,77,2],[10938,5,1],[10938,6,1],
[10938,16,1],[10938,17,2],[10938,20,2],[10938,44,1],[10938,45,1],
[10938,59,1],[10939,26,1],[10939,76,1],[10940,33,2],[10941,3,1],
[10941,13,3],[10941,15,2],[10941,26,12],[10941,44,1],[10941,51,2],
[10941,66,1],[10941,70,2],[10941,72,1],[10942,1,1],[10942,15,2],
[10942,17,1],[10942,24,1],[10943,2,1],[10943,47,1],[10944,13,1],
[10944,20,2],[10944,31,1],[10945,0,1],[10945,8,3],[10945,19,2],
[10945,21,1],[10945,22,2],[10945,25,10],[10945,27,2],[10945,28,5],
[10945,30,21],[10945,33,26],[10945,35,37],[10945,38,3],[10945,40,1],
[10945,50,1],[10945,52,24],[10945,54,1],[10945,66,1],[10945,70,1],
[10945,76,1],[10946,2,1],[10946,24,1],[10947,13,1],[10947,15,1],
[10947,17,4],[10947,39,4],[10948,4,1],[10948,22,2],[10948,27,3],
[10948,35,2],[10948,45,1],[10949,6,1],[10949,13,1],[10949,22,2],
[10949,24,2],[10949,42,1],[10950,26,1],[10950,27,2],[10951,1,1],
[10951,3,4],[10951,5,2],[10951,21,4],[10951,22,3],[10951,23,11],
[10951,24,6],[10951,25,4],[10951,26,11],[10951,27,6],[10951,28,20],
[10951,31,12],[10951,32,5],[10951,35,2],[10951,37,1],[10951,42,3],
[10951,43,13],[10951,45,1],[10951,47,3],[10951,48,3],[10951,50,1],
[10951,70,1],[10952,2,6],[10952,20,3],[10952,34,1],[10952,35,1],
[10952,44,1],[10952,49,1],[10952,53,1],[10952,65,1],[10952,79,1],
[10953,12,1],[10953,30,1],[10954,24,2],[10954,33,1],[10955,5,1],
[10955,28,1],[10955,55,1],[10956,24,1],[10956,32,1],[10956,46,1],
[10957,68,1],[10957,74,4],[10957,76,1],[10958,21,1],[10958,22,1],
[10959,21,1],[10959,26,1],[10959,28,10],[10959,55,2],[10960,22,3],
[10960,24,1],[10960,25,3],[10960,27,3],[10961,1,6],[10961,17,1],
[10961,33,1],[10962,23,1],[10962,26,1],[10962,27,2],[10962,28,4],
[10962,32,2],[10963,33,2],[10964,25,1],[10964,28,1],[10965,0,15],
[10965,1,1],[10965,8,7],[10965,9,1],[10965,10,2],[10965,11,3],
[10965,14,3],[10965,16,5],[10965,19,3],[10965,28,1],[10965,30,1],
[10965,34,23],[10965,37,1],[10965,45,1],[10965,46,1],[10965,49,2],
[10965,57,2],[10965,58,1],[10965,59,1],[10965,65,5],[10965,67,1],
[10965,71,1],[10965,73,2],[10966,42,1],[10966,51,2],[10967,5,1],
[10967,40,1],[10968,3,1],[10968,16,1],[10968,22,2],[10968,23,2],
[10968,27,1],[10968,28,9],[10968,31,2],[10968,41,1],[10968,59,6],
[10968,63,2],[10968,64,1],[10968,68,1],[10968,70,3],[10968,74,1],
[10968,75,1],[10968,76,2],[10969,30,1],[10969,45,1],[10970,59,2],
[10971,2,2],[10971,52,1],[10972,3,2],[10973,11,1],[10973,16,1],
[10974,28,1],[10974,31,3],[10974,43,1],[10974,47,1],[10975,5,2],
[10975,8,1],[10975,22,1],[10975,26,1],[10975,27,2],[10975,28,3],
[10975,32,1],[10975,35,4],[10975,43,1],[10976,5,1],[10976,24,1],

[10976,27,1],[10976,34,1],[10976,47,2],[10976,48,2],[10977,17,1],
[10977,53,4],[10977,55,1],[10978,15,3],[10979,0,1],[10979,1,2],
[10979,10,1],[10979,16,1],[10979,19,2],[10979,37,1],[10979,45,2],
[10979,57,2],[10979,58,1],[10979,67,2],[10979,71,2],[10979,73,1],
[10980,12,1],[10980,20,2],[10981,3,1],[10981,68,1],[10982,24,1],
[10982,29,1],[10982,65,1],[10983,33,2],[10984,23,2],[10984,27,2],
[10985,11,1],[10985,12,1],[10986,13,1],[10986,17,5],[10986,30,2],
[10986,45,1],[10986,53,3],[10987,6,1],[10987,24,1],[10987,27,2],
[10987,31,1],[10987,35,1],[10988,1,3],[10988,13,1],[10989,23,2],
[10989,26,1],[10990,22,1],[10990,35,1],[10990,47,1],[10990,66,1],
[10990,74,1],[10991,21,2],[10991,26,1],[10991,32,2],[10991,43,2],
[10992,1,1],[10992,2,16],[10992,3,2],[10992,22,1],[10992,25,1],
[10992,26,1],[10992,30,2],[10992,37,2],[10992,43,2],[10992,53,1],
[10993,33,4],[10994,53,2],[10994,55,1],[10995,7,4],[10996,3,1],
[10996,24,5],[10996,26,2],[10996,33,1],[10996,56,4],[10996,69,1],
[10997,1,1],[10997,3,1],[10997,4,3],[10997,5,1],[10997,10,1],
[10997,11,1],[10997,13,2],[10997,18,1],[10997,19,1],[10997,25,2],
[10997,27,1],[10997,28,1],[10997,33,1],[10997,35,2],[10997,37,2],
[10997,38,1],[10997,46,1],[10997,50,2],[10997,55,3],[10997,56,3],
[10997,60,2],[10997,62,1],[10997,63,1],[10997,64,1],[10997,66,6],
[10997,67,1],[10997,70,1],[10997,74,1],[10997,75,1],[10997,76,1],
[10998,24,7],[10999,28,8],[10999,30,1],[10999,33,1],[10999,72,1],
[11000,19,1],[11000,26,1],[11001,28,1],[11001,35,1],[11001,47,1],
[11001,58,1],[11002,28,2],[11003,25,1],[11003,33,5],[11003,38,1],
[11004,32,2],[11005,27,1],[11005,68,1],[11006,26,1],[11006,28,1],
[11006,38,1],[11006,40,1],[11006,44,1],[11006,50,1],[11006,61,1],
[11006,68,1],[11006,69,2],[11006,70,2],[11007,44,12],[11007,66,9],
[11008,2,2],[11008,9,2],[11008,23,2],[11008,27,2],[11008,28,2],
[11008,31,1],[11008,33,2],[11009,3,5],[11009,4,1],[11009,21,1],
[11009,22,5],[11009,23,14],[11009,25,3],[11009,26,1],[11009,27,7],
[11009,28,4],[11009,30,1],[11009,32,8],[11009,33,4],[11009,35,1],
[11009,43,1],[11009,56,3],[11009,62,1],[11010,24,1],[11010,25,1],
[11010,27,1],[11010,38,1],[11011,1,2],[11011,2,1],[11011,3,2],
[11011,5,1],[11011,6,2],[11011,13,3],[11011,20,2],[11011,21,1],
[11011,23,3],[11011,24,1],[11011,27,12],[11011,31,1],[11011,32,1],
[11011,33,9],[11011,35,4],[11011,46,1],[11011,47,1],[11011,52,1],
[11011,65,1],[11011,75,1],[11012,3,1],[11012,22,1],[11012,28,1],
[11012,33,1],[11013,1,2],[11013,29,2],[11013,39,1],[11013,77,1],
[11014,1,1],[11014,15,1],[11014,19,1],[11014,20,11],[11014,22,4],
[11014,23,4],[11014,24,1],[11014,28,2],[11014,30,1],[11014,35,1],
[11014,36,1],[11014,57,1],[11015,5,1],[11015,20,1],[11015,22,3],
[11016,21,4],[11017,35,1],[11017,68,1],[11018,3,2],[11019,23,1],
[11019,40,1],[11020,23,1],[11020,47,1],[11021,21,1],[11021,56,1],
[11022,28,1],[11022,32,1],[11023,26,1],[11023,62,1],[11024,6,1],
[11024,24,2],[11024,43,1],[11025,55,1],[11025,67,1],[11026,23,1],
[11026,27,2],[11026,47,1],[11026,48,1],[11027,6,1],[11027,22,3],
[11027,23,1],[11027,27,5],[11027,33,1],[11027,35,4],[11027,47,1],
[11027,52,1],[11027,76,2],[11028,15,1],[11028,53,1],[11029,1,1],
[11029,12,1],[11029,13,1],[11030,3,1],[11030,24,1],[11031,17,3],
[11031,53,1],[11032,20,1],[11032,39,2],[11032,77,1],[11033,33,5],

[11033,39,1],[11033,75,1],[11033,76,1],[11034,27,1],[11034,28,2],
[11035,31,1],[11035,32,1],[11036,3,1],[11036,28,3],[11036,44,2],
[11036,66,1],[11036,70,1],[11037,40,3],[11037,68,3],[11037,69,3],
[11037,70,4],[11037,72,2],[11038,28,1],[11038,69,1],[11039,35,4],
[11040,2,1],[11040,33,1],[11041,5,1],[11041,27,1],[11041,32,1],
[11041,46,1],[11042,42,4],[11043,3,1],[11043,6,1],[11043,22,3],
[11043,23,1],[11043,24,5],[11043,25,1],[11043,27,2],[11043,28,1],
[11043,33,2],[11043,35,2],[11043,37,1],[11043,42,3],[11043,43,1],
[11043,56,2],[11043,64,1],[11043,68,1],[11043,74,3],[11044,17,1],
[11044,53,1],[11045,74,1],[11045,75,1],[11046,28,1],[11046,31,1],
[11046,38,2],[11046,76,1],[11047,47,2],[11048,3,3],[11048,31,1],
[11049,31,1],[11049,33,1],[11049,35,1],[11050,60,2],[11050,75,1],
[11051,15,1],[11051,17,1],[11052,4,1],[11052,38,1],[11052,44,6],
[11052,50,1],[11052,66,9],[11053,8,1],[11053,21,1],[11053,26,1],
[11053,32,4],[11053,35,1],[11053,38,1],[11053,68,1],[11054,2,112],
[11054,24,1],[11054,28,3],[11054,33,2],[11054,35,1],[11054,42,1],
[11055,12,2],[11055,73,1],[11056,17,1],[11056,55,1],[11057,2,4],
[11058,23,2],[11059,23,1],[11059,27,8],[11059,28,3],[11059,47,7],
[11060,33,3],[11060,52,2],[11061,13,1],[11061,39,1],[11061,47,1],
[11062,3,1],[11062,30,1],[11063,0,1],[11063,11,1],[11063,12,2],
[11063,19,1],[11063,36,2],[11063,53,1],[11064,15,2],[11064,17,1],
[11064,53,1],[11064,55,1],[11064,76,1],[11065,20,1],[11065,27,1],
[11066,28,2],[11067,35,1],[11067,48,1],[11068,5,1],[11068,6,1],
[11068,23,1],[11068,24,2],[11068,25,1],[11068,27,7],[11068,47,1],
[11068,62,1],[11069,28,2],[11070,12,2],[11071,2,15],[11071,4,1],
[11072,22,1],[11072,76,1],[11073,22,1],[11073,27,4],[11073,33,2],
[11073,38,1],[11073,47,2],[11073,60,1],[11074,1,1],[11074,4,1],
[11074,17,1],[11074,25,1],[11074,32,1],[11074,50,5],[11074,53,5],
[11074,55,1],[11075,6,1],[11075,28,1],[11075,33,1],[11076,1,1],
[11076,13,4],[11076,15,1],[11076,20,10],[11076,65,1],[11077,27,2],
[11077,35,1],[11077,74,1],[11078,11,1],[11078,59,4],[11079,2,1],
[11079,8,1],[11079,25,1],[11079,28,3],[11079,32,1],[11079,62,2],
[11079,70,1],[11079,77,1],[11080,3,1],[11080,24,1],[11080,26,3],
[11080,28,1],[11080,43,1],[11080,63,1],[11081,62,4],[11082,2,1],
[11082,7,1],[11082,19,1],[11082,22,6],[11082,23,14],[11082,24,9],
[11082,25,2],[11082,26,2],[11082,27,49],[11082,28,3],[11082,32,4],
[11082,33,2],[11082,35,10],[11082,37,1],[11082,42,1],[11082,43,1],
[11082,48,1],[11082,66,1],[11082,68,1],[11083,56,1],[11083,58,1],
[11084,7,1],[11084,12,1],[11085,22,1],[11085,23,1],[11086,27,9],
[11086,35,1],[11087,9,1],[11087,10,1],[11087,14,1],[11087,16,2],
[11087,19,2],[11087,41,1],[11087,49,1],[11087,54,1],[11087,58,1],
[11087,59,1],[11087,71,1],[11087,73,1],[11087,75,1],[11088,5,1],
[11088,22,1],[11088,24,1],[11089,3,3],[11089,4,1],[11089,23,16],
[11089,24,1],[11089,26,2],[11089,28,1],[11089,32,1],[11089,33,1],
[11089,35,1],[11089,43,13],[11089,47,1],[11089,62,1],[11090,1,4],
[11090,15,1],[11091,12,1],[11091,39,4],[11091,55,1],[11092,56,3],
[11093,2,2],[11093,9,1],[11093,22,1],[11093,28,2],[11093,30,1],
[11093,39,1],[11093,55,1],[11094,8,1],[11094,52,1],[11094,53,1],
[11095,2,1],[11095,28,1],[11096,32,2],[11096,34,1],[11096,51,1],
[11097,17,1],[11097,20,1],[11097,53,1],[11098,5,1],[11098,23,1],

[11098,28,4],[11098,33,1],[11098,39,1],[11098,43,7],[11098,56,1],
[11098,62,1],[11098,63,1],[11099,9,2],[11099,24,1],[11099,28,1],
[11099,43,1],[11100,1,2],[11100,19,1],[11100,62,6],[11101,2,3],
[11101,28,1],[11101,31,2],[11101,46,1],[11102,0,1],[11102,8,5],
[11102,24,3],[11102,27,1],[11102,31,1],[11102,32,2],[11102,35,1],
[11102,39,1],[11102,42,1],[11103,1,2],[11103,13,1],[11104,24,2],
[11104,32,3],[11104,35,1],[11104,78,1],[11105,5,1],[11105,24,2],
[11105,26,1],[11105,27,1],[11105,28,3],[11105,31,2],[11105,32,2],
[11105,47,1],[11105,48,1],[11105,56,3],[11105,62,2],[11105,63,1],
[11106,13,1],[11106,60,1],[11107,22,1],[11107,28,1],[11107,32,7],
[11107,33,46],[11107,35,28],[11108,30,1],[11108,53,1],[11109,9,1],
[11109,15,2],[11109,17,1],[11109,19,1],[11109,28,1],[11109,55,1],
[11109,56,1],[11109,57,1],[11109,67,1],[11109,77,1],[11110,23,1],
[11110,25,4],[11110,31,1],[11110,43,1],[11110,47,1],[11111,3,1],
[11111,5,1],[11112,5,1],[11112,21,2],[11113,20,1],[11113,22,2],
[11113,23,1],[11113,28,1],[11114,23,1],[11114,24,1],[11115,3,1],
[11115,6,1],[11115,44,1],[11115,56,2],[11115,61,1],[11115,69,1],
[11116,23,1],[11116,28,15],[11116,51,1],[11117,24,1],[11117,27,2],
[11118,22,1],[11118,23,2],[11118,27,2],[11118,47,3],[11118,51,3],
[11118,56,1],[11118,61,1],[11118,79,1],[11119,15,1],[11119,55,1],
[11119,77,1],[11120,44,1],[11120,66,1],[11121,11,1],[11121,35,1],
[11121,53,1],[11121,63,1],[11122,6,1],[11122,21,1],[11122,27,2],
[11122,50,1],[11122,52,1],[11123,4,1],[11123,8,1],[11123,24,2],
[11123,31,2],[11123,43,1],[11124,10,1],[11124,77,1],[11125,23,1],
[11125,24,1],[11125,32,1],[11125,36,1],[11126,2,6],[11126,62,1],
[11127,34,1],[11127,55,1],[11128,25,1],[11128,33,3],[11129,32,2],
[11130,13,1],[11130,41,1],[11130,77,1],[11131,15,1],[11131,23,1],
[11131,76,4],[11132,3,2],[11132,6,1],[11133,3,1],[11133,12,1],
[11133,22,1],[11133,24,1],[11133,26,1],[11133,28,1],[11133,43,1],
[11134,3,4],[11134,6,1],[11134,27,2],[11134,35,1],[11134,47,2],
[11135,0,1],[11135,15,2],[11135,59,1],[11135,60,1],[11135,65,1],
[11136,2,1],[11136,3,3],[11136,5,4],[11136,21,1],[11136,22,6],
[11136,23,9],[11136,24,6],[11136,25,3],[11136,27,7],[11136,28,2],
[11136,30,1],[11136,31,3],[11136,32,3],[11136,33,2],[11136,35,2],
[11136,43,3],[11136,47,1],[11136,56,1],[11136,62,1],[11137,9,3],
[11137,20,1],[11137,23,3],[11137,24,1],[11137,29,1],[11137,33,1],
[11137,39,2],[11137,43,1],[11137,60,1],[11137,77,1],[11138,35,2],
[11139,15,3],[11139,44,1],[11139,66,1],[11140,27,1],[11140,28,1],
[11141,0,2],[11141,1,43],[11141,2,36],[11141,3,1],[11141,4,1],
[11141,7,1],[11141,8,6],[11141,12,14],[11141,13,41],[11141,15,1],
[11141,16,6],[11141,17,2],[11141,18,1],[11141,20,3],[11141,23,7],
[11141,24,2],[11141,25,2],[11141,26,1],[11141,27,17],[11141,28,10],
[11141,29,5],[11141,30,11],[11141,31,4],[11141,32,1],[11141,33,3],
[11141,34,5],[11141,35,7],[11141,36,2],[11141,37,2],[11141,38,2],
[11141,39,29],[11141,40,1],[11141,42,2],[11141,43,5],[11141,44,1],
[11141,47,6],[11141,48,8],[11141,50,3],[11141,51,2],[11141,52,1],
[11141,53,2],[11141,55,1],[11141,59,2],[11141,62,2],[11141,73,1],
[11141,74,1],[11141,78,1],[11141,79,1],[11142,32,1],[11142,62,1],
[11143,3,1],[11143,17,1],[11143,22,1],[11143,31,1],[11143,43,1],
[11143,53,1],[11144,28,2],[11145,22,1],[11145,31,1],[11146,4,2],

[11146,6,1],[11146,56,1],[11146,70,2],[11147,2,1],[11147,3,2],
[11147,21,1],[11147,22,2],[11147,28,3],[11147,31,1],[11147,42,1],
[11147,43,1],[11147,50,1],[11147,56,1],[11147,63,1],[11148,26,1],
[11148,27,1],[11148,44,1],[11149,49,1],[11149,53,1],[11149,70,1],
[11150,3,1],[11150,23,1],[11151,4,1],[11151,5,1],[11151,21,1],
[11151,35,1],[11151,42,1],[11152,33,2],[11153,63,7],[11153,74,2],
[11153,75,1],[11153,76,3],[11154,2,1],[11154,44,1],[11155,3,1],
[11155,23,1],[11155,25,3],[11155,26,1],[11156,2,4],[11156,3,2],
[11156,5,2],[11156,8,1],[11156,21,2],[11156,23,1],[11156,27,2],
[11156,28,1],[11156,31,3],[11156,32,2],[11156,35,1],[11156,37,1],
[11156,38,1],[11156,43,1],[11156,50,1],[11156,60,1],[11157,23,8],
[11157,26,1],[11157,33,4],[11157,38,1],[11157,56,1],[11158,51,2],
[11159,26,4],[11159,43,3],[11160,22,6],[11160,24,3],[11160,27,3],
[11160,35,1],[11161,27,26],[11161,28,4],[11161,33,1],[11161,35,21],
[11162,26,1],[11162,47,1],[11163,3,1],[11163,5,2],[11163,17,1],
[11163,20,1],[11163,21,7],[11163,22,20],[11163,23,7],[11163,24,7],
[11163,25,11],[11163,26,6],[11163,27,9],[11163,28,1],[11163,30,3],
[11163,31,2],[11163,32,5],[11163,35,4],[11163,38,2],[11163,39,1],
[11163,42,1],[11163,43,1],[11163,45,1],[11163,50,1],[11163,53,3],
[11163,62,4],[11164,4,2],[11164,5,2],[11164,6,2],[11164,21,1],
[11164,26,2],[11164,27,1],[11164,28,1],[11164,37,5],[11164,40,1],
[11164,44,3],[11164,45,1],[11164,47,2],[11164,50,3],[11164,56,3],
[11164,62,1],[11164,74,3],[11165,28,6],[11166,5,2],[11166,50,1],
[11167,5,1],[11167,56,1],[11168,1,1],[11168,5,1],[11168,6,3],
[11168,10,3],[11168,11,1],[11168,12,2],[11168,13,2],[11168,15,2],
[11168,16,1],[11168,20,2],[11168,22,1],[11168,24,1],[11168,26,6],
[11168,38,3],[11168,41,1],[11168,45,1],[11168,53,2],[11168,55,2],
[11168,59,1],[11168,64,1],[11168,65,1],[11168,71,1],[11169,28,17],
[11170,2,2],[11171,30,2],[11172,2,1],[11172,25,1],[11172,28,6],
[11172,42,2],[11173,3,1],[11173,9,1],[11173,10,1],[11173,23,1],
[11173,57,1],[11174,28,1],[11174,42,2],[11174,56,1],[11175,20,16],
[11175,28,1],[11175,42,4],[11176,13,1],[11176,62,1],[11177,20,1],
[11177,21,1],[11178,5,1],[11178,27,1],[11179,4,12],[11179,5,6],
[11179,8,3],[11179,9,4],[11179,10,4],[11179,12,3],[11179,13,1],
[11179,14,1],[11179,15,13],[11179,16,2],[11179,17,44],[11179,18,1],
[11179,22,1],[11179,23,1],[11179,25,1],[11179,28,1],[11179,29,1],
[11179,30,1],[11179,32,1],[11179,34,2],[11179,35,2],[11179,37,2],
[11179,38,2],[11179,39,1],[11179,41,1],[11179,44,3],[11179,45,1],
[11179,46,2],[11179,50,6],[11179,53,42],[11179,54,1],[11179,55,11],
[11179,59,1],[11179,63,1],[11179,65,1],[11179,66,7],[11179,77,1],
[11179,78,1],[11180,3,3],[11180,24,2],[11180,42,1],[11180,62,1],
[11181,28,9],[11181,44,2],[11181,66,2],[11182,25,2],[11182,28,3],
[11182,32,1],[11183,28,1],[11183,44,2],[11183,66,1],[11184,1,2],
[11184,9,3],[11184,10,1],[11184,18,1],[11184,19,3],[11184,28,2],
[11184,36,1],[11184,45,2],[11184,46,1],[11184,49,1],[11184,53,2],
[11184,57,1],[11184,67,1],[11184,75,1],[11184,76,1],[11185,4,1],
[11185,5,1],[11185,11,1],[11185,27,1],[11185,28,1],[11185,41,1],
[11185,53,1],[11185,55,1],[11185,59,1],[11186,23,1],[11186,28,1],
[11186,30,3],[11186,64,1],[11187,15,1],[11187,73,1],[11188,1,8],
[11188,29,1],[11188,39,1],[11189,2,1],[11189,30,1],[11190,0,1],

[11190,2,4],[11190,20,1],[11190,31,2],[11190,39,1],[11190,45,1],
[11190,59,1],[11190,65,1],[11191,20,1],[11191,59,2],[11192,9,1],
[11192,17,1],[11193,12,9],[11193,23,1],[11193,24,2],[11193,26,1],
[11193,28,3],[11193,33,1],[11193,35,1],[11193,38,1],[11193,39,1],
[11193,42,1],[11193,56,1],[11193,63,2],[11193,66,2],[11193,68,3],
[11193,70,1],[11194,0,1],[11194,1,8],[11194,2,4],[11194,3,4],
[11194,4,2],[11194,6,8],[11194,8,1],[11194,12,5],[11194,13,7],
[11194,16,1],[11194,17,2],[11194,21,2],[11194,23,1],[11194,24,2],
[11194,26,3],[11194,27,1],[11194,28,1],[11194,29,1],[11194,30,30],
[11194,32,1],[11194,33,1],[11194,34,3],[11194,35,3],[11194,36,1],
[11194,37,2],[11194,39,4],[11194,40,1],[11194,43,2],[11194,47,10],
[11194,48,1],[11194,50,2],[11194,51,1],[11194,53,2],[11194,63,1],
[11194,68,1],[11194,70,1],[11194,73,3],[11194,79,1],[11195,2,1],
[11195,3,2],[11195,8,1],[11195,23,3],[11195,25,1],[11195,27,2],
[11195,28,1],[11195,30,2],[11195,31,1],[11195,32,2],[11195,37,1],
[11195,47,2],[11196,32,1],[11196,62,2],[11197,27,16],[11197,43,1],
[11198,37,2],[11199,4,1],[11199,35,1],[11199,42,1],[11199,50,1],
[11200,28,1],[11200,48,4],[11201,26,1],[11201,28,1],[11202,63,4],
[11203,65,1],[11203,71,1],[11204,25,2],[11205,2,1],[11205,33,1],
[11206,6,1],[11206,31,1],[11206,34,1],[11206,43,1],[11207,28,1],
[11207,32,1],[11207,44,1],[11207,47,1],[11208,3,2],[11209,28,1],
[11209,76,1],[11210,2,3],[11210,6,1],[11211,6,1],[11211,22,1],
[11211,28,1],[11212,0,1],[11212,1,10],[11212,2,3],[11212,3,3],
[11212,4,128],[11212,5,15],[11212,8,1],[11212,9,15],[11212,10,19],
[11212,11,8],[11212,12,9],[11212,13,6],[11212,14,3],[11212,15,340],
[11212,16,15],[11212,17,201],[11212,18,2],[11212,19,18],[11212,20,9],
[11212,22,4],[11212,23,6],[11212,25,5],[11212,26,2],[11212,27,2],
[11212,28,13],[11212,29,2],[11212,30,1],[11212,31,2],[11212,32,7],
[11212,33,5],[11212,34,6],[11212,35,6],[11212,36,2],[11212,37,5],
[11212,38,35],[11212,39,1],[11212,40,2],[11212,41,6],[11212,42,1],
[11212,43,2],[11212,44,390],[11212,45,13],[11212,46,8],[11212,47,1],
[11212,48,1],[11212,49,6],[11212,50,143],[11212,51,1],[11212,53,227],
[11212,54,6],[11212,55,205],[11212,56,5],[11212,57,7],[11212,58,9],
[11212,59,9],[11212,60,4],[11212,61,1],[11212,62,1],[11212,63,2],
[11212,64,3],[11212,65,8],[11212,66,294],[11212,67,4],[11212,68,3],
[11212,69,1],[11212,70,12],[11212,71,8],[11212,72,1],[11212,73,2],
[11212,74,3],[11212,75,4],[11212,76,1],[11212,77,7],[11212,78,3],
[11212,79,1],[11213,4,1],[11213,28,3],[11213,40,1],[11213,68,1],
[11213,70,6],[11214,10,2],[11215,20,1],[11215,27,1],[11216,5,2],
[11216,50,1],[11217,24,2],[11218,28,1],[11218,72,1],[11219,28,1],
[11219,72,1],[11220,28,1],[11220,61,2],[11221,4,1],[11221,42,1],
[11221,44,3],[11222,16,1],[11222,18,1],[11223,2,1],[11223,26,1],
[11223,32,1],[11223,56,1],[11223,68,1],[11224,32,2],[11225,5,1],
[11225,6,1],[11225,22,1],[11225,26,2],[11225,28,3],[11225,32,1],
[11225,33,2],[11225,43,1],[11225,63,1],[11226,27,1],[11226,35,1],
[11227,28,1],[11227,43,1],[11228,20,1],[11228,25,1],[11228,72,1],
[11229,30,2],[11230,2,1],[11230,27,1],[11230,28,1],[11230,31,1],
[11230,35,1],[11231,28,2],[11232,3,2],[11232,6,1],[11232,22,1],
[11232,23,1],[11232,28,6],[11232,33,2],[11232,35,1],[11232,40,2],
[11232,44,1],[11232,51,1],[11232,62,1],[11232,74,3],[11232,75,1],

[11232,76,1],[11233,1,2],[11233,12,1],[11233,13,1],[11234,17,2],
[11234,18,1],[11234,49,1],[11234,53,7],[11234,55,1],[11234,65,1],
[11235,20,3],[11236,27,2],[11236,28,1],[11237,38,11],[11237,75,1],
[11238,23,1],[11238,28,1],[11239,28,1],[11239,68,1],[11239,70,2],
[11239,76,1],[11240,21,1],[11240,27,1],[11240,43,1],[11240,48,1],
[11241,13,1],[11241,27,1],[11242,3,1],[11242,4,1],[11242,25,1],
[11242,27,5],[11243,2,2],[11244,23,2],[11244,47,1],[11245,51,4],
[11246,15,1],[11246,17,1],[11247,2,5],[11248,23,1],[11248,24,1],
[11248,27,15],[11248,47,1],[11248,56,2],[11248,76,1],[11249,3,1],
[11249,28,1],[11249,48,2],[11250,39,2],[11251,62,2],[11252,9,1],
[11252,77,1],[11253,28,1],[11253,38,1],[11253,52,47],[11254,28,1],
[11254,47,1],[11255,57,1],[11255,67,1],[11256,15,1],[11256,23,1],
[11256,24,2],[11256,30,1],[11256,33,1],[11256,47,4],[11256,51,1],
[11257,21,1],[11257,66,2],[11258,18,1],[11258,35,1],[11258,39,1],
[11259,2,3],[11260,9,1],[11260,12,1],[11260,18,1],[11261,27,1],
[11261,70,1],[11262,31,1],[11262,47,1],[11263,2,2],[11263,26,1],
[11263,31,4],[11263,48,1],[11264,15,2],[11264,55,3],[11265,21,1],
[11265,23,1],[11265,32,2],[11265,48,1],[11266,28,1],[11266,38,1],
[11266,44,3],[11266,47,1],[11266,51,1],[11266,61,1],[11267,33,1],
[11267,65,1],[11268,40,1],[11268,68,1],[11268,72,1],[11269,44,1],
[11269,50,1],[11269,66,1],[11270,42,2],[11271,2,1],[11271,3,1],
[11271,6,2],[11271,20,1],[11271,23,4],[11271,24,2],[11271,25,3],
[11271,27,1],[11271,32,6],[11271,33,3],[11271,35,3],[11271,42,3],
[11271,43,8],[11271,51,1],[11271,68,2],[11271,70,2],[11271,75,1],
[11272,6,1],[11272,32,1],[11272,40,1],[11272,74,1],[11273,8,1],
[11273,42,1],[11274,15,2],[11274,28,2],[11274,32,1],[11274,41,2],
[11274,52,1],[11274,79,1],[11275,27,1],[11275,43,1],[11276,12,1],
[11276,18,1],[11276,28,1],[11276,29,1],[11276,46,1],[11276,55,1],
[11277,26,1],[11277,38,1],[11278,15,2],[11278,17,1],[11278,53,1],
[11278,55,1],[11279,17,1],[11279,44,1],[11279,53,1],[11280,24,2],
[11281,22,1],[11281,27,1],[11282,23,1],[11282,28,2],[11283,23,1],
[11283,24,1],[11283,38,8],[11284,1,1],[11284,15,2],[11284,28,1],
[11284,50,1],[11284,55,2],[11284,66,1],[11285,30,1],[11285,45,1],
[11286,3,4],[11286,22,1],[11286,38,2],[11287,3,1],[11287,5,1],
[11287,21,1],[11287,22,1],[11287,23,5],[11287,24,3],[11287,32,1],
[11287,35,1],[11287,42,13],[11287,43,2],[11287,47,1],[11287,56,1],
[11288,2,1],[11288,23,1],[11288,25,1],[11288,27,1],[11288,28,1],
[11288,32,1],[11288,35,1],[11289,15,1],[11289,28,3],[11289,40,1],
[11289,44,1],[11289,50,1],[11289,52,1],[11289,56,2],[11290,1,2],
[11291,24,1],[11291,27,1],[11291,28,6],[11291,33,1],[11291,43,1],
[11292,3,2],[11292,21,1],[11292,23,1],[11292,24,1],[11292,25,2],
[11292,26,1],[11292,27,3],[11292,28,2],[11292,31,3],[11292,32,4],
[11292,33,1],[11292,42,7],[11292,43,1],[11292,63,1],[11293,25,1],
[11293,28,1],[11294,28,2],[11295,48,2],[11296,2,1],[11296,25,1],
[11297,31,1],[11297,38,1],[11298,20,3],[11298,23,1],[11298,26,1],
[11298,35,1],[11299,27,1],[11299,28,1],[11300,28,1],[11300,31,3],
[11300,44,1],[11301,2,1],[11301,23,1],[11302,15,1],[11302,17,2],
[11302,53,3],[11302,55,1],[11303,24,2],[11304,28,1],[11304,53,2],
[11305,15,3],[11305,30,1],[11305,55,4],[11306,2,5],[11306,3,5],
[11306,21,2],[11306,22,1],[11306,23,5],[11306,24,4],[11306,26,3],

[11306,27,1],[11306,28,4],[11306,31,1],[11306,32,2],[11306,33,3],
[11306,35,2],[11306,37,2],[11306,42,2],[11306,43,15],[11306,47,1],
[11306,48,1],[11306,56,1],[11307,27,1],[11307,40,1],[11307,44,1],
[11308,5,1],[11308,15,1],[11308,31,1],[11308,55,1],[11309,0,142],
[11309,1,2],[11309,7,121],[11309,8,2],[11309,9,6],[11309,10,6],
[11309,11,4],[11309,12,5],[11309,13,5],[11309,14,3],[11309,16,12],
[11309,18,9],[11309,19,3],[11309,29,1],[11309,30,3],[11309,31,4],
[11309,36,4],[11309,37,2],[11309,41,1],[11309,45,8],[11309,46,4],
[11309,57,6],[11309,60,1],[11309,62,1],[11309,64,1],[11309,65,6],
[11309,67,1],[11309,73,6],[11309,77,12],[11309,78,1],[11310,2,2],
[11310,3,3],[11310,20,1],[11310,23,2],[11310,24,4],[11310,27,9],
[11310,35,6],[11310,38,1],[11310,42,3],[11310,44,1],[11310,46,1],
[11310,47,1],[11310,48,1],[11310,50,2],[11310,52,1],[11310,56,2],
[11310,60,1],[11310,76,1],[11311,1,6],[11311,2,5],[11311,3,4],
[11311,4,122],[11311,5,14],[11311,6,2],[11311,17,1],[11311,21,4],
[11311,22,3],[11311,23,3],[11311,24,9],[11311,25,5],[11311,26,5],
[11311,27,6],[11311,28,95],[11311,31,4],[11311,32,8],[11311,33,12],
[11311,35,6],[11311,38,108],[11311,40,5],[11311,42,6],[11311,43,5],
[11311,44,1058],[11311,47,5],[11311,48,4],[11311,50,142],[11311,51,2],
[11311,52,1],[11311,56,4],[11311,61,1],[11311,62,10],[11311,63,4],
[11311,66,963],[11311,68,2],[11311,69,6],[11311,70,13],[11311,72,2],
[11311,74,14],[11311,75,8],[11311,76,6],[11311,79,1],[11312,4,1],
[11312,15,2],[11312,31,1],[11312,55,2],[11313,1,1],[11313,12,1],
[11313,13,1],[11313,39,1],[11314,28,2],[11315,1,1],[11315,20,6],
[11315,31,1],[11315,32,1],[11316,62,2],[11317,6,3],[11317,21,1],
[11317,23,1],[11317,24,1],[11317,26,1],[11317,28,1],[11317,31,4],
[11317,38,1],[11317,40,1],[11317,42,1],[11317,43,7],[11317,48,1],
[11317,62,1],[11317,76,1],[11318,26,2],[11318,35,1],[11319,14,1],
[11319,32,1],[11320,15,1],[11320,53,3],[11320,55,2],[11321,2,5],
[11322,28,1],[11322,33,1],[11322,35,2],[11323,26,1],[11323,66,1],
[11324,26,1],[11324,27,1],[11324,32,1],[11325,12,7],[11325,29,7],
[11326,70,1],[11326,76,1],[11327,2,1],[11327,21,1],[11327,23,5],
[11327,26,2],[11327,28,2],[11327,31,1],[11327,33,2],[11327,42,6],
[11328,4,7],[11328,38,2],[11328,44,2],[11328,50,11],[11328,66,6],
[11329,23,8],[11329,24,2],[11329,28,1],[11329,68,1],[11330,2,1],
[11330,3,3],[11330,5,1],[11330,13,3],[11330,21,1],[11330,22,4],
[11330,23,4],[11330,24,11],[11330,26,2],[11330,27,9],[11330,28,4],
[11330,31,1],[11330,32,1],[11330,33,13],[11330,35,1],[11330,40,1],
[11330,42,1],[11330,43,3],[11330,44,1],[11330,48,1],[11330,51,1],
[11330,62,2],[11330,68,1],[11330,70,2],[11330,74,1],[11331,27,1],
[11331,32,1],[11331,35,1],[11332,3,1],[11332,30,1],[11332,33,4],
[11332,35,1],[11332,68,1],[11332,70,1],[11332,76,1],[11333,23,2],
[11334,12,4],[11334,13,1],[11335,32,1],[11335,43,1],[11336,3,1],
[11336,5,1],[11337,27,1],[11337,68,1],[11338,19,1],[11338,30,1],
[11338,77,1],[11339,2,4],[11339,3,1],[11339,28,1],[11339,35,1],
[11339,43,1],[11339,62,2],[11340,1,1],[11340,2,9],[11340,6,1],
[11340,9,2],[11340,12,1],[11340,28,2],[11340,30,6],[11340,38,1],
[11340,47,1],[11340,59,1],[11341,1,1],[11341,6,3],[11341,8,2],
[11341,23,2],[11341,24,1],[11341,27,6],[11341,32,6],[11341,33,2],
[11341,34,2],[11341,35,6],[11342,2,3],[11342,68,1],[11343,28,6],

[11344,10,1],[11344,55,1],[11345,4,1],[11345,28,28],[11346,2,2],
[11346,5,2],[11346,9,1],[11346,21,3],[11346,22,1],[11346,23,4],
[11346,25,3],[11346,26,1],[11346,28,1],[11346,31,3],[11346,33,1],
[11346,35,3],[11346,40,2],[11346,42,1],[11346,43,2],[11346,47,2],
[11346,48,3],[11346,50,1],[11346,58,1],[11346,64,1],[11346,72,1],
[11347,23,2],[11347,24,1],[11347,47,1],[11348,3,1],[11348,6,1],
[11348,10,1],[11348,13,1],[11348,19,1],[11348,24,12],[11348,28,2],
[11348,31,1],[11348,32,1],[11348,37,1],[11348,41,1],[11348,53,1],
[11348,76,1],[11349,3,1],[11349,6,4],[11349,24,1],[11349,38,1],
[11349,56,1],[11349,70,1],[11349,72,2],[11349,74,5],[11350,1,3],
[11350,29,1],[11351,20,1],[11351,28,1],[11351,43,1],[11352,4,1],
[11352,22,3],[11352,23,1],[11352,25,1],[11352,26,1],[11352,27,4],
[11352,33,9],[11352,35,1],[11352,62,1],[11352,68,1],[11353,2,2],
[11353,28,1],[11354,1,46],[11354,2,45],[11354,3,31],[11354,4,1],
[11354,5,26],[11354,6,5],[11354,7,1],[11354,8,2],[11354,9,7],
[11354,10,9],[11354,11,5],[11354,12,4],[11354,13,73],[11354,14,2],
[11354,15,3],[11354,16,3],[11354,17,3],[11354,18,5],[11354,19,4],
[11354,20,118],[11354,21,26],[11354,22,44],[11354,23,84],
[11354,24,41],[11354,25,17],[11354,26,29],[11354,27,59],[11354,28,42],
[11354,29,1],[11354,30,14],[11354,31,31],[11354,32,41],[11354,33,17],
[11354,34,12],[11354,35,28],[11354,36,3],[11354,37,5],[11354,38,7],
[11354,39,11],[11354,40,3],[11354,41,1],[11354,42,18],[11354,43,32],
[11354,44,2],[11354,45,9],[11354,46,6],[11354,47,5],[11354,48,13],
[11354,50,1],[11354,51,1],[11354,52,2],[11354,53,3],[11354,54,1],
[11354,55,7],[11354,56,5],[11354,57,3],[11354,58,1],[11354,59,19],
[11354,60,6],[11354,62,9],[11354,63,2],[11354,65,1],[11354,66,3],
[11354,67,3],[11354,68,3],[11354,69,1],[11354,70,7],[11354,71,1],
[11354,72,1],[11354,73,7],[11354,74,3],[11354,75,1],[11354,76,1],
[11354,77,3],[11354,78,2],[11355,25,1],[11355,50,1],[11355,62,1],
[11355,69,1],[11355,70,3],[11355,72,1],[11355,76,1],[11356,2,1],
[11356,15,1],[11356,18,2],[11356,20,2],[11356,21,1],[11356,23,3],
[11356,32,1],[11356,73,1],[11357,20,2],[11357,22,3],[11357,23,2],
[11357,31,2],[11357,32,2],[11357,33,4],[11358,33,15],[11359,1,7],
[11359,8,2],[11359,9,10],[11359,10,9],[11359,11,2],[11359,12,10],
[11359,13,9],[11359,14,3],[11359,15,3],[11359,16,5],[11359,17,4],
[11359,19,6],[11359,20,3],[11359,29,3],[11359,30,2],[11359,34,2],
[11359,36,3],[11359,37,3],[11359,39,8],[11359,41,4],[11359,45,8],
[11359,49,2],[11359,53,6],[11359,54,2],[11359,55,4],[11359,57,1],
[11359,58,2],[11359,59,4],[11359,64,2],[11359,65,5],[11359,71,1],
[11359,73,2],[11359,74,1],[11359,77,3],[11359,78,1],[11360,0,1],
[11360,1,5],[11360,3,1],[11360,8,1],[11360,10,1],[11360,13,3],
[11360,17,2],[11360,19,1],[11360,20,3],[11360,26,2],[11360,27,1],
[11360,28,2],[11360,29,1],[11360,30,7],[11360,32,2],[11360,34,1],
[11360,39,4],[11360,42,2],[11360,48,2],[11360,53,2],[11360,55,1],
[11360,73,1],[11360,77,1],[11361,35,1],[11361,56,1],[11362,32,3],
[11363,5,2],[11363,21,5],[11363,22,5],[11363,23,8],[11363,24,4],
[11363,26,3],[11363,27,5],[11363,28,6],[11363,35,1],[11363,42,1],
[11363,43,1],[11363,61,1],[11364,5,1],[11364,20,1],[11364,35,1],
[11365,28,1],[11365,68,1],[11366,59,2],[11367,23,1],[11367,33,1],
[11368,25,2],[11368,27,1],[11368,42,1],[11369,3,1],[11369,23,1],

[11369,24,1],[11369,27,1],[11370,5,1],[11370,24,6],[11370,62,2],
[11371,15,1],[11371,18,1],[11371,55,4],[11372,5,1],[11372,15,6],
[11372,17,1],[11373,22,1],[11373,24,1],[11373,50,1],[11373,69,2],
[11374,5,1],[11374,16,1],[11374,19,1],[11374,28,2],[11374,32,1],
[11374,35,1],[11374,42,1],[11374,43,1],[11374,52,1],[11374,59,1],
[11374,62,1],[11374,76,2],[11375,27,1],[11375,33,1],[11376,27,1],
[11376,33,3],[11376,47,1],[11376,62,1],[11376,68,1],[11377,0,9],
[11377,37,1],[11377,45,1],[11377,60,1],[11378,61,1],[11378,68,4],
[11379,7,8],[11379,9,1],[11380,25,15],[11380,27,1],[11380,75,1],
[11381,15,2],[11381,53,1],[11381,55,1],[11382,24,3],[11382,34,4],
[11383,33,1],[11383,43,1],[11384,25,1],[11384,28,1],[11384,32,1],
[11384,43,1],[11384,48,1],[11385,10,1],[11385,21,1],[11386,27,1],
[11386,35,1],[11386,62,1],[11387,3,7],[11387,21,1],[11387,23,3],
[11387,25,3],[11387,26,1],[11387,27,13],[11387,28,1],[11387,30,3],
[11387,32,2],[11387,35,7],[11387,42,1],[11387,43,8],[11387,48,7],
[11387,50,1],[11387,56,1],[11387,64,1],[11388,2,1],[11388,8,1],
[11388,13,1],[11388,22,7],[11388,23,46],[11388,26,1],[11388,27,1],
[11388,28,3],[11388,33,21],[11388,38,3],[11388,39,1],[11388,46,1],
[11388,47,1],[11388,50,1],[11388,55,1],[11388,64,1],[11388,66,1],
[11388,70,2],[11388,73,1],[11388,74,1],[11388,76,2],[11389,22,3],
[11389,28,1],[11390,2,1],[11390,68,1],[11391,17,1],[11391,53,1],
[11392,31,2],[11393,21,7],[11394,2,1],[11394,3,1],[11394,4,19],
[11394,5,1],[11394,15,6],[11394,17,21],[11394,23,2],[11394,24,1],
[11394,28,4],[11394,31,3],[11394,32,3],[11394,33,3],[11394,35,1],
[11394,38,2],[11394,39,1],[11394,42,1],[11394,43,1],[11394,44,4],
[11394,50,30],[11394,53,28],[11394,55,6],[11394,63,1],[11394,66,4],
[11394,76,1],[11395,6,1],[11395,13,1],[11395,27,1],[11395,47,2],
[11395,56,9],[11396,63,1],[11396,70,1],[11396,74,1],[11397,15,1],
[11397,55,1],[11398,22,1],[11398,38,1],[11399,20,7],[11399,31,1],
[11400,2,1],[11400,28,1],[11400,42,1],[11401,26,1],[11401,27,1],
[11401,28,1],[11401,29,1],[11401,30,1],[11401,31,1],[11401,38,1],
[11402,6,1],[11402,28,1],[11403,27,7],[11404,3,1],[11404,44,1],
[11405,75,2],[11405,76,1],[11406,10,1],[11406,32,1],[11406,58,1],
[11406,67,1],[11407,45,2],[11408,28,2],[11409,2,1],[11409,32,3],
[11410,28,87],[11411,0,15],[11411,1,17],[11411,2,6],[11411,3,61],
[11411,4,1],[11411,6,1],[11411,7,7],[11411,8,5],[11411,9,14],
[11411,10,14],[11411,11,14],[11411,12,27],[11411,13,22],[11411,14,10],
[11411,15,10],[11411,16,16],[11411,17,5],[11411,18,6],[11411,19,17],
[11411,20,36],[11411,21,1],[11411,22,2],[11411,23,139],[11411,24,17],
[11411,26,8],[11411,27,5],[11411,28,4],[11411,29,18],[11411,30,16],
[11411,31,2],[11411,32,4],[11411,33,2],[11411,34,46],[11411,35,6],
[11411,36,2],[11411,37,5],[11411,38,1],[11411,39,26],[11411,40,2],
[11411,41,13],[11411,42,4],[11411,43,3],[11411,44,1],[11411,45,11],
[11411,46,8],[11411,47,13],[11411,48,1],[11411,49,5],[11411,50,2],
[11411,53,11],[11411,54,6],[11411,55,2],[11411,57,6],[11411,58,3],
[11411,59,8],[11411,60,1],[11411,62,6],[11411,64,1],[11411,65,13],
[11411,66,1],[11411,67,7],[11411,69,3],[11411,70,1],[11411,71,9],
[11411,73,3],[11411,74,2],[11411,76,1],[11411,77,5],[11411,78,2],
[11412,44,1],[11412,66,1],[11413,2,2],[11413,26,1],[11413,31,4],
[11414,3,3],[11415,4,1],[11415,50,1],[11416,1,3],[11416,13,1],

[11416,20,1],[11417,26,1],[11417,42,1],[11418,2,1],[11418,5,1],
[11419,23,2],[11420,20,1],[11420,55,1],[11420,73,1],[11421,28,1],
[11421,74,1],[11422,26,4],[11423,12,1],[11423,13,1],[11423,31,1],
[11423,45,1],[11423,54,1],[11423,57,1],[11424,2,2],[11425,2,3],
[11426,22,1],[11426,23,2],[11426,27,1],[11427,32,3],[11428,9,1],
[11428,20,1],[11428,27,1],[11428,30,1],[11429,3,1],[11429,4,1],
[11429,28,8],[11429,50,1],[11430,18,1],[11430,22,2],[11430,26,1],
[11430,27,3],[11430,28,1],[11430,30,1],[11430,33,9],[11430,35,9],
[11430,42,1],[11430,48,1],[11430,62,7],[11431,4,1],[11431,38,1],
[11431,44,7],[11431,66,10],[11432,9,2],[11432,10,1],[11432,20,1],
[11432,30,31],[11432,36,1],[11432,41,1],[11432,46,1],[11432,57,1],
[11432,65,2],[11432,71,2],[11432,77,2],[11433,2,1],[11433,4,1],
[11433,5,4],[11433,6,1],[11433,9,1],[11433,10,3],[11433,16,1],
[11433,19,1],[11433,21,1],[11433,22,1],[11433,23,13],[11433,24,1],
[11433,25,2],[11433,27,2],[11433,28,3],[11433,29,1],[11433,31,2],
[11433,32,1],[11433,33,1],[11433,34,1],[11433,35,2],[11433,38,2],
[11433,40,6],[11433,41,1],[11433,44,2],[11433,47,3],[11433,50,1],
[11433,51,1],[11433,56,1],[11433,57,2],[11433,58,1],[11433,59,1],
[11433,62,1],[11433,63,139],[11433,65,1],[11433,68,4],[11433,69,3],
[11433,70,6],[11433,72,5],[11433,74,90],[11433,75,88],[11433,76,156],
[11434,30,2],[11434,38,1],[11435,3,3],[11435,42,1],[11435,56,1],
[11435,74,1],[11436,4,4],[11436,5,3],[11436,6,1],[11436,12,1],
[11436,15,5],[11436,17,4],[11436,23,1],[11436,28,9],[11436,31,2],
[11436,35,1],[11436,38,11],[11436,42,1],[11436,44,43],[11436,50,4],
[11436,51,1],[11436,53,2],[11436,55,23],[11436,66,47],[11436,76,2],
[11437,55,2],[11438,24,1],[11438,26,1],[11439,15,1],[11439,43,1],
[11440,20,2],[11440,23,3],[11440,32,2],[11440,41,1],[11440,52,1],
[11440,59,1],[11441,2,1],[11441,5,2],[11441,13,1],[11441,19,2],
[11441,26,2],[11441,27,1],[11441,59,1],[11442,35,17],[11443,2,2],
[11443,42,1],[11444,66,2],[11445,32,1],[11445,47,2],[11446,47,1],
[11446,70,1],[11447,38,1],[11447,50,1],[11448,28,1],[11448,33,1],
[11448,52,1],[11449,3,1],[11449,23,1],[11449,26,1],[11449,27,1],
[11449,33,1],[11449,35,1],[11449,43,1],[11450,3,1],[11450,5,1],
[11450,9,1],[11450,10,1],[11450,24,1],[11450,37,1],[11450,45,1],
[11450,77,1],[11451,2,1],[11451,14,1],[11451,28,2],[11451,31,2],
[11451,33,2],[11451,48,1],[11451,51,1],[11451,62,2],[11452,0,9],
[11452,8,2],[11452,9,1],[11452,10,1],[11452,16,1],[11452,34,3],
[11452,45,1],[11452,46,1],[11452,64,1],[11452,65,1],[11452,67,2],
[11453,4,1],[11453,50,1],[11453,55,1],[11454,3,1],[11454,22,2],
[11454,24,1],[11454,26,1],[11454,31,1],[11454,47,1],[11454,48,2],
[11454,50,1],[11455,20,1],[11455,27,1],[11456,1,1],[11456,5,1],
[11456,10,1],[11456,11,1],[11456,15,3],[11456,17,13],[11456,19,1],
[11456,20,1],[11456,46,1],[11456,53,20],[11456,54,1],[11456,55,7],
[11456,57,1],[11456,65,2],[11456,77,1],[11457,32,3],[11457,38,1],
[11457,43,2],[11457,59,1],[11457,62,4],[11458,22,2],[11458,27,1],
[11458,40,1],[11459,4,1],[11459,9,1],[11459,20,1],[11460,2,1],
[11460,3,1],[11460,5,1],[11460,13,2],[11460,21,1],[11460,22,1],
[11460,25,1],[11460,30,1],[11460,33,2],[11460,35,3],[11460,39,4],
[11460,43,1],[11460,49,1],[11460,51,2],[11460,52,1],[11460,63,1],
[11460,74,1],[11460,75,1],[11461,2,1],[11461,45,1],[11462,12,1],

[11462,17,1],[11462,53,2],[11463,1,3],[11463,2,2],[11463,4,2],
[11463,12,2],[11463,14,1],[11463,15,45],[11463,17,13],[11463,20,1],
[11463,22,22],[11463,23,249],[11463,24,1],[11463,28,3],[11463,29,1],
[11463,34,1],[11463,38,4],[11463,39,2],[11463,40,1],[11463,41,1],
[11463,44,1],[11463,46,1],[11463,50,2],[11463,53,9],[11463,54,1],
[11463,55,32],[11463,59,1],[11463,63,1],[11463,66,1],[11464,8,1],
[11464,11,1],[11464,16,1],[11464,57,1],[11464,65,1],[11465,27,3],
[11466,0,4],[11466,1,7],[11466,8,114],[11466,9,1],[11466,10,4],
[11466,11,1],[11466,12,2],[11466,13,1],[11466,14,1],[11466,15,2],
[11466,20,2],[11466,29,9],[11466,30,5],[11466,31,5],[11466,34,3],
[11466,36,1],[11466,37,2],[11466,39,1],[11466,53,2],[11466,59,1],
[11466,67,1],[11466,73,1],[11466,77,2],[11467,55,2],[11468,24,1],
[11468,28,2],[11469,1,1],[11469,2,3],[11469,5,1],[11469,7,1],
[11469,8,1],[11469,9,1],[11469,11,1],[11469,14,1],[11469,16,1],
[11469,19,1],[11469,22,1],[11469,23,2],[11469,24,1],[11469,25,1],
[11469,30,1],[11469,31,2],[11469,33,1],[11469,43,2],[11469,46,1],
[11469,49,1],[11469,53,1],[11469,68,1],[11469,71,1],[11469,78,2],
[11470,5,2],[11470,6,7],[11470,21,2],[11470,23,2],[11470,24,1],
[11470,26,1],[11470,27,4],[11470,28,1],[11470,30,4],[11470,35,2],
[11470,39,1],[11470,47,5],[11470,53,1],[11470,59,1],[11470,66,1],
[11470,68,1],[11471,5,1],[11471,21,1],[11471,32,1],[11471,33,5],
[11472,9,1],[11472,15,1],[11472,77,1],[11473,2,1],[11473,21,1],
[11474,27,1],[11474,32,3],[11475,26,1],[11475,28,1],[11475,47,1],
[11476,17,2],[11476,29,1],[11477,8,2],[11477,34,2],[11477,73,1],
[11478,2,1],[11478,5,1],[11478,68,1],[11479,24,1],[11479,26,1],
[11479,28,1],[11479,55,1],[11480,5,2],[11480,9,1],[11480,10,3],
[11480,13,5],[11480,24,1],[11480,25,3],[11480,26,2],[11480,27,2],
[11480,30,1],[11480,33,141],[11480,35,1],[11480,38,1],[11480,39,1],
[11480,43,1],[11480,44,2],[11480,47,1],[11480,48,1],[11480,55,1],
[11480,59,1],[11480,76,1],[11481,13,1],[11481,29,1],[11482,6,1],
[11482,70,2],[11483,20,2],[11484,24,1],[11484,28,2],[11485,3,1],
[11485,6,1],[11485,28,10],[11485,40,1],[11485,68,1],[11485,70,1],
[11486,29,2],[11487,2,3],[11487,23,1],[11487,27,2],[11487,30,1],
[11487,31,20],[11487,34,6],[11487,39,3],[11488,4,6],[11488,32,1],
[11488,35,1],[11488,38,1],[11488,50,7],[11489,17,1],[11489,53,1],
[11490,2,30],[11490,4,1],[11490,28,5],[11490,30,1],[11491,3,4],
[11491,24,9],[11491,28,5],[11491,35,1],[11491,70,1],[11492,2,1],
[11492,3,1],[11492,6,1],[11492,26,1],[11492,30,1],[11492,31,1],
[11493,12,1],[11493,28,2],[11493,55,1],[11494,0,1],[11494,10,1],
[11494,20,1],[11495,1,1],[11495,9,7],[11495,12,1],[11495,13,1],
[11495,28,11],[11495,53,2],[11495,58,1],[11495,60,1],[11495,70,1],
[11495,73,1],[11496,12,1],[11496,30,2],[11497,2,4],[11497,23,1],
[11497,27,1],[11497,30,1],[11497,31,1],[11497,43,1],[11497,60,1],
[11498,4,2],[11499,4,1],[11499,20,1],[11499,21,2],[11499,24,1],
[11500,43,5],[11501,0,77],[11501,1,3215],[11501,2,114],[11501,3,142],
[11501,4,58],[11501,5,497],[11501,6,53],[11501,7,73],[11501,8,72],
[11501,9,105],[11501,10,137],[11501,11,55],[11501,12,299],
[11501,13,1099],[11501,14,31],[11501,15,337],[11501,16,48],
[11501,17,66],[11501,18,60],[11501,19,85],[11501,20,1686],
[11501,21,145],[11501,22,81],[11501,23,395],[11501,24,578],

[11501,25,57],[11501,26,185],[11501,27,239],[11501,28,254],
[11501,29,219],[11501,30,117],[11501,31,181],[11501,32,363],
[11501,33,239],[11501,34,133],[11501,35,429],[11501,36,86],
[11501,37,61],[11501,38,321],[11501,39,136],[11501,40,25],
[11501,41,54],[11501,42,185],[11501,43,249],[11501,44,144],
[11501,45,48],[11501,46,43],[11501,47,91],[11501,48,17],[11501,49,26],
[11501,50,70],[11501,51,11],[11501,52,31],[11501,53,67],[11501,54,26],
[11501,55,210],[11501,56,83],[11501,57,58],[11501,58,33],
[11501,59,340],[11501,60,41],[11501,61,8],[11501,62,183],
[11501,63,14],[11501,64,23],[11501,65,69],[11501,66,115],
[11501,67,40],[11501,68,18],[11501,69,59],[11501,70,57],[11501,71,35],
[11501,72,14],[11501,73,39],[11501,74,56],[11501,75,317],
[11501,76,25],[11501,77,268],[11501,78,24],[11501,79,31],[11502,22,1],
[11502,24,1],[11502,28,1],[11502,33,2],[11502,43,1],[11502,47,1],
[11502,70,1],[11503,5,1],[11503,25,6],[11504,21,1],[11504,40,1],
[11504,56,1],[11504,66,2],[11505,26,1],[11505,28,1],[11505,31,1],
[11505,38,1],[11505,42,1],[11505,48,1],[11505,56,3],[11506,1,1],
[11506,15,1],[11506,16,1],[11506,18,1],[11506,34,1],[11506,41,1],
[11506,46,2],[11506,56,1],[11506,67,1],[11506,71,1],[11506,76,1],
[11507,2,4],[11508,4,1],[11508,23,1],[11508,28,12],[11508,44,10],
[11508,66,6],[11509,26,1],[11509,28,4],[11509,38,2],[11509,50,1],
[11509,56,1],[11509,69,1],[11510,1,1],[11510,15,1],[11511,4,1],
[11511,22,1],[11511,23,1],[11511,40,1],[11511,56,1],[11511,66,1],
[11512,3,1],[11512,22,2],[11512,23,3],[11512,24,4],[11512,27,1],
[11512,32,4],[11512,33,1],[11512,38,1],[11512,47,1],[11512,51,1],
[11512,56,2],[11513,15,2],[11514,22,1],[11514,32,1],[11514,35,2],
[11515,25,1],[11515,38,1],[11516,6,1],[11516,20,1],[11516,23,1],
[11516,27,1],[11516,31,1],[11516,33,1],[11516,48,1],[11517,2,1],
[11517,23,1],[11517,24,2],[11517,28,2],[11517,33,1],[11518,1,1],
[11518,2,3],[11519,1,9],[11519,36,1],[11520,62,2],[11521,3,1],
[11521,28,1],[11522,8,1],[11522,15,1],[11522,30,6],[11522,32,1],
[11522,65,1],[11523,3,1],[11523,26,1],[11523,28,1],[11523,47,1],
[11523,61,1],[11524,0,14],[11524,1,43],[11524,2,1],[11524,3,2],
[11524,4,3],[11524,6,1],[11524,7,13],[11524,8,14],[11524,9,70],
[11524,10,122],[11524,11,40],[11524,12,43],[11524,13,26],
[11524,14,37],[11524,15,35],[11524,16,65],[11524,17,22],[11524,18,30],
[11524,19,78],[11524,20,25],[11524,21,1],[11524,22,2],[11524,23,7],
[11524,24,4],[11524,25,2],[11524,26,2],[11524,28,2],[11524,29,9],
[11524,30,10],[11524,31,2],[11524,32,3],[11524,33,3],[11524,34,16],
[11524,35,2],[11524,36,24],[11524,37,36],[11524,38,2],[11524,39,17],
[11524,40,8],[11524,41,56],[11524,42,1],[11524,43,1],[11524,44,1],
[11524,45,60],[11524,46,46],[11524,47,1],[11524,48,1],[11524,49,23],
[11524,50,4],[11524,51,4],[11524,52,1],[11524,53,21],[11524,54,30],
[11524,55,18],[11524,56,3],[11524,57,51],[11524,58,38],[11524,59,33],
[11524,60,10],[11524,61,1],[11524,63,8],[11524,64,8],[11524,65,61],
[11524,66,4],[11524,67,26],[11524,68,4],[11524,70,6],[11524,71,44],
[11524,72,1],[11524,73,43],[11524,74,11],[11524,75,9],[11524,76,14],
[11524,77,39],[11524,78,15],[11524,79,64],[11525,25,4],[11525,28,2],
[11525,44,1],[11526,2,1],[11526,17,1],[11527,20,1],[11527,21,1],
[11527,42,1],[11528,5,2],[11528,8,1],[11528,13,2],[11528,15,3],

[11528,20,12],[11528,22,1],[11528,23,1],[11528,33,1],[11528,44,1],
[11528,51,1],[11528,55,7],[11528,56,1],[11528,66,1],[11528,71,1],
[11528,73,1],[11528,74,1],[11528,77,1],[11529,2,3],[11529,6,1],
[11529,24,1],[11530,1,2],[11530,3,1],[11530,5,1],[11530,6,1],
[11530,15,1],[11530,47,1],[11531,38,4],[11532,0,2],[11532,1,52],
[11532,2,1],[11532,3,19],[11532,4,1],[11532,5,6],[11532,7,1],
[11532,8,5],[11532,9,16],[11532,10,17],[11532,11,9],[11532,12,9],
[11532,13,52],[11532,14,10],[11532,15,20],[11532,16,7],[11532,17,2],
[11532,18,3],[11532,19,10],[11532,20,32],[11532,21,1],[11532,22,8],
[11532,23,74],[11532,24,15],[11532,25,5],[11532,26,22],[11532,27,66],
[11532,28,23],[11532,29,3],[11532,31,3],[11532,32,12],[11532,34,24],
[11532,35,2],[11532,36,5],[11532,37,7],[11532,38,1],[11532,39,1],
[11532,41,9],[11532,42,2],[11532,43,19],[11532,45,8],[11532,46,7],
[11532,47,13],[11532,48,1],[11532,49,9],[11532,50,2],[11532,53,8],
[11532,54,5],[11532,55,7],[11532,56,1],[11532,57,9],[11532,58,3],
[11532,59,19],[11532,60,2],[11532,64,2],[11532,65,18],[11532,66,1],
[11532,67,7],[11532,68,1],[11532,70,2],[11532,71,1],[11532,72,1],
[11532,73,8],[11532,74,2],[11532,77,9],[11532,78,2],[11533,28,4],
[11534,35,56],[11534,44,1],[11534,50,1],[11534,51,1],[11534,69,1],
[11535,3,1],[11535,22,1],[11535,23,1],[11536,9,1],[11536,11,1],
[11536,60,1],[11536,67,1],[11537,30,2],[11538,0,6],[11538,1,2],
[11538,7,1],[11538,8,2],[11538,9,1],[11538,12,3],[11538,14,1],
[11538,17,1],[11538,19,2],[11538,20,1],[11538,25,1],[11538,27,4],
[11538,28,6],[11538,29,9],[11538,30,107],[11538,32,2],[11538,33,14],
[11538,35,21],[11538,39,1],[11538,52,12],[11538,55,1],[11538,56,1],
[11538,58,1],[11538,62,2],[11538,65,1],[11538,71,2],[11539,0,1],
[11539,13,1],[11539,64,1],[11540,28,2],[11541,8,13],[11541,9,1],
[11541,10,1],[11541,30,44],[11541,45,4],[11541,46,1],[11541,67,3],
[11541,71,1],[11542,9,1],[11542,23,1],[11543,24,3],[11543,28,1],
[11543,42,4],[11543,44,1],[11543,66,1],[11544,0,1],[11544,1,1],
[11544,8,1],[11544,10,1],[11544,11,1],[11544,14,1],[11544,15,19],
[11544,17,6],[11544,19,1],[11544,21,1],[11544,34,1],[11544,37,2],
[11544,45,3],[11544,46,1],[11544,53,3],[11544,55,23],[11544,59,1],
[11544,67,1],[11545,2,1],[11545,32,1],[11546,23,4],[11546,25,9],
[11546,28,1],[11546,32,36],[11546,35,2],[11546,42,1],[11546,43,2],
[11546,44,2],[11546,50,1],[11546,51,1],[11546,66,1],[11546,68,1],
[11546,69,2],[11546,70,1],[11547,2,5],[11547,22,1],[11547,24,1],
[11547,52,1],[11548,76,2],[11549,3,1],[11549,23,1],[11549,24,1],
[11549,28,3],[11549,43,3],[11549,56,1],[11550,17,1],[11550,53,1],
[11551,2,1],[11551,4,1],[11551,24,1],[11551,27,1],[11552,6,1],
[11552,22,2],[11552,24,1],[11552,26,1],[11552,28,1],[11552,33,1],
[11552,42,5],[11552,47,4],[11552,48,6],[11552,64,1],[11552,75,1],
[11553,24,1],[11553,40,1],[11554,50,1],[11554,66,1],[11555,29,1],
[11555,77,1],[11556,17,1],[11556,53,1],[11556,73,1],[11557,27,1],
[11557,28,2],[11557,35,1],[11558,10,1],[11558,20,1],[11559,6,1],
[11559,35,1],[11560,7,1],[11560,10,4],[11560,12,1],[11560,15,1],
[11560,19,1],[11560,33,1],[11560,41,1],[11560,46,2],[11560,49,2],
[11560,53,1],[11560,58,1],[11560,60,1],[11560,73,2],[11560,77,2],
[11561,28,1],[11561,32,1],[11562,20,1],[11562,31,1],[11563,11,1],
[11563,78,1],[11564,2,1],[11564,27,1],[11565,27,1],[11565,42,1],

[11565,51,1],[11565,62,1],[11565,63,2],[11565,69,1],[11565,72,1],
[11565,74,10],[11565,75,1],[11565,76,13],[11566,2,2],[11566,3,1],
[11566,7,3],[11566,21,1],[11566,22,3],[11566,23,1],[11566,24,2],
[11566,30,1],[11566,32,3],[11566,42,1],[11566,45,1],[11566,55,1],
[11566,56,1],[11566,59,1],[11566,60,2],[11566,68,2],[11566,76,1],
[11567,5,1],[11567,17,7],[11567,18,1],[11567,22,1],[11567,28,2],
[11567,45,1],[11567,49,1],[11567,53,9],[11567,55,1],[11567,74,1],
[11567,75,2],[11567,76,1],[11568,3,1],[11568,10,1],[11568,23,2],
[11568,30,2],[11568,47,2],[11568,48,1],[11568,55,1],[11568,69,1],
[11569,2,1],[11569,40,1],[11570,24,1],[11570,76,1],[11571,2,1],
[11571,3,1],[11571,26,2],[11571,39,1],[11572,24,4],[11572,27,1],
[11572,32,2],[11572,33,1],[11572,74,1],[11573,4,1],[11573,9,4],
[11573,28,3],[11573,31,1],[11573,44,2],[11573,50,2],[11573,66,2],
[11573,75,1],[11574,23,3],[11574,24,1],[11574,33,2],[11574,74,6],
[11575,4,13],[11575,5,1],[11575,17,1],[11575,23,2],[11575,24,1],
[11575,25,1],[11575,27,2],[11575,35,1],[11575,50,8],[11575,56,1],
[11575,67,1],[11576,2,17],[11576,23,2],[11576,32,1],[11576,38,1],
[11576,56,1],[11577,20,1],[11577,43,1],[11578,33,2],[11579,27,2],
[11579,50,1],[11579,74,3],[11580,27,1],[11580,28,2],[11580,35,6],
[11580,40,1],[11580,52,32],[11581,10,1],[11581,17,1],[11582,20,4],
[11582,23,2],[11582,39,2],[11583,17,1],[11583,29,1],[11583,53,1],
[11584,2,1],[11584,28,1],[11585,24,1],[11585,56,1],[11586,21,7],
[11586,31,1],[11587,24,1],[11587,66,1],[11588,5,1],[11588,24,1],
[11588,42,1],[11589,1,1],[11589,6,1],[11589,8,1],[11589,23,4],
[11589,25,1],[11590,24,2],[11590,28,1],[11591,28,1],[11591,63,2],
[11592,3,1],[11592,5,1],[11592,47,1],[11592,54,1],[11592,62,3],
[11593,15,1],[11593,28,2],[11593,32,1],[11593,43,1],[11593,76,1],
[11594,27,1],[11594,31,1],[11595,5,3],[11595,62,6],[11595,70,1],
[11596,7,1],[11596,62,1],[11597,27,2],[11597,35,9],[11598,15,1],
[11598,53,1],[11599,32,3],[11600,28,11],[11601,27,2],[11601,28,1],
[11601,35,2],[11601,43,1],[11601,68,1],[11602,56,2],[11603,15,1],
[11603,53,1],[11604,62,5],[11605,27,2],[11606,28,1],[11606,31,1],
[11606,44,1],[11607,0,1],[11607,3,2],[11607,6,2],[11607,12,1],
[11607,13,2],[11607,15,1],[11607,16,1],[11607,17,1],[11607,20,9],
[11607,22,1],[11607,23,3],[11607,27,1],[11607,30,1],[11607,31,1],
[11607,32,1],[11607,33,1],[11607,35,2],[11607,38,2],[11607,39,1],
[11607,41,1],[11607,45,1],[11607,47,1],[11607,50,1],[11607,53,1],
[11607,55,1],[11607,56,3],[11607,57,1],[11607,58,1],[11607,72,1],
[11607,76,1],[11608,3,1],[11608,32,1],[11609,25,8],[11609,28,1],
[11610,74,8],[11611,1,1],[11611,30,3],[11611,47,1],[11611,48,1],
[11611,65,1],[11612,7,1],[11612,17,1],[11613,2,1],[11613,13,3],
[11613,21,1],[11613,48,1],[11614,33,20],[11615,17,1],[11615,36,1],
[11615,53,1],[11616,17,6],[11616,53,8],[11617,51,3],[11617,75,1],
[11618,23,1],[11618,27,2],[11618,32,1],[11618,42,1],[11619,5,2],
[11619,21,2],[11619,22,1],[11619,25,7],[11619,26,3],[11619,27,3],
[11619,31,1],[11619,32,2],[11619,33,2],[11620,1,3],[11620,4,1],
[11620,8,1],[11620,13,2],[11620,15,2],[11620,18,1],[11620,22,8],
[11620,24,2],[11620,25,4],[11620,27,6],[11620,28,14],[11620,29,1],
[11620,30,141],[11620,32,9],[11620,33,51],[11620,34,2],[11620,35,22],
[11620,38,4],[11620,39,2],[11620,41,1],[11620,45,1],[11620,49,1],

[11620,51,1],[11620,52,16],[11620,53,1],[11620,55,1],[11620,57,1],
[11620,60,1],[11620,61,1],[11620,62,1],[11620,65,2],[11620,67,1],
[11620,70,2],[11620,71,1],[11621,23,2],[11621,26,1],[11621,28,1],
[11621,40,1],[11621,43,1],[11621,70,1],[11622,2,30],[11622,23,2],
[11622,24,1],[11622,25,1],[11622,31,1],[11622,47,1],[11623,3,1],
[11623,23,2],[11623,33,5],[11623,35,2],[11624,2,2],[11624,5,1],
[11624,22,2],[11624,23,1],[11624,24,1],[11624,26,1],[11624,33,1],
[11624,34,2],[11624,35,1],[11624,43,7],[11624,59,1],[11624,74,1],
[11625,2,6],[11625,9,1],[11625,12,1],[11625,30,1],[11625,39,1],
[11626,14,1],[11626,28,1],[11627,21,3],[11627,31,1],[11628,3,2],
[11628,8,2],[11628,13,1],[11628,20,1],[11628,23,2],[11628,24,1],
[11628,26,2],[11628,27,1],[11628,28,2],[11628,31,2],[11628,35,1],
[11628,37,1],[11628,38,1],[11628,41,1],[11628,47,1],[11628,55,1],
[11628,56,2],[11628,58,1],[11628,59,1],[11628,65,1],[11629,9,1],
[11629,28,2],[11630,19,1],[11630,34,1],[11631,28,1],[11631,70,1],
[11632,23,1],[11632,24,1],[11632,39,1],[11632,62,1],[11633,56,1],
[11633,62,1],[11633,70,1],[11633,79,1],[11634,51,1],[11634,60,1],
[11635,2,3],[11636,3,1],[11636,17,1],[11636,19,1],[11636,22,1],
[11636,23,1],[11636,26,1],[11636,27,3],[11636,28,2],[11636,32,3],
[11636,35,2],[11636,74,1],[11636,75,1],[11637,22,1],[11637,28,1],
[11638,21,1],[11638,24,1],[11638,25,1],[11638,26,1],[11638,32,1],
[11638,52,1],[11639,2,2],[11640,44,1],[11640,45,1],[11641,3,1],
[11641,23,1],[11641,43,1],[11642,0,26],[11642,7,17],[11642,8,1],
[11642,10,2],[11642,11,1],[11642,12,2],[11642,13,3],[11642,14,1],
[11642,16,1],[11642,17,1],[11642,18,2],[11642,30,1],[11642,31,2],
[11642,32,1],[11642,36,2],[11642,41,1],[11642,45,2],[11642,46,1],
[11642,54,1],[11642,62,1],[11642,64,1],[11642,65,3],[11642,73,4],
[11642,77,1],[11642,78,1],[11643,22,2],[11643,27,2],[11643,35,1],
[11643,38,1],[11643,76,1],[11644,13,1],[11644,17,2],[11644,30,69],
[11644,33,9],[11644,34,2],[11644,53,6],[11644,55,2],[11644,59,2],
[11645,25,1],[11645,32,1],[11646,27,1],[11646,56,2],[11647,3,2],
[11647,6,1],[11647,23,1],[11647,24,2],[11647,27,1],[11647,28,1],
[11647,33,1],[11647,35,1],[11647,42,1],[11648,4,1],[11648,66,1],
[11649,23,1],[11649,24,1],[11649,31,3],[11650,23,1],[11650,26,1],
[11651,28,8],[11651,40,1],[11651,44,1],[11652,51,20],[11653,33,2],
[11654,3,4],[11655,1,2],[11656,6,1],[11656,27,5],[11657,2,2],
[11657,13,1],[11658,1,1],[11658,6,1],[11658,12,2],[11658,17,1],
[11658,40,1],[11658,47,1],[11658,51,8],[11658,70,4],[11658,72,4],
[11659,24,1],[11659,56,1],[11660,9,1],[11660,19,1],[11661,28,14],
[11662,32,1],[11662,41,1],[11662,50,2],[11662,53,1],[11662,58,1],
[11663,26,1],[11663,28,1],[11663,31,36],[11664,20,1],[11664,21,1],
[11665,2,2],[11665,21,1],[11665,23,4],[11665,26,1],[11665,28,1],
[11665,32,2],[11665,40,1],[11666,15,2],[11666,17,1],[11667,5,6],
[11667,8,1],[11667,21,3],[11667,27,2],[11667,39,1],[11667,52,1],
[11668,2,1],[11668,3,1],[11669,22,1],[11669,27,1],[11669,28,1],
[11669,62,2],[11670,2,6],[11670,3,1],[11670,5,1],[11670,8,5],
[11670,23,1],[11670,24,2],[11670,27,1],[11670,28,3],[11670,30,2],
[11670,31,1],[11670,43,2],[11670,56,3],[11671,23,2],[11672,28,17],
[11672,42,1],[11673,0,1],[11673,1,12],[11673,2,4],[11673,3,3],
[11673,9,3],[11673,10,4],[11673,12,4],[11673,13,13],[11673,14,1],

[11673,15,3],[11673,17,1],[11673,19,3],[11673,20,1],[11673,22,3],
[11673,25,3],[11673,27,1],[11673,28,2],[11673,29,1],[11673,30,3],
[11673,31,4],[11673,33,4],[11673,34,1],[11673,35,2],[11673,36,3],
[11673,37,2],[11673,39,1],[11673,41,3],[11673,42,2],[11673,43,2],
[11673,44,1],[11673,45,2],[11673,47,1],[11673,48,2],[11673,54,1],
[11673,55,3],[11673,59,3],[11673,60,2],[11673,65,3],[11673,67,1],
[11673,68,1],[11673,73,3],[11673,75,1],[11673,77,2],[11674,24,1],
[11674,27,3],[11674,34,1],[11674,39,1],[11674,42,1],[11674,50,1],
[11675,23,1],[11675,24,2],[11675,43,1],[11675,57,1],[11676,22,2],
[11676,24,1],[11676,26,1],[11676,27,8],[11676,28,1],[11676,32,1],
[11676,35,2],[11676,43,83],[11676,50,2],[11676,61,1],[11676,63,1],
[11676,66,3],[11676,70,1],[11676,74,1],[11677,1,3],[11677,71,1],
[11678,44,1],[11678,74,4],[11679,17,1],[11679,65,1],[11680,22,2],
[11680,72,1],[11681,10,1],[11681,34,1],[11682,3,1],[11682,25,1],
[11682,27,1],[11682,28,3],[11682,44,1],[11682,66,1],[11683,38,1],
[11683,51,1],[11683,74,1],[11684,5,1],[11684,44,1],[11684,66,5],
[11685,42,1],[11685,44,1],[11686,27,30],[11687,22,1],[11687,23,1],
[11687,28,2],[11687,56,1],[11688,2,1],[11688,5,3],[11688,8,1],
[11688,9,1],[11688,10,1],[11688,17,1],[11688,18,1],[11688,19,3],
[11688,21,2],[11688,22,8],[11688,23,1],[11688,24,3],[11688,25,15],
[11688,27,14],[11688,28,21],[11688,30,38],[11688,31,1],[11688,32,55],
[11688,33,107],[11688,34,1],[11688,35,35],[11688,38,1],[11688,40,6],
[11688,42,1],[11688,44,1],[11688,47,1],[11688,52,52],[11688,55,1],
[11688,56,1],[11688,57,1],[11688,58,1],[11688,59,1],[11688,60,1],
[11688,63,1],[11688,66,1],[11688,68,1],[11688,69,1],[11688,70,3],
[11688,72,1],[11688,74,1],[11688,76,1],[11689,28,1],[11689,69,1],
[11690,11,1],[11690,49,1],[11691,32,3],[11692,1,2],[11693,22,1],
[11693,26,1],[11693,31,1],[11693,32,1],[11693,50,1],[11694,58,1],
[11694,75,1],[11695,7,7],[11696,2,1],[11696,21,1],[11696,26,1],
[11697,22,3],[11697,24,1],[11697,27,1],[11698,4,1],[11698,22,1],
[11698,70,1],[11698,74,1],[11699,0,1],[11699,1,6],[11699,4,1],
[11699,5,1],[11699,8,1],[11699,9,1],[11699,12,11],[11699,15,3],
[11699,17,1],[11699,18,1],[11699,20,2],[11699,22,1],[11699,28,3],
[11699,29,14],[11699,30,3],[11699,38,1],[11699,39,1],[11699,40,1],
[11699,41,1],[11699,50,2],[11699,53,3],[11699,55,2],[11699,56,1],
[11699,70,1],[11699,77,1],[11700,14,1],[11700,61,1],[11701,6,1],
[11701,27,1],[11701,35,1],[11701,62,8],[11702,12,1],[11702,13,1],
[11702,37,1],[11702,55,1],[11702,57,1],[11702,66,1],[11703,4,2],
[11703,5,1],[11703,6,1],[11703,23,1],[11703,24,1],[11703,28,20],
[11703,33,1],[11703,40,1],[11703,50,1],[11703,62,1],[11703,68,1],
[11703,69,1],[11703,74,1],[11703,76,1],[11704,0,1],[11704,7,1],
[11704,16,1],[11704,53,1],[11705,3,1],[11705,6,1],[11705,24,1],
[11705,27,1],[11706,12,1],[11706,14,1],[11707,2,3],[11708,5,1],
[11708,38,1],[11709,5,4],[11709,25,13],[11709,32,15],[11709,40,3],
[11709,56,1],[11709,61,1],[11709,68,3],[11709,70,1],[11709,72,1],
[11710,26,4],[11710,27,5],[11710,42,1],[11711,15,3],[11711,55,2],
[11711,61,2],[11711,68,1],[11711,72,1],[11712,15,1],[11712,16,1],
[11712,28,1],[11713,62,6],[11714,62,2],[11715,2,2],[11716,24,1],
[11716,32,12],[11716,63,1],[11716,70,1],[11716,75,1],[11717,24,2],
[11718,20,3],[11718,26,1],[11719,24,1],[11719,74,5],[11720,51,1],

[11720,69,1],[11721,10,2],[11721,20,1],[11721,39,2],[11721,59,2],
[11722,21,1],[11722,23,2],[11722,26,1],[11722,27,1],[11722,31,2],
[11722,68,1],[11722,75,1],[11723,0,2],[11723,1,7],[11723,8,1],
[11723,9,3],[11723,10,3],[11723,11,1],[11723,13,1],[11723,14,1],
[11723,15,6],[11723,16,2],[11723,17,1],[11723,18,1],[11723,19,4],
[11723,20,3],[11723,29,1],[11723,30,2],[11723,32,1],[11723,34,1],
[11723,37,3],[11723,39,1],[11723,41,1],[11723,45,2],[11723,49,1],
[11723,53,2],[11723,55,4],[11723,58,1],[11723,59,2],[11723,60,1],
[11723,64,1],[11723,65,4],[11723,67,2],[11723,73,1],[11723,77,2],
[11724,3,1],[11724,56,1],[11725,2,1],[11725,3,1],[11725,12,2],
[11725,16,1],[11725,27,1],[11725,30,1],[11725,45,3],[11726,4,1],
[11726,5,1],[11726,25,7],[11726,27,2],[11727,35,1],[11727,68,1],
[11727,78,1],[11728,28,2],[11728,43,1],[11729,23,1],[11729,27,5],
[11729,28,1],[11730,1,10],[11730,2,1],[11730,3,2],[11730,4,4],
[11730,5,6],[11730,9,2],[11730,10,2],[11730,12,1],[11730,13,17],
[11730,14,1],[11730,15,1],[11730,16,3],[11730,19,3],[11730,20,9],
[11730,21,1],[11730,22,4],[11730,26,1],[11730,27,1],[11730,28,3],
[11730,29,2],[11730,30,3],[11730,31,1],[11730,32,2],[11730,33,1],
[11730,34,1],[11730,35,4],[11730,38,1],[11730,39,9],[11730,40,1],
[11730,41,2],[11730,42,2],[11730,43,1],[11730,44,2],[11730,45,1],
[11730,46,3],[11730,47,3],[11730,50,1],[11730,55,1],[11730,56,7],
[11730,58,1],[11730,62,2],[11730,64,2],[11730,65,3],[11730,67,1],
[11730,70,1],[11730,72,1],[11730,74,1],[11730,75,1],[11730,78,1],
[11731,20,1],[11731,22,1],[11731,24,1],[11731,28,2],[11731,29,1],
[11731,36,1],[11731,48,1],[11731,59,1],[11732,1,5],[11732,2,9],
[11732,10,1],[11732,34,2],[11733,43,1],[11733,68,1],[11734,12,2],
[11735,15,1],[11735,55,1],[11736,22,1],[11736,23,1],[11736,27,2],
[11736,28,1],[11736,33,1],[11737,5,1],[11737,15,3],[11737,17,3],
[11737,23,1],[11737,25,2],[11737,28,1],[11737,34,1],[11737,35,1],
[11737,41,1],[11737,50,4],[11737,53,6],[11737,55,4],[11737,74,1],
[11737,76,1],[11737,77,1],[11738,17,4],[11738,53,1],[11738,56,1],
[11739,20,1],[11739,54,1],[11740,25,4],[11741,1,7],[11741,2,1],
[11741,3,1],[11741,10,1],[11741,12,1],[11741,13,3],[11741,18,2],
[11741,20,5],[11741,22,1],[11741,23,2],[11741,24,1],[11741,25,1],
[11741,28,9],[11741,29,1],[11741,33,1],[11741,37,1],[11741,46,2],
[11741,47,1],[11741,48,1],[11741,55,1],[11741,59,1],[11741,64,1],
[11741,65,1],[11741,67,1],[11741,72,1],[11741,73,3],[11741,77,1],
[11742,28,2],[11743,0,1],[11743,1,1],[11743,9,1],[11743,10,2],
[11743,12,3],[11743,15,13],[11743,16,2],[11743,17,3],[11743,19,3],
[11743,23,1],[11743,28,1],[11743,37,1],[11743,45,1],[11743,49,1],
[11743,53,3],[11743,55,6],[11743,57,3],[11743,58,2],[11743,59,3],
[11743,60,1],[11743,64,1],[11743,65,1],[11743,67,3],[11743,71,1],
[11743,77,2],[11744,8,1],[11744,13,1],[11744,30,1],[11745,24,1],
[11745,27,2],[11745,56,2],[11745,70,1],[11746,10,1],[11746,15,1],
[11747,23,2],[11747,31,1],[11748,4,1],[11748,5,1],[11748,24,1],
[11748,26,1],[11748,28,1],[11748,31,1],[11748,44,1],[11748,47,1],
[11748,51,2],[11748,56,7],[11748,61,1],[11748,68,1],[11748,69,4],
[11749,1,1],[11749,11,1],[11749,18,1],[11749,28,1],[11749,31,1],
[11749,37,1],[11750,4,1],[11750,21,4],[11750,25,1],[11750,26,2],
[11750,27,2],[11750,28,2],[11750,42,1],[11750,43,1],[11750,47,1],

[11750,52,1],[11750,63,1],[11750,66,1],[11751,30,5],[11752,6,1],
[11752,28,2],[11753,4,1],[11753,28,19],[11753,40,1],[11753,42,1],
[11753,47,1],[11753,50,1],[11753,68,1],[11753,70,2],[11753,72,1],
[11753,74,1],[11753,76,2],[11753,79,1],[11754,23,1],[11754,24,11],
[11754,27,2],[11754,28,1],[11754,32,3],[11754,33,1],[11754,74,1],
[11754,76,1],[11755,31,3],[11756,20,1],[11756,31,3],[11756,33,9],
[11756,45,1],[11757,43,2],[11758,1,1],[11758,2,1],[11758,28,2],
[11759,3,2],[11759,23,5],[11759,24,4],[11759,56,3],[11760,1,1],
[11760,73,1],[11760,77,2],[11761,2,1],[11761,28,1],[11761,32,1],
[11762,2,4],[11762,27,1],[11763,32,20],[11764,33,2],[11764,68,1],
[11765,28,1],[11765,51,1],[11765,62,1],[11766,28,68],[11767,21,6],
[11767,25,7],[11767,31,1],[11767,32,2],[11767,33,4],[11767,42,8],
[11767,43,5],[11767,48,1],[11767,62,3],[11768,23,2],[11768,26,2],
[11768,28,1],[11769,1,1],[11769,3,2],[11769,5,6],[11769,6,2],
[11769,8,3],[11769,19,1],[11769,21,1],[11769,22,12],[11769,23,12],
[11769,24,9],[11769,25,17],[11769,26,20],[11769,27,32],[11769,28,6],
[11769,30,11],[11769,31,22],[11769,32,21],[11769,33,5],[11769,35,7],
[11769,39,1],[11769,42,3],[11769,43,17],[11769,47,2],[11769,50,1],
[11769,61,1],[11769,62,2],[11769,65,1],[11769,74,1],[11770,22,2],
[11770,26,8],[11770,43,4],[11771,26,3],[11771,43,3],[11772,13,2],
[11773,56,1],[11773,78,1],[11774,15,1],[11774,28,1],[11774,47,1],
[11775,9,1],[11775,39,1],[11776,12,2],[11777,1,1],[11777,7,1],
[11777,13,1],[11777,15,1],[11777,39,1],[11777,41,1],[11777,53,1],
[11777,65,1],[11778,12,4],[11778,19,1],[11778,29,3],[11779,8,1],
[11779,17,1],[11779,26,1],[11779,29,1],[11779,33,1],[11780,4,1],
[11780,22,1],[11781,5,1],[11781,23,2],[11781,28,1],[11782,32,1],
[11782,42,1],[11783,3,1],[11783,5,4],[11783,6,3],[11783,22,2],
[11783,23,1],[11783,24,20],[11783,27,3],[11783,28,9],[11783,32,2],
[11783,34,1],[11783,35,24],[11783,47,3],[11783,51,3],[11783,56,43],
[11783,62,2],[11783,65,1],[11783,66,1],[11783,70,1],[11783,75,1],
[11784,24,1],[11784,28,1],[11785,20,1],[11785,22,1],[11785,28,1],
[11785,35,1],[11785,42,4],[11785,45,3],[11786,2,9],[11786,3,2],
[11786,13,1],[11786,23,4],[11786,24,3],[11786,25,2],[11786,26,3],
[11786,28,1],[11786,33,2],[11786,42,3],[11786,47,2],[11786,48,3],
[11786,56,1],[11786,63,1],[11787,12,2],[11787,13,1],[11787,14,1],
[11787,27,1],[11787,29,2],[11787,36,1],[11787,37,1],[11787,39,2],
[11788,2,2],[11788,47,1],[11789,32,2],[11790,22,1],[11790,27,1],
[11791,3,2],[11792,1,1],[11792,13,2],[11792,15,3],[11792,20,1],
[11792,38,1],[11792,55,1],[11792,59,1],[11792,70,1],[11793,0,1],
[11793,20,1],[11793,23,1],[11793,24,2],[11793,25,5],[11793,28,4],
[11793,31,1],[11793,39,1],[11794,23,1],[11794,24,3],[11794,28,2],
[11794,35,1],[11794,38,1],[11794,46,1],[11794,56,1],[11795,10,1],
[11795,14,1],[11795,16,2],[11795,19,1],[11795,20,1],[11795,22,4],
[11795,27,6],[11795,30,51],[11795,32,2],[11795,33,19],[11795,35,12],
[11795,37,6],[11795,52,2],[11795,57,1],[11795,58,1],[11795,65,1],
[11795,67,1],[11795,70,1],[11795,77,1],[11796,1,1],[11796,29,1],
[11797,4,1],[11797,21,2],[11797,26,1],[11797,28,6],[11797,52,1],
[11797,66,1],[11798,30,1],[11798,45,1],[11799,2,1],[11799,6,1],
[11799,24,1],[11799,63,13],[11799,74,8],[11799,75,4],[11799,76,12],
[11800,2,5],[11800,5,5],[11800,6,1],[11800,13,1],[11800,21,2],

[11800,22,1],[11800,23,15],[11800,24,8],[11800,25,3],[11800,26,24],
[11800,27,2],[11800,28,8],[11800,30,1],[11800,31,4],[11800,32,1],
[11800,33,9],[11800,34,2],[11800,35,3],[11800,37,2],[11800,38,1],
[11800,40,1],[11800,42,6],[11800,43,1],[11800,47,1],[11800,48,6],
[11800,56,1],[11800,62,16],[11800,63,1],[11800,69,1],[11800,70,3],
[11800,72,1],[11800,75,1],[11800,76,1],[11800,78,2],[11801,28,2],
[11801,32,2],[11802,35,2],[11803,4,1],[11803,20,1],[11803,50,2],
[11803,75,1],[11803,76,1],[11804,25,3],[11804,27,1],[11804,32,1],
[11805,47,1],[11805,56,1],[11806,13,3],[11807,1,1],[11807,2,6],
[11807,3,2],[11807,8,2],[11807,10,1],[11807,14,1],[11807,20,7],
[11807,23,6],[11807,25,6],[11807,27,34],[11807,28,7],[11807,32,3],
[11807,33,1],[11807,35,14],[11807,40,1],[11807,42,2],[11807,47,1],
[11807,55,1],[11807,69,1],[11807,70,1],[11807,72,1],[11807,74,1],
[11808,1,6],[11808,9,1],[11808,15,1],[11808,20,1],[11808,24,2],
[11808,28,1],[11809,2,1],[11809,32,1],[11809,37,1],[11810,4,3],
[11810,38,2],[11810,40,1],[11810,44,11],[11810,56,1],[11810,66,4],
[11810,75,1],[11811,8,2],[11812,5,2],[11812,10,1],[11812,20,1],
[11812,21,4],[11812,23,13],[11812,28,1],[11812,32,1],[11812,33,3],
[11812,42,2],[11812,46,1],[11812,50,2],[11812,62,1],[11812,72,2],
[11812,76,1],[11813,35,3],[11814,2,1],[11814,17,1],[11814,22,7],
[11814,27,1],[11814,28,2],[11814,33,1],[11814,43,1],[11814,50,1],
[11814,55,1],[11814,66,1],[11815,1,3],[11815,2,4],[11815,3,2],
[11815,4,1],[11815,8,2],[11815,15,2],[11815,17,1],[11815,20,11],
[11815,21,11],[11815,22,1],[11815,23,4],[11815,24,3],[11815,26,43],
[11815,29,2],[11815,31,9],[11815,33,2],[11815,34,1],[11815,35,5],
[11815,36,1],[11815,39,53],[11815,42,2],[11815,43,12],[11815,47,5],
[11815,48,18],[11815,53,1],[11815,55,1],[11815,59,1],[11815,70,1],
[11816,4,1],[11816,28,1],[11816,50,1],[11817,25,6],[11818,11,1],
[11818,77,1],[11819,2,19],[11819,3,8],[11819,5,1],[11819,9,1],
[11819,17,1],[11819,19,1],[11819,21,2],[11819,22,7],[11819,23,9],
[11819,24,4],[11819,25,2],[11819,26,4],[11819,27,5],[11819,28,14],
[11819,30,4],[11819,31,14],[11819,32,11],[11819,33,34],[11819,35,44],
[11819,38,6],[11819,40,2],[11819,42,8],[11819,43,6],[11819,50,1],
[11819,52,50],[11819,63,1],[11819,66,3],[11819,68,1],[11819,69,2],
[11819,72,4],[11819,74,1],[11819,75,3],[11819,76,1],[11820,2,1],
[11820,24,1],[11821,2,2],[11821,3,2],[11821,5,1],[11821,8,1],
[11821,22,1],[11821,23,5],[11821,24,6],[11821,26,2],[11821,28,4],
[11821,31,2],[11821,32,10],[11821,38,1],[11821,43,3],[11821,44,1],
[11821,50,1],[11821,56,3],[11821,76,1],[11822,13,2],[11822,16,1],
[11822,20,5],[11822,29,1],[11822,50,1],[11822,59,1],[11823,20,1],
[11823,21,1],[11823,23,12],[11823,24,3],[11823,25,14],[11823,35,6],
[11823,50,1],[11824,2,1],[11824,3,1],[11824,21,1],[11824,37,1],
[11824,42,6],[11824,45,1],[11824,47,8],[11825,21,1],[11825,22,4],
[11825,23,1],[11825,27,1],[11825,35,4],[11825,47,1],[11826,27,5],
[11827,22,1],[11827,66,1],[11828,20,1],[11828,55,2],[11829,14,1],
[11829,23,3],[11829,25,1],[11829,26,1],[11829,27,2],[11829,28,1],
[11829,31,1],[11830,38,1],[11830,66,1],[11831,10,1],[11831,11,1],
[11831,17,6],[11831,28,1],[11831,31,1],[11831,53,3],[11831,63,1],
[11831,67,1],[11832,9,1],[11832,13,1],[11832,29,2],[11832,30,1],
[11832,31,1],[11833,2,8],[11833,3,3],[11833,8,2],[11833,10,1],

[11833,12,1],[11833,25,1],[11833,28,2],[11833,30,4],[11833,31,17],
[11833,38,1],[11833,42,1],[11833,43,2],[11833,60,2],[11834,44,1],
[11834,66,1],[11835,26,1],[11835,31,1],[11836,8,1],[11836,10,2],
[11836,11,1],[11836,12,2],[11836,15,1],[11836,45,1],[11836,53,1],
[11836,55,1],[11836,58,1],[11836,71,1],[11837,1,2],[11837,2,1],
[11837,3,1],[11837,4,26],[11837,5,3],[11837,15,35],[11837,17,11],
[11837,20,1],[11837,22,4],[11837,23,2],[11837,25,3],[11837,26,1],
[11837,28,10],[11837,30,1],[11837,31,5],[11837,32,1],[11837,35,1],
[11837,37,1],[11837,38,3],[11837,40,2],[11837,42,1],[11837,43,1],
[11837,44,41],[11837,47,1],[11837,48,1],[11837,50,30],[11837,51,1],
[11837,53,10],[11837,55,24],[11837,59,1],[11837,66,57],[11837,70,1],
[11837,72,1],[11837,74,1],[11837,76,3],[11838,21,1],[11838,24,1],
[11838,27,1],[11838,42,1],[11839,28,1],[11839,32,1],[11840,72,2],
[11841,15,3],[11841,28,2],[11841,39,1],[11841,44,2],[11841,50,2],
[11841,53,1],[11841,55,1],[11841,66,5],[11842,5,2],[11842,24,1],
[11842,26,9],[11842,33,1],[11842,39,1],[11842,43,4],[11842,62,1],
[11842,69,1],[11842,70,1],[11843,24,2],[11843,42,1],[11844,20,1],
[11844,26,1],[11844,27,3],[11844,31,1],[11844,32,1],[11844,33,1],
[11844,37,1],[11844,50,1],[11844,56,2],[11845,1,2],[11846,0,2],
[11847,18,1],[11847,29,1],[11848,21,1],[11848,24,1],[11848,32,3],
[11848,47,1],[11849,25,1],[11849,32,1],[11850,1,2],[11850,12,2],
[11850,13,3],[11850,20,1],[11850,28,2],[11850,30,1],[11850,45,1],
[11850,52,1],[11850,59,1],[11850,62,1],[11850,65,1],[11851,23,1],
[11851,24,2],[11851,27,5],[11851,28,2],[11851,31,1],[11851,32,1],
[11851,33,2],[11851,35,1],[11851,38,1],[11851,52,1],[11852,17,1],
[11852,41,1],[11853,30,2],[11853,46,1],[11854,10,1],[11854,15,2],
[11854,17,1],[11854,55,6],[11855,32,8],[11856,5,1],[11856,21,1],
[11856,38,2],[11856,52,1],[11857,62,2],[11858,1,3],[11858,12,1],
[11858,18,1],[11858,25,4],[11858,39,1],[11858,77,1],[11859,53,1],
[11859,55,1],[11860,5,1],[11860,12,1],[11860,22,1],[11860,33,1],
[11860,43,1],[11860,57,1],[11860,66,1],[11861,24,2],[11861,28,1],
[11861,35,1],[11861,56,1],[11862,2,1],[11862,28,1],[11863,15,1],
[11863,53,1],[11864,26,1],[11864,27,1],[11864,28,3],[11864,44,3],
[11864,48,1],[11864,61,6],[11864,66,1],[11864,68,1],[11865,44,2],
[11865,66,1],[11866,24,1],[11866,28,1],[11866,31,1],[11867,15,1],
[11867,24,1],[11867,27,1],[11867,28,5],[11867,42,1],[11868,3,1],
[11868,20,1],[11869,9,1],[11869,20,1],[11870,28,1],[11870,31,1],
[11871,31,2],[11872,0,13],[11872,1,16],[11872,2,1],[11872,3,52],
[11872,4,1],[11872,5,1],[11872,6,1],[11872,7,4],[11872,8,14],
[11872,9,15],[11872,10,10],[11872,12,22],[11872,13,4],[11872,14,2],
[11872,15,2],[11872,16,3],[11872,17,3],[11872,18,1],[11872,19,10],
[11872,20,64],[11872,21,2],[11872,22,1],[11872,23,103],[11872,24,3],
[11872,25,6],[11872,27,1],[11872,28,15],[11872,29,14],[11872,30,6],
[11872,31,1],[11872,32,1],[11872,33,2],[11872,34,7],[11872,35,8],
[11872,36,9],[11872,37,4],[11872,38,2],[11872,39,12],[11872,40,1],
[11872,41,4],[11872,42,1],[11872,45,4],[11872,46,1],[11872,47,1],
[11872,48,2],[11872,49,2],[11872,50,1],[11872,53,4],[11872,55,3],
[11872,56,2],[11872,57,3],[11872,58,4],[11872,59,6],[11872,60,1],
[11872,62,3],[11872,63,1],[11872,64,2],[11872,65,8],[11872,66,1],
[11872,67,5],[11872,70,3],[11872,71,2],[11872,72,1],[11872,73,2],

[11872,74,3],[11872,75,2],[11872,76,2],[11872,77,6],[11872,78,1],
[11873,24,2],[11874,2,1],[11874,14,1],[11874,25,1],[11874,31,1],
[11874,35,1],[11874,43,1],[11874,56,1],[11874,72,1],[11875,1,2],
[11875,7,4],[11875,8,1],[11875,9,3],[11875,10,9],[11875,11,2],
[11875,12,3],[11875,14,2],[11875,15,1],[11875,16,5],[11875,18,1],
[11875,19,7],[11875,20,2],[11875,28,8],[11875,29,1],[11875,30,1],
[11875,32,1],[11875,34,1],[11875,35,2],[11875,36,1],[11875,37,4],
[11875,38,1],[11875,39,1],[11875,41,4],[11875,45,3],[11875,46,1],
[11875,49,1],[11875,53,1],[11875,55,1],[11875,57,2],[11875,58,4],
[11875,59,2],[11875,63,3],[11875,64,1],[11875,65,9],[11875,67,2],
[11875,70,3],[11875,71,2],[11875,72,1],[11875,73,2],[11875,75,2],
[11875,76,2],[11875,77,2],[11876,8,1],[11876,20,1],[11877,1,1],
[11877,20,2],[11877,23,2],[11878,22,1],[11878,23,2],[11878,31,1],
[11878,32,2],[11878,44,3],[11878,51,4],[11878,66,3],[11878,69,2],
[11878,75,1],[11879,12,1],[11879,60,1],[11880,20,1],[11880,36,1],
[11881,28,1],[11881,50,1],[11882,1,1],[11882,20,4],[11882,29,1],
[11882,39,1],[11882,55,1],[11883,43,2],[11884,2,1],[11884,22,1],
[11884,27,1],[11884,28,1],[11884,31,2],[11885,32,4],[11885,41,1],
[11885,70,1],[11886,2,7],[11886,62,1],[11887,47,2],[11887,66,1],
[11887,72,1],[11888,3,1],[11888,23,1],[11889,9,2],[11889,10,1],
[11889,11,3],[11889,14,1],[11889,17,1],[11889,37,2],[11889,41,1],
[11890,0,1],[11890,15,1],[11890,37,1],[11890,39,1],[11890,65,1],
[11890,77,1],[11890,78,1],[11891,22,1],[11891,32,1],[11891,39,1],
[11892,63,6],[11892,74,3],[11892,75,9],[11892,76,13],[11893,9,1],
[11893,27,1],[11893,44,1],[11894,15,1],[11894,44,1],[11894,51,1],
[11895,25,1],[11895,27,1],[11896,10,1],[11896,16,1],[11896,41,1],
[11896,45,1],[11897,12,1],[11897,29,1],[11898,44,5],[11898,66,1],
[11899,22,1],[11899,23,1],[11899,28,1],[11899,42,1],[11900,1,1],
[11900,7,1],[11900,8,2],[11900,9,5],[11900,10,9],[11900,11,3],
[11900,12,2],[11900,13,2],[11900,14,3],[11900,16,3],[11900,17,2],
[11900,18,1],[11900,19,5],[11900,20,1],[11900,24,1],[11900,28,4],
[11900,29,1],[11900,34,1],[11900,36,1],[11900,39,1],[11900,41,1],
[11900,45,3],[11900,46,3],[11900,49,2],[11900,54,3],[11900,56,1],
[11900,57,3],[11900,58,2],[11900,59,2],[11900,63,1],[11900,64,1],
[11900,65,5],[11900,66,1],[11900,67,2],[11900,70,1],[11900,71,3],
[11900,73,2],[11900,74,2],[11900,75,1],[11901,1,2],[11901,39,1],
[11902,3,1],[11902,5,1],[11902,6,1],[11902,11,1],[11902,13,1],
[11902,17,1],[11902,20,6],[11902,21,1],[11902,22,1],[11902,23,2],
[11902,26,3],[11902,29,1],[11902,30,3],[11902,33,1],[11902,35,2],
[11902,42,2],[11902,46,2],[11902,56,1],[11902,59,1],[11902,74,1],
[11903,9,2],[11904,34,1],[11904,64,1],[11905,9,1],[11905,59,3],
[11905,67,1],[11906,1,3],[11906,11,1],[11906,12,1],[11906,13,9],
[11906,15,2],[11906,16,1],[11906,19,1],[11906,25,2],[11906,36,3],
[11906,37,1],[11906,46,1],[11906,55,1],[11906,65,1],[11907,22,1],
[11907,26,2],[11907,33,1],[11907,43,4],[11908,32,1],[11908,50,1],
[11908,53,1],[11909,5,1],[11909,23,3],[11909,32,1],[11909,42,1],
[11910,28,1],[11910,31,1],[11911,25,1],[11911,31,1],[11911,62,2],
[11912,25,2],[11912,28,1],[11913,27,5],[11913,43,2],[11914,4,1],
[11914,23,1],[11914,28,1],[11914,32,3],[11914,33,1],[11914,38,1],
[11914,52,1],[11914,61,3],[11914,62,2],[11914,66,1],[11915,8,1],

[11915,9,2],[11915,15,1],[11915,28,1],[11915,58,1],[11915,65,1],
[11916,15,1],[11916,53,1],[11917,5,1],[11917,8,1],[11917,20,1],
[11917,22,1],[11917,23,2],[11917,24,2],[11917,27,2],[11917,30,2],
[11917,32,3],[11917,33,1],[11917,34,1],[11917,37,1],[11917,42,1],
[11917,43,1],[11917,48,1],[11918,3,1],[11918,31,1],[11919,3,29],
[11919,4,1],[11919,5,1],[11919,24,1],[11919,25,1],[11919,27,10],
[11919,28,1],[11919,37,1],[11919,38,8],[11919,40,1],[11919,42,1],
[11919,52,27],[11919,69,1],[11919,72,2],[11920,28,2],[11921,19,1],
[11921,28,1],[11921,65,1],[11921,78,1],[11922,56,4],[11923,2,3],
[11923,10,1],[11923,31,2],[11923,33,1],[11923,59,1],[11924,40,1],
[11924,56,3],[11925,0,3],[11925,1,618],[11925,8,19],[11925,9,2],
[11925,10,1],[11925,12,20],[11925,13,28],[11925,15,7],[11925,16,1],
[11925,17,16],[11925,18,1],[11925,20,8],[11925,29,21],[11925,30,4],
[11925,31,1],[11925,34,13],[11925,36,8],[11925,39,15],[11925,53,9],
[11925,55,14],[11925,59,5],[11925,60,1],[11925,67,2],[11925,73,5],
[11926,3,4],[11926,23,1],[11926,24,13],[11926,28,1],[11926,32,1],
[11927,13,1],[11927,20,1],[11927,30,1],[11927,31,2],[11927,47,1],
[11927,54,1],[11928,2,5],[11929,28,7],[11930,3,1],[11930,5,1],
[11930,23,2],[11930,32,1],[11931,39,1],[11931,59,1],[11932,35,3],
[11933,1,1],[11933,3,2],[11933,4,1],[11933,12,1],[11933,17,1],
[11933,19,1],[11933,20,1],[11933,23,2],[11933,24,3],[11933,27,4],
[11933,33,3],[11933,35,2],[11933,39,1],[11933,53,1],[11933,55,2],
[11933,56,3],[11933,57,1],[11934,25,3],[11935,13,1],[11935,69,1],
[11936,4,2],[11936,16,1],[11936,20,1],[11936,22,2],[11936,23,5],
[11936,26,1],[11936,36,2],[11936,39,1],[11936,42,1],[11936,59,1],
[11936,62,1],[11937,3,1],[11937,26,3],[11937,28,2],[11937,31,1],
[11937,33,4],[11937,46,1],[11938,26,1],[11938,48,2],[11939,9,2],
[11939,29,1],[11940,9,1],[11940,18,1],[11940,20,1],[11940,27,3],
[11940,28,3],[11940,35,6],[11940,40,1],[11940,72,1],[11941,2,7],
[11941,5,1],[11941,23,1],[11942,23,5],[11942,25,2],[11942,26,1],
[11942,31,1],[11942,43,6],[11942,48,1],[11943,28,1],[11943,32,1],
[11944,15,1],[11944,17,1],[11945,9,2],[11945,19,1],[11945,63,2],
[11945,76,1],[11946,27,32],[11946,28,1],[11946,35,23],[11946,50,1],
[11946,70,2],[11946,76,1],[11947,8,1],[11947,28,69],[11948,17,1],
[11948,29,1],[11948,55,2],[11948,73,1],[11948,77,1],[11949,2,1],
[11949,3,1],[11949,4,1],[11949,22,1],[11949,24,2],[11949,25,1],
[11949,31,1],[11949,40,2],[11949,42,1],[11949,52,1],[11949,63,22],
[11949,66,1],[11949,72,2],[11949,74,28],[11949,75,20],[11949,76,27],
[11950,5,1],[11950,21,1],[11950,22,30],[11950,23,4],[11950,24,2],
[11950,25,5],[11950,26,4],[11950,27,19],[11950,31,6],[11950,32,8],
[11950,35,7],[11950,38,1],[11950,39,1],[11950,40,1],[11950,42,1],
[11950,43,15],[11950,48,2],[11950,58,1],[11950,59,2],[11951,5,1],
[11951,51,2],[11951,68,1],[11952,31,4],[11953,3,1],[11953,20,1],
[11953,27,2],[11953,28,2],[11953,43,1],[11954,23,1],[11954,24,1],
[11954,27,2],[11955,28,1],[11955,35,4],[11956,3,2],[11956,27,1],
[11957,61,2],[11958,59,2],[11959,4,1],[11959,25,2],[11959,31,1],
[11959,35,1],[11959,40,1],[11959,50,1],[11959,63,2],[11959,66,1],
[11959,68,2],[11959,69,1],[11959,70,2],[11959,72,1],[11959,75,1],
[11959,76,1],[11960,20,3],[11961,19,1],[11961,21,2],[11961,23,1],
[11961,24,1],[11961,27,1],[11961,32,1],[11961,35,1],[11961,40,1],

[11961,44,1],[11961,50,1],[11961,56,1],[11961,61,5],[11961,62,2],
[11961,68,2],[11961,70,12],[11961,72,2],[11962,13,1],[11962,20,1],
[11963,2,1],[11963,24,1],[11963,30,1],[11964,22,1],[11964,27,1],
[11965,1,1],[11965,22,1],[11965,28,1],[11965,31,1],[11965,33,1],
[11966,15,3],[11966,53,1],[11967,3,3],[11967,5,1],[11967,6,6],
[11967,23,1],[11967,29,1],[11967,42,2],[11967,47,2],[11967,52,1],
[11967,56,13],[11967,63,1],[11967,68,4],[11967,69,1],[11967,70,2],
[11967,74,14],[11967,76,3],[11968,22,2],[11968,27,1],[11969,15,2],
[11969,53,1],[11970,5,1],[11970,25,1],[11970,32,2],[11970,35,1],
[11970,38,1],[11971,5,2],[11972,2,2],[11973,2,1],[11973,33,1],
[11973,42,1],[11973,47,1],[11974,3,2],[11974,5,2],[11974,6,4],
[11974,22,4],[11974,31,1],[11974,32,4],[11974,33,4],[11974,35,3],
[11974,38,1],[11974,39,1],[11974,42,3],[11974,43,2],[11974,55,1],
[11974,56,3],[11974,68,6],[11974,72,1],[11975,4,1],[11975,22,1],
[11975,25,2],[11975,28,1],[11975,32,1],[11975,38,1],[11975,43,1],
[11975,50,2],[11975,53,2],[11976,28,1],[11976,44,1],[11977,3,2],
[11977,5,1],[11977,21,4],[11977,23,4],[11977,26,3],[11977,27,7],
[11977,28,1],[11977,31,2],[11977,32,5],[11977,34,1],[11977,35,3],
[11977,42,1],[11977,43,5],[11977,50,1],[11977,52,1],[11977,56,1],
[11977,68,1],[11978,28,1],[11978,32,5],[11978,44,1],[11979,28,1],
[11979,55,2],[11980,0,2],[11980,34,1],[11981,24,1],[11981,40,1],
[11982,12,1],[11982,35,1],[11983,20,7],[11983,73,1],[11984,31,2],
[11984,48,2],[11984,56,2],[11985,3,1],[11985,5,1],[11985,25,1],
[11985,31,5],[11985,43,1],[11985,56,1],[11985,68,1],[11986,21,1],
[11986,22,1],[11986,23,2],[11986,27,5],[11986,31,1],[11986,35,1],
[11987,12,1],[11987,15,16],[11987,45,1],[11987,55,47],[11987,65,2],
[11988,1,1],[11988,11,1],[11988,55,1],[11989,17,1],[11989,28,10],
[11989,50,1],[11990,3,1],[11990,24,1],[11990,32,1],[11991,32,2],
[11992,25,2],[11993,35,2],[11994,0,2],[11994,1,1],[11994,2,3],
[11994,3,5],[11994,4,1],[11994,5,4],[11994,6,4],[11994,10,1],
[11994,13,2],[11994,15,1],[11994,17,1],[11994,20,9],[11994,21,2],
[11994,22,6],[11994,23,5],[11994,24,2],[11994,25,1],[11994,26,1],
[11994,27,16],[11994,28,3],[11994,30,2],[11994,32,2],[11994,33,6],
[11994,34,1],[11994,35,4],[11994,38,1],[11994,39,1],[11994,45,1],
[11994,54,1],[11994,57,1],[11994,58,1],[11994,59,2],[11994,65,3],
[11994,70,1],[11994,71,1],[11995,20,1],[11995,28,3],[11995,37,1],
[11995,54,1],[11995,55,1],[11995,70,2],[11996,2,1],[11996,31,1],
[11997,28,2],[11997,47,1],[11998,1,1],[11998,5,2],[11998,13,1],
[11998,20,1],[11998,23,1],[11998,24,1],[11998,31,1],[11998,43,1],
[11998,74,1],[11998,76,2],[11999,1,1],[11999,13,1],[11999,15,1],
[11999,67,1],[12000,63,1],[12000,76,1],[12001,24,1],[12001,28,1],
[12002,44,1],[12002,66,2],[12003,27,4],[12003,31,1],[12003,40,1],
[12003,43,6],[12003,74,1],[12004,5,1],[12004,52,1],[12005,27,2],
[12006,45,1],[12006,55,1],[12007,31,5],[12007,33,1],[12008,13,1],
[12008,73,7],[12009,0,1],[12009,12,1],[12010,22,2],[12011,30,1],
[12011,31,2],[12011,62,2],[12012,8,5],[12012,34,4],[12013,0,2],
[12013,1,4],[12013,7,2],[12013,8,2],[12013,9,23],[12013,10,27],
[12013,11,6],[12013,12,3],[12013,13,3],[12013,14,7],[12013,15,4],
[12013,16,14],[12013,17,3],[12013,18,9],[12013,19,17],[12013,20,3],
[12013,24,2],[12013,29,3],[12013,34,5],[12013,36,7],[12013,37,14],

[12013,41,11],[12013,45,20],[12013,46,8],[12013,49,5],[12013,54,7],
[12013,55,5],[12013,57,10],[12013,58,10],[12013,59,10],[12013,60,4],
[12013,64,2],[12013,65,19],[12013,67,10],[12013,71,5],[12013,73,10],
[12013,74,1],[12013,77,10],[12013,78,3],[12014,1,1],[12014,22,7],
[12014,25,2],[12014,27,9],[12014,28,1],[12014,30,9],[12014,32,1],
[12014,33,17],[12014,35,6],[12014,36,1],[12014,38,2],[12014,52,6],
[12015,10,1],[12015,15,1],[12015,17,1],[12015,20,1],[12015,60,1],
[12015,65,1],[12016,41,1],[12016,65,1],[12017,0,1],[12017,5,1],
[12017,7,2],[12017,8,7],[12017,9,2],[12017,10,2],[12017,12,1],
[12017,15,1],[12017,18,1],[12017,19,1],[12017,20,1],[12017,22,2],
[12017,24,1],[12017,25,4],[12017,27,4],[12017,28,9],[12017,30,17],
[12017,32,3],[12017,33,27],[12017,35,22],[12017,37,3],[12017,38,1],
[12017,39,1],[12017,44,1],[12017,47,1],[12017,49,1],[12017,50,1],
[12017,52,29],[12017,53,1],[12017,65,1],[12017,66,1],[12017,74,2],
[12017,77,2],[12018,2,1],[12018,4,14],[12018,5,2],[12018,15,17],
[12018,17,14],[12018,18,1],[12018,19,1],[12018,20,1],[12018,24,3],
[12018,31,2],[12018,38,1],[12018,42,1],[12018,44,4],[12018,47,1],
[12018,50,11],[12018,53,11],[12018,55,7],[12018,58,1],[12018,65,2],
[12018,66,3],[12018,68,1],[12018,71,1],[12019,1,67],[12019,3,1],
[12019,4,1],[12019,5,1],[12019,7,2],[12019,8,1],[12019,9,2],
[12019,10,1],[12019,12,2],[12019,13,2],[12019,14,1],[12019,15,2],
[12019,18,1],[12019,19,2],[12019,20,2],[12019,21,1],[12019,24,2],
[12019,33,2],[12019,34,1],[12019,37,1],[12019,43,2],[12019,44,1],
[12019,55,2],[12019,59,1],[12019,61,2],[12019,66,1],[12019,68,1],
[12019,69,1],[12019,77,2],[12019,78,1],[12020,2,1],[12020,4,1],
[12020,33,2],[12020,35,2],[12020,51,1],[12020,70,1],[12020,74,1],
[12021,15,2],[12021,21,1],[12021,31,1],[12021,38,1],[12022,1,1],
[12022,3,1],[12022,4,2],[12022,8,1],[12022,13,4],[12022,19,1],
[12022,20,1],[12022,21,1],[12022,26,1],[12022,27,2],[12022,28,2],
[12022,29,1],[12022,31,1],[12022,32,1],[12022,35,1],[12022,37,2],
[12022,41,1],[12022,47,1],[12022,50,1],[12022,55,1],[12022,56,1],
[12022,62,1],[12022,74,1],[12022,76,1],[12022,77,1],[12023,3,1],
[12023,47,1],[12024,1,1],[12024,53,1],[12025,2,2],[12026,5,2],
[12026,22,1],[12026,52,1],[12027,28,2],[12027,32,245],[12027,33,1],
[12028,20,5],[12028,27,1],[12028,29,1],[12029,3,1],[12029,13,2],
[12029,22,4],[12029,24,2],[12029,26,2],[12029,27,1],[12029,33,1],
[12029,35,1],[12029,42,1],[12029,45,1],[12029,47,1],[12030,32,1],
[12030,33,2],[12030,35,1],[12030,42,2],[12031,22,1],[12031,27,1],
[12031,35,2],[12032,2,1],[12032,5,1],[12032,26,1],[12032,28,2],
[12032,31,1],[12032,42,3],[12032,50,1],[12033,6,1],[12033,24,1],
[12033,33,1],[12034,1,2],[12034,5,1],[12034,8,1],[12034,9,1],
[12034,12,4],[12034,13,4],[12034,15,1],[12034,19,1],[12034,20,1],
[12034,22,1],[12034,26,1],[12034,27,1],[12034,30,1],[12034,31,3],
[12034,33,12],[12034,52,1],[12034,53,1],[12034,63,2],[12034,76,4],
[12034,77,1],[12035,22,1],[12035,43,1],[12036,27,2],[12037,66,1],
[12037,74,1],[12038,3,1],[12038,6,1],[12038,22,2],[12038,26,1],
[12038,27,1],[12038,28,27],[12038,38,5],[12038,42,6],[12038,47,1],
[12038,48,8],[12038,62,1],[12038,63,1],[12039,3,1],[12039,5,1],
[12039,23,1],[12040,2,1],[12040,31,2],[12040,58,1],[12041,50,1],
[12041,77,1],[12042,4,1],[12042,26,1],[12043,21,1],[12043,27,2],

[12043,33,1],[12043,47,1],[12044,7,1],[12044,49,1],[12045,2,2],
[12046,12,1],[12046,24,1],[12046,37,2],[12046,53,1],[12047,28,1],
[12047,66,1],[12048,48,1],[12048,62,1],[12049,3,1],[12049,23,1],
[12049,24,2],[12049,32,2],[12049,35,1],[12049,37,1],[12049,40,1],
[12049,44,1],[12049,56,1],[12049,68,2],[12049,69,2],[12049,70,5],
[12049,74,1],[12050,4,1],[12050,16,1],[12050,17,30],[12050,50,2],
[12050,53,42],[12050,55,1],[12051,44,1],[12051,66,1],[12052,1,3],
[12052,3,10],[12052,4,1],[12052,5,1],[12052,6,3],[12052,23,6],
[12052,24,42],[12052,25,1],[12052,26,1],[12052,27,2],[12052,28,13],
[12052,32,1],[12052,33,2],[12052,37,1],[12052,38,3],[12052,40,1],
[12052,46,1],[12052,47,2],[12052,56,3],[12052,62,3],[12052,63,1],
[12053,1,2],[12053,22,2],[12053,27,1],[12053,30,1],[12053,45,2],
[12054,19,1],[12054,27,1],[12055,5,1],[12055,24,1],[12055,32,3],
[12055,33,3],[12055,40,1],[12055,42,2],[12055,43,1],[12055,48,1],
[12055,76,2],[12056,22,1],[12056,47,1],[12056,48,1],[12057,74,1],
[12057,76,2],[12058,22,2],[12058,24,1],[12058,27,1],[12059,2,1],
[12059,23,1],[12059,26,1],[12059,28,2],[12059,31,1],[12059,35,1],
[12060,3,1],[12060,25,1],[12060,28,3],[12060,30,1],[12060,32,1],
[12060,33,5],[12060,35,4],[12060,52,2],[12060,74,1],[12061,9,1],
[12061,15,1],[12062,1,1],[12062,16,1],[12062,20,1],[12062,28,1],
[12062,46,1],[12063,21,2],[12063,47,1],[12064,1,1],[12064,56,1],
[12065,2,8],[12065,3,9],[12065,5,5],[12065,6,30],[12065,8,2],
[12065,11,2],[12065,20,12],[12065,21,13],[12065,22,87],[12065,23,25],
[12065,24,24],[12065,25,1],[12065,26,16],[12065,27,73],[12065,28,9],
[12065,29,1],[12065,30,2],[12065,31,17],[12065,32,8],[12065,33,16],
[12065,34,3],[12065,35,28],[12065,37,1],[12065,38,18],[12065,39,1],
[12065,40,2],[12065,42,22],[12065,43,20],[12065,44,1],[12065,47,10],
[12065,48,5],[12065,51,1],[12065,56,3],[12065,59,4],[12065,63,2],
[12065,65,1],[12065,66,2],[12065,68,1],[12065,69,1],[12065,70,2],
[12065,72,4],[12065,74,4],[12065,75,2],[12065,76,2],[12066,5,1],
[12066,26,1],[12066,27,3],[12066,32,7],[12066,43,1],[12066,69,1],
[12066,70,1],[12067,25,6],[12067,27,1],[12068,5,1],[12068,22,1],
[12068,23,1],[12069,1,2],[12070,10,1],[12070,22,2],[12070,24,1],
[12070,27,1],[12070,43,1],[12070,48,2],[12071,63,1],[12071,74,2],
[12071,75,1],[12072,21,1],[12072,22,3],[12072,27,2],[12072,43,3],
[12072,62,16],[12072,68,1],[12073,25,2],[12074,33,3],[12075,22,1],
[12075,32,1],[12076,2,7],[12076,42,4],[12076,43,4],[12077,2,2],
[12077,20,1],[12077,21,2],[12077,22,1],[12077,23,17],[12077,24,16],
[12077,26,6],[12077,28,3],[12077,31,8],[12077,32,5],[12077,33,1],
[12077,36,1],[12077,38,2],[12077,39,1],[12077,40,1],[12077,42,2],
[12077,43,2],[12077,47,2],[12077,48,1],[12077,56,1],[12077,59,3],
[12077,62,6],[12077,68,1],[12078,3,3],[12078,6,1],[12078,23,1],
[12078,24,11],[12078,28,2],[12078,33,1],[12078,38,1],[12079,15,1],
[12079,23,2],[12079,25,27],[12079,26,2],[12079,28,1],[12079,30,5],
[12079,37,2],[12079,38,2],[12079,48,24],[12079,52,3],[12080,13,1],
[12080,59,1],[12081,2,3],[12081,53,1],[12082,24,1],[12082,28,2],
[12083,6,4],[12083,23,1],[12083,24,4],[12083,27,2],[12083,28,2],
[12083,35,3],[12083,56,1],[12083,66,1],[12083,74,3],[12084,28,1],
[12084,42,2],[12085,10,2],[12085,19,1],[12085,67,1],[12085,73,1],
[12086,28,6],[12087,32,2],[12087,74,1],[12088,27,2],[12089,28,1],

[12089,43,1],[12090,32,19],[12091,4,3],[12091,10,1],[12091,15,8],
[12091,17,4],[12091,20,2],[12091,32,1],[12091,44,1],[12091,50,1],
[12091,53,1],[12091,55,5],[12091,66,1],[12092,1,1],[12092,20,1],
[12092,39,1],[12093,2,1],[12093,3,1],[12093,4,58],[12093,5,16],
[12093,9,2],[12093,10,1],[12093,13,1],[12093,16,1],[12093,18,1],
[12093,19,1],[12093,22,1],[12093,23,6],[12093,25,2],[12093,26,1],
[12093,28,11],[12093,30,1],[12093,32,3],[12093,35,3],[12093,36,1],
[12093,38,10],[12093,40,2],[12093,41,1],[12093,42,1],[12093,43,1],
[12093,44,34],[12093,45,1],[12093,50,83],[12093,53,1],[12093,57,1],
[12093,58,1],[12093,62,1],[12093,63,1],[12093,64,1],[12093,66,28],
[12093,67,1],[12093,69,1],[12093,70,2],[12093,73,1],[12093,75,1],
[12093,76,2],[12094,15,2],[12094,28,9],[12095,17,1],[12095,53,3],
[12096,17,1],[12096,53,1],[12097,23,2],[12097,28,2],[12098,3,2],
[12098,26,5],[12098,27,4],[12098,32,4],[12098,43,1],[12099,9,11],
[12099,10,1],[12099,19,1],[12099,28,16],[12099,37,1],[12099,39,1],
[12099,41,1],[12099,50,1],[12099,55,1],[12099,63,1],[12099,65,1],
[12099,67,1],[12099,71,1],[12099,74,1],[12100,6,2],[12100,22,1],
[12101,22,1],[12101,59,1],[12102,23,1],[12102,27,1],[12102,28,1],
[12103,10,2],[12103,34,1],[12103,57,2],[12103,65,2],[12104,0,1],
[12104,1,1],[12104,9,2],[12104,10,2],[12104,12,1],[12104,15,5],
[12104,30,1],[12104,55,4],[12104,71,1],[12105,28,2],[12106,3,2],
[12106,24,1],[12106,28,2],[12106,32,1],[12106,33,1],[12106,48,2],
[12106,56,2],[12107,12,1],[12107,16,1],[12107,19,1],[12107,73,1],
[12108,1,1],[12108,41,1],[12109,2,1],[12109,32,9],[12110,20,1],
[12110,24,3],[12110,27,2],[12110,28,3],[12110,31,1],[12110,35,1],
[12111,22,1],[12111,27,1],[12111,32,1],[12111,47,1],[12112,20,2],
[12112,23,1],[12112,39,3],[12112,78,1],[12113,31,3],[12113,38,1],
[12114,23,1],[12114,24,2],[12114,32,4],[12115,5,1],[12115,32,1],
[12116,5,1],[12116,22,1],[12116,32,5],[12117,22,2],[12118,30,2],
[12119,42,1],[12119,70,1],[12120,22,4],[12120,27,1],[12120,28,1],
[12120,35,3],[12121,15,1],[12121,21,5],[12121,43,4],[12122,1,17],
[12122,2,63],[12122,3,33],[12122,4,10],[12122,5,63],[12122,6,5],
[12122,8,8],[12122,9,2],[12122,10,1],[12122,12,9],[12122,13,22],
[12122,14,1],[12122,15,13],[12122,17,3],[12122,18,1],[12122,20,21],
[12122,21,5],[12122,22,27],[12122,23,18],[12122,24,25],[12122,25,9],
[12122,26,16],[12122,27,33],[12122,28,24],[12122,29,2],[12122,30,6],
[12122,31,5],[12122,32,15],[12122,33,98],[12122,34,5],[12122,35,14],
[12122,37,14],[12122,38,4],[12122,39,3],[12122,40,2],[12122,41,2],
[12122,42,158],[12122,43,18],[12122,44,11],[12122,47,16],[12122,48,2],
[12122,50,14],[12122,51,8],[12122,52,3],[12122,53,1],[12122,55,6],
[12122,56,14],[12122,57,1],[12122,59,4],[12122,60,5],[12122,61,1],
[12122,62,5],[12122,63,2],[12122,65,3],[12122,66,13],[12122,67,2],
[12122,68,5],[12122,69,1],[12122,70,7],[12122,71,2],[12122,74,6],
[12122,75,5],[12122,76,8],[12122,77,3],[12123,3,1],[12123,6,1],
[12123,26,2],[12123,27,1],[12123,28,1],[12123,34,1],[12123,41,1],
[12123,44,1],[12124,2,3],[12124,3,3],[12124,5,1],[12124,21,1],
[12124,23,2],[12124,24,2],[12124,26,4],[12124,27,7],[12124,28,2],
[12124,31,6],[12124,35,2],[12124,37,3],[12124,39,1],[12124,40,1],
[12124,42,4],[12124,43,1],[12124,45,1],[12124,56,1],[12124,62,3],
[12125,2,1],[12125,20,1],[12126,8,1],[12126,30,1],[12127,2,2],

[12127,5,1],[12127,8,1],[12127,17,1],[12127,20,1],[12127,23,2],
[12127,26,3],[12127,28,2],[12127,31,2],[12127,35,2],[12127,39,1],
[12127,42,1],[12127,47,1],[12127,76,1],[12128,8,1],[12128,24,1],
[12128,26,1],[12128,27,1],[12128,31,1],[12128,39,2],[12129,1,3],
[12129,9,2],[12129,17,12],[12129,20,2],[12129,25,4],[12129,27,1],
[12129,30,10],[12129,37,2],[12129,39,6],[12129,45,4],[12129,53,12],
[12129,59,1],[12129,64,1],[12129,65,1],[12130,44,2],[12131,9,1],
[12131,10,1],[12131,11,1],[12131,39,1],[12131,54,1],[12131,59,2],
[12132,17,1],[12132,20,1],[12133,1,3],[12133,3,1],[12133,13,1],
[12133,20,1],[12133,34,1],[12133,42,1],[12133,46,1],[12133,64,1],
[12133,65,1],[12134,20,1],[12134,36,1],[12135,2,2],[12135,5,1],
[12135,46,1],[12135,65,1],[12135,74,1],[12136,50,1],[12136,53,1],
[12137,21,1],[12137,22,1],[12137,23,1],[12138,27,5],[12138,28,1],
[12138,47,1],[12139,2,2],[12139,32,1],[12140,1,1],[12140,5,1],
[12140,25,6],[12140,32,5],[12140,35,1],[12140,42,1],[12140,47,3],
[12140,61,3],[12140,62,2],[12141,56,1],[12141,70,1],[12142,22,2],
[12142,23,1],[12142,28,1],[12142,35,2],[12143,33,2],[12144,27,2],
[12145,24,2],[12146,21,1],[12146,28,1],[12147,59,1],[12147,60,1],
[12148,4,2],[12148,35,1],[12148,50,1],[12149,22,4],[12149,27,3],
[12149,28,1],[12149,35,2],[12150,2,2],[12150,5,1],[12150,22,1],
[12150,43,1],[12151,37,2],[12152,2,1],[12152,3,1],[12152,27,2],
[12152,28,14],[12152,31,1],[12152,33,3],[12152,35,2],[12152,40,1],
[12152,52,2],[12152,70,1],[12152,72,2],[12152,75,1],[12152,76,1],
[12153,4,4],[12153,5,2],[12153,44,1],[12153,50,1],[12154,20,2],
[12155,20,2],[12155,23,1],[12155,28,5],[12156,19,1],[12156,28,1],
[12156,30,2],[12156,74,1],[12157,37,1],[12157,50,1],[12157,53,1],
[12157,55,1],[12158,28,7],[12158,40,1],[12159,24,1],[12159,43,2],
[12159,56,21],[12159,76,1],[12160,36,1],[12160,43,2],[12161,25,1],
[12161,26,1],[12162,21,1],[12162,22,4],[12162,23,1],[12162,27,1],
[12162,38,1],[12163,4,1],[12163,23,1],[12164,21,1],[12164,31,1],
[12165,40,1],[12165,68,1],[12165,70,1],[12165,74,1],[12166,23,1],
[12166,33,1],[12167,28,1],[12167,31,1],[12168,2,13],[12168,4,1],
[12168,20,1],[12168,22,1],[12168,28,1],[12169,27,1],[12169,28,1],
[12170,6,1],[12170,33,1],[12171,12,1],[12171,16,1],[12172,21,1],
[12172,28,9],[12172,38,1],[12172,43,2],[12172,59,1],[12173,2,2],
[12173,27,1],[12173,28,1],[12173,42,1],[12174,2,1],[12174,24,1],
[12174,28,1],[12174,78,1],[12175,28,1],[12175,70,1],[12176,10,1],
[12176,13,1],[12176,15,3],[12176,16,1],[12176,55,4],[12177,2,2],
[12177,24,1],[12177,27,2],[12177,35,1],[12178,33,1],[12178,48,1],
[12179,3,1],[12179,23,1],[12179,56,1],[12180,3,2],[12180,24,1],
[12181,3,1],[12181,70,1],[12182,6,1],[12182,56,4],[12183,1,6],
[12183,13,2],[12183,31,1],[12184,24,2],[12185,15,3],[12185,32,1],
[12185,55,3],[12186,24,1],[12186,27,1],[12186,28,1],[12186,33,1],
[12186,42,1],[12187,2,2],[12187,42,1],[12187,43,2],[12188,9,1],
[12188,20,1],[12188,22,1],[12188,23,5],[12188,24,11],[12188,26,5],
[12188,27,5],[12188,28,1],[12188,32,9],[12188,35,1],[12188,36,1],
[12188,42,1],[12188,43,1],[12188,48,1],[12188,59,3],[12188,76,1],
[12189,25,1],[12189,56,1],[12190,2,5],[12191,27,1],[12191,28,1],
[12192,22,1],[12192,26,2],[12192,27,2],[12192,35,1],[12192,56,1],
[12192,72,1],[12193,25,2],[12193,28,2],[12193,32,2],[12194,4,1],

[12194,27,1],[12195,3,1],[12195,6,1],[12196,27,1],[12196,47,1],
[12197,5,5],[12197,21,1],[12198,24,1],[12198,32,1],[12198,47,1],
[12198,70,1],[12199,2,1],[12199,4,16],[12199,5,2],[12199,21,1],
[12199,23,1],[12199,24,1],[12199,28,5],[12199,31,2],[12199,32,1],
[12199,33,2],[12199,35,1],[12199,38,68],[12199,40,1],[12199,43,1],
[12199,44,17],[12199,50,31],[12199,56,1],[12199,66,81],[12199,68,1],
[12199,70,1],[12199,72,1],[12199,75,1],[12200,3,1],[12200,22,1],
[12200,32,3],[12200,56,2],[12200,65,1],[12201,2,5],[12202,23,2],
[12202,47,3],[12203,2,2],[12203,5,1],[12203,15,1],[12203,21,1],
[12203,31,1],[12204,2,1],[12204,20,2],[12204,23,3],[12204,62,4],
[12205,37,3],[12206,2,4],[12207,2,2],[12207,23,1],[12207,26,1],
[12207,30,1],[12207,31,1],[12207,32,1],[12208,24,1],[12208,32,1],
[12208,59,1],[12209,3,1],[12209,24,1],[12210,28,2],[12211,28,2],
[12212,24,1],[12212,27,1],[12212,47,2],[12213,25,1],[12213,27,6],
[12213,42,2],[12214,22,1],[12214,23,1],[12214,25,1],[12214,51,2],
[12214,66,1],[12214,68,1],[12215,20,2],[12215,22,1],[12215,31,1],
[12215,34,1],[12215,39,1],[12216,1,2],[12216,2,3],[12216,3,3],
[12216,6,1],[12216,8,1],[12216,9,1],[12216,12,17],[12216,13,2],
[12216,15,1],[12216,16,3],[12216,20,2],[12216,22,1],[12216,23,4],
[12216,24,2],[12216,25,133],[12216,26,10],[12216,27,4],[12216,28,1],
[12216,29,5],[12216,30,1],[12216,31,6],[12216,33,1],[12216,34,3],
[12216,39,11],[12216,43,1],[12216,44,1],[12216,47,1],[12216,48,5],
[12216,50,1],[12216,51,1],[12216,53,3],[12216,55,1],[12216,62,1],
[12216,66,2],[12216,79,1],[12217,25,5],[12218,59,2],[12219,3,1],
[12219,37,1],[12220,2,3],[12220,3,3],[12220,4,7],[12220,5,7],
[12220,6,2],[12220,21,2],[12220,23,4],[12220,24,6],[12220,25,14],
[12220,26,3],[12220,27,6],[12220,28,80],[12220,31,2],[12220,32,2],
[12220,33,2],[12220,35,5],[12220,38,3],[12220,40,12],[12220,43,3],
[12220,44,6],[12220,47,3],[12220,50,2],[12220,51,1],[12220,52,1],
[12220,61,1],[12220,62,3],[12220,63,7],[12220,66,1],[12220,68,11],
[12220,69,4],[12220,70,18],[12220,72,9],[12220,74,12],[12220,75,6],
[12220,76,12],[12221,5,1],[12221,21,2],[12221,23,1],[12221,26,1],
[12221,27,8],[12221,30,2],[12221,31,1],[12221,32,1],[12221,35,1],
[12222,38,1],[12222,44,1],[12223,1,2],[12224,22,3],[12225,4,1],
[12225,5,2],[12226,24,3],[12226,43,1],[12227,9,1],[12227,28,1],
[12227,55,1],[12228,21,2],[12229,28,3],[12229,32,3],[12229,44,1],
[12230,3,2],[12230,28,1],[12231,28,2],[12232,2,2],[12232,31,1],
[12233,3,1],[12233,22,1],[12233,23,1],[12233,24,1],[12233,26,1],
[12233,43,2],[12234,6,1],[12234,17,1],[12234,24,1],[12234,27,1],
[12234,31,1],[12235,1,2],[12235,7,1],[12235,9,2],[12235,10,1],
[12235,13,2],[12235,14,3],[12235,17,2],[12235,18,1],[12235,20,4],
[12235,34,1],[12235,45,2],[12235,53,1],[12235,54,1],[12235,55,1],
[12235,57,1],[12235,65,2],[12235,77,1],[12236,23,1],[12236,28,1],
[12237,2,7],[12237,8,8],[12237,12,1],[12237,29,2],[12237,31,1],
[12237,36,1],[12237,41,1],[12238,20,2],[12238,28,1],[12238,31,4],
[12238,56,1],[12239,3,1],[12239,9,1],[12239,37,1],[12240,5,1],
[12240,46,1],[12240,72,1],[12241,24,1],[12241,31,1],[12242,1,1],
[12242,10,1],[12242,16,2],[12242,19,1],[12242,46,1],[12242,65,1],
[12242,73,1],[12243,1,1],[12243,2,1],[12243,31,1],[12244,15,1],
[12244,20,1],[12244,55,1],[12245,6,5],[12245,28,1],[12245,56,9],

[12246,9,2],[12247,28,1],[12247,35,3],[12248,35,4],[12249,27,1],
[12249,56,1],[12250,27,1],[12250,28,1],[12251,28,3],[12251,41,1],
[12251,47,2],[12251,48,1],[12251,56,4],[12252,17,1],[12252,45,1],
[12252,53,1],[12253,2,3],[12253,3,1],[12253,4,2],[12253,5,1],
[12253,22,2],[12253,23,4],[12253,24,3],[12253,25,3],[12253,26,2],
[12253,27,2],[12253,28,23],[12253,31,4],[12253,32,2],[12253,33,2],
[12253,35,5],[12253,38,6],[12253,42,3],[12253,43,2],[12253,44,4],
[12253,47,2],[12253,50,2],[12253,51,1],[12253,62,1016],[12253,63,1],
[12253,65,1],[12253,66,3],[12253,69,1],[12253,70,3],[12253,75,1],
[12253,76,3],[12253,77,7],[12254,7,1],[12254,9,1],[12255,33,1],
[12255,47,1],[12256,10,1],[12256,15,4],[12256,16,1],[12256,17,2],
[12256,20,1],[12256,27,1],[12256,53,3],[12256,54,1],[12256,55,3],
[12257,1,1],[12257,7,2],[12257,9,1],[12257,10,1],[12257,14,1],
[12257,45,1],[12257,53,1],[12258,2,2],[12259,22,1],[12259,27,1],
[12260,3,1],[12260,12,1],[12260,20,3],[12260,22,1],[12260,23,1],
[12260,26,1],[12260,27,4],[12260,28,4],[12260,29,1],[12260,31,1],
[12260,32,1],[12260,43,1],[12261,20,2],[12262,2,2],[12263,34,2],
[12264,2,1],[12264,11,1],[12265,4,1],[12265,27,1],[12265,28,2],
[12265,39,1],[12265,40,1],[12265,42,1],[12265,44,1],[12265,70,1],
[12265,74,1],[12266,5,2],[12266,17,2],[12267,10,1],[12267,46,1],
[12268,63,3],[12268,74,1],[12268,75,2],[12268,76,1],[12269,2,2],
[12270,12,1],[12270,17,1],[12270,19,2],[12270,46,2],[12270,57,1],
[12270,59,1],[12270,64,1],[12270,65,1],[12270,71,1],[12270,77,1],
[12271,0,6],[12271,1,15],[12271,7,1],[12271,8,1],[12271,9,5],
[12271,10,10],[12271,11,2],[12271,12,808],[12271,13,4],[12271,14,3],
[12271,15,6],[12271,16,7],[12271,17,4],[12271,18,1],[12271,19,3],
[12271,20,5],[12271,27,1],[12271,29,22],[12271,30,6],[12271,32,1],
[12271,34,1],[12271,36,2],[12271,37,5],[12271,39,5],[12271,41,3],
[12271,45,4],[12271,46,2],[12271,49,2],[12271,53,2],[12271,54,4],
[12271,55,5],[12271,57,2],[12271,58,2],[12271,59,4],[12271,60,1],
[12271,65,1],[12271,67,3],[12271,70,4],[12271,73,3],[12271,77,5],
[12271,78,1],[12272,28,7],[12273,25,3],[12273,32,2],[12274,5,1],
[12274,25,1],[12275,6,1],[12275,22,1],[12275,24,1],[12275,27,2],
[12275,35,1],[12275,44,1],[12275,51,1],[12275,70,1],[12276,2,20],
[12276,3,2],[12276,8,1],[12276,19,1],[12276,21,1],[12276,22,4],
[12276,23,2],[12276,24,2],[12276,26,1],[12276,27,4],[12276,31,4],
[12276,34,1],[12276,35,1],[12276,42,1],[12276,43,1],[12276,47,6],
[12276,51,1],[12276,56,3],[12276,60,2],[12276,68,1],[12276,70,2],
[12276,75,1],[12277,4,2],[12277,26,1],[12277,27,3],[12277,62,1],
[12278,62,2],[12279,2,1],[12279,35,2],[12279,43,1],[12280,23,1],
[12280,27,1],[12281,33,1],[12281,43,1],[12282,3,1],[12282,27,1],
[12282,33,1],[12282,55,1],[12282,70,1],[12283,23,1],[12283,25,1],
[12283,27,1],[12283,28,1],[12284,19,1],[12284,49,1],[12285,28,1],
[12285,62,1],[12286,3,1],[12286,22,1],[12286,48,1],[12287,3,2],
[12287,6,2],[12287,11,1],[12287,21,1],[12287,23,1],[12287,26,3],
[12287,27,3],[12287,31,4],[12287,32,5],[12287,42,2],[12287,43,1],
[12287,47,1],[12287,48,1],[12287,52,1],[12287,64,1],[12287,66,1],
[12287,70,1],[12288,51,2],[12288,61,1],[12288,69,1],[12289,5,2],
[12290,15,2],[12290,34,1],[12290,35,1],[12290,68,2],[12290,70,1],
[12290,76,1],[12290,78,1],[12291,28,4],[12292,0,3],[12293,0,1],

[12293,1,1],[12293,15,2],[12293,17,2],[12293,41,1],[12293,53,2],
[12293,55,17],[12293,73,1],[12294,1,5],[12295,8,1],[12295,78,1],
[12296,28,5],[12297,1,1],[12297,10,1],[12297,31,1],[12298,74,2],
[12298,75,1],[12298,76,1],[12299,27,1],[12299,35,6],[12299,44,1],
[12300,13,1],[12300,29,1],[12301,30,1],[12301,46,1],[12302,1,2],
[12302,11,1],[12302,16,1],[12302,18,1],[12302,19,1],[12302,20,1],
[12302,34,1],[12302,37,1],[12302,41,1],[12302,57,1],[12302,67,1],
[12303,2,1],[12303,4,34],[12303,5,6],[12303,9,1],[12303,13,1],
[12303,15,1],[12303,17,1],[12303,22,2],[12303,23,1],[12303,25,2],
[12303,26,1],[12303,27,1],[12303,28,4],[12303,31,1],[12303,32,2],
[12303,33,1],[12303,34,3],[12303,35,2],[12303,38,1],[12303,42,2],
[12303,43,3],[12303,44,7],[12303,45,1],[12303,50,38],[12303,53,1],
[12303,55,1],[12303,58,1],[12303,59,1],[12303,66,3],[12303,76,1],
[12304,1,2],[12304,2,34],[12304,3,29],[12304,4,1],[12304,5,8],
[12304,6,2],[12304,9,5],[12304,10,4],[12304,11,3],[12304,12,1],
[12304,13,6],[12304,14,3],[12304,15,1],[12304,16,3],[12304,18,1],
[12304,19,1],[12304,20,28],[12304,21,18],[12304,22,6],[12304,23,51],
[12304,24,59],[12304,25,21],[12304,26,44],[12304,27,36],[12304,28,44],
[12304,29,1],[12304,30,2],[12304,31,49],[12304,32,28],[12304,33,3],
[12304,34,1],[12304,35,10],[12304,36,3],[12304,37,6],[12304,39,8],
[12304,40,1],[12304,41,1],[12304,42,28],[12304,43,34],[12304,44,2],
[12304,45,1],[12304,46,2],[12304,47,17],[12304,48,15],[12304,50,3],
[12304,52,4],[12304,55,2],[12304,56,2],[12304,58,1],[12304,59,5],
[12304,60,2],[12304,62,33],[12304,63,1],[12304,64,1],[12304,65,3],
[12304,66,1],[12304,69,2],[12304,70,1],[12304,71,2],[12304,72,2],
[12304,74,1],[12304,75,1],[12304,76,2],[12304,77,2],[12305,5,1],
[12305,30,1],[12306,43,1],[12306,56,1],[12307,1,2],[12307,3,1],
[12307,20,1],[12307,23,3],[12307,24,1],[12307,26,2],[12307,28,1],
[12307,31,1],[12308,5,1],[12308,26,1],[12308,28,1],[12309,37,1],
[12309,48,1],[12309,60,1],[12309,78,1],[12310,1,1],[12310,5,1],
[12310,9,1],[12310,34,17],[12310,77,1],[12311,49,1],[12311,63,7],
[12311,74,7],[12311,75,4],[12311,76,11],[12312,0,19],[12312,1,47],
[12312,7,8],[12312,8,8],[12312,9,77],[12312,10,113],[12312,11,38],
[12312,12,38],[12312,13,23],[12312,14,19],[12312,15,28],[12312,16,47],
[12312,17,20],[12312,18,21],[12312,19,70],[12312,20,33],[12312,29,13],
[12312,30,7],[12312,34,19],[12312,36,15],[12312,37,31],[12312,39,11],
[12312,41,37],[12312,45,48],[12312,46,16],[12312,49,17],[12312,53,21],
[12312,54,22],[12312,55,16],[12312,57,35],[12312,58,23],[12312,59,30],
[12312,60,9],[12312,64,7],[12312,65,58],[12312,67,25],[12312,71,21],
[12312,73,28],[12312,77,30],[12312,78,14],[12313,24,1],[12313,26,3],
[12313,33,8],[12313,50,1],[12314,17,1],[12314,20,1],[12314,41,1],
[12314,60,1],[12314,78,1],[12315,2,23],[12315,5,3],[12315,6,1],
[12315,17,2],[12315,20,2],[12315,21,2],[12315,22,2],[12315,23,14],
[12315,25,1],[12315,26,1],[12315,27,1],[12315,28,1],[12315,30,1],
[12315,31,3],[12315,32,3],[12315,35,3],[12315,39,1],[12315,40,1],
[12315,42,1],[12315,46,1],[12315,47,1],[12315,50,1],[12315,53,1],
[12315,59,3],[12315,60,2],[12315,62,6],[12316,2,2],[12317,9,1],
[12317,15,2],[12317,20,14],[12317,22,1],[12317,23,2],[12317,25,1],
[12317,32,6],[12317,50,1],[12317,51,1],[12317,66,1],[12317,74,2],
[12318,23,1],[12318,26,1],[12319,28,1],[12319,75,1],[12320,22,1],

[12320,27,1],[12321,0,4],[12321,1,4],[12321,2,6],[12321,7,1],
[12321,8,4],[12321,9,4],[12321,10,9],[12321,11,3],[12321,12,6],
[12321,13,10],[12321,14,2],[12321,15,5],[12321,16,5],[12321,17,1],
[12321,18,2],[12321,19,11],[12321,20,3],[12321,28,11],[12321,29,1],
[12321,30,1],[12321,31,3],[12321,34,152],[12321,36,1],[12321,37,1],
[12321,39,2],[12321,41,4],[12321,45,4],[12321,53,4],[12321,54,2],
[12321,55,4],[12321,56,1],[12321,57,3],[12321,58,2],[12321,59,2],
[12321,65,3],[12321,67,2],[12321,71,2],[12321,73,1],[12321,77,9],
[12322,35,1],[12322,38,1],[12323,20,2],[12323,24,2],[12323,53,1],
[12324,1,2],[12324,8,2],[12324,12,1],[12324,13,2],[12324,23,1],
[12324,26,1],[12324,27,2],[12324,29,1],[12324,34,1],[12324,35,2],
[12324,43,3],[12324,73,1],[12325,22,1],[12325,23,2],[12325,24,1],
[12325,27,2],[12325,28,1],[12325,32,2],[12325,35,2],[12326,8,1],
[12326,30,1],[12326,43,1],[12327,13,2],[12327,59,1],[12328,20,1],
[12328,21,3],[12328,22,1],[12328,23,2],[12328,24,1],[12328,27,1],
[12328,32,3],[12328,35,1],[12328,39,3],[12328,42,1],[12328,47,1],
[12328,59,1],[12328,62,3],[12328,76,1],[12329,2,1],[12329,28,1],
[12330,23,1],[12330,27,1],[12331,2,5],[12331,8,1],[12331,20,2],
[12332,28,1],[12332,42,2],[12332,68,1],[12332,70,1],[12333,2,1],
[12333,22,1],[12333,27,2],[12333,28,1],[12334,4,1],[12334,66,1],
[12335,3,2],[12335,12,2],[12335,15,1],[12336,22,2],[12336,28,1],
[12337,20,2],[12338,14,1],[12338,22,1],[12338,30,1],[12338,31,2],
[12338,46,1],[12338,48,1],[12339,2,1],[12339,3,1],[12339,21,1],
[12340,2,1],[12340,21,2],[12340,42,1],[12340,46,1],[12340,62,2],
[12341,21,1],[12341,22,1],[12341,23,4],[12341,24,1],[12341,26,1],
[12341,33,1],[12341,42,4],[12341,47,1],[12341,48,4],[12342,31,1],
[12342,62,7],[12343,25,3],[12344,4,2],[12344,38,1],[12344,44,1],
[12344,50,2],[12345,21,1],[12345,51,1],[12345,63,13],[12345,74,14],
[12345,75,6],[12345,76,28],[12346,15,3],[12346,24,1],[12346,28,4],
[12346,45,1],[12346,55,2],[12346,62,1],[12346,70,1],[12346,74,1],
[12347,28,2],[12348,46,1],[12348,59,1],[12349,23,1],[12349,43,3],
[12350,5,1],[12350,6,1],[12350,21,3],[12350,23,2],[12350,24,4],
[12350,25,1],[12350,26,3],[12350,27,3],[12350,28,1],[12350,31,5],
[12350,32,8],[12350,37,1],[12350,42,1],[12350,43,3],[12351,14,1],
[12351,73,1],[12352,28,1],[12352,33,1],[12353,5,1],[12353,25,1],
[12353,27,2],[12353,33,1],[12353,47,1],[12354,28,1],[12354,42,2],
[12355,3,1],[12355,4,1],[12355,6,7],[12355,21,4],[12355,23,6],
[12355,24,3],[12355,25,1],[12355,26,2],[12355,27,3],[12355,28,14],
[12355,30,4],[12355,33,5],[12355,35,4],[12355,47,8],[12355,50,1],
[12355,51,4],[12355,53,1],[12355,62,1],[12355,79,1],[12356,2,4],
[12356,28,1],[12357,2,2],[12357,3,1],[12357,8,1],[12357,20,1],
[12357,31,1],[12357,33,1],[12357,55,1],[12357,64,1],[12358,22,1],
[12358,23,1],[12358,27,1],[12358,28,3],[12359,29,1],[12359,39,1],
[12360,46,1],[12360,72,1],[12360,74,1],[12360,76,1],[12361,20,1],
[12361,24,1],[12361,28,1],[12361,32,1],[12361,34,3],[12361,48,1],
[12361,55,1],[12362,62,2],[12363,5,1],[12363,35,1],[12364,27,2],
[12365,10,1],[12365,53,1],[12365,70,1],[12366,12,2],[12366,39,1],
[12366,46,1],[12367,0,2],[12368,4,1],[12368,28,1],[12368,50,1],
[12369,22,1],[12369,24,1],[12369,26,1],[12369,27,1],[12369,34,1],
[12369,39,1],[12370,3,18],[12370,5,2],[12370,6,2],[12370,10,1],

[12370,15,4],[12370,17,7],[12370,18,1],[12370,19,1],[12370,21,5],
[12370,22,4],[12370,23,4],[12370,24,22],[12370,25,6],[12370,26,3],
[12370,27,9],[12370,28,5],[12370,31,4],[12370,32,15],[12370,33,12],
[12370,39,1],[12370,42,1],[12370,43,10],[12370,47,1],[12370,48,2],
[12370,50,1],[12370,53,5],[12370,55,2],[12370,56,2],[12370,59,1],
[12370,62,1],[12370,63,1],[12370,66,1],[12370,76,2],[12371,23,1],
[12371,26,1],[12371,43,1],[12372,2,1],[12372,28,1],[12372,31,3],
[12373,15,1],[12373,44,1],[12373,55,1],[12374,1,2],[12374,15,1],
[12374,20,1],[12374,29,2],[12374,39,6],[12375,24,4],[12375,28,3],
[12375,30,1],[12375,32,3],[12375,42,1],[12375,43,1],[12375,59,1],
[12376,8,1],[12376,15,1],[12376,23,2],[12376,24,1],[12376,27,2],
[12376,28,2],[12376,35,1],[12376,45,1],[12376,60,1],[12376,76,1],
[12377,5,2],[12377,15,1],[12377,16,1],[12377,20,1],[12377,23,1],
[12377,24,1],[12377,26,1],[12377,27,1],[12377,32,1],[12377,39,1],
[12377,42,1],[12377,44,2],[12377,55,2],[12377,56,1],[12377,59,1],
[12377,77,1],[12378,43,2],[12379,3,1],[12379,22,2],[12379,27,7],
[12379,28,1],[12379,35,1],[12380,1,1],[12380,30,1],[12381,22,1],
[12381,69,2],[12382,28,1],[12382,63,1],[12383,2,1],[12383,9,1],
[12383,30,1],[12383,43,1],[12384,6,2],[12384,35,2],[12385,1,3],
[12385,15,1],[12386,13,1],[12386,22,2],[12386,27,1],[12386,38,1],
[12386,42,1],[12387,6,1],[12387,24,1],[12387,28,1],[12387,43,1],
[12388,17,1],[12388,53,3],[12389,19,1],[12389,20,1],[12389,21,1],
[12389,23,1],[12389,27,9],[12389,28,1],[12389,31,1],[12389,36,1],
[12390,66,2],[12391,4,2],[12391,50,5],[12391,56,1],[12391,66,1],
[12392,3,1],[12392,8,2],[12392,20,1],[12392,22,3],[12392,24,1],
[12392,27,1],[12392,37,1],[12393,2,2],[12393,10,1],[12393,20,1],
[12393,23,2],[12393,26,2],[12393,33,1],[12393,42,1],[12393,52,1],
[12393,68,1],[12394,21,1],[12394,25,1],[12394,27,1],[12394,32,1],
[12395,9,1],[12395,17,1],[12396,4,1],[12396,28,1],[12397,63,1],
[12397,74,2],[12397,76,1],[12398,22,1],[12398,47,1],[12398,72,1],
[12399,3,1],[12399,22,1],[12399,27,1],[12399,31,1],[12399,45,1],
[12400,25,3],[12401,12,1],[12401,15,9],[12401,39,1],[12401,46,1],
[12401,49,1],[12401,55,36],[12401,71,2],[12402,2,1],[12402,6,1],
[12402,27,1],[12402,31,1],[12402,33,1],[12402,38,1],[12402,40,2],
[12402,42,1],[12402,48,1],[12402,50,1],[12402,63,1],[12402,68,1],
[12402,69,2],[12402,72,1],[12402,74,3],[12403,25,1],[12403,56,7],
[12404,28,3],[12405,2,3],[12405,25,1],[12405,28,2],[12406,8,1],
[12406,65,1],[12407,31,3],[12408,23,5],[12409,32,3],[12410,1,1],
[12410,15,1],[12410,17,1],[12410,53,1],[12411,2,2],[12411,15,1],
[12411,23,6],[12411,26,3],[12411,31,1],[12411,33,2],[12411,39,1],
[12411,75,1],[12412,1,1],[12412,5,1],[12412,13,6],[12412,15,1],
[12412,18,1],[12412,20,1],[12412,22,1],[12412,23,2],[12412,25,5],
[12412,26,3],[12412,27,2],[12412,28,2],[12412,32,6],[12412,34,1],
[12412,35,2],[12412,36,1],[12412,39,3],[12412,43,1],[12412,45,1],
[12412,46,1],[12412,52,1],[12412,62,15],[12413,16,2],[12413,20,4],
[12413,32,1],[12413,33,1],[12413,61,1],[12414,25,1],[12414,32,1],
[12415,42,2],[12416,4,1],[12416,24,1],[12416,33,1],[12416,35,1],
[12417,8,1],[12417,15,1],[12417,58,1],[12418,1,1],[12418,3,1],
[12418,15,1],[12418,27,1],[12418,35,1],[12418,39,1],[12418,70,1],
[12419,5,1],[12419,27,1],[12419,28,8],[12419,32,7],[12419,33,1],

[12419,35,2],[12419,72,1],[12419,75,2],[12420,35,4],[12420,44,1],
[12421,44,2],[12422,28,3],[12422,42,1],[12423,7,1],[12423,9,1],
[12423,23,1],[12423,27,1],[12423,28,1],[12423,62,1],[12424,25,1],
[12424,31,1],[12424,40,1],[12424,61,1],[12424,70,1],[12425,31,1],
[12425,38,1],[12426,3,1],[12426,22,2],[12427,0,1],[12427,22,1],
[12427,23,1],[12427,25,9],[12427,26,1],[12427,27,3],[12427,28,6],
[12427,30,4],[12427,33,55],[12427,35,37],[12427,44,1],[12427,62,2],
[12427,65,1],[12427,67,1],[12427,74,1],[12428,2,15],[12428,3,30],
[12428,4,3],[12428,5,8],[12428,6,8],[12428,8,1],[12428,10,2],
[12428,12,1],[12428,19,1],[12428,20,5],[12428,21,17],[12428,22,21],
[12428,23,38],[12428,24,37],[12428,25,19],[12428,26,26],[12428,27,54],
[12428,28,25],[12428,30,5],[12428,31,18],[12428,32,26],[12428,33,12],
[12428,34,1],[12428,35,47],[12428,36,1],[12428,37,3],[12428,38,6],
[12428,39,4],[12428,40,6],[12428,41,1],[12428,42,12],[12428,43,22],
[12428,44,6],[12428,45,2],[12428,46,2],[12428,47,10],[12428,48,8],
[12428,50,8],[12428,51,1],[12428,52,5],[12428,56,9],[12428,57,1],
[12428,58,1],[12428,59,4],[12428,60,1],[12428,61,2],[12428,62,6],
[12428,63,7],[12428,64,1],[12428,65,1],[12428,66,2],[12428,67,2],
[12428,68,5],[12428,69,2],[12428,70,12],[12428,72,1],[12428,74,3],
[12428,75,1],[12428,76,4],[12428,77,1],[12428,78,2],[12429,3,1],
[12429,25,1],[12429,26,2],[12429,27,2],[12429,32,1],[12429,35,1],
[12429,43,1],[12430,6,1],[12430,51,2],[12430,56,1],[12430,69,1],
[12431,20,1],[12431,22,2],[12431,23,3],[12431,24,6],[12431,25,12],
[12431,26,1],[12431,27,1],[12431,30,1],[12431,32,1],[12431,33,4],
[12431,35,1],[12431,56,1],[12432,9,2],[12432,19,1],[12432,20,1],
[12432,41,1],[12432,46,1],[12432,60,1],[12433,3,1],[12433,23,1],
[12433,24,2],[12433,28,1],[12434,31,2],[12435,2,1],[12435,55,1],
[12436,21,2],[12437,9,3],[12437,15,11],[12437,55,1],[12438,31,2],
[12438,39,1],[12438,42,1],[12439,5,5],[12439,15,1],[12439,43,1],
[12439,70,1],[12440,27,2],[12440,47,1],[12441,13,1],[12441,15,1],
[12441,19,1],[12441,23,1],[12441,24,1],[12441,25,1],[12441,33,1],
[12441,38,1],[12441,46,1],[12441,56,5],[12442,2,1],[12442,3,12],
[12442,4,4],[12442,5,3],[12442,6,6],[12442,15,1],[12442,16,1],
[12442,21,2],[12442,22,5],[12442,23,5],[12442,24,3],[12442,26,11],
[12442,27,8],[12442,28,1],[12442,31,5],[12442,33,1],[12442,35,2],
[12442,37,4],[12442,38,6],[12442,40,1],[12442,42,33],[12442,43,3],
[12442,44,3],[12442,46,4],[12442,47,19],[12442,48,92],[12442,50,3],
[12442,52,1],[12442,56,2],[12442,61,3],[12442,62,2],[12442,65,1],
[12442,70,3],[12442,74,1],[12442,75,3],[12442,76,1],[12443,13,2],
[12443,58,1],[12443,77,1],[12444,5,1],[12444,21,1],[12444,23,5],
[12444,24,3],[12444,25,1],[12444,26,1],[12444,27,5],[12444,28,2],
[12444,35,4],[12444,43,2],[12445,14,1],[12445,16,1],[12445,77,1],
[12446,1,1],[12446,10,5],[12446,11,2],[12446,12,2],[12446,15,1],
[12446,17,2],[12446,19,2],[12446,28,1],[12446,37,1],[12446,45,1],
[12446,46,1],[12446,47,1],[12446,57,1],[12446,58,1],[12446,65,2],
[12446,67,1],[12446,71,3],[12446,78,1],[12447,24,2],[12447,43,6],
[12447,76,1],[12448,13,1],[12448,26,2],[12448,45,1],[12448,76,1],
[12449,1,2],[12449,24,1],[12449,36,1],[12449,43,1],[12450,2,2],
[12450,3,1],[12450,4,1],[12450,21,1],[12450,22,1],[12450,24,1],
[12450,27,1],[12450,28,17],[12450,31,2],[12450,38,1],[12450,42,2],

[12450,44,1],[12450,47,1],[12450,50,1],[12450,62,2],[12450,66,1],
[12450,79,1],[12451,2,1],[12451,3,6],[12451,16,1],[12451,29,1],
[12451,35,3],[12451,73,3],[12451,74,1],[12452,30,2],[12453,27,3],
[12454,33,9],[12455,15,1],[12455,27,1],[12455,28,3],[12455,35,1],
[12455,39,1],[12456,26,1],[12456,43,2],[12457,21,1],[12457,23,2],
[12457,25,2],[12457,26,3],[12457,27,1],[12457,28,23],[12457,32,1],
[12457,33,1],[12457,40,2],[12457,42,2],[12457,43,1],[12457,44,1],
[12457,47,1],[12457,50,3],[12457,56,1],[12457,62,1],[12457,63,1],
[12457,70,1],[12457,72,1],[12457,74,1],[12458,25,1],[12458,31,4],
[12458,39,2],[12458,43,3],[12459,3,7],[12459,5,2],[12459,6,3],
[12459,15,1],[12459,22,1],[12459,23,2],[12459,24,16],[12459,25,54],
[12459,26,2],[12459,27,6],[12459,28,5],[12459,31,1],[12459,32,5],
[12459,33,5],[12459,38,2],[12459,42,3],[12459,43,1],[12459,46,1],
[12459,47,9],[12459,48,3],[12459,50,1],[12459,52,1],[12459,56,2],
[12459,57,1],[12459,72,1],[12459,74,1],[12459,77,1],[12460,5,1],
[12460,22,1],[12460,33,1],[12461,28,3],[12462,17,4],[12462,20,1],
[12462,23,1],[12462,32,2],[12462,53,8],[12463,1,1],[12463,12,2],
[12464,28,8],[12465,48,4],[12466,2,1],[12466,47,1],[12466,50,1],
[12466,63,1],[12467,23,2],[12467,24,1],[12468,32,1],[12468,56,1],
[12469,1,2],[12469,2,2],[12469,3,3],[12469,13,1],[12469,17,1],
[12469,19,1],[12469,22,2],[12469,23,1],[12469,27,4],[12469,28,1],
[12469,29,1],[12469,31,1],[12469,32,3],[12469,35,5],[12469,37,44],
[12469,39,1],[12469,48,2],[12469,56,15],[12469,62,1],[12469,70,1],
[12469,76,1],[12469,78,1],[12470,19,1],[12470,50,2],[12471,2,5],
[12471,27,1],[12472,20,2],[12472,24,3],[12472,35,1],[12473,22,1],
[12473,28,1],[12474,23,1],[12474,70,1],[12475,21,1],[12475,27,2],
[12475,35,1],[12476,42,1],[12476,69,2],[12476,70,2],[12477,30,4],
[12478,35,4],[12479,1,13],[12479,7,2],[12479,8,7],[12479,9,1],
[12479,12,7],[12479,13,3],[12479,15,1],[12479,17,2],[12479,19,1],
[12479,20,3],[12479,29,1],[12479,30,1],[12479,34,6],[12479,36,4],
[12479,37,2],[12479,39,8],[12479,53,6],[12479,55,2],[12479,73,3],
[12480,0,2],[12480,1,17],[12480,2,6],[12480,7,1],[12480,8,3],
[12480,12,65],[12480,13,18],[12480,15,3],[12480,16,2],[12480,17,5],
[12480,20,3],[12480,22,1],[12480,23,1],[12480,26,1],[12480,29,47],
[12480,30,5],[12480,34,1],[12480,36,4],[12480,39,4],[12480,42,1],
[12480,53,7],[12480,55,3],[12480,56,2],[12480,59,1],[12480,70,1],
[12480,72,1],[12480,73,1],[12481,36,1],[12481,41,2],[12481,60,1],
[12482,2,1],[12482,5,2],[12482,20,1],[12482,21,2],[12482,22,5],
[12482,24,1],[12482,25,3],[12482,27,2],[12482,38,1],[12482,42,1],
[12482,50,1],[12483,25,2],[12483,42,1],[12484,15,1],[12484,28,3],
[12484,53,1],[12484,55,2],[12485,9,1],[12485,28,2],[12485,70,1],
[12486,23,1],[12486,25,1],[12486,28,1],[12486,70,1],[12487,6,1],
[12487,22,1],[12487,24,1],[12487,25,1],[12487,28,1],[12487,33,2],
[12487,47,1],[12488,15,1],[12488,44,1],[12488,66,1],[12488,73,1],
[12489,20,1],[12489,26,1],[12490,40,3],[12490,68,1],[12490,69,2],
[12490,70,9],[12490,72,3],[12491,24,5],[12492,27,1],[12492,44,9],
[12492,47,1],[12492,50,1],[12492,66,3],[12493,2,2],[12494,3,1],
[12494,28,1],[12495,23,4],[12495,27,1],[12495,35,1],[12496,23,3],
[12496,42,1],[12496,59,1],[12497,2,3],[12497,22,2],[12497,24,2],
[12497,25,4],[12497,27,2],[12497,31,1],[12497,42,1],[12497,43,1],

[12497,50,1],[12498,28,26],[12499,26,1],[12499,28,5],[12500,3,1],
[12500,26,1],[12500,27,3],[12500,47,1],[12501,47,1],[12501,52,15],
[12502,0,1],[12502,1,4],[12502,2,3],[12502,3,5],[12502,4,65],
[12502,5,8],[12502,7,1],[12502,9,7],[12502,10,11],[12502,11,2],
[12502,12,7],[12502,13,6],[12502,14,1],[12502,15,70],[12502,16,1],
[12502,17,136],[12502,18,1],[12502,19,5],[12502,20,11],[12502,21,1],
[12502,22,1],[12502,23,6],[12502,24,8],[12502,25,1],[12502,26,1],
[12502,27,4],[12502,28,34],[12502,29,4],[12502,30,1],[12502,31,1],
[12502,33,2],[12502,34,2],[12502,35,4],[12502,36,1],[12502,37,2],
[12502,38,10],[12502,39,2],[12502,41,6],[12502,42,3],[12502,43,1],
[12502,44,75],[12502,45,3],[12502,46,3],[12502,47,1],[12502,49,3],
[12502,50,67],[12502,51,1],[12502,53,121],[12502,55,40],[12502,56,3],
[12502,57,2],[12502,58,2],[12502,59,3],[12502,60,2],[12502,63,1],
[12502,65,1],[12502,66,81],[12502,67,1],[12502,70,1],[12502,72,3],
[12502,73,1],[12502,74,2],[12502,75,5],[12502,76,1],[12502,77,4],
[12503,2,2],[12503,47,2],[12504,26,1],[12504,27,3],[12504,28,1],
[12504,32,3],[12504,52,1],[12505,51,3],[12506,1,1],[12506,3,1],
[12506,22,1],[12506,26,1],[12506,32,1],[12506,42,1],[12506,43,1],
[12506,65,1],[12507,5,1],[12507,20,1],[12507,28,2],[12507,32,1],
[12507,70,1],[12508,24,1],[12508,47,2],[12508,50,1],[12509,3,2],
[12509,20,2],[12509,23,1],[12509,28,1],[12509,32,1],[12510,23,1],
[12510,27,1],[12511,74,3],[12511,75,1],[12511,76,1],[12512,26,1],
[12512,28,1],[12512,43,1],[12513,55,2],[12514,57,1],[12514,65,1],
[12515,61,1],[12515,68,2],[12515,69,1],[12515,70,13],[12515,72,2],
[12516,13,1],[12516,46,1],[12517,25,1],[12517,35,3],[12518,26,1],
[12518,74,1],[12519,5,1],[12519,9,1],[12519,23,1],[12519,42,1],
[12519,69,1],[12519,71,1],[12520,23,1],[12520,30,1],[12521,1,1],
[12521,23,1],[12521,28,3],[12522,1,1],[12522,3,5],[12522,5,1],
[12522,13,3],[12522,18,1],[12522,21,1],[12522,22,26],[12522,23,18],
[12522,24,29],[12522,26,6],[12522,27,12],[12522,28,8],[12522,32,2],
[12522,33,3],[12522,35,7],[12522,38,1],[12522,42,5],[12522,43,4],
[12522,56,2],[12522,70,1],[12522,73,1],[12523,28,1],[12523,76,1],
[12524,1,1],[12524,5,1],[12524,6,1],[12524,20,1],[12524,21,1],
[12524,24,4],[12524,25,1],[12524,43,1],[12524,48,1],[12524,55,1],
[12524,59,1],[12524,63,1],[12524,65,1],[12524,70,1],[12524,77,1],
[12525,25,3],[12526,35,3],[12527,9,1],[12527,13,1],[12527,34,1],
[12527,44,2],[12527,50,1],[12527,53,1],[12527,55,1],[12527,66,2],
[12528,23,1],[12528,43,1],[12529,28,8],[12529,62,1],[12529,70,1],
[12529,74,1],[12530,0,1],[12530,7,5],[12530,11,1],[12530,12,1],
[12530,13,1],[12530,14,1],[12530,16,1],[12530,23,1],[12530,47,1],
[12530,62,1],[12530,64,1],[12530,77,1],[12531,3,2],[12531,20,1],
[12531,21,1],[12531,23,3],[12531,24,1],[12531,28,1],[12531,39,1],
[12531,42,3],[12531,43,1],[12531,47,3],[12531,62,2],[12532,25,6],
[12533,23,4],[12533,25,1],[12533,43,2],[12534,43,2],[12535,6,1],
[12535,50,1],[12535,66,1],[12536,1,1],[12536,20,1],[12536,29,3],
[12537,16,1],[12537,17,11],[12537,22,1],[12537,24,1],[12537,25,1],
[12537,28,1],[12537,30,2],[12537,34,2],[12537,50,2],[12537,53,21],
[12538,23,1],[12538,24,1],[12538,26,1],[12538,42,1],[12538,43,2],
[12539,23,1],[12539,27,1],[12539,33,2],[12539,62,1],[12540,44,1],
[12540,50,1],[12541,2,2],[12541,28,1],[12542,3,1],[12542,17,2],

[12542,23,1],[12542,24,1],[12542,27,2],[12542,28,1],[12542,53,3],
[12542,54,1],[12542,55,1],[12542,56,1],[12543,62,2],[12544,4,1],
[12544,5,2],[12544,23,3],[12544,24,2],[12544,28,1],[12544,33,1],
[12544,35,2],[12544,40,1],[12544,50,3],[12544,62,1],[12544,66,1],
[12544,74,2],[12545,1,8],[12545,5,1],[12545,55,1],[12546,23,1],
[12546,28,1],[12546,35,1],[12547,2,5],[12547,13,2],[12547,24,2],
[12547,26,1],[12547,28,2],[12547,31,1],[12547,32,2],[12548,1,1],
[12548,13,1],[12548,27,1],[12548,52,1],[12549,43,2],[12550,24,1],
[12550,40,1],[12550,44,1],[12550,50,1],[12550,66,1],[12551,28,1],
[12551,55,1],[12552,4,1],[12552,50,2],[12553,28,1],[12553,42,1],
[12553,44,1],[12554,1,2],[12554,2,1],[12554,13,2],[12554,27,1],
[12554,28,1],[12554,29,1],[12554,30,1],[12554,33,1],[12554,35,2],
[12554,42,2],[12554,54,1],[12554,55,1],[12554,60,1],[12554,63,1],
[12554,71,1],[12555,2,2],[12556,1,1],[12556,6,1],[12556,56,6],
[12556,66,1],[12557,3,1],[12557,32,1],[12557,42,1],[12558,4,1],
[12558,28,1],[12559,16,1],[12559,59,1],[12560,26,1],[12560,32,4],
[12560,35,1],[12561,1,3],[12562,24,1],[12562,35,1],[12562,51,1],
[12562,56,4],[12563,4,4],[12563,5,2],[12563,28,12],[12563,45,2],
[12563,50,4],[12563,68,1],[12563,77,1],[12564,1,5],[12564,2,8],
[12564,3,4],[12564,7,1],[12564,13,13],[12564,19,1],[12564,20,1],
[12564,22,1],[12564,23,2],[12564,25,1],[12564,27,3],[12564,30,1],
[12564,32,1],[12564,39,1],[12564,42,2],[12564,55,1],[12564,59,2],
[12565,27,7],[12565,28,2],[12565,35,1],[12565,47,1],[12565,62,1],
[12565,72,1],[12566,28,1],[12566,44,1],[12566,66,1],[12567,5,1],
[12567,28,2],[12568,2,9],[12568,3,5],[12568,4,1],[12568,5,8],
[12568,6,1],[12568,11,5],[12568,20,2],[12568,21,16],[12568,22,6],
[12568,23,37],[12568,24,15],[12568,25,53],[12568,26,16],[12568,27,20],
[12568,28,15],[12568,30,1],[12568,31,10],[12568,32,37],[12568,33,15],
[12568,35,16],[12568,37,10],[12568,38,1],[12568,39,4],[12568,40,6],
[12568,42,5],[12568,43,19],[12568,48,35],[12568,51,1],[12568,52,1],
[12568,56,24],[12568,59,3],[12568,60,1],[12568,61,1],[12568,63,3],
[12568,68,3],[12568,69,1],[12568,70,4],[12568,74,3],[12568,75,2],
[12568,76,2],[12568,78,1],[12569,23,3],[12569,25,3],[12569,26,2],
[12569,27,1],[12569,31,4],[12569,32,1],[12569,34,1],[12569,35,4],
[12569,51,1],[12569,55,1],[12570,11,2],[12571,9,3],[12571,12,2],
[12571,13,1],[12571,14,2],[12571,17,1],[12571,18,1],[12571,19,1],
[12571,20,2],[12571,34,1],[12571,37,2],[12571,41,1],[12571,46,1],
[12571,49,1],[12571,55,1],[12571,58,2],[12571,73,1],[12572,35,1],
[12572,44,1],[12573,19,1],[12573,20,4],[12573,22,1],[12573,23,13],
[12573,24,1],[12573,25,10],[12573,28,1],[12573,30,3],[12573,31,3],
[12573,32,7],[12573,39,7],[12573,42,2],[12573,45,1],[12573,65,1],
[12573,70,1],[12574,28,26],[12574,63,1],[12574,75,3],[12574,76,2],
[12575,38,1],[12575,68,2],[12576,20,2],[12577,21,1],[12577,26,1],
[12578,6,1],[12578,23,1],[12579,31,4],[12580,50,1],[12580,53,1],
[12581,23,1],[12581,28,1],[12581,42,1],[12581,47,2],[12581,66,1],
[12582,23,6],[12582,38,1],[12583,21,1],[12583,55,1],[12584,18,2],
[12584,67,1],[12585,24,1],[12585,28,3],[12585,31,14],[12585,35,1],
[12585,42,14],[12585,43,1],[12585,52,1],[12585,56,1],[12586,1,40],
[12586,7,1],[12586,8,2],[12586,12,2],[12586,13,1],[12586,29,2],
[12586,36,1],[12586,53,2],[12587,24,2],[12588,19,2],[12589,22,1],

[12589,28,1],[12589,33,3],[12589,47,2],[12590,27,1],[12590,56,1],
[12590,66,1],[12590,70,1],[12591,0,2],[12591,3,1],[12591,15,1],
[12591,23,1],[12591,30,1],[12591,39,1],[12591,47,1],[12592,24,1],
[12592,43,1],[12593,33,6],[12594,12,1],[12594,74,1],[12595,28,2],
[12595,32,1],[12596,46,1],[12596,54,1],[12597,22,1],[12597,26,2],
[12597,27,1],[12597,31,1],[12597,59,1],[12598,5,1],[12598,24,1],
[12598,27,1],[12599,70,2],[12600,28,1],[12600,35,1],[12600,48,1],
[12600,70,1],[12601,0,2],[12601,1,8],[12601,8,1],[12601,9,9],
[12601,10,6],[12601,12,4],[12601,13,1],[12601,14,3],[12601,15,1],
[12601,16,2],[12601,17,4],[12601,18,2],[12601,19,2],[12601,20,1],
[12601,36,1],[12601,37,2],[12601,39,2],[12601,41,5],[12601,45,10],
[12601,46,2],[12601,53,5],[12601,54,1],[12601,55,2],[12601,58,1],
[12601,59,1],[12601,64,1],[12601,65,1],[12601,67,1],[12601,71,1],
[12601,73,2],[12601,75,1],[12601,77,3],[12602,26,1],[12602,27,2],
[12603,17,1],[12603,53,1],[12604,3,1],[12604,22,1],[12604,23,2],
[12604,40,1],[12605,4,4],[12605,9,1],[12605,11,1],[12605,15,1],
[12605,17,5],[12605,24,1],[12605,28,31],[12605,30,1],[12605,50,2],
[12605,53,12],[12605,55,6],[12605,63,1],[12605,66,2],[12606,15,1],
[12606,51,1],[12606,59,1],[12606,73,1],[12607,4,9],[12607,8,1],
[12607,10,2],[12607,13,1],[12607,14,1],[12607,15,20],[12607,17,4],
[12607,18,1],[12607,26,1],[12607,32,1],[12607,40,2],[12607,50,4],
[12607,53,1],[12607,55,8],[12607,65,1],[12607,66,1],[12607,67,1],
[12607,73,1],[12608,15,11],[12608,17,1],[12608,53,4],[12608,55,2],
[12609,33,3],[12609,52,1],[12610,4,1],[12610,24,1],[12610,33,1],
[12610,44,25],[12610,50,2],[12610,66,10],[12611,3,1],[12611,20,2],
[12611,23,1],[12611,25,1],[12611,27,2],[12611,28,1],[12611,32,1],
[12611,33,1],[12611,35,1],[12611,43,2],[12612,17,1],[12612,37,1],
[12612,55,1],[12613,35,1],[12613,44,1],[12614,28,1],[12614,35,1],
[12615,10,1],[12615,15,5],[12615,32,1],[12615,55,4],[12616,15,4],
[12616,53,1],[12616,55,3],[12616,65,1],[12617,1,4],[12617,2,9],
[12617,3,4],[12617,6,2],[12617,8,1],[12617,9,2],[12617,12,2],
[12617,13,4],[12617,17,1],[12617,19,1],[12617,20,21],[12617,21,4],
[12617,22,3],[12617,23,4],[12617,24,9],[12617,25,1],[12617,26,3],
[12617,27,13],[12617,28,19],[12617,31,1],[12617,32,5],[12617,33,7],
[12617,34,1],[12617,35,9],[12617,37,1],[12617,38,1],[12617,41,1],
[12617,42,4],[12617,45,3],[12617,47,3],[12617,48,1],[12617,51,2],
[12617,53,1],[12617,54,1],[12617,56,2],[12617,59,1],[12617,62,3],
[12617,65,2],[12617,68,1],[12617,74,8],[12618,15,3],[12618,44,1],
[12618,50,2],[12618,53,1],[12618,55,3],[12618,66,2],[12619,52,1],
[12619,62,1],[12620,28,2],[12621,20,2],[12622,27,2],[12622,28,1],
[12622,35,1],[12622,57,1],[12623,30,1],[12623,31,2],[12624,22,1],
[12624,27,1],[12624,28,1],[12624,50,1],[12625,51,1],[12625,68,1],
[12626,33,2],[12627,22,1],[12627,31,1],[12628,2,10],[12629,56,1],
[12629,68,1],[12630,23,22],[12630,28,1],[12631,19,1],[12631,20,1],
[12632,1,1],[12632,2,24],[12632,3,2],[12632,4,1],[12632,5,1],
[12632,6,1],[12632,8,7],[12632,10,1],[12632,11,1],[12632,17,2],
[12632,22,2],[12632,23,4],[12632,24,1],[12632,25,1],[12632,28,1],
[12632,30,5],[12632,31,1],[12632,34,1],[12632,35,1],[12632,36,1],
[12632,41,1],[12632,42,1],[12632,43,2],[12632,50,1],[12632,61,1],
[12632,62,1],[12632,77,2],[12633,68,1],[12633,74,3],[12634,3,2],

[12634,4,4],[12634,5,3],[12634,6,1],[12634,9,1],[12634,10,1],
[12634,12,1],[12634,15,8],[12634,16,1],[12634,17,9],[12634,23,2],
[12634,24,1],[12634,27,1],[12634,28,2],[12634,32,1],[12634,33,1],
[12634,38,3],[12634,39,1],[12634,42,1],[12634,44,1],[12634,47,2],
[12634,48,9],[12634,50,1],[12634,52,3],[12634,53,11],[12634,55,6],
[12634,58,3],[12634,59,1],[12634,65,2],[12634,69,1],[12634,70,3],
[12634,71,2],[12634,77,1],[12635,22,2],[12635,35,1],[12636,2,1],
[12636,4,2],[12636,5,2],[12636,6,1],[12636,24,2],[12636,27,1],
[12636,32,1],[12636,52,1],[12636,55,1],[12636,63,1],[12636,65,1],
[12637,2,1],[12637,22,1],[12637,24,1],[12637,31,1],[12637,56,1],
[12637,61,34],[12637,62,2],[12637,74,1],[12638,25,2],[12638,27,2],
[12638,31,1],[12638,35,1],[12638,76,1],[12639,1,2],[12639,3,1],
[12639,4,2],[12639,17,1],[12639,20,70],[12639,21,1],[12639,22,19],
[12639,23,6],[12639,24,6],[12639,25,1],[12639,26,1],[12639,27,3],
[12639,28,2],[12639,29,1],[12639,31,1],[12639,35,5],[12639,38,1],
[12639,43,40],[12639,44,1],[12639,45,2],[12639,47,1],[12639,48,2],
[12639,55,3],[12639,59,3],[12639,65,1],[12639,71,1],[12639,74,1],
[12639,77,1],[12640,28,3],[12641,40,1],[12641,53,1],[12642,2,1],
[12642,3,2],[12642,5,1],[12642,23,1],[12642,26,1],[12642,32,1],
[12642,39,2],[12642,47,1],[12643,2,4],[12643,3,8],[12643,6,6],
[12643,13,5],[12643,14,1],[12643,21,1],[12643,22,3],[12643,23,34],
[12643,24,46],[12643,25,2],[12643,26,1],[12643,27,6],[12643,28,6],
[12643,30,1],[12643,31,2],[12643,32,2],[12643,33,1],[12643,35,4],
[12643,42,1],[12643,43,2],[12643,47,1],[12643,48,3],[12643,56,1],
[12643,70,1],[12643,76,2],[12644,5,1],[12644,22,1],[12644,28,12],
[12644,32,1],[12644,35,1],[12644,40,1],[12644,72,1],[12645,23,2],
[12645,33,1],[12646,2,2],[12646,31,4],[12647,10,1],[12647,11,2],
[12647,12,1],[12647,17,1],[12647,37,1],[12647,59,1],[12647,73,1],
[12648,28,44],[12648,63,1],[12648,75,1],[12649,11,1],[12649,67,1],
[12650,26,2],[12650,27,1],[12650,28,1],[12650,31,1],[12650,35,1],
[12651,38,1],[12651,66,1],[12652,27,1],[12652,28,1],[12653,4,6],
[12653,5,1],[12653,9,1],[12653,15,1],[12653,17,3],[12653,21,1],
[12653,28,11],[12653,50,5],[12653,53,2],[12654,0,1],[12654,9,1],
[12654,10,1],[12654,14,1],[12654,19,1],[12654,54,1],[12654,57,1],
[12654,65,1],[12655,23,3],[12655,28,1],[12655,42,1],[12656,28,2],
[12656,40,1],[12656,61,1],[12657,23,2],[12657,27,1],[12657,38,1],
[12658,28,2],[12659,40,1],[12659,69,1],[12659,70,1],[12660,6,1],
[12660,48,2],[12660,66,1],[12661,22,4],[12661,35,1],[12662,23,3],
[12662,28,1],[12662,39,1],[12663,1,3],[12664,50,1],[12664,66,1],
[12665,21,4],[12665,44,1],[12666,5,2],[12666,23,1],[12666,32,1],
[12666,38,2],[12666,40,1],[12666,55,1],[12666,60,1],[12666,62,2],
[12667,3,1],[12667,5,1],[12667,6,1],[12667,21,1],[12667,22,1],
[12667,23,2],[12667,25,1],[12667,26,4],[12667,27,16],[12667,28,3],
[12667,32,7],[12667,33,1],[12667,35,12],[12667,38,1],[12667,43,1],
[12667,44,2],[12667,50,1],[12667,66,1],[12668,23,1],[12668,24,2],
[12668,27,1],[12669,3,3],[12669,21,1],[12669,27,1],[12669,31,2],
[12669,32,2],[12669,33,1],[12669,35,1],[12669,38,2],[12669,40,2],
[12669,43,1],[12669,44,1],[12669,50,1],[12669,62,1],[12669,70,3],
[12669,74,1],[12669,75,1],[12669,76,1],[12670,2,1],[12670,26,1],
[12670,32,1],[12670,35,1],[12670,56,1],[12671,1,2],[12671,6,1],

[12671,13,5],[12671,17,1],[12671,19,2],[12671,20,1],[12671,22,1],
[12671,30,1],[12671,33,3],[12671,35,1],[12671,40,1],[12671,47,1],
[12671,53,1],[12671,55,1],[12671,56,3],[12671,66,1],[12671,67,1],
[12671,76,1],[12672,21,2],[12672,23,5],[12672,24,1],[12672,25,1],
[12672,26,1],[12673,22,1],[12673,31,1],[12673,70,1],[12673,79,6],
[12674,3,2],[12674,15,1],[12674,22,1],[12674,41,1],[12675,25,1],
[12675,28,3],[12675,39,4],[12676,32,2],[12677,5,2],[12677,23,1],
[12677,26,1],[12677,27,1],[12677,31,1],[12677,50,1],[12677,51,1],
[12677,56,1],[12677,70,4],[12677,79,1],[12678,8,1],[12678,43,1],
[12679,9,1],[12679,13,1],[12679,74,1],[12679,75,1],[12680,15,1],
[12680,26,1],[12680,33,1],[12680,35,2],[12680,55,1],[12680,66,1],
[12680,76,1],[12681,5,2],[12681,26,4],[12681,31,1],[12681,33,1],
[12681,43,2],[12682,16,1],[12682,37,1],[12682,77,1],[12683,3,1],
[12683,17,1],[12683,22,3],[12683,23,2],[12683,24,4],[12683,25,3],
[12683,26,1],[12683,27,11],[12683,28,1],[12683,31,3],[12683,32,2],
[12683,35,2],[12683,52,1],[12683,53,1],[12683,62,1],[12684,2,8],
[12684,3,3],[12685,19,1],[12685,25,2],[12685,50,1],[12686,44,2],
[12687,4,5],[12687,10,1],[12687,17,18],[12687,22,4],[12687,23,2],
[12687,24,7],[12687,25,1],[12687,27,4],[12687,30,4],[12687,31,1],
[12687,32,3],[12687,34,1],[12687,35,3],[12687,41,1],[12687,47,1],
[12687,50,6],[12687,53,13],[12688,38,1],[12688,66,1],[12689,17,1],
[12689,53,1],[12689,58,1],[12690,1,1],[12690,9,1],[12690,10,3],
[12690,12,1],[12690,13,3],[12690,14,1],[12690,16,1],[12690,17,1],
[12690,18,2],[12690,19,2],[12690,20,1],[12690,34,1],[12690,39,1],
[12690,41,2],[12690,45,2],[12690,46,1],[12690,49,1],[12690,53,1],
[12690,57,1],[12690,65,1],[12690,67,1],[12690,73,4],[12691,53,2],
[12692,2,1],[12692,22,1],[12692,35,2],[12693,12,2],[12694,2,3],
[12695,28,63],[12696,40,2],[12697,8,2],[12697,24,1],[12697,28,2],
[12697,35,2],[12697,38,1],[12697,52,6],[12697,70,1],[12697,74,1],
[12698,38,2],[12699,16,2],[12699,20,2],[12699,37,1],[12699,64,1],
[12700,5,3],[12700,35,5],[12701,4,1],[12701,9,1],[12701,15,1],
[12701,17,1],[12701,19,1],[12701,28,1],[12701,42,1],[12701,46,1],
[12701,52,1],[12701,55,1],[12701,66,1],[12702,2,4],[12703,7,1],
[12703,15,1],[12703,53,2],[12703,55,2],[12704,70,8],[12705,2,3],
[12705,28,1],[12706,24,1],[12706,27,1],[12706,47,1],[12707,15,10],
[12707,17,1],[12707,37,1],[12707,54,1],[12707,55,12],[12707,65,1],
[12707,71,1],[12708,1,1],[12708,28,2],[12708,30,1],[12708,35,1],
[12708,52,1],[12708,77,1],[12709,25,2],[12710,19,1],[12710,20,1],
[12710,65,1],[12711,28,1],[12711,44,1],[12711,51,1],[12712,2,69],
[12712,4,1],[12712,5,2],[12712,24,1],[12712,25,2],[12712,27,1],
[12712,43,1],[12712,50,1],[12712,70,1],[12712,79,1],[12713,24,1],
[12713,28,3],[12713,42,1],[12714,22,1],[12714,35,1],[12715,0,3],
[12715,8,5],[12715,12,2],[12715,17,1],[12715,55,1],[12716,8,1],
[12716,14,1],[12716,23,1],[12716,28,1],[12716,76,1],[12717,26,1],
[12717,33,2],[12718,16,1],[12718,23,1],[12719,4,1],[12719,22,1],
[12719,24,2],[12719,50,2],[12720,15,10],[12720,17,1],[12720,29,1],
[12720,55,3],[12720,65,1],[12721,2,1],[12721,28,1],[12721,56,1],
[12722,1,5],[12722,2,1],[12722,9,1],[12722,17,1],[12722,28,1],
[12722,31,1],[12723,25,1],[12723,28,1],[12723,40,1],[12723,48,1],
[12723,56,17],[12723,69,1],[12723,70,1],[12723,74,1],[12724,22,1],

[12724,48,1],[12725,9,1],[12725,20,1],[12725,23,1],[12725,24,3],
[12725,39,1],[12725,62,1],[12726,28,1],[12726,31,1],[12727,1,1],
[12727,4,1],[12727,5,2],[12727,9,2],[12727,11,1],[12727,22,1],
[12727,26,1],[12727,33,1],[12727,35,1],[12727,40,1],[12727,42,2],
[12727,44,1],[12727,68,1],[12728,15,1],[12728,53,2],[12729,2,3],
[12730,66,1],[12730,76,1],[12731,21,1],[12731,33,1],[12732,63,2],
[12733,8,1],[12733,31,1],[12733,32,1],[12734,47,2],[12734,51,1],
[12735,22,2],[12735,25,5],[12735,27,1],[12735,30,1],[12735,32,1],
[12735,33,9],[12735,35,5],[12735,38,1],[12735,39,1],[12735,52,3],
[12735,61,1],[12736,1,10],[12736,2,1],[12736,13,3],[12736,15,1],
[12736,23,1],[12736,24,2],[12736,27,1],[12736,29,4],[12736,30,1],
[12736,31,2],[12736,32,1],[12736,39,5],[12736,42,1],[12736,43,1],
[12736,46,3],[12736,47,2],[12736,55,1],[12736,59,1],[12736,70,2],
[12737,13,1],[12737,21,1],[12737,28,1],[12737,47,1],[12738,34,1],
[12738,36,1],[12739,11,1],[12739,37,1],[12740,34,1],[12740,37,4],
[12740,70,1],[12741,9,1],[12741,23,1],[12741,29,1],[12741,34,1],
[12741,57,1],[12742,22,1],[12742,47,2],[12742,76,2],[12743,28,1],
[12743,79,1],[12744,24,1],[12744,35,2],[12745,3,1],[12745,26,1],
[12745,28,12],[12745,32,1],[12745,43,1],[12745,44,1],[12745,52,7],
[12745,62,2],[12746,30,2],[12747,19,1],[12747,55,1],[12748,3,1],
[12748,22,1],[12748,43,2],[12749,2,1],[12749,3,2],[12749,31,1],
[12749,50,1],[12750,32,2],[12751,2,1],[12751,47,1],[12752,38,1],
[12752,44,4],[12752,66,2],[12753,39,2],[12754,1,2],[12754,10,1],
[12754,19,1],[12754,20,3],[12754,22,2],[12754,23,2],[12754,24,1],
[12754,29,1],[12754,35,2],[12754,39,1],[12754,41,1],[12754,42,1],
[12754,44,3],[12754,47,2],[12754,51,8],[12754,55,2],[12754,56,1],
[12754,66,1],[12754,70,1],[12754,73,1],[12754,76,1],[12755,25,2],
[12755,27,2],[12755,28,1],[12755,33,1],[12756,28,2],[12757,5,1],
[12757,52,1],[12758,3,3],[12758,4,1],[12758,13,1],[12758,19,1],
[12758,20,1],[12758,23,1],[12758,38,2],[12758,67,1],[12758,73,1],
[12759,23,1],[12759,25,1],[12760,23,1],[12760,39,1],[12761,47,2],
[12762,30,2],[12763,17,8],[12763,53,17],[12764,2,5],[12764,27,1],
[12765,13,1],[12765,20,2],[12765,33,2],[12765,35,4],[12766,33,2],
[12766,70,1],[12767,74,2],[12767,75,1],[12767,76,1],[12768,24,1],
[12768,25,2],[12768,26,2],[12768,27,1],[12768,32,1],[12768,35,2],
[12768,43,1],[12768,51,2],[12768,68,1],[12769,5,1],[12769,32,1],
[12769,74,1],[12770,8,1],[12770,26,1],[12770,43,1],[12771,22,1],
[12771,24,1],[12772,28,1],[12772,42,1],[12773,63,1],[12773,79,2],
[12774,38,2],[12775,23,2],[12775,28,1],[12776,13,1],[12776,23,5],
[12776,24,3],[12776,28,1],[12776,31,1],[12777,2,2],[12777,3,1],
[12777,22,1],[12777,24,3],[12777,26,1],[12777,27,1],[12777,28,21],
[12777,31,2],[12777,35,4],[12777,40,5],[12777,42,1],[12777,43,1],
[12777,44,1],[12777,50,3],[12777,52,1],[12777,56,3],[12777,62,1],
[12777,63,2],[12777,68,5],[12777,70,11],[12777,72,1],[12777,74,2],
[12777,76,4],[12777,79,1],[12778,15,1],[12778,38,1],[12779,27,1],
[12779,28,2],[12779,42,1],[12780,0,2],[12780,1,1],[12780,8,1],
[12780,9,3],[12780,10,7],[12780,11,2],[12780,13,2],[12780,14,2],
[12780,15,1],[12780,16,3],[12780,17,2],[12780,18,4],[12780,19,7],
[12780,20,2],[12780,23,1],[12780,29,1],[12780,36,1],[12780,41,6],
[12780,45,1],[12780,46,4],[12780,53,3],[12780,55,1],[12780,57,2],

[12780,58,2],[12780,65,5],[12780,67,1],[12780,71,1],[12780,73,3],
[12780,76,1],[12780,77,6],[12780,78,1],[12781,13,1],[12781,64,1],
[12782,23,1],[12782,27,1],[12782,35,15],[12782,44,1],[12783,24,7],
[12783,51,1],[12783,62,1],[12784,1,2],[12784,7,1],[12785,22,2],
[12786,25,2],[12786,42,1],[12786,43,2],[12787,2,2],[12788,24,1],
[12788,26,2],[12789,28,8],[12789,38,1],[12789,63,1],[12789,75,3],
[12789,76,1],[12790,24,3],[12791,6,1],[12791,13,1],[12791,20,1],
[12791,21,1],[12791,22,3],[12791,23,4],[12791,24,1],[12791,32,1],
[12791,33,1],[12791,42,3],[12791,43,1],[12791,52,1],[12791,59,1],
[12792,28,2],[12793,29,1],[12793,46,1],[12794,35,1],[12794,66,1],
[12795,30,2],[12795,50,1],[12795,51,1],[12795,62,1],[12796,23,1],
[12796,62,2],[12797,3,1],[12797,5,1],[12797,25,1],[12797,26,2],
[12797,27,1],[12797,28,6],[12797,31,1],[12797,38,12],[12797,40,1],
[12797,44,91],[12797,48,1],[12797,50,3],[12797,62,1],[12797,66,74],
[12797,75,1],[12797,76,2],[12798,9,1],[12798,57,1],[12799,15,1],
[12799,17,1],[12799,53,2],[12799,55,1],[12800,36,2],[12801,38,1],
[12801,44,1],[12801,66,1],[12802,1,2],[12802,7,1],[12802,8,1],
[12802,9,2],[12802,11,1],[12802,12,1],[12802,13,1],[12802,14,2],
[12802,15,2],[12802,16,2],[12802,18,2],[12802,19,1],[12802,34,2],
[12802,37,3],[12802,39,1],[12802,41,1],[12802,45,5],[12802,46,1],
[12802,49,1],[12802,54,1],[12802,60,1],[12802,65,3],[12802,67,1],
[12802,73,2],[12802,77,2],[12803,0,2],[12803,1,2],[12803,2,3],
[12803,3,10],[12803,5,6],[12803,6,1],[12803,8,1],[12803,10,3],
[12803,12,8],[12803,13,12],[12803,15,1],[12803,17,1],[12803,19,1],
[12803,20,37],[12803,21,8],[12803,22,10],[12803,23,52],[12803,24,7],
[12803,25,10],[12803,26,26],[12803,27,27],[12803,28,16],[12803,31,29],
[12803,32,41],[12803,33,9],[12803,34,1],[12803,35,12],[12803,36,5],
[12803,37,3],[12803,38,2],[12803,39,8],[12803,41,1],[12803,42,13],
[12803,43,17],[12803,44,1],[12803,46,1],[12803,47,7],[12803,48,6],
[12803,56,13],[12803,57,1],[12803,58,1],[12803,59,11],[12803,61,1],
[12803,62,3],[12803,65,2],[12803,69,1],[12803,70,2],[12803,71,1],
[12803,78,1],[12804,3,2],[12805,4,1],[12805,22,1],[12805,23,1],
[12805,24,1],[12805,32,1],[12805,43,1],[12805,51,1],[12805,66,1],
[12806,54,1],[12806,65,1],[12806,71,1],[12807,28,1],[12807,31,1],
[12808,52,1],[12808,58,1],[12809,2,1],[12809,32,1],[12810,12,1],
[12810,21,1],[12810,23,1],[12810,24,3],[12810,26,1],[12810,27,1],
[12810,28,7],[12810,30,1],[12810,50,1],[12811,2,6],[12811,3,6],
[12811,4,2],[12811,5,3],[12811,6,3],[12811,21,2],[12811,22,1],
[12811,23,3],[12811,24,4],[12811,25,6],[12811,26,3],[12811,27,5],
[12811,28,34],[12811,31,7],[12811,32,2],[12811,33,1],[12811,35,2],
[12811,38,3],[12811,40,2],[12811,42,2],[12811,43,1],[12811,44,5],
[12811,47,2],[12811,50,3],[12811,52,1],[12811,56,4],[12811,62,2],
[12811,63,2],[12811,66,3],[12811,70,10],[12811,74,8],[12811,75,2],
[12811,76,4],[12812,2,1],[12812,27,2],[12812,42,1],[12812,72,1],
[12812,74,1],[12813,24,1],[12813,47,1],[12813,61,1],[12814,24,1],
[12814,28,2],[12814,35,1],[12815,1,1],[12815,23,1],[12816,5,5],
[12816,6,2],[12816,27,1],[12816,32,1],[12816,35,1],[12816,40,2],
[12816,43,1],[12816,44,3],[12816,51,1],[12816,52,1],[12816,60,1],
[12816,62,1],[12816,66,1],[12816,70,2],[12816,72,1],[12817,12,1],
[12817,29,1],[12817,54,1],[12818,27,1],[12818,28,1],[12819,28,4],

[12820,5,1],[12820,23,1],[12821,33,13],[12822,1,1],[12822,23,1],
[12822,35,1],[12823,20,1],[12823,22,1],[12824,26,1],[12824,38,1],
[12825,9,1],[12825,41,1],[12825,71,1],[12826,28,2],[12826,32,1],
[12826,48,1],[12827,28,1],[12827,52,12],[12828,17,1],[12828,20,1],
[12829,19,1],[12829,30,3],[12830,5,1],[12830,22,3],[12830,23,1],
[12830,27,5],[12830,28,5],[12830,33,1],[12830,35,4],[12831,28,1],
[12831,45,1],[12832,3,1],[12832,28,1],[12832,42,1],[12832,59,1],
[12833,1,1],[12833,20,2],[12834,3,1],[12834,20,1],[12834,26,1],
[12835,22,1],[12835,27,1],[12836,19,1],[12836,35,1],[12837,1,4],
[12837,3,3],[12837,5,1],[12837,13,1],[12837,22,2],[12837,23,6],
[12837,24,1],[12837,25,3],[12837,26,1],[12837,31,1],[12837,32,1],
[12837,47,1],[12837,78,1],[12838,2,2],[12839,3,1],[12839,23,1],
[12839,25,1],[12839,26,1],[12839,27,2],[12839,37,1],[12839,63,1],
[12840,10,2],[12840,49,1],[12840,65,1],[12841,2,1],[12841,5,1],
[12841,13,1],[12841,20,4],[12841,21,1],[12841,22,1],[12841,24,1],
[12841,37,3],[12841,42,1],[12841,43,1],[12841,48,1],[12842,20,2],
[12842,21,1],[12842,54,1],[12843,3,1],[12843,6,1],[12843,19,1],
[12843,20,1],[12843,23,2],[12843,24,2],[12843,28,1],[12843,32,4],
[12843,37,1],[12843,43,1],[12844,25,2],[12844,70,1],[12845,32,2],
[12846,23,2],[12847,5,1],[12847,23,1],[12847,26,3],[12847,31,1],
[12847,32,1],[12847,39,1],[12847,43,1],[12848,24,1],[12848,32,6],
[12849,15,1],[12849,44,3],[12849,66,1],[12850,28,3],[12851,25,2],
[12851,27,1],[12851,32,1],[12851,33,1],[12851,42,1],[12851,47,1],
[12852,33,1],[12852,48,1],[12852,56,4],[12853,22,1],[12853,27,1],
[12853,35,1],[12854,1,3],[12854,9,3],[12854,10,4],[12854,12,1],
[12854,15,1],[12854,16,1],[12854,19,4],[12854,20,1],[12854,30,2],
[12854,34,3],[12854,36,1],[12854,37,1],[12854,45,1],[12854,46,1],
[12854,54,1],[12854,58,1],[12854,59,5],[12854,65,3],[12854,71,1],
[12854,73,1],[12854,77,1],[12855,1,2],[12855,13,3],[12855,26,3],
[12855,29,1],[12856,32,4],[12857,3,2],[12857,24,1],[12858,3,1],
[12858,14,1],[12858,73,1],[12859,24,1],[12859,28,1],[12860,5,5],
[12860,6,1],[12860,15,3],[12860,21,3],[12860,22,1],[12860,25,1],
[12860,30,2],[12860,31,2],[12860,40,2],[12860,42,1],[12860,43,1],
[12860,47,1],[12860,51,1],[12860,52,1],[12860,55,1],[12860,63,1],
[12860,69,1],[12860,70,1],[12860,72,2],[12860,74,1],[12860,75,3],
[12861,21,1],[12861,27,1],[12861,33,1],[12861,35,1],[12861,48,1],
[12861,50,1],[12861,63,1],[12861,74,2],[12862,26,1],[12862,31,1],
[12862,32,1],[12862,66,1],[12863,3,1],[12863,25,1],[12863,27,1],
[12863,33,1],[12863,35,2],[12863,62,1],[12864,1,2],[12864,13,1],
[12864,39,2],[12865,3,2],[12865,22,1],[12865,24,2],[12865,25,1],
[12865,26,1],[12865,28,3],[12865,35,1],[12865,38,1],[12865,47,1],
[12865,56,4],[12865,68,1],[12865,69,1],[12865,70,1],[12865,74,2],
[12866,51,1],[12866,69,2],[12867,5,1],[12867,21,1],[12868,31,2],
[12869,3,1],[12869,62,1],[12870,27,1],[12870,28,1],[12871,3,1],
[12871,28,1],[12872,2,1],[12872,3,2],[12872,4,1],[12872,20,1],
[12872,22,1],[12872,23,1],[12872,26,1],[12872,32,2],[12872,56,1],
[12873,1,6],[12873,8,1],[12873,12,1],[12873,17,3],[12873,20,3],
[12873,29,3],[12873,30,2],[12873,34,2],[12873,36,1],[12873,37,2],
[12873,39,3],[12873,53,2],[12873,55,2],[12873,73,2],[12873,78,1],
[12874,2,2],[12875,21,1],[12875,27,1],[12875,32,1],[12875,55,1],

[12875,70,1],[12876,2,1],[12876,28,1],[12877,15,1],[12877,39,1],
[12878,32,2],[12879,28,1],[12879,33,1],[12879,43,1],[12880,1,8],
[12880,20,1],[12880,28,1],[12880,32,1],[12880,53,1],[12880,62,1],
[12881,1,1],[12881,13,2],[12881,15,2],[12881,20,6],[12881,23,1],
[12881,24,1],[12881,77,1],[12882,2,1],[12882,22,1],[12882,23,1],
[12882,25,3],[12882,27,1],[12882,31,4],[12882,32,1],[12882,35,1],
[12882,52,1],[12883,0,2],[12883,1,3],[12883,8,1],[12883,9,1],
[12883,13,3],[12883,20,5],[12883,34,1],[12883,59,15],[12884,1,1],
[12884,2,2],[12884,3,64],[12884,4,3],[12884,5,7],[12884,6,36],
[12884,9,1],[12884,10,1],[12884,14,2],[12884,18,1],[12884,21,10],
[12884,22,10],[12884,23,10],[12884,24,22],[12884,25,45],[12884,26,24],
[12884,27,1],[12884,28,11],[12884,29,5],[12884,30,5],[12884,31,9],
[12884,32,22],[12884,33,118],[12884,35,3],[12884,37,25],[12884,38,10],
[12884,39,8],[12884,40,5],[12884,41,1],[12884,43,3],[12884,45,2],
[12884,46,5],[12884,47,23],[12884,48,93],[12884,50,2],[12884,52,4],
[12884,56,34],[12884,58,1],[12884,59,1],[12884,60,5],[12884,61,11],
[12884,62,1],[12884,63,2],[12884,64,4],[12884,65,2],[12884,66,3],
[12884,67,1],[12884,68,3],[12884,69,3],[12884,70,20],[12884,72,1],
[12884,73,1],[12884,74,4],[12884,75,2],[12884,76,3],[12884,78,6],
[12885,1,1],[12885,9,2],[12885,41,1],[12885,45,1],[12885,64,1],
[12885,73,1],[12886,32,5],[12887,75,1],[12887,76,2],[12888,37,1],
[12888,39,1],[12889,28,1],[12889,66,1],[12890,2,4],[12890,3,1],
[12890,4,1],[12890,5,2],[12890,9,1],[12890,12,5],[12890,16,1],
[12890,19,1],[12890,20,4],[12890,21,1],[12890,22,1],[12890,23,5],
[12890,24,2],[12890,26,1],[12890,28,7],[12890,32,1],[12890,33,1],
[12890,43,1],[12890,45,1],[12890,47,1],[12890,50,1],[12890,68,1],
[12891,16,1],[12891,19,1],[12892,27,1],[12892,43,3],[12893,9,1],
[12893,15,1],[12893,17,2],[12893,59,1],[12894,2,1],[12894,23,2],
[12894,43,3],[12895,2,9],[12896,1,1],[12896,9,1],[12896,15,1],
[12896,19,1],[12896,29,1],[12896,30,1],[12897,28,2],[12897,50,2],
[12897,66,3],[12898,1,2],[12898,2,1],[12898,13,2],[12898,24,3],
[12898,26,2],[12898,39,1],[12899,3,1],[12899,27,1],[12900,26,1],
[12900,31,2],[12900,39,2],[12900,43,2],[12901,24,1],[12901,28,2],
[12901,40,1],[12902,3,1],[12902,24,1],[12902,42,1],[12902,43,2],
[12903,25,2],[12903,26,1],[12903,28,1],[12903,31,1],[12903,33,1],
[12904,30,3],[12905,9,2],[12905,11,1],[12905,15,1],[12905,28,2],
[12905,40,1],[12905,50,1],[12905,71,1],[12906,1,1],[12906,20,1],
[12907,3,2],[12907,5,1],[12907,10,1],[12907,30,1],[12907,31,1],
[12907,40,1],[12907,46,1],[12907,56,1],[12907,59,2],[12907,72,2],
[12908,1,1],[12908,10,1],[12908,32,1],[12909,25,3],[12910,1,1],
[12910,3,1],[12910,4,1],[12910,5,1],[12910,9,1],[12910,11,1],
[12910,13,4],[12910,15,1],[12910,20,2],[12910,23,7],[12910,25,2],
[12910,27,2],[12910,28,1],[12910,32,3],[12910,43,2],[12910,58,1],
[12910,62,1],[12910,72,1],[12911,2,1],[12911,4,1],[12911,5,9],
[12911,8,1],[12911,11,1],[12911,21,2],[12911,23,1],[12911,25,1],
[12911,28,107],[12911,35,2],[12911,36,1],[12911,38,3],[12911,40,1],
[12911,42,1],[12911,43,5],[12911,44,1],[12911,52,4],[12911,53,1],
[12911,56,1],[12911,59,12],[12911,61,1],[12911,62,2],[12911,68,1],
[12911,69,1],[12911,72,1],[12911,76,1],[12911,77,1],[12912,2,1],
[12912,40,1],[12912,68,2],[12912,70,1],[12913,5,11],[12913,22,7],

[12913,24,1],[12913,42,2],[12913,52,2],[12914,27,2],[12914,44,1],
[12914,62,1],[12915,1,1],[12915,2,4],[12915,3,1],[12915,7,1],
[12915,10,1],[12915,13,1],[12915,19,3],[12915,20,4],[12915,24,1],
[12915,27,3],[12915,31,4],[12915,34,1],[12915,35,1],[12915,39,1],
[12915,46,1],[12915,60,1],[12915,62,4],[12915,77,1],[12915,78,1],
[12916,21,1],[12916,26,1],[12917,28,1],[12917,52,1],[12918,24,7],
[12918,46,1],[12918,66,1],[12919,22,2],[12919,23,6],[12920,35,2],
[12921,34,1],[12921,56,1],[12921,66,1],[12922,27,7],[12922,35,1],
[12923,6,1],[12923,27,1],[12924,4,1],[12924,5,1],[12924,6,1],
[12924,10,1],[12924,11,1],[12924,13,1],[12924,15,1],[12924,20,1],
[12924,22,7],[12924,24,10],[12924,26,2],[12924,27,1],[12924,28,1],
[12924,30,1],[12924,33,2],[12924,37,1],[12924,38,1],[12924,40,1],
[12924,42,2],[12924,47,13],[12924,50,3],[12924,56,1],[12924,59,1],
[12924,66,1],[12924,69,1],[12924,72,1],[12924,74,1],[12925,0,1],
[12925,3,4],[12925,17,1],[12925,23,1],[12925,26,1],[12925,43,1],
[12925,53,5],[12925,56,2],[12925,66,1],[12926,43,1],[12926,56,2],
[12927,23,2],[12927,24,2],[12927,35,2],[12928,2,3],[12928,23,1],
[12928,31,1],[12929,23,1],[12929,25,1],[12929,28,1],[12929,43,1],
[12930,22,1],[12930,25,2],[12930,28,1],[12930,32,1],[12931,35,3],
[12931,42,10],[12931,44,1],[12931,48,1],[12932,4,1],[12932,25,1],
[12932,31,2],[12932,35,1],[12932,44,1],[12932,53,1],[12932,55,1],
[12932,62,1],[12933,1,5],[12933,12,2],[12933,13,1],[12933,29,3],
[12933,34,1],[12933,55,1],[12933,59,1],[12933,73,8],[12934,0,1],
[12934,5,1],[12934,10,3],[12934,16,1],[12934,18,1],[12934,25,1],
[12934,31,2],[12934,34,1],[12934,37,1],[12934,41,5],[12934,43,1],
[12934,65,1],[12934,66,1],[12934,67,1],[12934,68,1],[12934,69,1],
[12934,70,4],[12934,71,1],[12934,72,1],[12934,75,1],[12934,76,1],
[12935,3,1],[12935,6,1],[12935,23,2],[12935,27,1],[12935,40,1],
[12935,50,1],[12935,68,1],[12935,70,1],[12935,74,2],[12936,22,4],
[12936,23,1],[12936,24,6],[12936,25,1],[12936,47,1],[12936,61,1],
[12936,62,4],[12937,1,3],[12937,7,1],[12937,8,1],[12937,20,2],
[12937,28,12],[12937,39,1],[12937,71,1],[12937,75,1],[12938,17,1],
[12938,28,1],[12938,53,1],[12938,55,1],[12938,68,1],[12939,28,1],
[12939,43,1],[12940,25,2],[12940,27,1],[12940,28,1],[12940,56,1],
[12941,2,8],[12942,25,1],[12942,27,1],[12942,55,1],[12943,3,1],
[12943,5,1],[12943,23,1],[12943,27,2],[12943,32,1],[12944,24,2],
[12944,27,8],[12944,28,3],[12944,33,16],[12944,34,1],[12944,35,3],
[12945,2,7],[12945,23,1],[12945,31,1],[12945,39,1],[12946,0,3],
[12947,15,1],[12947,22,1],[12948,31,3],[12948,39,1],[12949,22,1],
[12949,28,1],[12949,47,1],[12950,1,1],[12950,10,1],[12950,60,2],
[12951,3,1],[12951,5,1],[12951,6,2],[12951,24,3],[12951,28,1],
[12951,31,1],[12952,23,1],[12952,24,2],[12952,28,1],[12952,38,1],
[12952,42,1],[12952,47,1],[12952,56,1],[12952,68,1],[12952,74,1],
[12953,27,1],[12953,28,1],[12953,41,1],[12953,60,1],[12954,10,1],
[12954,11,1],[12954,12,1],[12954,17,1],[12954,19,2],[12954,20,1],
[12954,36,1],[12954,45,2],[12954,55,2],[12954,76,1],[12955,1,1],
[12955,3,2],[12955,13,2],[12955,20,1],[12955,23,2],[12955,24,7],
[12955,26,1],[12955,27,6],[12955,31,2],[12955,33,1],[12955,34,1],
[12955,39,1],[12955,43,3],[12956,15,1],[12956,23,1],[12956,24,2],
[12956,28,4],[12956,44,1],[12956,50,1],[12956,55,1],[12956,74,2],

[12957,3,2],[12958,60,2],[12959,5,6],[12959,13,1],[12959,18,1],
[12959,20,1],[12959,21,2],[12959,22,2],[12959,23,5],[12959,24,1],
[12959,26,14],[12959,28,3],[12959,31,4],[12959,32,7],[12959,33,1],
[12959,35,1],[12959,42,2],[12959,43,23],[12959,44,1],[12959,48,1],
[12959,66,1],[12960,22,1],[12960,39,1],[12960,52,1],[12961,38,1],
[12961,66,1],[12962,56,2],[12963,1,2],[12963,29,1],[12964,3,3],
[12964,28,1],[12964,68,1],[12964,70,1],[12965,32,2],[12966,2,2],
[12967,31,2],[12968,28,1],[12968,31,4],[12969,18,1],[12969,19,2],
[12969,41,1],[12969,45,1],[12969,58,1],[12969,65,1],[12969,67,1],
[12970,28,1],[12970,43,1],[12971,12,1],[12971,16,1],[12971,46,1],
[12971,58,1],[12972,6,1],[12972,28,1],[12972,47,1],[12973,1,1],
[12973,12,1],[12973,20,1],[12973,43,1],[12974,28,1],[12974,31,5],
[12974,33,1],[12974,42,4],[12975,31,1],[12975,42,5],[12975,43,3],
[12976,12,1],[12976,29,1],[12977,2,1],[12977,23,1],[12977,31,1],
[12977,34,1],[12977,42,1],[12978,3,1],[12978,22,1],[12978,47,1],
[12979,20,2],[12979,22,1],[12980,2,1],[12980,3,1],[12980,13,1],
[12980,20,4],[12980,31,1],[12981,1,1],[12981,20,1],[12982,25,1],
[12982,28,1],[12983,21,1],[12983,28,1],[12984,8,1],[12984,22,1],
[12985,30,2],[12986,7,1],[12986,62,1],[12987,7,1],[12987,65,1],
[12988,17,1],[12988,53,1],[12989,10,1],[12989,28,1],[12990,19,1],
[12990,40,1],[12990,65,1],[12991,28,1],[12991,31,1],[12992,27,5],
[12992,28,1],[12992,35,3],[12993,29,6],[12994,2,2],[12994,54,1],
[12995,10,1],[12995,21,1],[12996,28,4],[12997,41,3],[12998,1,1],
[12998,13,1],[12998,15,1],[12998,44,2],[12998,50,1],[12999,3,1],
[12999,5,1],[12999,20,1],[12999,23,2],[12999,27,1],[12999,28,4],
[13000,28,1],[13000,53,1],[13000,75,1],[13001,1,1],[13001,15,1],
[13001,17,1],[13001,20,6],[13001,24,2],[13001,35,3],[13001,47,1],
[13001,51,2],[13001,68,2],[13001,69,1],[13002,2,4],[13003,5,1],
[13003,37,1],[13003,38,1],[13003,78,1],[13004,2,1],[13004,30,1],
[13005,3,5],[13005,5,4],[13005,24,1],[13005,25,1],[13005,35,2],
[13005,38,1],[13005,42,1],[13005,47,3],[13005,56,2],[13005,66,2],
[13005,68,1],[13005,70,1],[13006,6,1],[13006,56,1],[13006,77,1],
[13007,34,1],[13007,46,1],[13008,12,1],[13008,19,1],[13008,65,1],
[13008,73,1],[13008,78,1],[13009,28,3],[13010,3,1],[13010,24,1],
[13011,24,1],[13011,26,1],[13011,28,1],[13012,2,3],[13013,15,1],
[13013,55,1],[13014,1,2],[13014,13,1],[13014,15,2],[13014,16,2],
[13014,17,1],[13014,20,1],[13014,55,1],[13014,71,1],[13014,73,1],
[13015,20,1],[13015,27,1],[13015,32,4],[13016,51,1],[13016,61,1],
[13017,1,1],[13017,7,1],[13017,28,2],[13017,39,1],[13017,41,1],
[13017,54,1],[13017,67,1],[13018,3,1],[13018,6,1],[13018,15,3],
[13018,17,1],[13018,23,2],[13018,24,8],[13018,25,1],[13018,26,3],
[13018,27,3],[13018,33,4],[13018,42,4],[13018,43,1],[13018,47,1],
[13018,48,5],[13018,50,1],[13018,53,1],[13018,55,1],[13018,56,11],
[13018,70,1],[13019,56,3],[13019,74,1],[13020,2,1],[13020,3,1],
[13020,4,1],[13020,18,5],[13020,19,2],[13020,21,1],[13020,24,2],
[13020,25,2],[13020,26,2],[13020,27,12],[13020,31,1],[13020,32,8],
[13020,35,4],[13020,37,2],[13020,40,2],[13020,42,5],[13020,46,1],
[13020,47,2],[13020,48,2],[13020,55,2],[13020,56,16],[13020,61,8],
[13020,62,1],[13020,63,1],[13020,66,1],[13020,70,13],[13021,2,1],
[13021,28,2],[13021,35,3],[13021,43,1],[13021,59,1],[13021,63,1],

[13022,2,2],[13022,30,1],[13022,72,1],[13023,28,1],[13023,56,1],
[13024,11,1],[13024,19,1],[13024,24,1],[13024,35,1],[13024,41,1],
[13024,48,1],[13024,63,7],[13024,75,2],[13024,76,5],[13025,2,1],
[13025,27,2],[13025,62,2],[13025,76,1],[13026,7,1],[13026,24,1],
[13026,42,11],[13026,43,2],[13026,53,1],[13026,60,1],[13026,61,1],
[13027,23,1],[13027,28,1],[13028,1,3],[13029,21,1],[13029,25,2],
[13030,26,2],[13030,28,2],[13031,1,1],[13031,34,1],[13031,73,1],
[13032,31,2],[13033,28,3],[13033,36,1],[13034,22,2],[13034,23,3],
[13034,24,2],[13034,25,2],[13034,27,1],[13034,31,2],[13034,35,3],
[13035,1,3],[13035,13,3],[13035,15,1],[13035,19,1],[13035,20,6],
[13035,34,2],[13035,36,1],[13035,39,1],[13035,46,1],[13035,58,1],
[13035,59,4],[13035,60,1],[13035,64,2],[13035,78,1],[13036,68,5],
[13036,72,1],[13037,3,1],[13037,24,1],[13037,25,3],[13037,28,1],
[13037,32,1],[13038,2,1],[13038,3,1],[13038,5,2],[13038,8,1],
[13038,13,1],[13038,20,2],[13038,21,1],[13038,25,4],[13038,27,6],
[13038,28,3],[13038,32,5],[13038,35,2],[13038,56,1],[13038,63,1],
[13038,67,1],[13038,75,1],[13039,8,1],[13039,19,1],[13039,22,1],
[13039,25,1],[13039,28,1],[13039,30,3],[13039,32,1],[13039,33,12],
[13039,35,1],[13039,38,1],[13039,45,1],[13039,52,7],[13039,63,1],
[13039,70,1],[13040,1,11],[13040,7,5],[13040,8,2],[13040,10,1],
[13040,12,1],[13040,13,1],[13040,17,1],[13040,34,1],[13040,53,1],
[13040,74,1],[13041,0,1],[13041,8,1],[13041,9,5],[13041,10,7],
[13041,11,1],[13041,12,2],[13041,16,5],[13041,18,1],[13041,19,3],
[13041,29,1],[13041,30,4],[13041,34,3],[13041,36,1],[13041,37,2],
[13041,39,1],[13041,41,1],[13041,45,1],[13041,46,2],[13041,54,2],
[13041,57,2],[13041,58,1],[13041,65,3],[13041,67,1],[13041,71,1],
[13041,73,4],[13041,77,1],[13042,1,2],[13042,13,1],[13042,24,1],
[13042,31,1],[13042,66,1],[13043,4,1],[13043,22,1],[13043,31,1],
[13043,32,1],[13044,0,4],[13044,1,45],[13044,2,20],[13044,3,13],
[13044,5,7],[13044,6,6],[13044,7,6],[13044,8,44],[13044,9,7],
[13044,10,7],[13044,11,21],[13044,12,189],[13044,13,25],[13044,14,1],
[13044,15,3],[13044,16,21],[13044,17,1],[13044,18,2],[13044,19,8],
[13044,20,9],[13044,21,4],[13044,22,9],[13044,23,20],[13044,24,38],
[13044,25,47],[13044,26,24],[13044,27,23],[13044,28,15],[13044,29,37],
[13044,30,9],[13044,31,18],[13044,32,33],[13044,33,50],[13044,35,29],
[13044,37,15],[13044,38,2],[13044,39,61],[13044,40,27],[13044,41,4],
[13044,42,25],[13044,43,19],[13044,44,3],[13044,45,3],[13044,46,2],
[13044,47,6],[13044,48,29],[13044,50,4],[13044,51,5],[13044,52,2],
[13044,53,4],[13044,54,3],[13044,55,3],[13044,56,13],[13044,57,6],
[13044,58,5],[13044,59,15],[13044,60,27],[13044,61,2],[13044,62,3],
[13044,63,2],[13044,64,9],[13044,65,4],[13044,66,2],[13044,67,4],
[13044,68,1],[13044,69,2],[13044,70,26],[13044,71,2],[13044,72,2],
[13044,73,1],[13044,74,5],[13044,75,2],[13044,77,3],[13044,78,5],
[13045,10,1],[13045,54,1],[13045,77,1],[13046,41,1],[13046,65,1],
[13047,2,2],[13047,25,3],[13047,56,1],[13047,66,1],[13048,5,1],
[13048,20,1],[13048,23,1],[13048,24,1],[13048,25,1],[13048,26,2],
[13048,30,1],[13048,31,2],[13048,35,1],[13048,43,1],[13048,72,1],
[13049,30,1],[13049,55,1],[13050,20,1],[13050,27,1],[13050,37,3],
[13050,39,1],[13050,40,1],[13050,60,1],[13050,64,3],[13050,74,1],
[13051,2,2],[13051,28,1],[13051,43,1],[13052,20,1],[13052,24,1],

[13052,26,2],[13052,27,2],[13052,32,1],[13052,33,1],[13052,34,2],
[13052,40,1],[13052,55,1],[13052,59,4],[13052,76,1],[13053,9,1],
[13053,22,1],[13053,34,1],[13053,39,1],[13053,53,1],[13054,2,3],
[13055,2,1],[13055,24,1],[13056,24,1],[13056,56,1],[13057,43,2],
[13058,25,1],[13058,47,1],[13058,63,12],[13058,70,1],[13058,74,7],
[13058,75,8],[13058,76,10],[13059,44,4],[13059,50,1],[13059,66,8],
[13060,4,1],[13060,5,1],[13060,24,2],[13060,66,1],[13061,5,1],
[13061,27,2],[13061,55,1],[13062,21,2],[13062,31,3],[13062,33,1],
[13063,63,3],[13063,74,1],[13063,75,1],[13063,76,4],[13064,32,3],
[13064,33,2],[13064,39,1],[13064,42,1],[13065,31,1],[13065,62,2],
[13066,2,1],[13066,10,1],[13066,26,1],[13066,34,1],[13066,45,1],
[13067,9,3],[13067,28,1],[13068,15,1],[13068,34,1],[13069,28,1],
[13069,72,5],[13070,10,1],[13070,17,1],[13070,20,2],[13070,58,1],
[13071,20,2],[13071,39,1],[13071,50,1],[13071,58,1],[13071,70,1],
[13071,72,2],[13071,78,1],[13072,21,3],[13073,23,1],[13073,24,1],
[13073,28,3],[13074,24,1],[13074,68,1],[13075,21,2],[13075,26,1],
[13075,31,5],[13075,33,5],[13075,43,3],[13076,2,1],[13076,22,1],
[13077,1,2],[13077,3,6],[13077,23,15],[13077,24,48],[13077,28,8],
[13077,32,1],[13077,33,2],[13077,47,3],[13078,30,1],[13078,63,1],
[13079,28,1],[13079,44,1],[13079,75,1],[13080,2,10],[13081,2,42],
[13081,28,1],[13081,66,1],[13082,25,3],[13083,22,1],[13083,44,1],
[13084,28,3],[13085,28,1],[13085,70,1],[13086,26,1],[13086,43,1],
[13087,23,1],[13087,25,1],[13087,40,1],[13087,56,4],[13088,2,1],
[13088,4,3],[13088,28,6],[13088,31,1],[13088,38,14],[13088,44,50],
[13088,50,1],[13088,56,1],[13088,62,2],[13088,66,42],[13088,68,2],
[13088,70,2],[13088,72,1],[13089,2,10],[13089,27,2],[13090,28,17],
[13090,76,1],[13091,12,1],[13091,35,1],[13092,9,1],[13092,11,3],
[13092,19,1],[13092,55,1],[13093,62,2],[13094,1,1],[13094,2,1],
[13094,5,2],[13094,9,2],[13094,10,1],[13094,13,2],[13094,15,1],
[13094,19,1],[13094,20,26],[13094,22,3],[13094,23,6],[13094,24,8],
[13094,27,2],[13094,28,2],[13094,30,1],[13094,32,8],[13094,34,3],
[13094,35,4],[13094,36,1],[13094,37,1],[13094,38,1],[13094,39,4],
[13094,42,2],[13094,43,2],[13094,45,1],[13094,47,1],[13094,49,1],
[13094,52,2],[13094,55,1],[13094,59,4],[13094,65,1],[13094,72,1],
[13095,6,1],[13095,13,2],[13095,20,1],[13095,22,1],[13095,27,1],
[13095,35,1],[13095,43,2],[13095,56,1],[13095,75,1],[13095,76,1],
[13096,20,1],[13096,26,2],[13096,56,1],[13097,31,6],[13097,39,1],
[13098,21,1],[13098,24,2],[13098,42,1],[13099,28,1],[13099,62,1],
[13100,9,1],[13100,54,1],[13100,55,1],[13100,59,1],[13100,77,1],
[13101,28,4],[13102,3,10],[13102,22,1],[13102,23,12],[13102,24,12],
[13102,25,3],[13102,26,1],[13102,27,10],[13102,28,5],[13102,31,6],
[13102,32,6],[13102,33,1],[13102,35,1],[13102,38,1],[13102,42,7],
[13102,43,1],[13102,48,6],[13102,56,7],[13102,62,3],[13102,75,3],
[13103,5,1],[13103,6,1],[13103,8,1],[13103,16,1],[13103,20,2],
[13103,22,2],[13103,23,1],[13103,35,1],[13103,37,1],[13103,39,2],
[13103,53,1],[13103,56,1],[13103,64,1],[13104,25,1],[13104,33,3],
[13104,39,1],[13105,26,1],[13105,28,2],[13106,24,1],[13106,31,1],
[13107,26,1],[13107,28,2],[13107,44,1],[13108,27,1],[13108,31,2],
[13108,42,1],[13108,61,1],[13109,2,8],[13109,31,2],[13110,1,2],
[13110,2,1],[13110,10,1],[13110,11,1],[13110,16,1],[13110,21,1],

[13110,22,1],[13110,23,1],[13110,27,1],[13110,28,1],[13110,35,3],
[13110,37,1],[13110,42,1],[13110,50,1],[13110,74,1],[13111,23,1],
[13111,27,1],[13112,15,1],[13112,28,2],[13112,44,1],[13112,55,5],
[13113,15,2],[13113,50,1],[13114,1,1],[13114,24,1],[13115,24,1],
[13115,55,1],[13116,13,1],[13116,73,2],[13117,22,2],[13117,23,1],
[13117,24,2],[13117,27,2],[13117,35,2],[13117,43,4],[13118,20,1],
[13118,22,2],[13118,33,2],[13118,44,3],[13118,56,2],[13118,66,1],
[13118,74,2],[13119,44,1],[13119,47,1],[13119,66,2],[13120,23,1],
[13120,24,1],[13120,28,1],[13120,31,8],[13120,44,1],[13121,20,1],
[13121,31,1],[13122,31,4],[13123,24,1],[13123,28,1],[13124,28,5],
[13124,32,1],[13124,44,1],[13124,69,1],[13124,70,1],[13125,2,4],
[13125,5,1],[13125,21,1],[13125,22,1],[13125,37,1],[13125,38,6],
[13125,42,2],[13125,43,1],[13125,46,1],[13125,47,1],[13125,50,1],
[13125,60,3],[13125,62,1],[13125,70,1],[13125,75,1],[13126,17,1],
[13126,28,1],[13126,31,1],[13127,31,1],[13127,68,2],[13127,72,1],
[13127,75,2],[13128,21,1],[13128,39,1],[13129,24,1],[13129,32,1],
[13130,1,2],[13130,13,11],[13130,39,1],[13131,0,1],[13131,8,1],
[13132,3,1],[13132,28,1],[13132,35,11],[13132,68,1],[13133,12,1],
[13133,35,6],[13133,41,1],[13134,28,1],[13134,75,1],[13135,31,2],
[13136,9,9],[13137,35,1],[13137,38,1],[13137,44,11],[13137,66,11],
[13137,72,1],[13138,3,1],[13138,28,1],[13139,5,1],[13139,33,3],
[13139,35,2],[13139,42,1],[13139,70,1],[13140,5,1],[13140,8,1],
[13140,21,1],[13140,23,2],[13140,24,4],[13140,26,2],[13140,27,5],
[13140,28,2],[13140,30,1],[13140,31,7],[13140,32,3],[13140,39,1],
[13140,43,3],[13140,47,2],[13141,22,1],[13141,24,1],[13141,31,1],
[13141,69,1],[13142,1,1],[13142,8,1],[13142,15,2],[13142,19,1],
[13142,20,1],[13142,28,2],[13143,28,19],[13143,38,1],[13143,68,2],
[13143,70,2],[13143,72,1],[13144,4,1],[13144,24,1],[13145,8,2],
[13146,8,1],[13146,29,1],[13147,23,1],[13147,25,2],[13147,32,1],
[13147,40,1],[13147,63,47],[13147,68,1],[13147,69,1],[13147,70,1],
[13147,74,21],[13147,75,30],[13147,76,55],[13148,1,2],[13148,29,2],
[13149,66,1],[13149,69,1],[13150,10,1],[13150,27,1],[13150,28,1],
[13150,32,1],[13151,55,2],[13152,1,2],[13152,7,1],[13152,30,1],
[13153,1,1],[13153,20,3],[13154,68,5],[13154,72,1],[13155,27,1],
[13155,47,1],[13156,5,2],[13156,21,1],[13156,46,1],[13157,5,3],
[13157,6,1],[13157,27,1],[13157,33,4],[13157,48,1],[13157,62,1],
[13158,24,1],[13158,28,2],[13158,33,41],[13158,35,12],[13158,38,3],
[13159,4,2],[13159,9,1],[13160,28,1],[13160,56,1],[13161,32,2],
[13161,42,6],[13162,2,1],[13162,5,2],[13162,9,1],[13162,10,3],
[13162,16,1],[13162,25,1],[13162,26,1],[13162,29,1],[13162,31,1],
[13162,32,1],[13162,40,4],[13162,41,1],[13162,44,1],[13162,50,2],
[13162,58,1],[13162,63,77],[13162,68,1],[13162,69,2],[13162,70,5],
[13162,74,90],[13162,75,59],[13162,76,126],[13163,1,1],[13163,7,1],
[13163,9,2],[13163,11,2],[13163,13,1],[13163,16,1],[13163,19,1],
[13163,41,1],[13163,49,1],[13163,65,2],[13163,77,1],[13164,9,2],
[13164,10,1],[13164,11,2],[13164,13,1],[13164,14,1],[13164,15,2],
[13164,19,1],[13164,34,1],[13164,49,1],[13164,55,1],[13164,59,1],
[13164,60,1],[13165,3,1],[13165,22,2],[13165,28,2],[13165,40,1],
[13165,44,2],[13165,47,2],[13165,50,1],[13165,51,1],[13165,56,5],
[13165,61,1],[13165,62,1],[13165,68,3],[13165,70,1],[13166,2,2],

[13166,28,1],[13167,2,3],[13167,3,2],[13167,23,3],[13167,28,1],
[13167,30,1],[13167,40,1],[13168,22,7],[13168,27,1],[13168,28,1],
[13168,35,2],[13169,12,1],[13169,23,1],[13169,34,1],[13170,0,1],
[13170,26,1],[13170,43,5],[13170,48,3],[13171,2,5],[13172,56,3],
[13173,4,1],[13173,21,1],[13173,26,1],[13173,42,1],[13173,47,1],
[13174,32,2],[13175,5,1],[13175,10,1],[13175,20,1],[13175,23,1],
[13175,34,3],[13175,39,1],[13175,62,3],[13176,22,1],[13176,23,3],
[13176,28,2],[13177,3,1],[13177,24,1],[13177,27,1],[13177,37,1],
[13177,43,1],[13178,26,2],[13178,27,1],[13178,32,1],[13179,26,1],
[13179,42,1],[13180,3,1],[13180,9,5],[13180,20,1],[13180,23,1],
[13180,24,1],[13180,26,2],[13180,28,9],[13180,31,5],[13180,33,1],
[13180,56,1],[13181,25,2],[13181,28,1],[13182,1,1],[13182,2,2],
[13182,4,1],[13182,8,1],[13182,9,2],[13182,10,2],[13182,15,40],
[13182,16,1],[13182,17,10],[13182,20,1],[13182,28,1],[13182,30,1],
[13182,37,3],[13182,42,1],[13182,44,1],[13182,50,1],[13182,53,6],
[13182,55,201],[13182,56,1],[13182,57,1],[13182,66,1],[13182,69,1],
[13183,1,1],[13183,12,1],[13183,20,1],[13184,17,1],[13184,53,1],
[13185,10,1],[13185,15,1],[13185,32,1],[13185,77,1],[13186,32,2],
[13187,3,1],[13187,35,1],[13187,46,1],[13187,47,1],[13187,51,1],
[13187,57,1],[13187,74,1],[13188,2,1],[13188,28,1],[13188,35,2],
[13188,66,1],[13189,27,1],[13189,50,1],[13189,53,1],[13190,3,1],
[13190,20,1],[13190,22,3],[13190,23,5],[13190,24,1],[13191,48,1],
[13191,74,1],[13192,22,2],[13192,38,1],[13193,1,1],[13193,4,1],
[13193,15,10],[13193,53,1],[13194,2,24],[13195,22,1],[13195,23,1],
[13195,53,1],[13196,1,3],[13197,2,2],[13197,23,2],[13197,27,1],
[13197,47,1],[13198,22,3],[13198,24,1],[13198,26,2],[13198,28,1],
[13198,32,1],[13198,33,1],[13198,35,2],[13198,43,1],[13198,45,1],
[13198,56,1],[13199,27,6],[13199,28,2],[13199,33,1],[13199,35,1],
[13199,69,1],[13200,28,2],[13201,8,2],[13201,12,1],[13202,28,2],
[13203,28,3],[13204,28,1],[13204,61,7],[13204,62,3],[13204,69,2],
[13204,70,2],[13205,2,6],[13205,3,3],[13205,5,2],[13205,6,1],
[13205,7,1],[13205,20,7],[13205,22,5],[13205,23,28],[13205,24,9],
[13205,25,6],[13205,26,4],[13205,27,7],[13205,28,6],[13205,30,2],
[13205,32,12],[13205,33,2],[13205,34,1],[13205,35,14],[13205,38,1],
[13205,39,10],[13205,42,3],[13205,43,4],[13205,48,13],[13205,51,1],
[13205,56,2],[13205,59,2],[13205,62,2],[13205,77,1],[13206,6,1],
[13206,26,1],[13206,31,1],[13207,22,1],[13207,23,1],[13207,78,1],
[13208,32,16],[13208,35,4],[13209,2,3],[13209,24,1],[13210,22,1],
[13210,28,1],[13211,2,3],[13211,5,1],[13211,21,1],[13211,22,2],
[13211,23,3],[13211,24,11],[13211,25,2],[13211,26,1],[13211,27,11],
[13211,28,3],[13211,32,3],[13211,33,2],[13211,35,4],[13211,38,3],
[13211,43,1],[13211,52,1],[13211,56,8],[13212,4,7],[13212,5,2],
[13212,6,1],[13212,9,1],[13212,28,2],[13212,38,1],[13212,40,1],
[13212,50,6],[13212,63,1],[13212,66,1],[13212,70,1],[13213,15,3],
[13213,28,1],[13213,44,3],[13213,55,3],[13214,23,2],[13215,2,1],
[13215,3,1],[13215,20,1],[13215,23,1],[13215,36,1],[13215,42,1],
[13215,60,1],[13216,10,1],[13216,23,1],[13216,30,15],[13216,33,3],
[13216,35,3],[13216,38,1],[13216,52,5],[13216,53,2],[13216,58,1],
[13216,62,1],[13217,23,1],[13217,27,4],[13217,62,1],[13218,5,1],
[13218,6,4],[13218,21,1],[13218,23,1],[13218,25,1],[13218,27,9],

[13218,28,2],[13218,32,5],[13218,33,1],[13218,35,4],[13218,43,1],
[13218,44,1],[13218,56,2],[13219,3,1],[13219,5,2],[13219,22,1],
[13219,23,1],[13219,26,3],[13219,27,1],[13219,28,1],[13220,25,3],
[13220,26,1],[13221,31,1],[13221,33,1],[13222,5,1],[13222,52,1],
[13222,61,1],[13222,63,5],[13222,66,1],[13222,70,2],[13222,72,1],
[13222,74,1],[13222,75,4],[13222,76,11],[13223,23,1],[13223,24,1],
[13223,26,1],[13223,33,1],[13223,35,2],[13223,43,1],[13224,62,2],
[13225,15,1],[13225,20,1],[13226,0,1],[13226,42,1],[13227,62,2],
[13228,25,1],[13228,28,1],[13228,31,1],[13229,23,1],[13229,27,2],
[13229,43,1],[13229,50,1],[13230,62,2],[13231,1,1],[13231,20,2],
[13232,26,2],[13232,42,1],[13232,43,2],[13233,5,1],[13233,25,1],
[13233,43,1],[13233,50,1],[13234,3,10],[13234,4,8],[13234,5,2],
[13234,6,1],[13234,9,1],[13234,10,1],[13234,13,2],[13234,17,1],
[13234,21,2],[13234,22,1],[13234,23,15],[13234,24,7],[13234,25,7],
[13234,26,5],[13234,27,21],[13234,28,5],[13234,30,1],[13234,31,6],
[13234,33,3],[13234,35,14],[13234,40,1],[13234,42,10],[13234,43,3],
[13234,44,2],[13234,45,1],[13234,47,7],[13234,48,4],[13234,50,4],
[13234,55,1],[13234,56,6],[13234,66,2],[13234,72,1],[13234,74,1],
[13234,76,1],[13235,1,2],[13236,15,1],[13236,22,2],[13237,31,2],
[13238,3,1],[13238,51,4],[13238,70,1],[13239,23,2],[13239,28,6],
[13239,32,2],[13239,60,1],[13240,27,2],[13241,0,13],[13241,2,2],
[13242,5,1],[13242,38,1],[13242,52,1],[13243,22,1],[13243,32,2],
[13243,56,1],[13244,27,1],[13244,28,1],[13244,35,2],[13244,55,1],
[13245,26,1],[13245,28,1],[13245,61,2],[13245,63,1],[13246,2,5],
[13246,3,1],[13246,4,1],[13246,6,1],[13246,13,1],[13246,16,1],
[13246,17,1],[13246,19,1],[13246,20,2],[13246,22,1],[13246,23,1],
[13246,24,1],[13246,25,3],[13246,27,4],[13246,30,2],[13246,31,2],
[13246,32,1],[13246,35,1],[13246,36,1],[13246,39,1],[13246,42,1],
[13246,43,5],[13246,44,1],[13246,45,1],[13246,54,1],[13246,59,1],
[13246,61,1],[13246,65,1],[13246,68,1],[13246,70,2],[13246,73,1],
[13246,75,1],[13247,22,2],[13247,23,1],[13247,35,1],[13248,23,1],
[13248,27,1],[13249,3,5],[13250,3,1],[13250,5,2],[13250,22,3],
[13250,24,1],[13250,25,1],[13250,26,1],[13250,35,1],[13250,44,1],
[13250,66,1],[13250,74,1],[13250,75,1],[13251,23,1],[13251,32,1],
[13252,44,1],[13252,63,14],[13252,70,1],[13252,74,16],[13252,75,2],
[13252,76,14],[13253,5,1],[13253,6,11],[13253,24,1],[13253,28,2],
[13253,75,3],[13254,37,1],[13254,47,1],[13255,21,2],[13256,0,2],
[13256,1,37],[13256,3,1],[13256,4,4],[13256,7,13],[13256,8,9],
[13256,9,31],[13256,10,35],[13256,11,27],[13256,12,28],[13256,13,27],
[13256,14,11],[13256,15,75],[13256,16,20],[13256,17,10],[13256,18,11],
[13256,19,26],[13256,20,27],[13256,23,1],[13256,24,1],[13256,26,1],
[13256,27,3],[13256,28,6],[13256,29,10],[13256,30,8],[13256,31,2],
[13256,32,1],[13256,34,17],[13256,35,1],[13256,36,8],[13256,37,15],
[13256,38,2],[13256,39,10],[13256,41,33],[13256,42,3],[13256,44,1],
[13256,45,11],[13256,46,8],[13256,48,1],[13256,49,6],[13256,50,2],
[13256,52,1],[13256,53,11],[13256,54,9],[13256,55,51],[13256,56,1],
[13256,57,11],[13256,58,15],[13256,59,19],[13256,60,3],[13256,62,3],
[13256,63,1],[13256,64,5],[13256,65,20],[13256,66,3],[13256,67,6],
[13256,68,2],[13256,70,1],[13256,71,5],[13256,73,8],[13256,74,1],
[13256,76,7],[13256,77,18],[13256,78,2],[13256,79,1],[13257,6,1],

[13257,24,2],[13257,28,1],[13258,1,7],[13259,13,1],[13259,34,1],
[13259,65,1],[13260,5,1],[13260,24,1],[13261,38,1],[13261,44,1],
[13262,26,2],[13262,35,2],[13263,2,2],[13263,3,1],[13263,20,1],
[13263,24,1],[13263,25,3],[13263,28,1],[13263,42,1],[13263,56,10],
[13263,60,2],[13263,74,1],[13264,2,2],[13265,63,2],[13265,74,1],
[13265,76,1],[13266,4,1],[13266,50,1],[13267,21,1],[13267,23,4],
[13267,28,1],[13267,31,1],[13267,56,1],[13268,17,3],[13268,19,1],
[13268,53,3],[13269,32,3],[13270,5,1],[13270,27,1],[13271,0,2],
[13271,1,23],[13271,2,20],[13271,3,32],[13271,4,2],[13271,5,6],
[13271,6,8],[13271,8,2],[13271,9,4],[13271,10,5],[13271,11,4],
[13271,12,4],[13271,13,30],[13271,14,2],[13271,15,3],[13271,16,3],
[13271,18,1],[13271,19,6],[13271,20,94],[13271,21,7],[13271,22,31],
[13271,23,33],[13271,24,42],[13271,25,12],[13271,26,25],[13271,27,47],
[13271,28,22],[13271,30,8],[13271,31,31],[13271,32,42],[13271,33,13],
[13271,34,15],[13271,35,31],[13271,36,4],[13271,37,3],[13271,38,2],
[13271,39,22],[13271,40,1],[13271,41,2],[13271,42,19],[13271,43,16],
[13271,44,2],[13271,45,6],[13271,46,2],[13271,47,6],[13271,48,5],
[13271,49,2],[13271,52,1],[13271,54,3],[13271,55,2],[13271,56,3],
[13271,57,3],[13271,58,1],[13271,59,11],[13271,60,1],[13271,61,1],
[13271,62,2],[13271,65,2],[13271,66,3],[13271,68,1],[13271,70,2],
[13271,71,1],[13271,72,1],[13271,73,1],[13271,74,1],[13271,76,1],
[13271,77,2],[13271,78,1],[13272,8,1],[13272,23,1],[13272,25,1],
[13272,27,3],[13272,30,1],[13272,31,1],[13273,0,3],[13273,1,31],
[13273,2,1],[13273,3,1],[13273,4,2],[13273,6,3],[13273,7,9],
[13273,9,7],[13273,10,2],[13273,11,8],[13273,12,103],[13273,13,19],
[13273,14,2],[13273,15,2],[13273,16,10],[13273,17,1],[13273,18,1],
[13273,19,2],[13273,20,10],[13273,21,1],[13273,22,2],[13273,24,6],
[13273,26,3],[13273,27,3],[13273,28,1],[13273,29,14],[13273,30,1],
[13273,32,2],[13273,33,4],[13273,34,1],[13273,35,1],[13273,36,3],
[13273,38,3],[13273,39,2],[13273,40,3],[13273,41,1],[13273,42,4],
[13273,43,1],[13273,44,2],[13273,46,2],[13273,47,3],[13273,48,4],
[13273,50,4],[13273,51,11],[13273,54,2],[13273,55,4],[13273,56,6],
[13273,57,1],[13273,61,1],[13273,65,6],[13273,66,2],[13273,67,2],
[13273,68,9],[13273,69,11],[13273,70,11],[13273,72,3],[13273,73,1],
[13273,74,4],[13273,77,17],[13273,78,1],[13273,79,1],[13274,15,1],
[13274,37,1],[13275,20,1],[13275,26,1],[13276,3,1],[13276,5,1],
[13277,8,1],[13277,19,1],[13278,28,1],[13278,33,1],[13279,2,4],
[13280,8,1],[13280,22,1],[13280,28,2],[13280,31,2],[13280,42,1],
[13281,19,1],[13281,40,1],[13281,43,1],[13281,63,6],[13281,70,1],
[13281,74,2],[13281,75,5],[13281,76,17],[13282,23,2],[13282,24,1],
[13282,31,2],[13282,43,1],[13282,52,1],[13283,23,10],[13284,23,1],
[13284,28,1],[13284,35,1],[13284,62,1],[13285,28,1],[13285,50,1],
[13286,1,8],[13286,2,2],[13286,12,2],[13286,29,1],[13286,70,1],
[13286,74,1],[13287,22,2],[13288,2,2],[13288,3,2],[13288,5,2],
[13288,22,1],[13288,24,1],[13288,25,2],[13288,26,2],[13288,32,2],
[13288,35,1],[13288,38,3],[13288,40,5],[13288,44,2],[13288,50,1],
[13288,56,1],[13288,62,2],[13288,63,3],[13288,68,1],[13288,69,2],
[13288,70,8],[13288,72,9],[13288,74,5],[13288,76,3],[13289,22,1],
[13289,30,1],[13290,27,1],[13290,32,2],[13291,31,8],[13292,3,1],
[13292,15,1],[13293,24,1],[13293,27,1],[13293,33,1],[13293,35,1],

[13294,44,1],[13294,53,1],[13295,3,1],[13295,25,1],[13296,9,4],
[13296,15,1],[13296,20,1],[13296,28,1],[13297,41,1],[13297,55,1],
[13298,45,1],[13298,58,1],[13299,2,6],[13299,20,1],[13299,21,4],
[13299,22,1],[13299,23,2],[13299,26,2],[13299,31,1],[13299,37,1],
[13299,53,1],[13299,60,1],[13299,68,1],[13300,62,2],[13301,13,2],
[13301,39,1],[13301,41,1],[13301,59,1],[13301,60,2],[13301,64,1],
[13302,8,2],[13303,1,2],[13303,14,1],[13303,20,3],[13304,23,1],
[13304,47,1],[13304,74,1],[13305,18,1],[13305,53,1],[13305,56,1],
[13306,3,2],[13306,6,1],[13306,40,1],[13306,47,1],[13306,48,1],
[13306,56,1],[13306,68,1],[13307,2,1],[13307,23,1],[13308,24,1],
[13308,32,1],[13308,33,1],[13308,35,1],[13308,56,2],[13309,2,1],
[13309,3,4],[13309,10,1],[13309,14,1],[13309,30,2],[13309,38,1],
[13309,47,1],[13309,55,1],[13309,60,1],[13309,63,1],[13310,33,1],
[13310,63,1],[13310,74,2],[13310,75,1],[13310,76,13],[13311,33,6],
[13312,2,1],[13312,39,1],[13312,47,1],[13313,1,1],[13313,2,2],
[13313,3,8],[13313,5,1],[13313,20,12],[13313,21,6],[13313,22,69],
[13313,23,24],[13313,24,20],[13313,25,4],[13313,26,19],[13313,27,68],
[13313,28,13],[13313,31,9],[13313,32,16],[13313,33,7],[13313,34,4],
[13313,35,22],[13313,38,3],[13313,39,4],[13313,42,7],[13313,43,7],
[13313,45,1],[13313,47,2],[13313,48,1],[13313,52,1],[13313,56,1],
[13313,59,2],[13313,62,1],[13314,10,1],[13314,12,2],[13315,2,2],
[13316,1,2],[13316,39,1],[13317,2,1],[13317,47,1],[13318,2,1],
[13318,22,1],[13318,24,2],[13318,26,2],[13318,27,1],[13318,31,1],
[13318,59,1],[13319,15,1],[13319,27,1],[13319,30,2],[13319,33,1],
[13319,42,1],[13320,2,1],[13320,5,7],[13320,20,1],[13320,21,7],
[13320,22,2],[13320,23,2],[13320,25,1],[13320,26,19],[13320,28,1],
[13320,31,2],[13320,32,10],[13320,33,1],[13320,35,2],[13320,42,2],
[13320,43,27],[13320,51,1],[13320,70,1],[13320,74,1],[13321,1,8],
[13321,7,2],[13321,13,2],[13321,29,5],[13322,5,1],[13322,22,1],
[13322,27,8],[13322,31,1],[13322,35,6],[13323,24,1],[13323,27,1],
[13323,28,1],[13324,2,2],[13324,30,1],[13324,74,1],[13325,27,5],
[13326,35,1],[13326,43,1],[13326,47,1],[13327,28,2],[13327,33,5],
[13328,14,2],[13328,17,1],[13328,19,1],[13328,36,1],[13328,37,1],
[13328,57,1],[13328,65,1],[13329,5,1],[13329,28,1],[13329,57,1],
[13330,3,1],[13330,4,1],[13330,21,3],[13330,22,5],[13330,23,2],
[13330,27,2],[13330,35,4],[13330,43,1],[13330,44,1],[13330,59,1],
[13330,69,1],[13330,74,1],[13331,15,1],[13331,58,1],[13332,23,6],
[13332,25,4],[13332,26,1],[13332,28,1],[13332,35,1],[13332,42,2],
[13332,47,1],[13332,48,1],[13332,56,1],[13333,2,3],[13333,3,2],
[13333,6,2],[13333,10,4],[13333,14,1],[13333,15,2],[13333,16,1],
[13333,18,1],[13333,20,11],[13333,21,3],[13333,22,1],[13333,23,8],
[13333,24,13],[13333,25,4],[13333,26,1],[13333,27,8],[13333,28,2],
[13333,31,7],[13333,32,2],[13333,33,2],[13333,34,10],[13333,35,10],
[13333,37,1],[13333,39,3],[13333,40,1],[13333,41,1],[13333,42,7],
[13333,43,11],[13333,44,1],[13333,47,1],[13333,50,2],[13333,53,2],
[13333,54,1],[13333,55,1],[13333,56,1],[13333,59,1],[13333,65,3],
[13333,69,1],[13333,74,2],[13333,76,1],[13334,11,1],[13334,12,1],
[13334,19,1],[13334,37,1],[13334,64,1],[13335,3,1],[13335,5,1],
[13335,16,1],[13335,21,2],[13335,22,2],[13335,23,7],[13335,24,14],
[13335,25,1],[13335,26,5],[13335,27,4],[13335,28,2],[13335,30,1],

[13335,31,3],[13335,32,8],[13335,33,1],[13335,34,2],[13335,35,1],
[13335,43,1],[13335,47,1],[13335,59,2],[13335,62,3],[13336,5,1],
[13336,63,1],[13336,74,1],[13336,76,5],[13337,21,2],[13337,26,3],
[13337,31,8],[13337,33,2],[13337,42,6],[13337,62,2],[13338,27,5],
[13339,20,2],[13339,23,1],[13339,24,1],[13339,27,1],[13339,28,1],
[13339,47,3],[13340,8,1],[13340,24,1],[13340,35,1],[13340,61,2],
[13340,62,1],[13341,12,1],[13341,13,1],[13341,16,1],[13341,17,1],
[13342,22,1],[13342,62,1],[13343,22,1],[13343,24,2],[13343,30,2],
[13343,36,1],[13343,43,1],[13343,73,2],[13344,20,2],[13344,21,1],
[13344,22,5],[13344,27,2],[13344,31,1],[13344,47,1],[13344,72,1],
[13345,17,2],[13345,53,1],[13346,9,1],[13346,45,1],[13347,3,1],
[13347,5,2],[13347,8,1],[13347,21,2],[13347,22,1],[13347,24,3],
[13347,25,2],[13347,26,1],[13347,27,2],[13347,28,7],[13347,31,2],
[13347,32,1],[13347,35,1],[13347,43,6],[13347,72,1],[13348,22,1],
[13348,23,1],[13349,2,2],[13350,17,1],[13350,53,1],[13351,4,1],
[13351,30,1],[13352,22,1],[13352,43,1],[13353,30,1],[13353,47,1],
[13354,2,3],[13354,12,2],[13354,13,1],[13354,63,1],[13354,70,1],
[13355,44,1],[13355,76,1],[13356,26,1],[13356,27,1],[13357,3,1],
[13357,22,1],[13357,23,2],[13357,24,4],[13357,25,1],[13357,26,1],
[13357,27,4],[13357,28,1],[13357,31,5],[13357,33,1],[13357,37,1],
[13357,42,1],[13357,47,2],[13357,53,1],[13357,62,1],[13357,63,1],
[13357,68,1],[13357,70,1],[13357,74,1],[13358,4,1],[13358,25,1],
[13358,28,6],[13358,30,3],[13358,33,1],[13358,35,2],[13358,40,1],
[13358,52,1],[13359,2,5],[13359,3,5],[13359,4,1],[13359,5,4],
[13359,6,4],[13359,8,3],[13359,12,1],[13359,13,2],[13359,15,1],
[13359,20,7],[13359,21,6],[13359,22,13],[13359,23,32],[13359,24,27],
[13359,26,8],[13359,27,13],[13359,28,3],[13359,30,1],[13359,31,2],
[13359,32,2],[13359,33,11],[13359,34,2],[13359,35,27],[13359,40,1],
[13359,41,3],[13359,42,1],[13359,43,18],[13359,46,1],[13359,47,5],
[13359,48,4],[13359,55,2],[13359,59,4],[13359,62,1],[13359,63,2],
[13359,65,1],[13359,68,2],[13359,70,1],[13359,72,1],[13359,78,3],
[13360,1,2],[13361,2,3],[13361,3,3],[13361,23,1],[13361,28,1],
[13361,44,1],[13361,56,1],[13361,74,1],[13362,30,1],[13362,39,1],
[13362,43,1],[13362,48,1],[13363,47,1],[13363,69,1],[13364,5,1],
[13364,32,1],[13364,55,1],[13365,16,1],[13365,63,1],[13365,72,1],
[13366,28,1],[13366,41,2],[13366,45,1],[13366,53,1],[13367,13,2],
[13368,20,1],[13368,24,1],[13368,27,2],[13369,24,4],[13369,28,1],
[13369,33,1],[13370,3,3],[13370,4,1],[13370,6,4],[13370,23,1],
[13370,24,1],[13370,25,1],[13370,28,5],[13370,32,190],[13370,33,1],
[13370,35,2],[13370,39,1],[13370,40,1],[13370,47,1],[13370,56,1],
[13370,70,2],[13370,74,1],[13370,76,1],[13371,28,1],[13371,37,2],
[13372,26,1],[13372,37,1],[13372,63,7],[13372,66,1],[13372,68,1],
[13372,72,1],[13372,74,4],[13372,76,7],[13373,9,1],[13373,15,2],
[13373,17,1],[13374,23,3],[13375,8,1],[13375,23,1],[13375,24,1],
[13375,51,1],[13375,77,1],[13376,25,2],[13377,7,5],[13377,10,1],
[13377,12,6],[13377,19,1],[13377,29,2],[13377,32,1],[13377,68,3],
[13377,69,1],[13378,3,9],[13378,4,2],[13378,5,1],[13378,6,3],
[13378,7,1],[13378,22,20],[13378,23,12],[13378,24,23],[13378,25,6],
[13378,26,5],[13378,27,8],[13378,28,19],[13378,31,4],[13378,32,3],
[13378,33,15],[13378,35,14],[13378,36,1],[13378,38,1],[13378,39,1],

[13378,42,1],[13378,43,4],[13378,44,1],[13378,47,1],[13378,56,3],
[13378,59,3],[13378,61,3],[13378,62,1],[13378,63,3],[13378,68,1],
[13378,69,1],[13378,70,4],[13378,74,1],[13379,27,1],[13379,28,4],
[13379,35,1],[13379,43,1],[13379,63,1],[13379,72,1],[13380,4,2],
[13380,22,1],[13380,28,1],[13380,44,1],[13381,3,4],[13381,12,5],
[13381,46,1],[13381,58,1],[13381,59,1],[13382,1,1],[13382,53,1],
[13383,25,7],[13383,39,1],[13383,48,22],[13384,33,1],[13384,41,1],
[13384,67,1],[13385,3,2],[13385,20,1],[13385,24,1],[13385,28,3],
[13385,31,1],[13385,50,1],[13386,1,31],[13386,5,1],[13386,12,2],
[13386,13,1],[13386,20,1],[13386,21,1],[13386,23,1],[13386,39,1],
[13386,51,15],[13386,55,1],[13387,28,3],[13388,2,1],[13388,23,1],
[13388,32,1],[13389,12,2],[13389,17,1],[13390,24,1],[13390,26,1],
[13390,31,3],[13390,32,2],[13390,33,1],[13391,12,1],[13391,23,1],
[13392,0,1],[13392,1,1],[13392,8,2],[13392,9,1],[13392,10,2],
[13392,11,1],[13392,12,1],[13392,13,2],[13392,18,3],[13392,19,3],
[13392,20,2],[13392,34,1],[13392,39,2],[13392,41,3],[13392,46,1],
[13392,54,1],[13392,55,1],[13392,57,1],[13392,58,2],[13392,59,2],
[13392,65,4],[13392,67,1],[13392,73,1],[13393,3,2],[13393,27,5],
[13393,28,1],[13393,47,3],[13393,72,1],[13394,6,1],[13394,28,1],
[13394,42,1],[13395,5,1],[13395,21,1],[13395,33,1],[13395,70,1],
[13396,28,1],[13396,42,2],[13397,2,1],[13397,28,1],[13398,1,1],
[13398,4,1],[13398,15,11],[13398,17,1],[13398,29,1],[13398,46,1],
[13398,53,2],[13398,55,4],[13398,66,1],[13399,21,1],[13399,25,1],
[13400,5,1],[13400,22,1],[13400,28,1],[13401,2,68],[13401,28,3],
[13402,1,1],[13402,2,6],[13402,13,1],[13403,24,1],[13403,66,1],
[13404,6,1],[13404,23,1],[13404,30,1],[13404,33,1],[13404,42,1],
[13404,75,2],[13405,2,1],[13405,30,1],[13405,34,2],[13406,3,1],
[13406,23,2],[13406,24,2],[13406,28,2],[13406,35,1],[13407,9,1],
[13407,37,1],[13408,15,1],[13408,55,1],[13409,2,6],[13409,3,4],
[13409,4,1],[13409,5,6],[13409,6,1],[13409,8,2],[13409,20,2],
[13409,21,1],[13409,22,6],[13409,23,3],[13409,24,14],[13409,25,1],
[13409,26,5],[13409,28,4],[13409,30,1],[13409,31,1],[13409,32,1],
[13409,35,2],[13409,37,1],[13409,38,1],[13409,40,1],[13409,42,2],
[13409,43,5],[13409,46,1],[13409,50,2],[13409,51,2],[13409,56,3],
[13409,60,3],[13409,62,1],[13409,68,2],[13409,69,1],[13409,70,1],
[13409,78,1],[13410,44,4],[13410,50,1],[13410,66,3],[13411,28,1],
[13411,61,1],[13412,6,1],[13412,22,3],[13412,25,1],[13412,27,3],
[13412,32,4],[13412,44,1],[13412,62,1],[13412,75,1],[13412,76,1],
[13413,9,1],[13413,12,1],[13413,15,19],[13413,17,11],[13413,19,2],
[13413,21,1],[13413,45,1],[13413,53,13],[13413,54,1],[13413,55,13],
[13413,63,1],[13413,67,1],[13413,72,1],[13414,13,1],[13414,18,1],
[13415,8,2],[13416,22,1],[13416,28,1],[13417,1,3],[13418,20,1],
[13418,21,2],[13418,23,1],[13418,24,3],[13418,27,1],[13418,33,3],
[13418,35,3],[13418,76,1],[13419,55,2],[13420,28,5],[13420,32,4],
[13421,1,256],[13421,3,2],[13421,8,8],[13421,10,3],[13421,12,8],
[13421,13,7],[13421,15,1],[13421,16,3],[13421,17,4],[13421,20,4],
[13421,29,13],[13421,30,1],[13421,34,7],[13421,36,1],[13421,37,1],
[13421,39,6],[13421,53,2],[13421,55,2],[13421,59,1],[13421,65,2],
[13421,73,1],[13421,78,1],[13422,13,1],[13422,27,1],[13422,32,2],
[13422,62,3],[13423,31,1],[13423,42,1],[13424,5,1],[13424,22,1],

[13424,33,1],[13425,9,11],[13425,28,2],[13426,31,3],[13427,2,2],
[13427,6,1],[13427,23,1],[13427,24,3],[13427,26,2],[13427,27,2],
[13427,31,2],[13427,42,1],[13427,62,2],[13428,21,1],[13428,25,1],
[13429,2,2],[13429,9,1],[13429,30,2],[13429,39,1],[13430,5,2],
[13430,21,1],[13430,22,2],[13430,23,8],[13430,24,9],[13430,25,1],
[13430,26,1],[13430,27,6],[13430,32,1],[13430,33,5],[13430,37,1],
[13430,44,1],[13430,47,3],[13430,52,1],[13430,56,2],[13430,64,1],
[13431,15,2],[13431,28,6],[13432,56,1],[13432,62,1],[13433,10,1],
[13433,16,1],[13433,39,1],[13434,1,1],[13434,2,3],[13434,3,5],
[13434,13,3],[13434,14,1],[13434,15,1],[13434,20,3],[13434,23,2],
[13434,24,5],[13434,25,2],[13434,27,1],[13434,28,2],[13434,31,2],
[13434,32,1],[13434,33,5],[13434,34,1],[13434,36,1],[13434,39,2],
[13434,40,1],[13434,61,1],[13434,62,1],[13434,65,1],[13434,73,1],
[13434,77,1],[13435,0,2],[13435,1,15],[13435,7,1],[13435,8,2],
[13435,9,4],[13435,10,2],[13435,11,1],[13435,12,416],[13435,13,3],
[13435,15,5],[13435,16,5],[13435,17,4],[13435,19,1],[13435,20,1],
[13435,25,1],[13435,28,1],[13435,29,10],[13435,30,4],[13435,31,27],
[13435,34,4],[13435,36,1],[13435,37,1],[13435,39,1],[13435,45,2],
[13435,53,2],[13435,55,2],[13435,57,1],[13435,60,1],[13435,65,1],
[13435,67,2],[13435,73,3],[13435,77,2],[13435,78,2],[13436,28,1],
[13436,43,1],[13437,77,1],[13437,78,1],[13438,22,2],[13438,31,1],
[13438,38,1],[13438,43,2],[13439,1,5],[13439,7,1],[13439,8,1],
[13439,9,2],[13439,11,1],[13439,12,3],[13439,13,2],[13439,14,2],
[13439,15,3],[13439,17,4],[13439,18,1],[13439,20,6],[13439,24,1],
[13439,29,2],[13439,30,1],[13439,34,2],[13439,39,2],[13439,41,2],
[13439,43,1],[13439,45,3],[13439,53,3],[13439,55,1],[13439,57,1],
[13439,71,1],[13439,73,1],[13439,77,1],[13440,12,1],[13440,30,1],
[13441,7,1],[13441,27,2],[13442,0,1],[13442,9,1],[13442,11,1],
[13442,13,2],[13442,14,1],[13442,16,1],[13442,19,1],[13442,36,2],
[13442,39,1],[13442,41,1],[13442,49,1],[13442,55,1],[13442,58,1],
[13443,3,1],[13443,5,1],[13443,23,1],[13443,24,1],[13443,27,2],
[13443,28,2],[13443,35,1],[13443,39,1],[13443,43,1],[13443,48,1],
[13443,52,1],[13444,21,2],[13445,3,1],[13445,13,1],[13445,59,1],
[13446,27,1],[13446,35,1],[13446,38,1],[13446,50,1],[13447,5,1],
[13447,23,1],[13447,25,1],[13447,27,1],[13447,31,1],[13447,33,1],
[13448,2,2],[13448,3,3],[13448,30,1],[13448,47,2],[13449,28,1],
[13449,75,1],[13450,2,2],[13450,13,1],[13450,14,1],[13450,22,1],
[13450,23,1],[13450,28,1],[13450,42,1],[13450,68,1],[13450,70,1],
[13451,40,1],[13451,48,8],[13451,54,1],[13452,5,1],[13452,27,4],
[13452,33,1],[13452,35,2],[13452,43,3],[13452,47,1],[13453,13,1],
[13453,27,1],[13453,60,1],[13454,35,2],[13454,38,1],[13454,56,1],
[13455,24,1],[13455,43,1],[13456,27,1],[13456,28,2],[13456,32,1],
[13457,0,2],[13457,1,14],[13457,7,1],[13457,8,1],[13457,9,4],
[13457,10,9],[13457,11,3],[13457,12,11],[13457,13,3],[13457,14,3],
[13457,15,6],[13457,16,4],[13457,17,4],[13457,18,2],[13457,19,8],
[13457,20,1],[13457,29,3],[13457,30,5],[13457,33,1],[13457,34,4],
[13457,37,3],[13457,39,1],[13457,41,8],[13457,45,6],[13457,46,1],
[13457,49,1],[13457,53,7],[13457,54,1],[13457,55,4],[13457,57,4],
[13457,58,3],[13457,59,4],[13457,60,2],[13457,64,2],[13457,65,9],
[13457,67,1],[13457,71,1],[13457,73,1],[13457,77,3],[13457,78,1],

[13458,8,4],[13458,21,1],[13458,22,7],[13458,23,3],[13458,24,1],
[13458,25,1],[13458,27,1],[13458,28,1],[13458,38,1],[13458,43,3],
[13459,15,4],[13459,24,1],[13459,28,1],[13459,55,2],[13459,70,1],
[13460,13,1],[13460,17,1],[13460,24,1],[13460,27,3],[13461,23,1],
[13461,28,1],[13461,30,1],[13462,35,2],[13462,38,1],[13463,2,2],
[13463,56,1],[13464,2,3],[13464,27,2],[13464,31,15],[13464,44,1],
[13465,13,1],[13465,36,1],[13466,1,1],[13466,17,1],[13466,67,1],
[13467,2,1],[13467,5,1],[13467,27,1],[13467,47,1],[13467,48,2],
[13467,62,3],[13468,40,1],[13468,70,1],[13469,20,1],[13469,30,2],
[13469,68,1],[13470,1,2],[13470,8,1],[13470,9,9],[13470,10,2],
[13470,11,1],[13470,12,1],[13470,13,4],[13470,15,15],[13470,17,182],
[13470,19,3],[13470,20,4],[13470,28,4],[13470,29,1],[13470,45,1],
[13470,50,2],[13470,53,233],[13470,55,11],[13470,57,1],[13470,58,1],
[13470,61,1],[13470,62,1],[13470,64,1],[13470,67,1],[13470,73,1],
[13470,77,1],[13471,2,3],[13471,3,2],[13471,8,1],[13471,22,2],
[13471,24,2],[13471,28,1],[13471,39,1],[13471,56,1],[13471,69,1],
[13472,2,1],[13472,71,1],[13473,2,3],[13473,3,7],[13473,5,2],
[13473,6,6],[13473,8,1],[13473,22,18],[13473,23,10],[13473,24,1],
[13473,25,41],[13473,26,4],[13473,27,10],[13473,28,3],[13473,31,2],
[13473,32,5],[13473,35,7],[13473,37,2],[13473,39,2],[13473,40,2],
[13473,42,6],[13473,43,2],[13473,47,3],[13473,51,1],[13473,53,1],
[13473,55,1],[13473,56,1],[13473,62,5],[13473,63,2],[13473,64,1],
[13473,68,2],[13473,72,2],[13473,73,1],[13473,74,1],[13473,76,1],
[13474,15,1],[13474,55,2],[13475,15,1],[13475,55,1],[13476,23,3],
[13476,24,1],[13476,26,1],[13476,27,3],[13476,30,1],[13476,42,4],
[13476,43,1],[13477,2,1],[13477,5,1],[13478,1,1],[13478,39,1],
[13479,2,1],[13479,5,1],[13479,23,1],[13479,25,1],[13479,27,1],
[13479,63,10],[13479,66,1],[13479,70,1],[13479,74,10],[13479,75,7],
[13479,76,13],[13480,12,1],[13480,65,1],[13481,33,5],[13482,15,1],
[13482,55,3],[13483,15,1],[13483,17,2],[13483,53,2],[13484,5,1],
[13484,55,1],[13485,0,1],[13485,1,183],[13485,7,1],[13485,9,3],
[13485,10,3],[13485,12,913],[13485,13,9],[13485,14,2],[13485,15,1],
[13485,16,1],[13485,17,1],[13485,18,2],[13485,19,2],[13485,20,1],
[13485,25,5],[13485,29,10],[13485,37,3],[13485,39,1],[13485,41,1],
[13485,45,3],[13485,46,3],[13485,54,2],[13485,55,1],[13485,59,1],
[13485,65,2],[13485,67,2],[13485,71,1],[13485,73,2],[13485,77,1],
[13485,78,1],[13486,2,13],[13486,28,1],[13486,31,1],[13486,39,2],
[13487,4,1],[13487,13,1],[13487,15,4],[13487,20,1],[13487,23,1],
[13487,35,1],[13487,44,1],[13487,45,1],[13487,49,1],[13487,50,1],
[13487,53,1],[13487,55,1],[13487,61,1],[13487,62,6],[13487,66,1],
[13487,69,1],[13488,31,4],[13489,2,2],[13489,5,2],[13489,19,1],
[13489,23,1],[13489,25,3],[13489,27,1],[13489,28,1],[13489,32,1],
[13489,35,1],[13489,61,2],[13489,62,1],[13490,8,1],[13490,27,5],
[13490,34,5],[13490,39,2],[13490,59,1],[13491,4,6],[13491,31,1],
[13491,44,12],[13491,50,5],[13491,56,2],[13491,66,16],[13492,25,1],
[13492,27,2],[13492,28,1],[13492,31,1],[13492,42,1],[13492,75,1],
[13493,2,3],[13493,3,2],[13493,22,9],[13493,23,1],[13493,24,7],
[13493,25,6],[13493,26,5],[13493,27,8],[13493,28,2],[13493,31,5],
[13493,32,8],[13493,35,3],[13493,50,1],[13493,52,4],[13493,62,3],
[13494,51,2],[13495,2,26],[13495,62,1],[13496,5,1],[13496,74,2],

[13497,13,1],[13497,20,1],[13497,22,1],[13497,45,1],[13498,28,1],
[13498,51,1],[13499,1,1],[13499,2,6],[13499,3,4],[13499,6,1],
[13499,13,1],[13499,21,6],[13499,22,3],[13499,24,7],[13499,25,2],
[13499,26,3],[13499,27,1],[13499,28,2],[13499,31,26],[13499,42,2],
[13499,43,4],[13499,47,3],[13499,50,2],[13499,51,1],[13499,55,1],
[13499,59,1],[13499,60,3],[13499,64,1],[13499,68,1],[13500,50,1],
[13500,66,1],[13501,26,1],[13501,39,1],[13501,56,1],[13501,60,1],
[13502,25,2],[13502,26,1],[13502,27,1],[13502,28,2],[13503,38,2],
[13504,17,1],[13504,22,1],[13504,27,1],[13504,30,1],[13504,32,1],
[13504,33,1],[13504,35,3],[13504,38,1],[13504,39,2],[13504,50,1],
[13505,0,11],[13505,1,98],[13505,7,30],[13505,8,52],[13505,9,198],
[13505,10,212],[13505,11,80],[13505,12,90],[13505,13,64],
[13505,14,67],[13505,15,114],[13505,16,114],[13505,17,73],
[13505,18,79],[13505,19,174],[13505,20,81],[13505,23,2],[13505,29,31],
[13505,30,33],[13505,34,42],[13505,36,44],[13505,37,70],[13505,39,32],
[13505,41,123],[13505,45,104],[13505,46,73],[13505,49,47],
[13505,53,69],[13505,54,60],[13505,55,82],[13505,57,81],[13505,58,75],
[13505,59,82],[13505,60,31],[13505,64,27],[13505,65,152],
[13505,67,97],[13505,69,1],[13505,71,66],[13505,73,65],[13505,77,90],
[13505,78,24],[13506,27,1],[13506,35,1],[13507,21,2],[13508,21,2],
[13508,23,4],[13508,24,2],[13508,26,2],[13508,27,5],[13508,31,1],
[13508,35,1],[13508,43,2],[13508,50,1],[13508,70,1],[13509,1,1],
[13509,31,1],[13509,55,1],[13510,22,3],[13510,27,1],[13511,1,1],
[13511,9,1],[13511,10,1],[13511,13,1],[13511,36,1],[13511,39,1],
[13511,53,3],[13511,55,1],[13511,57,1],[13511,59,2],[13511,62,1],
[13511,71,1],[13512,21,6],[13512,26,1],[13512,27,9],[13512,31,3],
[13512,35,1],[13512,38,1],[13512,42,1],[13513,8,1],[13513,9,1],
[13513,10,1],[13514,1,1],[13514,2,2],[13514,8,1],[13514,16,1],
[13514,20,72],[13514,21,3],[13514,23,10],[13514,24,1],[13514,25,1],
[13514,30,2],[13514,31,16],[13514,32,2],[13514,36,2],[13514,40,1],
[13514,42,14],[13514,43,2],[13514,55,1],[13514,58,1],[13514,63,1],
[13514,67,1],[13515,9,1],[13515,29,1],[13516,50,1],[13516,53,2],
[13517,3,1],[13517,5,1],[13517,19,1],[13517,37,1],[13517,44,1],
[13517,56,2],[13518,1,1],[13518,15,1],[13518,59,1],[13518,67,1],
[13519,24,1],[13519,28,1],[13520,9,1],[13520,62,1],[13521,15,1],
[13521,53,1],[13522,28,2],[13523,23,3],[13523,24,1],[13523,25,1],
[13523,27,4],[13523,28,1],[13523,33,1],[13523,38,1],[13523,56,1],
[13524,24,1],[13524,31,1],[13524,33,1],[13524,52,1],[13525,28,2],
[13525,68,2],[13525,70,3],[13525,72,2],[13526,4,1],[13526,50,1],
[13526,66,1],[13527,4,1],[13527,55,1],[13528,4,1],[13528,21,1],
[13528,24,1],[13528,27,2],[13528,32,2],[13528,33,2],[13528,35,1],
[13528,43,1],[13528,60,1],[13528,70,1],[13529,35,8],[13530,6,1],
[13530,44,1],[13531,5,1],[13531,23,4],[13531,24,5],[13531,26,1],
[13531,27,3],[13531,28,4],[13531,35,1],[13532,0,5],[13532,1,79],
[13532,2,2],[13532,4,2],[13532,7,1],[13532,8,22],[13532,10,1],
[13532,11,1],[13532,12,28],[13532,13,27],[13532,15,14],[13532,16,1],
[13532,17,19],[13532,20,10],[13532,21,4],[13532,23,7],[13532,24,2],
[13532,25,2],[13532,27,1],[13532,29,9],[13532,30,4],[13532,34,20],
[13532,36,12],[13532,37,7],[13532,39,11],[13532,42,1],[13532,45,1],
[13532,46,1],[13532,48,1],[13532,51,1],[13532,53,16],[13532,54,1],

[13532,55,6],[13532,59,6],[13532,65,2],[13532,73,9],[13532,78,1],
[13533,25,2],[13534,28,1],[13534,33,1],[13535,14,1],[13535,26,2],
[13535,55,1],[13535,67,1],[13536,0,1],[13536,7,1],[13537,20,2],
[13537,21,1],[13537,27,9],[13537,28,1],[13537,33,1],[13537,35,5],
[13537,43,8],[13537,52,2],[13538,23,1],[13538,66,1],[13539,5,1],
[13539,8,1],[13539,10,1],[13539,12,5],[13539,20,1],[13539,21,1],
[13539,23,7],[13539,24,1],[13539,25,4],[13539,26,2],[13539,27,2],
[13539,28,3],[13539,30,43],[13539,31,8],[13539,32,2],[13539,36,4],
[13539,39,1],[13539,42,6],[13539,43,2],[13539,59,4],[13540,25,1],
[13540,47,4],[13541,3,1],[13541,5,3],[13541,22,2],[13541,27,10],
[13541,28,1],[13541,31,2],[13541,32,10],[13541,35,4],[13541,42,2],
[13542,52,1],[13542,74,1],[13543,70,5],[13543,72,2],[13544,25,2],
[13545,1,10],[13545,2,6],[13545,3,4],[13545,5,1],[13545,6,1],
[13545,7,1],[13545,10,4],[13545,11,2],[13545,12,1],[13545,13,17],
[13545,16,1],[13545,19,1],[13545,20,5],[13545,21,1],[13545,22,1],
[13545,23,1],[13545,24,1],[13545,26,1],[13545,27,1],[13545,28,6],
[13545,30,3],[13545,31,2],[13545,32,1],[13545,34,1],[13545,35,1],
[13545,36,1],[13545,37,4],[13545,39,1],[13545,40,1],[13545,41,1],
[13545,42,3],[13545,45,1],[13545,47,1],[13545,52,1],[13545,53,1],
[13545,56,1],[13545,57,2],[13545,64,1],[13545,66,1],[13545,69,1],
[13545,73,2],[13546,4,1],[13546,27,1],[13547,28,1],[13547,50,1],
[13548,3,1],[13548,21,1],[13548,22,1],[13548,24,2],[13548,25,3],
[13548,31,1],[13548,32,1],[13548,35,1],[13548,38,1],[13549,21,2],
[13549,22,1],[13549,24,1],[13550,14,1],[13550,28,4],[13550,47,1],
[13550,55,1],[13551,28,2],[13551,70,1],[13552,17,1],[13552,55,1],
[13553,2,2],[13553,10,1],[13553,12,39],[13553,22,1],[13553,23,2],
[13553,29,10],[13553,30,2],[13553,42,1],[13553,45,1],[13553,65,3],
[13553,70,1],[13553,74,1],[13554,17,1],[13554,27,1],[13554,31,1],
[13555,0,2],[13555,41,2],[13555,46,1],[13555,55,1],[13555,77,1],
[13556,27,2],[13557,2,2],[13558,2,11],[13559,23,1],[13559,24,1],
[13559,27,2],[13560,22,3],[13560,35,2],[13560,68,1],[13561,1,5],
[13561,2,3],[13561,3,2],[13561,4,18],[13561,5,5],[13561,7,2],
[13561,8,1],[13561,9,5],[13561,10,3],[13561,11,2],[13561,12,3],
[13561,15,134],[13561,17,50],[13561,18,2],[13561,19,4],[13561,20,4],
[13561,21,2],[13561,23,7],[13561,24,2],[13561,25,4],[13561,26,1],
[13561,27,5],[13561,28,22],[13561,29,1],[13561,30,1],[13561,31,3],
[13561,32,6],[13561,33,1],[13561,34,2],[13561,35,2],[13561,37,1],
[13561,38,6],[13561,39,1],[13561,40,2],[13561,42,2],[13561,43,1],
[13561,44,17],[13561,45,4],[13561,46,3],[13561,47,1],[13561,48,1],
[13561,50,16],[13561,51,1],[13561,52,2],[13561,53,46],[13561,55,108],
[13561,56,1],[13561,58,2],[13561,59,1],[13561,62,2],[13561,63,7],
[13561,64,1],[13561,65,3],[13561,66,16],[13561,67,2],[13561,68,2],
[13561,69,2],[13561,70,9],[13561,72,3],[13561,74,7],[13561,75,1],
[13561,76,3],[13561,79,1],[13562,1,1],[13562,4,2],[13562,5,2],
[13562,7,1],[13562,9,2],[13562,10,5],[13562,11,1],[13562,14,1],
[13562,15,1],[13562,16,1],[13562,17,1],[13562,19,2],[13562,20,1],
[13562,22,2],[13562,27,1],[13562,34,2],[13562,36,1],[13562,37,1],
[13562,38,1],[13562,41,1],[13562,45,1],[13562,46,1],[13562,49,1],
[13562,50,6],[13562,53,3],[13562,55,1],[13562,58,2],[13562,65,4],
[13562,66,1],[13562,77,1],[13563,3,1],[13563,28,3],[13563,33,4],

[13564,32,4],[13565,36,1],[13565,55,1],[13566,6,1],[13566,56,1],
[13566,70,1],[13567,9,1],[13567,10,2],[13567,15,1],[13567,16,1],
[13567,19,1],[13567,36,1],[13567,46,1],[13567,54,1],[13567,57,1],
[13567,59,1],[13567,64,1],[13567,77,1],[13568,1,20],[13568,5,2],
[13568,7,1],[13568,12,1],[13568,13,1],[13568,21,2],[13568,23,1],
[13568,30,1],[13568,34,1],[13568,37,2],[13568,48,1],[13568,55,1],
[13569,3,1],[13569,13,2],[13569,15,2],[13569,16,2],[13569,19,1],
[13569,20,5],[13569,23,2],[13569,24,1],[13569,30,2],[13569,33,3],
[13569,34,2],[13569,35,1],[13569,38,1],[13569,41,2],[13569,47,1],
[13569,49,1],[13569,55,2],[13569,56,2],[13569,59,1],[13569,60,2],
[13569,62,1],[13569,67,1],[13569,71,1],[13570,28,3],[13571,28,1],
[13571,47,1],[13571,56,3],[13572,24,2],[13573,33,7],[13573,35,3],
[13573,38,1],[13573,52,3],[13574,2,16],[13574,6,1],[13574,21,1],
[13574,24,1],[13574,25,1],[13574,28,1],[13574,33,1],[13574,35,1],
[13575,6,2],[13575,28,1],[13575,33,1],[13575,47,4],[13575,56,3],
[13576,12,1],[13576,22,1],[13576,23,2],[13576,34,4],[13576,43,2],
[13577,31,2],[13578,1,1],[13578,7,1],[13578,12,1],[13578,29,1],
[13578,39,1],[13579,38,1],[13579,44,1],[13579,65,1],[13580,49,1],
[13580,59,1],[13581,20,1],[13581,35,4],[13582,4,1],[13582,6,1],
[13582,31,1],[13583,13,3],[13583,22,1],[13583,23,3],[13583,28,2],
[13583,31,1],[13583,32,1],[13583,39,2],[13583,60,1],[13583,62,8],
[13584,35,2],[13584,76,1],[13585,3,1],[13585,47,2],[13586,1,2],
[13586,2,1],[13586,3,36],[13586,4,3],[13586,5,47],[13586,6,3],
[13586,12,1],[13586,13,1],[13586,15,1],[13586,17,1],[13586,20,3],
[13586,21,97],[13586,22,21],[13586,23,249],[13586,24,179],
[13586,25,3],[13586,26,120],[13586,27,179],[13586,28,95],
[13586,31,93],[13586,32,52],[13586,33,33],[13586,34,1],[13586,35,99],
[13586,36,1],[13586,37,1],[13586,38,6],[13586,39,1],[13586,40,3],
[13586,42,23],[13586,43,112],[13586,44,2],[13586,46,1],[13586,47,4],
[13586,50,1],[13586,56,4],[13586,59,2],[13586,63,1],[13586,67,1],
[13586,68,2],[13586,69,3],[13586,70,5],[13586,72,2],[13586,74,5],
[13586,75,3],[13587,22,1],[13587,24,1],[13587,44,1],[13588,56,2],
[13589,62,2],[13590,2,2],[13590,3,1],[13590,23,1],[13590,24,1],
[13590,27,1],[13590,31,1],[13590,35,4],[13591,27,1],[13591,28,1],
[13591,31,1],[13592,12,1],[13592,28,1],[13592,44,2],[13592,51,1],
[13593,2,2],[13593,3,1],[13593,25,2],[13593,43,1],[13594,3,1],
[13594,28,2],[13594,47,1],[13595,9,10],[13595,17,1],[13595,28,1],
[13596,2,1],[13596,7,1],[13597,2,1],[13597,3,1],[13597,5,5],
[13597,20,1],[13597,24,5],[13597,25,3],[13597,26,4],[13597,27,18],
[13597,28,4],[13597,32,3],[13597,42,1],[13597,47,1],[13597,56,1],
[13597,59,1],[13597,73,1],[13598,22,1],[13598,23,1],[13598,24,1],
[13599,28,1],[13599,62,3],[13600,6,1],[13600,28,3],[13601,0,1],
[13601,1,6],[13601,6,1],[13601,7,1],[13601,8,2],[13601,9,1],
[13601,12,1],[13601,15,2],[13601,23,1],[13601,24,2],[13601,25,2],
[13601,26,1],[13601,27,5],[13601,28,9],[13601,29,1],[13601,31,2],
[13601,32,1],[13601,33,1],[13601,34,2],[13601,35,4],[13601,36,2],
[13601,38,1],[13601,39,4],[13601,42,1],[13601,47,1],[13601,50,2],
[13601,51,2],[13601,53,1],[13601,55,1],[13601,62,2],[13601,67,1],
[13601,72,2],[13601,74,1],[13601,75,2],[13601,76,1],[13601,79,1],
[13602,2,2],[13603,4,1],[13603,36,1],[13603,70,1],[13604,2,2],

[13604,3,1],[13604,23,2],[13604,42,1],[13604,46,2],[13604,47,2],
[13604,68,1],[13605,6,1],[13605,51,2],[13605,56,1],[13606,0,1],
[13606,1,68],[13606,2,63],[13606,3,47],[13606,4,5],[13606,5,34],
[13606,6,41],[13606,7,2],[13606,8,8],[13606,9,26],[13606,10,18],
[13606,11,3],[13606,12,33],[13606,13,143],[13606,14,8],[13606,15,17],
[13606,16,12],[13606,17,6],[13606,18,9],[13606,19,17],[13606,20,304],
[13606,21,31],[13606,22,60],[13606,23,91],[13606,24,43],[13606,25,23],
[13606,26,29],[13606,27,159],[13606,28,55],[13606,29,4],[13606,30,40],
[13606,31,32],[13606,32,64],[13606,33,110],[13606,34,56],
[13606,35,96],[13606,36,7],[13606,37,10],[13606,38,14],[13606,39,36],
[13606,40,4],[13606,41,15],[13606,42,56],[13606,43,22],[13606,44,10],
[13606,45,18],[13606,46,10],[13606,47,25],[13606,48,41],[13606,49,5],
[13606,50,5],[13606,51,5],[13606,52,1],[13606,53,5],[13606,54,3],
[13606,55,7],[13606,56,11],[13606,57,6],[13606,58,8],[13606,59,20],
[13606,60,10],[13606,62,16],[13606,63,1],[13606,64,2],[13606,65,17],
[13606,66,3],[13606,67,6],[13606,68,7],[13606,69,3],[13606,70,7],
[13606,71,3],[13606,72,4],[13606,73,4],[13606,74,35],[13606,75,4],
[13606,76,7],[13606,77,9],[13606,78,7],[13607,2,2],[13607,3,2],
[13607,8,2],[13608,32,1],[13608,33,1],[13608,59,2],[13609,23,1],
[13609,24,3],[13609,27,2],[13609,32,1],[13609,37,1],[13609,47,4],
[13610,3,1],[13610,23,2],[13611,26,1],[13611,43,1],[13612,16,1],
[13612,34,1],[13613,6,1],[13613,24,2],[13613,32,5],[13613,35,2],
[13613,71,1],[13614,43,1],[13614,60,1],[13614,77,1],[13615,0,1],
[13615,1,2],[13615,13,1],[13615,20,3],[13616,1,1],[13616,7,1],
[13616,9,1],[13616,14,1],[13616,71,1],[13617,27,1],[13617,28,1],
[13618,1,1],[13618,13,1],[13618,18,1],[13619,0,1],[13619,3,17],
[13619,4,1],[13619,5,16],[13619,6,4],[13619,15,1],[13619,20,2],
[13619,21,17],[13619,22,61],[13619,23,42],[13619,24,36],[13619,25,29],
[13619,26,24],[13619,27,71],[13619,28,27],[13619,30,2],[13619,31,12],
[13619,32,47],[13619,33,13],[13619,35,30],[13619,38,7],[13619,39,2],
[13619,40,2],[13619,42,12],[13619,43,8],[13619,47,5],[13619,48,2],
[13619,50,1],[13619,52,1],[13619,54,1],[13619,56,7],[13619,59,2],
[13619,62,7],[13619,63,1],[13619,66,2],[13619,67,1],[13619,69,1],
[13619,70,1],[13619,72,1],[13619,74,1],[13619,75,4],[13619,76,1],
[13620,1,2],[13620,6,1],[13620,7,1],[13620,9,2],[13620,10,4],
[13620,11,1],[13620,12,3],[13620,14,1],[13620,15,1],[13620,17,2],
[13620,18,2],[13620,20,1],[13620,23,1],[13620,28,2],[13620,29,2],
[13620,31,1],[13620,33,1],[13620,39,1],[13620,40,2],[13620,45,1],
[13620,55,2],[13620,56,1],[13620,57,1],[13620,59,1],[13620,61,1],
[13620,63,1],[13620,64,2],[13620,65,3],[13620,66,1],[13620,74,2],
[13620,75,3],[13620,76,7],[13621,39,2],[13622,3,1],[13622,23,1],
[13622,24,2],[13622,28,2],[13622,62,1],[13623,9,2],[13623,11,1],
[13624,28,2],[13625,15,2],[13626,10,1],[13626,13,1],[13626,16,1],
[13627,20,1],[13627,22,7],[13627,24,2],[13627,25,17],[13627,26,1],
[13627,27,1],[13627,28,10],[13627,30,5],[13627,32,43],[13627,33,60],
[13627,35,107],[13627,38,6],[13627,46,1],[13627,50,1],[13627,52,62],
[13627,66,1],[13627,69,1],[13628,26,1],[13628,43,7],[13629,15,1],
[13629,34,1],[13630,3,1],[13630,8,2],[13630,18,1],[13630,27,2],
[13630,28,1],[13630,30,1],[13630,31,1],[13630,34,5],[13630,35,4],
[13630,37,2],[13630,38,1],[13630,47,1],[13630,53,2],[13630,55,1],

[13630,60,1],[13630,62,5],[13631,1,4],[13631,5,1],[13631,8,1],
[13631,9,1],[13631,10,1],[13631,13,7],[13631,15,1],[13631,17,1],
[13631,19,1],[13631,20,58],[13631,21,1],[13631,22,2],[13631,23,7],
[13631,24,1],[13631,26,3],[13631,27,6],[13631,28,4],[13631,30,1],
[13631,32,1],[13631,33,1],[13631,35,6],[13631,36,1],[13631,37,3],
[13631,40,2],[13631,41,1],[13631,42,6],[13631,43,2],[13631,45,3],
[13631,46,1],[13631,50,1],[13631,59,2],[13631,62,16],[13631,65,2],
[13631,67,1],[13631,70,2],[13631,72,1],[13632,2,1],[13632,3,1],
[13632,4,14],[13632,22,1],[13632,24,1],[13632,25,1],[13632,28,3],
[13632,50,29],[13632,66,1],[13632,70,1],[13633,1,1],[13633,2,1],
[13633,10,1],[13633,20,1],[13633,24,1],[13633,31,1],[13633,41,1],
[13633,53,1],[13634,20,1],[13634,23,1],[13634,27,2],[13634,28,1],
[13634,35,6],[13634,43,1],[13634,50,1],[13634,59,1],[13634,62,1],
[13635,0,1],[13635,1,1],[13635,5,1],[13635,8,1],[13635,13,3],
[13635,14,1],[13635,15,1],[13635,18,7],[13635,20,9],[13635,22,1],
[13635,39,4],[13635,52,1],[13635,55,2],[13635,59,1],[13635,77,28],
[13636,26,1],[13636,27,3],[13636,35,2],[13636,62,1],[13637,2,1],
[13637,3,1],[13637,23,1],[13637,27,1],[13637,35,2],[13638,20,1],
[13638,26,1],[13639,24,1],[13639,28,5],[13639,30,3],[13639,33,3],
[13639,35,1],[13639,38,1],[13639,52,2],[13639,75,1],[13640,23,1],
[13640,28,3],[13640,63,2],[13640,75,4],[13641,1,1],[13641,2,5],
[13641,3,1],[13641,5,2],[13641,8,5],[13641,13,4],[13641,14,1],
[13641,15,1],[13641,20,1],[13641,21,1],[13641,23,11],[13641,24,2],
[13641,25,2],[13641,26,7],[13641,27,7],[13641,28,4],[13641,30,7],
[13641,31,1],[13641,32,18],[13641,33,1],[13641,37,2],[13641,38,1],
[13641,41,1],[13641,42,6],[13641,43,1],[13641,47,5],[13641,48,2],
[13641,53,2],[13641,55,1],[13641,59,1],[13641,60,1],[13641,62,7],
[13641,63,1],[13641,65,2],[13641,66,1],[13641,67,1],[13641,71,1],
[13641,74,1],[13641,78,1],[13642,3,1],[13642,20,1],[13642,57,1],
[13643,1,1],[13643,5,2],[13643,9,1],[13643,12,1],[13643,13,1],
[13643,26,1],[13643,28,5],[13643,33,3],[13643,35,2],[13643,47,3],
[13643,58,2],[13643,59,1],[13644,31,1],[13644,42,1],[13644,43,1],
[13645,31,2],[13646,32,1],[13646,53,1],[13646,61,1],[13646,66,1],
[13647,13,1],[13647,14,1],[13647,18,1],[13647,19,2],[13647,41,1],
[13647,58,2],[13647,65,2],[13647,67,1],[13647,73,1],[13647,77,1],
[13648,55,2],[13649,1,1],[13649,13,1],[13649,15,1],[13649,17,1],
[13649,34,1],[13649,57,1],[13649,59,1],[13649,64,2],[13649,65,1],
[13649,67,1],[13650,12,6],[13650,13,1],[13650,20,1],[13650,23,1],
[13650,28,22],[13650,29,1],[13650,68,1],[13650,75,1],[13651,2,4],
[13652,21,1],[13652,32,4],[13652,39,3],[13653,2,2],[13654,21,2],
[13655,62,12],[13656,28,18],[13656,51,1],[13657,4,1],[13657,10,1],
[13657,22,2],[13657,23,1],[13657,25,1],[13657,31,5],[13657,33,1],
[13657,34,2],[13657,35,1],[13657,39,3],[13657,42,1],[13657,43,2],
[13658,0,2],[13658,21,1],[13658,33,1],[13658,35,1],[13658,39,2],
[13658,40,1],[13658,43,1],[13658,48,1],[13658,59,1],[13659,3,1],
[13659,6,1],[13659,27,1],[13659,47,1],[13660,24,1],[13660,75,1],
[13661,5,1],[13661,24,1],[13661,27,2],[13661,35,1],[13662,4,1],
[13662,27,1],[13662,43,1],[13662,51,1],[13662,62,1],[13663,21,1],
[13663,53,1],[13664,29,1],[13664,67,1],[13665,25,1],[13665,31,1],
[13665,70,1],[13666,7,1],[13666,45,1],[13666,72,1],[13667,9,5],

[13667,20,1],[13667,58,1],[13667,60,1],[13667,73,1],[13668,2,2],
[13668,9,1],[13668,11,1],[13668,19,1],[13668,20,13],[13668,28,1],
[13668,31,2],[13668,42,2],[13668,43,2],[13669,13,1],[13669,17,1],
[13669,27,1],[13670,7,4],[13671,2,1],[13671,28,1],[13672,32,5],
[13672,33,1],[13673,8,1],[13673,10,1],[13674,5,3],[13674,28,1],
[13675,46,1],[13675,58,1],[13676,23,1],[13676,28,1],[13677,26,1],
[13677,42,1],[13678,26,1],[13678,31,1],[13678,43,2],[13679,0,71],
[13679,1,306],[13679,7,58],[13679,8,63],[13679,9,445],[13679,10,515],
[13679,11,199],[13679,12,240],[13679,13,272],[13679,14,121],
[13679,15,319],[13679,16,352],[13679,17,144],[13679,18,145],
[13679,19,467],[13679,20,312],[13679,29,69],[13679,30,74],
[13679,34,125],[13679,36,85],[13679,37,184],[13679,39,112],
[13679,41,230],[13679,45,305],[13679,46,139],[13679,49,129],
[13679,53,145],[13679,54,116],[13679,55,216],[13679,57,234],
[13679,58,187],[13679,59,206],[13679,60,69],[13679,64,56],
[13679,65,382],[13679,67,139],[13679,71,160],[13679,73,144],
[13679,77,216],[13679,78,47],[13680,4,1],[13680,23,4],[13680,28,1],
[13680,43,1],[13681,22,4],[13681,23,3],[13681,24,5],[13681,26,1],
[13681,27,4],[13681,28,1],[13681,32,5],[13681,33,1],[13681,35,4],
[13681,38,2],[13681,47,1],[13682,37,2],[13683,4,1],[13683,15,6],
[13683,17,1],[13683,44,2],[13683,46,1],[13683,53,2],[13683,55,1],
[13683,59,1],[13684,23,1],[13684,35,1],[13685,7,1],[13685,15,3],
[13685,24,2],[13685,28,4],[13685,38,4],[13685,44,36],[13685,50,2],
[13685,51,1],[13685,53,1],[13685,61,1],[13685,65,1],[13685,66,21],
[13685,68,1],[13685,70,1],[13685,74,1],[13686,1,2],[13686,12,1],
[13686,15,1],[13686,31,1],[13686,73,1],[13687,21,3],[13687,31,16],
[13688,32,2],[13689,13,1],[13689,60,1],[13690,23,6],[13691,1,1],
[13691,77,2],[13692,3,1],[13692,6,1],[13693,39,3],[13694,31,1],
[13694,71,1],[13695,20,1],[13695,39,4],[13696,28,1],[13696,74,2],
[13696,76,1],[13697,8,1],[13697,9,2],[13697,13,1],[13697,20,5],
[13697,27,1],[13697,28,4],[13697,70,1],[13698,0,1],[13698,1,84],
[13698,2,7],[13698,3,1],[13698,5,2],[13698,6,6],[13698,8,3],
[13698,11,4],[13698,12,14],[13698,13,18],[13698,15,2],[13698,16,46],
[13698,17,6],[13698,20,11],[13698,23,12],[13698,24,12],[13698,26,4],
[13698,27,5],[13698,28,2],[13698,29,2],[13698,30,3],[13698,32,2],
[13698,33,1],[13698,34,5],[13698,35,3],[13698,36,6],[13698,37,1],
[13698,39,3],[13698,41,1],[13698,42,3],[13698,46,1],[13698,47,8],
[13698,48,1],[13698,53,4],[13698,55,7],[13698,59,2],[13698,62,1],
[13698,66,1],[13698,68,1],[13698,70,1],[13698,73,2],[13698,78,1],
[13699,25,1],[13699,28,1],[13699,41,1],[13699,62,2],[13699,72,1],
[13700,23,1],[13700,25,101],[13700,28,2],[13700,44,1],[13701,30,1],
[13701,32,10],[13702,4,1],[13702,20,1],[13702,28,5],[13702,50,1],
[13703,35,1],[13703,74,1],[13704,2,4],[13705,0,1],[13705,1,1],
[13705,39,1],[13705,65,1],[13706,27,1],[13706,42,1],[13707,5,1],
[13707,21,1],[13708,26,1],[13708,28,1],[13709,3,2],[13709,22,2],
[13710,5,1],[13710,8,3],[13710,15,1],[13710,23,9],[13710,24,3],
[13710,25,17],[13710,26,5],[13710,27,5],[13710,28,3],[13710,32,1],
[13710,33,15],[13710,35,2],[13710,40,1],[13710,42,1],[13710,44,1],
[13710,48,1],[13710,51,1],[13710,54,1],[13710,68,1],[13710,69,3],
[13710,70,8],[13710,72,3],[13710,75,1],[13710,76,2],[13711,26,1],

[13711,27,1],[13711,31,2],[13711,32,1],[13711,34,1],[13711,36,1],
[13711,43,1],[13712,3,1],[13712,23,1],[13712,24,2],[13712,27,1],
[13712,28,4],[13712,29,1],[13712,31,1],[13712,42,3],[13712,43,1],
[13712,47,3],[13712,59,1],[13712,74,1],[13713,31,1],[13713,78,3],
[13714,2,1],[13714,20,1],[13714,22,2],[13714,23,13],[13714,24,10],
[13714,27,11],[13714,28,2],[13714,44,1],[13714,70,1],[13715,27,2],
[13716,13,1],[13716,59,1],[13717,5,1],[13717,32,1],[13718,23,1],
[13718,66,1],[13718,79,1],[13719,3,1],[13719,25,1],[13720,5,1],
[13720,28,8],[13720,63,1],[13720,72,1],[13721,15,1],[13721,28,2],
[13721,35,1],[13722,3,1],[13722,20,1],[13722,21,2],[13722,24,3],
[13722,25,1],[13722,26,1],[13722,27,1],[13722,28,2],[13722,31,1],
[13722,43,2],[13722,59,2],[13723,27,2],[13723,35,1],[13724,4,1],
[13724,5,2],[13724,25,1],[13724,76,1],[13725,3,1],[13725,24,2],
[13725,32,1],[13725,40,2],[13725,42,1],[13725,43,1],[13725,47,3],
[13725,51,2],[13725,56,2],[13725,66,1],[13725,68,2],[13725,70,2],
[13726,15,3],[13726,17,1],[13726,53,2],[13726,55,1],[13727,2,29],
[13727,23,1],[13727,28,1],[13728,15,1],[13728,53,1],[13729,39,2],
[13729,48,1],[13729,78,1],[13730,28,2],[13730,35,1],[13731,30,1],
[13731,42,1],[13732,12,1],[13732,13,1],[13733,2,1],[13733,6,1],
[13733,20,2],[13733,23,3],[13733,25,1],[13733,26,3],[13733,27,1],
[13733,30,1],[13733,32,3],[13733,71,1],[13734,68,3],[13734,69,1],
[13734,70,1],[13734,72,1],[13735,22,1],[13735,27,1],[13736,21,1],
[13736,22,1],[13736,24,1],[13736,25,1],[13736,27,156],[13736,28,3],
[13736,35,2],[13736,42,1],[13737,11,1],[13737,53,1],[13738,2,2],
[13739,5,3],[13740,40,1],[13740,44,1],[13741,1,2],[13741,2,3],
[13741,9,1],[13741,21,1],[13741,25,1],[13741,47,2],[13741,58,1],
[13741,62,1],[13742,2,2],[13742,67,1],[13743,0,1],[13743,1,6],
[13743,2,1],[13743,3,6],[13743,5,3],[13743,9,1],[13743,10,5],
[13743,11,2],[13743,12,1],[13743,13,31],[13743,15,1],[13743,16,2],
[13743,17,1],[13743,18,2],[13743,19,2],[13743,20,10],[13743,21,1],
[13743,22,5],[13743,23,9],[13743,24,15],[13743,25,4],[13743,26,1],
[13743,27,4],[13743,28,16],[13743,29,2],[13743,30,1],[13743,31,1],
[13743,32,2],[13743,34,3],[13743,35,1],[13743,39,7],[13743,42,3],
[13743,43,2],[13743,45,1],[13743,46,3],[13743,47,1],[13743,55,1],
[13743,58,1],[13743,59,2],[13743,64,1],[13743,65,2],[13743,71,1],
[13743,73,1],[13743,77,1],[13744,24,1],[13744,25,1],[13744,28,1],
[13744,42,1],[13745,1,3],[13745,39,1],[13746,3,1],[13746,4,16],
[13746,5,4],[13746,10,1],[13746,16,1],[13746,18,1],[13746,22,4],
[13746,23,4],[13746,25,2],[13746,26,1],[13746,28,6],[13746,32,1],
[13746,33,1],[13746,35,2],[13746,38,11],[13746,42,1],[13746,44,25],
[13746,50,16],[13746,52,1],[13746,56,1],[13746,63,1],[13746,66,25],
[13746,70,2],[13746,72,1],[13746,76,1],[13747,23,1],[13747,59,1],
[13748,27,1],[13748,35,1],[13748,48,1],[13748,56,1],[13748,70,1],
[13749,26,1],[13749,35,1],[13749,62,3],[13750,25,8],[13750,44,1],
[13751,28,7],[13752,20,2],[13752,26,1],[13752,30,1],[13752,74,1],
[13753,24,2],[13753,35,1],[13754,0,8],[13754,1,6],[13754,7,4],
[13754,8,5],[13754,9,9],[13754,10,7],[13754,11,3],[13754,12,36],
[13754,13,6],[13754,14,2],[13754,15,3],[13754,16,3],[13754,17,5],
[13754,18,5],[13754,19,8],[13754,20,6],[13754,29,11],[13754,30,8],
[13754,31,11],[13754,34,1],[13754,36,1],[13754,37,2],[13754,39,3],

[13754,41,3],[13754,45,5],[13754,46,5],[13754,53,4],[13754,54,1],
[13754,55,7],[13754,57,3],[13754,58,3],[13754,59,3],[13754,60,1],
[13754,65,6],[13754,67,3],[13754,71,3],[13754,73,3],[13754,77,1],
[13754,78,2],[13755,2,2],[13756,44,6],[13756,50,1],[13756,66,3],
[13756,68,1],[13757,21,1],[13757,23,1],[13757,26,2],[13757,35,1],
[13757,40,1],[13757,42,1],[13757,43,1],[13757,44,2],[13757,61,9],
[13757,62,1],[13757,63,1],[13757,66,1],[13757,68,9],[13757,69,1],
[13757,70,50],[13757,72,36],[13758,23,6],[13758,24,2],[13758,26,1],
[13758,27,4],[13758,31,3],[13758,32,1],[13758,33,2],[13758,47,1],
[13758,48,1],[13759,5,1],[13759,19,1],[13759,21,1],[13759,23,10],
[13759,24,1],[13759,26,8],[13759,27,1],[13759,31,1],[13759,32,7],
[13759,35,1],[13759,39,2],[13759,42,18],[13759,43,13],[13759,62,1],
[13760,2,2],[13761,22,1],[13761,26,1],[13761,62,2],[13762,0,23],
[13762,1,37],[13762,2,7],[13762,5,3],[13762,6,1],[13762,7,19],
[13762,8,20],[13762,9,46],[13762,10,61],[13762,11,49],[13762,12,73],
[13762,13,13],[13762,14,21],[13762,15,8],[13762,16,48],[13762,17,7],
[13762,18,31],[13762,19,48],[13762,20,55],[13762,21,1],[13762,22,1],
[13762,23,4],[13762,24,5],[13762,25,9],[13762,26,1],[13762,27,5],
[13762,28,17],[13762,29,13],[13762,30,7],[13762,31,7],[13762,32,11],
[13762,33,7],[13762,34,40],[13762,35,5],[13762,36,15],[13762,37,34],
[13762,39,9],[13762,41,23],[13762,42,1],[13762,43,3],[13762,44,1],
[13762,45,35],[13762,46,18],[13762,48,4],[13762,49,14],[13762,51,6],
[13762,53,5],[13762,54,23],[13762,55,7],[13762,56,3],[13762,57,28],
[13762,58,19],[13762,59,27],[13762,60,6],[13762,61,3],[13762,62,11],
[13762,63,3],[13762,64,4],[13762,65,32],[13762,66,1],[13762,67,26],
[13762,68,3],[13762,69,6],[13762,70,11],[13762,71,20],[13762,72,2],
[13762,73,70],[13762,74,3],[13762,76,25],[13762,77,36],[13762,78,8],
[13762,79,1],[13763,8,1],[13763,9,1],[13763,10,1],[13763,11,1],
[13763,13,1],[13763,14,2],[13763,16,1],[13763,19,2],[13763,20,2],
[13763,36,1],[13763,59,1],[13763,64,1],[13763,73,2],[13763,77,1],
[13763,78,1],[13764,24,1],[13764,32,2],[13765,21,2],[13765,23,2],
[13765,28,1],[13765,31,1],[13765,39,1],[13765,43,1],[13766,2,2],
[13767,2,10],[13767,3,2],[13767,4,1],[13767,6,1],[13767,21,1],
[13767,24,4],[13767,26,2],[13767,35,1],[13767,44,1],[13767,47,1],
[13767,63,1],[13767,66,1],[13767,68,1],[13767,70,3],[13768,23,1],
[13768,28,18],[13769,0,10],[13769,7,11],[13769,10,1],[13769,14,2],
[13769,16,1],[13769,18,1],[13769,36,1],[13769,57,1],[13769,59,1],
[13769,77,1],[13770,1,1],[13770,9,1],[13770,13,2],[13770,28,1],
[13770,30,2],[13770,37,1],[13770,78,1],[13771,40,1],[13771,63,1],
[13772,5,1],[13772,25,1],[13772,27,1],[13773,20,4],[13773,22,2],
[13773,32,1],[13773,35,3],[13773,40,1],[13773,62,1],[13774,0,1],
[13774,44,1],[13774,60,1],[13775,35,1],[13775,47,1],[13776,32,2],
[13777,2,1],[13777,3,1],[13777,20,2],[13777,21,2],[13777,24,2],
[13777,25,13],[13777,26,1],[13777,27,122],[13777,28,10],[13777,35,50],
[13777,40,2],[13777,44,1],[13777,45,1],[13777,62,9],[13777,72,1],
[13778,15,1],[13778,17,1],[13778,53,3],[13779,31,2],[13780,26,1],
[13780,52,1],[13781,20,1],[13781,45,1],[13782,15,1],[13782,24,1],
[13782,25,1],[13782,60,2],[13783,3,1],[13783,5,1],[13783,28,2],
[13783,33,2],[13783,42,1],[13783,62,87],[13783,66,1],[13783,73,1],
[13783,74,1],[13784,0,5],[13784,1,1],[13784,12,2],[13784,13,1],

[13784,15,2],[13784,17,4],[13784,20,1],[13784,30,2],[13784,34,1],
[13784,45,1],[13784,53,1],[13784,54,1],[13784,57,1],[13784,59,2],
[13785,27,2],[13785,32,2],[13785,40,1],[13785,56,1],[13786,13,1],
[13786,24,1],[13787,47,2],[13787,56,1],[13788,28,1],[13788,33,2],
[13788,50,1],[13789,2,2],[13789,22,1],[13790,43,2],[13791,27,4],
[13792,28,3],[13793,2,11],[13793,3,5],[13793,10,3],[13793,13,1],
[13793,21,4],[13793,22,1],[13793,23,10],[13793,24,5],[13793,25,3],
[13793,26,5],[13793,27,14],[13793,28,14],[13793,30,1],[13793,31,3],
[13793,32,2],[13793,33,3],[13793,35,3],[13793,39,1],[13793,42,3],
[13793,43,6],[13793,46,1],[13793,47,1],[13793,51,1],[13793,52,1],
[13793,62,3],[13794,28,1],[13794,31,2],[13795,15,1],[13795,44,2],
[13796,38,1],[13796,44,3],[13796,50,2],[13796,66,7],[13797,2,3],
[13797,28,1],[13798,51,4],[13798,68,1],[13799,3,1],[13799,33,4],
[13800,13,1],[13800,24,4],[13800,27,6],[13800,28,1],[13800,29,2],
[13800,32,1],[13800,33,1],[13800,35,3],[13800,36,1],[13800,42,2],
[13800,76,1],[13801,2,4],[13801,4,1],[13801,28,1],[13802,56,2],
[13803,27,1],[13803,31,1],[13804,32,5],[13804,38,1],[13805,22,1],
[13805,26,1],[13805,31,1],[13805,32,1],[13806,3,2],[13806,24,2],
[13806,27,1],[13806,31,1],[13806,32,1],[13806,35,1],[13807,12,1],
[13807,29,1],[13808,9,2],[13808,28,4],[13808,32,1],[13808,44,1],
[13808,69,1],[13809,3,2],[13809,6,1],[13809,25,1],[13809,26,1],
[13809,33,2],[13809,55,1],[13810,2,1],[13810,3,1],[13810,4,1],
[13810,23,1],[13810,24,2],[13810,26,1],[13810,27,2],[13810,28,21],
[13810,31,1],[13810,32,1],[13810,35,2],[13810,38,2],[13810,44,1],
[13810,47,2],[13810,56,1],[13810,66,2],[13810,69,1],[13810,70,3],
[13810,75,1],[13810,76,3],[13810,79,1],[13811,0,1],[13811,1,9],
[13811,2,16],[13811,7,1],[13811,8,2],[13811,9,6],[13811,10,1],
[13811,12,20],[13811,13,107],[13811,17,6],[13811,19,3],[13811,20,3],
[13811,21,1],[13811,29,3],[13811,30,2],[13811,31,2],[13811,34,5],
[13811,36,1],[13811,39,6],[13811,41,2],[13811,45,2],[13811,46,2],
[13811,47,1],[13811,48,3],[13811,53,2],[13811,64,1],[13811,73,1],
[13811,77,1],[13811,78,1],[13812,1,1],[13812,2,18],[13812,3,6],
[13812,4,1],[13812,5,5],[13812,6,3],[13812,8,1],[13812,16,1],
[13812,21,14],[13812,22,14],[13812,23,226],[13812,24,24],
[13812,25,11],[13812,26,12],[13812,27,142],[13812,28,34],[13812,30,4],
[13812,31,41],[13812,32,6],[13812,33,12],[13812,34,1],[13812,35,3],
[13812,37,1],[13812,38,15],[13812,40,4],[13812,42,448],[13812,43,7],
[13812,44,8],[13812,47,5],[13812,48,5],[13812,50,8],[13812,51,2],
[13812,52,1],[13812,53,1],[13812,55,3],[13812,56,2],[13812,59,1],
[13812,61,2],[13812,62,12],[13812,63,2],[13812,66,6],[13812,68,1],
[13812,69,3],[13812,70,8],[13812,72,3],[13812,74,10],[13812,75,3],
[13812,76,10],[13813,3,1],[13813,9,1],[13813,26,1],[13813,35,1],
[13813,37,10],[13814,1,1],[13814,71,1],[13815,42,2],[13815,43,1],
[13816,3,1],[13816,23,1],[13816,24,1],[13816,27,2],[13816,47,2],
[13817,3,2],[13817,23,1],[13817,35,3],[13817,42,1],[13817,47,1],
[13817,51,4],[13817,62,3],[13818,27,2],[13818,35,1],[13819,15,2],
[13819,26,1],[13819,39,1],[13819,46,1],[13819,53,1],[13819,72,1],
[13819,74,2],[13819,75,3],[13819,76,2],[13820,1,1],[13820,20,1],
[13821,12,1],[13821,53,1],[13822,21,1],[13822,47,1],[13823,19,1],
[13823,65,1],[13824,27,2],[13824,28,1],[13825,9,1],[13825,13,2],

[13825,20,1],[13825,41,2],[13825,45,2],[13825,60,1],[13825,77,1],
[13826,9,1],[13826,42,1],[13827,5,1],[13827,27,2],[13827,32,2],
[13827,56,1],[13828,28,1],[13828,32,26],[13829,28,1],[13829,35,1],
[13830,8,1],[13830,11,1],[13830,28,1],[13830,62,1],[13831,23,1],
[13831,28,1],[13831,32,3],[13832,30,1],[13832,39,2],[13833,20,1],
[13833,26,1],[13833,27,1],[13833,39,2],[13834,28,3],[13834,68,1],
[13835,3,1],[13835,23,1],[13835,26,1],[13836,1,10],[13836,6,1],
[13836,8,1],[13836,13,1],[13836,20,2],[13836,23,13],[13836,24,1],
[13836,26,1],[13836,29,9],[13836,31,2],[13836,33,10],[13836,34,2],
[13836,36,3],[13836,39,2],[13836,43,1],[13836,68,1],[13836,78,1],
[13837,8,1],[13837,9,1],[13837,12,1],[13837,45,1],[13837,55,1],
[13838,3,1],[13838,22,4],[13838,23,2],[13838,24,1],[13838,27,6],
[13838,28,1],[13838,32,5],[13838,33,1],[13838,34,2],[13838,35,1],
[13838,54,1],[13839,2,5],[13839,22,1],[13839,31,10],[13839,53,1],
[13840,1,1],[13840,9,3],[13840,10,1],[13840,11,2],[13840,16,1],
[13840,19,1],[13840,20,1],[13840,22,1],[13840,24,1],[13840,28,10],
[13840,45,3],[13840,69,1],[13840,71,1],[13840,72,1],[13840,73,1],
[13840,77,1],[13841,31,1],[13841,32,1],[13841,43,3],[13841,62,1],
[13842,17,2],[13843,44,1],[13843,66,1],[13844,3,1],[13844,4,1],
[13844,5,2],[13844,25,1],[13844,31,1],[13844,42,1],[13844,48,1],
[13844,55,1],[13844,74,1],[13844,75,1],[13845,9,2],[13845,59,1],
[13845,65,1],[13845,73,1],[13846,2,3],[13846,28,1],[13847,66,3],
[13848,25,5],[13849,23,1],[13849,32,2],[13849,56,1],[13849,74,1],
[13850,17,1],[13850,55,1],[13851,0,1],[13851,1,2],[13851,2,61],
[13851,3,45],[13851,4,41],[13851,5,93],[13851,6,22],[13851,8,3],
[13851,9,1],[13851,12,2],[13851,13,1],[13851,15,1],[13851,17,2],
[13851,19,2],[13851,20,1],[13851,21,29],[13851,22,29],[13851,23,74],
[13851,24,75],[13851,25,108],[13851,26,56],[13851,27,75],
[13851,28,836],[13851,31,63],[13851,32,64],[13851,33,29],
[13851,35,55],[13851,36,1],[13851,37,2],[13851,38,45],
[13851,40,14211],[13851,42,35],[13851,43,25],[13851,44,50],
[13851,45,2],[13851,47,32],[13851,48,27],[13851,50,51],[13851,51,11],
[13851,52,14],[13851,56,35],[13851,58,2],[13851,61,79],[13851,62,24],
[13851,63,143],[13851,66,59],[13851,68,5115],[13851,69,4604],
[13851,70,22937],[13851,72,9815],[13851,73,1],[13851,74,159],
[13851,75,92],[13851,76,190],[13851,77,1],[13851,79,5],[13852,12,1],
[13852,32,1],[13853,2,1],[13853,28,1],[13854,1,2],[13854,8,1],
[13854,22,1],[13854,23,4],[13854,24,5],[13854,25,1],[13854,26,2],
[13854,28,2],[13854,31,2],[13854,32,2],[13854,34,1],[13854,43,2],
[13854,47,1],[13854,59,3],[13855,7,1],[13855,10,1],[13855,12,1],
[13855,19,2],[13855,34,1],[13855,57,1],[13855,71,1],[13856,21,1],
[13856,28,1],[13856,42,12],[13856,59,1],[13857,2,1],[13857,24,1],
[13857,78,1],[13858,2,3],[13858,27,2],[13858,31,1],[13858,62,1],
[13859,1,8],[13859,2,31],[13859,3,4],[13859,4,1],[13859,5,5],
[13859,6,4],[13859,7,2],[13859,9,2],[13859,10,3],[13859,12,1],
[13859,13,15],[13859,15,1],[13859,17,2],[13859,20,43],[13859,21,2],
[13859,22,3],[13859,23,19],[13859,24,16],[13859,25,2],[13859,26,3],
[13859,27,10],[13859,28,9],[13859,31,7],[13859,32,2],[13859,33,14],
[13859,35,15],[13859,37,3],[13859,39,2],[13859,40,3],[13859,42,6],
[13859,43,1],[13859,44,1],[13859,45,2],[13859,46,1],[13859,47,7],

[13859,48,6],[13859,50,1],[13859,51,2],[13859,54,1],[13859,56,5],
[13859,57,1],[13859,62,1],[13859,63,1],[13859,64,1],[13859,66,2],
[13859,68,2],[13859,69,1],[13859,70,4],[13859,72,1],[13859,73,1],
[13859,74,9],[13859,75,2],[13859,76,5],[13859,77,1],[13860,20,3],
[13861,2,2],[13861,24,1],[13861,31,1],[13861,38,1],[13862,2,2],
[13862,28,2],[13862,33,1],[13862,35,1],[13862,42,1],[13863,3,1],
[13863,5,1],[13863,23,1],[13863,28,3],[13863,32,2],[13863,34,2],
[13863,35,1],[13863,40,1],[13863,43,1],[13863,48,1],[13863,62,49],
[13863,70,1],[13863,74,3],[13864,25,1],[13864,28,4],[13864,35,1],
[13864,62,1],[13865,28,1],[13865,31,1],[13866,28,1],[13866,75,1],
[13867,2,1],[13867,3,11],[13867,9,1],[13867,10,1],[13867,13,1],
[13867,28,1],[13867,29,1],[13868,1,1],[13868,2,1],[13868,21,1],
[13868,28,7],[13868,35,1],[13868,44,1],[13868,63,1],[13868,75,1],
[13869,1,2],[13869,15,1],[13869,36,1],[13869,41,1],[13869,45,2],
[13869,54,1],[13869,64,1],[13869,71,1],[13870,5,2],[13870,6,1],
[13870,27,1],[13870,32,2],[13870,35,4],[13870,78,1],[13871,3,6],
[13871,8,3],[13871,23,2],[13871,24,8],[13871,25,1],[13871,26,2],
[13871,27,2],[13871,28,2],[13871,30,2],[13871,31,4],[13871,32,2],
[13871,37,4],[13871,42,4],[13871,43,1],[13871,44,1],[13871,45,1],
[13871,47,4],[13871,59,1],[13871,65,1],[13871,76,1],[13872,39,1],
[13872,50,1],[13873,56,3],[13874,20,1],[13874,24,1],[13874,31,1],
[13874,32,1],[13874,37,1],[13874,42,1],[13874,43,1],[13874,56,1],
[13874,60,1],[13874,70,1],[13875,3,1],[13875,26,1],[13875,42,1],
[13876,15,1],[13876,23,1],[13876,51,1],[13876,59,2],[13877,22,1],
[13877,23,3],[13877,24,3],[13877,25,1],[13877,26,2],[13877,27,7],
[13877,32,4],[13877,33,1],[13877,35,1],[13877,38,1],[13877,43,1],
[13877,62,1],[13878,28,1],[13878,42,2],[13879,30,1],[13879,32,1],
[13879,42,1],[13880,20,1],[13880,24,1],[13880,39,1],[13881,6,2],
[13881,27,1],[13882,17,1],[13882,20,1],[13882,22,2],[13882,23,1],
[13882,24,3],[13882,25,2],[13882,27,1],[13882,32,1],[13882,35,2],
[13882,42,1],[13882,43,1],[13882,59,1],[13882,62,1],[13882,76,1],
[13883,9,1],[13883,27,1],[13883,28,2],[13884,1,1],[13884,29,1],
[13885,1,1],[13885,29,1],[13885,45,1],[13886,17,4],[13886,53,3],
[13887,42,2],[13888,59,1],[13888,65,1],[13889,23,1],[13889,28,1],
[13889,47,1],[13890,30,2],[13891,26,2],[13892,2,1],[13892,53,1],
[13893,30,2],[13894,9,4],[13894,28,3],[13895,16,1],[13895,27,2],
[13896,40,1],[13896,63,2],[13896,74,4],[13896,75,2],[13896,76,1],
[13897,22,1],[13897,26,2],[13897,74,1],[13898,63,1],[13898,74,2],
[13898,75,2],[13898,76,3],[13899,2,2],[13899,41,1],[13900,3,1],
[13900,25,10],[13900,35,1],[13900,44,1],[13900,66,1],[13901,3,1],
[13901,23,2],[13901,28,3],[13901,38,1],[13901,43,1],[13901,48,1],
[13902,2,1],[13902,5,1],[13902,24,1],[13902,25,1],[13902,27,1],
[13902,32,1],[13902,42,1],[13902,43,1],[13902,63,25],[13902,68,1],
[13902,69,1],[13902,70,1],[13902,74,13],[13902,75,7],[13902,76,37],
[13903,23,2],[13904,28,4],[13904,33,1],[13905,30,1],[13905,71,1],
[13906,37,1],[13906,48,1],[13907,55,2],[13908,24,3],[13908,27,1],
[13909,5,2],[13909,23,1],[13909,28,1],[13909,44,1],[13910,5,1],
[13910,21,1],[13910,30,2],[13911,12,1],[13911,59,2],[13911,73,1],
[13912,5,1],[13912,28,1],[13912,74,3],[13913,24,1],[13913,31,15],
[13913,43,1],[13914,19,1],[13914,35,1],[13915,6,1],[13915,24,2],

[13915,27,2],[13915,33,3],[13915,35,1],[13915,37,2],[13915,40,2],
[13915,43,2],[13916,2,1],[13916,44,1],[13917,23,1],[13917,27,1],
[13918,2,1],[13918,21,1],[13918,24,1],[13918,25,1],[13918,26,1],
[13918,32,1],[13918,33,1],[13918,38,1],[13918,43,3],[13918,47,1],
[13919,1,1],[13919,25,1],[13919,33,1],[13919,37,1],[13920,28,1],
[13920,56,1],[13921,32,1],[13921,50,1],[13921,62,1],[13922,2,2],
[13923,38,1],[13923,66,1],[13924,43,1],[13924,69,1],[13925,2,1],
[13925,33,2],[13926,28,2],[13927,62,3],[13928,2,5],[13929,5,1],
[13929,22,2],[13929,31,2],[13929,35,1],[13930,12,1],[13930,16,6],
[13931,2,4],[13931,3,1],[13931,6,1],[13931,8,1],[13931,9,2],
[13931,15,1],[13931,21,1],[13931,22,2],[13931,23,4],[13931,24,1],
[13931,27,5],[13931,28,18],[13931,30,1],[13931,31,2],[13931,32,1],
[13931,34,1],[13931,42,1],[13931,47,3],[13931,56,1],[13931,60,1],
[13931,62,1],[13931,74,1],[13932,23,1],[13932,56,1],[13933,1,1],
[13933,15,1],[13934,1,2],[13934,20,2],[13935,20,1],[13935,33,1],
[13936,9,39],[13936,28,4],[13936,34,1],[13936,37,1],[13936,41,1],
[13937,2,1],[13937,25,1],[13937,27,1],[13937,35,1],[13937,43,1],
[13938,2,2],[13938,3,3],[13938,23,2],[13938,24,2],[13938,30,1],
[13938,31,3],[13938,37,1],[13938,43,2],[13938,60,1],[13938,70,1],
[13939,28,3],[13940,4,1],[13940,26,1],[13940,42,1],[13941,11,1],
[13941,17,1],[13942,1,4],[13942,2,1],[13942,6,2],[13942,8,2],
[13942,12,12],[13942,13,8],[13942,15,1],[13942,17,1],[13942,20,3],
[13942,23,8],[13942,24,1],[13942,26,2],[13942,27,1],[13942,30,3],
[13942,31,1],[13942,32,1],[13942,33,3],[13942,34,4],[13942,45,3],
[13942,46,2],[13942,48,1],[13942,53,1],[13942,59,3],[13942,73,2],
[13943,28,6],[13944,47,2],[13945,68,1],[13945,72,1],[13946,22,1],
[13946,25,8],[13947,15,6],[13947,28,1],[13947,34,1],[13947,55,1],
[13947,74,1],[13948,5,2],[13949,18,1],[13949,53,1],[13950,59,4],
[13951,27,1],[13951,51,1],[13952,21,2],[13952,28,1],[13952,31,7],
[13952,33,5],[13952,42,2],[13953,10,1],[13953,56,1],[13954,1,1],
[13954,10,1],[13954,15,3],[13954,41,1],[13954,55,4],[13955,19,1],
[13955,78,1],[13956,17,1],[13956,55,1],[13957,2,2],[13957,5,1],
[13957,26,1],[13957,31,1],[13957,43,1],[13958,0,4],[13958,1,24],
[13958,2,1],[13958,3,3],[13958,4,13],[13958,5,7],[13958,7,1],
[13958,8,8],[13958,9,31],[13958,10,28],[13958,11,12],[13958,12,20],
[13958,13,10],[13958,14,6],[13958,15,79],[13958,16,14],[13958,17,18],
[13958,18,5],[13958,19,25],[13958,20,11],[13958,21,2],[13958,22,11],
[13958,23,14],[13958,25,7],[13958,26,1],[13958,28,11],[13958,29,17],
[13958,30,4],[13958,32,3],[13958,33,4],[13958,34,9],[13958,35,7],
[13958,36,4],[13958,37,7],[13958,38,9],[13958,39,14],[13958,41,13],
[13958,42,1],[13958,43,1],[13958,44,38],[13958,45,23],[13958,46,5],
[13958,49,4],[13958,50,20],[13958,53,18],[13958,54,4],[13958,55,30],
[13958,56,2],[13958,57,8],[13958,58,16],[13958,59,16],[13958,60,6],
[13958,64,2],[13958,65,19],[13958,66,27],[13958,67,9],[13958,71,4],
[13958,73,11],[13958,74,1],[13958,76,1],[13958,77,9],[13959,23,2],
[13959,26,1],[13959,28,1],[13959,31,3],[13959,32,1],[13960,6,1],
[13960,21,4],[13960,22,1],[13960,28,1],[13960,30,1],[13960,31,1],
[13960,33,1],[13960,42,2],[13960,43,9],[13960,53,1],[13961,1,3],
[13961,10,1],[13961,12,3],[13961,13,3],[13961,14,3],[13961,20,12],
[13961,21,1],[13961,23,1],[13961,24,2],[13961,26,3],[13961,27,1],

[13961,31,1],[13961,34,1],[13961,35,2],[13961,36,1],[13961,43,1],
[13961,59,1],[13961,62,1],[13961,65,1],[13962,2,1],[13962,37,1],
[13963,12,1],[13963,28,2],[13963,79,1],[13964,9,1],[13964,15,4],
[13965,0,8],[13965,9,1],[13965,12,1],[13965,37,1],[13965,65,1],
[13966,4,1],[13966,23,23],[13966,24,1],[13966,25,1],[13966,28,1],
[13966,32,1],[13966,38,1],[13966,42,2],[13966,44,1],[13966,50,2],
[13966,56,1],[13966,63,1],[13967,19,1],[13967,23,1],[13967,25,1],
[13967,27,1],[13967,32,1],[13968,14,1],[13968,16,1],[13969,63,2],
[13969,74,3],[13969,75,1],[13969,76,4],[13970,44,3],[13971,23,1],
[13971,24,1],[13971,25,1],[13971,35,1],[13972,30,1],[13972,78,1],
[13973,20,3],[13973,42,2],[13974,13,1],[13974,20,2],[13974,22,1],
[13974,23,3],[13974,24,2],[13974,25,1],[13974,28,1],[13974,31,1],
[13974,34,2],[13974,36,1],[13974,39,5],[13974,47,1],[13975,15,1],
[13975,28,1],[13975,70,1],[13976,24,1],[13976,28,1],[13976,31,1],
[13977,9,1],[13977,11,1],[13978,2,1],[13978,3,4],[13978,9,1],
[13978,13,1],[13978,22,1],[13978,23,2],[13978,24,1],[13978,26,1],
[13978,28,2],[13978,30,4],[13978,31,2],[13978,37,1],[13978,43,1],
[13978,56,2],[13978,60,2],[13978,74,1],[13979,12,1],[13979,25,3],
[13979,28,4],[13979,30,1],[13979,33,7],[13979,35,11],[13980,21,2],
[13980,23,1],[13980,27,1],[13980,28,12],[13980,31,3],[13980,33,4],
[13980,42,1],[13980,44,2],[13980,63,1],[13980,68,1],[13980,70,1],
[13980,75,1],[13980,76,2],[13981,3,1],[13981,5,4],[13981,33,13],
[13981,38,1],[13981,56,1],[13981,72,1],[13982,18,1],[13982,19,2],
[13982,37,1],[13982,65,1],[13983,23,2],[13984,1,2],[13984,30,2],
[13984,31,6],[13984,47,1],[13985,27,1],[13985,42,1],[13986,25,1],
[13986,30,1],[13986,47,1],[13987,10,1],[13987,62,1],[13988,27,2],
[13989,30,1],[13989,55,1],[13990,0,3],[13990,2,4],[13990,11,1],
[13990,20,1],[13990,30,1],[13990,31,10],[13990,32,1],[13990,36,1],
[13990,39,1],[13990,41,1],[13990,62,3],[13991,28,1],[13991,70,1],
[13992,1,1],[13992,3,1],[13992,5,2],[13992,13,1],[13992,15,2],
[13992,20,1],[13992,23,23],[13992,24,3],[13992,26,1],[13992,27,6],
[13992,29,1],[13992,32,4],[13992,35,8],[13992,36,1],[13992,43,1],
[13992,57,1],[13992,62,5],[13992,67,1],[13993,22,1],[13993,23,1],
[13993,26,1],[13993,43,1],[13993,69,1],[13994,33,2],[13995,23,3],
[13995,27,1],[13995,39,1],[13995,72,1],[13996,23,4],[13996,24,2],
[13996,28,1],[13996,32,3],[13996,33,8],[13996,43,1],[13996,47,1],
[13997,35,2],[13997,56,1],[13998,24,4],[13998,28,3],[13999,24,1],
[13999,28,1],[14000,17,1],[14000,28,15],[14000,55,3],[14001,1,2],
[14001,30,1],[14001,32,34],[14001,63,1],[14002,21,1],[14002,23,1],
[14002,24,2],[14002,25,1],[14002,27,1],[14002,32,1],[14003,25,2],
[14004,0,1],[14004,1,2],[14004,9,2],[14004,10,4],[14004,12,4],
[14004,13,2],[14004,14,1],[14004,16,1],[14004,17,1],[14004,18,1],
[14004,19,3],[14004,20,1],[14004,29,2],[14004,30,2],[14004,36,1],
[14004,37,2],[14004,41,1],[14004,45,1],[14004,53,1],[14004,59,1],
[14004,65,2],[14004,67,3],[14004,73,1],[14005,22,1],[14005,23,1],
[14005,26,1],[14005,27,2],[14005,72,1],[14005,75,1],[14006,3,1],
[14006,28,1],[14007,6,1],[14007,23,3],[14007,24,40],[14007,28,5],
[14007,33,1],[14008,0,1],[14008,1,1],[14008,2,2],[14008,4,7],
[14008,5,4],[14008,8,1],[14008,9,6],[14008,13,1],[14008,15,42],
[14008,17,56],[14008,19,1],[14008,21,1],[14008,22,1],[14008,23,2],

[14008,25,2],[14008,26,1],[14008,27,2],[14008,28,12],[14008,30,1],
[14008,31,1],[14008,35,6],[14008,37,1],[14008,38,2],[14008,39,2],
[14008,40,1],[14008,42,3],[14008,43,2],[14008,44,1],[14008,47,1],
[14008,50,6],[14008,52,1],[14008,53,70],[14008,55,22],[14008,59,1],
[14008,62,1],[14008,66,2],[14008,74,2],[14008,76,1],[14008,78,1],
[14009,69,2],[14009,70,1],[14010,12,1],[14010,22,1],[14010,23,1],
[14010,59,1],[14011,25,1],[14011,28,17],[14011,33,1],[14011,52,1],
[14012,21,2],[14013,0,2],[14013,1,6],[14013,9,11],[14013,10,13],
[14013,11,4],[14013,12,2],[14013,13,5],[14013,15,8],[14013,16,2],
[14013,17,26],[14013,18,2],[14013,19,6],[14013,20,4],[14013,28,3],
[14013,29,2],[14013,30,5],[14013,34,2],[14013,36,1],[14013,37,2],
[14013,40,2],[14013,41,7],[14013,45,7],[14013,46,3],[14013,50,1],
[14013,53,33],[14013,55,11],[14013,57,3],[14013,58,1],[14013,59,3],
[14013,60,1],[14013,65,9],[14013,67,6],[14013,70,1],[14013,71,1],
[14013,72,1],[14013,73,1],[14013,74,1],[14013,77,5],[14014,4,1],
[14014,28,9],[14015,2,1],[14015,35,1],[14016,31,2],[14017,24,2],
[14017,27,1],[14017,28,1],[14017,31,1],[14017,42,1],[14017,44,1],
[14017,47,1],[14018,5,1],[14018,20,1],[14018,33,1],[14018,41,1],
[14018,42,1],[14019,0,1],[14019,1,4],[14019,3,1],[14019,4,1],
[14019,5,1],[14019,6,4],[14019,10,1],[14019,13,2],[14019,14,2],
[14019,15,1],[14019,17,1],[14019,20,1],[14019,21,1],[14019,22,5],
[14019,23,3],[14019,24,13],[14019,26,4],[14019,27,2],[14019,28,5],
[14019,32,2],[14019,33,3],[14019,35,2],[14019,38,2],[14019,40,1],
[14019,43,3],[14019,44,3],[14019,47,4],[14019,48,1],[14019,50,1],
[14019,51,7],[14019,53,1],[14019,56,5],[14019,59,2],[14019,62,1],
[14019,66,2],[14019,68,3],[14019,69,2],[14019,70,4],[14019,74,8],
[14019,76,1],[14019,77,1],[14020,8,2],[14020,17,2],[14020,77,1],
[14021,27,1],[14021,38,1],[14022,28,1],[14022,42,1],[14023,1,1],
[14023,13,2],[14024,4,1],[14024,6,1],[14024,15,2],[14024,44,2],
[14024,55,1],[14024,66,1],[14024,67,1],[14025,66,2],[14026,2,1],
[14026,3,2],[14026,5,1],[14026,21,5],[14026,23,13],[14026,24,2],
[14026,25,5],[14026,26,2],[14026,27,8],[14026,33,3],[14026,35,3],
[14026,43,1],[14026,44,1],[14026,56,1],[14026,59,2],[14027,5,1],
[14027,6,2],[14027,24,1],[14027,47,1],[14027,51,2],[14027,69,1],
[14027,70,3],[14027,74,5],[14028,35,2],[14029,28,1],[14029,68,1],
[14030,8,1],[14030,9,2],[14030,10,4],[14030,11,1],[14030,12,1],
[14030,13,1],[14030,14,1],[14030,16,1],[14030,19,1],[14030,20,1],
[14030,37,1],[14030,57,1],[14030,58,2],[14030,65,2],[14030,71,2],
[14030,77,2],[14031,28,2],[14031,40,1],[14031,68,3],[14031,69,1],
[14032,21,1],[14032,26,1],[14033,15,1],[14033,55,1],[14034,2,2],
[14035,4,3],[14035,15,1],[14035,17,6],[14035,24,1],[14035,44,2],
[14035,50,4],[14035,53,8],[14035,55,11],[14036,5,3],[14036,10,1],
[14036,14,1],[14036,21,1],[14036,22,4],[14036,24,1],[14036,25,3],
[14036,27,5],[14036,28,2],[14036,30,2],[14036,31,1],[14036,33,14],
[14036,35,19],[14036,38,5],[14036,40,2],[14036,44,1],[14036,46,1],
[14036,52,42],[14036,56,1],[14036,58,1],[14036,62,1],[14036,66,1],
[14036,68,1],[14036,70,1],[14036,76,3],[14037,13,1],[14037,17,1],
[14037,23,2],[14037,26,1],[14037,28,1],[14037,31,1],[14037,35,2],
[14037,42,1],[14037,78,1],[14038,9,3],[14038,10,3],[14038,11,2],
[14038,13,1],[14038,18,1],[14038,19,3],[14038,34,1],[14038,37,1],

[14038,41,4],[14038,57,1],[14038,65,1],[14038,67,2],[14038,73,1],
[14039,0,1],[14039,1,18],[14039,10,1],[14039,11,1],[14039,12,2],
[14039,13,6],[14039,15,8],[14039,18,1],[14039,19,2],[14039,20,11],
[14039,50,1],[14039,53,2],[14039,55,16],[14039,59,4],[14039,60,2],
[14039,67,1],[14039,77,1],[14040,5,1],[14040,24,2],[14040,27,1],
[14040,32,1],[14040,40,1],[14040,43,1],[14040,63,1],[14041,19,1],
[14041,64,1],[14042,25,2],[14043,28,1],[14043,35,1],[14044,4,1],
[14044,24,1],[14044,26,1],[14044,42,1],[14044,56,1],[14045,28,1],
[14045,35,1],[14045,38,1],[14045,55,1],[14045,56,1],[14046,22,1],
[14046,25,1],[14046,26,1],[14047,26,3],[14048,1,1],[14048,3,2],
[14048,6,1],[14048,11,1],[14048,13,2],[14048,20,7],[14048,22,3],
[14048,23,1],[14048,24,1],[14048,26,2],[14048,27,4],[14048,28,1],
[14048,30,1],[14048,33,1],[14048,38,4],[14048,45,2],[14048,47,1],
[14048,51,1],[14048,68,1],[14048,70,1],[14048,77,1],[14049,3,1],
[14049,27,1],[14049,56,13],[14050,1,1],[14050,2,1],[14050,3,1],
[14050,4,1],[14050,5,1],[14050,10,1],[14050,15,2],[14050,21,2],
[14050,22,1],[14050,23,8],[14050,24,1],[14050,26,1],[14050,27,4],
[14050,28,1],[14050,30,1],[14050,32,1],[14050,33,1],[14050,35,1],
[14050,44,3],[14050,47,2],[14050,49,1],[14050,51,2],[14050,56,1],
[14050,66,1],[14050,68,2],[14050,69,2],[14050,74,2],[14050,75,1],
[14051,28,2],[14051,56,1],[14052,3,1],[14052,4,2],[14052,21,1],
[14052,22,1],[14052,23,1],[14052,24,1],[14052,28,8],[14052,31,1],
[14052,33,2],[14052,38,182],[14052,40,1],[14052,48,1],[14052,62,1],
[14052,66,1],[14052,68,1],[14053,45,2],[14053,55,1],[14054,23,1],
[14054,28,2],[14055,27,2],[14056,28,1],[14056,33,3],[14057,1,2],
[14057,7,1],[14057,9,2],[14057,10,5],[14057,12,1],[14057,13,1],
[14057,14,3],[14057,16,1],[14057,19,4],[14057,20,2],[14057,30,4],
[14057,36,1],[14057,37,7],[14057,41,2],[14057,46,1],[14057,54,1],
[14057,55,3],[14057,57,1],[14057,58,3],[14057,59,1],[14057,60,1],
[14057,65,3],[14057,67,2],[14057,77,2],[14058,6,1],[14058,22,2],
[14059,5,1],[14059,23,1],[14059,25,1],[14059,32,1],[14060,2,1],
[14060,17,1],[14060,22,1],[14060,28,94],[14060,38,1],[14060,42,1],
[14060,66,1],[14060,69,1],[14060,74,1],[14060,76,1],[14061,22,1],
[14061,23,2],[14061,35,1],[14062,23,1],[14062,27,1],[14063,24,1],
[14063,50,1],[14064,1,1],[14064,55,1],[14065,23,2],[14065,31,1],
[14066,6,1],[14066,42,1],[14067,4,1],[14067,15,1],[14067,17,1],
[14068,32,10],[14068,35,1],[14069,23,2],[14070,2,6],[14071,2,2],
[14071,3,23],[14071,5,8],[14071,6,13],[14071,13,5],[14071,14,1],
[14071,16,1],[14071,19,1],[14071,20,39],[14071,21,21],[14071,22,33],
[14071,23,30],[14071,24,49],[14071,25,9],[14071,26,14],[14071,27,62],
[14071,28,20],[14071,29,1],[14071,31,14],[14071,32,45],[14071,33,31],
[14071,35,28],[14071,37,13],[14071,38,4],[14071,39,4],[14071,42,16],
[14071,43,26],[14071,46,3],[14071,47,13],[14071,48,20],[14071,50,1],
[14071,55,1],[14071,56,24],[14071,57,1],[14071,59,10],[14071,60,2],
[14071,62,1],[14071,68,2],[14071,69,1],[14071,70,2],[14071,72,3],
[14071,75,1],[14071,76,2],[14071,77,1],[14072,1,1],[14072,17,1],
[14072,23,1],[14072,27,2],[14072,28,4],[14072,30,1],[14072,31,1],
[14072,33,1],[14072,35,2],[14072,40,1],[14072,42,1],[14072,44,2],
[14072,47,2],[14072,52,1],[14072,55,2],[14072,61,1],[14072,63,2],
[14072,66,1],[14072,68,1],[14072,70,2],[14072,74,3],[14072,76,1],

[14073,4,1],[14073,28,40],[14073,69,1],[14073,74,8],[14073,75,1],
[14074,25,1],[14074,32,2],[14075,28,4],[14076,2,3],[14076,28,2],
[14077,32,1],[14077,33,1],[14078,8,3],[14078,10,1],[14078,20,6],
[14078,22,4],[14078,23,2],[14078,24,1],[14078,27,1],[14078,28,1],
[14078,29,1],[14078,32,2],[14078,33,1],[14078,34,2],[14078,35,5],
[14078,42,1],[14078,59,3],[14079,2,2],[14079,66,1],[14080,4,1],
[14080,11,1],[14080,42,1],[14080,52,1],[14080,61,4],[14080,66,1],
[14081,2,5],[14081,3,1],[14081,4,1],[14081,5,5],[14081,6,1],
[14081,8,3],[14081,20,4],[14081,22,1],[14081,24,5],[14081,25,1],
[14081,26,1],[14081,27,1],[14081,33,2],[14081,38,1],[14081,39,1],
[14081,42,1],[14081,43,1],[14081,46,1],[14081,47,1],[14081,50,1],
[14081,55,1],[14081,60,1],[14081,62,1],[14082,5,1],[14082,28,4],
[14083,56,2],[14083,66,2],[14084,8,2],[14084,57,1],[14085,5,1],
[14085,21,1],[14085,22,1],[14085,23,1],[14085,35,2],[14085,38,1],
[14086,28,1],[14086,53,1],[14087,3,1],[14087,23,1],[14087,32,1],
[14088,28,3],[14088,32,4],[14089,2,1],[14089,4,1],[14089,23,3],
[14089,26,1],[14089,28,1],[14089,42,1],[14089,44,1],[14089,48,1],
[14089,50,1],[14089,66,1],[14090,3,1],[14090,24,1],[14091,62,3],
[14092,25,1],[14092,28,1],[14092,47,1],[14092,70,1],[14093,1,1],
[14093,3,1],[14093,8,1],[14093,11,1],[14093,13,2],[14093,15,5],
[14093,20,23],[14093,23,2],[14093,24,3],[14093,25,3],[14093,27,4],
[14093,30,1],[14093,31,1],[14093,33,4],[14093,35,5],[14093,38,2],
[14093,39,2],[14093,40,1],[14093,42,1],[14093,44,1],[14093,46,1],
[14093,50,1],[14093,53,1],[14093,54,1],[14093,55,3],[14093,62,1],
[14094,15,4],[14094,55,7],[14094,57,1],[14095,28,2],[14095,31,1],
[14096,22,6],[14096,32,1],[14097,18,1],[14097,30,1],[14097,59,1],
[14098,24,1],[14098,46,1],[14099,2,3],[14099,15,1],[14099,28,1],
[14099,43,1],[14100,42,2],[14101,2,2],[14102,2,4],[14102,60,1],
[14103,1,2],[14103,11,1],[14103,13,1],[14103,15,1],[14103,19,3],
[14103,30,1],[14103,36,1],[14103,41,1],[14103,45,1],[14103,60,1],
[14103,78,1],[14104,13,1],[14104,19,1],[14104,20,1],[14104,45,1],
[14104,53,1],[14104,54,1],[14104,65,1],[14104,73,1],[14104,77,1],
[14105,22,1],[14105,32,1],[14106,28,3],[14106,38,2],[14106,70,1],
[14107,56,2],[14108,32,3],[14109,2,1],[14109,3,1],[14109,5,1],
[14109,7,1],[14109,10,1],[14109,11,1],[14109,13,1],[14109,15,2],
[14109,20,19],[14109,21,1],[14109,22,10],[14109,23,90],[14109,24,24],
[14109,25,4],[14109,26,2],[14109,28,7],[14109,30,1],[14109,31,10],
[14109,33,3],[14109,35,20],[14109,39,1],[14109,42,1],[14109,46,2],
[14109,48,1],[14109,49,1],[14109,50,1],[14109,52,1],[14109,57,1],
[14109,59,25],[14109,66,1],[14109,73,1],[14110,0,1],[14110,1,157],
[14110,2,14],[14110,3,4],[14110,4,2],[14110,5,1],[14110,6,1],
[14110,9,4],[14110,10,4],[14110,12,1],[14110,13,48],[14110,14,1],
[14110,15,4],[14110,16,1],[14110,17,2],[14110,18,2],[14110,19,7],
[14110,20,7],[14110,21,1],[14110,22,4],[14110,23,19],[14110,24,6],
[14110,25,4],[14110,26,9],[14110,27,9],[14110,28,5],[14110,30,16],
[14110,31,13],[14110,32,3],[14110,33,11],[14110,35,1],[14110,36,1],
[14110,37,2],[14110,39,11],[14110,40,1],[14110,41,3],[14110,42,8],
[14110,43,12],[14110,44,1],[14110,45,3],[14110,46,3],[14110,47,5],
[14110,50,1],[14110,53,2],[14110,54,1],[14110,55,3],[14110,56,1],
[14110,57,4],[14110,58,3],[14110,59,13],[14110,60,10],[14110,62,1],

[14110,65,4],[14110,67,1],[14110,73,1],[14110,75,1],[14110,77,13],
[14110,78,1],[14111,13,1],[14111,27,1],[14112,27,4],[14113,28,10],
[14113,34,1],[14114,31,2],[14115,28,9],[14116,1,1],[14116,11,1],
[14116,16,1],[14116,73,1],[14116,77,1],[14117,26,2],[14117,28,1],
[14117,43,1],[14118,1,6],[14118,3,5],[14118,13,2],[14118,18,1],
[14118,24,1],[14118,28,2],[14118,37,1],[14118,59,1],[14118,64,1],
[14118,74,1],[14118,75,1],[14119,21,1],[14119,62,1],[14120,5,1],
[14120,23,1],[14120,28,5],[14120,34,2],[14120,47,1],[14121,33,1],
[14121,48,1],[14122,5,1],[14122,27,1],[14123,56,1],[14123,63,2],
[14123,68,1],[14123,70,1],[14123,74,2],[14123,75,3],[14123,76,2],
[14124,19,1],[14124,20,1],[14124,28,1],[14124,30,2],[14124,55,1],
[14125,3,1],[14125,5,1],[14125,22,4],[14125,23,1],[14125,24,3],
[14125,26,1],[14125,27,2],[14125,28,2],[14125,31,2],[14125,35,2],
[14125,42,2],[14125,43,2],[14125,47,1],[14126,23,1],[14126,28,1],
[14126,30,1],[14126,50,1],[14127,0,7],[14127,1,1],[14127,2,34],
[14127,8,63],[14127,9,2],[14127,10,2],[14127,12,3],[14127,20,1],
[14127,21,5],[14127,23,2],[14127,24,3],[14127,25,12],[14127,28,26],
[14127,29,2],[14127,31,54],[14127,34,17],[14127,35,4],[14127,36,11],
[14127,42,6],[14127,43,6],[14127,44,1],[14127,47,1],[14127,53,1],
[14127,55,1],[14127,56,2],[14127,62,6],[14127,65,2],[14127,67,1],
[14127,68,2],[14127,71,1],[14127,73,2],[14128,2,1],[14128,5,1],
[14128,28,1],[14129,35,1],[14129,56,1],[14130,22,1],[14130,46,1],
[14131,27,3],[14132,5,1],[14132,28,8],[14133,31,2],[14134,21,1],
[14134,31,1],[14135,21,2],[14136,44,1],[14136,51,10],[14137,25,1],
[14137,27,1],[14137,32,1],[14138,4,1],[14138,31,2],[14139,1,1],
[14139,10,1],[14139,15,1],[14140,0,2],[14140,13,1],[14141,4,1],
[14141,17,2],[14141,53,1],[14142,8,1],[14142,19,1],[14143,5,1],
[14143,21,2],[14143,24,1],[14143,27,2],[14143,32,1],[14143,47,1],
[14143,79,1],[14144,0,2],[14144,2,2],[14144,14,1],[14144,20,1],
[14145,6,10],[14145,21,1],[14145,22,10],[14145,23,1],[14145,24,38],
[14145,25,137],[14145,26,12],[14145,27,31],[14145,28,5],[14145,31,1],
[14145,32,1],[14145,35,39],[14145,43,1],[14145,56,1],[14145,66,1],
[14145,74,1],[14146,23,3],[14147,3,1],[14147,6,1],[14147,21,1],
[14147,23,2],[14147,31,5],[14147,33,1],[14147,37,1],[14147,42,2],
[14147,47,2],[14147,56,8],[14147,60,1],[14147,74,1],[14148,24,1],
[14148,25,2],[14148,28,1],[14148,33,17],[14148,35,8],[14148,38,4],
[14148,52,48],[14149,28,1],[14149,68,1],[14149,70,1],[14150,6,1],
[14150,23,2],[14150,24,1],[14150,27,2],[14150,28,13],[14150,35,1],
[14150,40,2],[14150,44,1],[14150,63,1],[14150,66,1],[14150,68,1],
[14150,70,1],[14150,76,1],[14151,25,2],[14152,4,4],[14152,27,1],
[14152,38,1],[14152,50,7],[14152,66,1],[14152,72,1],[14153,2,2],
[14154,23,1],[14154,42,1],[14154,44,1],[14154,52,1],[14154,61,1],
[14154,70,1],[14154,76,1],[14155,20,1],[14155,31,2],[14156,2,1],
[14156,30,1],[14157,11,1],[14157,18,1],[14158,23,1],[14158,25,5],
[14158,28,2],[14159,24,1],[14159,35,1],[14160,31,8],[14160,33,2],
[14161,2,14],[14162,24,1],[14162,28,2],[14162,35,1],[14163,2,3],
[14163,3,4],[14163,62,5],[14163,63,1],[14164,3,2],[14164,20,1],
[14164,28,1],[14164,51,1],[14164,56,6],[14165,32,7],[14166,25,2],
[14167,20,4],[14167,25,2],[14167,32,1],[14167,42,1],[14167,53,1],
[14167,55,1],[14168,23,1],[14168,38,1],[14169,63,2],[14170,1,1],

[14170,9,1],[14170,10,1],[14170,25,9],[14170,28,1],[14170,45,1],
[14170,73,1],[14171,4,1],[14171,44,2],[14172,22,1],[14172,33,1],
[14172,35,2],[14172,48,1],[14172,56,3],[14173,0,1],[14173,23,1],
[14173,27,1],[14173,37,2],[14173,56,1],[14174,28,3],[14174,33,1],
[14175,28,1],[14175,43,1],[14175,44,1],[14176,5,1],[14176,23,1],
[14176,24,1],[14176,27,1],[14176,31,1],[14176,62,1],[14177,17,1],
[14177,53,1],[14178,2,1],[14178,3,1],[14178,21,1],[14178,22,4],
[14178,23,1],[14178,24,2],[14178,27,2],[14178,28,4],[14178,33,1],
[14179,27,1],[14179,32,1],[14179,35,1],[14180,8,1],[14180,13,2],
[14180,14,1],[14180,15,1],[14180,17,1],[14180,18,1],[14180,19,1],
[14180,23,1],[14180,28,3],[14180,39,1],[14180,41,1],[14180,54,1],
[14180,55,1],[14180,74,1],[14180,75,1],[14180,77,3],[14181,11,1],
[14181,59,1],[14182,22,1],[14182,51,1],[14183,21,1],[14183,26,2],
[14183,31,2],[14183,35,1],[14183,62,1],[14184,25,2],[14184,42,1],
[14185,23,17],[14185,29,1],[14186,3,1],[14186,26,1],[14186,28,2],
[14186,32,1],[14186,35,1],[14186,37,3],[14186,38,1],[14186,41,1],
[14186,47,3],[14186,56,4],[14186,61,2],[14186,62,3],[14186,72,1],
[14187,22,2],[14187,24,2],[14187,26,1],[14188,3,3],[14188,5,1],
[14188,23,1],[14188,27,4],[14188,70,1],[14189,27,1],[14189,31,1],
[14189,62,1],[14190,3,1],[14190,27,1],[14190,28,6],[14191,22,1],
[14191,25,31],[14191,35,1],[14191,43,5],[14192,5,1],[14192,25,5],
[14192,28,1],[14192,56,1],[14193,2,1],[14193,37,2],[14194,1,2],
[14194,29,1],[14194,78,1],[14195,21,1],[14195,25,1],[14195,26,2],
[14195,75,1],[14196,0,8],[14196,2,38],[14196,5,1],[14196,8,1],
[14196,12,1],[14196,20,1],[14196,23,2],[14196,24,1],[14196,26,1],
[14196,30,3],[14196,31,4],[14196,32,6],[14196,38,1],[14196,39,4],
[14196,42,6],[14196,43,1],[14196,47,2],[14196,48,1],[14197,3,1],
[14197,5,1],[14197,28,2],[14197,32,1],[14197,47,1],[14198,5,3],
[14199,22,456],[14199,28,8],[14199,32,73],[14199,62,4],[14199,68,1],
[14200,13,1],[14200,19,3],[14200,49,1],[14200,64,2],[14201,22,1],
[14201,23,1],[14201,24,1],[14201,28,1],[14201,68,1],[14202,3,1],
[14202,26,1],[14202,27,1],[14202,28,17],[14202,44,1],[14202,66,1],
[14203,1,1],[14203,10,1],[14203,13,3],[14203,20,1],[14203,25,2],
[14203,28,13],[14203,38,1],[14203,48,1],[14203,56,2],[14203,59,1],
[14203,63,1],[14204,14,1],[14204,28,1],[14205,5,1],[14205,23,2],
[14205,26,1],[14205,32,1],[14205,39,2],[14205,43,1],[14206,1,2],
[14206,2,7],[14206,3,2],[14206,13,5],[14206,20,1],[14206,23,2],
[14206,24,4],[14206,25,2],[14206,27,3],[14206,28,3],[14206,31,1],
[14206,32,4],[14206,35,5],[14206,37,1],[14206,42,2],[14206,43,1],
[14206,56,1],[14206,70,1],[14206,74,1],[14206,75,1],[14206,78,1],
[14207,5,1],[14207,28,1],[14208,5,1],[14208,21,1],[14208,24,1],
[14208,35,4],[14208,42,2],[14208,43,2],[14209,23,1],[14209,27,13],
[14209,72,1],[14210,4,4],[14210,15,2],[14210,17,12],[14210,44,3],
[14210,50,4],[14210,53,11],[14210,55,11],[14211,35,2],[14211,43,1],
[14211,74,1],[14212,15,1],[14212,23,1],[14212,68,1],[14213,1,1],
[14213,42,1],[14214,5,1],[14214,28,1],[14214,62,1],[14214,70,1],
[14215,23,1],[14215,25,1],[14215,43,2],[14216,28,14],[14217,75,2],
[14217,76,1],[14218,15,1],[14218,51,1],[14219,32,2],[14220,5,1],
[14220,49,1],[14220,50,1],[14221,22,2],[14221,24,2],[14221,27,2],
[14222,23,1],[14222,24,1],[14222,43,1],[14223,30,2],[14223,48,3],

[14224,20,1],[14224,21,1],[14224,27,1],[14224,35,1],[14224,43,1],
[14224,47,3],[14224,48,2],[14225,5,1],[14225,23,1],[14226,15,1],
[14226,53,1],[14227,17,1],[14227,35,1],[14227,60,2],[14227,78,1],
[14228,35,1],[14228,67,1],[14229,3,1],[14229,24,1],[14229,32,3],
[14229,61,1],[14230,6,1],[14230,32,1],[14230,43,1],[14230,62,1],
[14231,28,2],[14232,10,1],[14232,23,1],[14232,27,2],[14232,45,1],
[14233,31,2],[14234,23,2],[14235,28,2],[14236,34,12],[14236,62,1],
[14237,17,1],[14237,50,1],[14237,53,2],[14237,66,1],[14238,22,2],
[14238,23,2],[14238,24,1],[14238,26,1],[14238,27,1],[14238,28,1],
[14238,30,1],[14238,32,1],[14238,39,2],[14239,27,4],[14240,6,2],
[14240,51,1],[14240,68,1],[14241,13,1],[14241,62,1],[14242,9,1],
[14242,30,1],[14243,0,1],[14243,14,1],[14243,27,4],[14243,62,2],
[14244,42,2],[14245,28,2],[14246,42,1],[14246,62,1],[14247,0,1],
[14247,1,6],[14247,2,12],[14247,4,2],[14247,5,4],[14247,7,2],
[14247,8,4],[14247,9,5],[14247,10,2],[14247,11,1],[14247,12,6],
[14247,13,1],[14247,14,1],[14247,15,3],[14247,17,9],[14247,19,1],
[14247,20,9],[14247,22,9],[14247,23,13],[14247,24,6],[14247,25,6],
[14247,26,3],[14247,27,11],[14247,28,6],[14247,29,2],[14247,30,13],
[14247,31,8],[14247,32,13],[14247,33,1],[14247,35,1],[14247,37,3],
[14247,39,2],[14247,41,2],[14247,42,14],[14247,43,9],[14247,45,2],
[14247,47,3],[14247,50,3],[14247,51,1],[14247,53,9],[14247,55,1],
[14247,56,3],[14247,58,1],[14247,59,2],[14247,61,2],[14247,62,5],
[14247,65,1],[14247,66,1],[14247,67,4],[14247,71,1],[14247,73,5],
[14247,77,1],[14248,1,2],[14248,2,1],[14248,8,1],[14248,12,10],
[14248,34,1],[14248,53,1],[14249,66,1],[14249,74,1],[14250,2,3],
[14250,5,2],[14250,22,1],[14250,23,3],[14250,24,5],[14250,25,4],
[14250,26,4],[14250,31,1],[14250,32,1],[14250,33,4],[14250,42,1],
[14250,43,2],[14250,68,1],[14251,17,1],[14251,24,1],[14251,28,1],
[14251,31,2],[14251,35,1],[14252,3,1],[14252,21,2],[14252,22,9],
[14252,23,1],[14252,24,2],[14252,25,1],[14252,26,2],[14252,27,3],
[14252,31,3],[14252,33,1],[14252,34,1],[14252,43,3],[14253,2,2],
[14253,28,1],[14254,25,2],[14255,2,45],[14255,23,1],[14255,28,3],
[14255,31,3],[14255,33,1],[14255,39,1],[14255,62,1],[14256,42,2],
[14257,37,5],[14257,56,1],[14257,57,1],[14258,9,4],[14258,15,2],
[14258,20,1],[14258,28,1],[14258,29,1],[14258,37,1],[14258,41,1],
[14258,55,3],[14258,65,1],[14258,77,1],[14259,20,1],[14259,41,1],
[14259,47,1],[14260,17,1],[14260,53,1],[14261,13,1],[14261,20,3],
[14261,60,1],[14262,0,1],[14262,2,2],[14262,3,1],[14262,4,3],
[14262,5,6],[14262,6,4],[14262,9,1],[14262,10,1],[14262,11,8],
[14262,13,3],[14262,15,2],[14262,17,1],[14262,19,1],[14262,20,11],
[14262,21,3],[14262,22,3],[14262,23,2],[14262,24,6],[14262,25,3],
[14262,26,2],[14262,27,6],[14262,28,15],[14262,29,1],[14262,31,4],
[14262,32,2],[14262,33,2],[14262,35,4],[14262,37,1],[14262,38,1],
[14262,39,1],[14262,40,1],[14262,42,3],[14262,44,1],[14262,45,1],
[14262,46,2],[14262,47,5],[14262,50,1],[14262,51,9],[14262,56,14],
[14262,61,4],[14262,62,13],[14262,63,2],[14262,68,7],[14262,69,14],
[14262,70,53],[14262,71,1],[14262,74,15],[14262,75,3],[14262,76,1],
[14262,77,5],[14263,28,1],[14263,32,1],[14264,2,1],[14264,24,2],
[14265,28,5],[14266,44,1],[14266,66,1],[14267,1,6],[14267,12,1],
[14267,21,1],[14267,23,1],[14267,26,1],[14267,27,1],[14267,30,1],

[14267,33,2],[14267,39,1],[14267,47,2],[14268,28,1],[14268,32,1],
[14269,9,1],[14269,23,1],[14269,24,1],[14269,32,8],[14269,37,1],
[14269,47,1],[14269,48,1],[14269,57,1],[14269,70,1],[14270,5,1],
[14270,35,3],[14271,23,1],[14271,25,3],[14271,26,1],[14271,31,1],
[14271,37,1],[14271,41,1],[14271,62,2],[14271,70,1],[14271,78,1],
[14272,5,1],[14272,22,1],[14272,23,1],[14272,25,2],[14272,27,2],
[14272,35,2],[14272,47,2],[14272,48,1],[14272,76,1],[14273,31,5],
[14274,0,1],[14274,4,18],[14274,5,3],[14274,7,1],[14274,17,5],
[14274,23,1],[14274,24,1],[14274,25,2],[14274,28,3],[14274,30,1],
[14274,35,1],[14274,44,1],[14274,50,21],[14274,53,4],[14274,55,2],
[14274,62,2],[14274,63,1],[14275,1,2],[14275,2,4],[14275,3,10],
[14275,4,2],[14275,6,1],[14275,8,5],[14275,10,1],[14275,12,1],
[14275,13,1],[14275,15,2],[14275,17,7],[14275,19,1],[14275,20,20],
[14275,21,2],[14275,23,4],[14275,24,1],[14275,27,3],[14275,28,9],
[14275,29,1],[14275,30,6],[14275,31,32],[14275,34,1],[14275,35,1],
[14275,36,1],[14275,37,2],[14275,39,2],[14275,40,2],[14275,43,2],
[14275,46,1],[14275,48,1],[14275,50,2],[14275,53,5],[14275,55,4],
[14275,56,2],[14275,59,4],[14275,60,2],[14275,63,2],[14275,65,5],
[14275,66,1],[14275,72,1],[14275,73,1],[14275,74,1],[14276,1,1],
[14276,10,1],[14276,12,1],[14276,71,1],[14277,28,1],[14277,53,1],
[14278,30,1],[14278,53,1],[14278,55,1],[14278,58,1],[14279,24,2],
[14280,9,1],[14280,11,1],[14281,28,2],[14282,0,5],[14282,1,72],
[14282,7,86],[14282,8,12],[14282,9,3],[14282,10,2],[14282,11,1],
[14282,12,213],[14282,13,31],[14282,15,5],[14282,17,15],[14282,20,4],
[14282,24,1],[14282,25,1],[14282,27,1],[14282,29,82],[14282,30,12],
[14282,34,11],[14282,35,2],[14282,36,8],[14282,37,1],[14282,39,35],
[14282,41,1],[14282,45,3],[14282,46,1],[14282,53,12],[14282,55,9],
[14282,59,3],[14282,65,1],[14282,73,37],[14283,15,1],[14283,50,1],
[14283,55,1],[14283,67,1],[14283,74,1],[14283,75,1],[14284,27,2],
[14284,28,1],[14285,24,1],[14285,35,1],[14286,32,1],[14286,72,1],
[14287,23,15],[14287,47,1],[14288,2,12],[14288,4,1],[14288,19,1],
[14288,23,1],[14288,24,2],[14288,28,2],[14288,31,1],[14288,37,6],
[14288,62,1],[14288,63,1],[14289,22,6],[14289,23,4],[14289,28,1],
[14289,35,3],[14289,45,1],[14290,27,1],[14290,47,1],[14290,59,1],
[14290,62,1],[14290,70,1],[14291,62,1],[14291,70,1],[14292,2,7],
[14292,28,1],[14293,31,5],[14294,28,1],[14294,50,1],[14295,20,1],
[14295,46,1],[14296,3,1],[14296,23,1],[14296,27,4],[14296,30,1],
[14297,5,1],[14297,24,1],[14297,25,1],[14298,37,1],[14298,59,1],
[14299,2,1],[14299,5,1],[14299,8,2],[14299,9,1],[14299,22,1],
[14299,26,1],[14299,34,1],[14299,57,1],[14300,3,1],[14300,72,1],
[14301,5,1],[14301,15,1],[14301,49,1],[14301,56,1],[14302,24,1],
[14302,28,2],[14302,38,1],[14303,8,2],[14303,22,2],[14303,27,1],
[14303,33,1],[14303,50,1],[14303,65,1],[14304,2,1],[14304,3,3],
[14304,8,1],[14304,13,2],[14304,17,1],[14304,20,3],[14304,24,1],
[14304,28,1],[14304,30,1],[14304,31,5],[14304,35,1],[14304,42,1],
[14304,53,1],[14304,64,2],[14304,69,1],[14304,70,2],[14304,75,1],
[14305,2,1],[14305,3,2],[14305,5,1],[14305,23,1],[14305,24,1],
[14305,27,1],[14305,28,1],[14305,30,2],[14305,35,1],[14305,38,1],
[14305,58,1],[14305,70,1],[14306,2,1],[14306,3,1],[14306,20,3],
[14306,23,1],[14306,26,1],[14306,34,2],[14306,47,1],[14306,54,1],

[14307,13,2],[14307,20,4],[14307,21,1],[14307,23,1],[14307,28,1],
[14307,31,1],[14307,33,1],[14307,35,1],[14307,39,1],[14307,43,2],
[14307,47,1],[14307,52,1],[14307,53,2],[14308,5,1],[14308,31,1],
[14309,15,1],[14309,27,1],[14309,28,5],[14309,32,2],[14309,48,1],
[14309,52,1],[14309,62,1],[14310,5,1],[14310,56,1],[14311,27,1],
[14311,28,1],[14311,38,1],[14311,44,7],[14311,66,9],[14312,21,1],
[14312,23,1],[14312,24,1],[14312,42,1],[14313,2,1],[14313,63,1],
[14314,9,1],[14314,53,1],[14315,1,1],[14315,2,12],[14315,3,3],
[14315,4,1],[14315,5,2],[14315,13,1],[14315,16,1],[14315,20,2],
[14315,21,4],[14315,22,2],[14315,23,4],[14315,25,1],[14315,26,3],
[14315,27,7],[14315,32,1],[14315,33,9],[14315,35,8],[14315,41,1],
[14315,42,6],[14315,50,1],[14315,65,1],[14315,70,1],[14315,76,1],
[14315,77,3],[14316,4,1],[14316,28,9],[14316,32,2],[14316,39,1],
[14317,1,2],[14317,7,1],[14317,13,1],[14317,15,3],[14317,17,2],
[14317,27,1],[14317,28,5],[14317,34,1],[14317,55,2],[14317,67,1],
[14318,70,4],[14319,24,1],[14319,40,1],[14319,41,1],[14319,46,1],
[14320,22,2],[14320,35,1],[14321,5,1],[14321,23,1],[14321,28,1],
[14321,32,1],[14321,47,1],[14321,62,2],[14322,0,1],[14322,1,3],
[14322,3,1],[14322,7,2],[14322,8,1],[14322,9,1],[14322,10,3],
[14322,11,1],[14322,12,5],[14322,15,4],[14322,18,5],[14322,19,4],
[14322,20,6],[14322,22,1],[14322,23,1],[14322,24,1],[14322,28,2],
[14322,33,1],[14322,34,1],[14322,36,4],[14322,37,4],[14322,38,1],
[14322,39,1],[14322,40,1],[14322,41,1],[14322,45,1],[14322,46,2],
[14322,52,1],[14322,53,4],[14322,55,4],[14322,57,3],[14322,59,3],
[14322,62,1],[14322,63,3],[14322,70,1],[14322,71,3],[14322,73,2],
[14322,74,13],[14322,75,4],[14322,76,21],[14322,77,3],[14323,2,22],
[14323,23,1],[14324,20,4],[14324,21,1],[14324,30,4],[14324,31,1],
[14324,34,5],[14324,44,1],[14325,28,10],[14326,20,1],[14326,23,1],
[14326,33,2],[14327,5,1],[14327,22,3],[14327,24,1],[14327,25,1],
[14327,26,1],[14327,28,3],[14327,44,1],[14328,24,4],[14328,26,1],
[14328,27,1],[14328,31,1],[14328,35,3],[14328,43,3],[14328,46,1],
[14328,53,1],[14328,69,3],[14328,74,1],[14329,15,1],[14329,20,1],
[14330,3,6],[14330,10,1],[14330,13,5],[14330,15,1],[14330,16,1],
[14330,20,4],[14330,22,2],[14330,23,8],[14330,25,8],[14330,26,6],
[14330,27,7],[14330,28,7],[14330,31,1],[14330,32,8],[14330,33,1],
[14330,35,1],[14330,42,1],[14330,44,1],[14330,58,1],[14330,72,1],
[14331,28,1],[14331,31,1],[14331,46,1],[14332,70,2],[14333,8,1],
[14333,23,1],[14333,40,1],[14333,59,1],[14333,78,1],[14334,4,1],
[14334,6,5],[14334,20,3],[14334,22,4],[14334,23,2],[14334,24,1],
[14334,27,6],[14334,32,1],[14334,33,5],[14334,35,11],[14334,42,3],
[14334,47,1],[14334,66,1],[14334,74,3],[14334,75,1],[14335,2,2],
[14335,11,1],[14336,33,7],[14337,2,6],[14337,3,10],[14337,4,1],
[14337,5,8],[14337,6,6],[14337,21,5],[14337,22,8],[14337,23,16],
[14337,24,72],[14337,25,15],[14337,26,8],[14337,27,25],[14337,28,16],
[14337,30,3],[14337,31,6],[14337,32,12],[14337,33,1],[14337,35,13],
[14337,38,5],[14337,39,2],[14337,42,2],[14337,43,3],[14337,47,8],
[14337,48,2],[14337,50,2],[14337,52,1],[14337,56,10],[14337,59,1],
[14337,62,2],[14338,1,6],[14338,2,51],[14338,3,21],[14338,4,1],
[14338,5,14],[14338,6,3],[14338,7,1],[14338,8,6],[14338,10,2],
[14338,12,1],[14338,13,2],[14338,14,1],[14338,15,1],[14338,17,4],

[14338,18,2],[14338,19,2],[14338,20,16],[14338,21,8],[14338,22,28],
[14338,23,62],[14338,24,48],[14338,25,21],[14338,26,44],[14338,27,56],
[14338,28,42],[14338,30,3],[14338,31,23],[14338,32,53],[14338,33,12],
[14338,34,6],[14338,35,29],[14338,36,1],[14338,37,3],[14338,38,3],
[14338,39,9],[14338,40,2],[14338,41,2],[14338,42,37],[14338,43,29],
[14338,44,1],[14338,47,21],[14338,48,9],[14338,50,4],[14338,52,1],
[14338,56,3],[14338,57,1],[14338,59,1],[14338,60,4],[14338,61,4],
[14338,62,11],[14338,63,1],[14338,64,1],[14338,66,3],[14338,67,1],
[14338,68,6],[14338,69,2],[14338,70,8],[14338,72,2],[14338,74,6],
[14338,75,1],[14338,76,4],[14338,77,3],[14338,78,1],[14339,1,2],
[14339,3,2],[14339,6,1],[14339,7,2],[14339,12,1],[14339,13,1],
[14339,25,2],[14339,41,1],[14339,47,2],[14339,50,1],[14339,56,2],
[14339,64,1],[14340,21,1],[14340,22,3],[14340,23,1],[14340,24,2],
[14340,25,1],[14340,33,1],[14340,40,1],[14340,51,1],[14341,1,2],
[14341,5,1],[14341,6,3],[14341,12,1],[14341,13,5],[14341,20,2],
[14341,24,1],[14341,26,1],[14341,31,1],[14341,36,1],[14341,40,1],
[14341,50,1],[14341,56,5],[14341,65,1],[14341,66,1],[14341,68,1],
[14341,69,1],[14341,72,1],[14342,23,1],[14342,33,1],[14343,2,1],
[14343,8,2],[14343,10,3],[14344,8,1],[14344,20,3],[14344,27,1],
[14344,35,1],[14344,36,2],[14345,12,1],[14345,24,2],[14345,35,1],
[14345,36,1],[14346,28,1],[14346,66,1],[14347,2,10],[14347,25,1],
[14348,3,2],[14348,4,4],[14348,5,2],[14348,10,1],[14348,14,2],
[14348,15,12],[14348,16,1],[14348,17,17],[14348,19,1],[14348,20,1],
[14348,21,1],[14348,23,3],[14348,24,3],[14348,27,3],[14348,28,4],
[14348,31,1],[14348,32,1],[14348,33,1],[14348,35,1],[14348,36,1],
[14348,37,1],[14348,40,2],[14348,41,1],[14348,44,1],[14348,45,2],
[14348,46,1],[14348,50,4],[14348,53,23],[14348,55,16],[14348,56,1],
[14348,58,2],[14348,61,1],[14348,62,1],[14348,63,5],[14348,65,1],
[14348,66,2],[14348,68,1],[14348,69,1],[14348,70,1],[14348,71,1],
[14348,72,1],[14348,73,1],[14348,74,2],[14348,75,2],[14348,76,1],
[14348,77,1],[14349,43,12],[14349,75,1],[14350,43,1],[14350,61,1],
[14351,23,2],[14351,47,1],[14351,68,2],[14352,9,1],[14352,34,1],
[14352,73,1],[14353,24,2],[14353,25,3],[14353,26,2],[14353,27,1],
[14353,28,1],[14353,35,5],[14353,43,2],[14353,47,1],[14354,27,3],
[14354,28,1],[14354,77,1],[14355,23,1],[14355,24,1],[14355,25,1],
[14355,27,1],[14355,32,2],[14356,2,9],[14356,3,1],[14356,9,1],
[14356,12,1],[14356,13,1],[14356,15,1],[14356,17,1],[14356,19,1],
[14356,20,1],[14356,21,15],[14356,22,1],[14356,25,6],[14356,26,1],
[14356,28,2],[14356,30,1],[14356,31,7],[14356,35,1],[14356,39,47],
[14356,42,18],[14356,43,15],[14356,47,1],[14356,48,2],[14356,60,1],
[14357,1,1],[14357,22,3],[14357,24,1],[14357,25,4],[14357,38,1],
[14358,9,1],[14358,59,1],[14359,2,6],[14359,3,5],[14359,5,1],
[14359,6,1],[14359,21,3],[14359,22,2],[14359,23,14],[14359,24,7],
[14359,25,3],[14359,26,3],[14359,27,16],[14359,28,20],[14359,31,14],
[14359,32,6],[14359,33,2],[14359,34,2],[14359,35,9],[14359,42,4],
[14359,43,6],[14359,47,10],[14359,48,2],[14359,56,3],[14359,59,5],
[14359,62,3],[14359,63,1],[14360,9,4],[14360,28,8],[14360,75,1],
[14361,2,1],[14361,23,1],[14361,24,1],[14361,27,1],[14362,3,1],
[14362,4,42],[14362,5,4],[14362,6,1],[14362,17,7],[14362,21,1],
[14362,22,4],[14362,23,1],[14362,24,8],[14362,25,2],[14362,26,1],

[14362,27,3],[14362,28,2],[14362,32,5],[14362,33,1],[14362,35,6],
[14362,36,1],[14362,38,1],[14362,39,1],[14362,42,1],[14362,47,1],
[14362,50,40],[14362,53,6],[14362,62,2],[14362,66,2],[14363,4,1],
[14363,15,3],[14363,17,2],[14363,20,1],[14363,44,1],[14363,50,1],
[14363,53,1],[14363,55,4],[14363,58,1],[14364,0,1],[14364,1,18],
[14364,4,1],[14364,8,1],[14364,11,1],[14364,12,66],[14364,13,3],
[14364,15,1],[14364,16,39],[14364,17,2],[14364,20,5],[14364,23,1],
[14364,25,1],[14364,29,32],[14364,30,5],[14364,34,2],[14364,36,5],
[14364,39,4],[14364,41,1],[14364,45,1],[14364,53,7],[14364,55,3],
[14364,59,2],[14364,71,1],[14364,73,1],[14365,28,2],[14366,31,2],
[14367,20,1],[14367,32,1],[14367,39,2],[14367,42,1],[14367,53,1],
[14368,22,1],[14368,23,1],[14368,25,1],[14368,26,1],[14368,33,6],
[14368,43,1],[14368,48,1],[14369,1,2],[14369,11,2],[14369,12,3],
[14369,16,1],[14369,18,1],[14369,19,1],[14369,20,4],[14369,23,1],
[14369,26,1],[14369,29,1],[14369,30,1],[14369,31,1],[14369,35,1],
[14369,37,1],[14369,38,37],[14369,39,2],[14369,40,1],[14369,55,2],
[14369,59,1],[14369,63,1],[14369,65,1],[14369,67,1],[14369,71,1],
[14369,77,2],[14370,1,4],[14370,5,1],[14370,13,2],[14370,19,1],
[14370,20,1],[14370,21,2],[14370,23,2],[14370,25,1],[14370,27,1],
[14370,30,1],[14370,31,1],[14370,32,1],[14370,34,2],[14370,39,2],
[14370,40,1],[14370,41,1],[14370,43,1],[14370,56,2],[14370,57,1],
[14370,62,2],[14371,13,3],[14371,20,1],[14371,24,1],[14371,34,1],
[14372,4,11],[14372,17,1],[14372,25,2],[14372,26,1],[14372,27,1],
[14372,28,1],[14372,38,2],[14372,43,1],[14372,44,15],[14372,50,11],
[14372,63,1],[14372,66,8],[14372,70,1],[14372,75,1],[14372,76,2],
[14373,23,2],[14374,60,3],[14375,3,1],[14375,22,3],[14375,32,2],
[14375,40,2],[14375,44,1],[14375,56,6],[14375,68,1],[14375,69,1],
[14375,70,2],[14376,1,6],[14376,2,1],[14376,3,17],[14376,5,3],
[14376,6,2],[14376,8,3],[14376,10,5],[14376,11,1],[14376,13,9],
[14376,14,2],[14376,15,2],[14376,16,1],[14376,18,2],[14376,19,1],
[14376,20,10],[14376,21,3],[14376,22,21],[14376,23,160],[14376,24,81],
[14376,25,8],[14376,26,29],[14376,27,18],[14376,28,65],[14376,31,12],
[14376,32,11],[14376,33,4],[14376,34,3],[14376,35,5],[14376,38,1],
[14376,41,2],[14376,42,6],[14376,43,6],[14376,44,2],[14376,45,2],
[14376,46,1],[14376,47,2],[14376,49,1],[14376,54,1],[14376,55,2],
[14376,58,2],[14376,59,6],[14376,60,1],[14376,62,3],[14376,64,1],
[14376,65,2],[14376,70,1],[14376,71,2],[14376,72,1],[14376,73,2],
[14376,76,1],[14376,78,1],[14377,2,1],[14377,3,2],[14377,5,3],
[14377,21,2],[14377,25,1],[14377,27,1],[14377,28,5],[14377,35,2],
[14377,39,1],[14377,47,3],[14377,50,1],[14377,51,8],[14377,59,2],
[14377,66,1],[14377,70,2],[14377,72,1],[14377,74,8],[14377,75,8],
[14377,76,12],[14378,9,1],[14378,12,1],[14378,59,1],[14379,27,2],
[14380,2,1],[14380,23,14],[14380,42,1],[14380,68,1],[14381,32,1],
[14381,51,2],[14382,24,2],[14382,25,1],[14382,27,1],[14382,33,1],
[14382,35,2],[14383,8,1],[14383,42,1],[14383,43,1],[14384,1,1],
[14384,10,3],[14384,22,1],[14384,23,1],[14384,26,1],[14384,43,1],
[14384,53,1],[14385,28,3],[14386,10,1],[14386,31,3],[14387,25,14],
[14387,28,1],[14388,28,1],[14388,62,6],[14389,28,1],[14389,62,1],
[14390,6,2],[14390,23,2],[14390,26,1],[14390,28,2],[14390,32,1],
[14390,35,1],[14390,38,2],[14390,40,1],[14390,44,4],[14390,47,3],

[14390,48,1],[14390,56,1],[14390,66,7],[14390,68,49],[14390,69,1],
[14390,70,5],[14390,74,3],[14390,75,1],[14390,76,2],[14391,14,1],
[14391,38,1],[14391,57,2],[14392,1,20],[14392,2,2],[14392,8,1],
[14392,12,1],[14392,13,6],[14392,17,1],[14392,20,1],[14392,23,1],
[14392,24,2],[14392,28,1],[14392,29,2],[14392,34,1],[14392,36,1],
[14392,39,1],[14392,45,1],[14392,47,2],[14392,48,2],[14392,53,3],
[14393,0,1],[14393,1,183],[14393,6,1],[14393,7,1],[14393,8,1],
[14393,9,1],[14393,10,1],[14393,12,2],[14393,13,5],[14393,15,1],
[14393,17,2],[14393,20,2],[14393,26,1],[14393,29,5],[14393,30,1],
[14393,34,3],[14393,36,2],[14393,39,7],[14393,45,1],[14393,53,3],
[14393,55,2],[14393,58,1],[14393,59,1],[14393,73,1],[14394,5,2],
[14394,47,1],[14394,56,1],[14395,1,1],[14395,9,2],[14395,17,1],
[14395,19,2],[14395,36,1],[14395,37,1],[14395,41,1],[14395,45,1],
[14395,58,1],[14395,59,1],[14395,65,2],[14395,71,1],[14395,73,1],
[14395,78,1],[14396,28,1],[14396,33,1],[14397,1,1],[14397,11,4],
[14398,22,6],[14398,35,4],[14399,1,1],[14399,8,1],[14400,3,1],
[14400,6,1],[14400,22,5],[14400,23,3],[14400,25,1],[14400,26,2],
[14400,27,9],[14400,32,2],[14400,35,3],[14400,42,5],[14400,43,2],
[14400,62,1],[14400,68,1],[14400,70,1],[14401,2,1],[14401,63,1],
[14402,2,6],[14402,4,1],[14402,5,1],[14402,8,1],[14402,16,1],
[14402,20,8],[14402,21,1],[14402,22,2],[14402,23,1],[14402,24,2],
[14402,26,1],[14402,27,2],[14402,30,1],[14402,33,1],[14402,35,1],
[14402,39,2],[14402,55,2],[14402,56,2],[14402,58,1],[14402,59,1],
[14402,62,2],[14402,64,1],[14402,70,1],[14402,71,1],[14402,74,1],
[14402,75,2],[14403,20,1],[14403,59,1],[14404,29,1],[14404,30,1],
[14404,38,1],[14404,58,1],[14405,24,10],[14405,25,2],[14406,70,1],
[14406,76,1],[14407,17,1],[14407,53,2],[14408,1,6],[14408,2,101],
[14408,3,32],[14408,4,6],[14408,5,26],[14408,6,8],[14408,8,2],
[14408,9,3],[14408,10,1],[14408,11,1],[14408,12,1],[14408,13,3],
[14408,16,1],[14408,17,6],[14408,18,1],[14408,19,2],[14408,20,14],
[14408,21,42],[14408,22,70],[14408,23,102],[14408,24,53],
[14408,25,99],[14408,26,79],[14408,27,81],[14408,28,29],[14408,30,14],
[14408,31,81],[14408,32,60],[14408,33,29],[14408,35,38],[14408,36,2],
[14408,37,4],[14408,38,6],[14408,39,21],[14408,40,11],[14408,42,48],
[14408,43,71],[14408,44,5],[14408,45,1],[14408,46,1],[14408,47,45],
[14408,48,32],[14408,50,3],[14408,51,3],[14408,52,4],[14408,53,12],
[14408,54,1],[14408,55,3],[14408,56,15],[14408,58,1],[14408,59,9],
[14408,60,11],[14408,61,1],[14408,62,21],[14408,63,5],[14408,64,1],
[14408,65,1],[14408,66,5],[14408,68,3],[14408,69,4],[14408,70,20],
[14408,71,1],[14408,72,6],[14408,73,2],[14408,74,6],[14408,75,4],
[14408,76,7],[14408,78,5],[14408,79,1],[14409,1,3],[14409,4,1],
[14409,13,1],[14409,17,1],[14409,20,3],[14409,24,3],[14409,25,697],
[14409,27,2],[14409,28,16],[14409,31,3],[14409,33,234],[14409,35,2],
[14409,39,1],[14409,42,12],[14409,51,1],[14409,59,1],[14410,2,9],
[14410,28,1],[14411,30,3],[14412,5,1],[14412,10,1],[14412,13,1],
[14412,15,1],[14412,19,1],[14412,39,1],[14412,44,1],[14412,52,13],
[14413,4,1],[14413,5,1],[14413,26,1],[14413,27,8],[14413,31,3],
[14413,50,1],[14413,51,1],[14413,57,1],[14413,68,1],[14413,72,1],
[14413,74,1],[14413,75,1],[14414,56,2],[14415,34,5],[14416,1,1],
[14416,6,1],[14416,23,1],[14416,31,2],[14416,48,1],[14417,10,1],

[14417,30,3],[14418,55,3],[14419,2,16],[14419,4,1],[14419,6,1],
[14419,31,2],[14419,32,1],[14419,49,1],[14419,68,1],[14419,76,1],
[14420,2,8],[14420,19,1],[14420,20,2],[14420,23,3],[14420,27,1],
[14420,48,1],[14421,2,4],[14421,23,1],[14421,24,1],[14421,28,2],
[14421,32,1],[14422,31,2],[14423,0,7],[14423,1,1],[14423,2,8],
[14423,7,1],[14423,9,4],[14423,10,3],[14423,12,5],[14423,13,6],
[14423,15,1],[14423,17,1],[14423,19,6],[14423,20,3],[14423,21,5],
[14423,28,2],[14423,29,2],[14423,30,1],[14423,31,42],[14423,32,1],
[14423,36,6],[14423,37,1],[14423,42,1],[14423,45,6],[14423,46,1],
[14423,49,1],[14423,52,2],[14423,54,3],[14423,57,1],[14423,58,1],
[14423,60,1],[14423,62,1],[14423,65,3],[14423,67,1],[14423,71,2],
[14423,73,3],[14423,77,4],[14424,40,4],[14425,2,3],[14425,26,1],
[14425,27,2],[14425,28,1],[14425,31,4],[14426,32,1],[14426,56,1],
[14427,26,1],[14427,27,2],[14428,26,5],[14429,53,1],[14429,55,1],
[14430,0,1],[14430,36,2],[14430,73,1],[14431,15,1],[14431,17,3],
[14431,53,4],[14432,3,1],[14432,23,1],[14432,35,2],[14432,38,1],
[14433,4,1],[14433,50,1],[14433,69,1],[14434,1,1],[14434,9,1],
[14434,10,2],[14434,16,1],[14434,53,1],[14434,59,1],[14435,2,1],
[14435,32,1],[14435,35,1],[14435,43,1],[14435,74,1],[14436,2,1],
[14436,31,6],[14437,25,2],[14438,1,1],[14438,3,1],[14438,28,2],
[14438,42,2],[14438,60,1],[14439,2,1],[14439,3,1],[14440,4,1],
[14440,5,1],[14440,21,1],[14440,22,1],[14440,25,1],[14440,50,1],
[14441,2,2],[14441,3,3],[14441,5,1],[14441,19,1],[14441,23,2],
[14441,24,7],[14441,25,4],[14441,26,1],[14441,27,5],[14441,28,1],
[14441,31,11],[14441,32,2],[14441,33,2],[14441,43,2],[14441,67,2],
[14442,1,79],[14442,3,2],[14442,9,3],[14442,10,1],[14442,11,1],
[14442,12,1],[14442,15,1],[14442,18,1],[14442,28,1],[14442,29,6],
[14442,36,1],[14442,43,1],[14442,44,1],[14442,46,1],[14442,48,3],
[14442,54,1],[14442,57,1],[14442,59,1],[14442,65,1],[14442,67,1],
[14443,20,1],[14443,23,1],[14443,24,2],[14443,27,2],[14443,30,1],
[14444,22,2],[14444,25,2],[14444,27,2],[14444,30,1],[14444,33,4],
[14444,35,7],[14444,52,2],[14444,74,1],[14445,1,2],[14445,15,1],
[14446,9,1],[14446,30,1],[14447,2,7],[14447,21,1],[14447,35,1],
[14448,44,1],[14448,66,1],[14449,2,1],[14449,8,4],[14449,20,1],
[14449,22,1],[14449,23,1],[14449,24,3],[14449,26,2],[14449,27,2],
[14449,30,1],[14449,31,1],[14449,32,3],[14449,34,4],[14449,36,1],
[14449,39,1],[14449,43,1],[14450,27,1],[14450,31,1],[14450,50,1],
[14451,9,1],[14451,12,1],[14452,23,6],[14453,33,2],[14453,35,1],
[14453,52,1],[14454,28,1],[14454,32,4],[14455,23,4],[14456,2,1],
[14456,33,1],[14456,56,1],[14456,68,3],[14457,21,1],[14457,70,1],
[14458,1,1],[14458,4,1],[14458,12,1],[14458,13,1],[14458,16,1],
[14458,17,2],[14458,18,1],[14458,19,2],[14458,20,2],[14458,29,1],
[14458,30,1],[14458,36,1],[14458,37,1],[14458,43,1],[14458,45,2],
[14458,53,2],[14458,73,1],[14458,74,1],[14459,34,1],[14459,76,1],
[14460,12,1],[14460,58,1],[14460,65,1],[14461,2,1],[14461,32,1],
[14461,62,1],[14462,2,2],[14463,23,1],[14463,24,3],[14464,1,2],
[14464,2,5],[14464,6,1],[14464,13,3],[14464,23,1],[14464,24,4],
[14464,33,1],[14464,37,1],[14464,42,2],[14464,43,2],[14464,47,3],
[14464,68,1],[14464,70,1],[14464,73,1],[14464,76,2],[14465,9,1],
[14465,10,1],[14466,2,3],[14466,25,1],[14466,43,1],[14467,21,1],

[14467,31,1],[14467,33,2],[14467,43,2],[14468,28,1],[14468,60,1],
[14469,13,1],[14469,20,1],[14469,29,1],[14469,59,1],[14469,70,1],
[14470,20,1],[14470,28,1],[14470,31,2],[14470,63,1],[14471,28,14],
[14471,70,1],[14471,76,2],[14472,62,3],[14473,6,1],[14473,13,1],
[14473,24,1],[14473,27,2],[14473,32,1],[14473,35,1],[14473,36,2],
[14473,37,1],[14473,38,1],[14473,45,1],[14474,12,1],[14474,34,1],
[14474,41,2],[14474,57,1],[14475,11,1],[14475,17,1],[14475,37,1],
[14475,45,1],[14475,65,1],[14475,67,1],[14476,3,1],[14476,4,22],
[14476,10,1],[14476,11,1],[14476,13,1],[14476,15,1],[14476,17,1],
[14476,18,1],[14476,19,2],[14476,20,1],[14476,26,1],[14476,33,1],
[14476,37,1],[14476,38,5],[14476,41,1],[14476,43,2],[14476,44,32],
[14476,46,1],[14476,50,31],[14476,55,1],[14476,56,1],[14476,57,1],
[14476,59,2],[14476,62,1],[14476,66,30],[14476,70,1],[14476,71,1],
[14476,77,1],[14477,3,3],[14477,24,1],[14477,26,1],[14477,27,3],
[14477,42,1],[14477,43,1],[14477,44,1],[14477,47,1],[14477,62,1],
[14478,27,1],[14478,32,4],[14478,35,3],[14479,1,1],[14479,2,2],
[14479,3,2],[14479,9,2],[14479,13,2],[14479,18,1],[14479,20,4],
[14479,21,1],[14479,22,1],[14479,23,14],[14479,24,1],[14479,25,4],
[14479,27,9],[14479,30,1],[14479,31,4],[14479,39,1],[14479,42,2],
[14479,43,2],[14479,53,1],[14480,62,2],[14481,23,1],[14481,31,1],
[14481,39,6],[14481,43,1],[14482,2,1],[14482,5,2],[14482,6,3],
[14482,9,1],[14482,23,6],[14482,24,302],[14482,27,11],[14482,28,6],
[14482,32,2],[14482,33,1],[14482,35,5],[14482,42,2],[14482,47,1],
[14482,50,1],[14482,66,1],[14482,67,1],[14482,69,1],[14482,70,3],
[14483,12,1],[14483,15,3],[14483,19,1],[14483,33,1],[14483,53,1],
[14483,55,3],[14484,17,1],[14484,70,1],[14485,5,2],[14486,1,2],
[14486,10,1],[14486,12,48],[14486,15,1],[14486,18,2],[14486,19,2],
[14486,34,1],[14486,45,2],[14486,73,1],[14487,37,2],[14488,13,1],
[14488,18,1],[14488,19,1],[14489,23,2],[14489,24,4],[14489,42,3],
[14489,62,1],[14489,70,1],[14490,3,1],[14490,12,1],[14490,23,1],
[14490,37,1],[14491,4,1],[14491,9,3],[14491,13,1],[14491,28,241],
[14491,41,1],[14491,63,3],[14491,74,1],[14491,75,5],[14491,76,6],
[14492,24,1],[14492,27,1],[14492,31,2],[14493,28,2],[14493,44,1],
[14493,69,1],[14493,72,1],[14494,2,17],[14494,3,1],[14494,17,1],
[14494,24,3],[14494,30,1],[14494,31,1],[14494,35,1],[14494,39,1],
[14494,47,1],[14495,2,1],[14495,3,1],[14495,24,1],[14495,26,2],
[14495,28,7],[14495,70,2],[14496,24,3],[14496,27,1],[14496,32,4],
[14496,40,1],[14497,24,1],[14497,35,1],[14498,6,1],[14498,34,1],
[14499,2,4],[14500,3,2],[14500,5,2],[14500,6,3],[14500,21,1],
[14500,23,7],[14500,24,3],[14500,25,1],[14500,27,6],[14500,28,8],
[14500,31,3],[14500,32,3],[14500,33,2],[14500,35,3],[14500,38,2],
[14500,42,1],[14500,43,1],[14501,5,1],[14501,35,3],[14501,43,1],
[14501,44,1],[14502,13,1],[14502,31,1],[14502,43,1],[14502,47,1],
[14502,51,1],[14503,10,2],[14503,13,2],[14503,14,1],[14503,16,1],
[14503,17,1],[14503,18,1],[14503,19,2],[14503,29,1],[14503,37,1],
[14503,41,1],[14503,45,2],[14503,63,1],[14503,67,1],[14503,77,1],
[14504,21,2],[14504,27,5],[14504,28,3],[14504,35,1],[14504,42,1],
[14504,47,1],[14504,51,1],[14504,74,1],[14505,9,1],[14505,20,1],
[14505,28,2],[14505,34,1],[14506,32,2],[14506,51,4],[14506,56,2],
[14506,69,1],[14507,22,1],[14507,23,7],[14507,24,2],[14507,27,8],

[14507,28,1],[14507,30,1],[14507,35,2],[14507,39,1],[14507,62,1],
[14508,2,1],[14508,3,1],[14508,21,1],[14508,42,1],[14509,5,1],
[14509,12,1],[14509,55,1],[14510,24,1],[14510,27,1],[14510,55,1],
[14511,20,1],[14511,35,1],[14512,22,3],[14512,26,1],[14512,28,2],
[14512,33,1],[14512,34,1],[14512,50,1],[14513,24,1],[14513,33,1],
[14513,38,1],[14513,40,1],[14513,42,1],[14513,47,1],[14513,76,2],
[14514,3,1],[14514,20,1],[14514,59,1],[14514,65,1],[14515,15,1],
[14515,20,1],[14516,15,1],[14516,17,1],[14517,27,1],[14517,33,2],
[14517,43,1],[14518,21,2],[14518,23,1],[14518,43,3],[14519,8,1],
[14519,19,1],[14519,23,1],[14519,27,1],[14520,21,2],[14521,4,1],
[14521,62,2],[14522,5,5],[14522,32,1],[14522,52,1],[14522,62,9],
[14522,68,1],[14523,2,1],[14523,28,1],[14523,31,1],[14524,1,1],
[14524,12,1],[14524,26,1],[14524,27,2],[14524,32,1],[14524,39,2],
[14524,45,1],[14525,47,2],[14526,13,1],[14526,17,1],[14526,28,1],
[14526,30,1],[14526,65,1],[14527,2,13],[14527,20,1],[14527,24,1],
[14527,28,1],[14527,31,1],[14527,32,1],[14527,33,1],[14527,43,3],
[14527,46,1],[14527,60,1],[14527,72,1],[14527,76,1],[14528,20,3],
[14528,34,3],[14529,39,2],[14530,20,1],[14530,71,1],[14531,30,1],
[14531,45,1],[14531,65,1],[14532,26,1],[14532,28,2],[14533,3,1],
[14533,40,1],[14533,50,1],[14533,63,1],[14533,73,1],[14534,5,1],
[14534,23,11],[14534,27,4],[14534,28,3],[14534,31,3],[14534,43,1],
[14534,74,1],[14535,28,1],[14535,32,8],[14536,4,1],[14536,15,2],
[14536,53,1],[14537,23,1],[14537,56,1],[14538,17,1],[14538,53,1],
[14539,2,1],[14539,15,1],[14539,28,1],[14539,31,1],[14540,31,4],
[14540,37,1],[14541,24,2],[14541,31,2],[14541,45,1],[14542,4,1],
[14542,66,1],[14543,56,6],[14544,1,1],[14544,2,8],[14544,31,7],
[14545,39,2],[14546,26,1],[14546,43,1],[14547,1,1],[14547,14,1],
[14547,15,1],[14547,20,2],[14547,39,1],[14547,42,1],[14547,59,1],
[14547,78,1],[14548,15,1],[14548,50,1],[14549,27,1],[14549,33,1],
[14550,0,1],[14550,1,2],[14550,9,2],[14550,10,1],[14550,12,3],
[14550,14,1],[14550,17,2],[14550,20,1],[14550,36,1],[14550,37,1],
[14550,39,1],[14550,41,1],[14550,44,1],[14550,45,3],[14550,46,1],
[14550,53,1],[14550,54,1],[14550,55,1],[14550,58,2],[14550,59,1],
[14550,60,1],[14550,63,1],[14550,65,2],[14550,66,1],[14550,67,1],
[14550,73,2],[14551,6,2],[14551,27,1],[14551,46,1],[14552,3,3],
[14552,5,1],[14552,20,2],[14552,22,3],[14552,23,4],[14552,24,2],
[14552,27,3],[14552,28,1],[14552,31,3],[14552,32,3],[14552,35,6],
[14552,39,2],[14552,42,3],[14552,43,3],[14552,46,1],[14552,70,1],
[14553,1,16],[14553,2,310],[14553,3,5],[14553,4,1],[14553,5,3],
[14553,6,2],[14553,9,8],[14553,10,1],[14553,12,1],[14553,13,28],
[14553,15,1],[14553,17,1],[14553,19,1],[14553,20,2],[14553,21,53],
[14553,22,3],[14553,23,48],[14553,24,41],[14553,25,4],[14553,26,74],
[14553,27,11],[14553,28,21],[14553,30,8],[14553,31,189],[14553,32,39],
[14553,33,31],[14553,35,13],[14553,37,1],[14553,38,1],[14553,39,38],
[14553,40,2],[14553,41,3],[14553,42,308],[14553,43,61],[14553,44,1],
[14553,45,1],[14553,46,3],[14553,47,25],[14553,48,7],[14553,49,1],
[14553,50,1],[14553,52,1],[14553,55,1],[14553,56,1],[14553,57,2],
[14553,62,2],[14553,64,1],[14553,65,2],[14553,66,2],[14553,68,1],
[14553,70,9],[14553,72,1],[14553,74,4],[14553,75,1],[14553,77,2],
[14553,78,4],[14554,5,1],[14554,18,1],[14554,66,1],[14555,17,2],

[14555,19,1],[14555,53,2],[14555,57,1],[14556,3,3],[14557,2,2],
[14558,31,1],[14558,35,1],[14559,5,1],[14559,23,1],[14559,24,2],
[14559,31,1],[14559,35,1],[14559,55,1],[14559,77,1],[14560,0,1],
[14560,1,1],[14560,2,1],[14560,3,4],[14560,4,30],[14560,5,9],
[14560,6,2],[14560,9,3],[14560,10,1],[14560,12,3],[14560,15,16],
[14560,16,1],[14560,17,10],[14560,21,1],[14560,22,1],[14560,23,4],
[14560,24,2],[14560,26,4],[14560,27,3],[14560,28,18],[14560,29,1],
[14560,31,1],[14560,32,1],[14560,33,2],[14560,34,1],[14560,35,2],
[14560,37,1],[14560,38,10],[14560,39,1],[14560,42,3],[14560,43,2],
[14560,44,162],[14560,45,2],[14560,47,4],[14560,48,3],[14560,49,1],
[14560,50,33],[14560,51,3],[14560,52,2],[14560,53,16],[14560,55,8],
[14560,56,1],[14560,58,1],[14560,61,1],[14560,62,1],[14560,66,125],
[14560,67,1],[14560,68,3],[14560,70,2],[14560,74,2],[14560,75,3],
[14560,76,2],[14560,79,1],[14561,42,1],[14561,52,1],[14562,2,2],
[14562,20,1],[14562,21,1],[14562,22,6],[14562,23,1],[14562,24,5],
[14562,25,1],[14562,26,2],[14562,27,3],[14562,30,2],[14562,31,3],
[14562,33,2],[14562,35,6],[14562,38,1],[14562,43,2],[14562,63,1],
[14562,70,1],[14562,79,1],[14563,31,2],[14564,2,2],[14564,22,1],
[14565,2,1],[14565,26,1],[14565,33,1],[14566,2,2],[14567,5,1],
[14567,47,1],[14567,62,2],[14568,16,2],[14568,41,1],[14568,46,1],
[14568,58,1],[14568,67,1],[14569,1,1],[14569,10,1],[14569,20,2],
[14569,28,1],[14569,35,1],[14570,1,1],[14570,13,1],[14570,17,1],
[14571,4,24],[14571,5,3],[14571,12,1],[14571,15,9],[14571,17,1],
[14571,20,1],[14571,21,1],[14571,22,1],[14571,23,1],[14571,24,1],
[14571,27,1],[14571,28,5],[14571,31,1],[14571,32,1],[14571,34,1],
[14571,38,4],[14571,40,1],[14571,42,1],[14571,44,75],[14571,47,2],
[14571,48,1],[14571,50,26],[14571,53,1],[14571,55,7],[14571,62,1],
[14571,66,66],[14571,68,2],[14571,70,1],[14571,74,1],[14571,76,2],
[14572,2,1],[14572,3,1],[14572,6,1],[14572,9,1],[14572,13,1],
[14572,20,2],[14572,22,1],[14572,23,2],[14572,24,23],[14572,25,1],
[14572,27,3],[14572,28,6],[14572,33,1],[14572,35,4],[14572,42,2],
[14572,48,1],[14572,58,1],[14573,21,1],[14573,25,2],[14573,42,1],
[14574,0,1],[14574,3,1],[14574,20,1],[14574,24,1],[14574,32,1],
[14574,37,1],[14574,42,2],[14574,46,1],[14574,59,2],[14575,2,1],
[14575,3,1],[14575,5,1],[14575,9,4],[14575,15,2],[14575,17,1],
[14575,20,1],[14575,23,1],[14575,28,1],[14575,50,1],[14575,53,5],
[14575,61,1],[14575,64,1],[14575,66,1],[14576,21,4],[14576,50,1],
[14577,15,1],[14577,49,1],[14577,55,2],[14578,24,1],[14578,51,1],
[14578,68,1],[14578,74,1],[14579,2,1],[14579,3,1],[14579,47,2],
[14580,8,1],[14580,28,10],[14580,34,1],[14581,28,2],[14582,24,1],
[14582,33,1],[14583,4,1],[14583,21,1],[14583,28,2],[14583,35,1],
[14583,76,2],[14584,1,1],[14584,39,1],[14585,10,1],[14585,41,1],
[14586,23,2],[14587,2,5],[14588,0,5],[14588,1,4],[14588,7,6],
[14588,8,10],[14588,9,11],[14588,10,13],[14588,11,6],[14588,12,15],
[14588,13,4],[14588,14,6],[14588,16,10],[14588,17,1],[14588,18,8],
[14588,19,8],[14588,20,7],[14588,22,1],[14588,24,1],[14588,29,3],
[14588,34,6],[14588,36,2],[14588,37,7],[14588,39,4],[14588,41,2],
[14588,45,3],[14588,46,3],[14588,49,2],[14588,51,1],[14588,53,1],
[14588,54,4],[14588,57,4],[14588,58,2],[14588,59,6],[14588,62,1],
[14588,63,1],[14588,64,3],[14588,65,8],[14588,67,2],[14588,71,2],

[14588,73,19],[14588,77,5],[14589,28,1],[14589,62,2],[14590,10,1],
[14590,22,1],[14591,63,2],[14592,28,4],[14592,35,1],[14592,69,1],
[14593,8,1],[14593,20,1],[14593,68,2],[14594,21,2],[14595,28,53],
[14595,75,1],[14596,33,2],[14596,44,1],[14597,30,2],[14597,52,1],
[14598,2,1],[14598,26,1],[14598,50,1],[14598,64,1],[14599,3,3],
[14599,6,1],[14599,26,1],[14599,28,1],[14599,31,1],[14599,38,1],
[14600,24,1],[14600,71,1],[14601,1,10],[14601,2,19],[14601,3,2],
[14601,4,1],[14601,8,3],[14601,10,2],[14601,12,4],[14601,13,8],
[14601,14,1],[14601,15,1],[14601,17,1],[14601,19,1],[14601,20,13],
[14601,21,2],[14601,22,1],[14601,23,12],[14601,24,6],[14601,26,6],
[14601,27,2],[14601,29,5],[14601,30,23],[14601,31,23],[14601,32,1],
[14601,33,5],[14601,34,2],[14601,37,2],[14601,38,1],[14601,39,7],
[14601,40,1],[14601,41,1],[14601,42,7],[14601,43,15],[14601,45,1],
[14601,46,5],[14601,47,1],[14601,51,1],[14601,52,2],[14601,54,1],
[14601,55,1],[14601,58,1],[14601,59,7],[14601,60,5],[14601,63,2],
[14601,64,4],[14601,65,2],[14601,68,1],[14601,69,1],[14601,70,1],
[14601,71,1],[14601,72,1],[14601,74,1],[14601,75,2],[14601,76,1],
[14601,77,1],[14601,78,2],[14602,1,3],[14602,2,39],[14602,3,19],
[14602,5,14],[14602,6,2],[14602,7,1],[14602,10,1],[14602,12,1],
[14602,13,3],[14602,20,1],[14602,21,3],[14602,22,11],[14602,23,30],
[14602,24,35],[14602,25,18],[14602,26,11],[14602,27,57],[14602,28,39],
[14602,30,1],[14602,31,54],[14602,32,48],[14602,33,14],[14602,35,26],
[14602,37,3],[14602,38,6],[14602,39,6],[14602,40,4],[14602,42,17],
[14602,43,20],[14602,44,2],[14602,45,1],[14602,47,9],[14602,48,11],
[14602,50,1],[14602,52,3],[14602,56,2],[14602,59,4],[14602,60,2],
[14602,61,2],[14602,62,6],[14602,63,3],[14602,64,1],[14602,66,2],
[14602,68,1],[14602,70,4],[14602,75,1],[14602,76,1],[14602,78,1],
[14603,27,1],[14603,28,2],[14603,55,1],[14604,32,4],[14604,43,4],
[14604,48,1],[14605,2,9],[14605,3,8],[14605,12,1],[14605,15,1],
[14605,17,1],[14605,22,4],[14605,23,2],[14605,24,2],[14605,26,1],
[14605,27,5],[14605,28,2],[14605,31,5],[14605,33,2],[14605,34,1],
[14605,35,1],[14605,37,2],[14605,42,2],[14605,43,1],[14605,47,1],
[14605,55,1],[14605,56,2],[14605,60,1],[14606,6,1],[14606,22,1],
[14606,38,2],[14606,47,51],[14607,25,2],[14608,12,1],[14608,23,1],
[14608,27,4],[14608,28,1],[14608,32,1],[14608,35,1],[14608,42,1],
[14609,19,1],[14609,34,1],[14609,50,1],[14610,22,1],[14610,23,2],
[14610,27,2],[14610,31,1],[14610,35,2],[14611,4,1],[14611,24,8],
[14611,31,3],[14611,33,1],[14611,76,1],[14612,27,3],[14613,2,1],
[14613,15,1],[14613,27,3],[14613,28,1],[14613,32,1],[14613,34,5],
[14613,35,8],[14613,37,3],[14613,39,2],[14613,62,3],[14614,28,1],
[14614,31,1],[14614,70,1],[14614,76,1],[14615,21,1],[14615,22,1],
[14615,24,1],[14615,26,1],[14615,35,1],[14616,23,1],[14616,24,1],
[14616,26,1],[14616,32,2],[14616,50,1],[14616,76,1],[14617,38,1],
[14617,42,3],[14618,3,1],[14618,13,1],[14618,23,1],[14618,26,1],
[14619,2,3],[14619,20,1],[14619,27,1],[14619,28,2],[14620,27,1],
[14620,33,1],[14621,2,1],[14621,5,1],[14621,17,1],[14621,22,1],
[14621,24,1],[14621,25,1],[14621,34,1],[14621,43,2],[14621,74,1],
[14622,10,1],[14622,20,1],[14623,1,1],[14623,3,2],[14623,9,1],
[14623,15,5],[14623,16,1],[14623,23,2],[14623,24,1],[14623,31,1],
[14623,36,1],[14623,50,1],[14623,55,11],[14623,56,2],[14623,61,1],

[14623,68,1],[14623,72,1],[14624,24,1],[14624,74,1],[14625,23,1],
[14625,32,1],[14626,21,1],[14626,25,1],[14627,27,1],[14627,34,1],
[14628,53,2],[14629,15,1],[14629,17,1],[14629,55,1],[14630,23,1],
[14630,40,1],[14630,47,1],[14631,30,1],[14631,35,1],[14631,64,2],
[14632,23,5],[14633,3,1],[14633,22,1],[14633,27,2],[14633,35,1],
[14634,2,1],[14634,4,1],[14634,5,1],[14634,6,4],[14634,20,2],
[14634,22,3],[14634,23,1],[14634,24,4],[14634,26,4],[14634,27,6],
[14634,31,2],[14634,32,4],[14634,35,1],[14634,42,1],[14634,43,1],
[14634,47,5],[14634,51,3],[14634,56,7],[14634,68,2],[14634,76,1],
[14635,2,3],[14635,21,1],[14635,24,1],[14635,31,1],[14636,23,1],
[14636,43,1],[14637,9,1],[14637,36,1],[14637,47,1],[14638,3,2],
[14638,23,1],[14638,24,1],[14638,27,2],[14638,28,1],[14638,32,1],
[14638,39,2],[14638,42,2],[14638,47,1],[14638,50,1],[14639,2,2],
[14639,42,1],[14640,27,2],[14641,5,1],[14641,6,1],[14641,8,1],
[14641,22,8],[14641,23,4],[14641,25,1],[14641,27,1],[14641,35,6],
[14641,38,1],[14641,55,1],[14641,56,2],[14642,63,2],[14642,76,2],
[14643,1,1],[14643,20,1],[14644,3,3],[14644,23,1],[14644,56,5],
[14644,61,1],[14645,17,1],[14645,19,1],[14645,78,1],[14646,2,2],
[14646,3,1],[14646,5,1],[14646,22,6],[14646,23,4],[14646,24,17],
[14646,25,4],[14646,26,1],[14646,27,14],[14646,30,1],[14646,31,2],
[14646,32,6],[14646,33,1],[14646,35,5],[14646,38,2],[14646,48,1],
[14646,52,1],[14646,56,5],[14646,59,1],[14647,2,2],[14647,22,1],
[14647,28,2],[14647,31,2],[14647,34,1],[14647,59,1],[14647,62,13],
[14647,75,1],[14648,28,1],[14648,40,2],[14649,2,1],[14649,23,1],
[14649,50,1],[14649,70,1],[14650,1,2],[14651,15,1],[14651,55,1],
[14652,1,1],[14652,12,2],[14653,2,4],[14653,33,1],[14653,62,2],
[14654,1,2],[14654,9,1],[14654,10,2],[14654,15,19],[14654,17,8],
[14654,19,1],[14654,20,1],[14654,37,1],[14654,45,1],[14654,53,3],
[14654,55,29],[14654,65,1],[14654,78,1],[14655,2,1],[14655,27,1],
[14656,28,1],[14656,56,12],[14657,24,4],[14658,0,8],[14658,1,25],
[14658,3,13],[14658,4,1],[14658,5,7],[14658,6,4],[14658,8,7],
[14658,9,25],[14658,10,13],[14658,11,2],[14658,12,9],[14658,13,60],
[14658,14,4],[14658,15,10],[14658,16,7],[14658,17,3],[14658,18,3],
[14658,19,3],[14658,20,123],[14658,21,2],[14658,22,4],[14658,23,17],
[14658,24,68],[14658,25,1],[14658,26,3],[14658,27,12],[14658,28,112],
[14658,29,2],[14658,30,4],[14658,31,2],[14658,32,7],[14658,33,28],
[14658,34,3],[14658,35,15],[14658,36,7],[14658,37,1],[14658,39,7],
[14658,40,1],[14658,41,2],[14658,42,5],[14658,43,4],[14658,44,2],
[14658,45,3],[14658,46,2],[14658,47,1],[14658,49,2],[14658,52,4],
[14658,53,7],[14658,54,2],[14658,55,10],[14658,56,1],[14658,57,1],
[14658,58,3],[14658,59,5],[14658,60,1],[14658,62,1],[14658,65,4],
[14658,66,2],[14658,67,1],[14658,68,1],[14658,71,1],[14658,74,2],
[14658,75,2],[14658,76,4],[14658,77,1],[14659,2,2],[14659,5,1],
[14659,24,2],[14659,26,1],[14659,27,3],[14659,28,11],[14659,32,1],
[14659,33,1],[14659,35,2],[14659,56,1],[14660,13,1],[14660,20,1],
[14661,0,1],[14661,7,1],[14661,8,2],[14661,9,2],[14661,36,1],
[14661,41,1],[14661,58,2],[14661,73,1],[14662,3,8],[14662,6,1],
[14662,13,1],[14662,24,1],[14662,30,1],[14662,31,2],[14662,33,1],
[14662,42,1],[14662,47,1],[14662,51,3],[14662,64,1],[14662,69,1],
[14663,2,1],[14663,12,1],[14663,28,1],[14663,62,1],[14663,73,1],

[14664,22,1],[14664,25,1],[14664,30,1],[14664,60,1],[14665,15,1],
[14665,58,1],[14666,4,1],[14666,22,1],[14666,44,1],[14666,66,1],
[14667,28,1],[14667,31,1],[14667,43,1],[14667,62,1],[14668,2,4],
[14668,20,1],[14668,23,2],[14668,25,1],[14668,26,1],[14668,27,2],
[14668,31,5],[14668,33,2],[14668,35,3],[14668,42,1],[14668,43,1],
[14668,71,1],[14669,55,1],[14669,58,1],[14670,1,3],[14670,12,1],
[14670,13,4],[14670,23,1],[14670,27,3],[14670,30,1],[14670,32,1],
[14670,39,1],[14670,42,1],[14670,51,1],[14670,69,6],[14670,70,2],
[14670,73,1],[14670,74,1],[14671,12,2],[14671,13,1],[14671,78,1],
[14672,3,2],[14672,23,4],[14672,31,1],[14672,32,1],[14672,42,1],
[14672,47,3],[14673,2,2],[14674,20,2],[14674,22,1],[14674,23,1],
[14675,5,1],[14675,35,1],[14675,52,1],[14675,66,1],[14676,22,1],
[14676,38,1],[14677,26,1],[14677,56,2],[14677,69,1],[14677,70,1],
[14678,28,3],[14678,32,5],[14679,35,1],[14679,43,1],[14680,2,1],
[14680,27,1],[14680,28,1],[14680,35,2],[14681,32,1],[14681,56,1],
[14682,17,1],[14682,34,1],[14682,39,1],[14683,44,1],[14683,66,1],
[14684,10,2],[14684,27,1],[14685,28,9],[14685,72,1],[14686,25,2],
[14686,32,1],[14686,56,1],[14686,69,1],[14687,1,2],[14688,21,1],
[14688,27,1],[14689,23,1],[14689,28,1],[14690,20,2],[14690,23,1],
[14690,24,1],[14690,26,1],[14690,27,8],[14690,31,1],[14690,48,1],
[14690,62,1],[14691,23,1],[14691,24,1],[14691,28,1],[14691,42,1],
[14691,55,2],[14691,62,1],[14692,5,2],[14693,23,4],[14694,3,1],
[14694,22,1],[14694,27,1],[14694,28,1],[14694,56,1],[14694,57,1],
[14694,60,1],[14695,25,3],[14695,27,1],[14696,2,2],[14697,32,2],
[14698,5,1],[14698,47,1],[14699,3,1],[14699,30,1],[14699,33,1],
[14699,55,1],[14700,21,6],[14700,24,1],[14700,42,3],[14701,33,4],
[14702,0,2],[14702,1,1],[14702,8,1],[14702,9,1],[14702,11,1],
[14702,12,10],[14702,13,2],[14702,20,6],[14702,24,1],[14702,53,1],
[14702,59,1],[14702,73,4],[14702,77,1],[14703,27,2],[14704,2,1],
[14704,5,1],[14704,6,1],[14704,20,1],[14704,21,1],[14704,22,4],
[14704,24,1],[14704,27,2],[14704,30,1],[14704,68,1],[14704,74,1],
[14704,76,1],[14705,27,1],[14705,47,2],[14706,1,1],[14706,8,1],
[14706,13,1],[14707,5,2],[14707,21,2],[14707,23,1],[14707,24,1],
[14707,27,1],[14707,28,6],[14707,52,1],[14708,38,2],[14709,7,2],
[14709,16,1],[14709,77,1],[14710,2,3],[14710,6,1],[14710,25,1],
[14710,32,2],[14710,42,1],[14710,79,1],[14711,23,4],[14712,1,3],
[14712,2,1],[14712,3,23],[14712,5,15],[14712,6,12],[14712,8,2],
[14712,9,1],[14712,10,2],[14712,11,1],[14712,12,1],[14712,13,6],
[14712,16,1],[14712,17,2],[14712,19,4],[14712,20,10],[14712,22,3],
[14712,23,4],[14712,24,22],[14712,26,8],[14712,27,23],[14712,28,8],
[14712,29,1],[14712,30,2],[14712,31,1],[14712,32,4],[14712,33,11],
[14712,34,1],[14712,35,8],[14712,39,5],[14712,40,1],[14712,42,1],
[14712,43,5],[14712,44,1],[14712,45,6],[14712,46,1],[14712,47,4],
[14712,50,2],[14712,53,1],[14712,54,1],[14712,55,3],[14712,56,8],
[14712,57,2],[14712,59,3],[14712,60,1],[14712,62,1],[14712,65,2],
[14712,67,1],[14712,69,1],[14712,73,3],[14712,76,1],[14712,78,1],
[14713,5,1],[14713,28,1],[14714,33,1],[14714,68,1],[14715,20,4],
[14715,22,5],[14715,26,1],[14715,37,1],[14716,0,6],[14716,1,3],
[14716,2,1],[14716,3,50],[14716,4,1],[14716,7,1],[14716,8,2],
[14716,9,4],[14716,10,1],[14716,11,3],[14716,12,5],[14716,13,1],

[14716,15,1],[14716,16,2],[14716,17,2],[14716,18,2],[14716,20,6],
[14716,23,80],[14716,28,1],[14716,29,1],[14716,30,2],[14716,34,1],
[14716,35,1],[14716,36,3],[14716,37,2],[14716,39,4],[14716,41,1],
[14716,42,2],[14716,43,1],[14716,47,1],[14716,53,1],[14716,55,2],
[14716,56,1],[14716,58,3],[14716,62,3],[14716,65,3],[14716,71,2],
[14716,73,3],[14716,77,2],[14717,75,2],[14717,76,1],[14718,2,1],
[14718,28,1],[14718,56,1],[14719,3,1],[14719,24,1],[14719,31,1],
[14720,2,1],[14720,4,1],[14720,9,1],[14720,10,1],[14720,51,1],
[14721,20,1],[14721,26,1],[14722,24,1],[14722,28,3],[14722,33,1],
[14722,69,1],[14722,74,1],[14723,46,1],[14723,62,1],[14724,5,1],
[14724,21,2],[14725,28,1],[14725,37,1],[14725,47,2],[14726,22,1],
[14726,41,3],[14727,5,1],[14727,70,1],[14728,3,2],[14728,23,2],
[14728,27,1],[14728,31,2],[14728,33,2],[14729,15,3],[14729,55,1],
[14730,3,1],[14730,65,1],[14731,22,5],[14731,28,1],[14732,35,1],
[14732,45,1],[14732,76,1],[14733,5,1],[14733,15,2],[14733,17,1],
[14733,18,1],[14733,20,3],[14733,21,1],[14733,23,1],[14733,49,1],
[14733,55,1],[14733,69,1],[14733,75,1],[14734,8,1],[14734,9,1],
[14734,13,1],[14734,20,2],[14734,34,1],[14734,60,1],[14734,67,1],
[14735,2,1],[14735,70,5],[14735,72,19],[14736,28,2],[14736,53,1],
[14737,9,1],[14737,17,1],[14737,39,1],[14738,10,1],[14738,15,2],
[14738,17,1],[14738,20,1],[14738,50,1],[14738,53,2],[14738,55,1],
[14739,15,1],[14739,23,1],[14739,42,1],[14739,50,1],[14740,31,1],
[14740,43,1],[14741,1,3],[14741,26,1],[14742,19,1],[14742,36,1],
[14742,57,1],[14743,1,7],[14743,8,9],[14743,12,2],[14743,17,2],
[14743,20,2],[14743,29,2],[14743,34,3],[14743,36,1],[14743,39,3],
[14743,53,2],[14743,59,1],[14743,65,1],[14743,73,3],[14743,78,1],
[14744,17,1],[14744,53,1],[14745,1,4],[14745,2,3],[14745,4,1],
[14745,7,1],[14745,8,2],[14745,15,3],[14745,20,2],[14745,24,1],
[14745,28,3],[14745,42,1],[14745,43,2],[14745,51,1],[14745,55,4],
[14745,60,1],[14745,66,1],[14745,67,1],[14745,68,1],[14746,23,1],
[14746,31,2],[14747,21,1],[14747,24,1],[14747,32,1],[14748,3,1],
[14748,13,1],[14748,20,2],[14748,26,1],[14748,35,5],[14748,43,1],
[14749,2,2],[14750,2,1],[14750,26,1],[14750,62,1],[14751,2,1],
[14751,21,1],[14751,23,1],[14752,1,1],[14752,23,1],[14752,27,2],
[14752,47,1],[14753,25,2],[14753,48,1],[14754,28,1],[14754,31,1],
[14755,5,1],[14755,10,1],[14755,23,1],[14755,26,3],[14755,30,1],
[14755,42,1],[14756,16,1],[14756,31,1],[14756,39,1],[14756,43,1],
[14757,2,3],[14757,7,1],[14757,12,54],[14757,13,3],[14757,17,1],
[14757,18,1],[14757,19,3],[14757,22,1],[14757,24,1],[14757,27,1],
[14757,28,1],[14757,29,6],[14757,30,2],[14757,33,1],[14757,34,2],
[14757,35,1],[14757,36,1],[14757,58,1],[14757,62,1],[14757,63,1],
[14757,68,2],[14757,70,1],[14757,77,2],[14757,78,1],[14758,22,1],
[14758,51,2],[14759,27,1],[14759,28,1],[14760,0,54],[14760,2,7],
[14760,9,1],[14760,12,1],[14760,15,1],[14760,16,1],[14760,34,3],
[14760,41,1],[14760,57,1],[14760,62,3],[14760,65,1],[14760,71,1],
[14761,4,1],[14761,6,2],[14761,23,1],[14761,24,1],[14761,33,1],
[14761,39,3],[14761,42,1],[14761,50,1],[14761,55,1],[14761,57,1],
[14761,66,1],[14761,72,1],[14762,28,1],[14762,32,1],[14762,42,1],
[14762,43,1],[14763,2,1],[14763,3,3],[14764,3,1],[14764,31,1],
[14764,63,1],[14765,15,2],[14766,1,2],[14767,2,8],[14767,22,1],

[14767,31,2],[14768,27,3],[14768,37,1],[14769,10,1],[14769,14,1],
[14769,15,1],[14769,65,1],[14769,71,1],[14770,1,1],[14770,13,1],
[14770,19,1],[14770,29,1],[14771,5,1],[14771,35,1],[14772,22,4],
[14772,23,1],[14772,24,1],[14772,32,1],[14773,23,3],[14774,25,1],
[14774,28,1],[14774,56,2],[14775,2,2],[14776,27,1],[14776,62,1],
[14777,17,2],[14777,39,1],[14777,53,1],[14777,77,1],[14778,6,4],
[14778,23,1],[14778,27,1],[14778,34,1],[14778,35,1],[14778,56,1],
[14778,70,1],[14779,5,1],[14779,21,2],[14779,24,1],[14779,27,1],
[14780,23,2],[14780,25,1],[14780,27,2],[14780,28,1],[14780,31,1],
[14780,32,3],[14780,42,1],[14780,59,2],[14781,1,1],[14781,5,1],
[14781,35,1],[14782,15,1],[14782,44,1],[14782,55,1],[14782,56,1],
[14783,2,1],[14783,27,1],[14783,28,1],[14784,53,1],[14784,77,1],
[14785,32,1],[14785,61,1],[14785,70,1],[14785,74,4],[14786,23,1],
[14786,47,1],[14786,50,1],[14787,2,1],[14787,27,1],[14787,45,1],
[14788,20,3],[14788,27,1],[14788,33,1],[14789,25,1],[14789,32,1],
[14790,51,1],[14790,74,1],[14791,1,10],[14791,13,3],[14791,39,2],
[14792,3,1],[14792,4,1],[14792,5,1],[14792,6,1],[14792,8,2],
[14792,22,2],[14792,24,6],[14792,27,1],[14792,56,4],[14792,60,1],
[14793,18,1],[14793,24,1],[14793,27,1],[14793,41,1],[14793,55,2],
[14794,20,1],[14794,53,1],[14794,55,2],[14795,3,1],[14795,23,1],
[14795,24,1],[14795,25,1],[14795,42,2],[14796,6,1],[14796,70,1],
[14797,20,2],[14797,22,3],[14797,26,1],[14797,32,1],[14797,33,1],
[14798,4,1],[14798,28,1],[14799,3,2],[14799,5,1],[14799,25,2],
[14799,28,1],[14799,32,4],[14799,43,2],[14800,3,1],[14800,27,3],
[14801,1,1],[14801,4,1],[14801,5,1],[14801,9,1],[14801,12,2],
[14801,17,1],[14801,18,1],[14801,19,1],[14801,24,1],[14801,28,1],
[14801,44,3],[14801,53,1],[14801,55,1],[14801,58,2],[14801,64,1],
[14801,65,1],[14801,66,1],[14801,67,1],[14802,73,2],[14803,2,2],
[14804,28,4],[14805,1,6],[14805,7,6],[14805,8,8],[14805,9,38],
[14805,10,37],[14805,11,11],[14805,12,10],[14805,13,8],[14805,14,6],
[14805,15,11],[14805,16,19],[14805,17,4],[14805,18,8],[14805,19,24],
[14805,20,14],[14805,29,10],[14805,30,7],[14805,34,10],[14805,36,6],
[14805,37,15],[14805,39,2],[14805,41,17],[14805,45,19],[14805,46,11],
[14805,49,11],[14805,53,8],[14805,54,8],[14805,55,16],[14805,57,18],
[14805,58,18],[14805,59,13],[14805,60,5],[14805,64,2],[14805,65,21],
[14805,67,17],[14805,71,5],[14805,73,12],[14805,77,19],[14805,78,4],
[14806,3,3],[14806,52,1],[14807,38,2],[14808,28,5],[14808,63,1],
[14808,74,3],[14808,76,1],[14809,23,2],[14809,27,6],[14810,1,2],
[14810,8,1],[14810,11,1],[14810,13,1],[14810,15,1],[14810,31,1],
[14810,35,2],[14811,15,1],[14811,55,2],[14811,65,1],[14812,2,1],
[14812,3,1],[14812,9,1],[14812,19,1],[14812,20,1],[14812,22,2],
[14812,24,4],[14812,26,2],[14812,27,2],[14812,28,1],[14812,42,1],
[14812,43,1],[14813,15,6],[14813,53,2],[14813,55,1],[14814,28,1],
[14814,47,1],[14815,28,1],[14815,35,3],[14816,22,1],[14816,25,5],
[14816,27,1],[14816,33,5],[14816,35,10],[14816,52,3],[14816,63,1],
[14817,23,2],[14817,28,9],[14817,44,1],[14818,1,1],[14818,2,1],
[14818,11,1],[14818,20,2],[14818,22,1],[14818,23,1],[14818,28,1],
[14818,34,1],[14818,39,2],[14818,41,1],[14818,45,1],[14818,53,1],
[14818,56,1],[14818,65,1],[14818,71,1],[14818,74,1],[14819,2,6],
[14819,3,1],[14819,27,1],[14820,0,1],[14820,1,5],[14820,4,9],

[14820,5,2],[14820,8,2],[14820,9,4],[14820,10,3],[14820,11,2],
[14820,12,5],[14820,13,1],[14820,15,109],[14820,16,1],[14820,17,50],
[14820,19,1],[14820,20,8],[14820,21,1],[14820,24,1],[14820,28,4],
[14820,29,3],[14820,33,2],[14820,34,1],[14820,36,1],[14820,37,1],
[14820,38,1],[14820,41,2],[14820,42,1],[14820,44,3],[14820,45,1],
[14820,46,3],[14820,50,9],[14820,53,56],[14820,54,1],[14820,55,70],
[14820,57,2],[14820,58,1],[14820,59,3],[14820,60,1],[14820,64,1],
[14820,65,3],[14820,66,3],[14820,67,2],[14820,68,1],[14820,69,1],
[14820,70,1],[14820,71,1],[14820,72,2],[14820,73,3],[14820,77,4],
[14821,1,21],[14821,6,1],[14821,7,6],[14821,8,1],[14821,13,29],
[14821,17,1],[14821,21,2],[14821,23,1],[14821,25,1],[14821,26,5],
[14821,28,1],[14821,29,1],[14821,31,5],[14821,34,1],[14821,39,1],
[14821,42,1],[14821,51,1],[14821,59,1],[14822,1,2],[14822,9,1],
[14822,12,1],[14822,15,2],[14822,16,1],[14822,19,1],[14822,29,6],
[14822,30,1],[14822,34,1],[14822,49,1],[14822,60,1],[14822,67,2],
[14822,71,1],[14822,73,1],[14823,13,1],[14823,20,2],[14823,36,1],
[14824,3,1],[14824,24,3],[14824,26,1],[14824,38,2],[14824,44,1],
[14824,56,3],[14824,66,1],[14824,74,1],[14825,19,1],[14825,20,1],
[14826,8,4],[14826,17,1],[14826,26,1],[14826,42,1],[14827,12,1],
[14827,18,1],[14827,20,1],[14827,21,3],[14827,22,4],[14827,23,8],
[14827,24,5],[14827,25,7],[14827,26,4],[14827,27,15],[14827,28,3],
[14827,31,6],[14827,32,5],[14827,33,4],[14827,35,6],[14827,39,8],
[14827,43,1],[14827,56,1],[14827,57,1],[14827,68,1],[14828,32,2],
[14829,52,1],[14829,70,1],[14830,2,1],[14830,27,1],[14830,42,1],
[14830,53,1],[14831,2,1],[14831,5,1],[14831,28,1],[14831,63,1],
[14832,1,1],[14832,13,1],[14832,16,1],[14832,54,1],[14832,59,1],
[14832,65,1],[14833,1,1],[14833,12,4],[14833,16,1],[14834,6,3],
[14834,27,1],[14834,28,1],[14834,33,1],[14834,42,2],[14835,28,18],
[14835,44,1],[14836,15,1],[14836,17,1],[14836,38,1],[14836,50,1],
[14836,53,3],[14837,8,1],[14837,10,1],[14837,17,1],[14837,20,1],
[14837,25,5],[14837,28,9],[14837,30,9],[14837,31,1],[14837,33,17],
[14837,35,30],[14837,38,1],[14837,43,1],[14837,52,51],[14837,62,1],
[14837,70,1],[14838,31,3],[14838,42,2],[14839,3,1],[14839,26,1],
[14839,28,1],[14839,56,3],[14839,66,1],[14840,3,1],[14840,8,1],
[14840,23,2],[14840,27,1],[14840,31,1],[14841,28,2],[14842,3,1],
[14842,6,1],[14842,22,7],[14842,23,1],[14842,24,3],[14842,25,3],
[14842,27,2],[14842,28,2],[14842,32,1],[14842,33,1],[14842,38,3],
[14842,56,4],[14843,4,3],[14843,16,1],[14843,17,3],[14843,32,1],
[14843,50,1],[14843,53,10],[14843,55,3],[14843,63,1],[14844,1,3],
[14844,3,3],[14844,6,1],[14844,8,1],[14844,10,1],[14844,11,1],
[14844,12,1],[14844,13,11],[14844,16,3],[14844,20,15],[14844,21,2],
[14844,22,3],[14844,23,3],[14844,24,4],[14844,25,2],[14844,26,4],
[14844,27,2],[14844,31,5],[14844,33,5],[14844,35,1],[14844,37,1],
[14844,38,2],[14844,39,2],[14844,42,8],[14844,43,5],[14844,44,1],
[14844,46,1],[14844,47,2],[14844,48,6],[14844,56,1],[14844,57,1],
[14844,58,1],[14844,75,3],[14844,78,3],[14845,2,1],[14845,4,5],
[14845,5,1],[14845,6,2],[14845,21,2],[14845,22,3],[14845,24,6],
[14845,25,1],[14845,27,5],[14845,28,10],[14845,31,6],[14845,32,3],
[14845,33,1],[14845,35,2],[14845,38,218],[14845,40,5],[14845,43,3],
[14845,44,2],[14845,50,4],[14845,63,1],[14845,66,2],[14845,74,2],

[14845,75,1],[14845,76,1],[14846,6,1],[14846,15,1],[14847,25,1],
[14847,28,1],[14848,4,1],[14848,24,1],[14848,25,1],[14848,62,1],
[14849,35,1],[14849,50,1],[14849,51,2],[14849,66,1],[14849,70,2],
[14850,42,1],[14850,70,1],[14850,72,1],[14851,40,11],[14851,68,3],
[14851,69,5],[14851,70,6],[14851,72,2],[14852,9,2],[14852,15,5],
[14852,18,1],[14852,55,2],[14853,3,1],[14853,19,1],[14853,21,1],
[14853,24,2],[14853,26,3],[14853,28,1],[14853,30,1],[14853,32,2],
[14853,43,1],[14853,56,3],[14853,62,1],[14853,65,1],[14854,10,1],
[14854,32,1],[14854,43,1],[14854,44,1],[14855,2,1],[14855,68,2],
[14856,3,1],[14856,21,1],[14856,23,2],[14856,24,1],[14856,26,1],
[14856,27,1],[14856,28,1],[14856,31,1],[14856,32,3],[14856,39,1],
[14856,42,3],[14856,43,5],[14856,47,1],[14857,27,2],[14857,62,1],
[14857,74,1],[14858,1,1],[14858,9,1],[14858,14,1],[14858,27,1],
[14858,29,1],[14858,30,1],[14858,32,1],[14858,46,1],[14858,54,2],
[14858,62,11],[14858,66,1],[14858,72,1],[14859,15,3],[14860,20,1],
[14860,49,2],[14861,4,1],[14861,35,1],[14862,23,1],[14862,40,1],
[14862,68,1],[14863,2,2],[14863,71,1],[14864,0,1],[14864,7,1],
[14864,9,3],[14864,10,8],[14864,11,3],[14864,12,1],[14864,13,1],
[14864,14,4],[14864,16,8],[14864,19,9],[14864,20,1],[14864,36,2],
[14864,37,2],[14864,41,8],[14864,45,5],[14864,46,1],[14864,49,4],
[14864,54,1],[14864,55,2],[14864,57,2],[14864,58,10],[14864,59,4],
[14864,60,1],[14864,64,1],[14864,65,11],[14864,67,3],[14864,71,3],
[14864,73,5],[14864,77,5],[14864,78,2],[14865,4,1],[14865,8,8],
[14865,28,6],[14866,4,1],[14866,6,1],[14866,28,1],[14866,69,9],
[14867,28,2],[14867,42,1],[14868,25,4],[14869,2,1],[14869,27,1],
[14869,28,1],[14870,2,1],[14870,28,1],[14870,31,1],[14870,56,1],
[14871,2,1],[14871,28,1],[14872,15,1],[14872,18,1],[14872,23,2],
[14872,27,1],[14872,31,1],[14872,32,2],[14872,38,1],[14872,52,1],
[14873,22,2],[14873,24,2],[14873,25,2],[14873,27,1],[14873,35,2],
[14873,56,1],[14873,76,1],[14874,4,1],[14874,28,1],[14874,66,1],
[14875,24,2],[14876,9,1],[14876,39,1],[14877,1,2],[14878,40,1],
[14878,44,1],[14879,3,1],[14879,8,1],[14879,17,1],[14879,22,1],
[14879,27,1],[14879,35,1],[14879,50,1],[14879,67,1],[14880,23,1],
[14880,34,2],[14881,22,1],[14881,27,1],[14881,61,1],[14881,62,1],
[14882,23,5],[14883,17,1],[14883,65,1],[14884,26,18],[14884,76,1],
[14885,31,1],[14885,33,1],[14885,50,1],[14886,2,3],[14886,21,1],
[14886,22,1],[14886,23,2],[14886,24,1],[14886,27,1],[14886,40,1],
[14886,43,1],[14886,47,1],[14886,48,1],[14887,32,7],[14888,20,1],
[14888,27,1],[14889,22,1],[14889,31,1],[14890,20,1],[14890,23,1],
[14890,24,1],[14890,26,1],[14890,28,1],[14890,31,1],[14890,47,1],
[14890,65,1],[14891,44,3],[14892,3,3],[14893,15,1],[14893,55,1],
[14893,59,1],[14893,67,1],[14894,42,1],[14894,46,1],[14895,5,1],
[14895,23,1],[14895,24,2],[14895,25,1],[14895,28,6],[14895,32,2],
[14895,56,1],[14896,17,2],[14897,15,1],[14897,44,1],[14897,55,4],
[14898,2,2],[14898,4,1],[14898,8,1],[14898,15,1],[14898,22,3],
[14898,23,1],[14898,24,1],[14898,26,1],[14898,39,1],[14898,50,1],
[14898,55,1],[14899,28,2],[14900,35,1],[14900,59,1],[14901,9,1],
[14901,12,1],[14901,28,1],[14902,25,1],[14902,32,1],[14902,43,1],
[14903,12,1],[14903,15,2],[14903,39,1],[14903,46,1],[14904,21,2],
[14904,22,1],[14904,39,1],[14904,62,1],[14905,2,3],[14905,27,1],

[14906,3,3],[14906,4,1],[14906,5,1],[14906,6,1],[14906,13,1],
[14906,22,5],[14906,23,12],[14906,24,38],[14906,25,1],[14906,26,8],
[14906,27,3],[14906,28,2],[14906,31,2],[14906,32,3],[14906,33,12],
[14906,35,1],[14906,42,5],[14906,43,6],[14906,47,2],[14906,50,2],
[14906,51,3],[14906,56,3],[14906,63,1],[14906,66,1],[14906,68,2],
[14906,75,1],[14907,2,1],[14907,24,1],[14907,25,1],[14907,42,2],
[14908,21,1],[14908,22,2],[14908,27,4],[14908,31,1],[14908,43,1],
[14908,48,1],[14909,3,1],[14909,30,1],[14909,33,8],[14909,34,3],
[14909,35,1],[14909,38,1],[14909,44,1],[14909,52,3],[14909,62,1],
[14910,39,3],[14911,2,3],[14911,28,3],[14912,15,1],[14912,20,2],
[14912,55,1],[14913,5,2],[14913,22,1],[14913,25,2],[14913,33,1],
[14914,28,1],[14914,47,2],[14915,44,1],[14915,66,1],[14916,5,2],
[14916,28,1],[14916,31,1],[14917,24,1],[14917,27,1],[14917,42,5],
[14918,8,1],[14918,28,1],[14919,20,1],[14919,26,1],[14919,27,1],
[14919,30,1],[14919,32,1],[14919,39,1],[14919,42,4],[14919,45,1],
[14920,3,20],[14920,5,3],[14920,6,1],[14920,10,1],[14920,13,1],
[14920,20,9],[14920,21,1],[14920,22,3],[14920,23,12],[14920,24,7],
[14920,26,10],[14920,27,7],[14920,28,7],[14920,30,1],[14920,31,10],
[14920,32,1],[14920,33,2],[14920,35,1],[14920,36,1],[14920,42,4],
[14920,43,9],[14920,46,1],[14920,47,1],[14920,56,1],[14920,64,1],
[14920,65,1],[14920,67,1],[14920,74,1],[14920,75,1],[14921,28,2],
[14921,68,4],[14921,72,12],[14922,13,1],[14922,27,1],[14923,9,1],
[14923,15,5],[14923,17,1],[14923,36,1],[14923,55,4],[14924,7,1],
[14924,9,1],[14924,24,1],[14924,27,1],[14924,30,3],[14924,45,2],
[14924,57,2],[14924,62,8],[14924,73,1],[14925,28,2],[14926,3,2],
[14927,53,4],[14928,24,2],[14928,27,1],[14928,30,1],[14929,24,1],
[14929,28,1],[14929,33,2],[14930,20,1],[14930,27,2],[14930,35,1],
[14931,5,3],[14931,24,3],[14931,30,1],[14931,35,2],[14931,43,1],
[14931,55,1],[14932,1,3],[14932,12,1],[14932,57,1],[14933,22,2],
[14934,5,1],[14934,24,2],[14934,25,1],[14934,27,5],[14934,33,1],
[14934,35,4],[14934,43,1],[14934,52,1],[14935,27,1],[14935,47,1],
[14935,50,1],[14936,3,1],[14936,21,1],[14936,23,1],[14936,32,1],
[14936,35,1],[14936,42,1],[14937,21,2],[14938,5,1],[14938,10,1],
[14938,14,1],[14938,22,2],[14938,23,1],[14938,24,2],[14938,25,1],
[14938,26,1],[14938,27,2],[14938,32,1],[14938,35,2],[14938,43,1],
[14938,47,1],[14938,50,1],[14938,61,1],[14938,69,1],[14939,3,1],
[14939,5,2],[14939,9,1],[14939,11,1],[14939,12,1],[14939,19,2],
[14939,21,1],[14939,22,1],[14939,24,3],[14939,27,2],[14939,28,13],
[14939,31,1],[14939,40,2],[14939,43,1],[14939,48,1],[14939,61,1],
[14939,63,8],[14939,66,1],[14939,74,20],[14939,75,8],[14939,76,14],
[14940,20,1],[14940,29,1],[14941,2,2],[14942,22,1],[14942,23,2],
[14942,27,1],[14942,28,1],[14943,27,1],[14943,35,1],[14943,43,1],
[14944,1,1],[14944,29,1],[14944,34,1],[14945,25,2],[14946,3,2],
[14946,6,2],[14946,26,1],[14946,44,2],[14946,56,11],[14946,66,1],
[14947,3,1],[14947,5,2],[14947,6,5],[14947,23,1],[14947,24,5],
[14947,27,2],[14947,28,1],[14947,32,1],[14947,36,1],[14947,44,1],
[14947,47,2],[14947,50,1],[14948,22,2],[14948,26,2],[14948,27,1],
[14948,35,1],[14948,43,1],[14948,50,1],[14949,44,1],[14949,74,1],
[14950,25,3],[14951,35,3],[14952,24,1],[14952,46,1],[14953,2,2],
[14953,3,1],[14953,26,1],[14953,29,1],[14953,31,1],[14953,46,1],

[14953,73,2],[14954,6,1],[14954,35,1],[14954,47,1],[14954,56,2],
[14955,21,1],[14955,66,1],[14956,31,1],[14956,42,1],[14957,23,1],
[14957,31,1],[14957,63,3],[14957,74,5],[14957,75,6],[14957,76,3],
[14958,5,1],[14958,21,1],[14958,23,1],[14958,28,2],[14958,38,1],
[14958,50,1],[14958,66,1],[14958,74,1],[14959,27,3],[14959,35,1],
[14959,75,1],[14960,39,1],[14960,54,1],[14961,1,1],[14961,13,1],
[14962,1,1],[14962,9,1],[14962,10,3],[14962,11,1],[14962,12,1],
[14962,16,2],[14962,19,1],[14962,20,1],[14962,29,1],[14962,36,1],
[14962,41,2],[14962,59,2],[14962,71,1],[14962,73,1],[14962,77,1],
[14963,22,3],[14963,28,1],[14963,31,1],[14964,13,1],[14964,20,1],
[14965,23,4],[14965,35,1],[14966,8,1],[14966,15,1],[14967,1,3],
[14967,4,6],[14967,5,3],[14967,14,1],[14967,15,23],[14967,17,58],
[14967,18,2],[14967,20,2],[14967,24,1],[14967,34,1],[14967,35,1],
[14967,38,2],[14967,41,1],[14967,42,1],[14967,44,3],[14967,46,1],
[14967,50,11],[14967,53,65],[14967,54,1],[14967,55,53],[14967,56,2],
[14967,58,1],[14967,62,1],[14967,64,3],[14967,77,3],[14968,23,1],
[14968,26,1],[14968,27,4],[14968,35,1],[14968,56,2],[14969,3,2],
[14969,5,1],[14969,6,1],[14969,22,1],[14969,24,1],[14969,25,1],
[14969,26,4],[14969,27,4],[14969,28,3],[14969,32,40],[14969,35,2],
[14969,56,3],[14970,24,1],[14970,25,1],[14971,24,1],[14971,28,1],
[14971,70,1],[14972,21,1],[14972,22,3],[14972,28,3],[14973,15,2],
[14973,20,1],[14974,3,1],[14974,4,1],[14974,5,4],[14974,10,1],
[14974,19,1],[14974,28,1],[14974,40,2],[14974,41,1],[14974,50,2],
[14974,63,51],[14974,68,3],[14974,70,3],[14974,74,52],[14974,75,64],
[14974,76,86],[14974,77,1],[14975,2,2],[14976,4,1],[14976,5,4],
[14976,10,1],[14976,19,1],[14976,25,1],[14976,27,1],[14976,28,1],
[14976,33,1],[14976,40,4],[14976,41,1],[14976,42,1],[14976,43,1],
[14976,44,1],[14976,49,1],[14976,62,1],[14976,63,48],[14976,66,1],
[14976,68,2],[14976,70,3],[14976,72,5],[14976,74,53],[14976,75,44],
[14976,76,55],[14977,25,2],[14978,23,2],[14979,2,3],[14979,24,3],
[14979,28,1],[14979,34,1],[14979,42,1],[14979,55,2],[14980,3,1],
[14980,23,1],[14980,26,1],[14980,27,1],[14980,33,1],[14980,35,1],
[14980,43,1],[14980,44,1],[14980,62,1],[14980,63,1],[14980,70,1],
[14980,74,2],[14980,76,2],[14981,15,2],[14981,55,1],[14981,56,1],
[14981,66,1],[14981,75,1],[14982,6,1],[14982,15,1],[14982,26,1],
[14982,40,2],[14982,44,1],[14982,66,1],[14982,70,1],[14982,75,1],
[14983,26,1],[14983,66,1],[14984,13,5],[14984,39,1],[14985,2,10],
[14985,13,2],[14986,24,2],[14987,24,1],[14987,62,1],[14988,33,2],
[14988,35,1],[14988,52,4],[14989,7,1],[14989,11,1],[14989,15,1],
[14989,16,1],[14989,17,1],[14989,37,1],[14989,45,1],[14989,55,3],
[14989,65,1],[14989,77,3],[14990,2,1],[14990,25,1],[14990,41,1],
[14990,51,1],[14990,69,2],[14991,7,1],[14991,10,1],[14992,23,1],
[14992,32,1],[14992,59,1],[14993,26,1],[14993,62,1],[14994,2,3],
[14994,23,1],[14994,31,1],[14994,42,1],[14994,43,1],[14994,71,1],
[14995,25,1],[14995,26,1],[14995,27,1],[14995,28,4],[14995,38,1],
[14995,66,1],[14996,12,1],[14996,15,1],[14996,16,1],[14996,21,1],
[14996,25,1],[14996,27,1],[14996,34,1],[14996,61,1],[14996,69,1],
[14996,74,20],[14996,76,1],[14997,12,1],[14997,20,1],[14997,29,1],
[14998,2,11],[14998,3,9],[14998,5,6],[14998,9,1],[14998,12,1],
[14998,15,1],[14998,18,1],[14998,20,4],[14998,21,5],[14998,22,14],

[14998,23,83],[14998,24,38],[14998,25,4],[14998,26,21],[14998,27,44],
[14998,28,16],[14998,30,1],[14998,31,22],[14998,32,31],[14998,33,5],
[14998,34,2],[14998,35,17],[14998,38,1],[14998,39,3],[14998,42,5],
[14998,43,21],[14998,47,3],[14998,48,1],[14998,52,3],[14998,53,1],
[14998,56,2],[14998,63,1],[14998,65,1],[14998,71,1],[14999,4,2],
[14999,23,4],[14999,27,5],[14999,31,6],[14999,38,1],[14999,40,1],
[14999,42,4],[14999,47,1],[14999,48,2],[14999,50,1],[14999,62,1],
[14999,63,1],[14999,66,8],[14999,68,2],[14999,70,1],[14999,72,1],
[14999,74,1],[14999,75,1],[14999,76,2],[15000,17,1],[15000,53,1],
[15000,55,1],[15001,23,1],[15001,28,1],[15002,3,3],[15002,8,1],
[15002,16,1],[15002,20,4],[15002,27,5],[15002,42,2],[15002,45,1],
[15002,46,1],[15002,60,3],[15002,77,1],[15003,19,1],[15003,20,1],
[15004,17,1],[15004,24,1],[15004,37,1],[15004,45,1],[15004,78,1],
[15005,43,3],[15005,48,7],[15005,56,1],[15005,78,1],[15006,5,1],
[15006,28,1],[15007,35,1],[15007,52,17],[15008,6,1],[15008,24,1],
[15008,27,1],[15008,28,1],[15008,47,2],[15009,23,1],[15009,33,1],
[15010,21,1],[15010,26,1],[15011,11,1],[15011,24,1],[15011,28,1],
[15011,50,1],[15011,56,2],[15012,9,1],[15012,23,1],[15012,25,1],
[15012,28,1],[15012,38,1],[15012,55,2],[15013,2,3],[15014,23,3],
[15014,27,2],[15014,56,1],[15014,70,1],[15015,3,2],[15016,8,1],
[15016,10,1],[15016,14,1],[15016,15,4],[15016,60,1],[15017,30,2],
[15018,30,2],[15018,46,1],[15019,27,1],[15019,33,1],[15020,27,1],
[15020,68,3],[15020,70,1],[15021,2,2],[15022,5,1],[15022,26,1],
[15023,22,1],[15023,23,1],[15023,32,3],[15024,22,1],[15024,74,1],
[15025,2,1],[15025,21,1],[15025,23,1],[15025,26,5],[15025,27,1],
[15025,35,2],[15025,40,1],[15025,43,3],[15025,44,1],[15025,56,1],
[15025,62,1],[15025,72,1],[15026,31,2],[15027,22,1],[15027,50,1],
[15028,30,2],[15029,22,1],[15029,43,1],[15029,45,1],[15030,5,2],
[15031,3,1],[15031,24,1],[15031,43,1],[15031,48,2],[15031,63,1],
[15032,36,1],[15032,73,1],[15033,4,1],[15033,24,1],[15033,28,1],
[15034,8,1],[15034,9,1],[15034,12,1],[15034,16,2],[15034,17,1],
[15034,47,1],[15034,53,1],[15035,34,1],[15035,66,1],[15036,35,1],
[15036,38,1],[15036,50,1],[15036,72,1],[15036,76,1],[15037,20,2],
[15038,4,2],[15038,14,1],[15039,28,1],[15039,35,1],[15040,10,1],
[15040,27,3],[15040,28,1],[15040,64,1],[15041,67,1],[15041,77,1],
[15042,27,1],[15042,33,1],[15043,2,5],[15043,31,4],[15044,24,1],
[15044,27,1],[15044,35,2],[15044,43,1],[15045,1,1],[15045,3,1],
[15045,20,1],[15045,23,1],[15045,27,1],[15045,28,1],[15046,12,1],
[15046,28,1],[15046,33,1],[15046,45,1],[15046,50,1],[15046,53,2],
[15046,70,1],[15047,5,1],[15047,15,2],[15047,21,1],[15047,23,1],
[15047,24,1],[15047,28,1],[15047,40,1],[15047,55,2],[15047,66,1],
[15048,9,1],[15048,10,1],[15048,15,2],[15048,45,1],[15048,55,1],
[15049,23,1],[15049,24,1],[15049,25,9],[15049,27,1],[15049,31,2],
[15049,33,1],[15049,35,5],[15049,42,1],[15049,43,4],[15050,2,3],
[15050,5,1],[15050,17,1],[15050,22,4],[15050,23,2],[15050,26,1],
[15050,27,1],[15050,33,1],[15051,5,1],[15051,28,4],[15051,59,1],
[15052,24,3],[15052,27,3],[15052,35,4],[15052,62,1],[15053,2,1],
[15053,28,1],[15053,35,9],[15053,70,1],[15054,24,2],[15055,28,1],
[15055,44,1],[15056,32,2],[15057,3,2],[15057,23,2],[15057,27,1],
[15057,30,1],[15057,77,1],[15058,9,1],[15058,45,1],[15059,23,1],

[15059,56,1],[15059,59,1],[15060,5,2],[15060,13,2],[15060,22,1],
[15060,30,1],[15060,33,3],[15060,34,2],[15060,56,1],[15060,69,1],
[15061,22,1],[15061,24,1],[15061,27,1],[15061,31,1],[15061,32,1],
[15061,35,2],[15062,5,1],[15062,6,1],[15062,28,1],[15063,45,1],
[15063,59,1],[15064,28,7],[15064,32,1],[15065,10,1],[15065,39,1],
[15066,27,6],[15066,35,1],[15066,40,1],[15066,51,1],[15066,62,1],
[15066,79,1],[15067,35,1],[15067,42,1],[15068,23,1],[15068,26,1],
[15068,27,2],[15068,43,1],[15069,4,1],[15069,28,2],[15070,8,1],
[15070,25,1],[15071,18,1],[15071,36,1],[15072,46,1],[15072,65,1],
[15073,22,1],[15073,28,1],[15074,31,3],[15075,28,1],[15075,50,2],
[15076,55,1],[15076,75,1],[15077,24,1],[15077,67,1],[15077,68,1],
[15078,5,1],[15078,22,1],[15079,5,1],[15079,23,1],[15079,27,1],
[15079,43,1],[15079,56,2],[15079,70,1],[15079,72,1],[15080,2,1],
[15080,5,1],[15080,23,3],[15080,24,10],[15080,25,3],[15080,27,4],
[15080,30,1],[15080,31,3],[15080,32,3],[15080,35,3],[15080,48,1],
[15080,50,2],[15080,56,4],[15080,62,1],[15080,66,1],[15081,2,8],
[15081,3,5],[15081,4,2],[15081,5,14],[15081,6,9],[15081,13,5],
[15081,20,4],[15081,21,6],[15081,22,10],[15081,23,12],[15081,24,11],
[15081,25,11],[15081,26,9],[15081,27,59],[15081,28,18],[15081,30,1],
[15081,31,6],[15081,32,8],[15081,33,26],[15081,35,48],[15081,37,2],
[15081,38,4],[15081,39,1],[15081,40,3],[15081,42,12],[15081,43,19],
[15081,44,1],[15081,46,1],[15081,47,12],[15081,48,9],[15081,50,2],
[15081,51,1],[15081,56,3],[15081,59,2],[15081,60,2],[15081,62,2],
[15081,63,2],[15081,65,1],[15081,67,1],[15081,68,2],[15081,69,2],
[15081,70,3],[15081,74,3],[15081,75,1],[15081,76,2],[15082,3,1],
[15082,23,1],[15082,27,1],[15082,55,1],[15082,62,1],[15083,3,4],
[15083,5,1],[15083,22,1],[15083,23,6],[15083,24,5],[15083,27,4],
[15083,28,2],[15083,33,1],[15083,35,1],[15083,47,1],[15083,56,1],
[15084,37,1],[15084,57,1],[15085,23,2],[15085,24,1],[15085,35,1],
[15086,21,2],[15086,22,3],[15086,23,2],[15086,24,4],[15086,25,1],
[15086,27,7],[15086,28,1],[15086,31,3],[15086,32,2],[15086,35,1],
[15086,45,1],[15086,53,1],[15087,27,1],[15087,35,1],[15088,5,1],
[15088,22,2],[15088,31,1],[15088,32,4],[15088,47,1],[15088,50,2],
[15088,55,1],[15088,63,1],[15088,70,1],[15088,76,1],[15089,6,1],
[15089,26,2],[15089,28,16],[15089,32,1],[15089,38,2],[15089,42,2],
[15089,46,1],[15089,47,1],[15089,48,7],[15089,50,1],[15089,75,1],
[15090,0,3],[15090,7,1],[15091,2,2],[15091,27,2],[15091,42,1],
[15091,62,1],[15092,22,1],[15092,23,2],[15092,24,3],[15092,28,2],
[15092,31,1],[15093,56,2],[15094,28,1],[15094,31,1],[15095,6,1],
[15095,21,2],[15095,25,1],[15096,22,2],[15096,27,3],[15096,35,2],
[15096,42,4],[15096,74,1],[15097,2,13],[15097,5,1],[15097,18,1],
[15097,22,1],[15097,23,1],[15097,24,1],[15097,26,1],[15097,27,1],
[15097,28,3],[15097,32,1],[15097,40,1],[15097,43,3],[15097,56,1],
[15097,68,1],[15098,45,1],[15098,53,1],[15099,4,1],[15099,62,1],
[15099,63,1],[15099,70,1],[15099,72,1],[15099,74,1],[15100,35,3],
[15101,30,2],[15102,23,1],[15102,24,7],[15102,56,1],[15103,4,1],
[15103,6,1],[15103,9,1],[15103,17,1],[15103,23,1],[15103,25,2],
[15103,28,1],[15103,31,1],[15103,33,29],[15103,39,1],[15103,47,1],
[15103,48,2],[15103,55,1],[15103,65,1],[15103,78,8],[15104,51,2],
[15105,35,1],[15105,47,7],[15106,3,1],[15106,24,1],[15107,22,4],

[15107,31,1],[15108,28,1],[15108,66,1],[15109,9,1],[15109,12,1],
[15109,37,1],[15109,70,1],[15110,25,1],[15110,28,3],[15110,68,1],
[15111,4,1],[15111,22,1],[15111,42,1],[15111,43,1],[15112,24,1],
[15112,31,1],[15112,62,1],[15113,2,4],[15113,3,1],[15113,5,1],
[15113,6,2],[15113,10,1],[15113,20,1],[15113,23,1],[15113,26,1],
[15113,35,1],[15113,42,1],[15113,44,2],[15113,56,1],[15113,60,1],
[15113,62,1],[15114,1,1],[15114,8,1],[15114,9,1],[15114,10,1],
[15114,13,1],[15114,20,1],[15114,23,1],[15114,27,1],[15114,28,1],
[15114,36,1],[15114,62,2],[15115,2,2],[15116,2,1],[15116,23,1],
[15116,28,1],[15116,31,2],[15116,43,1],[15117,2,1],[15117,3,1],
[15117,24,1],[15117,28,2],[15118,1,1],[15118,3,1],[15118,22,1],
[15118,23,2],[15118,24,8],[15118,25,1],[15118,27,1],[15118,28,5],
[15118,47,1],[15118,76,1],[15119,9,1],[15119,10,1],[15119,11,1],
[15119,16,2],[15119,41,1],[15119,45,1],[15119,49,1],[15119,57,1],
[15119,59,2],[15119,65,3],[15119,73,1],[15119,77,1],[15120,27,1],
[15120,35,1],[15121,27,1],[15121,31,1],[15121,45,1],[15121,72,1],
[15122,0,1],[15122,9,1],[15122,10,1],[15122,12,1],[15122,13,1],
[15122,15,2],[15122,17,2],[15122,18,1],[15122,19,1],[15122,20,2],
[15122,28,1],[15122,29,1],[15122,30,1],[15122,36,1],[15122,37,3],
[15122,39,2],[15122,41,2],[15122,45,1],[15122,46,2],[15122,53,2],
[15122,55,1],[15122,59,2],[15122,62,1],[15122,65,1],[15122,73,1],
[15122,77,1],[15123,13,1],[15123,20,1],[15123,39,1],[15124,2,4],
[15125,27,7],[15125,28,1],[15125,35,7],[15126,3,1],[15126,4,1],
[15126,24,2],[15126,26,1],[15126,35,1],[15126,43,1],[15126,47,1],
[15126,51,1],[15127,2,1],[15127,43,1],[15128,22,2],[15129,27,2],
[15130,21,1],[15130,23,1],[15130,24,1],[15130,25,5],[15130,26,1],
[15130,28,3],[15130,35,1],[15131,2,2],[15131,3,1],[15131,8,2],
[15131,27,3],[15131,30,1],[15131,32,1],[15131,46,1],[15131,60,1],
[15132,5,2],[15132,6,1],[15132,22,5],[15132,23,1],[15132,24,8],
[15132,26,4],[15132,27,2],[15132,28,1],[15132,43,8],[15132,56,1],
[15132,60,1],[15133,3,1],[15133,23,1],[15133,32,2],[15133,38,1],
[15134,0,1],[15134,2,6],[15135,1,1],[15135,53,1],[15136,35,3],
[15137,0,1331],[15137,1,12],[15137,2,1],[15137,7,1249],[15137,8,24],
[15137,9,25],[15137,10,60],[15137,11,27],[15137,12,59],[15137,13,3],
[15137,14,13],[15137,15,11],[15137,16,72],[15137,17,8],[15137,18,62],
[15137,19,49],[15137,20,10],[15137,27,1],[15137,28,1],[15137,29,5],
[15137,30,26],[15137,31,32],[15137,33,1],[15137,34,14],[15137,35,1],
[15137,36,14],[15137,37,37],[15137,39,11],[15137,41,28],[15137,42,1],
[15137,44,1],[15137,45,64],[15137,46,39],[15137,49,13],[15137,50,1],
[15137,52,1],[15137,53,9],[15137,54,13],[15137,55,6],[15137,56,1],
[15137,57,41],[15137,58,18],[15137,59,22],[15137,60,3],[15137,62,29],
[15137,63,1],[15137,64,2],[15137,65,56],[15137,67,15],[15137,70,2],
[15137,71,18],[15137,73,150],[15137,74,2],[15137,76,1],[15137,77,52],
[15138,25,8],[15138,26,1],[15138,28,1],[15138,32,1],[15138,50,1],
[15138,59,1],[15138,62,1],[15139,21,4],[15140,74,2],[15140,75,5],
[15141,22,1],[15141,35,1],[15142,31,4],[15143,28,5],[15143,44,3],
[15143,66,1],[15144,28,2],[15145,10,1],[15145,20,1],[15145,36,1],
[15146,25,2],[15147,5,5],[15147,9,1],[15147,16,1],[15147,25,4],
[15147,26,1],[15147,27,2],[15147,28,15],[15147,30,10],[15147,33,14],
[15147,35,18],[15147,38,7],[15147,47,1],[15147,52,45],[15147,62,1],

[15147,63,1],[15147,68,1],[15147,70,1],[15147,75,1],[15147,76,1],
[15148,24,1],[15148,31,3],[15148,56,1],[15149,27,1],[15149,31,1],
[15150,23,1],[15150,31,1],[15150,47,1],[15151,3,1],[15151,21,1],
[15151,23,2],[15151,25,2],[15151,26,3],[15151,27,2],[15151,28,1],
[15151,31,29],[15151,33,1],[15151,42,13],[15151,43,11],[15151,47,2],
[15151,48,1],[15151,70,1],[15152,34,1],[15152,53,1],[15153,15,1],
[15153,53,1],[15154,3,4],[15154,6,1],[15154,24,3],[15154,26,1],
[15154,28,5],[15154,31,1],[15154,56,4],[15155,23,1],[15155,31,2],
[15156,2,3],[15156,24,1],[15156,28,1],[15156,31,5],[15156,32,3],
[15156,37,1],[15157,1,3],[15157,7,1],[15157,29,1],[15158,31,1],
[15158,43,1],[15159,32,11],[15159,44,1],[15160,1,1],[15160,30,1],
[15160,60,1],[15161,1,2],[15161,5,2],[15161,9,1],[15162,22,1],
[15162,23,1],[15162,57,1],[15163,15,6],[15163,53,1],[15163,55,2],
[15164,2,1],[15164,26,1],[15165,1,1],[15165,12,1],[15166,25,1],
[15166,40,2],[15167,18,1],[15167,20,2],[15167,23,9],[15167,27,9],
[15167,34,2],[15168,0,1],[15168,9,2],[15168,12,1],[15168,13,1],
[15168,14,2],[15168,19,1],[15168,28,1],[15168,31,2],[15168,45,1],
[15168,46,1],[15168,49,1],[15168,53,1],[15168,67,1],[15168,77,2],
[15169,5,1],[15169,23,3],[15169,26,4],[15169,27,2],[15169,28,2],
[15169,31,1],[15169,35,1],[15169,42,1],[15169,43,8],[15169,56,1],
[15169,66,1],[15170,79,3],[15171,4,5],[15171,23,1],[15171,24,1],
[15171,28,4],[15171,32,1],[15171,35,1],[15171,38,12],[15171,40,1],
[15171,44,7],[15171,50,5],[15171,56,2],[15171,61,1],[15171,66,8],
[15171,68,1],[15171,75,2],[15171,76,1],[15172,26,1],[15172,28,1],
[15173,2,4],[15173,28,1],[15173,43,1],[15174,68,1],[15174,69,2],
[15175,27,1],[15175,28,1],[15176,25,1],[15176,34,1],[15176,47,2],
[15177,3,1],[15177,27,2],[15177,31,1],[15177,47,1],[15178,48,7],
[15179,3,1],[15179,5,1],[15179,6,1],[15179,22,7],[15179,24,5],
[15179,27,2],[15179,28,2],[15179,32,81],[15179,35,1],[15179,43,5],
[15179,60,3],[15179,68,1],[15180,2,3],[15180,4,1],[15180,9,1],
[15180,13,1],[15180,22,1],[15180,27,1],[15180,42,1],[15180,44,1],
[15180,56,1],[15181,24,1],[15181,28,1],[15181,74,1],[15182,22,1],
[15182,23,1],[15182,32,1],[15183,28,2],[15184,5,1],[15184,23,1],
[15184,25,1],[15185,5,3],[15185,6,1],[15185,23,2],[15185,24,1],
[15185,26,1],[15185,35,2],[15185,43,1],[15186,2,7],[15186,28,1],
[15187,0,1],[15187,1,193],[15187,2,1],[15187,3,6],[15187,4,1],
[15187,5,4],[15187,6,1],[15187,7,2],[15187,8,2],[15187,9,2],
[15187,10,4],[15187,11,7],[15187,12,21],[15187,13,68],[15187,15,5],
[15187,16,2],[15187,18,3],[15187,19,7],[15187,20,16],[15187,21,1],
[15187,22,3],[15187,23,81],[15187,24,38],[15187,25,20],[15187,26,12],
[15187,27,3],[15187,28,7],[15187,29,11],[15187,30,3],[15187,31,9],
[15187,32,5],[15187,33,47],[15187,34,21],[15187,35,3],[15187,36,2],
[15187,37,3],[15187,38,3],[15187,39,7],[15187,40,2],[15187,41,2],
[15187,42,4],[15187,43,3],[15187,45,4],[15187,46,2],[15187,47,1],
[15187,48,4],[15187,50,2],[15187,51,1],[15187,52,3],[15187,53,1],
[15187,54,1],[15187,55,2],[15187,56,1],[15187,57,1],[15187,58,1],
[15187,59,4],[15187,62,7],[15187,63,3],[15187,65,1],[15187,66,2],
[15187,69,1],[15187,70,1],[15187,71,1],[15187,72,1],[15187,73,4],
[15187,74,1],[15187,76,1],[15187,77,3],[15187,78,2],[15188,1,1],
[15188,2,3],[15188,5,1],[15188,6,3],[15188,11,1],[15188,20,1],

[15188,23,6],[15188,25,6],[15188,26,1],[15188,27,6],[15188,28,3],
[15188,31,31],[15188,32,5],[15188,33,1],[15188,34,4],[15188,35,3],
[15188,37,2],[15188,39,3],[15188,42,5],[15188,43,3],[15188,48,5],
[15188,55,1],[15188,56,16],[15188,61,6],[15188,63,1],[15188,68,1],
[15188,78,2],[15189,1,1],[15189,9,1],[15189,13,1],[15189,14,1],
[15189,19,3],[15189,34,1],[15189,57,1],[15189,67,2],[15189,71,1],
[15189,77,1],[15190,24,1],[15190,33,2],[15190,42,1],[15191,9,4],
[15192,0,1],[15192,1,3],[15192,2,1],[15192,3,2],[15192,5,1],
[15192,9,4],[15192,10,1],[15192,12,3],[15192,13,3],[15192,14,1],
[15192,15,74],[15192,16,1],[15192,17,1],[15192,18,1],[15192,21,2],
[15192,23,1],[15192,24,3],[15192,25,3],[15192,26,2],[15192,27,3],
[15192,28,28],[15192,32,1],[15192,33,2],[15192,35,3],[15192,38,1],
[15192,40,1],[15192,42,1],[15192,43,1],[15192,45,2],[15192,50,3],
[15192,53,3],[15192,54,2],[15192,55,45],[15192,56,2],[15192,58,1],
[15192,59,2],[15192,60,2],[15192,70,1],[15192,71,1],[15192,74,3],
[15192,75,1],[15192,76,1],[15192,77,2],[15192,78,1],[15193,42,1],
[15193,74,1],[15194,0,10],[15194,1,20],[15194,2,7],[15194,3,4],
[15194,4,172],[15194,5,8],[15194,7,2],[15194,8,7],[15194,9,8],
[15194,10,2],[15194,12,11],[15194,13,10],[15194,15,311],[15194,16,3],
[15194,17,931],[15194,18,1],[15194,19,2],[15194,20,19],[15194,21,2],
[15194,22,5],[15194,23,7],[15194,24,6],[15194,25,8],[15194,26,6],
[15194,27,12],[15194,28,70],[15194,29,2],[15194,30,10],[15194,31,4],
[15194,32,7],[15194,33,4],[15194,34,8],[15194,35,8],[15194,36,2],
[15194,37,5],[15194,38,8],[15194,39,10],[15194,40,4],[15194,41,1],
[15194,42,4],[15194,43,6],[15194,44,192],[15194,46,1],[15194,47,2],
[15194,48,3],[15194,49,1],[15194,50,305],[15194,51,5],[15194,52,2],
[15194,53,672],[15194,54,1],[15194,55,83],[15194,56,4],[15194,58,1],
[15194,59,3],[15194,60,3],[15194,62,7],[15194,63,4],[15194,65,1],
[15194,66,124],[15194,67,2],[15194,69,1],[15194,70,1],[15194,72,2],
[15194,73,2],[15194,74,2],[15194,75,3],[15194,76,1],[15194,78,1],
[15194,79,4],[15195,1,1],[15195,15,1],[15195,16,1],[15195,37,12],
[15195,55,2],[15195,77,1],[15196,2,2],[15196,5,3],[15196,22,1],
[15196,23,1],[15196,25,1],[15196,30,1],[15196,35,2],[15196,56,1],
[15196,68,1],[15197,15,1],[15197,53,1],[15197,55,1],[15198,23,1],
[15198,25,1],[15199,4,3],[15199,32,1],[15199,50,1],[15200,26,2],
[15201,39,1],[15201,62,1],[15202,15,3],[15202,20,1],[15202,36,1],
[15202,45,1],[15202,55,1],[15203,28,1],[15203,69,1],[15204,1,2],
[15204,3,4],[15204,7,3],[15204,12,3],[15204,13,2],[15204,15,1],
[15204,17,1],[15204,25,5],[15204,28,1],[15204,31,1],[15204,37,1],
[15204,42,2],[15204,46,1],[15204,47,3],[15204,51,1],[15204,56,3],
[15204,60,2],[15204,64,1],[15204,74,1],[15205,2,1],[15205,3,1],
[15205,4,1],[15205,8,1],[15205,23,1],[15205,27,1],[15205,32,2],
[15205,33,13],[15205,35,2],[15205,48,1],[15206,1,1],[15206,13,2],
[15206,19,1],[15206,20,3],[15206,55,2],[15206,56,1],[15207,8,2],
[15208,4,2],[15208,5,3],[15208,13,1],[15208,20,1],[15208,22,2],
[15208,23,1],[15208,24,1],[15208,25,2],[15208,27,1],[15208,28,1],
[15208,38,1],[15208,40,1],[15208,42,1],[15208,52,1],[15208,56,1],
[15208,70,1],[15209,27,1],[15209,64,1],[15210,27,1],[15210,35,1],
[15210,43,7],[15211,24,1],[15211,32,1],[15212,3,1],[15212,21,1],
[15212,24,1],[15213,1,1],[15213,9,1],[15213,10,1],[15213,13,1],

[15213,20,1],[15213,28,2],[15213,29,1],[15213,34,1],[15213,42,1],
[15213,58,1],[15213,59,1],[15214,33,2],[15215,24,1],[15215,56,1],
[15216,1,2],[15216,35,1],[15216,47,1],[15216,51,1],[15217,21,1],
[15217,27,1],[15217,32,1],[15217,33,1],[15217,62,1],[15218,23,2],
[15219,31,1],[15219,32,1],[15219,47,1],[15220,4,1],[15220,31,1],
[15220,42,1],[15220,56,1],[15221,23,1],[15221,31,1],[15221,42,1],
[15221,59,1],[15221,71,1],[15222,25,1],[15222,32,3],[15223,2,2],
[15224,22,1],[15224,27,1],[15224,33,1],[15225,1,1],[15225,12,1],
[15225,13,3],[15225,21,1],[15225,32,2],[15225,33,2],[15225,39,1],
[15225,42,1],[15225,46,1],[15225,59,1],[15225,68,1],[15225,71,1],
[15226,3,1],[15226,6,1],[15226,23,1],[15227,1,7],[15227,21,2],
[15227,23,1],[15227,24,1],[15227,25,1],[15227,26,1],[15227,27,3],
[15227,28,2],[15227,31,2],[15227,34,11],[15227,36,1],[15227,43,2],
[15227,62,1],[15228,6,1],[15228,21,1],[15228,22,3],[15228,23,2],
[15228,27,3],[15228,32,1],[15228,33,2],[15228,35,2],[15228,42,1],
[15228,43,1],[15228,47,1],[15228,49,1],[15228,56,3],[15228,74,1],
[15228,76,1],[15229,32,2],[15230,2,2],[15230,24,1],[15231,2,21],
[15231,3,3],[15231,4,1],[15231,5,1],[15231,6,1],[15231,15,1],
[15231,19,1],[15231,22,2],[15231,23,4],[15231,24,4],[15231,26,1],
[15231,30,5],[15231,31,1],[15231,34,1],[15231,35,3],[15231,43,1],
[15231,47,3],[15231,59,3],[15231,61,1],[15231,63,1],[15231,72,2],
[15231,76,1],[15232,15,1],[15232,18,1],[15232,34,1],[15233,17,1],
[15233,55,2],[15233,56,1],[15233,58,1],[15233,65,1],[15233,77,1],
[15234,48,5],[15235,1,1],[15235,24,1],[15235,32,1],[15235,38,1],
[15235,44,1],[15235,65,1],[15235,66,1],[15235,70,1],[15236,39,5],
[15237,1,3],[15237,2,1],[15237,3,2],[15237,20,3],[15237,22,1],
[15237,23,4],[15237,24,3],[15237,26,1],[15237,28,4],[15237,31,2],
[15237,32,2],[15237,34,1],[15238,28,1],[15238,51,2],[15239,10,1],
[15239,15,1],[15239,53,1],[15240,28,1],[15240,43,2],[15241,2,1],
[15241,3,1],[15241,22,4],[15241,26,1],[15241,35,1],[15241,43,1],
[15241,47,1],[15241,56,3],[15241,68,1],[15241,70,1],[15242,25,5],
[15243,3,1],[15243,40,1],[15244,27,5],[15244,28,1],[15244,35,6],
[15244,40,1],[15244,44,1],[15245,8,1],[15245,26,1],[15245,28,2],
[15246,32,2],[15247,22,1],[15247,28,1],[15247,33,1],[15247,66,1],
[15248,1,18],[15248,46,1],[15248,57,1],[15249,31,1],[15249,32,2],
[15249,48,2],[15250,1,1],[15250,24,1],[15250,44,1],[15250,77,1],
[15251,3,1],[15251,35,1],[15252,2,1],[15252,3,1],[15252,16,3],
[15252,17,1],[15252,22,28],[15252,25,13],[15252,27,26],[15252,28,27],
[15252,29,1],[15252,30,17],[15252,32,52],[15252,33,128],[15252,34,1],
[15252,35,40],[15252,38,6],[15252,52,31],[15252,59,1],[15252,62,2],
[15252,66,1],[15252,67,1],[15252,73,4],[15252,76,1],[15253,17,1],
[15253,28,1],[15254,26,1],[15254,60,1],[15255,5,1],[15255,22,1],
[15256,26,1],[15256,28,1],[15256,43,1],[15256,47,1],[15257,24,1],
[15257,28,1],[15258,28,47],[15258,31,1],[15258,38,1],[15258,44,1],
[15259,8,1],[15259,25,2],[15259,33,3],[15259,35,7],[15260,4,1],
[15260,17,1],[15260,44,2],[15260,50,2],[15260,66,2],[15261,6,2],
[15261,22,1],[15261,39,1],[15261,42,1],[15261,51,2],[15261,56,1],
[15261,62,1],[15261,68,2],[15261,70,4],[15261,74,2],[15261,75,1],
[15262,37,1],[15262,57,1],[15263,9,1],[15263,15,3],[15263,19,3],
[15263,28,1],[15263,39,2],[15263,41,1],[15263,55,8],[15263,65,1],

[15264,2,9],[15264,3,1],[15264,9,1],[15264,12,1],[15264,17,1],
[15264,20,2],[15264,21,2],[15264,22,1],[15264,23,6],[15264,26,2],
[15264,27,7],[15264,30,1],[15264,31,4],[15264,32,2],[15264,36,1],
[15264,39,5],[15264,42,1],[15264,43,2],[15264,47,1],[15264,58,1],
[15264,68,1],[15265,2,2],[15265,40,1],[15266,1,1],[15266,57,1],
[15266,59,1],[15267,2,1],[15267,28,2],[15268,2,2],[15268,10,1],
[15268,20,15],[15268,23,6],[15268,26,1],[15268,30,1],[15268,31,3],
[15268,32,6],[15268,33,1],[15268,39,1],[15268,42,3],[15268,45,1],
[15268,47,1],[15269,2,2],[15269,22,1],[15269,24,2],[15269,26,1],
[15269,35,1],[15270,23,1],[15270,27,1],[15271,1,1],[15271,8,1],
[15271,9,2],[15271,11,1],[15271,12,1],[15271,19,1],[15271,20,1],
[15271,29,1],[15271,45,1],[15271,57,1],[15271,59,1],[15271,71,1],
[15272,23,1],[15272,42,1],[15273,2,3],[15273,3,1],[15273,20,1],
[15273,22,1],[15273,26,1],[15273,27,1],[15273,56,2],[15274,21,3],
[15274,27,2],[15275,26,1],[15275,27,2],[15275,28,1],[15276,2,2],
[15276,5,3],[15276,10,1],[15276,22,1],[15276,27,2],[15276,30,1],
[15276,33,1],[15276,47,1],[15276,48,1],[15276,53,1],[15276,60,1],
[15277,15,1],[15277,16,1],[15278,0,3],[15278,39,1],[15279,29,3],
[15280,13,1],[15280,20,1],[15280,23,1],[15280,28,1],[15280,31,1],
[15281,2,1],[15281,5,1],[15281,6,2],[15281,13,1],[15281,20,1],
[15281,21,1],[15281,22,2],[15281,23,1],[15281,24,4],[15281,26,3],
[15281,27,2],[15281,33,3],[15281,42,3],[15281,43,1],[15281,59,1],
[15282,25,1],[15282,70,2],[15283,15,1],[15283,20,1],[15283,57,1],
[15283,71,1],[15283,77,1],[15284,17,4],[15284,50,2],[15284,53,4],
[15285,2,1],[15285,20,1],[15285,27,1],[15286,28,1],[15286,55,1],
[15287,20,2],[15288,6,1],[15288,23,3],[15288,50,1],[15288,56,1],
[15289,28,2],[15289,31,1],[15289,63,1],[15290,21,2],[15290,25,4],
[15290,28,2],[15290,33,15],[15290,37,1],[15290,68,1],[15291,28,1],
[15291,31,1],[15292,20,1],[15292,56,1],[15292,70,1],[15293,3,1],
[15293,23,1],[15293,24,2],[15293,28,1],[15294,1,1],[15294,8,1],
[15294,23,3],[15294,31,1],[15294,33,2],[15294,34,1],[15294,39,1],
[15294,42,1],[15294,43,2],[15295,24,1],[15295,25,2],[15295,27,3],
[15295,28,3],[15295,35,2],[15295,36,1],[15295,47,1],[15295,48,1],
[15295,50,1],[15295,51,1],[15295,53,1],[15296,2,1],[15296,12,9],
[15296,29,1],[15297,4,2],[15297,50,6],[15297,53,1],[15298,31,3],
[15299,2,4],[15299,20,1],[15299,30,1],[15300,10,2],[15300,20,3],
[15300,21,1],[15300,23,14],[15300,24,4],[15300,25,2],[15300,26,1],
[15300,27,7],[15300,28,6],[15300,31,1],[15300,32,2],[15300,35,3],
[15300,37,1],[15300,47,1],[15300,48,2],[15300,59,2],[15300,68,1],
[15300,69,1],[15300,70,1],[15300,72,1],[15301,24,1],[15301,42,1],
[15302,2,1],[15302,21,1],[15302,23,1],[15302,27,1],[15302,33,1],
[15303,1,3],[15303,10,1],[15303,13,2],[15303,20,1],[15303,35,1],
[15303,41,1],[15303,67,1],[15303,78,1],[15304,21,1],[15304,33,1],
[15304,42,1],[15305,6,1],[15305,24,2],[15305,47,1],[15305,51,1],
[15305,61,1],[15305,68,1],[15305,69,2],[15305,70,2],[15306,21,2],
[15306,22,2],[15306,26,1],[15306,32,1],[15306,65,1],[15306,74,2],
[15306,77,1],[15307,22,1],[15307,28,1],[15308,1,3],[15308,12,9],
[15308,13,9],[15308,28,1],[15309,4,6],[15309,38,1],[15309,44,1],
[15309,50,6],[15309,66,3],[15310,5,1],[15310,6,1],[15310,22,3],
[15310,24,9],[15310,25,21],[15310,26,9],[15310,32,1],[15310,35,1],

[15310,42,1],[15310,43,1],[15310,47,3],[15311,24,1],[15311,27,1],
[15311,35,1],[15312,27,1],[15312,28,38],[15313,2,1],[15313,35,2],
[15313,61,1],[15313,62,1],[15313,68,1],[15314,20,7],[15315,28,1],
[15315,55,1],[15316,40,1],[15316,63,3],[15316,68,1],[15316,74,2],
[15316,75,1],[15316,76,4],[15317,20,1],[15317,34,1],[15317,49,1],
[15318,20,2],[15318,24,1],[15318,37,1],[15318,38,1],[15318,42,2],
[15318,53,1],[15318,59,1],[15319,20,1],[15319,54,1],[15320,5,1],
[15320,17,1],[15320,38,1],[15321,25,1],[15321,39,1],[15322,20,1],
[15322,27,1],[15322,43,1],[15323,23,1],[15323,25,1],[15323,45,1],
[15323,59,4],[15323,65,1],[15324,0,1],[15324,1,17],[15324,2,2],
[15324,12,3],[15324,13,3],[15324,15,2],[15324,16,1],[15324,19,1],
[15324,20,1],[15324,31,8],[15324,42,2],[15324,45,1],[15324,55,1],
[15324,77,1],[15325,32,2],[15326,1,3],[15326,10,1],[15326,20,10],
[15326,22,1],[15326,23,4],[15326,24,3],[15326,26,1],[15326,27,2],
[15326,28,1],[15326,30,1],[15326,32,5],[15326,33,4],[15326,43,1],
[15326,49,1],[15326,59,1],[15327,9,1],[15327,10,3],[15327,14,1],
[15327,15,1],[15327,19,1],[15327,20,2],[15327,30,1],[15327,34,1],
[15327,41,1],[15327,45,2],[15327,46,2],[15327,49,1],[15327,53,1],
[15327,54,1],[15327,59,3],[15327,60,1],[15327,73,1],[15328,16,1],
[15328,71,1],[15329,25,1],[15329,28,1],[15330,2,1],[15330,6,1],
[15330,22,6],[15330,23,3],[15330,24,20],[15330,25,1],[15330,26,1],
[15330,27,10],[15330,28,11],[15330,32,2],[15330,35,2],[15330,38,4],
[15330,48,1],[15330,56,2],[15330,61,1],[15330,62,1],[15331,2,3],
[15331,3,6],[15331,5,4],[15331,6,1],[15331,8,2],[15331,10,1],
[15331,12,1],[15331,20,19],[15331,21,8],[15331,22,15],[15331,23,117],
[15331,24,42],[15331,25,2],[15331,26,37],[15331,27,23],[15331,28,28],
[15331,30,2],[15331,31,25],[15331,32,28],[15331,33,3],[15331,34,1],
[15331,35,11],[15331,39,1],[15331,42,9],[15331,43,18],[15331,47,4],
[15331,48,1],[15331,51,1],[15331,56,1],[15331,59,11],[15331,60,1],
[15331,62,1],[15331,63,1],[15331,68,2],[15331,69,1],[15331,70,1],
[15331,73,1],[15331,76,3],[15332,3,1],[15332,23,1],[15332,25,1],
[15332,28,1],[15332,47,1],[15333,23,1],[15333,63,3],[15333,72,1],
[15333,74,4],[15333,75,1],[15333,76,10],[15334,10,1],[15334,15,1],
[15335,2,1],[15335,24,1],[15335,33,1],[15335,67,1],[15335,68,44],
[15335,69,1],[15335,72,1],[15336,21,1],[15336,31,1],[15337,23,87],
[15337,26,1],[15337,27,1],[15337,28,1],[15337,35,1],[15337,38,1],
[15337,44,1],[15337,66,2],[15338,22,1],[15338,33,1],[15339,0,1],
[15339,1,7],[15339,8,1],[15339,9,4],[15339,10,11],[15339,11,2],
[15339,12,3],[15339,13,3],[15339,15,4],[15339,16,3],[15339,17,5],
[15339,18,1],[15339,19,5],[15339,20,2],[15339,25,2],[15339,29,1],
[15339,30,3],[15339,34,2],[15339,37,1],[15339,39,3],[15339,41,2],
[15339,45,3],[15339,46,1],[15339,49,1],[15339,53,4],[15339,55,6],
[15339,57,1],[15339,58,8],[15339,59,4],[15339,60,2],[15339,64,1],
[15339,65,8],[15339,67,3],[15339,71,2],[15339,73,1],[15339,77,2],
[15340,5,1],[15340,26,1],[15340,28,1],[15340,70,1],[15341,3,1],
[15341,23,1],[15342,26,1],[15342,27,3],[15342,47,1],[15343,25,2],
[15343,26,1],[15343,33,3],[15344,2,4],[15344,5,1],[15344,22,1],
[15344,27,1],[15344,28,1],[15344,56,2],[15345,15,1],[15345,44,1],
[15345,66,1],[15346,5,2],[15346,15,1],[15346,20,2],[15346,21,6],
[15346,22,3],[15346,23,3],[15346,24,1],[15346,25,5],[15346,26,11],

[15346,27,8],[15346,28,3],[15346,31,3],[15346,32,9],[15346,33,5],
[15346,35,8],[15346,42,1],[15346,43,5],[15346,47,2],[15346,52,1],
[15347,2,1],[15347,3,1],[15347,22,1],[15347,24,1],[15347,27,1],
[15347,28,4],[15347,33,1],[15347,35,2],[15347,38,1],[15347,40,1],
[15347,63,1],[15347,66,1],[15348,5,1],[15348,21,4],[15348,26,6],
[15348,28,1],[15348,31,4],[15348,35,1],[15348,43,3],[15349,55,1],
[15349,65,1],[15350,28,5],[15350,33,1],[15351,16,1],[15351,19,1],
[15352,3,3],[15352,28,1],[15352,47,2],[15353,21,4],[15354,15,1],
[15354,55,1],[15355,20,4],[15355,22,2],[15355,23,1],[15356,31,3],
[15356,68,1],[15357,2,4],[15357,3,1],[15357,31,2],[15357,43,1],
[15357,60,1],[15358,31,1],[15358,33,1],[15358,42,1],[15359,8,1],
[15359,21,1],[15359,36,1],[15359,45,2],[15359,46,1],[15359,51,1],
[15359,54,2],[15359,79,1],[15360,4,3],[15360,17,4],[15360,36,1],
[15360,44,1],[15360,50,2],[15360,53,2],[15360,55,4],[15361,22,1],
[15361,33,1],[15362,42,3],[15363,13,1],[15363,22,2],[15363,23,1],
[15363,25,3],[15363,27,1],[15363,30,4],[15363,31,2],[15363,32,2],
[15363,33,9],[15363,35,13],[15363,38,3],[15363,50,1],[15363,52,15],
[15363,56,3],[15363,58,1],[15363,63,1],[15363,65,1],[15363,66,1],
[15363,72,2],[15363,75,1],[15364,28,2],[15364,50,1],[15364,52,8],
[15365,4,1],[15365,15,1],[15365,31,1],[15365,35,1],[15365,74,1],
[15366,23,1],[15366,26,1],[15366,27,1],[15366,43,1],[15367,28,10],
[15367,32,1],[15367,47,1],[15367,62,1],[15368,2,1],[15368,28,1],
[15369,77,1],[15369,79,1],[15370,28,9],[15371,31,2],[15372,5,1],
[15372,6,1],[15372,24,5],[15372,28,1],[15372,35,1],[15373,28,4],
[15374,53,1],[15374,73,1],[15375,28,2],[15375,74,1],[15376,26,3],
[15376,28,1],[15377,5,3],[15377,24,6],[15377,26,15],[15377,33,1],
[15377,38,1],[15377,42,3],[15377,43,12],[15377,44,1],[15377,50,1],
[15378,26,1],[15378,70,1],[15379,2,1],[15379,57,1],[15379,60,1],
[15379,71,1],[15380,26,1],[15380,28,1],[15381,24,1],[15381,27,1],
[15381,31,2],[15382,31,1],[15382,53,1],[15383,22,1],[15383,25,1],
[15383,28,1],[15383,48,2],[15384,56,1],[15384,74,1],[15385,2,1],
[15385,25,1],[15385,32,1],[15385,48,1],[15386,36,3],[15386,60,1],
[15387,23,2],[15387,24,2],[15387,27,1],[15387,32,1],[15387,35,3],
[15387,62,1],[15388,3,2],[15388,9,1],[15388,15,1],[15388,24,1],
[15388,25,1],[15388,30,2],[15388,44,2],[15388,51,1],[15388,56,2],
[15388,58,1],[15388,65,1],[15388,68,2],[15388,69,3],[15388,74,2],
[15388,76,1],[15389,28,1],[15389,74,1],[15390,0,1],[15390,8,1],
[15390,20,1],[15390,36,1],[15391,20,3],[15392,1,6],[15392,13,1],
[15393,20,1],[15393,23,1],[15393,24,1],[15393,35,1],[15393,45,1],
[15394,27,1],[15394,31,1],[15395,7,1],[15395,9,4],[15395,18,1],
[15395,19,1],[15395,36,1],[15395,45,1],[15395,46,1],[15395,65,1],
[15396,2,2],[15397,2,1],[15397,23,1],[15398,27,2],[15398,70,1],
[15398,74,2],[15399,25,1],[15399,26,1],[15399,28,5],[15399,35,1],
[15399,42,2],[15399,43,1],[15399,44,2],[15399,48,1],[15399,62,1],
[15399,69,1],[15399,70,1],[15399,72,1],[15400,1,1],[15400,8,1],
[15400,13,22],[15400,16,1],[15400,20,1],[15400,21,3],[15400,25,1],
[15400,26,1],[15400,28,1],[15400,31,1],[15400,42,1],[15400,43,3],
[15400,45,1],[15400,46,1],[15400,53,1],[15401,2,2],[15401,3,1],
[15401,4,2],[15401,5,1],[15401,10,1],[15401,19,1],[15401,21,1],
[15401,22,1],[15401,23,2],[15401,24,1],[15401,25,3],[15401,26,1],

[15401,27,1],[15401,33,3],[15401,35,1],[15401,38,1],[15401,40,2],
[15401,42,1],[15401,44,1],[15401,50,5],[15401,62,2],[15401,63,1],
[15401,66,4],[15401,68,1],[15401,70,2],[15401,72,1],[15402,2,1],
[15402,4,11],[15402,5,1],[15402,15,1],[15402,17,6],[15402,50,16],
[15402,53,6],[15402,55,1],[15402,62,1],[15402,70,1],[15403,38,3],
[15404,62,3],[15405,28,1],[15405,33,1],[15406,23,1],[15406,26,4],
[15406,28,1],[15406,31,39],[15406,33,1],[15406,39,3],[15406,40,1],
[15406,42,1],[15406,47,1],[15406,52,2],[15406,60,1],[15406,70,2],
[15407,31,3],[15408,5,1],[15408,21,1],[15409,9,1],[15409,57,1],
[15410,44,1],[15410,66,2],[15411,18,1],[15411,77,1],[15412,2,4],
[15412,3,1],[15412,6,2],[15412,10,1],[15412,13,2],[15412,15,1],
[15412,22,1],[15412,24,4],[15412,26,1],[15412,27,5],[15412,30,1],
[15412,33,1],[15412,35,1],[15412,38,1],[15412,43,1],[15412,47,1],
[15412,52,1],[15412,74,1],[15413,2,4],[15413,42,1],[15414,27,6],
[15414,28,1],[15414,32,1],[15414,64,1],[15415,23,1],[15415,32,1],
[15415,43,1],[15416,8,1],[15416,9,1],[15416,13,1],[15416,27,2],
[15416,39,1],[15417,13,1],[15417,65,1],[15418,75,2],[15418,76,1],
[15419,24,1],[15419,26,1],[15419,31,1],[15420,2,4],[15420,42,1],
[15421,40,1],[15421,46,1],[15421,66,1],[15422,15,3],[15422,17,4],
[15422,19,1],[15422,53,1],[15423,23,1],[15423,31,43],[15423,33,5],
[15423,35,1],[15423,39,2],[15423,42,6],[15423,52,2],[15423,69,1],
[15424,1,3],[15424,11,1],[15424,13,2],[15424,15,1],[15424,17,1],
[15424,19,1],[15424,20,2],[15424,55,2],[15424,73,1],[15425,4,1],
[15425,15,1],[15425,17,2],[15425,50,1],[15425,53,3],[15425,55,3],
[15426,5,1],[15426,27,1],[15427,33,4],[15428,22,1],[15428,24,1],
[15428,27,1],[15428,28,3],[15429,19,1],[15429,20,9],[15429,26,1],
[15429,39,1],[15429,65,1],[15430,2,3],[15430,5,1],[15430,21,1],
[15430,24,1],[15431,2,1],[15431,25,12],[15431,31,1],[15432,2,2],
[15432,31,1],[15433,22,1],[15433,28,1],[15433,39,1],[15434,6,2],
[15434,48,1],[15435,5,1],[15435,24,1],[15435,28,1],[15435,31,1],
[15435,35,1],[15435,52,1],[15436,28,1],[15436,44,1],[15436,72,1],
[15437,6,4],[15437,24,1],[15437,27,2],[15437,42,1],[15437,44,1],
[15437,55,1],[15437,68,1],[15437,74,3],[15438,3,2],[15438,16,2],
[15438,20,1],[15438,36,1],[15438,38,1],[15438,45,1],[15438,55,1],
[15438,77,2],[15439,28,1],[15439,43,1],[15440,1,1],[15440,15,6],
[15440,55,2],[15440,66,1],[15441,75,1],[15441,76,1],[15442,10,1],
[15442,19,1],[15443,21,1],[15443,24,2],[15443,25,14],[15443,26,1],
[15443,29,1],[15443,31,1],[15444,3,1],[15444,6,1],[15444,9,1],
[15444,20,1],[15444,23,1],[15444,24,1],[15444,28,1],[15444,31,1],
[15444,33,1],[15444,53,1],[15445,44,2],[15446,28,9],[15447,13,2],
[15447,17,1],[15447,77,1],[15448,2,1],[15448,3,3],[15448,5,1],
[15448,13,2],[15448,20,2],[15448,22,1],[15448,23,1],[15448,24,6],
[15448,27,1],[15448,28,3],[15448,30,2],[15448,31,3],[15448,33,1],
[15448,42,2],[15448,43,1],[15448,45,1],[15448,46,1],[15448,47,1],
[15448,48,1],[15448,50,1],[15448,59,1],[15448,60,3],[15448,67,1],
[15448,69,1],[15449,35,5],[15449,52,28],[15450,31,6],[15450,56,1],
[15451,1,1],[15451,59,1],[15451,65,1],[15452,0,1],[15452,1,2],
[15452,10,1],[15452,12,1],[15452,14,1],[15452,16,1],[15452,17,1],
[15452,19,2],[15452,37,1],[15452,39,1],[15452,46,1],[15452,54,1],
[15452,55,1],[15452,57,1],[15452,59,2],[15452,60,1],[15452,71,1],

[15453,9,1],[15453,66,2],[15454,15,1],[15454,17,2],[15454,44,1],
[15454,53,1],[15454,55,1],[15455,53,2],[15455,55,1],[15456,2,3],
[15456,3,1],[15456,5,1],[15456,43,1],[15456,50,1],[15457,2,1],
[15457,3,1],[15457,16,1],[15457,70,1],[15458,17,6],[15458,53,5],
[15459,25,5],[15460,68,2],[15461,1,1],[15461,29,1],[15462,28,4],
[15463,1,1],[15463,13,1],[15463,15,1],[15463,23,1],[15463,38,1],
[15463,39,1],[15463,55,1],[15464,22,1],[15464,27,1],[15464,56,1],
[15465,8,2],[15466,9,2],[15466,34,1],[15466,45,1],[15466,60,1],
[15466,65,2],[15466,67,1],[15466,71,1],[15467,23,1],[15467,28,1],
[15468,2,1],[15468,3,1],[15468,12,4],[15468,22,1],[15468,26,1],
[15468,29,2],[15468,42,1],[15469,31,1],[15469,43,1],[15469,56,1],
[15469,60,1],[15469,62,1],[15469,65,1],[15470,2,1],[15470,28,1],
[15471,13,1],[15471,44,1],[15472,3,1],[15472,24,1],[15473,2,1],
[15473,27,1],[15473,62,1],[15474,8,1],[15474,23,1],[15475,2,14],
[15475,56,1],[15476,26,1],[15476,28,1],[15477,4,1],[15477,5,2],
[15477,32,2],[15478,26,1],[15478,35,1],[15478,56,1],[15479,29,1],
[15479,58,1],[15480,63,11],[15480,74,7],[15480,75,9],[15480,76,15],
[15481,22,2],[15481,23,2],[15481,28,1],[15482,51,1],[15482,62,1],
[15483,22,1],[15483,33,1],[15484,24,1],[15484,26,3],[15485,22,2],
[15485,43,22],[15486,40,2],[15486,70,1],[15487,0,1],[15487,24,1],
[15488,28,1],[15488,33,1],[15489,2,15],[15489,27,1],[15490,38,2],
[15491,3,1],[15491,10,1],[15491,13,1],[15491,67,1],[15492,2,18],
[15492,3,24],[15492,5,3],[15492,6,2],[15492,8,1],[15492,10,1],
[15492,19,1],[15492,20,4],[15492,21,7],[15492,22,21],[15492,23,28],
[15492,24,31],[15492,25,23],[15492,26,12],[15492,27,69],[15492,28,15],
[15492,30,4],[15492,31,19],[15492,32,17],[15492,33,5],[15492,34,2],
[15492,35,28],[15492,36,1],[15492,38,1],[15492,39,3],[15492,42,6],
[15492,43,17],[15492,44,1],[15492,45,1],[15492,46,1],[15492,47,6],
[15492,48,1],[15492,50,1],[15492,55,1],[15492,56,1],[15492,59,1],
[15492,62,3],[15492,65,1],[15493,23,2],[15493,27,3],[15493,31,1],
[15493,35,2],[15493,43,1],[15494,17,1],[15494,23,1],[15495,24,1],
[15495,47,1],[15496,24,1],[15496,26,1],[15497,0,2],[15497,1,2],
[15497,5,1],[15497,10,1],[15497,12,5],[15497,15,5],[15497,17,36],
[15497,20,2],[15497,28,9],[15497,29,2],[15497,32,1],[15497,36,2],
[15497,39,6],[15497,53,37],[15497,55,9],[15497,59,1],[15497,67,1],
[15498,1,1],[15498,12,1],[15498,39,2],[15499,3,1],[15499,59,1],
[15500,1,1],[15500,2,1],[15500,9,1],[15500,27,1],[15500,28,3],
[15501,58,1],[15501,64,1],[15502,5,1],[15502,56,1],[15503,22,1],
[15503,24,2],[15503,26,15],[15503,32,2],[15503,43,63],[15503,72,1],
[15504,25,2],[15505,15,3],[15506,28,1],[15506,31,6],[15506,43,1],
[15506,50,1],[15507,2,1],[15507,56,1],[15508,3,1],[15508,22,1],
[15508,25,1],[15508,26,1],[15508,28,16],[15508,40,4],[15508,62,1],
[15508,63,1],[15508,70,1],[15509,3,1],[15509,23,3],[15509,24,1],
[15509,27,2],[15509,35,1],[15509,77,1],[15510,12,1],[15510,23,1],
[15510,24,1],[15510,26,1],[15510,35,1],[15510,42,3],[15510,47,1],
[15510,50,1],[15510,66,1],[15511,32,2],[15511,54,1],[15512,32,2],
[15513,12,1],[15513,14,1],[15513,20,1],[15513,55,1],[15513,59,1],
[15514,1,1],[15514,13,1],[15515,23,1],[15515,51,6],[15516,33,1],
[15516,43,2],[15517,3,1],[15517,26,1],[15517,28,1],[15518,27,10],
[15518,35,1],[15518,63,1],[15518,70,1],[15519,20,1],[15519,24,1],

[15520,29,5],[15521,13,1],[15521,34,1],[15521,36,1],[15522,28,1],
[15522,70,1],[15523,22,2],[15523,61,2],[15523,68,2],[15523,70,1],
[15524,2,2],[15524,5,3],[15524,20,3],[15524,23,1],[15524,26,2],
[15524,28,1],[15524,31,6],[15524,42,5],[15524,47,1],[15525,23,1],
[15525,24,1],[15526,2,1],[15526,3,1],[15526,4,1],[15526,27,1],
[15527,63,2],[15527,72,1],[15527,74,3],[15527,75,5],[15527,76,1],
[15528,26,1],[15528,32,4],[15529,22,2],[15529,25,1],[15530,2,2],
[15530,3,2],[15530,15,1],[15530,22,1],[15530,23,1],[15530,24,4],
[15530,25,1],[15530,27,3],[15530,28,1],[15530,42,1],[15530,53,1],
[15530,56,1],[15530,59,1],[15530,62,1],[15531,5,3],[15532,28,1],
[15532,31,6],[15533,8,1],[15533,9,1],[15533,11,1],[15533,45,1],
[15533,67,1],[15534,33,2],[15535,27,4],[15535,32,10],[15536,8,2],
[15536,24,1],[15536,29,1],[15536,30,1],[15536,32,6],[15536,42,1],
[15536,53,1],[15537,8,1],[15537,27,1],[15538,8,1],[15538,24,1],
[15538,43,1],[15539,2,1],[15539,3,1],[15539,20,1],[15540,28,6],
[15540,42,3],[15540,66,1],[15541,0,1],[15541,1,254],[15541,8,1],
[15541,9,4],[15541,10,2],[15541,11,2],[15541,12,3],[15541,13,4],
[15541,15,1],[15541,16,1],[15541,18,1],[15541,19,4],[15541,26,1],
[15541,29,1],[15541,30,1],[15541,33,4],[15541,36,1],[15541,37,3],
[15541,39,1],[15541,41,1],[15541,43,2],[15541,46,2],[15541,47,1],
[15541,53,1],[15541,54,2],[15541,57,3],[15541,59,1],[15541,64,2],
[15541,65,1],[15541,67,1],[15541,71,1],[15542,2,1],[15542,37,1],
[15542,47,1],[15543,19,1],[15543,57,1],[15544,27,1],[15544,62,1],
[15545,24,1],[15545,47,1],[15545,56,2],[15546,2,2],[15546,3,2],
[15546,8,4],[15546,20,1],[15546,23,2],[15546,24,1],[15546,26,1],
[15546,28,3],[15546,32,1],[15546,43,1],[15546,47,8],[15546,53,1],
[15546,59,3],[15546,74,1],[15547,28,2],[15548,5,3],[15548,9,1],
[15548,10,1],[15548,12,2],[15548,24,1],[15548,25,1],[15548,28,1],
[15548,35,3],[15548,45,2],[15548,65,1],[15549,51,2],[15549,69,1],
[15550,1,1],[15550,24,1],[15550,30,1],[15550,33,2],[15551,3,3],
[15551,24,1],[15551,25,1],[15551,32,4],[15551,42,1],[15551,43,1],
[15551,47,1],[15551,56,1],[15551,72,1],[15551,74,1],[15551,76,1],
[15552,4,2],[15552,17,5],[15552,20,1],[15552,21,1],[15552,24,1],
[15552,27,1],[15552,28,3],[15552,34,1],[15552,47,1],[15552,53,7],
[15553,23,5],[15554,23,3],[15554,24,1],[15554,27,1],[15554,28,1],
[15554,35,1],[15555,4,2],[15555,15,1],[15555,23,1],[15555,27,1],
[15555,28,3],[15555,43,1],[15555,44,1],[15555,50,2],[15555,66,2],
[15555,72,1],[15556,27,1],[15556,56,1],[15557,2,1],[15557,27,1],
[15558,26,2],[15558,43,1],[15559,25,2],[15560,35,5],[15561,22,2],
[15561,23,1],[15561,26,1],[15561,27,2],[15561,31,1],[15561,32,1],
[15561,35,2],[15561,39,1],[15562,10,1],[15562,31,1],[15562,56,1],
[15563,28,1],[15563,33,1],[15563,70,1],[15564,15,1],[15564,51,4],
[15564,56,3],[15565,33,3],[15565,35,4],[15566,13,1],[15566,20,1],
[15566,27,9],[15566,28,6],[15566,35,13],[15566,52,1],[15567,48,2],
[15568,35,4],[15568,62,7],[15568,76,1],[15569,28,1],[15569,33,2],
[15570,1,1],[15570,5,5],[15570,6,1],[15570,23,2],[15570,24,1],
[15570,27,1],[15570,33,1],[15570,37,1],[15570,38,1],[15570,43,1],
[15570,53,1],[15570,56,1],[15571,31,2],[15572,23,2],[15572,24,2],
[15572,27,1],[15572,28,2],[15572,31,3],[15572,43,1],[15573,23,1],
[15573,27,1],[15573,33,1],[15574,15,1],[15574,53,1],[15575,23,2],

[15576,27,1],[15576,32,1],[15576,42,1],[15576,43,3],[15577,1,3],
[15577,5,1],[15577,13,1],[15577,15,1],[15577,20,1],[15577,23,1],
[15577,28,2],[15577,39,1],[15578,42,1],[15578,43,1],[15579,13,1],
[15579,19,1],[15579,41,1],[15579,65,1],[15580,48,1],[15580,56,1],
[15580,61,1],[15581,9,1],[15581,11,1],[15582,23,2],[15582,35,3],
[15583,3,1],[15583,31,1],[15583,47,1],[15584,31,1],[15584,63,4],
[15584,74,6],[15584,75,6],[15584,76,5],[15585,1,1],[15585,9,1],
[15585,11,1],[15585,12,1],[15585,16,1],[15585,18,1],[15585,58,1],
[15585,59,1],[15585,65,1],[15585,67,1],[15586,22,3],[15586,42,1],
[15587,9,1],[15587,19,1],[15587,34,1],[15587,57,1],[15587,73,1],
[15588,28,1],[15588,74,1],[15589,21,4],[15589,25,1],[15589,26,3],
[15589,33,1],[15589,43,3],[15589,47,2],[15589,48,1],[15590,62,3],
[15591,5,1],[15591,6,1],[15591,23,4],[15591,25,1],[15591,32,5],
[15591,33,1],[15591,35,1],[15592,42,3],[15593,23,1],[15593,28,1],
[15593,56,1],[15594,1,1],[15594,3,1],[15594,5,1],[15594,13,1],
[15594,16,1],[15594,24,1],[15594,29,1],[15594,50,1],[15594,53,1],
[15594,55,1],[15594,56,3],[15595,26,1],[15595,28,2],[15595,38,1],
[15596,12,2],[15596,45,1],[15596,55,1],[15597,21,1],[15597,27,1],
[15598,26,1],[15598,31,1],[15598,42,1],[15599,37,3],[15600,27,1],
[15600,32,1],[15601,31,2],[15602,23,1],[15602,25,2],[15602,31,2],
[15603,3,2],[15603,26,1],[15604,1,1],[15604,13,1],[15605,3,1],
[15605,63,8],[15605,66,1],[15605,74,4],[15605,75,3],[15605,76,7],
[15606,24,2],[15607,28,1],[15607,47,1],[15608,28,1],[15608,32,1],
[15609,43,2],[15610,9,1],[15610,20,29],[15610,78,1],[15611,1,54],
[15611,2,1],[15611,4,1],[15611,8,3],[15611,10,1],[15611,12,3],
[15611,13,7],[15611,15,1],[15611,17,2],[15611,20,1],[15611,21,3],
[15611,23,1],[15611,24,1],[15611,25,5],[15611,26,2],[15611,27,1],
[15611,28,1],[15611,29,3],[15611,30,1],[15611,33,9],[15611,34,4],
[15611,36,1],[15611,39,18],[15611,42,12],[15611,43,1],[15611,47,1],
[15611,48,1],[15611,52,1],[15611,53,1],[15611,55,2],[15611,59,7],
[15611,63,1],[15611,68,1],[15611,72,1],[15611,74,1],[15612,28,7],
[15613,5,4],[15613,26,1],[15613,38,10],[15613,42,6],[15613,56,1],
[15614,23,1],[15614,24,1],[15614,28,1],[15614,33,1],[15614,37,1],
[15615,2,2],[15615,33,1],[15616,2,1],[15616,5,1],[15616,22,1],
[15616,23,1],[15616,24,2],[15616,27,4],[15616,31,1],[15616,32,3],
[15616,35,1],[15616,43,1],[15616,62,2],[15617,27,2],[15618,1,103],
[15618,2,6],[15618,5,4],[15618,7,11],[15618,8,35],[15618,9,2],
[15618,10,1],[15618,11,1],[15618,12,88],[15618,13,13],[15618,14,1],
[15618,15,1],[15618,16,1],[15618,19,1],[15618,20,3],[15618,21,45],
[15618,22,1],[15618,23,106],[15618,24,26],[15618,25,195],
[15618,26,109],[15618,27,5],[15618,28,13],[15618,29,11],[15618,31,65],
[15618,33,130],[15618,34,6],[15618,35,8],[15618,36,1],[15618,37,3],
[15618,38,2],[15618,39,10],[15618,40,2],[15618,42,79],[15618,43,143],
[15618,44,4],[15618,46,2],[15618,47,2],[15618,48,8],[15618,51,1],
[15618,52,10],[15618,53,1],[15618,54,1],[15618,55,2],[15618,56,1],
[15618,57,2],[15618,59,1],[15618,62,2],[15618,63,1],[15618,65,1],
[15618,66,4],[15618,67,1],[15618,68,2],[15618,69,1],[15618,70,6],
[15618,72,2],[15618,73,1],[15618,74,1],[15618,77,1],[15618,78,2],
[15619,28,4],[15619,35,1],[15620,26,1],[15620,28,2],[15621,1,1],
[15621,9,2],[15621,10,4],[15621,11,1],[15621,19,3],[15621,45,1],

[15621,46,1],[15621,57,1],[15621,65,4],[15621,67,2],[15621,71,2],
[15621,77,1],[15622,0,1],[15622,9,1],[15622,65,1],[15623,21,2],
[15623,23,2],[15623,24,4],[15623,26,5],[15623,28,1],[15623,33,1],
[15623,43,9],[15623,47,1],[15623,68,1],[15624,1,1],[15624,13,2],
[15624,20,2],[15624,21,1],[15624,22,3],[15624,23,1],[15624,24,2],
[15624,27,4],[15624,28,4],[15624,31,2],[15624,32,1],[15624,34,1],
[15624,35,3],[15624,39,1],[15624,42,1],[15624,43,1],[15625,28,1],
[15625,35,5],[15626,15,1],[15626,55,3],[15627,22,2],[15627,27,2],
[15627,28,13],[15628,26,2],[15629,5,2],[15629,74,1],[15630,20,1],
[15630,35,1],[15630,52,1],[15631,47,2],[15632,15,2],[15633,33,1],
[15633,66,1],[15634,1,1],[15634,21,1],[15635,19,1],[15635,20,1],
[15636,39,2],[15637,1,1],[15637,2,38],[15637,3,13],[15637,5,4],
[15637,8,4],[15637,9,1],[15637,10,3],[15637,12,1],[15637,16,2],
[15637,19,1],[15637,20,15],[15637,22,2],[15637,23,5],[15637,24,6],
[15637,25,2],[15637,26,2],[15637,27,4],[15637,28,3],[15637,30,4],
[15637,31,5],[15637,32,3],[15637,33,1],[15637,34,2],[15637,35,1],
[15637,39,6],[15637,41,1],[15637,42,3],[15637,43,2],[15637,47,3],
[15637,48,2],[15637,52,1],[15637,56,1],[15637,58,1],[15637,59,1],
[15637,60,1],[15637,65,1],[15637,70,1],[15637,71,1],[15637,73,2],
[15638,2,1],[15638,3,1],[15638,6,1],[15638,28,1],[15638,56,1],
[15639,8,2],[15639,59,1],[15640,26,1],[15640,27,1],[15640,28,4],
[15640,33,1],[15640,43,1],[15641,23,1],[15641,32,1],[15641,42,1],
[15642,3,2],[15642,26,1],[15642,27,1],[15642,30,2],[15642,48,2],
[15643,31,2],[15644,4,1],[15644,28,1],[15645,46,1],[15645,64,1],
[15646,22,1],[15646,50,1],[15647,3,1],[15647,6,1],[15647,27,2],
[15648,2,2],[15648,28,1],[15649,3,2],[15649,5,1],[15649,24,2],
[15649,40,1],[15649,48,1],[15649,51,1],[15649,61,1],[15650,0,1],
[15650,1,3],[15650,3,2],[15650,7,6],[15650,11,1],[15650,13,1],
[15650,16,1],[15650,24,3],[15650,27,1],[15650,28,2],[15650,29,11],
[15650,32,5],[15650,41,1],[15650,57,1],[15650,58,1],[15650,62,5],
[15650,65,1],[15650,69,1],[15650,71,1],[15651,5,1],[15651,28,1],
[15651,31,1],[15651,66,1],[15651,71,1],[15652,23,1],[15652,56,1],
[15653,8,1],[15653,17,2],[15653,53,1],[15654,40,2],[15654,63,2],
[15654,70,1],[15654,74,6],[15654,75,5],[15654,76,5],[15655,28,2],
[15656,8,1],[15656,21,1],[15656,22,2],[15656,23,1],[15656,26,1],
[15656,27,1],[15656,32,1],[15656,62,1],[15656,70,2],[15657,27,1],
[15657,33,1],[15657,56,1],[15658,20,1],[15658,32,1],[15658,33,1],
[15658,35,2],[15658,50,1],[15659,27,1],[15659,47,1],[15660,23,7],
[15661,1,1],[15661,13,1],[15661,37,1],[15661,57,1],[15661,73,1],
[15662,67,1],[15662,77,1],[15663,2,1],[15663,3,3],[15663,6,4],
[15663,11,1],[15663,23,5],[15663,24,26],[15663,25,2],[15663,26,3],
[15663,28,3],[15663,32,1],[15663,42,1],[15663,47,1],[15663,56,7],
[15664,24,7],[15664,28,1],[15664,32,8],[15664,35,1],[15664,50,1],
[15664,56,1],[15664,66,1],[15665,15,2],[15665,28,1],[15665,55,1],
[15666,55,2],[15667,3,1],[15667,28,4],[15667,63,1],[15667,74,1],
[15667,76,1],[15668,19,1],[15668,30,1],[15669,20,1],[15669,39,1],
[15670,24,1],[15670,44,1],[15671,10,1],[15671,18,1],[15671,28,1],
[15671,31,1],[15671,35,1],[15671,53,1],[15671,57,1],[15671,64,1],
[15671,73,1],[15672,3,2],[15672,5,1],[15672,22,1],[15672,23,2],
[15672,24,2],[15672,27,2],[15672,32,1],[15672,40,1],[15672,42,1],

[15672,43,1],[15672,56,5],[15672,66,1],[15673,25,1],[15673,56,1],
[15674,28,2],[15674,55,1],[15675,22,2],[15675,23,1],[15675,27,1],
[15675,31,1],[15675,35,1],[15675,44,1],[15675,51,1],[15675,62,1],
[15676,1,1],[15676,54,1],[15676,65,1],[15677,24,1],[15677,27,1],
[15677,28,1],[15677,31,1],[15678,28,1],[15678,44,1],[15678,66,1],
[15679,1,1],[15679,37,1],[15680,28,4],[15681,23,1],[15681,28,1],
[15682,3,1],[15682,9,1],[15683,0,3],[15683,8,1],[15683,9,3],
[15683,10,1],[15683,11,1],[15683,12,1],[15683,14,1],[15683,15,1],
[15683,16,2],[15683,19,2],[15683,45,1],[15683,46,1],[15683,49,2],
[15683,54,1],[15683,57,2],[15683,58,1],[15683,59,1],[15683,65,5],
[15683,67,1],[15683,77,1],[15683,78,1],[15684,25,2],[15684,27,1],
[15684,40,1],[15684,47,1],[15685,3,1],[15685,8,1],[15685,62,1],
[15685,68,1],[15686,6,3],[15686,13,1],[15686,19,1],[15686,23,4],
[15686,24,11],[15686,28,2],[15686,35,1],[15687,4,1],[15687,5,1],
[15687,17,2],[15687,18,1],[15687,22,1],[15687,28,4],[15687,50,1],
[15687,53,2],[15687,70,1],[15688,23,3],[15688,24,2],[15688,26,1],
[15688,28,1],[15688,32,1],[15689,25,1],[15689,52,2],[15690,22,4],
[15690,26,1],[15690,33,1],[15690,35,1],[15690,56,1],[15691,9,1],
[15691,12,1],[15691,29,1],[15691,57,1],[15692,5,5],[15692,15,1],
[15692,20,1],[15692,21,4],[15692,24,1],[15692,27,2],[15692,32,1],
[15692,33,1],[15692,35,1],[15692,42,1],[15692,47,1],[15692,49,1],
[15692,50,1],[15692,52,1],[15692,59,1],[15692,66,2],[15692,68,1],
[15692,70,1],[15692,72,1],[15692,79,1],[15693,7,1],[15693,9,2],
[15693,10,3],[15693,19,2],[15693,41,1],[15693,45,1],[15693,53,1],
[15693,59,1],[15693,60,1],[15693,73,5],[15693,78,2],[15694,59,1],
[15694,71,1],[15695,22,1],[15695,27,1],[15696,2,1],[15696,24,1],
[15696,28,1],[15697,23,1],[15697,33,1],[15697,70,1],[15698,1,1],
[15698,10,1],[15698,23,1],[15698,25,1],[15698,28,1],[15698,38,1],
[15698,61,1],[15698,69,1],[15698,70,2],[15698,75,1],[15699,40,1],
[15699,56,1],[15699,69,1],[15700,19,2],[15700,53,1],[15700,55,1],
[15701,12,1],[15701,15,1],[15701,17,1],[15701,55,1],[15702,5,1],
[15702,6,1],[15702,9,1],[15702,25,1],[15702,31,1],[15702,32,1],
[15702,40,1],[15702,56,2],[15702,60,1],[15702,68,1],[15702,70,1],
[15702,76,1],[15703,8,2],[15704,3,1],[15704,22,1],[15704,39,2],
[15705,15,5],[15706,45,1],[15706,57,1],[15706,71,1],[15707,2,13],
[15707,8,5],[15707,31,2],[15707,34,5],[15707,39,1],[15708,35,1],
[15708,66,1],[15708,72,1],[15709,2,3],[15710,28,2],[15710,63,1],
[15710,74,1],[15710,75,3],[15710,76,2],[15711,27,2],[15712,28,1],
[15712,47,1],[15712,52,1],[15713,28,2],[15713,60,1],[15714,4,2],
[15714,10,1],[15714,17,2],[15714,19,1],[15714,20,1],[15714,23,1],
[15714,25,2],[15714,28,1],[15714,41,2],[15714,45,1],[15714,50,11],
[15714,77,1],[15715,25,4],[15716,10,1],[15716,41,1],[15717,2,7],
[15717,8,2],[15717,13,1],[15717,19,1],[15717,20,1],[15717,22,2],
[15717,24,1],[15717,28,1],[15717,30,5],[15717,33,1],[15717,47,1],
[15717,59,2],[15717,65,1],[15717,78,1],[15718,1,1],[15718,2,1],
[15718,13,1],[15718,28,9],[15718,31,5],[15718,33,2],[15718,43,1],
[15719,2,1],[15719,72,1],[15720,5,2],[15720,24,1],[15720,30,1],
[15720,75,1],[15721,15,2],[15721,55,1],[15722,13,1],[15722,15,1],
[15723,2,1],[15723,4,5],[15723,26,1],[15723,28,3],[15723,33,1],
[15723,38,4],[15723,42,1],[15723,44,42],[15723,50,19],[15723,66,38],

[15723,70,1],[15723,74,1],[15723,76,1],[15724,28,2],[15725,23,1],
[15725,24,3],[15726,2,3],[15726,3,2],[15726,5,1],[15726,24,1],
[15726,28,1],[15726,30,1],[15726,31,2],[15726,45,1],[15727,53,1],
[15727,55,1],[15728,6,1],[15728,69,1],[15728,70,7],[15728,72,1],
[15729,5,2],[15729,23,1],[15729,24,1],[15729,31,1],[15729,35,1],
[15729,62,4],[15730,7,1],[15730,34,1],[15731,0,1],[15731,1,2],
[15731,9,3],[15731,10,2],[15731,11,2],[15731,12,3],[15731,13,2],
[15731,14,2],[15731,15,1],[15731,16,3],[15731,17,1],[15731,19,4],
[15731,29,2],[15731,30,2],[15731,34,1],[15731,35,1],[15731,36,2],
[15731,37,1],[15731,39,1],[15731,41,3],[15731,49,1],[15731,53,2],
[15731,54,1],[15731,57,2],[15731,58,2],[15731,65,2],[15731,67,1],
[15731,71,1],[15731,73,3],[15731,77,3],[15731,78,1],[15732,31,5],
[15733,2,1],[15733,12,1],[15733,23,1],[15733,24,1],[15733,27,7],
[15733,31,1],[15733,32,2],[15733,62,2],[15733,70,1],[15734,23,2],
[15734,24,1],[15734,66,1],[15735,20,1],[15735,27,1],[15736,12,2],
[15737,18,1],[15737,28,9],[15737,38,1],[15737,41,1],[15737,45,1],
[15737,63,1],[15737,65,1],[15737,68,12],[15737,72,1],[15738,2,3],
[15738,8,1],[15739,2,3],[15739,10,1],[15739,24,1],[15739,34,5],
[15739,44,1],[15739,61,1],[15739,65,1],[15740,1,1],[15740,15,17],
[15740,17,4],[15740,20,1],[15740,44,1],[15740,53,3],[15740,55,2],
[15740,58,1],[15740,66,2],[15741,3,1],[15741,22,1],[15741,23,1],
[15741,27,1],[15741,31,1],[15741,35,1],[15741,40,2],[15741,44,5],
[15741,48,1],[15741,50,2],[15741,62,1],[15741,63,1],[15741,66,8],
[15741,69,1],[15741,70,1],[15741,74,2],[15741,76,2],[15742,10,1],
[15742,12,1],[15743,12,1],[15743,25,1],[15743,32,1],[15743,33,8],
[15743,35,10],[15743,76,1],[15744,5,1],[15744,28,1],[15744,42,1],
[15745,4,1],[15745,27,1],[15745,46,2],[15746,5,1],[15746,13,1],
[15746,20,1],[15746,23,1],[15746,68,3],[15746,70,2],[15746,74,1],
[15747,28,2],[15748,5,1],[15748,17,1],[15748,22,2],[15748,24,1],
[15748,27,1],[15748,28,1],[15749,14,1],[15749,20,1],[15749,28,1],
[15750,28,4],[15750,68,1],[15751,20,1],[15751,37,4],[15752,24,1],
[15752,25,1],[15752,34,1],[15753,3,1],[15753,26,1],[15753,33,1],
[15753,35,1],[15753,42,2],[15753,43,1],[15753,56,1],[15754,4,21],
[15754,5,2],[15754,22,1],[15754,23,1],[15754,26,1],[15754,27,2],
[15754,28,3],[15754,31,1],[15754,32,4],[15754,35,1],[15754,38,14],
[15754,44,62],[15754,47,2],[15754,50,21],[15754,63,2],[15754,66,65],
[15754,74,2],[15755,20,3],[15755,42,2],[15756,21,5],[15757,24,2],
[15757,26,2],[15757,27,3],[15758,2,1],[15758,47,15],[15758,74,3],
[15759,28,1],[15759,50,1],[15760,2,8],[15760,42,1],[15761,2,1],
[15761,28,4],[15761,44,2],[15761,50,1],[15761,55,1],[15761,66,1],
[15762,42,2],[15763,4,1],[15763,17,1],[15763,27,1],[15763,44,1],
[15763,50,4],[15763,55,1],[15763,66,1],[15764,1,5],[15764,7,4],
[15764,8,1],[15764,9,12],[15764,10,21],[15764,11,8],[15764,12,4],
[15764,13,5],[15764,14,5],[15764,15,3],[15764,16,16],[15764,17,2],
[15764,18,2],[15764,19,14],[15764,20,4],[15764,28,3],[15764,30,1],
[15764,34,1],[15764,36,5],[15764,37,7],[15764,39,2],[15764,41,15],
[15764,45,8],[15764,46,6],[15764,49,1],[15764,53,2],[15764,54,6],
[15764,55,7],[15764,57,6],[15764,58,3],[15764,59,5],[15764,60,4],
[15764,64,1],[15764,65,11],[15764,67,8],[15764,70,1],[15764,71,8],
[15764,73,6],[15764,74,1],[15764,77,11],[15764,78,1],[15764,79,13],

[15765,23,1],[15765,43,1],[15766,3,1],[15766,22,1],[15766,26,2],
[15766,35,2],[15766,42,1],[15766,51,1],[15767,40,1],[15767,62,1],
[15768,3,1],[15768,5,2],[15768,24,3],[15768,26,1],[15768,31,1],
[15768,43,2],[15769,1,1],[15769,2,6],[15769,3,8],[15769,4,1],
[15769,5,1],[15769,6,1],[15769,8,1],[15769,9,1],[15769,10,1],
[15769,11,1],[15769,13,5],[15769,14,1],[15769,15,1],[15769,16,1],
[15769,18,1],[15769,19,1],[15769,20,4],[15769,21,1],[15769,22,1],
[15769,23,3],[15769,25,2],[15769,26,3],[15769,27,12],[15769,28,4],
[15769,30,5],[15769,31,2],[15769,32,5],[15769,34,1],[15769,35,7],
[15769,37,1],[15769,39,2],[15769,40,1],[15769,42,12],[15769,43,2],
[15769,45,1],[15769,47,3],[15769,59,1],[15769,61,1],[15769,62,4],
[15769,63,1],[15769,65,3],[15769,67,1],[15769,70,1],[15770,11,2],
[15770,71,2],[15771,1,3],[15771,2,4],[15771,3,2],[15771,5,1],
[15771,9,3],[15771,14,1],[15771,15,1],[15771,19,1],[15771,26,1],
[15771,27,1],[15771,30,3],[15771,31,4],[15771,32,1],[15771,33,1],
[15771,35,1],[15771,39,1],[15771,47,2],[15771,59,1],[15771,60,1],
[15771,66,1],[15771,75,1],[15772,2,2],[15772,3,2],[15772,15,1],
[15772,27,1],[15772,55,1],[15773,28,6],[15774,4,1],[15774,22,5],
[15774,23,1],[15774,26,2],[15774,38,1],[15774,43,27],[15774,68,1],
[15774,79,1],[15775,23,1],[15775,24,3],[15775,28,1],[15775,31,1],
[15775,32,2],[15775,62,1],[15776,2,8],[15776,28,1],[15776,31,2],
[15777,27,1],[15777,34,1],[15778,7,1],[15778,10,1],[15779,55,2],
[15780,20,1],[15780,27,1],[15781,32,2],[15782,28,1],[15782,63,1],
[15783,2,1],[15783,64,1],[15784,1,1],[15784,19,2],[15784,22,2],
[15784,24,2],[15784,40,1],[15784,69,2],[15785,27,1],[15785,28,1],
[15786,8,3],[15786,35,1],[15787,20,3],[15787,31,1],[15788,22,1],
[15788,24,1],[15788,26,1],[15788,27,1],[15788,28,11],[15788,32,1],
[15788,33,1],[15788,43,1],[15788,44,1],[15788,50,1],[15788,69,1],
[15788,74,1],[15788,75,1],[15788,76,2],[15789,48,2],[15790,3,2],
[15790,4,1],[15790,5,1],[15790,10,1],[15790,24,1],[15790,27,3],
[15790,32,1],[15790,38,2],[15790,40,1],[15790,43,1],[15790,63,34],
[15790,66,1],[15790,69,2],[15790,70,2],[15790,71,1],[15790,72,1],
[15790,74,21],[15790,75,28],[15790,76,38],[15791,15,1],[15791,55,2],
[15792,17,1],[15792,53,1],[15793,2,13],[15793,3,3],[15793,5,4],
[15793,8,3],[15793,9,1],[15793,10,1],[15793,11,2],[15793,12,1],
[15793,17,1],[15793,19,1],[15793,20,11],[15793,21,1],[15793,22,1],
[15793,23,2],[15793,24,2],[15793,25,1],[15793,26,4],[15793,27,3],
[15793,28,1],[15793,30,1],[15793,31,7],[15793,32,1],[15793,34,4],
[15793,35,3],[15793,37,1],[15793,38,1],[15793,41,1],[15793,42,1],
[15793,43,4],[15793,46,2],[15793,47,3],[15793,51,1],[15793,53,1],
[15793,54,2],[15793,56,3],[15793,59,2],[15793,62,1],[15793,68,1],
[15793,69,1],[15793,73,2],[15793,76,1],[15793,77,1],[15794,66,3],
[15795,9,1],[15795,20,1],[15795,53,1],[15796,20,1],[15796,24,1],
[15796,28,2],[15796,78,1],[15797,24,2],[15797,27,1],[15797,32,1],
[15797,35,1],[15797,56,4],[15798,11,1],[15798,20,1],[15799,2,4],
[15799,3,3],[15799,6,1],[15799,8,2],[15799,13,2],[15799,16,1],
[15799,19,1],[15799,22,1],[15799,23,3],[15799,24,2],[15799,26,1],
[15799,27,3],[15799,30,3],[15799,31,1],[15799,37,2],[15799,42,3],
[15799,43,1],[15799,47,2],[15799,53,1],[15799,59,1],[15799,60,1],
[15799,69,1],[15799,73,1],[15800,3,1],[15800,50,1],[15801,0,2],

[15801,1,12],[15801,2,20],[15801,3,2],[15801,5,2],[15801,9,1],
[15801,12,4],[15801,13,7],[15801,15,1],[15801,17,2],[15801,20,6],
[15801,23,1],[15801,24,4],[15801,27,2],[15801,30,4],[15801,33,2],
[15801,39,3],[15801,42,4],[15801,43,3],[15801,51,1],[15801,53,1],
[15801,56,1],[15801,62,1],[15801,66,1],[15801,68,1],[15801,74,2],
[15801,77,1],[15802,18,1],[15802,32,4],[15802,56,1],[15803,6,1],
[15803,21,2],[15803,27,4],[15803,33,2],[15803,35,1],[15803,61,1],
[15803,74,2],[15804,11,1],[15804,19,1],[15805,28,1],[15805,69,1],
[15806,23,1],[15806,31,2],[15806,39,1],[15807,48,5],[15808,1,1],
[15808,2,2],[15808,3,4],[15808,4,1],[15808,22,1],[15808,23,2],
[15808,24,3],[15808,26,2],[15808,27,2],[15808,31,5],[15808,32,1],
[15808,35,1],[15808,43,1],[15808,50,1],[15808,53,2],[15808,64,2],
[15808,68,1],[15808,74,1],[15809,17,1],[15809,53,1],[15810,13,1],
[15810,53,1],[15811,35,1],[15811,50,1],[15812,5,1],[15812,9,1],
[15812,28,2],[15812,33,1],[15812,42,1],[15812,52,1],[15812,55,1],
[15812,61,1],[15813,9,1],[15813,28,4],[15814,2,4],[15815,2,4],
[15816,26,11],[15816,31,2],[15816,33,1],[15816,43,5],[15816,56,1],
[15817,44,2],[15817,66,1],[15818,25,1],[15818,26,1],[15818,27,3],
[15818,33,2],[15818,62,2],[15818,74,1],[15819,1,2],[15819,9,1],
[15819,10,1],[15819,11,1],[15819,12,3],[15819,13,1],[15819,14,1],
[15819,16,2],[15819,17,2],[15819,19,1],[15819,28,1],[15819,30,3],
[15819,36,3],[15819,37,2],[15819,39,3],[15819,41,2],[15819,53,4],
[15819,55,2],[15819,57,1],[15819,58,2],[15819,59,1],[15819,65,2],
[15819,67,2],[15819,76,3],[15820,26,1],[15820,51,1],[15820,52,1],
[15821,13,1],[15821,22,1],[15821,27,1],[15821,28,6],[15821,30,2],
[15821,32,15],[15821,33,10],[15821,35,9],[15821,38,2],[15821,48,1],
[15821,50,1],[15821,52,29],[15822,25,4],[15822,28,1],[15823,6,1],
[15823,21,1],[15823,24,1],[15823,30,1],[15824,23,1],[15824,32,6],
[15824,37,2],[15824,56,2],[15825,21,1],[15825,42,1],[15826,0,2],
[15826,1,21],[15826,2,8],[15826,3,12],[15826,4,174],[15826,5,27],
[15826,7,2],[15826,8,5],[15826,9,16],[15826,10,13],[15826,11,1],
[15826,12,11],[15826,13,9],[15826,14,5],[15826,15,82],[15826,16,6],
[15826,17,75],[15826,18,3],[15826,19,6],[15826,20,17],[15826,21,1],
[15826,22,10],[15826,23,18],[15826,24,3],[15826,25,7],[15826,26,5],
[15826,27,6],[15826,28,30],[15826,29,3],[15826,31,6],[15826,32,10],
[15826,33,2],[15826,34,6],[15826,35,10],[15826,36,2],[15826,37,4],
[15826,38,25],[15826,39,8],[15826,40,4],[15826,41,2],[15826,42,9],
[15826,43,3],[15826,44,78],[15826,45,7],[15826,46,5],[15826,47,1],
[15826,48,3],[15826,49,3],[15826,50,165],[15826,51,1],[15826,52,1],
[15826,53,73],[15826,54,3],[15826,55,73],[15826,56,4],[15826,57,5],
[15826,58,2],[15826,59,6],[15826,61,2],[15826,62,6],[15826,63,6],
[15826,64,3],[15826,65,5],[15826,66,79],[15826,67,3],[15826,68,3],
[15826,69,4],[15826,70,15],[15826,71,3],[15826,72,6],[15826,74,2],
[15826,75,1],[15826,76,3],[15826,77,11],[15826,78,1],[15827,38,1],
[15827,44,9],[15827,50,2],[15827,63,1],[15827,66,3],[15828,15,2],
[15828,20,2],[15828,21,1],[15828,41,1],[15828,55,1],[15828,59,1],
[15828,65,1],[15828,78,1],[15829,28,1],[15829,32,1],[15829,50,1],
[15830,3,1],[15830,28,1],[15831,15,3],[15831,55,1],[15832,27,3],
[15832,40,1],[15833,0,23473],[15833,1,272],[15833,2,1],[15833,4,1],
[15833,7,3440],[15833,8,266],[15833,9,968],[15833,10,990],

[15833,11,473],[15833,12,676],[15833,13,165],[15833,14,240],
[15833,15,77],[15833,16,830],[15833,17,144],[15833,18,458],
[15833,19,876],[15833,20,77],[15833,24,1],[15833,25,4],[15833,26,1],
[15833,28,6],[15833,29,84],[15833,30,435],[15833,31,36],[15833,32,1],
[15833,34,275],[15833,35,3],[15833,36,327],[15833,37,356],
[15833,39,127],[15833,41,480],[15833,44,1],[15833,45,856],
[15833,46,378],[15833,49,186],[15833,50,1],[15833,52,2],
[15833,53,154],[15833,54,270],[15833,55,110],[15833,57,674],
[15833,58,317],[15833,59,367],[15833,60,86],[15833,62,29],
[15833,63,1],[15833,64,86],[15833,65,934],[15833,67,351],[15833,68,1],
[15833,69,2],[15833,70,21],[15833,71,224],[15833,72,55],
[15833,73,979],[15833,74,1],[15833,77,494],[15833,78,72],[15834,35,1],
[15834,56,1],[15835,74,1],[15835,76,1],[15836,2,3],[15837,0,8],
[15837,1,958],[15837,2,1],[15837,4,1],[15837,5,2],[15837,6,1],
[15837,7,100],[15837,8,39],[15837,9,5],[15837,10,9],[15837,12,162],
[15837,13,551],[15837,14,2],[15837,15,13],[15837,16,5],[15837,17,34],
[15837,18,1],[15837,19,1],[15837,20,19],[15837,21,9],[15837,23,4],
[15837,24,3],[15837,25,5],[15837,26,4],[15837,27,4],[15837,28,7],
[15837,29,117],[15837,30,17],[15837,31,5],[15837,32,226],
[15837,33,213],[15837,34,42],[15837,35,1],[15837,36,18],[15837,37,19],
[15837,39,406],[15837,42,1],[15837,43,13],[15837,44,1],[15837,45,4],
[15837,47,2],[15837,48,3],[15837,50,3],[15837,51,1],[15837,52,1],
[15837,53,43],[15837,54,1],[15837,55,20],[15837,57,3],[15837,58,1],
[15837,59,11],[15837,60,3],[15837,62,2],[15837,64,1],[15837,65,2],
[15837,66,1],[15837,67,2],[15837,70,1],[15837,71,3],[15837,73,11],
[15837,74,1],[15837,76,2],[15837,77,5],[15837,78,3],[15838,23,2],
[15838,47,13],[15839,1,3],[15839,29,1],[15840,1,2],[15840,8,3],
[15840,16,1],[15840,22,2],[15840,23,1],[15840,27,2],[15840,28,1],
[15840,30,2],[15840,32,1],[15840,37,1],[15840,39,4],[15840,43,1],
[15840,56,1],[15841,2,1],[15841,22,1],[15842,27,4],[15843,9,1],
[15843,71,1],[15844,33,3],[15844,35,1],[15844,52,3],[15844,70,1],
[15845,3,1],[15845,22,2],[15845,28,19],[15845,35,1],[15845,74,1],
[15846,25,1],[15846,35,1],[15847,2,2],[15847,26,1],[15847,32,3],
[15847,43,1],[15848,0,1],[15848,7,1],[15848,10,1],[15848,14,1],
[15848,16,1],[15848,20,11],[15848,21,4],[15848,22,19],[15848,23,4],
[15848,24,1],[15848,25,2],[15848,27,7],[15848,28,3],[15848,33,2],
[15848,34,1],[15848,35,2],[15848,36,1],[15848,38,5],[15848,42,1],
[15848,43,1],[15848,45,4],[15848,65,2],[15848,73,1],[15848,78,1],
[15849,0,1],[15849,1,6],[15849,2,15],[15849,3,14],[15849,5,6],
[15849,6,2],[15849,8,3],[15849,9,6],[15849,10,1],[15849,12,1],
[15849,13,20],[15849,14,1],[15849,15,1],[15849,16,2],[15849,17,3],
[15849,18,1],[15849,19,3],[15849,20,12],[15849,21,2],[15849,22,1],
[15849,23,19],[15849,24,8],[15849,25,3],[15849,26,1],[15849,27,5],
[15849,28,6],[15849,30,8],[15849,31,4],[15849,32,6],[15849,33,2],
[15849,34,3],[15849,35,9],[15849,36,1],[15849,37,2],[15849,38,1],
[15849,39,2],[15849,41,1],[15849,42,6],[15849,43,7],[15849,44,1],
[15849,45,2],[15849,47,7],[15849,49,1],[15849,50,1],[15849,52,1],
[15849,53,1],[15849,55,1],[15849,57,3],[15849,59,4],[15849,60,2],
[15849,62,1],[15849,64,1],[15849,65,2],[15849,67,1],[15849,77,3],
[15850,35,25],[15850,56,1],[15851,0,42],[15851,8,1],[15851,17,1],

[15851,19,1],[15851,22,1],[15852,1,2],[15852,10,1],[15852,26,1],
[15852,28,1],[15853,3,1],[15853,23,1],[15853,24,3],[15853,27,2],
[15853,28,1],[15853,31,1],[15853,44,1],[15853,47,1],[15854,3,2],
[15854,28,1],[15854,35,1],[15854,47,2],[15854,51,1],[15855,26,1],
[15855,74,1],[15856,1,2],[15856,2,1],[15856,3,1],[15856,5,4],
[15856,10,2],[15856,12,1],[15856,13,1],[15856,15,13],[15856,20,1],
[15856,21,1],[15856,22,1],[15856,23,1],[15856,25,1],[15856,27,1],
[15856,31,1],[15856,35,1],[15856,44,1],[15856,47,1],[15856,55,16],
[15856,59,1],[15857,9,2],[15858,14,1],[15858,20,2],[15858,22,1],
[15858,27,1],[15858,62,1],[15858,74,2],[15859,27,2],[15859,35,1],
[15859,43,1],[15859,48,2],[15860,9,2],[15860,13,1],[15860,14,1],
[15860,16,2],[15860,19,1],[15860,28,2],[15860,45,1],[15860,54,1],
[15860,57,1],[15860,58,1],[15860,59,4],[15860,64,1],[15860,65,1],
[15860,67,1],[15860,77,1],[15861,9,1],[15861,13,1],[15862,20,1],
[15862,24,1],[15862,31,2],[15863,1,127],[15863,3,1],[15863,9,3],
[15863,10,2],[15863,12,4],[15863,13,4],[15863,14,1],[15863,16,1],
[15863,17,1],[15863,20,1],[15863,29,3],[15863,34,4],[15863,36,2],
[15863,39,5],[15863,53,1],[15863,55,2],[15864,23,1],[15864,42,1],
[15865,5,1],[15865,26,1],[15865,43,1],[15866,23,5],[15866,24,1],
[15866,25,1],[15866,26,3],[15866,28,1],[15866,32,2],[15866,42,1],
[15867,21,1],[15867,35,1],[15868,15,1],[15868,17,1],[15869,6,1],
[15869,28,1],[15870,24,1],[15870,31,1],[15871,5,1],[15871,74,1],
[15872,15,2],[15873,9,1],[15873,10,3],[15873,17,1],[15873,18,1],
[15873,19,1],[15873,22,1],[15873,28,2],[15873,34,1],[15873,37,1],
[15873,39,1],[15873,47,1],[15873,48,1],[15873,55,1],[15873,58,1],
[15873,67,2],[15873,68,2],[15873,70,1],[15873,73,1],[15873,74,1],
[15873,75,1],[15873,77,1],[15874,45,1],[15874,77,1],[15875,8,1],
[15875,16,1],[15875,39,1],[15876,2,1],[15876,22,1],[15877,28,2],
[15878,2,1],[15878,31,1],[15879,1,2],[15879,7,1],[15879,10,2],
[15879,12,2],[15879,14,2],[15879,15,1],[15879,49,2],[15879,57,2],
[15879,58,1],[15879,59,3],[15879,64,1],[15880,6,3],[15880,21,1],
[15880,23,2],[15880,26,2],[15880,27,2],[15880,37,1],[15880,42,1],
[15880,43,1],[15880,48,2],[15881,2,2],[15881,3,1],[15881,20,3],
[15881,23,8],[15881,30,3],[15881,42,2],[15881,47,2],[15881,48,1],
[15882,22,1],[15882,23,1],[15882,24,1],[15882,31,1],[15882,42,2],
[15882,43,1],[15882,47,2],[15883,4,1],[15883,53,1],[15883,55,4],
[15884,56,2],[15885,8,2],[15886,15,2],[15886,23,1],[15886,40,1],
[15886,53,1],[15886,55,1],[15887,34,1],[15887,39,2],[15888,3,1],
[15888,63,3],[15888,66,1],[15888,74,3],[15888,75,2],[15888,76,2],
[15889,32,2],[15889,62,1],[15890,8,7],[15890,9,1],[15890,45,1],
[15891,9,1],[15891,68,1],[15892,63,1],[15892,76,2],[15893,28,1],
[15893,66,2],[15894,31,1],[15894,52,1],[15895,24,2],[15895,31,1],
[15896,7,1],[15896,9,1],[15896,11,2],[15896,12,1],[15896,15,3],
[15896,16,1],[15896,29,1],[15896,46,1],[15896,55,1],[15896,57,1],
[15896,64,1],[15896,65,1],[15896,67,1],[15896,71,1],[15897,2,2],
[15897,27,2],[15897,35,2],[15898,55,2],[15899,15,1],[15899,44,1],
[15900,20,1],[15900,39,2],[15900,46,1],[15901,5,1],[15901,66,1],
[15902,3,1],[15902,22,1],[15902,28,1],[15903,61,2],[15904,1,1],
[15904,5,1],[15904,6,3],[15904,24,4],[15904,26,2],[15904,27,1],
[15904,31,1],[15904,33,1],[15904,56,3],[15904,68,3],[15904,70,3],

[15905,2,1],[15905,28,1],[15905,31,4],[15906,22,2],[15906,59,1],
[15906,62,1],[15907,26,1],[15907,31,1],[15908,44,2],[15908,53,2],
[15908,66,1],[15909,18,1],[15909,73,1],[15910,3,1],[15910,23,1],
[15910,26,1],[15910,27,1],[15910,33,2],[15911,2,1],[15911,20,1],
[15911,26,2],[15911,31,2],[15911,35,1],[15911,43,1],[15911,55,1],
[15912,15,2],[15912,17,1],[15912,55,2],[15913,35,4],[15914,2,10],
[15915,20,1],[15915,23,1],[15916,0,1],[15916,30,3],[15916,71,1],
[15917,2,2],[15918,3,1],[15918,20,1],[15918,27,2],[15918,32,1],
[15918,34,1],[15918,40,1],[15918,47,1],[15918,56,3],[15918,74,2],
[15918,77,1],[15919,5,1],[15919,28,1],[15920,23,4],[15920,34,1],
[15921,1,1],[15921,2,6],[15921,17,1],[15921,20,1],[15922,3,1],
[15922,4,23],[15922,5,2],[15922,6,1],[15922,10,1],[15922,15,11],
[15922,22,2],[15922,23,4],[15922,24,4],[15922,25,1],[15922,26,6],
[15922,27,6],[15922,28,100],[15922,31,2],[15922,33,3],[15922,35,1],
[15922,38,5],[15922,40,2],[15922,42,2],[15922,43,4],[15922,44,46],
[15922,47,5],[15922,50,27],[15922,52,1],[15922,53,3],[15922,55,15],
[15922,56,2],[15922,61,1],[15922,62,3],[15922,63,2],[15922,66,51],
[15922,68,2],[15922,69,2],[15922,70,1],[15922,72,2],[15922,74,1],
[15922,75,3],[15922,79,1],[15923,30,2],[15923,34,1],[15923,53,1],
[15924,26,2],[15924,28,1],[15924,35,1],[15925,21,1],[15925,39,1],
[15926,23,1],[15926,26,1],[15926,27,1],[15926,43,1],[15927,31,8],
[15928,2,1],[15928,3,1],[15928,4,1],[15928,5,1],[15928,15,1],
[15928,17,2],[15928,24,1],[15928,27,1],[15928,32,1],[15928,35,2],
[15928,40,3],[15928,42,1],[15928,43,1],[15928,53,3],[15928,55,2],
[15928,58,1],[15928,60,1],[15928,63,1],[15928,66,1],[15928,68,2],
[15928,69,1],[15928,72,2],[15928,74,1],[15928,75,2],[15928,76,1],
[15929,2,1],[15929,15,1],[15929,30,3],[15929,47,1],[15930,3,2],
[15930,6,1],[15930,22,1],[15930,27,1],[15930,56,1],[15931,20,2],
[15931,22,1],[15931,24,2],[15931,26,1],[15931,35,2],[15931,40,1],
[15931,43,1],[15931,45,2],[15931,46,1],[15931,77,1],[15932,4,1],
[15932,44,2],[15932,50,3],[15933,9,1],[15933,27,1],[15934,32,2],
[15934,69,1],[15934,72,1],[15935,15,1],[15935,28,83],[15936,1,1],
[15936,14,1],[15936,28,1],[15936,32,1],[15936,33,9],[15936,35,1],
[15936,52,3],[15937,32,1],[15937,43,1],[15938,35,2],[15939,2,7],
[15939,5,1],[15939,22,2],[15939,23,1],[15939,26,1],[15939,32,2],
[15939,35,1],[15939,40,1],[15939,42,1],[15939,47,1],[15940,20,1],
[15940,79,1],[15941,3,2],[15941,6,1],[15941,23,1],[15941,28,1],
[15941,35,2],[15941,38,3],[15941,45,2],[15942,23,1],[15942,26,1],
[15943,2,1],[15943,63,1],[15943,70,1],[15944,8,1],[15944,23,1],
[15945,5,3],[15945,21,2],[15945,47,1],[15946,34,2],[15946,40,1],
[15946,61,1],[15946,70,1],[15947,19,1],[15947,28,1],[15947,35,1],
[15947,50,1],[15948,3,1],[15948,33,1],[15949,12,1],[15949,13,1],
[15949,15,27],[15949,17,7],[15949,20,1],[15949,29,1],[15949,34,1],
[15949,41,1],[15949,46,1],[15949,53,7],[15949,55,19],[15949,57,1],
[15950,28,3],[15951,3,1],[15951,26,1],[15951,28,1],[15951,31,1],
[15951,37,4],[15952,4,1],[15952,10,1],[15952,14,1],[15952,15,1],
[15952,17,20],[15952,19,1],[15952,20,2],[15952,23,2],[15952,25,1],
[15952,26,1],[15952,27,2],[15952,28,6],[15952,31,1],[15952,33,1],
[15952,35,1],[15952,36,1],[15952,38,1],[15952,40,3],[15952,42,1],
[15952,45,3],[15952,46,1],[15952,47,1],[15952,50,1],[15952,52,1],

[15952,53,24],[15952,55,1],[15952,63,3],[15952,66,1],[15952,67,1],
[15952,70,2],[15952,72,3],[15952,74,6],[15952,75,1],[15952,76,3],
[15952,78,1],[15953,18,1],[15953,46,1],[15954,4,2],[15954,5,3],
[15954,9,1],[15954,11,1],[15954,19,1],[15954,23,1],[15954,26,1],
[15954,28,1],[15954,35,1],[15954,49,1],[15954,50,2],[15954,63,10],
[15954,65,2],[15954,74,10],[15954,75,3],[15954,76,21],[15955,2,2],
[15955,22,1],[15955,27,1],[15955,32,1],[15955,35,1],[15956,1,75],
[15956,2,5],[15956,3,1],[15956,4,2],[15956,5,12],[15956,6,2],
[15956,8,1],[15956,12,1],[15956,13,45],[15956,15,3],[15956,16,6],
[15956,17,3],[15956,20,3],[15956,21,6],[15956,22,2],[15956,23,10],
[15956,24,2],[15956,25,9],[15956,26,15],[15956,27,1],[15956,28,1],
[15956,29,6],[15956,30,1],[15956,31,28],[15956,33,22],[15956,34,2],
[15956,35,2],[15956,36,2],[15956,37,1],[15956,39,3],[15956,40,2],
[15956,42,5],[15956,43,91],[15956,47,8],[15956,50,3],[15956,51,3],
[15956,52,1],[15956,53,3],[15956,55,3],[15956,59,3],[15956,65,1],
[15956,68,1],[15956,73,1],[15956,76,3],[15956,77,2],[15957,2,1],
[15957,31,1],[15958,5,1],[15958,21,1],[15958,22,1],[15958,26,2],
[15958,28,10],[15958,31,1],[15958,32,4],[15958,35,1],[15958,43,4],
[15959,3,2],[15959,47,1],[15960,28,1],[15960,35,1],[15960,70,1],
[15961,1,10],[15961,13,4],[15961,26,5],[15961,29,2],[15961,40,1],
[15961,64,1],[15961,65,1],[15962,5,1],[15962,26,1],[15962,28,76],
[15962,48,1],[15962,70,1],[15962,74,1],[15962,75,1],[15963,3,1],
[15963,24,1],[15963,70,1],[15964,22,1],[15964,27,1],[15964,35,1],
[15964,68,1],[15965,42,5],[15965,43,1],[15966,9,1],[15966,10,1],
[15966,20,4],[15966,26,1],[15966,28,1],[15966,35,1],[15966,42,1],
[15966,49,1],[15966,58,1],[15967,46,1],[15967,68,1],[15968,2,2],
[15969,17,1],[15969,21,1],[15969,28,1],[15969,35,1],[15969,75,1],
[15970,21,1],[15970,23,4],[15970,42,2],[15970,43,2],[15971,10,2],
[15971,11,1],[15971,12,1],[15971,13,1],[15971,16,2],[15971,18,1],
[15971,21,1],[15971,27,1],[15971,31,1],[15971,34,1],[15971,35,1],
[15971,39,1],[15971,43,1],[15971,45,2],[15971,54,2],[15971,63,1],
[15971,74,6],[15971,76,3],[15972,3,2],[15972,10,1],[15972,20,1],
[15972,25,1],[15972,27,2],[15972,70,1],[15973,21,1],[15973,24,1],
[15974,23,2],[15974,24,1],[15974,32,4],[15974,42,1],[15975,25,2],
[15976,2,1],[15976,23,3],[15977,24,1],[15977,26,1],[15978,24,1],
[15978,38,1],[15979,4,1],[15979,28,1],[15980,31,1],[15980,43,1],
[15980,59,2],[15981,26,1],[15981,35,1],[15982,32,4],[15983,12,1],
[15983,13,1],[15983,45,3],[15983,59,2],[15983,78,1],[15984,25,1],
[15984,28,2],[15984,38,1],[15985,2,2],[15985,3,3],[15985,4,1],
[15985,5,2],[15985,11,1],[15985,13,2],[15985,17,1],[15985,21,7],
[15985,23,1],[15985,25,1],[15985,26,2],[15985,30,1],[15985,31,4],
[15985,42,12],[15985,43,4],[15985,44,1],[15985,48,1],[15985,61,1],
[15985,69,1],[15986,2,1],[15986,30,1],[15986,34,4],[15986,39,4],
[15986,62,2],[15987,15,1],[15987,28,1],[15987,66,1],[15988,6,1],
[15988,13,1],[15988,23,3],[15988,24,1],[15988,28,1],[15988,37,1],
[15988,52,1],[15988,59,3],[15988,62,1],[15988,75,1],[15989,28,2],
[15989,31,3],[15990,22,2],[15990,28,1],[15991,21,1],[15991,42,1],
[15992,4,1],[15992,15,4],[15992,17,4],[15992,53,7],[15992,55,4],
[15993,2,3],[15993,47,1],[15994,25,1],[15994,26,1],[15994,28,1],
[15994,43,1],[15994,66,1],[15995,31,1],[15995,74,2],[15996,35,21],

[15996,78,3],[15997,3,1],[15997,5,1],[15997,6,1],[15997,9,1],
[15997,25,1],[15997,28,3],[15997,33,1],[15997,35,5],[15997,50,1],
[15997,68,1],[15998,32,3],[15999,24,1],[15999,28,1],[16000,27,3],
[16000,70,1],[16001,33,8],[16002,23,2],[16002,24,2],[16002,26,1],
[16002,27,1],[16002,56,1],[16003,5,4],[16003,17,1],[16003,38,1],
[16003,66,1],[16004,26,3],[16005,40,1],[16005,73,1],[16006,10,1],
[16006,20,1],[16007,15,1],[16007,17,1],[16007,53,1],[16008,2,1],
[16008,3,1],[16008,4,4],[16008,5,89],[16008,6,2],[16008,23,7],
[16008,24,2],[16008,25,4],[16008,26,1],[16008,27,5],[16008,28,12],
[16008,30,1],[16008,31,1],[16008,32,1],[16008,33,169],[16008,35,2],
[16008,38,1],[16008,42,2],[16008,43,1],[16008,44,2],[16008,47,1],
[16008,48,1],[16008,50,1],[16008,52,1],[16008,63,1],[16008,66,2],
[16008,70,2],[16008,74,2],[16008,75,2],[16008,76,3],[16009,2,1],
[16009,4,1],[16009,5,3],[16009,6,2],[16009,11,1],[16009,13,4],
[16009,15,1],[16009,16,1],[16009,19,1],[16009,20,1],[16009,21,3],
[16009,22,4],[16009,24,3],[16009,25,1],[16009,26,1],[16009,27,3],
[16009,29,1],[16009,31,2],[16009,33,9],[16009,38,2],[16009,42,1],
[16009,43,1],[16009,44,1],[16009,50,2],[16009,56,1],[16009,66,3],
[16009,70,1],[16009,74,2],[16009,77,2],[16010,3,2],[16011,8,1],
[16011,64,1],[16012,2,1],[16012,24,1],[16012,35,1],[16012,56,1],
[16013,27,7],[16013,35,1],[16014,1,2],[16014,9,1],[16014,20,2],
[16014,23,3],[16014,25,2],[16014,26,1],[16014,27,1],[16014,28,1],
[16014,31,1],[16014,34,3],[16014,35,1],[16014,39,2],[16014,41,1],
[16014,42,2],[16014,43,1],[16014,66,1],[16014,77,1],[16015,38,1],
[16015,76,1],[16016,21,1],[16016,22,1],[16016,26,1],[16017,5,1],
[16017,22,2],[16018,3,1],[16018,31,1],[16018,32,1],[16019,2,1],
[16019,25,1],[16019,28,1],[16019,56,2],[16019,61,1],[16019,78,1],
[16020,38,1],[16020,66,1],[16021,37,1],[16021,54,1],[16021,71,1],
[16022,10,1],[16022,15,3],[16022,45,1],[16023,33,2],[16024,1,1],
[16024,28,1],[16025,3,2],[16025,27,2],[16025,32,1],[16025,48,1],
[16026,2,2],[16026,20,2],[16027,4,1],[16027,44,2],[16028,2,1],
[16028,22,1],[16028,25,3],[16028,26,1],[16028,31,1],[16028,32,1],
[16028,43,1],[16028,62,2],[16029,27,2],[16030,9,1],[16030,15,1],
[16030,36,1],[16031,23,1],[16031,43,1],[16032,6,1],[16032,17,2],
[16032,52,1],[16032,74,1],[16033,28,1],[16033,40,1],[16034,28,2],
[16035,20,1],[16035,40,1],[16035,45,1],[16035,59,1],[16036,8,2],
[16036,34,1],[16036,59,1],[16037,24,3],[16037,26,1],[16037,27,1],
[16037,31,1],[16037,32,3],[16037,37,1],[16037,42,3],[16037,48,1],
[16037,50,1],[16037,56,8],[16037,57,1],[16037,61,1],[16037,70,5],
[16037,78,1],[16038,40,1],[16038,70,1],[16039,20,2],[16039,23,1],
[16039,24,1],[16039,27,1],[16039,28,1],[16039,48,1],[16040,28,4],
[16041,2,1],[16041,12,8],[16041,13,1],[16041,16,1],[16041,24,1],
[16041,28,1],[16041,42,1],[16041,62,26],[16041,77,4],[16042,4,1],
[16042,38,1],[16042,44,2],[16042,50,1],[16042,66,1],[16042,70,1],
[16043,2,1],[16043,28,3],[16043,68,1],[16044,5,1],[16044,24,4],
[16044,27,1],[16044,38,1],[16045,2,1],[16045,5,1],[16045,27,1],
[16045,31,1],[16045,32,1],[16045,37,1],[16045,53,1],[16045,60,2],
[16045,78,1],[16046,3,1],[16046,5,1],[16046,27,1],[16046,28,1],
[16047,50,1],[16047,66,1],[16048,24,1],[16048,39,1],[16048,44,1],
[16049,26,2],[16050,35,1],[16050,41,1],[16050,56,1],[16050,68,3],

[16050,69,3],[16050,72,1],[16051,4,1],[16051,5,1],[16051,37,5],
[16051,59,1],[16052,5,2],[16052,8,1],[16052,23,1],[16052,28,1],
[16052,74,2],[16053,3,1],[16053,5,2],[16053,21,4],[16053,22,7],
[16053,23,3],[16053,24,4],[16053,25,1],[16053,26,4],[16053,27,27],
[16053,28,7],[16053,31,2],[16053,32,9],[16053,33,1],[16053,35,4],
[16053,38,1],[16053,43,4],[16053,47,4],[16053,79,1],[16054,17,2],
[16054,50,1],[16054,53,3],[16055,28,1],[16055,32,1],[16055,46,1],
[16056,28,1],[16056,38,1],[16057,2,1],[16057,21,2],[16057,25,1],
[16057,26,2],[16057,31,4],[16057,33,1],[16057,43,2],[16058,4,1],
[16058,21,1],[16058,23,1],[16058,28,5],[16058,74,1],[16059,35,2],
[16060,23,1],[16060,25,1],[16060,27,1],[16060,49,1],[16061,28,48],
[16062,25,1],[16062,27,1],[16062,32,5],[16062,35,1],[16062,52,1],
[16062,62,2],[16063,21,1],[16063,28,20],[16063,34,1],[16064,1,2],
[16064,29,1],[16065,3,1],[16065,28,1],[16066,1,1],[16066,15,1],
[16066,53,1],[16067,33,2],[16068,33,1],[16068,43,1],[16069,0,1],
[16069,1,5],[16069,2,3],[16069,3,6],[16069,4,18],[16069,5,4],
[16069,6,2],[16069,9,2],[16069,10,1],[16069,12,3],[16069,13,1],
[16069,15,25],[16069,16,2],[16069,17,1],[16069,18,1],[16069,20,9],
[16069,21,4],[16069,22,6],[16069,23,5],[16069,24,7],[16069,25,2],
[16069,26,9],[16069,27,6],[16069,28,41],[16069,29,1],[16069,30,1],
[16069,31,7],[16069,32,5],[16069,33,6],[16069,34,4],[16069,35,4],
[16069,36,2],[16069,38,5],[16069,39,3],[16069,40,4],[16069,42,7],
[16069,43,2],[16069,44,30],[16069,45,1],[16069,47,6],[16069,50,20],
[16069,51,1],[16069,53,2],[16069,54,2],[16069,55,10],[16069,57,1],
[16069,58,1],[16069,59,2],[16069,60,1],[16069,61,1],[16069,63,3],
[16069,65,1],[16069,66,35],[16069,67,1],[16069,68,2],[16069,69,2],
[16069,70,5],[16069,72,1],[16069,73,1],[16069,74,2],[16069,75,1],
[16069,76,1],[16069,77,1],[16070,13,2],[16070,39,2],[16071,15,1],
[16071,55,1],[16072,2,1],[16072,23,3],[16073,30,1],[16073,37,2],
[16073,48,2],[16074,0,3],[16074,1,550],[16074,2,148],[16074,3,2],
[16074,4,2],[16074,5,1],[16074,6,2],[16074,7,2],[16074,8,32],
[16074,9,5],[16074,10,4],[16074,11,2],[16074,12,171],[16074,13,471],
[16074,15,7],[16074,16,27],[16074,17,35],[16074,18,1],[16074,19,1],
[16074,20,19],[16074,21,1],[16074,22,1],[16074,23,2],[16074,24,5],
[16074,25,117],[16074,26,24],[16074,27,4],[16074,28,26],[16074,29,46],
[16074,30,6],[16074,31,129],[16074,32,50],[16074,33,483],
[16074,34,29],[16074,35,3],[16074,36,17],[16074,37,2],[16074,38,2],
[16074,39,136],[16074,40,1],[16074,42,5],[16074,43,23],[16074,44,1],
[16074,46,8],[16074,48,2],[16074,51,4],[16074,53,33],[16074,55,9],
[16074,56,1],[16074,57,1],[16074,58,1],[16074,59,5],[16074,60,4],
[16074,63,1],[16074,66,2],[16074,68,3],[16074,70,3],[16074,71,1],
[16074,72,1],[16074,73,11],[16074,74,1],[16074,76,2],[16074,77,1],
[16074,78,1],[16074,79,1],[16075,0,1],[16075,36,1],[16075,65,1],
[16076,25,2],[16077,31,1],[16077,68,1],[16077,75,1],[16077,76,1],
[16078,23,2],[16079,42,4],[16079,61,1],[16080,2,1],[16080,4,1],
[16080,9,1],[16080,15,6],[16080,17,7],[16080,20,2],[16080,28,2],
[16080,36,1],[16080,37,1],[16080,47,1],[16080,50,1],[16080,53,13],
[16080,55,8],[16081,17,1],[16081,65,1],[16082,4,1],[16082,11,1],
[16082,13,1],[16082,33,1],[16082,74,1],[16083,27,2],[16084,27,1],
[16084,28,1],[16084,35,2],[16085,40,1],[16085,50,1],[16085,63,2],

[16086,3,7],[16086,28,1],[16087,28,1],[16087,32,1],[16088,28,2],
[16088,48,1],[16089,4,1],[16089,10,1],[16089,16,1],[16089,19,1],
[16089,20,11],[16089,22,3],[16089,23,72],[16089,24,34],[16089,25,13],
[16089,26,8],[16089,27,173],[16089,28,6],[16089,32,6],[16089,33,2],
[16089,34,1],[16089,35,33],[16089,39,5],[16089,40,3],[16089,43,1],
[16089,48,1],[16089,55,1],[16089,59,6],[16089,60,1],[16089,62,3],
[16089,66,1],[16089,69,1],[16089,70,3],[16089,72,3],[16089,74,2],
[16090,2,1],[16090,3,1],[16090,27,2],[16090,62,7],[16091,5,1],
[16091,24,1],[16092,63,1],[16092,69,1],[16093,5,2],[16093,23,1],
[16093,28,1],[16093,52,2],[16094,23,2],[16094,27,1],[16095,15,1],
[16095,17,4],[16095,24,1],[16095,50,1],[16095,53,2],[16095,55,6],
[16096,23,1],[16096,27,1],[16096,41,1],[16097,22,3],[16097,23,1],
[16097,27,1],[16098,15,2],[16099,31,18],[16099,33,7],[16099,43,1],
[16099,76,3],[16100,27,2],[16101,9,1],[16101,28,1],[16102,28,1],
[16102,33,2],[16103,5,1],[16103,24,1],[16103,28,1],[16104,3,1],
[16104,6,1],[16104,35,1],[16104,56,1],[16105,11,1],[16105,20,1],
[16106,19,1],[16106,20,6],[16106,23,5],[16106,44,1],[16107,20,3],
[16107,22,1],[16108,15,1],[16108,17,1],[16108,53,3],[16109,28,1],
[16109,56,1],[16110,28,2],[16111,32,5],[16112,4,1],[16112,25,1],
[16112,28,1],[16112,75,1],[16113,43,2],[16114,25,1],[16114,28,1],
[16114,32,2],[16115,1,2],[16116,4,3],[16116,9,11],[16116,12,1],
[16116,15,3],[16116,17,21],[16116,19,1],[16116,28,48],[16116,42,1],
[16116,50,10],[16116,53,14],[16116,54,1],[16116,55,16],[16116,66,2],
[16116,78,1],[16117,13,1],[16117,26,1],[16117,34,5],[16118,15,2],
[16119,28,1],[16119,31,1],[16120,2,1],[16120,30,1],[16121,25,1],
[16121,28,1],[16121,32,1],[16122,25,1],[16122,35,1],[16123,32,2],
[16123,44,1],[16124,25,13],[16124,42,1],[16125,1,1],[16125,2,1],
[16125,3,4],[16125,5,1],[16125,6,3],[16125,9,2],[16125,10,2],
[16125,12,1],[16125,15,2],[16125,16,1],[16125,17,1],[16125,19,1],
[16125,20,1],[16125,22,1],[16125,23,3],[16125,24,10],[16125,25,1],
[16125,26,1],[16125,27,3],[16125,28,14],[16125,29,1],[16125,30,1],
[16125,31,2],[16125,32,6],[16125,35,2],[16125,38,3],[16125,40,1],
[16125,41,1],[16125,42,50],[16125,43,3],[16125,44,8],[16125,45,1],
[16125,46,1],[16125,47,385],[16125,48,1],[16125,50,4],[16125,51,15],
[16125,53,2],[16125,56,15],[16125,59,1],[16125,66,5],[16125,67,1],
[16125,68,1],[16125,69,1],[16125,70,6],[16125,71,1],[16125,72,1],
[16125,74,15],[16125,76,1],[16125,77,1],[16126,3,3],[16126,24,1],
[16126,27,4],[16126,28,1],[16126,35,3],[16126,42,1],[16127,12,2],
[16127,28,2],[16128,2,1],[16128,20,1],[16128,23,1],[16128,39,1],
[16129,25,2],[16129,40,1],[16129,43,1],[16130,38,1],[16130,39,2],
[16131,2,3],[16132,20,1],[16132,26,1],[16132,28,1],[16133,35,2],
[16134,22,1],[16134,24,1],[16134,35,1],[16134,56,1],[16135,1,1],
[16135,16,1],[16136,28,1],[16136,74,1],[16136,76,5],[16137,0,2],
[16137,1,1],[16137,12,1],[16137,13,2],[16137,20,1],[16137,22,1],
[16137,24,2],[16137,25,1],[16137,27,1],[16137,28,4],[16137,29,3],
[16137,31,1],[16137,34,1],[16137,38,1],[16137,41,1],[16137,49,1],
[16137,53,1],[16137,62,1],[16138,3,1],[16138,13,1],[16139,5,2],
[16140,6,1],[16140,20,1],[16140,22,1],[16140,27,2],[16140,37,2],
[16140,70,1],[16141,13,1],[16141,55,1],[16142,27,2],[16143,5,2],
[16143,21,1],[16143,26,1],[16143,27,3],[16143,35,2],[16143,62,1],

[16144,0,29],[16144,1,2],[16144,7,13],[16144,10,1],[16144,14,2],
[16144,16,1],[16144,17,1],[16144,18,1],[16144,31,2],[16144,45,1],
[16144,46,1],[16144,64,1],[16144,71,1],[16144,73,5],[16144,77,2],
[16145,26,1],[16145,52,1],[16146,6,2],[16146,22,1],[16146,27,5],
[16146,28,2],[16146,35,1],[16146,56,2],[16147,28,1],[16147,63,1],
[16148,4,1],[16148,24,1],[16148,25,1],[16148,32,2],[16149,13,1],
[16149,39,1],[16150,1,1],[16150,2,1],[16150,4,1],[16150,5,1],
[16150,8,1],[16150,20,1],[16150,21,1],[16150,23,3],[16150,24,2],
[16150,26,1],[16150,27,36],[16150,28,12],[16150,32,2],[16150,33,1],
[16150,35,20],[16150,42,1],[16150,43,1],[16150,48,5],[16150,56,1],
[16150,66,1],[16150,70,2],[16150,72,1],[16150,74,1],[16150,76,1],
[16151,3,1],[16151,5,1],[16151,13,1],[16151,20,1],[16151,23,1],
[16151,28,4],[16151,32,3],[16151,33,1],[16151,39,1],[16151,43,1],
[16151,45,1],[16152,21,1],[16152,32,1],[16152,42,1],[16153,56,1],
[16153,61,1],[16154,21,1],[16154,23,1],[16154,24,1],[16154,28,8],
[16154,31,1],[16154,32,1],[16154,44,1],[16154,50,1],[16154,66,1],
[16155,18,1],[16155,70,1],[16156,42,2],[16157,1,1],[16157,10,1],
[16157,12,2],[16158,43,2],[16159,3,1],[16159,21,1],[16159,22,1],
[16159,23,2],[16159,25,3],[16159,27,1],[16159,28,1],[16159,31,1],
[16159,32,1],[16159,42,1],[16159,43,6],[16160,27,1],[16160,28,1],
[16160,35,1],[16160,70,1],[16161,20,1],[16161,53,1],[16162,4,2],
[16162,17,11],[16162,29,1],[16162,37,1],[16162,46,1],[16162,53,6],
[16162,71,1],[16163,12,1],[16163,15,1],[16163,27,1],[16163,49,1],
[16163,77,1],[16164,13,2],[16165,6,1],[16165,56,1],[16166,3,2],
[16166,5,1],[16166,21,3],[16166,22,3],[16166,23,5],[16166,24,1],
[16166,26,1],[16166,27,1],[16166,28,3],[16166,43,1],[16167,31,2],
[16168,28,1],[16168,48,1],[16169,2,4],[16169,45,1],[16170,28,1],
[16170,56,1],[16171,28,5],[16172,1,1],[16172,39,2],[16173,13,1],
[16173,45,1],[16174,27,1],[16174,28,1],[16175,1,2],[16175,13,1],
[16175,14,1],[16175,25,3],[16175,27,2],[16175,30,7],[16175,52,4],
[16176,24,2],[16177,63,1],[16177,74,1],[16177,76,1],[16178,10,1],
[16178,32,1],[16178,33,1],[16178,38,1],[16178,43,1],[16179,4,1],
[16179,31,1],[16179,32,1],[16179,40,1],[16179,63,39],[16179,68,1],
[16179,70,2],[16179,74,25],[16179,75,30],[16179,76,33],[16180,5,1],
[16180,32,4],[16180,35,1],[16180,70,1],[16181,31,1],[16181,42,6],
[16182,9,1],[16182,23,11],[16182,26,6],[16182,27,1],[16182,28,5],
[16182,32,1],[16182,33,3],[16182,52,2],[16182,56,1],[16183,28,1],
[16183,66,2],[16184,2,4],[16184,3,1],[16184,5,1],[16184,23,4],
[16184,24,1],[16184,25,1],[16184,31,2],[16184,42,1],[16184,68,2],
[16185,12,1],[16185,45,1],[16185,65,1],[16185,73,1],[16186,9,1],
[16186,10,1],[16186,13,1],[16186,76,1],[16187,3,1],[16187,26,1],
[16187,28,3],[16187,31,2],[16187,42,1],[16188,26,1],[16188,27,1],
[16188,63,12],[16188,66,1],[16188,74,4],[16188,75,8],[16188,76,10],
[16189,24,1],[16189,28,1],[16189,38,1],[16189,42,1],[16189,62,1],
[16190,46,1],[16190,65,1],[16191,38,1],[16191,44,3],[16191,66,1],
[16192,24,1],[16192,75,1],[16193,24,4],[16193,25,5],[16193,32,5],
[16194,2,3],[16194,3,1],[16194,22,1],[16194,53,1],[16194,59,2],
[16194,64,1],[16195,32,1],[16195,42,1],[16196,2,1],[16196,47,1],
[16197,20,1],[16197,53,1],[16198,2,1],[16198,48,1],[16199,2,1],
[16199,56,1],[16199,70,2],[16200,27,1],[16200,75,1],[16201,33,2],

[16202,43,1],[16202,67,1],[16203,1,11],[16203,2,1],[16203,7,2],
[16203,9,2],[16203,10,3],[16203,12,271],[16203,14,1],[16203,15,3],
[16203,16,1],[16203,18,2],[16203,19,1],[16203,20,1],[16203,23,15],
[16203,27,3],[16203,29,4],[16203,30,1],[16203,35,2],[16203,36,2],
[16203,37,1],[16203,39,2],[16203,41,3],[16203,45,2],[16203,46,1],
[16203,53,1],[16203,57,1],[16203,58,1],[16203,59,2],[16203,65,4],
[16203,67,2],[16203,73,1],[16203,77,5],[16203,78,1],[16204,9,1],
[16204,20,1],[16204,24,1],[16204,33,1],[16204,42,1],[16205,2,2],
[16205,28,1],[16206,21,1],[16206,23,1],[16206,24,1],[16206,28,2],
[16206,47,1],[16206,55,2],[16207,2,2],[16208,32,7],[16209,44,1],
[16209,66,1],[16210,2,4],[16211,4,3],[16211,22,1],[16211,24,1],
[16211,27,1],[16211,28,7],[16211,31,1],[16211,32,1],[16211,38,14],
[16211,43,2],[16211,44,86],[16211,47,1],[16211,50,3],[16211,51,1],
[16211,55,1],[16211,62,1],[16211,66,76],[16211,70,1],[16211,75,1],
[16212,30,1],[16212,31,1],[16213,21,1],[16213,47,2],[16214,31,1],
[16214,35,1],[16215,8,2],[16215,19,1],[16215,31,1],[16215,47,1],
[16216,26,1],[16216,27,2],[16216,28,1],[16217,6,1],[16217,24,1],
[16217,27,1],[16217,32,1],[16217,33,1],[16217,48,1],[16217,56,2],
[16218,27,1],[16218,31,1],[16218,62,1],[16219,2,29],[16219,22,1],
[16219,23,1],[16219,25,1],[16219,27,1],[16219,30,1],[16219,31,5],
[16219,32,1],[16219,38,1],[16219,47,1],[16219,62,1],[16219,70,1],
[16219,78,1],[16220,8,1],[16220,35,2],[16221,23,1],[16221,24,2],
[16222,28,1],[16222,35,1],[16223,24,2],[16223,27,2],[16223,28,3],
[16223,31,3],[16223,32,8],[16223,35,1],[16223,42,1],[16224,5,1],
[16224,22,1],[16224,23,4],[16224,25,1],[16224,27,3],[16225,28,1],
[16225,50,1],[16225,66,1],[16226,24,2],[16226,28,1],[16226,47,5],
[16227,5,2],[16227,27,1],[16227,28,22],[16227,50,1],[16227,66,1],
[16228,2,2],[16229,1,2],[16229,7,1],[16229,9,1],[16229,12,1],
[16229,13,1],[16229,17,3],[16229,20,1],[16229,37,1],[16229,39,2],
[16229,55,1],[16229,77,1],[16230,26,2],[16231,24,1],[16231,28,1],
[16232,20,1],[16232,24,1],[16232,34,1],[16232,42,1],[16232,78,1],
[16233,21,6],[16233,33,5],[16234,5,1],[16234,8,1],[16234,22,1],
[16234,25,3],[16234,26,4],[16234,27,1],[16234,28,1],[16234,32,6],
[16234,34,1],[16234,43,1],[16234,62,1],[16235,1,48],[16235,2,4],
[16235,5,3],[16235,6,2],[16235,9,3],[16235,10,1],[16235,12,2],
[16235,13,13],[16235,14,4],[16235,15,2],[16235,17,2],[16235,20,4],
[16235,21,7],[16235,22,3],[16235,23,3],[16235,24,4],[16235,25,1],
[16235,28,3],[16235,29,1],[16235,32,1],[16235,33,6],[16235,35,3],
[16235,39,1],[16235,42,3],[16235,43,4],[16235,44,1],[16235,45,3],
[16235,47,3],[16235,48,1],[16235,49,1],[16235,54,1],[16235,55,1],
[16235,60,1],[16235,65,2],[16235,69,1],[16235,70,2],[16235,73,1],
[16235,74,1],[16235,76,1],[16235,78,1],[16236,9,1],[16236,10,1],
[16236,20,21],[16236,41,1],[16236,43,1],[16237,4,1],[16237,47,1],
[16238,28,2],[16239,27,6],[16240,24,3],[16241,23,1],[16241,28,1],
[16242,3,5],[16242,4,17],[16242,5,7],[16242,6,5],[16242,9,2],
[16242,14,1],[16242,15,2],[16242,17,8],[16242,19,1],[16242,22,1],
[16242,23,3],[16242,24,15],[16242,25,3],[16242,26,2],[16242,27,21],
[16242,28,7],[16242,31,7],[16242,32,6],[16242,33,1],[16242,34,1],
[16242,35,7],[16242,38,3],[16242,40,1],[16242,42,3],[16242,43,1],
[16242,44,1],[16242,45,2],[16242,46,1],[16242,49,1],[16242,50,15],

[16242,53,5],[16242,54,2],[16242,55,1],[16242,56,4],[16242,60,1],
[16242,65,2],[16242,66,2],[16242,67,1],[16242,70,2],[16242,71,1],
[16242,73,1],[16242,77,1],[16243,68,2],[16244,22,1],[16244,26,1],
[16244,28,3],[16244,45,1],[16244,68,1],[16245,56,1],[16245,74,1],
[16246,26,1],[16246,44,1],[16246,50,1],[16246,52,1],[16247,23,1],
[16247,35,1],[16248,37,1],[16248,71,1],[16249,1,33],[16249,2,47],
[16249,3,53],[16249,4,26],[16249,5,57],[16249,6,14],[16249,8,6],
[16249,9,2],[16249,13,3],[16249,17,36],[16249,18,1],[16249,20,19],
[16249,21,79],[16249,22,344],[16249,23,330],[16249,24,502],
[16249,25,278],[16249,26,85],[16249,27,477],[16249,28,162],
[16249,30,90],[16249,31,114],[16249,32,213],[16249,33,43],
[16249,34,30],[16249,35,206],[16249,36,4],[16249,37,2],[16249,38,93],
[16249,39,42],[16249,40,2],[16249,41,1],[16249,42,38],[16249,43,63],
[16249,45,19],[16249,47,37],[16249,48,16],[16249,50,67],[16249,51,1],
[16249,52,11],[16249,53,69],[16249,54,1],[16249,56,36],[16249,57,1],
[16249,59,9],[16249,60,1],[16249,62,61],[16249,65,1],[16249,66,2],
[16249,67,1],[16249,68,1],[16249,74,1],[16250,5,1],[16250,27,1],
[16250,39,1],[16251,1,1],[16251,15,1],[16251,19,1],[16251,36,1],
[16251,58,1],[16252,5,1],[16252,8,1],[16252,20,2],[16252,21,1],
[16252,22,3],[16252,23,2],[16252,24,1],[16252,27,8],[16252,33,2],
[16252,35,11],[16252,38,1],[16252,42,1],[16252,46,1],[16252,47,1],
[16253,20,1],[16253,21,1],[16253,23,1],[16253,26,1],[16253,31,1],
[16253,78,1],[16254,1,1],[16254,2,1],[16254,9,1],[16254,15,1],
[16254,19,1],[16254,23,2],[16254,25,1],[16254,27,1],[16254,32,1],
[16254,43,1],[16254,48,1],[16254,54,1],[16254,60,1],[16255,21,1],
[16255,31,1],[16255,43,2],[16256,6,1],[16256,21,1],[16256,24,5],
[16256,26,1],[16256,27,1],[16256,32,3],[16256,40,1],[16256,43,1],
[16256,47,3],[16256,51,1],[16256,63,1],[16256,66,1],[16256,68,3],
[16256,75,1],[16257,3,1],[16257,5,1],[16257,21,1],[16257,22,1],
[16257,23,1],[16257,25,1],[16257,26,2],[16257,35,2],[16257,44,1],
[16257,62,2],[16257,63,5],[16257,66,1],[16257,68,2],[16257,74,11],
[16257,75,3],[16257,76,11],[16258,25,1],[16258,27,1],[16258,33,4],
[16259,26,6],[16259,39,1],[16259,42,2],[16260,6,1],[16260,27,1],
[16261,21,1],[16261,59,2],[16262,2,3],[16263,24,1],[16263,43,1],
[16264,52,13],[16265,26,2],[16266,27,1],[16266,48,1],[16266,66,1],
[16267,15,1],[16267,28,1],[16268,21,1],[16268,31,3],[16269,44,1],
[16269,55,1],[16270,20,1],[16270,22,1],[16270,28,1],[16270,68,1],
[16271,8,2],[16271,25,2],[16271,35,4],[16272,10,1],[16272,39,18],
[16273,30,2],[16274,19,1],[16274,59,1],[16274,78,1],[16275,29,1],
[16275,65,1],[16276,31,2],[16277,2,1],[16277,3,1],[16277,22,2],
[16277,31,1],[16277,35,1],[16278,24,1],[16278,32,1],[16279,33,1],
[16279,35,3],[16279,39,4],[16280,1,1],[16280,8,1],[16280,23,2],
[16280,28,1],[16280,29,1],[16280,30,2],[16280,32,1],[16281,0,1],
[16281,1,43],[16281,2,9],[16281,3,11],[16281,4,1],[16281,5,2],
[16281,7,3],[16281,8,11],[16281,9,1],[16281,10,1],[16281,12,13],
[16281,13,81],[16281,15,5],[16281,16,6],[16281,17,1],[16281,20,47],
[16281,22,14],[16281,23,10],[16281,24,8],[16281,25,2],[16281,26,29],
[16281,27,55],[16281,28,6],[16281,29,4],[16281,30,13],[16281,31,15],
[16281,32,22],[16281,33,16],[16281,34,24],[16281,35,14],[16281,36,4],
[16281,39,30],[16281,42,5],[16281,43,21],[16281,45,1],[16281,47,21],

[16281,48,41],[16281,51,3],[16281,52,2],[16281,53,9],[16281,54,1],
[16281,55,1],[16281,59,3],[16281,60,1],[16281,65,1],[16281,66,1],
[16281,69,1],[16281,70,1],[16281,73,6],[16281,78,1],[16281,79,2],
[16282,39,2],[16283,48,3],[16283,78,1],[16284,63,22],[16284,74,26],
[16284,75,30],[16284,76,42],[16284,79,1],[16285,27,2],[16286,20,1],
[16286,27,1],[16286,28,1],[16287,2,4],[16287,3,16],[16287,5,10],
[16287,8,1],[16287,12,1],[16287,16,1],[16287,17,1],[16287,21,2],
[16287,22,5],[16287,23,23],[16287,24,23],[16287,25,9],[16287,26,28],
[16287,27,20],[16287,28,18],[16287,30,1],[16287,31,8],[16287,32,16],
[16287,35,5],[16287,37,1],[16287,39,1],[16287,41,2],[16287,42,5],
[16287,43,15],[16287,47,2],[16287,48,2],[16287,52,1],[16287,53,1],
[16287,56,3],[16287,62,1],[16287,76,1],[16288,2,13],[16288,21,4],
[16289,9,1],[16289,18,1],[16289,34,1],[16290,24,2],[16291,24,1],
[16291,28,1],[16291,30,1],[16292,2,1],[16292,32,1],[16293,1,15],
[16293,5,1],[16293,13,8],[16293,16,1],[16293,20,5],[16293,21,2],
[16293,23,5],[16293,24,1],[16293,25,5],[16293,26,1],[16293,27,1],
[16293,28,1],[16293,31,5],[16293,33,1],[16293,37,1],[16293,39,6],
[16293,42,10],[16293,43,2],[16293,47,1],[16293,58,1],[16293,60,1],
[16293,66,1],[16293,78,1],[16294,24,1],[16294,68,1],[16295,24,1],
[16295,26,2],[16295,28,3],[16296,44,2],[16296,66,3],[16297,1,1],
[16297,2,1],[16297,3,2],[16297,4,48],[16297,5,14],[16297,9,2],
[16297,12,1],[16297,13,1],[16297,15,18],[16297,16,1],[16297,17,127],
[16297,22,2],[16297,23,3],[16297,24,2],[16297,25,3],[16297,26,3],
[16297,27,1],[16297,28,10],[16297,31,3],[16297,33,4],[16297,34,2],
[16297,35,4],[16297,37,2],[16297,38,2],[16297,42,1],[16297,43,2],
[16297,44,15],[16297,45,2],[16297,47,1],[16297,50,100],[16297,51,1],
[16297,53,63],[16297,55,22],[16297,56,1],[16297,60,1],[16297,61,1],
[16297,63,3],[16297,65,1],[16297,66,29],[16297,69,2],[16297,70,1],
[16297,75,1],[16297,76,1],[16297,77,1],[16297,78,1],[16297,79,1],
[16298,3,1],[16298,31,1],[16298,33,1],[16299,20,1],[16299,35,1],
[16299,48,1],[16300,29,1],[16300,60,1],[16301,4,1],[16301,5,2],
[16301,6,4],[16301,23,2],[16301,25,1],[16301,70,1],[16302,7,1],
[16302,12,2],[16302,13,3],[16302,32,1],[16302,39,1],[16303,51,1],
[16303,56,1],[16304,0,1],[16304,45,1],[16305,28,2],[16306,42,1],
[16306,47,1],[16307,28,2],[16307,44,1],[16308,2,1],[16308,3,1],
[16308,22,1],[16308,23,1],[16308,27,1],[16308,31,1],[16308,44,4],
[16308,47,1],[16308,56,2],[16308,66,7],[16308,68,1],[16309,26,1],
[16309,27,5],[16310,20,1],[16310,34,1],[16310,59,1],[16311,24,1],
[16311,30,1],[16312,33,1],[16312,76,1],[16313,21,1],[16313,44,1],
[16314,13,1],[16314,20,1],[16315,0,2],[16315,2,2],[16315,3,3],
[16315,4,470],[16315,5,11],[16315,7,2],[16315,8,1],[16315,9,2],
[16315,10,2],[16315,15,25],[16315,17,47],[16315,21,4],[16315,22,8],
[16315,24,1],[16315,25,4],[16315,26,1],[16315,27,4],[16315,28,39],
[16315,29,1],[16315,31,3],[16315,32,2],[16315,33,3],[16315,35,1],
[16315,40,2],[16315,42,1],[16315,44,3],[16315,48,1],[16315,50,592],
[16315,51,1],[16315,53,85],[16315,55,25],[16315,56,1],[16315,62,5],
[16315,63,7],[16315,65,1],[16315,66,7],[16315,68,1],[16315,69,3],
[16315,70,1],[16315,71,2],[16315,74,2],[16315,75,1],[16316,1,3],
[16316,4,7],[16316,7,1],[16316,8,1],[16316,10,2],[16316,12,3],
[16316,13,1],[16316,14,1],[16316,17,1],[16316,36,1],[16316,38,3],

[16316,40,1],[16316,41,2],[16316,44,2],[16316,45,1],[16316,50,5],
[16316,53,2],[16316,57,1],[16316,60,1],[16316,65,1],[16316,66,6],
[16316,67,1],[16316,70,1],[16316,77,1],[16317,2,2],[16317,47,1],
[16318,35,2],[16318,47,1],[16319,2,2],[16319,3,8],[16319,4,86],
[16319,5,8],[16319,9,2],[16319,13,1],[16319,15,1],[16319,21,3],
[16319,22,7],[16319,23,3],[16319,24,7],[16319,25,8],[16319,26,1],
[16319,27,9],[16319,28,54],[16319,31,18],[16319,32,11],[16319,33,2],
[16319,35,80],[16319,38,79],[16319,40,2],[16319,42,1],[16319,44,707],
[16319,48,3],[16319,50,100],[16319,51,1],[16319,52,1],[16319,55,5],
[16319,56,1],[16319,61,1],[16319,63,1],[16319,65,2],[16319,66,567],
[16319,68,4],[16319,69,1],[16319,70,3],[16319,72,3],[16319,74,5],
[16319,75,4],[16319,76,5],[16319,78,1],[16320,3,3],[16320,15,1],
[16320,23,5],[16320,24,3],[16320,26,1],[16320,27,3],[16320,28,1],
[16320,31,2],[16320,48,2],[16321,63,1],[16321,74,1],[16321,75,1],
[16321,76,1],[16322,2,1],[16322,3,1],[16322,21,1],[16322,77,1],
[16323,21,1],[16323,26,1],[16324,4,1],[16324,15,1],[16325,1,2],
[16325,3,1],[16325,13,1],[16325,23,2],[16325,30,1],[16325,64,1],
[16325,65,1],[16325,76,1],[16326,56,1],[16326,68,1],[16326,69,1],
[16327,1,2],[16327,13,1],[16327,18,1],[16327,20,1],[16328,2,1],
[16328,35,1],[16329,5,1],[16329,23,6],[16329,28,1],[16330,22,1],
[16330,62,1],[16331,2,2],[16332,27,2],[16332,30,4],[16332,33,25],
[16332,35,7],[16332,52,4],[16332,66,1],[16332,76,1],[16333,31,2],
[16333,35,1],[16334,2,1],[16334,39,1],[16334,57,1],[16335,9,1],
[16335,20,1],[16336,5,1],[16336,15,1],[16336,22,1],[16336,23,1],
[16336,26,1],[16336,56,2],[16337,1,1],[16337,9,1],[16338,23,1],
[16338,28,1],[16339,24,3],[16339,27,2],[16339,40,1],[16340,2,1],
[16340,8,3],[16340,9,2],[16340,10,1],[16340,12,47],[16340,13,9],
[16340,15,1],[16340,17,1],[16340,19,2],[16340,21,1],[16340,22,1],
[16340,25,75],[16340,26,34],[16340,28,4],[16340,29,7],[16340,30,5],
[16340,31,154],[16340,33,2],[16340,43,39],[16340,48,7],[16340,57,1],
[16340,59,2],[16340,60,1],[16340,62,5],[16340,63,2],[16340,65,1],
[16340,67,1],[16340,68,1],[16340,70,1],[16340,73,2],[16340,77,1],
[16340,78,2],[16341,27,1],[16341,35,1],[16342,4,1],[16342,41,1],
[16343,21,1],[16343,24,1],[16343,35,1],[16343,68,1],[16344,3,2],
[16344,30,1],[16344,31,1],[16344,39,1],[16344,40,1],[16344,43,1],
[16344,46,1],[16344,56,1],[16344,64,2],[16345,5,1],[16345,52,1],
[16346,2,1],[16346,25,1],[16346,32,1],[16347,2,6],[16347,3,1],
[16347,17,1],[16347,22,1],[16347,23,1],[16347,24,1],[16347,37,4],
[16347,42,2],[16347,46,1],[16347,48,1],[16347,62,1],[16348,59,1],
[16348,71,1],[16349,9,2],[16349,36,1],[16349,41,1],[16349,65,1],
[16350,22,1],[16350,24,1],[16350,27,1],[16350,35,1],[16350,70,1],
[16351,2,1],[16351,9,1],[16351,10,1],[16351,23,1],[16351,28,1],
[16352,27,1],[16352,48,1],[16353,23,1],[16353,27,2],[16353,28,3],
[16354,2,38],[16354,3,9],[16354,4,1],[16354,5,2],[16354,6,1],
[16354,8,2],[16354,11,4],[16354,15,1],[16354,19,1],[16354,20,3],
[16354,22,3],[16354,24,2],[16354,25,2],[16354,26,1],[16354,27,4],
[16354,28,3],[16354,30,3],[16354,31,1],[16354,35,2],[16354,36,1],
[16354,38,1],[16354,39,1],[16354,41,1],[16354,42,1],[16354,43,1],
[16354,45,1],[16354,48,1],[16354,50,1],[16354,52,1],[16354,53,4],
[16354,56,3],[16354,58,1],[16354,60,1],[16354,62,1],[16354,64,1],

[16354,65,1],[16354,69,1],[16354,70,1],[16354,76,1],[16354,77,1],
[16355,28,1],[16355,69,3],[16355,70,3],[16355,72,1],[16356,7,9],
[16356,45,1],[16357,7,1],[16357,8,1],[16357,10,2],[16357,19,1],
[16357,20,2],[16357,21,1],[16357,22,1],[16357,23,1],[16357,24,1],
[16357,25,1],[16357,27,1],[16357,28,1],[16357,30,1],[16357,31,2],
[16357,32,1],[16357,33,37],[16357,35,17],[16357,38,3],[16357,52,3],
[16357,68,1],[16357,70,1],[16357,74,1],[16357,76,1],[16358,27,1],
[16358,66,1],[16359,25,1],[16359,49,1],[16359,56,1],[16360,3,2],
[16360,4,1],[16360,6,1],[16360,8,1],[16360,10,1],[16360,23,2],
[16360,25,1],[16360,32,2],[16360,35,1],[16360,59,1],[16360,74,2],
[16361,22,1],[16361,23,2],[16361,24,1],[16361,25,1],[16361,27,1],
[16361,32,1],[16361,59,1],[16362,3,2],[16362,33,2],[16362,78,1],
[16363,24,1],[16363,27,1],[16363,35,1],[16364,2,2],[16365,22,1],
[16365,23,1],[16365,25,2],[16366,44,1],[16366,66,1],[16367,6,1],
[16367,47,1],[16368,31,1],[16368,35,1],[16369,17,1],[16369,49,1],
[16370,52,7],[16371,28,1],[16371,30,1],[16371,58,1],[16372,56,5],
[16373,27,1],[16373,28,1],[16374,3,1],[16374,4,1],[16374,62,8],
[16375,2,2],[16376,9,2],[16376,10,2],[16376,16,1],[16376,78,1],
[16377,57,1],[16377,59,1],[16377,71,1],[16378,6,1],[16378,22,1],
[16378,77,1],[16379,2,3],[16380,2,2],[16380,3,1],[16380,22,2],
[16380,23,1],[16380,24,1],[16380,28,1],[16380,38,1],[16381,22,1],
[16381,28,1],[16381,31,1],[16381,35,1],[16382,13,1],[16382,22,1],
[16382,23,1],[16382,27,1],[16382,33,1],[16382,35,2],[16382,42,1],
[16382,65,1],[16383,24,1],[16383,32,2],[16384,2,2],[16384,32,1],
[16385,14,1],[16385,36,1],[16385,49,1],[16386,15,2],[16386,17,1],
[16386,38,1],[16386,53,2],[16387,32,1],[16387,34,3],[16387,47,1],
[16388,25,2],[16389,5,1],[16389,33,18],[16390,2,1],[16390,30,1],
[16390,39,1],[16391,23,2],[16391,32,4],[16391,75,1],[16392,9,1],
[16392,10,1],[16392,12,1],[16392,23,1],[16392,41,1],[16392,78,1],
[16393,24,1],[16393,31,1],[16394,3,11],[16394,5,1],[16394,6,5],
[16394,9,2],[16394,10,1],[16394,13,5],[16394,16,1],[16394,21,5],
[16394,23,7],[16394,24,33],[16394,25,4],[16394,26,11],[16394,27,2],
[16394,28,14],[16394,30,1],[16394,31,5],[16394,32,11],[16394,33,7],
[16394,35,1],[16394,40,2],[16394,42,8],[16394,43,47],[16394,47,2],
[16394,48,1],[16394,53,1],[16394,56,7],[16394,59,7],[16394,60,3],
[16394,62,1],[16394,66,2],[16395,2,2],[16395,5,1],[16395,12,1],
[16395,17,1],[16395,22,1],[16395,70,1],[16396,1,2],[16396,2,1],
[16396,3,4],[16396,4,1],[16396,5,3],[16396,6,1],[16396,12,1],
[16396,13,2],[16396,15,4],[16396,17,2],[16396,21,1],[16396,22,7],
[16396,23,6],[16396,24,2],[16396,26,1],[16396,27,5],[16396,31,2],
[16396,32,3],[16396,33,2],[16396,35,6],[16396,38,4],[16396,40,3],
[16396,41,1],[16396,42,2],[16396,43,2],[16396,44,5],[16396,47,4],
[16396,50,2],[16396,51,6],[16396,52,1],[16396,53,4],[16396,55,2],
[16396,56,6],[16396,58,1],[16396,60,1],[16396,62,1],[16396,66,5],
[16396,68,9],[16396,69,1],[16396,70,2],[16396,74,2],[16396,76,3],
[16397,2,10],[16397,6,1],[16397,20,1],[16397,23,1],[16397,24,1],
[16397,25,12],[16397,27,1],[16397,28,2],[16397,31,4],[16397,33,4],
[16397,38,1],[16397,42,1],[16397,48,1],[16397,56,1],[16397,74,1],
[16398,5,1],[16398,26,1],[16399,2,2],[16400,24,1],[16400,27,1],
[16400,28,1],[16401,2,1],[16401,28,1],[16401,30,1],[16401,35,1],

[16401,70,1],[16402,27,1],[16402,28,1],[16403,40,1],[16403,63,4],
[16403,74,4],[16403,75,3],[16403,76,10],[16404,7,4],[16404,39,2],
[16404,50,1],[16404,62,1],[16404,64,1],[16405,3,1],[16405,5,1],
[16405,22,6],[16405,23,6],[16405,24,1],[16405,26,1],[16405,27,2],
[16405,28,2],[16405,35,1],[16405,43,2],[16405,47,1],[16405,56,2],
[16406,25,3],[16407,15,1],[16407,55,1],[16408,8,1],[16408,11,1],
[16409,12,2],[16409,20,1],[16409,30,1],[16410,1,15],[16410,2,1],
[16410,29,1],[16410,30,3],[16410,33,1],[16410,37,1],[16410,38,1],
[16410,42,1],[16410,47,2],[16410,57,1],[16410,58,1],[16410,65,2],
[16410,77,1],[16411,9,1],[16411,13,1],[16411,19,1],[16411,58,1],
[16412,1,5],[16412,15,2],[16413,2,3],[16413,3,1],[16414,0,3],
[16414,1,30],[16414,2,18],[16414,3,16],[16414,5,1],[16414,6,1],
[16414,7,2],[16414,8,2],[16414,9,10],[16414,10,11],[16414,11,5],
[16414,12,6],[16414,13,33],[16414,14,3],[16414,15,8],[16414,16,8],
[16414,17,4],[16414,19,14],[16414,20,59],[16414,21,2],[16414,22,15],
[16414,23,73],[16414,24,19],[16414,25,10],[16414,26,10],[16414,27,62],
[16414,28,35],[16414,29,1],[16414,30,14],[16414,31,15],[16414,32,16],
[16414,33,9],[16414,34,9],[16414,35,32],[16414,36,1],[16414,37,7],
[16414,39,3],[16414,40,2],[16414,41,4],[16414,42,8],[16414,43,8],
[16414,44,1],[16414,45,7],[16414,47,5],[16414,48,2],[16414,49,3],
[16414,50,5],[16414,51,1],[16414,52,1],[16414,53,4],[16414,54,2],
[16414,55,5],[16414,56,9],[16414,57,5],[16414,58,5],[16414,59,13],
[16414,60,4],[16414,61,1],[16414,62,1],[16414,64,3],[16414,65,6],
[16414,66,3],[16414,67,3],[16414,68,1],[16414,70,4],[16414,71,2],
[16414,73,4],[16414,74,1],[16414,77,6],[16414,78,6],[16415,31,1],
[16415,33,4],[16416,22,1],[16416,24,2],[16416,31,3],[16416,42,1],
[16417,1,19],[16417,7,1],[16417,12,2],[16417,27,1],[16417,34,1],
[16417,36,2],[16417,73,1],[16418,47,3],[16419,6,3],[16419,20,7],
[16419,22,2],[16419,23,13],[16419,24,11],[16419,25,4],[16419,26,1],
[16419,27,1],[16419,31,1],[16419,32,3],[16419,33,1],[16419,35,1],
[16419,39,1],[16419,42,3],[16419,44,1],[16419,48,1],[16419,51,2],
[16419,56,9],[16419,62,1],[16419,68,1],[16419,69,4],[16419,74,21],
[16420,1,1],[16420,15,1],[16420,44,3],[16420,66,1],[16421,24,3],
[16422,28,1],[16422,40,1],[16422,43,5],[16423,1,1],[16423,2,2],
[16423,3,3],[16423,5,8],[16423,6,1],[16423,7,2],[16423,8,3],
[16423,10,2],[16423,11,4],[16423,13,4],[16423,15,4],[16423,19,2],
[16423,20,8],[16423,21,3],[16423,22,1],[16423,24,6],[16423,26,2],
[16423,27,2],[16423,33,5],[16423,34,1],[16423,35,1],[16423,42,1],
[16423,43,1],[16423,44,2],[16423,45,1],[16423,47,1],[16423,48,1],
[16423,50,1],[16423,51,2],[16423,53,1],[16423,55,3],[16423,56,5],
[16423,57,1],[16423,62,1],[16423,63,1],[16423,66,1],[16423,68,1],
[16423,70,2],[16423,73,1],[16423,74,4],[16423,77,1],[16424,34,1],
[16424,45,1],[16425,2,1],[16425,26,1],[16426,2,1],[16426,3,1],
[16427,28,1],[16427,44,1],[16427,50,1],[16427,55,1],[16428,26,1],
[16428,28,1],[16428,35,1],[16428,43,1],[16429,2,1],[16429,10,2],
[16429,12,19],[16429,15,1],[16429,27,1],[16429,29,9],[16429,33,1],
[16429,40,1],[16429,59,1],[16429,62,1],[16429,64,1],[16429,65,2],
[16430,27,2],[16431,23,2],[16432,24,2],[16432,26,1],[16432,28,1],
[16432,32,3],[16432,35,1],[16432,52,1],[16433,28,2],[16433,32,1],
[16434,17,1],[16434,19,1],[16434,25,1],[16434,44,1],[16434,52,4],

[16434,74,1],[16434,76,1],[16435,28,1],[16435,30,1],[16436,2,8],
[16437,9,3],[16438,22,1],[16438,28,1],[16439,2,6],[16439,47,1],
[16439,65,1],[16440,27,1],[16440,35,1],[16441,56,4],[16442,10,1],
[16442,23,1],[16442,46,1],[16442,67,1],[16443,2,1],[16443,30,1],
[16444,26,8],[16444,28,1],[16444,43,9],[16444,47,1],[16445,10,1],
[16445,24,1],[16445,56,2],[16445,60,1],[16446,3,1],[16446,28,24],
[16447,1,1],[16447,28,1],[16447,39,1],[16448,0,1],[16448,5,1],
[16448,12,1],[16448,20,1],[16448,25,1],[16448,27,1],[16448,28,1],
[16448,35,1],[16448,39,1],[16448,42,1],[16448,43,1],[16448,44,1],
[16448,51,1],[16448,63,1],[16448,70,2],[16449,3,1],[16449,26,1],
[16449,27,1],[16449,32,1],[16450,9,1],[16450,15,3],[16450,16,1],
[16450,18,1],[16450,29,1],[16450,41,1],[16450,53,1],[16451,0,1],
[16451,1,464],[16451,3,1],[16451,8,8],[16451,10,3],[16451,11,1],
[16451,12,27],[16451,13,22],[16451,15,6],[16451,17,7],[16451,20,4],
[16451,23,1],[16451,29,9],[16451,34,3],[16451,36,4],[16451,39,16],
[16451,53,5],[16451,55,7],[16451,58,1],[16451,59,1],[16451,65,2],
[16451,73,5],[16452,9,1],[16452,71,1],[16453,1,1],[16453,39,2],
[16454,1,10],[16454,2,1],[16454,3,3],[16454,5,9],[16454,6,2],
[16454,8,1],[16454,10,1],[16454,11,1],[16454,12,1],[16454,13,2],
[16454,15,5],[16454,19,1],[16454,20,1],[16454,21,5],[16454,22,5],
[16454,23,4],[16454,24,3],[16454,25,25],[16454,26,6],[16454,27,7],
[16454,32,2],[16454,33,6],[16454,35,12],[16454,37,1],[16454,38,1],
[16454,39,1],[16454,40,1],[16454,42,1],[16454,43,2],[16454,44,5],
[16454,47,1],[16454,50,2],[16454,51,3],[16454,55,2],[16454,56,3],
[16454,57,1],[16454,66,5],[16454,72,1],[16454,76,1],[16454,77,1],
[16454,79,1],[16455,28,2],[16456,28,6],[16457,28,3],[16458,22,1],
[16458,45,1],[16459,6,1],[16459,15,1],[16459,21,1],[16459,23,1],
[16459,40,1],[16459,47,1],[16459,51,1],[16459,56,1],[16459,61,3],
[16459,68,2],[16459,69,2],[16459,70,6],[16460,5,1],[16460,72,1],
[16461,21,1],[16461,27,1],[16462,21,1],[16462,22,1],[16462,50,1],
[16462,68,1],[16462,69,1],[16462,70,8],[16462,72,1],[16463,4,1],
[16463,5,2],[16463,23,1],[16463,24,1],[16463,26,1],[16463,27,1],
[16463,28,11],[16463,32,1],[16463,33,1],[16463,35,1],[16463,38,1],
[16463,40,3],[16463,42,1],[16463,43,1],[16463,44,2],[16463,62,1],
[16463,68,2],[16463,70,2],[16463,74,1],[16463,76,4],[16464,20,1],
[16464,62,1],[16465,3,5],[16465,21,1],[16465,23,2],[16465,47,1],
[16465,50,1],[16466,0,1],[16466,1,2],[16466,2,3],[16466,3,29],
[16466,4,3],[16466,5,8],[16466,6,5],[16466,7,1],[16466,8,1],
[16466,9,1],[16466,10,1],[16466,11,3],[16466,12,1],[16466,13,1],
[16466,14,1],[16466,15,1],[16466,16,2],[16466,17,1],[16466,19,2],
[16466,20,4],[16466,21,6],[16466,22,2],[16466,23,6],[16466,24,17],
[16466,25,1],[16466,26,6],[16466,27,5],[16466,28,10],[16466,31,26],
[16466,32,9],[16466,33,36],[16466,35,5],[16466,37,5],[16466,38,3],
[16466,42,4],[16466,43,1],[16466,44,2],[16466,45,3],[16466,46,2],
[16466,47,12],[16466,48,1],[16466,49,2],[16466,50,4],[16466,51,1],
[16466,52,2],[16466,53,1],[16466,54,1],[16466,55,2],[16466,56,28],
[16466,57,1],[16466,60,1],[16466,62,2],[16466,63,1],[16466,66,3],
[16466,68,5],[16466,70,5],[16466,72,1],[16466,73,1],[16466,74,4],
[16466,75,2],[16466,76,4],[16466,78,1],[16467,22,1],[16467,23,1],
[16467,24,1],[16467,31,1],[16467,42,1],[16467,70,1],[16468,0,1],

[16468,1,132],[16468,6,26],[16468,7,2],[16468,8,11],[16468,9,4],
[16468,12,11],[16468,13,68],[16468,15,3],[16468,17,4],[16468,20,5],
[16468,22,2],[16468,23,1],[16468,26,3],[16468,28,3],[16468,29,4],
[16468,30,6],[16468,31,1],[16468,34,9],[16468,36,6],[16468,37,2],
[16468,39,14],[16468,43,4],[16468,45,9],[16468,47,1],[16468,53,4],
[16468,55,3],[16468,70,2],[16468,73,3],[16469,2,1],[16469,33,1],
[16469,40,1],[16469,44,1],[16469,63,12],[16469,68,2],[16469,70,1],
[16469,72,2],[16469,74,13],[16469,75,3],[16469,76,2],[16470,2,3],
[16470,3,4],[16470,5,3],[16470,17,1],[16470,21,1],[16470,22,1],
[16470,23,2],[16470,24,3],[16470,26,2],[16470,27,4],[16470,28,3],
[16470,35,1],[16470,43,1],[16470,47,5],[16470,56,3],[16470,78,2],
[16471,3,1],[16471,24,1],[16471,27,1],[16471,28,7],[16471,47,1],
[16471,69,1],[16472,47,1],[16472,61,1],[16472,68,1],[16472,76,1],
[16473,44,2],[16474,3,1],[16474,6,1],[16474,22,2],[16474,27,3],
[16474,32,1],[16474,33,1],[16474,35,4],[16474,56,1],[16474,69,1],
[16475,3,3],[16475,5,1],[16475,6,2],[16475,21,1],[16475,22,4],
[16475,23,10],[16475,24,6],[16475,26,2],[16475,27,7],[16475,28,2],
[16475,31,1],[16475,33,1],[16475,35,1],[16475,40,1],[16475,42,1],
[16475,56,2],[16475,60,2],[16475,62,1],[16475,70,1],[16476,21,38],
[16476,24,2],[16476,63,1],[16476,66,1],[16476,70,2],[16476,75,1],
[16477,25,3],[16477,28,1],[16477,35,1],[16478,35,2],[16479,3,1],
[16479,42,1],[16479,47,2],[16480,5,1],[16480,9,1],[16480,22,1],
[16480,35,3],[16480,45,1],[16480,47,1],[16480,58,1],[16480,63,1],
[16481,6,1],[16481,27,1],[16481,28,1],[16481,35,1],[16482,27,2],
[16483,21,1],[16483,31,1],[16484,22,2],[16484,25,2],[16484,43,1],
[16485,2,4],[16486,1,1],[16486,2,2],[16486,3,4],[16486,5,1],
[16486,20,3],[16486,23,3],[16486,24,1],[16486,25,8],[16486,26,2],
[16486,27,2],[16486,28,1],[16486,31,1],[16486,32,15],[16486,33,1],
[16486,34,1],[16486,35,1],[16486,37,1],[16486,39,2],[16486,40,1],
[16486,42,1],[16486,43,3],[16486,47,1],[16486,48,1],[16486,52,3],
[16486,56,1],[16486,59,1],[16486,62,33],[16487,10,1],[16487,78,1],
[16488,3,1],[16488,6,3],[16488,22,2],[16488,24,1],[16488,26,1],
[16488,27,3],[16488,28,1],[16488,43,1],[16488,46,1],[16488,57,1],
[16488,62,1],[16488,75,1],[16489,20,1],[16489,23,2],[16489,27,1],
[16489,28,2],[16489,33,14],[16490,3,1],[16490,6,1],[16490,17,1],
[16490,22,1],[16490,23,2],[16490,24,2],[16490,27,7],[16490,33,1],
[16490,35,1],[16490,54,1],[16490,56,1],[16490,79,1],[16491,1,22],
[16491,2,2],[16491,5,1],[16491,12,1],[16491,13,3],[16491,18,1],
[16491,29,2],[16491,30,1],[16491,33,2],[16491,34,1],[16491,37,2],
[16491,39,7],[16491,54,1],[16491,57,1],[16491,65,1],[16491,69,4],
[16491,70,3],[16492,5,1],[16492,35,2],[16493,1,1],[16493,9,3],
[16493,10,2],[16493,11,1],[16493,12,3],[16493,14,2],[16493,19,1],
[16493,20,1],[16493,28,3],[16494,10,1],[16494,17,2],[16494,55,1],
[16494,59,1],[16495,22,1],[16495,63,1],[16496,4,3],[16496,23,1],
[16496,26,1],[16496,27,1],[16496,28,1],[16496,33,1],[16496,40,2],
[16496,43,1],[16496,62,1],[16496,63,1],[16496,68,1],[16496,69,1],
[16496,70,2],[16496,72,2],[16496,75,3],[16497,24,1],[16497,26,1],
[16498,2,1],[16498,31,3],[16499,51,11],[16500,28,1],[16500,32,8],
[16501,2,1],[16501,23,1],[16502,1,1],[16502,12,1],[16502,15,1],
[16502,17,1],[16502,29,1],[16502,34,2],[16502,41,1],[16502,46,1],

[16502,54,1],[16502,65,1],[16502,71,2],[16502,77,1],[16503,28,3],
[16504,16,1],[16504,26,1],[16504,28,1],[16504,44,1],[16504,68,1],
[16505,62,3],[16506,28,7],[16507,22,2],[16507,23,2],[16507,24,1],
[16507,27,1],[16507,28,1],[16507,38,1],[16508,56,1],[16508,69,1],
[16509,2,11],[16509,3,16],[16509,4,22],[16509,5,15],[16509,6,1],
[16509,8,1],[16509,16,7],[16509,19,1],[16509,21,5],[16509,22,13],
[16509,23,21],[16509,24,33],[16509,25,15],[16509,26,20],[16509,27,30],
[16509,28,84],[16509,31,32],[16509,32,14],[16509,33,16],[16509,35,24],
[16509,38,21],[16509,39,1],[16509,40,11],[16509,42,9],[16509,43,9],
[16509,44,28],[16509,45,2],[16509,47,8],[16509,48,6],[16509,50,24],
[16509,52,6],[16509,56,10],[16509,61,1],[16509,62,15],[16509,63,13],
[16509,66,29],[16509,68,10],[16509,69,2],[16509,70,17],[16509,72,8],
[16509,74,17],[16509,75,18],[16509,76,19],[16509,79,1],[16510,21,1],
[16510,22,8],[16510,25,1],[16510,26,1],[16510,28,1],[16510,33,3],
[16510,35,1],[16510,43,1],[16510,50,1],[16510,51,1],[16510,64,1],
[16510,70,1],[16510,74,1],[16511,10,2],[16511,13,1],[16511,19,2],
[16511,20,6],[16511,23,1],[16511,24,2],[16511,27,2],[16511,33,2],
[16511,38,1],[16511,39,1],[16511,44,1],[16511,58,1],[16511,78,1],
[16512,2,3],[16512,42,2],[16513,1,1],[16513,17,1],[16513,30,1],
[16514,2,1],[16514,30,1],[16514,42,3],[16514,43,1],[16514,47,1],
[16514,60,1],[16515,23,1],[16515,26,4],[16515,33,1],[16515,37,1],
[16515,43,1],[16516,28,3],[16517,26,1],[16517,32,2],[16518,7,4],
[16518,15,1],[16519,24,2],[16520,4,1],[16520,22,2],[16520,25,2],
[16520,27,7],[16520,28,5],[16520,30,4],[16520,33,12],[16520,35,24],
[16520,40,1],[16520,52,19],[16520,73,1],[16520,74,1],[16521,21,1],
[16521,24,1],[16521,30,1],[16521,32,1],[16522,25,1],[16522,32,3],
[16523,2,2],[16524,26,1],[16524,31,1],[16524,63,1],[16525,21,1],
[16525,25,4],[16525,26,1],[16526,2,1],[16526,24,1],[16526,26,1],
[16526,31,1],[16526,60,1],[16527,42,6],[16527,66,1],[16528,26,1],
[16528,28,4],[16528,33,1],[16529,2,2],[16529,3,4],[16529,4,79],
[16529,5,10],[16529,10,1],[16529,15,25],[16529,17,5],[16529,19,1],
[16529,21,1],[16529,22,6],[16529,23,10],[16529,24,5],[16529,25,5],
[16529,26,7],[16529,27,9],[16529,28,24],[16529,30,1],[16529,31,14],
[16529,32,5],[16529,33,2],[16529,35,2],[16529,38,87],[16529,39,1],
[16529,40,2],[16529,42,1],[16529,44,900],[16529,47,6],[16529,48,2],
[16529,50,88],[16529,51,3],[16529,52,2],[16529,53,8],[16529,55,26],
[16529,56,4],[16529,61,1],[16529,62,4],[16529,63,3],[16529,66,722],
[16529,68,1],[16529,69,2],[16529,70,5],[16529,72,3],[16529,74,5],
[16529,75,2],[16529,76,2],[16530,26,2],[16530,27,1],[16530,28,1],
[16530,53,1],[16531,35,2],[16532,0,1],[16532,1,1],[16532,7,1],
[16532,8,1],[16532,9,2],[16532,10,2],[16532,11,1],[16532,12,3],
[16532,13,2],[16532,15,7],[16532,17,2],[16532,18,1],[16532,19,2],
[16532,29,1],[16532,37,1],[16532,38,1],[16532,39,1],[16532,41,1],
[16532,45,2],[16532,46,1],[16532,53,1],[16532,54,3],[16532,55,3],
[16532,57,3],[16532,58,1],[16532,59,2],[16532,63,3],[16532,65,4],
[16532,70,1],[16532,71,1],[16532,73,1],[16532,74,4],[16532,75,3],
[16532,76,2],[16532,77,1],[16532,78,1],[16533,10,1],[16533,15,4],
[16533,17,2],[16533,44,2],[16533,53,2],[16533,55,2],[16533,66,1],
[16534,5,1],[16534,15,1],[16534,17,29],[16534,19,3],[16534,20,1],
[16534,25,1],[16534,27,1],[16534,28,3],[16534,34,1],[16534,45,1],

[16534,49,1],[16534,50,1],[16534,53,30],[16534,55,1],[16534,57,3],
[16534,58,1],[16534,59,1],[16534,65,2],[16534,74,1],[16535,2,1],
[16535,4,2],[16535,10,1],[16535,15,7],[16535,17,8],[16535,28,1],
[16535,41,1],[16535,44,1],[16535,49,1],[16535,52,1],[16535,53,13],
[16535,55,8],[16535,56,1],[16535,62,1],[16535,65,1],[16535,66,1],
[16536,43,1],[16536,47,1],[16537,24,2],[16537,47,1],[16538,17,1],
[16538,59,1],[16539,24,1],[16539,27,2],[16540,68,2],[16541,23,1],
[16541,33,1],[16542,22,1],[16542,35,2],[16543,1,1],[16543,13,2],
[16543,20,1],[16544,23,2],[16544,27,3],[16544,43,1],[16545,2,2],
[16545,31,2],[16545,32,1],[16545,62,1],[16546,27,1],[16546,28,1],
[16546,35,1],[16546,42,1],[16547,2,1],[16547,47,1],[16548,1,3],
[16548,7,1],[16548,8,1],[16548,9,4],[16548,10,2],[16548,11,2],
[16548,12,3],[16548,14,6],[16548,16,2],[16548,17,2],[16548,18,2],
[16548,19,2],[16548,20,1],[16548,29,1],[16548,30,1],[16548,34,1],
[16548,36,1],[16548,37,1],[16548,39,1],[16548,41,1],[16548,45,2],
[16548,49,1],[16548,53,1],[16548,54,2],[16548,55,1],[16548,57,1],
[16548,58,2],[16548,59,2],[16548,65,2],[16548,67,2],[16548,77,3],
[16549,25,1],[16549,32,1],[16550,23,2],[16551,26,2],[16552,11,2],
[16552,39,1],[16552,41,1],[16552,60,1],[16552,73,27],[16552,77,1],
[16553,28,2],[16554,35,1],[16554,42,1],[16555,21,1],[16555,26,1],
[16556,5,2],[16556,21,1],[16556,22,2],[16556,23,5],[16556,24,1],
[16556,26,1],[16556,27,2],[16556,33,3],[16556,35,1],[16556,43,3],
[16556,47,1],[16557,2,1],[16557,4,2],[16557,26,1],[16557,28,2],
[16557,35,1],[16557,44,4],[16557,50,4],[16557,62,1],[16557,66,1],
[16557,76,1],[16558,2,1],[16558,4,14],[16558,5,1],[16558,15,12],
[16558,22,1],[16558,24,2],[16558,25,1],[16558,26,2],[16558,31,5],
[16558,38,6],[16558,40,1],[16558,42,1],[16558,43,1],[16558,44,9],
[16558,50,8],[16558,53,1],[16558,55,3],[16558,56,2],[16558,63,1],
[16558,66,15],[16558,70,1],[16558,74,1],[16558,75,2],[16558,76,2],
[16559,27,2],[16559,35,2],[16560,26,1],[16560,35,1],[16561,1,3],
[16561,7,1],[16561,8,2],[16561,9,8],[16561,10,8],[16561,11,7],
[16561,12,7],[16561,13,2],[16561,14,2],[16561,15,2],[16561,16,8],
[16561,17,1],[16561,18,1],[16561,19,4],[16561,20,3],[16561,30,2],
[16561,34,2],[16561,37,5],[16561,39,3],[16561,41,3],[16561,45,7],
[16561,46,4],[16561,49,4],[16561,53,1],[16561,54,3],[16561,55,8],
[16561,57,5],[16561,58,5],[16561,59,4],[16561,60,1],[16561,64,2],
[16561,65,4],[16561,67,5],[16561,71,3],[16561,73,3],[16561,77,8],
[16561,78,3],[16562,22,2],[16562,27,3],[16562,32,2],[16562,42,1],
[16563,24,1],[16563,25,1],[16563,27,1],[16563,31,1],[16564,8,3],
[16564,21,1],[16564,27,1],[16564,30,1],[16564,39,1],[16564,43,1],
[16564,55,1],[16565,13,1],[16565,21,1],[16565,27,3],[16565,28,1],
[16565,35,1],[16565,36,2],[16565,47,2],[16566,5,1],[16566,20,1],
[16566,26,1],[16567,24,2],[16567,28,7],[16568,1,1],[16568,5,1],
[16568,22,1],[16568,23,4],[16568,26,1],[16568,27,7],[16568,28,3],
[16568,29,1],[16568,33,1],[16568,37,1],[16568,42,1],[16568,46,1],
[16569,2,1],[16569,4,1],[16569,5,4],[16569,9,1],[16569,10,1],
[16569,15,3],[16569,20,1],[16569,21,1],[16569,22,5],[16569,24,1],
[16569,27,3],[16569,31,1],[16569,32,1],[16569,40,1],[16569,44,3],
[16569,47,1],[16569,50,1],[16569,52,1],[16569,55,2],[16569,66,2],
[16569,68,1],[16569,70,2],[16569,76,2],[16570,11,1],[16570,20,1],

[16571,8,2],[16571,15,1],[16571,16,4],[16571,37,1],[16571,54,1],
[16572,23,1],[16572,25,3],[16572,26,1],[16572,27,4],[16572,33,1],
[16572,35,2],[16573,28,1],[16573,56,1],[16574,32,3],[16575,8,2],
[16575,28,1],[16575,31,1],[16576,3,1],[16576,7,1],[16576,10,1],
[16576,20,2],[16576,23,1],[16576,25,2],[16576,26,1],[16576,27,11],
[16576,28,2],[16576,32,9],[16576,34,2],[16576,48,1],[16576,65,1],
[16576,66,1],[16577,3,1],[16577,26,1],[16577,31,1],[16578,36,1],
[16578,77,1],[16579,2,1],[16579,22,1],[16579,23,1],[16579,24,1],
[16579,28,1],[16580,20,1],[16580,22,4],[16580,23,1],[16580,35,1],
[16580,41,1],[16581,4,1],[16581,5,1],[16581,24,2],[16581,28,1],
[16581,31,3],[16581,42,1],[16582,2,3],[16583,35,3],[16583,38,1],
[16584,23,1],[16584,28,1],[16584,35,1],[16584,43,1],[16585,2,3],
[16586,2,1],[16586,5,2],[16586,22,2],[16586,24,3],[16586,25,1],
[16586,27,3],[16586,28,1],[16586,33,1],[16586,40,1],[16586,43,1],
[16586,47,1],[16587,44,1],[16587,66,1],[16588,5,2],[16588,22,1],
[16588,24,1],[16588,27,2],[16589,4,1],[16589,44,1],[16589,66,1],
[16590,5,1],[16590,20,1],[16590,23,1],[16590,25,2],[16590,26,1],
[16590,27,1],[16590,28,2],[16590,31,1],[16590,32,1],[16590,42,1],
[16590,43,2],[16590,62,2],[16591,2,3],[16591,5,1],[16591,24,1],
[16591,26,1],[16591,28,1],[16592,12,13],[16592,21,1],[16593,1,1],
[16593,9,1],[16593,19,1],[16593,23,2],[16593,38,1],[16593,46,1],
[16593,49,1],[16593,50,1],[16593,54,1],[16593,62,3],[16593,65,2],
[16593,73,1],[16594,10,1],[16594,19,1],[16594,39,1],[16594,54,1],
[16595,17,1],[16595,53,1],[16595,55,2],[16596,4,1],[16596,25,2],
[16596,31,1],[16596,38,1],[16596,40,1],[16597,22,1],[16597,23,3],
[16597,27,3],[16597,28,1],[16597,32,1],[16597,35,1],[16598,24,1],
[16598,32,1],[16599,15,1],[16599,46,1],[16600,23,1],[16600,68,1],
[16600,75,1],[16601,44,2],[16602,10,1],[16602,23,1],[16602,26,1],
[16602,35,1],[16602,55,1],[16602,60,1],[16602,62,1],[16602,68,141],
[16602,70,6],[16602,72,10],[16602,76,1],[16603,2,2],[16603,5,3],
[16603,21,2],[16603,22,1],[16603,23,1],[16603,27,3],[16603,32,2],
[16603,35,2],[16603,38,1],[16603,42,1],[16604,28,2],[16604,69,1],
[16605,2,2],[16605,3,1],[16605,21,1],[16605,23,2],[16605,24,1],
[16605,26,2],[16605,27,12],[16605,32,3],[16605,35,14],[16605,42,26],
[16605,48,1],[16605,76,1],[16606,3,1],[16606,15,1],[16606,22,1],
[16606,40,1],[16606,47,1],[16606,72,1],[16607,3,5],[16607,6,5],
[16607,23,2],[16607,24,2],[16607,56,19],[16608,3,1],[16608,67,1],
[16609,26,2],[16609,35,1],[16609,50,1],[16610,0,1],[16610,1,9],
[16610,5,2],[16610,9,3],[16610,10,6],[16610,11,35],[16610,12,15],
[16610,13,12],[16610,15,5],[16610,16,2],[16610,17,1],[16610,18,1],
[16610,19,1],[16610,20,10],[16610,24,1],[16610,28,2],[16610,29,23],
[16610,30,3],[16610,32,1],[16610,34,2],[16610,35,2],[16610,36,3],
[16610,39,2],[16610,41,1],[16610,43,1],[16610,53,2],[16610,54,4],
[16610,55,4],[16610,57,2],[16610,58,2],[16610,60,1],[16610,64,1],
[16610,65,3],[16610,68,1],[16610,72,1],[16610,73,1],[16610,77,6],
[16611,2,8],[16611,3,1],[16611,5,2],[16611,22,2],[16611,31,4],
[16611,38,2],[16611,42,3],[16611,47,1],[16612,28,15],[16612,69,1],
[16613,0,1],[16613,9,1],[16613,29,1],[16614,8,1],[16614,28,1],
[16615,23,1],[16615,24,6],[16615,27,1],[16615,28,1],[16615,32,1],
[16615,33,1],[16615,39,1],[16615,56,3],[16615,74,1],[16616,23,1],

[16616,27,1],[16616,32,1],[16617,3,1],[16617,35,1],[16618,27,1],
[16618,28,1],[16619,25,3],[16620,4,2],[16620,22,1],[16620,43,1],
[16620,44,2],[16620,66,2],[16621,24,1],[16621,25,4],[16621,33,1],
[16621,35,1],[16622,29,1],[16622,32,6],[16623,22,1],[16623,23,3],
[16623,24,1],[16623,27,3],[16623,28,1],[16623,35,1],[16623,37,1],
[16624,33,1],[16624,40,1],[16624,45,1],[16624,69,1],[16624,76,1],
[16625,22,1],[16625,27,1],[16626,12,1],[16626,14,1],[16626,34,1],
[16627,19,1],[16627,23,1],[16627,24,2],[16627,26,2],[16627,27,1],
[16627,37,1],[16627,42,3],[16627,46,1],[16627,59,2],[16627,62,9],
[16628,27,1],[16628,35,1],[16628,42,1],[16629,23,1],[16629,40,1],
[16630,2,1],[16630,28,5],[16631,32,10],[16632,74,2],[16633,56,1],
[16633,68,1],[16633,69,1],[16634,2,3],[16634,7,1],[16634,9,1],
[16634,23,4],[16634,30,3],[16634,31,2],[16634,42,28],[16634,47,3],
[16635,13,1],[16635,18,1],[16636,2,2],[16636,9,1],[16636,17,2],
[16636,50,1],[16636,53,1],[16636,65,1],[16636,72,1],[16636,74,1],
[16637,15,2],[16638,3,3],[16638,26,1],[16638,47,1],[16639,3,2],
[16639,5,1],[16639,6,6],[16639,8,1],[16639,21,2],[16639,22,1],
[16639,23,3],[16639,24,1],[16639,25,7],[16639,26,5],[16639,27,4],
[16639,28,1],[16639,31,2],[16639,32,2],[16639,35,1],[16639,47,4],
[16639,56,3],[16640,1,1],[16640,8,3],[16640,29,1],[16641,1,1],
[16641,10,1],[16641,12,1],[16641,37,1],[16641,46,1],[16641,57,1],
[16641,64,2],[16641,65,1],[16642,28,2],[16642,40,1],[16642,62,1],
[16643,23,1],[16643,27,2],[16643,47,1],[16643,56,1],[16643,69,1],
[16643,71,1],[16644,19,1],[16644,36,1],[16645,22,2],[16645,24,1],
[16645,27,1],[16645,28,3],[16645,30,4],[16645,35,7],[16645,38,1],
[16645,40,1],[16645,41,1],[16645,46,1],[16645,52,11],[16645,63,1],
[16645,69,1],[16646,2,4],[16646,27,1],[16646,28,1],[16646,34,1],
[16647,74,1],[16647,76,1],[16648,4,9],[16648,10,1],[16648,25,1],
[16648,28,1],[16648,29,1],[16648,31,1],[16648,32,1],[16648,38,10],
[16648,42,1],[16648,44,14],[16648,49,1],[16648,50,19],[16648,53,1],
[16648,56,1],[16648,64,1],[16648,65,1],[16648,66,22],[16648,72,1],
[16648,76,1],[16649,24,5],[16649,56,2],[16650,67,1],[16650,77,1],
[16651,33,1],[16651,72,1],[16652,35,3],[16653,21,2],[16653,23,1],
[16653,30,1],[16653,35,1],[16654,20,1],[16654,23,1],[16654,26,1],
[16654,39,1],[16655,28,69],[16655,72,1],[16656,26,1],[16656,32,1],
[16656,35,1],[16657,2,3],[16657,5,4],[16657,23,2],[16657,24,1],
[16657,28,2],[16657,33,3],[16657,37,1],[16657,41,1],[16657,43,1],
[16657,45,1],[16657,47,3],[16657,55,1],[16657,56,4],[16657,60,1],
[16657,66,1],[16658,2,1],[16658,21,1],[16659,10,1],[16659,34,4],
[16659,46,1],[16660,13,1],[16660,53,1],[16661,21,1],[16661,25,1],
[16661,27,1],[16661,28,1],[16662,3,1],[16662,62,1],[16663,25,2],
[16663,28,1],[16663,35,1],[16663,42,1],[16663,51,2],[16664,24,3],
[16664,59,1],[16664,70,1],[16665,28,1],[16665,70,1],[16666,23,1],
[16666,25,1],[16666,28,2],[16667,4,2],[16668,28,3],[16668,62,19],
[16669,23,4],[16670,1,6],[16670,12,2],[16670,25,1],[16670,34,1],
[16671,5,1],[16671,20,8],[16671,22,1],[16671,23,1],[16671,24,2],
[16671,26,3],[16671,27,5],[16671,28,2],[16671,32,1],[16671,38,2],
[16671,39,2],[16671,56,1],[16671,60,1],[16671,65,1],[16671,73,1],
[16672,5,1],[16672,25,2],[16672,28,14],[16672,40,2],[16672,44,1],
[16672,51,1],[16672,63,1],[16672,68,1],[16672,69,1],[16672,70,6],

[16672,74,1],[16672,75,2],[16672,76,2],[16673,9,1],[16673,24,1],
[16673,33,1],[16673,37,1],[16674,7,1],[16674,9,1],[16674,11,1],
[16674,12,3],[16674,13,1],[16674,14,1],[16674,16,2],[16674,17,1],
[16674,18,1],[16674,27,4],[16674,28,1],[16674,39,4],[16674,43,1],
[16674,45,1],[16674,50,1],[16674,57,1],[16674,59,1],[16674,77,1],
[16675,1,1],[16675,12,1],[16675,20,1],[16675,53,1],[16675,64,1],
[16675,65,1],[16675,71,1],[16676,27,1],[16676,47,2],[16677,2,1],
[16677,5,1],[16677,21,1],[16677,31,1],[16677,32,1],[16677,40,1],
[16677,42,1],[16677,62,1],[16678,1,14],[16678,2,1],[16678,3,9],
[16678,4,307],[16678,5,17],[16678,6,4],[16678,7,1],[16678,8,2],
[16678,9,7],[16678,10,5],[16678,11,3],[16678,12,1],[16678,13,3],
[16678,14,1],[16678,15,24],[16678,16,6],[16678,17,6],[16678,18,1],
[16678,19,5],[16678,20,26],[16678,21,5],[16678,22,9],[16678,23,12],
[16678,24,7],[16678,25,4],[16678,26,3],[16678,27,4],[16678,28,24],
[16678,30,2],[16678,31,4],[16678,32,7],[16678,33,4],[16678,34,1],
[16678,35,13],[16678,37,1],[16678,38,28],[16678,39,2],[16678,40,3],
[16678,41,1],[16678,42,4],[16678,43,2],[16678,44,224],[16678,45,2],
[16678,47,10],[16678,48,5],[16678,49,2],[16678,50,356],[16678,51,7],
[16678,53,4],[16678,55,19],[16678,56,8],[16678,57,2],[16678,58,4],
[16678,59,5],[16678,60,1],[16678,61,1],[16678,62,2],[16678,63,5],
[16678,64,1],[16678,65,3],[16678,66,171],[16678,67,2],[16678,68,3],
[16678,69,2],[16678,70,18],[16678,71,4],[16678,72,4],[16678,73,1],
[16678,74,22],[16678,75,5],[16678,76,5],[16678,77,1],[16678,78,1],
[16679,16,1],[16679,21,2],[16679,22,1],[16679,77,1],[16680,45,1],
[16680,65,1],[16681,8,3],[16681,11,1],[16681,22,1],[16681,28,1],
[16681,31,2],[16681,62,1],[16682,25,1],[16682,28,1],[16682,34,1],
[16682,59,1],[16682,78,1],[16683,27,1],[16683,39,1],[16684,22,1],
[16684,23,2],[16684,24,1],[16684,25,4],[16684,26,1],[16684,27,2],
[16684,35,5],[16684,40,1],[16684,43,5],[16684,62,1],[16685,9,1],
[16685,11,1],[16686,28,2],[16687,21,2],[16687,26,1],[16687,31,2],
[16687,32,6],[16688,39,2],[16689,28,5],[16689,31,10],[16689,32,1],
[16689,38,1],[16690,29,2],[16690,39,2],[16691,9,1],[16691,13,8],
[16691,16,1],[16691,23,4],[16691,26,1],[16691,28,2],[16691,40,1],
[16691,56,1],[16691,78,2],[16692,28,1],[16692,40,1],[16693,27,1],
[16693,66,1],[16694,1,3],[16694,10,1],[16694,12,1],[16694,13,2],
[16694,15,5],[16694,16,2],[16694,20,1],[16694,34,1],[16694,36,1],
[16694,37,1],[16694,41,1],[16694,49,1],[16694,53,1],[16694,55,2],
[16694,77,1],[16695,25,1],[16695,28,1],[16695,33,1],[16695,47,1],
[16695,50,1],[16696,2,1],[16696,20,1],[16696,22,1],[16696,27,1],
[16696,32,1],[16696,34,2],[16697,9,1],[16697,18,1],[16698,55,1],
[16698,77,1],[16699,2,1],[16699,24,1],[16699,42,2],[16700,24,1],
[16700,63,3],[16700,76,1],[16701,2,7],[16701,28,1],[16701,50,1],
[16702,28,3],[16703,0,2],[16703,57,1],[16704,25,1],[16704,31,2],
[16704,33,5],[16704,39,3],[16704,42,2],[16705,3,1],[16705,22,1],
[16705,23,1],[16705,32,10],[16705,48,2],[16706,1,2],[16706,29,1],
[16707,28,8],[16707,63,3],[16708,2,1],[16708,3,1],[16708,20,1],
[16708,23,1],[16708,26,3],[16708,27,2],[16708,28,5],[16708,32,1],
[16709,15,1],[16709,17,1],[16709,55,1],[16710,4,2],[16710,26,1],
[16710,28,2],[16710,32,1],[16710,44,2],[16710,66,1],[16711,25,2],
[16711,28,1],[16711,33,10],[16712,26,1],[16712,32,1],[16712,35,1],

[16712,56,18],[16712,74,1],[16712,76,2],[16713,1,4],[16713,8,2],
[16713,13,5],[16713,17,1],[16713,36,1],[16714,27,4],[16714,35,1],
[16714,50,1],[16715,25,1],[16715,28,1],[16716,27,1],[16716,28,1],
[16716,61,1],[16717,20,1],[16717,29,3],[16718,24,1],[16718,27,1],
[16718,32,2],[16718,33,1],[16718,43,1],[16719,34,1],[16719,53,1],
[16720,1,1],[16720,21,1],[16721,63,1],[16721,76,1],[16722,4,1],
[16722,21,1],[16722,22,4],[16722,24,4],[16722,32,1],[16722,74,1],
[16723,26,1],[16723,27,1],[16724,6,1],[16724,32,1],[16725,43,1],
[16725,62,1],[16726,28,1],[16726,66,1],[16727,3,2],[16727,5,1],
[16727,16,1],[16727,23,13],[16727,24,5],[16727,25,2],[16727,26,1],
[16727,27,1],[16727,28,1],[16727,31,5],[16727,32,7],[16727,33,2],
[16727,35,5],[16727,42,1],[16727,43,2],[16727,48,1],[16727,52,2],
[16727,56,1],[16727,74,1],[16727,78,1],[16728,1,1],[16728,6,1],
[16728,13,1],[16728,19,1],[16728,24,1],[16728,28,1],[16728,41,1],
[16729,29,2],[16730,23,1],[16730,50,1],[16730,56,1],[16730,66,1],
[16731,24,3],[16732,29,2],[16732,58,1],[16733,0,1],[16733,8,1],
[16733,12,3],[16733,13,2],[16733,20,1],[16733,28,1],[16733,29,1],
[16733,36,1],[16733,39,1],[16733,42,1],[16734,63,2],[16734,76,3],
[16735,6,4],[16735,22,1],[16735,27,1],[16735,35,1],[16736,28,1],
[16736,62,1],[16737,6,2],[16737,23,5],[16737,24,2],[16737,25,5],
[16737,26,3],[16737,28,1],[16737,32,1],[16737,33,2],[16737,43,3],
[16737,47,3],[16738,1,1],[16738,7,1],[16738,9,2],[16738,10,2],
[16738,30,1],[16738,45,1],[16738,53,1],[16738,58,2],[16738,64,1],
[16738,71,1],[16738,76,1],[16739,2,1],[16739,26,1],[16739,28,1],
[16739,32,1],[16739,43,1],[16740,2,4],[16740,69,1],[16741,23,12],
[16741,24,1],[16742,4,2],[16742,22,1],[16742,24,4],[16742,25,3],
[16742,26,1],[16742,27,5],[16742,43,1],[16742,50,1],[16742,53,1],
[16742,62,1],[16743,1,1],[16743,10,1],[16743,16,1],[16743,54,1],
[16744,40,1],[16744,48,2],[16745,22,4],[16746,24,2],[16747,42,3],
[16748,22,1],[16748,32,1],[16749,13,1],[16749,15,3],[16749,17,1],
[16749,37,1],[16749,55,1],[16749,59,1],[16750,21,1],[16750,24,1],
[16751,43,1],[16751,69,1],[16752,23,4],[16753,4,1],[16753,5,1],
[16753,28,1],[16753,50,2],[16754,13,1],[16754,15,1],[16754,19,1],
[16754,41,1],[16754,53,1],[16754,59,1],[16755,10,1],[16755,21,1],
[16755,23,1],[16755,25,1],[16755,28,2],[16756,1,3],[16756,2,1],
[16756,20,1],[16756,24,1],[16756,45,1],[16756,55,1],[16756,64,1],
[16757,23,1],[16757,32,1],[16758,23,7],[16758,24,3],[16758,27,1],
[16758,28,1],[16758,30,2],[16758,31,1],[16758,32,1],[16758,43,2],
[16758,47,1],[16758,48,3],[16759,0,1],[16759,7,1],[16759,8,1],
[16759,9,2],[16759,10,1],[16759,12,1],[16759,15,1],[16759,16,1],
[16759,17,1],[16759,19,1],[16759,29,2],[16759,39,1],[16759,45,3],
[16759,65,1],[16759,77,1],[16760,2,2],[16760,29,1],[16761,28,9],
[16762,23,1],[16762,24,3],[16762,56,2],[16763,10,1],[16763,37,1],
[16763,59,1],[16763,60,1],[16763,71,1],[16764,28,1],[16764,32,16],
[16765,28,2],[16766,0,1],[16766,7,1],[16767,59,1],[16767,67,1],
[16768,4,1],[16768,15,2],[16768,17,1],[16768,55,1],[16768,66,1],
[16769,15,5],[16769,53,2],[16770,5,1],[16770,8,3],[16771,0,1],
[16771,25,2],[16771,26,1],[16771,31,1],[16771,33,1],[16771,42,1],
[16771,43,1],[16771,47,1],[16771,56,2],[16771,64,1],[16772,1,1],
[16772,10,1],[16772,15,1],[16772,20,1],[16772,27,1],[16772,35,1],

[16772,62,3],[16773,2,1],[16773,25,1],[16773,28,1],[16773,43,1],
[16773,70,1],[16773,74,1],[16774,23,1],[16774,24,1],[16774,28,1],
[16774,56,1],[16775,5,1],[16775,6,1],[16775,23,2],[16775,24,14],
[16775,27,1],[16775,28,1],[16775,31,5],[16775,33,1],[16775,42,5],
[16775,47,1],[16775,52,2],[16775,70,2],[16776,17,1],[16776,39,1],
[16776,53,2],[16777,9,1],[16777,19,1],[16777,28,1],[16777,53,1],
[16778,56,5],[16779,25,1],[16779,31,1],[16779,79,1],[16780,15,1],
[16780,33,1],[16780,48,1],[16780,55,2],[16781,13,1],[16781,16,1],
[16781,37,1],[16782,23,3],[16783,5,2],[16783,23,1],[16783,24,18],
[16783,27,1],[16783,32,2],[16783,33,2],[16783,42,2],[16783,48,1],
[16783,56,9],[16783,68,1],[16783,76,1],[16784,55,3],[16785,28,1],
[16785,48,1],[16786,28,1],[16786,76,1],[16787,35,1],[16787,47,1],
[16788,0,1],[16788,1,163],[16788,2,3],[16788,4,4],[16788,5,5],
[16788,7,3],[16788,9,6],[16788,10,1],[16788,11,4],[16788,12,151],
[16788,13,118],[16788,14,1],[16788,15,7],[16788,16,2],[16788,17,1],
[16788,18,3],[16788,19,2],[16788,20,9],[16788,21,5],[16788,22,3],
[16788,23,5],[16788,24,2],[16788,25,34],[16788,26,15],[16788,27,4],
[16788,28,10],[16788,29,35],[16788,30,2],[16788,31,3],[16788,33,92],
[16788,34,1],[16788,35,1],[16788,36,2],[16788,37,6],[16788,38,8],
[16788,39,7],[16788,41,2],[16788,42,9],[16788,43,8],[16788,44,3],
[16788,46,1],[16788,47,2],[16788,48,1],[16788,49,3],[16788,50,3],
[16788,52,1],[16788,53,5],[16788,55,3],[16788,56,3],[16788,57,1],
[16788,58,2],[16788,59,1],[16788,60,2],[16788,61,1],[16788,63,3],
[16788,64,1],[16788,65,3],[16788,66,1],[16788,67,3],[16788,69,1],
[16788,72,1],[16788,75,4],[16788,76,2],[16788,77,3],[16788,78,6],
[16789,28,4],[16790,12,1],[16790,55,1],[16791,28,1],[16791,40,1],
[16792,3,2],[16792,26,1],[16793,5,1],[16793,8,1],[16793,28,2],
[16793,44,1],[16794,3,1],[16794,23,1],[16794,26,1],[16795,35,3],
[16796,55,1],[16796,76,1],[16797,14,1],[16797,23,1],[16797,28,1],
[16797,38,1],[16797,40,10],[16797,63,1],[16797,65,1],[16797,68,23],
[16797,69,5],[16797,70,10],[16797,72,13],[16797,75,1],[16797,76,1],
[16797,77,1],[16798,20,5],[16798,25,2],[16798,27,2],[16798,42,1],
[16799,23,1],[16799,37,1],[16800,4,1],[16800,24,1],[16800,28,1],
[16800,50,1],[16800,51,1],[16800,53,2],[16801,26,1],[16801,56,1],
[16802,2,4],[16802,3,10],[16802,8,1],[16802,9,1],[16802,21,3],
[16802,23,3],[16802,24,2],[16802,26,6],[16802,28,5],[16802,30,13],
[16802,31,30],[16802,32,4],[16802,33,1],[16802,35,1],[16802,36,1],
[16802,37,1],[16802,42,42],[16802,43,3],[16802,46,1],[16802,47,11],
[16802,48,1],[16802,51,1],[16802,53,1],[16802,57,2],[16802,58,1],
[16802,69,1],[16802,74,1],[16803,2,1],[16803,13,1],[16804,20,1],
[16804,31,1],[16804,76,1],[16805,9,2],[16805,13,1],[16805,28,6],
[16805,33,1],[16805,43,1],[16806,9,1],[16806,11,1],[16806,49,1],
[16807,15,3],[16807,17,2],[16807,20,1],[16807,34,1],[16807,53,2],
[16807,55,2],[16808,66,2],[16809,0,5],[16809,1,5],[16809,2,12],
[16809,8,3],[16809,9,3],[16809,12,11],[16809,13,8],[16809,15,1],
[16809,16,6],[16809,18,1],[16809,19,6],[16809,20,108],[16809,23,4],
[16809,24,8],[16809,26,1],[16809,27,3],[16809,28,3],[16809,29,2],
[16809,30,2],[16809,31,12],[16809,32,35],[16809,34,22],[16809,35,2],
[16809,36,1],[16809,39,6],[16809,42,4],[16809,45,1],[16809,46,2],
[16809,49,1],[16809,54,1],[16809,55,2],[16809,57,1],[16809,59,3],

[16809,60,1],[16809,71,1],[16809,72,1],[16809,77,1],[16810,1,2],
[16810,8,1],[16810,10,1],[16810,11,1],[16810,18,1],[16810,19,1],
[16810,20,1],[16810,58,2],[16810,65,1],[16810,73,1],[16810,77,1],
[16811,21,3],[16812,1,3],[16812,20,1],[16812,22,2],[16812,23,1],
[16812,43,1],[16813,4,1],[16813,17,1],[16813,28,1],[16814,20,1],
[16814,23,1],[16814,28,1],[16814,42,1],[16814,43,1],[16815,28,1],
[16815,62,1],[16816,1,1],[16816,20,5],[16816,23,1],[16816,24,1],
[16816,27,1],[16817,2,2],[16818,2,1],[16818,3,1],[16818,13,1],
[16818,15,2],[16818,17,1],[16818,20,4],[16818,22,2],[16818,23,1],
[16818,25,1],[16818,27,1],[16818,44,1],[16818,47,3],[16818,69,1],
[16818,70,1],[16818,74,1],[16819,2,2],[16819,5,4],[16819,27,1],
[16819,28,3],[16819,43,1],[16819,53,1],[16819,62,1],[16820,45,1],
[16820,53,1],[16820,65,1],[16820,73,1],[16821,42,2],[16822,2,1],
[16822,4,1],[16822,9,2],[16822,11,1],[16822,15,13],[16822,16,3],
[16822,17,6],[16822,18,1],[16822,19,1],[16822,20,1],[16822,28,5],
[16822,30,1],[16822,32,2],[16822,35,1],[16822,36,1],[16822,37,2],
[16822,38,2],[16822,40,1],[16822,45,2],[16822,49,1],[16822,50,1],
[16822,53,2],[16822,54,1],[16822,55,7],[16822,58,1],[16822,64,1],
[16822,65,2],[16822,73,1],[16822,75,1],[16822,76,2],[16823,1,3],
[16823,17,1],[16823,34,2],[16823,53,1],[16824,35,2],[16824,37,5],
[16824,47,1],[16824,63,1],[16825,1,2],[16825,10,1],[16825,12,1],
[16825,13,3],[16825,24,1],[16825,27,3],[16825,35,1],[16825,39,1],
[16825,43,1],[16825,44,1],[16825,56,2],[16825,58,1],[16825,59,1],
[16826,6,6],[16826,25,1],[16826,32,2],[16826,40,2],[16826,51,3],
[16826,63,1],[16826,66,1],[16826,68,7],[16826,70,2],[16826,72,2],
[16826,74,2],[16827,0,1],[16827,9,1],[16828,21,1],[16828,23,1],
[16829,2,1],[16829,3,1],[16829,77,1],[16830,28,53],[16830,63,2],
[16830,74,1],[16831,16,1],[16831,74,1],[16831,76,1],[16832,62,2],
[16833,42,2],[16833,43,5],[16834,20,3],[16834,34,1],[16835,8,1],
[16835,15,1],[16835,43,1],[16835,55,1],[16835,66,1],[16836,17,2],
[16836,19,1],[16837,61,1],[16837,62,14],[16838,3,1],[16838,27,1],
[16838,31,1],[16838,33,3],[16838,39,1],[16839,28,1],[16839,31,1],
[16840,2,3],[16841,1,1],[16841,6,1],[16841,10,1],[16841,12,1],
[16841,22,1],[16841,59,1],[16841,65,1],[16841,77,1],[16842,23,1],
[16842,46,1],[16843,0,1],[16843,24,1],[16843,27,1],[16843,28,1],
[16843,37,1],[16843,59,1],[16844,24,1],[16844,32,1],[16845,10,2],
[16845,12,1],[16845,29,1],[16845,37,1],[16845,39,1],[16846,2,1],
[16846,21,2],[16847,4,3],[16847,15,1],[16847,17,1],[16847,22,5],
[16847,24,1],[16847,27,1],[16847,31,1],[16847,44,3],[16847,50,4],
[16847,53,1],[16847,75,1],[16848,0,1],[16848,1,1],[16848,7,1],
[16848,11,1],[16848,13,3],[16848,15,1],[16848,17,1],[16848,18,1],
[16848,20,1],[16848,29,2],[16848,34,1],[16848,37,1],[16848,59,1],
[16848,60,1],[16849,9,2],[16849,20,1],[16850,34,2],[16851,1,18],
[16851,7,1],[16851,9,5],[16851,10,11],[16851,11,3],[16851,12,8],
[16851,13,10],[16851,14,5],[16851,15,15],[16851,16,5],[16851,17,4],
[16851,18,4],[16851,19,4],[16851,20,7],[16851,21,3],[16851,25,1],
[16851,26,2],[16851,27,9],[16851,28,1],[16851,30,3],[16851,31,2],
[16851,32,2],[16851,34,6],[16851,35,2],[16851,36,2],[16851,39,4],
[16851,40,1],[16851,41,1],[16851,42,3],[16851,45,7],[16851,46,5],
[16851,52,1],[16851,53,2],[16851,54,3],[16851,55,9],[16851,57,6],

[16851,58,4],[16851,59,6],[16851,60,1],[16851,62,2],[16851,64,1],
[16851,65,10],[16851,66,1],[16851,67,7],[16851,73,7],[16851,76,1],
[16851,77,4],[16851,78,1],[16852,2,1],[16852,43,2],[16853,21,5],
[16853,28,1],[16853,56,1],[16854,56,2],[16855,28,2],[16856,3,2],
[16856,5,2],[16856,25,1],[16856,34,1],[16856,35,2],[16856,76,1],
[16857,28,83],[16857,75,1],[16858,6,3],[16858,10,1],[16858,32,3],
[16858,56,1],[16859,31,1],[16859,32,1],[16859,74,1],[16860,10,1],
[16860,47,1],[16860,59,1],[16861,2,2],[16861,28,2],[16862,6,1],
[16862,39,1],[16862,53,1],[16863,1,2],[16864,8,1],[16864,9,1],
[16864,12,23],[16864,14,1],[16864,23,1],[16864,33,1],[16864,41,2],
[16864,71,1],[16865,4,1],[16865,5,1],[16865,6,2],[16865,8,1],
[16865,13,2],[16865,20,2],[16865,22,1],[16865,23,1],[16865,24,2],
[16865,34,1],[16865,51,1],[16865,56,3],[16865,68,1],[16865,70,1],
[16865,72,1],[16866,15,1],[16866,17,1],[16867,5,1],[16867,28,1],
[16868,24,1],[16868,47,1],[16869,20,2],[16869,26,1],[16869,31,3],
[16869,35,1],[16870,1,1],[16870,30,1],[16871,0,1],[16871,8,6],
[16871,10,1],[16871,11,1],[16871,12,2],[16871,13,2],[16871,19,1],
[16871,20,1],[16871,25,1],[16871,31,15],[16871,32,1],[16871,34,1],
[16871,35,1],[16871,39,1],[16871,46,1],[16871,54,1],[16871,58,1],
[16871,59,1],[16871,62,2],[16871,64,1],[16872,35,2],[16873,6,1],
[16873,68,4],[16873,69,1],[16874,28,2],[16875,2,2],[16875,26,1],
[16876,15,4],[16876,17,1],[16876,19,1],[16876,21,3],[16876,23,1],
[16876,27,1],[16876,33,2],[16876,36,1],[16876,37,1],[16876,42,1],
[16876,46,1],[16876,53,2],[16876,54,1],[16876,55,1],[16876,68,1],
[16877,17,1],[16877,25,1],[16877,34,1],[16878,4,1],[16878,5,1],
[16878,9,1],[16878,10,1],[16878,12,1],[16878,13,3],[16878,15,3],
[16878,20,2],[16878,21,5],[16878,24,1],[16878,30,2],[16878,33,1],
[16878,36,1],[16878,39,2],[16878,47,1],[16878,48,2],[16878,53,1],
[16878,54,1],[16878,55,2],[16878,56,1],[16878,57,1],[16878,58,1],
[16878,61,2],[16878,66,1],[16878,70,3],[16878,72,3],[16878,74,1],
[16878,75,1],[16879,20,1],[16879,27,1],[16880,3,1],[16880,21,16],
[16880,22,1],[16880,23,1],[16880,25,2],[16880,26,14],[16880,31,9],
[16880,33,4],[16880,43,3],[16880,48,2],[16881,0,11],[16881,1,624],
[16881,3,1],[16881,7,75],[16881,8,40],[16881,9,7],[16881,10,6],
[16881,12,1014],[16881,13,121],[16881,14,1],[16881,15,19],
[16881,16,26],[16881,17,29],[16881,18,2],[16881,19,5],[16881,20,38],
[16881,25,193],[16881,26,1],[16881,28,2],[16881,29,156],[16881,30,21],
[16881,31,37],[16881,32,1],[16881,34,47],[16881,36,23],[16881,37,3],
[16881,39,216],[16881,42,1],[16881,43,8],[16881,44,1],[16881,45,4],
[16881,46,1],[16881,47,1],[16881,49,1],[16881,50,2],[16881,52,2],
[16881,53,28],[16881,54,1],[16881,55,25],[16881,58,1],[16881,59,5],
[16881,62,2],[16881,66,1],[16881,70,1],[16881,71,2],[16881,73,12],
[16881,77,14],[16881,78,4],[16882,1,2],[16882,2,8],[16882,3,14],
[16882,4,3],[16882,5,42],[16882,6,1],[16882,7,1],[16882,9,1],
[16882,10,1],[16882,12,2],[16882,13,10],[16882,14,1],[16882,15,1],
[16882,16,1],[16882,17,2],[16882,18,2],[16882,19,1],[16882,20,92],
[16882,21,260],[16882,22,72],[16882,23,214],[16882,24,99],
[16882,25,53],[16882,26,319],[16882,27,674],[16882,28,103],
[16882,29,1],[16882,30,1],[16882,31,197],[16882,32,30],[16882,33,59],
[16882,34,2],[16882,35,401],[16882,36,1],[16882,37,1],[16882,38,5],

[16882,39,18],[16882,40,6],[16882,41,2],[16882,42,138],[16882,43,336],
[16882,44,2],[16882,45,3],[16882,47,11],[16882,48,6],[16882,49,2],
[16882,50,5],[16882,52,3],[16882,53,1],[16882,54,1],[16882,56,3],
[16882,59,15],[16882,62,2],[16882,63,3],[16882,65,1],[16882,66,6],
[16882,67,2],[16882,68,4],[16882,69,3],[16882,70,11],[16882,71,1],
[16882,72,8],[16882,74,8],[16882,75,1],[16882,76,3],[16882,78,10],
[16883,2,1],[16883,22,1],[16883,23,4],[16884,0,2],[16884,1,2],
[16884,2,1],[16884,5,1],[16884,8,4],[16884,9,7],[16884,10,7],
[16884,13,1],[16884,16,1],[16884,17,3],[16884,18,1],[16884,19,5],
[16884,20,2],[16884,22,76],[16884,24,3],[16884,25,42],[16884,27,65],
[16884,28,35],[16884,29,1],[16884,30,61],[16884,31,3],[16884,32,61],
[16884,33,161],[16884,34,1],[16884,35,144],[16884,37,2],[16884,38,7],
[16884,39,1],[16884,43,1],[16884,45,2],[16884,46,2],[16884,49,2],
[16884,52,412],[16884,53,3],[16884,54,2],[16884,57,3],[16884,58,1],
[16884,70,1],[16884,71,1],[16884,75,1],[16884,76,1],[16884,77,1],
[16885,2,4],[16885,23,1],[16885,24,4],[16885,26,1],[16885,31,1],
[16885,32,1],[16885,43,2],[16886,27,1],[16886,28,1],[16886,35,5],
[16886,38,1],[16887,28,1],[16887,47,1],[16888,22,3],[16888,27,3],
[16888,30,1],[16888,35,1],[16889,27,1],[16889,35,2],[16890,27,1],
[16890,28,1],[16891,0,1],[16891,12,1],[16891,27,1],[16891,28,1],
[16891,32,1],[16891,35,3],[16891,71,1],[16892,0,1],[16892,1,1],
[16892,3,1],[16892,4,16],[16892,5,2],[16892,7,1],[16892,8,1],
[16892,9,2],[16892,15,55],[16892,17,3],[16892,21,1],[16892,22,1],
[16892,23,1],[16892,24,1],[16892,25,5],[16892,26,1],[16892,27,3],
[16892,28,14],[16892,31,5],[16892,32,5],[16892,33,2],[16892,35,2],
[16892,37,2],[16892,38,3],[16892,39,1],[16892,40,2],[16892,42,2],
[16892,43,2],[16892,44,11],[16892,46,1],[16892,47,2],[16892,50,19],
[16892,51,1],[16892,53,5],[16892,55,30],[16892,56,2],[16892,62,2],
[16892,66,16],[16892,70,2],[16892,72,1],[16892,74,3],[16892,75,2],
[16892,76,1],[16893,5,1],[16893,28,1],[16893,52,1],[16894,1,1],
[16894,20,1],[16894,31,2],[16894,62,20],[16894,77,1],[16895,15,2],
[16895,55,1],[16896,20,2],[16896,24,1],[16896,27,1],[16897,6,1],
[16897,27,2],[16897,28,1],[16898,9,1],[16898,12,1],[16898,30,1],
[16898,40,1],[16898,43,1],[16898,55,1],[16898,58,1],[16898,60,1],
[16898,68,1],[16899,27,2],[16900,6,1],[16900,22,2],[16900,23,3],
[16900,24,2],[16900,27,1],[16900,35,1],[16901,24,1],[16901,35,1],
[16901,47,1],[16902,24,2],[16902,26,4],[16902,27,3],[16902,32,3],
[16902,42,1],[16902,43,19],[16902,44,1],[16902,56,1],[16902,72,1],
[16902,76,1],[16903,5,3],[16903,42,1],[16904,3,3],[16904,24,5],
[16904,28,1],[16904,47,1],[16904,56,8],[16905,5,1],[16905,27,1],
[16905,28,1],[16906,10,1],[16906,29,1],[16906,46,1],[16906,73,1],
[16906,77,1],[16907,28,2],[16908,2,1],[16908,25,1],[16908,27,1],
[16908,28,1],[16908,31,1],[16909,28,2],[16910,35,3],[16911,17,1],
[16911,66,1],[16912,5,2],[16912,27,1],[16912,35,1],[16912,43,1],
[16913,12,2],[16913,29,1],[16914,1,2],[16914,4,2],[16914,17,1],
[16914,23,1],[16914,30,1],[16914,32,1],[16914,42,1],[16914,50,1],
[16914,55,1],[16915,59,2],[16916,7,2],[16917,7,4],[16918,32,2],
[16919,1,1],[16919,39,1],[16920,51,2],[16920,70,12],[16921,1,8],
[16921,29,4],[16921,39,1],[16922,32,1],[16922,54,1],[16922,59,4],
[16922,60,1],[16923,4,10],[16923,5,2],[16923,28,2],[16923,32,1],

[16923,38,4],[16923,44,24],[16923,50,19],[16923,56,2],[16923,66,23],
[16924,24,1],[16924,43,1],[16925,1,1],[16925,3,1],[16925,25,1],
[16926,23,2],[16926,28,2],[16926,43,1],[16927,6,1],[16927,27,1],
[16927,32,1],[16928,66,2],[16929,2,32],[16929,31,1],[16930,23,1],
[16930,28,1],[16931,31,1],[16931,33,1],[16932,28,15],[16932,74,1],
[16933,2,1],[16933,3,2],[16933,5,3],[16933,10,1],[16933,15,4],
[16933,20,2],[16933,22,2],[16933,23,1],[16933,24,1],[16933,26,1],
[16933,27,2],[16933,38,1],[16933,40,1],[16933,42,1],[16933,43,3],
[16933,50,1],[16933,51,1],[16933,55,1],[16933,56,1],[16933,62,1],
[16933,67,1],[16934,56,2],[16935,28,15],[16935,42,2],[16935,44,1],
[16936,4,1],[16936,5,1],[16936,16,1],[16936,22,2],[16936,32,1],
[16936,38,2],[16936,44,8],[16936,50,2],[16936,63,1],[16936,66,19],
[16936,68,1],[16937,3,2],[16938,5,2],[16938,52,1],[16939,4,1],
[16939,5,1],[16939,7,1],[16939,17,5],[16939,50,1],[16939,53,1],
[16939,55,1],[16940,24,1],[16940,26,1],[16941,22,2],[16941,42,1],
[16942,21,1],[16942,22,1],[16942,23,5],[16942,26,1],[16942,27,1],
[16942,31,2],[16942,32,4],[16943,26,2],[16943,31,1],[16944,44,1],
[16944,53,1],[16945,13,2],[16945,18,1],[16945,26,1],[16946,56,4],
[16947,5,1],[16947,48,6],[16948,4,1],[16948,37,1],[16948,44,2],
[16948,46,1],[16948,50,1],[16948,53,1],[16948,66,3],[16949,3,1],
[16949,21,1],[16949,22,1],[16949,23,1],[16949,27,1],[16949,37,2],
[16949,42,1],[16949,44,1],[16949,54,1],[16949,78,2],[16950,2,1],
[16950,8,2],[16950,24,5],[16950,30,1],[16950,33,1],[16950,34,1],
[16950,59,1],[16950,60,2],[16950,69,1],[16951,1,2],[16951,17,1],
[16951,20,1],[16951,29,1],[16951,34,1],[16951,39,7],[16952,29,2],
[16953,23,1],[16953,24,2],[16953,27,3],[16953,28,2],[16953,33,2],
[16953,35,2],[16953,42,1],[16953,48,1],[16954,35,1],[16954,56,6],
[16955,55,1],[16955,66,1],[16956,2,1],[16956,10,2],[16956,12,2],
[16956,13,2],[16956,15,2],[16956,17,1],[16956,20,1],[16956,28,1],
[16956,29,1],[16956,35,1],[16956,36,1],[16956,44,1],[16956,48,1],
[16956,51,1],[16956,57,1],[16956,66,1],[16957,3,1],[16957,21,1],
[16958,37,3],[16959,1,1],[16959,2,4],[16959,5,5],[16959,6,5],
[16959,7,1],[16959,9,1],[16959,13,4],[16959,15,2],[16959,20,8],
[16959,21,4],[16959,22,4],[16959,23,14],[16959,24,5],[16959,26,7],
[16959,27,11],[16959,28,1],[16959,30,1],[16959,31,4],[16959,32,10],
[16959,33,2],[16959,34,1],[16959,35,3],[16959,39,2],[16959,40,1],
[16959,42,9],[16959,43,9],[16959,47,2],[16959,50,2],[16959,51,4],
[16959,57,1],[16959,60,1],[16959,62,3],[16959,66,1],[16959,67,1],
[16959,68,1],[16959,70,3],[16959,72,1],[16959,78,1],[16960,19,1],
[16960,29,1],[16960,55,1],[16960,58,1],[16960,59,1],[16960,60,1],
[16960,65,1],[16960,67,1],[16961,26,1],[16961,48,2],[16962,2,1],
[16962,4,1],[16962,5,2],[16962,16,1],[16962,17,1],[16962,21,26],
[16962,23,2],[16962,28,2],[16962,66,1],[16962,70,1],[16963,0,1],
[16963,1,2],[16963,12,2],[16963,17,1],[16963,30,11],[16963,58,1],
[16964,2,1],[16964,3,2],[16964,5,10],[16964,6,2],[16964,20,1],
[16964,21,3],[16964,22,3],[16964,23,10],[16964,24,11],[16964,26,4],
[16964,27,6],[16964,28,4],[16964,31,4],[16964,32,11],[16964,33,9],
[16964,35,3],[16964,38,1],[16964,39,5],[16964,42,4],[16964,43,4],
[16964,46,1],[16964,47,5],[16964,48,15],[16964,62,11],[16964,76,1],
[16965,28,1],[16965,31,1],[16966,1,4],[16966,4,1],[16966,13,1],

[16966,24,1],[16966,25,4],[16966,56,1],[16966,58,1],[16966,61,2],
[16966,65,1],[16967,27,1],[16967,28,1],[16967,33,1],[16968,27,1],
[16968,47,1],[16968,52,1],[16969,2,2],[16969,32,2],[16970,1,1],
[16970,29,2],[16971,20,1],[16971,24,1],[16971,32,1],[16971,35,2],
[16972,2,3],[16972,17,1],[16972,60,1],[16972,69,1],[16973,1,10],
[16973,38,1],[16974,2,1],[16974,3,2],[16974,23,2],[16974,24,2],
[16974,25,4],[16974,27,3],[16974,31,2],[16974,35,1],[16975,25,2],
[16975,26,1],[16975,28,2],[16975,32,1],[16975,33,7],[16975,35,9],
[16975,38,1],[16975,52,2],[16976,5,1],[16976,36,1],[16976,41,1],
[16976,59,5],[16976,78,1],[16977,28,2],[16977,43,1],[16978,24,2],
[16979,11,1],[16979,29,1],[16979,31,1],[16979,63,2],[16980,2,1],
[16980,23,2],[16980,25,3],[16980,27,5],[16980,28,2],[16981,35,1],
[16981,42,1],[16982,16,1],[16982,17,1],[16982,35,1],[16982,37,1],
[16982,65,1],[16982,73,2],[16983,21,1],[16983,22,2],[16983,24,2],
[16983,26,2],[16983,27,3],[16983,28,1],[16983,31,1],[16983,35,4],
[16983,42,2],[16983,47,1],[16984,28,2],[16985,28,2],[16986,24,1],
[16986,28,1],[16987,76,2],[16988,1,2],[16988,4,6],[16988,17,9],
[16988,19,2],[16988,20,1],[16988,22,1],[16988,25,1],[16988,26,1],
[16988,29,1],[16988,31,1],[16988,38,1],[16988,39,1],[16988,44,1],
[16988,50,2],[16988,53,7],[16988,55,4],[16988,56,1],[16988,59,1],
[16988,66,3],[16989,2,1],[16989,24,1],[16990,20,1],[16990,22,5],
[16990,23,3],[16990,27,4],[16990,28,2],[16990,33,1],[16990,35,2],
[16990,38,1],[16990,47,1],[16991,28,1],[16991,62,1],[16992,10,1],
[16992,69,1],[16993,26,2],[16993,44,1],[16993,56,1],[16994,1,1],
[16994,2,3],[16994,5,1],[16994,13,3],[16994,19,1],[16994,20,3],
[16994,22,1],[16994,24,7],[16994,26,1],[16994,27,1],[16994,28,1],
[16994,29,1],[16994,31,3],[16994,39,7],[16994,40,1],[16994,42,5],
[16994,43,7],[16994,47,1],[16994,62,6],[16994,77,1],[16995,20,1],
[16995,24,1],[16996,28,1],[16996,40,1],[16997,20,1],[16997,27,1],
[16997,28,2],[16998,20,3],[16999,1,3],[16999,2,1],[16999,21,1],
[16999,39,1],[16999,42,1],[16999,50,1],[17000,20,1],[17000,28,1],
[17001,15,2],[17002,30,2],[17003,0,1],[17003,3,1],[17003,4,1],
[17003,24,1],[17003,29,1],[17003,30,1],[17003,62,1],[17003,65,1],
[17003,70,1],[17004,27,2],[17005,0,1],[17005,27,1],[17006,2,1],
[17006,23,1],[17006,28,2],[17006,32,61],[17006,33,1],[17006,56,1],
[17006,62,12],[17007,23,4],[17007,24,1],[17007,28,1],[17008,31,5],
[17009,32,1],[17009,47,1],[17010,3,3],[17010,22,3],[17010,23,2],
[17010,24,3],[17010,26,2],[17010,27,1],[17010,28,2],[17010,32,2],
[17011,34,1],[17011,48,1],[17012,3,1],[17012,43,3],[17013,11,1],
[17013,45,1],[17013,59,1],[17013,78,1],[17014,29,1],[17014,41,1],
[17014,45,1],[17014,57,1],[17014,59,1],[17014,65,1],[17014,67,1],
[17015,15,1],[17015,55,1],[17016,28,21],[17016,32,1],[17016,73,1],
[17017,27,2],[17018,44,2],[17018,63,1],[17018,66,2],[17019,2,1],
[17019,28,1],[17019,31,1],[17020,3,4],[17021,22,2],[17021,25,5],
[17021,27,3],[17021,28,5],[17021,30,1],[17021,33,19],[17021,35,13],
[17021,38,1],[17021,52,4],[17022,23,1],[17022,28,1],[17022,33,1],
[17022,35,3],[17022,42,1],[17022,56,2],[17023,3,1],[17023,22,1],
[17023,23,3],[17023,24,1],[17023,26,1],[17023,27,1],[17023,28,2],
[17023,33,1],[17023,43,1],[17023,74,1],[17024,5,1],[17024,13,1],
[17024,20,2],[17024,25,1],[17024,48,1],[17025,3,1],[17025,31,1],

[17025,44,1],[17025,60,1],[17026,20,1],[17026,26,1],[17026,59,1],
[17027,2,8],[17027,27,1],[17028,44,1],[17028,50,1],[17028,66,2],
[17029,53,1],[17029,59,2],[17030,4,3],[17030,17,4],[17030,25,1],
[17030,27,1],[17030,50,5],[17030,53,5],[17031,66,2],[17032,20,1],
[17032,32,1],[17032,59,1],[17033,41,1],[17033,77,1],[17034,12,1],
[17034,27,1],[17035,38,1],[17035,44,1],[17036,32,2],[17037,21,1],
[17037,42,1],[17038,21,2],[17038,31,1],[17039,21,1],[17039,66,1],
[17040,28,2],[17040,32,1],[17041,9,1],[17041,30,1],[17042,23,1],
[17042,25,8],[17042,27,1],[17042,56,10],[17043,19,1],[17043,20,1],
[17044,1,1],[17044,3,1],[17044,4,1],[17044,5,7],[17044,6,2],
[17044,13,1],[17044,20,1],[17044,22,1],[17044,24,1],[17044,27,2],
[17044,35,1],[17044,38,1],[17044,42,1],[17044,44,1],[17044,48,1],
[17044,50,1],[17044,51,1],[17044,70,5],[17044,71,1],[17044,74,1],
[17044,75,1],[17045,0,2],[17045,1,8],[17045,2,1],[17045,3,2],
[17045,4,20],[17045,5,2],[17045,8,4],[17045,9,8],[17045,10,3],
[17045,11,2],[17045,12,6],[17045,13,5],[17045,14,1],[17045,15,97],
[17045,17,65],[17045,19,4],[17045,20,7],[17045,22,2],[17045,28,2],
[17045,29,3],[17045,30,1],[17045,31,1],[17045,34,3],[17045,36,2],
[17045,37,1],[17045,38,4],[17045,39,1],[17045,41,2],[17045,44,10],
[17045,45,4],[17045,47,1],[17045,48,1],[17045,50,21],[17045,53,87],
[17045,54,2],[17045,55,191],[17045,56,1],[17045,65,1],[17045,66,8],
[17045,67,1],[17045,70,1],[17045,73,2],[17045,76,1],[17045,77,1],
[17045,78,1],[17046,5,1],[17046,26,3],[17046,63,1],[17046,66,1],
[17047,0,1],[17047,1,1],[17047,9,1],[17047,10,2],[17047,12,5],
[17047,13,1],[17047,15,3],[17047,16,1],[17047,17,1],[17047,19,2],
[17047,21,1],[17047,23,2],[17047,24,1],[17047,28,1],[17047,30,2],
[17047,31,5],[17047,39,1],[17047,41,1],[17047,47,1],[17047,55,1],
[17047,58,1],[17047,60,1],[17047,71,1],[17047,78,1],[17048,3,1],
[17048,19,1],[17048,21,2],[17048,22,2],[17048,23,1],[17048,27,1],
[17048,35,1],[17048,43,1],[17048,48,1],[17048,59,1],[17049,0,1],
[17049,1,2],[17049,7,1],[17049,9,1],[17049,10,2],[17049,11,1],
[17049,15,1],[17049,19,3],[17049,34,1],[17049,39,1],[17049,45,4],
[17049,46,1],[17049,49,1],[17049,57,1],[17049,65,1],[17049,71,1],
[17049,78,1],[17050,2,3],[17050,3,2],[17050,4,1],[17050,6,3],
[17050,18,1],[17050,21,1],[17050,22,1],[17050,23,5],[17050,24,2],
[17050,26,2],[17050,27,4],[17050,30,1],[17050,35,3],[17050,41,1],
[17050,43,2],[17050,69,1],[17051,41,1],[17051,58,1],[17052,21,1],
[17052,27,1],[17052,62,2],[17053,57,1],[17053,58,1],[17054,1,6],
[17054,13,1],[17054,15,1],[17054,30,1],[17055,8,1],[17055,13,4],
[17055,20,1],[17055,59,1],[17056,28,1],[17056,32,1],[17056,35,1],
[17057,28,1],[17057,56,1],[17058,21,1],[17058,22,1],[17058,23,2],
[17058,25,12],[17058,27,7],[17058,28,1],[17058,35,5],[17059,2,1],
[17059,3,2],[17059,47,1],[17060,2,2],[17060,28,1],[17061,8,3],
[17062,77,3],[17063,28,1],[17063,35,1],[17064,22,4],[17064,23,4],
[17065,24,1],[17065,54,1],[17066,24,3],[17066,25,1],[17066,27,1],
[17066,31,4],[17066,32,2],[17066,42,1],[17066,52,1],[17066,59,2],
[17067,2,2],[17067,7,1],[17067,10,1],[17067,11,1],[17067,12,1],
[17067,13,1],[17067,19,1],[17067,20,3],[17067,23,3],[17067,24,2],
[17067,27,3],[17067,30,5],[17067,33,3],[17067,37,2],[17067,39,1],
[17067,43,1],[17067,45,2],[17067,47,1],[17067,48,1],[17067,55,1],

[17067,56,1],[17067,66,1],[17067,77,2],[17068,28,1],[17068,44,3],
[17068,66,2],[17068,74,1],[17069,3,1],[17069,27,1],[17070,3,1],
[17070,5,1],[17070,6,1],[17070,15,1],[17070,16,1],[17070,20,2],
[17070,22,1],[17070,23,3],[17070,24,2],[17070,27,1],[17070,28,1],
[17070,32,1],[17070,35,1],[17070,40,1],[17070,47,1],[17070,51,1],
[17070,61,1],[17070,62,1],[17070,63,1],[17070,66,1],[17070,69,1],
[17070,70,1],[17070,75,2],[17071,28,1],[17071,55,1],[17071,74,1],
[17072,22,1],[17072,28,2],[17072,35,15],[17073,1,1],[17073,2,3],
[17073,3,1],[17073,4,1],[17073,9,1],[17073,10,1],[17073,13,3],
[17073,15,1],[17073,20,4],[17073,22,2],[17073,23,7],[17073,24,2],
[17073,26,2],[17073,27,1],[17073,28,2],[17073,29,2],[17073,31,11],
[17073,32,3],[17073,35,2],[17073,39,3],[17073,42,1],[17073,43,6],
[17073,45,2],[17073,47,3],[17073,52,1],[17073,55,1],[17073,59,2],
[17073,65,3],[17074,20,1],[17074,63,1],[17075,9,1],[17075,38,1],
[17076,28,1],[17076,43,1],[17077,32,1],[17077,68,1],[17078,5,3],
[17078,22,1],[17078,35,1],[17079,23,1],[17079,31,1],[17079,74,3],
[17079,75,2],[17079,76,6],[17080,3,3],[17080,13,4],[17080,31,1],
[17080,33,7],[17080,60,1],[17080,75,2],[17081,2,4],[17081,17,1],
[17081,22,1],[17081,28,1],[17081,44,1],[17082,9,1],[17082,26,2],
[17082,37,1],[17083,20,1],[17083,60,1],[17084,4,2],[17084,15,1],
[17084,17,1],[17084,28,4],[17084,31,1],[17084,38,1],[17084,50,1],
[17084,66,2],[17085,3,1],[17085,6,3],[17085,22,4],[17085,23,1],
[17085,24,1],[17085,27,3],[17085,35,3],[17085,46,1],[17085,48,2],
[17085,62,1],[17086,3,2],[17086,22,2],[17086,23,8],[17086,24,1],
[17086,27,1],[17086,35,1],[17086,44,2],[17087,3,1],[17087,24,1],
[17087,27,2],[17087,43,1],[17087,59,1],[17087,69,1],[17088,8,1],
[17088,20,1],[17088,31,1],[17088,32,1],[17089,2,1],[17089,5,3],
[17089,44,1],[17089,56,1],[17090,26,3],[17090,42,2],[17090,60,1],
[17091,1,1],[17091,3,1],[17091,28,1],[17091,32,3],[17091,43,1],
[17092,2,2],[17092,42,1],[17093,2,1],[17093,5,1],[17093,8,1],
[17093,28,1],[17093,33,1],[17093,68,1],[17094,21,1],[17094,25,6],
[17094,26,9],[17094,28,1],[17094,43,3],[17095,17,1],[17095,27,1],
[17095,78,1],[17096,28,26],[17096,35,2],[17097,4,2],[17097,5,1],
[17097,50,1],[17098,42,2],[17099,24,1],[17099,32,1],[17099,47,1],
[17099,52,1],[17100,6,1],[17100,25,1],[17100,26,1],[17100,39,1],
[17100,57,1],[17101,12,1],[17101,13,4],[17101,20,2],[17101,21,1],
[17101,22,5],[17101,23,1],[17101,24,2],[17101,25,1],[17101,26,6],
[17101,27,4],[17101,28,1],[17101,30,1],[17101,31,1],[17101,33,3],
[17101,38,3],[17101,42,6],[17101,43,5],[17101,45,1],[17101,74,1],
[17102,35,1],[17102,42,1],[17102,68,3],[17102,69,1],[17102,70,1],
[17103,24,1],[17103,68,2],[17103,69,2],[17103,74,1],[17104,4,1],
[17104,10,1],[17104,14,1],[17104,15,3],[17104,50,1],[17104,55,1],
[17104,66,4],[17104,68,1],[17105,2,1],[17105,27,2],[17105,47,1],
[17105,70,1],[17106,6,1],[17106,28,2],[17107,22,1],[17107,28,1],
[17107,33,17],[17107,35,14],[17107,52,7],[17108,23,1],[17108,24,2],
[17108,26,1],[17108,27,2],[17109,1,1],[17109,23,11],[17109,26,2],
[17109,27,1],[17109,62,1],[17109,72,1],[17109,77,1],[17110,15,1],
[17110,17,1],[17110,19,1],[17110,20,1],[17110,53,3],[17110,55,1],
[17111,35,2],[17111,44,1],[17112,2,2],[17112,29,1],[17112,31,1],
[17113,17,1],[17113,24,1],[17113,35,1],[17114,4,1],[17114,5,6],

[17114,21,1],[17114,28,1],[17114,33,1],[17115,27,4],[17116,15,2],
[17117,15,1],[17117,55,1],[17118,2,1],[17118,33,1],[17119,3,2],
[17119,23,1],[17119,27,1],[17119,31,2],[17119,47,1],[17120,17,3],
[17120,28,3],[17120,42,1],[17120,50,1],[17120,53,6],[17120,55,2],
[17120,66,1],[17121,28,6],[17121,42,1],[17122,54,1],[17122,57,1],
[17122,60,1],[17123,2,1],[17123,23,1],[17123,39,1],[17123,43,1],
[17124,23,2],[17124,24,1],[17124,68,1],[17124,69,1],[17125,24,1],
[17125,27,1],[17125,47,1],[17126,27,1],[17126,47,2],[17127,27,1],
[17127,32,1],[17127,43,1],[17128,10,1],[17128,19,1],[17128,24,1],
[17128,28,1],[17129,65,1],[17129,74,1],[17130,28,1],[17130,47,2],
[17131,25,6],[17131,28,3],[17132,2,1],[17132,8,1],[17132,31,1],
[17133,1,2],[17133,13,3],[17133,20,1],[17133,53,1],[17133,55,3],
[17133,67,1],[17133,73,2],[17134,28,5],[17134,48,1],[17134,63,1],
[17134,74,1],[17134,76,2],[17135,8,2],[17135,20,1],[17135,34,3],
[17135,36,2],[17135,37,1],[17136,32,7],[17137,2,1],[17137,74,1],
[17138,22,1],[17138,34,3],[17139,28,2],[17139,35,3],[17139,62,1],
[17140,1,2],[17140,7,1],[17140,20,2],[17140,55,1],[17141,27,1],
[17141,28,1],[17141,32,1],[17141,35,1],[17142,35,2],[17143,21,1],
[17143,33,1],[17144,2,1],[17144,27,1],[17145,2,1],[17145,5,2],
[17145,24,1],[17145,25,2],[17145,27,1],[17145,38,1],[17145,44,1],
[17145,50,1],[17145,56,1],[17145,62,1],[17145,68,1],[17145,74,1],
[17146,0,11],[17146,1,80],[17146,7,10],[17146,8,12],[17146,9,86],
[17146,10,123],[17146,11,40],[17146,12,49],[17146,13,46],
[17146,14,27],[17146,15,50],[17146,16,68],[17146,17,23],[17146,18,26],
[17146,19,85],[17146,20,44],[17146,21,1],[17146,27,1],[17146,28,5],
[17146,29,14],[17146,30,19],[17146,31,1],[17146,34,26],[17146,35,1],
[17146,36,24],[17146,37,38],[17146,39,16],[17146,40,1],[17146,41,63],
[17146,45,63],[17146,46,31],[17146,48,1],[17146,49,32],[17146,53,32],
[17146,54,34],[17146,55,41],[17146,57,50],[17146,58,53],[17146,59,46],
[17146,60,9],[17146,63,1],[17146,64,9],[17146,65,67],[17146,67,39],
[17146,70,1],[17146,71,24],[17146,72,3],[17146,73,34],[17146,77,45],
[17146,78,8],[17147,25,1],[17147,27,2],[17147,28,1],[17148,28,2],
[17148,31,3],[17149,38,1],[17149,61,1],[17150,2,1],[17150,3,2],
[17150,5,3],[17150,9,1],[17150,13,2],[17150,15,2],[17150,17,1],
[17150,20,4],[17150,21,1],[17150,22,1],[17150,23,1],[17150,24,1],
[17150,25,1],[17150,27,4],[17150,32,2],[17150,33,1],[17150,35,1],
[17150,41,1],[17150,42,1],[17150,43,3],[17150,46,1],[17150,63,1],
[17150,66,1],[17150,73,1],[17150,74,1],[17150,76,1],[17151,3,1],
[17151,32,1],[17151,50,1],[17151,55,1],[17151,66,1],[17152,0,3],
[17152,1,3],[17152,9,1],[17152,11,2],[17152,12,1],[17152,17,1],
[17152,20,1],[17152,30,1],[17152,36,1],[17152,53,1],[17152,60,1],
[17152,65,1],[17153,47,2],[17154,28,1],[17154,48,5],[17155,2,8],
[17156,27,1],[17156,33,2],[17156,38,1],[17156,52,3],[17157,20,1],
[17157,57,1],[17158,23,1],[17158,24,1],[17159,2,1],[17159,25,1],
[17160,3,1],[17160,43,1],[17161,27,1],[17161,28,1],[17162,21,1],
[17162,27,1],[17162,33,1],[17163,6,1],[17163,62,1],[17164,13,1],
[17164,43,1],[17165,29,3],[17166,30,2],[17166,60,2],[17167,25,8],
[17167,34,1],[17168,31,17],[17169,21,2],[17169,23,1],[17169,27,1],
[17169,28,1],[17169,43,2],[17170,1,1],[17170,15,1],[17170,22,1],
[17170,23,1],[17170,30,1],[17170,35,2],[17170,56,1],[17170,57,1],

[17170,74,1],[17171,23,1],[17171,24,4],[17171,27,1],[17171,59,1],
[17172,47,1],[17172,56,1],[17173,24,1],[17173,26,1],[17173,28,2],
[17173,31,1],[17173,56,1],[17173,74,2],[17174,22,2],[17175,21,2],
[17175,24,2],[17175,25,1],[17175,26,2],[17175,31,6],[17175,32,1],
[17175,43,3],[17176,55,1],[17176,70,1],[17177,23,2],[17178,12,1],
[17178,21,1],[17179,23,1],[17179,42,1],[17180,38,2],[17180,50,1],
[17180,66,1],[17181,1,1],[17181,17,1],[17181,53,1],[17182,23,2],
[17182,25,1],[17182,27,2],[17182,31,1],[17182,35,1],[17182,56,1],
[17182,78,2],[17183,3,1],[17183,21,1],[17183,23,1],[17183,24,4],
[17183,26,1],[17183,27,1],[17183,28,3],[17183,33,1],[17183,34,1],
[17183,43,1],[17183,47,1],[17183,56,3],[17183,76,1],[17184,17,1],
[17184,19,1],[17184,35,1],[17185,4,2],[17185,6,1],[17185,13,1],
[17185,19,1],[17185,20,1],[17185,22,2],[17185,23,1],[17185,28,1],
[17185,38,2],[17185,47,1],[17185,50,2],[17185,51,1],[17185,56,1],
[17185,65,1],[17185,68,4],[17185,71,1],[17185,72,1],[17185,74,1],
[17186,22,1],[17186,24,1],[17186,27,2],[17186,33,1],[17186,74,1],
[17187,24,1],[17187,48,1],[17188,28,1],[17188,32,3],[17188,43,1],
[17189,6,1],[17189,23,2],[17190,22,2],[17191,24,2],[17192,9,1],
[17192,22,1],[17192,26,1],[17192,60,1],[17193,5,1],[17193,27,1],
[17193,35,2],[17193,63,22],[17193,74,10],[17193,75,8],[17193,76,24],
[17194,13,1],[17194,39,2],[17195,19,1],[17195,73,1],[17196,1,26],
[17196,9,4],[17196,10,1],[17196,12,3],[17196,13,6],[17196,19,2],
[17196,20,1],[17196,22,1],[17196,23,10],[17196,28,1],[17196,32,4],
[17196,35,1],[17196,37,1],[17196,44,1],[17196,45,1],[17196,56,1],
[17196,62,1],[17196,65,1],[17196,70,1],[17196,77,1],[17196,78,1],
[17197,20,1],[17197,30,1],[17198,23,1],[17198,35,1],[17198,59,1],
[17198,65,1],[17199,23,1],[17199,27,2],[17199,35,2],[17199,38,1],
[17199,40,1],[17199,47,1],[17199,62,1],[17200,6,2],[17200,22,1],
[17200,27,4],[17200,28,1],[17200,35,4],[17201,4,3],[17201,5,1],
[17201,19,1],[17201,27,1],[17201,44,2],[17201,50,4],[17201,66,1],
[17201,78,1],[17202,32,6],[17202,33,1],[17203,24,3],[17203,26,1],
[17203,28,4],[17203,42,1],[17203,43,2],[17204,8,1],[17204,9,2],
[17204,10,1],[17204,11,4],[17204,12,1],[17204,14,2],[17204,15,1],
[17204,16,3],[17204,19,3],[17204,20,1],[17204,39,1],[17204,41,3],
[17204,45,1],[17204,49,3],[17204,54,1],[17204,59,3],[17204,64,1],
[17204,65,2],[17204,71,1],[17204,73,1],[17204,77,1],[17205,43,3],
[17205,52,2],[17206,6,1],[17206,21,1],[17206,28,3],[17207,1,1],
[17207,2,4],[17207,8,1],[17207,32,1],[17207,37,1],[17207,41,1],
[17208,53,2],[17209,20,1],[17209,27,1],[17210,2,1],[17210,70,1],
[17211,1,1],[17211,15,7],[17211,16,1],[17211,30,1],[17211,36,1],
[17212,23,18],[17212,35,3],[17212,51,1],[17212,62,2],[17212,70,1],
[17213,1,1],[17213,9,1],[17213,10,4],[17213,15,1],[17213,19,1],
[17213,23,2],[17213,24,1],[17213,27,2],[17213,30,24],[17213,31,1],
[17213,33,8],[17213,35,6],[17213,40,1],[17213,52,3],[17213,54,1],
[17213,58,2],[17213,63,1],[17213,65,1],[17213,66,1],[17213,67,1],
[17213,70,2],[17213,71,1],[17214,13,1],[17214,21,3],[17214,23,1],
[17214,26,7],[17214,39,1],[17215,4,1],[17215,25,1],[17215,28,1],
[17215,70,1],[17215,79,1],[17216,24,1],[17216,25,1],[17217,3,3],
[17217,5,2],[17217,20,1],[17217,21,1],[17217,23,4],[17217,24,2],
[17217,27,1],[17217,32,2],[17217,46,1],[17218,23,1],[17218,51,1],

[17219,34,1],[17219,55,2],[17219,67,1],[17220,2,4],[17221,28,2],
[17221,63,1],[17221,74,3],[17222,1,1],[17222,5,2],[17222,21,2],
[17222,24,1],[17222,28,8],[17222,30,1],[17223,27,2],[17224,24,1],
[17224,28,3],[17224,33,1],[17225,1,1],[17225,12,1],[17225,45,1],
[17226,0,1],[17226,9,1],[17227,33,1],[17227,37,1],[17227,46,1],
[17227,70,1],[17228,0,1],[17228,1,61],[17228,2,5],[17228,5,1],
[17228,7,1],[17228,8,6],[17228,9,1],[17228,11,2],[17228,12,69],
[17228,13,14],[17228,15,6],[17228,16,96],[17228,17,7],[17228,20,7],
[17228,23,3],[17228,25,1],[17228,29,74],[17228,30,5],[17228,34,4],
[17228,36,7],[17228,37,1],[17228,39,15],[17228,53,4],[17228,55,6],
[17228,58,1],[17228,59,1],[17228,68,1],[17228,70,1],[17228,73,3],
[17229,23,3],[17230,66,2],[17231,28,1],[17231,62,1],[17232,28,1],
[17232,31,1],[17233,25,1],[17233,33,4],[17233,35,5],[17233,52,2],
[17233,79,1],[17234,20,1],[17234,27,1],[17234,28,1],[17235,10,1],
[17235,11,1],[17235,13,1],[17235,19,1],[17235,20,1],[17235,36,1],
[17235,41,1],[17235,46,1],[17235,54,1],[17235,59,2],[17235,65,2],
[17235,73,1],[17235,77,1],[17236,17,1],[17236,42,1],[17237,2,3],
[17237,4,22],[17237,23,1],[17237,24,1],[17237,35,1],[17237,40,2],
[17237,44,1],[17237,47,1],[17237,49,1],[17237,50,15],[17237,51,8],
[17237,55,1],[17237,61,2],[17237,66,1],[17237,68,3],[17237,69,2],
[17237,70,5],[17237,75,3],[17238,4,1],[17238,5,1],[17238,26,1],
[17238,28,1],[17238,36,1],[17239,12,1],[17239,15,1],[17239,37,1],
[17239,55,1],[17240,5,1],[17240,6,1],[17240,23,1],[17240,51,1],
[17240,56,1],[17241,28,2],[17241,31,2],[17241,48,1],[17242,51,2],
[17243,9,1],[17243,32,2],[17243,39,1],[17244,1,1],[17244,10,1],
[17244,16,1],[17244,19,1],[17244,49,1],[17244,57,2],[17244,58,1],
[17245,3,1],[17245,27,2],[17245,28,1],[17246,22,2],[17247,21,1],
[17247,31,2],[17247,32,6],[17247,43,1],[17248,2,1],[17248,3,1],
[17248,8,2],[17248,11,1],[17248,20,7],[17248,23,3],[17248,27,8],
[17248,30,5],[17248,31,17],[17248,32,3],[17248,34,1],[17248,39,1],
[17248,45,1],[17248,47,1],[17248,56,1],[17248,57,1],[17248,73,1],
[17248,77,1],[17249,26,1],[17249,35,1],[17250,15,1],[17250,55,2],
[17251,23,1],[17251,35,1],[17251,79,1],[17252,27,1],[17252,28,2],
[17253,28,2],[17254,13,1],[17254,25,1],[17254,29,1],[17255,40,33],
[17255,68,4],[17255,69,2],[17255,70,29],[17255,72,11],[17256,25,2],
[17256,27,1],[17256,32,2],[17256,40,1],[17256,61,2],[17256,63,1],
[17256,66,1],[17257,27,1],[17257,42,1],[17257,47,1],[17257,62,3],
[17258,2,40],[17258,5,1],[17258,24,2],[17258,31,5],[17258,39,1],
[17259,17,1],[17259,45,1],[17259,53,1],[17260,3,1],[17260,5,2],
[17260,28,2],[17261,23,1],[17261,25,1],[17261,28,1],[17261,35,1],
[17262,2,2],[17263,2,2],[17263,5,1],[17263,27,1],[17263,28,3],
[17263,32,1],[17263,33,1],[17263,47,1],[17263,59,2],[17264,8,2],
[17265,41,1],[17265,50,1],[17265,53,1],[17266,23,1],[17266,32,14],
[17266,38,1],[17266,47,14],[17266,50,1],[17266,51,1],[17267,24,1],
[17267,28,1],[17268,25,2],[17268,31,3],[17268,34,2],[17268,65,1],
[17268,74,1],[17269,21,4],[17270,74,1],[17270,76,1],[17271,13,2],
[17271,23,1],[17271,25,5],[17271,33,1],[17272,22,1],[17272,27,6],
[17272,28,1],[17272,35,3],[17272,76,1],[17273,4,1],[17273,8,1],
[17273,12,6],[17273,20,1],[17273,39,1],[17273,47,1],[17273,53,2],
[17273,67,1],[17274,1,1],[17274,2,1],[17274,8,2],[17274,21,1],

[17274,24,3],[17274,25,1],[17274,26,1],[17274,27,2],[17274,28,2],
[17274,31,1],[17274,32,1],[17274,33,1],[17274,35,1],[17274,42,1],
[17274,43,1],[17275,14,1],[17275,20,13],[17275,31,1],[17275,42,2],
[17275,43,1],[17275,58,2],[17275,62,3],[17276,4,1],[17276,25,1],
[17277,12,1],[17277,24,1],[17277,26,1],[17277,31,1],[17278,1,1],
[17278,13,1],[17278,20,1],[17278,23,2],[17278,24,2],[17278,28,1],
[17278,30,1],[17279,9,1],[17279,16,1],[17279,28,6],[17279,65,1],
[17280,3,1],[17280,6,1],[17280,8,1],[17280,22,1],[17280,23,1],
[17280,27,1],[17280,31,1],[17280,35,1],[17280,38,1],[17280,59,1],
[17281,5,1],[17281,9,3],[17281,18,2],[17281,24,3],[17281,27,3],
[17281,28,10],[17281,32,2],[17281,35,2],[17281,38,1],[17281,39,1],
[17281,42,3],[17281,47,2],[17281,50,1],[17281,56,4],[17281,61,2],
[17281,70,2],[17282,10,1],[17282,13,1],[17282,19,1],[17282,21,1],
[17282,23,1],[17282,24,3],[17282,25,2],[17282,27,1],[17282,30,1],
[17282,31,1],[17282,32,3],[17282,33,2],[17282,34,1],[17282,40,7],
[17282,41,2],[17282,44,1],[17282,50,2],[17282,56,2],[17282,59,1],
[17282,63,99],[17282,68,3],[17282,69,1],[17282,70,5],[17282,72,4],
[17282,73,1],[17282,74,98],[17282,75,101],[17282,76,159],[17283,19,1],
[17283,35,1],[17284,22,1],[17284,28,3],[17284,33,2],[17284,56,1],
[17284,61,1],[17285,2,1],[17285,5,1],[17285,25,1],[17286,3,1],
[17286,6,1],[17286,15,1],[17286,16,1],[17286,20,5],[17286,21,3],
[17286,23,1],[17286,24,1],[17286,26,1],[17286,35,1],[17286,39,1],
[17286,42,1],[17286,44,1],[17286,45,1],[17286,51,1],[17286,54,1],
[17286,56,3],[17286,61,1],[17286,74,1],[17287,1,1],[17287,2,1],
[17287,3,2],[17287,5,2],[17287,6,14],[17287,7,1],[17287,8,4],
[17287,9,1],[17287,10,1],[17287,11,1],[17287,12,1],[17287,15,1],
[17287,16,10],[17287,20,1],[17287,21,1],[17287,22,12],[17287,23,43],
[17287,24,2],[17287,25,3],[17287,26,20],[17287,27,70],[17287,28,5],
[17287,29,1],[17287,31,6],[17287,32,3],[17287,33,21],[17287,35,15],
[17287,38,2],[17287,40,2],[17287,42,3],[17287,43,10],[17287,47,1],
[17287,49,1],[17287,51,4],[17287,56,1],[17287,57,2],[17287,59,1],
[17287,60,3],[17287,62,1],[17287,64,1],[17287,65,1],[17287,70,3],
[17287,72,1],[17287,74,3],[17287,76,1],[17288,2,1],[17288,3,4],
[17288,4,1],[17288,22,4],[17288,23,4],[17288,24,1],[17288,25,1],
[17288,26,3],[17288,27,6],[17288,28,1],[17288,31,4],[17288,32,4],
[17288,33,1],[17288,35,2],[17288,38,2],[17288,43,1],[17288,47,1],
[17288,68,1],[17288,75,1],[17289,24,1],[17289,39,1],[17290,6,1],
[17290,33,1],[17291,24,2],[17292,1,1],[17292,5,1],[17292,13,3],
[17292,15,1],[17292,17,1],[17292,24,3],[17292,36,1],[17292,47,1],
[17292,55,1],[17293,2,4],[17293,39,1],[17294,28,13],[17294,33,4],
[17295,5,1],[17295,6,1],[17295,23,1],[17295,28,1],[17296,1,7],
[17296,12,4],[17296,13,1],[17296,17,1],[17296,22,1],[17296,23,5],
[17296,24,1],[17296,27,2],[17296,29,1],[17296,32,2],[17296,35,1],
[17296,43,1],[17296,59,2],[17297,1,14],[17298,21,1],[17298,22,2],
[17298,23,1],[17298,25,1],[17298,26,2],[17298,27,2],[17298,35,1],
[17298,39,1],[17299,15,3],[17299,17,2],[17299,44,1],[17299,46,1],
[17299,53,2],[17299,55,3],[17300,2,1],[17300,4,2],[17300,5,2],
[17300,10,2],[17300,12,1],[17300,15,34],[17300,17,6],[17300,22,1],
[17300,23,1],[17300,26,2],[17300,30,1],[17300,31,1],[17300,32,3],
[17300,38,5],[17300,42,2],[17300,44,11],[17300,46,1],[17300,47,1],

[17300,50,7],[17300,53,8],[17300,55,25],[17300,56,1],[17300,60,1],
[17300,66,4],[17300,70,1],[17300,71,1],[17300,72,1],[17301,0,2],
[17301,1,2],[17301,7,2],[17301,9,1],[17301,10,7],[17301,13,2],
[17301,15,1],[17301,16,3],[17301,18,2],[17301,19,5],[17301,20,1],
[17301,30,2],[17301,37,3],[17301,41,1],[17301,45,2],[17301,53,1],
[17301,55,1],[17301,58,1],[17301,59,4],[17301,65,2],[17301,67,1],
[17301,71,2],[17301,77,1],[17301,78,1],[17302,1,2],[17303,48,2],
[17303,51,1],[17304,21,1],[17304,23,4],[17304,24,1],[17304,35,1],
[17304,38,1],[17304,50,1],[17304,76,1],[17305,8,1],[17305,12,1],
[17305,19,1],[17305,20,1],[17305,46,1],[17306,3,2],[17306,23,1],
[17306,28,3],[17307,6,1],[17307,23,5],[17307,28,1],[17307,39,1],
[17307,74,6],[17308,28,8],[17308,31,1],[17308,43,1],[17309,44,2],
[17309,66,1],[17310,8,1],[17310,10,2],[17310,12,1],[17310,13,1],
[17310,15,1],[17310,16,1],[17310,18,1],[17310,20,1],[17310,31,1],
[17310,46,1],[17310,49,1],[17310,54,1],[17310,55,2],[17310,58,1],
[17310,59,1],[17310,66,1],[17310,71,1],[17310,76,1],[17310,77,1],
[17311,2,1],[17311,17,1],[17311,22,2],[17311,28,1],[17311,35,1],
[17311,42,1],[17311,53,1],[17312,27,1],[17312,32,2],[17312,56,1],
[17313,1,4],[17313,27,1],[17313,33,1],[17313,39,4],[17313,53,1],
[17314,21,1],[17314,28,2],[17315,2,2],[17315,4,1],[17315,23,1],
[17315,77,1],[17316,28,1],[17316,32,8],[17317,4,1],[17317,17,7],
[17317,50,1],[17317,53,7],[17317,75,1],[17317,76,1],[17318,3,3],
[17318,22,1],[17318,23,3],[17318,24,13],[17318,28,3],[17318,56,6],
[17319,15,3],[17319,20,3],[17319,23,1],[17319,24,2],[17319,43,1],
[17319,50,1],[17319,55,1],[17319,56,1],[17319,62,1],[17320,1,1],
[17320,2,1],[17320,6,1],[17320,8,1],[17320,12,1],[17320,13,1],
[17320,20,1],[17320,22,10],[17320,23,2],[17320,25,21],[17320,26,1],
[17320,27,7],[17320,28,11],[17320,30,18],[17320,32,11],[17320,33,54],
[17320,35,30],[17320,38,5],[17320,44,1],[17320,52,46],[17320,62,1],
[17320,66,1],[17320,67,1],[17320,70,3],[17321,27,1],[17321,55,1],
[17321,60,1],[17321,61,3],[17321,62,1],[17321,69,5],[17321,70,2],
[17322,22,1],[17322,25,4],[17322,28,4],[17322,42,1],[17323,12,1],
[17323,18,1],[17323,28,1],[17323,32,1],[17323,33,1],[17323,39,5],
[17324,8,1],[17324,24,1],[17324,27,4],[17324,42,1],[17325,12,1],
[17325,15,1],[17325,17,4],[17325,29,1],[17325,53,3],[17326,3,1],
[17326,26,1],[17326,35,1],[17327,33,1],[17327,34,1],[17328,4,1],
[17328,21,1],[17328,24,1],[17328,39,1],[17328,42,1],[17329,2,2],
[17329,18,1],[17329,30,2],[17330,4,5],[17330,10,2],[17330,12,1],
[17330,15,11],[17330,17,15],[17330,20,1],[17330,27,1],[17330,30,1],
[17330,32,1],[17330,37,2],[17330,50,9],[17330,53,16],[17330,55,24],
[17330,59,1],[17330,62,1],[17330,63,1],[17330,67,1],[17330,70,1],
[17330,71,1],[17331,2,1],[17331,5,1],[17331,12,1],[17331,15,1],
[17331,24,1],[17331,25,1],[17331,26,1],[17331,42,1],[17331,59,1],
[17331,62,1],[17332,20,1],[17332,22,2],[17332,35,1],[17332,43,2],
[17333,25,3],[17334,21,1],[17334,26,2],[17334,53,1],[17335,8,1],
[17335,9,1],[17335,11,1],[17335,16,1],[17335,34,1],[17335,37,1],
[17335,73,1],[17336,2,4],[17336,20,1],[17336,27,1],[17336,30,1],
[17337,5,1],[17337,27,36],[17337,32,1],[17337,35,1],[17337,36,1],
[17337,43,4],[17337,50,1],[17337,52,1],[17338,15,1],[17338,46,1],
[17338,53,2],[17338,54,1],[17338,55,11],[17338,76,1],[17339,2,2],

[17339,21,1],[17339,22,1],[17339,24,1],[17339,31,1],[17339,33,2],
[17339,47,1],[17339,60,1],[17340,24,1],[17340,27,1],[17340,31,1],
[17341,20,1],[17341,47,1],[17342,4,1],[17342,22,1],[17342,24,2],
[17342,27,1],[17342,35,2],[17342,42,1],[17343,33,17],[17343,35,3],
[17343,52,3],[17344,2,1],[17344,5,1],[17344,6,2],[17344,8,1],
[17344,20,2],[17344,22,1],[17344,23,1],[17344,24,1],[17344,27,7],
[17344,28,3],[17344,31,1],[17344,32,5],[17344,33,3],[17344,35,2],
[17344,38,1],[17344,45,1],[17344,69,1],[17344,76,1],[17345,3,1],
[17345,28,1],[17346,2,2],[17346,3,1],[17346,6,1],[17346,10,1],
[17346,20,3],[17346,22,4],[17346,27,1],[17346,35,1],[17346,47,6],
[17346,53,1],[17346,55,1],[17346,59,1],[17346,60,1],[17347,5,1],
[17347,6,1],[17347,24,3],[17347,27,8],[17347,38,1],[17347,56,1],
[17347,68,1],[17348,15,3],[17348,55,4],[17349,8,1],[17349,20,1],
[17349,27,1],[17349,34,1],[17349,36,2],[17349,73,1],[17350,1,1],
[17350,3,1],[17350,5,1],[17350,9,1],[17350,10,1],[17350,27,1],
[17350,41,1],[17350,42,1],[17350,43,1],[17350,49,1],[17350,52,1],
[17350,59,1],[17350,67,1],[17350,73,1],[17351,2,2],[17351,3,1],
[17351,6,1],[17351,8,1],[17351,31,1],[17351,33,1],[17351,45,1],
[17351,47,1],[17351,68,1],[17352,1,4],[17352,7,48],[17352,8,3],
[17352,9,1],[17352,12,2],[17352,13,4],[17352,15,1],[17352,17,5],
[17352,20,2],[17352,29,2],[17352,34,3],[17352,36,3],[17352,39,7],
[17352,53,4],[17352,55,2],[17352,59,3],[17352,73,25],[17353,0,2],
[17353,1,11],[17353,7,71],[17353,9,12],[17353,10,6],[17353,11,4],
[17353,12,4],[17353,13,14],[17353,14,50],[17353,15,15],[17353,16,40],
[17353,17,8],[17353,18,24],[17353,19,10],[17353,20,13],[17353,29,3],
[17353,30,2],[17353,34,6],[17353,37,2],[17353,39,2],[17353,41,2],
[17353,45,5],[17353,46,1],[17353,49,3],[17353,53,4],[17353,54,2],
[17353,55,8],[17353,57,1],[17353,58,6],[17353,59,3],[17353,60,3],
[17353,65,8],[17353,67,1],[17353,71,4],[17353,72,1],[17353,73,2],
[17353,77,233],[17353,78,1],[17354,17,1],[17354,27,1],[17354,36,1],
[17354,39,1],[17355,1,1],[17355,29,1],[17355,53,1],[17356,20,1],
[17356,53,1],[17357,2,13],[17358,23,2],[17358,33,1],[17358,39,1],
[17358,58,1],[17359,22,2],[17359,35,1],[17360,2,54],[17360,24,1],
[17360,28,2],[17360,46,1],[17360,47,1],[17360,56,2],[17361,1,6],
[17361,2,3],[17361,3,4],[17361,4,1],[17361,8,1],[17361,9,1],
[17361,12,1],[17361,13,13],[17361,17,1],[17361,20,5],[17361,21,2],
[17361,23,8],[17361,25,1],[17361,26,11],[17361,28,1],[17361,30,1],
[17361,31,1],[17361,35,1],[17361,39,5],[17361,43,2],[17361,47,10],
[17361,48,1],[17361,51,2],[17361,55,1],[17361,59,4],[17361,68,1],
[17361,70,1],[17361,74,1],[17362,12,1],[17362,20,1],[17362,26,1],
[17362,28,1],[17363,62,3],[17364,13,2],[17364,29,1],[17364,42,1],
[17364,43,1],[17365,15,2],[17365,53,1],[17366,4,1],[17366,44,1],
[17367,30,3],[17368,33,3],[17369,1,2],[17369,8,1],[17369,27,1],
[17369,39,2],[17370,5,1],[17370,8,5],[17370,9,1],[17370,28,1],
[17370,31,16],[17370,34,2],[17370,35,1],[17370,36,1],[17370,39,1],
[17370,53,1],[17370,54,1],[17370,55,1],[17371,27,5],[17371,28,1],
[17371,74,1],[17372,1,1],[17372,2,36],[17372,3,3],[17372,4,1],
[17372,20,3],[17372,22,2],[17372,23,1],[17372,24,2],[17372,26,1],
[17372,27,4],[17372,28,1],[17372,29,1],[17372,31,7],[17372,38,1],
[17372,39,1],[17372,42,1],[17372,43,2],[17372,47,1],[17372,50,1],

[17372,53,1],[17372,56,1],[17372,60,1],[17372,74,1],[17373,17,1],
[17373,39,1],[17374,6,3],[17375,7,3],[17375,10,1],[17375,77,1],
[17376,17,1],[17376,20,1],[17376,24,1],[17376,27,1],[17376,44,2],
[17376,53,1],[17376,54,1],[17376,55,3],[17376,72,1],[17377,21,1],
[17377,22,3],[17377,27,1],[17377,32,1],[17377,42,10],[17378,26,1],
[17378,27,1],[17379,0,10],[17379,7,3],[17379,8,1],[17379,10,1],
[17379,19,1],[17379,39,1],[17379,53,1],[17379,77,1],[17379,78,1],
[17380,2,8],[17380,3,2],[17380,26,1],[17380,28,1],[17380,30,2],
[17380,31,1],[17381,2,1],[17381,55,1],[17382,2,1],[17382,37,1],
[17383,3,1],[17383,27,1],[17383,55,1],[17383,62,2],[17384,23,1],
[17384,27,2],[17384,28,2],[17384,35,1],[17384,60,1],[17385,32,1],
[17385,48,1],[17386,4,1],[17386,55,1],[17386,70,2],[17387,1,6],
[17387,12,3],[17387,17,2],[17387,29,10],[17387,36,1],[17387,39,2],
[17387,55,1],[17388,35,3],[17388,79,1],[17389,3,1],[17389,8,1],
[17389,15,2],[17389,17,1],[17389,31,1],[17389,47,1],[17389,60,1],
[17389,70,1],[17390,4,1],[17390,10,1],[17390,11,1],[17390,27,1],
[17390,28,1],[17390,37,1],[17390,45,1],[17390,68,1],[17390,76,1],
[17391,10,1],[17391,20,1],[17392,10,1],[17392,41,1],[17393,2,4],
[17393,3,1],[17393,20,1],[17393,24,1],[17394,26,1],[17394,28,1],
[17395,23,3],[17396,47,3],[17397,2,1],[17397,23,1],[17398,0,1],
[17398,2,7],[17398,3,1],[17398,4,1],[17398,9,1],[17398,17,1],
[17398,20,2],[17398,28,1],[17398,31,2],[17398,34,1],[17398,43,1],
[17398,55,1],[17399,2,1],[17399,28,1],[17400,5,6],[17400,21,2],
[17400,24,2],[17400,26,1],[17400,30,2],[17400,47,1],[17401,2,1],
[17401,4,17],[17401,5,1],[17401,24,2],[17401,28,3],[17401,50,30],
[17401,66,1],[17402,25,1],[17402,30,1],[17402,33,13],[17402,38,2],
[17402,50,1],[17402,52,1],[17403,2,1],[17403,3,1],[17403,50,1],
[17404,2,1],[17404,28,1],[17405,2,1],[17405,31,1],[17406,2,2],
[17407,44,1],[17407,66,1],[17408,1,1],[17408,4,2],[17408,5,1],
[17408,13,1],[17408,15,2],[17408,17,3],[17408,19,2],[17408,20,1],
[17408,22,1],[17408,24,1],[17408,28,1],[17408,33,1],[17408,34,1],
[17408,38,2],[17408,44,5],[17408,45,1],[17408,50,2],[17408,53,4],
[17408,54,1],[17408,55,2],[17408,65,1],[17408,66,4],[17408,69,1],
[17408,70,3],[17408,73,1],[17408,74,1],[17408,76,2],[17409,15,1],
[17409,41,1],[17410,27,2],[17411,5,1],[17411,26,1],[17411,53,1],
[17412,2,2],[17412,3,1],[17412,8,1],[17412,13,1],[17412,21,2],
[17412,22,3],[17412,23,17],[17412,24,3],[17412,26,2],[17412,27,1],
[17412,31,17],[17412,32,1],[17412,33,1],[17412,42,1],[17412,43,1],
[17412,70,1],[17413,28,1],[17413,32,4],[17414,21,2],[17414,22,1],
[17414,35,1],[17415,2,6],[17415,28,4],[17416,8,2],[17416,33,1],
[17416,35,1],[17416,38,1],[17417,40,1],[17417,51,14],[17417,56,1],
[17417,68,1],[17417,79,1],[17418,3,1],[17418,27,1],[17419,2,7],
[17419,3,1],[17419,13,1],[17419,20,2],[17419,21,1],[17419,31,7],
[17419,33,1],[17419,39,2],[17419,59,1],[17419,60,1],[17420,2,4],
[17421,3,3],[17421,42,2],[17421,44,1],[17421,47,1],[17421,59,1],
[17422,3,1],[17422,22,1],[17423,21,1],[17423,22,2],[17423,23,2],
[17423,25,11],[17423,27,1],[17423,32,1],[17423,33,3],[17423,43,1],
[17423,62,2],[17424,25,5],[17425,10,1],[17425,23,2],[17426,7,16],
[17426,29,1],[17427,5,1],[17427,23,1],[17427,27,1],[17427,62,1],
[17428,13,1],[17428,20,1],[17429,19,1],[17429,37,1],[17429,59,1],

[17430,17,3],[17430,18,1],[17430,36,1],[17430,53,5],[17430,55,4],
[17431,28,1],[17431,47,1],[17432,1,1],[17432,3,1],[17432,8,1],
[17432,20,2],[17432,24,4],[17432,27,1],[17432,30,5],[17432,43,1],
[17432,56,2],[17433,2,2],[17434,53,3],[17434,66,1],[17435,39,1],
[17435,53,1],[17436,27,1],[17436,28,5],[17437,15,3],[17437,55,2],
[17438,25,1],[17438,28,1],[17438,31,13],[17438,33,3],[17438,68,1],
[17439,12,1],[17439,53,1],[17440,3,1],[17440,15,1],[17440,20,1],
[17440,24,5],[17440,33,1],[17440,35,4],[17440,43,1],[17440,55,4],
[17441,44,1],[17441,66,1],[17442,28,1],[17442,70,1],[17443,42,1],
[17443,47,1],[17444,56,2],[17445,1,8],[17445,12,4],[17445,13,2],
[17445,17,1],[17445,29,1],[17445,30,1],[17445,39,12],[17445,53,1],
[17445,55,1],[17445,78,1],[17446,32,2],[17447,26,1],[17447,27,1],
[17447,43,3],[17447,62,2],[17448,27,3],[17449,9,1],[17449,57,1],
[17450,20,1],[17450,21,1],[17451,22,1],[17451,23,1],[17451,24,1],
[17451,25,1],[17451,27,1],[17451,32,1],[17451,35,1],[17451,38,1],
[17451,42,1],[17451,44,2],[17451,50,1],[17451,52,1],[17451,63,3],
[17451,70,1],[17451,72,1],[17451,75,1],[17451,76,2],[17452,25,5],
[17452,28,1],[17452,62,6],[17453,1,1],[17453,9,1],[17453,20,1],
[17453,78,1],[17454,31,2],[17455,1,1],[17455,15,4],[17455,20,3],
[17455,24,1],[17455,37,1],[17455,50,1],[17455,55,2],[17455,56,1],
[17455,65,1],[17455,73,1],[17455,74,1],[17456,12,2],[17457,71,1],
[17457,74,1],[17458,20,3],[17458,27,3],[17458,39,1],[17459,3,1],
[17459,10,1],[17459,20,1],[17459,24,1],[17459,38,1],[17459,74,1],
[17460,11,1],[17460,39,1],[17461,20,2],[17462,42,2],[17463,22,2],
[17463,23,2],[17463,24,2],[17463,25,1],[17463,27,2],[17463,28,35],
[17463,33,1],[17463,47,2],[17464,2,1],[17464,25,1],[17465,3,1],
[17465,70,1],[17466,4,1],[17466,28,2],[17466,74,2],[17467,20,1],
[17467,24,1],[17467,35,2],[17468,10,1],[17468,58,1],[17468,65,1],
[17469,56,1],[17469,74,1],[17470,63,1],[17470,72,1],[17471,1,1],
[17471,13,2],[17471,20,2],[17471,29,1],[17471,78,1],[17472,20,1],
[17472,22,6],[17472,23,1],[17472,27,4],[17472,28,1],[17472,35,6],
[17472,75,1],[17473,38,1],[17473,46,1],[17473,66,1],[17474,2,10],
[17474,5,1],[17475,11,1],[17475,12,3],[17475,28,4],[17475,31,1],
[17475,71,1],[17476,9,1],[17476,13,4],[17476,15,1],[17476,20,2],
[17476,23,1],[17476,28,3],[17476,47,1],[17476,51,2],[17476,59,1],
[17476,74,1],[17476,75,1],[17477,21,3],[17477,22,1],[17477,26,1],
[17477,27,2],[17477,43,3],[17478,3,1],[17478,21,1],[17478,22,1],
[17478,24,12],[17478,25,4],[17478,26,2],[17478,27,1],[17478,28,2],
[17478,31,2],[17478,33,1],[17478,35,1],[17478,39,1],[17478,42,5],
[17478,47,1],[17478,48,1],[17478,65,1],[17479,44,1],[17479,68,3],
[17480,28,1],[17480,38,1],[17481,5,1],[17481,21,1],[17482,9,3],
[17482,12,1],[17482,18,1],[17482,19,1],[17482,41,1],[17482,45,1],
[17482,46,1],[17482,49,1],[17482,58,1],[17482,59,1],[17482,65,2],
[17483,5,1],[17483,22,1],[17483,23,1],[17483,24,1],[17483,26,2],
[17483,27,2],[17483,31,1],[17483,32,1],[17483,35,2],[17483,38,1],
[17483,42,1],[17483,47,1],[17483,50,1],[17483,51,1],[17483,62,3],
[17483,66,1],[17484,21,1],[17484,24,2],[17485,9,1],[17485,10,3],
[17485,19,2],[17485,24,1],[17485,45,1],[17485,53,1],[17485,55,2],
[17486,26,1],[17486,64,1],[17487,24,1],[17487,31,2],[17488,20,1],
[17488,23,2],[17488,26,1],[17488,31,2],[17488,32,1],[17488,43,1],

[17489,4,1],[17489,28,1],[17489,66,1],[17490,23,1],[17490,24,1],
[17491,25,1],[17491,47,1],[17492,16,1],[17492,19,1],[17492,28,1],
[17492,45,1],[17492,75,1],[17493,31,2],[17494,24,2],[17495,5,2],
[17495,28,2],[17495,32,3],[17495,61,1],[17495,70,2],[17496,27,1],
[17496,73,1],[17497,22,3],[17497,70,1],[17498,15,2],[17499,23,1],
[17499,30,1],[17500,44,1],[17500,50,1],[17501,2,1],[17501,27,1],
[17501,47,1],[17502,1,3],[17503,8,1],[17503,27,2],[17503,28,2],
[17504,1,1],[17504,15,1],[17504,17,1],[17504,64,1],[17505,23,1],
[17505,35,1],[17506,28,13],[17507,2,1],[17507,50,1],[17508,13,2],
[17508,26,3],[17508,28,1],[17508,33,4],[17508,40,1],[17508,41,1],
[17508,48,7],[17508,66,1],[17508,78,1],[17509,37,1],[17509,73,1],
[17510,22,1],[17510,23,1],[17510,24,1],[17510,25,1],[17510,26,2],
[17510,27,1],[17510,28,1],[17510,30,2],[17510,43,2],[17510,47,1],
[17510,62,1],[17510,76,1],[17511,5,1],[17511,55,1],[17512,28,2],
[17512,32,2],[17513,3,8],[17513,5,1],[17513,15,1],[17513,16,1],
[17513,43,1],[17513,44,1],[17513,52,1],[17513,67,1],[17514,15,1],
[17514,17,1],[17515,21,1],[17515,42,1],[17516,21,1],[17516,23,1],
[17516,27,2],[17516,33,4],[17516,70,1],[17517,22,1],[17517,63,1],
[17518,27,1],[17518,31,1],[17518,43,1],[17518,68,1],[17519,40,3],
[17519,68,4],[17519,69,3],[17519,70,5],[17519,72,3],[17520,15,1],
[17520,28,2],[17521,1,1],[17521,2,1],[17521,20,1],[17521,29,1],
[17522,15,1],[17522,34,1],[17523,28,4],[17524,1,1],[17524,10,1],
[17524,13,1],[17524,15,1],[17524,19,1],[17524,34,1],[17524,55,2],
[17525,29,1],[17525,39,1],[17526,23,2],[17526,24,6],[17526,38,1],
[17527,0,1],[17527,4,1],[17527,32,6],[17527,35,5],[17528,2,1],
[17528,7,1],[17528,13,1],[17528,20,2],[17528,22,1],[17528,23,2],
[17528,24,1],[17528,31,1],[17528,43,2],[17528,62,1],[17529,15,2],
[17529,27,1],[17529,38,1],[17529,55,1],[17530,20,1],[17530,31,1],
[17530,32,1],[17530,72,1],[17531,21,2],[17532,1,52],[17532,2,14],
[17532,5,3],[17532,8,1],[17532,9,1],[17532,10,2],[17532,13,8],
[17532,19,1],[17532,20,10],[17532,21,2],[17532,22,3],[17532,25,1],
[17532,27,2],[17532,28,9],[17532,29,3],[17532,30,2],[17532,31,3],
[17532,32,2],[17532,33,1],[17532,35,2],[17532,36,1],[17532,38,2],
[17532,40,1],[17532,41,1],[17532,42,1],[17532,44,3],[17532,45,1],
[17532,49,1],[17532,50,1],[17532,60,1],[17532,62,1],[17532,63,2],
[17532,70,1],[17532,73,2],[17533,3,3],[17533,4,1],[17533,5,3],
[17533,6,1],[17533,22,6],[17533,26,1],[17533,27,1],[17533,32,1],
[17533,35,1],[17533,47,1],[17533,51,1],[17533,56,6],[17533,68,1],
[17533,70,1],[17534,14,1],[17534,20,1],[17535,35,1],[17535,50,1],
[17535,53,1],[17536,2,1],[17536,3,1],[17536,5,2],[17536,24,1],
[17537,23,1],[17537,48,1],[17538,1,5],[17538,7,1],[17538,12,1],
[17538,39,1],[17539,28,1],[17539,62,1],[17540,24,1],[17540,26,1],
[17540,28,3],[17540,32,1],[17540,66,1],[17540,72,1],[17541,23,1],
[17541,24,1],[17541,31,1],[17541,33,2],[17541,35,1],[17541,42,1],
[17542,9,2],[17543,28,1],[17543,35,1],[17544,26,1],[17544,42,1],
[17545,4,1],[17545,27,2],[17545,56,1],[17545,69,1],[17546,21,3],
[17546,22,3],[17546,23,1],[17546,24,1],[17546,25,2],[17546,27,2],
[17546,28,2],[17546,32,2],[17547,2,1],[17547,25,2],[17548,21,1],
[17548,43,1],[17549,28,1],[17549,31,15],[17549,32,1],[17550,21,1],
[17550,43,1],[17551,5,3],[17551,6,1],[17551,20,2],[17551,22,5],

[17551,23,8],[17551,26,2],[17551,27,2],[17551,28,1],[17551,31,3],
[17551,33,25],[17551,35,3],[17551,40,1],[17551,42,6],[17551,43,13],
[17551,47,3],[17551,48,2],[17551,50,1],[17551,62,2],[17551,63,1],
[17551,69,1],[17551,72,1],[17552,0,1],[17552,1,1],[17552,20,2],
[17552,30,3],[17552,40,1],[17552,46,1],[17552,55,1],[17552,62,1],
[17553,2,1],[17553,3,1],[17553,21,1],[17553,26,14],[17553,31,1],
[17553,32,1],[17553,33,2],[17553,43,3],[17553,48,1],[17553,56,7],
[17553,64,1],[17554,28,1],[17554,40,1],[17554,50,1],[17554,62,11],
[17554,70,1],[17555,9,3],[17555,34,1],[17555,45,1],[17555,58,1],
[17555,65,1],[17555,77,1],[17556,3,1],[17556,31,1],[17557,4,1],
[17557,13,1],[17557,19,2],[17557,20,1],[17557,24,2],[17557,28,1],
[17557,30,3],[17557,47,1],[17557,50,2],[17558,3,1],[17558,4,3],
[17558,5,1],[17558,14,1],[17558,25,1],[17558,26,1],[17558,28,2],
[17558,33,1],[17558,38,11],[17558,44,19],[17558,50,3],[17558,56,1],
[17558,66,21],[17559,4,1],[17559,9,1],[17559,39,2],[17560,25,1],
[17560,48,1],[17561,17,1],[17561,55,1],[17562,12,1],[17562,34,1],
[17562,46,1],[17563,27,2],[17564,1,1],[17564,2,1],[17564,3,9],
[17564,5,1],[17564,6,1],[17564,16,1],[17564,22,6],[17564,24,1],
[17564,26,1],[17564,30,2],[17564,31,1],[17564,32,1],[17564,33,3],
[17564,39,1],[17564,46,1],[17564,50,2],[17564,53,1],[17564,54,2],
[17564,56,2],[17564,58,1],[17564,60,1],[17564,62,1],[17564,69,2],
[17564,72,1],[17564,76,2],[17565,2,2],[17565,31,13],[17565,47,1],
[17566,39,1],[17566,41,1],[17566,59,2],[17567,0,1],[17567,1,10],
[17567,3,2],[17567,9,1],[17567,12,1],[17567,13,4],[17567,15,1],
[17567,24,8],[17567,41,1],[17567,43,1],[17567,77,1],[17568,14,1],
[17568,16,1],[17568,77,1],[17569,16,1],[17569,20,1],[17569,24,1],
[17569,67,1],[17570,2,4],[17570,3,1],[17570,20,1],[17570,47,1],
[17570,64,1],[17571,4,1],[17571,12,1],[17571,15,1],[17571,27,1],
[17571,32,1],[17571,35,1],[17571,46,1],[17571,48,1],[17571,53,1],
[17571,57,1],[17571,64,1],[17571,67,1],[17572,1,1],[17572,39,4],
[17573,27,1],[17573,28,1],[17573,35,17],[17574,2,1],[17574,3,1],
[17574,21,4],[17574,22,1],[17575,1,2],[17576,23,1],[17576,28,1],
[17576,35,1],[17577,0,1],[17577,7,2],[17577,10,1],[17577,13,1],
[17577,14,1],[17577,20,1],[17577,45,1],[17577,46,1],[17577,47,1],
[17577,64,1],[17578,20,3],[17579,25,1],[17579,28,1],[17579,31,1],
[17579,32,2],[17580,43,2],[17581,2,3],[17581,23,4],[17581,24,5],
[17581,33,8],[17581,35,1],[17581,38,1],[17581,42,1],[17581,43,1],
[17581,47,1],[17581,51,1],[17581,66,1],[17582,22,1],[17582,23,6],
[17582,24,2],[17582,43,1],[17582,66,1],[17582,76,1],[17583,62,4],
[17584,15,1],[17584,17,1],[17585,6,3],[17585,22,2],[17585,26,2],
[17585,27,5],[17585,35,1],[17585,42,1],[17586,50,1],[17586,76,2],
[17587,21,1],[17587,23,1],[17587,26,4],[17587,27,1],[17587,31,2],
[17587,42,1],[17587,43,1],[17588,2,1],[17588,28,1],[17589,3,1],
[17589,27,1],[17590,9,1],[17590,17,1],[17591,15,3],[17591,34,1],
[17591,55,1],[17592,22,1],[17592,40,1],[17593,3,18],[17593,6,4],
[17593,12,1],[17593,21,1],[17593,23,1],[17593,26,1],[17593,28,1],
[17593,30,1],[17593,32,1],[17593,42,1],[17593,47,3],[17593,62,2],
[17593,64,1],[17594,9,1],[17594,23,2],[17595,63,3],[17595,74,2],
[17595,75,1],[17595,76,3],[17596,15,1],[17596,21,3],[17596,31,1],
[17596,44,1],[17596,51,4],[17596,61,1],[17596,66,2],[17596,70,1],

[17596,72,1],[17596,74,1],[17597,23,2],[17597,26,1],[17597,27,3],
[17597,35,3],[17597,52,1],[17597,70,1],[17598,17,2],[17598,28,6],
[17598,50,1],[17598,53,1],[17598,59,1],[17599,28,2],[17599,43,1],
[17600,1,1],[17600,57,1],[17601,27,2],[17602,2,1],[17602,23,2],
[17602,31,1],[17602,70,1],[17603,21,1],[17603,24,1],[17604,21,1],
[17604,22,1],[17605,15,1],[17605,37,3],[17605,42,2],[17605,53,1],
[17605,55,1],[17605,76,1],[17606,1,1],[17606,7,1],[17606,10,1],
[17606,12,1],[17606,15,2],[17606,20,1],[17606,37,1],[17606,41,1],
[17606,49,1],[17606,73,1],[17606,77,1],[17607,6,1],[17607,7,1],
[17607,8,2],[17607,9,5],[17607,10,1],[17607,15,1],[17607,17,2],
[17607,28,1],[17607,31,2],[17607,32,1],[17607,34,1],[17607,37,1],
[17607,39,1],[17607,45,1],[17607,49,1],[17607,53,1],[17607,55,1],
[17607,67,1],[17607,74,1],[17608,13,1],[17608,24,11],[17608,25,1],
[17608,28,1],[17608,62,1],[17609,25,33],[17609,28,1],[17609,70,1],
[17610,2,2],[17610,28,1],[17610,56,1],[17611,28,2],[17612,2,1],
[17612,22,1],[17612,24,1],[17612,26,1],[17612,28,4],[17612,31,1],
[17612,33,2],[17612,48,1],[17612,62,2],[17612,68,1],[17612,69,1],
[17612,70,1],[17613,2,1],[17613,4,1],[17613,24,1],[17613,27,3],
[17613,28,3],[17614,3,2],[17614,74,1],[17615,1,4],[17615,2,1],
[17615,13,2],[17615,19,1],[17615,20,2],[17615,27,2],[17615,40,1],
[17615,59,2],[17615,62,6],[17616,30,1],[17616,71,1],[17617,22,1],
[17617,27,1],[17617,28,1],[17618,9,1],[17618,13,1],[17618,23,1],
[17618,34,2],[17618,55,1],[17618,57,1],[17618,67,1],[17619,23,1],
[17619,44,1],[17620,28,7],[17621,10,1],[17621,37,1],[17621,39,1],
[17621,55,1],[17622,2,15],[17622,5,2],[17622,6,1],[17622,8,1],
[17622,17,1],[17622,21,1],[17622,22,6],[17622,23,31],[17622,24,3],
[17622,25,1],[17622,26,4],[17622,27,8],[17622,28,3],[17622,31,3],
[17622,32,3],[17622,33,1],[17622,34,1],[17622,35,3],[17622,38,1],
[17622,42,1],[17622,43,5],[17622,47,3],[17622,53,2],[17622,62,2],
[17622,63,1],[17622,66,2],[17622,68,1],[17622,70,1],[17623,63,1],
[17623,74,2],[17623,76,1],[17624,28,2],[17625,26,1],[17625,50,1],
[17626,28,1],[17626,31,1],[17627,28,7],[17627,38,1],[17627,52,17],
[17628,44,4],[17629,23,2],[17629,25,2],[17629,27,3],[17629,28,1],
[17630,3,2],[17630,8,1],[17630,20,1],[17630,22,1],[17630,23,1],
[17630,25,2],[17630,27,5],[17630,28,4],[17630,35,3],[17630,39,1],
[17630,70,1],[17631,1,1],[17631,2,1],[17631,5,4],[17631,13,1],
[17631,20,2],[17631,28,1],[17631,55,1],[17631,77,1],[17632,27,2],
[17632,28,1],[17632,32,2],[17632,33,2],[17632,40,1],[17632,47,1],
[17633,35,1],[17633,74,1],[17634,4,6],[17634,5,1],[17634,6,1],
[17634,25,1],[17634,26,1],[17634,29,1],[17634,44,7],[17634,50,4],
[17634,66,7],[17635,23,3],[17635,31,1],[17635,35,2],[17635,62,1],
[17636,2,8],[17636,5,3],[17636,13,1],[17636,30,1],[17636,59,1],
[17636,60,2],[17636,70,2],[17637,1,3],[17637,2,2],[17637,6,1],
[17637,12,1],[17637,13,12],[17637,20,1],[17637,22,2],[17637,23,4],
[17637,24,2],[17637,25,1],[17637,27,2],[17637,29,1],[17637,31,1],
[17637,33,1],[17637,34,2],[17637,35,3],[17637,36,1],[17637,39,1],
[17637,41,1],[17637,42,1],[17637,44,1],[17637,50,1],[17637,56,2],
[17638,2,3],[17639,22,1],[17639,24,1],[17640,23,1],[17640,31,1],
[17641,16,1],[17641,20,1],[17641,35,1],[17641,47,1],[17641,69,1],
[17641,70,1],[17642,32,6],[17643,0,2],[17643,2,14],[17643,31,2],

[17643,46,1],[17644,21,1],[17644,28,1],[17644,74,1],[17645,1,2],
[17645,6,1],[17645,29,1],[17645,79,1],[17646,2,2],[17646,5,2],
[17646,23,3],[17646,24,1],[17646,27,2],[17646,28,3],[17646,30,1],
[17646,74,1],[17646,75,1],[17647,2,1],[17647,31,1],[17647,32,1],
[17647,35,1],[17648,17,1],[17648,76,1],[17649,1,1],[17649,12,1],
[17650,8,1],[17650,10,1],[17651,2,1],[17651,17,2],[17651,23,1],
[17651,31,2],[17651,42,3],[17651,44,1],[17651,50,1],[17652,3,8],
[17652,4,1],[17652,5,1],[17652,6,13],[17652,22,3],[17652,23,17],
[17652,24,82],[17652,26,2],[17652,28,23],[17652,33,2],[17652,38,3],
[17652,42,1],[17652,47,5],[17652,48,1],[17652,56,25],[17652,62,2],
[17652,68,1],[17652,70,1],[17652,74,1],[17653,15,2],[17654,24,1],
[17654,32,1],[17654,35,1],[17654,48,1],[17654,76,1],[17655,27,1],
[17655,35,2],[17656,1,2],[17656,13,2],[17656,24,2],[17656,31,1],
[17657,2,2],[17657,47,1],[17658,22,1],[17658,23,2],[17658,27,1],
[17658,47,1],[17658,59,1],[17659,18,1],[17659,50,1],[17659,53,1],
[17660,12,1],[17660,38,1],[17661,5,1],[17661,6,1],[17661,23,1],
[17661,56,2],[17661,63,2],[17662,19,1],[17662,23,1],[17662,34,1],
[17663,3,1],[17663,5,1],[17663,21,1],[17663,22,1],[17663,24,1],
[17663,38,12],[17663,43,1],[17663,47,1],[17663,74,1],[17664,28,1],
[17664,74,1],[17665,61,1],[17665,68,1],[17665,70,1],[17666,13,2],
[17666,20,1],[17666,39,1],[17667,28,1],[17667,32,1],[17668,8,1],
[17668,12,1],[17668,13,1],[17668,17,1],[17668,34,1],[17669,43,7],
[17670,22,1],[17670,31,1],[17670,48,1],[17671,8,1],[17671,17,1],
[17671,20,2],[17671,21,2],[17671,23,2],[17671,25,1],[17671,27,5],
[17671,28,1],[17671,29,1],[17671,31,2],[17671,32,4],[17671,33,1],
[17671,34,1],[17671,43,1],[17671,44,1],[17672,4,8],[17672,5,1],
[17672,9,1],[17672,15,4],[17672,16,1],[17672,17,8],[17672,23,1],
[17672,24,2],[17672,25,1],[17672,32,1],[17672,44,1],[17672,50,11],
[17672,53,20],[17672,55,10],[17672,66,1],[17673,2,1],[17673,3,1],
[17673,5,1],[17673,6,1],[17673,22,1],[17673,23,1],[17673,26,1],
[17673,27,4],[17673,28,1],[17673,31,1],[17674,15,2],[17674,17,1],
[17674,53,2],[17675,5,1],[17675,15,1],[17675,24,2],[17675,28,2],
[17675,32,1],[17675,33,1],[17675,38,1],[17675,41,1],[17675,43,1],
[17675,44,1],[17675,45,1],[17675,58,2],[17676,63,1],[17676,74,2],
[17676,75,2],[17676,76,1],[17677,1,3],[17677,3,1],[17677,5,1],
[17677,13,4],[17677,17,1],[17677,20,22],[17677,21,2],[17677,23,8],
[17677,24,3],[17677,25,2],[17677,26,4],[17677,27,5],[17677,28,5],
[17677,31,6],[17677,32,11],[17677,33,1],[17677,34,2],[17677,35,8],
[17677,37,5],[17677,39,1],[17677,42,3],[17677,47,1],[17677,57,1],
[17677,59,3],[17677,65,1],[17677,73,1],[17677,76,1],[17678,28,32],
[17678,74,1],[17678,76,1],[17679,26,1],[17679,28,1],[17680,24,1],
[17680,40,1],[17681,10,1],[17681,62,1],[17682,28,1],[17682,44,1],
[17682,47,1],[17682,53,1],[17683,77,3],[17684,26,1],[17684,29,1],
[17684,53,1],[17685,4,2],[17685,27,3],[17685,32,7],[17685,33,1],
[17685,47,1],[17686,38,4],[17687,50,1],[17687,53,1],[17688,0,1],
[17688,8,1],[17688,13,3],[17688,17,1],[17688,73,1],[17689,24,1],
[17689,26,1],[17690,0,2],[17690,1,11],[17690,7,1],[17690,8,4],
[17690,9,27],[17690,10,24],[17690,11,3],[17690,12,25],[17690,13,8],
[17690,14,6],[17690,15,18],[17690,16,8],[17690,17,8],[17690,18,6],
[17690,19,17],[17690,20,19],[17690,29,7],[17690,30,9],[17690,34,6],

[17690,36,2],[17690,37,8],[17690,39,5],[17690,41,7],[17690,45,10],
[17690,46,6],[17690,49,7],[17690,53,15],[17690,54,3],[17690,55,14],
[17690,57,8],[17690,58,5],[17690,59,9],[17690,60,2],[17690,64,4],
[17690,65,17],[17690,67,4],[17690,71,3],[17690,73,8],[17690,77,9],
[17690,78,5],[17691,2,1],[17691,23,1],[17691,27,2],[17691,32,1],
[17691,50,1],[17691,70,1],[17692,9,1],[17692,15,1],[17692,55,1],
[17692,77,1],[17693,30,3],[17694,0,1],[17694,35,2],[17695,3,1],
[17695,12,1],[17695,26,1],[17695,27,1],[17696,0,4],[17696,1,16],
[17696,2,4],[17696,8,1],[17696,9,2],[17696,10,2],[17696,12,5],
[17696,13,37],[17696,15,3],[17696,18,1],[17696,19,1],[17696,20,1],
[17696,21,65],[17696,23,174],[17696,24,1],[17696,25,19],[17696,26,46],
[17696,28,18],[17696,29,2],[17696,31,240],[17696,32,1],[17696,33,23],
[17696,34,1],[17696,38,1],[17696,39,5],[17696,40,1],[17696,42,83],
[17696,43,192],[17696,45,1],[17696,50,3],[17696,51,1],[17696,57,2],
[17696,58,2],[17696,59,1],[17696,62,9],[17696,64,1],[17696,74,1],
[17696,76,1],[17696,77,4],[17697,5,1],[17697,32,7],[17698,12,1],
[17698,15,1],[17698,55,1],[17699,62,2],[17700,22,1],[17700,23,3],
[17700,28,12],[17700,35,1],[17701,27,2],[17702,3,1],[17702,28,1],
[17703,4,1],[17703,28,1],[17704,2,6],[17704,3,7],[17704,4,3],
[17704,5,2],[17704,6,1],[17704,9,1],[17704,10,1],[17704,21,3],
[17704,22,2],[17704,23,5],[17704,24,5],[17704,25,6],[17704,26,5],
[17704,27,8],[17704,28,33],[17704,31,3],[17704,32,3],[17704,33,2],
[17704,34,2],[17704,35,2],[17704,37,1],[17704,38,4],[17704,40,44],
[17704,41,1],[17704,43,3],[17704,44,9],[17704,47,4],[17704,50,2],
[17704,51,1],[17704,52,1],[17704,53,1],[17704,54,1],[17704,56,4],
[17704,58,1],[17704,61,2],[17704,62,2],[17704,63,15],[17704,65,1],
[17704,66,4],[17704,68,2557],[17704,69,101],[17704,70,156],
[17704,72,119],[17704,74,12],[17704,75,16],[17704,76,17],[17705,4,1],
[17705,13,1],[17705,17,1],[17705,24,1],[17705,53,4],[17706,10,1],
[17706,14,1],[17707,50,1],[17707,66,1],[17708,5,1],[17708,23,1],
[17708,24,1],[17708,25,1],[17708,26,1],[17708,27,1],[17708,28,1],
[17708,32,1],[17708,43,1],[17708,46,1],[17709,14,1],[17709,16,1],
[17709,18,1],[17709,35,1],[17709,37,14],[17709,48,1],[17709,56,2],
[17709,61,1],[17709,70,1],[17709,75,1],[17709,76,2],[17709,78,5],
[17710,28,2],[17711,74,1],[17711,75,3],[17712,23,1],[17712,28,1],
[17712,55,1],[17712,58,1],[17712,76,3],[17713,3,1],[17713,8,1],
[17713,20,1],[17713,31,1],[17713,32,2],[17714,28,1],[17714,44,1],
[17715,26,1],[17715,63,6],[17715,74,8],[17715,75,6],[17715,76,4],
[17716,3,1],[17716,6,1],[17716,15,1],[17716,16,1],[17716,23,1],
[17716,24,1],[17716,38,1],[17716,45,2],[17716,68,1],[17717,34,2],
[17717,48,1],[17718,35,2],[17719,35,5],[17719,61,1],[17720,24,1],
[17720,56,1],[17720,61,12],[17720,63,1],[17721,23,1],[17721,28,1],
[17722,3,1],[17722,22,1],[17722,23,1],[17722,26,2],[17722,32,2],
[17722,62,1],[17723,55,3],[17724,24,4],[17724,27,13],[17724,35,1],
[17724,40,1],[17725,3,2],[17725,6,7],[17725,21,1],[17725,22,4],
[17725,23,11],[17725,24,5],[17725,26,3],[17725,27,16],[17725,28,1],
[17725,32,1],[17725,33,8],[17725,35,15],[17725,38,1],[17725,42,4],
[17725,43,2],[17725,46,1],[17725,48,1],[17725,50,2],[17725,56,1],
[17725,62,1],[17725,66,2],[17725,70,1],[17725,74,5],[17725,76,1],
[17726,5,1],[17726,15,2],[17726,55,1],[17727,28,1],[17727,32,2],

[17728,2,2],[17728,27,2],[17728,31,7],[17729,30,2],[17729,65,1],
[17730,39,1],[17730,69,1],[17731,5,4],[17731,21,4],[17731,42,1],
[17731,47,1],[17731,50,1],[17731,66,2],[17732,9,1],[17732,34,1],
[17733,3,1],[17733,22,8],[17733,23,75],[17733,24,1],[17733,28,1],
[17733,31,1],[17733,38,2],[17733,40,1],[17733,47,1],[17733,63,1],
[17733,68,1],[17733,70,1],[17733,72,1],[17733,76,1],[17734,1,1],
[17734,15,1],[17734,17,2],[17734,30,1],[17734,53,4],[17734,55,2],
[17735,1,4],[17735,3,2],[17735,6,8],[17735,7,1],[17735,8,1],
[17735,9,1],[17735,10,1],[17735,12,4],[17735,13,2],[17735,14,1],
[17735,15,7],[17735,16,1],[17735,18,1],[17735,22,1],[17735,26,12],
[17735,27,4],[17735,28,1],[17735,30,1],[17735,31,1],[17735,34,2],
[17735,36,1],[17735,38,8],[17735,40,1],[17735,46,2],[17735,53,3],
[17735,54,1],[17735,55,4],[17735,57,1],[17735,58,1],[17735,59,4],
[17735,62,3],[17735,65,2],[17735,70,1],[17735,73,1],[17735,77,1],
[17736,1,5],[17736,7,1],[17736,39,2],[17737,29,1],[17737,30,1],
[17738,21,1],[17738,32,2],[17738,62,1],[17739,2,2],[17740,4,1],
[17740,28,3],[17741,2,1],[17741,5,1],[17741,15,1],[17741,16,1],
[17741,25,5],[17741,45,1],[17741,55,5],[17741,59,1],[17742,21,1],
[17742,27,1],[17742,38,1],[17742,69,1],[17743,2,2],[17744,2,3],
[17744,30,1],[17745,3,1],[17745,27,1],[17745,28,6],[17745,32,1],
[17745,35,1],[17745,40,1],[17745,48,1],[17745,70,1],[17746,25,2],
[17746,26,1],[17746,32,1],[17746,51,1],[17746,52,1],[17747,17,1],
[17747,27,1],[17747,35,1],[17747,56,1],[17748,2,1],[17748,30,2],
[17748,43,1],[17748,48,3],[17749,5,1],[17749,9,1],[17749,20,2],
[17749,23,2],[17749,27,2],[17749,28,1],[17749,32,5],[17749,34,1],
[17749,35,2],[17749,43,1],[17749,47,1],[17749,52,1],[17750,27,3],
[17750,28,1],[17751,21,5],[17751,22,1],[17751,25,2],[17751,26,10],
[17751,31,6],[17751,33,1],[17751,43,4],[17751,48,1],[17751,52,1],
[17751,56,1],[17752,0,4],[17752,1,8],[17752,7,2],[17752,8,3],
[17752,9,26],[17752,10,34],[17752,11,8],[17752,12,12],[17752,13,9],
[17752,14,6],[17752,15,3],[17752,16,20],[17752,17,3],[17752,18,8],
[17752,19,30],[17752,20,2],[17752,24,1],[17752,25,1],[17752,28,3],
[17752,29,1],[17752,32,1],[17752,33,3],[17752,34,4],[17752,36,9],
[17752,37,7],[17752,39,2],[17752,41,15],[17752,45,17],[17752,46,14],
[17752,49,8],[17752,53,3],[17752,54,10],[17752,55,6],[17752,57,7],
[17752,58,14],[17752,59,8],[17752,60,2],[17752,61,1],[17752,62,1],
[17752,63,1],[17752,64,2],[17752,65,19],[17752,66,1],[17752,67,8],
[17752,69,1],[17752,71,6],[17752,73,10],[17752,74,2],[17752,77,11],
[17752,78,8],[17752,79,2],[17753,1,1],[17753,3,2],[17753,21,1],
[17753,23,2],[17753,28,4],[17753,31,1],[17753,32,1],[17753,33,13],
[17753,38,9],[17753,42,1],[17753,52,47],[17753,74,1],[17754,25,1],
[17754,26,1],[17754,56,4],[17755,24,1],[17755,33,2],[17756,2,7],
[17756,28,2],[17756,60,1],[17756,65,1],[17756,76,1],[17757,10,1],
[17757,34,1],[17757,59,1],[17758,26,1],[17758,59,1],[17759,37,1],
[17759,47,1],[17760,27,3],[17760,39,1],[17760,76,1],[17761,26,3],
[17762,22,1],[17762,23,2],[17762,26,1],[17763,28,5],[17763,55,2],
[17763,74,1],[17764,23,2],[17764,35,1],[17765,22,1],[17765,23,1],
[17765,27,1],[17765,35,2],[17765,47,1],[17766,15,2],[17767,13,2],
[17767,15,1],[17767,16,1],[17767,34,1],[17767,43,1],[17767,45,1],
[17767,55,1],[17767,56,1],[17768,10,1],[17768,19,1],[17768,73,3],

[17769,32,3],[17770,2,1],[17770,42,1],[17771,1,2],[17772,3,1],
[17772,33,1],[17773,13,1],[17773,15,1],[17773,16,1],[17773,23,2],
[17773,25,1],[17773,27,1],[17773,28,1],[17774,13,2],[17774,23,3],
[17774,24,1],[17775,8,5],[17775,27,1],[17775,29,1],[17775,62,1],
[17776,4,1],[17776,50,1],[17777,19,1],[17777,56,3],[17777,75,1],
[17778,10,1],[17778,16,1],[17778,36,1],[17778,57,1],[17778,65,1],
[17778,71,1],[17779,22,1],[17779,27,1],[17779,65,1],[17779,68,1],
[17779,76,2],[17780,23,1],[17780,32,1],[17781,9,3],[17781,10,1],
[17781,11,1],[17781,14,1],[17781,15,1],[17781,19,1],[17781,41,2],
[17781,45,1],[17781,57,4],[17781,58,1],[17781,60,1],[17781,65,2],
[17781,73,1],[17781,77,2],[17782,0,1],[17782,2,12],[17782,8,1],
[17782,20,2],[17782,22,2],[17782,23,2],[17782,24,2],[17782,26,1],
[17782,27,1],[17782,28,2],[17782,31,1],[17782,32,1],[17782,34,1],
[17782,42,1],[17782,43,2],[17782,44,1],[17782,46,1],[17782,47,4],
[17782,56,1],[17782,72,1],[17782,76,2],[17783,27,1],[17783,28,1],
[17783,47,1],[17783,58,1],[17784,2,5],[17784,42,3],[17784,47,1],
[17785,28,1],[17785,70,1],[17786,5,1],[17786,22,1],[17786,27,2],
[17787,28,1],[17787,38,1],[17788,32,3],[17788,33,2],[17789,24,1],
[17789,71,1],[17790,15,2],[17790,17,2],[17790,53,2],[17790,55,3],
[17791,25,1],[17791,35,1],[17791,39,8],[17791,68,1],[17792,23,1],
[17792,27,4],[17792,44,1],[17792,48,1],[17793,3,2],[17793,6,1],
[17793,9,2],[17793,20,2],[17793,21,1],[17793,23,1],[17793,28,1],
[17793,32,2],[17793,35,2],[17793,39,3],[17793,42,1],[17793,45,1],
[17793,47,1],[17793,68,1],[17794,3,2],[17794,23,1],[17794,25,1],
[17794,35,1],[17795,28,1],[17795,50,1],[17796,2,8],[17796,31,1],
[17797,2,146],[17797,20,2],[17797,21,1],[17797,28,1],[17797,31,1],
[17797,47,1],[17797,59,3],[17797,62,1],[17797,68,1],[17798,1,2],
[17798,5,3],[17798,15,3],[17798,20,1],[17798,27,1],[17798,33,1],
[17798,40,1],[17798,43,1],[17798,44,1],[17798,47,1],[17798,55,5],
[17798,69,1],[17798,74,1],[17798,76,1],[17799,3,1],[17799,28,1],
[17799,38,1],[17799,75,1],[17800,2,2],[17800,31,6],[17800,39,2],
[17801,26,1],[17801,28,3],[17801,35,2],[17802,31,2],[17802,47,1],
[17803,1,1],[17803,9,1],[17803,12,5],[17804,22,1],[17804,48,1],
[17805,9,2],[17805,18,1],[17805,54,1],[17805,65,1],[17805,77,3],
[17806,15,1],[17806,65,1],[17807,1,3],[17807,33,1],[17808,21,1],
[17808,28,12],[17808,31,1],[17809,4,2],[17810,9,1],[17810,10,2],
[17810,16,1],[17810,18,1],[17810,19,1],[17810,20,2],[17810,27,1],
[17810,31,1],[17810,32,1],[17810,34,1],[17810,37,1],[17810,41,3],
[17810,53,1],[17810,54,2],[17810,65,1],[17810,67,1],[17811,36,1],
[17811,41,1],[17812,1,6],[17812,8,1],[17812,12,1],[17812,13,11],
[17812,17,2],[17812,20,1],[17812,30,5],[17812,34,2],[17812,36,1],
[17812,47,1],[17812,50,1],[17812,73,1],[17813,3,1],[17813,29,2],
[17814,16,1],[17814,24,1],[17814,27,1],[17814,28,1],[17814,30,1],
[17814,32,1],[17814,34,2],[17815,27,1],[17815,28,1],[17815,49,1],
[17816,59,1],[17816,64,1],[17817,4,14],[17817,5,3],[17817,22,1],
[17817,28,5],[17817,32,1],[17817,38,4],[17817,42,1],[17817,44,45],
[17817,50,17],[17817,66,42],[17817,70,2],[17817,72,1],[17817,75,1],
[17817,76,1],[17818,10,1],[17818,67,1],[17819,1,1],[17819,13,1],
[17819,15,1],[17819,20,1],[17819,23,2],[17819,56,1],[17820,23,1],
[17820,33,2],[17821,3,6],[17822,19,1],[17822,22,1],[17822,49,1],

[17822,65,1],[17823,5,1],[17823,25,2],[17824,5,1],[17824,35,1],
[17825,1,1],[17825,29,1],[17826,1,2],[17826,11,1],[17826,29,1],
[17826,65,1],[17827,2,3]],"rows": [{"id": "denovo3", "metadata":
{"taxonomy": ["Bacteria", "__Chlorobi", "__Chlorobia",
"__Chlorobiales", "__Chlorobiaceae", "__Prosthecochloris"]}},{"id":
"denovo4", "metadata": {"taxonomy": ["Bacteria", "__Proteobacteria",
"__Gammaproteobacteria", "__Thiotrichales", "__EV818SWSAP88",
"__g"]}},{"id": "denovo7", "metadata": {"taxonomy": ["Bacteria",
"__Verrucomicrobia", "__Verrucomicrobiae", "__Verrucomicrobiales",
"__Verrucomicrobiaceae", "__Persicirhabdus"]}},{"id": "denovo9",
"metadata": {"taxonomy": ["Bacteria", "__Actinobacteria",
"__Actinobacteria", "__Kineosporiales", "__Kineosporiaceae",
"__Kineococcus"]}},{"id": "denovo13", "metadata": {"taxonomy":
["Bacteria", "__Proteobacteria", "__Alphaproteobacteria",
"__Rhodobacterales", "__Rhodobacteraceae"]}},{"id": "denovo14",
"metadata": {"taxonomy": ["Bacteria", "__Proteobacteria",
"__Alphaproteobacteria", "__Rhodospirillales",
"__Rhodospirillaceae"]}},{"id": "denovo20", "metadata": {"taxonomy":
["Bacteria", "__Proteobacteria", "__Gammaproteobacteria",
"__Alteromonadales", "__Alteromonadaceae", "__g"]}},{"id": "denovo23",
"metadata": {"taxonomy": ["Bacteria", "__Planctomycetes",
"__Phycisphaerae", "__Phycisphaerales", "__Phycisphaeraceae",
"__Phycisphaera"]}},{"id": "denovo24", "metadata": {"taxonomy":
["Bacteria", "__Proteobacteria", "__Gammaproteobacteria", "__BD3-1",
"__f", "__g"]}},{"id": "denovo27", "metadata": {"taxonomy":
["Bacteria", "__Proteobacteria", "__Deltaproteobacteria", "__Sh765B-
TzT-29", "__f", "__g"]}},{"id": "denovo30", "metadata": {"taxonomy":
["Bacteria", "__Proteobacteria", "__Alphaproteobacteria",
"__Caulobacterales", "__Caulobacteraceae", "__g"]}},{"id": "denovo33",
"metadata": {"taxonomy": ["Bacteria", "__Proteobacteria",
"__Gammaproteobacteria", "__Thiotrichales", "__Piscirickettsiaceae",
"__Piscirickettsia"]}},{"id": "denovo35", "metadata": {"taxonomy":
["Bacteria", "__Proteobacteria", "__Gammaproteobacteria",
"__Legionellales", "__Coxiellaceae", "__Coxiella"]}},{"id":
"denovo36", "metadata": {"taxonomy": ["Bacteria", "__BD1-5", "__c",
"__o", "__f", "__g"]}},{"id": "denovo38", "metadata": {"taxonomy":
["Bacteria", "__Planctomycetes", "__Phycisphaerae",
"__Phycisphaerales", "__Phycisphaeraceae",
"__Urania-1B-19_marine_sediment_group"]}},{"id": "denovo40",
"metadata": {"taxonomy": ["Bacteria", "__Planctomycetes",
"__Planctomycetacia", "__Planctomycetales", "__Planctomycetaceae",
"__Pir4_lineage"]}},{"id": "denovo42", "metadata": {"taxonomy":
["Bacteria", "__Proteobacteria", "__Alphaproteobacteria",
"__Rhodobacterales", "__Rhodobacteraceae", "__Tateyamaria"]}},{"id":
"denovo46", "metadata": {"taxonomy": ["Bacteria", "__Proteobacteria",
"__Gammaproteobacteria", "__Xanthomonadales", "__Sinobacteraceae",
"__JTB255_marine_benthic_group"]}},{"id": "denovo47", "metadata":
{"taxonomy": ["Bacteria", "__Planctomycetes", "__Phycisphaerae",
"__Phycisphaerales", "__Phycisphaeraceae", "__Phycisphaera"]}},{"id":
"denovo48", "metadata": {"taxonomy": ["Bacteria", "__Proteobacteria",

"__Alphaproteobacteria", "__Rhizobiales", "__Hyphomicrobiaceae", "__Pedomicrobium"]}},{"id": "denovo51", "metadata": {"taxonomy": ["Bacteria", "__Proteobacteria", "__Gammaproteobacteria", "__Alteromonadales", "__Alteromonadaceae", "__BD1-7_clade"]}},{"id": "denovo53", "metadata": {"taxonomy": ["Bacteria", "__Proteobacteria", "__Gammaproteobacteria", "__Order_Incertae_Sedis", "__Family_Incertae_Sedis", "__Marinicella"]}},{"id": "denovo56", "metadata": {"taxonomy": ["Bacteria", "__Proteobacteria", "__Alphaproteobacteria", "__Rhodospirillales", "__Rhodospirillaceae", "__Defluviicoccus"]}},{"id": "denovo59", "metadata": {"taxonomy": ["Bacteria", "__TM6", "__c", "__o", "__f", "__g"]}},{"id": "denovo63", "metadata": {"taxonomy": ["Bacteria", "__Proteobacteria", "__Deltaproteobacteria", "__Desulfobacterales", "__Desulfobacteraceae", "__Sva0081_sediment_group"]}},{"id": "denovo65", "metadata": {"taxonomy": ["Bacteria", "__Planctomycetes", "__Phycisphaerae", "__S-70", "__f", "__g"]}},{"id": "denovo66", "metadata": {"taxonomy": ["Bacteria", "__Actinobacteria", "__Acidimicrobiia", "__Acidimicrobiales", "__uncultured", "__g"]}},{"id": "denovo67", "metadata": {"taxonomy": ["Bacteria", "__Proteobacteria", "__Gammaproteobacteria", "__Oceanospirillales", "__OM182_clade", "__g"]}},{"id": "denovo70", "metadata": {"taxonomy": ["Bacteria", "__Acidobacteria", "__Holophagae", "__NS72", "__f", "__g"]}},{"id": "denovo72", "metadata": {"taxonomy": ["Bacteria", "__Proteobacteria", "__Alphaproteobacteria", "__Rhodobacterales", "__Rhodobacteraceae"]}},{"id": "denovo73", "metadata": {"taxonomy": ["Bacteria", "__Proteobacteria", "__Alphaproteobacteria", "__Rhodobacterales", "__Rhodobacteraceae"]}},{"id": "denovo77", "metadata": {"taxonomy": ["Bacteria", "__Proteobacteria", "__Alphaproteobacteria", "__Rhodospirillales", "__Rhodospirillaceae", "__Thalassospira"]}},{"id": "denovo81", "metadata": {"taxonomy": ["Bacteria", "__Proteobacteria", "__Gammaproteobacteria", "__NKB5", "__f", "__g"]}},{"id": "denovo84", "metadata": {"taxonomy": ["Bacteria", "__Proteobacteria", "__Gammaproteobacteria", "__Xanthomonadales", "__Sinobacteraceae", "__JTB255_marine_benthic_group"]}},{"id": "denovo86", "metadata": {"taxonomy": ["Bacteria", "__Proteobacteria", "__Gammaproteobacteria", "__Alteromonadales"]}},{"id": "denovo87", "metadata": {"taxonomy": ["Bacteria", "__Proteobacteria", "__Gammaproteobacteria", "__Oceanospirillales", "__Hahellaceae", "__Endozoicomonas"]}},{"id": "denovo89", "metadata": {"taxonomy": ["Bacteria", "__Bacteroidetes", "__Sphingobacteriia", "__Sphingobacteriales", "__Saprospiraceae", "__g"]}},{"id": "denovo91", "metadata": {"taxonomy": ["Bacteria", "__Bacteroidetes", "__Cytophagia", "__Cytophagales", "__Flammeovirgaceae", "__Flexithrix"]}},{"id": "denovo92", "metadata": {"taxonomy": ["Bacteria", "__Proteobacteria", "__Gammaproteobacteria", "__Oceanospirillales", "__Alcanivoracaceae", "__Kangiella"]}},{"id": "denovo94", "metadata": {"taxonomy": ["Bacteria", "__Proteobacteria", "__Gammaproteobacteria"]}},{"id": "denovo95", "metadata": {"taxonomy": ["Bacteria", "__Proteobacteria", "__Gammaproteobacteria", "__Oceanospirillales", "__Oleiphilaceae", "__Oleiphilus"]}},{"id":

"denovo96", "metadata": {"taxonomy": ["Bacteria", "__Proteobacteria", "__Deltaproteobacteria", "__Desulfobacterales", "__Nitrospinaceae", "__Nitrospina"]}},{"id": "denovo100", "metadata": {"taxonomy": ["Bacteria", "__Proteobacteria", "__Alphaproteobacteria", "__Rhodospirillales", "__Rhodospirillaceae", "__g"]}},{"id": "denovo102", "metadata": {"taxonomy": ["Bacteria", "__Chloroflexi", "__SAR202_clade", "__o", "__f", "__g"]}},{"id": "denovo103", "metadata": {"taxonomy": ["Bacteria", "__Proteobacteria", "__Alphaproteobacteria", "__Rhodobacterales", "__Rhodobacteraceae", "__Ruegeria"]}},{"id": "denovo105", "metadata": {"taxonomy": ["Bacteria", "__Actinobacteria", "__Acidimicrobiia", "__Acidimicrobiales", "__OCS155_marine_group", "__g"]}},{"id": "denovo108", "metadata": {"taxonomy": ["Bacteria", "__Bacteroidetes", "__Sphingobacteriia", "__Sphingobacteriales", "__Saprospiraceae", "__g"]}},{"id": "denovo109", "metadata": {"taxonomy": ["Bacteria", "__Proteobacteria", "__Deltaproteobacteria", "__Bdellovibrionales", "__Bdellovibrionaceae", "__OM27_clade"]}},{"id": "denovo111", "metadata": {"taxonomy": ["Bacteria", "__Proteobacteria", "__Gammaproteobacteria", "__Alteromonadales", "__Alteromonadaceae", "__Candidatus_Endobugula"]}},{"id": "denovo112", "metadata": {"taxonomy": ["Bacteria", "__Bacteroidetes", "__Sphingobacteriia", "__Sphingobacteriales", "__Saprospiraceae", "__g"]}},{"id": "denovo116", "metadata": {"taxonomy": ["Bacteria", "__Bacteroidetes", "__Flavobacteria", "__Flavobacteriales", "__Cryomorphaceae", "__Owenweeksia"]}},{"id": "denovo119", "metadata": {"taxonomy": ["Bacteria", "__Candidate_division_WS3", "__c", "__o", "__f", "__g"]}},{"id": "denovo121", "metadata": {"taxonomy": ["Bacteria", "__Proteobacteria", "__Alphaproteobacteria", "__Rhodobacterales", "__Rhodobacteraceae"]}},{"id": "denovo122", "metadata": {"taxonomy": ["Bacteria", "__Proteobacteria", "__Alphaproteobacteria", "__Rhodospirillales"]}},{"id": "denovo125", "metadata": {"taxonomy": ["Bacteria", "__Cyanobacteria", "__Cyanobacteria", "__uncultured", "__f", "__g"]}},{"id": "denovo127", "metadata": {"taxonomy": ["Bacteria", "__Proteobacteria", "__Gammaproteobacteria", "__Order_Incertae_Sedis", "__Family_Incertae_Sedis", "__Marinicella"]}},{"id": "denovo131", "metadata": {"taxonomy": ["Bacteria", "__Proteobacteria", "__Gammaproteobacteria", "__Alteromonadales", "__Colwelliaceae", "__Thalassomonas"]}},{"id": "denovo138", "metadata": {"taxonomy": ["Bacteria", "__Bacteroidetes"]}},{"id": "denovo140", "metadata": {"taxonomy": ["Bacteria", "__Proteobacteria", "__SPOTSOCT00m83", "__o", "__f", "__g"]}},{"id": "denovo145", "metadata": {"taxonomy": ["Bacteria", "__Bacteroidetes", "__Flavobacteria", "__Flavobacteriales", "__Flavobacteriaceae", "__Meridianimaribacter"]}},{"id": "denovo157", "metadata": {"taxonomy": ["Bacteria", "__Bacteroidetes", "__Bacteroidia", "__Bacteroidales", "__Marinilabiaceae", "__g"]}}, {"id": "denovo162", "metadata": {"taxonomy": ["Bacteria", "__Gemmatimonadetes", "__Gemmatimonadetes", "__Kazan-1B-37", "__f", "__g"]}},{"id": "denovo164", "metadata": {"taxonomy": ["Bacteria", "__Proteobacteria", "__Gammaproteobacteria", "__Pseudomonadales",

"__Moraxellaceae", "__Acinetobacter"]}},{"id": "denovo166",
"metadata": {"taxonomy": ["Bacteria", "__Proteobacteria",
"__Gammaproteobacteria", "__Alteromonadales", "__Alteromonadaceae"]}},
{"id": "denovo175", "metadata": {"taxonomy": ["Bacteria",
"__Proteobacteria", "__Alphaproteobacteria", "__Rhodobacterales",
"__Rhodobacteraceae", "__Ruegeria"]}},{"id": "denovo180", "metadata":
{"taxonomy": ["Bacteria", "__Proteobacteria", "__Alphaproteobacteria",
"__Rhodospirillales", "__Rhodospirillaceae", "__Thalassospira"]}},
{"id": "denovo182", "metadata": {"taxonomy": ["Bacteria",
"__Proteobacteria", "__Deltaproteobacteria", "__Bdellovibrionales",
"__Bacteriovoracaceae", "__Peredibacter"]}},{"id": "denovo185",
"metadata": {"taxonomy": ["Bacteria", "__Bacteroidetes"]}},{"id":
"denovo187", "metadata": {"taxonomy": ["Bacteria", "__Proteobacteria",
"__Gammaproteobacteria", "__Chromatiales", "__Chromatiaceae",
"__Nitrosococcus"]}},{"id": "denovo188", "metadata": {"taxonomy":
["Unclassified"]}},{"id": "denovo190", "metadata": {"taxonomy":
["Bacteria", "__Proteobacteria", "__Deltaproteobacteria", "__GR-
WP33-30", "__f", "__g"]}},{"id": "denovo191", "metadata": {"taxonomy":
["Bacteria", "__Proteobacteria", "__Alphaproteobacteria", "__DB1-14",
"__f", "__g"]}},{"id": "denovo193", "metadata": {"taxonomy":
["Bacteria", "__Proteobacteria", "__Alphaproteobacteria",
"__SAR11_clade", "__Surface_1", "__Candidatus_Pelagibacter"]}},{"id":
"denovo199", "metadata": {"taxonomy": ["Bacteria", "__Proteobacteria",
"__Deltaproteobacteria", "__Bdellovibrionales",
"__Bdellovibrionaceae", "__Bdellovibrio"]}},{"id": "denovo203",
"metadata": {"taxonomy": ["Bacteria", "__TM6", "__c", "__o", "__f",
"__g"]}},{"id": "denovo204", "metadata": {"taxonomy": ["Bacteria",
"__Proteobacteria", "__Alphaproteobacteria", "__Rhodobacterales",
"__Rhodobacteraceae", "__Tropicimonas"]}},{"id": "denovo211",
"metadata": {"taxonomy": ["Bacteria", "__Proteobacteria",
"__Alphaproteobacteria", "__Rhizobiales", "__Hyphomicrobiaceae",
"__Hyphomicrobium"]}},{"id": "denovo212", "metadata": {"taxonomy":
["Bacteria", "__Proteobacteria", "__Alphaproteobacteria",
"__Rhodobacterales", "__Rhodobacteraceae", "__g"]}},{"id":
"denovo214", "metadata": {"taxonomy": ["Bacteria", "__Proteobacteria",
"__Deltaproteobacteria", "__Desulfobacterales",
"__Desulfobacteraceae", "__Desulfofaba"]}},{"id": "denovo216",
"metadata": {"taxonomy": ["Bacteria", "__Proteobacteria",
"__Gammaproteobacteria", "__Alteromonadales", "__Colwelliaceae",
"__Thalassomonas"]}},{"id": "denovo218", "metadata": {"taxonomy":
["Bacteria", "__Deferribacteres", "__Deferribacteres",
"__Deferribacterales", "__PAUC34f", "__g"]}},{"id": "denovo222",
"metadata": {"taxonomy": ["Bacteria", "__Proteobacteria",
"__Deltaproteobacteria", "__Sh765B-TzT-29", "__f", "__g"]}},{"id":
"denovo224", "metadata": {"taxonomy": ["Bacteria", "__Proteobacteria",
"__Alphaproteobacteria"]}},{"id": "denovo225", "metadata":
{"taxonomy": ["Bacteria", "__Proteobacteria", "__Alphaproteobacteria",
"__Caulobacterales", "__Hyphomonadaceae", "__g"]}},{"id": "denovo230",
"metadata": {"taxonomy": ["Bacteria", "__Proteobacteria",
"__Alphaproteobacteria", "__Rhodobacterales", "__Rhodobacteraceae"]}},

{"id": "denovo234", "metadata": {"taxonomy": ["Bacteria", "__Proteobacteria", "__Alphaproteobacteria", "__Rhizobiales", "__Phyllobacteriaceae", "__Phyllobacterium"]}},{"id": "denovo237", "metadata": {"taxonomy": ["Bacteria", "__Proteobacteria", "__Alphaproteobacteria"]}},{"id": "denovo238", "metadata": {"taxonomy": ["Bacteria", "__Proteobacteria", "__Gammaproteobacteria"]}},{"id": "denovo239", "metadata": {"taxonomy": ["Bacteria", "__Proteobacteria", "__Alphaproteobacteria", "__Rhodospirillales", "__Rhodospirillaceae", "__Pelagibius"]}},{"id": "denovo241", "metadata": {"taxonomy": ["Bacteria", "__Chloroflexi", "__Caldilineae", "__Caldilineales", "__Caldilineaceae", "__Caldilinea"]}},{"id": "denovo242", "metadata": {"taxonomy": ["Bacteria", "__Proteobacteria", "__CF2", "__o", "__f", "__g"]}},{"id": "denovo243", "metadata": {"taxonomy": ["Bacteria", "__Proteobacteria", "__Gammaproteobacteria", "__Pseudomonadales", "__Moraxellaceae", "__Acinetobacter"]}},{"id": "denovo248", "metadata": {"taxonomy": ["Bacteria", "__Cyanobacteria", "__Cyanobacteria", "__SubsectionIII", "__FamilyI", "__Pseudanabaena"]}},{"id": "denovo251", "metadata": {"taxonomy": ["Bacteria", "__Fusobacteria", "__Fusobacteria", "__MSBL6", "__f", "__g"]}},{"id": "denovo257", "metadata": {"taxonomy": ["Bacteria", "__Proteobacteria", "__Gammaproteobacteria", "__Alteromonadales", "__Alteromonadaceae", "__Candidatus_Endobugula"]}},{"id": "denovo262", "metadata": {"taxonomy": ["Bacteria", "__Proteobacteria", "__Gammaproteobacteria", "__HOC36", "__f", "__g"]}},{"id": "denovo264", "metadata": {"taxonomy": ["Bacteria", "__Proteobacteria", "__Gammaproteobacteria", "__Enterobacteriales", "__Enterobacteriaceae", "__Shimwellia"]}},{"id": "denovo265", "metadata": {"taxonomy": ["Bacteria", "__Bacteroidetes", "__Sphingobacteriia", "__Sphingobacteriales", "__Saprospiraceae", "__g"]}},{"id": "denovo267", "metadata": {"taxonomy": ["Bacteria", "__Proteobacteria", "__Gammaproteobacteria", "__KI89A_clade", "__f", "__g"]}},{"id": "denovo269", "metadata": {"taxonomy": ["Bacteria", "__Proteobacteria", "__Deltaproteobacteria", "__Desulfobacterales", "__Desulfobulbaceae", "__Desulfobulbus"]}},{"id": "denovo273", "metadata": {"taxonomy": ["Bacteria"]}},{"id": "denovo274", "metadata": {"taxonomy": ["Bacteria", "__Chloroflexi", "__SAR202_clade", "__o", "__f", "__g"]}},{"id": "denovo277", "metadata": {"taxonomy": ["Bacteria", "__Proteobacteria", "__Gammaproteobacteria", "__Oceanospirillales", "__Hahellaceae", "__Endozoicomonas"]}},{"id": "denovo278", "metadata": {"taxonomy": ["Bacteria", "__Chlamydiae", "__Chlamydiae", "__Chlamydiales", "__Simkaniaceae"]}},{"id": "denovo282", "metadata": {"taxonomy": ["Bacteria", "__Proteobacteria", "__Alphaproteobacteria", "__Rhodobacterales", "__Rhodobacteraceae", "__Pelagicola"]}},{"id": "denovo284", "metadata": {"taxonomy": ["Bacteria", "__Proteobacteria", "__Gammaproteobacteria", "__Oceanospirillales", "__Alcanivoracaceae", "__Kangiella"]}},{"id": "denovo288", "metadata": {"taxonomy": ["Bacteria", "__Proteobacteria", "__Alphaproteobacteria", "__Rhodospirillales", "__Rhodospirillaceae",

"__AEGEAN-169_marine_group"]}},{"id": "denovo289", "metadata": {"taxonomy": ["Bacteria", "__Proteobacteria", "__Alphaproteobacteria", "__Rhizobiales", "__Rhizobiaceae", "__Rhizobium"]}},{"id": "denovo290", "metadata": {"taxonomy": ["Bacteria", "__Proteobacteria", "__Alphaproteobacteria", "__Rhizobiales", "__Phyllobacteriaceae", "__Nitratireductor"]}},{"id": "denovo291", "metadata": {"taxonomy": ["Bacteria", "__Proteobacteria", "__Deltaproteobacteria", "__Myxococcales", "__0319-6G20", "__g"]}},{"id": "denovo292", "metadata": {"taxonomy": ["Bacteria", "__Bacteroidetes", "__Sphingobacteriia", "__Sphingobacteriales", "__Saprospiraceae", "__g"]}},{"id": "denovo296", "metadata": {"taxonomy": ["Bacteria", "__Proteobacteria", "__Alphaproteobacteria", "__Rhodospirillales", "__Rhodospirillaceae", "__g"]}},{"id": "denovo297", "metadata": {"taxonomy": ["Bacteria", "__Proteobacteria", "__Deltaproteobacteria", "__Myxococcales", "__0319-6G20", "__g"]}},{"id": "denovo298", "metadata": {"taxonomy": ["Bacteria", "__Proteobacteria", "__Deltaproteobacteria", "__Bdellovibrionales", "__Bacteriovoracaceae", "__Peredibacter"]}},{"id": "denovo300", "metadata": {"taxonomy": ["Bacteria", "__Proteobacteria", "__Alphaproteobacteria", "__Sphingomonadales", "__Erythrobacteraceae"]}},{"id": "denovo301", "metadata": {"taxonomy": ["Bacteria", "__Proteobacteria", "__Deltaproteobacteria", "__Sh765B-TzT-29", "__f", "__g"]}},{"id": "denovo302", "metadata": {"taxonomy": ["Bacteria", "__Proteobacteria", "__Alphaproteobacteria", "__DB1-14", "__f", "__g"]}},{"id": "denovo303", "metadata": {"taxonomy": ["Bacteria", "__Proteobacteria", "__Alphaproteobacteria", "__Rhodospirillales", "__MSB-1E8", "__g"]}},{"id": "denovo304", "metadata": {"taxonomy": ["Bacteria", "__Cyanobacteria", "__SHA-109", "__o", "__f", "__g"]}},{"id": "denovo311", "metadata": {"taxonomy": ["Bacteria", "__Cyanobacteria", "__Cyanobacteria", "__SubsectionIII", "__FamilyI", "__Phormidium"]}},{"id": "denovo313", "metadata": {"taxonomy": ["Bacteria", "__Bacteroidetes", "__Flavobacteria", "__Flavobacteriales", "__Flavobacteriaceae", "__NS4_marine_group"]}},{"id": "denovo314", "metadata": {"taxonomy": ["Bacteria", "__Acidobacteria", "__Acidobacteria", "__BPC102", "__f", "__g"]}},{"id": "denovo315", "metadata": {"taxonomy": ["Bacteria", "__Proteobacteria", "__Gammaproteobacteria", "__CS-B046", "__f", "__g"]}},{"id": "denovo316", "metadata": {"taxonomy": ["Bacteria", "__Bacteroidetes", "__Flavobacteria", "__Flavobacteriales", "__Flavobacteriaceae", "__Formosa"]}},{"id": "denovo317", "metadata": {"taxonomy": ["Bacteria", "__Verrucomicrobia", "__Verrucomicrobiae", "__Verrucomicrobiales", "__DEV007", "__g"]}},{"id": "denovo318", "metadata": {"taxonomy": ["Bacteria", "__Chloroflexi", "__Anaerolineae", "__Anaerolineales", "__Anaerolineaceae", "__g"]}},{"id": "denovo319", "metadata": {"taxonomy": ["Bacteria", "__Proteobacteria", "__Alphaproteobacteria", "__Rhodobacterales", "__Rhodobacteraceae"]}},{"id": "denovo320", "metadata": {"taxonomy": ["Bacteria", "__Proteobacteria", "__Alphaproteobacteria", "__Rhodobacterales", "__Rhodobacteraceae", "__Loktanella"]}},{"id": "denovo324", "metadata": {"taxonomy": ["Bacteria", "__Proteobacteria",

"__Alphaproteobacteria", "__Rhizobiales", "__Phyllobacteriaceae", "__Cohaesibacter"]}},{"id": "denovo327", "metadata": {"taxonomy": ["Bacteria", "__Proteobacteria", "__Alphaproteobacteria", "__4-Org1-14", "__f", "__g"]}},{"id": "denovo328", "metadata": {"taxonomy": ["Bacteria", "__Proteobacteria", "__Deltaproteobacteria", "__Desulfobacterales", "__Desulfobacteraceae", "__g"]}},{"id": "denovo330", "metadata": {"taxonomy": ["Bacteria", "__Proteobacteria", "__Gammaproteobacteria", "__Thiotrichales"]}},{"id": "denovo332", "metadata": {"taxonomy": ["Bacteria", "__Proteobacteria", "__Gammaproteobacteria", "__Xanthomonadales", "__Sinobacteraceae", "__JTB255_marine_benthic_group"]}},{"id": "denovo333", "metadata": {"taxonomy": ["Bacteria", "__Chlorobi", "__Ignavibacteria", "__Ignavibacteriales", "__IheB3-7", "__g"]}},{"id": "denovo337", "metadata": {"taxonomy": ["Bacteria", "__Proteobacteria", "__Alphaproteobacteria", "__Rhodospirillales", "__Rhodospirillaceae", "__Defluviicoccus"]}},{"id": "denovo338", "metadata": {"taxonomy": ["Bacteria"]}},{"id": "denovo340", "metadata": {"taxonomy": ["Bacteria", "__Proteobacteria", "__Alphaproteobacteria", "__Rickettsiales", "__SAR116_clade", "__g"]}},{"id": "denovo341", "metadata": {"taxonomy": ["Bacteria", "__Proteobacteria", "__Deltaproteobacteria", "__Sh765B-TzT-29", "__f", "__g"]}},{"id": "denovo355", "metadata": {"taxonomy": ["Bacteria", "__Bacteroidetes", "__Flavobacteria", "__Flavobacteriales", "__Flavobacteriaceae", "__Chryseobacterium"]}},{"id": "denovo356", "metadata": {"taxonomy": ["Bacteria", "__Proteobacteria", "__Gammaproteobacteria"]}},{"id": "denovo359", "metadata": {"taxonomy": ["Bacteria", "__Proteobacteria", "__Alphaproteobacteria", "__Rhizobiales"]}},{"id": "denovo362", "metadata": {"taxonomy": ["Bacteria", "__Tenericutes", "__Mollicutes", "__Candidatus_Phytoplasma", "__f", "__g"]}},{"id": "denovo363", "metadata": {"taxonomy": ["Bacteria", "__Chlorobi", "__Chlorobia", "__Chlorobiales", "__OPB56", "__g"]}},{"id": "denovo365", "metadata": {"taxonomy": ["Bacteria", "__Bacteroidetes", "__Cytophagia", "__Order_III_Incertae_Sedis", "__B2706-C7", "__g"]}},{"id": "denovo366", "metadata": {"taxonomy": ["Bacteria", "__Proteobacteria", "__Alphaproteobacteria", "__DB1-14", "__f", "__g"]}},{"id": "denovo371", "metadata": {"taxonomy": ["Bacteria", "__Acidobacteria", "__RB25", "__o", "__f", "__g"]}},{"id": "denovo373", "metadata": {"taxonomy": ["Bacteria", "__Proteobacteria", "__Gammaproteobacteria", "__Oceanospirillales", "__Hahellaceae", "__Endozoicomonas"]}},{"id": "denovo374", "metadata": {"taxonomy": ["Bacteria", "__Chlorobi", "__Chlorobia", "__Chlorobiales", "__OPB56", "__g"]}},{"id": "denovo375", "metadata": {"taxonomy": ["Bacteria", "__Proteobacteria", "__Alphaproteobacteria", "__Rhizobiales", "__Rhizobiaceae", "__Shinella"]}},{"id": "denovo380", "metadata": {"taxonomy": ["Bacteria", "__Proteobacteria", "__Alphaproteobacteria", "__Caulobacterales", "__Caulobacteraceae", "__Brevundimonas"]}},{"id": "denovo381", "metadata": {"taxonomy": ["Bacteria", "__Proteobacteria", "__Gammaproteobacteria", "__HOC36", "__f", "__g"]}},{"id": "denovo388", "metadata": {"taxonomy": ["Bacteria", "__Proteobacteria", "__Gammaproteobacteria", "__E01-9C-26_marine_group", "__f", "__g"]}},

{"id": "denovo389", "metadata": {"taxonomy": ["Bacteria", "__Bacteroidetes"]}},{"id": "denovo390", "metadata": {"taxonomy": ["Bacteria", "__Proteobacteria", "__Alphaproteobacteria", "__Sphingomonadales", "__Sphingomonadaceae", "__Sphingomonas"]}}, {"id": "denovo391", "metadata": {"taxonomy": ["Bacteria", "__Proteobacteria", "__Betaproteobacteria", "__Neisseriales", "__Neisseriaceae", "__Neisseria"]}},{"id": "denovo395", "metadata": {"taxonomy": ["Bacteria", "__Bacteroidetes", "__Flavobacteria", "__Flavobacteriales", "__Flavobacteriaceae", "__Polaribacter"]}}, {"id": "denovo397", "metadata": {"taxonomy": ["Bacteria", "__Proteobacteria", "__Alphaproteobacteria"]}},{"id": "denovo399", "metadata": {"taxonomy": ["Bacteria", "__Proteobacteria", "__Alphaproteobacteria", "__SB1-18", "__f", "__g"]}},{"id": "denovo406", "metadata": {"taxonomy": ["Bacteria", "__Proteobacteria", "__Alphaproteobacteria", "__Rhodospirillales", "__Rhodospirillaceae", "__Defluviicoccus"]}},{"id": "denovo408", "metadata": {"taxonomy": ["Bacteria", "__Bacteroidetes", "__Flavobacteria", "__Flavobacteriales", "__Flavobacteriaceae", "__NS5_marine_group"]}}, {"id": "denovo410", "metadata": {"taxonomy": ["Bacteria", "__Proteobacteria", "__Deltaproteobacteria", "__Sh765B-TzT-29", "__f", "__g"]}},{"id": "denovo413", "metadata": {"taxonomy": ["Bacteria", "__Proteobacteria", "__Gammaproteobacteria", "__NKB5", "__f", "__g"]}},{"id": "denovo414", "metadata": {"taxonomy": ["Bacteria", "__Bacteroidetes", "__Flavobacteria", "__Flavobacteriales", "__Cryomorphaceae", "__Owenweeksia"]}},{"id": "denovo415", "metadata": {"taxonomy": ["Bacteria", "__Bacteroidetes", "__Flavobacteria", "__Flavobacteriales", "__Flavobacteriaceae"]}},{"id": "denovo418", "metadata": {"taxonomy": ["Bacteria", "__Acidobacteria", "__Acidobacteria", "__DA023", "__f", "__g"]}},{"id": "denovo423", "metadata": {"taxonomy": ["Bacteria", "__Proteobacteria", "__Alphaproteobacteria", "__Rhodobacterales", "__Rhodobacteraceae", "__Roseovarius"]}},{"id": "denovo429", "metadata": {"taxonomy": ["Bacteria", "__Proteobacteria", "__Betaproteobacteria", "__Rhodocyclales", "__Rhodocyclaceae", "__Methyloversatilis"]}},{"id": "denovo430", "metadata": {"taxonomy": ["Bacteria", "__Cyanobacteria", "__Cyanobacteria", "__SubsectionIII", "__FamilyI"]}},{"id": "denovo432", "metadata": {"taxonomy": ["Bacteria", "__Proteobacteria", "__Alphaproteobacteria", "__Rhizobiales", "__Phyllobacteriaceae", "__g"]}},{"id": "denovo433", "metadata": {"taxonomy": ["Bacteria", "__Firmicutes", "__Clostridia", "__Clostridiales", "__Clostridiaceae"]}},{"id": "denovo434", "metadata": {"taxonomy": ["Bacteria", "__Proteobacteria", "__Gammaproteobacteria"]}},{"id": "denovo438", "metadata": {"taxonomy": ["Bacteria", "__Proteobacteria", "__Alphaproteobacteria", "__Rhodobacterales", "__Rhodobacteraceae"]}}, {"id": "denovo443", "metadata": {"taxonomy": ["Bacteria", "__Proteobacteria", "__Alphaproteobacteria", "__Rhodobacterales", "__Rhodobacteraceae", "__Roseobacter_clade_NAC11-7_lineage"]}},{"id": "denovo449", "metadata": {"taxonomy": ["Bacteria", "__Proteobacteria", "__Alphaproteobacteria", "__Rhodospirillales", "__Rhodospirillaceae", "__g"]}},{"id": "denovo455", "metadata": {"taxonomy": ["Bacteria",

"__Proteobacteria", "__Gammaproteobacteria", "__aaa34a10", "__f", "__g"]}},{"id": "denovo456", "metadata": {"taxonomy": ["Bacteria", "__Acidobacteria", "__Holophagae", "__CA002", "__f", "__g"]}},{"id": "denovo457", "metadata": {"taxonomy": ["Bacteria", "__Acidobacteria", "__Holophagae", "__CA002", "__f", "__g"]}},{"id": "denovo460", "metadata": {"taxonomy": ["Bacteria", "__Proteobacteria", "__Alphaproteobacteria", "__Rhizobiales", "__Hyphomicrobiaceae", "__g"]}},{"id": "denovo461", "metadata": {"taxonomy": ["Bacteria", "__Proteobacteria", "__Gammaproteobacteria", "__Xanthomonadales", "__Sinobacteraceae", "__g"]}},{"id": "denovo462", "metadata": {"taxonomy": ["Bacteria", "__Cyanobacteria", "__Cyanobacteria"]}},{"id": "denovo465", "metadata": {"taxonomy": ["Bacteria", "__Proteobacteria", "__JTB23", "__o", "__f", "__g"]}},{"id": "denovo467", "metadata": {"taxonomy": ["Bacteria", "__Proteobacteria", "__Deltaproteobacteria", "__Myxococcales", "__Sorangiineae", "__g"]}},{"id": "denovo468", "metadata": {"taxonomy": ["Bacteria", "__Cyanobacteria", "__Cyanobacteria", "__SubsectionII"]}},{"id": "denovo470", "metadata": {"taxonomy": ["Bacteria", "__Proteobacteria", "__Alphaproteobacteria", "__DB1-14", "__f", "__g"]}},{"id": "denovo473", "metadata": {"taxonomy": ["Bacteria", "__Proteobacteria", "__Alphaproteobacteria", "__Rhizobiales", "__Phyllobacteriaceae"]}},{"id": "denovo476", "metadata": {"taxonomy": ["Bacteria", "__Proteobacteria", "__Alphaproteobacteria", "__Rhodobacterales", "__Rhodobacteraceae"]}},{"id": "denovo477", "metadata": {"taxonomy": ["Bacteria", "__Proteobacteria", "__Alphaproteobacteria"]}},{"id": "denovo478", "metadata": {"taxonomy": ["Bacteria", "__Planctomycetes", "__Planctomycetacia", "__Planctomycetales", "__Planctomycetaceae", "__Blastopirellula"]}},{"id": "denovo480", "metadata": {"taxonomy": ["Bacteria", "__Proteobacteria", "__AEGEAN-245", "__o", "__f", "__g"]}},{"id": "denovo483", "metadata": {"taxonomy": ["Bacteria", "__Proteobacteria", "__Deltaproteobacteria", "__Sh765B-TzT-29", "__f", "__g"]}},{"id": "denovo484", "metadata": {"taxonomy": ["Bacteria", "__Proteobacteria", "__Gammaproteobacteria", "__Alteromonadales", "__Alteromonadaceae", "__Haliea"]}},{"id": "denovo487", "metadata": {"taxonomy": ["Bacteria", "__Bacteroidetes", "__Cytophagia", "__Cytophagales", "__Flammeovirgaceae"]}},{"id": "denovo489", "metadata": {"taxonomy": ["Bacteria", "__Proteobacteria", "__Betaproteobacteria", "__Methylophilales", "__Methylophilaceae", "__OM43_clade"]}},{"id": "denovo494", "metadata": {"taxonomy": ["Bacteria", "__Proteobacteria", "__Deltaproteobacteria", "__Desulfobacterales", "__Desulfobacteraceae", "__Desulfosalsimonas"]}},{"id": "denovo495", "metadata": {"taxonomy": ["Bacteria", "__Bacteroidetes", "__Cytophagia", "__Cytophagales", "__Flammeovirgaceae", "__g"]}},{"id": "denovo496", "metadata": {"taxonomy": ["Bacteria", "__Candidate_division_WS3", "__c", "__o", "__f", "__g"]}},{"id": "denovo498", "metadata": {"taxonomy": ["Bacteria", "__Proteobacteria", "__Gammaproteobacteria", "__Oceanospirillales", "__Hahellaceae", "__Endozoicomonas"]}},{"id": "denovo499", "metadata": {"taxonomy": ["Bacteria", "__Acidobacteria", "__RB25", "__o", "__f", "__g"]}},{"id": "denovo503", "metadata":

{"taxonomy": ["Bacteria", "__Bacteroidetes", "__Sphingobacteriia", "__Sphingobacteriales", "__Saprospiraceae", "__g"]}},{"id": "denovo504", "metadata": {"taxonomy": ["Archaea", "__Euryarchaeota", "__Halobacteria", "__Halobacteriales", "__Deep_Sea_Hydrothermal_Vent_Gp_6(DHVEG-6)", "__g"]}},{"id": "denovo506", "metadata": {"taxonomy": ["Bacteria", "__Bacteroidetes", "__Flavobacteria", "__Flavobacteriales", "__Flavobacteriaceae", "__Chryseobacterium"]}},{"id": "denovo511", "metadata": {"taxonomy": ["Bacteria", "__Bacteroidetes", "__Flavobacteria", "__Flavobacteriales", "__Cryomorphaceae", "__Owenweeksia"]}},{"id": "denovo512", "metadata": {"taxonomy": ["Bacteria", "__Acidobacteria", "__Holophagae", "__CA002", "__f", "__g"]}},{"id": "denovo514", "metadata": {"taxonomy": ["Bacteria", "__Proteobacteria", "__Alphaproteobacteria"]}},{"id": "denovo516", "metadata": {"taxonomy": ["Bacteria", "__Proteobacteria", "__Alphaproteobacteria", "__Rhodobacterales", "__Rhodobacteraceae", "__Phaeobacter"]}},{"id": "denovo517", "metadata": {"taxonomy": ["Bacteria", "__Planctomycetes", "__Pla3_lineage", "__o", "__f", "__g"]}},{"id": "denovo519", "metadata": {"taxonomy": ["Bacteria", "__Bacteroidetes", "__Flavobacteria", "__Flavobacteriales", "__Flavobacteriaceae"]}},{"id": "denovo521", "metadata": {"taxonomy": ["Bacteria", "__Proteobacteria", "__Gammaproteobacteria", "__Xanthomonadales", "__Sinobacteraceae", "__JTB255_marine_benthic_group"]}},{"id": "denovo522", "metadata": {"taxonomy": ["Bacteria", "__Proteobacteria", "__Gammaproteobacteria", "__Alteromonadales", "__Celerinatantimonadaceae", "__Celerinatantimonas"]}},{"id": "denovo525", "metadata": {"taxonomy": ["Bacteria", "__Proteobacteria", "__Deltaproteobacteria", "__Myxococcales", "__Haliangiaceae", "__Haliangium"]}},{"id": "denovo528", "metadata": {"taxonomy": ["Bacteria", "__Proteobacteria", "__Alphaproteobacteria", "__OCS116_clade", "__f", "__g"]}},{"id": "denovo529", "metadata": {"taxonomy": ["Bacteria", "__Proteobacteria", "__Gammaproteobacteria", "__Pseudomonadales", "__Moraxellaceae", "__Acinetobacter"]}},{"id": "denovo530", "metadata": {"taxonomy": ["Bacteria", "__Acidobacteria", "__Holophagae", "__Sva0725", "__f", "__g"]}},{"id": "denovo535", "metadata": {"taxonomy": ["Bacteria", "__Proteobacteria", "__Epsilonproteobacteria", "__Campylobacterales", "__Helicobacteraceae", "__Sulfurovum"]}},{"id": "denovo536", "metadata": {"taxonomy": ["Bacteria", "__Proteobacteria", "__Deltaproteobacteria", "__Myxococcales", "__Cystobacterineae", "__g"]}},{"id": "denovo539", "metadata": {"taxonomy": ["Bacteria", "__Actinobacteria", "__Actinobacteria", "__Corynebacteriales", "__uncultured", "__g"]}},{"id": "denovo540", "metadata": {"taxonomy": ["Bacteria", "__Proteobacteria", "__Alphaproteobacteria", "__Rhodobacterales", "__Rhodobacteraceae", "__Rubribacterium"]}},{"id": "denovo544", "metadata": {"taxonomy": ["Bacteria", "__Actinobacteria", "__Actinobacteria", "__Micrococcales", "__Brevibacteriaceae", "__Brevibacterium"]}},{"id": "denovo545", "metadata": {"taxonomy": ["Unclassified"]}},{"id": "denovo546", "metadata": {"taxonomy": ["Bacteria", "__Proteobacteria",

"__Deltaproteobacteria", "__Desulfuromonadales", "__GR-WP33-58",
"__g"]}},{"id": "denovo549", "metadata": {"taxonomy": ["Bacteria",
"__Proteobacteria", "__Gammaproteobacteria"]}},{"id": "denovo550",
"metadata": {"taxonomy": ["Bacteria", "__Proteobacteria",
"__Gammaproteobacteria", "__Sva0071", "__f", "__g"]}},{"id":
"denovo551", "metadata": {"taxonomy": ["Bacteria", "__Proteobacteria",
"__Alphaproteobacteria", "__DB1-14", "__f", "__g"]}},{"id":
"denovo552", "metadata": {"taxonomy": ["Unclassified"]}},{"id":
"denovo558", "metadata": {"taxonomy": ["Bacteria", "__Bacteroidetes",
"__Cytophagia", "__Cytophagales", "__Flammeovirgaceae",
"__Persicobacter"]}},{"id": "denovo560", "metadata": {"taxonomy":
["Bacteria", "__Bacteroidetes", "__Cytophagia",
"__Order_II_Incertae_Sedis", "__Rhodothermaceae", "__Rubricoccus"]}},
{"id": "denovo563", "metadata": {"taxonomy": ["Bacteria",
"__Acidobacteria", "__Holophagae", "__CA002", "__f", "__g"]}},{"id":
"denovo566", "metadata": {"taxonomy": ["Bacteria", "__Proteobacteria",
"__Gammaproteobacteria", "__Alteromonadales", "__Alteromonadaceae",
"__BD1-7_clade"]}},{"id": "denovo575", "metadata": {"taxonomy":
["Bacteria"]}},{"id": "denovo576", "metadata": {"taxonomy":
["Bacteria", "__Chloroflexi", "__SAR202_clade", "__o", "__f",
"__g"]}},{"id": "denovo578", "metadata": {"taxonomy": ["Bacteria",
"__Verrucomicrobia", "__Arctic97B-4_marine_group", "__o", "__f",
"__g"]}},{"id": "denovo586", "metadata": {"taxonomy": ["Bacteria",
"__Actinobacteria", "__Actinobacteria", "__Corynebacteriales",
"__Corynebacteriaceae", "__Corynebacterium"]}},{"id": "denovo587",
"metadata": {"taxonomy": ["Bacteria", "__Proteobacteria",
"__Gammaproteobacteria"]}},{"id": "denovo589", "metadata":
{"taxonomy": ["Bacteria", "__Chloroflexi", "__Caldilineae",
"__Caldilineales", "__Caldilineaceae", "__Caldilinea"]}},{"id":
"denovo590", "metadata": {"taxonomy": ["Bacteria", "__Proteobacteria",
"__Alphaproteobacteria", "__Rhodospirillales"]}},{"id": "denovo594",
"metadata": {"taxonomy": ["Bacteria", "__Cyanobacteria",
"__Cyanobacteria", "__SubsectionI", "__FamilyI", "__Synechococcus"]}},
{"id": "denovo597", "metadata": {"taxonomy": ["Bacteria",
"__Bacteroidetes", "__Flavobacteria", "__Flavobacteriales",
"__Flavobacteriaceae", "__Algibacter"]}},{"id": "denovo599",
"metadata": {"taxonomy": ["Bacteria", "__Bacteroidetes",
"__Flavobacteria", "__Flavobacteriales", "__Flavobacteriaceae",
"__Tenacibaculum"]}},{"id": "denovo600", "metadata": {"taxonomy":
["Bacteria", "__Proteobacteria", "__Alphaproteobacteria"]}},{"id":
"denovo607", "metadata": {"taxonomy": ["Bacteria", "__Actinobacteria",
"__Acidimicrobiia", "__Acidimicrobiales", "__Sva0996_marine_group",
"__g"]}},{"id": "denovo609", "metadata": {"taxonomy": ["Bacteria",
"__Nitrospirae", "__Nitrospira", "__Nitrospirales",
"__Nitrospiraceae", "__g"]}},{"id": "denovo610", "metadata":
{"taxonomy": ["Bacteria", "__Proteobacteria", "__Alphaproteobacteria",
"__Rhodobacterales", "__Rhodobacteraceae"]}},{"id": "denovo612",
"metadata": {"taxonomy": ["Bacteria", "__Planctomycetes",
"__Phycisphaerae", "__Phycisphaerales", "__Phycisphaeraceae",
"__SM1A02"]}},{"id": "denovo617", "metadata": {"taxonomy":

["Bacteria", "__Proteobacteria", "__Gammaproteobacteria", "__Alteromonadales", "__Colwelliaceae", "__Thalassomonas"]}},{"id": "denovo618", "metadata": {"taxonomy": ["Bacteria", "__Bacteroidetes", "__Cytophagia", "__Cytophagales", "__Flammeovirgaceae", "__Candidatus_Amoebophilus"]}},{"id": "denovo624", "metadata": {"taxonomy": ["Bacteria", "__Bacteroidetes", "__Flavobacteria", "__Flavobacteriales", "__Flavobacteriaceae"]}},{"id": "denovo625", "metadata": {"taxonomy": ["Bacteria", "__Proteobacteria"]}},{"id": "denovo637", "metadata": {"taxonomy": ["Bacteria", "__Chloroflexi", "__Caldilineae", "__Caldilineales", "__Caldilineaceae", "__Caldilinea"]}},{"id": "denovo641", "metadata": {"taxonomy": ["Bacteria", "__Proteobacteria", "__Gammaproteobacteria", "__Oceanospirillales", "__Hahellaceae", "__Kistimonas"]}},{"id": "denovo642", "metadata": {"taxonomy": ["Bacteria", "__Proteobacteria", "__Gammaproteobacteria", "__Enterobacteriales", "__Enterobacteriaceae", "__Escherichia-Shigella"]}},{"id": "denovo643", "metadata": {"taxonomy": ["Bacteria", "__Proteobacteria", "__Gammaproteobacteria", "__Alteromonadales", "__Alteromonadaceae", "__Haliea"]}},{"id": "denovo644", "metadata": {"taxonomy": ["Bacteria", "__Spirochaetes", "__Spirochaetes", "__Spirochaetales", "__Spirochaetaceae", "__Spirochaeta"]}},{"id": "denovo645", "metadata": {"taxonomy": ["Bacteria", "__Proteobacteria", "__Deltaproteobacteria", "__Myxococcales", "__Nannocystaceae", "__Enhygromyxa"]}},{"id": "denovo648", "metadata": {"taxonomy": ["Bacteria", "__Proteobacteria", "__Deltaproteobacteria", "__Sh765B-TzT-29", "__f", "__g"]}},{"id": "denovo650", "metadata": {"taxonomy": ["Bacteria", "__Proteobacteria", "__Gammaproteobacteria", "__Alteromonadales", "__Alteromonadaceae", "__Haliea"]}},{"id": "denovo652", "metadata": {"taxonomy": ["Bacteria", "__Acidobacteria", "__RB25", "__o", "__f", "__g"]}},{"id": "denovo653", "metadata": {"taxonomy": ["Bacteria", "__Proteobacteria", "__Gammaproteobacteria", "__Alteromonadales", "__Alteromonadaceae", "__Haliea"]}},{"id": "denovo654", "metadata": {"taxonomy": ["Bacteria", "__Proteobacteria", "__Alphaproteobacteria", "__Rhodobacterales", "__Rhodobacteraceae"]}},{"id": "denovo661", "metadata": {"taxonomy": ["Bacteria", "__Proteobacteria", "__Alphaproteobacteria", "__SAR11_clade", "__Surface_1", "__Candidatus_Pelagibacter"]}},{"id": "denovo662", "metadata": {"taxonomy": ["Bacteria", "__Proteobacteria", "__Deltaproteobacteria", "__Desulfobacterales", "__Desulfobacteraceae"]}},{"id": "denovo665", "metadata": {"taxonomy": ["Bacteria", "__Proteobacteria", "__Alphaproteobacteria", "__Rhodospirillales", "__Rhodospirillaceae", "__g"]}},{"id": "denovo669", "metadata": {"taxonomy": ["Bacteria", "__Proteobacteria", "__Alphaproteobacteria", "__Rhizobiales", "__Rhodobiaceae", "__Rhodobium"]}},{"id": "denovo670", "metadata": {"taxonomy": ["Bacteria", "__Proteobacteria", "__Alphaproteobacteria", "__DB1-14", "__f", "__g"]}},{"id": "denovo673", "metadata": {"taxonomy": ["Bacteria", "__Proteobacteria", "__Gammaproteobacteria", "__Alteromonadales", "__Alteromonadaceae", "__BD1-7_clade"]}},{"id": "denovo678", "metadata": {"taxonomy": ["Bacteria", "__Proteobacteria",

"__Alphaproteobacteria", "__Rhodobacterales", "__Rhodobacteraceae"]}},
{"id": "denovo682", "metadata": {"taxonomy": ["Bacteria",
"__Proteobacteria", "__Gammaproteobacteria", "__Xanthomonadales",
"__Sinobacteraceae", "__JTB255_marine_benthic_group"]}},{"id":
"denovo684", "metadata": {"taxonomy": ["Bacteria", "__Bacteroidetes",
"__Flavobacteria", "__Flavobacteriales", "__Flavobacteriaceae"]}},
{"id": "denovo686", "metadata": {"taxonomy": ["Bacteria",
"__Proteobacteria", "__Gammaproteobacteria", "__Pseudomonadales",
"__Moraxellaceae", "__Acinetobacter"]}},{"id": "denovo689",
"metadata": {"taxonomy": ["Bacteria", "__Proteobacteria",
"__Alphaproteobacteria", "__SAR11_clade", "__Surface_1", "__g"]}},
{"id": "denovo690", "metadata": {"taxonomy": ["Bacteria",
"__Cyanobacteria", "__MLE1-12", "__o", "__f", "__g"]}},{"id":
"denovo691", "metadata": {"taxonomy": ["Bacteria", "__Acidobacteria",
"__Acidobacteria", "__PAUC26f", "__f", "__g"]}},{"id": "denovo693",
"metadata": {"taxonomy": ["Bacteria", "__Proteobacteria",
"__Deltaproteobacteria", "__Myxococcales", "__Sorangiineae",
"__Sandaracinaceae"]}},{"id": "denovo694", "metadata": {"taxonomy":
["Bacteria", "__Bacteroidetes", "__Flavobacteria",
"__Flavobacteriales", "__Flavobacteriaceae"]}},{"id": "denovo695",
"metadata": {"taxonomy": ["Bacteria", "__Cyanobacteria",
"__Cyanobacteria", "__SubsectionIII", "__FamilyI"]}},{"id":
"denovo701", "metadata": {"taxonomy": ["Bacteria", "__Proteobacteria",
"__Alphaproteobacteria", "__Rhizobiales"]}},{"id": "denovo708",
"metadata": {"taxonomy": ["Archaea", "__Euryarchaeota",
"__Halobacteria", "__Halobacteriales",
"__Deep_Sea_Hydrothermal_Vent_Gp_6(DHVEG-6)",
"__Candidatus_Parvarchaeum"]}},{"id": "denovo712", "metadata":
{"taxonomy": ["Archaea", "__Thaumarchaeota", "__Marine_Group_I",
"__Candidatus_Nitrosopumilus", "__f", "__g"]}},{"id": "denovo714",
"metadata": {"taxonomy": ["Bacteria", "__Proteobacteria",
"__Deltaproteobacteria", "__Myxococcales", "__Sorangiineae", "__g"]}},
{"id": "denovo716", "metadata": {"taxonomy": ["Bacteria",
"__Proteobacteria", "__Alphaproteobacteria", "__Sphingomonadales",
"__Sphingomonadaceae", "__Novosphingobium"]}},{"id": "denovo717",
"metadata": {"taxonomy": ["Bacteria", "__Proteobacteria",
"__Deltaproteobacteria", "__Bdellovibrionales",
"__Bacteriovoracaceae", "__Bacteriovorax"]}},{"id": "denovo719",
"metadata": {"taxonomy": ["Bacteria", "__Proteobacteria",
"__Gammaproteobacteria", "__HOC36", "__f", "__g"]}},{"id":
"denovo720", "metadata": {"taxonomy": ["Bacteria", "__Proteobacteria",
"__Alphaproteobacteria", "__Sphingomonadales", "__Erythrobacteraceae",
"__Erythrobacter"]}},{"id": "denovo726", "metadata": {"taxonomy":
["Bacteria", "__Proteobacteria", "__Deltaproteobacteria", "__GR-
WP33-30", "__f", "__g"]}},{"id": "denovo729", "metadata": {"taxonomy":
["Bacteria", "__Cyanobacteria", "__Cyanobacteria", "__SubsectionIII",
"__FamilyI", "__Phormidium"]}},{"id": "denovo730", "metadata":
{"taxonomy": ["Bacteria", "__Planctomycetes", "__Planctomycetacia",
"__Planctomycetales", "__Planctomycetaceae", "__g"]}},{"id":
"denovo734", "metadata": {"taxonomy": ["Bacteria", "__Chlamydiae",

"__Chlamydiae", "__Chlamydiales", "__Simkaniaceae", "__g"]}},{"id": "denovo737", "metadata": {"taxonomy": ["Bacteria", "__Bacteroidetes", "__Sphingobacteriia", "__Sphingobacteriales", "__Saprospiraceae", "__g"]}},{"id": "denovo740", "metadata": {"taxonomy": ["Bacteria", "__Proteobacteria", "__Gammaproteobacteria", "__Order_Incertae_Sedis", "__Family_Incertae_Sedis", "__Thiohalophilus"]}},{"id": "denovo743", "metadata": {"taxonomy": ["Bacteria", "__Bacteroidetes", "__Cytophagia", "__Order_II_Incertae_Sedis", "__Rhodothermaceae", "__g"]}},{"id": "denovo744", "metadata": {"taxonomy": ["Bacteria", "__Acidobacteria", "__RB25", "__o", "__f", "__g"]}},{"id": "denovo745", "metadata": {"taxonomy": ["Bacteria", "__Bacteroidetes", "__Flavobacteria", "__Flavobacteriales", "__Flavobacteriaceae", "__NS5_marine_group"]}},{"id": "denovo746", "metadata": {"taxonomy": ["Bacteria"]}},{"id": "denovo747", "metadata": {"taxonomy": ["Bacteria", "__Proteobacteria", "__Alphaproteobacteria"]}},{"id": "denovo750", "metadata": {"taxonomy": ["Bacteria", "__Acidobacteria", "__Holophagae", "__TK85", "__f", "__g"]}},{"id": "denovo751", "metadata": {"taxonomy": ["Bacteria", "__Deferribacteres", "__Deferribacteres", "__Deferribacterales", "__SAR406_clade(Marine_group_A)", "__g"]}},{"id": "denovo752", "metadata": {"taxonomy": ["Bacteria", "__Nitrospirae", "__Nitrospira", "__Nitrospirales", "__OPB95", "__g"]}},{"id": "denovo753", "metadata": {"taxonomy": ["Bacteria", "__Proteobacteria", "__Gammaproteobacteria"]}},{"id": "denovo756", "metadata": {"taxonomy": ["Bacteria", "__Candidate_division_KB1", "__c", "__o", "__f", "__g"]}},{"id": "denovo762", "metadata": {"taxonomy": ["Bacteria", "__Proteobacteria", "__Gammaproteobacteria"]}},{"id": "denovo766", "metadata": {"taxonomy": ["Bacteria", "__Proteobacteria", "__Gammaproteobacteria", "__Pseudomonadales", "__Moraxellaceae", "__Acinetobacter"]}},{"id": "denovo769", "metadata": {"taxonomy": ["Bacteria", "__Bacteroidetes", "__Flavobacteria", "__Flavobacteriales", "__Flavobacteriaceae", "__Zeaxanthinibacter"]}},{"id": "denovo770", "metadata": {"taxonomy": ["Bacteria", "__Proteobacteria", "__Alphaproteobacteria", "__Rhizobiales", "__Rhodobiaceae", "__Anderseniella"]}},{"id": "denovo778", "metadata": {"taxonomy": ["Bacteria", "__Acidobacteria", "__Acidobacteria", "__BPC015", "__f", "__g"]}},{"id": "denovo781", "metadata": {"taxonomy": ["Bacteria", "__Proteobacteria", "__Alphaproteobacteria", "__Rhizobiales"]}},{"id": "denovo782", "metadata": {"taxonomy": ["Bacteria", "__Proteobacteria", "__Alphaproteobacteria", "__Sphingomonadales"]}},{"id": "denovo784", "metadata": {"taxonomy": ["Bacteria", "__Acidobacteria", "__RB25", "__o", "__f", "__g"]}},{"id": "denovo797", "metadata": {"taxonomy": ["Bacteria", "__Proteobacteria", "__Gammaproteobacteria", "__Sva0071", "__f", "__g"]}},{"id": "denovo798", "metadata": {"taxonomy": ["Bacteria", "__Chloroflexi", "__Thermomicrobia", "__AKYG1722", "__f", "__g"]}},{"id": "denovo800", "metadata": {"taxonomy": ["Bacteria", "__Proteobacteria", "__Deltaproteobacteria", "__GR-WP33-30", "__f", "__g"]}},{"id": "denovo804", "metadata": {"taxonomy": ["Bacteria", "__Proteobacteria", "__Gammaproteobacteria", "__BD7-8_marine_group",

"__f", "__g"]}},{"id": "denovo805", "metadata": {"taxonomy": ["Bacteria", "__Bacteroidetes", "__Flavobacteria", "__Flavobacteriales", "__Flavobacteriaceae", "__g"]}},{"id": "denovo806", "metadata": {"taxonomy": ["Bacteria", "__Proteobacteria", "__Alphaproteobacteria", "__Rhodobacterales", "__Rhodobacteraceae", "__Maritimibacter"]}},{"id": "denovo811", "metadata": {"taxonomy": ["Bacteria", "__Proteobacteria", "__Deltaproteobacteria", "__Syntrophobacterales", "__Syntrophaceae", "__Desulfobacca"]}},{"id": "denovo813", "metadata": {"taxonomy": ["Bacteria", "__Spirochaetes", "__Spirochaetes", "__Spirochaetales", "__Leptospiraceae", "__g"]}},{"id": "denovo814", "metadata": {"taxonomy": ["Bacteria", "__Proteobacteria", "__Deltaproteobacteria", "__Desulfobacterales", "__Desulfobacteraceae"]}},{"id": "denovo816", "metadata": {"taxonomy": ["Bacteria", "__Proteobacteria", "__Deltaproteobacteria", "__Myxococcales", "__Haliangiaceae", "__Haliangium"]}},{"id": "denovo817", "metadata": {"taxonomy": ["Bacteria", "__Proteobacteria", "__Deltaproteobacteria", "__Myxococcales", "__Nannocystaceae", "__g"]}},{"id": "denovo819", "metadata": {"taxonomy": ["Bacteria", "__Proteobacteria", "__Alphaproteobacteria", "__Rhodobacterales", "__Rhodobacteraceae"]}},{"id": "denovo821", "metadata": {"taxonomy": ["Bacteria", "__Proteobacteria", "__Deltaproteobacteria", "__Myxococcales", "__Nannocystaceae", "__g"]}},{"id": "denovo827", "metadata": {"taxonomy": ["Bacteria", "__Proteobacteria", "__Alphaproteobacteria", "__Sphingomonadales", "__Sphingomonadaceae"]}},{"id": "denovo829", "metadata": {"taxonomy": ["Bacteria", "__Proteobacteria", "__Alphaproteobacteria", "__Rhodobacterales", "__Rhodobacteraceae"]}},{"id": "denovo830", "metadata": {"taxonomy": ["Bacteria", "__Planctomycetes", "__Planctomycetacia", "__Planctomycetales", "__Planctomycetaceae"]}},{"id": "denovo831", "metadata": {"taxonomy": ["Bacteria", "__Chloroflexi", "__Caldilineae", "__Caldilineales", "__Caldilineaceae", "__g"]}},{"id": "denovo833", "metadata": {"taxonomy": ["Bacteria", "__Proteobacteria", "__Gammaproteobacteria", "__Oceanospirillales", "__Oceanospirillaceae", "__Pseudospirillum"]}},{"id": "denovo836", "metadata": {"taxonomy": ["Bacteria", "__Proteobacteria", "__Alphaproteobacteria", "__Rhodobacterales", "__Rhodobacteraceae", "__Roseobacter_clade_AS-21_lineage"]}},{"id": "denovo839", "metadata": {"taxonomy": ["Bacteria", "__Proteobacteria", "__Gammaproteobacteria", "__Alteromonadales", "__Psychromonadaceae", "__Psychromonas"]}},{"id": "denovo840", "metadata": {"taxonomy": ["Bacteria", "__Proteobacteria", "__Alphaproteobacteria", "__OCS116_clade", "__f", "__g"]}},{"id": "denovo841", "metadata": {"taxonomy": ["Bacteria", "__Planctomycetes"]}},{"id": "denovo843", "metadata": {"taxonomy": ["Bacteria", "__Proteobacteria", "__Deltaproteobacteria", "__Sh765B-TzT-29", "__f", "__g"]}},{"id": "denovo844", "metadata": {"taxonomy": ["Bacteria", "__Chloroflexi", "__Caldilineae", "__Caldilineales", "__Caldilineaceae", "__g"]}},{"id": "denovo846", "metadata": {"taxonomy": ["Bacteria", "__Proteobacteria", "__Deltaproteobacteria", "__Desulfobacterales", "__Desulfobacteraceae"]}},{"id": "denovo847", "metadata": {"taxonomy":

["Bacteria", "__Bacteroidetes", "__Flavobacteria", "__Flavobacteriales", "__Flavobacteriaceae", "__g"]}},{"id": "denovo849", "metadata": {"taxonomy": ["Bacteria", "__Proteobacteria", "__Alphaproteobacteria", "__Caulobacterales", "__Hyphomonadaceae", "__Hellea"]}},{"id": "denovo856", "metadata": {"taxonomy": ["Bacteria", "__Spirochaetes", "__Spirochaetes", "__Spirochaetales", "__Spirochaetaceae", "__Spirochaeta"]}},{"id": "denovo857", "metadata": {"taxonomy": ["Bacteria", "__Proteobacteria", "__Alphaproteobacteria", "__Rhodospirillales", "__Rhodospirillaceae"]}},{"id": "denovo858", "metadata": {"taxonomy": ["Bacteria", "__Bacteroidetes"]}},{"id": "denovo860", "metadata": {"taxonomy": ["Bacteria", "__Cyanobacteria", "__Cyanobacteria", "__SubsectionIII", "__FamilyI", "__g"]}},{"id": "denovo862", "metadata": {"taxonomy": ["Bacteria", "__Candidate_division_KB1", "__c", "__o", "__f", "__g"]}},{"id": "denovo863", "metadata": {"taxonomy": ["Bacteria", "__Proteobacteria", "__Gammaproteobacteria", "__Chromatiales", "__Chromatiaceae", "__Nitrosococcus"]}},{"id": "denovo870", "metadata": {"taxonomy": ["Bacteria", "__Cyanobacteria", "__Cyanobacteria", "__SubsectionIV", "__FamilyII", "__Rivularia"]}},{"id": "denovo873", "metadata": {"taxonomy": ["Bacteria", "__Proteobacteria", "__Alphaproteobacteria", "__Rhodobacterales", "__Rhodobacteraceae", "__Marinosulfonomonas"]}},{"id": "denovo877", "metadata": {"taxonomy": ["Bacteria", "__Proteobacteria", "__Deltaproteobacteria", "__Sh765B-TzT-29", "__f", "__g"]}},{"id": "denovo879", "metadata": {"taxonomy": ["Bacteria", "__Firmicutes", "__Clostridia", "__Clostridiales", "__Lachnospiraceae"]}},{"id": "denovo886", "metadata": {"taxonomy": ["Bacteria", "__Proteobacteria", "__Alphaproteobacteria", "__Rhodobacterales", "__Rhodobacteraceae"]}},{"id": "denovo887", "metadata": {"taxonomy": ["Bacteria", "__Proteobacteria", "__Deltaproteobacteria", "__Myxococcales", "__Cystobacterineae", "__g"]}},{"id": "denovo889", "metadata": {"taxonomy": ["Bacteria", "__Proteobacteria", "__Alphaproteobacteria", "__DB1-14", "__f", "__g"]}},{"id": "denovo896", "metadata": {"taxonomy": ["Bacteria", "__Chloroflexi", "__SAR202_clade", "__o", "__f", "__g"]}},{"id": "denovo909", "metadata": {"taxonomy": ["Bacteria", "__Proteobacteria", "__Alphaproteobacteria", "__Rhizobiales", "__Rhodobiaceae", "__Rhodobium"]}},{"id": "denovo914", "metadata": {"taxonomy": ["Bacteria", "__Proteobacteria", "__Alphaproteobacteria", "__Kordiimonadales", "__Kordiimonadaceae", "__Kordiimonas"]}},{"id": "denovo923", "metadata": {"taxonomy": ["Bacteria", "__TM6", "__c", "__o", "__f", "__g"]}},{"id": "denovo925", "metadata": {"taxonomy": ["Bacteria", "__Candidate_division_BRC1", "__c", "__o", "__f", "__g"]}},{"id": "denovo927", "metadata": {"taxonomy": ["Bacteria", "__Proteobacteria", "__Gammaproteobacteria", "__Pseudomonadales", "__Moraxellaceae", "__Acinetobacter"]}},{"id": "denovo928", "metadata": {"taxonomy": ["Bacteria", "__Verrucomicrobia", "__Verrucomicrobiae", "__Verrucomicrobiales", "__DEV007", "__g"]}},{"id": "denovo931", "metadata": {"taxonomy": ["Bacteria", "__Proteobacteria", "__Alphaproteobacteria", "__Caulobacterales", "__Hyphomonadaceae"]}},

{"id": "denovo932", "metadata": {"taxonomy": ["Bacteria", "__Bacteroidetes", "__Flavobacteria", "__Flavobacteriales", "__Flavobacteriaceae", "__NS5_marine_group"]}},{"id": "denovo933", "metadata": {"taxonomy": ["Bacteria", "__Candidate_division_WS3", "__c", "__o", "__f", "__g"]}},{"id": "denovo934", "metadata": {"taxonomy": ["Bacteria", "__Proteobacteria", "__Gammaproteobacteria", "__Xanthomonadales", "__Sinobacteraceae", "__JTB255_marine_benthic_group"]}},{"id": "denovo938", "metadata": {"taxonomy": ["Bacteria", "__Proteobacteria", "__Alphaproteobacteria", "__Rhodospirillales", "__Rhodospirillaceae", "__Defluviicoccus"]}},{"id": "denovo940", "metadata": {"taxonomy": ["Bacteria", "__Actinobacteria", "__Actinobacteria", "__Frankiales", "__Acidothermaceae", "__Acidothermus"]}},{"id": "denovo943", "metadata": {"taxonomy": ["Bacteria", "__Proteobacteria", "__Gammaproteobacteria", "__Alteromonadales", "__Colwelliaceae", "__Colwellia"]}},{"id": "denovo944", "metadata": {"taxonomy": ["Bacteria", "__Proteobacteria", "__Gammaproteobacteria"]}},{"id": "denovo948", "metadata": {"taxonomy": ["Bacteria", "__Proteobacteria", "__Gammaproteobacteria", "__Chromatiales"]}},{"id": "denovo949", "metadata": {"taxonomy": ["Bacteria", "__Firmicutes", "__Clostridia", "__Clostridiales", "__Clostridiaceae", "__Caminicella"]}},{"id": "denovo953", "metadata": {"taxonomy": ["Bacteria", "__Bacteroidetes", "__Flavobacteria", "__Flavobacteriales", "__Flavobacteriaceae"]}},{"id": "denovo954", "metadata": {"taxonomy": ["Bacteria", "__Bacteroidetes", "__Cytophagia", "__Cytophagales", "__Flammeovirgaceae", "__g"]}},{"id": "denovo955", "metadata": {"taxonomy": ["Bacteria", "__Proteobacteria", "__Alphaproteobacteria", "__Rhodobacterales", "__Rhodobacteraceae", "__Marinovum"]}},{"id": "denovo957", "metadata": {"taxonomy": ["Bacteria", "__Bacteroidetes", "__Sphingobacteriia", "__Sphingobacteriales", "__Saprospiraceae", "__g"]}},{"id": "denovo958", "metadata": {"taxonomy": ["Bacteria", "__Bacteroidetes", "__Sphingobacteriia", "__Sphingobacteriales", "__Saprospiraceae", "__g"]}},{"id": "denovo964", "metadata": {"taxonomy": ["Bacteria", "__Proteobacteria", "__Alphaproteobacteria", "__DB1-14", "__f", "__g"]}},{"id": "denovo966", "metadata": {"taxonomy": ["Bacteria", "__Gemmatimonadetes", "__Gemmatimonadetes", "__PAUC43f_marine_benthic_group", "__f", "__g"]}},{"id": "denovo969", "metadata": {"taxonomy": ["Bacteria", "__Proteobacteria", "__Deltaproteobacteria", "__Myxococcales", "__Haliangiaceae", "__Haliangium"]}},{"id": "denovo970", "metadata": {"taxonomy": ["Bacteria", "__Bacteroidetes", "__Flavobacteria", "__Flavobacteriales", "__Flavobacteriaceae", "__Aquimarina"]}},{"id": "denovo971", "metadata": {"taxonomy": ["Bacteria", "__Proteobacteria", "__Deltaproteobacteria", "__Desulfobacterales", "__Desulfobacteraceae", "__Desulfospira"]}},{"id": "denovo973", "metadata": {"taxonomy": ["Bacteria", "__Proteobacteria", "__Alphaproteobacteria", "__Rhodobacterales", "__Rhodobacteraceae"]}},{"id": "denovo974", "metadata": {"taxonomy": ["Bacteria", "__Bacteroidetes", "__Flavobacteria", "__Flavobacteriales", "__NS9_marine_group", "__g"]}},{"id": "denovo978", "metadata":

{"taxonomy": ["Bacteria", "__Proteobacteria", "__Gammaproteobacteria", "__B38", "__f", "__g"]}},{"id": "denovo979", "metadata": {"taxonomy": ["Bacteria", "__Bacteroidetes", "__Flavobacteria", "__Flavobacteriales", "__Flavobacteriaceae", "__Tenacibaculum"]}}, {"id": "denovo981", "metadata": {"taxonomy": ["Bacteria", "__Bacteroidetes", "__Sphingobacteriia", "__Sphingobacteriales", "__Saprospiraceae", "__g"]}},{"id": "denovo982", "metadata": {"taxonomy": ["Bacteria", "__Bacteroidetes", "__Sphingobacteriia", "__Sphingobacteriales", "__NS11-12_marine_group", "__g"]}},{"id": "denovo983", "metadata": {"taxonomy": ["Bacteria", "__Proteobacteria", "__Gammaproteobacteria", "__Pseudomonadales", "__Moraxellaceae", "__Acinetobacter"]}},{"id": "denovo990", "metadata": {"taxonomy": ["Bacteria", "__Proteobacteria", "__Alphaproteobacteria", "__Rhodobacterales", "__Rhodobacteraceae"]}},{"id": "denovo997", "metadata": {"taxonomy": ["Bacteria", "__Proteobacteria", "__Gammaproteobacteria", "__KI89A_clade", "__f", "__g"]}},{"id": "denovo999", "metadata": {"taxonomy": ["Bacteria", "__Proteobacteria", "__Alphaproteobacteria", "__Rhodobacterales", "__Rhodobacteraceae", "__Ruegeria"]}},{"id": "denovo1000", "metadata": {"taxonomy": ["Bacteria", "__Candidate_division_BRC1", "__c", "__o", "__f", "__g"]}},{"id": "denovo1001", "metadata": {"taxonomy": ["Bacteria", "__Proteobacteria", "__Alphaproteobacteria", "__Rhodospirillales", "__Rhodospirillaceae"]}},{"id": "denovo1003", "metadata": {"taxonomy": ["Bacteria", "__Acidobacteria", "__Acidobacteria", "__PAUC26f", "__f", "__g"]}},{"id": "denovo1004", "metadata": {"taxonomy": ["Bacteria", "__Proteobacteria", "__Gammaproteobacteria", "__Pseudomonadales", "__Moraxellaceae", "__Acinetobacter"]}},{"id": "denovo1011", "metadata": {"taxonomy": ["Bacteria", "__Bacteroidetes", "__Flavobacteria", "__Flavobacteriales", "__Flavobacteriaceae", "__Winogradskyella"]}},{"id": "denovo1012", "metadata": {"taxonomy": ["Bacteria", "__Bacteroidetes", "__Flavobacteria", "__Flavobacteriales", "__Flavobacteriaceae", "__Winogradskyella"]}}, {"id": "denovo1014", "metadata": {"taxonomy": ["Bacteria", "__Chloroflexi", "__Caldilineae", "__Caldilineales", "__Caldilineaceae", "__g"]}},{"id": "denovo1022", "metadata": {"taxonomy": ["Bacteria", "__Proteobacteria", "__Deltaproteobacteria", "__Myxococcales", "__Cystobacterineae", "__g"]}},{"id": "denovo1023", "metadata": {"taxonomy": ["Bacteria", "__Proteobacteria", "__Alphaproteobacteria", "__Rhodospirillales"]}},{"id": "denovo1025", "metadata": {"taxonomy": ["Bacteria", "__Proteobacteria", "__Gammaproteobacteria", "__Legionellales", "__Coxiellaceae", "__Aquicella"]}},{"id": "denovo1028", "metadata": {"taxonomy": ["Bacteria", "__Proteobacteria", "__Gammaproteobacteria", "__Oceanospirillales"]}},{"id": "denovo1030", "metadata": {"taxonomy": ["Bacteria", "__Actinobacteria", "__Actinobacteria", "__Frankiales", "__Acidothermaceae", "__Acidothermus"]}},{"id": "denovo1033", "metadata": {"taxonomy": ["Bacteria", "__Proteobacteria", "__Alphaproteobacteria", "__SAR11_clade", "__Surface_1", "__Candidatus_Pelagibacter"]}},{"id": "denovo1041", "metadata": {"taxonomy": ["Bacteria", "__RF3", "__c", "__o", "__f", "__g"]}},

{"id": "denovo1054", "metadata": {"taxonomy": ["Bacteria", "__Proteobacteria", "__Betaproteobacteria", "__Burkholderiales", "__Oxalobacteraceae", "__Massilia"]}},{"id": "denovo1055", "metadata": {"taxonomy": ["Bacteria", "__Proteobacteria", "__Deltaproteobacteria", "__Sh765B-TzT-29", "__f", "__g"]}},{"id": "denovo1056", "metadata": {"taxonomy": ["Bacteria", "__Proteobacteria", "__Gammaproteobacteria", "__Order_Incertae_Sedis", "__Family_Incertae_Sedis", "__Marinicella"]}},{"id": "denovo1061", "metadata": {"taxonomy": ["Bacteria", "__Proteobacteria", "__Deltaproteobacteria", "__Sh765B-TzT-29", "__f", "__g"]}},{"id": "denovo1063", "metadata": {"taxonomy": ["Bacteria", "__Proteobacteria", "__Alphaproteobacteria", "__Rhodobacterales", "__Rhodobacteraceae"]}},{"id": "denovo1070", "metadata": {"taxonomy": ["Bacteria", "__Proteobacteria", "__Gammaproteobacteria", "__Xanthomonadales", "__Sinobacteraceae", "__JTB255_marine_benthic_group"]}},{"id": "denovo1075", "metadata": {"taxonomy": ["Bacteria", "__Proteobacteria", "__Gammaproteobacteria", "__Legionellales", "__Coxiellaceae", "__Coxiella"]}},{"id": "denovo1077", "metadata": {"taxonomy": ["Bacteria", "__Cyanobacteria", "__Cyanobacteria", "__SubsectionII", "__FamilyII", "__Pleurocapsa"]}},{"id": "denovo1078", "metadata": {"taxonomy": ["Bacteria", "__Actinobacteria", "__Acidimicrobiia", "__Acidimicrobiales", "__OCS155_marine_group", "__g"]}},{"id": "denovo1079", "metadata": {"taxonomy": ["Bacteria", "__Proteobacteria", "__Deltaproteobacteria", "__Myxococcales", "__0319-6G20", "__g"]}},{"id": "denovo1083", "metadata": {"taxonomy": ["Bacteria", "__Proteobacteria", "__Gammaproteobacteria", "__Alteromonadales", "__Alteromonadaceae", "__OM60(NOR5)_clade"]}},{"id": "denovo1086", "metadata": {"taxonomy": ["Bacteria", "__Proteobacteria", "__Gammaproteobacteria", "__E01-9C-26_marine_group", "__f", "__g"]}},{"id": "denovo1089", "metadata": {"taxonomy": ["Bacteria", "__Proteobacteria", "__Deltaproteobacteria", "__Desulfobacterales", "__Desulfobacteraceae", "__Sva0081_sediment_group"]}},{"id": "denovo1091", "metadata": {"taxonomy": ["Bacteria", "__Chlorobi", "__Chlorobia", "__Chlorobiales", "__Chlorobiaceae", "__g"]}},{"id": "denovo1093", "metadata": {"taxonomy": ["Bacteria", "__Bacteroidetes", "__Flavobacteria", "__Flavobacteriales", "__Cryomorphaceae", "__Owenweeksia"]}},{"id": "denovo1095", "metadata": {"taxonomy": ["Bacteria", "__Bacteroidetes", "__Flavobacteria", "__Flavobacteriales", "__Flavobacteriaceae", "__Aquimarina"]}},{"id": "denovo1097", "metadata": {"taxonomy": ["Bacteria", "__Proteobacteria", "__Gammaproteobacteria", "__Oceanospirillales"]}},{"id": "denovo1098", "metadata": {"taxonomy": ["Bacteria", "__Proteobacteria", "__Gammaproteobacteria", "__Alteromonadales", "__Pseudoalteromonadaceae", "__Pseudoalteromonas"]}},{"id": "denovo1101", "metadata": {"taxonomy": ["Bacteria", "__Actinobacteria", "__Actinobacteria", "__Corynebacteriales", "__Dietziaceae", "__Dietzia"]}},{"id": "denovo1102", "metadata": {"taxonomy": ["Bacteria", "__Proteobacteria", "__Alphaproteobacteria", "__SAR11_clade", "__Surface_1", "__Candidatus_Pelagibacter"]}},{"id": "denovo1105", "metadata": {"taxonomy": ["Bacteria",

"__Proteobacteria", "__Gammaproteobacteria", "__Alteromonadales", "__Alteromonadaceae", "__Candidatus_Endobugula"]}},{"id": "denovo1106", "metadata": {"taxonomy": ["Bacteria", "__Cyanobacteria", "__Cyanobacteria", "__SubsectionIII", "__FamilyI", "__Phormidium"]}}, {"id": "denovo1108", "metadata": {"taxonomy": ["Bacteria", "__Firmicutes", "__Clostridia", "__Clostridiales", "__Christensenellaceae"]}},{"id": "denovo1113", "metadata": {"taxonomy": ["Bacteria", "__Acidobacteria", "__Holophagae", "__CA002", "__f", "__g"]}},{"id": "denovo1114", "metadata": {"taxonomy": ["Bacteria", "__Proteobacteria", "__Gammaproteobacteria", "__BD3-1", "__f", "__g"]}},{"id": "denovo1115", "metadata": {"taxonomy": ["Bacteria", "__Proteobacteria", "__Alphaproteobacteria", "__DB1-14", "__f", "__g"]}},{"id": "denovo1117", "metadata": {"taxonomy": ["Bacteria", "__Bacteroidetes", "__Flavobacteria", "__Flavobacteriales", "__Flavobacteriaceae", "__Gilvibacter"]}},{"id": "denovo1119", "metadata": {"taxonomy": ["Bacteria", "__Proteobacteria", "__Gammaproteobacteria", "__Vibrionales", "__Vibrionaceae", "__Vibrio"]}},{"id": "denovo1120", "metadata": {"taxonomy": ["Bacteria", "__Planctomycetes", "__Planctomycetacia", "__Planctomycetales", "__Planctomycetaceae", "__Blastopirellula"]}}, {"id": "denovo1123", "metadata": {"taxonomy": ["Bacteria", "__Proteobacteria", "__Deltaproteobacteria", "__Bdellovibrionales", "__Bacteriovoracaceae", "__Peredibacter"]}},{"id": "denovo1127", "metadata": {"taxonomy": ["Bacteria", "__Proteobacteria", "__Gammaproteobacteria", "__Alteromonadales", "__Alteromonadaceae", "__Simiduia"]}},{"id": "denovo1129", "metadata": {"taxonomy": ["Bacteria", "__Planctomycetes", "__Planctomycetacia", "__Planctomycetales", "__Planctomycetaceae", "__Rhodopirellula"]}}, {"id": "denovo1132", "metadata": {"taxonomy": ["Bacteria", "__Bacteroidetes", "__Cytophagia", "__Cytophagales", "__Flammeovirgaceae", "__Candidatus_Amoebophilus"]}},{"id": "denovo1134", "metadata": {"taxonomy": ["Bacteria", "__Proteobacteria", "__Gammaproteobacteria"]}},{"id": "denovo1135", "metadata": {"taxonomy": ["Bacteria", "__Proteobacteria", "__Deltaproteobacteria", "__Sh765B-TzT-29", "__f", "__g"]}},{"id": "denovo1137", "metadata": {"taxonomy": ["Bacteria", "__Proteobacteria", "__Alphaproteobacteria", "__Rhodobacterales", "__Rhodobacteraceae"]}},{"id": "denovo1139", "metadata": {"taxonomy": ["Bacteria", "__Cyanobacteria", "__Cyanobacteria", "__SubsectionIII", "__FamilyI", "__Leptolyngbya"]}},{"id": "denovo1141", "metadata": {"taxonomy": ["Bacteria", "__Proteobacteria", "__Gammaproteobacteria"]}},{"id": "denovo1143", "metadata": {"taxonomy": ["Bacteria", "__Proteobacteria", "__Gammaproteobacteria", "__E01-9C-26_marine_group", "__f", "__g"]}},{"id": "denovo1144", "metadata": {"taxonomy": ["Bacteria", "__Proteobacteria", "__Deltaproteobacteria", "__Desulfobacterales", "__Desulfobulbaceae"]}},{"id": "denovo1148", "metadata": {"taxonomy": ["Bacteria", "__Bacteroidetes", "__Flavobacteria", "__Flavobacteriales", "__Cryomorphaceae", "__Owenweeksia"]}},{"id": "denovo1149", "metadata": {"taxonomy": ["Bacteria",

"__Proteobacteria", "__Gammaproteobacteria", "__Pseudomonadales", "__Moraxellaceae", "__Acinetobacter"]}},{"id": "denovo1154", "metadata": {"taxonomy": ["Bacteria", "__Proteobacteria", "__Deltaproteobacteria", "__Myxococcales", "__Cystobacterineae", "__g"]}},{"id": "denovo1155", "metadata": {"taxonomy": ["Bacteria", "__Proteobacteria", "__Gammaproteobacteria", "__KI89A_clade", "__f", "__g"]}},{"id": "denovo1157", "metadata": {"taxonomy": ["Bacteria", "__Proteobacteria", "__Deltaproteobacteria", "__Myxococcales", "__Cystobacterineae", "__g"]}},{"id": "denovo1159", "metadata": {"taxonomy": ["Bacteria", "__Bacteroidetes", "__VC2.1_Bac22", "__o", "__f", "__g"]}},{"id": "denovo1164", "metadata": {"taxonomy": ["Bacteria", "__Candidate_division_WS3", "__c", "__o", "__f", "__g"]}},{"id": "denovo1165", "metadata": {"taxonomy": ["Bacteria", "__Proteobacteria", "__Gammaproteobacteria", "__Order_Incertae_Sedis", "__Family_Incertae_Sedis", "__Marinicella"]}},{"id": "denovo1166", "metadata": {"taxonomy": ["Bacteria", "__Proteobacteria", "__Deltaproteobacteria", "__Bdellovibrionales", "__Bacteriovoracaceae", "__Peredibacter"]}},{"id": "denovo1170", "metadata": {"taxonomy": ["Bacteria", "__Proteobacteria", "__Gammaproteobacteria", "__E01-9C-26_marine_group", "__f", "__g"]}},{"id": "denovo1171", "metadata": {"taxonomy": ["Bacteria", "__Bacteroidetes", "__Flavobacteria", "__Flavobacteriales", "__Flavobacteriaceae", "__Actibacter"]}},{"id": "denovo1176", "metadata": {"taxonomy": ["Bacteria", "__Cyanobacteria", "__Cyanobacteria", "__SubsectionIII", "__FamilyI", "__Spirulina"]}},{"id": "denovo1178", "metadata": {"taxonomy": ["Bacteria", "__Bacteroidetes", "__Flavobacteria", "__Flavobacteriales", "__Cryomorphaceae", "__NS7_marine_group"]}},{"id": "denovo1181", "metadata": {"taxonomy": ["Bacteria", "__Proteobacteria", "__Alphaproteobacteria", "__Rhizobiales", "__Rhodobiaceae", "__Rhodobium"]}},{"id": "denovo1183", "metadata": {"taxonomy": ["Bacteria", "__Proteobacteria", "__Alphaproteobacteria", "__OCS116_clade", "__f", "__g"]}},{"id": "denovo1184", "metadata": {"taxonomy": ["Bacteria", "__Proteobacteria", "__Gammaproteobacteria", "__Alteromonadales", "__Alteromonadaceae", "__Teredinibacter"]}},{"id": "denovo1189", "metadata": {"taxonomy": ["Bacteria", "__Proteobacteria", "__Gammaproteobacteria", "__Alteromonadales", "__Pseudoalteromonadaceae", "__Pseudoalteromonas"]}},{"id": "denovo1193", "metadata": {"taxonomy": ["Bacteria", "__Verrucomicrobia", "__Verrucomicrobiae", "__Verrucomicrobiales", "__DEV007", "__g"]}},{"id": "denovo1196", "metadata": {"taxonomy": ["Bacteria", "__Proteobacteria", "__Gammaproteobacteria", "__Alteromonadales", "__Pseudoalteromonadaceae", "__Pseudoalteromonas"]}},{"id": "denovo1198", "metadata": {"taxonomy": ["Bacteria", "__Proteobacteria", "__Gammaproteobacteria", "__KI89A_clade", "__f", "__g"]}},{"id": "denovo1200", "metadata": {"taxonomy": ["Bacteria", "__Proteobacteria", "__Alphaproteobacteria", "__Rhodobacterales", "__Rhodobacteraceae"]}},{"id": "denovo1206", "metadata": {"taxonomy": ["Bacteria", "__Proteobacteria", "__Alphaproteobacteria", "__Rhodobacterales", "__Rhodobacteraceae",

"__Ruegeria"]}},{"id": "denovo1208", "metadata": {"taxonomy": ["Bacteria", "__Elusimicrobia", "__Elusimicrobia", "__Lineage_IIb", "__f", "__g"]}},{"id": "denovo1210", "metadata": {"taxonomy": ["Bacteria", "__Proteobacteria", "__Alphaproteobacteria", "__Rickettsiales", "__SAR116_clade", "__g"]}},{"id": "denovo1213", "metadata": {"taxonomy": ["Bacteria", "__Bacteroidetes", "__Cytophagia", "__Order_II_Incertae_Sedis", "__Rhodothermaceae", "__g"]}},{"id": "denovo1219", "metadata": {"taxonomy": ["Bacteria", "__Proteobacteria", "__Alphaproteobacteria", "__Rhodobacterales", "__Rhodobacteraceae", "__Marinosulfonomonas"]}},{"id": "denovo1220", "metadata": {"taxonomy": ["Bacteria", "__Spirochaetes", "__Spirochaetes", "__Spirochaetales", "__Spirochaetaceae", "__Spirochaeta"]}},{"id": "denovo1222", "metadata": {"taxonomy": ["Bacteria", "__Proteobacteria", "__Alphaproteobacteria", "__Rhodobacterales", "__Rhodobacteraceae", "__Pseudovibrio"]}},{"id": "denovo1225", "metadata": {"taxonomy": ["Bacteria", "__Bacteroidetes", "__Sphingobacteriia", "__Sphingobacteriales", "__Saprospiraceae", "__g"]}},{"id": "denovo1226", "metadata": {"taxonomy": ["Bacteria", "__Chlorobi", "__Chlorobia", "__Chlorobiales", "__Chlorobiaceae"]}},{"id": "denovo1231", "metadata": {"taxonomy": ["Bacteria", "__Proteobacteria", "__Alphaproteobacteria", "__Rhizobiales"]}},{"id": "denovo1235", "metadata": {"taxonomy": ["Bacteria", "__Proteobacteria", "__Gammaproteobacteria", "__Xanthomonadales", "__Sinobacteraceae", "__JTB255_marine_benthic_group"]}},{"id": "denovo1237", "metadata": {"taxonomy": ["Bacteria", "__Proteobacteria", "__Gammaproteobacteria", "__BD7-8_marine_group", "__f", "__g"]}},{"id": "denovo1238", "metadata": {"taxonomy": ["Bacteria", "__Proteobacteria", "__Gammaproteobacteria", "__BD7-8_marine_group", "__f", "__g"]}},{"id": "denovo1248", "metadata": {"taxonomy": ["Bacteria", "__Proteobacteria", "__Alphaproteobacteria", "__Rhodobacterales", "__Rhodobacteraceae", "__g"]}},{"id": "denovo1251", "metadata": {"taxonomy": ["Bacteria", "__Bacteroidetes", "__Cytophagia", "__Cytophagales", "__Flammeovirgaceae", "__Reichenbachiella"]}},{"id": "denovo1253", "metadata": {"taxonomy": ["Bacteria", "__Proteobacteria", "__Deltaproteobacteria", "__Myxococcales", "__Cystobacterineae", "__g"]}},{"id": "denovo1254", "metadata": {"taxonomy": ["Bacteria", "__Proteobacteria", "__Gammaproteobacteria", "__Xanthomonadales", "__Sinobacteraceae", "__JTB255_marine_benthic_group"]}},{"id": "denovo1256", "metadata": {"taxonomy": ["Bacteria", "__Proteobacteria", "__Deltaproteobacteria", "__SAR324_clade(Marine_group_B)", "__f", "__g"]}},{"id": "denovo1257", "metadata": {"taxonomy": ["Bacteria", "__Proteobacteria", "__Gammaproteobacteria", "__KI89A_clade", "__f", "__g"]}},{"id": "denovo1258", "metadata": {"taxonomy": ["Bacteria", "__Chlamydiae", "__Chlamydiae", "__Chlamydiales", "__Simkaniaceae", "__g"]}},{"id": "denovo1259", "metadata": {"taxonomy": ["Bacteria", "__Proteobacteria", "__Gammaproteobacteria", "__Alteromonadales", "__Alteromonadaceae", "__Haliea"]}},{"id": "denovo1262", "metadata": {"taxonomy": ["Bacteria", "__Chloroflexi", "__Caldilineae",

"__Caldilineales", "__Caldilineaceae", "__g"]}},{"id": "denovo1263", "metadata": {"taxonomy": ["Bacteria", "__Verrucomicrobia", "__Opitutae", "__Puniceicoccales", "__Puniceicoccaceae"]}},{"id": "denovo1267", "metadata": {"taxonomy": ["Bacteria", "__Bacteroidetes", "__Flavobacteria", "__Flavobacteriales", "__Flavobacteriaceae", "__Croceitalea"]}},{"id": "denovo1270", "metadata": {"taxonomy": ["Bacteria", "__Bacteroidetes", "__Sphingobacteriia", "__Sphingobacteriales", "__Saprospiraceae", "__g"]}},{"id": "denovo1271", "metadata": {"taxonomy": ["Bacteria", "__Proteobacteria", "__Alphaproteobacteria", "__Rhizobiales"]}},{"id": "denovo1272", "metadata": {"taxonomy": ["Bacteria", "__Proteobacteria", "__Alphaproteobacteria", "__Rickettsiales", "__Rickettsiaceae", "__g"]}},{"id": "denovo1276", "metadata": {"taxonomy": ["Bacteria", "__Acidobacteria", "__RB25", "__o", "__f", "__g"]}},{"id": "denovo1280", "metadata": {"taxonomy": ["Bacteria", "__Proteobacteria", "__Alphaproteobacteria"]}},{"id": "denovo1282", "metadata": {"taxonomy": ["Bacteria", "__Proteobacteria", "__Alphaproteobacteria", "__Rhodobacterales", "__Rhodobacteraceae", "__g"]}},{"id": "denovo1285", "metadata": {"taxonomy": ["Bacteria", "__Proteobacteria", "__Gammaproteobacteria", "__Xanthomonadales", "__Sinobacteraceae", "__JTB255_marine_benthic_group"]}},{"id": "denovo1292", "metadata": {"taxonomy": ["Bacteria", "__Proteobacteria", "__Deltaproteobacteria", "__Myxococcales", "__Haliangiaceae", "__Haliangium"]}},{"id": "denovo1298", "metadata": {"taxonomy": ["Bacteria", "__Proteobacteria", "__Alphaproteobacteria", "__Rhodospirillales", "__Rhodospirillaceae", "__g"]}},{"id": "denovo1300", "metadata": {"taxonomy": ["Bacteria", "__Proteobacteria", "__Gammaproteobacteria", "__E01-9C-26_marine_group", "__f", "__g"]}},{"id": "denovo1303", "metadata": {"taxonomy": ["Bacteria", "__Chloroflexi", "__Caldilineae", "__Caldilineales", "__Caldilineaceae", "__g"]}},{"id": "denovo1305", "metadata": {"taxonomy": ["Bacteria", "__Gemmatimonadetes", "__Gemmatimonadetes", "__BD2-11_terrestrial_group", "__f", "__g"]}},{"id": "denovo1307", "metadata": {"taxonomy": ["Bacteria", "__Proteobacteria", "__Gammaproteobacteria", "__Vibrionales", "__Vibrionaceae", "__Photobacterium"]}},{"id": "denovo1311", "metadata": {"taxonomy": ["Bacteria", "__Proteobacteria", "__Alphaproteobacteria", "__Rickettsiales", "__SM2D12", "__g"]}},{"id": "denovo1313", "metadata": {"taxonomy": ["Bacteria", "__Proteobacteria", "__Alphaproteobacteria", "__DB1-14", "__f", "__g"]}},{"id": "denovo1324", "metadata": {"taxonomy": ["Bacteria", "__Bacteroidetes", "__Cytophagia", "__Order_II_Incertae_Sedis", "__Rhodothermaceae", "__g"]}},{"id": "denovo1327", "metadata": {"taxonomy": ["Bacteria", "__Proteobacteria", "__Deltaproteobacteria", "__Desulfuromonadales", "__Desulfuromonadaceae", "__g"]}},{"id": "denovo1328", "metadata": {"taxonomy": ["Bacteria", "__Chlamydiae", "__Chlamydiae", "__Chlamydiales", "__Simkaniaceae", "__Candidatus_Rhabdochlamydia"]}},{"id": "denovo1329", "metadata": {"taxonomy": ["Bacteria", "__Proteobacteria", "__Alphaproteobacteria", "__Rhodobacterales",

"__Rhodobacteraceae"]}},{"id": "denovo1330", "metadata": {"taxonomy": ["Bacteria", "__Proteobacteria", "__Alphaproteobacteria", "__DB1-14", "__f", "__g"]}},{"id": "denovo1332", "metadata": {"taxonomy": ["Bacteria", "__Cyanobacteria", "__Cyanobacteria", "__SubsectionI", "__FamilyI", "__Synechococcus"]}},{"id": "denovo1335", "metadata": {"taxonomy": ["Bacteria", "__Proteobacteria", "__Alphaproteobacteria", "__SAR11_clade", "__Surface_2", "__g"]}},{"id": "denovo1337", "metadata": {"taxonomy": ["Bacteria", "__Proteobacteria", "__Gammaproteobacteria"]}},{"id": "denovo1338", "metadata": {"taxonomy": ["Bacteria", "__Proteobacteria", "__Gammaproteobacteria", "__Alteromonadales", "__Alteromonadaceae", "__OM60(NOR5)_clade"]}},{"id": "denovo1341", "metadata": {"taxonomy": ["Bacteria", "__Proteobacteria", "__Deltaproteobacteria", "__Desulfuromonadales", "__GR-WP33-58", "__g"]}},{"id": "denovo1342", "metadata": {"taxonomy": ["Bacteria", "__Bacteroidetes", "__Flavobacteria", "__Flavobacteriales", "__Flavobacteriaceae", "__NS2b_marine_group"]}},{"id": "denovo1343", "metadata": {"taxonomy": ["Bacteria", "__Verrucomicrobia", "__Verrucomicrobiae", "__Verrucomicrobiales", "__Verrucomicrobiaceae", "__g"]}},{"id": "denovo1345", "metadata": {"taxonomy": ["Bacteria", "__Proteobacteria", "__Gammaproteobacteria", "__E01-9C-26_marine_group", "__f", "__g"]}},{"id": "denovo1350", "metadata": {"taxonomy": ["Bacteria", "__Chlorobi", "__Chlorobia", "__Chlorobiales", "__Chlorobiaceae", "__Prosthecochloris"]}},{"id": "denovo1359", "metadata": {"taxonomy": ["Unclassified"]}},{"id": "denovo1360", "metadata": {"taxonomy": ["Bacteria", "__Bacteroidetes", "__Sphingobacteriia", "__Sphingobacteriales", "__Saprospiraceae", "__g"]}},{"id": "denovo1363", "metadata": {"taxonomy": ["Bacteria", "__Proteobacteria", "__Alphaproteobacteria", "__Rhodobacterales", "__Rhodobacteraceae", "__g"]}},{"id": "denovo1365", "metadata": {"taxonomy": ["Bacteria", "__Acidobacteria", "__Holophagae", "__TK85", "__f", "__g"]}},{"id": "denovo1370", "metadata": {"taxonomy": ["Bacteria", "__Verrucomicrobia", "__Verrucomicrobiae", "__Verrucomicrobiales", "__Rubritaleaceae", "__Rubritalea"]}},{"id": "denovo1372", "metadata": {"taxonomy": ["Bacteria", "__Proteobacteria", "__Gammaproteobacteria", "__Pseudomonadales", "__Moraxellaceae", "__Acinetobacter"]}},{"id": "denovo1377", "metadata": {"taxonomy": ["Bacteria", "__Spirochaetes", "__Spirochaetes", "__Spirochaetales", "__Spirochaetaceae", "__Spirochaeta"]}},{"id": "denovo1379", "metadata": {"taxonomy": ["Bacteria", "__Proteobacteria", "__Alphaproteobacteria", "__Rhodobacterales", "__Rhodobacteraceae"]}},{"id": "denovo1383", "metadata": {"taxonomy": ["Bacteria", "__Proteobacteria", "__Gammaproteobacteria", "__Chromatiales", "__Granulosicoccaceae", "__Granulosicoccus"]}},{"id": "denovo1385", "metadata": {"taxonomy": ["Bacteria", "__Acidobacteria", "__Acidobacteria", "__32-21", "__f", "__g"]}},{"id": "denovo1390", "metadata": {"taxonomy": ["Bacteria", "__Proteobacteria", "__Deltaproteobacteria", "__Myxococcales", "__Cystobacterineae", "__g"]}},{"id": "denovo1392", "metadata": {"taxonomy": ["Bacteria", "__Proteobacteria", "__Gammaproteobacteria", "__Alteromonadales", "__Alteromonadaceae", "__Congregibacter"]}},

{"id": "denovo1394", "metadata": {"taxonomy": ["Bacteria", "__Proteobacteria", "__Gammaproteobacteria", "__Alteromonadales", "__Alteromonadaceae", "__BD1-7_clade"]}},{"id": "denovo1397", "metadata": {"taxonomy": ["Bacteria", "__Actinobacteria", "__Acidimicrobiia", "__Acidimicrobiales", "__OCS155_marine_group", "__g"]}},{"id": "denovo1402", "metadata": {"taxonomy": ["Bacteria", "__Proteobacteria", "__Gammaproteobacteria", "__Xanthomonadales", "__Sinobacteraceae", "__JTB255_marine_benthic_group"]}},{"id": "denovo1404", "metadata": {"taxonomy": ["Bacteria", "__Bacteroidetes", "__Flavobacteria", "__Flavobacteriales", "__Flavobacteriaceae", "__Gilvibacter"]}},{"id": "denovo1405", "metadata": {"taxonomy": ["Bacteria", "__Cyanobacteria", "__Cyanobacteria", "__SubsectionI", "__FamilyI", "__Synechococcus"]}},{"id": "denovo1408", "metadata": {"taxonomy": ["Bacteria", "__Proteobacteria", "__Alphaproteobacteria", "__Rhodospirillales", "__Rhodospirillaceae", "__g"]}},{"id": "denovo1411", "metadata": {"taxonomy": ["Bacteria", "__Bacteroidetes", "__Flavobacteria", "__Flavobacteriales", "__Cryomorphaceae", "__Owenweeksia"]}},{"id": "denovo1413", "metadata": {"taxonomy": ["Bacteria", "__Cyanobacteria", "__Cyanobacteria", "__SubsectionIII", "__FamilyI"]}},{"id": "denovo1414", "metadata": {"taxonomy": ["Bacteria", "__Proteobacteria", "__Deltaproteobacteria", "__Desulfuromonadales", "__GR-WP33-58", "__g"]}},{"id": "denovo1415", "metadata": {"taxonomy": ["Archaea", "__Euryarchaeota", "__Halobacteria", "__Halobacteriales", "__Deep_Sea_Hydrothermal_Vent_Gp_6(DHVEG-6)", "__g"]}},{"id": "denovo1416", "metadata": {"taxonomy": ["Unclassified"]}},{"id": "denovo1417", "metadata": {"taxonomy": ["Bacteria", "__Chloroflexi", "__Chloroflexi", "__Chloroflexales", "__Chloroflexaceae", "__Roseiflexus"]}},{"id": "denovo1425", "metadata": {"taxonomy": ["Bacteria", "__Cyanobacteria", "__Cyanobacteria", "__SubsectionIII", "__FamilyI", "__Phormidium"]}},{"id": "denovo1427", "metadata": {"taxonomy": ["Bacteria", "__Proteobacteria", "__Gammaproteobacteria", "__NKB5", "__f", "__g"]}},{"id": "denovo1429", "metadata": {"taxonomy": ["Bacteria", "__Chloroflexi", "__Anaerolineae", "__Anaerolineales", "__Anaerolineaceae", "__g"]}},{"id": "denovo1430", "metadata": {"taxonomy": ["Bacteria", "__Proteobacteria", "__Betaproteobacteria", "__Burkholderiales", "__Comamonadaceae"]}},{"id": "denovo1431", "metadata": {"taxonomy": ["Bacteria", "__Chloroflexi", "__Caldilineae", "__Caldilineales", "__Caldilineaceae", "__g"]}},{"id": "denovo1432", "metadata": {"taxonomy": ["Bacteria", "__Proteobacteria", "__Alphaproteobacteria", "__Rhodospirillales", "__Rhodospirillaceae", "__g"]}},{"id": "denovo1436", "metadata": {"taxonomy": ["Bacteria", "__Proteobacteria", "__Deltaproteobacteria", "__Bdellovibrionales", "__Bdellovibrionaceae", "__Bdellovibrio"]}},{"id": "denovo1437", "metadata": {"taxonomy": ["Bacteria", "__Proteobacteria", "__Gammaproteobacteria", "__Oceanospirillales", "__SAR86_clade", "__g"]}},{"id": "denovo1438", "metadata": {"taxonomy": ["Bacteria", "__Proteobacteria", "__Gammaproteobacteria", "__Vibrionales", "__Vibrionaceae", "__Vibrio"]}},{"id": "denovo1440", "metadata":

{"taxonomy": ["Bacteria", "__Proteobacteria", "__Gammaproteobacteria", "__Legionellales", "__Legionellaceae", "__Legionella"]}},{"id": "denovo1442", "metadata": {"taxonomy": ["Bacteria", "__Chlamydiae", "__Chlamydiae", "__Chlamydiales", "__cvE6", "__g"]}},{"id": "denovo1443", "metadata": {"taxonomy": ["Bacteria", "__Proteobacteria", "__Gammaproteobacteria", "__B38", "__f", "__g"]}},{"id": "denovo1444", "metadata": {"taxonomy": ["Bacteria", "__Chloroflexi", "__KD4-96", "__o", "__f", "__g"]}},{"id": "denovo1445", "metadata": {"taxonomy": ["Bacteria", "__Proteobacteria", "__Alphaproteobacteria", "__Rhizobiales", "__Hyphomicrobiaceae", "__g"]}},{"id": "denovo1446", "metadata": {"taxonomy": ["Bacteria", "__Proteobacteria", "__Alphaproteobacteria", "__Caulobacterales", "__Hyphomonadaceae", "__g"]}},{"id": "denovo1448", "metadata": {"taxonomy": ["Bacteria", "__Candidate_division_WS3", "__c", "__o", "__f", "__g"]}},{"id": "denovo1450", "metadata": {"taxonomy": ["Bacteria", "__Proteobacteria", "__Gammaproteobacteria", "__Xanthomonadales", "__Sinobacteraceae", "__JTB255_marine_benthic_group"]}},{"id": "denovo1451", "metadata": {"taxonomy": ["Bacteria", "__Proteobacteria", "__Alphaproteobacteria", "__Rhodobacterales", "__Rhodobacteraceae"]}},{"id": "denovo1452", "metadata": {"taxonomy": ["Bacteria", "__Bacteroidetes", "__Flavobacteria", "__Flavobacteriales", "__Cryomorphaceae", "__Owenweeksia"]}},{"id": "denovo1454", "metadata": {"taxonomy": ["Bacteria", "__Proteobacteria", "__Gammaproteobacteria", "__Pseudomonadales", "__Moraxellaceae", "__Acinetobacter"]}},{"id": "denovo1457", "metadata": {"taxonomy": ["Bacteria", "__Proteobacteria", "__Deltaproteobacteria", "__Myxococcales", "__Haliangiaceae", "__Haliangium"]}},{"id": "denovo1458", "metadata": {"taxonomy": ["Bacteria", "__Proteobacteria", "__Gammaproteobacteria", "__Vibrionales", "__Vibrionaceae", "__Aliivibrio"]}},{"id": "denovo1459", "metadata": {"taxonomy": ["Bacteria", "__Planctomycetes", "__Planctomycetacia", "__Planctomycetales", "__Planctomycetaceae", "__Pirellula"]}},{"id": "denovo1461", "metadata": {"taxonomy": ["Bacteria", "__Bacteroidetes", "__Flavobacteria", "__Flavobacteriales", "__Flavobacteriaceae", "__Algibacter"]}},{"id": "denovo1463", "metadata": {"taxonomy": ["Bacteria", "__Firmicutes", "__Clostridia", "__Clostridiales", "__Clostridiaceae", "__Clostridium"]}},{"id": "denovo1464", "metadata": {"taxonomy": ["Bacteria", "__Acidobacteria", "__Acidobacteria", "__BPC102", "__f", "__g"]}},{"id": "denovo1465", "metadata": {"taxonomy": ["Bacteria", "__Proteobacteria", "__Gammaproteobacteria", "__Alteromonadales", "__Alteromonadaceae", "__OM60(NOR5)_clade"]}},{"id": "denovo1466", "metadata": {"taxonomy": ["Bacteria", "__Proteobacteria", "__Alphaproteobacteria", "__Rhodospirillales", "__Rhodospirillaceae"]}},{"id": "denovo1467", "metadata": {"taxonomy": ["Bacteria", "__Proteobacteria", "__Alphaproteobacteria", "__Rhodobacterales", "__Rhodobacteraceae"]}},{"id": "denovo1468", "metadata": {"taxonomy": ["Bacteria", "__Proteobacteria", "__Gammaproteobacteria", "__Alteromonadales",

"__Colwelliaceae", "__Thalassomonas"]}},{"id": "denovo1471", "metadata": {"taxonomy": ["Bacteria", "__Cyanobacteria", "__Cyanobacteria", "__SubsectionI", "__FamilyI", "__Prochlorococcus"]}},{"id": "denovo1477", "metadata": {"taxonomy": ["Bacteria", "__Proteobacteria", "__Alphaproteobacteria", "__DB1-14", "__f", "__g"]}},{"id": "denovo1478", "metadata": {"taxonomy": ["Bacteria", "__Proteobacteria", "__Deltaproteobacteria", "__Sh765B-TzT-29", "__f", "__g"]}},{"id": "denovo1479", "metadata": {"taxonomy": ["Bacteria", "__Proteobacteria", "__Gammaproteobacteria", "__Oceanospirillales", "__Hahellaceae", "__Endozoicomonas"]}},{"id": "denovo1480", "metadata": {"taxonomy": ["Bacteria", "__Proteobacteria", "__Alphaproteobacteria", "__Rhodobacterales", "__Rhodobacteraceae"]}},{"id": "denovo1482", "metadata": {"taxonomy": ["Bacteria", "__Proteobacteria", "__Alphaproteobacteria", "__Rhodospirillales", "__Rhodospirillaceae", "__g"]}},{"id": "denovo1489", "metadata": {"taxonomy": ["Bacteria", "__Proteobacteria", "__Alphaproteobacteria", "__Rhodospirillales", "__Rhodospirillaceae", "__g"]}},{"id": "denovo1492", "metadata": {"taxonomy": ["Bacteria", "__Proteobacteria", "__Alphaproteobacteria", "__Rhodobacterales", "__Rhodobacteraceae", "__g"]}},{"id": "denovo1496", "metadata": {"taxonomy": ["Bacteria", "__Cyanobacteria", "__4C0d-2", "__o", "__f", "__g"]}},{"id": "denovo1497", "metadata": {"taxonomy": ["Bacteria", "__Proteobacteria", "__Deltaproteobacteria", "__GR-WP33-30", "__f", "__g"]}},{"id": "denovo1499", "metadata": {"taxonomy": ["Bacteria", "__Proteobacteria", "__Alphaproteobacteria", "__Rhodospirillales", "__Rhodospirillaceae", "__g"]}},{"id": "denovo1500", "metadata": {"taxonomy": ["Bacteria", "__Bacteroidetes", "__Flavobacteria", "__Flavobacteriales", "__Flavobacteriaceae", "__g"]}},{"id": "denovo1507", "metadata": {"taxonomy": ["Bacteria", "__Proteobacteria", "__Gammaproteobacteria"]}},{"id": "denovo1509", "metadata": {"taxonomy": ["Bacteria", "__Proteobacteria", "__Alphaproteobacteria", "__Rhodobacterales", "__Rhodobacteraceae", "__Octadecabacter"]}},{"id": "denovo1510", "metadata": {"taxonomy": ["Bacteria", "__Proteobacteria", "__Alphaproteobacteria", "__Rhodospirillales", "__Rhodospirillaceae", "__Oceanibaculum"]}},{"id": "denovo1512", "metadata": {"taxonomy": ["Bacteria", "__Proteobacteria", "__Alphaproteobacteria", "__Rhodospirillales", "__Rhodospirillaceae", "__Defluviicoccus"]}},{"id": "denovo1515", "metadata": {"taxonomy": ["Bacteria", "__Planctomycetes", "__OM190", "__o", "__f", "__g"]}},{"id": "denovo1517", "metadata": {"taxonomy": ["Bacteria", "__Planctomycetes", "__OM190", "__o", "__f", "__g"]}},{"id": "denovo1518", "metadata": {"taxonomy": ["Bacteria", "__Bacteroidetes", "__Cytophagia", "__Order_III_Incertae_Sedis", "__Family_Incertae_Sedis", "__Balneola"]}},{"id": "denovo1524", "metadata": {"taxonomy": ["Bacteria", "__Proteobacteria", "__Gammaproteobacteria"]}},{"id": "denovo1525", "metadata": {"taxonomy": ["Bacteria", "__Planctomycetes", "__OM190", "__o", "__f", "__g"]}},{"id": "denovo1530", "metadata": {"taxonomy": ["Bacteria", "__Proteobacteria", "__Gammaproteobacteria", "__Alteromonadales", "__Ferrimonadaceae", "__Ferrimonas"]}},{"id": "denovo1531",

"metadata": {"taxonomy": ["Bacteria", "__Proteobacteria", "__Gammaproteobacteria", "__Pseudomonadales", "__Moraxellaceae", "__Acinetobacter"]}},{"id": "denovo1538", "metadata": {"taxonomy": ["Bacteria", "__Actinobacteria", "__Acidimicrobiia", "__Acidimicrobiales", "__Sva0996_marine_group", "__g"]}},{"id": "denovo1542", "metadata": {"taxonomy": ["Bacteria", "__Planctomycetes", "__Planctomycetacia", "__Planctomycetales", "__Planctomycetaceae", "__Blastopirellula"]}},{"id": "denovo1543", "metadata": {"taxonomy": ["Bacteria", "__Proteobacteria", "__Alphaproteobacteria", "__Rhodospirillales", "__Rhodospirillaceae", "__Defluviicoccus"]}},{"id": "denovo1544", "metadata": {"taxonomy": ["Bacteria", "__Proteobacteria", "__Gammaproteobacteria", "__Alteromonadales", "__Colwelliaceae", "__Thalassomonas"]}},{"id": "denovo1545", "metadata": {"taxonomy": ["Bacteria", "__Proteobacteria", "__Deltaproteobacteria", "__Desulfobacterales", "__Desulfobacteraceae", "__Desulfospira"]}},{"id": "denovo1550", "metadata": {"taxonomy": ["Bacteria", "__Proteobacteria", "__Gammaproteobacteria", "__Pseudomonadales", "__Pseudomonadaceae", "__Pseudomonas"]}},{"id": "denovo1551", "metadata": {"taxonomy": ["Bacteria"]}},{"id": "denovo1552", "metadata": {"taxonomy": ["Bacteria", "__Proteobacteria", "__Gammaproteobacteria", "__Pseudomonadales", "__Moraxellaceae", "__Acinetobacter"]}},{"id": "denovo1553", "metadata": {"taxonomy": ["Bacteria", "__Planctomycetes", "__OM190", "__o", "__f", "__g"]}},{"id": "denovo1556", "metadata": {"taxonomy": ["Bacteria", "__Proteobacteria", "__Alphaproteobacteria", "__Rhizobiales", "__Rhizobiaceae", "__Rhizobium"]}},{"id": "denovo1557", "metadata": {"taxonomy": ["Bacteria", "__Bacteroidetes", "__Sphingobacteriia", "__Sphingobacteriales", "__Saprospiraceae", "__g"]}},{"id": "denovo1558", "metadata": {"taxonomy": ["Bacteria", "__Proteobacteria", "__Gammaproteobacteria", "__Alteromonadales", "__Alteromonadaceae", "__OM60(NOR5)_clade"]}},{"id": "denovo1559", "metadata": {"taxonomy": ["Bacteria", "__Proteobacteria", "__Alphaproteobacteria", "__SB1-18", "__f", "__g"]}},{"id": "denovo1561", "metadata": {"taxonomy": ["Bacteria", "__Proteobacteria", "__Gammaproteobacteria", "__Oceanospirillales", "__OM182_clade", "__g"]}},{"id": "denovo1562", "metadata": {"taxonomy": ["Bacteria", "__Proteobacteria", "__SPOTSOCT00m83", "__o", "__f", "__g"]}},{"id": "denovo1563", "metadata": {"taxonomy": ["Bacteria", "__Cyanobacteria", "__MLE1-12", "__o", "__f", "__g"]}},{"id": "denovo1570", "metadata": {"taxonomy": ["Bacteria", "__Proteobacteria", "__Gammaproteobacteria", "__Pseudomonadales", "__Moraxellaceae", "__Acinetobacter"]}},{"id": "denovo1571", "metadata": {"taxonomy": ["Bacteria", "__Acidobacteria", "__Holophagae", "__CA002", "__f", "__g"]}},{"id": "denovo1574", "metadata": {"taxonomy": ["Bacteria", "__Bacteroidetes", "__Sphingobacteriia", "__Sphingobacteriales", "__Saprospiraceae", "__g"]}},{"id": "denovo1575", "metadata": {"taxonomy": ["Bacteria", "__Proteobacteria", "__Betaproteobacteria", "__Burkholderiales", "__Alcaligenaceae", "__Derxia"]}},{"id": "denovo1577", "metadata":

{"taxonomy": ["Bacteria", "__Proteobacteria",
"__Alphaproteobacteria"]}},{"id": "denovo1578", "metadata":
{"taxonomy": ["Bacteria", "__Proteobacteria", "__Alphaproteobacteria",
"__Caulobacterales", "__Hyphomonadaceae", "__Hellea"]}},{"id":
"denovo1579", "metadata": {"taxonomy": ["Bacteria", "__Bacteroidetes",
"__Sphingobacteriia", "__Sphingobacteriales", "__Chitinophagaceae",
"__g"]}},{"id": "denovo1580", "metadata": {"taxonomy": ["Bacteria",
"__Acidobacteria", "__RB25", "__o", "__f", "__g"]}},{"id":
"denovo1582", "metadata": {"taxonomy": ["Bacteria",
"__Proteobacteria", "__Gammaproteobacteria", "__Alteromonadales",
"__Pseudoalteromonadaceae", "__Pseudoalteromonas"]}},{"id":
"denovo1584", "metadata": {"taxonomy": ["Bacteria", "__Bacteroidetes",
"__Flavobacteria", "__Flavobacteriales", "__Cryomorphaceae",
"__NS7_marine_group"]}},{"id": "denovo1585", "metadata": {"taxonomy":
["Bacteria", "__Bacteroidetes", "__Flavobacteria",
"__Flavobacteriales", "__Cryomorphaceae", "__Owenweeksia"]}},{"id":
"denovo1587", "metadata": {"taxonomy": ["Bacteria", "__Cyanobacteria",
"__Cyanobacteria", "__SubsectionII", "__FamilyII",
"__Chroococcidiopsis"]}},{"id": "denovo1588", "metadata": {"taxonomy":
["Bacteria", "__Proteobacteria", "__Gammaproteobacteria",
"__Alteromonadales", "__Alteromonadaceae", "__Haliea"]}},{"id":
"denovo1589", "metadata": {"taxonomy": ["Bacteria",
"__Proteobacteria", "__Gammaproteobacteria", "__Alteromonadales",
"__Alteromonadaceae", "__Aestuariibacter"]}},{"id": "denovo1594",
"metadata": {"taxonomy": ["Bacteria", "__Proteobacteria",
"__Gammaproteobacteria", "__Alteromonadales", "__Alteromonadaceae",
"__OM60(NOR5)_clade"]}},{"id": "denovo1596", "metadata": {"taxonomy":
["Bacteria", "__Proteobacteria", "__Gammaproteobacteria",
"__Pseudomonadales", "__Moraxellaceae", "__Acinetobacter"]}},{"id":
"denovo1600", "metadata": {"taxonomy": ["Bacteria",
"__Bacteroidetes"]}},{"id": "denovo1605", "metadata": {"taxonomy":
["Bacteria", "__Cyanobacteria", "__Cyanobacteria", "__SubsectionIII",
"__FamilyI"]}},{"id": "denovo1608", "metadata": {"taxonomy":
["Bacteria", "__Gemmatimonadetes", "__Gemmatimonadetes",
"__BD2-11_terrestrial_group", "__f", "__g"]}},{"id": "denovo1611",
"metadata": {"taxonomy": ["Bacteria", "__Proteobacteria",
"__Alphaproteobacteria", "__SAR11_clade", "__Surface_1",
"__Candidatus_Pelagibacter"]}},{"id": "denovo1613", "metadata":
{"taxonomy": ["Bacteria", "__Proteobacteria", "__Alphaproteobacteria",
"__Rickettsiales", "__S25-593", "__g"]}},{"id": "denovo1616",
"metadata": {"taxonomy": ["Bacteria", "__Cyanobacteria",
"__ML635J-21", "__o", "__f", "__g"]}},{"id": "denovo1621", "metadata":
{"taxonomy": ["Bacteria", "__Proteobacteria", "__Gammaproteobacteria",
"__Xanthomonadales", "__Sinobacteraceae",
"__JTB255_marine_benthic_group"]}},{"id": "denovo1622", "metadata":
{"taxonomy": ["Bacteria", "__Proteobacteria", "__Alphaproteobacteria",
"__Rhodobacterales", "__Rhodobacteraceae"]}},{"id": "denovo1627",
"metadata": {"taxonomy": ["Bacteria", "__Bacteroidetes",
"__Sphingobacteriia", "__Sphingobacteriales", "__Saprospiraceae",
"__g"]}},{"id": "denovo1630", "metadata": {"taxonomy": ["Bacteria",

"__Proteobacteria", "__Alphaproteobacteria", "__Rickettsiales", "__SAR116_clade", "__g"]}},{"id": "denovo1632", "metadata": {"taxonomy": ["Bacteria", "__Proteobacteria", "__Deltaproteobacteria", "__Myxococcales", "__0319-6G20", "__g"]}},{"id": "denovo1634", "metadata": {"taxonomy": ["Bacteria", "__Proteobacteria", "__Gammaproteobacteria", "__Pseudomonadales", "__Moraxellaceae", "__Acinetobacter"]}},{"id": "denovo1636", "metadata": {"taxonomy": ["Bacteria", "__Verrucomicrobia", "__Verrucomicrobiae", "__Verrucomicrobiales", "__DEV007", "__g"]}},{"id": "denovo1637", "metadata": {"taxonomy": ["Bacteria", "__Proteobacteria", "__Gammaproteobacteria", "__Oceanospirillales", "__Oleiphilaceae", "__Oleiphilus"]}},{"id": "denovo1638", "metadata": {"taxonomy": ["Bacteria", "__Proteobacteria", "__Deltaproteobacteria", "__Bdellovibrionales", "__Bdellovibrionaceae", "__OM27_clade"]}},{"id": "denovo1641", "metadata": {"taxonomy": ["Bacteria", "__Proteobacteria", "__Gammaproteobacteria", "__Oceanospirillales", "__OM182_clade", "__g"]}},{"id": "denovo1642", "metadata": {"taxonomy": ["Bacteria", "__Chlamydiae", "__Chlamydiae", "__Chlamydiales", "__Simkaniaceae", "__g"]}},{"id": "denovo1644", "metadata": {"taxonomy": ["Bacteria", "__Proteobacteria", "__JTB23", "__o", "__f", "__g"]}},{"id": "denovo1645", "metadata": {"taxonomy": ["Bacteria", "__Proteobacteria", "__Gammaproteobacteria"]}},{"id": "denovo1646", "metadata": {"taxonomy": ["Bacteria", "__Cyanobacteria", "__Cyanobacteria", "__SubsectionII", "__FamilyII", "__Pleurocapsa"]}},{"id": "denovo1647", "metadata": {"taxonomy": ["Bacteria", "__Proteobacteria", "__Betaproteobacteria", "__Burkholderiales", "__Comamonadaceae"]}},{"id": "denovo1648", "metadata": {"taxonomy": ["Bacteria", "__Bacteroidetes", "__Cytophagia", "__Cytophagales", "__Flammeovirgaceae", "__g"]}},{"id": "denovo1660", "metadata": {"taxonomy": ["Bacteria", "__Proteobacteria", "__Alphaproteobacteria", "__Rhodospirillales", "__Rhodospirillaceae", "__g"]}},{"id": "denovo1663", "metadata": {"taxonomy": ["Bacteria", "__Proteobacteria", "__Alphaproteobacteria", "__Rickettsiales", "__SAR116_clade", "__Candidatus_Puniceispirillum"]}},{"id": "denovo1666", "metadata": {"taxonomy": ["Bacteria", "__Bacteroidetes", "__Cytophagia", "__Cytophagales", "__Flammeovirgaceae", "__Fulvivirga"]}},{"id": "denovo1670", "metadata": {"taxonomy": ["Bacteria", "__Proteobacteria", "__Gammaproteobacteria", "__Vibrionales", "__Vibrionaceae", "__Vibrio"]}},{"id": "denovo1672", "metadata": {"taxonomy": ["Bacteria", "__Actinobacteria", "__Actinobacteria", "__Corynebacteriales", "__Mycobacteriaceae", "__Mycobacterium"]}},{"id": "denovo1674", "metadata": {"taxonomy": ["Bacteria", "__Proteobacteria", "__Alphaproteobacteria", "__Caulobacterales", "__Hyphomonadaceae", "__Hellea"]}},{"id": "denovo1675", "metadata": {"taxonomy": ["Bacteria", "__Proteobacteria", "__Alphaproteobacteria", "__Rhodospirillales", "__Rhodospirillaceae", "__Defluviicoccus"]}},{"id": "denovo1679", "metadata": {"taxonomy": ["Bacteria", "__Proteobacteria", "__Gammaproteobacteria"]}},{"id": "denovo1682", "metadata": {"taxonomy": ["Bacteria", "__Proteobacteria", "__Gammaproteobacteria",

"__MD2904-B13", "__f", "__g"]}},{"id": "denovo1687", "metadata": {"taxonomy": ["Bacteria", "__Proteobacteria", "__Gammaproteobacteria", "__Oceanospirillales", "__Hahellaceae", "__Endozoicomonas"]}},{"id": "denovo1688", "metadata": {"taxonomy": ["Bacteria", "__Proteobacteria", "__Alphaproteobacteria", "__Rhodobacterales", "__Rhodobacteraceae"]}},{"id": "denovo1690", "metadata": {"taxonomy": ["Bacteria", "__Proteobacteria", "__Alphaproteobacteria", "__SB1-18", "__f", "__g"]}},{"id": "denovo1695", "metadata": {"taxonomy": ["Bacteria", "__Proteobacteria", "__Gammaproteobacteria", "__NKB5", "__f", "__g"]}},{"id": "denovo1696", "metadata": {"taxonomy": ["Bacteria", "__Proteobacteria", "__Alphaproteobacteria", "__Rhodobacterales", "__Rhodobacteraceae"]}},{"id": "denovo1697", "metadata": {"taxonomy": ["Bacteria", "__Proteobacteria", "__Gammaproteobacteria", "__Xanthomonadales", "__Xanthomonadaceae", "__Thermomonas"]}},{"id": "denovo1700", "metadata": {"taxonomy": ["Bacteria", "__Proteobacteria", "__Deltaproteobacteria", "__FW113", "__f", "__g"]}},{"id": "denovo1701", "metadata": {"taxonomy": ["Bacteria", "__Proteobacteria", "__Gammaproteobacteria", "__Oceanospirillales", "__Oceanospirillaceae", "__Pseudospirillum"]}},{"id": "denovo1703", "metadata": {"taxonomy": ["Bacteria", "__Proteobacteria", "__Deltaproteobacteria", "__Desulfuromonadales", "__GR-WP33-58", "__g"]}},{"id": "denovo1706", "metadata": {"taxonomy": ["Bacteria", "__Proteobacteria", "__Alphaproteobacteria", "__Rhodospirillales"]}},{"id": "denovo1707", "metadata": {"taxonomy": ["Bacteria", "__Proteobacteria", "__TA18", "__o", "__f", "__g"]}},{"id": "denovo1712", "metadata": {"taxonomy": ["Bacteria", "__Proteobacteria", "__Alphaproteobacteria", "__Rhizobiales", "__Phyllobacteriaceae", "__g"]}},{"id": "denovo1713", "metadata": {"taxonomy": ["Bacteria", "__Cyanobacteria", "__Cyanobacteria", "__SubsectionIII", "__FamilyI"]}},{"id": "denovo1716", "metadata": {"taxonomy": ["Bacteria", "__Bacteroidetes", "__Sphingobacteriia", "__Sphingobacteriales", "__Saprospiraceae", "__g"]}},{"id": "denovo1719", "metadata": {"taxonomy": ["Bacteria", "__Proteobacteria", "__Alphaproteobacteria", "__Rhodobacterales", "__Rhodobacteraceae"]}},{"id": "denovo1723", "metadata": {"taxonomy": ["Archaea", "__Thaumarchaeota", "__Marine_Group_I", "__o", "__f", "__g"]}},{"id": "denovo1724", "metadata": {"taxonomy": ["Bacteria", "__Bacteroidetes", "__Cytophagia", "__Order_II_Incertae_Sedis", "__Rhodothermaceae", "__g"]}},{"id": "denovo1725", "metadata": {"taxonomy": ["Bacteria", "__Proteobacteria", "__Deltaproteobacteria", "__Desulfuromonadales"]}},{"id": "denovo1726", "metadata": {"taxonomy": ["Bacteria", "__Cyanobacteria", "__Cyanobacteria"]}},{"id": "denovo1728", "metadata": {"taxonomy": ["Bacteria", "__Proteobacteria", "__Alphaproteobacteria", "__Rhizobiales", "__Hyphomicrobiaceae", "__Cucumibacter"]}},{"id": "denovo1731", "metadata": {"taxonomy": ["Bacteria", "__Proteobacteria", "__Gammaproteobacteria", "__NKB5", "__f", "__g"]}},{"id": "denovo1733", "metadata": {"taxonomy": ["Bacteria", "__Proteobacteria", "__Deltaproteobacteria", "__Myxococcales", "__Haliangiaceae", "__Haliangium"]}},{"id": "denovo1736", "metadata":

{"taxonomy": ["Bacteria", "__Proteobacteria", "__Gammaproteobacteria", "__Alteromonadales", "__Ferrimonadaceae", "__Ferrimonas"]}},{"id": "denovo1739", "metadata": {"taxonomy": ["Bacteria", "__Proteobacteria", "__Deltaproteobacteria", "__Desulfobacterales", "__Desulfobacteraceae", "__SEEP-SRB1"]}},{"id": "denovo1741", "metadata": {"taxonomy": ["Bacteria", "__Proteobacteria", "__Alphaproteobacteria", "__DB1-14", "__f", "__g"]}},{"id": "denovo1749", "metadata": {"taxonomy": ["Bacteria", "__Proteobacteria", "__Deltaproteobacteria", "__Desulfobacterales", "__Desulfobacteraceae", "__Desulfospira"]}},{"id": "denovo1750", "metadata": {"taxonomy": ["Bacteria", "__Bacteroidetes", "__Flavobacteria", "__Flavobacteriales", "__Flavobacteriaceae", "__Gilvibacter"]}},{"id": "denovo1751", "metadata": {"taxonomy": ["Bacteria", "__Bacteroidetes", "__Cytophagia", "__Order_II_Incertae_Sedis", "__Rhodothermaceae", "__g"]}},{"id": "denovo1754", "metadata": {"taxonomy": ["Bacteria", "__Proteobacteria", "__Alphaproteobacteria", "__Rhodospirillales", "__Rhodospirillaceae", "__g"]}},{"id": "denovo1760", "metadata": {"taxonomy": ["Bacteria", "__Proteobacteria", "__Alphaproteobacteria", "__Rickettsiales", "__TK34", "__g"]}},{"id": "denovo1765", "metadata": {"taxonomy": ["Bacteria", "__Proteobacteria", "__Gammaproteobacteria", "__Pseudomonadales", "__Moraxellaceae", "__Acinetobacter"]}},{"id": "denovo1767", "metadata": {"taxonomy": ["Bacteria", "__Proteobacteria", "__Deltaproteobacteria", "__Desulfurellales", "__Desulfurellaceae", "__g"]}},{"id": "denovo1769", "metadata": {"taxonomy": ["Bacteria", "__Proteobacteria"]}},{"id": "denovo1773", "metadata": {"taxonomy": ["Bacteria", "__Proteobacteria", "__Gammaproteobacteria", "__Oceanospirillales", "__Oceanospirillaceae"]}},{"id": "denovo1774", "metadata": {"taxonomy": ["Bacteria", "__Proteobacteria", "__Deltaproteobacteria", "__Desulfobacterales", "__Desulfobulbaceae", "__Desulfobulbus"]}},{"id": "denovo1775", "metadata": {"taxonomy": ["Bacteria", "__Proteobacteria", "__Deltaproteobacteria", "__Desulfobacterales", "__Desulfobacteraceae"]}},{"id": "denovo1777", "metadata": {"taxonomy": ["Bacteria", "__Proteobacteria", "__Deltaproteobacteria", "__Myxococcales", "__Sandaracinaceae", "__Sandaracinus"]}},{"id": "denovo1778", "metadata": {"taxonomy": ["Bacteria", "__Proteobacteria", "__Deltaproteobacteria", "__Desulfobacterales", "__Desulfobacteraceae", "__g"]}},{"id": "denovo1779", "metadata": {"taxonomy": ["Bacteria", "__Proteobacteria", "__Alphaproteobacteria", "__Rhodospirillales", "__Rhodospirillaceae", "__Nisaea"]}},{"id": "denovo1784", "metadata": {"taxonomy": ["Bacteria", "__Proteobacteria", "__Alphaproteobacteria", "__Sphingomonadales", "__Sphingomonadaceae", "__Sphingomonas"]}},{"id": "denovo1786", "metadata": {"taxonomy": ["Bacteria", "__Proteobacteria", "__Alphaproteobacteria", "__Rhodospirillales"]}},{"id": "denovo1800", "metadata": {"taxonomy": ["Bacteria", "__Cyanobacteria", "__Cyanobacteria", "__SubsectionIII", "__FamilyI", "__Phormidium"]}},{"id": "denovo1805", "metadata": {"taxonomy": ["Bacteria", "__Proteobacteria", "__Gammaproteobacteria"]}},{"id": "denovo1807",

"metadata": {"taxonomy": ["Bacteria", "__Proteobacteria",
"__Deltaproteobacteria", "__Desulfobacterales",
"__Desulfobacteraceae", "__Sva0081_sediment_group"]}},{"id":
"denovo1818", "metadata": {"taxonomy": ["Bacteria",
"__Proteobacteria", "__Deltaproteobacteria", "__Bdellovibrionales",
"__Bacteriovoracaceae", "__Peredibacter"]}},{"id": "denovo1819",
"metadata": {"taxonomy": ["Bacteria", "__Chloroflexi",
"__SAR202_clade", "__o", "__f", "__g"]}},{"id": "denovo1821",
"metadata": {"taxonomy": ["Bacteria", "__Proteobacteria",
"__Gammaproteobacteria", "__Order_Incertae_Sedis",
"__Family_Incertae_Sedis", "__Sedimenticola"]}},{"id": "denovo1831",
"metadata": {"taxonomy": ["Bacteria", "__Candidate_division_TM7",
"__c", "__o", "__f", "__g"]}},{"id": "denovo1832", "metadata":
{"taxonomy": ["Bacteria", "__Proteobacteria", "__Gammaproteobacteria",
"__Pseudomonadales", "__Moraxellaceae", "__Acinetobacter"]}},{"id":
"denovo1833", "metadata": {"taxonomy": ["Bacteria",
"__Candidate_division_OD1", "__c", "__o", "__f", "__g"]}},{"id":
"denovo1834", "metadata": {"taxonomy": ["Bacteria", "__Bacteroidetes",
"__Cytophagia", "__Cytophagales", "__Flammeovirgaceae",
"__Persicobacter"]}},{"id": "denovo1836", "metadata": {"taxonomy":
["Bacteria", "__Proteobacteria", "__Alphaproteobacteria",
"__Rhodobacterales", "__Rhodobacteraceae", "__Maritimibacter"]}},
{"id": "denovo1838", "metadata": {"taxonomy": ["Bacteria",
"__Proteobacteria", "__Deltaproteobacteria", "__Myxococcales",
"__0319-6G20", "__g"]}},{"id": "denovo1845", "metadata": {"taxonomy":
["Archaea", "__Euryarchaeota", "__Thermoplasmata",
"__Thermoplasmatales", "__Marine_Group_II", "__g"]}},{"id":
"denovo1846", "metadata": {"taxonomy": ["Bacteria",
"__Proteobacteria", "__Alphaproteobacteria", "__Rhodospirillales",
"__Rhodospirillaceae", "__g"]}},{"id": "denovo1847", "metadata":
{"taxonomy": ["Bacteria", "__Proteobacteria", "__Alphaproteobacteria",
"__Rhodobacterales", "__Rhodobacteraceae"]}},{"id": "denovo1848",
"metadata": {"taxonomy": ["Bacteria", "__Proteobacteria",
"__Gammaproteobacteria", "__Xanthomonadales", "__Sinobacteraceae"]}},
{"id": "denovo1851", "metadata": {"taxonomy": ["Bacteria",
"__Proteobacteria", "__Gammaproteobacteria", "__Alteromonadales",
"__Alteromonadaceae", "__Haliea"]}},{"id": "denovo1853", "metadata":
{"taxonomy": ["Bacteria", "__Proteobacteria", "__Elev-16S-509", "__o",
"__f", "__g"]}},{"id": "denovo1857", "metadata": {"taxonomy":
["Bacteria", "__Gemmatimonadetes", "__Gemmatimonadetes",
"__PAUC43f_marine_benthic_group", "__f", "__g"]}},{"id": "denovo1858",
"metadata": {"taxonomy": ["Bacteria", "__Proteobacteria",
"__Deltaproteobacteria", "__Bdellovibrionales",
"__Bdellovibrionaceae", "__OM27_clade"]}},{"id": "denovo1861",
"metadata": {"taxonomy": ["Bacteria", "__Bacteroidetes",
"__Flavobacteria", "__Flavobacteriales", "__NS9_marine_group",
"__g"]}},{"id": "denovo1862", "metadata": {"taxonomy": ["Bacteria",
"__Proteobacteria", "__Gammaproteobacteria", "__Order_Incertae_Sedis",
"__Family_Incertae_Sedis", "__Marinicella"]}},{"id": "denovo1865",
"metadata": {"taxonomy": ["Bacteria", "__Chloroflexi",

"__Caldilineae", "__Caldilineales", "__Caldilineaceae", "__g"]}},
{"id": "denovo1866", "metadata": {"taxonomy": ["Bacteria",
"__Proteobacteria", "__Gammaproteobacteria", "__Order_Incertae_Sedis",
"__Family_Incertae_Sedis", "__Marinicella"]}},{"id": "denovo1867",
"metadata": {"taxonomy": ["Bacteria", "__Cyanobacteria",
"__Cyanobacteria", "__SubsectionIII", "__FamilyI", "__Phormidium"]}},
{"id": "denovo1871", "metadata": {"taxonomy": ["Bacteria",
"__Proteobacteria", "__Alphaproteobacteria", "__Rhodobacterales",
"__Rhodobacteraceae", "__Phaeobacter"]}},{"id": "denovo1872",
"metadata": {"taxonomy": ["Bacteria", "__Proteobacteria",
"__Alphaproteobacteria", "__DB1-14", "__f", "__g"]}},{"id":
"denovo1874", "metadata": {"taxonomy": ["Bacteria",
"__Proteobacteria", "__Alphaproteobacteria", "__Rhizobiales",
"__Brucellaceae"]}},{"id": "denovo1876", "metadata": {"taxonomy":
["Bacteria", "__Proteobacteria", "__Gammaproteobacteria",
"__Chromatiales", "__Chromatiaceae", "__Nitrosococcus"]}},{"id":
"denovo1880", "metadata": {"taxonomy": ["Bacteria", "__Bacteroidetes",
"__Cytophagia", "__Cytophagales", "__Flammeovirgaceae"]}},{"id":
"denovo1881", "metadata": {"taxonomy": ["Bacteria",
"__Planctomycetes", "__Planctomycetacia", "__Planctomycetales",
"__Planctomycetaceae", "__g"]}},{"id": "denovo1882", "metadata":
{"taxonomy": ["Bacteria", "__Proteobacteria", "__Deltaproteobacteria",
"__Sh765B-TzT-29", "__f", "__g"]}},{"id": "denovo1886", "metadata":
{"taxonomy": ["Bacteria", "__Proteobacteria", "__Deltaproteobacteria",
"__Sh765B-TzT-29", "__f", "__g"]}},{"id": "denovo1888", "metadata":
{"taxonomy": ["Bacteria", "__Proteobacteria", "__Gammaproteobacteria",
"__Oceanospirillales", "__Oceanospirillaceae", "__Amphritea"]}},{"id":
"denovo1890", "metadata": {"taxonomy": ["Bacteria", "__Bacteroidetes",
"__Flavobacteria", "__Flavobacteriales", "__Flavobacteriaceae",
"__Gilvibacter"]}},{"id": "denovo1893", "metadata": {"taxonomy":
["Bacteria", "__Proteobacteria", "__Alphaproteobacteria",
"__Rhodobacterales", "__Rhodobacteraceae", "__g"]}},{"id":
"denovo1896", "metadata": {"taxonomy": ["Bacteria", "__Bacteroidetes",
"__Sphingobacteriia", "__Sphingobacteriales", "__Saprospiraceae",
"__g"]}},{"id": "denovo1899", "metadata": {"taxonomy": ["Bacteria",
"__Proteobacteria", "__Deltaproteobacteria", "__Desulfobacterales",
"__Desulfobacteraceae", "__Desulfobacula"]}},{"id": "denovo1901",
"metadata": {"taxonomy": ["Bacteria", "__Proteobacteria",
"__Alphaproteobacteria", "__Rhodospirillales", "__Rhodospirillaceae",
"__Pelagibius"]}},{"id": "denovo1902", "metadata": {"taxonomy":
["Bacteria", "__Proteobacteria", "__Alphaproteobacteria",
"__Rhizobiales", "__Phyllobacteriaceae", "__Ahrensia"]}},{"id":
"denovo1904", "metadata": {"taxonomy": ["Bacteria",
"__Proteobacteria", "__Deltaproteobacteria", "__Sh765B-TzT-29", "__f",
"__g"]}},{"id": "denovo1905", "metadata": {"taxonomy": ["Bacteria",
"__Proteobacteria", "__Gammaproteobacteria", "__Pseudomonadales",
"__Moraxellaceae", "__Acinetobacter"]}},{"id": "denovo1906",
"metadata": {"taxonomy": ["Bacteria", "__Bacteroidetes",
"__Sphingobacteriia", "__Sphingobacteriales", "__Saprospiraceae",
"__Aureispira"]}},{"id": "denovo1908", "metadata": {"taxonomy":

["Bacteria", "__Proteobacteria", "__Alphaproteobacteria",
"__Rhodospirillales", "__Rhodospirillaceae", "__Pelagibius"]}},{"id":
"denovo1909", "metadata": {"taxonomy": ["Bacteria", "__TM6", "__c",
"__o", "__f", "__g"]}},{"id": "denovo1912", "metadata": {"taxonomy":
["Bacteria", "__Bacteroidetes", "__Sphingobacteriia",
"__Sphingobacteriales", "__Saprospiraceae", "__g"]}},{"id":
"denovo1913", "metadata": {"taxonomy": ["Bacteria",
"__Proteobacteria", "__Alphaproteobacteria", "__Rhodobacterales",
"__Rhodobacteraceae", "__g"]}},{"id": "denovo1914", "metadata":
{"taxonomy": ["Bacteria", "__Proteobacteria", "__Alphaproteobacteria",
"__DB1-14", "__f", "__g"]}},{"id": "denovo1916", "metadata":
{"taxonomy": ["Bacteria", "__Proteobacteria", "__Deltaproteobacteria",
"__Desulfobacterales", "__Desulfobacteraceae", "__Desulfobacter"]}},
{"id": "denovo1917", "metadata": {"taxonomy": ["Bacteria",
"__Proteobacteria", "__Alphaproteobacteria", "__Rickettsiales",
"__SM2D12", "__g"]}},{"id": "denovo1918", "metadata": {"taxonomy":
["Bacteria", "__Acidobacteria", "__RB25", "__o", "__f", "__g"]}},
{"id": "denovo1919", "metadata": {"taxonomy": ["Bacteria",
"__Proteobacteria", "__Deltaproteobacteria",
"__SAR324_clade(Marine_group_B)", "__f", "__g"]}},{"id": "denovo1921",
"metadata": {"taxonomy": ["Bacteria", "__Bacteroidetes",
"__Flavobacteria", "__Flavobacteriales", "__Flavobacteriaceae"]}},
{"id": "denovo1923", "metadata": {"taxonomy": ["Bacteria",
"__Proteobacteria", "__Alphaproteobacteria", "__Caulobacterales",
"__Hyphomonadaceae", "__Hyphomonas"]}},{"id": "denovo1925",
"metadata": {"taxonomy": ["Bacteria", "__Proteobacteria",
"__Alphaproteobacteria", "__Rhodospirillales", "__Rhodospirillaceae",
"__Defluviicoccus"]}},{"id": "denovo1934", "metadata": {"taxonomy":
["Bacteria", "__Planctomycetes", "__Phycisphaerae",
"__Phycisphaerales"]}},{"id": "denovo1938", "metadata": {"taxonomy":
["Bacteria", "__Proteobacteria", "__Gammaproteobacteria", "__BD3-1",
"__f", "__g"]}},{"id": "denovo1939", "metadata": {"taxonomy":
["Bacteria", "__Proteobacteria", "__Deltaproteobacteria",
"__Desulfobacterales", "__Desulfobacteraceae", "__Desulfofaba"]}},
{"id": "denovo1941", "metadata": {"taxonomy": ["Bacteria",
"__Proteobacteria", "__Alphaproteobacteria", "__OCS116_clade", "__f",
"__g"]}},{"id": "denovo1945", "metadata": {"taxonomy": ["Bacteria",
"__Actinobacteria", "__Acidimicrobiia", "__Acidimicrobiales",
"__Sva0996_marine_group", "__g"]}},{"id": "denovo1949", "metadata":
{"taxonomy": ["Bacteria", "__Bacteroidetes", "__Flavobacteria",
"__Flavobacteriales", "__Cryomorphaceae"]}},{"id": "denovo1952",
"metadata": {"taxonomy": ["Bacteria", "__Proteobacteria",
"__Gammaproteobacteria", "__KI89A_clade", "__f", "__g"]}},{"id":
"denovo1953", "metadata": {"taxonomy": ["Bacteria",
"__Actinobacteria", "__Acidimicrobiia", "__Acidimicrobiales",
"__OCS155_marine_group", "__g"]}},{"id": "denovo1956", "metadata":
{"taxonomy": ["Bacteria", "__Bacteroidetes", "__Flavobacteria",
"__Flavobacteriales", "__Flavobacteriaceae", "__Aquimarina"]}},{"id":
"denovo1957", "metadata": {"taxonomy": ["Bacteria", "__Bacteroidetes",
"__Cytophagia", "__Cytophagales", "__Flammeovirgaceae",

"__Ekhidna"]}},{"id": "denovo1963", "metadata": {"taxonomy": ["Bacteria", "__Bacteroidetes", "__Sphingobacteriia", "__Sphingobacteriales"]}},{"id": "denovo1965", "metadata": {"taxonomy": ["Bacteria", "__Acidobacteria", "__Acidobacteria", "__BPC015", "__f", "__g"]}},{"id": "denovo1966", "metadata": {"taxonomy": ["Bacteria", "__Proteobacteria", "__Epsilonproteobacteria", "__Campylobacterales", "__Campylobacteraceae", "__Arcobacter"]}},{"id": "denovo1967", "metadata": {"taxonomy": ["Bacteria", "__Proteobacteria", "__Alphaproteobacteria", "__Rhizobiales", "__uncultured", "__g"]}}, {"id": "denovo1969", "metadata": {"taxonomy": ["Bacteria", "__Proteobacteria", "__Gammaproteobacteria", "__E01-9C-26_marine_group", "__f", "__g"]}},{"id": "denovo1970", "metadata": {"taxonomy": ["Bacteria", "__Acidobacteria", "__Holophagae", "__CA002", "__f", "__g"]}},{"id": "denovo1973", "metadata": {"taxonomy": ["Bacteria", "__Proteobacteria", "__Alphaproteobacteria", "__Rhizobiales", "__Rhodobiaceae", "__Rhodobium"]}},{"id": "denovo1975", "metadata": {"taxonomy": ["Bacteria", "__Proteobacteria", "__Alphaproteobacteria", "__DB1-14", "__f", "__g"]}},{"id": "denovo1976", "metadata": {"taxonomy": ["Bacteria", "__Actinobacteria", "__Acidimicrobiia", "__Acidimicrobiales", "__OCS155_marine_group", "__g"]}},{"id": "denovo1981", "metadata": {"taxonomy": ["Bacteria", "__Planctomycetes", "__Planctomycetacia", "__Planctomycetales", "__Planctomycetaceae", "__Pir4_lineage"]}},{"id": "denovo1985", "metadata": {"taxonomy": ["Bacteria", "__Proteobacteria", "__Gammaproteobacteria", "__Order_Incertae_Sedis", "__Family_Incertae_Sedis", "__Arenicella"]}},{"id": "denovo1987", "metadata": {"taxonomy": ["Bacteria", "__Proteobacteria", "__Deltaproteobacteria", "__Myxococcales", "__Cystobacterineae", "__g"]}},{"id": "denovo1988", "metadata": {"taxonomy": ["Bacteria", "__Planctomycetes", "__OM190", "__o", "__f", "__g"]}},{"id": "denovo1992", "metadata": {"taxonomy": ["Bacteria", "__Proteobacteria", "__Alphaproteobacteria", "__Parvularculales", "__Parvularculaceae", "__Parvularcula"]}},{"id": "denovo1997", "metadata": {"taxonomy": ["Bacteria", "__Proteobacteria", "__Alphaproteobacteria", "__Rhodobacterales", "__Rhodobacteraceae", "__Roseobacter_clade_AS-21_lineage"]}},{"id": "denovo1998", "metadata": {"taxonomy": ["Bacteria", "__Proteobacteria", "__Gammaproteobacteria", "__Thiotrichales", "__Piscirickettsiaceae", "__g"]}},{"id": "denovo1999", "metadata": {"taxonomy": ["Bacteria", "__Proteobacteria", "__Alphaproteobacteria", "__Rhodospirillales", "__Rhodospirillaceae", "__Defluviicoccus"]}},{"id": "denovo2000", "metadata": {"taxonomy": ["Bacteria", "__Acidobacteria", "__Holophagae", "__Sva0725", "__f", "__g"]}},{"id": "denovo2001", "metadata": {"taxonomy": ["Bacteria", "__Proteobacteria", "__Gammaproteobacteria", "__Alteromonadales", "__Colwelliaceae", "__Thalassomonas"]}},{"id": "denovo2002", "metadata": {"taxonomy": ["Bacteria", "__Proteobacteria", "__Gammaproteobacteria", "__Chromatiales", "__Ectothiorhodospiraceae", "__Acidiferrobacter"]}},

{"id": "denovo2013", "metadata": {"taxonomy": ["Bacteria", "__Cyanobacteria", "__Cyanobacteria", "__SubsectionI", "__FamilyI", "__g"]}},{"id": "denovo2014", "metadata": {"taxonomy": ["Bacteria", "__Proteobacteria", "__Gammaproteobacteria"]}},{"id": "denovo2018", "metadata": {"taxonomy": ["Bacteria", "__Proteobacteria", "__Gammaproteobacteria", "__NKB5", "__f", "__g"]}},{"id": "denovo2020", "metadata": {"taxonomy": ["Bacteria", "__Proteobacteria", "__Gammaproteobacteria", "__Order_Incertae_Sedis", "__Family_Incertae_Sedis", "__Marinicella"]}},{"id": "denovo2021", "metadata": {"taxonomy": ["Bacteria", "__Proteobacteria", "__Deltaproteobacteria", "__Desulfobacterales", "__Desulfobacteraceae", "__Desulfobacula"]}},{"id": "denovo2022", "metadata": {"taxonomy": ["Bacteria", "__Proteobacteria", "__Deltaproteobacteria", "__GR-WP33-30", "__f", "__g"]}},{"id": "denovo2023", "metadata": {"taxonomy": ["Archaea", "__Thaumarchaeota", "__Marine_Group_I", "__Candidatus_Nitrosopumilus", "__f", "__g"]}},{"id": "denovo2025", "metadata": {"taxonomy": ["Bacteria", "__Acidobacteria", "__Acidobacteria", "__PAUC26f", "__f", "__g"]}},{"id": "denovo2027", "metadata": {"taxonomy": ["Bacteria", "__Proteobacteria", "__Alphaproteobacteria", "__Parvularculales", "__Parvularculaceae", "__Parvularcula"]}},{"id": "denovo2031", "metadata": {"taxonomy": ["Bacteria", "__Proteobacteria", "__Deltaproteobacteria", "__SAR324_clade(Marine_group_B)", "__f", "__g"]}},{"id": "denovo2033", "metadata": {"taxonomy": ["Bacteria", "__Proteobacteria", "__Gammaproteobacteria", "__Legionellales", "__Coxiellaceae", "__Coxiella"]}},{"id": "denovo2034", "metadata": {"taxonomy": ["Bacteria", "__Verrucomicrobia", "__Verrucomicrobiae", "__Verrucomicrobiales", "__Rubritaleaceae", "__Rubritalea"]}},{"id": "denovo2036", "metadata": {"taxonomy": ["Bacteria", "__Bacteroidetes", "__Sphingobacteriia", "__Sphingobacteriales", "__Saprospiraceae", "__g"]}},{"id": "denovo2040", "metadata": {"taxonomy": ["Bacteria", "__Proteobacteria", "__Alphaproteobacteria"]}},{"id": "denovo2041", "metadata": {"taxonomy": ["Bacteria", "__Chloroflexi", "__Chloroflexi", "__Chloroflexales"]}},{"id": "denovo2043", "metadata": {"taxonomy": ["Bacteria", "__Gemmatimonadetes", "__Gemmatimonadetes", "__Gemmatimonadales", "__Gemmatimonadaceae", "__g"]}},{"id": "denovo2047", "metadata": {"taxonomy": ["Bacteria", "__Planctomycetes", "__OM190", "__o", "__f", "__g"]}},{"id": "denovo2048", "metadata": {"taxonomy": ["Bacteria", "__Actinobacteria", "__Acidimicrobiia", "__Acidimicrobiales", "__uncultured", "__g"]}},{"id": "denovo2055", "metadata": {"taxonomy": ["Bacteria", "__Proteobacteria", "__Gammaproteobacteria", "__Oceanospirillales", "__Hahellaceae", "__Endozoicomonas"]}},{"id": "denovo2056", "metadata": {"taxonomy": ["Bacteria", "__Proteobacteria", "__TA18", "__o", "__f", "__g"]}},{"id": "denovo2057", "metadata": {"taxonomy": ["Bacteria", "__Bacteroidetes", "__Flavobacteria", "__Flavobacteriales", "__Flavobacteriaceae", "__Sufflavibacter"]}},{"id": "denovo2062", "metadata": {"taxonomy": ["Bacteria", "__Proteobacteria", "__Gammaproteobacteria", "__Alteromonadales", "__Alteromonadaceae"]}},{"id": "denovo2063",

"metadata": {"taxonomy": ["Bacteria", "__Proteobacteria", "__Deltaproteobacteria", "__Desulfuromonadales", "__GR-WP33-58", "__g"]}},{"id": "denovo2069", "metadata": {"taxonomy": ["Bacteria", "__Proteobacteria", "__Gammaproteobacteria", "__Alteromonadales", "__Colwelliaceae", "__Thalassomonas"]}},{"id": "denovo2073", "metadata": {"taxonomy": ["Bacteria", "__Cyanobacteria", "__Cyanobacteria"]}},{"id": "denovo2074", "metadata": {"taxonomy": ["Bacteria", "__Proteobacteria", "__Gammaproteobacteria", "__Pseudomonadales", "__Moraxellaceae", "__Acinetobacter"]}},{"id": "denovo2075", "metadata": {"taxonomy": ["Bacteria", "__Proteobacteria", "__Deltaproteobacteria", "__Desulfobacterales", "__Desulfobulbaceae", "__Desulfopila"]}},{"id": "denovo2076", "metadata": {"taxonomy": ["Bacteria", "__Proteobacteria", "__Deltaproteobacteria", "__Sh765B-TzT-29", "__f", "__g"]}},{"id": "denovo2077", "metadata": {"taxonomy": ["Bacteria", "__Proteobacteria", "__Deltaproteobacteria", "__Desulfobacterales", "__Desulfobacteraceae"]}},{"id": "denovo2078", "metadata": {"taxonomy": ["Bacteria", "__Proteobacteria", "__Deltaproteobacteria", "__Bdellovibrionales", "__Bdellovibrionaceae", "__OM27_clade"]}},{"id": "denovo2082", "metadata": {"taxonomy": ["Bacteria", "__Proteobacteria", "__Alphaproteobacteria", "__Rhodospirillales", "__Rhodospirillaceae", "__g"]}},{"id": "denovo2083", "metadata": {"taxonomy": ["Bacteria", "__Firmicutes", "__Clostridia", "__Clostridiales", "__Clostridiaceae", "__Clostridium"]}},{"id": "denovo2085", "metadata": {"taxonomy": ["Bacteria", "__Spirochaetes", "__Spirochaetes", "__Spirochaetales", "__Spirochaetaceae", "__Spirochaeta"]}},{"id": "denovo2086", "metadata": {"taxonomy": ["Bacteria", "__Proteobacteria", "__Gammaproteobacteria", "__Alteromonadales", "__Alteromonadaceae", "__OM60(NOR5)_clade"]}},{"id": "denovo2088", "metadata": {"taxonomy": ["Bacteria", "__RF3", "__c", "__o", "__f", "__g"]}},{"id": "denovo2094", "metadata": {"taxonomy": ["Bacteria", "__Proteobacteria", "__Alphaproteobacteria", "__Sphingomonadales", "__Erythrobacteraceae"]}},{"id": "denovo2100", "metadata": {"taxonomy": ["Bacteria", "__Firmicutes", "__Clostridia", "__Clostridiales", "__Kazan-2B-17", "__g"]}},{"id": "denovo2106", "metadata": {"taxonomy": ["Bacteria", "__Bacteroidetes", "__Sphingobacteriia", "__Sphingobacteriales", "__Saprospiraceae", "__g"]}},{"id": "denovo2107", "metadata": {"taxonomy": ["Bacteria", "__Bacteroidetes", "__Flavobacteria", "__Flavobacteriales", "__Flavobacteriaceae", "__Flagellimonas"]}},{"id": "denovo2113", "metadata": {"taxonomy": ["Bacteria", "__Proteobacteria", "__Alphaproteobacteria", "__Rhizobiales", "__Phyllobacteriaceae", "__Hoeflea"]}},{"id": "denovo2116", "metadata": {"taxonomy": ["Bacteria", "__Proteobacteria", "__Gammaproteobacteria", "__Alteromonadales", "__Alteromonadaceae", "__OM60(NOR5)_clade"]}},{"id": "denovo2121", "metadata": {"taxonomy": ["Bacteria", "__Deferribacteres", "__Deferribacteres", "__Deferribacterales", "__SAR406_clade(Marine_group_A)", "__g"]}},{"id": "denovo2122", "metadata": {"taxonomy": ["Bacteria", "__Chlamydiae", "__Chlamydiae", "__Chlamydiales", "__Chlamydiaceae", "__g"]}},{"id": "denovo2130",

"metadata": {"taxonomy": ["Bacteria", "__Actinobacteria",
"__Acidimicrobiia", "__Acidimicrobiales", "__Acidimicrobiaceae",
"__g"]}},{"id": "denovo2132", "metadata": {"taxonomy": ["Bacteria",
"__Acidobacteria", "__Holophagae", "__NKB17", "__f", "__g"]}},{"id":
"denovo2137", "metadata": {"taxonomy": ["Bacteria", "__Bacteroidetes",
"__Flavobacteria", "__Flavobacteriales", "__Cryomorphaceae",
"__Owenweeksia"]}},{"id": "denovo2139", "metadata": {"taxonomy":
["Bacteria", "__Proteobacteria", "__Gammaproteobacteria",
"__Oceanospirillales", "__OM182_clade", "__g"]}},{"id": "denovo2140",
"metadata": {"taxonomy": ["Bacteria", "__Deferribacteres",
"__Deferribacteres", "__Deferribacterales", "__Family_Incertae_Sedis",
"__Caldithrix"]}},{"id": "denovo2143", "metadata": {"taxonomy":
["Bacteria", "__Proteobacteria", "__Gammaproteobacteria",
"__Alteromonadales", "__Alteromonadaceae", "__OM60(NOR5)_clade"]}},
{"id": "denovo2144", "metadata": {"taxonomy": ["Bacteria",
"__Proteobacteria", "__Alphaproteobacteria", "__Rhodospirillales",
"__Rhodospirillaceae", "__g"]}},{"id": "denovo2146", "metadata":
{"taxonomy": ["Bacteria", "__Proteobacteria", "__Gammaproteobacteria",
"__Alteromonadales", "__Alteromonadaceae"]}},{"id": "denovo2150",
"metadata": {"taxonomy": ["Bacteria", "__Actinobacteria",
"__Actinobacteria", "__Micrococcales", "__Micrococcaceae",
"__Rothia"]}},{"id": "denovo2151", "metadata": {"taxonomy":
["Bacteria", "__Proteobacteria", "__Alphaproteobacteria",
"__Rhodobacterales", "__Rhodobacteraceae"]}},{"id": "denovo2152",
"metadata": {"taxonomy": ["Bacteria", "__Bacteroidetes", "__BD2-2",
"__o", "__f", "__g"]}},{"id": "denovo2154", "metadata": {"taxonomy":
["Bacteria", "__Proteobacteria", "__Alphaproteobacteria",
"__Sphingomonadales"]}},{"id": "denovo2157", "metadata": {"taxonomy":
["Bacteria", "__Bacteroidetes", "__Sphingobacteriia",
"__Sphingobacteriales", "__Saprospiraceae", "__g"]}},{"id":
"denovo2161", "metadata": {"taxonomy": ["Bacteria", "__Chloroflexi",
"__SAR202_clade", "__o", "__f", "__g"]}},{"id": "denovo2166",
"metadata": {"taxonomy": ["Bacteria", "__Proteobacteria",
"__Deltaproteobacteria", "__Sh765B-TzT-29", "__f", "__g"]}},{"id":
"denovo2171", "metadata": {"taxonomy": ["Bacteria",
"__Proteobacteria", "__Alphaproteobacteria", "__Caulobacterales",
"__Hyphomonadaceae", "__Hyphomonas"]}},{"id": "denovo2175",
"metadata": {"taxonomy": ["Bacteria", "__Proteobacteria",
"__Alphaproteobacteria", "__Rhodobacterales", "__Rhodobacteraceae"]}},
{"id": "denovo2176", "metadata": {"taxonomy": ["Bacteria",
"__Bacteroidetes", "__Cytophagia", "__Cytophagales",
"__Cyclobacteriaceae", "__Rhodonellum"]}},{"id": "denovo2179",
"metadata": {"taxonomy": ["Bacteria", "__Proteobacteria",
"__Gammaproteobacteria"]}},{"id": "denovo2180", "metadata":
{"taxonomy": ["Bacteria", "__Proteobacteria", "__Gammaproteobacteria",
"__Alteromonadales", "__Colwelliaceae", "__Thalassomonas"]}},{"id":
"denovo2183", "metadata": {"taxonomy": ["Bacteria",
"__Proteobacteria", "__Gammaproteobacteria", "__Alteromonadales",
"__Pseudoalteromonadaceae", "__Pseudoalteromonas"]}},{"id":
"denovo2185", "metadata": {"taxonomy": ["Bacteria",

"__Proteobacteria", "__Gammaproteobacteria", "__Oceanospirillales", "__Oceanospirillaceae", "__Neptuniibacter_"]}},{"id": "denovo2187", "metadata": {"taxonomy": ["Bacteria", "__Proteobacteria", "__Alphaproteobacteria", "__Sphingomonadales", "__GOBB3-C201", "__g"]}},{"id": "denovo2191", "metadata": {"taxonomy": ["Bacteria", "__Proteobacteria", "__AEGEAN-245", "__o", "__f", "__g"]}},{"id": "denovo2194", "metadata": {"taxonomy": ["Bacteria", "__Proteobacteria", "__Gammaproteobacteria", "__Oceanospirillales", "__Litoricolaceae", "__Litoricola"]}},{"id": "denovo2199", "metadata": {"taxonomy": ["Bacteria", "__Bacteroidetes", "__Flavobacteria", "__Flavobacteriales", "__Cryomorphaceae", "__Owenweeksia"]}},{"id": "denovo2201", "metadata": {"taxonomy": ["Bacteria", "__Proteobacteria", "__Alphaproteobacteria", "__Rhodospirillales", "__Rhodospirillaceae"]}},{"id": "denovo2210", "metadata": {"taxonomy": ["Bacteria", "__Proteobacteria", "__Deltaproteobacteria", "__Sh765B-TzT-29", "__f", "__g"]}},{"id": "denovo2216", "metadata": {"taxonomy": ["Bacteria", "__Actinobacteria", "__Acidimicrobiia", "__Acidimicrobiales", "__OCS155_marine_group", "__g"]}},{"id": "denovo2217", "metadata": {"taxonomy": ["Bacteria", "__Proteobacteria", "__Gammaproteobacteria", "__Xanthomonadales", "__Sinobacteraceae", "__g"]}},{"id": "denovo2219", "metadata": {"taxonomy": ["Bacteria"]}},{"id": "denovo2220", "metadata": {"taxonomy": ["Bacteria", "__Proteobacteria", "__Gammaproteobacteria", "__Oceanospirillales", "__Hahellaceae", "__Endozoicomonas"]}},{"id": "denovo2221", "metadata": {"taxonomy": ["Bacteria", "__Bacteroidetes", "__Sphingobacteriia", "__Sphingobacteriales", "__Saprospiraceae", "__g"]}},{"id": "denovo2222", "metadata": {"taxonomy": ["Bacteria", "__Proteobacteria", "__Gammaproteobacteria", "__Alteromonadales", "__Alteromonadaceae", "__Candidatus_Endobugula"]}},{"id": "denovo2225", "metadata": {"taxonomy": ["Bacteria", "__Proteobacteria", "__Deltaproteobacteria", "__Bdellovibrionales", "__Bdellovibrionaceae", "__Bdellovibrio"]}},{"id": "denovo2226", "metadata": {"taxonomy": ["Archaea", "__Euryarchaeota", "__Thermoplasmata", "__Thermoplasmatales", "__Marine_Group_II", "__g"]}},{"id": "denovo2228", "metadata": {"taxonomy": ["Bacteria", "__Actinobacteria", "__Thermoleophilia", "__Gaiellales", "__uncultured", "__g"]}},{"id": "denovo2229", "metadata": {"taxonomy": ["Bacteria", "__Planctomycetes", "__Phycisphaerae", "__Phycisphaerales", "__Phycisphaeraceae", "__Phycisphaera"]}},{"id": "denovo2231", "metadata": {"taxonomy": ["Bacteria", "__Proteobacteria", "__Deltaproteobacteria", "__Bdellovibrionales", "__Bacteriovoracaceae", "__Peredibacter"]}},{"id": "denovo2234", "metadata": {"taxonomy": ["Bacteria", "__Proteobacteria", "__Alphaproteobacteria", "__Rhizobiales", "__Brucellaceae", "__g"]}},{"id": "denovo2235", "metadata": {"taxonomy": ["Bacteria", "__Proteobacteria", "__Alphaproteobacteria", "__Rhodobacterales", "__Rhodobacteraceae"]}},{"id": "denovo2236", "metadata": {"taxonomy": ["Bacteria", "__Candidate_division_OD1", "__c", "__o", "__f", "__g"]}},{"id": "denovo2243", "metadata": {"taxonomy": ["Bacteria", "__Proteobacteria", "__Alphaproteobacteria", "__DB1-14", "__f",

"__g"]}},{"id": "denovo2248", "metadata": {"taxonomy": ["Bacteria", "__Deferribacteres", "__Deferribacteres", "__Deferribacterales", "__PAUC34f", "__g"]}},{"id": "denovo2252", "metadata": {"taxonomy": ["Bacteria", "__Proteobacteria", "__Alphaproteobacteria", "__Rhodobacterales", "__Rhodobacteraceae"]}},{"id": "denovo2254", "metadata": {"taxonomy": ["Bacteria", "__Proteobacteria", "__Alphaproteobacteria", "__Sphingomonadales"]}},{"id": "denovo2258", "metadata": {"taxonomy": ["Bacteria", "__Proteobacteria", "__Alphaproteobacteria", "__Sneathiellales", "__Sneathiellaceae", "__Sneathiella"]}},{"id": "denovo2263", "metadata": {"taxonomy": ["Bacteria", "__Cyanobacteria", "__Cyanobacteria", "__SubsectionI", "__FamilyI", "__Acaryochloris"]}},{"id": "denovo2264", "metadata": {"taxonomy": ["Bacteria", "__Firmicutes", "__Clostridia", "__Clostridiales", "__Lachnospiraceae"]}},{"id": "denovo2267", "metadata": {"taxonomy": ["Bacteria", "__Proteobacteria", "__Alphaproteobacteria", "__Rhodospirillales", "__Rhodospirillaceae", "__Pelagibius"]}},{"id": "denovo2269", "metadata": {"taxonomy": ["Bacteria", "__Proteobacteria", "__Deltaproteobacteria", "__Bdellovibrionales", "__Bacteriovoracaceae", "__Bacteriovorax"]}},{"id": "denovo2276", "metadata": {"taxonomy": ["Bacteria", "__Proteobacteria", "__Gammaproteobacteria", "__Thiotrichales", "__Thiotrichaceae", "__g"]}},{"id": "denovo2281", "metadata": {"taxonomy": ["Bacteria", "__Acidobacteria", "__Holophagae", "__CA002", "__f", "__g"]}},{"id": "denovo2283", "metadata": {"taxonomy": ["Bacteria", "__Gemmatimonadetes", "__Gemmatimonadetes", "__BD2-11_terrestrial_group", "__f", "__g"]}},{"id": "denovo2284", "metadata": {"taxonomy": ["Bacteria", "__Acidobacteria", "__Holophagae", "__NS72", "__f", "__g"]}},{"id": "denovo2286", "metadata": {"taxonomy": ["Bacteria", "__Proteobacteria", "__Gammaproteobacteria", "__Oceanospirillales"]}},{"id": "denovo2288", "metadata": {"taxonomy": ["Bacteria", "__Proteobacteria", "__Alphaproteobacteria", "__Rhodobacterales", "__Rhodobacteraceae"]}},{"id": "denovo2290", "metadata": {"taxonomy": ["Bacteria", "__Acidobacteria", "__Holophagae", "__CA002", "__f", "__g"]}},{"id": "denovo2295", "metadata": {"taxonomy": ["Bacteria", "__Proteobacteria", "__Alphaproteobacteria", "__Rhodospirillales", "__Rhodospirillaceae", "__Pelagibius"]}},{"id": "denovo2298", "metadata": {"taxonomy": ["Bacteria", "__Proteobacteria", "__Deltaproteobacteria", "__Desulfobacterales", "__Desulfobacteraceae"]}},{"id": "denovo2302", "metadata": {"taxonomy": ["Bacteria", "__Proteobacteria", "__Alphaproteobacteria", "__OCS116_clade", "__f", "__g"]}},{"id": "denovo2306", "metadata": {"taxonomy": ["Archaea", "__Euryarchaeota", "__Thermoplasmata", "__Thermoplasmatales", "__Marine_Group_II", "__g"]}},{"id": "denovo2313", "metadata": {"taxonomy": ["Bacteria", "__Proteobacteria", "__Alphaproteobacteria", "__Rhodobacterales", "__Rhodobacteraceae"]}},{"id": "denovo2314", "metadata": {"taxonomy": ["Bacteria", "__Proteobacteria", "__Deltaproteobacteria", "__Sh765B-TzT-29", "__f", "__g"]}},{"id": "denovo2320", "metadata": {"taxonomy": ["Bacteria", "__Bacteroidetes", "__BD2-2", "__o", "__f", "__g"]}},

{"id": "denovo2322", "metadata": {"taxonomy": ["Bacteria", "__Bacteroidetes", "__Flavobacteria", "__Flavobacteriales", "__Flavobacteriaceae", "__Kordia"]}},{"id": "denovo2332", "metadata": {"taxonomy": ["Bacteria", "__Firmicutes", "__Clostridia", "__Clostridiales", "__Family_XII_Incertae_Sedis", "__Fusibacter"]}}, {"id": "denovo2342", "metadata": {"taxonomy": ["Bacteria", "__Proteobacteria", "__Deltaproteobacteria", "__Myxococcales", "__0319-6G20", "__g"]}},{"id": "denovo2347", "metadata": {"taxonomy": ["Bacteria"]}},{"id": "denovo2351", "metadata": {"taxonomy": ["Bacteria", "__Proteobacteria", "__Deltaproteobacteria", "__Sh765B-TzT-29", "__f", "__g"]}},{"id": "denovo2352", "metadata": {"taxonomy": ["Bacteria", "__Proteobacteria", "__Alphaproteobacteria", "__Rhodospirillales", "__Rhodospirillaceae", "__Defluviicoccus"]}}, {"id": "denovo2353", "metadata": {"taxonomy": ["Bacteria", "__Proteobacteria", "__Deltaproteobacteria", "__Myxococcales", "__Elev-16S-1158", "__g"]}},{"id": "denovo2358", "metadata": {"taxonomy": ["Bacteria", "__Chloroflexi", "__Caldilineae", "__Caldilineales", "__Caldilineaceae", "__Caldilinea"]}},{"id": "denovo2360", "metadata": {"taxonomy": ["Bacteria", "__Cyanobacteria", "__Cyanobacteria", "__SubsectionIII", "__FamilyI", "__Phormidium"]}}, {"id": "denovo2361", "metadata": {"taxonomy": ["Bacteria", "__Bacteroidetes", "__Flavobacteria", "__Flavobacteriales", "__NS9_marine_group", "__g"]}},{"id": "denovo2362", "metadata": {"taxonomy": ["Bacteria", "__Proteobacteria", "__Gammaproteobacteria", "__BD7-8_marine_group", "__f", "__g"]}},{"id": "denovo2363", "metadata": {"taxonomy": ["Bacteria", "__Proteobacteria", "__Alphaproteobacteria", "__Sphingomonadales", "__Erythrobacteraceae", "__Erythrobacter"]}},{"id": "denovo2365", "metadata": {"taxonomy": ["Bacteria", "__Bacteroidetes", "__Sphingobacteriia", "__Sphingobacteriales", "__Saprospiraceae", "__g"]}},{"id": "denovo2368", "metadata": {"taxonomy": ["Bacteria", "__Proteobacteria", "__Alphaproteobacteria", "__Rhodobacterales", "__Rhodobacteraceae", "__Labrenzia"]}},{"id": "denovo2369", "metadata": {"taxonomy": ["Bacteria", "__Proteobacteria", "__Alphaproteobacteria", "__Rhodobacterales", "__Rhodobacteraceae"]}}, {"id": "denovo2370", "metadata": {"taxonomy": ["Bacteria", "__Bacteroidetes", "__Cytophagia", "__Cytophagales", "__Flammeovirgaceae", "__g"]}},{"id": "denovo2374", "metadata": {"taxonomy": ["Bacteria", "__Proteobacteria", "__Gammaproteobacteria", "__Oceanospirillales", "__Hahellaceae", "__Endozoicomonas"]}},{"id": "denovo2377", "metadata": {"taxonomy": ["Bacteria", "__Acidobacteria", "__Acidobacteria", "__BPC015", "__f", "__g"]}},{"id": "denovo2379", "metadata": {"taxonomy": ["Bacteria", "__Proteobacteria", "__Alphaproteobacteria", "__Rhizobiales"]}},{"id": "denovo2381", "metadata": {"taxonomy": ["Bacteria", "__Cyanobacteria", "__Cyanobacteria", "__SubsectionIII", "__FamilyI", "__Halomicronema"]}},{"id": "denovo2382", "metadata": {"taxonomy": ["Bacteria", "__Proteobacteria", "__Betaproteobacteria", "__Burkholderiales", "__Burkholderiaceae", "__Limnobacter"]}},{"id": "denovo2383", "metadata": {"taxonomy": ["Bacteria",

"__Proteobacteria", "__Gammaproteobacteria",
"__E01-9C-26_marine_group", "__f", "__g"]}},{"id": "denovo2384",
"metadata": {"taxonomy": ["Bacteria", "__Verrucomicrobia",
"__Verrucomicrobiae", "__Verrucomicrobiales", "__DEV007", "__g"]}},
{"id": "denovo2385", "metadata": {"taxonomy": ["Bacteria",
"__Proteobacteria", "__Gammaproteobacteria", "__Alteromonadales",
"__Alteromonadaceae", "__Haliea"]}},{"id": "denovo2386", "metadata":
{"taxonomy": ["Bacteria", "__Lentisphaerae", "__Lentisphaeria"]}},
{"id": "denovo2388", "metadata": {"taxonomy": ["Bacteria",
"__Proteobacteria", "__Deltaproteobacteria", "__Sh765B-TzT-29", "__f",
"__g"]}},{"id": "denovo2389", "metadata": {"taxonomy": ["Bacteria",
"__Proteobacteria", "__Alphaproteobacteria", "__DB1-14", "__f",
"__g"]}},{"id": "denovo2390", "metadata": {"taxonomy": ["Bacteria",
"__Proteobacteria", "__Alphaproteobacteria", "__Rhodobacterales",
"__Rhodobacteraceae", "__Ruegeria"]}},{"id": "denovo2392", "metadata":
{"taxonomy": ["Bacteria", "__Proteobacteria", "__Gammaproteobacteria",
"__Vibrionales", "__Vibrionaceae", "__Photobacterium"]}},{"id":
"denovo2401", "metadata": {"taxonomy": ["Bacteria",
"__Proteobacteria", "__Deltaproteobacteria", "__Bdellovibrionales",
"__Bdellovibrionaceae", "__Bdellovibrio"]}},{"id": "denovo2409",
"metadata": {"taxonomy": ["Bacteria", "__Proteobacteria",
"__Gammaproteobacteria", "__E01-9C-26_marine_group", "__f", "__g"]}},
{"id": "denovo2413", "metadata": {"taxonomy": ["Bacteria",
"__Proteobacteria", "__Gammaproteobacteria", "__Alteromonadales",
"__Alteromonadaceae", "__Teredinibacter"]}},{"id": "denovo2421",
"metadata": {"taxonomy": ["Bacteria", "__Chloroflexi",
"__Caldilineae", "__Caldilineales", "__Caldilineaceae",
"__Caldilinea"]}},{"id": "denovo2423", "metadata": {"taxonomy":
["Bacteria", "__Proteobacteria", "__Gammaproteobacteria",
"__Pseudomonadales", "__Moraxellaceae", "__Acinetobacter"]}},{"id":
"denovo2429", "metadata": {"taxonomy": ["Bacteria",
"__Planctomycetes"]}},{"id": "denovo2430", "metadata": {"taxonomy":
["Bacteria", "__Candidate_division_WS3", "__c", "__o", "__f",
"__g"]}},{"id": "denovo2436", "metadata": {"taxonomy": ["Bacteria",
"__Proteobacteria", "__Alphaproteobacteria", "__Rickettsiales",
"__TK34", "__g"]}},{"id": "denovo2441", "metadata": {"taxonomy":
["Bacteria", "__Cyanobacteria", "__Cyanobacteria", "__SubsectionII",
"__FamilyII", "__Pleurocapsa"]}},{"id": "denovo2444", "metadata":
{"taxonomy": ["Bacteria", "__Proteobacteria", "__TA18", "__o", "__f",
"__g"]}},{"id": "denovo2445", "metadata": {"taxonomy": ["Bacteria",
"__Bacteroidetes", "__Flavobacteria", "__Flavobacteriales",
"__Flavobacteriaceae", "__Tenacibaculum"]}},{"id": "denovo2447",
"metadata": {"taxonomy": ["Bacteria", "__Proteobacteria",
"__Gammaproteobacteria", "__Order_Incertae_Sedis",
"__Family_Incertae_Sedis", "__Arenicella"]}},{"id": "denovo2451",
"metadata": {"taxonomy": ["Bacteria", "__Verrucomicrobia",
"__OPB35_soil_group", "__o", "__f", "__g"]}},{"id": "denovo2455",
"metadata": {"taxonomy": ["Bacteria", "__Proteobacteria",
"__Deltaproteobacteria", "__Desulfovibrionales",
"__Desulfovibrionaceae", "__Desulfovibrio"]}},{"id": "denovo2459",

"metadata": {"taxonomy": ["Bacteria", "__Bacteroidetes", "__Sphingobacteriia", "__Sphingobacteriales", "__Saprospiraceae", "__g"]}},{"id": "denovo2461", "metadata": {"taxonomy": ["Bacteria", "__Proteobacteria", "__Deltaproteobacteria", "__Desulfobacterales", "__Desulfobacteraceae", "__Desulfobacter"]}},{"id": "denovo2466", "metadata": {"taxonomy": ["Bacteria", "__Planctomycetes", "__Pla3_lineage", "__o", "__f", "__g"]}},{"id": "denovo2469", "metadata": {"taxonomy": ["Bacteria", "__Proteobacteria", "__Deltaproteobacteria", "__SAR324_clade(Marine_group_B)", "__f", "__g"]}},{"id": "denovo2472", "metadata": {"taxonomy": ["Bacteria", "__Proteobacteria", "__Alphaproteobacteria", "__Rhodospirillales", "__Rhodospirillaceae", "__g"]}},{"id": "denovo2474", "metadata": {"taxonomy": ["Bacteria", "__Proteobacteria", "__Gammaproteobacteria", "__Alteromonadales", "__Pseudoalteromonadaceae", "__Algicola"]}}, {"id": "denovo2475", "metadata": {"taxonomy": ["Bacteria", "__Proteobacteria", "__Alphaproteobacteria", "__Rhodobacterales", "__Rhodobacteraceae", "__g"]}},{"id": "denovo2477", "metadata": {"taxonomy": ["Bacteria", "__Actinobacteria", "__Acidimicrobiia", "__Acidimicrobiales", "__OCS155_marine_group", "__g"]}},{"id": "denovo2478", "metadata": {"taxonomy": ["Bacteria", "__Proteobacteria", "__Gammaproteobacteria", "__Order_Incertae_Sedis", "__Family_Incertae_Sedis", "__Marinicella"]}},{"id": "denovo2479", "metadata": {"taxonomy": ["Bacteria", "__Proteobacteria", "__Gammaproteobacteria", "__Alteromonadales", "__Alteromonadaceae", "__Teredinibacter"]}},{"id": "denovo2481", "metadata": {"taxonomy": ["Bacteria", "__Proteobacteria", "__SPOTSOCT00m83", "__o", "__f", "__g"]}},{"id": "denovo2482", "metadata": {"taxonomy": ["Bacteria", "__Proteobacteria", "__Deltaproteobacteria", "__Desulfobacterales", "__Desulfobulbaceae", "__g"]}},{"id": "denovo2485", "metadata": {"taxonomy": ["Bacteria", "__Firmicutes", "__Clostridia", "__Halanaerobiales", "__64K2", "__g"]}},{"id": "denovo2487", "metadata": {"taxonomy": ["Bacteria", "__Proteobacteria", "__Deltaproteobacteria", "__SAR324_clade(Marine_group_B)", "__f", "__g"]}},{"id": "denovo2488", "metadata": {"taxonomy": ["Bacteria", "__Firmicutes", "__Clostridia", "__Clostridiales", "__Clostridiaceae", "__Clostridium"]}},{"id": "denovo2491", "metadata": {"taxonomy": ["Bacteria", "__Proteobacteria", "__Alphaproteobacteria", "__E6aD10", "__f", "__g"]}},{"id": "denovo2495", "metadata": {"taxonomy": ["Bacteria", "__Proteobacteria", "__Gammaproteobacteria", "__Alteromonadales", "__Alteromonadaceae", "__Haliea"]}},{"id": "denovo2496", "metadata": {"taxonomy": ["Bacteria", "__Proteobacteria", "__Deltaproteobacteria", "__Desulfobacterales", "__Desulfobulbaceae", "__Desulfobulbus"]}},{"id": "denovo2500", "metadata": {"taxonomy": ["Bacteria", "__Proteobacteria", "__Alphaproteobacteria", "__Rhodobacterales", "__Rhodobacteraceae", "__Pseudovibrio"]}},{"id": "denovo2501", "metadata": {"taxonomy": ["Bacteria", "__Proteobacteria", "__Alphaproteobacteria", "__Rhodobacterales", "__Rhodobacteraceae", "__Loktanella"]}},{"id": "denovo2502", "metadata": {"taxonomy": ["Bacteria", "__Proteobacteria", "__Gammaproteobacteria", "__Alteromonadales",

"__Colwelliaceae", "__Thalassomonas"]}},{"id": "denovo2504", "metadata": {"taxonomy": ["Bacteria", "__Proteobacteria", "__Alphaproteobacteria", "__Rickettsiales", "__SAR116_clade", "__g"]}},{"id": "denovo2505", "metadata": {"taxonomy": ["Bacteria", "__Proteobacteria", "__Betaproteobacteria", "__Methylophilales", "__Methylophilaceae", "__Methylophilus"]}},{"id": "denovo2506", "metadata": {"taxonomy": ["Bacteria", "__Proteobacteria", "__Deltaproteobacteria"]}},{"id": "denovo2507", "metadata": {"taxonomy": ["Bacteria", "__Proteobacteria", "__Alphaproteobacteria", "__Rickettsiales", "__SAR116_clade", "__g"]}},{"id": "denovo2510", "metadata": {"taxonomy": ["Bacteria", "__Proteobacteria", "__Alphaproteobacteria", "__Rhodobacterales", "__Rhodobacteraceae", "__Rubribacterium"]}},{"id": "denovo2512", "metadata": {"taxonomy": ["Bacteria", "__Proteobacteria", "__Betaproteobacteria", "__Methylophilales", "__Methylophilaceae"]}},{"id": "denovo2513", "metadata": {"taxonomy": ["Bacteria", "__Proteobacteria", "__Gammaproteobacteria", "__Chromatiales", "__Chromatiaceae", "__Nitrosococcus"]}},{"id": "denovo2515", "metadata": {"taxonomy": ["Bacteria", "__Acidobacteria", "__Holophagae", "__CA002", "__f", "__g"]}},{"id": "denovo2516", "metadata": {"taxonomy": ["Bacteria", "__Proteobacteria", "__Alphaproteobacteria"]}},{"id": "denovo2518", "metadata": {"taxonomy": ["Bacteria", "__Proteobacteria", "__Alphaproteobacteria", "__Rhodobacterales", "__Rhodobacteraceae"]}}, {"id": "denovo2519", "metadata": {"taxonomy": ["Bacteria", "__Proteobacteria", "__Deltaproteobacteria", "__Myxococcales"]}}, {"id": "denovo2524", "metadata": {"taxonomy": ["Bacteria", "__Proteobacteria", "__Alphaproteobacteria", "__DB1-14", "__f", "__g"]}},{"id": "denovo2525", "metadata": {"taxonomy": ["Bacteria", "__Proteobacteria", "__Deltaproteobacteria", "__Desulfobacterales", "__Desulfobacteraceae", "__Desulfofaba"]}},{"id": "denovo2529", "metadata": {"taxonomy": ["Bacteria", "__Proteobacteria", "__Deltaproteobacteria", "__Desulfuromonadales", "__GR-WP33-58", "__g"]}},{"id": "denovo2532", "metadata": {"taxonomy": ["Bacteria", "__Chloroflexi", "__Caldilineae", "__Caldilineales", "__Caldilineaceae", "__Caldilinea"]}},{"id": "denovo2539", "metadata": {"taxonomy": ["Archaea", "__Euryarchaeota", "__Thermoplasmata", "__Thermoplasmatales", "__Marine_Group_II", "__g"]}},{"id": "denovo2540", "metadata": {"taxonomy": ["Bacteria", "__Acidobacteria", "__RB25", "__o", "__f", "__g"]}},{"id": "denovo2543", "metadata": {"taxonomy": ["Bacteria", "__Candidate_division_OD1", "__c", "__o", "__f", "__g"]}},{"id": "denovo2544", "metadata": {"taxonomy": ["Bacteria", "__Proteobacteria", "__Gammaproteobacteria", "__Oceanospirillales", "__Oceanospirillaceae", "__Marinobacterium"]}}, {"id": "denovo2547", "metadata": {"taxonomy": ["Bacteria", "__Proteobacteria", "__Alphaproteobacteria", "__Rhodobacterales", "__Rhodobacteraceae", "__Pseudovibrio"]}},{"id": "denovo2553", "metadata": {"taxonomy": ["Bacteria", "__Proteobacteria", "__Alphaproteobacteria", "__Kordiimonadales", "__Kordiimonadaceae", "__Kordiimonas"]}},{"id": "denovo2554", "metadata": {"taxonomy": ["Bacteria", "__Proteobacteria", "__Gammaproteobacteria",

"__Pseudomonadales", "__Moraxellaceae", "__Acinetobacter"]}},{"id": "denovo2561", "metadata": {"taxonomy": ["Bacteria", "__Bacteroidetes", "__Flavobacteria", "__Flavobacteriales", "__Cryomorphaceae", "__Owenweeksia"]}},{"id": "denovo2571", "metadata": {"taxonomy": ["Bacteria", "__Bacteroidetes", "__Sphingobacteriia", "__Sphingobacteriales", "__Saprospiraceae", "__Aureispira"]}},{"id": "denovo2573", "metadata": {"taxonomy": ["Bacteria", "__Acidobacteria", "__Holophagae", "__CA002", "__f", "__g"]}},{"id": "denovo2578", "metadata": {"taxonomy": ["Bacteria", "__Proteobacteria", "__Alphaproteobacteria", "__Rhodobacterales", "__Rhodobacteraceae", "__g"]}},{"id": "denovo2585", "metadata": {"taxonomy": ["Bacteria", "__Proteobacteria", "__Gammaproteobacteria", "__Order_Incertae_Sedis", "__Family_Incertae_Sedis", "__Arenicella"]}},{"id": "denovo2587", "metadata": {"taxonomy": ["Bacteria", "__Proteobacteria", "__Alphaproteobacteria", "__Sphingomonadales", "__Erythrobacteraceae", "__Erythrobacter"]}},{"id": "denovo2588", "metadata": {"taxonomy": ["Bacteria", "__Proteobacteria", "__Alphaproteobacteria"]}},{"id": "denovo2592", "metadata": {"taxonomy": ["Bacteria", "__Proteobacteria", "__Alphaproteobacteria", "__Rhodobacterales", "__Rhodobacteraceae", "__Roseobacter_clade_CHAB-I-5_lineage"]}},{"id": "denovo2595", "metadata": {"taxonomy": ["Bacteria", "__Proteobacteria", "__Alphaproteobacteria", "__Rhodobacterales", "__Rhodobacteraceae", "__g"]}},{"id": "denovo2599", "metadata": {"taxonomy": ["Bacteria", "__Proteobacteria", "__Gammaproteobacteria", "__Xanthomonadales", "__Sinobacteraceae", "__JTB255_marine_benthic_group"]}},{"id": "denovo2602", "metadata": {"taxonomy": ["Bacteria", "__Proteobacteria", "__Alphaproteobacteria", "__Kordiimonadales", "__Kordiimonadaceae", "__Kordiimonas"]}},{"id": "denovo2603", "metadata": {"taxonomy": ["Bacteria", "__Proteobacteria", "__Gammaproteobacteria", "__KI89A_clade", "__f", "__g"]}},{"id": "denovo2605", "metadata": {"taxonomy": ["Bacteria", "__Firmicutes", "__Clostridia", "__Clostridiales", "__Lachnospiraceae"]}},{"id": "denovo2606", "metadata": {"taxonomy": ["Bacteria", "__Firmicutes", "__Bacilli", "__Bacillales"]}},{"id": "denovo2610", "metadata": {"taxonomy": ["Bacteria", "__Proteobacteria", "__Gammaproteobacteria"]}},{"id": "denovo2611", "metadata": {"taxonomy": ["Bacteria", "__Proteobacteria", "__Deltaproteobacteria", "__Myxococcales", "__Sorangiineae", "__Sandaracinaceae"]}},{"id": "denovo2612", "metadata": {"taxonomy": ["Bacteria", "__Bacteroidetes", "__Sphingobacteriia", "__Sphingobacteriales", "__Saprospiraceae", "__g"]}},{"id": "denovo2613", "metadata": {"taxonomy": ["Bacteria", "__Proteobacteria", "__Gammaproteobacteria", "__Oceanospirillales", "__Alcanivoracaceae", "__Kangiella"]}},{"id": "denovo2618", "metadata": {"taxonomy": ["Bacteria", "__Cyanobacteria", "__Cyanobacteria", "__SubsectionIII", "__FamilyI", "__Planktothricoides"]}},{"id": "denovo2619", "metadata": {"taxonomy": ["Bacteria", "__Bacteroidetes", "__Flavobacteria", "__Flavobacteriales", "__Flavobacteriaceae"]}},{"id": "denovo2626", "metadata": {"taxonomy": ["Bacteria", "__Proteobacteria",

"__Epsilonproteobacteria", "__Campylobacterales",
"__Helicobacteraceae", "__Sulfurovum"]}},{"id": "denovo2629",
"metadata": {"taxonomy": ["Bacteria", "__Proteobacteria",
"__Gammaproteobacteria", "__Oceanospirillales", "__Hahellaceae",
"__Endozoicomonas"]}},{"id": "denovo2632", "metadata": {"taxonomy":
["Bacteria", "__Cyanobacteria", "__Cyanobacteria", "__SubsectionII",
"__FamilyII", "__Pleurocapsa"]}},{"id": "denovo2633", "metadata":
{"taxonomy": ["Bacteria", "__Proteobacteria", "__Alphaproteobacteria",
"__Sphingomonadales", "__Erythrobacteraceae", "__Erythrobacter"]}},
{"id": "denovo2634", "metadata": {"taxonomy": ["Bacteria",
"__Proteobacteria", "__Gammaproteobacteria", "__Alteromonadales",
"__Alteromonadaceae", "__Haliea"]}},{"id": "denovo2636", "metadata":
{"taxonomy": ["Bacteria", "__Proteobacteria", "__Alphaproteobacteria",
"__Rhodobacterales", "__Rhodobacteraceae", "__g"]}},{"id":
"denovo2637", "metadata": {"taxonomy": ["Bacteria",
"__Proteobacteria", "__Alphaproteobacteria", "__Rhodobacterales",
"__Rhodobacteraceae", "__Maritimibacter"]}},{"id": "denovo2638",
"metadata": {"taxonomy": ["Bacteria", "__Proteobacteria",
"__Alphaproteobacteria", "__OCS116_clade", "__f", "__g"]}},{"id":
"denovo2643", "metadata": {"taxonomy": ["Bacteria",
"__Verrucomicrobia", "__Verrucomicrobiae", "__Verrucomicrobiales",
"__Rubritaleaceae", "__Rubritalea"]}},{"id": "denovo2646", "metadata":
{"taxonomy": ["Bacteria", "__Proteobacteria", "__Gammaproteobacteria",
"__Alteromonadales", "__Alteromonadaceae", "__OM60(NOR5)_clade"]}},
{"id": "denovo2649", "metadata": {"taxonomy": ["Bacteria",
"__Bacteroidetes", "__Flavobacteria", "__Flavobacteriales",
"__Flavobacteriaceae", "__Chryseobacterium"]}},{"id": "denovo2656",
"metadata": {"taxonomy": ["Bacteria", "__Proteobacteria",
"__Deltaproteobacteria", "__GR-WP33-30", "__f", "__g"]}},{"id":
"denovo2657", "metadata": {"taxonomy": ["Bacteria",
"__Proteobacteria", "__Deltaproteobacteria", "__Myxococcales",
"__Haliangiaceae", "__Haliangium"]}},{"id": "denovo2660", "metadata":
{"taxonomy": ["Bacteria", "__Proteobacteria", "__Gammaproteobacteria",
"__Alteromonadales", "__Alteromonadaceae"]}},{"id": "denovo2662",
"metadata": {"taxonomy": ["Bacteria", "__Fibrobacteres",
"__Fibrobacteria", "__Fibrobacterales", "__B122", "__g"]}},{"id":
"denovo2668", "metadata": {"taxonomy": ["Bacteria", "__Bacteroidetes",
"__Flavobacteria", "__Flavobacteriales", "__Flavobacteriaceae",
"__Aquimarina"]}},{"id": "denovo2669", "metadata": {"taxonomy":
["Bacteria", "__Nitrospirae", "__Nitrospira", "__Nitrospirales",
"__Nitrospiraceae", "__Nitrospira"]}},{"id": "denovo2672", "metadata":
{"taxonomy": ["Bacteria", "__Verrucomicrobia", "__Verrucomicrobiae",
"__Verrucomicrobiales", "__Verrucomicrobiaceae", "__Haloferula"]}},
{"id": "denovo2677", "metadata": {"taxonomy": ["Bacteria",
"__Proteobacteria", "__Gammaproteobacteria",
"__E01-9C-26_marine_group", "__f", "__g"]}},{"id": "denovo2680",
"metadata": {"taxonomy": ["Bacteria", "__Proteobacteria",
"__Alphaproteobacteria"]}},{"id": "denovo2681", "metadata":
{"taxonomy": ["Bacteria", "__Bacteroidetes", "__Flavobacteria",
"__Flavobacteriales", "__Cryomorphaceae", "__Owenweeksia"]}},{"id":

"denovo2682", "metadata": {"taxonomy": ["Bacteria", "__Proteobacteria", "__Alphaproteobacteria", "__Kordiimonadales", "__Kordiimonadaceae", "__Kordiimonas"]}},{"id": "denovo2683", "metadata": {"taxonomy": ["Bacteria", "__Proteobacteria", "__Gammaproteobacteria", "__Pseudomonadales", "__Moraxellaceae", "__Acinetobacter"]}},{"id": "denovo2688", "metadata": {"taxonomy": ["Bacteria", "__Bacteroidetes", "__Sphingobacteriia", "__Sphingobacteriales", "__Saprospiraceae", "__Lewinella"]}},{"id": "denovo2690", "metadata": {"taxonomy": ["Bacteria", "__Proteobacteria", "__Alphaproteobacteria", "__Rhizobiales", "__Rhodobiaceae", "__Rhodobium"]}},{"id": "denovo2698", "metadata": {"taxonomy": ["Bacteria", "__Cyanobacteria", "__ML635J-21", "__o", "__f", "__g"]}},{"id": "denovo2702", "metadata": {"taxonomy": ["Bacteria", "__Proteobacteria", "__Gammaproteobacteria", "__NKB5", "__f", "__g"]}},{"id": "denovo2704", "metadata": {"taxonomy": ["Bacteria", "__Proteobacteria", "__Gammaproteobacteria", "__Order_Incertae_Sedis", "__Family_Incertae_Sedis", "__Marinicella"]}},{"id": "denovo2705", "metadata": {"taxonomy": ["Bacteria", "__Proteobacteria", "__Gammaproteobacteria"]}},{"id": "denovo2706", "metadata": {"taxonomy": ["Bacteria", "__Acidobacteria", "__Holophagae", "__CA002", "__f", "__g"]}},{"id": "denovo2707", "metadata": {"taxonomy": ["Bacteria", "__Proteobacteria", "__Alphaproteobacteria"]}},{"id": "denovo2712", "metadata": {"taxonomy": ["Bacteria", "__Proteobacteria", "__Alphaproteobacteria", "__OCS116_clade", "__f", "__g"]}},{"id": "denovo2715", "metadata": {"taxonomy": ["Bacteria", "__Proteobacteria", "__Alphaproteobacteria", "__Sphingomonadales", "__Sphingomonadaceae", "__Novosphingobium"]}},{"id": "denovo2717", "metadata": {"taxonomy": ["Bacteria", "__Proteobacteria", "__Alphaproteobacteria", "__Rhodobacterales", "__Rhodobacteraceae", "__g"]}},{"id": "denovo2721", "metadata": {"taxonomy": ["Bacteria", "__Bacteroidetes", "__Cytophagia", "__Cytophagales", "__Flammeovirgaceae", "__Candidatus_Amoebophilus"]}},{"id": "denovo2723", "metadata": {"taxonomy": ["Bacteria", "__Chloroflexi", "__Caldilineae", "__Caldilineales", "__Caldilineaceae", "__Caldilinea"]}},{"id": "denovo2726", "metadata": {"taxonomy": ["Bacteria", "__Bacteroidetes", "__Flavobacteria", "__Flavobacteriales", "__Cryomorphaceae", "__Crocinitomix"]}},{"id": "denovo2727", "metadata": {"taxonomy": ["Bacteria", "__Bacteroidetes"]}},{"id": "denovo2729", "metadata": {"taxonomy": ["Bacteria", "__Bacteroidetes", "__Flavobacteria", "__Flavobacteriales", "__Flavobacteriaceae"]}},{"id": "denovo2730", "metadata": {"taxonomy": ["Bacteria", "__Proteobacteria", "__Gammaproteobacteria"]}},{"id": "denovo2731", "metadata": {"taxonomy": ["Bacteria", "__Proteobacteria", "__Gammaproteobacteria", "__Order_Incertae_Sedis", "__Family_Incertae_Sedis", "__Thiohalophilus"]}},{"id": "denovo2732", "metadata": {"taxonomy": ["Bacteria", "__Proteobacteria", "__Gammaproteobacteria", "__Alteromonadales", "__Alteromonadaceae", "__Haliea"]}},{"id": "denovo2735", "metadata": {"taxonomy": ["Bacteria", "__Planctomycetes", "__Planctomycetacia", "__Planctomycetales",

"__Planctomycetaceae", "__Planctomyces"]}},{"id": "denovo2736", "metadata": {"taxonomy": ["Bacteria", "__Proteobacteria", "__Gammaproteobacteria", "__Alteromonadales", "__Shewanellaceae", "__Shewanella"]}},{"id": "denovo2738", "metadata": {"taxonomy": ["Archaea", "__Euryarchaeota", "__Thermoplasmata", "__Thermoplasmatales", "__Marine_Group_II", "__g"]}},{"id": "denovo2739", "metadata": {"taxonomy": ["Bacteria", "__Proteobacteria", "__Gammaproteobacteria", "__NKB5", "__f", "__g"]}},{"id": "denovo2743", "metadata": {"taxonomy": ["Bacteria", "__Proteobacteria"]}},{"id": "denovo2748", "metadata": {"taxonomy": ["Bacteria", "__Bacteroidetes", "__Sphingobacteriia", "__Sphingobacteriales", "__Saprospiraceae", "__Lewinella"]}},{"id": "denovo2752", "metadata": {"taxonomy": ["Bacteria", "__Cyanobacteria", "__SM2F09", "__o", "__f", "__g"]}},{"id": "denovo2762", "metadata": {"taxonomy": ["Archaea", "__Thaumarchaeota", "__Marine_Group_I", "__Candidatus_Nitrosopumilus", "__f", "__g"]}},{"id": "denovo2766", "metadata": {"taxonomy": ["Bacteria", "__Proteobacteria", "__Alphaproteobacteria", "__Rhodospirillales", "__Rhodospirillaceae", "__g"]}},{"id": "denovo2769", "metadata": {"taxonomy": ["Bacteria", "__Proteobacteria", "__Gammaproteobacteria", "__Chromatiales", "__Chromatiaceae", "__Nitrosococcus"]}},{"id": "denovo2771", "metadata": {"taxonomy": ["Bacteria", "__Proteobacteria", "__Gammaproteobacteria", "__Vibrionales", "__Vibrionaceae"]}},{"id": "denovo2775", "metadata": {"taxonomy": ["Bacteria", "__Cyanobacteria", "__ML635J-21", "__o", "__f", "__g"]}},{"id": "denovo2776", "metadata": {"taxonomy": ["Bacteria", "__Spirochaetes", "__Spirochaetes", "__Spirochaetales", "__Spirochaetaceae", "__Spirochaeta"]}},{"id": "denovo2779", "metadata": {"taxonomy": ["Archaea", "__Euryarchaeota", "__Halobacteria", "__Halobacteriales", "__Deep_Sea_Hydrothermal_Vent_Gp_6(DHVEG-6)", "__Candidatus_Parvarchaeum"]}},{"id": "denovo2783", "metadata": {"taxonomy": ["Bacteria", "__Planctomycetes", "__028H05-P-BN-P5", "__o", "__f", "__g"]}},{"id": "denovo2786", "metadata": {"taxonomy": ["Bacteria", "__Proteobacteria", "__Alphaproteobacteria", "__Rhizobiales", "__Methylobacteriaceae", "__Methylobacterium"]}},{"id": "denovo2791", "metadata": {"taxonomy": ["Bacteria", "__Proteobacteria", "__Gammaproteobacteria", "__Order_Incertae_Sedis", "__Family_Incertae_Sedis", "__Marinicella"]}},{"id": "denovo2792", "metadata": {"taxonomy": ["Bacteria"]}},{"id": "denovo2793", "metadata": {"taxonomy": ["Bacteria", "__Proteobacteria", "__Gammaproteobacteria", "__KI89A_clade", "__f", "__g"]}},{"id": "denovo2795", "metadata": {"taxonomy": ["Bacteria", "__Proteobacteria", "__Deltaproteobacteria", "__Desulfuromonadales", "__GR-WP33-58", "__g"]}},{"id": "denovo2796", "metadata": {"taxonomy": ["Bacteria", "__Proteobacteria", "__Gammaproteobacteria", "__Alteromonadales", "__Alteromonadaceae", "__OM60(NOR5)_clade"]}},{"id": "denovo2806", "metadata": {"taxonomy": ["Bacteria", "__Acidobacteria", "__RB25", "__o", "__f", "__g"]}},{"id": "denovo2809", "metadata": {"taxonomy": ["Bacteria", "__Proteobacteria", "__Gammaproteobacteria", "__Alteromonadales",

"__Moritellaceae", "__Paramoritella"]}},{"id": "denovo2813", "metadata": {"taxonomy": ["Bacteria", "__Proteobacteria", "__Gammaproteobacteria", "__Oceanospirillales", "__Alcanivoracaceae", "__Kangiella"]}},{"id": "denovo2815", "metadata": {"taxonomy": ["Bacteria", "__Actinobacteria", "__Acidimicrobiia", "__Acidimicrobiales", "__OCS155_marine_group", "__g"]}},{"id": "denovo2817", "metadata": {"taxonomy": ["Bacteria", "__Chloroflexi", "__SAR202_clade", "__o", "__f", "__g"]}},{"id": "denovo2819", "metadata": {"taxonomy": ["Bacteria", "__Gemmatimonadetes", "__Gemmatimonadetes", "__BD2-11_terrestrial_group", "__f", "__g"]}}, {"id": "denovo2823", "metadata": {"taxonomy": ["Bacteria", "__Proteobacteria", "__Gammaproteobacteria", "__Alteromonadales", "__Alteromonadaceae", "__Aestuariibacter"]}},{"id": "denovo2824", "metadata": {"taxonomy": ["Bacteria", "__Acidobacteria", "__Acidobacteria", "__Candidatus_Chloracidobacterium", "__f", "__g"]}},{"id": "denovo2829", "metadata": {"taxonomy": ["Bacteria", "__Proteobacteria", "__Alphaproteobacteria", "__Sphingomonadales", "__Sphingomonadaceae", "__Sphingomonas"]}},{"id": "denovo2832", "metadata": {"taxonomy": ["Bacteria", "__Proteobacteria", "__Alphaproteobacteria", "__Caulobacterales", "__Hyphomonadaceae", "__g"]}},{"id": "denovo2833", "metadata": {"taxonomy": ["Bacteria", "__Proteobacteria", "__Alphaproteobacteria", "__Rhodobacterales", "__Rhodobacteraceae", "__Roseobacter_clade_NAC11-7_lineage"]}},{"id": "denovo2835", "metadata": {"taxonomy": ["Bacteria", "__Proteobacteria", "__Deltaproteobacteria", "__Myxococcales", "__Sorangiineae", "__Sandaracinaceae"]}},{"id": "denovo2838", "metadata": {"taxonomy": ["Bacteria", "__Proteobacteria", "__Deltaproteobacteria", "__Desulfobacterales", "__Nitrospinaceae", "__Nitrospina"]}},{"id": "denovo2839", "metadata": {"taxonomy": ["Bacteria", "__Proteobacteria", "__Alphaproteobacteria"]}},{"id": "denovo2845", "metadata": {"taxonomy": ["Bacteria", "__Proteobacteria", "__Gammaproteobacteria", "__Enterobacteriales", "__Enterobacteriaceae", "__Shimwellia"]}},{"id": "denovo2846", "metadata": {"taxonomy": ["Bacteria", "__Proteobacteria"]}},{"id": "denovo2847", "metadata": {"taxonomy": ["Bacteria"]}},{"id": "denovo2848", "metadata": {"taxonomy": ["Bacteria", "__Proteobacteria", "__Alphaproteobacteria", "__DB1-14", "__f", "__g"]}},{"id": "denovo2849", "metadata": {"taxonomy": ["Bacteria", "__Proteobacteria", "__Gammaproteobacteria", "__Sva0071", "__f", "__g"]}},{"id": "denovo2851", "metadata": {"taxonomy": ["Bacteria", "__Cyanobacteria", "__Cyanobacteria", "__SubsectionII", "__FamilyII", "__Pleurocapsa"]}},{"id": "denovo2857", "metadata": {"taxonomy": ["Bacteria", "__Actinobacteria", "__Thermoleophilia", "__Gaiellales", "__uncultured", "__g"]}},{"id": "denovo2860", "metadata": {"taxonomy": ["Bacteria", "__Proteobacteria", "__Deltaproteobacteria", "__Bdellovibrionales", "__Bdellovibrionaceae", "__OM27_clade"]}}, {"id": "denovo2861", "metadata": {"taxonomy": ["Bacteria", "__Actinobacteria", "__Acidimicrobiia", "__Acidimicrobiales", "__OCS155_marine_group", "__g"]}},{"id": "denovo2864", "metadata": {"taxonomy": ["Bacteria", "__Proteobacteria", "__Gammaproteobacteria",

"__E01-9C-26_marine_group", "__f", "__g"]}},{"id": "denovo2870", "metadata": {"taxonomy": ["Bacteria", "__Proteobacteria", "__Alphaproteobacteria", "__Rickettsiales"]}},{"id": "denovo2871", "metadata": {"taxonomy": ["Bacteria", "__Proteobacteria", "__Gammaproteobacteria", "__Oceanospirillales", "__OM182_clade", "__g"]}},{"id": "denovo2874", "metadata": {"taxonomy": ["Bacteria", "__Proteobacteria", "__Alphaproteobacteria", "__DB1-14", "__f", "__g"]}},{"id": "denovo2875", "metadata": {"taxonomy": ["Bacteria", "__Chloroflexi", "__SAR202_clade", "__o", "__f", "__g"]}},{"id": "denovo2877", "metadata": {"taxonomy": ["Bacteria", "__Proteobacteria", "__Deltaproteobacteria", "__Bdellovibrionales", "__Bdellovibrionaceae", "__Bdellovibrio"]}},{"id": "denovo2881", "metadata": {"taxonomy": ["Bacteria", "__Bacteroidetes", "__Sphingobacteriia", "__Sphingobacteriales", "__Saprospiraceae", "__Aureispira"]}},{"id": "denovo2883", "metadata": {"taxonomy": ["Bacteria", "__Proteobacteria", "__Deltaproteobacteria", "__Desulfobacterales", "__Desulfobulbaceae"]}},{"id": "denovo2884", "metadata": {"taxonomy": ["Bacteria", "__Proteobacteria"]}},{"id": "denovo2885", "metadata": {"taxonomy": ["Bacteria", "__Proteobacteria", "__Alphaproteobacteria", "__Rhizobiales", "__Phyllobacteriaceae"]}},{"id": "denovo2887", "metadata": {"taxonomy": ["Bacteria", "__Proteobacteria", "__Alphaproteobacteria", "__Rickettsiales", "__Rickettsiaceae", "__g"]}},{"id": "denovo2888", "metadata": {"taxonomy": ["Bacteria", "__Proteobacteria", "__Gammaproteobacteria", "__Xanthomonadales", "__Sinobacteraceae", "__JTB255_marine_benthic_group"]}},{"id": "denovo2890", "metadata": {"taxonomy": ["Bacteria", "__Proteobacteria", "__Gammaproteobacteria", "__Oceanospirillales", "__Hahellaceae", "__Endozoicomonas"]}},{"id": "denovo2891", "metadata": {"taxonomy": ["Bacteria", "__Proteobacteria", "__Betaproteobacteria", "__Burkholderiales", "__Comamonadaceae", "__Aquabacterium"]}},{"id": "denovo2894", "metadata": {"taxonomy": ["Bacteria", "__Proteobacteria", "__Alphaproteobacteria", "__Rhodobacterales", "__Rhodobacteraceae", "__Ruegeria"]}},{"id": "denovo2895", "metadata": {"taxonomy": ["Bacteria", "__Proteobacteria", "__Gammaproteobacteria", "__Order_Incertae_Sedis", "__Family_Incertae_Sedis", "__Marinicella"]}},{"id": "denovo2896", "metadata": {"taxonomy": ["Bacteria", "__Proteobacteria", "__Gammaproteobacteria", "__KI89A_clade", "__f", "__g"]}},{"id": "denovo2901", "metadata": {"taxonomy": ["Bacteria", "__Cyanobacteria", "__Cyanobacteria", "__SubsectionIV", "__FamilyII", "__Rivularia"]}},{"id": "denovo2902", "metadata": {"taxonomy": ["Bacteria", "__Fusobacteria", "__Fusobacteria", "__Fusobacteriales", "__Fusobacteriaceae", "__Propionigenium"]}},{"id": "denovo2903", "metadata": {"taxonomy": ["Bacteria", "__Proteobacteria", "__Epsilonproteobacteria", "__Campylobacterales", "__Helicobacteraceae", "__Sulfurovum"]}},{"id": "denovo2906", "metadata": {"taxonomy": ["Bacteria", "__Proteobacteria", "__Gammaproteobacteria", "__Order_Incertae_Sedis", "__Family_Incertae_Sedis", "__Marinicella"]}},{"id": "denovo2911", "metadata": {"taxonomy": ["Bacteria", "__Proteobacteria",

"__Alphaproteobacteria", "__Rhodobacterales", "__Rhodobacteraceae", "__Labrenzia"]}},{"id": "denovo2919", "metadata": {"taxonomy": ["Bacteria", "__Acidobacteria", "__Holophagae", "__NS72", "__f", "__g"]}},{"id": "denovo2921", "metadata": {"taxonomy": ["Bacteria", "__Planctomycetes", "__Planctomycetacia", "__Planctomycetales", "__Planctomycetaceae", "__Rhodopirellula"]}},{"id": "denovo2930", "metadata": {"taxonomy": ["Bacteria", "__Proteobacteria", "__Alphaproteobacteria", "__Rickettsiales", "__SAR116_clade", "__g"]}},{"id": "denovo2932", "metadata": {"taxonomy": ["Bacteria", "__Proteobacteria", "__Alphaproteobacteria", "__Rhodospirillales", "__Rhodospirillaceae", "__g"]}},{"id": "denovo2933", "metadata": {"taxonomy": ["Bacteria", "__Proteobacteria", "__Alphaproteobacteria", "__Rhodobacterales", "__Rhodobacteraceae", "__Roseobacter_clade_AS-21_lineage"]}},{"id": "denovo2937", "metadata": {"taxonomy": ["Bacteria", "__Proteobacteria", "__Deltaproteobacteria", "__Desulfobacterales", "__Desulfobacteraceae", "__SEEP-SRB1"]}},{"id": "denovo2939", "metadata": {"taxonomy": ["Bacteria", "__Bacteroidetes", "__Flavobacteria", "__Flavobacteriales", "__Cryomorphaceae"]}},{"id": "denovo2943", "metadata": {"taxonomy": ["Bacteria", "__Proteobacteria", "__Alphaproteobacteria", "__Rhizobiales", "__Phyllobacteriaceae", "__Nitratireductor"]}},{"id": "denovo2944", "metadata": {"taxonomy": ["Bacteria", "__Chloroflexi", "__JG30-KF-CM66", "__o", "__f", "__g"]}},{"id": "denovo2947", "metadata": {"taxonomy": ["Bacteria", "__Proteobacteria", "__Deltaproteobacteria", "__Sh765B-TzT-29", "__f", "__g"]}},{"id": "denovo2952", "metadata": {"taxonomy": ["Bacteria", "__Proteobacteria", "__Alphaproteobacteria", "__Rhodospirillales", "__Rhodospirillaceae", "__Thalassospira"]}},{"id": "denovo2953", "metadata": {"taxonomy": ["Bacteria", "__Cyanobacteria", "__Cyanobacteria", "__SubsectionII", "__FamilyII", "__Pleurocapsa"]}},{"id": "denovo2956", "metadata": {"taxonomy": ["Bacteria", "__Actinobacteria", "__Acidimicrobiia", "__Acidimicrobiales", "__OCS155_marine_group", "__g"]}},{"id": "denovo2959", "metadata": {"taxonomy": ["Bacteria", "__Proteobacteria", "__Alphaproteobacteria", "__Rhodospirillales", "__Rhodospirillaceae", "__g"]}},{"id": "denovo2961", "metadata": {"taxonomy": ["Bacteria", "__Proteobacteria", "__Deltaproteobacteria", "__SAR324_clade(Marine_group_B)", "__f", "__g"]}},{"id": "denovo2966", "metadata": {"taxonomy": ["Bacteria", "__Proteobacteria", "__Deltaproteobacteria", "__Myxococcales", "__Nannocystineae", "__g"]}},{"id": "denovo2971", "metadata": {"taxonomy": ["Bacteria", "__Proteobacteria", "__Alphaproteobacteria", "__DB1-14", "__f", "__g"]}},{"id": "denovo2972", "metadata": {"taxonomy": ["Bacteria", "__Proteobacteria", "__Deltaproteobacteria", "__Desulfobacterales", "__Desulfobacteraceae", "__Sva0081_sediment_group"]}},{"id": "denovo2973", "metadata": {"taxonomy": ["Bacteria", "__Proteobacteria", "__Gammaproteobacteria", "__Order_Incertae_Sedis", "__Family_Incertae_Sedis", "__Arenicella"]}},{"id": "denovo2974", "metadata": {"taxonomy": ["Bacteria", "__Bacteroidetes", "__Flavobacteria", "__Flavobacteriales", "__Flavobacteriaceae",

"__Gilvibacter"]}},{"id": "denovo2975", "metadata": {"taxonomy": ["Bacteria", "__Proteobacteria", "__Gammaproteobacteria"]}},{"id": "denovo2977", "metadata": {"taxonomy": ["Bacteria", "__Proteobacteria", "__Alphaproteobacteria", "__Rhodobacterales", "__Rhodobacteraceae"]}},{"id": "denovo2978", "metadata": {"taxonomy": ["Bacteria", "__Proteobacteria", "__Alphaproteobacteria", "__Rhodospirillales"]}},{"id": "denovo2979", "metadata": {"taxonomy": ["Bacteria", "__Planctomycetes", "__Phycisphaerae", "__Phycisphaerales", "__Phycisphaeraceae", "__Urania-1B-19_marine_sediment_group"]}},{"id": "denovo2980", "metadata": {"taxonomy": ["Bacteria", "__Proteobacteria", "__Alphaproteobacteria", "__Rhodospirillales", "__MSB-1E8", "__g"]}},{"id": "denovo2981", "metadata": {"taxonomy": ["Bacteria", "__Proteobacteria", "__Gammaproteobacteria", "__Xanthomonadales", "__Sinobacteraceae", "__JTB255_marine_benthic_group"]}},{"id": "denovo2982", "metadata": {"taxonomy": ["Bacteria", "__Verrucomicrobia", "__Opitutae", "__Puniceicoccales", "__Puniceicoccaceae", "__Cerasicoccus"]}},{"id": "denovo2984", "metadata": {"taxonomy": ["Bacteria", "__Proteobacteria", "__Alphaproteobacteria", "__Rhodospirillales", "__Rhodospirillaceae"]}},{"id": "denovo2989", "metadata": {"taxonomy": ["Bacteria", "__Proteobacteria", "__Deltaproteobacteria", "__Sh765B-TzT-29", "__f", "__g"]}},{"id": "denovo2990", "metadata": {"taxonomy": ["Bacteria", "__Proteobacteria", "__Alphaproteobacteria", "__Sphingomonadales", "__Sphingomonadaceae", "__Sphingomonas"]}},{"id": "denovo2994", "metadata": {"taxonomy": ["Bacteria", "__Proteobacteria", "__Gammaproteobacteria", "__Order_Incertae_Sedis", "__Family_Incertae_Sedis", "__Arenicella"]}},{"id": "denovo2999", "metadata": {"taxonomy": ["Bacteria", "__Proteobacteria", "__Alphaproteobacteria", "__DB1-14", "__f", "__g"]}},{"id": "denovo3000", "metadata": {"taxonomy": ["Bacteria", "__Proteobacteria", "__Alphaproteobacteria", "__Rhodobacterales", "__Rhodobacteraceae"]}},{"id": "denovo3001", "metadata": {"taxonomy": ["Bacteria", "__Proteobacteria", "__Deltaproteobacteria"]}},{"id": "denovo3002", "metadata": {"taxonomy": ["Bacteria", "__Cyanobacteria", "__Cyanobacteria", "__SubsectionIII", "__FamilyI", "__Lyngbya"]}},{"id": "denovo3006", "metadata": {"taxonomy": ["Bacteria", "__Bacteroidetes", "__Sphingobacteriia", "__Sphingobacteriales", "__Saprospiraceae", "__g"]}},{"id": "denovo3007", "metadata": {"taxonomy": ["Bacteria", "__Proteobacteria", "__Deltaproteobacteria", "__Sh765B-TzT-29", "__f", "__g"]}},{"id": "denovo3008", "metadata": {"taxonomy": ["Bacteria", "__Proteobacteria", "__Deltaproteobacteria", "__Myxococcales", "__Sorangiineae", "__g"]}},{"id": "denovo3016", "metadata": {"taxonomy": ["Bacteria", "__Acidobacteria", "__Acidobacteria", "__PAUC26f", "__f", "__g"]}},{"id": "denovo3021", "metadata": {"taxonomy": ["Bacteria", "__Proteobacteria", "__Deltaproteobacteria", "__Desulfobacterales", "__Desulfobacteraceae", "__g"]}},{"id": "denovo3025", "metadata": {"taxonomy": ["Bacteria", "__Proteobacteria", "__Deltaproteobacteria", "__Sva0485", "__f", "__g"]}},{"id": "denovo3026", "metadata":

{"taxonomy": ["Bacteria", "__Proteobacteria", "__Alphaproteobacteria", "__Rhodospirillales", "__Rhodospirillaceae"]}},{"id": "denovo3027", "metadata": {"taxonomy": ["Bacteria", "__Proteobacteria", "__Alphaproteobacteria", "__Rhodobacterales", "__Rhodobacteraceae", "__Roseobacter_clade_NAC11-7_lineage"]}},{"id": "denovo3028", "metadata": {"taxonomy": ["Bacteria", "__Acidobacteria", "__Holophagae", "__NS72", "__f", "__g"]}},{"id": "denovo3033", "metadata": {"taxonomy": ["Bacteria", "__Proteobacteria", "__Alphaproteobacteria", "__Rhodobacterales", "__Rhodobacteraceae"]}},{"id": "denovo3035", "metadata": {"taxonomy": ["Bacteria", "__Planctomycetes", "__Planctomycetacia", "__Planctomycetales", "__Planctomycetaceae", "__Planctomyces"]}},{"id": "denovo3044", "metadata": {"taxonomy": ["Bacteria", "__Bacteroidetes", "__Sphingobacteriia", "__Sphingobacteriales", "__NS11-12_marine_group", "__g"]}},{"id": "denovo3046", "metadata": {"taxonomy": ["Bacteria", "__Bacteroidetes", "__Flavobacteria", "__Flavobacteriales", "__Flavobacteriaceae", "__Gilvibacter"]}},{"id": "denovo3050", "metadata": {"taxonomy": ["Bacteria", "__Proteobacteria", "__Epsilonproteobacteria", "__Campylobacterales", "__Helicobacteraceae", "__Sulfurovum"]}},{"id": "denovo3051", "metadata": {"taxonomy": ["Bacteria", "__Proteobacteria", "__Gammaproteobacteria", "__Vibrionales", "__Vibrionaceae", "__Vibrio"]}},{"id": "denovo3054", "metadata": {"taxonomy": ["Bacteria", "__Firmicutes", "__Clostridia", "__Clostridiales", "__Christensenellaceae"]}},{"id": "denovo3056", "metadata": {"taxonomy": ["Bacteria", "__Chloroflexi", "__Anaerolineae", "__Anaerolineales", "__Anaerolineaceae", "__g"]}},{"id": "denovo3057", "metadata": {"taxonomy": ["Bacteria", "__Proteobacteria", "__Gammaproteobacteria", "__Chromatiales", "__Chromatiaceae", "__Nitrosococcus"]}},{"id": "denovo3059", "metadata": {"taxonomy": ["Bacteria", "__Bacteroidetes", "__BD2-2", "__o", "__f", "__g"]}},{"id": "denovo3060", "metadata": {"taxonomy": ["Bacteria", "__Bacteroidetes", "__Sphingobacteriia", "__Sphingobacteriales", "__Saprospiraceae", "__g"]}},{"id": "denovo3061", "metadata": {"taxonomy": ["Bacteria", "__Bacteroidetes", "__Flavobacteria", "__Flavobacteriales", "__Flavobacteriaceae", "__Aquimarina"]}},{"id": "denovo3063", "metadata": {"taxonomy": ["Bacteria", "__Acidobacteria", "__Acidobacteria", "__BPC102", "__f", "__g"]}},{"id": "denovo3064", "metadata": {"taxonomy": ["Bacteria", "__Actinobacteria", "__Acidimicrobiia", "__Acidimicrobiales", "__uncultured", "__g"]}},{"id": "denovo3067", "metadata": {"taxonomy": ["Bacteria", "__Proteobacteria", "__Gammaproteobacteria", "__Legionellales", "__Coxiellaceae", "__Coxiella"]}},{"id": "denovo3070", "metadata": {"taxonomy": ["Bacteria", "__Proteobacteria", "__Alphaproteobacteria", "__Rhodobacterales", "__Rhodobacteraceae", "__Maritimibacter"]}},{"id": "denovo3072", "metadata": {"taxonomy": ["Bacteria", "__Proteobacteria", "__Alphaproteobacteria", "__Rhodospirillales", "__Rhodospirillaceae"]}},{"id": "denovo3076", "metadata": {"taxonomy": ["Bacteria", "__Proteobacteria", "__Gammaproteobacteria", "__Enterobacteriales", "__Enterobacteriaceae"]}},{"id": "denovo3081",

"metadata": {"taxonomy": ["Bacteria", "__Proteobacteria", "__Gammaproteobacteria", "__Oceanospirillales", "__Hahellaceae", "__Endozoicomonas"]}},{"id": "denovo3082", "metadata": {"taxonomy": ["Bacteria", "__Proteobacteria", "__Alphaproteobacteria", "__Rhodobacterales", "__Rhodobacteraceae", "__g"]}},{"id": "denovo3087", "metadata": {"taxonomy": ["Bacteria", "__Proteobacteria", "__Alphaproteobacteria", "__Rhodospirillales", "__Rhodospirillaceae", "__g"]}},{"id": "denovo3094", "metadata": {"taxonomy": ["Bacteria", "__Nitrospirae", "__Nitrospira", "__Nitrospirales", "__Nitrospiraceae", "__Nitrospira"]}},{"id": "denovo3096", "metadata": {"taxonomy": ["Bacteria", "__Proteobacteria"]}},{"id": "denovo3098", "metadata": {"taxonomy": ["Bacteria", "__Verrucomicrobia", "__Opitutae", "__Puniceicoccales", "__Puniceicoccaceae", "__Cerasicoccus"]}},{"id": "denovo3099", "metadata": {"taxonomy": ["Bacteria", "__Planctomycetes", "__Planctomycetacia", "__Planctomycetales", "__Planctomycetaceae", "__Blastopirellula"]}},{"id": "denovo3100", "metadata": {"taxonomy": ["Bacteria", "__Planctomycetes", "__Phycisphaerae", "__Phycisphaerales", "__Phycisphaeraceae", "__Urania-1B-19_marine_sediment_group"]}},{"id": "denovo3109", "metadata": {"taxonomy": ["Bacteria", "__Proteobacteria", "__Gammaproteobacteria", "__KI89A_clade", "__f", "__g"]}},{"id": "denovo3113", "metadata": {"taxonomy": ["Bacteria", "__Proteobacteria", "__Alphaproteobacteria"]}},{"id": "denovo3114", "metadata": {"taxonomy": ["Bacteria", "__Proteobacteria", "__Alphaproteobacteria", "__Rhizobiales", "__Rhodobiaceae", "__Rhodobium"]}},{"id": "denovo3115", "metadata": {"taxonomy": ["Bacteria", "__Bacteroidetes", "__Flavobacteria", "__Flavobacteriales", "__Flavobacteriaceae", "__Maritimimonas"]}},{"id": "denovo3116", "metadata": {"taxonomy": ["Bacteria", "__Proteobacteria", "__Alphaproteobacteria", "__Rhodobacterales", "__Rhodobacteraceae", "__Ruegeria"]}},{"id": "denovo3117", "metadata": {"taxonomy": ["Bacteria", "__Proteobacteria", "__Deltaproteobacteria", "__Desulfobacterales", "__Nitrospinaceae", "__Candidatus_Entotheonella"]}},{"id": "denovo3122", "metadata": {"taxonomy": ["Bacteria", "__Proteobacteria", "__Gammaproteobacteria", "__Xanthomonadales", "__Sinobacteraceae", "__JTB255_marine_benthic_group"]}},{"id": "denovo3124", "metadata": {"taxonomy": ["Bacteria", "__Bacteroidetes", "__Sphingobacteriia", "__Sphingobacteriales", "__NS11-12_marine_group", "__g"]}},{"id": "denovo3128", "metadata": {"taxonomy": ["Bacteria", "__Proteobacteria", "__Gammaproteobacteria", "__Alteromonadales", "__Alteromonadaceae", "__Dasania"]}},{"id": "denovo3132", "metadata": {"taxonomy": ["Bacteria", "__Proteobacteria", "__Alphaproteobacteria", "__Caulobacterales", "__Hyphomonadaceae", "__Hellea"]}},{"id": "denovo3149", "metadata": {"taxonomy": ["Bacteria", "__Proteobacteria", "__Alphaproteobacteria", "__Rhodobacterales", "__Rhodobacteraceae", "__Roseobacter_clade_NAC11-7_lineage"]}},{"id": "denovo3151", "metadata": {"taxonomy": ["Bacteria", "__Cyanobacteria", "__Cyanobacteria", "__SubsectionIII", "__FamilyI", "__Phormidium"]}},

{"id": "denovo3152", "metadata": {"taxonomy": ["Bacteria", "__Proteobacteria", "__Gammaproteobacteria"]}},{"id": "denovo3154", "metadata": {"taxonomy": ["Bacteria", "__Proteobacteria", "__Gammaproteobacteria", "__NKB5", "__f", "__g"]}},{"id": "denovo3155", "metadata": {"taxonomy": ["Bacteria", "__Proteobacteria", "__Alphaproteobacteria", "__DB1-14", "__f", "__g"]}},{"id": "denovo3157", "metadata": {"taxonomy": ["Bacteria", "__Proteobacteria", "__Gammaproteobacteria", "__SS1-B-09-64", "__f", "__g"]}},{"id": "denovo3158", "metadata": {"taxonomy": ["Bacteria", "__Proteobacteria", "__Deltaproteobacteria", "__Sh765B-TzT-29", "__f", "__g"]}},{"id": "denovo3159", "metadata": {"taxonomy": ["Bacteria", "__Proteobacteria", "__Alphaproteobacteria", "__Rhodobacterales", "__Rhodobacteraceae", "__Ruegeria"]}},{"id": "denovo3163", "metadata": {"taxonomy": ["Bacteria", "__Bacteroidetes", "__Cytophagia", "__Cytophagales", "__Flammeovirgaceae", "__Marivirga"]}},{"id": "denovo3164", "metadata": {"taxonomy": ["Bacteria", "__Proteobacteria", "__Gammaproteobacteria", "__Oceanospirillales"]}},{"id": "denovo3167", "metadata": {"taxonomy": ["Bacteria", "__Proteobacteria", "__Gammaproteobacteria"]}},{"id": "denovo3168", "metadata": {"taxonomy": ["Bacteria", "__Planctomycetes", "__Planctomycetacia", "__Brocadiales", "__Brocadiaceae"]}},{"id": "denovo3174", "metadata": {"taxonomy": ["Bacteria", "__Proteobacteria", "__Gammaproteobacteria", "__Oceanospirillales", "__Oceanospirillaceae", "__Pseudospirillum"]}},{"id": "denovo3175", "metadata": {"taxonomy": ["Bacteria", "__Deferribacteres", "__Deferribacteres", "__Deferribacterales", "__Family_Incertae_Sedis", "__Caldithrix"]}},{"id": "denovo3176", "metadata": {"taxonomy": ["Bacteria", "__Proteobacteria", "__Alphaproteobacteria", "__Rhizobiales", "__Rhodobiaceae", "__Rhodobium"]}},{"id": "denovo3177", "metadata": {"taxonomy": ["Bacteria", "__Actinobacteria", "__Actinobacteria", "__Micrococcales", "__Micrococcaceae", "__Nesterenkonia"]}},{"id": "denovo3179", "metadata": {"taxonomy": ["Bacteria", "__Proteobacteria", "__Alphaproteobacteria", "__DB1-14", "__f", "__g"]}},{"id": "denovo3183", "metadata": {"taxonomy": ["Bacteria", "__Proteobacteria"]}},{"id": "denovo3185", "metadata": {"taxonomy": ["Bacteria", "__Proteobacteria", "__Gammaproteobacteria", "__Enterobacteriales", "__Enterobacteriaceae"]}},{"id": "denovo3189", "metadata": {"taxonomy": ["Bacteria", "__Cyanobacteria", "__Cyanobacteria", "__SubsectionIII", "__FamilyI", "__Phormidium"]}},{"id": "denovo3190", "metadata": {"taxonomy": ["Bacteria", "__Proteobacteria", "__TA18", "__o", "__f", "__g"]}},{"id": "denovo3192", "metadata": {"taxonomy": ["Bacteria", "__Proteobacteria", "__Alphaproteobacteria", "__Rickettsiales", "__SM2D12", "__g"]}},{"id": "denovo3196", "metadata": {"taxonomy": ["Bacteria", "__Proteobacteria", "__Gammaproteobacteria", "__Alteromonadales", "__NB-1d", "__g"]}},{"id": "denovo3198", "metadata": {"taxonomy": ["Bacteria", "__Proteobacteria", "__Gammaproteobacteria", "__Alteromonadales", "__Colwelliaceae", "__Thalassomonas"]}},{"id": "denovo3199", "metadata": {"taxonomy":

["Bacteria", "__Firmicutes", "__Clostridia", "__Clostridiales", "__Christensenellaceae", "__Christensenella"]}},{"id": "denovo3200", "metadata": {"taxonomy": ["Bacteria", "__Cyanobacteria", "__Cyanobacteria", "__SubsectionIII", "__FamilyI", "__Phormidium"]}}, {"id": "denovo3202", "metadata": {"taxonomy": ["Bacteria", "__Proteobacteria", "__Alphaproteobacteria", "__SAR11_clade", "__Surface_1", "__g"]}},{"id": "denovo3206", "metadata": {"taxonomy": ["Bacteria", "__Proteobacteria", "__Gammaproteobacteria", "__Oceanospirillales", "__Oleiphilaceae", "__Oleiphilus"]}},{"id": "denovo3208", "metadata": {"taxonomy": ["Bacteria", "__Cyanobacteria", "__Cyanobacteria", "__SubsectionII", "__FamilyII", "__Pleurocapsa"]}}, {"id": "denovo3212", "metadata": {"taxonomy": ["Bacteria", "__Proteobacteria", "__Alphaproteobacteria", "__Rhodobacterales", "__Rhodobacteraceae", "__Rhodovulum"]}},{"id": "denovo3213", "metadata": {"taxonomy": ["Bacteria", "__Bacteroidetes", "__Cytophagia", "__Cytophagales", "__Flammeovirgaceae", "__Ekhidna"]}},{"id": "denovo3216", "metadata": {"taxonomy": ["Bacteria", "__Proteobacteria", "__Alphaproteobacteria", "__Rhizobiales"]}},{"id": "denovo3220", "metadata": {"taxonomy": ["Bacteria", "__Proteobacteria", "__Gammaproteobacteria", "__Order_Incertae_Sedis", "__Family_Incertae_Sedis", "__Sedimenticola"]}},{"id": "denovo3221", "metadata": {"taxonomy": ["Bacteria", "__Proteobacteria", "__Gammaproteobacteria", "__Oceanospirillales", "__OM182_clade", "__g"]}},{"id": "denovo3222", "metadata": {"taxonomy": ["Bacteria", "__Proteobacteria", "__Deltaproteobacteria", "__GR-WP33-30", "__f", "__g"]}},{"id": "denovo3223", "metadata": {"taxonomy": ["Bacteria", "__Proteobacteria", "__Gammaproteobacteria", "__Pseudomonadales", "__Moraxellaceae", "__Acinetobacter"]}},{"id": "denovo3224", "metadata": {"taxonomy": ["Bacteria", "__Proteobacteria", "__Gammaproteobacteria", "__E01-9C-26_marine_group", "__f", "__g"]}}, {"id": "denovo3235", "metadata": {"taxonomy": ["Bacteria", "__Proteobacteria", "__Alphaproteobacteria", "__DB1-14", "__f", "__g"]}},{"id": "denovo3236", "metadata": {"taxonomy": ["Bacteria", "__Cyanobacteria", "__Cyanobacteria", "__SubsectionIII", "__FamilyI", "__Halomicronema"]}},{"id": "denovo3238", "metadata": {"taxonomy": ["Bacteria", "__Proteobacteria", "__Gammaproteobacteria", "__Oceanospirillales", "__OM182_clade", "__g"]}},{"id": "denovo3239", "metadata": {"taxonomy": ["Bacteria", "__Proteobacteria", "__Betaproteobacteria", "__Burkholderiales", "__Alcaligenaceae", "__Achromobacter"]}},{"id": "denovo3242", "metadata": {"taxonomy": ["Bacteria", "__Actinobacteria", "__Acidimicrobiia", "__Acidimicrobiales", "__uncultured", "__g"]}},{"id": "denovo3244", "metadata": {"taxonomy": ["Bacteria", "__Cyanobacteria", "__Cyanobacteria"]}},{"id": "denovo3246", "metadata": {"taxonomy": ["Bacteria", "__Proteobacteria", "__Gammaproteobacteria", "__Xanthomonadales", "__Sinobacteraceae", "__JTB255_marine_benthic_group"]}},{"id": "denovo3252", "metadata": {"taxonomy": ["Bacteria", "__Firmicutes", "__Clostridia", "__Clostridiales", "__Clostridiaceae", "__Clostridiisalibacter"]}},

{"id": "denovo3253", "metadata": {"taxonomy": ["Bacteria", "__Proteobacteria", "__Gammaproteobacteria", "__Oceanospirillales", "__Oceanospirillaceae", "__Marinobacterium"]}},{"id": "denovo3254", "metadata": {"taxonomy": ["Bacteria", "__Spirochaetes", "__Spirochaetes", "__Spirochaetales", "__Leptospiraceae", "__g"]}}, {"id": "denovo3255", "metadata": {"taxonomy": ["Bacteria", "__Proteobacteria", "__Alphaproteobacteria", "__iodide-oxidizers", "__f", "__g"]}},{"id": "denovo3256", "metadata": {"taxonomy": ["Bacteria", "__Proteobacteria", "__Alphaproteobacteria"]}},{"id": "denovo3257", "metadata": {"taxonomy": ["Bacteria", "__Acidobacteria", "__Acidobacteria", "__DA023", "__f", "__g"]}},{"id": "denovo3261", "metadata": {"taxonomy": ["Bacteria", "__Proteobacteria", "__Alphaproteobacteria", "__DB1-14", "__f", "__g"]}},{"id": "denovo3267", "metadata": {"taxonomy": ["Bacteria", "__Proteobacteria", "__Gammaproteobacteria", "__Order_Incertae_Sedis", "__Family_Incertae_Sedis", "__Sedimenticola"]}},{"id": "denovo3268", "metadata": {"taxonomy": ["Bacteria", "__Proteobacteria", "__Alphaproteobacteria", "__OCS116_clade", "__f", "__g"]}},{"id": "denovo3270", "metadata": {"taxonomy": ["Bacteria", "__Proteobacteria", "__Gammaproteobacteria"]}},{"id": "denovo3273", "metadata": {"taxonomy": ["Bacteria", "__Proteobacteria", "__Alphaproteobacteria", "__Rickettsiales", "__SM2D12", "__g"]}}, {"id": "denovo3276", "metadata": {"taxonomy": ["Bacteria", "__Proteobacteria", "__Gammaproteobacteria", "__Xanthomonadales", "__Sinobacteraceae", "__JTB255_marine_benthic_group"]}},{"id": "denovo3278", "metadata": {"taxonomy": ["Bacteria", "__Deinococcus-Thermus", "__Deinococci", "__Deinococcales", "__Trueperaceae", "__Truepera"]}},{"id": "denovo3285", "metadata": {"taxonomy": ["Bacteria", "__Firmicutes", "__Clostridia", "__Clostridiales", "__Lachnospiraceae"]}},{"id": "denovo3292", "metadata": {"taxonomy": ["Bacteria", "__Proteobacteria", "__Alphaproteobacteria", "__Rhodobacterales", "__Rhodobacteraceae", "__Ruegeria"]}},{"id": "denovo3293", "metadata": {"taxonomy": ["Bacteria", "__Proteobacteria", "__Deltaproteobacteria", "__Bdellovibrionales", "__Bdellovibrionaceae", "__OM27_clade"]}},{"id": "denovo3295", "metadata": {"taxonomy": ["Bacteria", "__Nitrospirae", "__Nitrospira", "__Nitrospirales", "__Nitrospiraceae", "__Nitrospira"]}},{"id": "denovo3296", "metadata": {"taxonomy": ["Bacteria", "__Proteobacteria", "__Deltaproteobacteria", "__Desulfobacterales", "__Desulfobacteraceae"]}},{"id": "denovo3301", "metadata": {"taxonomy": ["Bacteria", "__Deferribacteres", "__Deferribacteres", "__Deferribacterales", "__PAUC34f", "__g"]}},{"id": "denovo3303", "metadata": {"taxonomy": ["Bacteria", "__Proteobacteria", "__Gammaproteobacteria", "__BD7-8_marine_group", "__f", "__g"]}}, {"id": "denovo3305", "metadata": {"taxonomy": ["Bacteria", "__Bacteroidetes", "__Sphingobacteriia", "__Sphingobacteriales", "__Saprospiraceae", "__g"]}},{"id": "denovo3306", "metadata": {"taxonomy": ["Bacteria", "__Candidate_division_TM7", "__c", "__o", "__f", "__g"]}},{"id": "denovo3308", "metadata": {"taxonomy": ["Bacteria", "__Bacteroidetes", "__Cytophagia", "__Cytophagales",

"__Flammeovirgaceae", "__Candidatus_Amoebophilus"]}},{"id":
"denovo3309", "metadata": {"taxonomy": ["Bacteria", "__Bacteroidetes",
"__Flavobacteria", "__Flavobacteriales", "__Flavobacteriaceae",
"__Polaribacter"]}},{"id": "denovo3312", "metadata": {"taxonomy":
["Bacteria", "__Cyanobacteria", "__Cyanobacteria", "__SubsectionIII",
"__FamilyI"]}},{"id": "denovo3317", "metadata": {"taxonomy":
["Bacteria", "__Bacteroidetes", "__Sphingobacteriia",
"__Sphingobacteriales", "__Saprospiraceae", "__g"]}},{"id":
"denovo3323", "metadata": {"taxonomy": ["Bacteria",
"__Proteobacteria", "__Gammaproteobacteria"]}},{"id": "denovo3324",
"metadata": {"taxonomy": ["Bacteria", "__Proteobacteria",
"__Alphaproteobacteria", "__Rhodospirillales", "__Rhodospirillaceae",
"__Thalassospira"]}},{"id": "denovo3329", "metadata": {"taxonomy":
["Bacteria", "__Proteobacteria", "__Gammaproteobacteria",
"__Pseudomonadales", "__Moraxellaceae", "__Acinetobacter"]}},{"id":
"denovo3333", "metadata": {"taxonomy": ["Bacteria", "__Acidobacteria",
"__RB25", "__o", "__f", "__g"]}},{"id": "denovo3334", "metadata":
{"taxonomy": ["Bacteria", "__Proteobacteria", "__Alphaproteobacteria",
"__Rhizobiales", "__Phyllobacteriaceae", "__g"]}},{"id": "denovo3336",
"metadata": {"taxonomy": ["Bacteria", "__Proteobacteria",
"__Deltaproteobacteria", "__Myxococcales", "__Nannocystaceae",
"__g"]}},{"id": "denovo3344", "metadata": {"taxonomy": ["Bacteria",
"__Proteobacteria", "__Gammaproteobacteria", "__Order_Incertae_Sedis",
"__Family_Incertae_Sedis", "__Marinicella"]}},{"id": "denovo3345",
"metadata": {"taxonomy": ["Bacteria", "__Cyanobacteria",
"__Cyanobacteria", "__SubsectionIII", "__FamilyI"]}},{"id":
"denovo3348", "metadata": {"taxonomy": ["Bacteria", "__Firmicutes",
"__Clostridia", "__Clostridiales", "__Lachnospiraceae"]}},{"id":
"denovo3351", "metadata": {"taxonomy": ["Bacteria",
"__Proteobacteria", "__Gammaproteobacteria", "__KI89A_clade", "__f",
"__g"]}},{"id": "denovo3353", "metadata": {"taxonomy": ["Bacteria",
"__Proteobacteria", "__Gammaproteobacteria", "__Alteromonadales",
"__Idiomarinaceae", "__Pseudidiomarina"]}},{"id": "denovo3354",
"metadata": {"taxonomy": ["Bacteria", "__Proteobacteria",
"__Gammaproteobacteria", "__Alteromonadales", "__Ferrimonadaceae",
"__Ferrimonas"]}},{"id": "denovo3355", "metadata": {"taxonomy":
["Bacteria", "__Bacteroidetes", "__Sphingobacteriia",
"__Sphingobacteriales", "__Saprospiraceae", "__g"]}},{"id":
"denovo3356", "metadata": {"taxonomy": ["Bacteria",
"__Proteobacteria", "__Deltaproteobacteria", "__Desulfobacterales",
"__Desulfobacteraceae", "__Desulforegula"]}},{"id": "denovo3357",
"metadata": {"taxonomy": ["Bacteria", "__Proteobacteria",
"__Gammaproteobacteria"]}},{"id": "denovo3358", "metadata":
{"taxonomy": ["Bacteria", "__Proteobacteria", "__Gammaproteobacteria",
"__Oceanospirillales", "__OM182_clade", "__g"]}},{"id": "denovo3360",
"metadata": {"taxonomy": ["Bacteria", "__Proteobacteria",
"__Alphaproteobacteria", "__OCS116_clade", "__f", "__g"]}},{"id":
"denovo3361", "metadata": {"taxonomy": ["Bacteria", "__Bacteroidetes",
"__Flavobacteria", "__Flavobacteriales", "__Flavobacteriaceae",
"__Polaribacter"]}},{"id": "denovo3365", "metadata": {"taxonomy":

["Bacteria", "__Verrucomicrobia", "__Verrucomicrobiae", "__Verrucomicrobiales", "__Rubritaleaceae", "__Rubritalea"]}},{"id": "denovo3368", "metadata": {"taxonomy": ["Bacteria", "__Proteobacteria", "__Deltaproteobacteria", "__Bdellovibrionales", "__Bacteriovoracaceae", "__Peredibacter"]}},{"id": "denovo3369", "metadata": {"taxonomy": ["Bacteria", "__Proteobacteria", "__Gammaproteobacteria", "__Oceanospirillales", "__Hahellaceae", "__Endozoicomonas"]}},{"id": "denovo3372", "metadata": {"taxonomy": ["Bacteria", "__Proteobacteria", "__Alphaproteobacteria", "__Rhizobiales", "__Phyllobacteriaceae"]}},{"id": "denovo3373", "metadata": {"taxonomy": ["Bacteria", "__Proteobacteria", "__Deltaproteobacteria", "__Myxococcales", "__Sorangiineae", "__g"]}},{"id": "denovo3374", "metadata": {"taxonomy": ["Bacteria", "__Proteobacteria", "__Deltaproteobacteria", "__Sh765B-TzT-29", "__f", "__g"]}},{"id": "denovo3378", "metadata": {"taxonomy": ["Bacteria", "__Proteobacteria", "__Alphaproteobacteria", "__Rhodospirillales", "__Rhodospirillaceae", "__g"]}},{"id": "denovo3379", "metadata": {"taxonomy": ["Bacteria", "__Proteobacteria", "__Gammaproteobacteria", "__Pseudomonadales", "__Moraxellaceae", "__Acinetobacter"]}},{"id": "denovo3380", "metadata": {"taxonomy": ["Bacteria", "__Firmicutes", "__Clostridia", "__Clostridiales", "__Family_XII_Incertae_Sedis", "__Fusibacter"]}},{"id": "denovo3381", "metadata": {"taxonomy": ["Bacteria", "__Bacteroidetes", "__Sphingobacteriia", "__Sphingobacteriales", "__Saprospiraceae", "__g"]}},{"id": "denovo3383", "metadata": {"taxonomy": ["Bacteria", "__Proteobacteria", "__Deltaproteobacteria", "__Sh765B-TzT-29", "__f", "__g"]}},{"id": "denovo3391", "metadata": {"taxonomy": ["Archaea", "__Euryarchaeota", "__Thermoplasmata", "__Thermoplasmatales", "__Marine_Group_II", "__g"]}},{"id": "denovo3396", "metadata": {"taxonomy": ["Bacteria", "__Proteobacteria"]}},{"id": "denovo3403", "metadata": {"taxonomy": ["Bacteria", "__Chloroflexi", "__Anaerolineae", "__Anaerolineales", "__Anaerolineaceae", "__g"]}},{"id": "denovo3404", "metadata": {"taxonomy": ["Bacteria", "__Proteobacteria", "__Alphaproteobacteria", "__Rhodobacterales", "__Rhodobacteraceae"]}},{"id": "denovo3405", "metadata": {"taxonomy": ["Bacteria", "__Proteobacteria", "__Alphaproteobacteria", "__Rhodospirillales", "__Rhodospirillaceae", "__g"]}},{"id": "denovo3408", "metadata": {"taxonomy": ["Bacteria", "__Proteobacteria", "__Gammaproteobacteria", "__Thiotrichales", "__Thiotrichaceae", "__Thiothrix"]}},{"id": "denovo3411", "metadata": {"taxonomy": ["Bacteria", "__Proteobacteria", "__Alphaproteobacteria", "__SAR11_clade", "__Surface_2", "__g"]}},{"id": "denovo3412", "metadata": {"taxonomy": ["Bacteria", "__Proteobacteria", "__Deltaproteobacteria", "__Myxococcales", "__Sandaracinaceae", "__Sandaracinus"]}},{"id": "denovo3416", "metadata": {"taxonomy": ["Bacteria", "__Proteobacteria", "__Gammaproteobacteria", "__BD7-8_marine_group", "__f", "__g"]}},{"id": "denovo3424", "metadata": {"taxonomy": ["Bacteria", "__Chloroflexi", "__SAR202_clade", "__o", "__f", "__g"]}},{"id": "denovo3428", "metadata": {"taxonomy": ["Bacteria", "__Proteobacteria"]}},{"id":

"denovo3432", "metadata": {"taxonomy": ["Bacteria",
"__Proteobacteria", "__Gammaproteobacteria", "__NKB5", "__f",
"__g"]}},{"id": "denovo3438", "metadata": {"taxonomy": ["Bacteria",
"__Proteobacteria", "__Alphaproteobacteria", "__Rhodobacterales",
"__Rhodobacteraceae"]}},{"id": "denovo3440", "metadata": {"taxonomy":
["Bacteria", "__Proteobacteria", "__Gammaproteobacteria",
"__Vibrionales", "__Vibrionaceae", "__Vibrio"]}},{"id": "denovo3441",
"metadata": {"taxonomy": ["Bacteria", "__Proteobacteria",
"__Alphaproteobacteria", "__Rhodobacterales", "__Rhodobacteraceae"]}},
{"id": "denovo3443", "metadata": {"taxonomy": ["Bacteria",
"__Planctomycetes", "__Phycisphaerae", "__Phycisphaerales",
"__Phycisphaeraceae", "__Urania-1B-19_marine_sediment_group"]}},{"id":
"denovo3444", "metadata": {"taxonomy": ["Bacteria",
"__Proteobacteria", "__Alphaproteobacteria", "__Rhodobacterales",
"__Rhodobacteraceae"]}},{"id": "denovo3445", "metadata": {"taxonomy":
["Bacteria", "__Firmicutes", "__Clostridia", "__Clostridiales",
"__Family_XVI_Incertae_Sedis", "__Carboxydocella"]}},{"id":
"denovo3446", "metadata": {"taxonomy": ["Bacteria",
"__Proteobacteria", "__Alphaproteobacteria", "__Sphingomonadales",
"__ctg-CGOF202", "__g"]}},{"id": "denovo3447", "metadata":
{"taxonomy": ["Bacteria", "__Proteobacteria", "__Gammaproteobacteria",
"__Oceanospirillales", "__Hahellaceae", "__Endozoicomonas"]}},{"id":
"denovo3449", "metadata": {"taxonomy": ["Bacteria",
"__Proteobacteria", "__Deltaproteobacteria", "__Sh765B-TzT-29", "__f",
"__g"]}},{"id": "denovo3454", "metadata": {"taxonomy": ["Bacteria",
"__Proteobacteria", "__Alphaproteobacteria", "__Rhodobacterales",
"__Rhodobacteraceae"]}},{"id": "denovo3455", "metadata": {"taxonomy":
["Bacteria", "__Actinobacteria", "__Acidimicrobiia",
"__Acidimicrobiales", "__uncultured", "__g"]}},{"id": "denovo3456",
"metadata": {"taxonomy": ["Bacteria", "__Proteobacteria",
"__Gammaproteobacteria"]}},{"id": "denovo3459", "metadata":
{"taxonomy": ["Bacteria", "__Proteobacteria", "__Alphaproteobacteria",
"__Rhodobacterales", "__Rhodobacteraceae", "__g"]}},{"id":
"denovo3460", "metadata": {"taxonomy": ["Bacteria",
"__Verrucomicrobia"]}},{"id": "denovo3463", "metadata": {"taxonomy":
["Bacteria", "__Proteobacteria", "__Alphaproteobacteria",
"__Rhizobiales", "__Phyllobacteriaceae", "__g"]}},{"id": "denovo3466",
"metadata": {"taxonomy": ["Bacteria", "__Proteobacteria",
"__Gammaproteobacteria"]}},{"id": "denovo3467", "metadata":
{"taxonomy": ["Bacteria", "__Chloroflexi", "__Caldilineae",
"__Caldilineales", "__Caldilineaceae", "__g"]}},{"id": "denovo3468",
"metadata": {"taxonomy": ["Bacteria", "__Chloroflexi",
"__Caldilineae", "__Caldilineales", "__Caldilineaceae",
"__Caldilinea"]}},{"id": "denovo3469", "metadata": {"taxonomy":
["Bacteria", "__Deferribacteres", "__Deferribacteres",
"__Deferribacterales", "__SAR406_clade(Marine_group_A)", "__g"]}},
{"id": "denovo3471", "metadata": {"taxonomy": ["Bacteria",
"__Bacteroidetes", "__Flavobacteria", "__Flavobacteriales",
"__Flavobacteriaceae"]}},{"id": "denovo3473", "metadata": {"taxonomy":
["Bacteria", "__Cyanobacteria", "__SHA-109", "__o", "__f", "__g"]}},

{"id": "denovo3481", "metadata": {"taxonomy": ["Bacteria", "__Nitrospirae", "__Nitrospira", "__Nitrospirales", "__Nitrospiraceae", "__Nitrospira"]}},{"id": "denovo3484", "metadata": {"taxonomy": ["Bacteria", "__Proteobacteria", "__Alphaproteobacteria", "__Rhodospirillales", "__Rhodospirillaceae", "__g"]}},{"id": "denovo3485", "metadata": {"taxonomy": ["Bacteria", "__Proteobacteria", "__Alphaproteobacteria", "__Rhodobacterales", "__Rhodobacteraceae", "__Rhodovulum"]}},{"id": "denovo3490", "metadata": {"taxonomy": ["Bacteria", "__Proteobacteria", "__Deltaproteobacteria", "__Sh765B-TzT-29", "__f", "__g"]}},{"id": "denovo3497", "metadata": {"taxonomy": ["Bacteria", "__Proteobacteria", "__Gammaproteobacteria"]}},{"id": "denovo3498", "metadata": {"taxonomy": ["Bacteria", "__Proteobacteria", "__Alphaproteobacteria", "__Rhodobacterales", "__Rhodobacteraceae", "__g"]}},{"id": "denovo3499", "metadata": {"taxonomy": ["Bacteria", "__Proteobacteria", "__Alphaproteobacteria", "__Kordiimonadales", "__Kordiimonadaceae", "__Kordiimonas"]}},{"id": "denovo3503", "metadata": {"taxonomy": ["Bacteria", "__Bacteroidetes", "__Cytophagia", "__Cytophagales", "__Flammeovirgaceae", "__g"]}},{"id": "denovo3504", "metadata": {"taxonomy": ["Bacteria", "__Chlamydiae", "__Chlamydiae", "__Chlamydiales"]}},{"id": "denovo3507", "metadata": {"taxonomy": ["Bacteria", "__Spirochaetes", "__Spirochaetes", "__Spirochaetales", "__Leptospiraceae", "__Turneriella"]}},{"id": "denovo3510", "metadata": {"taxonomy": ["Bacteria", "__Candidate_division_OD1", "__c", "__o", "__f", "__g"]}},{"id": "denovo3511", "metadata": {"taxonomy": ["Bacteria", "__Cyanobacteria", "__Cyanobacteria", "__SubsectionII", "__FamilyII", "__Pleurocapsa"]}},{"id": "denovo3513", "metadata": {"taxonomy": ["Bacteria", "__Proteobacteria", "__Alphaproteobacteria", "__Rhodospirillales", "__Rhodospirillaceae", "__g"]}},{"id": "denovo3515", "metadata": {"taxonomy": ["Bacteria", "__Cyanobacteria", "__4C0d-2", "__o", "__f", "__g"]}},{"id": "denovo3516", "metadata": {"taxonomy": ["Bacteria", "__Proteobacteria", "__Alphaproteobacteria", "__Parvularculales", "__Parvularculaceae", "__Parvularcula"]}},{"id": "denovo3517", "metadata": {"taxonomy": ["Bacteria", "__Proteobacteria", "__Gammaproteobacteria", "__KI89A_clade", "__f", "__g"]}},{"id": "denovo3518", "metadata": {"taxonomy": ["Bacteria", "__Candidate_division_BRC1", "__c", "__o", "__f", "__g"]}},{"id": "denovo3519", "metadata": {"taxonomy": ["Bacteria", "__Proteobacteria", "__Epsilonproteobacteria"]}},{"id": "denovo3520", "metadata": {"taxonomy": ["Bacteria", "__Proteobacteria", "__Alphaproteobacteria", "__Rhodobacterales", "__Rhodobacteraceae", "__Nereida"]}},{"id": "denovo3521", "metadata": {"taxonomy": ["Bacteria", "__Bacteroidetes", "__Flavobacteria", "__Flavobacteriales", "__Flavobacteriaceae", "__Tenacibaculum"]}},{"id": "denovo3523", "metadata": {"taxonomy": ["Bacteria", "__Firmicutes", "__Clostridia", "__Clostridiales", "__Lachnospiraceae"]}},{"id": "denovo3526", "metadata": {"taxonomy": ["Bacteria", "__Proteobacteria", "__Alphaproteobacteria", "__Rhodospirillales", "__Rhodospirillaceae", "__g"]}},{"id":

"denovo3531", "metadata": {"taxonomy": ["Bacteria", "__Proteobacteria", "__Deltaproteobacteria", "__Sh765B-TzT-29", "__f", "__g"]}},{"id": "denovo3535", "metadata": {"taxonomy": ["Bacteria"]}}, {"id": "denovo3538", "metadata": {"taxonomy": ["Bacteria", "__Proteobacteria", "__Gammaproteobacteria", "__Oceanospirillales", "__Hahellaceae", "__Endozoicomonas"]}},{"id": "denovo3539", "metadata": {"taxonomy": ["Bacteria", "__Proteobacteria", "__Alphaproteobacteria", "__Rhodobacterales", "__Rhodobacteraceae", "__Labrenzia"]}},{"id": "denovo3541", "metadata": {"taxonomy": ["Bacteria", "__Proteobacteria", "__Alphaproteobacteria", "__Rhizobiales", "__Brucellaceae", "__g"]}},{"id": "denovo3542", "metadata": {"taxonomy": ["Bacteria", "__Proteobacteria", "__Alphaproteobacteria", "__Rhodobacterales", "__Rhodobacteraceae"]}}, {"id": "denovo3545", "metadata": {"taxonomy": ["Bacteria", "__Proteobacteria", "__Gammaproteobacteria", "__HOC36", "__f", "__g"]}},{"id": "denovo3547", "metadata": {"taxonomy": ["Bacteria", "__Chloroflexi", "__Chloroflexi", "__Chloroflexales", "__Candidatus_Chlorothrix", "__g"]}},{"id": "denovo3549", "metadata": {"taxonomy": ["Bacteria", "__Proteobacteria", "__Alphaproteobacteria", "__Rhizobiales"]}},{"id": "denovo3551", "metadata": {"taxonomy": ["Bacteria", "__Acidobacteria", "__Holophagae", "__Sva0725", "__f", "__g"]}},{"id": "denovo3555", "metadata": {"taxonomy": ["Bacteria", "__Candidate_division_WS3", "__c", "__o", "__f", "__g"]}},{"id": "denovo3556", "metadata": {"taxonomy": ["Bacteria"]}},{"id": "denovo3557", "metadata": {"taxonomy": ["Bacteria", "__Fusobacteria", "__Fusobacteria", "__Fusobacteriales", "__Fusobacteriaceae", "__Propionigenium"]}},{"id": "denovo3558", "metadata": {"taxonomy": ["Bacteria", "__Bacteroidetes", "__Sphingobacteriia", "__Sphingobacteriales", "__Saprospiraceae", "__g"]}},{"id": "denovo3560", "metadata": {"taxonomy": ["Bacteria", "__Proteobacteria", "__Deltaproteobacteria"]}},{"id": "denovo3561", "metadata": {"taxonomy": ["Bacteria", "__Proteobacteria", "__Deltaproteobacteria", "__Desulfuromonadales", "__GR-WP33-58", "__g"]}},{"id": "denovo3563", "metadata": {"taxonomy": ["Bacteria", "__Proteobacteria", "__Alphaproteobacteria", "__Rhodospirillales", "__Rhodospirillaceae", "__g"]}},{"id": "denovo3566", "metadata": {"taxonomy": ["Bacteria", "__Proteobacteria", "__Gammaproteobacteria", "__SS1-B-09-64", "__f", "__g"]}},{"id": "denovo3574", "metadata": {"taxonomy": ["Bacteria", "__Verrucomicrobia", "__Candidatus_Methylacidiphilum", "__o", "__f", "__g"]}},{"id": "denovo3575", "metadata": {"taxonomy": ["Bacteria", "__Proteobacteria", "__Gammaproteobacteria", "__Alteromonadales", "__Alteromonadaceae", "__Simiduia"]}},{"id": "denovo3576", "metadata": {"taxonomy": ["Bacteria", "__Planctomycetes", "__Phycisphaerae", "__Phycisphaerales", "__Phycisphaeraceae", "__Phycisphaera"]}},{"id": "denovo3579", "metadata": {"taxonomy": ["Bacteria", "__Firmicutes", "__Clostridia", "__Halanaerobiales", "__64K2", "__g"]}},{"id": "denovo3584", "metadata": {"taxonomy": ["Bacteria", "__Proteobacteria", "__Alphaproteobacteria", "__Rickettsiales", "__SM2D12", "__g"]}},{"id": "denovo3586", "metadata": {"taxonomy":

["Bacteria", "__Proteobacteria", "__Deltaproteobacteria", "__Sh765B-TzT-29", "__f", "__g"]}},{"id": "denovo3587", "metadata": {"taxonomy": ["Bacteria", "__Proteobacteria", "__Deltaproteobacteria", "__SAR324_clade(Marine_group_B)", "__f", "__g"]}},{"id": "denovo3588", "metadata": {"taxonomy": ["Bacteria", "__Bacteroidetes", "__Sphingobacteriia", "__Sphingobacteriales", "__Saprospiraceae", "__g"]}},{"id": "denovo3590", "metadata": {"taxonomy": ["Bacteria", "__Proteobacteria", "__Gammaproteobacteria", "__Oceanospirillales", "__CrystalBog021C3", "__g"]}},{"id": "denovo3592", "metadata": {"taxonomy": ["Bacteria", "__Acidobacteria", "__Holophagae", "__CA002", "__f", "__g"]}},{"id": "denovo3593", "metadata": {"taxonomy": ["Bacteria", "__Planctomycetes", "__Pla3_lineage", "__o", "__f", "__g"]}},{"id": "denovo3596", "metadata": {"taxonomy": ["Bacteria", "__Chlorobi", "__Ignavibacteria", "__Ignavibacteriales", "__IheB3-7", "__g"]}},{"id": "denovo3599", "metadata": {"taxonomy": ["Bacteria", "__Proteobacteria", "__Gammaproteobacteria", "__Order_Incertae_Sedis", "__Family_Incertae_Sedis", "__Arenicella"]}},{"id": "denovo3601", "metadata": {"taxonomy": ["Bacteria", "__Proteobacteria", "__Alphaproteobacteria", "__Rhodobacterales", "__Rhodobacteraceae"]}},{"id": "denovo3604", "metadata": {"taxonomy": ["Bacteria", "__Bacteroidetes", "__Flavobacteria", "__Flavobacteriales", "__Cryomorphaceae", "__Owenweeksia"]}},{"id": "denovo3607", "metadata": {"taxonomy": ["Bacteria", "__Proteobacteria", "__Gammaproteobacteria", "__Alteromonadales", "__Alteromonadaceae", "__BD1-7_clade"]}},{"id": "denovo3615", "metadata": {"taxonomy": ["Bacteria", "__Proteobacteria", "__Deltaproteobacteria", "__Myxococcales", "__Sorangiineae", "__Sandaracinaceae"]}},{"id": "denovo3617", "metadata": {"taxonomy": ["Bacteria", "__Proteobacteria", "__Alphaproteobacteria", "__Kordiimonadales", "__Kordiimonadaceae", "__Kordiimonas"]}},{"id": "denovo3618", "metadata": {"taxonomy": ["Bacteria", "__Bacteroidetes"]}},{"id": "denovo3622", "metadata": {"taxonomy": ["Bacteria", "__Proteobacteria", "__Alphaproteobacteria", "__Rhodospirillales", "__Rhodospirillaceae", "__Pelagibius"]}},{"id": "denovo3623", "metadata": {"taxonomy": ["Bacteria"]}},{"id": "denovo3626", "metadata": {"taxonomy": ["Bacteria", "__Bacteroidetes", "__Flavobacteria", "__Flavobacteriales", "__Flavobacteriaceae", "__NS4_marine_group"]}},{"id": "denovo3627", "metadata": {"taxonomy": ["Bacteria", "__Proteobacteria", "__Deltaproteobacteria", "__Desulfuromonadales", "__GR-WP33-58", "__g"]}},{"id": "denovo3630", "metadata": {"taxonomy": ["Bacteria", "__Cyanobacteria", "__Cyanobacteria", "__SubsectionIII", "__FamilyI", "__Phormidium"]}},{"id": "denovo3633", "metadata": {"taxonomy": ["Bacteria", "__Proteobacteria", "__Gammaproteobacteria", "__Alteromonadales", "__Alteromonadaceae", "__Haliea"]}},{"id": "denovo3636", "metadata": {"taxonomy": ["Archaea", "__Euryarchaeota", "__Halobacteria", "__Halobacteriales", "__Deep_Sea_Hydrothermal_Vent_Gp_6(DHVEG-6)", "__g"]}},{"id": "denovo3642", "metadata": {"taxonomy": ["Bacteria", "__Spirochaetes", "__Spirochaetes", "__Spirochaetales", "__Leptospiraceae", "__g"]}},{"id": "denovo3645", "metadata":

{"taxonomy": ["Unclassified"]}},{"id": "denovo3652", "metadata":
{"taxonomy": ["Bacteria", "__Proteobacteria",
"__Gammaproteobacteria"]}},{"id": "denovo3653", "metadata":
{"taxonomy": ["Bacteria", "__Bacteroidetes", "__Flavobacteria",
"__Flavobacteriales", "__Flavobacteriaceae"]}},{"id": "denovo3657",
"metadata": {"taxonomy": ["Bacteria", "__Proteobacteria",
"__Gammaproteobacteria", "__Oceanospirillales", "__Hahellaceae",
"__Endozoicomonas"]}},{"id": "denovo3659", "metadata": {"taxonomy":
["Bacteria", "__Proteobacteria", "__Gammaproteobacteria",
"__Alteromonadales", "__Alteromonadaceae"]}},{"id": "denovo3663",
"metadata": {"taxonomy": ["Bacteria", "__Proteobacteria",
"__Alphaproteobacteria", "__Rhodobacterales", "__Rhodobacteraceae",
"__Tropicimonas"]}},{"id": "denovo3664", "metadata": {"taxonomy":
["Bacteria", "__Proteobacteria", "__Alphaproteobacteria", "__DB1-14",
"__f", "__g"]}},{"id": "denovo3667", "metadata": {"taxonomy":
["Bacteria", "__Proteobacteria", "__Alphaproteobacteria", "__DB1-14",
"__f", "__g"]}},{"id": "denovo3668", "metadata": {"taxonomy":
["Bacteria", "__Proteobacteria", "__Alphaproteobacteria",
"__Rhodobacterales", "__Rhodobacteraceae", "__g"]}},{"id":
"denovo3669", "metadata": {"taxonomy": ["Bacteria",
"__Proteobacteria", "__Alphaproteobacteria", "__Rhodospirillales",
"__Family_Incertae_Sedis", "__Geminicoccus"]}},{"id": "denovo3671",
"metadata": {"taxonomy": ["Bacteria", "__Firmicutes", "__Clostridia",
"__Clostridiales"]}},{"id": "denovo3673", "metadata": {"taxonomy":
["Bacteria", "__Proteobacteria", "__Gammaproteobacteria",
"__Oceanospirillales", "__Hahellaceae", "__Endozoicomonas"]}},{"id":
"denovo3680", "metadata": {"taxonomy": ["Bacteria",
"__Proteobacteria", "__Alphaproteobacteria", "__Rhodospirillales"]}},
{"id": "denovo3681", "metadata": {"taxonomy": ["Bacteria",
"__Cyanobacteria", "__Cyanobacteria", "__SubsectionI", "__FamilyI",
"__Cyanobacterium"]}},{"id": "denovo3687", "metadata": {"taxonomy":
["Bacteria", "__Proteobacteria", "__Gammaproteobacteria",
"__Enterobacteriales", "__Enterobacteriaceae", "__Escherichia-
Shigella"]}},{"id": "denovo3689", "metadata": {"taxonomy":
["Bacteria", "__Chloroflexi", "__SAR202_clade", "__o", "__f",
"__g"]}},{"id": "denovo3692", "metadata": {"taxonomy": ["Bacteria",
"__Proteobacteria", "__Gammaproteobacteria", "__Alteromonadales",
"__Alteromonadaceae", "__Aestuariibacter"]}},{"id": "denovo3694",
"metadata": {"taxonomy": ["Bacteria", "__Proteobacteria",
"__Alphaproteobacteria", "__Rhodobacterales", "__Rhodobacteraceae"]}},
{"id": "denovo3698", "metadata": {"taxonomy": ["Bacteria",
"__Bacteroidetes", "__Class_Incertae_Sedis", "__Order_Incertae_Sedis",
"__Family_Incertae_Sedis", "__Prolixibacter"]}},{"id": "denovo3703",
"metadata": {"taxonomy": ["Bacteria", "__Acidobacteria", "__RB25",
"__o", "__f", "__g"]}},{"id": "denovo3706", "metadata": {"taxonomy":
["Bacteria", "__Proteobacteria", "__Gammaproteobacteria",
"__Oceanospirillales", "__OM182_clade", "__g"]}},{"id": "denovo3707",
"metadata": {"taxonomy": ["Bacteria", "__Bacteroidetes",
"__Flavobacteria", "__Flavobacteriales", "__Flavobacteriaceae",
"__Polaribacter"]}},{"id": "denovo3708", "metadata": {"taxonomy":

["Bacteria", "__Proteobacteria", "__Alphaproteobacteria", "__Rhodobacterales", "__Rhodobacteraceae"]}},{"id": "denovo3711", "metadata": {"taxonomy": ["Bacteria", "__Proteobacteria", "__Alphaproteobacteria", "__Rhizobiales"]}},{"id": "denovo3715", "metadata": {"taxonomy": ["Bacteria", "__Proteobacteria", "__Alphaproteobacteria", "__Sphingomonadales", "__f", "__g"]}},{"id": "denovo3718", "metadata": {"taxonomy": ["Bacteria", "__Acidobacteria", "__Holophagae", "__Sva0725", "__f", "__g"]}},{"id": "denovo3729", "metadata": {"taxonomy": ["Bacteria", "__Proteobacteria", "__Alphaproteobacteria", "__Rickettsiales", "__Rickettsiaceae", "__Candidatus_Cryptoprodotis"]}},{"id": "denovo3730", "metadata": {"taxonomy": ["Bacteria", "__Proteobacteria", "__Deltaproteobacteria", "__Myxococcales", "__0319-6G20", "__g"]}},{"id": "denovo3731", "metadata": {"taxonomy": ["Bacteria", "__Proteobacteria", "__Gammaproteobacteria", "__Alteromonadales", "__Alteromonadaceae", "__OM60(NOR5)_clade"]}},{"id": "denovo3732", "metadata": {"taxonomy": ["Bacteria", "__Actinobacteria", "__Actinobacteria", "__Corynebacteriales", "__Corynebacteriaceae", "__Corynebacterium"]}},{"id": "denovo3735", "metadata": {"taxonomy": ["Bacteria", "__Candidate_division_WS3", "__c", "__o", "__f", "__g"]}},{"id": "denovo3739", "metadata": {"taxonomy": ["Bacteria", "__Proteobacteria", "__Gammaproteobacteria", "__Xanthomonadales", "__Sinobacteraceae", "__JTB255_marine_benthic_group"]}},{"id": "denovo3741", "metadata": {"taxonomy": ["Bacteria", "__Bacteroidetes"]}},{"id": "denovo3743", "metadata": {"taxonomy": ["Bacteria", "__Proteobacteria", "__Gammaproteobacteria", "__Xanthomonadales", "__Sinobacteraceae", "__JTB255_marine_benthic_group"]}},{"id": "denovo3745", "metadata": {"taxonomy": ["Bacteria", "__Proteobacteria", "__Deltaproteobacteria", "__Desulfobacterales", "__Desulfobulbaceae", "__Desulfobulbus"]}},{"id": "denovo3749", "metadata": {"taxonomy": ["Bacteria", "__Proteobacteria", "__Alphaproteobacteria", "__Rhodobacterales", "__Rhodobacteraceae"]}},{"id": "denovo3750", "metadata": {"taxonomy": ["Bacteria", "__Bacteroidetes", "__Flavobacteria", "__Flavobacteriales", "__Flavobacteriaceae", "__Tenacibaculum"]}},{"id": "denovo3762", "metadata": {"taxonomy": ["Bacteria", "__Proteobacteria", "__Gammaproteobacteria", "__Enterobacteriales", "__Enterobacteriaceae", "__Shimwellia"]}},{"id": "denovo3764", "metadata": {"taxonomy": ["Bacteria", "__Proteobacteria", "__Gammaproteobacteria", "__BD7-8_marine_group", "__f", "__g"]}},{"id": "denovo3765", "metadata": {"taxonomy": ["Bacteria", "__Proteobacteria", "__Alphaproteobacteria", "__Rhizobiales", "__Phyllobacteriaceae", "__Hoeflea"]}},{"id": "denovo3773", "metadata": {"taxonomy": ["Bacteria", "__Proteobacteria", "__Deltaproteobacteria", "__Desulfobacterales", "__Nitrospinaceae", "__Nitrospina"]}},{"id": "denovo3775", "metadata": {"taxonomy": ["Bacteria", "__Bacteroidetes", "__Cytophagia", "__Cytophagales", "__Flammeovirgaceae", "__g"]}},{"id": "denovo3781", "metadata": {"taxonomy": ["Bacteria", "__Proteobacteria", "__Betaproteobacteria", "__Nitrosomonadales", "__Gallionellaceae",

"__Candidatus_Nitrotoga"]}},{"id": "denovo3782", "metadata": {"taxonomy": ["Bacteria", "__Proteobacteria", "__Deltaproteobacteria", "__Desulfobacterales", "__Desulfobacteraceae"]}},{"id": "denovo3786", "metadata": {"taxonomy": ["Bacteria", "__Fusobacteria", "__Fusobacteria", "__Fusobacteriales", "__Fusobacteriaceae", "__Propionigenium"]}},{"id": "denovo3788", "metadata": {"taxonomy": ["Bacteria", "__Proteobacteria", "__Deltaproteobacteria", "__Myxococcales", "__Haliangiaceae", "__Haliangium"]}},{"id": "denovo3791", "metadata": {"taxonomy": ["Bacteria", "__Proteobacteria", "__Alphaproteobacteria", "__OCS116_clade", "__f", "__g"]}},{"id": "denovo3792", "metadata": {"taxonomy": ["Bacteria", "__Spirochaetes", "__Spirochaetes", "__Spirochaetales", "__Leptospiraceae", "__Turneriella"]}},{"id": "denovo3793", "metadata": {"taxonomy": ["Bacteria", "__Proteobacteria", "__Gammaproteobacteria", "__Xanthomonadales", "__Sinobacteraceae", "__JTB255_marine_benthic_group"]}},{"id": "denovo3794", "metadata": {"taxonomy": ["Bacteria", "__Proteobacteria", "__Alphaproteobacteria", "__Rhizobiales", "__Methylobacteriaceae", "__Methylobacterium"]}},{"id": "denovo3795", "metadata": {"taxonomy": ["Bacteria", "__Proteobacteria", "__Gammaproteobacteria", "__Pseudomonadales", "__Moraxellaceae", "__Acinetobacter"]}},{"id": "denovo3796", "metadata": {"taxonomy": ["Bacteria", "__Proteobacteria", "__Gammaproteobacteria", "__Oceanospirillales", "__Oceanospirillaceae", "__Pseudospirillum"]}},{"id": "denovo3800", "metadata": {"taxonomy": ["Bacteria", "__Proteobacteria", "__Deltaproteobacteria", "__Myxococcales", "__Nannocystaceae"]}},{"id": "denovo3806", "metadata": {"taxonomy": ["Bacteria", "__Proteobacteria", "__Alphaproteobacteria", "__Rhodospirillales", "__Rhodospirillaceae", "__Skermanella"]}},{"id": "denovo3809", "metadata": {"taxonomy": ["Bacteria", "__Proteobacteria", "__Alphaproteobacteria", "__Rhodobacterales", "__Rhodobacteraceae", "__Salinihabitans"]}},{"id": "denovo3810", "metadata": {"taxonomy": ["Bacteria", "__Proteobacteria", "__Alphaproteobacteria", "__Rhodospirillales", "__Rhodospirillaceae"]}},{"id": "denovo3816", "metadata": {"taxonomy": ["Bacteria", "__Proteobacteria", "__Gammaproteobacteria", "__KI89A_clade", "__f", "__g"]}},{"id": "denovo3820", "metadata": {"taxonomy": ["Bacteria", "__Spirochaetes", "__Spirochaetes", "__Spirochaetales", "__Leptospiraceae", "__g"]}},{"id": "denovo3821", "metadata": {"taxonomy": ["Bacteria", "__Bacteroidetes", "__Sphingobacteriia", "__Sphingobacteriales", "__Saprospiraceae", "__g"]}},{"id": "denovo3822", "metadata": {"taxonomy": ["Bacteria"]}},{"id": "denovo3826", "metadata": {"taxonomy": ["Bacteria", "__Proteobacteria", "__Gammaproteobacteria", "__Pseudomonadales", "__Moraxellaceae", "__Acinetobacter"]}},{"id": "denovo3829", "metadata": {"taxonomy": ["Bacteria", "__Proteobacteria", "__Alphaproteobacteria", "__Rhodobacterales", "__Rhodobacteraceae", "__Maritimibacter"]}},{"id": "denovo3831", "metadata": {"taxonomy": ["Bacteria", "__Bacteroidetes", "__Flavobacteria", "__Flavobacteriales", "__Flavobacteriaceae", "__Polaribacter"]}},{"id": "denovo3832", "metadata": {"taxonomy":

["Bacteria", "__Cyanobacteria", "__Cyanobacteria"]}},{"id": "denovo3834", "metadata": {"taxonomy": ["Bacteria", "__Firmicutes", "__Clostridia", "__Clostridiales", "__Peptococcaceae", "__g"]}},{"id": "denovo3835", "metadata": {"taxonomy": ["Bacteria", "__Proteobacteria", "__SPOTSOCT00m83", "__o", "__f", "__g"]}},{"id": "denovo3842", "metadata": {"taxonomy": ["Bacteria", "__Proteobacteria", "__Alphaproteobacteria", "__Rhodobacterales", "__Rhodobacteraceae", "__Loktanella"]}},{"id": "denovo3844", "metadata": {"taxonomy": ["Bacteria", "__Proteobacteria", "__Gammaproteobacteria", "__Pseudomonadales", "__Moraxellaceae", "__Acinetobacter"]}},{"id": "denovo3845", "metadata": {"taxonomy": ["Bacteria", "__Proteobacteria", "__Gammaproteobacteria", "__Sva0071", "__f", "__g"]}},{"id": "denovo3846", "metadata": {"taxonomy": ["Bacteria", "__Cyanobacteria", "__Cyanobacteria", "__SubsectionI", "__FamilyI", "__g"]}},{"id": "denovo3851", "metadata": {"taxonomy": ["Bacteria", "__Proteobacteria", "__Alphaproteobacteria", "__Caulobacterales", "__Hyphomonadaceae", "__g"]}},{"id": "denovo3856", "metadata": {"taxonomy": ["Bacteria", "__Proteobacteria", "__Alphaproteobacteria", "__Rhodobacterales", "__Rhodobacteraceae", "__g"]}},{"id": "denovo3857", "metadata": {"taxonomy": ["Bacteria", "__Proteobacteria", "__Alphaproteobacteria", "__Caulobacterales", "__Hyphomonadaceae", "__Hyphomonas"]}},{"id": "denovo3858", "metadata": {"taxonomy": ["Archaea", "__Thaumarchaeota", "__Marine_Group_I", "__Candidatus_Nitrosopumilus", "__f", "__g"]}},{"id": "denovo3859", "metadata": {"taxonomy": ["Bacteria", "__Proteobacteria", "__Gammaproteobacteria"]}},{"id": "denovo3865", "metadata": {"taxonomy": ["Bacteria", "__Proteobacteria", "__Gammaproteobacteria", "__Chromatiales"]}},{"id": "denovo3866", "metadata": {"taxonomy": ["Bacteria", "__Proteobacteria", "__Alphaproteobacteria", "__Sphingomonadales", "__Sphingomonadaceae", "__Sphingomonas"]}},{"id": "denovo3867", "metadata": {"taxonomy": ["Bacteria", "__Proteobacteria", "__Gammaproteobacteria"]}},{"id": "denovo3868", "metadata": {"taxonomy": ["Bacteria", "__Actinobacteria", "__Acidimicrobiia", "__Acidimicrobiales", "__Sva0996_marine_group", "__g"]}},{"id": "denovo3869", "metadata": {"taxonomy": ["Bacteria", "__Proteobacteria", "__Alphaproteobacteria"]}},{"id": "denovo3870", "metadata": {"taxonomy": ["Bacteria", "__Proteobacteria", "__Gammaproteobacteria", "__Alteromonadales", "__Alteromonadaceae"]}},{"id": "denovo3872", "metadata": {"taxonomy": ["Bacteria", "__Firmicutes", "__Clostridia", "__Clostridiales", "__Lachnospiraceae"]}},{"id": "denovo3873", "metadata": {"taxonomy": ["Bacteria", "__Bacteroidetes", "__Cytophagia", "__Cytophagales", "__Flammeovirgaceae", "__Ekhidna"]}},{"id": "denovo3874", "metadata": {"taxonomy": ["Bacteria", "__Proteobacteria", "__Alphaproteobacteria", "__Rhodobacterales", "__Rhodobacteraceae"]}},{"id": "denovo3875", "metadata": {"taxonomy": ["Bacteria", "__Proteobacteria", "__Gammaproteobacteria", "__Alteromonadales", "__Alteromonadaceae", "__Haliea"]}},{"id": "denovo3878", "metadata": {"taxonomy": ["Bacteria", "__Proteobacteria", "__Gammaproteobacteria",

"__Order_Incertae_Sedis", "__Family_Incertae_Sedis",
"__Sedimenticola"]}},{"id": "denovo3879", "metadata": {"taxonomy":
["Bacteria", "__Planctomycetes", "__Planctomycetacia",
"__Planctomycetales", "__Planctomycetaceae", "__Blastopirellula"]}},
{"id": "denovo3885", "metadata": {"taxonomy": ["Bacteria",
"__Proteobacteria", "__Gammaproteobacteria", "__Chromatiales",
"__Chromatiaceae", "__Nitrosococcus"]}},{"id": "denovo3886",
"metadata": {"taxonomy": ["Bacteria", "__Acidobacteria",
"__Holophagae", "__NS72", "__f", "__g"]}},{"id": "denovo3891",
"metadata": {"taxonomy": ["Bacteria", "__Proteobacteria",
"__Betaproteobacteria", "__Burkholderiales", "__Comamonadaceae",
"__Comamonas"]}},{"id": "denovo3900", "metadata": {"taxonomy":
["Bacteria", "__Proteobacteria", "__Alphaproteobacteria",
"__Rhizobiales", "__Rhodobiaceae", "__Anderseniella"]}},{"id":
"denovo3902", "metadata": {"taxonomy": ["Archaea", "__Euryarchaeota",
"__Thermoplasmata", "__Thermoplasmatales", "__Marine_Group_II",
"__g"]}},{"id": "denovo3907", "metadata": {"taxonomy": ["Bacteria",
"__Proteobacteria", "__Alphaproteobacteria", "__Caulobacterales",
"__Hyphomonadaceae", "__g"]}},{"id": "denovo3909", "metadata":
{"taxonomy": ["Bacteria", "__Proteobacteria", "__SPOTSOCT00m83",
"__o", "__f", "__g"]}},{"id": "denovo3912", "metadata": {"taxonomy":
["Bacteria", "__Proteobacteria", "__Alphaproteobacteria",
"__Rhodospirillales", "__Rhodospirillaceae"]}},{"id": "denovo3913",
"metadata": {"taxonomy": ["Bacteria", "__Proteobacteria",
"__Alphaproteobacteria", "__Rhodobacterales", "__Rhodobacteraceae",
"__Loktanella"]}},{"id": "denovo3914", "metadata": {"taxonomy":
["Bacteria", "__Proteobacteria", "__Alphaproteobacteria",
"__Kordiimonadales", "__Kordiimonadaceae", "__Kordiimonas"]}},{"id":
"denovo3915", "metadata": {"taxonomy": ["Bacteria",
"__Proteobacteria", "__Deltaproteobacteria", "__Desulfobacterales",
"__Desulfobulbaceae", "__Desulfobulbus"]}},{"id": "denovo3916",
"metadata": {"taxonomy": ["Bacteria", "__Proteobacteria",
"__Gammaproteobacteria", "__Alteromonadales", "__Shewanellaceae",
"__Shewanella"]}},{"id": "denovo3917", "metadata": {"taxonomy":
["Bacteria", "__Proteobacteria", "__Deltaproteobacteria",
"__Myxococcales", "__MSB-4B10", "__g"]}},{"id": "denovo3919",
"metadata": {"taxonomy": ["Bacteria", "__Spirochaetes",
"__Spirochaetes", "__Spirochaetales", "__Spirochaetaceae",
"__Spirochaeta"]}},{"id": "denovo3920", "metadata": {"taxonomy":
["Bacteria", "__Acidobacteria", "__Holophagae",
"__Acanthopleuribacterales", "__Acanthopleuribacteraceae",
"__Acanthopleuribacter"]}},{"id": "denovo3922", "metadata":
{"taxonomy": ["Bacteria", "__Proteobacteria", "__Deltaproteobacteria",
"__Sh765B-TzT-29", "__f", "__g"]}},{"id": "denovo3926", "metadata":
{"taxonomy": ["Bacteria", "__Proteobacteria", "__Gammaproteobacteria",
"__Order_Incertae_Sedis", "__Family_Incertae_Sedis",
"__Arenicella"]}},{"id": "denovo3927", "metadata": {"taxonomy":
["Bacteria", "__Proteobacteria", "__Gammaproteobacteria",
"__Alteromonadales", "__Alteromonadaceae", "__BD1-7_clade"]}},{"id":
"denovo3928", "metadata": {"taxonomy": ["Bacteria",

"__Proteobacteria", "__Alphaproteobacteria", "__Rhodobacterales", "__Rhodobacteraceae"]}},{"id": "denovo3929", "metadata": {"taxonomy": ["Bacteria", "__Proteobacteria", "__TA18", "__o", "__f", "__g"]}}, {"id": "denovo3937", "metadata": {"taxonomy": ["Bacteria", "__Proteobacteria", "__Deltaproteobacteria", "__Bdellovibrionales", "__Bdellovibrionaceae", "__Bdellovibrio"]}},{"id": "denovo3938", "metadata": {"taxonomy": ["Bacteria", "__Spirochaetes", "__Spirochaetes", "__Spirochaetales", "__Spirochaetaceae", "__Spirochaeta"]}},{"id": "denovo3939", "metadata": {"taxonomy": ["Bacteria", "__Bacteroidetes", "__Flavobacteria", "__Flavobacteriales", "__Flavobacteriaceae", "__Croceitalea"]}},{"id": "denovo3940", "metadata": {"taxonomy": ["Bacteria", "__Bacteroidetes", "__Flavobacteria", "__Flavobacteriales", "__Cryomorphaceae", "__Owenweeksia"]}},{"id": "denovo3942", "metadata": {"taxonomy": ["Bacteria", "__Proteobacteria", "__Gammaproteobacteria", "__Oceanospirillales", "__CrystalBog021C3", "__g"]}},{"id": "denovo3943", "metadata": {"taxonomy": ["Bacteria", "__Proteobacteria", "__Gammaproteobacteria", "__Alteromonadales", "__Pseudoalteromonadaceae", "__g"]}},{"id": "denovo3945", "metadata": {"taxonomy": ["Bacteria", "__Proteobacteria", "__Alphaproteobacteria", "__Rickettsiales"]}},{"id": "denovo3946", "metadata": {"taxonomy": ["Bacteria", "__Proteobacteria", "__Gammaproteobacteria", "__Enterobacteriales", "__Enterobacteriaceae", "__Escherichia-Shigella"]}},{"id": "denovo3948", "metadata": {"taxonomy": ["Bacteria", "__Bacteroidetes", "__Cytophagia", "__Cytophagales", "__Flammeovirgaceae"]}},{"id": "denovo3949", "metadata": {"taxonomy": ["Bacteria", "__Deferribacteres", "__Deferribacteres", "__Deferribacterales", "__SAR406_clade(Marine_group_A)", "__g"]}}, {"id": "denovo3950", "metadata": {"taxonomy": ["Bacteria", "__Chlorobi", "__Chlorobia", "__Chlorobiales", "__Chlorobiaceae", "__Prosthecochloris"]}},{"id": "denovo3954", "metadata": {"taxonomy": ["Bacteria", "__Bacteroidetes", "__Flavobacteria", "__Flavobacteriales", "__Cryomorphaceae", "__Crocinitomix"]}},{"id": "denovo3956", "metadata": {"taxonomy": ["Bacteria", "__Proteobacteria", "__Deltaproteobacteria", "__Sh765B-TzT-29", "__f", "__g"]}},{"id": "denovo3958", "metadata": {"taxonomy": ["Bacteria", "__Proteobacteria", "__Alphaproteobacteria", "__Sphingomonadales", "__Erythrobacteraceae"]}},{"id": "denovo3960", "metadata": {"taxonomy": ["Bacteria", "__Proteobacteria", "__Deltaproteobacteria", "__Desulfobacterales", "__Desulfobulbaceae"]}},{"id": "denovo3962", "metadata": {"taxonomy": ["Bacteria", "__Proteobacteria", "__Gammaproteobacteria", "__Oceanospirillales", "__MBAE14", "__g"]}}, {"id": "denovo3963", "metadata": {"taxonomy": ["Bacteria", "__Proteobacteria", "__Alphaproteobacteria", "__Rhodospirillales"]}}, {"id": "denovo3964", "metadata": {"taxonomy": ["Bacteria", "__Proteobacteria", "__Gammaproteobacteria", "__Alteromonadales", "__Alteromonadaceae", "__Haliea"]}},{"id": "denovo3965", "metadata": {"taxonomy": ["Bacteria", "__Planctomycetes", "__Phycisphaerae", "__CCM11a", "__f", "__g"]}},{"id": "denovo3966", "metadata": {"taxonomy": ["Bacteria", "__Proteobacteria", "__Alphaproteobacteria",

"__Rhizobiales", "__Phyllobacteriaceae", "__Cohaesibacter"]}},{"id":
"denovo3967", "metadata": {"taxonomy": ["Bacteria",
"__Proteobacteria", "__AEGEAN-245", "__o", "__f", "__g"]}},{"id":
"denovo3969", "metadata": {"taxonomy": ["Bacteria", "__Chloroflexi",
"__Caldilineae", "__Caldilineales", "__Caldilineaceae",
"__Caldilinea"]}},{"id": "denovo3970", "metadata": {"taxonomy":
["Bacteria", "__Proteobacteria", "__Alphaproteobacteria",
"__Rhizobiales", "__Hyphomicrobiaceae", "__Devosia"]}},{"id":
"denovo3973", "metadata": {"taxonomy": ["Bacteria",
"__Planctomycetes", "__OM190", "__o", "__f", "__g"]}},{"id":
"denovo3974", "metadata": {"taxonomy": ["Bacteria", "__Acidobacteria",
"__Acidobacteria", "__DA023", "__f", "__g"]}},{"id": "denovo3975",
"metadata": {"taxonomy": ["Bacteria", "__Proteobacteria",
"__Gammaproteobacteria", "__Order_Incertae_Sedis",
"__Family_Incertae_Sedis", "__Marinicella"]}},{"id": "denovo3976",
"metadata": {"taxonomy": ["Bacteria", "__Bacteroidetes",
"__Flavobacteria", "__Flavobacteriales", "__Flavobacteriaceae",
"__Maritimimonas"]}},{"id": "denovo3978", "metadata": {"taxonomy":
["Bacteria", "__Bacteroidetes", "__Sphingobacteriia",
"__Sphingobacteriales", "__Saprospiraceae"]}},{"id": "denovo3980",
"metadata": {"taxonomy": ["Bacteria", "__Proteobacteria",
"__Gammaproteobacteria", "__Alteromonadales", "__Alteromonadaceae"]}},
{"id": "denovo3981", "metadata": {"taxonomy": ["Bacteria",
"__Actinobacteria", "__Acidimicrobiia", "__Acidimicrobiales",
"__OCS155_marine_group", "__g"]}},{"id": "denovo3983", "metadata":
{"taxonomy": ["Bacteria", "__Proteobacteria", "__Alphaproteobacteria",
"__DB1-14", "__f", "__g"]}},{"id": "denovo3988", "metadata":
{"taxonomy": ["Bacteria", "__Proteobacteria", "__Gammaproteobacteria",
"__Enterobacteriales", "__Enterobacteriaceae"]}},{"id": "denovo3995",
"metadata": {"taxonomy": ["Bacteria", "__Proteobacteria",
"__Gammaproteobacteria", "__Chromatiales"]}},{"id": "denovo3996",
"metadata": {"taxonomy": ["Bacteria", "__Proteobacteria",
"__Deltaproteobacteria", "__Bdellovibrionales",
"__Bacteriovoracaceae", "__Peredibacter"]}},{"id": "denovo3998",
"metadata": {"taxonomy": ["Bacteria", "__Proteobacteria",
"__Gammaproteobacteria", "__Oceanospirillales", "__Hahellaceae",
"__Endozoicomonas"]}},{"id": "denovo4001", "metadata": {"taxonomy":
["Bacteria", "__Proteobacteria", "__Deltaproteobacteria",
"__Desulfobacterales", "__Nitrospinaceae",
"__Candidatus_Entotheonella"]}},{"id": "denovo4002", "metadata":
{"taxonomy": ["Bacteria", "__Proteobacteria", "__Gammaproteobacteria",
"__Pseudomonadales", "__Moraxellaceae", "__Acinetobacter"]}},{"id":
"denovo4004", "metadata": {"taxonomy": ["Bacteria", "__Firmicutes",
"__Bacilli", "__Lactobacillales", "__Streptococcaceae",
"__Streptococcus"]}},{"id": "denovo4005", "metadata": {"taxonomy":
["Bacteria", "__Proteobacteria", "__Alphaproteobacteria",
"__Rhodospirillales", "__Rhodospirillaceae", "__Thalassospira"]}},
{"id": "denovo4006", "metadata": {"taxonomy": ["Bacteria",
"__Proteobacteria", "__Alphaproteobacteria", "__Rickettsiales"]}},
{"id": "denovo4007", "metadata": {"taxonomy": ["Bacteria",

"__Bacteroidetes"]}},{"id": "denovo4008", "metadata": {"taxonomy": ["Bacteria", "__Proteobacteria", "__Gammaproteobacteria", "__Oceanospirillales", "__Oceanospirillaceae", "__g"]}},{"id": "denovo4010", "metadata": {"taxonomy": ["Bacteria", "__Proteobacteria", "__Deltaproteobacteria", "__Sh765B-TzT-29", "__f", "__g"]}},{"id": "denovo4011", "metadata": {"taxonomy": ["Bacteria", "__Proteobacteria", "__Alphaproteobacteria", "__SAR11_clade", "__Surface_4", "__g"]}},{"id": "denovo4015", "metadata": {"taxonomy": ["Bacteria", "__Actinobacteria", "__Actinobacteria", "__Micrococcales", "__Micrococcaceae", "__Micrococcus"]}},{"id": "denovo4019", "metadata": {"taxonomy": ["Bacteria", "__Bacteroidetes", "__Sphingobacteriia", "__Sphingobacteriales", "__Saprospiraceae", "__g"]}},{"id": "denovo4020", "metadata": {"taxonomy": ["Bacteria", "__Proteobacteria", "__Gammaproteobacteria", "__Methylococcales", "__IheB2-23", "__g"]}},{"id": "denovo4021", "metadata": {"taxonomy": ["Bacteria", "__Proteobacteria", "__Gammaproteobacteria", "__Pseudomonadales", "__Moraxellaceae", "__Acinetobacter"]}},{"id": "denovo4026", "metadata": {"taxonomy": ["Bacteria", "__Proteobacteria", "__Alphaproteobacteria", "__Rhodobacterales", "__Rhodobacteraceae"]}},{"id": "denovo4029", "metadata": {"taxonomy": ["Bacteria", "__Proteobacteria", "__Gammaproteobacteria", "__Alteromonadales", "__Shewanellaceae", "__Shewanella"]}},{"id": "denovo4032", "metadata": {"taxonomy": ["Bacteria", "__Candidate_division_OD1", "__c", "__o", "__f", "__g"]}},{"id": "denovo4036", "metadata": {"taxonomy": ["Bacteria", "__Proteobacteria", "__Alphaproteobacteria", "__SAR11_clade", "__Surface_1", "__g"]}},{"id": "denovo4038", "metadata": {"taxonomy": ["Bacteria", "__Proteobacteria", "__Gammaproteobacteria", "__Xanthomonadales", "__Sinobacteraceae", "__JTB255_marine_benthic_group"]}},{"id": "denovo4041", "metadata": {"taxonomy": ["Bacteria", "__Proteobacteria", "__Alphaproteobacteria", "__Rickettsiales", "__Rickettsiaceae", "__g"]}},{"id": "denovo4042", "metadata": {"taxonomy": ["Bacteria", "__Cyanobacteria", "__4C0d-2", "__o", "__f", "__g"]}},{"id": "denovo4043", "metadata": {"taxonomy": ["Bacteria", "__Proteobacteria", "__Alphaproteobacteria", "__OCS116_clade", "__f", "__g"]}},{"id": "denovo4045", "metadata": {"taxonomy": ["Bacteria", "__Proteobacteria", "__Gammaproteobacteria", "__Oceanospirillales", "__Hahellaceae", "__Endozoicomonas"]}},{"id": "denovo4050", "metadata": {"taxonomy": ["Bacteria", "__Proteobacteria", "__Deltaproteobacteria", "__Desulfobacterales", "__Desulfobulbaceae", "__Desulfofustis"]}},{"id": "denovo4054", "metadata": {"taxonomy": ["Bacteria", "__Proteobacteria", "__Gammaproteobacteria", "__Oceanospirillales", "__Hahellaceae", "__Endozoicomonas"]}},{"id": "denovo4060", "metadata": {"taxonomy": ["Bacteria", "__Spirochaetes", "__Spirochaetes", "__Spirochaetales", "__Leptospiraceae", "__g"]}},{"id": "denovo4061", "metadata": {"taxonomy": ["Bacteria", "__Proteobacteria", "__Gammaproteobacteria", "__Oceanospirillales", "__SS1-B-06-26", "__g"]}},{"id": "denovo4065", "metadata": {"taxonomy": ["Bacteria", "__Proteobacteria", "__Alphaproteobacteria", "__Rhizobiales", "__Rhodobiaceae",

"__Rhodobium"]}},{"id": "denovo4066", "metadata": {"taxonomy": ["Bacteria", "__Bacteroidetes", "__Cytophagia", "__Cytophagales", "__Flammeovirgaceae"]}},{"id": "denovo4067", "metadata": {"taxonomy": ["Bacteria", "__Bacteroidetes", "__Sphingobacteriia", "__Sphingobacteriales", "__Saprospiraceae", "__g"]}},{"id": "denovo4069", "metadata": {"taxonomy": ["Bacteria", "__Proteobacteria", "__Alphaproteobacteria", "__DB1-14", "__f", "__g"]}},{"id": "denovo4070", "metadata": {"taxonomy": ["Bacteria", "__Proteobacteria", "__Alphaproteobacteria", "__Caulobacterales", "__Hyphomonadaceae", "__Hellea"]}},{"id": "denovo4072", "metadata": {"taxonomy": ["Bacteria", "__Proteobacteria", "__Gammaproteobacteria", "__Oceanospirillales", "__Hahellaceae", "__Endozoicomonas"]}},{"id": "denovo4075", "metadata": {"taxonomy": ["Bacteria", "__Proteobacteria", "__Gammaproteobacteria", "__E01-9C-26_marine_group", "__f", "__g"]}},{"id": "denovo4078", "metadata": {"taxonomy": ["Bacteria", "__Proteobacteria", "__Alphaproteobacteria", "__Rhizobiales", "__Phyllobacteriaceae", "__Hoeflea"]}},{"id": "denovo4082", "metadata": {"taxonomy": ["Bacteria", "__Proteobacteria", "__Alphaproteobacteria", "__Rhodospirillales"]}},{"id": "denovo4083", "metadata": {"taxonomy": ["Bacteria", "__Tenericutes", "__Mollicutes", "__Acholeplasmatales", "__Acholeplasmataceae", "__Acholeplasma"]}},{"id": "denovo4087", "metadata": {"taxonomy": ["Bacteria", "__Proteobacteria", "__Alphaproteobacteria", "__Rhizobiales", "__Phyllobacteriaceae"]}},{"id": "denovo4088", "metadata": {"taxonomy": ["Bacteria", "__Proteobacteria", "__Alphaproteobacteria", "__Rhizobiales", "__Phyllobacteriaceae", "__g"]}},{"id": "denovo4091", "metadata": {"taxonomy": ["Bacteria", "__Bacteroidetes", "__Flavobacteria", "__Flavobacteriales", "__Flavobacteriaceae", "__Muricauda"]}},{"id": "denovo4093", "metadata": {"taxonomy": ["Bacteria", "__Acidobacteria", "__Holophagae", "__CA002", "__f", "__g"]}},{"id": "denovo4097", "metadata": {"taxonomy": ["Bacteria", "__Proteobacteria", "__Alphaproteobacteria", "__Rhodospirillales", "__Rhodospirillaceae", "__AEGEAN-169_marine_group"]}},{"id": "denovo4098", "metadata": {"taxonomy": ["Bacteria", "__Firmicutes", "__Clostridia", "__Clostridiales", "__Clostridiaceae", "__Clostridium"]}},{"id": "denovo4100", "metadata": {"taxonomy": ["Bacteria", "__Bacteroidetes", "__Flavobacteria", "__Flavobacteriales", "__Cryomorphaceae", "__Owenweeksia"]}},{"id": "denovo4101", "metadata": {"taxonomy": ["Bacteria", "__Proteobacteria", "__Alphaproteobacteria", "__Rhodobacterales", "__Rhodobacteraceae"]}},{"id": "denovo4106", "metadata": {"taxonomy": ["Bacteria", "__Proteobacteria", "__Gammaproteobacteria", "__Order_Incertae_Sedis", "__Family_Incertae_Sedis", "__Marinicella"]}},{"id": "denovo4109", "metadata": {"taxonomy": ["Bacteria", "__Proteobacteria", "__Gammaproteobacteria", "__Oceanospirillales", "__E12-CR02-full", "__g"]}},{"id": "denovo4114", "metadata": {"taxonomy": ["Bacteria", "__Acidobacteria", "__Acidobacteria", "__PAUC26f", "__f", "__g"]}},{"id": "denovo4118", "metadata": {"taxonomy": ["Bacteria", "__Proteobacteria", "__Gammaproteobacteria", "__Vibrionales",

"__Vibrionaceae", "__Photobacterium"]}},{"id": "denovo4121", "metadata": {"taxonomy": ["Bacteria", "__Proteobacteria", "__Alphaproteobacteria", "__Rhodospirillales", "__Rhodospirillaceae"]}},{"id": "denovo4123", "metadata": {"taxonomy": ["Bacteria", "__Proteobacteria", "__Alphaproteobacteria", "__Rhizobiales", "__Phyllobacteriaceae", "__g"]}},{"id": "denovo4125", "metadata": {"taxonomy": ["Bacteria", "__Proteobacteria", "__Alphaproteobacteria", "__Rhodobacterales", "__Rhodobacteraceae", "__g"]}},{"id": "denovo4128", "metadata": {"taxonomy": ["Bacteria", "__Bacteroidetes", "__Flavobacteria", "__Flavobacteriales", "__Flavobacteriaceae", "__NS5_marine_group"]}},{"id": "denovo4130", "metadata": {"taxonomy": ["Bacteria", "__Proteobacteria", "__Alphaproteobacteria", "__Rhodospirillales", "__Rhodospirillaceae", "__Tistlia"]}},{"id": "denovo4140", "metadata": {"taxonomy": ["Bacteria", "__Actinobacteria", "__Acidimicrobiia", "__Acidimicrobiales", "__OCS155_marine_group", "__g"]}},{"id": "denovo4143", "metadata": {"taxonomy": ["Bacteria", "__Proteobacteria", "__Alphaproteobacteria", "__Rickettsiales", "__SM2D12", "__g"]}},{"id": "denovo4144", "metadata": {"taxonomy": ["Bacteria", "__Acidobacteria", "__RB25", "__o", "__f", "__g"]}},{"id": "denovo4148", "metadata": {"taxonomy": ["Bacteria", "__Proteobacteria", "__Gammaproteobacteria", "__HOC36", "__f", "__g"]}},{"id": "denovo4152", "metadata": {"taxonomy": ["Bacteria", "__Proteobacteria", "__Gammaproteobacteria", "__BD7-8_marine_group", "__f", "__g"]}},{"id": "denovo4153", "metadata": {"taxonomy": ["Bacteria", "__Actinobacteria", "__OPB41", "__o", "__f", "__g"]}},{"id": "denovo4154", "metadata": {"taxonomy": ["Bacteria", "__Cyanobacteria", "__Cyanobacteria", "__SubsectionIII", "__FamilyI", "__g"]}},{"id": "denovo4155", "metadata": {"taxonomy": ["Bacteria", "__Proteobacteria", "__Gammaproteobacteria", "__Alteromonadales", "__Alteromonadaceae", "__Haliea"]}},{"id": "denovo4156", "metadata": {"taxonomy": ["Bacteria", "__Cyanobacteria", "__Cyanobacteria", "__SubsectionI", "__FamilyI", "__Acaryochloris"]}},{"id": "denovo4160", "metadata": {"taxonomy": ["Bacteria", "__Proteobacteria", "__Alphaproteobacteria", "__Rickettsiales", "__SM2D12", "__g"]}},{"id": "denovo4161", "metadata": {"taxonomy": ["Bacteria", "__Proteobacteria", "__Gammaproteobacteria", "__Alteromonadales", "__Alteromonadaceae", "__Haliea"]}},{"id": "denovo4164", "metadata": {"taxonomy": ["Bacteria", "__Firmicutes", "__Clostridia", "__Clostridiales", "__Clostridiaceae", "__Clostridiisalibacter"]}},{"id": "denovo4168", "metadata": {"taxonomy": ["Bacteria", "__Proteobacteria", "__Alphaproteobacteria", "__Rhodobacterales", "__Rhodobacteraceae", "__Dinoroseobacter"]}},{"id": "denovo4170", "metadata": {"taxonomy": ["Bacteria", "__Planctomycetes", "__Planctomycetacia", "__Planctomycetales", "__Planctomycetaceae", "__Rhodopirellula"]}},{"id": "denovo4171", "metadata": {"taxonomy": ["Bacteria", "__Proteobacteria", "__Gammaproteobacteria", "__Alteromonadales", "__Alteromonadaceae", "__C1-B045"]}},{"id": "denovo4173", "metadata": {"taxonomy": ["Bacteria", "__Proteobacteria", "__Gammaproteobacteria",

"__Oceanospirillales", "__Oceanospirillaceae", "__Marinomonas"]}},
{"id": "denovo4174", "metadata": {"taxonomy": ["Bacteria",
"__Proteobacteria", "__Deltaproteobacteria", "__Bdellovibrionales",
"__Bdellovibrionaceae", "__Bdellovibrio"]}},{"id": "denovo4177",
"metadata": {"taxonomy": ["Bacteria", "__Proteobacteria",
"__Gammaproteobacteria", "__Alteromonadales", "__Alteromonadaceae",
"__BD1-7_clade"]}},{"id": "denovo4178", "metadata": {"taxonomy":
["Bacteria", "__Proteobacteria", "__Gammaproteobacteria", "__NKB5",
"__f", "__g"]}},{"id": "denovo4181", "metadata": {"taxonomy":
["Bacteria", "__Bacteroidetes", "__Sphingobacteriia",
"__Sphingobacteriales", "__Saprospiraceae", "__g"]}},{"id":
"denovo4183", "metadata": {"taxonomy": ["Bacteria",
"__Proteobacteria", "__Gammaproteobacteria", "__Legionellales",
"__Coxiellaceae", "__Coxiella"]}},{"id": "denovo4185", "metadata":
{"taxonomy": ["Bacteria", "__Proteobacteria", "__Gammaproteobacteria",
"__Legionellales", "__Legionellaceae", "__Legionella"]}},{"id":
"denovo4187", "metadata": {"taxonomy": ["Bacteria", "__Bacteroidetes",
"__Cytophagia", "__Order_II_Incertae_Sedis", "__Rhodothermaceae",
"__g"]}},{"id": "denovo4188", "metadata": {"taxonomy": ["Bacteria",
"__Bacteroidetes", "__Flavobacteria", "__Flavobacteriales",
"__Flavobacteriaceae", "__Zeaxanthinibacter"]}},{"id": "denovo4189",
"metadata": {"taxonomy": ["Bacteria", "__Proteobacteria",
"__Alphaproteobacteria", "__Sphingomonadales", "__Sphingomonadaceae",
"__Novosphingobium"]}},{"id": "denovo4190", "metadata": {"taxonomy":
["Bacteria", "__Proteobacteria", "__Gammaproteobacteria",
"__Xanthomonadales", "__Xanthomonadaceae", "__Silanimonas"]}},{"id":
"denovo4196", "metadata": {"taxonomy": ["Bacteria",
"__Proteobacteria", "__Alphaproteobacteria", "__Rhizobiales",
"__Phyllobacteriaceae", "__g"]}},{"id": "denovo4197", "metadata":
{"taxonomy": ["Bacteria", "__Proteobacteria", "__Gammaproteobacteria",
"__Pseudomonadales", "__Moraxellaceae", "__Acinetobacter"]}},{"id":
"denovo4199", "metadata": {"taxonomy": ["Bacteria", "__Deinococcus-
Thermus", "__Deinococci", "__Deinococcales", "__Trueperaceae",
"__Truepera"]}},{"id": "denovo4200", "metadata": {"taxonomy":
["Bacteria", "__Proteobacteria", "__Gammaproteobacteria",
"__Chromatiales"]}},{"id": "denovo4202", "metadata": {"taxonomy":
["Bacteria", "__Proteobacteria", "__Alphaproteobacteria",
"__OCS116_clade", "__f", "__g"]}},{"id": "denovo4203", "metadata":
{"taxonomy": ["Bacteria", "__Proteobacteria", "__Gammaproteobacteria",
"__Alteromonadales", "__Pseudoalteromonadaceae", "__Algicola"]}},
{"id": "denovo4204", "metadata": {"taxonomy": ["Bacteria",
"__Acidobacteria", "__Holophagae", "__CA002", "__f", "__g"]}},{"id":
"denovo4205", "metadata": {"taxonomy": ["Bacteria",
"__Proteobacteria", "__Gammaproteobacteria", "__Pseudomonadales",
"__Moraxellaceae", "__Acinetobacter"]}},{"id": "denovo4207",
"metadata": {"taxonomy": ["Bacteria", "__Chloroflexi",
"__SAR202_clade", "__o", "__f", "__g"]}},{"id": "denovo4212",
"metadata": {"taxonomy": ["Bacteria", "__Proteobacteria",
"__Deltaproteobacteria", "__Myxococcales", "__0319-6G20", "__g"]}},
{"id": "denovo4216", "metadata": {"taxonomy": ["Bacteria",

"__Proteobacteria", "__Alphaproteobacteria", "__Rickettsiales", "__SM2D12", "__g"]}},{"id": "denovo4220", "metadata": {"taxonomy": ["Bacteria", "__Proteobacteria", "__Alphaproteobacteria", "__Rhodospirillales", "__Rhodospirillaceae"]}},{"id": "denovo4221", "metadata": {"taxonomy": ["Bacteria", "__Proteobacteria", "__Gammaproteobacteria", "__Chromatiales", "__Chromatiaceae", "__Nitrosococcus"]}},{"id": "denovo4226", "metadata": {"taxonomy": ["Bacteria", "__Chloroflexi", "__Caldilineae", "__Caldilineales", "__Caldilineaceae", "__Caldilinea"]}},{"id": "denovo4228", "metadata": {"taxonomy": ["Bacteria", "__Firmicutes", "__Clostridia", "__Clostridiales", "__Clostridiaceae", "__Clostridiisalibacter"]}},{"id": "denovo4230", "metadata": {"taxonomy": ["Bacteria", "__Actinobacteria", "__Acidimicrobiia", "__Acidimicrobiales", "__uncultured", "__g"]}},{"id": "denovo4231", "metadata": {"taxonomy": ["Archaea", "__Euryarchaeota", "__Thermoplasmata", "__Marine_Benthic_Group_E", "__f", "__g"]}},{"id": "denovo4239", "metadata": {"taxonomy": ["Bacteria"]}},{"id": "denovo4242", "metadata": {"taxonomy": ["Bacteria", "__Actinobacteria", "__Actinobacteria", "__Corynebacteriales", "__Corynebacteriaceae", "__Corynebacterium"]}},{"id": "denovo4243", "metadata": {"taxonomy": ["Bacteria", "__Bacteroidetes", "__Flavobacteria", "__Flavobacteriales", "__Cryomorphaceae", "__Owenweeksia"]}},{"id": "denovo4245", "metadata": {"taxonomy": ["Bacteria", "__Proteobacteria", "__Gammaproteobacteria", "__Enterobacteriales", "__Enterobacteriaceae", "__Escherichia-Shigella"]}},{"id": "denovo4248", "metadata": {"taxonomy": ["Bacteria", "__Acidobacteria", "__RB25", "__o", "__f", "__g"]}},{"id": "denovo4249", "metadata": {"taxonomy": ["Bacteria", "__Proteobacteria", "__Gammaproteobacteria", "__Oceanospirillales"]}},{"id": "denovo4252", "metadata": {"taxonomy": ["Bacteria", "__Proteobacteria", "__Alphaproteobacteria", "__SAR11_clade", "__Surface_1"]}},{"id": "denovo4255", "metadata": {"taxonomy": ["Bacteria", "__Proteobacteria", "__Gammaproteobacteria", "__Alteromonadales", "__Alteromonadaceae", "__BD1-7_clade"]}},{"id": "denovo4257", "metadata": {"taxonomy": ["Bacteria", "__Firmicutes"]}},{"id": "denovo4262", "metadata": {"taxonomy": ["Bacteria", "__Proteobacteria", "__Alphaproteobacteria", "__Rhodospirillales", "__Rhodospirillaceae", "__g"]}},{"id": "denovo4267", "metadata": {"taxonomy": ["Bacteria", "__Proteobacteria", "__Gammaproteobacteria", "__Run-SP154", "__f", "__g"]}},{"id": "denovo4268", "metadata": {"taxonomy": ["Bacteria", "__Proteobacteria", "__Deltaproteobacteria"]}},{"id": "denovo4270", "metadata": {"taxonomy": ["Bacteria", "__Bacteroidetes", "__Sphingobacteriia", "__Sphingobacteriales", "__Saprospiraceae", "__g"]}},{"id": "denovo4271", "metadata": {"taxonomy": ["Bacteria", "__Proteobacteria", "__Alphaproteobacteria", "__Rhodobacterales", "__Rhodobacteraceae", "__Pseudovibrio"]}},{"id": "denovo4286", "metadata": {"taxonomy": ["Bacteria", "__Cyanobacteria", "__Cyanobacteria", "__SubsectionIV", "__FamilyII", "__Rivularia"]}},{"id": "denovo4287", "metadata": {"taxonomy": ["Bacteria", "__Bacteroidetes", "__Flavobacteria", "__Flavobacteriales",

"__Flavobacteriaceae", "__Leptobacterium"]}},{"id": "denovo4290", "metadata": {"taxonomy": ["Bacteria", "__Proteobacteria", "__Gammaproteobacteria", "__EC3", "__f", "__g"]}},{"id": "denovo4294", "metadata": {"taxonomy": ["Bacteria", "__Proteobacteria", "__Alphaproteobacteria", "__DB1-14", "__f", "__g"]}},{"id": "denovo4295", "metadata": {"taxonomy": ["Bacteria", "__Proteobacteria", "__Alphaproteobacteria", "__Rickettsiales"]}}, {"id": "denovo4296", "metadata": {"taxonomy": ["Bacteria", "__Firmicutes", "__Clostridia", "__Clostridiales", "__Clostridiaceae", "__Clostridium"]}},{"id": "denovo4298", "metadata": {"taxonomy": ["Bacteria", "__Bacteroidetes", "__Cytophagia", "__Order_III_Incertae_Sedis", "__Family_Incertae_Sedis", "__Balneola"]}},{"id": "denovo4302", "metadata": {"taxonomy": ["Bacteria", "__Bacteroidetes", "__Flavobacteria", "__Flavobacteriales", "__Flavobacteriaceae", "__NS5_marine_group"]}}, {"id": "denovo4305", "metadata": {"taxonomy": ["Bacteria", "__Proteobacteria", "__Gammaproteobacteria", "__Enterobacteriales", "__Enterobacteriaceae"]}},{"id": "denovo4310", "metadata": {"taxonomy": ["Bacteria", "__Proteobacteria", "__Alphaproteobacteria", "__Rhodospirillales", "__Rhodospirillaceae", "__Defluviicoccus"]}}, {"id": "denovo4315", "metadata": {"taxonomy": ["Bacteria", "__Proteobacteria", "__Gammaproteobacteria", "__Order_Incertae_Sedis", "__Family_Incertae_Sedis", "__Marinicella"]}},{"id": "denovo4316", "metadata": {"taxonomy": ["Bacteria", "__Proteobacteria", "__Alphaproteobacteria", "__Sphingomonadales", "__Sphingomonadaceae", "__Sphingomonas"]}},{"id": "denovo4317", "metadata": {"taxonomy": ["Bacteria", "__Proteobacteria", "__Deltaproteobacteria", "__Bdellovibrionales", "__Bdellovibrionaceae", "__OM27_clade"]}}, {"id": "denovo4318", "metadata": {"taxonomy": ["Bacteria", "__Verrucomicrobia", "__Opitutae", "__MB11C04_marine_group", "__f", "__g"]}},{"id": "denovo4322", "metadata": {"taxonomy": ["Bacteria", "__Proteobacteria", "__Alphaproteobacteria", "__Rhodospirillales", "__Rhodospirillaceae", "__g"]}},{"id": "denovo4324", "metadata": {"taxonomy": ["Bacteria", "__Proteobacteria", "__Gammaproteobacteria", "__Oceanospirillales"]}},{"id": "denovo4331", "metadata": {"taxonomy": ["Bacteria", "__Proteobacteria", "__Alphaproteobacteria", "__OCS116_clade", "__f", "__g"]}},{"id": "denovo4338", "metadata": {"taxonomy": ["Bacteria", "__Proteobacteria", "__Betaproteobacteria", "__Burkholderiales", "__Burkholderiaceae", "__Limnobacter"]}},{"id": "denovo4339", "metadata": {"taxonomy": ["Bacteria", "__Proteobacteria", "__Deltaproteobacteria", "__Sh765B-TzT-29", "__f", "__g"]}},{"id": "denovo4341", "metadata": {"taxonomy": ["Bacteria", "__Proteobacteria", "__Gammaproteobacteria", "__KI89A_clade", "__f", "__g"]}},{"id": "denovo4344", "metadata": {"taxonomy": ["Bacteria", "__Chloroflexi", "__SAR202_clade", "__o", "__f", "__g"]}},{"id": "denovo4345", "metadata": {"taxonomy": ["Bacteria", "__Proteobacteria", "__Alphaproteobacteria", "__OCS116_clade", "__f", "__g"]}},{"id": "denovo4350", "metadata": {"taxonomy": ["Bacteria", "__Proteobacteria", "__Gammaproteobacteria"]}},{"id": "denovo4351", "metadata": {"taxonomy": ["Bacteria", "__Proteobacteria",

"__Gammaproteobacteria", "__Chromatiales", "__Chromatiaceae"]}},{"id": "denovo4352", "metadata": {"taxonomy": ["Bacteria", "__Chlamydiae", "__Chlamydiae", "__Chlamydiales", "__Simkaniaceae", "__Simkania"]}}, {"id": "denovo4353", "metadata": {"taxonomy": ["Bacteria", "__Bacteroidetes", "__Cytophagia", "__Cytophagales", "__Flammeovirgaceae", "__g"]}},{"id": "denovo4355", "metadata": {"taxonomy": ["Bacteria", "__Proteobacteria", "__Alphaproteobacteria", "__Rhodospirillales", "__Rhodospirillaceae"]}},{"id": "denovo4358", "metadata": {"taxonomy": ["Bacteria", "__Bacteroidetes", "__Sphingobacteriia", "__Sphingobacteriales", "__Saprospiraceae", "__g"]}},{"id": "denovo4360", "metadata": {"taxonomy": ["Bacteria", "__Proteobacteria", "__Alphaproteobacteria", "__Rhodobacterales", "__Rhodobacteraceae"]}},{"id": "denovo4366", "metadata": {"taxonomy": ["Bacteria", "__Proteobacteria", "__Gammaproteobacteria", "__Pseudomonadales", "__Moraxellaceae", "__Acinetobacter"]}},{"id": "denovo4367", "metadata": {"taxonomy": ["Bacteria", "__Proteobacteria", "__Alphaproteobacteria", "__SAR11_clade", "__Surface_1", "__g"]}},{"id": "denovo4373", "metadata": {"taxonomy": ["Bacteria", "__Firmicutes", "__Clostridia", "__Clostridiales", "__Clostridiaceae"]}},{"id": "denovo4374", "metadata": {"taxonomy": ["Bacteria", "__Proteobacteria", "__Deltaproteobacteria", "__Desulfobacterales", "__Desulfobacteraceae", "__Desulfofaba"]}}, {"id": "denovo4382", "metadata": {"taxonomy": ["Bacteria", "__Proteobacteria", "__Alphaproteobacteria", "__Rickettsiales", "__S25-593", "__g"]}},{"id": "denovo4390", "metadata": {"taxonomy": ["Bacteria", "__Bacteroidetes", "__Cytophagia", "__Cytophagales", "__Flammeovirgaceae", "__Persicobacter"]}},{"id": "denovo4395", "metadata": {"taxonomy": ["Bacteria", "__Proteobacteria", "__Betaproteobacteria", "__Burkholderiales", "__Oxalobacteraceae", "__Massilia"]}},{"id": "denovo4402", "metadata": {"taxonomy": ["Bacteria", "__Proteobacteria", "__Gammaproteobacteria", "__Oceanospirillales", "__Hahellaceae", "__Endozoicomonas"]}},{"id": "denovo4408", "metadata": {"taxonomy": ["Bacteria", "__Proteobacteria", "__Gammaproteobacteria", "__Chromatiales", "__Chromatiaceae"]}},{"id": "denovo4411", "metadata": {"taxonomy": ["Bacteria", "__Chlamydiae", "__Chlamydiae", "__Chlamydiales", "__Parachlamydiaceae"]}},{"id": "denovo4412", "metadata": {"taxonomy": ["Bacteria", "__Bacteroidetes", "__Sphingobacteriia", "__Sphingobacteriales", "__Saprospiraceae", "__g"]}},{"id": "denovo4415", "metadata": {"taxonomy": ["Bacteria", "__BD1-5", "__c", "__o", "__f", "__g"]}},{"id": "denovo4416", "metadata": {"taxonomy": ["Bacteria", "__Bacteroidetes", "__Sphingobacteriia", "__Sphingobacteriales", "__Saprospiraceae", "__g"]}},{"id": "denovo4421", "metadata": {"taxonomy": ["Bacteria", "__Planctomycetes", "__C47", "__o", "__f", "__g"]}},{"id": "denovo4422", "metadata": {"taxonomy": ["Bacteria", "__Proteobacteria", "__Deltaproteobacteria", "__Desulfobacterales", "__Desulfobulbaceae"]}},{"id": "denovo4424", "metadata": {"taxonomy": ["Bacteria", "__Proteobacteria", "__Gammaproteobacteria", "__Alteromonadales", "__Shewanellaceae", "__Shewanella"]}},{"id":

"denovo4426", "metadata": {"taxonomy": ["Bacteria",
"__Proteobacteria", "__Alphaproteobacteria", "__Rhizobiales",
"__Phyllobacteriaceae", "__Phyllobacterium"]}},{"id": "denovo4434",
"metadata": {"taxonomy": ["Bacteria", "__Firmicutes", "__Clostridia",
"__Clostridiales", "__Kazan-2B-17", "__g"]}},{"id": "denovo4435",
"metadata": {"taxonomy": ["Bacteria", "__Verrucomicrobia",
"__Opitutae", "__RS-B22", "__f", "__g"]}},{"id": "denovo4437",
"metadata": {"taxonomy": ["Bacteria", "__Proteobacteria",
"__Alphaproteobacteria", "__Rhodobacterales", "__Rhodobacteraceae",
"__Roseibium"]}},{"id": "denovo4440", "metadata": {"taxonomy":
["Bacteria", "__Proteobacteria", "__Deltaproteobacteria",
"__Desulfobacterales", "__Desulfobacteraceae"]}},{"id": "denovo4441",
"metadata": {"taxonomy": ["Bacteria", "__Proteobacteria",
"__Alphaproteobacteria", "__Sphingomonadales", "__MN_122.2a",
"__g"]}},{"id": "denovo4445", "metadata": {"taxonomy": ["Bacteria",
"__Proteobacteria", "__Alphaproteobacteria", "__Parvularculales",
"__Parvularculaceae", "__Parvularcula"]}},{"id": "denovo4446",
"metadata": {"taxonomy": ["Bacteria", "__Proteobacteria",
"__Gammaproteobacteria", "__Alteromonadales",
"__Pseudoalteromonadaceae", "__Pseudoalteromonas"]}},{"id":
"denovo4450", "metadata": {"taxonomy": ["Bacteria", "__Acidobacteria",
"__Holophagae", "__NS72", "__f", "__g"]}},{"id": "denovo4454",
"metadata": {"taxonomy": ["Bacteria", "__Proteobacteria",
"__Gammaproteobacteria", "__Oceanospirillales", "__SAR86_clade",
"__g"]}},{"id": "denovo4457", "metadata": {"taxonomy": ["Bacteria",
"__Proteobacteria", "__Gammaproteobacteria"]}},{"id": "denovo4459",
"metadata": {"taxonomy": ["Bacteria", "__Cyanobacteria",
"__Cyanobacteria", "__SubsectionIII", "__FamilyI", "__Phormidium"]}},
{"id": "denovo4460", "metadata": {"taxonomy": ["Bacteria",
"__Bacteroidetes", "__Sphingobacteriia", "__Sphingobacteriales",
"__Saprospiraceae", "__g"]}},{"id": "denovo4464", "metadata":
{"taxonomy": ["Bacteria", "__Proteobacteria", "__Alphaproteobacteria",
"__Rhodobacterales", "__Rhodobacteraceae", "__Paracoccus"]}},{"id":
"denovo4465", "metadata": {"taxonomy": ["Bacteria",
"__Proteobacteria", "__Alphaproteobacteria", "__Rhizobiales",
"__Methylobacteriaceae", "__Microvirga"]}},{"id": "denovo4469",
"metadata": {"taxonomy": ["Bacteria", "__Proteobacteria",
"__Gammaproteobacteria"]}},{"id": "denovo4474", "metadata":
{"taxonomy": ["Bacteria", "__Proteobacteria", "__Alphaproteobacteria",
"__Rhodobacterales", "__Rhodobacteraceae", "__Ruegeria"]}},{"id":
"denovo4475", "metadata": {"taxonomy": ["Bacteria", "__Bacteroidetes",
"__Cytophagia", "__Cytophagales", "__Flammeovirgaceae",
"__Candidatus_Amoebophilus"]}},{"id": "denovo4476", "metadata":
{"taxonomy": ["Bacteria", "__Proteobacteria", "__Gammaproteobacteria",
"__Oceanospirillales", "__Hahellaceae", "__Endozoicomonas"]}},{"id":
"denovo4480", "metadata": {"taxonomy": ["Bacteria", "__Acidobacteria",
"__Holophagae", "__CA002", "__f", "__g"]}},{"id": "denovo4485",
"metadata": {"taxonomy": ["Bacteria", "__Verrucomicrobia",
"__Opitutae", "__Puniceicoccales", "__Puniceicoccaceae",
"__Pelagicoccus"]}},{"id": "denovo4486", "metadata": {"taxonomy":

["Bacteria", "__Chloroflexi", "__SAR202_clade", "__o", "__f", "__g"]}},{"id": "denovo4487", "metadata": {"taxonomy": ["Bacteria", "__Proteobacteria", "__Gammaproteobacteria", "__Oceanospirillales", "__Oceanospirillaceae", "__Neptunomonas"]}},{"id": "denovo4488", "metadata": {"taxonomy": ["Bacteria", "__Bacteroidetes", "__Flavobacteria", "__Flavobacteriales", "__Flavobacteriaceae", "__Leptobacterium"]}},{"id": "denovo4491", "metadata": {"taxonomy": ["Bacteria", "__Proteobacteria", "__Deltaproteobacteria", "__Sh765B-TzT-29", "__f", "__g"]}},{"id": "denovo4493", "metadata": {"taxonomy": ["Bacteria", "__Proteobacteria", "__Gammaproteobacteria", "__Oceanospirillales", "__Oceanospirillaceae", "__Pseudospirillum"]}},{"id": "denovo4497", "metadata": {"taxonomy": ["Bacteria", "__Cyanobacteria", "__Cyanobacteria", "__SubsectionIII", "__FamilyI"]}},{"id": "denovo4502", "metadata": {"taxonomy": ["Bacteria", "__Planctomycetes", "__Pla3_lineage", "__o", "__f", "__g"]}},{"id": "denovo4504", "metadata": {"taxonomy": ["Bacteria", "__Proteobacteria", "__Gammaproteobacteria", "__Oceanospirillales"]}},{"id": "denovo4508", "metadata": {"taxonomy": ["Bacteria", "__Proteobacteria", "__Alphaproteobacteria"]}},{"id": "denovo4511", "metadata": {"taxonomy": ["Bacteria", "__Proteobacteria"]}},{"id": "denovo4513", "metadata": {"taxonomy": ["Bacteria", "__Proteobacteria", "__Alphaproteobacteria", "__Rhodobacterales", "__Rhodobacteraceae"]}},{"id": "denovo4516", "metadata": {"taxonomy": ["Bacteria", "__Proteobacteria", "__Gammaproteobacteria", "__Alteromonadales", "__Alteromonadaceae", "__BD1-7_clade"]}},{"id": "denovo4519", "metadata": {"taxonomy": ["Bacteria", "__Actinobacteria", "__Thermoleophilia", "__Solirubrobacterales", "__Elev-16S-1332", "__g"]}},{"id": "denovo4524", "metadata": {"taxonomy": ["Bacteria", "__Proteobacteria", "__Betaproteobacteria", "__Burkholderiales", "__Alcaligenaceae", "__MWH-UniP1_aquatic_group"]}},{"id": "denovo4527", "metadata": {"taxonomy": ["Bacteria", "__Bacteroidetes", "__Cytophagia", "__Cytophagales", "__Flammeovirgaceae", "__g"]}},{"id": "denovo4528", "metadata": {"taxonomy": ["Bacteria", "__Proteobacteria", "__Alphaproteobacteria", "__Rhodospirillales", "__Candidatus_Alysiosphaera", "__g"]}},{"id": "denovo4533", "metadata": {"taxonomy": ["Bacteria", "__Gemmatimonadetes", "__Gemmatimonadetes", "__PAUC43f_marine_benthic_group", "__f", "__g"]}},{"id": "denovo4534", "metadata": {"taxonomy": ["Bacteria", "__Proteobacteria", "__Alphaproteobacteria", "__Rhizobiales", "__Hyphomicrobiaceae", "__Rhodoplanes"]}},{"id": "denovo4536", "metadata": {"taxonomy": ["Bacteria", "__Proteobacteria", "__Gammaproteobacteria", "__Oceanospirillales", "__Oleiphilaceae", "__Oleiphilus"]}},{"id": "denovo4538", "metadata": {"taxonomy": ["Bacteria", "__Bacteroidetes", "__Flavobacteria", "__Flavobacteriales", "__Cryomorphaceae", "__NS7_marine_group"]}},{"id": "denovo4540", "metadata": {"taxonomy": ["Bacteria", "__Candidate_division_WS3", "__c", "__o", "__f", "__g"]}},{"id": "denovo4547", "metadata": {"taxonomy": ["Bacteria", "__Bacteroidetes", "__Sphingobacteriia", "__Sphingobacteriales", "__Saprospiraceae", "__g"]}},{"id": "denovo4551", "metadata":

{"taxonomy": ["Bacteria", "__Planctomycetes", "__Phycisphaerae", "__Phycisphaerales", "__Phycisphaeraceae", "__Urania-1B-19_marine_sediment_group"]}},{"id": "denovo4552", "metadata": {"taxonomy": ["Bacteria", "__Proteobacteria", "__Deltaproteobacteria", "__Sh765B-TzT-29", "__f", "__g"]}},{"id": "denovo4553", "metadata": {"taxonomy": ["Bacteria", "__Proteobacteria", "__Alphaproteobacteria", "__DB1-14", "__f", "__g"]}},{"id": "denovo4554", "metadata": {"taxonomy": ["Bacteria", "__Proteobacteria", "__Gammaproteobacteria", "__Oceanospirillales", "__SS1-B-06-26", "__g"]}},{"id": "denovo4556", "metadata": {"taxonomy": ["Bacteria", "__Bacteroidetes", "__Cytophagia", "__Cytophagales", "__Flammeovirgaceae"]}},{"id": "denovo4559", "metadata": {"taxonomy": ["Bacteria", "__Proteobacteria", "__Betaproteobacteria", "__Methylophilales", "__Methylophilaceae"]}},{"id": "denovo4560", "metadata": {"taxonomy": ["Bacteria", "__Chlorobi", "__Chlorobia", "__Chlorobiales", "__Chlorobiaceae", "__Prosthecochloris"]}},{"id": "denovo4562", "metadata": {"taxonomy": ["Bacteria", "__Proteobacteria", "__Gammaproteobacteria"]}},{"id": "denovo4564", "metadata": {"taxonomy": ["Bacteria", "__Bacteroidetes", "__Flavobacteria", "__Flavobacteriales", "__Flavobacteriaceae", "__Leptobacterium"]}},{"id": "denovo4566", "metadata": {"taxonomy": ["Bacteria", "__Proteobacteria", "__Deltaproteobacteria", "__Desulfuromonadales", "__GR-WP33-58", "__g"]}},{"id": "denovo4569", "metadata": {"taxonomy": ["Bacteria", "__Proteobacteria", "__Deltaproteobacteria", "__Desulfuromonadales", "__Desulfuromonadaceae", "__Desulfuromusa"]}},{"id": "denovo4571", "metadata": {"taxonomy": ["Bacteria", "__Bacteroidetes", "__Bacteroidia", "__Bacteroidales", "__Marinilabiaceae", "__Marinifilum"]}},{"id": "denovo4572", "metadata": {"taxonomy": ["Bacteria", "__Proteobacteria", "__Alphaproteobacteria", "__MNG3", "__f", "__g"]}},{"id": "denovo4575", "metadata": {"taxonomy": ["Bacteria", "__Gemmatimonadetes", "__Gemmatimonadetes", "__BD2-11_terrestrial_group", "__f", "__g"]}},{"id": "denovo4576", "metadata": {"taxonomy": ["Bacteria", "__Proteobacteria", "__Gammaproteobacteria", "__Alteromonadales", "__Ferrimonadaceae", "__Ferrimonas"]}},{"id": "denovo4577", "metadata": {"taxonomy": ["Bacteria", "__Proteobacteria", "__Deltaproteobacteria", "__Desulfobacterales", "__Desulfobacteraceae", "__Sva0081_sediment_group"]}},{"id": "denovo4586", "metadata": {"taxonomy": ["Bacteria", "__Proteobacteria", "__SC3-20", "__o", "__f", "__g"]}},{"id": "denovo4589", "metadata": {"taxonomy": ["Bacteria", "__Proteobacteria", "__Betaproteobacteria", "__Burkholderiales", "__Burkholderiaceae", "__Limnobacter"]}},{"id": "denovo4593", "metadata": {"taxonomy": ["Bacteria", "__Proteobacteria", "__TA18", "__o", "__f", "__g"]}},{"id": "denovo4597", "metadata": {"taxonomy": ["Bacteria", "__Proteobacteria", "__Alphaproteobacteria", "__Rhizobiales", "__Rhodobiaceae", "__Anderseniella"]}},{"id": "denovo4598", "metadata": {"taxonomy": ["Bacteria", "__Bacteroidetes", "__Flavobacteria", "__Flavobacteriales", "__Flavobacteriaceae",

"__Chryseobacterium"]}},{"id": "denovo4599", "metadata": {"taxonomy": ["Bacteria", "__Cyanobacteria", "__Cyanobacteria", "__SubsectionIII", "__FamilyI"]}},{"id": "denovo4601", "metadata": {"taxonomy": ["Bacteria", "__Proteobacteria", "__Deltaproteobacteria", "__Bdellovibrionales", "__Bdellovibrionaceae", "__Bdellovibrio"]}}, {"id": "denovo4605", "metadata": {"taxonomy": ["Bacteria", "__Proteobacteria", "__Alphaproteobacteria", "__Rhodobacterales", "__Rhodobacteraceae", "__g"]}},{"id": "denovo4607", "metadata": {"taxonomy": ["Bacteria", "__Bacteroidetes", "__Flavobacteria", "__Flavobacteriales", "__Flavobacteriaceae"]}},{"id": "denovo4612", "metadata": {"taxonomy": ["Bacteria", "__Bacteroidetes", "__Flavobacteria", "__Flavobacteriales", "__Cryomorphaceae", "__Owenweeksia"]}},{"id": "denovo4615", "metadata": {"taxonomy": ["Bacteria", "__Proteobacteria", "__Alphaproteobacteria", "__Rhizobiales"]}},{"id": "denovo4617", "metadata": {"taxonomy": ["Bacteria"]}},{"id": "denovo4618", "metadata": {"taxonomy": ["Bacteria", "__Proteobacteria", "__Gammaproteobacteria", "__Alteromonadales", "__Idiomarinaceae", "__Idiomarina"]}},{"id": "denovo4625", "metadata": {"taxonomy": ["Bacteria", "__Proteobacteria", "__Gammaproteobacteria", "__Thiotrichales", "__Piscirickettsiaceae", "__Methylophaga"]}},{"id": "denovo4627", "metadata": {"taxonomy": ["Bacteria", "__Proteobacteria", "__Alphaproteobacteria", "__Caulobacterales", "__Caulobacteraceae", "__Brevundimonas"]}},{"id": "denovo4630", "metadata": {"taxonomy": ["Bacteria", "__Actinobacteria", "__Actinobacteria", "__Micrococcales", "__Micrococcaceae", "__Rothia"]}},{"id": "denovo4631", "metadata": {"taxonomy": ["Bacteria", "__Proteobacteria", "__Gammaproteobacteria", "__Thiotrichales", "__Thiotrichaceae", "__Cocleimonas"]}},{"id": "denovo4638", "metadata": {"taxonomy": ["Bacteria", "__Proteobacteria", "__Deltaproteobacteria", "__Desulfuromonadales", "__GR-WP33-58", "__g"]}},{"id": "denovo4640", "metadata": {"taxonomy": ["Bacteria", "__Actinobacteria", "__Acidimicrobiia", "__Acidimicrobiales", "__OCS155_marine_group", "__g"]}},{"id": "denovo4649", "metadata": {"taxonomy": ["Bacteria", "__Proteobacteria", "__Alphaproteobacteria", "__Rhodobacterales", "__Rhodobacteraceae", "__g"]}},{"id": "denovo4650", "metadata": {"taxonomy": ["Bacteria", "__Chlamydiae", "__Chlamydiae", "__Chlamydiales", "__Simkaniaceae", "__g"]}},{"id": "denovo4651", "metadata": {"taxonomy": ["Bacteria", "__Proteobacteria", "__Gammaproteobacteria", "__Oceanospirillales", "__Hahellaceae", "__Endozoicomonas"]}},{"id": "denovo4652", "metadata": {"taxonomy": ["Bacteria", "__Spirochaetes", "__Spirochaetes", "__Spirochaetales", "__Spirochaetaceae", "__Spirochaeta"]}},{"id": "denovo4653", "metadata": {"taxonomy": ["Bacteria", "__Cyanobacteria", "__Cyanobacteria", "__SubsectionIII", "__FamilyI", "__g"]}},{"id": "denovo4661", "metadata": {"taxonomy": ["Bacteria", "__Proteobacteria", "__Gammaproteobacteria", "__Legionellales", "__Coxiellaceae", "__Coxiella"]}},{"id": "denovo4666", "metadata": {"taxonomy": ["Bacteria", "__Proteobacteria", "__Alphaproteobacteria", "__Sphingomonadales"]}},

{"id": "denovo4669", "metadata": {"taxonomy": ["Bacteria", "__Chlorobi", "__Chlorobia", "__Chlorobiales", "__Chlorobiaceae", "__Prosthecochloris"]}},{"id": "denovo4670", "metadata": {"taxonomy": ["Bacteria", "__Proteobacteria", "__Gammaproteobacteria", "__Oceanospirillales", "__SAR86_clade", "__g"]}},{"id": "denovo4671", "metadata": {"taxonomy": ["Bacteria", "__Proteobacteria", "__Alphaproteobacteria", "__Rhodospirillales", "__Rhodospirillaceae"]}},{"id": "denovo4672", "metadata": {"taxonomy": ["Bacteria", "__Proteobacteria", "__Alphaproteobacteria", "__Rhodospirillales"]}},{"id": "denovo4677", "metadata": {"taxonomy": ["Bacteria", "__Proteobacteria", "__Alphaproteobacteria", "__Rhizobiales", "__Family_Incertae_Sedis", "__Bauldia"]}},{"id": "denovo4678", "metadata": {"taxonomy": ["Bacteria", "__Proteobacteria", "__Gammaproteobacteria", "__KI89A_clade", "__f", "__g"]}},{"id": "denovo4679", "metadata": {"taxonomy": ["Bacteria", "__Actinobacteria", "__Acidimicrobiia", "__Acidimicrobiales", "__Sva0996_marine_group", "__g"]}},{"id": "denovo4686", "metadata": {"taxonomy": ["Bacteria", "__Proteobacteria", "__Alphaproteobacteria", "__Rhodospirillales", "__Rhodospirillaceae", "__AEGEAN-169_marine_group"]}},{"id": "denovo4690", "metadata": {"taxonomy": ["Bacteria", "__Proteobacteria", "__Gammaproteobacteria", "__Alteromonadales", "__Alteromonadaceae", "__Umboniibacter"]}},{"id": "denovo4697", "metadata": {"taxonomy": ["Bacteria", "__Proteobacteria", "__Gammaproteobacteria"]}},{"id": "denovo4699", "metadata": {"taxonomy": ["Bacteria", "__Proteobacteria", "__Alphaproteobacteria", "__Rhodospirillales", "__Rhodospirillaceae", "__Pelagibius"]}},{"id": "denovo4700", "metadata": {"taxonomy": ["Bacteria", "__Bacteroidetes", "__Flavobacteria", "__Flavobacteriales", "__Flavobacteriaceae", "__Formosa"]}},{"id": "denovo4703", "metadata": {"taxonomy": ["Bacteria", "__Proteobacteria", "__Gammaproteobacteria", "__Alteromonadales", "__Alteromonadaceae"]}},{"id": "denovo4706", "metadata": {"taxonomy": ["Bacteria", "__Proteobacteria", "__Gammaproteobacteria", "__Chromatiales"]}},{"id": "denovo4708", "metadata": {"taxonomy": ["Bacteria", "__Planctomycetes", "__Planctomycetacia", "__Planctomycetales", "__Planctomycetaceae", "__Rhodopirellula"]}},{"id": "denovo4710", "metadata": {"taxonomy": ["Bacteria", "__Proteobacteria", "__Deltaproteobacteria", "__Myxococcales", "__Cystobacterineae", "__g"]}},{"id": "denovo4713", "metadata": {"taxonomy": ["Bacteria", "__Proteobacteria", "__Alphaproteobacteria", "__SAR11_clade", "__Surface_1", "__g"]}},{"id": "denovo4714", "metadata": {"taxonomy": ["Bacteria", "__Actinobacteria", "__Acidimicrobiia", "__Acidimicrobiales", "__Acidimicrobiaceae"]}},{"id": "denovo4718", "metadata": {"taxonomy": ["Bacteria", "__Planctomycetes", "__Planctomycetacia", "__Planctomycetales", "__Planctomycetaceae", "__Rhodopirellula"]}},{"id": "denovo4720", "metadata": {"taxonomy": ["Bacteria", "__Proteobacteria", "__Gammaproteobacteria", "__Alteromonadales", "__Alteromonadaceae"]}},{"id": "denovo4723", "metadata": {"taxonomy": ["Bacteria", "__Proteobacteria", "__Gammaproteobacteria", "__Pseudomonadales",

"__Moraxellaceae", "__Acinetobacter"]}},{"id": "denovo4724",
"metadata": {"taxonomy": ["Bacteria", "__Proteobacteria",
"__Betaproteobacteria", "__Methylophilales", "__Methylophilaceae",
"__Methylophilus"]}},{"id": "denovo4725", "metadata": {"taxonomy":
["Bacteria", "__Proteobacteria", "__Deltaproteobacteria",
"__Desulfovibrionales", "__Desulfovibrionaceae", "__Desulfovibrio"]}},
{"id": "denovo4728", "metadata": {"taxonomy": ["Bacteria",
"__Proteobacteria", "__Gammaproteobacteria"]}},{"id": "denovo4729",
"metadata": {"taxonomy": ["Bacteria", "__Proteobacteria",
"__Alphaproteobacteria"]}},{"id": "denovo4732", "metadata":
{"taxonomy": ["Bacteria", "__Proteobacteria", "__Alphaproteobacteria",
"__Rhodobacterales", "__Rhodobacteraceae"]}},{"id": "denovo4734",
"metadata": {"taxonomy": ["Bacteria", "__Proteobacteria",
"__Gammaproteobacteria"]}},{"id": "denovo4736", "metadata":
{"taxonomy": ["Bacteria", "__Proteobacteria",
"__Gammaproteobacteria"]}},{"id": "denovo4737", "metadata":
{"taxonomy": ["Bacteria", "__Proteobacteria", "__Gammaproteobacteria",
"__NKB5", "__f", "__g"]}},{"id": "denovo4742", "metadata":
{"taxonomy": ["Bacteria", "__Proteobacteria", "__Gammaproteobacteria",
"__Xanthomonadales", "__Sinobacteraceae",
"__JTB255_marine_benthic_group"]}},{"id": "denovo4744", "metadata":
{"taxonomy": ["Bacteria", "__Proteobacteria", "__Alphaproteobacteria",
"__Rhodobacterales", "__Rhodobacteraceae"]}},{"id": "denovo4745",
"metadata": {"taxonomy": ["Bacteria", "__Bacteroidetes",
"__Flavobacteria", "__Flavobacteriales", "__Flavobacteriaceae"]}},
{"id": "denovo4746", "metadata": {"taxonomy": ["Bacteria",
"__Spirochaetes", "__Spirochaetes", "__Spirochaetales",
"__Leptospiraceae", "__g"]}},{"id": "denovo4748", "metadata":
{"taxonomy": ["Bacteria", "__Proteobacteria", "__Alphaproteobacteria",
"__Rhodospirillales", "__Rhodospirillaceae"]}},{"id": "denovo4751",
"metadata": {"taxonomy": ["Bacteria", "__Proteobacteria",
"__Deltaproteobacteria", "__Sh765B-TzT-29", "__f", "__g"]}},{"id":
"denovo4752", "metadata": {"taxonomy": ["Bacteria",
"__Proteobacteria", "__Alphaproteobacteria", "__DB1-14", "__f",
"__g"]}},{"id": "denovo4753", "metadata": {"taxonomy": ["Bacteria",
"__Chloroflexi", "__SAR202_clade", "__o", "__f", "__g"]}},{"id":
"denovo4755", "metadata": {"taxonomy": ["Bacteria",
"__Proteobacteria", "__Alphaproteobacteria", "__Rhodospirillales",
"__Rhodospirillaceae", "__Defluviicoccus"]}},{"id": "denovo4761",
"metadata": {"taxonomy": ["Bacteria", "__Bacteroidetes",
"__Flavobacteria", "__Flavobacteriales", "__Flavobacteriaceae",
"__Meridianimaribacter"]}},{"id": "denovo4765", "metadata":
{"taxonomy": ["Bacteria", "__Proteobacteria", "__Alphaproteobacteria",
"__Rhodobacterales", "__Rhodobacteraceae",
"__Roseobacter_clade_AS-21_lineage"]}},{"id": "denovo4768",
"metadata": {"taxonomy": ["Bacteria", "__Cyanobacteria",
"__ML635J-21", "__o", "__f", "__g"]}},{"id": "denovo4774", "metadata":
{"taxonomy": ["Bacteria", "__Acidobacteria", "__Acidobacteria",
"__BPC102", "__f", "__g"]}},{"id": "denovo4776", "metadata":
{"taxonomy": ["Bacteria", "__Bacteroidetes", "__Sphingobacteriia",

"__Sphingobacteriales", "__Saprospiraceae", "__g"]}},{"id":
"denovo4777", "metadata": {"taxonomy": ["Bacteria",
"__Proteobacteria", "__Alphaproteobacteria", "__Rhodobacterales",
"__Rhodobacteraceae", "__Pseudovibrio"]}},{"id": "denovo4778",
"metadata": {"taxonomy": ["Bacteria", "__Actinobacteria",
"__Acidimicrobiia", "__Acidimicrobiales"]}},{"id": "denovo4785",
"metadata": {"taxonomy": ["Bacteria", "__Proteobacteria",
"__Alphaproteobacteria", "__Rickettsiales", "__SAR116_clade",
"__g"]}},{"id": "denovo4786", "metadata": {"taxonomy": ["Bacteria",
"__Bacteroidetes", "__Flavobacteria", "__Flavobacteriales",
"__Flavobacteriaceae", "__Costertonia"]}},{"id": "denovo4788",
"metadata": {"taxonomy": ["Bacteria", "__Proteobacteria",
"__Alphaproteobacteria", "__Rhodobacterales", "__Rhodobacteraceae",
"__Roseobacter"]}},{"id": "denovo4789", "metadata": {"taxonomy":
["Bacteria", "__Proteobacteria", "__Gammaproteobacteria",
"__Oceanospirillales", "__J8P41000-1F04", "__g"]}},{"id":
"denovo4790", "metadata": {"taxonomy": ["Bacteria",
"__Proteobacteria", "__Alphaproteobacteria", "__Rickettsiales",
"__Anaplasmataceae", "__Candidatus_Xenohaliotis"]}},{"id":
"denovo4791", "metadata": {"taxonomy": ["Bacteria",
"__Proteobacteria", "__Alphaproteobacteria", "__Rhodobacterales",
"__Rhodobacteraceae", "__Maritimibacter"]}},{"id": "denovo4793",
"metadata": {"taxonomy": ["Bacteria", "__Proteobacteria",
"__Gammaproteobacteria", "__Pseudomonadales", "__Moraxellaceae",
"__Acinetobacter"]}},{"id": "denovo4795", "metadata": {"taxonomy":
["Bacteria", "__Cyanobacteria", "__Cyanobacteria", "__SubsectionIII",
"__FamilyI", "__Phormidium"]}},{"id": "denovo4799", "metadata":
{"taxonomy": ["Bacteria", "__Cyanobacteria", "__Cyanobacteria",
"__SubsectionI", "__FamilyI", "__Synechococcus"]}},{"id":
"denovo4805", "metadata": {"taxonomy": ["Bacteria",
"__Proteobacteria", "__Gammaproteobacteria", "__Oceanospirillales",
"__SS1-B-06-26", "__g"]}},{"id": "denovo4806", "metadata":
{"taxonomy": ["Bacteria", "__Proteobacteria", "__Gammaproteobacteria",
"__Alteromonadales", "__Alteromonadaceae", "__Haliea"]}},{"id":
"denovo4807", "metadata": {"taxonomy": ["Bacteria", "__Cyanobacteria",
"__Cyanobacteria", "__SubsectionIII", "__FamilyI"]}},{"id":
"denovo4813", "metadata": {"taxonomy": ["Bacteria",
"__Proteobacteria", "__Alphaproteobacteria", "__Rhodobacterales",
"__Rhodobacteraceae", "__Vadicella"]}},{"id": "denovo4814",
"metadata": {"taxonomy": ["Bacteria", "__Bacteroidetes",
"__Flavobacteria", "__Flavobacteriales", "__Flavobacteriaceae",
"__NS5_marine_group"]}},{"id": "denovo4816", "metadata": {"taxonomy":
["Bacteria", "__Proteobacteria", "__Deltaproteobacteria",
"__Desulfobacterales", "__Desulfobacteraceae",
"__Sva0081_sediment_group"]}},{"id": "denovo4819", "metadata":
{"taxonomy": ["Bacteria", "__Proteobacteria", "__Gammaproteobacteria",
"__BD7-8_marine_group", "__f", "__g"]}},{"id": "denovo4822",
"metadata": {"taxonomy": ["Bacteria", "__Actinobacteria",
"__Acidimicrobiia", "__Acidimicrobiales", "__Sva0996_marine_group",
"__g"]}},{"id": "denovo4825", "metadata": {"taxonomy": ["Bacteria",

"__Proteobacteria", "__Deltaproteobacteria", "__Sh765B-TzT-29", "__f", "__g"]}},{"id": "denovo4826", "metadata": {"taxonomy": ["Bacteria", "__Acidobacteria", "__Holophagae", "__CA002", "__f", "__g"]}},{"id": "denovo4828", "metadata": {"taxonomy": ["Bacteria", "__Proteobacteria", "__Alphaproteobacteria", "__Rhodospirillales", "__Rhodospirillaceae", "__Pelagibius"]}},{"id": "denovo4831", "metadata": {"taxonomy": ["Bacteria", "__Proteobacteria", "__Gammaproteobacteria", "__Methylococcales", "__Hyd24-01", "__g"]}},{"id": "denovo4833", "metadata": {"taxonomy": ["Bacteria", "__Proteobacteria", "__Alphaproteobacteria", "__Rhizobiales"]}},{"id": "denovo4834", "metadata": {"taxonomy": ["Bacteria", "__Proteobacteria", "__Gammaproteobacteria", "__Enterobacteriales", "__Enterobacteriaceae", "__Shimwellia"]}},{"id": "denovo4837", "metadata": {"taxonomy": ["Bacteria", "__Proteobacteria", "__Alphaproteobacteria", "__Rhodobacterales", "__Rhodobacteraceae", "__Roseobacter_clade_CHAB-I-5_lineage"]}},{"id": "denovo4840", "metadata": {"taxonomy": ["Bacteria", "__Proteobacteria", "__Alphaproteobacteria", "__Rhodospirillales", "__Rhodospirillaceae", "__Thalassospira"]}},{"id": "denovo4845", "metadata": {"taxonomy": ["Bacteria", "__Proteobacteria", "__Alphaproteobacteria", "__OCS116_clade", "__f", "__g"]}},{"id": "denovo4851", "metadata": {"taxonomy": ["Bacteria", "__Proteobacteria", "__Alphaproteobacteria", "__Rhodobacterales", "__Rhodobacteraceae", "__Labrenzia"]}},{"id": "denovo4855", "metadata": {"taxonomy": ["Bacteria", "__Proteobacteria", "__Deltaproteobacteria", "__Sh765B-TzT-29", "__f", "__g"]}},{"id": "denovo4857", "metadata": {"taxonomy": ["Bacteria", "__Proteobacteria", "__Alphaproteobacteria", "__Rhodospirillales", "__Rhodospirillaceae", "__Nisaea"]}},{"id": "denovo4858", "metadata": {"taxonomy": ["Bacteria", "__Actinobacteria", "__Acidimicrobiia", "__Acidimicrobiales", "__uncultured", "__g"]}},{"id": "denovo4868", "metadata": {"taxonomy": ["Bacteria", "__Proteobacteria", "__SC3-20", "__o", "__f", "__g"]}},{"id": "denovo4874", "metadata": {"taxonomy": ["Bacteria", "__Proteobacteria", "__Deltaproteobacteria", "__Bdellovibrionales", "__Bdellovibrionaceae", "__OM27_clade"]}},{"id": "denovo4875", "metadata": {"taxonomy": ["Archaea", "__Euryarchaeota", "__Halobacteria", "__Halobacteriales"]}},{"id": "denovo4878", "metadata": {"taxonomy": ["Bacteria", "__Bacteroidetes", "__Sphingobacteriia", "__Sphingobacteriales", "__Saprospiraceae", "__g"]}},{"id": "denovo4879", "metadata": {"taxonomy": ["Bacteria", "__Proteobacteria", "__Alphaproteobacteria", "__Rhizobiales", "__Brucellaceae", "__g"]}},{"id": "denovo4880", "metadata": {"taxonomy": ["Bacteria", "__Proteobacteria", "__Alphaproteobacteria", "__Rhodobacterales", "__Rhodobacteraceae"]}},{"id": "denovo4889", "metadata": {"taxonomy": ["Bacteria", "__Proteobacteria", "__Deltaproteobacteria"]}},{"id": "denovo4902", "metadata": {"taxonomy": ["Bacteria", "__Proteobacteria", "__Deltaproteobacteria", "__Myxococcales", "__Cystobacterineae", "__g"]}},{"id": "denovo4906", "metadata": {"taxonomy": ["Bacteria", "__Proteobacteria", "__Deltaproteobacteria", "__Desulfobacterales", "__Desulfobacteraceae", "__g"]}},{"id": "denovo4911", "metadata":

{"taxonomy": ["Bacteria", "__Planctomycetes", "__Phycisphaerae", "__Phycisphaerales", "__Phycisphaeraceae", "__SM1A02"]}},{"id": "denovo4912", "metadata": {"taxonomy": ["Bacteria", "__Proteobacteria", "__Deltaproteobacteria", "__Desulfobacterales", "__Desulfobacteraceae", "__Desulfobacula"]}},{"id": "denovo4920", "metadata": {"taxonomy": ["Bacteria", "__Proteobacteria", "__Gammaproteobacteria", "__Vibrionales", "__Vibrionaceae", "__Vibrio"]}},{"id": "denovo4923", "metadata": {"taxonomy": ["Bacteria", "__TA06", "__c", "__o", "__f", "__g"]}},{"id": "denovo4925", "metadata": {"taxonomy": ["Bacteria", "__Proteobacteria", "__Alphaproteobacteria", "__Rhodobacterales", "__Rhodobacteraceae"]}},{"id": "denovo4926", "metadata": {"taxonomy": ["Bacteria", "__Planctomycetes", "__Planctomycetacia", "__Planctomycetales", "__Planctomycetaceae", "__g"]}},{"id": "denovo4927", "metadata": {"taxonomy": ["Bacteria", "__Proteobacteria", "__Gammaproteobacteria", "__Order_Incertae_Sedis", "__Family_Incertae_Sedis", "__Marinicella"]}},{"id": "denovo4928", "metadata": {"taxonomy": ["Bacteria", "__Chloroflexi", "__Caldilineae", "__Caldilineales", "__Caldilineaceae", "__g"]}},{"id": "denovo4932", "metadata": {"taxonomy": ["Bacteria", "__Proteobacteria", "__Gammaproteobacteria", "__Legionellales", "__Coxiellaceae", "__Coxiella"]}},{"id": "denovo4935", "metadata": {"taxonomy": ["Bacteria", "__Proteobacteria", "__Alphaproteobacteria", "__Rickettsiales", "__TK34", "__g"]}},{"id": "denovo4937", "metadata": {"taxonomy": ["Bacteria", "__Bacteroidetes"]}},{"id": "denovo4939", "metadata": {"taxonomy": ["Bacteria", "__Bacteroidetes", "__Flavobacteria", "__Flavobacteriales", "__Flavobacteriaceae", "__Gilvibacter"]}},{"id": "denovo4941", "metadata": {"taxonomy": ["Bacteria", "__Proteobacteria", "__Gammaproteobacteria", "__Chromatiales", "__Granulosicoccaceae", "__Granulosicoccus"]}},{"id": "denovo4942", "metadata": {"taxonomy": ["Bacteria", "__Proteobacteria", "__Gammaproteobacteria", "__Alteromonadales", "__Colwelliaceae", "__Thalassomonas"]}},{"id": "denovo4943", "metadata": {"taxonomy": ["Bacteria", "__Proteobacteria", "__Gammaproteobacteria", "__Alteromonadales", "__Alteromonadaceae", "__Haliea"]}},{"id": "denovo4944", "metadata": {"taxonomy": ["Bacteria", "__Proteobacteria", "__Alphaproteobacteria", "__Rhodospirillales", "__Rhodospirillaceae", "__Fodinicurvata"]}},{"id": "denovo4945", "metadata": {"taxonomy": ["Bacteria", "__Gemmatimonadetes", "__Gemmatimonadetes", "__BD2-11_terrestrial_group", "__f", "__g"]}},{"id": "denovo4946", "metadata": {"taxonomy": ["Bacteria", "__Proteobacteria", "__Gammaproteobacteria", "__Order_Incertae_Sedis", "__Family_Incertae_Sedis", "__Marinicella"]}},{"id": "denovo4950", "metadata": {"taxonomy": ["Bacteria", "__Verrucomicrobia", "__Verrucomicrobiae", "__Verrucomicrobiales", "__Verrucomicrobiaceae", "__Roseibacillus"]}},{"id": "denovo4953", "metadata": {"taxonomy": ["Bacteria", "__Proteobacteria", "__Gammaproteobacteria", "__Alteromonadales", "__Alteromonadaceae", "__g"]}},{"id": "denovo4955", "metadata": {"taxonomy": ["Bacteria", "__Chloroflexi",

"__SAR202_clade", "__o", "__f", "__g"]}},{"id": "denovo4956", "metadata": {"taxonomy": ["Bacteria", "__Proteobacteria", "__Deltaproteobacteria", "__Bdellovibrionales", "__Bdellovibrionaceae", "__Bdellovibrio"]}},{"id": "denovo4957", "metadata": {"taxonomy": ["Bacteria", "__Proteobacteria", "__Gammaproteobacteria", "__Oceanospirillales", "__Oceanospirillaceae", "__Pseudospirillum"]}},{"id": "denovo4961", "metadata": {"taxonomy": ["Bacteria", "__Proteobacteria", "__Alphaproteobacteria", "__4-Org1-14", "__f", "__g"]}},{"id": "denovo4968", "metadata": {"taxonomy": ["Bacteria", "__Proteobacteria", "__Gammaproteobacteria", "__Order_Incertae_Sedis", "__Family_Incertae_Sedis", "__Marinicella"]}},{"id": "denovo4970", "metadata": {"taxonomy": ["Bacteria", "__Bacteroidetes", "__Sphingobacteriia", "__Sphingobacteriales", "__Saprospiraceae", "__g"]}},{"id": "denovo4973", "metadata": {"taxonomy": ["Bacteria", "__Proteobacteria", "__Deltaproteobacteria", "__Desulfuromonadales", "__Sva1033", "__g"]}},{"id": "denovo4978", "metadata": {"taxonomy": ["Bacteria", "__Chlorobi", "__Chlorobia", "__Chlorobiales", "__Chlorobiaceae", "__Prosthecochloris"]}},{"id": "denovo4980", "metadata": {"taxonomy": ["Bacteria", "__Proteobacteria", "__Alphaproteobacteria", "__Rhodospirillales", "__Rhodospirillaceae"]}},{"id": "denovo4987", "metadata": {"taxonomy": ["Bacteria", "__Proteobacteria", "__Gammaproteobacteria", "__Vibrionales", "__Vibrionaceae", "__Photobacterium"]}},{"id": "denovo4988", "metadata": {"taxonomy": ["Bacteria", "__Proteobacteria", "__Gammaproteobacteria", "__Xanthomonadales", "__Sinobacteraceae", "__JTB255_marine_benthic_group"]}},{"id": "denovo4992", "metadata": {"taxonomy": ["Bacteria", "__Proteobacteria", "__Alphaproteobacteria", "__Rhizobiales", "__Phyllobacteriaceae"]}},{"id": "denovo4995", "metadata": {"taxonomy": ["Bacteria", "__Proteobacteria", "__Alphaproteobacteria", "__Rhodobacterales", "__Rhodobacteraceae"]}},{"id": "denovo4996", "metadata": {"taxonomy": ["Bacteria", "__Proteobacteria", "__Gammaproteobacteria", "__Xanthomonadales", "__Sinobacteraceae", "__JTB255_marine_benthic_group"]}},{"id": "denovo4999", "metadata": {"taxonomy": ["Bacteria", "__Proteobacteria", "__Alphaproteobacteria", "__Rhodospirillales", "__Rhodospirillaceae", "__g"]}},{"id": "denovo5001", "metadata": {"taxonomy": ["Bacteria", "__Proteobacteria", "__Alphaproteobacteria", "__Rhodospirillales"]}},{"id": "denovo5007", "metadata": {"taxonomy": ["Bacteria", "__Proteobacteria", "__Gammaproteobacteria", "__Alteromonadales", "__Alteromonadaceae", "__Candidatus_Endobugula"]}},{"id": "denovo5008", "metadata": {"taxonomy": ["Bacteria", "__Bacteroidetes", "__Sphingobacteriia", "__Sphingobacteriales", "__Chitinophagaceae", "__g"]}},{"id": "denovo5015", "metadata": {"taxonomy": ["Bacteria", "__Candidate_division_BRC1", "__c", "__o", "__f", "__g"]}},{"id": "denovo5016", "metadata": {"taxonomy": ["Bacteria", "__Bacteroidetes", "__Flavobacteria", "__Flavobacteriales", "__Cryomorphaceae", "__Owenweeksia"]}},{"id": "denovo5017", "metadata": {"taxonomy": ["Bacteria", "__Cyanobacteria", "__Cyanobacteria"]}},{"id":

"denovo5021", "metadata": {"taxonomy": ["Bacteria", "__Proteobacteria", "__Alphaproteobacteria", "__Rhodobacterales", "__Rhodobacteraceae", "__Roseobacter_clade_NAC11-7_lineage"]}},{"id": "denovo5022", "metadata": {"taxonomy": ["Bacteria", "__Proteobacteria", "__Alphaproteobacteria", "__Rickettsiales", "__S25-593", "__g"]}},{"id": "denovo5025", "metadata": {"taxonomy": ["Bacteria", "__Proteobacteria", "__Alphaproteobacteria", "__Rhodospirillales", "__Rhodospirillaceae", "__Thalassospira"]}},{"id": "denovo5027", "metadata": {"taxonomy": ["Bacteria", "__Proteobacteria", "__Alphaproteobacteria", "__Rhodospirillales", "__Rhodospirillaceae", "__Tistlia"]}},{"id": "denovo5028", "metadata": {"taxonomy": ["Bacteria", "__Proteobacteria", "__Alphaproteobacteria", "__Rhizobiales", "__Rhodobiaceae", "__Anderseniella"]}},{"id": "denovo5029", "metadata": {"taxonomy": ["Bacteria", "__Proteobacteria", "__Alphaproteobacteria"]}},{"id": "denovo5031", "metadata": {"taxonomy": ["Bacteria", "__Proteobacteria", "__Alphaproteobacteria", "__Rhizobiales", "__Phyllobacteriaceae"]}},{"id": "denovo5034", "metadata": {"taxonomy": ["Bacteria", "__Proteobacteria"]}},{"id": "denovo5044", "metadata": {"taxonomy": ["Bacteria"]}},{"id": "denovo5045", "metadata": {"taxonomy": ["Bacteria", "__Candidate_division_WS3", "__c", "__o", "__f", "__g"]}},{"id": "denovo5047", "metadata": {"taxonomy": ["Bacteria", "__Bacteroidetes", "__Flavobacteria", "__Flavobacteriales", "__Flavobacteriaceae", "__Tenacibaculum"]}},{"id": "denovo5054", "metadata": {"taxonomy": ["Bacteria", "__Proteobacteria", "__Gammaproteobacteria", "__Alteromonadales", "__Alteromonadaceae", "__g"]}},{"id": "denovo5056", "metadata": {"taxonomy": ["Bacteria", "__Cyanobacteria", "__Cyanobacteria", "__uncultured", "__f", "__g"]}},{"id": "denovo5057", "metadata": {"taxonomy": ["Bacteria", "__Proteobacteria", "__TA18", "__o", "__f", "__g"]}},{"id": "denovo5058", "metadata": {"taxonomy": ["Bacteria", "__Cyanobacteria", "__Cyanobacteria", "__SubsectionI", "__FamilyI", "__Synechococcus"]}},{"id": "denovo5059", "metadata": {"taxonomy": ["Bacteria", "__Proteobacteria", "__Gammaproteobacteria", "__Xanthomonadales", "__Sinobacteraceae", "__JTB255_marine_benthic_group"]}},{"id": "denovo5060", "metadata": {"taxonomy": ["Bacteria"]}},{"id": "denovo5061", "metadata": {"taxonomy": ["Bacteria", "__Proteobacteria", "__Gammaproteobacteria", "__Pseudomonadales", "__Moraxellaceae", "__Acinetobacter"]}},{"id": "denovo5064", "metadata": {"taxonomy": ["Bacteria", "__Firmicutes", "__Clostridia", "__Clostridiales", "__Clostridiaceae", "__Geosporobacter"]}},{"id": "denovo5068", "metadata": {"taxonomy": ["Bacteria", "__Verrucomicrobia", "__Verrucomicrobiae", "__Verrucomicrobiales", "__Verrucomicrobiaceae", "__g"]}},{"id": "denovo5075", "metadata": {"taxonomy": ["Bacteria", "__Proteobacteria", "__Gammaproteobacteria", "__BD7-8_marine_group", "__f", "__g"]}},{"id": "denovo5077", "metadata": {"taxonomy": ["Bacteria", "__Proteobacteria", "__Gammaproteobacteria", "__Chromatiales", "__Chromatiaceae", "__Nitrosococcus"]}},{"id": "denovo5085", "metadata": {"taxonomy": ["Bacteria", "__Proteobacteria", "__Gammaproteobacteria",

"__Chromatiales", "__Chromatiaceae"]}},{"id": "denovo5086", "metadata": {"taxonomy": ["Bacteria", "__Candidate_division_WS3", "__c", "__o", "__f", "__g"]}},{"id": "denovo5089", "metadata": {"taxonomy": ["Bacteria", "__Proteobacteria", "__Gammaproteobacteria", "__HOC36", "__f", "__g"]}},{"id": "denovo5090", "metadata": {"taxonomy": ["Bacteria", "__Proteobacteria", "__Deltaproteobacteria", "__Desulfuromonadales", "__GR-WP33-58", "__g"]}},{"id": "denovo5093", "metadata": {"taxonomy": ["Bacteria", "__Proteobacteria", "__Alphaproteobacteria", "__Rhizobiales", "__Rhodobiaceae", "__Rhodobium"]}},{"id": "denovo5095", "metadata": {"taxonomy": ["Bacteria", "__Proteobacteria", "__Gammaproteobacteria", "__Oceanospirillales", "__Oceanospirillaceae", "__Reinekea"]}},{"id": "denovo5096", "metadata": {"taxonomy": ["Bacteria", "__Proteobacteria", "__Deltaproteobacteria", "__Myxococcales", "__Nannocystineae", "__Nannocystaceae"]}},{"id": "denovo5098", "metadata": {"taxonomy": ["Bacteria", "__Proteobacteria", "__Gammaproteobacteria"]}},{"id": "denovo5099", "metadata": {"taxonomy": ["Bacteria", "__Proteobacteria", "__Gammaproteobacteria", "__Order_Incertae_Sedis", "__Family_Incertae_Sedis", "__Marinicella"]}},{"id": "denovo5100", "metadata": {"taxonomy": ["Bacteria", "__Proteobacteria", "__Betaproteobacteria"]}},{"id": "denovo5102", "metadata": {"taxonomy": ["Bacteria", "__Bacteroidetes", "__Flavobacteria", "__Flavobacteriales", "__Flavobacteriaceae", "__NS5_marine_group"]}},{"id": "denovo5109", "metadata": {"taxonomy": ["Bacteria", "__Chlorobi", "__Chlorobia", "__Chlorobiales", "__Chlorobiaceae", "__Prosthecochloris"]}},{"id": "denovo5115", "metadata": {"taxonomy": ["Bacteria", "__Proteobacteria", "__Gammaproteobacteria", "__Vibrionales", "__Vibrionaceae", "__Vibrio"]}},{"id": "denovo5117", "metadata": {"taxonomy": ["Bacteria", "__Proteobacteria", "__Alphaproteobacteria", "__Rickettsiales", "__LWSR-14", "__g"]}},{"id": "denovo5118", "metadata": {"taxonomy": ["Bacteria", "__Bacteroidetes", "__Cytophagia", "__Cytophagales", "__Flammeovirgaceae", "__Fulvivirga"]}},{"id": "denovo5121", "metadata": {"taxonomy": ["Bacteria", "__Chlorobi", "__Chlorobia", "__Chlorobiales", "__Chlorobiaceae", "__Prosthecochloris"]}},{"id": "denovo5125", "metadata": {"taxonomy": ["Bacteria", "__Proteobacteria", "__Deltaproteobacteria", "__Desulfobacterales", "__Desulfobacteraceae", "__Desulfosarcina"]}},{"id": "denovo5137", "metadata": {"taxonomy": ["Bacteria", "__Proteobacteria", "__Gammaproteobacteria", "__Sva0071", "__f", "__g"]}},{"id": "denovo5140", "metadata": {"taxonomy": ["Bacteria", "__Proteobacteria", "__Alphaproteobacteria", "__Rhodospirillales", "__Rhodospirillaceae"]}},{"id": "denovo5143", "metadata": {"taxonomy": ["Bacteria", "__Bacteroidetes", "__Flavobacteria", "__Flavobacteriales", "__Flavobacteriaceae", "__Gilvibacter"]}},{"id": "denovo5148", "metadata": {"taxonomy": ["Bacteria", "__Proteobacteria", "__Alphaproteobacteria", "__Rhizobiales", "__Rhodobiaceae"]}},{"id": "denovo5154", "metadata": {"taxonomy": ["Bacteria", "__Proteobacteria", "__Gammaproteobacteria",

"__Xanthomonadales", "__Sinobacteraceae",
"__JTB255_marine_benthic_group"]}},{"id": "denovo5156", "metadata":
{"taxonomy": ["Bacteria", "__Proteobacteria",
"__Gammaproteobacteria"]}},{"id": "denovo5159", "metadata":
{"taxonomy": ["Bacteria", "__Proteobacteria", "__Deltaproteobacteria",
"__Bdellovibrionales", "__Bacteriovoracaceae", "__Peredibacter"]}},
{"id": "denovo5160", "metadata": {"taxonomy": ["Bacteria",
"__Bacteroidetes", "__Flavobacteria", "__Flavobacteriales",
"__Cryomorphaceae", "__Fluviicola"]}},{"id": "denovo5161", "metadata":
{"taxonomy": ["Bacteria", "__Proteobacteria", "__Gammaproteobacteria",
"__Chromatiales", "__Granulosicoccaceae", "__Granulosicoccus"]}},
{"id": "denovo5166", "metadata": {"taxonomy": ["Bacteria",
"__Proteobacteria", "__Gammaproteobacteria",
"__E01-9C-26_marine_group", "__f", "__g"]}},{"id": "denovo5171",
"metadata": {"taxonomy": ["Bacteria", "__Proteobacteria",
"__Alphaproteobacteria", "__OCS116_clade", "__f", "__g"]}},{"id":
"denovo5172", "metadata": {"taxonomy": ["Bacteria",
"__Proteobacteria", "__TA18", "__o", "__f", "__g"]}},{"id":
"denovo5176", "metadata": {"taxonomy": ["Bacteria", "__Bacteroidetes",
"__Flavobacteria", "__Flavobacteriales", "__Flavobacteriaceae"]}},
{"id": "denovo5177", "metadata": {"taxonomy": ["Bacteria",
"__Proteobacteria", "__Deltaproteobacteria", "__Sh765B-TzT-29", "__f",
"__g"]}},{"id": "denovo5179", "metadata": {"taxonomy": ["Bacteria",
"__Cyanobacteria", "__Cyanobacteria", "__SubsectionIII", "__FamilyI",
"__Phormidium"]}},{"id": "denovo5185", "metadata": {"taxonomy":
["Bacteria", "__Proteobacteria", "__Alphaproteobacteria", "__DB1-14",
"__f", "__g"]}},{"id": "denovo5189", "metadata": {"taxonomy":
["Bacteria", "__Proteobacteria", "__Alphaproteobacteria"]}},{"id":
"denovo5194", "metadata": {"taxonomy": ["Bacteria",
"__Proteobacteria", "__Betaproteobacteria", "__Rhodocyclales",
"__Rhodocyclaceae", "__Sulfuritalea"]}},{"id": "denovo5195",
"metadata": {"taxonomy": ["Bacteria", "__Proteobacteria",
"__Gammaproteobacteria", "__Alteromonadales", "__Alteromonadaceae"]}},
{"id": "denovo5202", "metadata": {"taxonomy": ["Bacteria",
"__Proteobacteria", "__Gammaproteobacteria", "__Alteromonadales",
"__Alteromonadaceae", "__OM60(NOR5)_clade"]}},{"id": "denovo5207",
"metadata": {"taxonomy": ["Bacteria", "__Proteobacteria",
"__Alphaproteobacteria"]}},{"id": "denovo5211", "metadata":
{"taxonomy": ["Bacteria", "__Proteobacteria", "__Alphaproteobacteria",
"__Rhizobiales", "__Rhodobiaceae", "__Rhodobium"]}},{"id":
"denovo5215", "metadata": {"taxonomy": ["Bacteria",
"__Proteobacteria", "__Gammaproteobacteria", "__Order_Incertae_Sedis",
"__Family_Incertae_Sedis", "__Marinicella"]}},{"id": "denovo5218",
"metadata": {"taxonomy": ["Bacteria", "__Proteobacteria",
"__Deltaproteobacteria", "__Desulfobacterales",
"__Desulfobacteraceae", "__SEEP-SRB1"]}},{"id": "denovo5220",
"metadata": {"taxonomy": ["Bacteria", "__Proteobacteria",
"__Deltaproteobacteria", "__Sh765B-TzT-29", "__f", "__g"]}},{"id":
"denovo5221", "metadata": {"taxonomy": ["Bacteria",
"__Proteobacteria", "__Alphaproteobacteria", "__Rhodobacterales",

"__Rhodobacteraceae", "__Roseobacter_clade_NAC11-7_lineage"]}},{"id": "denovo5223", "metadata": {"taxonomy": ["Bacteria", "__Proteobacteria", "__Alphaproteobacteria", "__Rhizobiales", "__F0723", "__g"]}},{"id": "denovo5226", "metadata": {"taxonomy": ["Bacteria", "__Proteobacteria", "__Alphaproteobacteria", "__Rhodospirillales", "__Rhodospirillaceae", "__g"]}},{"id": "denovo5236", "metadata": {"taxonomy": ["Bacteria", "__Proteobacteria", "__Alphaproteobacteria", "__Rhodobacterales", "__Rhodobacteraceae", "__Pseudovibrio"]}},{"id": "denovo5237", "metadata": {"taxonomy": ["Bacteria", "__Acidobacteria", "__Holophagae"]}},{"id": "denovo5240", "metadata": {"taxonomy": ["Bacteria", "__Proteobacteria", "__Alphaproteobacteria", "__Rhizobiales", "__Rhodobiaceae", "__Rhodobium"]}},{"id": "denovo5242", "metadata": {"taxonomy": ["Bacteria", "__Proteobacteria", "__Gammaproteobacteria", "__Oceanospirillales", "__Hahellaceae", "__Endozoicomonas"]}},{"id": "denovo5243", "metadata": {"taxonomy": ["Bacteria", "__Proteobacteria", "__Alphaproteobacteria"]}},{"id": "denovo5244", "metadata": {"taxonomy": ["Bacteria", "__Proteobacteria", "__Gammaproteobacteria", "__Oceanospirillales", "__Oceanospirillaceae", "__Pseudospirillum"]}},{"id": "denovo5247", "metadata": {"taxonomy": ["Bacteria", "__Acidobacteria", "__RB25", "__o", "__f", "__g"]}},{"id": "denovo5248", "metadata": {"taxonomy": ["Bacteria", "__Proteobacteria", "__Alphaproteobacteria", "__Rhodobacterales", "__Rhodobacteraceae"]}},{"id": "denovo5250", "metadata": {"taxonomy": ["Bacteria", "__Proteobacteria", "__Alphaproteobacteria", "__Rhizobiales", "__Phyllobacteriaceae"]}},{"id": "denovo5251", "metadata": {"taxonomy": ["Bacteria", "__Proteobacteria", "__Gammaproteobacteria", "__Pseudomonadales", "__Moraxellaceae", "__Acinetobacter"]}},{"id": "denovo5254", "metadata": {"taxonomy": ["Bacteria", "__Proteobacteria", "__Deltaproteobacteria", "__Bdellovibrionales", "__Bdellovibrionaceae", "__Bdellovibrio"]}},{"id": "denovo5256", "metadata": {"taxonomy": ["Bacteria", "__Proteobacteria", "__Alphaproteobacteria", "__Caulobacterales", "__Hyphomonadaceae"]}},{"id": "denovo5257", "metadata": {"taxonomy": ["Bacteria", "__Gemmatimonadetes", "__Gemmatimonadetes", "__BD2-11_terrestrial_group", "__f", "__g"]}},{"id": "denovo5259", "metadata": {"taxonomy": ["Bacteria", "__Bacteroidetes", "__Sphingobacteriia", "__Sphingobacteriales", "__Saprospiraceae", "__g"]}},{"id": "denovo5260", "metadata": {"taxonomy": ["Bacteria", "__Planctomycetes", "__Planctomycetacia", "__Planctomycetales", "__Planctomycetaceae", "__Rhodopirellula"]}},{"id": "denovo5263", "metadata": {"taxonomy": ["Bacteria", "__Proteobacteria", "__Alphaproteobacteria", "__Rhodobacterales", "__Rhodobacteraceae", "__Rhodothalassium"]}},{"id": "denovo5264", "metadata": {"taxonomy": ["Bacteria", "__Acidobacteria", "__Holophagae", "__Sva0725", "__f", "__g"]}},{"id": "denovo5265", "metadata": {"taxonomy": ["Bacteria", "__Proteobacteria", "__Gammaproteobacteria", "__Alteromonadales", "__Alteromonadaceae", "__BD1-7_clade"]}},{"id": "denovo5266", "metadata": {"taxonomy": ["Bacteria", "__Actinobacteria",

"__Acidimicrobiia", "__Acidimicrobiales", "__OCS155_marine_group", "__g"]}},{"id": "denovo5267", "metadata": {"taxonomy": ["Bacteria", "__Proteobacteria", "__Alphaproteobacteria"]}},{"id": "denovo5268", "metadata": {"taxonomy": ["Bacteria", "__Actinobacteria", "__Acidimicrobiia", "__Acidimicrobiales", "__OCS155_marine_group", "__g"]}},{"id": "denovo5272", "metadata": {"taxonomy": ["Bacteria", "__Proteobacteria", "__Gammaproteobacteria", "__Oceanospirillales", "__E12-CR02-full", "__g"]}},{"id": "denovo5273", "metadata": {"taxonomy": ["Bacteria", "__Bacteroidetes", "__Sphingobacteriia", "__Sphingobacteriales", "__Saprospiraceae", "__g"]}},{"id": "denovo5277", "metadata": {"taxonomy": ["Bacteria", "__Proteobacteria", "__Gammaproteobacteria", "__Alteromonadales", "__Alteromonadaceae", "__Umboniibacter"]}},{"id": "denovo5278", "metadata": {"taxonomy": ["Archaea", "__Euryarchaeota", "__Thermoplasmata", "__Thermoplasmatales", "__Marine_Group_II", "__g"]}},{"id": "denovo5281", "metadata": {"taxonomy": ["Bacteria", "__Bacteroidetes", "__Flavobacteria", "__Flavobacteriales", "__Cryomorphaceae", "__Owenweeksia"]}},{"id": "denovo5286", "metadata": {"taxonomy": ["Bacteria", "__Proteobacteria", "__Gammaproteobacteria", "__Legionellales", "__Coxiellaceae", "__Coxiella"]}},{"id": "denovo5287", "metadata": {"taxonomy": ["Bacteria", "__Bacteroidetes", "__Flavobacteria", "__Flavobacteriales", "__Flavobacteriaceae", "__g"]}},{"id": "denovo5289", "metadata": {"taxonomy": ["Bacteria", "__Proteobacteria", "__Alphaproteobacteria", "__Rhodobacterales", "__Rhodobacteraceae"]}},{"id": "denovo5290", "metadata": {"taxonomy": ["Bacteria", "__Proteobacteria", "__Deltaproteobacteria", "__Sh765B-TzT-29", "__f", "__g"]}},{"id": "denovo5293", "metadata": {"taxonomy": ["Bacteria", "__Proteobacteria"]}},{"id": "denovo5295", "metadata": {"taxonomy": ["Bacteria", "__Planctomycetes", "__BD7-11", "__o", "__f", "__g"]}},{"id": "denovo5299", "metadata": {"taxonomy": ["Bacteria", "__Proteobacteria", "__Gammaproteobacteria", "__Alteromonadales", "__Alteromonadaceae", "__Haliea"]}},{"id": "denovo5303", "metadata": {"taxonomy": ["Bacteria", "__Proteobacteria", "__Alphaproteobacteria", "__Rhizobiales", "__Rhodobiaceae", "__Anderseniella"]}},{"id": "denovo5304", "metadata": {"taxonomy": ["Bacteria", "__Proteobacteria", "__Gammaproteobacteria", "__HOC36", "__f", "__g"]}},{"id": "denovo5306", "metadata": {"taxonomy": ["Bacteria", "__Proteobacteria", "__Deltaproteobacteria", "__Myxococcales", "__Sorangiineae", "__Sandaracinaceae"]}},{"id": "denovo5316", "metadata": {"taxonomy": ["Bacteria", "__Planctomycetes", "__Planctomycetacia", "__Planctomycetales", "__Planctomycetaceae", "__Planctomyces"]}},{"id": "denovo5321", "metadata": {"taxonomy": ["Bacteria", "__Proteobacteria", "__Alphaproteobacteria"]}},{"id": "denovo5322", "metadata": {"taxonomy": ["Bacteria", "__Proteobacteria", "__Gammaproteobacteria", "__Chromatiales", "__Chromatiaceae", "__Nitrosococcus"]}},{"id": "denovo5323", "metadata": {"taxonomy": ["Bacteria", "__Proteobacteria", "__Gammaproteobacteria", "__E01-9C-26_marine_group", "__f", "__g"]}},

{"id": "denovo5324", "metadata": {"taxonomy": ["Bacteria", "__Bacteroidetes", "__Cytophagia", "__Order_II_Incertae_Sedis", "__Rhodothermaceae", "__g"]}},{"id": "denovo5325", "metadata": {"taxonomy": ["Bacteria", "__Proteobacteria", "__Gammaproteobacteria"]}},{"id": "denovo5329", "metadata": {"taxonomy": ["Bacteria", "__Acidobacteria", "__Acidobacteria", "__BPC015", "__f", "__g"]}},{"id": "denovo5331", "metadata": {"taxonomy": ["Bacteria", "__Proteobacteria", "__Deltaproteobacteria", "__Desulfobacterales", "__Desulfobacteraceae", "__Desulfofrigus"]}},{"id": "denovo5332", "metadata": {"taxonomy": ["Bacteria", "__Proteobacteria", "__Alphaproteobacteria", "__Rhodospirillales", "__Rhodospirillaceae", "__g"]}},{"id": "denovo5333", "metadata": {"taxonomy": ["Bacteria", "__Proteobacteria", "__Deltaproteobacteria", "__SAR324_clade(Marine_group_B)", "__f", "__g"]}},{"id": "denovo5339", "metadata": {"taxonomy": ["Bacteria", "__Acidobacteria", "__Holophagae", "__CA002", "__f", "__g"]}},{"id": "denovo5345", "metadata": {"taxonomy": ["Bacteria", "__Bacteroidetes", "__Flavobacteria", "__Flavobacteriales", "__Flavobacteriaceae", "__g"]}},{"id": "denovo5346", "metadata": {"taxonomy": ["Bacteria", "__Verrucomicrobia", "__Verrucomicrobiae", "__Verrucomicrobiales", "__DEV007", "__g"]}},{"id": "denovo5347", "metadata": {"taxonomy": ["Bacteria", "__Proteobacteria", "__Gammaproteobacteria"]}},{"id": "denovo5349", "metadata": {"taxonomy": ["Bacteria", "__Proteobacteria"]}},{"id": "denovo5354", "metadata": {"taxonomy": ["Bacteria", "__Proteobacteria", "__Alphaproteobacteria", "__Rhizobiales", "__Phyllobacteriaceae"]}},{"id": "denovo5357", "metadata": {"taxonomy": ["Bacteria", "__Proteobacteria", "__Gammaproteobacteria", "__Oceanospirillales", "__Oceanospirillaceae", "__Oceanospirillum"]}},{"id": "denovo5370", "metadata": {"taxonomy": ["Bacteria", "__Proteobacteria", "__Gammaproteobacteria", "__E01-9C-26_marine_group", "__f", "__g"]}},{"id": "denovo5374", "metadata": {"taxonomy": ["Bacteria", "__Chloroflexi", "__SAR202_clade", "__o", "__f", "__g"]}},{"id": "denovo5376", "metadata": {"taxonomy": ["Bacteria", "__Actinobacteria", "__Acidimicrobiia", "__Acidimicrobiales", "__Sva0996_marine_group", "__g"]}},{"id": "denovo5378", "metadata": {"taxonomy": ["Bacteria"]}},{"id": "denovo5380", "metadata": {"taxonomy": ["Bacteria", "__Proteobacteria", "__Gammaproteobacteria", "__Oceanospirillales", "__SS1-B-06-26", "__g"]}},{"id": "denovo5382", "metadata": {"taxonomy": ["Bacteria", "__Proteobacteria", "__Gammaproteobacteria", "__Xanthomonadales", "__Sinobacteraceae", "__JTB255_marine_benthic_group"]}},{"id": "denovo5386", "metadata": {"taxonomy": ["Bacteria", "__Proteobacteria", "__Alphaproteobacteria", "__Caulobacterales", "__Hyphomonadaceae", "__g"]}},{"id": "denovo5390", "metadata": {"taxonomy": ["Bacteria", "__Proteobacteria", "__Gammaproteobacteria", "__NKB5", "__f", "__g"]}},{"id": "denovo5394", "metadata": {"taxonomy": ["Bacteria"]}},{"id": "denovo5396", "metadata": {"taxonomy": ["Bacteria", "__Proteobacteria", "__Gammaproteobacteria", "__KI89A_clade", "__f", "__g"]}},{"id": "denovo5407", "metadata": {"taxonomy": ["Bacteria",

"__Bacteroidetes", "__Flavobacteria", "__Flavobacteriales",
"__Cryomorphaceae", "__Owenweeksia"]}},{"id": "denovo5409",
"metadata": {"taxonomy": ["Bacteria", "__Verrucomicrobia",
"__Verrucomicrobiae", "__Verrucomicrobiales", "__Verrucomicrobiaceae",
"__Roseibacillus"]}},{"id": "denovo5410", "metadata": {"taxonomy":
["Bacteria", "__Cyanobacteria", "__Cyanobacteria", "__SubsectionIII",
"__FamilyI"]}},{"id": "denovo5411", "metadata": {"taxonomy":
["Bacteria", "__Proteobacteria", "__Gammaproteobacteria"]}},{"id":
"denovo5416", "metadata": {"taxonomy": ["Bacteria", "__Acidobacteria",
"__Holophagae", "__CA002", "__f", "__g"]}},{"id": "denovo5421",
"metadata": {"taxonomy": ["Bacteria", "__Proteobacteria",
"__Gammaproteobacteria", "__Xanthomonadales", "__Sinobacteraceae",
"__JTB255_marine_benthic_group"]}},{"id": "denovo5422", "metadata":
{"taxonomy": ["Bacteria", "__Bacteroidetes", "__Flavobacteria",
"__Flavobacteriales", "__Cryomorphaceae", "__Owenweeksia"]}},{"id":
"denovo5424", "metadata": {"taxonomy": ["Bacteria", "__Bacteroidetes",
"__Cytophagia", "__Cytophagales", "__Flammeovirgaceae"]}},{"id":
"denovo5430", "metadata": {"taxonomy": ["Bacteria",
"__Proteobacteria", "__Alphaproteobacteria", "__DB1-14", "__f",
"__g"]}},{"id": "denovo5433", "metadata": {"taxonomy": ["Bacteria",
"__Firmicutes", "__Clostridia", "__Clostridiales",
"__Family_XII_Incertae_Sedis", "__Fusibacter"]}},{"id": "denovo5437",
"metadata": {"taxonomy": ["Bacteria", "__Proteobacteria",
"__Alphaproteobacteria", "__ss1-B-07-44", "__f", "__g"]}},{"id":
"denovo5441", "metadata": {"taxonomy": ["Bacteria",
"__Proteobacteria", "__Alphaproteobacteria", "__Rhodospirillales",
"__Rhodospirillaceae", "__Thalassospira"]}},{"id": "denovo5443",
"metadata": {"taxonomy": ["Bacteria", "__Proteobacteria",
"__Alphaproteobacteria", "__Rickettsiales"]}},{"id": "denovo5444",
"metadata": {"taxonomy": ["Bacteria", "__Proteobacteria",
"__Gammaproteobacteria", "__Alteromonadales", "__Alteromonadaceae",
"__C1-B045"]}},{"id": "denovo5445", "metadata": {"taxonomy":
["Bacteria", "__Proteobacteria", "__Alphaproteobacteria"]}},{"id":
"denovo5447", "metadata": {"taxonomy": ["Bacteria", "__Acidobacteria",
"__Holophagae", "__NS72", "__f", "__g"]}},{"id": "denovo5450",
"metadata": {"taxonomy": ["Bacteria", "__Bacteroidetes",
"__Sphingobacteriia", "__Sphingobacteriales", "__Saprospiraceae",
"__g"]}},{"id": "denovo5454", "metadata": {"taxonomy": ["Bacteria",
"__Proteobacteria", "__Gammaproteobacteria", "__Order_Incertae_Sedis",
"__Family_Incertae_Sedis", "__Arenicella"]}},{"id": "denovo5455",
"metadata": {"taxonomy": ["Bacteria", "__Proteobacteria",
"__Gammaproteobacteria", "__Oceanospirillales",
"__Oceanospirillaceae", "__Pseudospirillum"]}},{"id": "denovo5456",
"metadata": {"taxonomy": ["Bacteria", "__Bacteroidetes",
"__Flavobacteria", "__Flavobacteriales", "__Cryomorphaceae",
"__Owenweeksia"]}},{"id": "denovo5465", "metadata": {"taxonomy":
["Bacteria", "__Proteobacteria", "__Alphaproteobacteria",
"__Rhodobacterales", "__Rhodobacteraceae"]}},{"id": "denovo5474",
"metadata": {"taxonomy": ["Bacteria", "__Actinobacteria",
"__Actinobacteria", "__Micrococcales", "__Promicromonosporaceae",

"__Isoptericola"]}},{"id": "denovo5475", "metadata": {"taxonomy": ["Bacteria", "__Bacteroidetes", "__Sphingobacteriia", "__Sphingobacteriales", "__Chitinophagaceae", "__g"]}},{"id": "denovo5479", "metadata": {"taxonomy": ["Bacteria", "__Proteobacteria", "__Alphaproteobacteria", "__Rhizobiales", "__Hyphomicrobiaceae", "__Devosia"]}},{"id": "denovo5480", "metadata": {"taxonomy": ["Bacteria", "__Proteobacteria", "__Alphaproteobacteria", "__Rhodospirillales", "__Rhodospirillaceae", "__g"]}},{"id": "denovo5481", "metadata": {"taxonomy": ["Bacteria", "__Bacteroidetes", "__Sphingobacteriia", "__Sphingobacteriales", "__Saprospiraceae", "__g"]}},{"id": "denovo5485", "metadata": {"taxonomy": ["Bacteria", "__Bacteroidetes", "__Flavobacteria", "__Flavobacteriales", "__Cryomorphaceae", "__Crocinitomix"]}},{"id": "denovo5486", "metadata": {"taxonomy": ["Bacteria", "__Acidobacteria", "__Acidobacteria", "__PAUC26f", "__f", "__g"]}},{"id": "denovo5488", "metadata": {"taxonomy": ["Bacteria", "__Gemmatimonadetes", "__Gemmatimonadetes", "__PAUC43f_marine_benthic_group", "__f", "__g"]}},{"id": "denovo5489", "metadata": {"taxonomy": ["Bacteria", "__Bacteroidetes", "__Flavobacteria", "__Flavobacteriales", "__Cryomorphaceae", "__Owenweeksia"]}},{"id": "denovo5493", "metadata": {"taxonomy": ["Bacteria", "__Bacteroidetes", "__Sphingobacteriia", "__Sphingobacteriales", "__Saprospiraceae", "__g"]}},{"id": "denovo5497", "metadata": {"taxonomy": ["Bacteria", "__Proteobacteria", "__Gammaproteobacteria", "__Alteromonadales", "__Alteromonadaceae"]}},{"id": "denovo5501", "metadata": {"taxonomy": ["Bacteria", "__Proteobacteria", "__Alphaproteobacteria", "__Rickettsiales", "__SAR116_clade", "__g"]}},{"id": "denovo5504", "metadata": {"taxonomy": ["Bacteria", "__Proteobacteria", "__Alphaproteobacteria", "__Rhodospirillales"]}},{"id": "denovo5509", "metadata": {"taxonomy": ["Bacteria", "__Actinobacteria", "__Acidimicrobiia", "__Acidimicrobiales", "__OCS155_marine_group", "__g"]}},{"id": "denovo5513", "metadata": {"taxonomy": ["Bacteria", "__Proteobacteria", "__Alphaproteobacteria", "__Rhodospirillales", "__Rhodospirillaceae", "__g"]}},{"id": "denovo5516", "metadata": {"taxonomy": ["Bacteria", "__Bacteroidetes", "__Cytophagia", "__Cytophagales", "__Flammeovirgaceae", "__Candidatus_Amoebophilus"]}},{"id": "denovo5521", "metadata": {"taxonomy": ["Bacteria", "__Proteobacteria", "__SPOTSOCT00m83", "__o", "__f", "__g"]}},{"id": "denovo5523", "metadata": {"taxonomy": ["Bacteria", "__Proteobacteria", "__Alphaproteobacteria", "__Rhodobacterales", "__Rhodobacteraceae", "__Maritimibacter"]}},{"id": "denovo5525", "metadata": {"taxonomy": ["Bacteria", "__Bacteroidetes", "__Flavobacteria", "__Flavobacteriales", "__Flavobacteriaceae", "__NS4_marine_group"]}},{"id": "denovo5527", "metadata": {"taxonomy": ["Bacteria", "__Proteobacteria", "__Gammaproteobacteria", "__Alteromonadales", "__Colwelliaceae", "__Thalassomonas"]}},{"id": "denovo5528", "metadata": {"taxonomy": ["Bacteria", "__Proteobacteria", "__Alphaproteobacteria", "__Rickettsiales", "__SAR116_clade", "__g"]}},{"id": "denovo5529", "metadata": {"taxonomy": ["Bacteria", "__Actinobacteria",

"__Acidimicrobiia", "__Acidimicrobiales", "__Sva0996_marine_group", "__g"]}},{"id": "denovo5530", "metadata": {"taxonomy": ["Bacteria", "__Proteobacteria", "__Alphaproteobacteria", "__Rhodospirillales", "__Rhodospirillaceae", "__Pelagibius"]}},{"id": "denovo5531", "metadata": {"taxonomy": ["Bacteria", "__Proteobacteria", "__Deltaproteobacteria", "__Desulfobacterales", "__Desulfobulbaceae", "__Desulfopila"]}},{"id": "denovo5532", "metadata": {"taxonomy": ["Bacteria", "__Proteobacteria", "__Gammaproteobacteria", "__Order_Incertae_Sedis", "__Family_Incertae_Sedis", "__Marinicella"]}},{"id": "denovo5533", "metadata": {"taxonomy": ["Bacteria", "__Proteobacteria", "__Gammaproteobacteria", "__Oceanospirillales", "__Alcanivoracaceae", "__Kangiella"]}},{"id": "denovo5539", "metadata": {"taxonomy": ["Bacteria", "__Proteobacteria", "__Gammaproteobacteria", "__Alteromonadales", "__Alteromonadaceae", "__Haliea"]}},{"id": "denovo5543", "metadata": {"taxonomy": ["Bacteria", "__Actinobacteria", "__Acidimicrobiia", "__Acidimicrobiales", "__OCS155_marine_group", "__g"]}},{"id": "denovo5544", "metadata": {"taxonomy": ["Bacteria", "__Proteobacteria", "__Gammaproteobacteria", "__Alteromonadales", "__Alteromonadaceae", "__OM60(NOR5)_clade"]}},{"id": "denovo5545", "metadata": {"taxonomy": ["Archaea", "__Thaumarchaeota", "__Marine_Group_I", "__Candidatus_Nitrosopumilus", "__f", "__g"]}},{"id": "denovo5548", "metadata": {"taxonomy": ["Bacteria", "__Proteobacteria", "__Deltaproteobacteria", "__Bdellovibrionales", "__Bdellovibrionaceae", "__Bdellovibrio"]}},{"id": "denovo5551", "metadata": {"taxonomy": ["Bacteria", "__Proteobacteria", "__Alphaproteobacteria", "__Rickettsiales", "__SM2D12", "__g"]}},{"id": "denovo5554", "metadata": {"taxonomy": ["Bacteria", "__Proteobacteria", "__Alphaproteobacteria"]}},{"id": "denovo5556", "metadata": {"taxonomy": ["Bacteria", "__Acidobacteria", "__Holophagae", "__Acanthopleuribacterales", "__Acanthopleuribacteraceae", "__Acanthopleuribacter"]}},{"id": "denovo5559", "metadata": {"taxonomy": ["Bacteria", "__Proteobacteria", "__Alphaproteobacteria"]}},{"id": "denovo5563", "metadata": {"taxonomy": ["Bacteria", "__Bacteroidetes"]}},{"id": "denovo5564", "metadata": {"taxonomy": ["Bacteria", "__Proteobacteria", "__Alphaproteobacteria", "__Rhodospirillales", "__Rhodospirillaceae", "__Defluviicoccus"]}},{"id": "denovo5566", "metadata": {"taxonomy": ["Bacteria", "__Planctomycetes", "__OM190", "__o", "__f", "__g"]}},{"id": "denovo5569", "metadata": {"taxonomy": ["Bacteria", "__Cyanobacteria", "__Cyanobacteria", "__SubsectionIII", "__FamilyI", "__Phormidium"]}},{"id": "denovo5574", "metadata": {"taxonomy": ["Bacteria", "__Proteobacteria", "__Alphaproteobacteria", "__DB1-14", "__f", "__g"]}},{"id": "denovo5575", "metadata": {"taxonomy": ["Bacteria", "__Proteobacteria", "__Gammaproteobacteria", "__Chromatiales", "__Granulosicoccaceae", "__Granulosicoccus"]}},{"id": "denovo5576", "metadata": {"taxonomy": ["Bacteria", "__Proteobacteria", "__SPOTSOCT00m83", "__o", "__f", "__g"]}},{"id": "denovo5577", "metadata": {"taxonomy": ["Bacteria", "__Proteobacteria", "__Alphaproteobacteria", "__Rhizobiales",

"__Phyllobacteriaceae", "__g"]}},{"id": "denovo5579", "metadata": {"taxonomy": ["Bacteria", "__Proteobacteria", "__Gammaproteobacteria", "__Alteromonadales", "__Alteromonadaceae", "__BD1-7_clade"]}},{"id": "denovo5580", "metadata": {"taxonomy": ["Bacteria", "__Proteobacteria", "__Alphaproteobacteria", "__Rhodospirillales", "__Rhodospirillaceae", "__Tistlia"]}},{"id": "denovo5584", "metadata": {"taxonomy": ["Bacteria", "__Proteobacteria", "__Gammaproteobacteria", "__Alteromonadales", "__Moritellaceae", "__Paramoritella"]}},{"id": "denovo5585", "metadata": {"taxonomy": ["Bacteria", "__Proteobacteria", "__Gammaproteobacteria", "__Alteromonadales", "__Alteromonadaceae", "__OM60(NOR5)_clade"]}},{"id": "denovo5586", "metadata": {"taxonomy": ["Bacteria", "__Bacteroidetes", "__Cytophagia", "__Cytophagales", "__Flammeovirgaceae", "__Ekhidna"]}},{"id": "denovo5587", "metadata": {"taxonomy": ["Bacteria", "__Proteobacteria", "__Deltaproteobacteria", "__Desulfobacterales", "__Desulfobulbaceae"]}},{"id": "denovo5589", "metadata": {"taxonomy": ["Bacteria", "__Acidobacteria", "__RB25", "__o", "__f", "__g"]}},{"id": "denovo5596", "metadata": {"taxonomy": ["Bacteria", "__Proteobacteria", "__Deltaproteobacteria", "__Sh765B-TzT-29", "__f", "__g"]}},{"id": "denovo5601", "metadata": {"taxonomy": ["Bacteria", "__Proteobacteria", "__Alphaproteobacteria", "__Rhodospirillales", "__Rhodospirillaceae", "__g"]}},{"id": "denovo5603", "metadata": {"taxonomy": ["Bacteria", "__Chloroflexi"]}},{"id": "denovo5604", "metadata": {"taxonomy": ["Bacteria", "__Proteobacteria", "__Deltaproteobacteria", "__SAR324_clade(Marine_group_B)", "__f", "__g"]}},{"id": "denovo5606", "metadata": {"taxonomy": ["Bacteria", "__Actinobacteria", "__Actinobacteria", "__Corynebacteriales", "__Mycobacteriaceae", "__Mycobacterium"]}},{"id": "denovo5607", "metadata": {"taxonomy": ["Bacteria", "__Proteobacteria", "__Deltaproteobacteria", "__Myxococcales", "__Nannocystineae", "__g"]}},{"id": "denovo5608", "metadata": {"taxonomy": ["Bacteria", "__Proteobacteria", "__Epsilonproteobacteria", "__Campylobacterales", "__Helicobacteraceae", "__Sulfurimonas"]}},{"id": "denovo5611", "metadata": {"taxonomy": ["Bacteria", "__Acidobacteria", "__Holophagae", "__CA002", "__f", "__g"]}},{"id": "denovo5613", "metadata": {"taxonomy": ["Bacteria", "__Proteobacteria", "__Deltaproteobacteria", "__Desulfuromonadales"]}},{"id": "denovo5615", "metadata": {"taxonomy": ["Bacteria", "__Bacteroidetes", "__Flavobacteria", "__Flavobacteriales", "__Flavobacteriaceae", "__NS4_marine_group"]}},{"id": "denovo5620", "metadata": {"taxonomy": ["Bacteria", "__Proteobacteria", "__Gammaproteobacteria", "__Enterobacteriales", "__Enterobacteriaceae", "__Escherichia-Shigella"]}},{"id": "denovo5628", "metadata": {"taxonomy": ["Bacteria", "__Bacteroidetes", "__Flavobacteria", "__Flavobacteriales", "__Flavobacteriaceae", "__NS4_marine_group"]}},{"id": "denovo5633", "metadata": {"taxonomy": ["Bacteria", "__Proteobacteria", "__Deltaproteobacteria", "__Myxococcales", "__0319-6G20", "__g"]}},{"id": "denovo5634", "metadata": {"taxonomy": ["Bacteria", "__Proteobacteria", "__Betaproteobacteria",

"__Burkholderiales", "__Comamonadaceae"]}},{"id": "denovo5635", "metadata": {"taxonomy": ["Bacteria", "__Bacteroidetes", "__Flavobacteria", "__Flavobacteriales", "__Flavobacteriaceae", "__Tenacibaculum"]}},{"id": "denovo5636", "metadata": {"taxonomy": ["Bacteria", "__Proteobacteria", "__Alphaproteobacteria", "__Rhizobiales", "__Phyllobacteriaceae", "__g"]}},{"id": "denovo5637", "metadata": {"taxonomy": ["Bacteria", "__Proteobacteria", "__Gammaproteobacteria", "__Oceanospirillales", "__Hahellaceae", "__Endozoicomonas"]}},{"id": "denovo5638", "metadata": {"taxonomy": ["Bacteria", "__Acidobacteria", "__RB25", "__o", "__f", "__g"]}}, {"id": "denovo5640", "metadata": {"taxonomy": ["Bacteria", "__Proteobacteria", "__Deltaproteobacteria", "__Desulfobacterales", "__Desulfobacteraceae", "__Sva0081_sediment_group"]}},{"id": "denovo5643", "metadata": {"taxonomy": ["Bacteria", "__Candidate_division_OD1", "__c", "__o", "__f", "__g"]}},{"id": "denovo5645", "metadata": {"taxonomy": ["Bacteria"]}},{"id": "denovo5646", "metadata": {"taxonomy": ["Bacteria", "__Bacteroidetes", "__Sphingobacteriia", "__Sphingobacteriales", "__NS11-12_marine_group", "__g"]}},{"id": "denovo5647", "metadata": {"taxonomy": ["Bacteria", "__Proteobacteria", "__Gammaproteobacteria", "__Pseudomonadales", "__Moraxellaceae", "__Acinetobacter"]}},{"id": "denovo5650", "metadata": {"taxonomy": ["Bacteria", "__Proteobacteria", "__Alphaproteobacteria", "__Rhodobacterales", "__Rhodobacteraceae", "__Pseudovibrio"]}},{"id": "denovo5652", "metadata": {"taxonomy": ["Bacteria", "__Proteobacteria", "__Gammaproteobacteria", "__Pseudomonadales", "__Pseudomonadaceae", "__Pseudomonas"]}},{"id": "denovo5657", "metadata": {"taxonomy": ["Bacteria", "__Acidobacteria", "__Acidobacteria", "__DA023", "__f", "__g"]}},{"id": "denovo5659", "metadata": {"taxonomy": ["Bacteria", "__Bacteroidetes", "__Sphingobacteriia", "__Sphingobacteriales", "__Saprospiraceae", "__g"]}},{"id": "denovo5660", "metadata": {"taxonomy": ["Bacteria", "__Proteobacteria", "__Gammaproteobacteria", "__KI89A_clade", "__f", "__g"]}},{"id": "denovo5661", "metadata": {"taxonomy": ["Bacteria", "__Proteobacteria", "__Alphaproteobacteria", "__Rhodobacterales", "__Rhodobacteraceae", "__Ruegeria"]}},{"id": "denovo5663", "metadata": {"taxonomy": ["Bacteria", "__Proteobacteria", "__Alphaproteobacteria", "__Rhizobiales", "__F0723", "__g"]}},{"id": "denovo5664", "metadata": {"taxonomy": ["Bacteria", "__Bacteroidetes", "__Cytophagia", "__Cytophagales", "__Flammeovirgaceae", "__Fabibacter"]}},{"id": "denovo5665", "metadata": {"taxonomy": ["Bacteria", "__Proteobacteria", "__Deltaproteobacteria", "__Desulfobacterales", "__Desulfobacteraceae", "__Desulfospira"]}},{"id": "denovo5668", "metadata": {"taxonomy": ["Bacteria", "__Proteobacteria", "__Gammaproteobacteria", "__BD7-8_marine_group", "__f", "__g"]}}, {"id": "denovo5670", "metadata": {"taxonomy": ["Bacteria", "__Proteobacteria", "__Alphaproteobacteria", "__Rhodospirillales", "__MSB-1E8", "__g"]}},{"id": "denovo5671", "metadata": {"taxonomy": ["Bacteria", "__Bacteroidetes", "__Flavobacteria", "__Flavobacteriales", "__Cryomorphaceae", "__Owenweeksia"]}},{"id":

"denovo5673", "metadata": {"taxonomy": ["Bacteria", "__Proteobacteria", "__Alphaproteobacteria", "__SAR11_clade", "__Chesapeake-Delaware_Bay", "__g"]}},{"id": "denovo5678", "metadata": {"taxonomy": ["Bacteria", "__Proteobacteria", "__Alphaproteobacteria", "__Caulobacterales", "__Hyphomonadaceae", "__Hellea"]}},{"id": "denovo5679", "metadata": {"taxonomy": ["Bacteria", "__Proteobacteria", "__Alphaproteobacteria", "__Rhodobacterales", "__Rhodobacteraceae"]}},{"id": "denovo5685", "metadata": {"taxonomy": ["Bacteria", "__Proteobacteria", "__Alphaproteobacteria", "__Rhodospirillales", "__Rhodospirillaceae", "__AEGEAN-169_marine_group"]}},{"id": "denovo5689", "metadata": {"taxonomy": ["Bacteria", "__Proteobacteria", "__Alphaproteobacteria", "__Rhodospirillales", "__Rhodospirillaceae", "__AEGEAN-169_marine_group"]}},{"id": "denovo5692", "metadata": {"taxonomy": ["Bacteria", "__Proteobacteria", "__Alphaproteobacteria", "__Rhodobacterales", "__Rhodobacteraceae"]}},{"id": "denovo5695", "metadata": {"taxonomy": ["Bacteria", "__Spirochaetes", "__Spirochaetes", "__Spirochaetales", "__Leptospiraceae", "__g"]}},{"id": "denovo5698", "metadata": {"taxonomy": ["Bacteria", "__Candidate_division_WS3", "__c", "__o", "__f", "__g"]}},{"id": "denovo5699", "metadata": {"taxonomy": ["Bacteria", "__Proteobacteria", "__Gammaproteobacteria", "__Vibrionales", "__Vibrionaceae", "__Vibrio"]}},{"id": "denovo5700", "metadata": {"taxonomy": ["Bacteria", "__Planctomycetes", "__Planctomycetacia", "__Planctomycetales", "__Planctomycetaceae", "__Planctomyces"]}},{"id": "denovo5707", "metadata": {"taxonomy": ["Bacteria", "__Acidobacteria", "__Holophagae", "__CA002", "__f", "__g"]}},{"id": "denovo5715", "metadata": {"taxonomy": ["Bacteria", "__Proteobacteria", "__Gammaproteobacteria", "__Alteromonadales", "__Colwelliaceae", "__Colwellia"]}},{"id": "denovo5723", "metadata": {"taxonomy": ["Bacteria", "__Proteobacteria", "__Alphaproteobacteria", "__Rhodospirillales", "__Rhodospirillaceae", "__Thalassospira"]}},{"id": "denovo5727", "metadata": {"taxonomy": ["Bacteria", "__Cyanobacteria", "__Cyanobacteria", "__SubsectionI", "__FamilyI", "__g"]}},{"id": "denovo5736", "metadata": {"taxonomy": ["Bacteria", "__Proteobacteria", "__Gammaproteobacteria", "__Xanthomonadales", "__Sinobacteraceae", "__g"]}},{"id": "denovo5737", "metadata": {"taxonomy": ["Bacteria", "__Gemmatimonadetes", "__Gemmatimonadetes", "__Gemmatimonadales", "__Gemmatimonadaceae", "__g"]}},{"id": "denovo5738", "metadata": {"taxonomy": ["Bacteria", "__Planctomycetes", "__Planctomycetacia", "__Planctomycetales", "__Planctomycetaceae", "__Rhodopirellula"]}},{"id": "denovo5739", "metadata": {"taxonomy": ["Bacteria", "__Gemmatimonadetes", "__Gemmatimonadetes", "__BD2-11_terrestrial_group", "__f", "__g"]}},{"id": "denovo5741", "metadata": {"taxonomy": ["Bacteria", "__Proteobacteria", "__Alphaproteobacteria", "__Rhodospirillales", "__Rhodospirillaceae", "__Skermanella"]}},{"id": "denovo5742", "metadata": {"taxonomy": ["Bacteria", "__Firmicutes", "__Clostridia", "__Halanaerobiales", "__Halobacteroidaceae", "__g"]}},{"id": "denovo5744", "metadata": {"taxonomy": ["Bacteria", "__Chlamydiae",

"__Chlamydiae", "__Chlamydiales", "__Simkaniaceae",
"__Candidatus_Fritschea"]}},{"id": "denovo5745", "metadata":
{"taxonomy": ["Archaea", "__Thaumarchaeota", "__Marine_Group_I",
"__o", "__f", "__g"]}},{"id": "denovo5751", "metadata": {"taxonomy":
["Bacteria", "__Bacteroidetes", "__Sphingobacteriia",
"__Sphingobacteriales", "__NS11-12_marine_group", "__g"]}},{"id":
"denovo5752", "metadata": {"taxonomy": ["Bacteria",
"__Proteobacteria", "__Deltaproteobacteria", "__Sh765B-TzT-29", "__f",
"__g"]}},{"id": "denovo5757", "metadata": {"taxonomy": ["Bacteria",
"__Proteobacteria", "__Gammaproteobacteria", "__Thiotrichales",
"__Piscirickettsiaceae", "__g"]}},{"id": "denovo5759", "metadata":
{"taxonomy": ["Bacteria", "__Proteobacteria", "__Deltaproteobacteria",
"__Desulfobacterales", "__Desulfobacteraceae", "__Desulfosarcina"]}},
{"id": "denovo5765", "metadata": {"taxonomy": ["Bacteria",
"__Proteobacteria", "__Alphaproteobacteria", "__OCS116_clade", "__f",
"__g"]}},{"id": "denovo5766", "metadata": {"taxonomy": ["Bacteria",
"__Proteobacteria"]}},{"id": "denovo5771", "metadata": {"taxonomy":
["Bacteria", "__Proteobacteria", "__Deltaproteobacteria",
"__Bdellovibrionales", "__Bacteriovoracaceae", "__Peredibacter"]}},
{"id": "denovo5772", "metadata": {"taxonomy": ["Bacteria",
"__Proteobacteria", "__Gammaproteobacteria"]}},{"id": "denovo5773",
"metadata": {"taxonomy": ["Bacteria", "__Bacteroidetes",
"__Flavobacteria", "__Flavobacteriales", "__Cryomorphaceae",
"__Owenweeksia"]}},{"id": "denovo5774", "metadata": {"taxonomy":
["Bacteria", "__Proteobacteria", "__Gammaproteobacteria",
"__Alteromonadales", "__Colwelliaceae", "__Thalassomonas"]}},{"id":
"denovo5776", "metadata": {"taxonomy": ["Bacteria",
"__Proteobacteria", "__Alphaproteobacteria", "__Parvularculales",
"__Parvularculaceae", "__Parvularcula"]}},{"id": "denovo5777",
"metadata": {"taxonomy": ["Bacteria", "__Proteobacteria",
"__Gammaproteobacteria", "__Alteromonadales", "__Alteromonadaceae"]}},
{"id": "denovo5778", "metadata": {"taxonomy": ["Bacteria",
"__Proteobacteria", "__Gammaproteobacteria", "__Order_Incertae_Sedis",
"__Family_Incertae_Sedis", "__Thiohalophilus"]}},{"id": "denovo5779",
"metadata": {"taxonomy": ["Bacteria", "__Proteobacteria",
"__Alphaproteobacteria", "__Rickettsiales"]}},{"id": "denovo5789",
"metadata": {"taxonomy": ["Bacteria", "__Proteobacteria",
"__Alphaproteobacteria"]}},{"id": "denovo5801", "metadata":
{"taxonomy": ["Bacteria", "__Proteobacteria", "__Gammaproteobacteria",
"__Thiotrichales", "__Piscirickettsiaceae", "__g"]}},{"id":
"denovo5805", "metadata": {"taxonomy": ["Bacteria",
"__Proteobacteria", "__Alphaproteobacteria", "__Rhodobacterales",
"__Rhodobacteraceae", "__Roseobacter_clade_AS-21_lineage"]}},{"id":
"denovo5809", "metadata": {"taxonomy": ["Bacteria",
"__Proteobacteria", "__Gammaproteobacteria",
"__E01-9C-26_marine_group", "__f", "__g"]}},{"id": "denovo5810",
"metadata": {"taxonomy": ["Bacteria", "__Proteobacteria",
"__Alphaproteobacteria", "__Rhodospirillales", "__Rhodospirillaceae",
"__g"]}},{"id": "denovo5813", "metadata": {"taxonomy": ["Bacteria",
"__Gemmatimonadetes", "__Gemmatimonadetes",

"__BD2-11_terrestrial_group", "__f", "__g"]}},{"id": "denovo5818", "metadata": {"taxonomy": ["Bacteria", "__Verrucomicrobia", "__Opitutae"]}},{"id": "denovo5819", "metadata": {"taxonomy": ["Bacteria", "__Proteobacteria", "__Alphaproteobacteria", "__Rhodobacterales", "__Rhodobacteraceae"]}},{"id": "denovo5821", "metadata": {"taxonomy": ["Bacteria", "__Bacteroidetes", "__Flavobacteria", "__Flavobacteriales", "__Cryomorphaceae", "__Crocinitomix"]}},{"id": "denovo5823", "metadata": {"taxonomy": ["Bacteria", "__Proteobacteria", "__Gammaproteobacteria", "__KI89A_clade", "__f", "__g"]}},{"id": "denovo5825", "metadata": {"taxonomy": ["Bacteria", "__Proteobacteria", "__Alphaproteobacteria", "__Rhodobacterales", "__Rhodobacteraceae", "__Roseobacter_clade_OCT_lineage"]}},{"id": "denovo5827", "metadata": {"taxonomy": ["Archaea", "__Thaumarchaeota", "__Marine_Group_I", "__Cenarchaeales", "__Cenarchaeaceae", "__Cenarchaeum"]}},{"id": "denovo5828", "metadata": {"taxonomy": ["Bacteria", "__Cyanobacteria", "__MLE1-12", "__o", "__f", "__g"]}},{"id": "denovo5829", "metadata": {"taxonomy": ["Bacteria", "__Proteobacteria", "__Gammaproteobacteria", "__Vibrionales", "__Vibrionaceae", "__Vibrio"]}},{"id": "denovo5831", "metadata": {"taxonomy": ["Bacteria", "__Bacteroidetes"]}},{"id": "denovo5839", "metadata": {"taxonomy": ["Bacteria", "__Proteobacteria", "__Gammaproteobacteria", "__Vibrionales", "__Vibrionaceae", "__Vibrio"]}},{"id": "denovo5841", "metadata": {"taxonomy": ["Bacteria", "__Proteobacteria", "__Alphaproteobacteria", "__Rhodobacterales", "__Rhodobacteraceae", "__Wenxinia"]}},{"id": "denovo5842", "metadata": {"taxonomy": ["Bacteria", "__Proteobacteria", "__Alphaproteobacteria", "__Rhodospirillales", "__Rhodospirillaceae", "__g"]}},{"id": "denovo5843", "metadata": {"taxonomy": ["Bacteria", "__Proteobacteria", "__Gammaproteobacteria", "__Xanthomonadales", "__Sinobacteraceae", "__JTB255_marine_benthic_group"]}},{"id": "denovo5846", "metadata": {"taxonomy": ["Bacteria", "__Chlorobi", "__Chlorobia", "__Chlorobiales", "__Chlorobiaceae", "__Prosthecochloris"]}},{"id": "denovo5847", "metadata": {"taxonomy": ["Bacteria", "__Cyanobacteria", "__Cyanobacteria", "__SubsectionIII", "__FamilyI", "__Arthrospira"]}},{"id": "denovo5848", "metadata": {"taxonomy": ["Bacteria", "__Actinobacteria", "__Acidimicrobiia", "__Acidimicrobiales", "__OCS155_marine_group", "__g"]}},{"id": "denovo5851", "metadata": {"taxonomy": ["Bacteria", "__Bacteroidetes", "__Flavobacteria", "__Flavobacteriales", "__Flavobacteriaceae", "__Algibacter"]}},{"id": "denovo5854", "metadata": {"taxonomy": ["Bacteria", "__Verrucomicrobia", "__Opitutae", "__Puniceicoccales", "__Puniceicoccaceae", "__Lentimonas"]}},{"id": "denovo5857", "metadata": {"taxonomy": ["Bacteria", "__Nitrospirae", "__Nitrospira", "__Nitrospirales", "__Nitrospiraceae", "__Nitrospira"]}},{"id": "denovo5858", "metadata": {"taxonomy": ["Bacteria", "__Actinobacteria", "__Acidimicrobiia", "__Acidimicrobiales", "__Sva0996_marine_group", "__g"]}},{"id": "denovo5859", "metadata": {"taxonomy": ["Bacteria", "__Proteobacteria", "__Alphaproteobacteria", "__Rhodobacterales", "__Rhodobacteraceae", "__g"]}},{"id":

"denovo5860", "metadata": {"taxonomy": ["Bacteria", "__Acidobacteria", "__RB25", "__o", "__f", "__g"]}},{"id": "denovo5869", "metadata": {"taxonomy": ["Bacteria", "__Proteobacteria", "__Alphaproteobacteria", "__SAR11_clade", "__Surface_1", "__g"]}},{"id": "denovo5870", "metadata": {"taxonomy": ["Bacteria", "__Proteobacteria", "__Alphaproteobacteria", "__Rhodobacterales", "__Rhodobacteraceae", "__Loktanella"]}},{"id": "denovo5871", "metadata": {"taxonomy": ["Bacteria", "__Spirochaetes", "__Spirochaetes", "__Spirochaetales", "__Spirochaetaceae", "__Spirochaeta"]}},{"id": "denovo5872", "metadata": {"taxonomy": ["Bacteria", "__Proteobacteria", "__Deltaproteobacteria", "__Desulfuromonadales", "__Desulfuromonadaceae"]}},{"id": "denovo5873", "metadata": {"taxonomy": ["Bacteria", "__Deferribacteres", "__Deferribacteres", "__Deferribacterales", "__Family_Incertae_Sedis", "__Caldithrix"]}},{"id": "denovo5878", "metadata": {"taxonomy": ["Bacteria", "__Proteobacteria", "__Deltaproteobacteria", "__Myxococcales", "__Nannocystaceae", "__g"]}},{"id": "denovo5880", "metadata": {"taxonomy": ["Bacteria", "__Proteobacteria", "__Gammaproteobacteria", "__E01-9C-26_marine_group", "__f", "__g"]}},{"id": "denovo5881", "metadata": {"taxonomy": ["Bacteria", "__Bacteroidetes", "__Cytophagia", "__Order_II_Incertae_Sedis", "__Rhodothermaceae", "__Rubricoccus"]}},{"id": "denovo5885", "metadata": {"taxonomy": ["Bacteria", "__Spirochaetes", "__Spirochaetes", "__Spirochaetales", "__Spirochaetaceae", "__Spirochaeta"]}},{"id": "denovo5887", "metadata": {"taxonomy": ["Bacteria", "__Proteobacteria", "__Alphaproteobacteria", "__Rhodospirillales", "__Rhodospirillaceae", "__g"]}},{"id": "denovo5888", "metadata": {"taxonomy": ["Bacteria", "__Bacteroidetes", "__Flavobacteria", "__Flavobacteriales", "__Flavobacteriaceae", "__Muricauda"]}},{"id": "denovo5892", "metadata": {"taxonomy": ["Bacteria", "__Proteobacteria", "__Alphaproteobacteria", "__Rhodospirillales", "__Rhodospirillaceae", "__g"]}},{"id": "denovo5897", "metadata": {"taxonomy": ["Bacteria", "__Bacteroidetes", "__Cytophagia", "__Order_II_Incertae_Sedis", "__Rhodothermaceae", "__g"]}},{"id": "denovo5898", "metadata": {"taxonomy": ["Bacteria", "__Firmicutes", "__Clostridia", "__Clostridiales"]}},{"id": "denovo5903", "metadata": {"taxonomy": ["Bacteria", "__Firmicutes", "__Clostridia", "__Clostridiales", "__Clostridiaceae", "__Alkaliphilus"]}},{"id": "denovo5907", "metadata": {"taxonomy": ["Bacteria", "__Proteobacteria", "__Betaproteobacteria"]}},{"id": "denovo5909", "metadata": {"taxonomy": ["Bacteria", "__Proteobacteria", "__Gammaproteobacteria", "__Pseudomonadales", "__Moraxellaceae", "__Psychrobacter"]}},{"id": "denovo5913", "metadata": {"taxonomy": ["Bacteria", "__Proteobacteria", "__Deltaproteobacteria", "__Desulfobacterales", "__Desulfobulbaceae"]}},{"id": "denovo5916", "metadata": {"taxonomy": ["Bacteria", "__Proteobacteria", "__Deltaproteobacteria", "__Bdellovibrionales", "__Bacteriovoracaceae", "__Peredibacter"]}},{"id": "denovo5917", "metadata": {"taxonomy": ["Bacteria", "__Bacteroidetes", "__Flavobacteria", "__Flavobacteriales", "__NS9_marine_group", "__g"]}},{"id": "denovo5921", "metadata":

{"taxonomy": ["Bacteria", "__Proteobacteria", "__Alphaproteobacteria", "__Parvularculales", "__Parvularculaceae", "__Parvularcula"]}},{"id": "denovo5923", "metadata": {"taxonomy": ["Bacteria", "__Nitrospirae", "__Nitrospira", "__Nitrospirales", "__Nitrospiraceae", "__Nitrospira"]}},{"id": "denovo5924", "metadata": {"taxonomy": ["Bacteria", "__Proteobacteria", "__Alphaproteobacteria", "__Rickettsiales", "__SAR116_clade", "__Candidatus_Puniceispirillum"]}},{"id": "denovo5927", "metadata": {"taxonomy": ["Bacteria", "__Proteobacteria", "__Gammaproteobacteria", "__Oceanospirillales", "__Alcanivoracaceae", "__Kangiella"]}},{"id": "denovo5929", "metadata": {"taxonomy": ["Bacteria", "__Bacteroidetes", "__Flavobacteria", "__Flavobacteriales", "__Cryomorphaceae", "__Owenweeksia"]}},{"id": "denovo5930", "metadata": {"taxonomy": ["Bacteria", "__Acidobacteria", "__Acidobacteria", "__PAUC26f", "__f", "__g"]}},{"id": "denovo5932", "metadata": {"taxonomy": ["Bacteria", "__Proteobacteria", "__Alphaproteobacteria", "__Caulobacterales", "__Caulobacteraceae", "__Amorphus"]}},{"id": "denovo5935", "metadata": {"taxonomy": ["Bacteria", "__Cyanobacteria", "__Cyanobacteria", "__SubsectionII", "__FamilyII", "__Pleurocapsa"]}},{"id": "denovo5936", "metadata": {"taxonomy": ["Bacteria", "__Proteobacteria", "__Gammaproteobacteria", "__NKB5", "__f", "__g"]}},{"id": "denovo5940", "metadata": {"taxonomy": ["Bacteria", "__Proteobacteria", "__Gammaproteobacteria", "__Alteromonadales", "__Colwelliaceae", "__Colwellia"]}},{"id": "denovo5941", "metadata": {"taxonomy": ["Bacteria", "__Proteobacteria", "__Gammaproteobacteria", "__Xanthomonadales", "__Sinobacteraceae", "__JTB255_marine_benthic_group"]}},{"id": "denovo5942", "metadata": {"taxonomy": ["Bacteria", "__Firmicutes", "__Clostridia", "__Clostridiales", "__Veillonellaceae", "__Pelosinus"]}},{"id": "denovo5944", "metadata": {"taxonomy": ["Bacteria", "__Proteobacteria", "__Gammaproteobacteria", "__Pseudomonadales", "__Moraxellaceae", "__Acinetobacter"]}},{"id": "denovo5946", "metadata": {"taxonomy": ["Bacteria", "__Bacteroidetes", "__Sphingobacteriia", "__Sphingobacteriales", "__Saprospiraceae", "__g"]}},{"id": "denovo5947", "metadata": {"taxonomy": ["Bacteria", "__Bacteroidetes", "__Sphingobacteriia", "__Sphingobacteriales", "__Saprospiraceae", "__g"]}},{"id": "denovo5949", "metadata": {"taxonomy": ["Bacteria", "__Proteobacteria", "__Alphaproteobacteria", "__Rhodobacterales", "__Rhodobacteraceae", "__Pseudovibrio"]}},{"id": "denovo5951", "metadata": {"taxonomy": ["Bacteria", "__Proteobacteria", "__Deltaproteobacteria", "__Myxococcales", "__Cystobacterineae", "__g"]}},{"id": "denovo5954", "metadata": {"taxonomy": ["Bacteria", "__Bacteroidetes", "__Class_Incertae_Sedis", "__Order_Incertae_Sedis", "__Family_Incertae_Sedis", "__Prolixibacter"]}},{"id": "denovo5955", "metadata": {"taxonomy": ["Bacteria", "__Proteobacteria", "__Gammaproteobacteria", "__BD7-8_marine_group", "__f", "__g"]}},{"id": "denovo5959", "metadata": {"taxonomy": ["Bacteria", "__Proteobacteria", "__Gammaproteobacteria"]}},{"id": "denovo5962", "metadata": {"taxonomy": ["Bacteria", "__Proteobacteria", "__Alphaproteobacteria",

"__Rickettsiales", "__Rickettsiaceae", "__g"]}},{"id": "denovo5966", "metadata": {"taxonomy": ["Bacteria", "__Proteobacteria", "__Alphaproteobacteria", "__Rhodobacterales", "__Rhodobacteraceae", "__Pseudoruegeria"]}},{"id": "denovo5967", "metadata": {"taxonomy": ["Bacteria", "__Proteobacteria", "__Deltaproteobacteria", "__Desulfobacterales", "__Desulfobacteraceae", "__Desulfosarcina"]}}, {"id": "denovo5968", "metadata": {"taxonomy": ["Bacteria", "__Proteobacteria", "__Gammaproteobacteria", "__Oceanospirillales", "__OM182_clade", "__g"]}},{"id": "denovo5974", "metadata": {"taxonomy": ["Bacteria", "__Proteobacteria", "__Alphaproteobacteria", "__Rhodobacterales", "__Rhodobacteraceae"]}},{"id": "denovo5975", "metadata": {"taxonomy": ["Bacteria", "__Proteobacteria", "__Deltaproteobacteria", "__Myxococcales", "__Nannocystineae", "__g"]}},{"id": "denovo5976", "metadata": {"taxonomy": ["Bacteria", "__Proteobacteria", "__Gammaproteobacteria", "__Sva0071", "__f", "__g"]}},{"id": "denovo5977", "metadata": {"taxonomy": ["Bacteria", "__Proteobacteria", "__Deltaproteobacteria", "__Desulfobacterales", "__Desulfobacteraceae"]}},{"id": "denovo5978", "metadata": {"taxonomy": ["Bacteria", "__Proteobacteria", "__Alphaproteobacteria", "__DB1-14", "__f", "__g"]}},{"id": "denovo5980", "metadata": {"taxonomy": ["Bacteria", "__Proteobacteria", "__Deltaproteobacteria", "__Desulfobacterales", "__Desulfobulbaceae", "__Desulfofustis"]}}, {"id": "denovo5982", "metadata": {"taxonomy": ["Bacteria", "__Proteobacteria", "__Gammaproteobacteria"]}},{"id": "denovo5986", "metadata": {"taxonomy": ["Archaea", "__Thaumarchaeota", "__Marine_Group_I", "__Cenarchaeales", "__Cenarchaeaceae", "__Cenarchaeum"]}},{"id": "denovo5988", "metadata": {"taxonomy": ["Bacteria", "__Proteobacteria", "__Alphaproteobacteria", "__Rhodospirillales", "__Rhodospirillaceae", "__Thalassospira"]}}, {"id": "denovo5989", "metadata": {"taxonomy": ["Bacteria", "__Proteobacteria", "__Deltaproteobacteria", "__Bdellovibrionales", "__Bacteriovoracaceae", "__Bacteriovorax"]}},{"id": "denovo5994", "metadata": {"taxonomy": ["Bacteria", "__Proteobacteria", "__Alphaproteobacteria", "__SAR11_clade", "__Surface_1", "__Candidatus_Pelagibacter"]}},{"id": "denovo5995", "metadata": {"taxonomy": ["Bacteria", "__Cyanobacteria", "__Cyanobacteria", "__SubsectionI", "__FamilyI"]}},{"id": "denovo5998", "metadata": {"taxonomy": ["Bacteria", "__Proteobacteria", "__Alphaproteobacteria", "__Rhodospirillales", "__Rhodospirillaceae", "__Pelagibius"]}},{"id": "denovo6001", "metadata": {"taxonomy": ["Bacteria", "__Proteobacteria", "__Gammaproteobacteria", "__Oceanospirillales", "__Oceanospirillaceae", "__Pseudospirillum"]}},{"id": "denovo6004", "metadata": {"taxonomy": ["Bacteria", "__Bacteroidetes", "__Sphingobacteriia", "__Sphingobacteriales", "__Saprospiraceae", "__Lewinella"]}},{"id": "denovo6005", "metadata": {"taxonomy": ["Bacteria", "__Actinobacteria", "__Acidimicrobiia", "__Acidimicrobiales", "__Acidimicrobiaceae", "__Ilumatobacter"]}}, {"id": "denovo6006", "metadata": {"taxonomy": ["Bacteria", "__Proteobacteria", "__Betaproteobacteria", "__Burkholderiales", "__Comamonadaceae", "__Aquabacterium"]}},{"id": "denovo6009",

"metadata": {"taxonomy": ["Bacteria", "__Cyanobacteria", "__Cyanobacteria", "__SubsectionIII", "__FamilyI"]}},{"id": "denovo6011", "metadata": {"taxonomy": ["Bacteria", "__Proteobacteria", "__Gammaproteobacteria", "__Order_Incertae_Sedis", "__Family_Incertae_Sedis", "__Sedimenticola"]}},{"id": "denovo6012", "metadata": {"taxonomy": ["Bacteria", "__Proteobacteria", "__Gammaproteobacteria", "__Xanthomonadales", "__Sinobacteraceae", "__JTB255_marine_benthic_group"]}},{"id": "denovo6021", "metadata": {"taxonomy": ["Bacteria", "__Proteobacteria", "__Alphaproteobacteria", "__Rhizobiales", "__Bradyrhizobiaceae", "__Bosea"]}},{"id": "denovo6023", "metadata": {"taxonomy": ["Bacteria", "__Proteobacteria", "__Alphaproteobacteria"]}},{"id": "denovo6026", "metadata": {"taxonomy": ["Bacteria", "__Proteobacteria", "__Gammaproteobacteria"]}},{"id": "denovo6030", "metadata": {"taxonomy": ["Bacteria", "__Proteobacteria", "__Alphaproteobacteria", "__DB1-14", "__f", "__g"]}},{"id": "denovo6038", "metadata": {"taxonomy": ["Bacteria", "__Bacteroidetes", "__Flavobacteria", "__Flavobacteriales", "__Cryomorphaceae", "__Owenweeksia"]}},{"id": "denovo6039", "metadata": {"taxonomy": ["Bacteria", "__Proteobacteria", "__Deltaproteobacteria", "__Myxococcales", "__Cystobacterineae", "__g"]}},{"id": "denovo6050", "metadata": {"taxonomy": ["Bacteria", "__Proteobacteria", "__Alphaproteobacteria", "__Rhodospirillales", "__Rhodospirillaceae", "__AEGEAN-169_marine_group"]}},{"id": "denovo6052", "metadata": {"taxonomy": ["Bacteria", "__Proteobacteria", "__Gammaproteobacteria", "__Chromatiales", "__Ectothiorhodospiraceae", "__Thioalkalispira"]}}, {"id": "denovo6058", "metadata": {"taxonomy": ["Bacteria", "__Bacteroidetes", "__Flavobacteria", "__Flavobacteriales", "__Flavobacteriaceae", "__Lutibacter"]}},{"id": "denovo6062", "metadata": {"taxonomy": ["Bacteria", "__Cyanobacteria"]}},{"id": "denovo6065", "metadata": {"taxonomy": ["Bacteria", "__Proteobacteria", "__Gammaproteobacteria"]}},{"id": "denovo6069", "metadata": {"taxonomy": ["Bacteria", "__Proteobacteria", "__Alphaproteobacteria", "__Rhodospirillales", "__Rhodospirillaceae", "__g"]}},{"id": "denovo6072", "metadata": {"taxonomy": ["Bacteria", "__Bacteroidetes", "__Flavobacteria", "__Flavobacteriales", "__Flavobacteriaceae", "__Polaribacter"]}},{"id": "denovo6073", "metadata": {"taxonomy": ["Bacteria", "__Proteobacteria", "__Alphaproteobacteria", "__Rhodospirillales", "__Rhodospirillaceae", "__g"]}},{"id": "denovo6075", "metadata": {"taxonomy": ["Bacteria", "__Acidobacteria", "__Holophagae", "__CA002", "__f", "__g"]}},{"id": "denovo6081", "metadata": {"taxonomy": ["Bacteria", "__Bacteroidetes", "__Sphingobacteriia", "__Sphingobacteriales", "__NS11-12_marine_group", "__g"]}},{"id": "denovo6082", "metadata": {"taxonomy": ["Bacteria", "__Proteobacteria", "__Alphaproteobacteria", "__DB1-14", "__f", "__g"]}},{"id": "denovo6083", "metadata": {"taxonomy": ["Bacteria", "__Proteobacteria", "__Gammaproteobacteria", "__Oceanospirillales", "__Alcanivoracaceae", "__Kangiella"]}},{"id": "denovo6084", "metadata": {"taxonomy": ["Bacteria", "__Proteobacteria", "__Gammaproteobacteria", "__Alteromonadales",

"__Alteromonadaceae"]}},{"id": "denovo6086", "metadata": {"taxonomy":
["Bacteria", "__Proteobacteria", "__Gammaproteobacteria",
"__Order_Incertae_Sedis", "__Family_Incertae_Sedis",
"__Arenicella"]}},{"id": "denovo6093", "metadata": {"taxonomy":
["Bacteria", "__Proteobacteria", "__JTB23", "__o", "__f", "__g"]}},
{"id": "denovo6095", "metadata": {"taxonomy": ["Bacteria",
"__Cyanobacteria", "__Cyanobacteria", "__SubsectionIII", "__FamilyI",
"__g"]}},{"id": "denovo6096", "metadata": {"taxonomy": ["Bacteria",
"__Proteobacteria", "__Alphaproteobacteria", "__Rhodospirillales",
"__Rhodospirillaceae", "__g"]}},{"id": "denovo6097", "metadata":
{"taxonomy": ["Bacteria", "__Proteobacteria", "__Alphaproteobacteria",
"__SAR11_clade", "__Surface_1", "__Candidatus_Pelagibacter"]}},{"id":
"denovo6099", "metadata": {"taxonomy": ["Bacteria", "__Cyanobacteria",
"__Cyanobacteria", "__SubsectionIII", "__FamilyI", "__Phormidium"]}},
{"id": "denovo6113", "metadata": {"taxonomy": ["Bacteria",
"__Bacteroidetes", "__Flavobacteria", "__Flavobacteriales",
"__Flavobacteriaceae", "__Sufflavibacter"]}},{"id": "denovo6120",
"metadata": {"taxonomy": ["Bacteria", "__Actinobacteria",
"__Actinobacteria", "__Micrococcales", "__Microbacteriaceae"]}},{"id":
"denovo6121", "metadata": {"taxonomy": ["Bacteria",
"__Proteobacteria", "__Gammaproteobacteria", "__KI89A_clade", "__f",
"__g"]}},{"id": "denovo6123", "metadata": {"taxonomy": ["Bacteria",
"__Proteobacteria", "__Gammaproteobacteria", "__Xanthomonadales",
"__Sinobacteraceae", "__JTB255_marine_benthic_group"]}},{"id":
"denovo6126", "metadata": {"taxonomy": ["Bacteria",
"__Proteobacteria", "__Gammaproteobacteria", "__Xanthomonadales",
"__Sinobacteraceae", "__JTB255_marine_benthic_group"]}},{"id":
"denovo6130", "metadata": {"taxonomy": ["Bacteria",
"__Proteobacteria", "__Alphaproteobacteria", "__Rhizobiales",
"__Phyllobacteriaceae", "__Hoeflea"]}},{"id": "denovo6132",
"metadata": {"taxonomy": ["Bacteria", "__Planctomycetes",
"__Planctomycetacia", "__Planctomycetales", "__Planctomycetaceae",
"__Rhodopirellula"]}},{"id": "denovo6134", "metadata": {"taxonomy":
["Bacteria", "__Bacteroidetes", "__Flavobacteria",
"__Flavobacteriales", "__Cryomorphaceae", "__Owenweeksia"]}},{"id":
"denovo6138", "metadata": {"taxonomy": ["Bacteria",
"__Proteobacteria", "__Deltaproteobacteria", "__Myxococcales",
"__Sorangiineae", "__g"]}},{"id": "denovo6141", "metadata":
{"taxonomy": ["Bacteria", "__Proteobacteria", "__Deltaproteobacteria",
"__SAR324_clade(Marine_group_B)", "__f", "__g"]}},{"id": "denovo6142",
"metadata": {"taxonomy": ["Bacteria", "__Acidobacteria",
"__Acidobacteria", "__32-21", "__f", "__g"]}},{"id": "denovo6144",
"metadata": {"taxonomy": ["Bacteria", "__Proteobacteria",
"__Gammaproteobacteria", "__Alteromonadales", "__Alteromonadaceae",
"__Dasania"]}},{"id": "denovo6148", "metadata": {"taxonomy":
["Bacteria", "__Proteobacteria", "__TA18", "__o", "__f", "__g"]}},
{"id": "denovo6151", "metadata": {"taxonomy": ["Bacteria",
"__Proteobacteria", "__Deltaproteobacteria", "__Myxococcales",
"__0319-6G20", "__g"]}},{"id": "denovo6155", "metadata": {"taxonomy":
["Bacteria", "__Actinobacteria", "__Acidimicrobiia",

"__Acidimicrobiales", "__Sva0996_marine_group", "__g"]}},{"id":
"denovo6157", "metadata": {"taxonomy": ["Bacteria",
"__Proteobacteria", "__Alphaproteobacteria", "__Rhodospirillales",
"__Rhodospirillaceae"]}},{"id": "denovo6159", "metadata": {"taxonomy":
["Bacteria", "__Proteobacteria", "__Alphaproteobacteria",
"__SAR11_clade", "__Surface_1", "__Candidatus_Pelagibacter"]}},{"id":
"denovo6163", "metadata": {"taxonomy": ["Bacteria",
"__Proteobacteria", "__Gammaproteobacteria",
"__E01-9C-26_marine_group", "__f", "__g"]}},{"id": "denovo6164",
"metadata": {"taxonomy": ["Bacteria", "__Deferribacteres",
"__Deferribacteres", "__Deferribacterales", "__Family_Incertae_Sedis",
"__Caldithrix"]}},{"id": "denovo6172", "metadata": {"taxonomy":
["Bacteria", "__Proteobacteria", "__Gammaproteobacteria",
"__Order_Incertae_Sedis", "__Family_Incertae_Sedis",
"__Arenicella"]}},{"id": "denovo6174", "metadata": {"taxonomy":
["Bacteria", "__Proteobacteria", "__Deltaproteobacteria",
"__Bdellovibrionales", "__Bacteriovoracaceae", "__g"]}},{"id":
"denovo6177", "metadata": {"taxonomy": ["Bacteria",
"__Proteobacteria", "__Alphaproteobacteria", "__Rhodospirillales",
"__Rhodospirillaceae", "__g"]}},{"id": "denovo6180", "metadata":
{"taxonomy": ["Bacteria", "__Bacteroidetes", "__Cytophagia",
"__Cytophagales", "__Flammeovirgaceae",
"__Candidatus_Amoebophilus"]}},{"id": "denovo6182", "metadata":
{"taxonomy": ["Bacteria", "__Candidate_division_TM7", "__c", "__o",
"__f", "__g"]}},{"id": "denovo6183", "metadata": {"taxonomy":
["Bacteria", "__Proteobacteria", "__Alphaproteobacteria",
"__Rhodospirillales", "__Rhodospirillaceae", "__g"]}},{"id":
"denovo6185", "metadata": {"taxonomy": ["Bacteria", "__Bacteroidetes",
"__Flavobacteria", "__Flavobacteriales", "__Flavobacteriaceae",
"__NS3a_marine_group"]}},{"id": "denovo6189", "metadata": {"taxonomy":
["Archaea", "__Euryarchaeota", "__Halobacteria", "__Halobacteriales",
"__Deep_Sea_Euryarchaeotic_Group(DSEG)", "__g"]}},{"id": "denovo6194",
"metadata": {"taxonomy": ["Bacteria", "__Bacteroidetes"]}},{"id":
"denovo6195", "metadata": {"taxonomy": ["Bacteria",
"__Proteobacteria", "__Alphaproteobacteria", "__Rhizobiales",
"__Phyllobacteriaceae", "__Nitratireductor"]}},{"id": "denovo6203",
"metadata": {"taxonomy": ["Bacteria", "__Gemmatimonadetes",
"__Gemmatimonadetes", "__BD2-11_terrestrial_group", "__f", "__g"]}},
{"id": "denovo6205", "metadata": {"taxonomy": ["Bacteria",
"__Proteobacteria", "__Gammaproteobacteria", "__HOC36", "__f",
"__g"]}},{"id": "denovo6211", "metadata": {"taxonomy": ["Bacteria",
"__Proteobacteria", "__Gammaproteobacteria", "__Xanthomonadales",
"__Sinobacteraceae", "__JTB255_marine_benthic_group"]}},{"id":
"denovo6213", "metadata": {"taxonomy": ["Bacteria", "__Chlamydiae",
"__Chlamydiae", "__Chlamydiales", "__Simkaniaceae",
"__Candidatus_Fritschea"]}},{"id": "denovo6216", "metadata":
{"taxonomy": ["Bacteria", "__Acidobacteria", "__RB25", "__o", "__f",
"__g"]}},{"id": "denovo6222", "metadata": {"taxonomy": ["Bacteria",
"__Proteobacteria", "__Deltaproteobacteria", "__Sh765B-TzT-29", "__f",
"__g"]}},{"id": "denovo6224", "metadata": {"taxonomy": ["Archaea",

"__Euryarchaeota", "__Thermoplasmata", "__Thermoplasmatales", "__Marine_Group_II", "__g"]}},{"id": "denovo6225", "metadata": {"taxonomy": ["Bacteria", "__Bacteroidetes", "__Flavobacteria", "__Flavobacteriales", "__Flavobacteriaceae", "__Robiginitalea"]}}, {"id": "denovo6226", "metadata": {"taxonomy": ["Bacteria", "__Planctomycetes", "__vadinHA49", "__o", "__f", "__g"]}},{"id": "denovo6231", "metadata": {"taxonomy": ["Bacteria", "__Proteobacteria", "__Deltaproteobacteria", "__Desulfobacterales", "__Desulfobacteraceae"]}},{"id": "denovo6232", "metadata": {"taxonomy": ["Bacteria", "__Proteobacteria", "__Gammaproteobacteria", "__Alteromonadales", "__Alteromonadaceae", "__BD1-7_clade"]}},{"id": "denovo6234", "metadata": {"taxonomy": ["Bacteria", "__Bacteroidetes", "__Sphingobacteriia", "__Sphingobacteriales", "__Saprospiraceae", "__g"]}},{"id": "denovo6235", "metadata": {"taxonomy": ["Bacteria", "__Proteobacteria", "__Gammaproteobacteria", "__Alteromonadales", "__Alteromonadaceae", "__Haliea"]}},{"id": "denovo6237", "metadata": {"taxonomy": ["Bacteria", "__Cyanobacteria", "__Cyanobacteria", "__SubsectionIII", "__FamilyI", "__Phormidium"]}},{"id": "denovo6238", "metadata": {"taxonomy": ["Bacteria", "__Proteobacteria", "__Alphaproteobacteria", "__Rhodospirillales", "__Rhodospirillaceae"]}},{"id": "denovo6239", "metadata": {"taxonomy": ["Bacteria", "__Proteobacteria", "__Alphaproteobacteria", "__Rhodobacterales", "__Rhodobacteraceae"]}},{"id": "denovo6246", "metadata": {"taxonomy": ["Bacteria", "__Proteobacteria", "__Alphaproteobacteria", "__Rickettsiales"]}},{"id": "denovo6252", "metadata": {"taxonomy": ["Bacteria", "__Proteobacteria", "__Gammaproteobacteria", "__Alteromonadales", "__Alteromonadaceae", "__Candidatus_Endobugula"]}},{"id": "denovo6256", "metadata": {"taxonomy": ["Bacteria", "__Proteobacteria", "__Epsilonproteobacteria", "__Campylobacterales", "__Helicobacteraceae"]}},{"id": "denovo6259", "metadata": {"taxonomy": ["Archaea", "__Euryarchaeota", "__Halobacteria", "__Halobacteriales", "__Deep_Sea_Hydrothermal_Vent_Gp_6(DHVEG-6)", "__Candidatus_Parvarchaeum"]}},{"id": "denovo6260", "metadata": {"taxonomy": ["Bacteria", "__Proteobacteria", "__SPOTSOCT00m83", "__o", "__f", "__g"]}},{"id": "denovo6263", "metadata": {"taxonomy": ["Bacteria", "__Chloroflexi", "__Caldilineae", "__Caldilineales", "__Caldilineaceae", "__g"]}},{"id": "denovo6268", "metadata": {"taxonomy": ["Bacteria", "__Proteobacteria", "__Deltaproteobacteria", "__GR-WP33-30", "__f", "__g"]}},{"id": "denovo6270", "metadata": {"taxonomy": ["Bacteria", "__Proteobacteria", "__Deltaproteobacteria", "__Sh765B-TzT-29", "__f", "__g"]}},{"id": "denovo6273", "metadata": {"taxonomy": ["Bacteria", "__Proteobacteria", "__Deltaproteobacteria", "__Myxococcales", "__Cystobacterineae", "__g"]}},{"id": "denovo6276", "metadata": {"taxonomy": ["Bacteria", "__Bacteroidetes", "__Bacteroidia", "__Bacteroidales", "__Marinilabiaceae", "__g"]}}, {"id": "denovo6278", "metadata": {"taxonomy": ["Bacteria", "__Proteobacteria", "__Deltaproteobacteria", "__Myxococcales", "__0319-6G20", "__g"]}},{"id": "denovo6279", "metadata": {"taxonomy": ["Bacteria", "__Proteobacteria", "__Alphaproteobacteria",

"__Rhodospirillales", "__Rhodospirillaceae", "__Defluviicoccus"]}},
{"id": "denovo6281", "metadata": {"taxonomy": ["Bacteria",
"__Cyanobacteria", "__Cyanobacteria", "__SubsectionIII",
"__FamilyI"]}},{"id": "denovo6283", "metadata": {"taxonomy":
["Bacteria", "__Proteobacteria", "__Gammaproteobacteria",
"__Chromatiales", "__Ectothiorhodospiraceae", "__Acidiferrobacter"]}},
{"id": "denovo6285", "metadata": {"taxonomy": ["Bacteria"]}},{"id":
"denovo6290", "metadata": {"taxonomy": ["Bacteria",
"__Proteobacteria", "__Gammaproteobacteria", "__BD7-8_marine_group",
"__f", "__g"]}},{"id": "denovo6291", "metadata": {"taxonomy":
["Bacteria", "__Proteobacteria", "__Alphaproteobacteria",
"__Rhodobacterales", "__Rhodobacteraceae", "__Ruegeria"]}},{"id":
"denovo6292", "metadata": {"taxonomy": ["Bacteria",
"__Proteobacteria", "__Gammaproteobacteria"]}},{"id": "denovo6293",
"metadata": {"taxonomy": ["Bacteria", "__Proteobacteria", "__JTB23",
"__o", "__f", "__g"]}},{"id": "denovo6295", "metadata": {"taxonomy":
["Bacteria", "__Planctomycetes", "__Phycisphaerae",
"__Phycisphaerales", "__Phycisphaeraceae", "__Phycisphaera"]}},{"id":
"denovo6296", "metadata": {"taxonomy": ["Bacteria",
"__Proteobacteria", "__Gammaproteobacteria", "__Alteromonadales",
"__Alteromonadaceae", "__Haliea"]}},{"id": "denovo6300", "metadata":
{"taxonomy": ["Bacteria", "__Proteobacteria", "__Deltaproteobacteria",
"__Bdellovibrionales", "__Bdellovibrionaceae", "__Bdellovibrio"]}},
{"id": "denovo6301", "metadata": {"taxonomy": ["Bacteria",
"__Proteobacteria", "__Deltaproteobacteria", "__Myxococcales",
"__Haliangiaceae", "__Haliangium"]}},{"id": "denovo6302", "metadata":
{"taxonomy": ["Bacteria", "__Bacteroidetes", "__Cytophagia",
"__Order_II_Incertae_Sedis", "__Rhodothermaceae", "__g"]}},{"id":
"denovo6303", "metadata": {"taxonomy": ["Bacteria",
"__Bacteroidetes"]}},{"id": "denovo6304", "metadata": {"taxonomy":
["Bacteria", "__Gemmatimonadetes", "__Gemmatimonadetes",
"__Gemmatimonadales", "__Gemmatimonadaceae", "__g"]}},{"id":
"denovo6305", "metadata": {"taxonomy": ["Archaea", "__Thaumarchaeota",
"__Marine_Group_I", "__Candidatus_Nitrosopumilus", "__f", "__g"]}},
{"id": "denovo6306", "metadata": {"taxonomy": ["Bacteria",
"__Proteobacteria", "__Alphaproteobacteria", "__SAR11_clade",
"__Surface_1", "__Candidatus_Pelagibacter"]}},{"id": "denovo6309",
"metadata": {"taxonomy": ["Bacteria", "__Proteobacteria",
"__Gammaproteobacteria", "__Legionellales", "__Coxiellaceae",
"__Coxiella"]}},{"id": "denovo6311", "metadata": {"taxonomy":
["Bacteria", "__Proteobacteria", "__Alphaproteobacteria",
"__Rhodospirillales", "__Rhodospirillaceae", "__g"]}},{"id":
"denovo6313", "metadata": {"taxonomy": ["Bacteria", "__Bacteroidetes",
"__Flavobacteria", "__Flavobacteriales", "__Flavobacteriaceae",
"__Leptobacterium"]}},{"id": "denovo6318", "metadata": {"taxonomy":
["Bacteria", "__Proteobacteria", "__Deltaproteobacteria",
"__Desulfobacterales", "__Nitrospinaceae", "__Nitrospina"]}},{"id":
"denovo6320", "metadata": {"taxonomy": ["Bacteria",
"__Proteobacteria", "__Gammaproteobacteria", "__Xanthomonadales",
"__Sinobacteraceae", "__JTB255_marine_benthic_group"]}},{"id":

"denovo6321", "metadata": {"taxonomy": ["Bacteria", "__Chloroflexi", "__KD4-96", "__o", "__f", "__g"]}},{"id": "denovo6324", "metadata": {"taxonomy": ["Bacteria", "__Planctomycetes", "__Planctomycetacia", "__Planctomycetales", "__Planctomycetaceae", "__Planctomyces"]}}, {"id": "denovo6326", "metadata": {"taxonomy": ["Bacteria", "__Proteobacteria", "__Gammaproteobacteria", "__Alteromonadales", "__Alteromonadaceae", "__OM60(NOR5)_clade"]}},{"id": "denovo6328", "metadata": {"taxonomy": ["Bacteria", "__Bacteroidetes", "__Sphingobacteriia", "__Sphingobacteriales", "__Saprospiraceae", "__g"]}},{"id": "denovo6331", "metadata": {"taxonomy": ["Bacteria", "__Proteobacteria", "__Gammaproteobacteria", "__Order_Incertae_Sedis", "__Family_Incertae_Sedis", "__Marinicella"]}},{"id": "denovo6334", "metadata": {"taxonomy": ["Bacteria", "__Proteobacteria", "__Gammaproteobacteria", "__Alteromonadales", "__Alteromonadaceae", "__SAR92_clade"]}},{"id": "denovo6339", "metadata": {"taxonomy": ["Bacteria", "__Proteobacteria", "__Deltaproteobacteria", "__Bdellovibrionales", "__Bdellovibrionaceae", "__Bdellovibrio"]}}, {"id": "denovo6343", "metadata": {"taxonomy": ["Bacteria", "__Proteobacteria", "__Alphaproteobacteria", "__Sphingomonadales"]}}, {"id": "denovo6345", "metadata": {"taxonomy": ["Bacteria", "__Bacteroidetes", "__BD2-2", "__o", "__f", "__g"]}},{"id": "denovo6346", "metadata": {"taxonomy": ["Bacteria", "__Proteobacteria", "__Gammaproteobacteria"]}},{"id": "denovo6350", "metadata": {"taxonomy": ["Bacteria", "__Actinobacteria", "__Acidimicrobiia", "__Acidimicrobiales", "__OCS155_marine_group", "__g"]}},{"id": "denovo6355", "metadata": {"taxonomy": ["Bacteria", "__Proteobacteria", "__Alphaproteobacteria", "__Parvularculales", "__Parvularculaceae", "__Parvularcula"]}},{"id": "denovo6366", "metadata": {"taxonomy": ["Bacteria", "__Acidobacteria", "__Holophagae", "__CA002", "__f", "__g"]}},{"id": "denovo6367", "metadata": {"taxonomy": ["Bacteria", "__Proteobacteria", "__Alphaproteobacteria", "__Rickettsiales", "__LWSR-14", "__g"]}}, {"id": "denovo6369", "metadata": {"taxonomy": ["Bacteria", "__Proteobacteria", "__Deltaproteobacteria", "__Myxococcales", "__Sorangiineae", "__Sandaracinaceae"]}},{"id": "denovo6371", "metadata": {"taxonomy": ["Bacteria", "__Proteobacteria", "__Alphaproteobacteria", "__Rhodospirillales", "__Rhodospirillaceae", "__g"]}},{"id": "denovo6376", "metadata": {"taxonomy": ["Bacteria", "__Proteobacteria", "__Betaproteobacteria", "__Methylophilales", "__Methylophilaceae", "__OM43_clade"]}},{"id": "denovo6388", "metadata": {"taxonomy": ["Bacteria", "__Gemmatimonadetes", "__Gemmatimonadetes", "__Gemmatimonadales", "__Gemmatimonadaceae", "__g"]}},{"id": "denovo6390", "metadata": {"taxonomy": ["Bacteria", "__Proteobacteria", "__Alphaproteobacteria", "__MNG3", "__f", "__g"]}},{"id": "denovo6393", "metadata": {"taxonomy": ["Bacteria", "__Proteobacteria", "__Gammaproteobacteria"]}},{"id": "denovo6398", "metadata": {"taxonomy": ["Bacteria", "__Bacteroidetes"]}},{"id": "denovo6404", "metadata": {"taxonomy": ["Bacteria", "__Proteobacteria", "__Deltaproteobacteria", "__Myxococcales", "__Sorangiineae", "__Sandaracinaceae"]}},{"id": "denovo6405",

"metadata": {"taxonomy": ["Bacteria", "__Proteobacteria", "__Gammaproteobacteria", "__Xanthomonadales", "__Sinobacteraceae", "__JTB255_marine_benthic_group"]}},{"id": "denovo6407", "metadata": {"taxonomy": ["Bacteria", "__Proteobacteria", "__Alphaproteobacteria", "__DB1-14", "__f", "__g"]}},{"id": "denovo6408", "metadata": {"taxonomy": ["Bacteria", "__Proteobacteria", "__Alphaproteobacteria", "__Rhodospirillales", "__Rhodospirillaceae", "__Defluviicoccus"]}}, {"id": "denovo6409", "metadata": {"taxonomy": ["Bacteria", "__Proteobacteria", "__Alphaproteobacteria", "__Rhizobiales", "__Hyphomicrobiaceae", "__Filomicrobium"]}},{"id": "denovo6411", "metadata": {"taxonomy": ["Bacteria", "__Proteobacteria", "__Deltaproteobacteria", "__SAR324_clade(Marine_group_B)", "__f", "__g"]}},{"id": "denovo6413", "metadata": {"taxonomy": ["Bacteria", "__Proteobacteria", "__Alphaproteobacteria", "__Rhodospirillales", "__Rhodospirillaceae", "__Defluviicoccus"]}},{"id": "denovo6415", "metadata": {"taxonomy": ["Bacteria", "__Firmicutes", "__Clostridia", "__Clostridiales", "__Lachnospiraceae"]}},{"id": "denovo6416", "metadata": {"taxonomy": ["Bacteria"]}},{"id": "denovo6422", "metadata": {"taxonomy": ["Bacteria", "__Proteobacteria", "__Betaproteobacteria", "__Methylophilales", "__Methylophilaceae", "__OM43_clade"]}},{"id": "denovo6425", "metadata": {"taxonomy": ["Bacteria", "__Proteobacteria", "__Gammaproteobacteria", "__Oceanospirillales", "__Litoricolaceae", "__Litoricola"]}},{"id": "denovo6430", "metadata": {"taxonomy": ["Bacteria", "__Bacteroidetes", "__Flavobacteria", "__Flavobacteriales", "__Cryomorphaceae", "__Owenweeksia"]}},{"id": "denovo6431", "metadata": {"taxonomy": ["Bacteria", "__Proteobacteria", "__Gammaproteobacteria", "__Alteromonadales", "__Ferrimonadaceae", "__Ferrimonas"]}},{"id": "denovo6432", "metadata": {"taxonomy": ["Bacteria"]}},{"id": "denovo6433", "metadata": {"taxonomy": ["Bacteria", "__Bacteroidetes", "__Flavobacteria", "__Flavobacteriales", "__Flavobacteriaceae", "__Tenacibaculum"]}},{"id": "denovo6434", "metadata": {"taxonomy": ["Bacteria", "__Proteobacteria", "__Deltaproteobacteria", "__Bdellovibrionales", "__Bdellovibrionaceae", "__Bdellovibrio"]}}, {"id": "denovo6438", "metadata": {"taxonomy": ["Bacteria", "__Proteobacteria", "__Alphaproteobacteria"]}},{"id": "denovo6442", "metadata": {"taxonomy": ["Bacteria", "__Nitrospirae", "__Nitrospira", "__Nitrospirales", "__Nitrospiraceae", "__Nitrospira"]}},{"id": "denovo6446", "metadata": {"taxonomy": ["Bacteria", "__Proteobacteria", "__Gammaproteobacteria", "__Vibrionales", "__Vibrionaceae", "__Grimontia"]}},{"id": "denovo6449", "metadata": {"taxonomy": ["Bacteria", "__Proteobacteria", "__Deltaproteobacteria", "__Desulfobacterales", "__Desulfobulbaceae", "__g"]}},{"id": "denovo6450", "metadata": {"taxonomy": ["Bacteria", "__Firmicutes", "__Bacilli", "__Bacillales", "__Family_XI_Incertae_Sedis", "__Gemella"]}},{"id": "denovo6452", "metadata": {"taxonomy": ["Bacteria", "__Proteobacteria", "__CF2", "__o", "__f", "__g"]}}, {"id": "denovo6453", "metadata": {"taxonomy": ["Bacteria", "__Actinobacteria", "__Acidimicrobiia", "__Acidimicrobiales", "__OCS155_marine_group", "__g"]}},{"id": "denovo6455", "metadata":

{"taxonomy": ["Bacteria", "__Planctomycetes", "__vadinHA49", "__o", "__f", "__g"]}},{"id": "denovo6456", "metadata": {"taxonomy": ["Bacteria", "__Chloroflexi"]}},{"id": "denovo6461", "metadata": {"taxonomy": ["Bacteria", "__Proteobacteria", "__Alphaproteobacteria", "__Rhodobacterales", "__Rhodobacteraceae"]}},{"id": "denovo6463", "metadata": {"taxonomy": ["Bacteria", "__Cyanobacteria", "__Cyanobacteria", "__SubsectionII", "__FamilyII", "__Pleurocapsa"]}}, {"id": "denovo6464", "metadata": {"taxonomy": ["Bacteria", "__Proteobacteria", "__Deltaproteobacteria", "__Desulfobacterales", "__Nitrospinaceae", "__Nitrospina"]}},{"id": "denovo6467", "metadata": {"taxonomy": ["Bacteria", "__Proteobacteria", "__Deltaproteobacteria", "__Desulfobacterales", "__Desulfobulbaceae", "__Desulforhopalus"]}}, {"id": "denovo6468", "metadata": {"taxonomy": ["Bacteria", "__Proteobacteria", "__Gammaproteobacteria", "__Alteromonadales", "__Alteromonadaceae", "__OM60(NOR5)_clade"]}},{"id": "denovo6470", "metadata": {"taxonomy": ["Bacteria", "__Proteobacteria", "__Alphaproteobacteria", "__OCS116_clade", "__f", "__g"]}},{"id": "denovo6471", "metadata": {"taxonomy": ["Bacteria", "__Proteobacteria", "__Gammaproteobacteria", "__Alteromonadales", "__Pseudoalteromonadaceae", "__g"]}},{"id": "denovo6473", "metadata": {"taxonomy": ["Bacteria", "__Cyanobacteria", "__Cyanobacteria", "__SubsectionI", "__FamilyI", "__Acaryochloris"]}},{"id": "denovo6474", "metadata": {"taxonomy": ["Bacteria", "__Planctomycetes", "__Phycisphaerae", "__Phycisphaerales", "__Phycisphaeraceae", "__SM1A02"]}},{"id": "denovo6476", "metadata": {"taxonomy": ["Bacteria", "__Bacteroidetes", "__Sphingobacteriia", "__Sphingobacteriales", "__Saprospiraceae", "__Lewinella"]}},{"id": "denovo6477", "metadata": {"taxonomy": ["Bacteria", "__Chloroflexi", "__Caldilineae", "__Caldilineales", "__Caldilineaceae", "__Caldilinea"]}},{"id": "denovo6480", "metadata": {"taxonomy": ["Bacteria", "__Proteobacteria", "__Gammaproteobacteria", "__Chromatiales", "__Chromatiaceae", "__Rheinheimera"]}},{"id": "denovo6482", "metadata": {"taxonomy": ["Bacteria", "__Proteobacteria", "__Gammaproteobacteria", "__Pseudomonadales", "__Moraxellaceae", "__Acinetobacter"]}},{"id": "denovo6484", "metadata": {"taxonomy": ["Bacteria", "__Proteobacteria", "__Gammaproteobacteria", "__Oceanospirillales", "__OM182_clade", "__g"]}},{"id": "denovo6491", "metadata": {"taxonomy": ["Bacteria", "__Actinobacteria", "__Actinobacteria", "__Propionibacteriales", "__Propionibacteriaceae", "__Jiangella"]}},{"id": "denovo6502", "metadata": {"taxonomy": ["Bacteria", "__Actinobacteria", "__Actinobacteria", "__Actinomycetales", "__Actinomycetaceae", "__Actinobaculum"]}},{"id": "denovo6503", "metadata": {"taxonomy": ["Bacteria", "__Proteobacteria", "__Alphaproteobacteria", "__Rhodospirillales", "__Rhodospirillaceae", "__g"]}},{"id": "denovo6507", "metadata": {"taxonomy": ["Bacteria", "__Actinobacteria", "__Acidimicrobiia", "__Acidimicrobiales", "__OCS155_marine_group", "__g"]}},{"id": "denovo6508", "metadata": {"taxonomy": ["Bacteria", "__Proteobacteria", "__Gammaproteobacteria", "__Enterobacteriales", "__Enterobacteriaceae"]}},{"id": "denovo6509",

"metadata": {"taxonomy": ["Bacteria", "__Bacteroidetes",
"__Flavobacteria", "__Flavobacteriales", "__Flavobacteriaceae",
"__Maribacter"]}},{"id": "denovo6511", "metadata": {"taxonomy":
["Bacteria", "__Bacteroidetes", "__Sphingobacteriia",
"__Sphingobacteriales", "__Saprospiraceae", "__g"]}},{"id":
"denovo6515", "metadata": {"taxonomy": ["Bacteria",
"__Proteobacteria", "__Alphaproteobacteria"]}},{"id": "denovo6516",
"metadata": {"taxonomy": ["Bacteria", "__Proteobacteria",
"__Gammaproteobacteria", "__Order_Incertae_Sedis",
"__Family_Incertae_Sedis", "__Marinicella"]}},{"id": "denovo6520",
"metadata": {"taxonomy": ["Bacteria", "__Proteobacteria",
"__Deltaproteobacteria", "__Myxococcales", "__Cystobacterineae",
"__g"]}},{"id": "denovo6524", "metadata": {"taxonomy": ["Bacteria",
"__Bacteroidetes", "__Flavobacteria", "__Flavobacteriales",
"__Flavobacteriaceae", "__Ulvibacter"]}},{"id": "denovo6526",
"metadata": {"taxonomy": ["Bacteria", "__Chloroflexi"]}},{"id":
"denovo6528", "metadata": {"taxonomy": ["Bacteria", "__Acidobacteria",
"__Acidobacteria", "__BPC015", "__f", "__g"]}},{"id": "denovo6533",
"metadata": {"taxonomy": ["Bacteria", "__Proteobacteria",
"__Deltaproteobacteria", "__Desulfobacterales", "__Desulfobulbaceae",
"__Desulfobulbus"]}},{"id": "denovo6534", "metadata": {"taxonomy":
["Bacteria", "__Proteobacteria", "__Alphaproteobacteria",
"__Rhodobacterales", "__Rhodobacteraceae", "__g"]}},{"id":
"denovo6536", "metadata": {"taxonomy": ["Bacteria",
"__Proteobacteria", "__Gammaproteobacteria", "__Order_Incertae_Sedis",
"__Family_Incertae_Sedis", "__Marinicella"]}},{"id": "denovo6537",
"metadata": {"taxonomy": ["Bacteria", "__Proteobacteria",
"__Alphaproteobacteria", "__Rhodospirillales", "__Rhodospirillaceae",
"__g"]}},{"id": "denovo6540", "metadata": {"taxonomy": ["Bacteria",
"__Proteobacteria", "__Alphaproteobacteria", "__Rhizobiales",
"__Phyllobacteriaceae", "__Nitratireductor"]}},{"id": "denovo6543",
"metadata": {"taxonomy": ["Bacteria", "__Bacteroidetes",
"__Bacteroidia", "__Bacteroidales", "__Marinilabiaceae",
"__Marinifilum"]}},{"id": "denovo6544", "metadata": {"taxonomy":
["Bacteria", "__Proteobacteria", "__Alphaproteobacteria",
"__Rickettsiales", "__SM2D12", "__g"]}},{"id": "denovo6545",
"metadata": {"taxonomy": ["Bacteria", "__Bacteroidetes",
"__Flavobacteria", "__Flavobacteriales", "__Cryomorphaceae",
"__Owenweeksia"]}},{"id": "denovo6551", "metadata": {"taxonomy":
["Bacteria", "__Proteobacteria", "__Alphaproteobacteria",
"__Rhodobacterales", "__Rhodobacteraceae"]}},{"id": "denovo6552",
"metadata": {"taxonomy": ["Bacteria", "__Firmicutes", "__Clostridia",
"__Clostridiales", "__Clostridiaceae"]}},{"id": "denovo6553",
"metadata": {"taxonomy": ["Bacteria", "__Bacteroidetes",
"__Sphingobacteriia", "__Sphingobacteriales", "__Saprospiraceae",
"__Aureispira"]}},{"id": "denovo6558", "metadata": {"taxonomy":
["Bacteria", "__Proteobacteria", "__Alphaproteobacteria",
"__OCS116_clade", "__f", "__g"]}},{"id": "denovo6560", "metadata":
{"taxonomy": ["Bacteria", "__Proteobacteria", "__Deltaproteobacteria",
"__Sva0485", "__f", "__g"]}},{"id": "denovo6561", "metadata":

{"taxonomy": ["Bacteria", "__Proteobacteria", "__Deltaproteobacteria", "__Desulfobacterales", "__Nitrospinaceae", "__Candidatus_Entotheonella"]}},{"id": "denovo6563", "metadata": {"taxonomy": ["Bacteria", "__Proteobacteria", "__Gammaproteobacteria"]}},{"id": "denovo6566", "metadata": {"taxonomy": ["Bacteria", "__Cyanobacteria", "__Cyanobacteria", "__SubsectionII", "__FamilyII", "__Pleurocapsa"]}},{"id": "denovo6569", "metadata": {"taxonomy": ["Bacteria", "__Bacteroidetes", "__Flavobacteria", "__Flavobacteriales", "__Flavobacteriaceae"]}},{"id": "denovo6574", "metadata": {"taxonomy": ["Bacteria", "__Cyanobacteria", "__MLE1-12", "__o", "__f", "__g"]}},{"id": "denovo6577", "metadata": {"taxonomy": ["Bacteria", "__Proteobacteria", "__Alphaproteobacteria", "__Rhodobacterales", "__Rhodobacteraceae", "__Rhodobacter"]}},{"id": "denovo6586", "metadata": {"taxonomy": ["Bacteria", "__Firmicutes", "__Clostridia", "__Halanaerobiales", "__Halobacteroidaceae", "__g"]}},{"id": "denovo6595", "metadata": {"taxonomy": ["Bacteria", "__Proteobacteria", "__Gammaproteobacteria", "__Pseudomonadales", "__Moraxellaceae", "__Acinetobacter"]}},{"id": "denovo6599", "metadata": {"taxonomy": ["Bacteria", "__Proteobacteria", "__Betaproteobacteria", "__Rhodocyclales", "__Rhodocyclaceae", "__Propionivibrio"]}},{"id": "denovo6600", "metadata": {"taxonomy": ["Bacteria", "__Actinobacteria", "__Acidimicrobiia", "__Acidimicrobiales", "__OCS155_marine_group", "__g"]}},{"id": "denovo6601", "metadata": {"taxonomy": ["Bacteria", "__Bacteroidetes", "__Flavobacteria", "__Flavobacteriales", "__Flavobacteriaceae", "__NS5_marine_group"]}},{"id": "denovo6603", "metadata": {"taxonomy": ["Bacteria", "__Proteobacteria", "__Alphaproteobacteria", "__Rhodobacterales", "__Rhodobacteraceae", "__Roseobacter"]}},{"id": "denovo6604", "metadata": {"taxonomy": ["Bacteria", "__Proteobacteria", "__Gammaproteobacteria", "__Alteromonadales", "__Alteromonadaceae", "__Haliea"]}},{"id": "denovo6605", "metadata": {"taxonomy": ["Bacteria", "__Proteobacteria", "__Deltaproteobacteria", "__Desulfobacterales", "__Desulfobacteraceae", "__Desulfatirhabdium"]}},{"id": "denovo6612", "metadata": {"taxonomy": ["Bacteria", "__Bacteroidetes", "__BD2-2", "__o", "__f", "__g"]}},{"id": "denovo6614", "metadata": {"taxonomy": ["Bacteria", "__Proteobacteria", "__Gammaproteobacteria", "__Order_Incertae_Sedis", "__Family_Incertae_Sedis", "__Marinicella"]}},{"id": "denovo6617", "metadata": {"taxonomy": ["Bacteria", "__Proteobacteria", "__Deltaproteobacteria", "__Myxococcales", "__Sorangiineae", "__g"]}},{"id": "denovo6621", "metadata": {"taxonomy": ["Bacteria", "__Proteobacteria", "__CF2", "__o", "__f", "__g"]}},{"id": "denovo6622", "metadata": {"taxonomy": ["Bacteria", "__Proteobacteria", "__Alphaproteobacteria", "__Rhodospirillales"]}},{"id": "denovo6626", "metadata": {"taxonomy": ["Bacteria", "__Candidate_division_OD1", "__c", "__o", "__f", "__g"]}},{"id": "denovo6629", "metadata": {"taxonomy": ["Bacteria", "__Proteobacteria", "__Alphaproteobacteria", "__Rhizobiales", "__Rhizobiaceae", "__Rhizobium"]}},{"id": "denovo6630", "metadata":

{"taxonomy": ["Bacteria", "__Proteobacteria", "__Alphaproteobacteria", "__Rhizobiales", "__Hyphomicrobiaceae", "__Devosia"]}},{"id": "denovo6632", "metadata": {"taxonomy": ["Bacteria", "__Proteobacteria", "__Gammaproteobacteria", "__Chromatiales", "__Chromatiaceae", "__Nitrosococcus"]}},{"id": "denovo6633", "metadata": {"taxonomy": ["Bacteria", "__Proteobacteria", "__Gammaproteobacteria", "__EC3", "__f", "__g"]}},{"id": "denovo6634", "metadata": {"taxonomy": ["Bacteria", "__Firmicutes", "__Bacilli", "__Bacillales", "__Planococcaceae", "__Bhargavaea"]}},{"id": "denovo6643", "metadata": {"taxonomy": ["Bacteria", "__Proteobacteria", "__Gammaproteobacteria", "__Order_Incertae_Sedis", "__Family_Incertae_Sedis", "__Thiohalophilus"]}},{"id": "denovo6647", "metadata": {"taxonomy": ["Bacteria", "__Firmicutes", "__Clostridia", "__Clostridiales", "__Family_Incertae_Sedis", "__Dethiosulfatibacter"]}},{"id": "denovo6649", "metadata": {"taxonomy": ["Bacteria", "__Proteobacteria", "__Deltaproteobacteria", "__Sh765B-TzT-29", "__f", "__g"]}},{"id": "denovo6650", "metadata": {"taxonomy": ["Bacteria", "__Cyanobacteria", "__SM2F09", "__o", "__f", "__g"]}},{"id": "denovo6657", "metadata": {"taxonomy": ["Bacteria", "__Verrucomicrobia", "__Verrucomicrobiae", "__Verrucomicrobiales", "__Verrucomicrobiaceae", "__Persicirhabdus"]}},{"id": "denovo6658", "metadata": {"taxonomy": ["Bacteria", "__Proteobacteria", "__Alphaproteobacteria", "__OCS116_clade", "__f", "__g"]}},{"id": "denovo6660", "metadata": {"taxonomy": ["Bacteria", "__Proteobacteria", "__Gammaproteobacteria", "__Alteromonadales", "__Alteromonadaceae", "__BD1-7_clade"]}},{"id": "denovo6662", "metadata": {"taxonomy": ["Bacteria", "__Proteobacteria", "__Alphaproteobacteria", "__Rhodobacterales", "__Rhodobacteraceae", "__Roseobacter_clade_AS-21_lineage"]}},{"id": "denovo6663", "metadata": {"taxonomy": ["Bacteria", "__Proteobacteria", "__Deltaproteobacteria", "__Desulfobacterales", "__Desulfobulbaceae", "__Desulfobulbus"]}},{"id": "denovo6665", "metadata": {"taxonomy": ["Bacteria", "__Proteobacteria", "__Alphaproteobacteria", "__Rhodospirillales", "__Rhodospirillaceae", "__g"]}},{"id": "denovo6666", "metadata": {"taxonomy": ["Bacteria", "__Proteobacteria", "__Gammaproteobacteria", "__Oceanospirillales"]}},{"id": "denovo6669", "metadata": {"taxonomy": ["Bacteria", "__Proteobacteria", "__Alphaproteobacteria", "__Rhodobacterales", "__Rhodobacteraceae"]}},{"id": "denovo6677", "metadata": {"taxonomy": ["Bacteria", "__Proteobacteria", "__Alphaproteobacteria", "__Sphingomonadales", "__Sphingomonadaceae", "__Sphingomonas"]}},{"id": "denovo6679", "metadata": {"taxonomy": ["Bacteria", "__Proteobacteria", "__Alphaproteobacteria", "__Rickettsiales", "__Rickettsiaceae", "__g"]}},{"id": "denovo6681", "metadata": {"taxonomy": ["Bacteria", "__Proteobacteria", "__Gammaproteobacteria", "__Alteromonadales", "__Alteromonadaceae", "__Haliea"]}},{"id": "denovo6687", "metadata": {"taxonomy": ["Bacteria", "__Proteobacteria", "__Alphaproteobacteria", "__Rhodobacterales", "__Rhodobacteraceae"]}},{"id": "denovo6692", "metadata": {"taxonomy": ["Bacteria", "__Proteobacteria", "__Deltaproteobacteria",

"__Desulfobacterales", "__Desulfobacteraceae", "__SEEP-SRB1"]}},{"id":
"denovo6694", "metadata": {"taxonomy": ["Bacteria",
"__Proteobacteria", "__Alphaproteobacteria", "__Rickettsiales",
"__S25-593", "__g"]}},{"id": "denovo6698", "metadata": {"taxonomy":
["Bacteria", "__Verrucomicrobia", "__Verrucomicrobiae",
"__Verrucomicrobiales", "__Verrucomicrobiaceae", "__Roseibacillus"]}},
{"id": "denovo6699", "metadata": {"taxonomy": ["Bacteria",
"__Proteobacteria", "__Gammaproteobacteria", "__Alteromonadales",
"__Ferrimonadaceae", "__Ferrimonas"]}},{"id": "denovo6701",
"metadata": {"taxonomy": ["Bacteria", "__Proteobacteria",
"__Gammaproteobacteria"]}},{"id": "denovo6702", "metadata":
{"taxonomy": ["Bacteria", "__Acidobacteria", "__RB25", "__o", "__f",
"__g"]}},{"id": "denovo6704", "metadata": {"taxonomy": ["Bacteria",
"__Proteobacteria", "__Alphaproteobacteria", "__Rhodospirillales",
"__Rhodospirillaceae", "__Defluviicoccus"]}},{"id": "denovo6711",
"metadata": {"taxonomy": ["Bacteria", "__Proteobacteria",
"__Deltaproteobacteria", "__Sh765B-TzT-29", "__f", "__g"]}},{"id":
"denovo6716", "metadata": {"taxonomy": ["Bacteria",
"__Planctomycetes", "__Phycisphaerae", "__Phycisphaerales",
"__Phycisphaeraceae", "__SM1A02"]}},{"id": "denovo6719", "metadata":
{"taxonomy": ["Bacteria", "__Proteobacteria",
"__Gammaproteobacteria"]}},{"id": "denovo6722", "metadata":
{"taxonomy": ["Archaea", "__Thaumarchaeota", "__Marine_Group_I",
"__Candidatus_Nitrosopumilus", "__f", "__g"]}},{"id": "denovo6724",
"metadata": {"taxonomy": ["Bacteria", "__Proteobacteria",
"__Gammaproteobacteria", "__Vibrionales", "__Vibrionaceae"]}},{"id":
"denovo6725", "metadata": {"taxonomy": ["Bacteria",
"__Proteobacteria", "__Alphaproteobacteria", "__Caulobacterales",
"__Caulobacteraceae", "__Brevundimonas"]}},{"id": "denovo6726",
"metadata": {"taxonomy": ["Bacteria", "__Proteobacteria",
"__Gammaproteobacteria", "__Xanthomonadales", "__Sinobacteraceae",
"__JTB255_marine_benthic_group"]}},{"id": "denovo6727", "metadata":
{"taxonomy": ["Bacteria", "__Proteobacteria", "__Alphaproteobacteria",
"__Rhizobiales", "__Phyllobacteriaceae"]}},{"id": "denovo6728",
"metadata": {"taxonomy": ["Bacteria", "__Acidobacteria",
"__Acidobacteria", "__BPC102", "__f", "__g"]}},{"id": "denovo6731",
"metadata": {"taxonomy": ["Bacteria", "__Proteobacteria",
"__Deltaproteobacteria", "__Desulfurellales", "__Desulfurellaceae",
"__g"]}},{"id": "denovo6733", "metadata": {"taxonomy": ["Bacteria",
"__Proteobacteria", "__Alphaproteobacteria", "__SAR11_clade",
"__Surface_1", "__Candidatus_Pelagibacter"]}},{"id": "denovo6735",
"metadata": {"taxonomy": ["Bacteria", "__Acidobacteria",
"__Acidobacteria", "__BPC015", "__f", "__g"]}},{"id": "denovo6739",
"metadata": {"taxonomy": ["Bacteria", "__Bacteroidetes",
"__Flavobacteria", "__Flavobacteriales", "__Flavobacteriaceae",
"__Algibacter"]}},{"id": "denovo6741", "metadata": {"taxonomy":
["Bacteria", "__Bacteroidetes", "__Sphingobacteriia",
"__Sphingobacteriales", "__Chitinophagaceae", "__g"]}},{"id":
"denovo6742", "metadata": {"taxonomy": ["Bacteria",
"__Candidate_division_OD1", "__c", "__o", "__f", "__g"]}},{"id":

"denovo6744", "metadata": {"taxonomy": ["Bacteria", "__Bacteroidetes", "__Sphingobacteriia", "__Sphingobacteriales", "__Chitinophagaceae", "__g"]}},{"id": "denovo6746", "metadata": {"taxonomy": ["Bacteria", "__Proteobacteria", "__Deltaproteobacteria", "__SAR324_clade(Marine_group_B)", "__f", "__g"]}},{"id": "denovo6747", "metadata": {"taxonomy": ["Bacteria", "__Proteobacteria", "__Alphaproteobacteria", "__OCS116_clade", "__f", "__g"]}},{"id": "denovo6751", "metadata": {"taxonomy": ["Bacteria", "__Bacteroidetes", "__Sphingobacteriia", "__Sphingobacteriales", "__Saprospiraceae", "__g"]}},{"id": "denovo6759", "metadata": {"taxonomy": ["Bacteria", "__Bacteroidetes", "__Sphingobacteriia", "__Sphingobacteriales", "__Saprospiraceae", "__g"]}},{"id": "denovo6765", "metadata": {"taxonomy": ["Bacteria", "__Proteobacteria", "__Gammaproteobacteria", "__Oceanospirillales", "__Oceanospirillaceae", "__Oceanobacter"]}},{"id": "denovo6767", "metadata": {"taxonomy": ["Bacteria", "__Proteobacteria", "__Alphaproteobacteria", "__Rhizobiales", "__Phyllobacteriaceae", "__Ahrensia"]}},{"id": "denovo6773", "metadata": {"taxonomy": ["Bacteria", "__Proteobacteria", "__Alphaproteobacteria", "__Rhodobacterales", "__Rhodobacteraceae"]}},{"id": "denovo6781", "metadata": {"taxonomy": ["Bacteria", "__Verrucomicrobia", "__Verrucomicrobiae", "__Verrucomicrobiales", "__Verrucomicrobiaceae", "__g"]}},{"id": "denovo6782", "metadata": {"taxonomy": ["Bacteria", "__Actinobacteria", "__Acidimicrobiia", "__Acidimicrobiales", "__OCS155_marine_group", "__g"]}},{"id": "denovo6784", "metadata": {"taxonomy": ["Bacteria", "__Bacteroidetes", "__Flavobacteria", "__Flavobacteriales", "__Flavobacteriaceae", "__Algibacter"]}},{"id": "denovo6785", "metadata": {"taxonomy": ["Bacteria", "__Chloroflexi", "__Anaerolineae", "__Anaerolineales", "__Anaerolineaceae", "__g"]}},{"id": "denovo6793", "metadata": {"taxonomy": ["Bacteria", "__Proteobacteria", "__Gammaproteobacteria", "__Chromatiales", "__Chromatiaceae", "__Nitrosococcus"]}},{"id": "denovo6794", "metadata": {"taxonomy": ["Bacteria", "__Proteobacteria", "__Alphaproteobacteria", "__Rhodospirillales", "__Rhodospirillaceae", "__Thalassospira"]}},{"id": "denovo6799", "metadata": {"taxonomy": ["Bacteria", "__Proteobacteria", "__Alphaproteobacteria", "__Rhodobacterales", "__Rhodobacteraceae", "__Labrenzia"]}},{"id": "denovo6800", "metadata": {"taxonomy": ["Bacteria", "__Proteobacteria", "__Alphaproteobacteria", "__Rhodobacterales", "__Rhodobacteraceae"]}},{"id": "denovo6801", "metadata": {"taxonomy": ["Bacteria", "__Chloroflexi", "__SAR202_clade", "__o", "__f", "__g"]}},{"id": "denovo6804", "metadata": {"taxonomy": ["Bacteria", "__Spirochaetes", "__Spirochaetes", "__Spirochaetales", "__Leptospiraceae", "__Turneriella"]}},{"id": "denovo6805", "metadata": {"taxonomy": ["Bacteria", "__Proteobacteria", "__Alphaproteobacteria", "__Rhodobacterales", "__Rhodobacteraceae"]}},{"id": "denovo6808", "metadata": {"taxonomy": ["Bacteria", "__Bacteroidetes", "__Sphingobacteriia", "__Sphingobacteriales", "__NS11-12_marine_group", "__g"]}},{"id": "denovo6811", "metadata": {"taxonomy": ["Bacteria", "__Proteobacteria", "__Deltaproteobacteria",

"__Desulfobacterales", "__Desulfobulbaceae", "__g"]}},{"id": "denovo6812", "metadata": {"taxonomy": ["Bacteria", "__Bacteroidetes", "__Cytophagia", "__Cytophagales", "__Flammeovirgaceae", "__Reichenbachiella"]}},{"id": "denovo6815", "metadata": {"taxonomy": ["Bacteria"]}},{"id": "denovo6818", "metadata": {"taxonomy": ["Bacteria", "__Candidate_division_WS3", "__c", "__o", "__f", "__g"]}},{"id": "denovo6819", "metadata": {"taxonomy": ["Bacteria", "__Proteobacteria", "__Alphaproteobacteria", "__Rhodobacterales", "__Rhodobacteraceae", "__Rhodovulum"]}},{"id": "denovo6820", "metadata": {"taxonomy": ["Bacteria", "__Gemmatimonadetes", "__Gemmatimonadetes", "__PAUC43f_marine_benthic_group", "__f", "__g"]}},{"id": "denovo6823", "metadata": {"taxonomy": ["Bacteria", "__Planctomycetes", "__Planctomycetacia", "__Brocadiales", "__Brocadiaceae"]}},{"id": "denovo6824", "metadata": {"taxonomy": ["Bacteria", "__Verrucomicrobia", "__OPB35_soil_group", "__o", "__f", "__g"]}},{"id": "denovo6825", "metadata": {"taxonomy": ["Bacteria", "__Bacteroidetes", "__Flavobacteria", "__Flavobacteriales", "__Flavobacteriaceae", "__Aquimarina"]}},{"id": "denovo6826", "metadata": {"taxonomy": ["Bacteria", "__Bacteroidetes", "__Flavobacteria", "__Flavobacteriales", "__Cryomorphaceae", "__Owenweeksia"]}},{"id": "denovo6827", "metadata": {"taxonomy": ["Bacteria", "__Proteobacteria", "__Alphaproteobacteria", "__Rhodospirillales", "__Rhodospirillaceae", "__g"]}},{"id": "denovo6828", "metadata": {"taxonomy": ["Bacteria", "__Proteobacteria", "__Alphaproteobacteria", "__Sphingomonadales", "__Erythrobacteraceae"]}},{"id": "denovo6829", "metadata": {"taxonomy": ["Bacteria", "__Proteobacteria", "__Alphaproteobacteria", "__Rhodobacterales", "__Rhodobacteraceae", "__Roseobacter_clade_NAC11-7_lineage"]}},{"id": "denovo6830", "metadata": {"taxonomy": ["Bacteria", "__Proteobacteria", "__Alphaproteobacteria", "__Rhodospirillales", "__Rhodospirillaceae", "__Defluviicoccus"]}},{"id": "denovo6833", "metadata": {"taxonomy": ["Bacteria", "__Proteobacteria", "__Alphaproteobacteria", "__Rickettsiales", "__Candidatus_Captivus", "__g"]}},{"id": "denovo6837", "metadata": {"taxonomy": ["Bacteria", "__Bacteroidetes", "__Flavobacteria", "__Flavobacteriales", "__Flavobacteriaceae", "__Zeaxanthinibacter"]}},{"id": "denovo6839", "metadata": {"taxonomy": ["Bacteria", "__Proteobacteria", "__Deltaproteobacteria", "__Sh765B-TzT-29", "__f", "__g"]}},{"id": "denovo6844", "metadata": {"taxonomy": ["Bacteria", "__Cyanobacteria", "__Cyanobacteria", "__SubsectionI", "__FamilyI", "__Synechococcus"]}},{"id": "denovo6847", "metadata": {"taxonomy": ["Bacteria", "__Proteobacteria", "__Alphaproteobacteria", "__Rhodobacterales", "__Rhodobacteraceae"]}},{"id": "denovo6853", "metadata": {"taxonomy": ["Bacteria", "__Proteobacteria", "__Gammaproteobacteria", "__KI89A_clade", "__f", "__g"]}},{"id": "denovo6858", "metadata": {"taxonomy": ["Bacteria", "__Nitrospirae", "__Nitrospira", "__Nitrospirales", "__Nitrospiraceae", "__Nitrospira"]}},{"id": "denovo6859", "metadata": {"taxonomy": ["Bacteria", "__Proteobacteria", "__Gammaproteobacteria", "__Vibrionales", "__Vibrionaceae", "__Aliivibrio"]}},{"id":

"denovo6860", "metadata": {"taxonomy": ["Bacteria", "__Proteobacteria", "__SPOTSOCT00m83", "__o", "__f", "__g"]}},{"id": "denovo6865", "metadata": {"taxonomy": ["Bacteria", "__Proteobacteria", "__Gammaproteobacteria", "__Order_Incertae_Sedis", "__Family_Incertae_Sedis", "__Sedimenticola"]}},{"id": "denovo6868", "metadata": {"taxonomy": ["Bacteria", "__Proteobacteria", "__Alphaproteobacteria", "__Rhodobacterales", "__Rhodobacteraceae"]}},{"id": "denovo6870", "metadata": {"taxonomy": ["Bacteria", "__Proteobacteria", "__Alphaproteobacteria", "__Rhizobiales", "__Brucellaceae"]}},{"id": "denovo6872", "metadata": {"taxonomy": ["Bacteria", "__Proteobacteria", "__Gammaproteobacteria", "__KI89A_clade", "__f", "__g"]}},{"id": "denovo6878", "metadata": {"taxonomy": ["Bacteria", "__Proteobacteria", "__Alphaproteobacteria", "__Caulobacterales", "__Hyphomonadaceae", "__Maricaulis"]}},{"id": "denovo6880", "metadata": {"taxonomy": ["Bacteria", "__Proteobacteria", "__Gammaproteobacteria", "__EC3", "__f", "__g"]}},{"id": "denovo6881", "metadata": {"taxonomy": ["Bacteria", "__Proteobacteria", "__Alphaproteobacteria", "__Rhodospirillales", "__Family_Incertae_Sedis", "__Geminicoccus"]}},{"id": "denovo6884", "metadata": {"taxonomy": ["Bacteria", "__Proteobacteria", "__Alphaproteobacteria", "__Rhodobacterales", "__Rhodobacteraceae"]}},{"id": "denovo6885", "metadata": {"taxonomy": ["Bacteria", "__Bacteroidetes", "__Cytophagia", "__Cytophagales", "__Flammeovirgaceae", "__Candidatus_Amoebophilus"]}},{"id": "denovo6888", "metadata": {"taxonomy": ["Bacteria", "__Firmicutes", "__Clostridia", "__Clostridiales", "__Family_XI_Incertae_Sedis", "__Helcococcus"]}},{"id": "denovo6892", "metadata": {"taxonomy": ["Bacteria", "__Proteobacteria", "__Alphaproteobacteria", "__S26-47", "__f", "__g"]}},{"id": "denovo6893", "metadata": {"taxonomy": ["Bacteria", "__Cyanobacteria", "__Cyanobacteria", "__SubsectionI", "__FamilyI", "__Synechococcus"]}},{"id": "denovo6894", "metadata": {"taxonomy": ["Bacteria", "__Proteobacteria", "__Gammaproteobacteria", "__Enterobacteriales", "__Enterobacteriaceae", "__Escherichia-Shigella"]}},{"id": "denovo6903", "metadata": {"taxonomy": ["Bacteria", "__Proteobacteria", "__Alphaproteobacteria", "__Rhodobacterales", "__Rhodobacteraceae"]}},{"id": "denovo6905", "metadata": {"taxonomy": ["Bacteria", "__Proteobacteria", "__Gammaproteobacteria", "__Alteromonadales", "__Alteromonadaceae", "__BD2-7"]}},{"id": "denovo6906", "metadata": {"taxonomy": ["Bacteria", "__Proteobacteria", "__Gammaproteobacteria", "__Oceanospirillales", "__Oceanospirillaceae", "__Amphritea"]}},{"id": "denovo6909", "metadata": {"taxonomy": ["Bacteria", "__Proteobacteria", "__Gammaproteobacteria", "__Xanthomonadales", "__Sinobacteraceae", "__JTB255_marine_benthic_group"]}},{"id": "denovo6911", "metadata": {"taxonomy": ["Bacteria", "__Proteobacteria", "__Gammaproteobacteria", "__Order_Incertae_Sedis", "__Family_Incertae_Sedis", "__Marinicella"]}},{"id": "denovo6913", "metadata": {"taxonomy": ["Bacteria", "__Firmicutes", "__Clostridia", "__Clostridiales", "__Lachnospiraceae"]}},{"id": "denovo6918", "metadata": {"taxonomy": ["Bacteria", "__Bacteroidetes"]}},{"id":

"denovo6919", "metadata": {"taxonomy": ["Bacteria", "__Proteobacteria", "__Deltaproteobacteria", "__Sh765B-TzT-29", "__f", "__g"]}},{"id": "denovo6920", "metadata": {"taxonomy": ["Bacteria", "__Proteobacteria", "__Alphaproteobacteria", "__Rhodospirillales", "__Rhodospirillaceae", "__g"]}},{"id": "denovo6922", "metadata": {"taxonomy": ["Bacteria", "__Proteobacteria", "__Gammaproteobacteria", "__Chromatiales", "__Granulosicoccaceae", "__Granulosicoccus"]}}, {"id": "denovo6927", "metadata": {"taxonomy": ["Bacteria", "__Proteobacteria", "__Alphaproteobacteria", "__Rhizobiales", "__Hyphomicrobiaceae", "__Devosia"]}},{"id": "denovo6929", "metadata": {"taxonomy": ["Bacteria", "__Proteobacteria", "__Alphaproteobacteria", "__Rhodospirillales", "__Rhodospirillaceae", "__Rhodovibrio"]}},{"id": "denovo6930", "metadata": {"taxonomy": ["Bacteria", "__Proteobacteria", "__Gammaproteobacteria"]}},{"id": "denovo6931", "metadata": {"taxonomy": ["Bacteria", "__Proteobacteria", "__Deltaproteobacteria", "__Desulfobacterales", "__Desulfobulbaceae", "__Desulfobulbus"]}},{"id": "denovo6933", "metadata": {"taxonomy": ["Bacteria", "__Proteobacteria", "__Alphaproteobacteria", "__Rhodospirillales", "__Rhodospirillaceae"]}},{"id": "denovo6939", "metadata": {"taxonomy": ["Bacteria", "__Proteobacteria", "__Gammaproteobacteria", "__Alteromonadales", "__Colwelliaceae", "__Colwellia"]}},{"id": "denovo6944", "metadata": {"taxonomy": ["Bacteria", "__Proteobacteria", "__Alphaproteobacteria", "__Sphingomonadales", "__f", "__g"]}},{"id": "denovo6947", "metadata": {"taxonomy": ["Bacteria", "__Chloroflexi", "__Anaerolineae", "__Anaerolineales", "__Anaerolineaceae", "__g"]}},{"id": "denovo6950", "metadata": {"taxonomy": ["Unclassified"]}},{"id": "denovo6952", "metadata": {"taxonomy": ["Bacteria", "__Proteobacteria"]}},{"id": "denovo6953", "metadata": {"taxonomy": ["Bacteria", "__Proteobacteria", "__Gammaproteobacteria"]}},{"id": "denovo6959", "metadata": {"taxonomy": ["Bacteria", "__Proteobacteria", "__Alphaproteobacteria", "__Rhodobacterales", "__Rhodobacteraceae", "__Ruegeria"]}},{"id": "denovo6960", "metadata": {"taxonomy": ["Bacteria", "__Proteobacteria", "__Deltaproteobacteria", "__Desulfobacterales", "__Desulfobulbaceae", "__Desulfobulbus"]}}, {"id": "denovo6961", "metadata": {"taxonomy": ["Bacteria", "__Proteobacteria", "__Alphaproteobacteria", "__DB1-14", "__f", "__g"]}},{"id": "denovo6968", "metadata": {"taxonomy": ["Bacteria", "__Proteobacteria", "__Gammaproteobacteria", "__Pseudomonadales", "__Moraxellaceae", "__Acinetobacter"]}},{"id": "denovo6969", "metadata": {"taxonomy": ["Bacteria", "__Cyanobacteria", "__Cyanobacteria", "__SubsectionIII", "__FamilyI"]}},{"id": "denovo6974", "metadata": {"taxonomy": ["Bacteria", "__Firmicutes", "__Clostridia", "__Clostridiales", "__Clostridiaceae"]}},{"id": "denovo6980", "metadata": {"taxonomy": ["Bacteria", "__Bacteroidetes", "__Cytophagia", "__Cytophagales"]}},{"id": "denovo6981", "metadata": {"taxonomy": ["Bacteria", "__Proteobacteria", "__Deltaproteobacteria", "__Desulfobacterales", "__Nitrospinaceae", "__Candidatus_Entotheonella"]}},{"id": "denovo6983", "metadata": {"taxonomy": ["Bacteria", "__Cyanobacteria", "__Cyanobacteria",

"__SubsectionIII", "__FamilyI"]}},{"id": "denovo6986", "metadata": {"taxonomy": ["Bacteria", "__Cyanobacteria", "__Cyanobacteria", "__SubsectionI", "__FamilyI", "__g"]}},{"id": "denovo6989", "metadata": {"taxonomy": ["Bacteria", "__Proteobacteria", "__Alphaproteobacteria", "__Rhizobiales", "__Phyllobacteriaceae", "__Ahrensia"]}},{"id": "denovo6990", "metadata": {"taxonomy": ["Bacteria", "__Proteobacteria", "__Gammaproteobacteria", "__Alteromonadales", "__Colwelliaceae", "__Colwellia"]}},{"id": "denovo6995", "metadata": {"taxonomy": ["Bacteria", "__Proteobacteria", "__Gammaproteobacteria", "__Vibrionales", "__Vibrionaceae", "__Vibrio"]}},{"id": "denovo6996", "metadata": {"taxonomy": ["Bacteria", "__Proteobacteria", "__Gammaproteobacteria", "__Vibrionales", "__Vibrionaceae", "__Vibrio"]}},{"id": "denovo7005", "metadata": {"taxonomy": ["Bacteria", "__Proteobacteria", "__Alphaproteobacteria", "__Rhodospirillales", "__Rhodospirillaceae", "__Rhodovibrio"]}},{"id": "denovo7022", "metadata": {"taxonomy": ["Bacteria", "__Proteobacteria", "__Gammaproteobacteria", "__Xanthomonadales", "__Sinobacteraceae", "__JTB255_marine_benthic_group"]}},{"id": "denovo7033", "metadata": {"taxonomy": ["Bacteria", "__Bacteroidetes", "__Flavobacteria", "__Flavobacteriales", "__Flavobacteriaceae", "__Gilvibacter"]}},{"id": "denovo7039", "metadata": {"taxonomy": ["Bacteria", "__Proteobacteria", "__Alphaproteobacteria"]}},{"id": "denovo7042", "metadata": {"taxonomy": ["Bacteria", "__Proteobacteria", "__Alphaproteobacteria", "__Rhodobacterales", "__Rhodobacteraceae", "__Marinovum"]}},{"id": "denovo7045", "metadata": {"taxonomy": ["Bacteria", "__Proteobacteria", "__Deltaproteobacteria", "__Myxococcales", "__Nannocystineae", "__Nannocystaceae"]}},{"id": "denovo7054", "metadata": {"taxonomy": ["Bacteria", "__Bacteroidetes", "__Sphingobacteriia", "__Sphingobacteriales", "__Saprospiraceae", "__g"]}},{"id": "denovo7056", "metadata": {"taxonomy": ["Bacteria", "__Proteobacteria", "__Alphaproteobacteria", "__Rhodobacterales", "__Rhodobacteraceae", "__Roseobacter_clade_AS-21_lineage"]}},{"id": "denovo7057", "metadata": {"taxonomy": ["Bacteria", "__Proteobacteria", "__Alphaproteobacteria", "__Rhodospirillales", "__Rhodospirillaceae", "__Pelagibius"]}},{"id": "denovo7059", "metadata": {"taxonomy": ["Bacteria", "__Proteobacteria", "__Alphaproteobacteria", "__SAR11_clade", "__Surface_1", "__Candidatus_Pelagibacter"]}},{"id": "denovo7063", "metadata": {"taxonomy": ["Bacteria", "__Proteobacteria", "__Deltaproteobacteria", "__Sh765B-TzT-29", "__f", "__g"]}},{"id": "denovo7064", "metadata": {"taxonomy": ["Bacteria", "__Actinobacteria", "__Actinobacteria", "__Micrococcales", "__Micrococcaceae", "__Nesterenkonia"]}},{"id": "denovo7068", "metadata": {"taxonomy": ["Bacteria", "__Actinobacteria", "__Thermoleophilia", "__Gaiellales", "__uncultured", "__g"]}},{"id": "denovo7069", "metadata": {"taxonomy": ["Bacteria", "__Bacteroidetes", "__Flavobacteria", "__Flavobacteriales", "__Cryomorphaceae", "__Owenweeksia"]}},{"id": "denovo7070", "metadata": {"taxonomy": ["Bacteria", "__Proteobacteria", "__Deltaproteobacteria", "__Sh765B-TzT-29", "__f",

"__g"]}},{"id": "denovo7074", "metadata": {"taxonomy": ["Bacteria", "__Proteobacteria", "__Gammaproteobacteria", "__Alteromonadales", "__Alteromonadaceae", "__Aestuariibacter"]}},{"id": "denovo7075", "metadata": {"taxonomy": ["Bacteria", "__Proteobacteria", "__Gammaproteobacteria", "__BD7-8_marine_group", "__f", "__g"]}}, {"id": "denovo7082", "metadata": {"taxonomy": ["Bacteria", "__Proteobacteria", "__Alphaproteobacteria", "__DB1-14", "__f", "__g"]}},{"id": "denovo7083", "metadata": {"taxonomy": ["Bacteria", "__Acidobacteria", "__Acidobacteria", "__DA023", "__f", "__g"]}}, {"id": "denovo7085", "metadata": {"taxonomy": ["Bacteria", "__Actinobacteria", "__Acidimicrobiia", "__Acidimicrobiales", "__Sva0996_marine_group", "__g"]}},{"id": "denovo7087", "metadata": {"taxonomy": ["Bacteria", "__Proteobacteria", "__Gammaproteobacteria", "__Pseudomonadales", "__Pseudomonadaceae", "__Pseudomonas"]}},{"id": "denovo7088", "metadata": {"taxonomy": ["Bacteria", "__Planctomycetes", "__OM190", "__o", "__f", "__g"]}},{"id": "denovo7090", "metadata": {"taxonomy": ["Bacteria", "__Proteobacteria", "__Deltaproteobacteria", "__Myxococcales", "__Nannocystineae", "__Nannocystaceae"]}},{"id": "denovo7091", "metadata": {"taxonomy": ["Bacteria", "__Proteobacteria", "__Deltaproteobacteria", "__SAR324_clade(Marine_group_B)", "__f", "__g"]}},{"id": "denovo7092", "metadata": {"taxonomy": ["Bacteria", "__Cyanobacteria", "__Cyanobacteria", "__SubsectionIII", "__FamilyI", "__Phormidium"]}},{"id": "denovo7093", "metadata": {"taxonomy": ["Bacteria", "__Proteobacteria", "__Alphaproteobacteria", "__DB1-14", "__f", "__g"]}},{"id": "denovo7096", "metadata": {"taxonomy": ["Bacteria", "__Proteobacteria", "__Alphaproteobacteria", "__Rhodospirillales", "__Rhodospirillaceae", "__Defluviicoccus"]}}, {"id": "denovo7098", "metadata": {"taxonomy": ["Bacteria", "__Proteobacteria", "__Gammaproteobacteria", "__Oceanospirillales", "__Hahellaceae", "__Endozoicomonas"]}},{"id": "denovo7099", "metadata": {"taxonomy": ["Bacteria", "__Bacteroidetes", "__Cytophagia", "__Cytophagales", "__Flammeovirgaceae", "__g"]}}, {"id": "denovo7102", "metadata": {"taxonomy": ["Bacteria", "__Proteobacteria", "__Alphaproteobacteria", "__Rhizobiales", "__Rhodobiaceae", "__Rhodobium"]}},{"id": "denovo7103", "metadata": {"taxonomy": ["Bacteria", "__Proteobacteria", "__Gammaproteobacteria", "__Oceanospirillales", "__Oleiphilaceae", "__Oleiphilus"]}},{"id": "denovo7104", "metadata": {"taxonomy": ["Bacteria", "__Firmicutes", "__Bacilli"]}},{"id": "denovo7105", "metadata": {"taxonomy": ["Bacteria", "__Proteobacteria", "__Gammaproteobacteria", "__34P16", "__f", "__g"]}},{"id": "denovo7108", "metadata": {"taxonomy": ["Bacteria", "__Proteobacteria", "__Gammaproteobacteria", "__Chromatiales", "__Chromatiaceae", "__Nitrosococcus"]}},{"id": "denovo7115", "metadata": {"taxonomy": ["Bacteria", "__Bacteroidetes", "__Flavobacteria", "__Flavobacteriales", "__Flavobacteriaceae"]}}, {"id": "denovo7116", "metadata": {"taxonomy": ["Bacteria", "__Proteobacteria", "__Gammaproteobacteria", "__Alteromonadales", "__Alteromonadaceae", "__Haliea"]}},{"id": "denovo7118", "metadata": {"taxonomy": ["Bacteria", "__Bacteroidetes", "__Cytophagia",

"__Cytophagales", "__Flammeovirgaceae", "__g"]}},{"id": "denovo7122", "metadata": {"taxonomy": ["Bacteria", "__Proteobacteria", "__Gammaproteobacteria", "__Oceanospirillales", "__Hahellaceae", "__Endozoicomonas"]}},{"id": "denovo7128", "metadata": {"taxonomy": ["Bacteria", "__Acidobacteria", "__RB25", "__o", "__f", "__g"]}}, {"id": "denovo7131", "metadata": {"taxonomy": ["Bacteria", "__Cyanobacteria", "__Cyanobacteria", "__SubsectionIII", "__FamilyI"]}},{"id": "denovo7132", "metadata": {"taxonomy": ["Bacteria", "__Fusobacteria", "__Fusobacteria", "__Fusobacteriales", "__Fusobacteriaceae", "__Propionigenium"]}},{"id": "denovo7136", "metadata": {"taxonomy": ["Bacteria", "__Verrucomicrobia", "__Verrucomicrobiae", "__Verrucomicrobiales", "__Rubritaleaceae", "__Rubritalea"]}},{"id": "denovo7140", "metadata": {"taxonomy": ["Bacteria", "__Proteobacteria", "__Alphaproteobacteria", "__Rhizobiales"]}},{"id": "denovo7142", "metadata": {"taxonomy": ["Bacteria", "__Proteobacteria", "__Deltaproteobacteria", "__Bdellovibrionales", "__Bacteriovoracaceae", "__Bacteriovorax"]}}, {"id": "denovo7145", "metadata": {"taxonomy": ["Bacteria", "__Proteobacteria", "__Alphaproteobacteria", "__Caulobacterales", "__Hyphomonadaceae", "__Robiginitomaculum"]}},{"id": "denovo7146", "metadata": {"taxonomy": ["Bacteria", "__Bacteroidetes", "__Flavobacteria", "__Flavobacteriales", "__Flavobacteriaceae", "__Maritimimonas"]}},{"id": "denovo7148", "metadata": {"taxonomy": ["Bacteria", "__Proteobacteria", "__Alphaproteobacteria", "__Rickettsiales", "__Rickettsiaceae", "__g"]}},{"id": "denovo7149", "metadata": {"taxonomy": ["Bacteria", "__Chlorobi", "__Chlorobia", "__Chlorobiales", "__Chlorobiaceae", "__Prosthecochloris"]}},{"id": "denovo7150", "metadata": {"taxonomy": ["Bacteria", "__Proteobacteria", "__Gammaproteobacteria", "__Alteromonadales", "__Alteromonadaceae", "__Haliea"]}},{"id": "denovo7151", "metadata": {"taxonomy": ["Bacteria", "__Elusimicrobia", "__Elusimicrobia", "__Lineage_IIb", "__f", "__g"]}},{"id": "denovo7153", "metadata": {"taxonomy": ["Bacteria", "__Bacteroidetes", "__Sphingobacteriia", "__Sphingobacteriales", "__Saprospiraceae", "__g"]}},{"id": "denovo7157", "metadata": {"taxonomy": ["Bacteria", "__Cyanobacteria", "__SHA-109", "__o", "__f", "__g"]}},{"id": "denovo7159", "metadata": {"taxonomy": ["Bacteria", "__Firmicutes", "__Clostridia", "__Halanaerobiales", "__64K2", "__g"]}},{"id": "denovo7162", "metadata": {"taxonomy": ["Bacteria", "__Proteobacteria", "__Betaproteobacteria", "__Burkholderiales", "__Alcaligenaceae", "__MWH-UniP1_aquatic_group"]}},{"id": "denovo7163", "metadata": {"taxonomy": ["Bacteria", "__Proteobacteria", "__Alphaproteobacteria", "__Rhizobiales", "__Phyllobacteriaceae"]}},{"id": "denovo7164", "metadata": {"taxonomy": ["Bacteria", "__Proteobacteria", "__Alphaproteobacteria", "__Rhodospirillales", "__Rhodospirillaceae", "__AEGEAN-169_marine_group"]}},{"id": "denovo7168", "metadata": {"taxonomy": ["Bacteria", "__Chloroflexi", "__SAR202_clade", "__o", "__f", "__g"]}},{"id": "denovo7172", "metadata": {"taxonomy": ["Bacteria", "__Proteobacteria", "__Deltaproteobacteria", "__Sh765B-TzT-29", "__f", "__g"]}},{"id": "denovo7177", "metadata": {"taxonomy":

["Bacteria", "__Proteobacteria", "__Alphaproteobacteria", "__Rhodospirillales", "__Rhodospirillaceae", "__Thalassospira"]}}, {"id": "denovo7178", "metadata": {"taxonomy": ["Bacteria", "__Gemmatimonadetes", "__Gemmatimonadetes", "__BD2-11_terrestrial_group", "__f", "__g"]}},{"id": "denovo7179", "metadata": {"taxonomy": ["Bacteria", "__Proteobacteria", "__Alphaproteobacteria"]}},{"id": "denovo7181", "metadata": {"taxonomy": ["Bacteria", "__Proteobacteria", "__Deltaproteobacteria", "__Sh765B-TzT-29", "__f", "__g"]}},{"id": "denovo7186", "metadata": {"taxonomy": ["Bacteria", "__Acidobacteria", "__Holophagae", "__CA002", "__f", "__g"]}},{"id": "denovo7187", "metadata": {"taxonomy": ["Bacteria", "__Firmicutes", "__Clostridia", "__Halanaerobiales", "__Halobacteroidaceae", "__g"]}},{"id": "denovo7188", "metadata": {"taxonomy": ["Bacteria", "__Proteobacteria", "__Gammaproteobacteria", "__BD3-1", "__f", "__g"]}},{"id": "denovo7189", "metadata": {"taxonomy": ["Archaea", "__Thaumarchaeota", "__Marine_Group_I", "__Candidatus_Nitrosopumilus", "__f", "__g"]}},{"id": "denovo7190", "metadata": {"taxonomy": ["Bacteria", "__Proteobacteria", "__Gammaproteobacteria", "__Alteromonadales", "__Pseudoalteromonadaceae", "__Pseudoalteromonas"]}},{"id": "denovo7191", "metadata": {"taxonomy": ["Bacteria", "__Proteobacteria", "__Deltaproteobacteria", "__Sh765B-TzT-29", "__f", "__g"]}},{"id": "denovo7193", "metadata": {"taxonomy": ["Bacteria", "__Acidobacteria", "__Acidobacteria", "__AT-s3-28", "__f", "__g"]}},{"id": "denovo7197", "metadata": {"taxonomy": ["Bacteria", "__Proteobacteria", "__Alphaproteobacteria", "__Rhodospirillales", "__MSB-1E8", "__g"]}},{"id": "denovo7202", "metadata": {"taxonomy": ["Bacteria", "__Actinobacteria", "__Acidimicrobiia", "__Acidimicrobiales", "__Sva0996_marine_group", "__g"]}},{"id": "denovo7206", "metadata": {"taxonomy": ["Bacteria", "__Actinobacteria", "__Acidimicrobiia", "__Acidimicrobiales", "__Acidimicrobiaceae", "__g"]}},{"id": "denovo7207", "metadata": {"taxonomy": ["Bacteria", "__Proteobacteria", "__Alphaproteobacteria", "__Caulobacterales", "__Caulobacteraceae"]}},{"id": "denovo7209", "metadata": {"taxonomy": ["Bacteria", "__Proteobacteria", "__Gammaproteobacteria", "__HOC36", "__f", "__g"]}},{"id": "denovo7216", "metadata": {"taxonomy": ["Bacteria", "__Proteobacteria", "__Gammaproteobacteria"]}},{"id": "denovo7223", "metadata": {"taxonomy": ["Bacteria", "__Proteobacteria", "__Deltaproteobacteria", "__Desulfovibrionales", "__Desulfovibrionaceae", "__Desulfovibrio"]}},{"id": "denovo7227", "metadata": {"taxonomy": ["Bacteria", "__Proteobacteria", "__Gammaproteobacteria", "__Oceanospirillales", "__Oceanospirillaceae"]}},{"id": "denovo7228", "metadata": {"taxonomy": ["Bacteria", "__TM6", "__c", "__o", "__f", "__g"]}}, {"id": "denovo7231", "metadata": {"taxonomy": ["Bacteria", "__Proteobacteria", "__Gammaproteobacteria"]}},{"id": "denovo7234", "metadata": {"taxonomy": ["Bacteria", "__Bacteroidetes", "__Cytophagia", "__Cytophagales", "__Flammeovirgaceae"]}},{"id": "denovo7246", "metadata": {"taxonomy": ["Bacteria",

"__Proteobacteria", "__Alphaproteobacteria", "__Rhodobacterales", "__Rhodobacteraceae", "__Roseobacter_clade_AS-21_lineage"]}},{"id": "denovo7250", "metadata": {"taxonomy": ["Bacteria", "__Bacteroidetes", "__Flavobacteria", "__Flavobacteriales", "__Flavobacteriaceae", "__Croceitalea"]}},{"id": "denovo7256", "metadata": {"taxonomy": ["Bacteria", "__Proteobacteria", "__Alphaproteobacteria", "__Parvularculales", "__Parvularculaceae", "__Parvularcula"]}},{"id": "denovo7260", "metadata": {"taxonomy": ["Bacteria", "__Bacteroidetes", "__Flavobacteria", "__Flavobacteriales", "__Flavobacteriaceae", "__NS5_marine_group"]}},{"id": "denovo7262", "metadata": {"taxonomy": ["Bacteria", "__Proteobacteria", "__Deltaproteobacteria", "__Desulfuromonadales", "__Desulfuromonadaceae"]}},{"id": "denovo7266", "metadata": {"taxonomy": ["Bacteria", "__Proteobacteria", "__Alphaproteobacteria", "__Rhodobacterales", "__Rhodobacteraceae"]}},{"id": "denovo7269", "metadata": {"taxonomy": ["Bacteria", "__Proteobacteria", "__Gammaproteobacteria", "__Alteromonadales", "__Alteromonadaceae", "__Haliea"]}},{"id": "denovo7270", "metadata": {"taxonomy": ["Bacteria", "__Proteobacteria", "__Gammaproteobacteria", "__Alteromonadales", "__Alteromonadaceae", "__C1-B045"]}},{"id": "denovo7272", "metadata": {"taxonomy": ["Bacteria", "__Bacteroidetes", "__Flavobacteria", "__Flavobacteriales", "__Cryomorphaceae", "__Owenweeksia"]}},{"id": "denovo7276", "metadata": {"taxonomy": ["Bacteria", "__TM6", "__c", "__o", "__f", "__g"]}},{"id": "denovo7280", "metadata": {"taxonomy": ["Bacteria", "__Proteobacteria", "__Gammaproteobacteria", "__Alteromonadales", "__Alteromonadaceae", "__BD1-7_clade"]}},{"id": "denovo7287", "metadata": {"taxonomy": ["Bacteria", "__Proteobacteria", "__Alphaproteobacteria"]}},{"id": "denovo7289", "metadata": {"taxonomy": ["Bacteria", "__Verrucomicrobia", "__Opitutae", "__Puniceicoccales", "__Puniceicoccaceae", "__Pelagicoccus"]}},{"id": "denovo7293", "metadata": {"taxonomy": ["Bacteria", "__Proteobacteria", "__Alphaproteobacteria", "__Rickettsiales", "__TK34", "__g"]}},{"id": "denovo7295", "metadata": {"taxonomy": ["Bacteria", "__Proteobacteria", "__Alphaproteobacteria", "__Rhodobacterales", "__Rhodobacteraceae"]}},{"id": "denovo7298", "metadata": {"taxonomy": ["Bacteria", "__Proteobacteria", "__Deltaproteobacteria", "__Desulfobacterales", "__Desulfobacteraceae", "__Desulfococcus"]}},{"id": "denovo7299", "metadata": {"taxonomy": ["Bacteria", "__Proteobacteria", "__Alphaproteobacteria", "__Rhodobacterales", "__Rhodobacteraceae"]}},{"id": "denovo7302", "metadata": {"taxonomy": ["Bacteria", "__Proteobacteria", "__Gammaproteobacteria", "__Legionellales", "__Coxiellaceae", "__Coxiella"]}},{"id": "denovo7304", "metadata": {"taxonomy": ["Bacteria", "__Proteobacteria", "__Deltaproteobacteria", "__Desulfobacterales", "__Desulfobacteraceae", "__g"]}},{"id": "denovo7305", "metadata": {"taxonomy": ["Bacteria", "__Acidobacteria", "__Acidobacteria", "__Candidatus_Chloracidobacterium", "__f", "__g"]}},{"id": "denovo7309", "metadata": {"taxonomy": ["Bacteria", "__Proteobacteria", "__Deltaproteobacteria", "__GR-WP33-30", "__f", "__g"]}},{"id": "denovo7310", "metadata": {"taxonomy": ["Bacteria",

"__Proteobacteria", "__Gammaproteobacteria"]}},{"id": "denovo7312", "metadata": {"taxonomy": ["Bacteria", "__Proteobacteria", "__Gammaproteobacteria", "__BD7-8_marine_group", "__f", "__g"]}}, {"id": "denovo7315", "metadata": {"taxonomy": ["Bacteria", "__Proteobacteria", "__Gammaproteobacteria", "__KI89A_clade", "__f", "__g"]}},{"id": "denovo7317", "metadata": {"taxonomy": ["Bacteria", "__Verrucomicrobia", "__Verrucomicrobiae", "__Verrucomicrobiales", "__DEV007", "__g"]}},{"id": "denovo7318", "metadata": {"taxonomy": ["Bacteria", "__Bacteroidetes", "__Flavobacteria", "__Flavobacteriales", "__Flavobacteriaceae", "__g"]}},{"id": "denovo7320", "metadata": {"taxonomy": ["Bacteria", "__Proteobacteria", "__Alphaproteobacteria", "__Rhodospirillales", "__Rhodospirillaceae"]}},{"id": "denovo7321", "metadata": {"taxonomy": ["Bacteria", "__Proteobacteria", "__Gammaproteobacteria", "__HOC36", "__f", "__g"]}},{"id": "denovo7322", "metadata": {"taxonomy": ["Bacteria", "__Proteobacteria", "__Gammaproteobacteria", "__Pseudomonadales", "__Moraxellaceae", "__Acinetobacter"]}},{"id": "denovo7331", "metadata": {"taxonomy": ["Bacteria", "__Proteobacteria", "__Gammaproteobacteria", "__Xanthomonadales", "__Sinobacteraceae", "__JTB255_marine_benthic_group"]}},{"id": "denovo7334", "metadata": {"taxonomy": ["Bacteria", "__Proteobacteria", "__Gammaproteobacteria", "__Order_Incertae_Sedis", "__Family_Incertae_Sedis", "__Marinicella"]}},{"id": "denovo7335", "metadata": {"taxonomy": ["Bacteria", "__Proteobacteria", "__Gammaproteobacteria", "__Oceanospirillales", "__Hahellaceae", "__Endozoicomonas"]}},{"id": "denovo7339", "metadata": {"taxonomy": ["Bacteria", "__Bacteroidetes", "__Sphingobacteriia", "__Sphingobacteriales", "__Saprospiraceae", "__g"]}},{"id": "denovo7342", "metadata": {"taxonomy": ["Bacteria", "__Acidobacteria", "__Holophagae", "__CA002", "__f", "__g"]}},{"id": "denovo7345", "metadata": {"taxonomy": ["Bacteria", "__Proteobacteria", "__Deltaproteobacteria", "__Myxococcales", "__Cystobacterineae", "__g"]}},{"id": "denovo7348", "metadata": {"taxonomy": ["Bacteria", "__Proteobacteria", "__Deltaproteobacteria", "__Myxococcales", "__0319-6G20", "__g"]}},{"id": "denovo7351", "metadata": {"taxonomy": ["Bacteria", "__Proteobacteria", "__Alphaproteobacteria", "__Rhodobacterales", "__Rhodobacteraceae"]}},{"id": "denovo7353", "metadata": {"taxonomy": ["Bacteria", "__Acidobacteria", "__Holophagae", "__TK85", "__f", "__g"]}},{"id": "denovo7355", "metadata": {"taxonomy": ["Bacteria", "__Firmicutes", "__Clostridia", "__Clostridiales", "__Lachnospiraceae"]}},{"id": "denovo7357", "metadata": {"taxonomy": ["Bacteria", "__Cyanobacteria", "__Cyanobacteria", "__SubsectionIII", "__FamilyI"]}},{"id": "denovo7358", "metadata": {"taxonomy": ["Bacteria", "__Proteobacteria", "__Deltaproteobacteria", "__Desulfobacterales", "__Desulfobulbaceae"]}},{"id": "denovo7359", "metadata": {"taxonomy": ["Bacteria", "__Proteobacteria", "__Deltaproteobacteria", "__SAR324_clade(Marine_group_B)", "__f", "__g"]}},{"id": "denovo7361", "metadata": {"taxonomy": ["Bacteria", "__Proteobacteria", "__Alphaproteobacteria", "__Rhodospirillales", "__Rhodospirillaceae",

"__Defluviicoccus"]}},{"id": "denovo7363", "metadata": {"taxonomy": ["Bacteria", "__Proteobacteria", "__Candidatus_Thiobios", "__o", "__f", "__g"]}},{"id": "denovo7366", "metadata": {"taxonomy": ["Bacteria", "__Gemmatimonadetes", "__Gemmatimonadetes", "__BD2-11_terrestrial_group", "__f", "__g"]}},{"id": "denovo7370", "metadata": {"taxonomy": ["Bacteria", "__Proteobacteria", "__Gammaproteobacteria", "__Enterobacteriales", "__Enterobacteriaceae"]}},{"id": "denovo7371", "metadata": {"taxonomy": ["Bacteria", "__Proteobacteria", "__Alphaproteobacteria", "__Caulobacterales", "__Caulobacteraceae", "__Brevundimonas"]}},{"id": "denovo7372", "metadata": {"taxonomy": ["Archaea", "__Thaumarchaeota", "__Marine_Group_I", "__Candidatus_Nitrosopumilus", "__f", "__g"]}},{"id": "denovo7378", "metadata": {"taxonomy": ["Bacteria", "__Proteobacteria", "__Alphaproteobacteria", "__Rhodobacterales", "__Rhodobacteraceae"]}},{"id": "denovo7381", "metadata": {"taxonomy": ["Bacteria", "__Planctomycetes", "__Planctomycetacia", "__Planctomycetales", "__Planctomycetaceae", "__Planctomyces"]}},{"id": "denovo7385", "metadata": {"taxonomy": ["Bacteria", "__Proteobacteria", "__Gammaproteobacteria", "__Xanthomonadales", "__Sinobacteraceae", "__JTB255_marine_benthic_group"]}},{"id": "denovo7390", "metadata": {"taxonomy": ["Bacteria", "__Acidobacteria", "__Acidobacteria", "__Candidatus_Chloracidobacterium", "__f", "__g"]}},{"id": "denovo7391", "metadata": {"taxonomy": ["Bacteria", "__Proteobacteria", "__Deltaproteobacteria", "__Myxococcales", "__Nannocystaceae", "__g"]}},{"id": "denovo7399", "metadata": {"taxonomy": ["Archaea", "__Euryarchaeota", "__Halobacteria", "__Halobacteriales", "__Deep_Sea_Hydrothermal_Vent_Gp_6(DHVEG-6)", "__g"]}},{"id": "denovo7400", "metadata": {"taxonomy": ["Bacteria", "__Acidobacteria", "__RB25", "__o", "__f", "__g"]}},{"id": "denovo7402", "metadata": {"taxonomy": ["Bacteria", "__Bacteroidetes", "__Sphingobacteriia", "__Sphingobacteriales", "__Saprospiraceae", "__g"]}},{"id": "denovo7408", "metadata": {"taxonomy": ["Bacteria", "__Proteobacteria", "__Deltaproteobacteria", "__Sh765B-TzT-29", "__f", "__g"]}},{"id": "denovo7410", "metadata": {"taxonomy": ["Bacteria", "__Proteobacteria", "__Alphaproteobacteria", "__OCS116_clade", "__f", "__g"]}},{"id": "denovo7413", "metadata": {"taxonomy": ["Bacteria", "__Firmicutes", "__Clostridia", "__Clostridiales", "__Clostridiaceae", "__Clostridium"]}},{"id": "denovo7414", "metadata": {"taxonomy": ["Bacteria", "__Proteobacteria", "__Gammaproteobacteria", "__Order_Incertae_Sedis", "__Family_Incertae_Sedis", "__Arenicella"]}},{"id": "denovo7422", "metadata": {"taxonomy": ["Bacteria", "__TM6", "__c", "__o", "__f", "__g"]}},{"id": "denovo7424", "metadata": {"taxonomy": ["Bacteria", "__Proteobacteria", "__Deltaproteobacteria", "__Desulfobacterales", "__Desulfobacteraceae", "__Desulfosalsimonas"]}},{"id": "denovo7425", "metadata": {"taxonomy": ["Bacteria", "__Proteobacteria", "__Alphaproteobacteria", "__Rhodospirillales", "__Rhodospirillaceae"]}},{"id": "denovo7426", "metadata": {"taxonomy": ["Bacteria", "__Chloroflexi", "__SAR202_clade", "__o", "__f", "__g"]}},{"id": "denovo7427", "metadata": {"taxonomy": ["Bacteria",

"__Firmicutes", "__Clostridia", "__Clostridiales", "__Clostridiaceae", "__Anaerovirgula"]}},{"id": "denovo7433", "metadata": {"taxonomy": ["Bacteria", "__Proteobacteria", "__Alphaproteobacteria", "__Rhizobiales", "__Phyllobacteriaceae", "__Aquamicrobium"]}},{"id": "denovo7435", "metadata": {"taxonomy": ["Bacteria", "__Proteobacteria", "__Alphaproteobacteria", "__Rhodobacterales", "__Rhodobacteraceae"]}},{"id": "denovo7436", "metadata": {"taxonomy": ["Bacteria", "__Proteobacteria", "__Gammaproteobacteria", "__Order_Incertae_Sedis", "__Family_Incertae_Sedis", "__Marinicella"]}},{"id": "denovo7438", "metadata": {"taxonomy": ["Bacteria", "__Candidate_division_OD1", "__c", "__o", "__f", "__g"]}},{"id": "denovo7440", "metadata": {"taxonomy": ["Bacteria", "__Proteobacteria", "__Deltaproteobacteria", "__Myxococcales", "__Haliangiaceae", "__Haliangium"]}},{"id": "denovo7442", "metadata": {"taxonomy": ["Bacteria", "__Proteobacteria", "__Deltaproteobacteria", "__Sh765B-TzT-29", "__f", "__g"]}},{"id": "denovo7443", "metadata": {"taxonomy": ["Bacteria", "__Proteobacteria", "__Alphaproteobacteria", "__Sphingomonadales", "__Erythrobacteraceae", "__g"]}},{"id": "denovo7444", "metadata": {"taxonomy": ["Bacteria", "__Proteobacteria", "__Gammaproteobacteria", "__Chromatiales", "__Granulosicoccaceae", "__Granulosicoccus"]}},{"id": "denovo7446", "metadata": {"taxonomy": ["Bacteria", "__Bacteroidetes", "__Sphingobacteriia", "__Sphingobacteriales", "__PHOS-HE51", "__g"]}},{"id": "denovo7452", "metadata": {"taxonomy": ["Bacteria", "__Gemmatimonadetes", "__Gemmatimonadetes", "__BD2-11_terrestrial_group", "__f", "__g"]}},{"id": "denovo7458", "metadata": {"taxonomy": ["Bacteria", "__Candidate_division_TM7", "__c", "__o", "__f", "__g"]}},{"id": "denovo7460", "metadata": {"taxonomy": ["Bacteria", "__Proteobacteria", "__Gammaproteobacteria", "__Pseudomonadales", "__Moraxellaceae", "__Acinetobacter"]}},{"id": "denovo7465", "metadata": {"taxonomy": ["Bacteria", "__Proteobacteria", "__Alphaproteobacteria", "__OCS116_clade", "__f", "__g"]}},{"id": "denovo7466", "metadata": {"taxonomy": ["Bacteria", "__Cyanobacteria", "__Cyanobacteria", "__SubsectionI", "__FamilyI", "__Synechococcus"]}},{"id": "denovo7469", "metadata": {"taxonomy": ["Bacteria", "__Proteobacteria", "__Deltaproteobacteria", "__Bdellovibrionales", "__Bdellovibrionaceae", "__Bdellovibrio"]}},{"id": "denovo7470", "metadata": {"taxonomy": ["Bacteria", "__Proteobacteria", "__Alphaproteobacteria", "__Rhodospirillales", "__Rhodospirillaceae", "__Defluviicoccus"]}},{"id": "denovo7476", "metadata": {"taxonomy": ["Bacteria", "__Actinobacteria", "__Actinobacteria", "__Propionibacteriales", "__Propionibacteriaceae", "__Jiangella"]}},{"id": "denovo7477", "metadata": {"taxonomy": ["Bacteria", "__Candidate_division_BRC1", "__c", "__o", "__f", "__g"]}},{"id": "denovo7485", "metadata": {"taxonomy": ["Bacteria", "__Acidobacteria", "__Holophagae", "__CA002", "__f", "__g"]}},{"id": "denovo7486", "metadata": {"taxonomy": ["Bacteria", "__Bacteroidetes", "__Cytophagia", "__Order_II_Incertae_Sedis", "__Rhodothermaceae", "__g"]}},{"id": "denovo7488", "metadata": {"taxonomy": ["Bacteria", "__Proteobacteria", "__Gammaproteobacteria", "__BD7-8_marine_group",

"__f", "__g"]}},{"id": "denovo7490", "metadata": {"taxonomy": ["Bacteria", "__Proteobacteria", "__Alphaproteobacteria", "__DB1-14", "__f", "__g"]}},{"id": "denovo7499", "metadata": {"taxonomy": ["Bacteria", "__Bacteroidetes", "__Cytophagia", "__Cytophagales", "__Cytophagaceae"]}},{"id": "denovo7508", "metadata": {"taxonomy": ["Bacteria", "__Proteobacteria", "__Gammaproteobacteria", "__Alteromonadales", "__Alteromonadaceae", "__Candidatus_Endobugula"]}},{"id": "denovo7509", "metadata": {"taxonomy": ["Bacteria", "__Chloroflexi", "__Caldilineae", "__Caldilineales", "__Caldilineaceae", "__g"]}},{"id": "denovo7510", "metadata": {"taxonomy": ["Bacteria", "__Lentisphaerae", "__Lentisphaeria"]}},{"id": "denovo7514", "metadata": {"taxonomy": ["Bacteria", "__Chlamydiae", "__Chlamydiae", "__Chlamydiales"]}},{"id": "denovo7517", "metadata": {"taxonomy": ["Bacteria", "__Proteobacteria", "__Alphaproteobacteria", "__Rhizobiales", "__Rhodobiaceae", "__Rhodobium"]}},{"id": "denovo7522", "metadata": {"taxonomy": ["Bacteria", "__Proteobacteria", "__Alphaproteobacteria", "__Rhodobacterales", "__Rhodobacteraceae", "__Roseobacter"]}},{"id": "denovo7524", "metadata": {"taxonomy": ["Bacteria", "__Proteobacteria", "__Alphaproteobacteria", "__Rhizobiales", "__Brucellaceae", "__g"]}},{"id": "denovo7525", "metadata": {"taxonomy": ["Bacteria", "__Proteobacteria", "__Gammaproteobacteria", "__Legionellales", "__Coxiellaceae", "__Coxiella"]}},{"id": "denovo7529", "metadata": {"taxonomy": ["Bacteria", "__Nitrospirae", "__Nitrospira", "__Nitrospirales", "__Nitrospiraceae", "__Nitrospira"]}},{"id": "denovo7530", "metadata": {"taxonomy": ["Bacteria", "__Proteobacteria", "__Alphaproteobacteria", "__Rhodobacterales", "__Rhodobacteraceae"]}},{"id": "denovo7535", "metadata": {"taxonomy": ["Bacteria", "__Proteobacteria", "__Gammaproteobacteria", "__Vibrionales", "__Vibrionaceae"]}},{"id": "denovo7536", "metadata": {"taxonomy": ["Bacteria", "__Proteobacteria", "__Gammaproteobacteria", "__Chromatiales"]}},{"id": "denovo7537", "metadata": {"taxonomy": ["Bacteria", "__Proteobacteria", "__Gammaproteobacteria", "__Oceanospirillales", "__Hahellaceae", "__Endozoicomonas"]}},{"id": "denovo7538", "metadata": {"taxonomy": ["Bacteria", "__Proteobacteria", "__Alphaproteobacteria", "__Rhizobiales", "__JG34-KF-361", "__g"]}},{"id": "denovo7546", "metadata": {"taxonomy": ["Bacteria", "__Firmicutes", "__Clostridia", "__Clostridiales", "__Ruminococcaceae"]}},{"id": "denovo7548", "metadata": {"taxonomy": ["Bacteria", "__Proteobacteria", "__Gammaproteobacteria", "__Order_Incertae_Sedis", "__Family_Incertae_Sedis", "__Marinicella"]}},{"id": "denovo7553", "metadata": {"taxonomy": ["Bacteria", "__Proteobacteria", "__Alphaproteobacteria", "__OCS116_clade", "__f", "__g"]}},{"id": "denovo7554", "metadata": {"taxonomy": ["Archaea", "__Euryarchaeota", "__Halobacteria", "__Halobacteriales", "__Deep_Sea_Hydrothermal_Vent_Gp_6(DHVEG-6)", "__Candidatus_Parvarchaeum"]}},{"id": "denovo7555", "metadata": {"taxonomy": ["Bacteria", "__Proteobacteria", "__Alphaproteobacteria", "__Rickettsiales", "__SAR116_clade", "__g"]}},{"id": "denovo7557",

"metadata": {"taxonomy": ["Bacteria", "__Proteobacteria",
"__Gammaproteobacteria", "__Order_Incertae_Sedis",
"__Family_Incertae_Sedis", "__Sedimenticola"]}},{"id": "denovo7559",
"metadata": {"taxonomy": ["Bacteria", "__Bacteroidetes", "__BD2-2",
"__o", "__f", "__g"]}},{"id": "denovo7564", "metadata": {"taxonomy":
["Bacteria", "__Bacteroidetes", "__Flavobacteria",
"__Flavobacteriales", "__Flavobacteriaceae", "__Muricauda"]}},{"id":
"denovo7567", "metadata": {"taxonomy": ["Bacteria",
"__Proteobacteria", "__Deltaproteobacteria", "__GR-WP33-30", "__f",
"__g"]}},{"id": "denovo7568", "metadata": {"taxonomy": ["Bacteria",
"__Proteobacteria", "__Gammaproteobacteria", "__Oceanospirillales",
"__Alcanivoracaceae", "__Kangiella"]}},{"id": "denovo7569",
"metadata": {"taxonomy": ["Bacteria", "__Proteobacteria",
"__Alphaproteobacteria", "__Rhodobacterales", "__Rhodobacteraceae",
"__Ruegeria"]}},{"id": "denovo7571", "metadata": {"taxonomy":
["Bacteria", "__Elusimicrobia", "__Elusimicrobia", "__Lineage_IIb",
"__f", "__g"]}},{"id": "denovo7572", "metadata": {"taxonomy":
["Bacteria", "__Cyanobacteria", "__Cyanobacteria", "__SubsectionIII",
"__FamilyI", "__Lyngbya"]}},{"id": "denovo7574", "metadata":
{"taxonomy": ["Archaea", "__Thaumarchaeota", "__Marine_Group_I",
"__Candidatus_Nitrosopumilus", "__f", "__g"]}},{"id": "denovo7578",
"metadata": {"taxonomy": ["Bacteria", "__Proteobacteria",
"__Deltaproteobacteria", "__Myxococcales", "__Nannocystaceae",
"__g"]}},{"id": "denovo7579", "metadata": {"taxonomy": ["Bacteria"]}},
{"id": "denovo7583", "metadata": {"taxonomy": ["Bacteria",
"__Acidobacteria", "__Acidobacteria", "__BPC102", "__f", "__g"]}},
{"id": "denovo7584", "metadata": {"taxonomy": ["Bacteria",
"__Chloroflexi", "__Caldilineae", "__Caldilineales",
"__Caldilineaceae", "__Caldilinea"]}},{"id": "denovo7585", "metadata":
{"taxonomy": ["Bacteria", "__Proteobacteria", "__Deltaproteobacteria",
"__Desulfovibrionales", "__Desulfovibrionaceae", "__Desulfovibrio"]}},
{"id": "denovo7586", "metadata": {"taxonomy": ["Bacteria",
"__Proteobacteria", "__Gammaproteobacteria", "__NKB5", "__f",
"__g"]}},{"id": "denovo7589", "metadata": {"taxonomy": ["Bacteria",
"__Planctomycetes", "__Phycisphaerae", "__Phycisphaerales",
"__Phycisphaeraceae", "__Phycisphaera"]}},{"id": "denovo7593",
"metadata": {"taxonomy": ["Bacteria", "__Proteobacteria",
"__Deltaproteobacteria", "__Sh765B-TzT-29", "__f", "__g"]}},{"id":
"denovo7594", "metadata": {"taxonomy": ["Bacteria",
"__Proteobacteria", "__Alphaproteobacteria", "__Sphingomonadales",
"__MN_122.2a", "__g"]}},{"id": "denovo7597", "metadata": {"taxonomy":
["Bacteria", "__Proteobacteria", "__Deltaproteobacteria",
"__Desulfobacterales", "__Desulfobulbaceae"]}},{"id": "denovo7598",
"metadata": {"taxonomy": ["Bacteria", "__Proteobacteria",
"__Gammaproteobacteria", "__Oceanospirillales"]}},{"id": "denovo7604",
"metadata": {"taxonomy": ["Bacteria", "__Chlorobi", "__Chlorobia",
"__Chlorobiales", "__Chlorobiaceae", "__Prosthecochloris"]}},{"id":
"denovo7605", "metadata": {"taxonomy": ["Bacteria",
"__Proteobacteria", "__SC3-20", "__o", "__f", "__g"]}},{"id":
"denovo7608", "metadata": {"taxonomy": ["Bacteria",

"__Proteobacteria", "__Gammaproteobacteria", "__Thiotrichales", "__Thiotrichaceae", "__Thiothrix"]}},{"id": "denovo7609", "metadata": {"taxonomy": ["Bacteria", "__Chlorobi", "__Chlorobia", "__Chlorobiales", "__Chlorobiaceae", "__Prosthecochloris"]}},{"id": "denovo7614", "metadata": {"taxonomy": ["Bacteria", "__Proteobacteria", "__Gammaproteobacteria", "__Xanthomonadales", "__Sinobacteraceae", "__JTB255_marine_benthic_group"]}},{"id": "denovo7618", "metadata": {"taxonomy": ["Bacteria", "__Acidobacteria", "__Holophagae", "__Sva0725", "__f", "__g"]}},{"id": "denovo7619", "metadata": {"taxonomy": ["Bacteria", "__Bacteroidetes", "__Cytophagia", "__Cytophagales", "__Flammeovirgaceae", "__Persicobacter"]}},{"id": "denovo7626", "metadata": {"taxonomy": ["Bacteria", "__Acidobacteria", "__Holophagae", "__CA002", "__f", "__g"]}},{"id": "denovo7628", "metadata": {"taxonomy": ["Bacteria", "__Proteobacteria", "__Gammaproteobacteria", "__Oceanospirillales", "__OM182_clade", "__g"]}},{"id": "denovo7629", "metadata": {"taxonomy": ["Bacteria", "__Proteobacteria", "__Gammaproteobacteria", "__Alteromonadales", "__Alteromonadaceae"]}},{"id": "denovo7630", "metadata": {"taxonomy": ["Bacteria", "__Proteobacteria", "__Alphaproteobacteria", "__DB1-14", "__f", "__g"]}},{"id": "denovo7631", "metadata": {"taxonomy": ["Bacteria", "__Proteobacteria", "__Alphaproteobacteria", "__Rickettsiales", "__Ho(lab)", "__g"]}},{"id": "denovo7633", "metadata": {"taxonomy": ["Bacteria", "__Proteobacteria", "__Deltaproteobacteria", "__Sh765B-TzT-29", "__f", "__g"]}},{"id": "denovo7634", "metadata": {"taxonomy": ["Bacteria", "__Proteobacteria", "__Gammaproteobacteria", "__Alteromonadales", "__Alteromonadaceae", "__BD1-7_clade"]}},{"id": "denovo7639", "metadata": {"taxonomy": ["Bacteria", "__Proteobacteria", "__Gammaproteobacteria", "__Vibrionales", "__Vibrionaceae", "__Photobacterium"]}},{"id": "denovo7640", "metadata": {"taxonomy": ["Bacteria", "__Proteobacteria", "__Gammaproteobacteria", "__Pseudomonadales", "__Moraxellaceae", "__Acinetobacter"]}},{"id": "denovo7641", "metadata": {"taxonomy": ["Bacteria", "__Proteobacteria", "__Deltaproteobacteria", "__Sh765B-TzT-29", "__f", "__g"]}},{"id": "denovo7652", "metadata": {"taxonomy": ["Bacteria", "__Cyanobacteria", "__Cyanobacteria", "__SubsectionI", "__FamilyI", "__Synechococcus"]}},{"id": "denovo7653", "metadata": {"taxonomy": ["Bacteria", "__Proteobacteria", "__Deltaproteobacteria", "__Myxococcales", "__Nannocystineae", "__g"]}},{"id": "denovo7655", "metadata": {"taxonomy": ["Bacteria", "__Proteobacteria", "__Betaproteobacteria", "__Hydrogenophilales", "__Hydrogenophilaceae", "__Thiobacillus"]}},{"id": "denovo7657", "metadata": {"taxonomy": ["Bacteria", "__Bacteroidetes", "__Flavobacteria", "__Flavobacteriales", "__Flavobacteriaceae"]}},{"id": "denovo7665", "metadata": {"taxonomy": ["Bacteria", "__Proteobacteria", "__Alphaproteobacteria", "__MNG3", "__f", "__g"]}},{"id": "denovo7666", "metadata": {"taxonomy": ["Bacteria", "__Proteobacteria", "__Gammaproteobacteria", "__E01-9C-26_marine_group", "__f", "__g"]}},{"id": "denovo7668", "metadata": {"taxonomy": ["Bacteria", "__Proteobacteria",

"__Gammaproteobacteria", "__Vibrionales", "__Vibrionaceae",
"__Vibrio"]}},{"id": "denovo7671", "metadata": {"taxonomy":
["Bacteria", "__Proteobacteria", "__Deltaproteobacteria",
"__SAR324_clade(Marine_group_B)", "__f", "__g"]}},{"id": "denovo7673",
"metadata": {"taxonomy": ["Bacteria", "__Proteobacteria",
"__Alphaproteobacteria", "__OCS116_clade", "__f", "__g"]}},{"id":
"denovo7679", "metadata": {"taxonomy": ["Bacteria",
"__Proteobacteria", "__Deltaproteobacteria", "__Sh765B-TzT-29", "__f",
"__g"]}},{"id": "denovo7682", "metadata": {"taxonomy": ["Bacteria",
"__Proteobacteria", "__Deltaproteobacteria", "__Desulfobacterales",
"__Desulfobacteraceae"]}},{"id": "denovo7683", "metadata":
{"taxonomy": ["Bacteria", "__Proteobacteria", "__Gammaproteobacteria",
"__Order_Incertae_Sedis", "__Family_Incertae_Sedis",
"__Thiohalomonas"]}},{"id": "denovo7689", "metadata": {"taxonomy":
["Bacteria", "__Verrucomicrobia", "__Verrucomicrobiae",
"__Verrucomicrobiales", "__DEV007", "__g"]}},{"id": "denovo7691",
"metadata": {"taxonomy": ["Bacteria", "__Proteobacteria",
"__Alphaproteobacteria", "__Rhodobacterales", "__Rhodobacteraceae",
"__g"]}},{"id": "denovo7692", "metadata": {"taxonomy": ["Bacteria",
"__Proteobacteria", "__Gammaproteobacteria", "__KI89A_clade", "__f",
"__g"]}},{"id": "denovo7693", "metadata": {"taxonomy": ["Bacteria",
"__Proteobacteria", "__Alphaproteobacteria", "__Rhizobiales",
"__Rhizobiaceae", "__Shinella"]}},{"id": "denovo7697", "metadata":
{"taxonomy": ["Bacteria"]}},{"id": "denovo7704", "metadata":
{"taxonomy": ["Bacteria", "__Proteobacteria", "__Alphaproteobacteria",
"__Rhodobacterales", "__Rhodobacteraceae"]}},{"id": "denovo7706",
"metadata": {"taxonomy": ["Bacteria", "__Proteobacteria",
"__Alphaproteobacteria", "__Rickettsiales"]}},{"id": "denovo7708",
"metadata": {"taxonomy": ["Bacteria", "__Proteobacteria",
"__Alphaproteobacteria", "__Sphingomonadales", "__f", "__g"]}},{"id":
"denovo7709", "metadata": {"taxonomy": ["Bacteria", "__Bacteroidetes",
"__Cytophagia", "__Cytophagales", "__Cyclobacteriaceae",
"__Rhodonellum"]}},{"id": "denovo7714", "metadata": {"taxonomy":
["Bacteria", "__Candidate_division_BRC1", "__c", "__o", "__f",
"__g"]}},{"id": "denovo7715", "metadata": {"taxonomy": ["Bacteria",
"__Proteobacteria", "__Gammaproteobacteria", "__Oceanospirillales",
"__OM182_clade", "__g"]}},{"id": "denovo7716", "metadata":
{"taxonomy": ["Bacteria", "__Proteobacteria", "__Deltaproteobacteria",
"__Bdellovibrionales", "__Bacteriovoracaceae", "__Peredibacter"]}},
{"id": "denovo7720", "metadata": {"taxonomy": ["Bacteria",
"__Proteobacteria", "__Alphaproteobacteria", "__Rhodobacterales",
"__Rhodobacteraceae"]}},{"id": "denovo7722", "metadata": {"taxonomy":
["Bacteria", "__Proteobacteria", "__Alphaproteobacteria",
"__Rhodospirillales", "__Family_Incertae_Sedis", "__Geminicoccus"]}},
{"id": "denovo7723", "metadata": {"taxonomy": ["Bacteria",
"__Proteobacteria", "__Gammaproteobacteria"]}},{"id": "denovo7725",
"metadata": {"taxonomy": ["Bacteria", "__Proteobacteria",
"__Alphaproteobacteria", "__Rhizobiales", "__Phyllobacteriaceae"]}},
{"id": "denovo7726", "metadata": {"taxonomy": ["Bacteria",
"__Proteobacteria", "__Alphaproteobacteria", "__Rhodospirillales"]}},

{"id": "denovo7728", "metadata": {"taxonomy": ["Archaea", "__Euryarchaeota", "__Halobacteria", "__Halobacteriales", "__Deep_Sea_Hydrothermal_Vent_Gp_6(DHVEG-6)", "__g"]}},{"id": "denovo7733", "metadata": {"taxonomy": ["Bacteria", "__Proteobacteria", "__Gammaproteobacteria", "__Chromatiales", "__Chromatiaceae", "__Nitrosococcus"]}},{"id": "denovo7736", "metadata": {"taxonomy": ["Bacteria", "__Proteobacteria", "__Alphaproteobacteria", "__Rhodospirillales", "__Rhodospirillaceae", "__g"]}},{"id": "denovo7737", "metadata": {"taxonomy": ["Bacteria", "__Proteobacteria", "__Gammaproteobacteria", "__Alteromonadales", "__Alteromonadaceae", "__BD1-7_clade"]}},{"id": "denovo7744", "metadata": {"taxonomy": ["Bacteria", "__Proteobacteria", "__Alphaproteobacteria", "__Rhodospirillales", "__Rhodospirillaceae", "__Rhodospirillum"]}},{"id": "denovo7749", "metadata": {"taxonomy": ["Bacteria", "__Proteobacteria", "__Gammaproteobacteria", "__KI89A_clade", "__f", "__g"]}},{"id": "denovo7752", "metadata": {"taxonomy": ["Bacteria", "__Proteobacteria", "__Gammaproteobacteria", "__Alteromonadales", "__Alteromonadaceae", "__Haliea"]}},{"id": "denovo7753", "metadata": {"taxonomy": ["Bacteria", "__Proteobacteria", "__Gammaproteobacteria", "__Alteromonadales", "__Ferrimonadaceae", "__Ferrimonas"]}},{"id": "denovo7756", "metadata": {"taxonomy": ["Bacteria", "__Cyanobacteria", "__ML635J-21", "__o", "__f", "__g"]}},{"id": "denovo7760", "metadata": {"taxonomy": ["Bacteria", "__Proteobacteria", "__Deltaproteobacteria", "__Desulfobacterales", "__Desulfobulbaceae", "__Desulfotalea"]}},{"id": "denovo7762", "metadata": {"taxonomy": ["Bacteria", "__Proteobacteria", "__Alphaproteobacteria", "__Sphingomonadales", "__Erythrobacteraceae"]}},{"id": "denovo7766", "metadata": {"taxonomy": ["Bacteria", "__Proteobacteria", "__Gammaproteobacteria", "__KI89A_clade", "__f", "__g"]}},{"id": "denovo7770", "metadata": {"taxonomy": ["Bacteria", "__Bacteroidetes"]}},{"id": "denovo7772", "metadata": {"taxonomy": ["Bacteria", "__Proteobacteria", "__Deltaproteobacteria", "__Desulfobacterales", "__Desulfobulbaceae", "__Desulfobulbus"]}},{"id": "denovo7776", "metadata": {"taxonomy": ["Bacteria", "__Gemmatimonadetes", "__Gemmatimonadetes", "__BD2-11_terrestrial_group", "__f", "__g"]}},{"id": "denovo7781", "metadata": {"taxonomy": ["Bacteria", "__Proteobacteria", "__Gammaproteobacteria", "__Oceanospirillales", "__Hahellaceae", "__Endozoicomonas"]}},{"id": "denovo7783", "metadata": {"taxonomy": ["Bacteria", "__Bacteroidetes", "__Cytophagia", "__Cytophagales", "__Flammeovirgaceae", "__Candidatus_Amoebophilus"]}},{"id": "denovo7785", "metadata": {"taxonomy": ["Bacteria", "__Proteobacteria", "__Gammaproteobacteria", "__Pasteurellales", "__Pasteurellaceae", "__Haemophilus"]}},{"id": "denovo7787", "metadata": {"taxonomy": ["Bacteria", "__Nitrospirae", "__Nitrospira", "__Nitrospirales", "__Nitrospiraceae", "__Nitrospira"]}},{"id": "denovo7789", "metadata": {"taxonomy": ["Bacteria", "__Acidobacteria", "__Acidobacteria", "__PAUC26f", "__f", "__g"]}},{"id": "denovo7793", "metadata": {"taxonomy": ["Bacteria", "__Proteobacteria", "__Gammaproteobacteria", "__Pseudomonadales", "__Moraxellaceae",

"__Acinetobacter"]}},{"id": "denovo7794", "metadata": {"taxonomy": ["Bacteria", "__Cyanobacteria"]}},{"id": "denovo7796", "metadata": {"taxonomy": ["Bacteria", "__Proteobacteria", "__Alphaproteobacteria", "__Rickettsiales", "__Holosporaceae", "__Holospora"]}},{"id": "denovo7799", "metadata": {"taxonomy": ["Bacteria", "__Proteobacteria", "__Alphaproteobacteria", "__Rhizobiales", "__Phyllobacteriaceae", "__Cohaesibacter"]}},{"id": "denovo7801", "metadata": {"taxonomy": ["Bacteria", "__Proteobacteria", "__Gammaproteobacteria"]}},{"id": "denovo7807", "metadata": {"taxonomy": ["Bacteria", "__Proteobacteria", "__Deltaproteobacteria", "__Myxococcales", "__Nannocystaceae", "__g"]}},{"id": "denovo7808", "metadata": {"taxonomy": ["Bacteria", "__Proteobacteria", "__Alphaproteobacteria", "__Rhodobacterales", "__Rhodobacteraceae", "__g"]}},{"id": "denovo7815", "metadata": {"taxonomy": ["Bacteria", "__Proteobacteria", "__Alphaproteobacteria", "__Rhodobacterales", "__Rhodobacteraceae"]}},{"id": "denovo7818", "metadata": {"taxonomy": ["Bacteria", "__Chloroflexi", "__SAR202_clade", "__o", "__f", "__g"]}},{"id": "denovo7820", "metadata": {"taxonomy": ["Bacteria", "__Cyanobacteria", "__Cyanobacteria", "__SubsectionIII", "__FamilyI", "__Symploca"]}},{"id": "denovo7822", "metadata": {"taxonomy": ["Bacteria", "__Planctomycetes", "__Planctomycetacia", "__Planctomycetales", "__Planctomycetaceae", "__Blastopirellula"]}},{"id": "denovo7824", "metadata": {"taxonomy": ["Bacteria", "__Bacteroidetes", "__Flavobacteria", "__Flavobacteriales", "__Flavobacteriaceae", "__Muricola"]}},{"id": "denovo7826", "metadata": {"taxonomy": ["Bacteria", "__Gemmatimonadetes", "__Gemmatimonadetes", "__BD2-11_terrestrial_group", "__f", "__g"]}},{"id": "denovo7836", "metadata": {"taxonomy": ["Bacteria", "__Proteobacteria", "__Alphaproteobacteria", "__Rhodobacterales", "__Rhodobacteraceae", "__Roseobacter"]}},{"id": "denovo7838", "metadata": {"taxonomy": ["Bacteria", "__Proteobacteria", "__Gammaproteobacteria", "__Alteromonadales", "__Alteromonadaceae", "__C1-B045"]}},{"id": "denovo7846", "metadata": {"taxonomy": ["Bacteria", "__Bacteroidetes", "__VC2.1_Bac22", "__o", "__f", "__g"]}},{"id": "denovo7850", "metadata": {"taxonomy": ["Bacteria", "__Proteobacteria", "__Gammaproteobacteria", "__Sva0071", "__f", "__g"]}},{"id": "denovo7852", "metadata": {"taxonomy": ["Bacteria", "__Candidate_division_BRC1", "__c", "__o", "__f", "__g"]}},{"id": "denovo7853", "metadata": {"taxonomy": ["Bacteria", "__Chlamydiae", "__Chlamydiae", "__Chlamydiales", "__Simkaniaceae", "__Simkania"]}},{"id": "denovo7863", "metadata": {"taxonomy": ["Bacteria", "__Proteobacteria", "__Alphaproteobacteria", "__Rhodobacterales", "__Rhodobacteraceae"]}},{"id": "denovo7866", "metadata": {"taxonomy": ["Bacteria", "__Firmicutes", "__Clostridia", "__Clostridiales", "__Clostridiaceae", "__Clostridiisalibacter"]}},{"id": "denovo7867", "metadata": {"taxonomy": ["Bacteria", "__Actinobacteria", "__Acidimicrobiia", "__Acidimicrobiales", "__Sva0996_marine_group", "__g"]}},{"id": "denovo7868", "metadata": {"taxonomy": ["Bacteria", "__Cyanobacteria"]}},{"id": "denovo7869", "metadata": {"taxonomy": ["Bacteria", "__Proteobacteria", "__Gammaproteobacteria",

"__BD7-8_marine_group", "__f", "__g"]}},{"id": "denovo7871", "metadata": {"taxonomy": ["Bacteria", "__Proteobacteria", "__Alphaproteobacteria", "__OCS116_clade", "__f", "__g"]}},{"id": "denovo7872", "metadata": {"taxonomy": ["Bacteria", "__Proteobacteria", "__Alphaproteobacteria", "__Sphingomonadales", "__SWB04", "__g"]}},{"id": "denovo7875", "metadata": {"taxonomy": ["Bacteria", "__Cyanobacteria", "__Cyanobacteria", "__SubsectionIII", "__FamilyI", "__Leptolyngbya"]}},{"id": "denovo7881", "metadata": {"taxonomy": ["Bacteria", "__Proteobacteria", "__Gammaproteobacteria", "__Alteromonadales", "__Alteromonadaceae", "__Haliea"]}},{"id": "denovo7883", "metadata": {"taxonomy": ["Bacteria", "__Bacteroidetes", "__Flavobacteria", "__Flavobacteriales", "__Flavobacteriaceae", "__Algibacter"]}},{"id": "denovo7888", "metadata": {"taxonomy": ["Bacteria", "__Proteobacteria", "__Gammaproteobacteria", "__Oceanospirillales", "__Oceanospirillaceae", "__Pseudospirillum"]}},{"id": "denovo7889", "metadata": {"taxonomy": ["Bacteria", "__Proteobacteria", "__Alphaproteobacteria", "__Rhizobiales", "__Phyllobacteriaceae", "__Aquamicrobium"]}},{"id": "denovo7890", "metadata": {"taxonomy": ["Bacteria", "__Proteobacteria", "__Gammaproteobacteria", "__Vibrionales", "__Vibrionaceae", "__Photobacterium"]}},{"id": "denovo7892", "metadata": {"taxonomy": ["Bacteria", "__Firmicutes", "__Clostridia", "__Clostridiales", "__Clostridiaceae", "__Clostridium"]}},{"id": "denovo7896", "metadata": {"taxonomy": ["Bacteria", "__Firmicutes", "__Clostridia", "__Clostridiales", "__Family_XII_Incertae_Sedis", "__Fusibacter"]}},{"id": "denovo7901", "metadata": {"taxonomy": ["Bacteria", "__Planctomycetes", "__Planctomycetacia", "__Planctomycetales", "__Planctomycetaceae", "__Rhodopirellula"]}},{"id": "denovo7906", "metadata": {"taxonomy": ["Bacteria", "__Cyanobacteria", "__Cyanobacteria", "__SubsectionI", "__FamilyI", "__Synechococcus"]}},{"id": "denovo7915", "metadata": {"taxonomy": ["Bacteria", "__Bacteroidetes", "__Sphingobacteriia", "__Sphingobacteriales", "__Saprospiraceae", "__g"]}},{"id": "denovo7923", "metadata": {"taxonomy": ["Bacteria", "__Bacteroidetes", "__Flavobacteria", "__Flavobacteriales", "__Cryomorphaceae", "__Brumimicrobium"]}},{"id": "denovo7927", "metadata": {"taxonomy": ["Bacteria", "__Proteobacteria", "__Alphaproteobacteria", "__Rhodobacterales", "__Rhodobacteraceae", "__Roseobacter_clade_AS-21_lineage"]}},{"id": "denovo7928", "metadata": {"taxonomy": ["Bacteria", "__Cyanobacteria", "__4C0d-2", "__o", "__f", "__g"]}},{"id": "denovo7929", "metadata": {"taxonomy": ["Bacteria", "__Proteobacteria", "__Gammaproteobacteria", "__Xanthomonadales", "__Xanthomonadaceae", "__g"]}},{"id": "denovo7932", "metadata": {"taxonomy": ["Bacteria", "__Proteobacteria", "__Deltaproteobacteria", "__Desulfobacterales", "__Nitrospinaceae", "__Nitrospina"]}},{"id": "denovo7934", "metadata": {"taxonomy": ["Bacteria", "__Proteobacteria", "__Alphaproteobacteria", "__Rhodobacterales", "__Rhodobacteraceae", "__g"]}},{"id": "denovo7935", "metadata": {"taxonomy": ["Bacteria", "__Proteobacteria", "__Alphaproteobacteria", "__Rhodospirillales", "__Rhodospirillaceae", "__g"]}},{"id": "denovo7937", "metadata":

{"taxonomy": ["Bacteria", "__Proteobacteria", "__Gammaproteobacteria", "__Alteromonadales", "__Alteromonadaceae", "__Haliea"]}},{"id": "denovo7938", "metadata": {"taxonomy": ["Bacteria", "__Proteobacteria", "__Gammaproteobacteria", "__Vibrionales", "__Vibrionaceae", "__Vibrio"]}},{"id": "denovo7941", "metadata": {"taxonomy": ["Bacteria", "__Proteobacteria", "__Gammaproteobacteria"]}},{"id": "denovo7943", "metadata": {"taxonomy": ["Bacteria", "__Proteobacteria", "__Alphaproteobacteria", "__Caulobacterales", "__Caulobacteraceae", "__g"]}},{"id": "denovo7945", "metadata": {"taxonomy": ["Bacteria", "__Bacteroidetes", "__Sphingobacteriia", "__Sphingobacteriales"]}},{"id": "denovo7947", "metadata": {"taxonomy": ["Bacteria", "__Bacteroidetes", "__Cytophagia", "__Cytophagales", "__Flammeovirgaceae", "__Flexithrix"]}},{"id": "denovo7949", "metadata": {"taxonomy": ["Bacteria", "__Cyanobacteria", "__Cyanobacteria", "__SubsectionIII", "__FamilyI", "__Phormidium"]}},{"id": "denovo7952", "metadata": {"taxonomy": ["Bacteria", "__Candidate_division_WS3", "__c", "__o", "__f", "__g"]}},{"id": "denovo7956", "metadata": {"taxonomy": ["Bacteria", "__Acidobacteria", "__Acidobacteria", "__BPC015", "__f", "__g"]}},{"id": "denovo7962", "metadata": {"taxonomy": ["Bacteria", "__Proteobacteria", "__Alphaproteobacteria", "__Rhodospirillales", "__Rhodospirillaceae", "__g"]}},{"id": "denovo7968", "metadata": {"taxonomy": ["Bacteria", "__Chloroflexi", "__TK10", "__o", "__f", "__g"]}},{"id": "denovo7969", "metadata": {"taxonomy": ["Bacteria", "__Proteobacteria", "__Gammaproteobacteria", "__Oceanospirillales", "__Oceanospirillaceae"]}},{"id": "denovo7970", "metadata": {"taxonomy": ["Bacteria", "__Firmicutes", "__Clostridia", "__Clostridiales", "__Peptostreptococcaceae", "__g"]}},{"id": "denovo7972", "metadata": {"taxonomy": ["Bacteria", "__Cyanobacteria", "__Cyanobacteria", "__SubsectionI", "__FamilyI", "__Synechococcus"]}},{"id": "denovo7973", "metadata": {"taxonomy": ["Bacteria", "__Proteobacteria", "__Gammaproteobacteria", "__Alteromonadales", "__Alteromonadaceae", "__OM60(NOR5)_clade"]}},{"id": "denovo7974", "metadata": {"taxonomy": ["Bacteria", "__Proteobacteria", "__Gammaproteobacteria", "__Oceanospirillales", "__CrystalBog021C3", "__g"]}},{"id": "denovo7975", "metadata": {"taxonomy": ["Bacteria", "__Bacteroidetes", "__Cytophagia", "__Cytophagales", "__Flammeovirgaceae"]}},{"id": "denovo7981", "metadata": {"taxonomy": ["Bacteria", "__Proteobacteria", "__Deltaproteobacteria", "__Myxococcales", "__Cystobacterineae", "__g"]}},{"id": "denovo7983", "metadata": {"taxonomy": ["Bacteria", "__Proteobacteria", "__Gammaproteobacteria", "__Xanthomonadales", "__Sinobacteraceae"]}},{"id": "denovo7988", "metadata": {"taxonomy": ["Bacteria", "__Acidobacteria", "__Holophagae", "__Sva0725", "__f", "__g"]}},{"id": "denovo7989", "metadata": {"taxonomy": ["Bacteria", "__Proteobacteria", "__Alphaproteobacteria", "__Rhodospirillales", "__Rhodospirillaceae"]}},{"id": "denovo7990", "metadata": {"taxonomy": ["Bacteria", "__Bacteroidetes", "__Cytophagia", "__Order_II_Incertae_Sedis", "__Rhodothermaceae", "__g"]}},{"id": "denovo7991", "metadata": {"taxonomy": ["Bacteria",

"__Proteobacteria", "__Gammaproteobacteria", "__BD3-1", "__f",
"__g"]}},{"id": "denovo7993", "metadata": {"taxonomy": ["Bacteria",
"__Cyanobacteria", "__Cyanobacteria", "__SubsectionIII", "__FamilyI",
"__Lyngbya"]}},{"id": "denovo7995", "metadata": {"taxonomy":
["Bacteria", "__Proteobacteria", "__Alphaproteobacteria",
"__Sphingomonadales", "__Sphingomonadaceae", "__Sphingosinicella"]}},
{"id": "denovo7996", "metadata": {"taxonomy": ["Bacteria",
"__Proteobacteria", "__Deltaproteobacteria", "__Myxococcales",
"__0319-6G20", "__g"]}},{"id": "denovo7998", "metadata": {"taxonomy":
["Bacteria", "__Proteobacteria", "__Alphaproteobacteria",
"__Rhodospirillales", "__Rhodospirillaceae"]}},{"id": "denovo8000",
"metadata": {"taxonomy": ["Bacteria", "__Planctomycetes",
"__Planctomycetacia", "__Planctomycetales", "__Planctomycetaceae",
"__Rhodopirellula"]}},{"id": "denovo8001", "metadata": {"taxonomy":
["Bacteria", "__Proteobacteria", "__Deltaproteobacteria",
"__Myxococcales", "__Cystobacterineae", "__g"]}},{"id": "denovo8003",
"metadata": {"taxonomy": ["Bacteria", "__Firmicutes", "__Clostridia",
"__Clostridiales", "__Lachnospiraceae"]}},{"id": "denovo8007",
"metadata": {"taxonomy": ["Bacteria", "__Bacteroidetes",
"__Flavobacteria", "__Flavobacteriales", "__Cryomorphaceae",
"__Crocinitomix"]}},{"id": "denovo8010", "metadata": {"taxonomy":
["Bacteria", "__Proteobacteria", "__Betaproteobacteria"]}},{"id":
"denovo8011", "metadata": {"taxonomy": ["Bacteria",
"__Proteobacteria", "__Deltaproteobacteria", "__Desulfurellales",
"__Desulfurellaceae", "__g"]}},{"id": "denovo8016", "metadata":
{"taxonomy": ["Bacteria", "__Proteobacteria", "__Gammaproteobacteria",
"__Alteromonadales", "__Colwelliaceae", "__Thalassomonas"]}},{"id":
"denovo8017", "metadata": {"taxonomy": ["Bacteria",
"__Proteobacteria", "__Gammaproteobacteria", "__Pseudomonadales",
"__Moraxellaceae", "__g"]}},{"id": "denovo8021", "metadata":
{"taxonomy": ["Bacteria", "__Proteobacteria", "__Deltaproteobacteria",
"__Myxococcales", "__Cystobacterineae", "__g"]}},{"id": "denovo8023",
"metadata": {"taxonomy": ["Bacteria", "__Acidobacteria", "__RB25",
"__o", "__f", "__g"]}},{"id": "denovo8024", "metadata": {"taxonomy":
["Bacteria", "__Proteobacteria", "__Alphaproteobacteria",
"__Rhodospirillales", "__Rhodospirillaceae", "__g"]}},{"id":
"denovo8026", "metadata": {"taxonomy": ["Bacteria",
"__Proteobacteria", "__Alphaproteobacteria", "__Rhodobacterales",
"__Rhodobacteraceae", "__Marinovum"]}},{"id": "denovo8028",
"metadata": {"taxonomy": ["Bacteria", "__Proteobacteria",
"__Alphaproteobacteria", "__Rhizobiales", "__Hyphomicrobiaceae",
"__Devosia"]}},{"id": "denovo8029", "metadata": {"taxonomy":
["Bacteria", "__Chlamydiae", "__Chlamydiae", "__Chlamydiales",
"__Simkaniaceae"]}},{"id": "denovo8033", "metadata": {"taxonomy":
["Bacteria", "__Proteobacteria", "__Gammaproteobacteria",
"__Oceanospirillales", "__Hahellaceae", "__Endozoicomonas"]}},{"id":
"denovo8036", "metadata": {"taxonomy": ["Bacteria",
"__Proteobacteria", "__Gammaproteobacteria"]}},{"id": "denovo8037",
"metadata": {"taxonomy": ["Archaea", "__Euryarchaeota",
"__Halobacteria", "__Halobacteriales", "__Halobacteriaceae",

"__Halococcus"]}},{"id": "denovo8039", "metadata": {"taxonomy": ["Bacteria", "__Proteobacteria", "__Deltaproteobacteria", "__Bdellovibrionales", "__Bdellovibrionaceae", "__OM27_clade"]}}, {"id": "denovo8045", "metadata": {"taxonomy": ["Bacteria", "__Proteobacteria", "__Deltaproteobacteria", "__Myxococcales", "__0319-6G20", "__g"]}},{"id": "denovo8046", "metadata": {"taxonomy": ["Bacteria", "__Nitrospirae", "__Nitrospira", "__Nitrospirales", "__OPB95", "__g"]}},{"id": "denovo8049", "metadata": {"taxonomy": ["Bacteria", "__TM6", "__c", "__o", "__f", "__g"]}},{"id": "denovo8050", "metadata": {"taxonomy": ["Bacteria", "__Bacteroidetes", "__Cytophagia", "__Cytophagales", "__Flammeovirgaceae", "__Candidatus_Amoebophilus"]}},{"id": "denovo8051", "metadata": {"taxonomy": ["Bacteria", "__Bacteroidetes", "__Cytophagia", "__Order_II_Incertae_Sedis", "__Rhodothermaceae", "__Rubricoccus"]}}, {"id": "denovo8054", "metadata": {"taxonomy": ["Bacteria", "__Proteobacteria", "__Gammaproteobacteria", "__Alteromonadales", "__Alteromonadaceae", "__OM60(NOR5)_clade"]}},{"id": "denovo8060", "metadata": {"taxonomy": ["Bacteria", "__Proteobacteria", "__Gammaproteobacteria", "__HOC36", "__f", "__g"]}},{"id": "denovo8064", "metadata": {"taxonomy": ["Bacteria", "__Proteobacteria", "__Gammaproteobacteria", "__Xanthomonadales", "__Sinobacteraceae", "__JTB255_marine_benthic_group"]}},{"id": "denovo8065", "metadata": {"taxonomy": ["Bacteria", "__Bacteroidetes", "__Flavobacteria", "__Flavobacteriales", "__Flavobacteriaceae", "__Tenacibaculum"]}},{"id": "denovo8066", "metadata": {"taxonomy": ["Bacteria", "__Proteobacteria", "__Alphaproteobacteria", "__Rhodobacterales", "__Rhodobacteraceae"]}},{"id": "denovo8069", "metadata": {"taxonomy": ["Bacteria", "__Proteobacteria", "__Deltaproteobacteria", "__Bdellovibrionales", "__Bdellovibrionaceae", "__OM27_clade"]}},{"id": "denovo8070", "metadata": {"taxonomy": ["Bacteria", "__Proteobacteria", "__Gammaproteobacteria", "__Oceanospirillales", "__Hahellaceae", "__Endozoicomonas"]}},{"id": "denovo8076", "metadata": {"taxonomy": ["Bacteria", "__Proteobacteria", "__Gammaproteobacteria", "__Alteromonadales", "__Alteromonadaceae", "__Simiduia"]}},{"id": "denovo8077", "metadata": {"taxonomy": ["Bacteria", "__Proteobacteria", "__Deltaproteobacteria", "__Myxococcales", "__Sandaracinaceae", "__Sandaracinus"]}},{"id": "denovo8078", "metadata": {"taxonomy": ["Bacteria", "__Bacteroidetes", "__Cytophagia", "__Order_II_Incertae_Sedis", "__Rhodothermaceae", "__g"]}},{"id": "denovo8079", "metadata": {"taxonomy": ["Bacteria", "__Bacteroidetes", "__Cytophagia", "__Order_II_Incertae_Sedis", "__Rhodothermaceae", "__g"]}},{"id": "denovo8082", "metadata": {"taxonomy": ["Bacteria", "__Bacteroidetes", "__Flavobacteria", "__Flavobacteriales", "__Flavobacteriaceae", "__Formosa"]}},{"id": "denovo8086", "metadata": {"taxonomy": ["Bacteria", "__BD1-5", "__c", "__o", "__f", "__g"]}},{"id": "denovo8087", "metadata": {"taxonomy": ["Bacteria", "__Proteobacteria", "__Alphaproteobacteria", "__Rhodobacterales", "__Rhodobacteraceae", "__Roseobacter_clade_OCT_lineage"]}},{"id": "denovo8090", "metadata":

{"taxonomy": ["Bacteria", "__Cyanobacteria", "__Cyanobacteria", "__SubsectionIII", "__FamilyI", "__Planktothricoides"]}},{"id": "denovo8092", "metadata": {"taxonomy": ["Bacteria", "__Acidobacteria", "__Acidobacteria", "__AT-s3-28", "__f", "__g"]}},{"id": "denovo8094", "metadata": {"taxonomy": ["Bacteria", "__Bacteroidetes", "__Flavobacteria", "__Flavobacteriales", "__Flavobacteriaceae", "__NS5_marine_group"]}},{"id": "denovo8096", "metadata": {"taxonomy": ["Bacteria", "__Proteobacteria", "__Gammaproteobacteria", "__Alteromonadales", "__Alteromonadaceae"]}},{"id": "denovo8100", "metadata": {"taxonomy": ["Bacteria", "__Proteobacteria", "__Gammaproteobacteria", "__Pseudomonadales", "__Moraxellaceae", "__Acinetobacter"]}},{"id": "denovo8101", "metadata": {"taxonomy": ["Archaea", "__Euryarchaeota", "__Thermoplasmata", "__Thermoplasmatales", "__Marine_Group_II", "__g"]}},{"id": "denovo8102", "metadata": {"taxonomy": ["Bacteria", "__Proteobacteria", "__Alphaproteobacteria", "__Rhodobacterales", "__Rhodobacteraceae", "__g"]}},{"id": "denovo8105", "metadata": {"taxonomy": ["Bacteria", "__Bacteroidetes", "__Bacteroidia", "__Bacteroidales", "__Marinilabiaceae", "__Marinifilum"]}},{"id": "denovo8108", "metadata": {"taxonomy": ["Bacteria", "__Proteobacteria", "__Alphaproteobacteria", "__Rickettsiales"]}},{"id": "denovo8110", "metadata": {"taxonomy": ["Bacteria", "__Proteobacteria", "__Alphaproteobacteria", "__Caulobacterales", "__Hyphomonadaceae", "__g"]}},{"id": "denovo8116", "metadata": {"taxonomy": ["Bacteria", "__Acidobacteria", "__Acidobacteria", "__DA023", "__f", "__g"]}},{"id": "denovo8118", "metadata": {"taxonomy": ["Bacteria", "__Proteobacteria", "__Deltaproteobacteria", "__Myxococcales", "__Haliangiaceae", "__Haliangium"]}},{"id": "denovo8123", "metadata": {"taxonomy": ["Bacteria", "__Proteobacteria", "__Deltaproteobacteria"]}},{"id": "denovo8127", "metadata": {"taxonomy": ["Bacteria", "__Proteobacteria", "__Betaproteobacteria", "__Burkholderiales", "__Comamonadaceae", "__Tepidimonas"]}},{"id": "denovo8128", "metadata": {"taxonomy": ["Bacteria", "__Bacteroidetes", "__Cytophagia", "__Cytophagales", "__Flammeovirgaceae", "__g"]}},{"id": "denovo8130", "metadata": {"taxonomy": ["Bacteria", "__Acidobacteria", "__Holophagae", "__B276-D12", "__f", "__g"]}},{"id": "denovo8133", "metadata": {"taxonomy": ["Bacteria", "__Bacteroidetes", "__Flavobacteria", "__Flavobacteriales", "__Cryomorphaceae", "__Crocinitomix"]}},{"id": "denovo8134", "metadata": {"taxonomy": ["Bacteria", "__Bacteroidetes", "__Cytophagia", "__Cytophagales", "__Flammeovirgaceae"]}},{"id": "denovo8135", "metadata": {"taxonomy": ["Bacteria", "__Proteobacteria", "__Betaproteobacteria", "__Methylophilales", "__Methylophilaceae", "__Methylophilus"]}},{"id": "denovo8136", "metadata": {"taxonomy": ["Bacteria", "__Proteobacteria", "__Gammaproteobacteria", "__Alteromonadales", "__Alteromonadaceae", "__OM60(NOR5)_clade"]}},{"id": "denovo8139", "metadata": {"taxonomy": ["Bacteria", "__Chloroflexi", "__SAR202_clade", "__o", "__f", "__g"]}},{"id": "denovo8141", "metadata": {"taxonomy": ["Bacteria", "__Planctomycetes", "__Planctomycetacia", "__Planctomycetales",

"__Planctomycetaceae", "__Rhodopirellula"]}},{"id": "denovo8150",
"metadata": {"taxonomy": ["Bacteria", "__Proteobacteria",
"__Alphaproteobacteria", "__SAR11_clade", "__Surface_1",
"__Candidatus_Pelagibacter"]}},{"id": "denovo8151", "metadata":
{"taxonomy": ["Bacteria", "__Proteobacteria", "__Deltaproteobacteria",
"__Desulfovibrionales", "__Desulfovibrionaceae", "__Desulfovibrio"]}},
{"id": "denovo8153", "metadata": {"taxonomy": ["Bacteria",
"__Proteobacteria", "__Deltaproteobacteria", "__Myxococcales"]}},
{"id": "denovo8154", "metadata": {"taxonomy": ["Bacteria",
"__Bacteroidetes", "__Flavobacteria", "__Flavobacteriales",
"__Cryomorphaceae", "__Owenweeksia"]}},{"id": "denovo8157",
"metadata": {"taxonomy": ["Bacteria", "__Cyanobacteria",
"__Cyanobacteria", "__SubsectionIV"]}},{"id": "denovo8161",
"metadata": {"taxonomy": ["Bacteria", "__Cyanobacteria",
"__Cyanobacteria", "__o", "__f", "__g"]}},{"id": "denovo8162",
"metadata": {"taxonomy": ["Bacteria", "__Actinobacteria",
"__Actinobacteria", "__Micrococcales", "__Microbacteriaceae"]}},{"id":
"denovo8163", "metadata": {"taxonomy": ["Bacteria", "__Chlorobi",
"__Chlorobia", "__Chlorobiales", "__Chlorobiaceae",
"__Prosthecochloris"]}},{"id": "denovo8170", "metadata": {"taxonomy":
["Bacteria", "__Proteobacteria", "__Deltaproteobacteria", "__Sh765B-
TzT-29", "__f", "__g"]}},{"id": "denovo8173", "metadata": {"taxonomy":
["Bacteria", "__Firmicutes", "__Bacilli", "__Bacillales",
"__Bacillaceae", "__Bacillus"]}},{"id": "denovo8176", "metadata":
{"taxonomy": ["Bacteria", "__Cyanobacteria", "__Cyanobacteria",
"__SubsectionIII", "__FamilyI", "__Phormidium"]}},{"id": "denovo8178",
"metadata": {"taxonomy": ["Bacteria", "__Proteobacteria",
"__Deltaproteobacteria", "__Desulfobacterales",
"__Desulfobacteraceae", "__Desulfobacter"]}},{"id": "denovo8179",
"metadata": {"taxonomy": ["Bacteria", "__Proteobacteria",
"__Gammaproteobacteria", "__Xanthomonadales", "__Sinobacteraceae",
"__JTB255_marine_benthic_group"]}},{"id": "denovo8186", "metadata":
{"taxonomy": ["Bacteria", "__Proteobacteria", "__Gammaproteobacteria",
"__Alteromonadales", "__Colwelliaceae", "__Colwellia"]}},{"id":
"denovo8190", "metadata": {"taxonomy": ["Bacteria",
"__Proteobacteria", "__Gammaproteobacteria", "__KI89A_clade", "__f",
"__g"]}},{"id": "denovo8194", "metadata": {"taxonomy": ["Bacteria",
"__Proteobacteria", "__Gammaproteobacteria", "__Xanthomonadales",
"__Sinobacteraceae", "__JTB255_marine_benthic_group"]}},{"id":
"denovo8197", "metadata": {"taxonomy": ["Bacteria",
"__Proteobacteria", "__Gammaproteobacteria", "__Thiotrichales",
"__Piscirickettsiaceae", "__endosymbionts"]}},{"id": "denovo8198",
"metadata": {"taxonomy": ["Bacteria", "__Planctomycetes",
"__Phycisphaerae", "__Phycisphaerales", "__Phycisphaeraceae",
"__Phycisphaera"]}},{"id": "denovo8200", "metadata": {"taxonomy":
["Bacteria", "__Proteobacteria", "__Betaproteobacteria",
"__Burkholderiales", "__Comamonadaceae", "__Delftia"]}},{"id":
"denovo8201", "metadata": {"taxonomy": ["Bacteria",
"__Proteobacteria", "__Gammaproteobacteria", "__Alteromonadales",
"__Alteromonadaceae"]}},{"id": "denovo8203", "metadata": {"taxonomy":

["Bacteria", "__Proteobacteria", "__Gammaproteobacteria"]}},{"id": "denovo8205", "metadata": {"taxonomy": ["Bacteria", "__Bacteroidetes", "__Sphingobacteriia", "__Sphingobacteriales", "__Saprospiraceae", "__Lewinella"]}},{"id": "denovo8206", "metadata": {"taxonomy": ["Bacteria", "__Bacteroidetes", "__Sphingobacteriia", "__Sphingobacteriales", "__Saprospiraceae", "__g"]}},{"id": "denovo8208", "metadata": {"taxonomy": ["Bacteria", "__Proteobacteria", "__Alphaproteobacteria", "__DB1-14", "__f", "__g"]}},{"id": "denovo8212", "metadata": {"taxonomy": ["Bacteria", "__Proteobacteria", "__Deltaproteobacteria", "__Sh765B-TzT-29", "__f", "__g"]}},{"id": "denovo8214", "metadata": {"taxonomy": ["Bacteria", "__Chloroflexi", "__SAR202_clade", "__o", "__f", "__g"]}},{"id": "denovo8220", "metadata": {"taxonomy": ["Bacteria", "__Proteobacteria", "__Alphaproteobacteria", "__SAR11_clade", "__Surface_1", "__Candidatus_Pelagibacter"]}},{"id": "denovo8221", "metadata": {"taxonomy": ["Bacteria", "__Proteobacteria", "__Deltaproteobacteria", "__Myxococcales", "__Nannocystaceae", "__g"]}},{"id": "denovo8227", "metadata": {"taxonomy": ["Bacteria", "__Proteobacteria", "__Alphaproteobacteria", "__Rhizobiales", "__Hyphomicrobiaceae", "__Maritalea"]}},{"id": "denovo8229", "metadata": {"taxonomy": ["Bacteria", "__Proteobacteria", "__Gammaproteobacteria", "__Alteromonadales", "__Alteromonadaceae"]}},{"id": "denovo8231", "metadata": {"taxonomy": ["Bacteria", "__Firmicutes", "__Clostridia", "__Clostridiales", "__Ruminococcaceae", "__Acetivibrio"]}},{"id": "denovo8232", "metadata": {"taxonomy": ["Bacteria", "__Acidobacteria", "__Holophagae"]}},{"id": "denovo8236", "metadata": {"taxonomy": ["Bacteria", "__Proteobacteria", "__Gammaproteobacteria", "__Oceanospirillales", "__Hahellaceae", "__Endozoicomonas"]}},{"id": "denovo8242", "metadata": {"taxonomy": ["Bacteria", "__Proteobacteria", "__Gammaproteobacteria", "__Order_Incertae_Sedis", "__Family_Incertae_Sedis", "__Arenicella"]}},{"id": "denovo8249", "metadata": {"taxonomy": ["Bacteria", "__Bacteroidetes", "__Flavobacteria", "__Flavobacteriales", "__Flavobacteriaceae", "__Croceitalea"]}},{"id": "denovo8250", "metadata": {"taxonomy": ["Archaea", "__Euryarchaeota", "__Halobacteria", "__Halobacteriales", "__Deep_Sea_Hydrothermal_Vent_Gp_6(DHVEG-6)", "__Candidatus_Parvarchaeum"]}},{"id": "denovo8251", "metadata": {"taxonomy": ["Bacteria", "__Proteobacteria", "__Gammaproteobacteria", "__Oceanospirillales", "__Hahellaceae", "__Endozoicomonas"]}},{"id": "denovo8252", "metadata": {"taxonomy": ["Bacteria", "__Acidobacteria", "__RB25", "__o", "__f", "__g"]}},{"id": "denovo8254", "metadata": {"taxonomy": ["Bacteria", "__Proteobacteria", "__Alphaproteobacteria", "__Rhodospirillales", "__Rhodospirillaceae", "__Defluviicoccus"]}},{"id": "denovo8255", "metadata": {"taxonomy": ["Bacteria", "__Proteobacteria", "__Alphaproteobacteria", "__Rhodobacterales", "__Rhodobacteraceae"]}},{"id": "denovo8257", "metadata": {"taxonomy": ["Bacteria", "__Lentisphaerae", "__Lentisphaeria", "__Victivallales", "__Victivallaceae", "__g"]}},{"id": "denovo8260", "metadata": {"taxonomy": ["Bacteria", "__Bacteroidetes", "__Flavobacteria",

"__Flavobacteriales", "__Flavobacteriaceae"]}},{"id": "denovo8261", "metadata": {"taxonomy": ["Bacteria", "__Proteobacteria", "__Gammaproteobacteria", "__KI89A_clade", "__f", "__g"]}},{"id": "denovo8263", "metadata": {"taxonomy": ["Bacteria", "__Acidobacteria", "__Holophagae", "__NS72", "__f", "__g"]}},{"id": "denovo8264", "metadata": {"taxonomy": ["Bacteria", "__Actinobacteria", "__Acidimicrobiia", "__Acidimicrobiales", "__uncultured", "__g"]}}, {"id": "denovo8268", "metadata": {"taxonomy": ["Bacteria", "__Candidate_division_BRC1", "__c", "__o", "__f", "__g"]}},{"id": "denovo8273", "metadata": {"taxonomy": ["Bacteria", "__Proteobacteria", "__Gammaproteobacteria", "__Oceanospirillales", "__Oleiphilaceae", "__Oleiphilus"]}},{"id": "denovo8275", "metadata": {"taxonomy": ["Archaea", "__Thaumarchaeota", "__Marine_Group_I", "__Candidatus_Nitrosopumilus", "__f", "__g"]}},{"id": "denovo8277", "metadata": {"taxonomy": ["Bacteria", "__Proteobacteria", "__Alphaproteobacteria", "__Rhodospirillales", "__Rhodospirillaceae"]}},{"id": "denovo8279", "metadata": {"taxonomy": ["Bacteria", "__Proteobacteria", "__Alphaproteobacteria", "__Rhodospirillales", "__Rhodospirillaceae", "__g"]}},{"id": "denovo8282", "metadata": {"taxonomy": ["Bacteria", "__Proteobacteria", "__Deltaproteobacteria", "__Myxococcales", "__Nannocystaceae", "__g"]}},{"id": "denovo8284", "metadata": {"taxonomy": ["Bacteria", "__Bacteroidetes", "__Flavobacteria", "__Flavobacteriales", "__Flavobacteriaceae", "__Kordia"]}},{"id": "denovo8285", "metadata": {"taxonomy": ["Bacteria", "__Proteobacteria", "__Gammaproteobacteria", "__Pseudomonadales", "__Moraxellaceae", "__Acinetobacter"]}},{"id": "denovo8292", "metadata": {"taxonomy": ["Bacteria", "__Proteobacteria", "__Gammaproteobacteria", "__Oceanospirillales", "__Hahellaceae", "__Kistimonas"]}},{"id": "denovo8294", "metadata": {"taxonomy": ["Bacteria", "__Proteobacteria", "__Gammaproteobacteria", "__Chromatiales"]}},{"id": "denovo8295", "metadata": {"taxonomy": ["Bacteria", "__Proteobacteria", "__Alphaproteobacteria", "__Rhodospirillales", "__Rhodospirillaceae", "__Defluviicoccus"]}}, {"id": "denovo8298", "metadata": {"taxonomy": ["Bacteria", "__Proteobacteria", "__Alphaproteobacteria", "__Rhodobacterales", "__Rhodobacteraceae", "__Roseobacter_clade_OCT_lineage"]}},{"id": "denovo8300", "metadata": {"taxonomy": ["Bacteria", "__Firmicutes", "__Clostridia", "__Clostridiales", "__Lachnospiraceae", "__Catonella"]}},{"id": "denovo8304", "metadata": {"taxonomy": ["Bacteria", "__Proteobacteria", "__Deltaproteobacteria"]}},{"id": "denovo8308", "metadata": {"taxonomy": ["Bacteria", "__Proteobacteria", "__Gammaproteobacteria", "__Xanthomonadales", "__Sinobacteraceae", "__JTB255_marine_benthic_group"]}},{"id": "denovo8313", "metadata": {"taxonomy": ["Bacteria", "__Chloroflexi", "__Caldilineae", "__Caldilineales", "__Caldilineaceae", "__g"]}}, {"id": "denovo8314", "metadata": {"taxonomy": ["Bacteria", "__Proteobacteria", "__Gammaproteobacteria", "__Alteromonadales", "__Alteromonadaceae"]}},{"id": "denovo8318", "metadata": {"taxonomy": ["Bacteria", "__Proteobacteria", "__Deltaproteobacteria",

"__Myxococcales", "__Nannocystaceae", "__Nannocystis"]}},{"id": "denovo8324", "metadata": {"taxonomy": ["Bacteria", "__Acidobacteria", "__Holophagae", "__CA002", "__f", "__g"]}},{"id": "denovo8325", "metadata": {"taxonomy": ["Bacteria", "__Nitrospirae", "__Nitrospira", "__Nitrospirales", "__Nitrospiraceae", "__Nitrospira"]}},{"id": "denovo8327", "metadata": {"taxonomy": ["Bacteria", "__Actinobacteria", "__Actinobacteria", "__Micrococcales", "__Micrococcaceae", "__Nesterenkonia"]}},{"id": "denovo8328", "metadata": {"taxonomy": ["Archaea", "__Euryarchaeota", "__Thermoplasmata", "__Thermoplasmatales", "__Marine_Group_II", "__g"]}},{"id": "denovo8329", "metadata": {"taxonomy": ["Archaea", "__Euryarchaeota", "__Halobacteria", "__Halobacteriales", "__Deep_Sea_Hydrothermal_Vent_Gp_6(DHVEG-6)", "__Candidatus_Parvarchaeum"]}},{"id": "denovo8330", "metadata": {"taxonomy": ["Bacteria", "__Bacteroidetes", "__Sphingobacteriia", "__Sphingobacteriales", "__Saprospiraceae", "__g"]}},{"id": "denovo8333", "metadata": {"taxonomy": ["Bacteria", "__Bacteroidetes", "__Cytophagia", "__Cytophagales", "__Flammeovirgaceae", "__Ekhidna"]}},{"id": "denovo8337", "metadata": {"taxonomy": ["Bacteria", "__Firmicutes", "__Clostridia", "__Clostridiales", "__Lachnospiraceae"]}},{"id": "denovo8338", "metadata": {"taxonomy": ["Bacteria", "__Gemmatimonadetes", "__Gemmatimonadetes", "__BD2-11_terrestrial_group", "__f", "__g"]}},{"id": "denovo8339", "metadata": {"taxonomy": ["Bacteria", "__Planctomycetes", "__Phycisphaerae", "__Phycisphaerales", "__Phycisphaeraceae", "__SM1A02"]}},{"id": "denovo8341", "metadata": {"taxonomy": ["Bacteria", "__Bacteroidetes", "__Cytophagia", "__Cytophagales"]}},{"id": "denovo8342", "metadata": {"taxonomy": ["Bacteria", "__Proteobacteria", "__Gammaproteobacteria", "__KI89A_clade", "__f", "__g"]}},{"id": "denovo8343", "metadata": {"taxonomy": ["Bacteria", "__Bacteroidetes", "__Cytophagia", "__Cytophagales"]}},{"id": "denovo8345", "metadata": {"taxonomy": ["Bacteria", "__Proteobacteria", "__Deltaproteobacteria", "__Myxococcales", "__Cystobacterineae", "__g"]}},{"id": "denovo8347", "metadata": {"taxonomy": ["Bacteria", "__Chloroflexi", "__Caldilineae", "__Caldilineales", "__Caldilineaceae"]}},{"id": "denovo8348", "metadata": {"taxonomy": ["Bacteria", "__Firmicutes", "__Clostridia", "__Clostridiales", "__Clostridiaceae", "__Clostridiisalibacter"]}},{"id": "denovo8349", "metadata": {"taxonomy": ["Archaea", "__Euryarchaeota", "__Halobacteria", "__Halobacteriales", "__Deep_Sea_Hydrothermal_Vent_Gp_6(DHVEG-6)", "__Candidatus_Parvarchaeum"]}},{"id": "denovo8356", "metadata": {"taxonomy": ["Bacteria", "__Proteobacteria", "__Betaproteobacteria", "__Hydrogenophilales", "__Hydrogenophilaceae", "__Thiobacillus"]}},{"id": "denovo8359", "metadata": {"taxonomy": ["Bacteria", "__Proteobacteria", "__Alphaproteobacteria", "__Rhodospirillales", "__Rhodospirillaceae", "__Thalassospira"]}},{"id": "denovo8360", "metadata": {"taxonomy": ["Bacteria", "__Proteobacteria", "__Deltaproteobacteria", "__Myxococcales", "__Cystobacterineae", "__g"]}},{"id": "denovo8367", "metadata": {"taxonomy": ["Bacteria",

"__Acidobacteria", "__RB25", "__o", "__f", "__g"]}},{"id":
"denovo8368", "metadata": {"taxonomy": ["Bacteria", "__Bacteroidetes",
"__Sphingobacteriia", "__Sphingobacteriales", "__Saprospiraceae",
"__g"]}},{"id": "denovo8369", "metadata": {"taxonomy": ["Bacteria",
"__Proteobacteria", "__Gammaproteobacteria", "__Oceanospirillales",
"__B78-89", "__g"]}},{"id": "denovo8370", "metadata": {"taxonomy":
["Bacteria", "__Cyanobacteria", "__Cyanobacteria", "__SubsectionI",
"__FamilyI", "__Synechococcus"]}},{"id": "denovo8376", "metadata":
{"taxonomy": ["Bacteria", "__Proteobacteria", "__Alphaproteobacteria",
"__DB1-14", "__f", "__g"]}},{"id": "denovo8381", "metadata":
{"taxonomy": ["Bacteria", "__Proteobacteria", "__Deltaproteobacteria",
"__Desulfobacterales", "__Desulfobacteraceae", "__g"]}},{"id":
"denovo8383", "metadata": {"taxonomy": ["Bacteria",
"__Proteobacteria", "__Deltaproteobacteria"]}},{"id": "denovo8384",
"metadata": {"taxonomy": ["Bacteria", "__Proteobacteria",
"__Deltaproteobacteria", "__Bdellovibrionales",
"__Bacteriovoracaceae", "__Peredibacter"]}},{"id": "denovo8385",
"metadata": {"taxonomy": ["Bacteria", "__Proteobacteria",
"__Alphaproteobacteria", "__Kordiimonadales", "__Kordiimonadaceae",
"__Kordiimonas"]}},{"id": "denovo8386", "metadata": {"taxonomy":
["Bacteria", "__Proteobacteria", "__Gammaproteobacteria",
"__Alteromonadales", "__Colwelliaceae", "__Thalassomonas"]}},{"id":
"denovo8387", "metadata": {"taxonomy": ["Bacteria", "__Cyanobacteria",
"__Cyanobacteria", "__SubsectionI", "__FamilyI"]}},{"id":
"denovo8388", "metadata": {"taxonomy": ["Bacteria",
"__Bacteroidetes"]}},{"id": "denovo8390", "metadata": {"taxonomy":
["Bacteria", "__Proteobacteria", "__Deltaproteobacteria",
"__Desulfobacterales", "__Nitrospinaceae", "__Nitrospina"]}},{"id":
"denovo8391", "metadata": {"taxonomy": ["Bacteria",
"__Proteobacteria", "__Deltaproteobacteria", "__Myxococcales",
"__Sorangiineae", "__Sandaracinaceae"]}},{"id": "denovo8395",
"metadata": {"taxonomy": ["Bacteria", "__Planctomycetes",
"__Phycisphaerae", "__Phycisphaerales", "__Phycisphaeraceae",
"__Urania-1B-19_marine_sediment_group"]}},{"id": "denovo8396",
"metadata": {"taxonomy": ["Bacteria", "__Proteobacteria",
"__Gammaproteobacteria", "__Order_Incertae_Sedis",
"__Family_Incertae_Sedis", "__Marinicella"]}},{"id": "denovo8398",
"metadata": {"taxonomy": ["Bacteria", "__Proteobacteria",
"__Gammaproteobacteria", "__Oceanospirillales", "__Halomonadaceae",
"__Cobetia"]}},{"id": "denovo8400", "metadata": {"taxonomy":
["Bacteria", "__Proteobacteria", "__Gammaproteobacteria",
"__Vibrionales", "__Vibrionaceae", "__Vibrio"]}},{"id": "denovo8404",
"metadata": {"taxonomy": ["Bacteria", "__Proteobacteria",
"__Deltaproteobacteria", "__Desulfobacterales",
"__Desulfobacteraceae", "__Desulfofaba"]}},{"id": "denovo8408",
"metadata": {"taxonomy": ["Bacteria", "__Spirochaetes",
"__Spirochaetes", "__Spirochaetales", "__Spirochaetaceae",
"__Spirochaeta"]}},{"id": "denovo8414", "metadata": {"taxonomy":
["Bacteria", "__Proteobacteria", "__Gammaproteobacteria",
"__E01-9C-26_marine_group", "__f", "__g"]}},{"id": "denovo8415",

"metadata": {"taxonomy": ["Bacteria", "__Proteobacteria",
"__Gammaproteobacteria", "__Alteromonadales",
"__Pseudoalteromonadaceae", "__Algicola"]}},{"id": "denovo8417",
"metadata": {"taxonomy": ["Bacteria", "__Proteobacteria",
"__Deltaproteobacteria", "__Sh765B-TzT-29", "__f", "__g"]}},{"id":
"denovo8419", "metadata": {"taxonomy": ["Bacteria",
"__Proteobacteria", "__Deltaproteobacteria", "__Myxococcales",
"__Haliangiaceae", "__Haliangium"]}},{"id": "denovo8420", "metadata":
{"taxonomy": ["Bacteria", "__Proteobacteria", "__Alphaproteobacteria",
"__Rhodobacterales", "__Rhodobacteraceae"]}},{"id": "denovo8423",
"metadata": {"taxonomy": ["Bacteria", "__Proteobacteria",
"__Gammaproteobacteria", "__Chromatiales", "__Chromatiaceae",
"__Nitrosococcus"]}},{"id": "denovo8425", "metadata": {"taxonomy":
["Bacteria", "__Proteobacteria"]}},{"id": "denovo8428", "metadata":
{"taxonomy": ["Bacteria", "__Proteobacteria", "__Gammaproteobacteria",
"__Chromatiales", "__Chromatiaceae", "__Nitrosococcus"]}},{"id":
"denovo8430", "metadata": {"taxonomy": ["Bacteria",
"__Proteobacteria", "__Deltaproteobacteria", "__Desulfobacterales",
"__Desulfobacteraceae", "__Desulfofaba"]}},{"id": "denovo8433",
"metadata": {"taxonomy": ["Bacteria", "__Proteobacteria",
"__Alphaproteobacteria", "__Sphingomonadales", "__ctg-CGOF202",
"__g"]}},{"id": "denovo8434", "metadata": {"taxonomy": ["Bacteria",
"__Proteobacteria", "__Alphaproteobacteria", "__Rhodospirillales",
"__Rhodospirillaceae", "__g"]}},{"id": "denovo8438", "metadata":
{"taxonomy": ["Bacteria", "__Proteobacteria", "__Gammaproteobacteria",
"__EC3", "__f", "__g"]}},{"id": "denovo8441", "metadata": {"taxonomy":
["Bacteria", "__Cyanobacteria", "__Cyanobacteria", "__SubsectionIV",
"__FamilyI", "__Cylindrospermum"]}},{"id": "denovo8445", "metadata":
{"taxonomy": ["Bacteria", "__Proteobacteria", "__Gammaproteobacteria",
"__Xanthomonadales", "__Xanthomonadaceae", "__Stenotrophomonas"]}},
{"id": "denovo8446", "metadata": {"taxonomy": ["Bacteria",
"__Proteobacteria", "__Betaproteobacteria"]}},{"id": "denovo8450",
"metadata": {"taxonomy": ["Bacteria", "__Proteobacteria",
"__Alphaproteobacteria", "__SAR11_clade", "__Surface_2", "__g"]}},
{"id": "denovo8458", "metadata": {"taxonomy": ["Bacteria",
"__Proteobacteria", "__Gammaproteobacteria", "__NKB5", "__f",
"__g"]}},{"id": "denovo8459", "metadata": {"taxonomy": ["Bacteria",
"__Proteobacteria", "__Gammaproteobacteria"]}},{"id": "denovo8463",
"metadata": {"taxonomy": ["Bacteria", "__Actinobacteria",
"__Acidimicrobiia", "__Acidimicrobiales", "__OCS155_marine_group",
"__g"]}},{"id": "denovo8467", "metadata": {"taxonomy": ["Bacteria",
"__Proteobacteria", "__Gammaproteobacteria", "__Chromatiales",
"__Granulosicoccaceae", "__Granulosicoccus"]}},{"id": "denovo8468",
"metadata": {"taxonomy": ["Bacteria", "__Proteobacteria",
"__Deltaproteobacteria", "__Desulfobacterales",
"__Desulfobacteraceae"]}},{"id": "denovo8469", "metadata":
{"taxonomy": ["Bacteria", "__Proteobacteria", "__Deltaproteobacteria",
"__Desulfobacterales", "__Nitrospinaceae", "__Nitrospina"]}},{"id":
"denovo8471", "metadata": {"taxonomy": ["Bacteria",
"__Proteobacteria", "__Alphaproteobacteria", "__SAR11_clade",

"__Surface_2", "__g"]}},{"id": "denovo8472", "metadata": {"taxonomy": ["Bacteria", "__Proteobacteria", "__Alphaproteobacteria", "__DB1-14", "__f", "__g"]}},{"id": "denovo8475", "metadata": {"taxonomy": ["Bacteria", "__Proteobacteria", "__Alphaproteobacteria", "__Rhodobacterales", "__Rhodobacteraceae", "__Maritimibacter"]}}, {"id": "denovo8477", "metadata": {"taxonomy": ["Bacteria", "__Bacteroidetes", "__Sphingobacteriia", "__Sphingobacteriales", "__Saprospiraceae", "__Lewinella"]}},{"id": "denovo8478", "metadata": {"taxonomy": ["Bacteria", "__Acidobacteria", "__RB25", "__o", "__f", "__g"]}},{"id": "denovo8479", "metadata": {"taxonomy": ["Bacteria", "__Firmicutes", "__Clostridia", "__Clostridiales"]}},{"id": "denovo8484", "metadata": {"taxonomy": ["Bacteria", "__Proteobacteria", "__Deltaproteobacteria", "__Desulfuromonadales", "__GR-WP33-58", "__g"]}},{"id": "denovo8486", "metadata": {"taxonomy": ["Bacteria", "__Planctomycetes", "__Phycisphaerae", "__Phycisphaerales", "__Phycisphaeraceae", "__Phycisphaera"]}},{"id": "denovo8487", "metadata": {"taxonomy": ["Bacteria", "__Proteobacteria", "__Alphaproteobacteria", "__Rickettsiales", "__Rickettsiaceae", "__Rickettsia"]}},{"id": "denovo8488", "metadata": {"taxonomy": ["Bacteria", "__Actinobacteria"]}},{"id": "denovo8497", "metadata": {"taxonomy": ["Bacteria", "__Proteobacteria", "__Deltaproteobacteria", "__Myxococcales", "__Nannocystaceae", "__g"]}},{"id": "denovo8498", "metadata": {"taxonomy": ["Bacteria", "__TM6", "__c", "__o", "__f", "__g"]}},{"id": "denovo8501", "metadata": {"taxonomy": ["Bacteria", "__Proteobacteria", "__Alphaproteobacteria", "__Sphingomonadales", "__Sphingomonadaceae"]}},{"id": "denovo8507", "metadata": {"taxonomy": ["Bacteria", "__Proteobacteria", "__Gammaproteobacteria", "__KI89A_clade", "__f", "__g"]}},{"id": "denovo8510", "metadata": {"taxonomy": ["Bacteria", "__Bacteroidetes", "__Flavobacteria", "__Flavobacteriales", "__Flavobacteriaceae", "__Aquimarina"]}},{"id": "denovo8512", "metadata": {"taxonomy": ["Bacteria", "__Proteobacteria", "__Gammaproteobacteria", "__Pseudomonadales", "__Pseudomonadaceae", "__Pseudomonas"]}},{"id": "denovo8516", "metadata": {"taxonomy": ["Bacteria", "__Proteobacteria", "__Alphaproteobacteria", "__Rhizobiales", "__Hyphomicrobiaceae", "__Hyphomicrobium"]}},{"id": "denovo8524", "metadata": {"taxonomy": ["Bacteria", "__Proteobacteria", "__Alphaproteobacteria", "__Rhodobacterales", "__Rhodobacteraceae"]}},{"id": "denovo8530", "metadata": {"taxonomy": ["Bacteria", "__Proteobacteria", "__Gammaproteobacteria", "__Alteromonadales", "__Ferrimonadaceae", "__Ferrimonas"]}},{"id": "denovo8531", "metadata": {"taxonomy": ["Bacteria", "__Firmicutes", "__Clostridia", "__Clostridiales", "__Christensenellaceae"]}},{"id": "denovo8532", "metadata": {"taxonomy": ["Bacteria", "__Bacteroidetes", "__Flavobacteria", "__Flavobacteriales", "__Flavobacteriaceae", "__Tenacibaculum"]}}, {"id": "denovo8539", "metadata": {"taxonomy": ["Bacteria", "__Proteobacteria", "__Alphaproteobacteria", "__Rhodospirillales", "__Rhodospirillaceae", "__g"]}},{"id": "denovo8540", "metadata": {"taxonomy": ["Bacteria", "__Proteobacteria", "__Gammaproteobacteria",

"__NKB5", "__f", "__g"]}},{"id": "denovo8541", "metadata":
{"taxonomy": ["Bacteria", "__TM6", "__c", "__o", "__f", "__g"]}},
{"id": "denovo8550", "metadata": {"taxonomy": ["Bacteria",
"__Proteobacteria", "__Gammaproteobacteria", "__NKB5", "__f",
"__g"]}},{"id": "denovo8551", "metadata": {"taxonomy": ["Bacteria",
"__Proteobacteria", "__Gammaproteobacteria", "__Alteromonadales",
"__Alteromonadaceae", "__BD2-7"]}},{"id": "denovo8552", "metadata":
{"taxonomy": ["Bacteria", "__Proteobacteria", "__Gammaproteobacteria",
"__E01-9C-26_marine_group", "__f", "__g"]}},{"id": "denovo8553",
"metadata": {"taxonomy": ["Bacteria", "__Verrucomicrobia",
"__Opitutae", "__Puniceicoccales", "__Puniceicoccaceae",
"__Pelagicoccus"]}},{"id": "denovo8558", "metadata": {"taxonomy":
["Bacteria", "__Proteobacteria", "__Alphaproteobacteria",
"__SAR11_clade", "__Surface_1", "__g"]}},{"id": "denovo8562",
"metadata": {"taxonomy": ["Bacteria", "__BD1-5", "__c", "__o", "__f",
"__g"]}},{"id": "denovo8563", "metadata": {"taxonomy": ["Bacteria",
"__Deferribacteres", "__Deferribacteres", "__Deferribacterales",
"__PAUC34f", "__g"]}},{"id": "denovo8564", "metadata": {"taxonomy":
["Bacteria", "__Proteobacteria", "__Alphaproteobacteria",
"__Rhodobacterales", "__Rhodobacteraceae", "__Shimia"]}},{"id":
"denovo8573", "metadata": {"taxonomy": ["Bacteria", "__Firmicutes",
"__Bacilli", "__Bacillales", "__Bacillaceae", "__Bacillus"]}},{"id":
"denovo8575", "metadata": {"taxonomy": ["Bacteria",
"__Proteobacteria", "__Alphaproteobacteria", "__Rhodospirillales",
"__Rhodospirillaceae", "__Pelagibius"]}},{"id": "denovo8579",
"metadata": {"taxonomy": ["Bacteria", "__Proteobacteria",
"__Alphaproteobacteria", "__Rhodobacterales", "__Rhodobacteraceae"]}},
{"id": "denovo8581", "metadata": {"taxonomy": ["Bacteria",
"__Actinobacteria", "__Actinobacteria", "__Micrococcales",
"__Micrococcaceae", "__Nesterenkonia"]}},{"id": "denovo8589",
"metadata": {"taxonomy": ["Bacteria", "__Proteobacteria",
"__Alphaproteobacteria", "__Rhodospirillales",
"__Candidatus_Alysiosphaera", "__g"]}},{"id": "denovo8593",
"metadata": {"taxonomy": ["Bacteria", "__Proteobacteria",
"__Alphaproteobacteria", "__Rhodospirillales", "__Rhodospirillaceae",
"__g"]}},{"id": "denovo8594", "metadata": {"taxonomy": ["Archaea",
"__Thaumarchaeota", "__Marine_Group_I", "__Candidatus_Nitrosopumilus",
"__f", "__g"]}},{"id": "denovo8595", "metadata": {"taxonomy":
["Bacteria", "__Proteobacteria", "__Alphaproteobacteria",
"__Rickettsiales", "__Rickettsiaceae", "__Rickettsia"]}},{"id":
"denovo8596", "metadata": {"taxonomy": ["Bacteria",
"__Proteobacteria", "__Gammaproteobacteria", "__NKB5", "__f",
"__g"]}},{"id": "denovo8606", "metadata": {"taxonomy": ["Bacteria",
"__Proteobacteria", "__Alphaproteobacteria", "__Rhodobacterales",
"__Rhodobacteraceae"]}},{"id": "denovo8609", "metadata": {"taxonomy":
["Bacteria", "__Proteobacteria", "__Deltaproteobacteria",
"__Desulfuromonadales", "__GR-WP33-58", "__g"]}},{"id": "denovo8611",
"metadata": {"taxonomy": ["Bacteria", "__Proteobacteria",
"__Gammaproteobacteria", "__Alteromonadales", "__Ferrimonadaceae",
"__Ferrimonas"]}},{"id": "denovo8615", "metadata": {"taxonomy":

["Bacteria", "__Actinobacteria", "__Actinobacteria",
"__Micrococcales", "__Microbacteriaceae", "__Candidatus_Aquiluna"]}},
{"id": "denovo8616", "metadata": {"taxonomy": ["Bacteria",
"__Proteobacteria", "__Alphaproteobacteria", "__Rhodobacterales",
"__Rhodobacteraceae"]}},{"id": "denovo8619", "metadata": {"taxonomy":
["Bacteria", "__Bacteroidetes", "__Flavobacteria",
"__Flavobacteriales", "__NS9_marine_group", "__g"]}},{"id":
"denovo8621", "metadata": {"taxonomy": ["Bacteria",
"__Proteobacteria", "__Gammaproteobacteria", "__Chromatiales",
"__Chromatiaceae", "__Nitrosococcus"]}},{"id": "denovo8622",
"metadata": {"taxonomy": ["Bacteria", "__Cyanobacteria", "__SHA-109",
"__o", "__f", "__g"]}},{"id": "denovo8623", "metadata": {"taxonomy":
["Bacteria", "__Bacteroidetes"]}},{"id": "denovo8629", "metadata":
{"taxonomy": ["Bacteria", "__Proteobacteria",
"__Gammaproteobacteria"]}},{"id": "denovo8631", "metadata":
{"taxonomy": ["Bacteria", "__Proteobacteria", "__Gammaproteobacteria",
"__Xanthomonadales", "__Sinobacteraceae",
"__JTB255_marine_benthic_group"]}},{"id": "denovo8632", "metadata":
{"taxonomy": ["Bacteria", "__Proteobacteria", "__Alphaproteobacteria",
"__Rhodospirillales", "__Rhodospirillaceae", "__Defluviicoccus"]}},
{"id": "denovo8633", "metadata": {"taxonomy": ["Bacteria",
"__Proteobacteria", "__Gammaproteobacteria", "__Oceanospirillales",
"__Oceanospirillaceae", "__Pseudospirillum"]}},{"id": "denovo8636",
"metadata": {"taxonomy": ["Bacteria", "__Proteobacteria",
"__Deltaproteobacteria", "__Myxococcales", "__0319-6G20", "__g"]}},
{"id": "denovo8644", "metadata": {"taxonomy": ["Bacteria",
"__Proteobacteria", "__Deltaproteobacteria", "__Desulfuromonadales",
"__Sva1033", "__g"]}},{"id": "denovo8646", "metadata": {"taxonomy":
["Bacteria", "__Proteobacteria", "__Alphaproteobacteria",
"__Rickettsiales", "__SAR116_clade", "__g"]}},{"id": "denovo8648",
"metadata": {"taxonomy": ["Bacteria", "__Verrucomicrobia",
"__Verrucomicrobiae", "__Verrucomicrobiales", "__DEV007", "__g"]}},
{"id": "denovo8651", "metadata": {"taxonomy": ["Bacteria",
"__Cyanobacteria", "__Cyanobacteria", "__SubsectionII", "__FamilyI",
"__Xenococcus"]}},{"id": "denovo8663", "metadata": {"taxonomy":
["Bacteria", "__Bacteroidetes", "__Flavobacteria",
"__Flavobacteriales"]}},{"id": "denovo8666", "metadata": {"taxonomy":
["Bacteria", "__Proteobacteria", "__Alphaproteobacteria",
"__Rhodospirillales", "__Rhodospirillaceae", "__Pelagibius"]}},{"id":
"denovo8672", "metadata": {"taxonomy": ["Bacteria",
"__Proteobacteria", "__Alphaproteobacteria", "__Rhodobacterales",
"__Rhodobacteraceae"]}},{"id": "denovo8678", "metadata": {"taxonomy":
["Bacteria", "__Proteobacteria", "__Alphaproteobacteria",
"__SAR11_clade", "__Surface_1", "__g"]}},{"id": "denovo8679",
"metadata": {"taxonomy": ["Bacteria", "__Chloroflexi", "__KD4-96",
"__o", "__f", "__g"]}},{"id": "denovo8683", "metadata": {"taxonomy":
["Bacteria", "__Proteobacteria", "__Betaproteobacteria",
"__Methylophilales", "__Methylophilaceae", "__Methylophilus"]}},{"id":
"denovo8686", "metadata": {"taxonomy": ["Bacteria", "__Firmicutes",
"__Clostridia", "__Clostridiales", "__Clostridiaceae",

"__Clostridium"]}},{"id": "denovo8687", "metadata": {"taxonomy": ["Bacteria", "__Proteobacteria", "__Alphaproteobacteria", "__Parvularculales", "__Parvularculaceae", "__Parvularcula"]}},{"id": "denovo8696", "metadata": {"taxonomy": ["Bacteria", "__Proteobacteria", "__Deltaproteobacteria", "__Desulfobacterales", "__Desulfobulbaceae", "__Desulfobulbus"]}},{"id": "denovo8698", "metadata": {"taxonomy": ["Bacteria", "__Proteobacteria", "__Alphaproteobacteria", "__Rhodospirillales", "__Rhodospirillaceae", "__Pelagibius"]}},{"id": "denovo8699", "metadata": {"taxonomy": ["Bacteria", "__Proteobacteria", "__Alphaproteobacteria", "__DB1-14", "__f", "__g"]}},{"id": "denovo8700", "metadata": {"taxonomy": ["Bacteria", "__Acidobacteria", "__Holophagae", "__Acanthopleuribacterales", "__Acanthopleuribacteraceae", "__Acanthopleuribacter"]}},{"id": "denovo8706", "metadata": {"taxonomy": ["Bacteria", "__Proteobacteria", "__Alphaproteobacteria", "__Parvularculales", "__Parvularculaceae", "__Parvularcula"]}},{"id": "denovo8707", "metadata": {"taxonomy": ["Bacteria", "__Proteobacteria", "__Deltaproteobacteria", "__Bdellovibrionales", "__Bdellovibrionaceae", "__OM27_clade"]}},{"id": "denovo8708", "metadata": {"taxonomy": ["Bacteria", "__Proteobacteria", "__Alphaproteobacteria", "__DB1-14", "__f", "__g"]}},{"id": "denovo8711", "metadata": {"taxonomy": ["Bacteria", "__Proteobacteria", "__Alphaproteobacteria", "__Sphingomonadales", "__Sphingomonadaceae", "__Novosphingobium"]}},{"id": "denovo8712", "metadata": {"taxonomy": ["Bacteria", "__Proteobacteria", "__Gammaproteobacteria", "__Pseudomonadales", "__Moraxellaceae", "__Acinetobacter"]}},{"id": "denovo8713", "metadata": {"taxonomy": ["Bacteria", "__Proteobacteria", "__Alphaproteobacteria", "__SAR11_clade", "__Surface_2", "__g"]}},{"id": "denovo8715", "metadata": {"taxonomy": ["Bacteria", "__Proteobacteria", "__Alphaproteobacteria", "__Rhizobiales", "__Rhodobiaceae", "__Rhodobium"]}},{"id": "denovo8719", "metadata": {"taxonomy": ["Bacteria", "__Proteobacteria", "__Alphaproteobacteria", "__Rhodobacterales", "__Rhodobacteraceae"]}},{"id": "denovo8721", "metadata": {"taxonomy": ["Bacteria", "__Chloroflexi", "__TK10", "__o", "__f", "__g"]}},{"id": "denovo8725", "metadata": {"taxonomy": ["Bacteria", "__Proteobacteria", "__Alphaproteobacteria", "__Rhodospirillales", "__Rhodospirillaceae", "__g"]}},{"id": "denovo8727", "metadata": {"taxonomy": ["Bacteria", "__Proteobacteria", "__Gammaproteobacteria", "__Pseudomonadales", "__Moraxellaceae", "__Acinetobacter"]}},{"id": "denovo8728", "metadata": {"taxonomy": ["Bacteria", "__Cyanobacteria", "__ML635J-21", "__o", "__f", "__g"]}},{"id": "denovo8732", "metadata": {"taxonomy": ["Bacteria", "__Proteobacteria", "__Deltaproteobacteria"]}},{"id": "denovo8734", "metadata": {"taxonomy": ["Bacteria", "__Proteobacteria", "__Alphaproteobacteria", "__Rhizobiales", "__Rhodobiaceae", "__Rhodobium"]}},{"id": "denovo8735", "metadata": {"taxonomy": ["Bacteria", "__Proteobacteria", "__Alphaproteobacteria", "__Rhodobacterales", "__Rhodobacteraceae"]}},{"id": "denovo8737", "metadata": {"taxonomy":

["Bacteria", "__Bacteroidetes", "__Cytophagia", "__Cytophagales", "__Flammeovirgaceae", "__Ekhidna"]}},{"id": "denovo8739", "metadata": {"taxonomy": ["Bacteria", "__Acidobacteria", "__Holophagae", "__Acanthopleuribacterales", "__Acanthopleuribacteraceae", "__Acanthopleuribacter"]}},{"id": "denovo8740", "metadata": {"taxonomy": ["Bacteria", "__Proteobacteria", "__Alphaproteobacteria", "__Rhodobacterales", "__Rhodobacteraceae"]}},{"id": "denovo8742", "metadata": {"taxonomy": ["Bacteria", "__Planctomycetes", "__Planctomycetacia", "__Planctomycetales", "__Planctomycetaceae", "__Planctomyces"]}},{"id": "denovo8744", "metadata": {"taxonomy": ["Bacteria", "__Proteobacteria", "__Alphaproteobacteria", "__DB1-14", "__f", "__g"]}},{"id": "denovo8745", "metadata": {"taxonomy": ["Bacteria", "__Proteobacteria"]}},{"id": "denovo8748", "metadata": {"taxonomy": ["Bacteria", "__Proteobacteria", "__Gammaproteobacteria", "__Thiotrichales", "__Piscirickettsiaceae", "__Piscirickettsia"]}},{"id": "denovo8755", "metadata": {"taxonomy": ["Bacteria", "__TM6", "__c", "__o", "__f", "__g"]}},{"id": "denovo8762", "metadata": {"taxonomy": ["Bacteria", "__Proteobacteria", "__Gammaproteobacteria", "__KI89A_clade", "__f", "__g"]}},{"id": "denovo8763", "metadata": {"taxonomy": ["Bacteria", "__Proteobacteria", "__Alphaproteobacteria", "__Rickettsiales", "__LWSR-14", "__g"]}},{"id": "denovo8764", "metadata": {"taxonomy": ["Bacteria", "__Bacteroidetes", "__Sphingobacteriia", "__Sphingobacteriales", "__WCHB1-69", "__g"]}},{"id": "denovo8765", "metadata": {"taxonomy": ["Bacteria", "__Proteobacteria", "__Deltaproteobacteria", "__Desulfobacterales", "__Desulfobulbaceae", "__Desulfotalea"]}},{"id": "denovo8766", "metadata": {"taxonomy": ["Bacteria", "__Acidobacteria", "__Holophagae", "__NS72", "__f", "__g"]}},{"id": "denovo8768", "metadata": {"taxonomy": ["Bacteria", "__Bacteroidetes", "__Cytophagia", "__Cytophagales", "__Flammeovirgaceae", "__g"]}},{"id": "denovo8770", "metadata": {"taxonomy": ["Bacteria", "__Cyanobacteria", "__Cyanobacteria", "__SubsectionIII", "__FamilyI"]}},{"id": "denovo8781", "metadata": {"taxonomy": ["Bacteria", "__Proteobacteria", "__Gammaproteobacteria"]}},{"id": "denovo8782", "metadata": {"taxonomy": ["Bacteria", "__Proteobacteria", "__Gammaproteobacteria", "__Pseudomonadales", "__Moraxellaceae", "__Acinetobacter"]}},{"id": "denovo8785", "metadata": {"taxonomy": ["Bacteria", "__Proteobacteria", "__Gammaproteobacteria", "__Oceanospirillales", "__Hahellaceae", "__Hahella"]}},{"id": "denovo8789", "metadata": {"taxonomy": ["Bacteria", "__Proteobacteria", "__Betaproteobacteria", "__Burkholderiales", "__Comamonadaceae"]}},{"id": "denovo8790", "metadata": {"taxonomy": ["Bacteria", "__Proteobacteria", "__Gammaproteobacteria", "__Oceanospirillales", "__Oceanospirillaceae", "__Reinekea"]}},{"id": "denovo8792", "metadata": {"taxonomy": ["Bacteria", "__Proteobacteria", "__Gammaproteobacteria", "__Chromatiales", "__Chromatiaceae", "__Nitrosococcus"]}},{"id": "denovo8796", "metadata": {"taxonomy": ["Bacteria", "__Proteobacteria", "__Deltaproteobacteria", "__Myxococcales", "__Nannocystineae", "__g"]}},{"id": "denovo8798",

"metadata": {"taxonomy": ["Bacteria", "__Proteobacteria", "__Alphaproteobacteria", "__Rickettsiales", "__Family_Incertae_Sedis", "__Caedibacter"]}},{"id": "denovo8800", "metadata": {"taxonomy": ["Bacteria", "__Proteobacteria", "__Gammaproteobacteria", "__KI89A_clade", "__f", "__g"]}},{"id": "denovo8802", "metadata": {"taxonomy": ["Bacteria", "__Proteobacteria", "__Alphaproteobacteria", "__Rhizobiales", "__1174-901-12", "__g"]}},{"id": "denovo8803", "metadata": {"taxonomy": ["Bacteria", "__Proteobacteria", "__Gammaproteobacteria", "__Xanthomonadales", "__Sinobacteraceae", "__JTB255_marine_benthic_group"]}},{"id": "denovo8806", "metadata": {"taxonomy": ["Bacteria", "__Firmicutes", "__Clostridia", "__Clostridiales", "__Lachnospiraceae", "__g"]}},{"id": "denovo8807", "metadata": {"taxonomy": ["Bacteria", "__Proteobacteria", "__Alphaproteobacteria", "__DB1-14", "__f", "__g"]}},{"id": "denovo8809", "metadata": {"taxonomy": ["Bacteria", "__Bacteroidetes", "__Flavobacteria", "__Flavobacteriales", "__Flavobacteriaceae", "__g"]}},{"id": "denovo8811", "metadata": {"taxonomy": ["Bacteria", "__Proteobacteria", "__Gammaproteobacteria", "__Alteromonadales", "__Alteromonadaceae"]}},{"id": "denovo8812", "metadata": {"taxonomy": ["Bacteria", "__Proteobacteria", "__Deltaproteobacteria", "__GR-WP33-30", "__f", "__g"]}},{"id": "denovo8815", "metadata": {"taxonomy": ["Bacteria", "__Proteobacteria", "__Deltaproteobacteria", "__SAR324_clade(Marine_group_B)", "__f", "__g"]}},{"id": "denovo8821", "metadata": {"taxonomy": ["Bacteria", "__Chlorobi", "__Chlorobia", "__Chlorobiales", "__Chlorobiaceae", "__Prosthecochloris"]}},{"id": "denovo8822", "metadata": {"taxonomy": ["Bacteria", "__Proteobacteria", "__Alphaproteobacteria", "__Rhizobiales", "__Hyphomicrobiaceae", "__Devosia"]}},{"id": "denovo8825", "metadata": {"taxonomy": ["Bacteria", "__Proteobacteria", "__TA18", "__o", "__f", "__g"]}},{"id": "denovo8826", "metadata": {"taxonomy": ["Bacteria", "__Bacteroidetes", "__Cytophagia", "__Cytophagales", "__Flammeovirgaceae", "__Flexithrix"]}},{"id": "denovo8831", "metadata": {"taxonomy": ["Bacteria", "__Chloroflexi", "__Chloroflexi", "__Chloroflexales", "__Chloroflexaceae", "__Roseiflexus"]}},{"id": "denovo8832", "metadata": {"taxonomy": ["Bacteria", "__Proteobacteria", "__Alphaproteobacteria"]}},{"id": "denovo8833", "metadata": {"taxonomy": ["Bacteria", "__Proteobacteria", "__Alphaproteobacteria", "__Rhodobacterales", "__Rhodobacteraceae", "__g"]}},{"id": "denovo8835", "metadata": {"taxonomy": ["Bacteria", "__Proteobacteria", "__Alphaproteobacteria", "__Rhodobacterales", "__Rhodobacteraceae"]}},{"id": "denovo8836", "metadata": {"taxonomy": ["Bacteria", "__Chloroflexi", "__SAR202_clade", "__o", "__f", "__g"]}},{"id": "denovo8839", "metadata": {"taxonomy": ["Bacteria", "__Verrucomicrobia", "__Verrucomicrobiae", "__Verrucomicrobiales", "__Rubritaleaceae", "__Rubritalea"]}},{"id": "denovo8840", "metadata": {"taxonomy": ["Bacteria", "__Proteobacteria", "__Alphaproteobacteria", "__Rhodospirillales", "__Rhodospirillaceae", "__g"]}},{"id": "denovo8841", "metadata": {"taxonomy": ["Bacteria", "__Bacteroidetes", "__Flavobacteria", "__Flavobacteriales", "__Flavobacteriaceae",

"__Polaribacter"]}},{"id": "denovo8844", "metadata": {"taxonomy": ["Bacteria", "__Cyanobacteria", "__Cyanobacteria"]}},{"id": "denovo8847", "metadata": {"taxonomy": ["Bacteria", "__Proteobacteria", "__Gammaproteobacteria", "__Enterobacteriales", "__Enterobacteriaceae"]}},{"id": "denovo8856", "metadata": {"taxonomy": ["Bacteria", "__Bacteroidetes", "__Sphingobacteriia", "__Sphingobacteriales", "__Saprospiraceae", "__g"]}},{"id": "denovo8858", "metadata": {"taxonomy": ["Bacteria", "__Cyanobacteria", "__Cyanobacteria", "__SubsectionIII", "__FamilyI"]}},{"id": "denovo8859", "metadata": {"taxonomy": ["Bacteria", "__Proteobacteria", "__Alphaproteobacteria", "__Rhodospirillales", "__Candidatus_Alysiosphaera", "__g"]}},{"id": "denovo8860", "metadata": {"taxonomy": ["Bacteria", "__Acidobacteria", "__RB25", "__o", "__f", "__g"]}},{"id": "denovo8864", "metadata": {"taxonomy": ["Bacteria", "__Proteobacteria", "__Alphaproteobacteria", "__Rhodobacterales", "__Rhodobacteraceae", "__Roseobacter_clade_OCT_lineage"]}},{"id": "denovo8865", "metadata": {"taxonomy": ["Archaea", "__Euryarchaeota", "__Halobacteria", "__Halobacteriales", "__Deep_Sea_Hydrothermal_Vent_Gp_6(DHVEG-6)", "__g"]}},{"id": "denovo8867", "metadata": {"taxonomy": ["Bacteria", "__Bacteroidetes", "__Cytophagia", "__Cytophagales", "__Flammeovirgaceae", "__g"]}},{"id": "denovo8868", "metadata": {"taxonomy": ["Bacteria", "__Proteobacteria", "__Gammaproteobacteria", "__Xanthomonadales", "__Sinobacteraceae", "__JTB255_marine_benthic_group"]}},{"id": "denovo8869", "metadata": {"taxonomy": ["Bacteria", "__Proteobacteria", "__Deltaproteobacteria", "__Myxococcales", "__Nannocystaceae", "__g"]}},{"id": "denovo8871", "metadata": {"taxonomy": ["Bacteria", "__Chloroflexi", "__Caldilineae", "__Caldilineales", "__Caldilineaceae", "__g"]}},{"id": "denovo8874", "metadata": {"taxonomy": ["Bacteria", "__Proteobacteria", "__Gammaproteobacteria", "__Alteromonadales"]}},{"id": "denovo8882", "metadata": {"taxonomy": ["Bacteria", "__Proteobacteria", "__Gammaproteobacteria", "__Chromatiales", "__Chromatiaceae", "__Nitrosococcus"]}},{"id": "denovo8884", "metadata": {"taxonomy": ["Bacteria", "__Proteobacteria", "__Deltaproteobacteria", "__Myxococcales", "__0319-6G20", "__g"]}},{"id": "denovo8885", "metadata": {"taxonomy": ["Bacteria", "__Proteobacteria", "__SPOTSOCT00m83", "__o", "__f", "__g"]}},{"id": "denovo8886", "metadata": {"taxonomy": ["Bacteria", "__Bacteroidetes", "__Flavobacteria", "__Flavobacteriales", "__Flavobacteriaceae", "__Chryseobacterium"]}},{"id": "denovo8892", "metadata": {"taxonomy": ["Bacteria", "__Acidobacteria", "__RB25", "__o", "__f", "__g"]}},{"id": "denovo8896", "metadata": {"taxonomy": ["Bacteria", "__Proteobacteria", "__Deltaproteobacteria", "__Myxococcales", "__Haliangiaceae", "__Haliangium"]}},{"id": "denovo8897", "metadata": {"taxonomy": ["Bacteria", "__Proteobacteria", "__Alphaproteobacteria"]}},{"id": "denovo8898", "metadata": {"taxonomy": ["Bacteria", "__Proteobacteria", "__Gammaproteobacteria", "__Oceanospirillales", "__SAR86_clade", "__g"]}},{"id": "denovo8901", "metadata": {"taxonomy": ["Bacteria", "__Proteobacteria",

"__Gammaproteobacteria", "__Order_Incertae_Sedis",
"__Family_Incertae_Sedis", "__Sedimenticola"]}},{"id": "denovo8902",
"metadata": {"taxonomy": ["Bacteria", "__Proteobacteria",
"__Alphaproteobacteria", "__Rickettsiales", "__Rickettsiaceae",
"__g"]}},{"id": "denovo8904", "metadata": {"taxonomy": ["Bacteria",
"__Proteobacteria", "__Deltaproteobacteria", "__Bdellovibrionales",
"__Bdellovibrionaceae", "__Bdellovibrio"]}},{"id": "denovo8905",
"metadata": {"taxonomy": ["Bacteria", "__Bacteroidetes",
"__Sphingobacteriia", "__Sphingobacteriales", "__Saprospiraceae",
"__g"]}},{"id": "denovo8907", "metadata": {"taxonomy": ["Bacteria",
"__Firmicutes", "__Clostridia", "__Halanaerobiales", "__64K2",
"__g"]}},{"id": "denovo8908", "metadata": {"taxonomy": ["Bacteria",
"__Proteobacteria", "__Gammaproteobacteria"]}},{"id": "denovo8909",
"metadata": {"taxonomy": ["Bacteria", "__Proteobacteria",
"__Gammaproteobacteria"]}},{"id": "denovo8910", "metadata":
{"taxonomy": ["Bacteria", "__Bacteroidetes", "__Sphingobacteriia",
"__Sphingobacteriales", "__Saprospiraceae", "__g"]}},{"id":
"denovo8913", "metadata": {"taxonomy": ["Bacteria",
"__Proteobacteria", "__Gammaproteobacteria"]}},{"id": "denovo8915",
"metadata": {"taxonomy": ["Bacteria", "__Proteobacteria",
"__Alphaproteobacteria", "__Parvularculales", "__Parvularculaceae",
"__Parvularcula"]}},{"id": "denovo8916", "metadata": {"taxonomy":
["Bacteria", "__Proteobacteria", "__Alphaproteobacteria",
"__Rickettsiales", "__SM2D12", "__g"]}},{"id": "denovo8918",
"metadata": {"taxonomy": ["Bacteria", "__Proteobacteria",
"__Gammaproteobacteria", "__Legionellales", "__Coxiellaceae",
"__Coxiella"]}},{"id": "denovo8923", "metadata": {"taxonomy":
["Bacteria", "__Proteobacteria", "__Alphaproteobacteria", "__MNG3",
"__f", "__g"]}},{"id": "denovo8927", "metadata": {"taxonomy":
["Bacteria", "__Proteobacteria", "__Alphaproteobacteria",
"__Rhodospirillales", "__Rhodospirillaceae", "__Defluviicoccus"]}},
{"id": "denovo8928", "metadata": {"taxonomy": ["Bacteria",
"__Proteobacteria", "__Epsilonproteobacteria", "__Campylobacterales",
"__Campylobacteraceae", "__Arcobacter"]}},{"id": "denovo8929",
"metadata": {"taxonomy": ["Bacteria", "__Proteobacteria",
"__Alphaproteobacteria", "__Rhodobacterales", "__Rhodobacteraceae"]}},
{"id": "denovo8931", "metadata": {"taxonomy": ["Bacteria",
"__Planctomycetes", "__vadinHA49", "__o", "__f", "__g"]}},{"id":
"denovo8932", "metadata": {"taxonomy": ["Bacteria", "__Bacteroidetes",
"__Sphingobacteriia", "__Sphingobacteriales", "__Saprospiraceae",
"__g"]}},{"id": "denovo8933", "metadata": {"taxonomy": ["Bacteria",
"__Proteobacteria", "__Gammaproteobacteria", "__Pseudomonadales",
"__Moraxellaceae", "__Acinetobacter"]}},{"id": "denovo8936",
"metadata": {"taxonomy": ["Bacteria", "__Bacteroidetes",
"__Flavobacteria", "__Flavobacteriales", "__Cryomorphaceae",
"__Owenweeksia"]}},{"id": "denovo8941", "metadata": {"taxonomy":
["Bacteria", "__Proteobacteria", "__Gammaproteobacteria",
"__Enterobacteriales", "__Enterobacteriaceae"]}},{"id": "denovo8943",
"metadata": {"taxonomy": ["Bacteria", "__Proteobacteria",
"__Alphaproteobacteria", "__Rhodospirillales", "__Rhodospirillaceae",

"__g"]}},{"id": "denovo8944", "metadata": {"taxonomy": ["Bacteria", "__Proteobacteria", "__Alphaproteobacteria", "__Rhodobacterales", "__Rhodobacteraceae", "__Ruegeria"]}},{"id": "denovo8946", "metadata": {"taxonomy": ["Bacteria", "__Proteobacteria", "__Gammaproteobacteria", "__Pseudomonadales", "__Moraxellaceae", "__Acinetobacter"]}},{"id": "denovo8948", "metadata": {"taxonomy": ["Bacteria", "__Proteobacteria", "__Gammaproteobacteria", "__Oceanospirillales", "__Hahellaceae", "__Endozoicomonas"]}},{"id": "denovo8954", "metadata": {"taxonomy": ["Bacteria", "__Proteobacteria", "__Alphaproteobacteria", "__Rhodospirillales", "__Rhodospirillaceae", "__Pelagibius"]}},{"id": "denovo8956", "metadata": {"taxonomy": ["Bacteria", "__Proteobacteria", "__Alphaproteobacteria", "__Rhodobacterales", "__Rhodobacteraceae"]}},{"id": "denovo8957", "metadata": {"taxonomy": ["Bacteria", "__Chloroflexi", "__Thermomicrobia", "__AKYG1722", "__f", "__g"]}},{"id": "denovo8960", "metadata": {"taxonomy": ["Bacteria", "__Bacteroidetes", "__Cytophagia", "__Cytophagales", "__Flammeovirgaceae"]}},{"id": "denovo8961", "metadata": {"taxonomy": ["Bacteria", "__Proteobacteria", "__Deltaproteobacteria", "__Myxococcales", "__Sandaracinaceae", "__Sandaracinus"]}},{"id": "denovo8970", "metadata": {"taxonomy": ["Bacteria", "__Bacteroidetes", "__Cytophagia", "__Cytophagales", "__Cytophagaceae", "__Meniscus"]}},{"id": "denovo8972", "metadata": {"taxonomy": ["Bacteria", "__Proteobacteria", "__Gammaproteobacteria", "__Alteromonadales", "__Pseudoalteromonadaceae", "__g"]}},{"id": "denovo8979", "metadata": {"taxonomy": ["Bacteria", "__Acidobacteria", "__Acidobacteria", "__BPC102", "__f", "__g"]}},{"id": "denovo8980", "metadata": {"taxonomy": ["Bacteria", "__Proteobacteria", "__Alphaproteobacteria", "__Rhodobacterales", "__Rhodobacteraceae"]}},{"id": "denovo8981", "metadata": {"taxonomy": ["Bacteria", "__Firmicutes", "__Clostridia", "__Clostridiales", "__Clostridiaceae", "__Anaerovirgula"]}},{"id": "denovo8982", "metadata": {"taxonomy": ["Bacteria", "__Actinobacteria", "__Acidimicrobiia", "__Acidimicrobiales", "__OCS155_marine_group", "__g"]}},{"id": "denovo8984", "metadata": {"taxonomy": ["Bacteria", "__Proteobacteria", "__Alphaproteobacteria", "__Rickettsiales", "__SM2D12", "__g"]}},{"id": "denovo8985", "metadata": {"taxonomy": ["Bacteria", "__Proteobacteria", "__Gammaproteobacteria", "__Oceanospirillales"]}},{"id": "denovo8986", "metadata": {"taxonomy": ["Bacteria", "__Spirochaetes", "__Spirochaetes", "__Spirochaetales", "__Spirochaetaceae", "__Spirochaeta"]}},{"id": "denovo8987", "metadata": {"taxonomy": ["Bacteria", "__Bacteroidetes", "__Cytophagia", "__Cytophagales", "__Flammeovirgaceae", "__Marinoscillum"]}},{"id": "denovo8990", "metadata": {"taxonomy": ["Bacteria", "__Proteobacteria", "__Gammaproteobacteria", "__Alteromonadales", "__Alteromonadaceae", "__Haliea"]}},{"id": "denovo8992", "metadata": {"taxonomy": ["Bacteria", "__Proteobacteria", "__Alphaproteobacteria", "__Rhodospirillales", "__Rhodospirillaceae"]}},{"id": "denovo8996", "metadata": {"taxonomy": ["Bacteria", "__Proteobacteria", "__Alphaproteobacteria", "__Rhizobiales", "__Rhodobiaceae",

"__Rhodobium"]}},{"id": "denovo8997", "metadata": {"taxonomy": ["Bacteria", "__Gemmatimonadetes", "__Gemmatimonadetes", "__BD2-11_terrestrial_group", "__f", "__g"]}},{"id": "denovo8998", "metadata": {"taxonomy": ["Bacteria", "__Bacteroidetes", "__Flavobacteria", "__Flavobacteriales", "__Flavobacteriaceae", "__NS4_marine_group"]}},{"id": "denovo8999", "metadata": {"taxonomy": ["Bacteria", "__Proteobacteria", "__Deltaproteobacteria", "__Desulfobacterales", "__Desulfobulbaceae", "__g"]}},{"id": "denovo9001", "metadata": {"taxonomy": ["Bacteria", "__Proteobacteria", "__Alphaproteobacteria", "__Rhizobiales", "__Phyllobacteriaceae", "__Cohaesibacter"]}},{"id": "denovo9002", "metadata": {"taxonomy": ["Bacteria", "__Proteobacteria", "__Alphaproteobacteria", "__Rhodospirillales", "__Rhodospirillaceae", "__g"]}},{"id": "denovo9004", "metadata": {"taxonomy": ["Bacteria", "__Bacteroidetes", "__Cytophagia", "__Order_II_Incertae_Sedis", "__Rhodothermaceae", "__g"]}},{"id": "denovo9005", "metadata": {"taxonomy": ["Bacteria", "__Elusimicrobia", "__Elusimicrobia", "__MVP-88", "__f", "__g"]}},{"id": "denovo9006", "metadata": {"taxonomy": ["Bacteria"]}},{"id": "denovo9013", "metadata": {"taxonomy": ["Bacteria", "__Bacteroidetes", "__Flavobacteria", "__Flavobacteriales", "__Flavobacteriaceae", "__NS5_marine_group"]}},{"id": "denovo9016", "metadata": {"taxonomy": ["Bacteria", "__Proteobacteria", "__Deltaproteobacteria", "__SAR324_clade(Marine_group_B)", "__f", "__g"]}},{"id": "denovo9017", "metadata": {"taxonomy": ["Bacteria", "__Proteobacteria", "__Alphaproteobacteria", "__Rhodobacterales", "__Rhodobacteraceae"]}},{"id": "denovo9021", "metadata": {"taxonomy": ["Bacteria", "__Proteobacteria", "__Gammaproteobacteria", "__Pseudomonadales", "__Moraxellaceae", "__Acinetobacter"]}},{"id": "denovo9022", "metadata": {"taxonomy": ["Bacteria", "__Proteobacteria", "__Deltaproteobacteria", "__Sh765B-TzT-29", "__f", "__g"]}},{"id": "denovo9026", "metadata": {"taxonomy": ["Bacteria", "__Chloroflexi", "__Anaerolineae", "__Anaerolineales", "__Anaerolineaceae", "__g"]}},{"id": "denovo9028", "metadata": {"taxonomy": ["Bacteria", "__Cyanobacteria", "__Cyanobacteria", "__SubsectionIII", "__FamilyI", "__g"]}},{"id": "denovo9029", "metadata": {"taxonomy": ["Bacteria", "__Bacteroidetes", "__Sphingobacteriia", "__Sphingobacteriales", "__Saprospiraceae", "__g"]}},{"id": "denovo9030", "metadata": {"taxonomy": ["Archaea", "__Euryarchaeota", "__Thermoplasmata", "__Thermoplasmatales", "__VC2.1_Arc6", "__g"]}},{"id": "denovo9035", "metadata": {"taxonomy": ["Bacteria", "__Bacteroidetes", "__Flavobacteria", "__Flavobacteriales", "__Flavobacteriaceae"]}},{"id": "denovo9037", "metadata": {"taxonomy": ["Bacteria", "__Chloroflexi", "__Anaerolineae", "__Anaerolineales", "__Anaerolineaceae", "__g"]}},{"id": "denovo9041", "metadata": {"taxonomy": ["Bacteria", "__Bacteroidetes", "__Sphingobacteriia", "__Sphingobacteriales", "__Saprospiraceae", "__g"]}},{"id": "denovo9044", "metadata": {"taxonomy": ["Bacteria", "__Proteobacteria", "__Alphaproteobacteria", "__Rhodobacterales", "__Rhodobacteraceae", "__Pseudovibrio"]}},{"id": "denovo9045",

"metadata": {"taxonomy": ["Bacteria", "__Proteobacteria", "__Deltaproteobacteria", "__Myxococcales", "__0319-6G20", "__g"]}}, {"id": "denovo9048", "metadata": {"taxonomy": ["Bacteria", "__Proteobacteria", "__Gammaproteobacteria", "__KI89A_clade", "__f", "__g"]}},{"id": "denovo9052", "metadata": {"taxonomy": ["Bacteria", "__Cyanobacteria", "__4C0d-2", "__o", "__f", "__g"]}},{"id": "denovo9057", "metadata": {"taxonomy": ["Bacteria", "__Bacteroidetes", "__Flavobacteria", "__Flavobacteriales", "__Flavobacteriaceae", "__Zeaxanthinibacter"]}},{"id": "denovo9060", "metadata": {"taxonomy": ["Bacteria", "__Actinobacteria", "__Acidimicrobiia", "__Acidimicrobiales", "__OCS155_marine_group", "__g"]}},{"id": "denovo9061", "metadata": {"taxonomy": ["Bacteria"]}},{"id": "denovo9062", "metadata": {"taxonomy": ["Bacteria", "__Proteobacteria", "__Gammaproteobacteria"]}},{"id": "denovo9063", "metadata": {"taxonomy": ["Bacteria", "__Proteobacteria", "__Alphaproteobacteria", "__Rhodospirillales", "__Rhodospirillaceae", "__g"]}},{"id": "denovo9064", "metadata": {"taxonomy": ["Bacteria", "__Chloroflexi", "__Caldilineae", "__Caldilineales", "__Caldilineaceae", "__Caldilinea"]}},{"id": "denovo9065", "metadata": {"taxonomy": ["Bacteria", "__Firmicutes", "__Clostridia", "__Halanaerobiales", "__Halobacteroidaceae", "__g"]}},{"id": "denovo9067", "metadata": {"taxonomy": ["Bacteria", "__Bacteroidetes", "__Sphingobacteriia", "__Sphingobacteriales", "__Saprospiraceae", "__Lewinella"]}},{"id": "denovo9071", "metadata": {"taxonomy": ["Bacteria", "__Bacteroidetes", "__Flavobacteria", "__Flavobacteriales", "__Cryomorphaceae", "__Owenweeksia"]}},{"id": "denovo9078", "metadata": {"taxonomy": ["Bacteria", "__Proteobacteria", "__Alphaproteobacteria", "__Rhodobacterales", "__Rhodobacteraceae"]}},{"id": "denovo9080", "metadata": {"taxonomy": ["Bacteria", "__Proteobacteria", "__Deltaproteobacteria", "__Sh765B-TzT-29", "__f", "__g"]}},{"id": "denovo9082", "metadata": {"taxonomy": ["Bacteria", "__Chloroflexi", "__Anaerolineae", "__Anaerolineales", "__Anaerolineaceae", "__g"]}},{"id": "denovo9086", "metadata": {"taxonomy": ["Bacteria", "__Cyanobacteria", "__Cyanobacteria"]}}, {"id": "denovo9090", "metadata": {"taxonomy": ["Bacteria", "__Proteobacteria", "__Gammaproteobacteria", "__Oceanospirillales", "__MBAE14", "__g"]}},{"id": "denovo9091", "metadata": {"taxonomy": ["Bacteria", "__Proteobacteria", "__Gammaproteobacteria", "__KI89A_clade", "__f", "__g"]}},{"id": "denovo9094", "metadata": {"taxonomy": ["Bacteria", "__Bacteroidetes", "__Cytophagia", "__Cytophagales", "__Flammeovirgaceae", "__g"]}},{"id": "denovo9095", "metadata": {"taxonomy": ["Bacteria", "__Candidate_division_BRC1", "__c", "__o", "__f", "__g"]}},{"id": "denovo9096", "metadata": {"taxonomy": ["Bacteria", "__Proteobacteria", "__Alphaproteobacteria", "__MNG3", "__f", "__g"]}},{"id": "denovo9097", "metadata": {"taxonomy": ["Bacteria", "__Proteobacteria", "__Gammaproteobacteria", "__Alteromonadales", "__Alteromonadaceae", "__Haliea"]}},{"id": "denovo9098", "metadata": {"taxonomy": ["Bacteria", "__Proteobacteria", "__Gammaproteobacteria", "__Alteromonadales", "__Alteromonadaceae", "__Melitea"]}},{"id": "denovo9101", "metadata":

{"taxonomy": ["Bacteria", "__Proteobacteria", "__Deltaproteobacteria", "__Bdellovibrionales", "__Bdellovibrionaceae", "__Bdellovibrio"]}}, {"id": "denovo9107", "metadata": {"taxonomy": ["Bacteria", "__Gemmatimonadetes", "__Gemmatimonadetes", "__BD2-11_terrestrial_group", "__f", "__g"]}},{"id": "denovo9108", "metadata": {"taxonomy": ["Bacteria", "__Spirochaetes", "__Spirochaetes", "__Spirochaetales"]}},{"id": "denovo9109", "metadata": {"taxonomy": ["Bacteria", "__Cyanobacteria", "__Cyanobacteria", "__SubsectionI", "__FamilyI", "__Acaryochloris"]}}, {"id": "denovo9110", "metadata": {"taxonomy": ["Bacteria", "__Proteobacteria", "__Alphaproteobacteria", "__Sphingomonadales", "__Sphingomonadaceae", "__Zymomonas"]}},{"id": "denovo9116", "metadata": {"taxonomy": ["Bacteria", "__Bacteroidetes", "__Sphingobacteriia", "__Sphingobacteriales", "__Chitinophagaceae", "__g"]}},{"id": "denovo9117", "metadata": {"taxonomy": ["Bacteria", "__Proteobacteria", "__Gammaproteobacteria", "__EC3", "__f", "__g"]}}, {"id": "denovo9119", "metadata": {"taxonomy": ["Bacteria", "__Proteobacteria", "__Gammaproteobacteria", "__Oceanospirillales", "__Oceanospirillaceae", "__Pseudospirillum"]}},{"id": "denovo9120", "metadata": {"taxonomy": ["Bacteria", "__Proteobacteria", "__Alphaproteobacteria", "__DB1-14", "__f", "__g"]}},{"id": "denovo9122", "metadata": {"taxonomy": ["Bacteria", "__Proteobacteria", "__Deltaproteobacteria", "__Sh765B-TzT-29", "__f", "__g"]}},{"id": "denovo9123", "metadata": {"taxonomy": ["Bacteria", "__Proteobacteria", "__Alphaproteobacteria", "__Rhodospirillales", "__Rhodospirillaceae", "__Thalassospira"]}},{"id": "denovo9124", "metadata": {"taxonomy": ["Bacteria", "__Proteobacteria", "__Alphaproteobacteria", "__Rhodobacterales", "__Rhodobacteraceae", "__Roseovarius"]}},{"id": "denovo9135", "metadata": {"taxonomy": ["Bacteria", "__Bacteroidetes", "__Flavobacteria", "__Flavobacteriales", "__Cryomorphaceae", "__NS7_marine_group"]}}, {"id": "denovo9138", "metadata": {"taxonomy": ["Bacteria", "__Bacteroidetes", "__Flavobacteria", "__Flavobacteriales", "__Cryomorphaceae", "__Owenweeksia"]}},{"id": "denovo9139", "metadata": {"taxonomy": ["Bacteria", "__Acidobacteria", "__Acidobacteria", "__BPC015", "__f", "__g"]}},{"id": "denovo9142", "metadata": {"taxonomy": ["Bacteria", "__Proteobacteria", "__Alphaproteobacteria", "__Sphingomonadales", "__Sphingomonadaceae", "__Sphingomonas"]}},{"id": "denovo9143", "metadata": {"taxonomy": ["Bacteria", "__Bacteroidetes", "__Flavobacteria", "__Flavobacteriales", "__NS9_marine_group", "__g"]}},{"id": "denovo9148", "metadata": {"taxonomy": ["Bacteria", "__Bacteroidetes", "__Flavobacteria", "__Flavobacteriales", "__Flavobacteriaceae", "__NS5_marine_group"]}},{"id": "denovo9150", "metadata": {"taxonomy": ["Bacteria", "__Proteobacteria", "__Gammaproteobacteria", "__Thiotrichales", "__Thiotrichaceae", "__Thiothrix"]}},{"id": "denovo9158", "metadata": {"taxonomy": ["Bacteria", "__Proteobacteria", "__Alphaproteobacteria", "__Rhodobacterales", "__Rhodobacteraceae", "__Maritimibacter"]}},{"id": "denovo9160", "metadata": {"taxonomy": ["Bacteria", "__Firmicutes", "__Clostridia",

"__Clostridiales", "__Peptococcaceae", "__g"]}},{"id": "denovo9162",
"metadata": {"taxonomy": ["Bacteria", "__Proteobacteria",
"__Alphaproteobacteria", "__Rhodobacterales", "__Rhodobacteraceae",
"__Jannaschia"]}},{"id": "denovo9164", "metadata": {"taxonomy":
["Bacteria", "__Spirochaetes", "__Spirochaetes", "__Spirochaetales",
"__Spirochaetaceae", "__Spirochaeta"]}},{"id": "denovo9166",
"metadata": {"taxonomy": ["Bacteria", "__Firmicutes",
"__Clostridia"]}},{"id": "denovo9169", "metadata": {"taxonomy":
["Bacteria", "__Proteobacteria", "__Gammaproteobacteria",
"__Legionellales", "__Legionellaceae"]}},{"id": "denovo9172",
"metadata": {"taxonomy": ["Bacteria", "__Proteobacteria",
"__Alphaproteobacteria", "__Sphingomonadales",
"__Sphingomonadaceae"]}},{"id": "denovo9176", "metadata": {"taxonomy":
["Bacteria", "__Proteobacteria", "__Gammaproteobacteria",
"__Pseudomonadales", "__Moraxellaceae", "__Acinetobacter"]}},{"id":
"denovo9177", "metadata": {"taxonomy": ["Bacteria", "__Cyanobacteria",
"__Cyanobacteria", "__SubsectionI", "__FamilyI", "__Synechococcus"]}},
{"id": "denovo9178", "metadata": {"taxonomy": ["Bacteria",
"__Proteobacteria", "__Gammaproteobacteria", "__Pseudomonadales",
"__Moraxellaceae", "__Acinetobacter"]}},{"id": "denovo9179",
"metadata": {"taxonomy": ["Bacteria", "__Candidate_division_OD1",
"__c", "__o", "__f", "__g"]}},{"id": "denovo9181", "metadata":
{"taxonomy": ["Bacteria", "__Bacteroidetes", "__Class_Incertae_Sedis",
"__Order_Incertae_Sedis", "__Family_Incertae_Sedis",
"__Prolixibacter"]}},{"id": "denovo9182", "metadata": {"taxonomy":
["Bacteria", "__Chlorobi", "__Chlorobia", "__Chlorobiales",
"__Chlorobiaceae", "__Prosthecochloris"]}},{"id": "denovo9183",
"metadata": {"taxonomy": ["Bacteria", "__Chloroflexi",
"__Caldilineae", "__Caldilineales", "__Caldilineaceae", "__g"]}},
{"id": "denovo9184", "metadata": {"taxonomy": ["Bacteria",
"__Proteobacteria", "__Deltaproteobacteria", "__Desulfuromonadales",
"__Desulfuromonadaceae", "__Desulfuromonas"]}},{"id": "denovo9190",
"metadata": {"taxonomy": ["Bacteria", "__Proteobacteria",
"__Gammaproteobacteria", "__Xanthomonadales", "__Sinobacteraceae",
"__JTB255_marine_benthic_group"]}},{"id": "denovo9193", "metadata":
{"taxonomy": ["Bacteria", "__Chloroflexi", "__SAR202_clade", "__o",
"__f", "__g"]}},{"id": "denovo9197", "metadata": {"taxonomy":
["Bacteria", "__Proteobacteria"]}},{"id": "denovo9198", "metadata":
{"taxonomy": ["Bacteria", "__Proteobacteria", "__Alphaproteobacteria",
"__Rhodospirillales", "__Rhodospirillaceae", "__g"]}},{"id":
"denovo9199", "metadata": {"taxonomy": ["Bacteria",
"__Proteobacteria", "__Deltaproteobacteria", "__Sh765B-TzT-29", "__f",
"__g"]}},{"id": "denovo9201", "metadata": {"taxonomy": ["Bacteria",
"__Proteobacteria", "__Epsilonproteobacteria", "__Campylobacterales",
"__Campylobacteraceae", "__Arcobacter"]}},{"id": "denovo9205",
"metadata": {"taxonomy": ["Bacteria", "__Actinobacteria",
"__Acidimicrobiia", "__Acidimicrobiales", "__Sva0996_marine_group",
"__g"]}},{"id": "denovo9206", "metadata": {"taxonomy": ["Bacteria"]}},
{"id": "denovo9207", "metadata": {"taxonomy": ["Bacteria",
"__Bacteroidetes", "__Bacteroidia", "__Bacteroidales",

"__Marinilabiaceae", "__Marinifilum"]}},{"id": "denovo9209", "metadata": {"taxonomy": ["Bacteria", "__Bacteroidetes", "__Flavobacteria", "__Flavobacteriales", "__Flavobacteriaceae"]}}, {"id": "denovo9210", "metadata": {"taxonomy": ["Bacteria", "__Proteobacteria", "__Alphaproteobacteria", "__Rhodobacterales", "__Rhodobacteraceae"]}},{"id": "denovo9212", "metadata": {"taxonomy": ["Bacteria", "__Proteobacteria", "__JTB23", "__o", "__f", "__g"]}}, {"id": "denovo9217", "metadata": {"taxonomy": ["Bacteria"]}},{"id": "denovo9221", "metadata": {"taxonomy": ["Bacteria", "__Proteobacteria", "__Gammaproteobacteria", "__Legionellales", "__Legionellaceae", "__g"]}},{"id": "denovo9223", "metadata": {"taxonomy": ["Bacteria", "__Bacteroidetes", "__Sphingobacteriia", "__Sphingobacteriales", "__Chitinophagaceae", "__g"]}},{"id": "denovo9226", "metadata": {"taxonomy": ["Bacteria", "__Proteobacteria", "__Gammaproteobacteria", "__Oceanospirillales", "__Oceanospirillaceae", "__Pseudospirillum"]}},{"id": "denovo9228", "metadata": {"taxonomy": ["Bacteria", "__Proteobacteria", "__Deltaproteobacteria", "__Myxococcales", "__Haliangiaceae", "__Haliangium"]}},{"id": "denovo9229", "metadata": {"taxonomy": ["Bacteria", "__Firmicutes", "__Clostridia", "__Clostridiales"]}}, {"id": "denovo9232", "metadata": {"taxonomy": ["Bacteria", "__Proteobacteria", "__Gammaproteobacteria", "__Oceanospirillales", "__Hahellaceae", "__Endozoicomonas"]}},{"id": "denovo9234", "metadata": {"taxonomy": ["Bacteria", "__Proteobacteria", "__Deltaproteobacteria", "__Sh765B-TzT-29", "__f", "__g"]}},{"id": "denovo9235", "metadata": {"taxonomy": ["Bacteria", "__Proteobacteria", "__Alphaproteobacteria", "__Rhodobacterales", "__Rhodobacteraceae", "__Ruegeria"]}},{"id": "denovo9236", "metadata": {"taxonomy": ["Bacteria", "__Bacteroidetes", "__Sphingobacteriia", "__Sphingobacteriales", "__Saprospiraceae", "__g"]}},{"id": "denovo9238", "metadata": {"taxonomy": ["Bacteria", "__Proteobacteria", "__Gammaproteobacteria", "__Xanthomonadales", "__Sinobacteraceae", "__JTB255_marine_benthic_group"]}},{"id": "denovo9242", "metadata": {"taxonomy": ["Bacteria", "__Proteobacteria", "__Gammaproteobacteria", "__Order_Incertae_Sedis", "__Family_Incertae_Sedis", "__Marinicella"]}},{"id": "denovo9244", "metadata": {"taxonomy": ["Bacteria", "__Bacteroidetes", "__Sphingobacteriia", "__Sphingobacteriales", "__NS11-12_marine_group", "__g"]}},{"id": "denovo9246", "metadata": {"taxonomy": ["Bacteria", "__Acidobacteria", "__Acidobacteria", "__BPC102", "__f", "__g"]}},{"id": "denovo9248", "metadata": {"taxonomy": ["Bacteria", "__Proteobacteria", "__Gammaproteobacteria", "__Order_Incertae_Sedis", "__Family_Incertae_Sedis", "__Marinicella"]}},{"id": "denovo9251", "metadata": {"taxonomy": ["Bacteria", "__Cyanobacteria", "__Cyanobacteria", "__SubsectionIII", "__FamilyI", "__Symploca"]}},{"id": "denovo9255", "metadata": {"taxonomy": ["Bacteria", "__Planctomycetes", "__Phycisphaerae", "__Phycisphaerales", "__Phycisphaeraceae", "__Phycisphaera"]}},{"id": "denovo9256", "metadata": {"taxonomy": ["Bacteria", "__Proteobacteria", "__Alphaproteobacteria", "__Rhodobacterales",

"__Rhodobacteraceae"]}},{"id": "denovo9260", "metadata": {"taxonomy": ["Bacteria", "__Proteobacteria", "__Alphaproteobacteria", "__Rhodobacterales", "__Rhodobacteraceae", "__g"]}},{"id": "denovo9261", "metadata": {"taxonomy": ["Bacteria", "__Chlamydiae", "__Chlamydiae", "__Chlamydiales", "__cvE6", "__g"]}},{"id": "denovo9266", "metadata": {"taxonomy": ["Bacteria", "__Bacteroidetes", "__Flavobacteria", "__Flavobacteriales", "__Flavobacteriaceae", "__Aquimarina"]}},{"id": "denovo9269", "metadata": {"taxonomy": ["Bacteria", "__Proteobacteria", "__Alphaproteobacteria", "__Rhodobacterales", "__Rhodobacteraceae"]}},{"id": "denovo9270", "metadata": {"taxonomy": ["Bacteria", "__Acidobacteria", "__Holophagae", "__Acanthopleuribacterales", "__Acanthopleuribacteraceae", "__Acanthopleuribacter"]}},{"id": "denovo9272", "metadata": {"taxonomy": ["Bacteria", "__Proteobacteria", "__Gammaproteobacteria"]}},{"id": "denovo9276", "metadata": {"taxonomy": ["Bacteria", "__Acidobacteria", "__Acidobacteria", "__BPC102", "__f", "__g"]}},{"id": "denovo9278", "metadata": {"taxonomy": ["Bacteria", "__Proteobacteria", "__Deltaproteobacteria", "__Sh765B-TzT-29", "__f", "__g"]}},{"id": "denovo9279", "metadata": {"taxonomy": ["Bacteria", "__Proteobacteria", "__Alphaproteobacteria", "__Rhodospirillales", "__Rhodospirillaceae", "__g"]}},{"id": "denovo9283", "metadata": {"taxonomy": ["Bacteria", "__Proteobacteria", "__Alphaproteobacteria", "__Rhodospirillales", "__Rhodospirillaceae"]}},{"id": "denovo9285", "metadata": {"taxonomy": ["Bacteria", "__Bacteroidetes", "__Sphingobacteriia", "__Sphingobacteriales", "__Saprospiraceae", "__g"]}},{"id": "denovo9286", "metadata": {"taxonomy": ["Bacteria", "__Acidobacteria", "__Holophagae", "__CA002", "__f", "__g"]}},{"id": "denovo9287", "metadata": {"taxonomy": ["Bacteria", "__Actinobacteria", "__Acidimicrobiia", "__Acidimicrobiales", "__Sva0996_marine_group", "__g"]}},{"id": "denovo9291", "metadata": {"taxonomy": ["Bacteria", "__Proteobacteria", "__Deltaproteobacteria", "__Desulfobacterales", "__Nitrospinaceae", "__Nitrospina"]}},{"id": "denovo9292", "metadata": {"taxonomy": ["Bacteria", "__Proteobacteria", "__Alphaproteobacteria", "__Rhizobiales", "__Phyllobacteriaceae", "__g"]}},{"id": "denovo9294", "metadata": {"taxonomy": ["Bacteria", "__Proteobacteria", "__Gammaproteobacteria", "__Xanthomonadales", "__Sinobacteraceae", "__JTB255_marine_benthic_group"]}},{"id": "denovo9300", "metadata": {"taxonomy": ["Bacteria", "__Proteobacteria"]}},{"id": "denovo9306", "metadata": {"taxonomy": ["Bacteria", "__Planctomycetes", "__Phycisphaerae", "__Phycisphaerales", "__Phycisphaeraceae", "__SM1A02"]}},{"id": "denovo9307", "metadata": {"taxonomy": ["Bacteria", "__Proteobacteria", "__Alphaproteobacteria", "__Rhizobiales", "__Rhodobiaceae", "__Rhodobium"]}},{"id": "denovo9315", "metadata": {"taxonomy": ["Bacteria", "__Chloroflexi", "__Anaerolineae", "__Anaerolineales", "__Anaerolineaceae", "__g"]}},{"id": "denovo9324", "metadata": {"taxonomy": ["Bacteria", "__Proteobacteria", "__Gammaproteobacteria", "__Enterobacteriales", "__Enterobacteriaceae", "__Raoultella"]}},{"id": "denovo9325",

"metadata": {"taxonomy": ["Bacteria", "__Proteobacteria", "__Alphaproteobacteria", "__Rickettsiales", "__SM2D12", "__g"]}}, {"id": "denovo9327", "metadata": {"taxonomy": ["Bacteria", "__Proteobacteria", "__Gammaproteobacteria", "__Order_Incertae_Sedis", "__Family_Incertae_Sedis", "__Marinicella"]}},{"id": "denovo9328", "metadata": {"taxonomy": ["Bacteria", "__Bacteroidetes", "__Cytophagia", "__Cytophagales", "__Flammeovirgaceae", "__Ekhidna"]}},{"id": "denovo9331", "metadata": {"taxonomy": ["Bacteria", "__Proteobacteria", "__Alphaproteobacteria", "__Sphingomonadales", "__Erythrobacteraceae", "__g"]}},{"id": "denovo9332", "metadata": {"taxonomy": ["Bacteria", "__Firmicutes", "__Clostridia", "__Clostridiales"]}},{"id": "denovo9333", "metadata": {"taxonomy": ["Bacteria", "__Gemmatimonadetes", "__Gemmatimonadetes", "__Kazan-1B-37", "__f", "__g"]}},{"id": "denovo9334", "metadata": {"taxonomy": ["Bacteria", "__Verrucomicrobia", "__Verrucomicrobiae", "__Verrucomicrobiales", "__Rubritaleaceae", "__Rubritalea"]}},{"id": "denovo9341", "metadata": {"taxonomy": ["Bacteria", "__Proteobacteria", "__Deltaproteobacteria", "__Myxococcales", "__Nannocystaceae", "__g"]}},{"id": "denovo9345", "metadata": {"taxonomy": ["Bacteria", "__Proteobacteria", "__Alphaproteobacteria", "__Rhodobacterales", "__Rhodobacteraceae", "__Rhodovulum"]}},{"id": "denovo9348", "metadata": {"taxonomy": ["Bacteria", "__Proteobacteria", "__Gammaproteobacteria", "__NKB5", "__f", "__g"]}},{"id": "denovo9350", "metadata": {"taxonomy": ["Bacteria", "__Proteobacteria", "__Gammaproteobacteria", "__Alteromonadales", "__Alteromonadaceae", "__g"]}},{"id": "denovo9358", "metadata": {"taxonomy": ["Bacteria", "__Proteobacteria", "__Deltaproteobacteria", "__Sh765B-TzT-29", "__f", "__g"]}},{"id": "denovo9359", "metadata": {"taxonomy": ["Bacteria", "__Proteobacteria", "__Gammaproteobacteria", "__Oceanospirillales", "__SS1-B-06-26", "__g"]}},{"id": "denovo9363", "metadata": {"taxonomy": ["Bacteria", "__Proteobacteria", "__Alphaproteobacteria", "__Parvularculales", "__Parvularculaceae", "__Parvularcula"]}},{"id": "denovo9374", "metadata": {"taxonomy": ["Bacteria", "__Bacteroidetes", "__Flavobacteria", "__Flavobacteriales", "__Cryomorphaceae", "__NS7_marine_group"]}}, {"id": "denovo9375", "metadata": {"taxonomy": ["Bacteria", "__Proteobacteria", "__Alphaproteobacteria"]}},{"id": "denovo9376", "metadata": {"taxonomy": ["Bacteria", "__Chloroflexi", "__Caldilineae", "__Caldilineales", "__Caldilineaceae", "__g"]}}, {"id": "denovo9380", "metadata": {"taxonomy": ["Bacteria", "__Proteobacteria"]}},{"id": "denovo9384", "metadata": {"taxonomy": ["Bacteria", "__Bacteroidetes"]}},{"id": "denovo9388", "metadata": {"taxonomy": ["Bacteria", "__Proteobacteria", "__Alphaproteobacteria", "__Rickettsiales", "__Holosporaceae", "__Holospora"]}},{"id": "denovo9389", "metadata": {"taxonomy": ["Bacteria", "__Firmicutes", "__Bacilli", "__Bacillales", "__Staphylococcaceae", "__Staphylococcus"]}},{"id": "denovo9394", "metadata": {"taxonomy": ["Bacteria", "__Verrucomicrobia", "__Opitutae", "__Puniceicoccales", "__Puniceicoccaceae", "__g"]}},{"id": "denovo9397", "metadata": {"taxonomy": ["Bacteria", "__Candidate_division_WS3", "__c", "__o",

"__f", "__g"]}},{"id": "denovo9400", "metadata": {"taxonomy": ["Bacteria", "__Proteobacteria", "__Gammaproteobacteria", "__KI89A_clade", "__f", "__g"]}},{"id": "denovo9404", "metadata": {"taxonomy": ["Bacteria", "__Proteobacteria", "__Deltaproteobacteria", "__Desulfobacterales", "__Desulfobacteraceae", "__Sva0081_sediment_group"]}},{"id": "denovo9406", "metadata": {"taxonomy": ["Bacteria", "__Planctomycetes", "__OM190", "__o", "__f", "__g"]}},{"id": "denovo9411", "metadata": {"taxonomy": ["Bacteria", "__Acidobacteria", "__Holophagae", "__Acanthopleuribacterales", "__Acanthopleuribacteraceae", "__Acanthopleuribacter"]}},{"id": "denovo9415", "metadata": {"taxonomy": ["Bacteria", "__Proteobacteria", "__Alphaproteobacteria", "__Rhodobacterales", "__Rhodobacteraceae", "__Wenxinia"]}},{"id": "denovo9417", "metadata": {"taxonomy": ["Bacteria", "__Acidobacteria", "__Holophagae", "__Sva0725", "__f", "__g"]}},{"id": "denovo9423", "metadata": {"taxonomy": ["Bacteria", "__Proteobacteria", "__Alphaproteobacteria", "__Rhizobiales"]}},{"id": "denovo9424", "metadata": {"taxonomy": ["Bacteria", "__Proteobacteria", "__Gammaproteobacteria", "__Enterobacteriales", "__Enterobacteriaceae", "__Escherichia-Shigella"]}},{"id": "denovo9425", "metadata": {"taxonomy": ["Bacteria", "__Bacteroidetes", "__Flavobacteria", "__Flavobacteriales", "__Flavobacteriaceae", "__Winogradskyella"]}},{"id": "denovo9433", "metadata": {"taxonomy": ["Bacteria", "__Planctomycetes", "__Phycisphaerae", "__Phycisphaerales", "__Phycisphaeraceae", "__Urania-1B-19_marine_sediment_group"]}},{"id": "denovo9434", "metadata": {"taxonomy": ["Bacteria", "__Proteobacteria", "__Deltaproteobacteria", "__Bdellovibrionales", "__Bdellovibrionaceae", "__Bdellovibrio"]}},{"id": "denovo9435", "metadata": {"taxonomy": ["Bacteria", "__Proteobacteria", "__Gammaproteobacteria"]}},{"id": "denovo9439", "metadata": {"taxonomy": ["Bacteria", "__Proteobacteria", "__Deltaproteobacteria", "__Sh765B-TzT-29", "__f", "__g"]}},{"id": "denovo9440", "metadata": {"taxonomy": ["Bacteria", "__Proteobacteria", "__Alphaproteobacteria", "__Rhodobacterales", "__Rhodobacteraceae", "__Dinoroseobacter"]}},{"id": "denovo9443", "metadata": {"taxonomy": ["Bacteria", "__Acidobacteria", "__Acidobacteria", "__DA023", "__f", "__g"]}},{"id": "denovo9448", "metadata": {"taxonomy": ["Bacteria", "__Proteobacteria", "__Gammaproteobacteria", "__Xanthomonadales", "__Sinobacteraceae", "__JTB255_marine_benthic_group"]}},{"id": "denovo9449", "metadata": {"taxonomy": ["Bacteria", "__Proteobacteria", "__Deltaproteobacteria", "__Myxococcales", "__0319-6G20", "__g"]}},{"id": "denovo9450", "metadata": {"taxonomy": ["Bacteria", "__Bacteroidetes", "__Flavobacteria", "__Flavobacteriales", "__Flavobacteriaceae"]}},{"id": "denovo9453", "metadata": {"taxonomy": ["Bacteria", "__Proteobacteria", "__Deltaproteobacteria", "__Desulfuromonadales", "__GR-WP33-58", "__g"]}},{"id": "denovo9457", "metadata": {"taxonomy": ["Bacteria", "__Proteobacteria", "__Deltaproteobacteria", "__Sh765B-TzT-29", "__f", "__g"]}},{"id": "denovo9460", "metadata": {"taxonomy": ["Bacteria", "__Bacteroidetes", "__Flavobacteria", "__Flavobacteriales",

"__Cryomorphaceae", "__Owenweeksia"]}},{"id": "denovo9461", "metadata": {"taxonomy": ["Bacteria", "__Proteobacteria", "__Gammaproteobacteria", "__Alteromonadales", "__Alteromonadaceae"]}}, {"id": "denovo9463", "metadata": {"taxonomy": ["Bacteria", "__Proteobacteria", "__Deltaproteobacteria", "__Myxococcales", "__Cystobacterineae", "__g"]}},{"id": "denovo9464", "metadata": {"taxonomy": ["Bacteria", "__Gemmatimonadetes", "__Gemmatimonadetes", "__BD2-11_terrestrial_group", "__f", "__g"]}},{"id": "denovo9465", "metadata": {"taxonomy": ["Bacteria", "__Proteobacteria", "__Alphaproteobacteria", "__Rhodospirillales", "__Rhodospirillaceae", "__g"]}},{"id": "denovo9467", "metadata": {"taxonomy": ["Bacteria", "__Proteobacteria", "__Gammaproteobacteria"]}},{"id": "denovo9478", "metadata": {"taxonomy": ["Bacteria", "__Proteobacteria", "__Gammaproteobacteria", "__Vibrionales", "__Vibrionaceae", "__Aliivibrio"]}},{"id": "denovo9480", "metadata": {"taxonomy": ["Bacteria", "__Bacteroidetes"]}},{"id": "denovo9481", "metadata": {"taxonomy": ["Bacteria", "__Proteobacteria", "__Gammaproteobacteria", "__Oceanospirillales", "__OM182_clade", "__g"]}},{"id": "denovo9488", "metadata": {"taxonomy": ["Bacteria", "__Proteobacteria", "__Alphaproteobacteria", "__Rhizobiales", "__Rhizobiaceae", "__Shinella"]}},{"id": "denovo9489", "metadata": {"taxonomy": ["Bacteria", "__Chloroflexi", "__Caldilineae", "__Caldilineales", "__Caldilineaceae", "__g"]}},{"id": "denovo9494", "metadata": {"taxonomy": ["Bacteria", "__Proteobacteria", "__Gammaproteobacteria", "__Oceanospirillales", "__Oceanospirillaceae", "__Amphritea"]}},{"id": "denovo9495", "metadata": {"taxonomy": ["Bacteria", "__Proteobacteria", "__Gammaproteobacteria", "__Thiotrichales"]}}, {"id": "denovo9500", "metadata": {"taxonomy": ["Bacteria", "__Proteobacteria", "__Gammaproteobacteria", "__Xanthomonadales", "__Sinobacteraceae", "__JTB255_marine_benthic_group"]}},{"id": "denovo9504", "metadata": {"taxonomy": ["Bacteria", "__Proteobacteria", "__Gammaproteobacteria", "__Oceanospirillales", "__Hahellaceae"]}},{"id": "denovo9510", "metadata": {"taxonomy": ["Bacteria", "__Firmicutes", "__Clostridia", "__Clostridiales", "__Lachnospiraceae"]}},{"id": "denovo9512", "metadata": {"taxonomy": ["Bacteria", "__Proteobacteria", "__Deltaproteobacteria", "__Bdellovibrionales", "__Bdellovibrionaceae", "__OM27_clade"]}}, {"id": "denovo9515", "metadata": {"taxonomy": ["Bacteria", "__Proteobacteria", "__Gammaproteobacteria", "__E01-9C-26_marine_group", "__f", "__g"]}},{"id": "denovo9518", "metadata": {"taxonomy": ["Bacteria", "__Bacteroidetes", "__Sphingobacteriia", "__Sphingobacteriales", "__Saprospiraceae", "__g"]}},{"id": "denovo9519", "metadata": {"taxonomy": ["Bacteria", "__Proteobacteria", "__Deltaproteobacteria", "__Bdellovibrionales", "__Bdellovibrionaceae", "__OM27_clade"]}},{"id": "denovo9522", "metadata": {"taxonomy": ["Bacteria", "__Proteobacteria", "__Gammaproteobacteria", "__Pseudomonadales", "__Moraxellaceae", "__Perlucidibaca"]}},{"id": "denovo9523", "metadata": {"taxonomy": ["Bacteria", "__Bacteroidetes", "__Flavobacteria", "__Flavobacteriales", "__Cryomorphaceae", "__Owenweeksia"]}},{"id":

"denovo9524", "metadata": {"taxonomy": ["Bacteria", "__Candidate_division_WS3", "__c", "__o", "__f", "__g"]}},{"id": "denovo9525", "metadata": {"taxonomy": ["Bacteria"]}},{"id": "denovo9527", "metadata": {"taxonomy": ["Bacteria", "__Proteobacteria", "__Gammaproteobacteria", "__Chromatiales", "__Granulosicoccaceae", "__Granulosicoccus"]}},{"id": "denovo9529", "metadata": {"taxonomy": ["Bacteria", "__Candidate_division_WS3", "__c", "__o", "__f", "__g"]}},{"id": "denovo9532", "metadata": {"taxonomy": ["Bacteria", "__Proteobacteria", "__Gammaproteobacteria", "__Vibrionales", "__Vibrionaceae", "__Enterovibrio"]}},{"id": "denovo9534", "metadata": {"taxonomy": ["Bacteria", "__Proteobacteria", "__Alphaproteobacteria", "__SAR11_clade", "__Surface_1", "__g"]}},{"id": "denovo9538", "metadata": {"taxonomy": ["Bacteria", "__Proteobacteria", "__Deltaproteobacteria", "__SAR324_clade(Marine_group_B)", "__f", "__g"]}},{"id": "denovo9542", "metadata": {"taxonomy": ["Bacteria", "__Actinobacteria", "__Acidimicrobiia", "__Acidimicrobiales", "__Sva0996_marine_group", "__g"]}},{"id": "denovo9545", "metadata": {"taxonomy": ["Bacteria", "__Cyanobacteria", "__Cyanobacteria", "__SubsectionI", "__FamilyI", "__Cyanobacterium"]}},{"id": "denovo9546", "metadata": {"taxonomy": ["Bacteria", "__Proteobacteria", "__Gammaproteobacteria"]}},{"id": "denovo9549", "metadata": {"taxonomy": ["Bacteria", "__Proteobacteria", "__Alphaproteobacteria", "__Rhodospirillales", "__Rhodospirillaceae", "__Defluviicoccus"]}},{"id": "denovo9551", "metadata": {"taxonomy": ["Bacteria", "__Planctomycetes", "__OM190", "__o", "__f", "__g"]}},{"id": "denovo9554", "metadata": {"taxonomy": ["Bacteria", "__Proteobacteria", "__Alphaproteobacteria", "__Rhizobiales"]}},{"id": "denovo9557", "metadata": {"taxonomy": ["Bacteria", "__Proteobacteria", "__Gammaproteobacteria", "__E01-9C-26_marine_group", "__f", "__g"]}},{"id": "denovo9560", "metadata": {"taxonomy": ["Bacteria", "__Proteobacteria", "__Gammaproteobacteria", "__Xanthomonadales", "__Sinobacteraceae", "__JTB255_marine_benthic_group"]}},{"id": "denovo9562", "metadata": {"taxonomy": ["Bacteria", "__Proteobacteria", "__Alphaproteobacteria", "__Rickettsiales", "__SM2D12", "__g"]}},{"id": "denovo9563", "metadata": {"taxonomy": ["Bacteria", "__Proteobacteria", "__Alphaproteobacteria", "__Parvularculales", "__Parvularculaceae", "__Parvularcula"]}},{"id": "denovo9564", "metadata": {"taxonomy": ["Bacteria", "__Proteobacteria", "__Alphaproteobacteria", "__SAR11_clade", "__Surface_1", "__Candidatus_Pelagibacter"]}},{"id": "denovo9567", "metadata": {"taxonomy": ["Bacteria", "__Nitrospirae", "__Nitrospira", "__Nitrospirales", "__Nitrospiraceae", "__Nitrospira"]}},{"id": "denovo9569", "metadata": {"taxonomy": ["Bacteria", "__Bacteroidetes", "__Flavobacteria", "__Flavobacteriales", "__Flavobacteriaceae", "__Leptobacterium"]}},{"id": "denovo9570", "metadata": {"taxonomy": ["Bacteria", "__Proteobacteria", "__Alphaproteobacteria", "__Rhizobiales", "__Hyphomicrobiaceae", "__g"]}},{"id": "denovo9571", "metadata": {"taxonomy": ["Bacteria", "__Proteobacteria", "__Gammaproteobacteria", "__Alteromonadales", "__Colwelliaceae", "__Thalassomonas"]}},{"id":

"denovo9576", "metadata": {"taxonomy": ["Bacteria", "__Proteobacteria", "__Deltaproteobacteria", "__Sva0485", "__f", "__g"]}},{"id": "denovo9579", "metadata": {"taxonomy": ["Bacteria", "__Bacteroidetes", "__Flavobacteria", "__Flavobacteriales", "__Flavobacteriaceae", "__NS5_marine_group"]}},{"id": "denovo9580", "metadata": {"taxonomy": ["Bacteria", "__Bacteroidetes", "__Sphingobacteriia", "__Sphingobacteriales", "__Saprospiraceae", "__g"]}},{"id": "denovo9587", "metadata": {"taxonomy": ["Bacteria", "__Proteobacteria", "__Gammaproteobacteria", "__Oceanospirillales", "__Hahellaceae", "__Endozoicomonas"]}},{"id": "denovo9589", "metadata": {"taxonomy": ["Bacteria", "__Cyanobacteria", "__Cyanobacteria", "__SubsectionIII", "__FamilyI", "__Phormidium"]}},{"id": "denovo9593", "metadata": {"taxonomy": ["Bacteria", "__Cyanobacteria", "__Cyanobacteria", "__SubsectionIII", "__FamilyI", "__Lyngbya"]}},{"id": "denovo9594", "metadata": {"taxonomy": ["Bacteria", "__BD1-5", "__c", "__o", "__f", "__g"]}},{"id": "denovo9595", "metadata": {"taxonomy": ["Bacteria", "__Proteobacteria", "__Gammaproteobacteria", "__Pseudomonadales", "__Moraxellaceae", "__Acinetobacter"]}},{"id": "denovo9602", "metadata": {"taxonomy": ["Bacteria", "__Proteobacteria", "__Gammaproteobacteria", "__Order_Incertae_Sedis", "__Family_Incertae_Sedis", "__Marinicella"]}},{"id": "denovo9608", "metadata": {"taxonomy": ["Bacteria", "__Proteobacteria", "__Alphaproteobacteria", "__Rhizobiales", "__Brucellaceae", "__Ochrobactrum"]}},{"id": "denovo9609", "metadata": {"taxonomy": ["Bacteria", "__Proteobacteria", "__Alphaproteobacteria", "__Rhodobacterales", "__Rhodobacteraceae"]}},{"id": "denovo9612", "metadata": {"taxonomy": ["Bacteria", "__Proteobacteria", "__Deltaproteobacteria", "__Bdellovibrionales", "__Bdellovibrionaceae", "__Bdellovibrio"]}},{"id": "denovo9613", "metadata": {"taxonomy": ["Bacteria", "__Proteobacteria", "__Alphaproteobacteria", "__Rhodospirillales", "__Rhodospirillaceae", "__g"]}},{"id": "denovo9620", "metadata": {"taxonomy": ["Bacteria", "__Firmicutes", "__Clostridia", "__Clostridiales", "__Peptostreptococcaceae", "__g"]}},{"id": "denovo9622", "metadata": {"taxonomy": ["Bacteria", "__Bacteroidetes", "__Cytophagia", "__Cytophagales", "__Flammeovirgaceae", "__Ekhidna"]}},{"id": "denovo9623", "metadata": {"taxonomy": ["Bacteria", "__Proteobacteria", "__Alphaproteobacteria", "__Rhodobacterales", "__Rhodobacteraceae", "__Pseudovibrio"]}},{"id": "denovo9627", "metadata": {"taxonomy": ["Bacteria", "__Proteobacteria", "__Gammaproteobacteria", "__Pseudomonadales", "__Moraxellaceae", "__Acinetobacter"]}},{"id": "denovo9634", "metadata": {"taxonomy": ["Bacteria", "__Proteobacteria", "__Alphaproteobacteria", "__Rhodobacterales", "__Rhodobacteraceae", "__Ruegeria"]}},{"id": "denovo9635", "metadata": {"taxonomy": ["Bacteria", "__Proteobacteria", "__Gammaproteobacteria", "__Chromatiales", "__Granulosicoccaceae", "__Granulosicoccus"]}},{"id": "denovo9638", "metadata": {"taxonomy": ["Bacteria", "__Proteobacteria", "__Gammaproteobacteria", "__Pseudomonadales", "__Moraxellaceae",

"__Acinetobacter"]}},{"id": "denovo9639", "metadata": {"taxonomy": ["Bacteria", "__Chloroflexi", "__SAR202_clade", "__o", "__f", "__g"]}},{"id": "denovo9640", "metadata": {"taxonomy": ["Bacteria", "__Proteobacteria", "__Alphaproteobacteria", "__Rhodobacterales", "__Rhodobacteraceae", "__Thalassobius"]}},{"id": "denovo9641", "metadata": {"taxonomy": ["Bacteria", "__Proteobacteria", "__Deltaproteobacteria", "__Desulfobacterales", "__Desulfobacteraceae", "__Desulfospira"]}},{"id": "denovo9649", "metadata": {"taxonomy": ["Bacteria", "__Gemmatimonadetes", "__Gemmatimonadetes", "__Kazan-1B-37", "__f", "__g"]}},{"id": "denovo9650", "metadata": {"taxonomy": ["Bacteria", "__Bacteroidetes", "__Flavobacteria", "__Flavobacteriales", "__Flavobacteriaceae", "__Eudoraea"]}},{"id": "denovo9657", "metadata": {"taxonomy": ["Bacteria", "__Proteobacteria", "__Gammaproteobacteria", "__Vibrionales", "__Vibrionaceae", "__Vibrio"]}},{"id": "denovo9660", "metadata": {"taxonomy": ["Bacteria", "__Proteobacteria", "__Gammaproteobacteria", "__Order_Incertae_Sedis", "__Family_Incertae_Sedis", "__Arenicella"]}},{"id": "denovo9663", "metadata": {"taxonomy": ["Bacteria", "__Proteobacteria", "__Alphaproteobacteria", "__Rhodobacterales", "__Rhodobacteraceae", "__Pannonibacter"]}},{"id": "denovo9667", "metadata": {"taxonomy": ["Bacteria", "__Proteobacteria", "__Alphaproteobacteria"]}},{"id": "denovo9670", "metadata": {"taxonomy": ["Bacteria", "__Proteobacteria", "__Gammaproteobacteria", "__Enterobacteriales", "__Enterobacteriaceae"]}},{"id": "denovo9671", "metadata": {"taxonomy": ["Bacteria", "__Proteobacteria", "__Alphaproteobacteria", "__Rickettsiales", "__SAR116_clade", "__g"]}},{"id": "denovo9673", "metadata": {"taxonomy": ["Bacteria", "__Acidobacteria", "__Acidobacteria", "__PAUC26f", "__f", "__g"]}},{"id": "denovo9674", "metadata": {"taxonomy": ["Bacteria", "__Gemmatimonadetes", "__Gemmatimonadetes", "__AT425-EubC11_terrestrial_group", "__f", "__g"]}},{"id": "denovo9677", "metadata": {"taxonomy": ["Bacteria", "__Proteobacteria", "__Gammaproteobacteria", "__Xanthomonadales", "__Sinobacteraceae", "__JTB255_marine_benthic_group"]}},{"id": "denovo9678", "metadata": {"taxonomy": ["Bacteria", "__Proteobacteria", "__Deltaproteobacteria", "__Myxococcales", "__Nannocystineae", "__g"]}},{"id": "denovo9679", "metadata": {"taxonomy": ["Bacteria", "__Bacteroidetes", "__Flavobacteria", "__Flavobacteriales", "__Cryomorphaceae", "__Owenweeksia"]}},{"id": "denovo9680", "metadata": {"taxonomy": ["Bacteria", "__Proteobacteria", "__Alphaproteobacteria", "__ss1-B-07-44", "__f", "__g"]}},{"id": "denovo9681", "metadata": {"taxonomy": ["Bacteria", "__Proteobacteria", "__Alphaproteobacteria", "__Rhodospirillales", "__Rhodospirillaceae", "__Defluviicoccus"]}},{"id": "denovo9682", "metadata": {"taxonomy": ["Bacteria", "__Firmicutes", "__Bacilli"]}},{"id": "denovo9683", "metadata": {"taxonomy": ["Archaea", "__Euryarchaeota", "__Halobacteria", "__Halobacteriales", "__Deep_Sea_Hydrothermal_Vent_Gp_6(DHVEG-6)", "__Candidatus_Parvarchaeum"]}},{"id": "denovo9684", "metadata": {"taxonomy": ["Bacteria", "__Chlorobi", "__Ignavibacteria",

"__Ignavibacteriales", "__BSV26", "__g"]}},{"id": "denovo9687", "metadata": {"taxonomy": ["Bacteria", "__Proteobacteria", "__Deltaproteobacteria", "__Desulfobacterales", "__Nitrospinaceae", "__Candidatus_Entotheonella"]}},{"id": "denovo9690", "metadata": {"taxonomy": ["Bacteria", "__Proteobacteria", "__Gammaproteobacteria", "__Order_Incertae_Sedis", "__Family_Incertae_Sedis", "__Marinicella"]}},{"id": "denovo9700", "metadata": {"taxonomy": ["Bacteria", "__Proteobacteria", "__Alphaproteobacteria", "__DB1-14", "__f", "__g"]}},{"id": "denovo9701", "metadata": {"taxonomy": ["Bacteria", "__Proteobacteria", "__Alphaproteobacteria", "__Rhodobacterales", "__Rhodobacteraceae"]}},{"id": "denovo9702", "metadata": {"taxonomy": ["Bacteria", "__Proteobacteria", "__Alphaproteobacteria", "__Rhodobacterales", "__Rhodobacteraceae"]}},{"id": "denovo9709", "metadata": {"taxonomy": ["Bacteria", "__Proteobacteria", "__Gammaproteobacteria", "__KI89A_clade", "__f", "__g"]}},{"id": "denovo9712", "metadata": {"taxonomy": ["Bacteria", "__Proteobacteria", "__Alphaproteobacteria", "__Rhodobacterales", "__Rhodobacteraceae", "__g"]}},{"id": "denovo9718", "metadata": {"taxonomy": ["Bacteria", "__Bacteroidetes", "__Sphingobacteriia", "__Sphingobacteriales", "__Saprospiraceae", "__Lewinella"]}},{"id": "denovo9721", "metadata": {"taxonomy": ["Bacteria", "__Firmicutes", "__Clostridia", "__Halanaerobiales", "__64K2", "__g"]}},{"id": "denovo9722", "metadata": {"taxonomy": ["Bacteria", "__Chloroflexi", "__SAR202_clade", "__o", "__f", "__g"]}},{"id": "denovo9723", "metadata": {"taxonomy": ["Bacteria", "__Cyanobacteria", "__SM1D11", "__o", "__f", "__g"]}},{"id": "denovo9725", "metadata": {"taxonomy": ["Bacteria", "__Bacteroidetes", "__Flavobacteria", "__Flavobacteriales", "__Flavobacteriaceae", "__g"]}},{"id": "denovo9726", "metadata": {"taxonomy": ["Bacteria", "__Proteobacteria", "__Gammaproteobacteria", "__Oceanospirillales", "__Hahellaceae", "__Endozoicomonas"]}},{"id": "denovo9731", "metadata": {"taxonomy": ["Bacteria", "__Proteobacteria", "__Deltaproteobacteria", "__Sh765B-TzT-29", "__f", "__g"]}},{"id": "denovo9732", "metadata": {"taxonomy": ["Bacteria", "__Gemmatimonadetes", "__Gemmatimonadetes", "__BD2-11_terrestrial_group", "__f", "__g"]}},{"id": "denovo9733", "metadata": {"taxonomy": ["Bacteria", "__Proteobacteria", "__Deltaproteobacteria", "__SAR324_clade(Marine_group_B)", "__f", "__g"]}},{"id": "denovo9734", "metadata": {"taxonomy": ["Bacteria", "__Proteobacteria", "__Gammaproteobacteria"]}},{"id": "denovo9735", "metadata": {"taxonomy": ["Bacteria", "__Proteobacteria", "__Gammaproteobacteria", "__Oceanospirillales", "__MBAE14", "__g"]}},{"id": "denovo9738", "metadata": {"taxonomy": ["Bacteria", "__Proteobacteria", "__Deltaproteobacteria", "__Myxococcales", "__Cystobacterineae", "__g"]}},{"id": "denovo9739", "metadata": {"taxonomy": ["Bacteria", "__Proteobacteria", "__Alphaproteobacteria"]}},{"id": "denovo9741", "metadata": {"taxonomy": ["Bacteria", "__Proteobacteria", "__Gammaproteobacteria"]}},{"id": "denovo9746", "metadata": {"taxonomy": ["Bacteria", "__Proteobacteria", "__Gammaproteobacteria",

"__Alteromonadales", "__Pseudoalteromonadaceae", "__Pseudoalteromonas"]}},{"id": "denovo9747", "metadata": {"taxonomy": ["Bacteria", "__Verrucomicrobia", "__Verrucomicrobiae", "__Verrucomicrobiales", "__Verrucomicrobiaceae", "__Roseibacillus"]}}, {"id": "denovo9748", "metadata": {"taxonomy": ["Bacteria", "__Cyanobacteria", "__SM1D11", "__o", "__f", "__g"]}},{"id": "denovo9753", "metadata": {"taxonomy": ["Bacteria", "__Proteobacteria", "__Alphaproteobacteria", "__Rhizobiales", "__Rhizobiaceae", "__Rhizobium"]}},{"id": "denovo9757", "metadata": {"taxonomy": ["Bacteria", "__Proteobacteria", "__Gammaproteobacteria", "__Pseudomonadales", "__Moraxellaceae", "__Acinetobacter"]}},{"id": "denovo9758", "metadata": {"taxonomy": ["Bacteria", "__Proteobacteria", "__Deltaproteobacteria", "__Bdellovibrionales", "__Bdellovibrionaceae", "__Bdellovibrio"]}},{"id": "denovo9759", "metadata": {"taxonomy": ["Bacteria", "__Proteobacteria", "__Alphaproteobacteria", "__Rhodospirillales", "__Rhodospirillaceae", "__Pelagibius"]}},{"id": "denovo9761", "metadata": {"taxonomy": ["Bacteria", "__Proteobacteria", "__Gammaproteobacteria", "__Order_Incertae_Sedis", "__Family_Incertae_Sedis", "__Marinicella"]}},{"id": "denovo9764", "metadata": {"taxonomy": ["Bacteria", "__Proteobacteria", "__Alphaproteobacteria", "__Rhodospirillales", "__Rhodospirillaceae", "__AEGEAN-169_marine_group"]}},{"id": "denovo9766", "metadata": {"taxonomy": ["Bacteria", "__Proteobacteria", "__Gammaproteobacteria"]}},{"id": "denovo9767", "metadata": {"taxonomy": ["Bacteria", "__Proteobacteria", "__Deltaproteobacteria", "__Desulfuromonadales", "__GR-WP33-58", "__g"]}},{"id": "denovo9768", "metadata": {"taxonomy": ["Bacteria", "__Proteobacteria", "__Deltaproteobacteria", "__Sva0485", "__f", "__g"]}},{"id": "denovo9769", "metadata": {"taxonomy": ["Bacteria", "__Proteobacteria", "__Gammaproteobacteria"]}},{"id": "denovo9775", "metadata": {"taxonomy": ["Bacteria", "__Bacteroidetes", "__Cytophagia", "__Cytophagales", "__Flammeovirgaceae", "__Candidatus_Amoebophilus"]}},{"id": "denovo9777", "metadata": {"taxonomy": ["Bacteria", "__Proteobacteria", "__Gammaproteobacteria", "__Xanthomonadales", "__Sinobacteraceae", "__JTB255_marine_benthic_group"]}},{"id": "denovo9778", "metadata": {"taxonomy": ["Bacteria", "__Acidobacteria", "__Acidobacteria", "__PAUC26f", "__f", "__g"]}},{"id": "denovo9784", "metadata": {"taxonomy": ["Bacteria", "__Firmicutes", "__Clostridia", "__Clostridiales", "__Lachnospiraceae"]}},{"id": "denovo9785", "metadata": {"taxonomy": ["Bacteria", "__Proteobacteria", "__Gammaproteobacteria", "__BD7-8_marine_group", "__f", "__g"]}}, {"id": "denovo9788", "metadata": {"taxonomy": ["Bacteria", "__Proteobacteria", "__Alphaproteobacteria", "__Rhodospirillales", "__Rhodospirillaceae", "__Pelagibius"]}},{"id": "denovo9792", "metadata": {"taxonomy": ["Bacteria", "__Proteobacteria", "__Alphaproteobacteria", "__Rhizobiales", "__Phyllobacteriaceae", "__Ahrensia"]}},{"id": "denovo9795", "metadata": {"taxonomy": ["Bacteria", "__Bacteroidetes", "__Flavobacteria",

"__Flavobacteriales", "__Flavobacteriaceae", "__Muricauda"]}},{"id": "denovo9798", "metadata": {"taxonomy": ["Bacteria", "__Proteobacteria", "__Gammaproteobacteria", "__Pseudomonadales", "__Moraxellaceae", "__Acinetobacter"]}},{"id": "denovo9802", "metadata": {"taxonomy": ["Bacteria", "__Bacteroidetes", "__Flavobacteria", "__Flavobacteriales", "__Flavobacteriaceae", "__g"]}},{"id": "denovo9807", "metadata": {"taxonomy": ["Bacteria", "__Proteobacteria", "__Betaproteobacteria"]}},{"id": "denovo9809", "metadata": {"taxonomy": ["Bacteria", "__Proteobacteria", "__Alphaproteobacteria", "__Rhodobacterales", "__Rhodobacteraceae"]}},{"id": "denovo9810", "metadata": {"taxonomy": ["Bacteria", "__Proteobacteria", "__Gammaproteobacteria", "__Pseudomonadales", "__Moraxellaceae", "__Acinetobacter"]}},{"id": "denovo9823", "metadata": {"taxonomy": ["Bacteria", "__Gemmatimonadetes", "__Gemmatimonadetes", "__BD2-11_terrestrial_group", "__f", "__g"]}},{"id": "denovo9828", "metadata": {"taxonomy": ["Bacteria", "__Proteobacteria", "__Deltaproteobacteria", "__Desulfobacterales", "__Desulfobulbaceae", "__Desulfopila"]}},{"id": "denovo9831", "metadata": {"taxonomy": ["Bacteria", "__Proteobacteria", "__Alphaproteobacteria", "__Rhodospirillales", "__Rhodospirillaceae", "__g"]}},{"id": "denovo9834", "metadata": {"taxonomy": ["Bacteria", "__Proteobacteria", "__Gammaproteobacteria", "__BD3-1", "__f", "__g"]}},{"id": "denovo9837", "metadata": {"taxonomy": ["Bacteria", "__Firmicutes", "__Clostridia", "__Clostridiales", "__Ruminococcaceae", "__g"]}},{"id": "denovo9841", "metadata": {"taxonomy": ["Bacteria", "__Proteobacteria", "__Gammaproteobacteria"]}},{"id": "denovo9842", "metadata": {"taxonomy": ["Bacteria", "__Proteobacteria", "__Gammaproteobacteria", "__NKB5", "__f", "__g"]}},{"id": "denovo9846", "metadata": {"taxonomy": ["Bacteria", "__Proteobacteria", "__Gammaproteobacteria", "__Alteromonadales", "__Alteromonadaceae", "__BD2-7"]}},{"id": "denovo9847", "metadata": {"taxonomy": ["Bacteria", "__Proteobacteria", "__Gammaproteobacteria", "__Oceanospirillales"]}},{"id": "denovo9851", "metadata": {"taxonomy": ["Bacteria", "__Proteobacteria", "__Gammaproteobacteria", "__Alteromonadales", "__Alteromonadaceae"]}},{"id": "denovo9853", "metadata": {"taxonomy": ["Bacteria", "__Bacteroidetes", "__Cytophagia", "__Cytophagales", "__Flammeovirgaceae", "__Ekhidna"]}},{"id": "denovo9855", "metadata": {"taxonomy": ["Bacteria", "__Proteobacteria", "__Alphaproteobacteria", "__Rhizobiales"]}},{"id": "denovo9856", "metadata": {"taxonomy": ["Bacteria", "__Bacteroidetes", "__Sphingobacteriia", "__Sphingobacteriales", "__Saprospiraceae", "__g"]}},{"id": "denovo9858", "metadata": {"taxonomy": ["Bacteria", "__Cyanobacteria", "__Cyanobacteria", "__SubsectionIII", "__FamilyI"]}},{"id": "denovo9863", "metadata": {"taxonomy": ["Bacteria", "__Proteobacteria"]}},{"id": "denovo9866", "metadata": {"taxonomy": ["Bacteria", "__Proteobacteria", "__Betaproteobacteria", "__Burkholderiales", "__Burkholderiaceae", "__Polynucleobacter"]}},{"id": "denovo9875", "metadata": {"taxonomy": ["Bacteria", "__Proteobacteria", "__Alphaproteobacteria", "__Rhodospirillales",

"__Acetobacteraceae", "__Rhodovarius"]}},{"id": "denovo9884", "metadata": {"taxonomy": ["Bacteria", "__Fusobacteria", "__Fusobacteria", "__Fusobacteriales", "__Fusobacteriaceae", "__Propionigenium"]}},{"id": "denovo9885", "metadata": {"taxonomy": ["Bacteria", "__Proteobacteria", "__Deltaproteobacteria", "__Myxococcales", "__Haliangiaceae", "__Haliangium"]}},{"id": "denovo9886", "metadata": {"taxonomy": ["Bacteria", "__Proteobacteria", "__Gammaproteobacteria"]}},{"id": "denovo9887", "metadata": {"taxonomy": ["Bacteria", "__Proteobacteria", "__Deltaproteobacteria", "__Desulfobacterales", "__Desulfobulbaceae", "__Desulfopila"]}},{"id": "denovo9888", "metadata": {"taxonomy": ["Bacteria", "__Proteobacteria", "__Gammaproteobacteria"]}},{"id": "denovo9890", "metadata": {"taxonomy": ["Bacteria", "__TM6", "__c", "__o", "__f", "__g"]}},{"id": "denovo9899", "metadata": {"taxonomy": ["Bacteria", "__Proteobacteria", "__Alphaproteobacteria", "__Rickettsiales"]}},{"id": "denovo9900", "metadata": {"taxonomy": ["Bacteria", "__Proteobacteria", "__Deltaproteobacteria", "__Desulfobacterales", "__Desulfobacteraceae", "__Desulfococcus"]}},{"id": "denovo9909", "metadata": {"taxonomy": ["Bacteria", "__Acidobacteria", "__RB25", "__o", "__f", "__g"]}},{"id": "denovo9913", "metadata": {"taxonomy": ["Bacteria", "__Proteobacteria", "__Alphaproteobacteria"]}},{"id": "denovo9915", "metadata": {"taxonomy": ["Bacteria", "__Proteobacteria", "__Deltaproteobacteria", "__Sh765B-TzT-29", "__f", "__g"]}},{"id": "denovo9917", "metadata": {"taxonomy": ["Bacteria", "__Proteobacteria", "__Deltaproteobacteria", "__Desulfobacterales", "__Desulfobacteraceae"]}},{"id": "denovo9918", "metadata": {"taxonomy": ["Bacteria", "__Acidobacteria", "__RB25", "__o", "__f", "__g"]}},{"id": "denovo9919", "metadata": {"taxonomy": ["Bacteria", "__Cyanobacteria", "__Cyanobacteria", "__SubsectionIII", "__FamilyI"]}},{"id": "denovo9921", "metadata": {"taxonomy": ["Bacteria", "__Proteobacteria", "__Alphaproteobacteria", "__Rhodospirillales", "__Rhodospirillaceae", "__Pelagibius"]}},{"id": "denovo9923", "metadata": {"taxonomy": ["Bacteria", "__Chloroflexi", "__Caldilineae", "__Caldilineales", "__Caldilineaceae", "__Caldilinea"]}},{"id": "denovo9926", "metadata": {"taxonomy": ["Bacteria", "__Proteobacteria", "__Gammaproteobacteria", "__Oceanospirillales", "__TX4CB-01", "__g"]}},{"id": "denovo9928", "metadata": {"taxonomy": ["Bacteria", "__Proteobacteria", "__Alphaproteobacteria", "__Rhizobiales"]}},{"id": "denovo9932", "metadata": {"taxonomy": ["Bacteria", "__Proteobacteria", "__Gammaproteobacteria", "__Alteromonadales", "__Alteromonadaceae", "__Haliea"]}},{"id": "denovo9934", "metadata": {"taxonomy": ["Bacteria", "__Cyanobacteria", "__Cyanobacteria", "__SubsectionIII", "__FamilyI", "__Phormidium"]}},{"id": "denovo9935", "metadata": {"taxonomy": ["Bacteria", "__Proteobacteria", "__Gammaproteobacteria", "__Vibrionales", "__Vibrionaceae", "__Vibrio"]}},{"id": "denovo9937", "metadata": {"taxonomy": ["Bacteria", "__Proteobacteria", "__Alphaproteobacteria", "__Sphingomonadales", "__Family_Incertae_Sedis", "__Kiloniella"]}},{"id": "denovo9941",

"metadata": {"taxonomy": ["Bacteria", "__Proteobacteria",
"__Deltaproteobacteria", "__Bdellovibrionales",
"__Bdellovibrionaceae", "__OM27_clade"]}},{"id": "denovo9942",
"metadata": {"taxonomy": ["Bacteria", "__Proteobacteria",
"__Gammaproteobacteria"]}},{"id": "denovo9946", "metadata":
{"taxonomy": ["Bacteria", "__Verrucomicrobia", "__Verrucomicrobiae",
"__Verrucomicrobiales", "__Verrucomicrobiaceae", "__Roseibacillus"]}},
{"id": "denovo9955", "metadata": {"taxonomy": ["Bacteria",
"__Proteobacteria", "__Gammaproteobacteria", "__Alteromonadales",
"__Alteromonadaceae", "__BD1-7_clade"]}},{"id": "denovo9958",
"metadata": {"taxonomy": ["Bacteria", "__Nitrospirae", "__Nitrospira",
"__Nitrospirales", "__Nitrospiraceae", "__Nitrospira"]}},{"id":
"denovo9959", "metadata": {"taxonomy": ["Bacteria",
"__Proteobacteria", "__Gammaproteobacteria", "__Xanthomonadales",
"__Xanthomonadaceae", "__Luteimonas"]}},{"id": "denovo9960",
"metadata": {"taxonomy": ["Bacteria", "__Planctomycetes",
"__Phycisphaerae", "__CCM11a", "__f", "__g"]}},{"id": "denovo9961",
"metadata": {"taxonomy": ["Bacteria", "__Proteobacteria"]}},{"id":
"denovo9966", "metadata": {"taxonomy": ["Bacteria", "__Bacteroidetes",
"__Cytophagia", "__Cytophagales", "__Flammeovirgaceae", "__g"]}},
{"id": "denovo9968", "metadata": {"taxonomy": ["Bacteria",
"__Proteobacteria", "__Gammaproteobacteria"]}},{"id": "denovo9969",
"metadata": {"taxonomy": ["Bacteria", "__Acidobacteria",
"__Holophagae", "__Acanthopleuribacterales",
"__Acanthopleuribacteraceae", "__Acanthopleuribacter"]}},{"id":
"denovo9970", "metadata": {"taxonomy": ["Bacteria", "__Cyanobacteria",
"__Cyanobacteria", "__SubsectionI", "__FamilyI", "__Acaryochloris"]}},
{"id": "denovo9972", "metadata": {"taxonomy": ["Bacteria",
"__Bacteroidetes", "__Cytophagia", "__Cytophagales",
"__Flammeovirgaceae", "__g"]}},{"id": "denovo9973", "metadata":
{"taxonomy": ["Bacteria", "__Verrucomicrobia", "__S-
BQ2-57_soil_group", "__o", "__f", "__g"]}},{"id": "denovo9974",
"metadata": {"taxonomy": ["Bacteria", "__Proteobacteria",
"__Alphaproteobacteria", "__Rickettsiales", "__Rickettsiaceae",
"__Candidatus_Cryptoprodotis"]}},{"id": "denovo9976", "metadata":
{"taxonomy": ["Bacteria", "__Proteobacteria", "__Gammaproteobacteria",
"__Enterobacteriales", "__Enterobacteriaceae"]}},{"id": "denovo9978",
"metadata": {"taxonomy": ["Bacteria", "__Proteobacteria",
"__Deltaproteobacteria", "__SAR324_clade(Marine_group_B)", "__f",
"__g"]}},{"id": "denovo9979", "metadata": {"taxonomy": ["Bacteria",
"__Bacteroidetes", "__Flavobacteria", "__Flavobacteriales",
"__NS9_marine_group", "__g"]}},{"id": "denovo9981", "metadata":
{"taxonomy": ["Bacteria", "__Proteobacteria", "__Alphaproteobacteria",
"__DB1-14", "__f", "__g"]}},{"id": "denovo9982", "metadata":
{"taxonomy": ["Bacteria", "__Proteobacteria", "__Alphaproteobacteria",
"__Rickettsiales", "__Rickettsiaceae", "__g"]}},{"id": "denovo9987",
"metadata": {"taxonomy": ["Bacteria", "__Chloroflexi",
"__Caldilineae", "__Caldilineales", "__Caldilineaceae",
"__Caldilinea"]}},{"id": "denovo9992", "metadata": {"taxonomy":
["Bacteria", "__Proteobacteria", "__Alphaproteobacteria",

"__Sphingomonadales", "__Erythrobacteraceae", "__Erythrobacter"]}},
{"id": "denovo9996", "metadata": {"taxonomy": ["Bacteria",
"__Proteobacteria", "__Alphaproteobacteria"]}},{"id": "denovo9997",
"metadata": {"taxonomy": ["Bacteria", "__Proteobacteria",
"__Deltaproteobacteria", "__Myxococcales", "__Haliangiaceae",
"__Haliangium"]}},{"id": "denovo10004", "metadata": {"taxonomy":
["Bacteria", "__Proteobacteria", "__JTB23", "__o", "__f", "__g"]}},
{"id": "denovo10005", "metadata": {"taxonomy": ["Bacteria",
"__Proteobacteria", "__Alphaproteobacteria", "__Rhodospirillales",
"__Rhodospirillaceae", "__Thalassospira"]}},{"id": "denovo10007",
"metadata": {"taxonomy": ["Bacteria", "__Proteobacteria",
"__Alphaproteobacteria", "__Rhizobiales", "__Hyphomicrobiaceae"]}},
{"id": "denovo10011", "metadata": {"taxonomy": ["Bacteria",
"__Proteobacteria", "__TA18", "__o", "__f", "__g"]}},{"id":
"denovo10012", "metadata": {"taxonomy": ["Bacteria",
"__Proteobacteria", "__Gammaproteobacteria", "__Alteromonadales",
"__Pseudoalteromonadaceae", "__Pseudoalteromonas"]}},{"id":
"denovo10013", "metadata": {"taxonomy": ["Bacteria",
"__Proteobacteria", "__Alphaproteobacteria", "__OCS116_clade", "__f",
"__g"]}},{"id": "denovo10015", "metadata": {"taxonomy": ["Bacteria",
"__Proteobacteria", "__Alphaproteobacteria", "__Rhodobacterales",
"__Rhodobacteraceae"]}},{"id": "denovo10018", "metadata": {"taxonomy":
["Bacteria", "__Proteobacteria", "__Deltaproteobacteria",
"__Desulfobacterales", "__Nitrospinaceae", "__Nitrospina"]}},{"id":
"denovo10020", "metadata": {"taxonomy": ["Bacteria", "__Firmicutes",
"__Clostridia", "__Halanaerobiales", "__Halobacteroidaceae", "__g"]}},
{"id": "denovo10021", "metadata": {"taxonomy": ["Bacteria",
"__Proteobacteria", "__Alphaproteobacteria", "__Rhodospirillales",
"__Rhodospirillaceae", "__Defluviicoccus"]}},{"id": "denovo10025",
"metadata": {"taxonomy": ["Bacteria", "__Proteobacteria",
"__Gammaproteobacteria", "__Alteromonadales", "__Alteromonadaceae"]}},
{"id": "denovo10033", "metadata": {"taxonomy": ["Bacteria",
"__Acidobacteria", "__Acidobacteria", "__AT-s3-28", "__f", "__g"]}},
{"id": "denovo10035", "metadata": {"taxonomy": ["Bacteria",
"__Chloroflexi", "__SAR202_clade", "__o", "__f", "__g"]}},{"id":
"denovo10037", "metadata": {"taxonomy": ["Bacteria",
"__Planctomycetes", "__OM190", "__o", "__f", "__g"]}},{"id":
"denovo10039", "metadata": {"taxonomy": ["Bacteria",
"__Actinobacteria", "__Actinobacteria", "__Propionibacteriales",
"__Nocardioidaceae", "__Nocardioides"]}},{"id": "denovo10041",
"metadata": {"taxonomy": ["Bacteria", "__Proteobacteria",
"__Alphaproteobacteria", "__Rhodospirillales", "__Rhodospirillaceae",
"__g"]}},{"id": "denovo10054", "metadata": {"taxonomy": ["Bacteria",
"__Cyanobacteria", "__Cyanobacteria", "__SubsectionIII", "__FamilyI",
"__Oscillatoria"]}},{"id": "denovo10055", "metadata": {"taxonomy":
["Bacteria", "__Actinobacteria", "__Acidimicrobiia",
"__Acidimicrobiales", "__OCS155_marine_group", "__g"]}},{"id":
"denovo10060", "metadata": {"taxonomy": ["Bacteria",
"__Proteobacteria", "__Alphaproteobacteria", "__Rhizobiales"]}},{"id":
"denovo10063", "metadata": {"taxonomy": ["Bacteria",

"__Gemmatimonadetes", "__Gemmatimonadetes",
"__BD2-11_terrestrial_group", "__f", "__g"]}},{"id": "denovo10066",
"metadata": {"taxonomy": ["Bacteria", "__Cyanobacteria",
"__Cyanobacteria", "__SubsectionIII", "__FamilyI", "__g"]}},{"id":
"denovo10067", "metadata": {"taxonomy": ["Bacteria",
"__Proteobacteria", "__Betaproteobacteria", "__Methylophilales",
"__Methylophilaceae", "__Methylotenera"]}},{"id": "denovo10068",
"metadata": {"taxonomy": ["Bacteria", "__Proteobacteria",
"__Alphaproteobacteria", "__Kordiimonadales", "__Kordiimonadaceae",
"__Kordiimonas"]}},{"id": "denovo10070", "metadata": {"taxonomy":
["Bacteria", "__Proteobacteria", "__Gammaproteobacteria",
"__Alteromonadales", "__Alteromonadaceae", "__C1-B045"]}},{"id":
"denovo10076", "metadata": {"taxonomy": ["Bacteria",
"__Proteobacteria", "__Deltaproteobacteria", "__Myxococcales",
"__0319-6G20", "__g"]}},{"id": "denovo10081", "metadata": {"taxonomy":
["Bacteria", "__Proteobacteria", "__Gammaproteobacteria",
"__Xanthomonadales", "__Sinobacteraceae",
"__JTB255_marine_benthic_group"]}},{"id": "denovo10082", "metadata":
{"taxonomy": ["Bacteria", "__Proteobacteria", "__Deltaproteobacteria",
"__SAR324_clade(Marine_group_B)", "__f", "__g"]}},{"id":
"denovo10086", "metadata": {"taxonomy": ["Bacteria",
"__Proteobacteria", "__Gammaproteobacteria"]}},{"id": "denovo10088",
"metadata": {"taxonomy": ["Bacteria", "__Bacteroidetes",
"__Flavobacteria", "__Flavobacteriales", "__Cryomorphaceae",
"__Fluviicola"]}},{"id": "denovo10090", "metadata": {"taxonomy":
["Bacteria", "__Proteobacteria", "__Gammaproteobacteria", "__o",
"__f", "__g"]}},{"id": "denovo10097", "metadata": {"taxonomy":
["Bacteria", "__Proteobacteria", "__Alphaproteobacteria", "__DB1-14",
"__f", "__g"]}},{"id": "denovo10099", "metadata": {"taxonomy":
["Archaea", "__Euryarchaeota", "__Thermoplasmata",
"__Thermoplasmatales", "__Marine_Group_II", "__g"]}},{"id":
"denovo10103", "metadata": {"taxonomy": ["Bacteria", "__Firmicutes",
"__Bacilli", "__Bacillales", "__Bacillaceae",
"__Paucisalibacillus"]}},{"id": "denovo10106", "metadata":
{"taxonomy": ["Bacteria", "__Planctomycetes", "__Phycisphaerae",
"__Phycisphaerales", "__Phycisphaeraceae",
"__Urania-1B-19_marine_sediment_group"]}},{"id": "denovo10108",
"metadata": {"taxonomy": ["Bacteria", "__Proteobacteria",
"__Deltaproteobacteria", "__Sh765B-TzT-29", "__f", "__g"]}},{"id":
"denovo10111", "metadata": {"taxonomy": ["Bacteria",
"__Proteobacteria", "__Gammaproteobacteria"]}},{"id": "denovo10113",
"metadata": {"taxonomy": ["Bacteria", "__Bacteroidetes",
"__Flavobacteria", "__Flavobacteriales", "__Flavobacteriaceae",
"__Tenacibaculum"]}},{"id": "denovo10114", "metadata": {"taxonomy":
["Bacteria", "__Proteobacteria", "__Gammaproteobacteria",
"__Pseudomonadales", "__Moraxellaceae", "__Acinetobacter"]}},{"id":
"denovo10118", "metadata": {"taxonomy": ["Bacteria",
"__Bacteroidetes", "__Flavobacteria", "__Flavobacteriales",
"__Flavobacteriaceae", "__Maritimimonas"]}},{"id": "denovo10122",
"metadata": {"taxonomy": ["Bacteria", "__Proteobacteria",

"__Alphaproteobacteria", "__Rickettsiales", "__SAR116_clade", "__g"]}},{"id": "denovo10123", "metadata": {"taxonomy": ["Bacteria", "__Proteobacteria", "__Alphaproteobacteria", "__Rhodobacterales", "__Rhodobacteraceae", "__Thalassococcus"]}},{"id": "denovo10127", "metadata": {"taxonomy": ["Bacteria", "__Firmicutes", "__Clostridia", "__Clostridiales", "__Clostridiaceae", "__Caminicella"]}},{"id": "denovo10129", "metadata": {"taxonomy": ["Bacteria", "__Bacteroidetes", "__Sphingobacteriia", "__Sphingobacteriales", "__Saprospiraceae", "__g"]}},{"id": "denovo10134", "metadata": {"taxonomy": ["Bacteria", "__Proteobacteria", "__Alphaproteobacteria", "__Rhodospirillales", "__Rhodospirillaceae", "__g"]}},{"id": "denovo10135", "metadata": {"taxonomy": ["Bacteria", "__Proteobacteria", "__Deltaproteobacteria", "__Desulfuromonadales", "__Sva1033", "__g"]}},{"id": "denovo10136", "metadata": {"taxonomy": ["Bacteria", "__Firmicutes", "__Clostridia", "__Clostridiales", "__Lachnospiraceae"]}},{"id": "denovo10138", "metadata": {"taxonomy": ["Bacteria", "__Proteobacteria", "__Alphaproteobacteria", "__SAR11_clade", "__Surface_1"]}},{"id": "denovo10139", "metadata": {"taxonomy": ["Bacteria", "__Gemmatimonadetes", "__Gemmatimonadetes", "__Kazan-1B-37", "__f", "__g"]}},{"id": "denovo10141", "metadata": {"taxonomy": ["Bacteria", "__Proteobacteria", "__Gammaproteobacteria", "__Oceanospirillales", "__OM182_clade", "__g"]}},{"id": "denovo10143", "metadata": {"taxonomy": ["Bacteria", "__Proteobacteria", "__Alphaproteobacteria"]}},{"id": "denovo10148", "metadata": {"taxonomy": ["Bacteria", "__Proteobacteria", "__Gammaproteobacteria", "__Oceanospirillales"]}},{"id": "denovo10149", "metadata": {"taxonomy": ["Bacteria", "__Bacteroidetes", "__Flavobacteria", "__Flavobacteriales", "__Flavobacteriaceae", "__NS5_marine_group"]}},{"id": "denovo10150", "metadata": {"taxonomy": ["Bacteria", "__Proteobacteria", "__Gammaproteobacteria", "__Pseudomonadales", "__Moraxellaceae", "__Acinetobacter"]}},{"id": "denovo10154", "metadata": {"taxonomy": ["Bacteria", "__Proteobacteria", "__Deltaproteobacteria", "__Myxococcales", "__Haliangiaceae", "__Haliangium"]}},{"id": "denovo10158", "metadata": {"taxonomy": ["Bacteria", "__Bacteroidetes", "__Flavobacteria", "__Flavobacteriales", "__Flavobacteriaceae", "__NS5_marine_group"]}},{"id": "denovo10160", "metadata": {"taxonomy": ["Bacteria", "__Proteobacteria", "__Alphaproteobacteria", "__Sphingomonadales", "__Sphingomonadaceae"]}},{"id": "denovo10161", "metadata": {"taxonomy": ["Bacteria", "__Proteobacteria", "__Gammaproteobacteria", "__Alteromonadales", "__Alteromonadaceae", "__Haliea"]}},{"id": "denovo10162", "metadata": {"taxonomy": ["Bacteria", "__Bacteroidetes", "__Sphingobacteriia", "__Sphingobacteriales", "__Saprospiraceae", "__g"]}},{"id": "denovo10163", "metadata": {"taxonomy": ["Bacteria", "__Proteobacteria", "__Gammaproteobacteria", "__Pseudomonadales", "__Pseudomonadaceae", "__Pseudomonas"]}},{"id": "denovo10166", "metadata": {"taxonomy": ["Bacteria", "__Proteobacteria", "__CF2", "__o", "__f", "__g"]}},{"id": "denovo10168", "metadata": {"taxonomy": ["Bacteria", "__Bacteroidetes", "__Sphingobacteriia", "__Sphingobacteriales",

"__Saprospiraceae", "__g"]}},{"id": "denovo10178", "metadata": {"taxonomy": ["Bacteria", "__Proteobacteria", "__Gammaproteobacteria", "__Oceanospirillales", "__Alcanivoracaceae", "__Kangiella"]}},{"id": "denovo10180", "metadata": {"taxonomy": ["Bacteria", "__Proteobacteria", "__Deltaproteobacteria", "__Sh765B-TzT-29", "__f", "__g"]}},{"id": "denovo10183", "metadata": {"taxonomy": ["Bacteria", "__Cyanobacteria", "__Cyanobacteria", "__SubsectionI", "__FamilyI", "__Acaryochloris"]}},{"id": "denovo10185", "metadata": {"taxonomy": ["Bacteria", "__Proteobacteria", "__Alphaproteobacteria", "__Rickettsiales", "__Rickettsiaceae", "__g"]}},{"id": "denovo10191", "metadata": {"taxonomy": ["Bacteria", "__Actinobacteria", "__Acidimicrobiia", "__Acidimicrobiales", "__Sva0996_marine_group", "__g"]}},{"id": "denovo10195", "metadata": {"taxonomy": ["Bacteria", "__Proteobacteria", "__Alphaproteobacteria", "__Rhizobiales", "__Phyllobacteriaceae"]}},{"id": "denovo10201", "metadata": {"taxonomy": ["Bacteria", "__Bacteroidetes", "__Sphingobacteriia", "__Sphingobacteriales", "__WCHB1-69", "__g"]}},{"id": "denovo10202", "metadata": {"taxonomy": ["Bacteria", "__Proteobacteria", "__Gammaproteobacteria", "__Pseudomonadales", "__Moraxellaceae", "__Acinetobacter"]}},{"id": "denovo10203", "metadata": {"taxonomy": ["Bacteria", "__Proteobacteria", "__Deltaproteobacteria", "__Desulfobacterales", "__Nitrospinaceae", "__Candidatus_Entotheonella"]}},{"id": "denovo10205", "metadata": {"taxonomy": ["Bacteria", "__Bacteroidetes", "__Sphingobacteriia", "__Sphingobacteriales", "__Saprospiraceae", "__g"]}},{"id": "denovo10206", "metadata": {"taxonomy": ["Bacteria", "__Bacteroidetes", "__Flavobacteria", "__Flavobacteriales", "__Flavobacteriaceae", "__Aquimarina"]}},{"id": "denovo10218", "metadata": {"taxonomy": ["Bacteria", "__Proteobacteria", "__Deltaproteobacteria", "__GR-WP33-30", "__f", "__g"]}},{"id": "denovo10220", "metadata": {"taxonomy": ["Bacteria", "__Proteobacteria", "__Alphaproteobacteria", "__Rhodospirillales", "__Rhodospirillaceae"]}},{"id": "denovo10225", "metadata": {"taxonomy": ["Bacteria", "__Bacteroidetes", "__Flavobacteria", "__Flavobacteriales", "__Flavobacteriaceae", "__NS5_marine_group"]}},{"id": "denovo10228", "metadata": {"taxonomy": ["Bacteria", "__Bacteroidetes", "__Sphingobacteriia", "__Sphingobacteriales", "__Saprospiraceae"]}},{"id": "denovo10229", "metadata": {"taxonomy": ["Bacteria", "__Firmicutes", "__Clostridia", "__Clostridiales", "__Christensenellaceae", "__Christensenella"]}},{"id": "denovo10231", "metadata": {"taxonomy": ["Bacteria", "__Proteobacteria", "__Alphaproteobacteria", "__Rhodospirillales", "__Rhodospirillaceae", "__AEGEAN-169_marine_group"]}},{"id": "denovo10232", "metadata": {"taxonomy": ["Bacteria", "__Acidobacteria", "__Holophagae", "__CA002", "__f", "__g"]}},{"id": "denovo10233", "metadata": {"taxonomy": ["Bacteria", "__Proteobacteria", "__Alphaproteobacteria", "__Rhizobiales", "__Phyllobacteriaceae", "__g"]}},{"id": "denovo10234", "metadata": {"taxonomy": ["Bacteria", "__Proteobacteria", "__Alphaproteobacteria", "__Sphingomonadales"]}},{"id": "denovo10235", "metadata": {"taxonomy": ["Bacteria",

"__Proteobacteria", "__Gammaproteobacteria", "__Chromatiales", "__Chromatiaceae", "__Nitrosococcus"]}},{"id": "denovo10236", "metadata": {"taxonomy": ["Bacteria", "__Proteobacteria", "__Alphaproteobacteria"]}},{"id": "denovo10237", "metadata": {"taxonomy": ["Bacteria", "__Proteobacteria", "__Gammaproteobacteria", "__Oceanospirillales"]}},{"id": "denovo10239", "metadata": {"taxonomy": ["Bacteria", "__Proteobacteria", "__Alphaproteobacteria", "__SAR11_clade", "__Surface_1", "__Candidatus_Pelagibacter"]}},{"id": "denovo10240", "metadata": {"taxonomy": ["Bacteria", "__Bacteroidetes", "__Flavobacteria", "__Flavobacteriales", "__Flavobacteriaceae", "__Formosa"]}},{"id": "denovo10242", "metadata": {"taxonomy": ["Bacteria", "__Proteobacteria", "__Alphaproteobacteria", "__Rhodobacterales", "__Rhodobacteraceae"]}},{"id": "denovo10250", "metadata": {"taxonomy": ["Bacteria", "__Proteobacteria", "__Alphaproteobacteria", "__Rhizobiales", "__Phyllobacteriaceae", "__Cohaesibacter"]}},{"id": "denovo10256", "metadata": {"taxonomy": ["Bacteria", "__Proteobacteria", "__Deltaproteobacteria", "__Myxococcales", "__Sorangiineae", "__g"]}},{"id": "denovo10258", "metadata": {"taxonomy": ["Bacteria", "__Actinobacteria", "__Acidimicrobiia", "__Acidimicrobiales", "__Acidimicrobiaceae", "__g"]}},{"id": "denovo10262", "metadata": {"taxonomy": ["Bacteria", "__Firmicutes", "__Clostridia", "__Halanaerobiales", "__64K2", "__g"]}},{"id": "denovo10266", "metadata": {"taxonomy": ["Bacteria", "__Proteobacteria", "__Gammaproteobacteria", "__Xanthomonadales", "__Sinobacteraceae", "__JTB255_marine_benthic_group"]}},{"id": "denovo10268", "metadata": {"taxonomy": ["Bacteria"]}},{"id": "denovo10269", "metadata": {"taxonomy": ["Bacteria", "__Proteobacteria", "__Deltaproteobacteria"]}},{"id": "denovo10270", "metadata": {"taxonomy": ["Bacteria", "__Proteobacteria", "__Alphaproteobacteria", "__SAR11_clade", "__Surface_1", "__g"]}},{"id": "denovo10273", "metadata": {"taxonomy": ["Bacteria", "__Chloroflexi", "__JG30-KF-CM66", "__o", "__f", "__g"]}},{"id": "denovo10275", "metadata": {"taxonomy": ["Bacteria", "__Proteobacteria", "__Gammaproteobacteria", "__Pseudomonadales", "__Pseudomonadaceae", "__Pseudomonas"]}},{"id": "denovo10278", "metadata": {"taxonomy": ["Bacteria", "__Proteobacteria", "__Deltaproteobacteria", "__Sh765B-TzT-29", "__f", "__g"]}},{"id": "denovo10281", "metadata": {"taxonomy": ["Bacteria", "__Proteobacteria", "__Gammaproteobacteria", "__BD7-8_marine_group", "__f", "__g"]}},{"id": "denovo10282", "metadata": {"taxonomy": ["Bacteria", "__Acidobacteria", "__Acidobacteria", "__DA023", "__f", "__g"]}},{"id": "denovo10283", "metadata": {"taxonomy": ["Bacteria", "__Bacteroidetes", "__Flavobacteria", "__Flavobacteriales", "__Flavobacteriaceae", "__Algibacter"]}},{"id": "denovo10286", "metadata": {"taxonomy": ["Bacteria", "__Proteobacteria", "__Betaproteobacteria", "__Burkholderiales", "__Oxalobacteraceae", "__Massilia"]}},{"id": "denovo10287", "metadata": {"taxonomy": ["Bacteria", "__Proteobacteria", "__Gammaproteobacteria", "__BD7-8_marine_group", "__f", "__g"]}},{"id": "denovo10288", "metadata": {"taxonomy": ["Bacteria", "__Proteobacteria",

"__Deltaproteobacteria", "__GR-WP33-30", "__f", "__g"]}},{"id": "denovo10289", "metadata": {"taxonomy": ["Bacteria", "__Proteobacteria", "__Gammaproteobacteria", "__E01-9C-26_marine_group", "__f", "__g"]}},{"id": "denovo10291", "metadata": {"taxonomy": ["Bacteria", "__Proteobacteria", "__Alphaproteobacteria", "__Rhizobiales", "__Rhodobiaceae", "__Rhodobium"]}},{"id": "denovo10292", "metadata": {"taxonomy": ["Archaea", "__Euryarchaeota", "__Halobacteria", "__Halobacteriales", "__Deep_Sea_Hydrothermal_Vent_Gp_6(DHVEG-6)", "__Candidatus_Parvarchaeum"]}},{"id": "denovo10293", "metadata": {"taxonomy": ["Bacteria", "__Proteobacteria", "__Gammaproteobacteria"]}},{"id": "denovo10300", "metadata": {"taxonomy": ["Bacteria", "__Proteobacteria", "__Deltaproteobacteria", "__Desulfobacterales", "__Desulfobacteraceae", "__g"]}},{"id": "denovo10307", "metadata": {"taxonomy": ["Bacteria", "__Chloroflexi", "__SAR202_clade", "__o", "__f", "__g"]}},{"id": "denovo10308", "metadata": {"taxonomy": ["Bacteria", "__Bacteroidetes", "__Sphingobacteriia", "__Sphingobacteriales", "__Saprospiraceae", "__g"]}},{"id": "denovo10309", "metadata": {"taxonomy": ["Bacteria", "__Proteobacteria", "__Betaproteobacteria", "__Hydrogenophilales", "__Hydrogenophilaceae", "__Thiobacillus"]}},{"id": "denovo10312", "metadata": {"taxonomy": ["Bacteria", "__Actinobacteria", "__Acidimicrobiia", "__Acidimicrobiales", "__Acidimicrobiaceae", "__Ilumatobacter"]}},{"id": "denovo10313", "metadata": {"taxonomy": ["Bacteria", "__Spirochaetes", "__Spirochaetes", "__Spirochaetales", "__Spirochaetaceae", "__Spirochaeta"]}},{"id": "denovo10314", "metadata": {"taxonomy": ["Bacteria", "__Proteobacteria", "__Alphaproteobacteria", "__Caulobacterales", "__Hyphomonadaceae", "__Hellea"]}},{"id": "denovo10315", "metadata": {"taxonomy": ["Bacteria", "__Proteobacteria", "__Deltaproteobacteria", "__Desulfobacterales", "__Desulfobacteraceae", "__g"]}},{"id": "denovo10323", "metadata": {"taxonomy": ["Bacteria", "__Proteobacteria", "__Gammaproteobacteria", "__KI89A_clade", "__f", "__g"]}},{"id": "denovo10324", "metadata": {"taxonomy": ["Bacteria", "__Proteobacteria", "__Gammaproteobacteria", "__E01-9C-26_marine_group", "__f", "__g"]}},{"id": "denovo10332", "metadata": {"taxonomy": ["Bacteria", "__Planctomycetes", "__Pla3_lineage", "__o", "__f", "__g"]}},{"id": "denovo10336", "metadata": {"taxonomy": ["Bacteria", "__Cyanobacteria", "__Cyanobacteria", "__SubsectionIV"]}},{"id": "denovo10337", "metadata": {"taxonomy": ["Bacteria", "__Proteobacteria", "__Deltaproteobacteria", "__GR-WP33-30", "__f", "__g"]}},{"id": "denovo10339", "metadata": {"taxonomy": ["Bacteria", "__Proteobacteria", "__Deltaproteobacteria", "__Desulfobacterales", "__Desulfobulbaceae", "__Desulfopila"]}},{"id": "denovo10341", "metadata": {"taxonomy": ["Bacteria", "__Verrucomicrobia", "__Verrucomicrobiae", "__Verrucomicrobiales", "__Verrucomicrobiaceae", "__Roseibacillus"]}},{"id": "denovo10350", "metadata": {"taxonomy": ["Bacteria", "__Bacteroidetes", "__Flavobacteria", "__Flavobacteriales", "__Flavobacteriaceae", "__Psychroserpens"]}},

{"id": "denovo10352", "metadata": {"taxonomy": ["Bacteria", "__Proteobacteria", "__Gammaproteobacteria", "__Alteromonadales", "__Colwelliaceae", "__Thalassomonas"]}},{"id": "denovo10354", "metadata": {"taxonomy": ["Bacteria", "__Proteobacteria", "__Alphaproteobacteria", "__Rhodobacterales", "__Rhodobacteraceae"]}}, {"id": "denovo10355", "metadata": {"taxonomy": ["Bacteria", "__Chloroflexi", "__S085", "__o", "__f", "__g"]}},{"id": "denovo10360", "metadata": {"taxonomy": ["Bacteria", "__Firmicutes", "__Clostridia", "__Halanaerobiales", "__64K2", "__g"]}},{"id": "denovo10365", "metadata": {"taxonomy": ["Bacteria", "__Proteobacteria", "__Alphaproteobacteria", "__Rhodobacterales", "__Rhodobacteraceae"]}},{"id": "denovo10368", "metadata": {"taxonomy": ["Bacteria", "__Proteobacteria", "__Gammaproteobacteria", "__Alteromonadales", "__Colwelliaceae", "__Thalassomonas"]}},{"id": "denovo10372", "metadata": {"taxonomy": ["Bacteria", "__Proteobacteria", "__Gammaproteobacteria", "__Pseudomonadales", "__Moraxellaceae", "__Acinetobacter"]}},{"id": "denovo10377", "metadata": {"taxonomy": ["Bacteria", "__Proteobacteria", "__Alphaproteobacteria", "__Rhizobiales", "__Hyphomicrobiaceae", "__g"]}},{"id": "denovo10380", "metadata": {"taxonomy": ["Bacteria", "__Planctomycetes", "__Planctomycetacia", "__Planctomycetales", "__Planctomycetaceae", "__Pir4_lineage"]}},{"id": "denovo10381", "metadata": {"taxonomy": ["Bacteria", "__Proteobacteria", "__Alphaproteobacteria", "__Rickettsiales"]}},{"id": "denovo10382", "metadata": {"taxonomy": ["Bacteria", "__Proteobacteria", "__Alphaproteobacteria", "__Rhodospirillales", "__Rhodospirillaceae", "__Defluviicoccus"]}},{"id": "denovo10386", "metadata": {"taxonomy": ["Bacteria", "__Proteobacteria", "__Alphaproteobacteria", "__Rickettsiales", "__SM2D12", "__g"]}},{"id": "denovo10389", "metadata": {"taxonomy": ["Archaea", "__Euryarchaeota", "__Halobacteria", "__Halobacteriales", "__Deep_Sea_Hydrothermal_Vent_Gp_6(DHVEG-6)", "__Candidatus_Parvarchaeum"]}},{"id": "denovo10391", "metadata": {"taxonomy": ["Bacteria", "__Bacteroidetes", "__Sphingobacteriia", "__Sphingobacteriales", "__Saprospiraceae", "__g"]}},{"id": "denovo10392", "metadata": {"taxonomy": ["Bacteria", "__Proteobacteria", "__Deltaproteobacteria", "__Myxococcales", "__Cystobacterineae", "__g"]}},{"id": "denovo10394", "metadata": {"taxonomy": ["Bacteria", "__Proteobacteria", "__Deltaproteobacteria", "__Desulfobacterales", "__Desulfobacteraceae", "__Sva0081_sediment_group"]}},{"id": "denovo10396", "metadata": {"taxonomy": ["Bacteria", "__Bacteroidetes", "__Cytophagia", "__Cytophagales", "__Flammeovirgaceae", "__Ekhidna"]}},{"id": "denovo10398", "metadata": {"taxonomy": ["Bacteria", "__Firmicutes", "__Clostridia", "__Halanaerobiales", "__Halobacteroidaceae", "__g"]}}, {"id": "denovo10399", "metadata": {"taxonomy": ["Bacteria", "__Proteobacteria", "__Alphaproteobacteria", "__Rhizobiales", "__Brucellaceae"]}},{"id": "denovo10400", "metadata": {"taxonomy": ["Bacteria", "__Bacteroidetes", "__Sphingobacteriia", "__Sphingobacteriales", "__PHOS-HE51", "__g"]}},{"id": "denovo10403",

"metadata": {"taxonomy": ["Bacteria", "__BD1-5", "__c", "__o", "__f", "__g"]}},{"id": "denovo10405", "metadata": {"taxonomy": ["Bacteria", "__Proteobacteria", "__Alphaproteobacteria", "__Rhodospirillales", "__Rhodospirillaceae"]}},{"id": "denovo10407", "metadata": {"taxonomy": ["Bacteria", "__Acidobacteria", "__Acidobacteria", "__DA023", "__f", "__g"]}},{"id": "denovo10409", "metadata": {"taxonomy": ["Bacteria", "__Proteobacteria", "__Gammaproteobacteria", "__Chromatiales", "__Chromatiaceae", "__Nitrosococcus"]}},{"id": "denovo10410", "metadata": {"taxonomy": ["Bacteria", "__Proteobacteria", "__Deltaproteobacteria", "__Apal-E12", "__f", "__g"]}},{"id": "denovo10414", "metadata": {"taxonomy": ["Bacteria", "__Proteobacteria", "__Alphaproteobacteria", "__Rhizobiales", "__Phyllobacteriaceae"]}},{"id": "denovo10421", "metadata": {"taxonomy": ["Bacteria", "__Acidobacteria", "__Holophagae"]}},{"id": "denovo10423", "metadata": {"taxonomy": ["Bacteria", "__Proteobacteria"]}},{"id": "denovo10425", "metadata": {"taxonomy": ["Bacteria", "__Proteobacteria", "__Deltaproteobacteria", "__Bdellovibrionales", "__Bacteriovoracaceae", "__Peredibacter"]}},{"id": "denovo10433", "metadata": {"taxonomy": ["Bacteria", "__Proteobacteria", "__Alphaproteobacteria", "__Rhodospirillales", "__Rhodospirillaceae", "__Defluviicoccus"]}},{"id": "denovo10441", "metadata": {"taxonomy": ["Bacteria", "__Proteobacteria", "__Gammaproteobacteria", "__Order_Incertae_Sedis", "__Family_Incertae_Sedis", "__Marinicella"]}},{"id": "denovo10442", "metadata": {"taxonomy": ["Bacteria", "__Firmicutes", "__Bacilli", "__Lactobacillales", "__Streptococcaceae", "__Streptococcus"]}},{"id": "denovo10443", "metadata": {"taxonomy": ["Bacteria", "__Proteobacteria", "__Deltaproteobacteria", "__Sh765B-TzT-29", "__f", "__g"]}},{"id": "denovo10445", "metadata": {"taxonomy": ["Bacteria", "__Proteobacteria", "__Deltaproteobacteria", "__Desulfobacterales", "__Desulfobulbaceae", "__Desulfobulbus"]}},{"id": "denovo10450", "metadata": {"taxonomy": ["Bacteria", "__Proteobacteria", "__Gammaproteobacteria", "__Oceanospirillales", "__Oceanospirillaceae", "__g"]}},{"id": "denovo10459", "metadata": {"taxonomy": ["Bacteria", "__Firmicutes", "__Clostridia", "__Halanaerobiales", "__Halobacteroidaceae", "__g"]}},{"id": "denovo10463", "metadata": {"taxonomy": ["Bacteria", "__Proteobacteria", "__Gammaproteobacteria", "__KI89A_clade", "__f", "__g"]}},{"id": "denovo10465", "metadata": {"taxonomy": ["Bacteria", "__Proteobacteria", "__Gammaproteobacteria", "__Oceanospirillales", "__OM182_clade", "__g"]}},{"id": "denovo10466", "metadata": {"taxonomy": ["Bacteria", "__Bacteroidetes", "__Cytophagia", "__Cytophagales", "__Flammeovirgaceae", "__g"]}},{"id": "denovo10471", "metadata": {"taxonomy": ["Bacteria", "__Proteobacteria", "__Alphaproteobacteria", "__Rhodobacterales", "__Rhodobacteraceae", "__Roseobacter_clade_OCT_lineage"]}},{"id": "denovo10472", "metadata": {"taxonomy": ["Bacteria", "__Proteobacteria", "__Deltaproteobacteria", "__Desulfobacterales", "__Desulfobacteraceae"]}},{"id": "denovo10474", "metadata": {"taxonomy": ["Bacteria", "__Proteobacteria", "__Alphaproteobacteria", "__DB1-14", "__f", "__g"]}},{"id":

"denovo10475", "metadata": {"taxonomy": ["Bacteria", "__Proteobacteria", "__Gammaproteobacteria", "__Vibrionales", "__Vibrionaceae", "__Photobacterium"]}},{"id": "denovo10476", "metadata": {"taxonomy": ["Bacteria", "__Cyanobacteria", "__Cyanobacteria", "__SubsectionI", "__FamilyI", "__Synechococcus"]}}, {"id": "denovo10477", "metadata": {"taxonomy": ["Archaea", "__Euryarchaeota", "__Halobacteria", "__Halobacteriales", "__Deep_Sea_Hydrothermal_Vent_Gp_6(DHVEG-6)", "__g"]}},{"id": "denovo10479", "metadata": {"taxonomy": ["Bacteria", "__Proteobacteria", "__Alphaproteobacteria", "__Rhodobacterales", "__Rhodobacteraceae"]}},{"id": "denovo10480", "metadata": {"taxonomy": ["Bacteria", "__Acidobacteria", "__RB25", "__o", "__f", "__g"]}}, {"id": "denovo10485", "metadata": {"taxonomy": ["Bacteria", "__Proteobacteria", "__Deltaproteobacteria", "__Myxococcales", "__0319-6G20", "__g"]}},{"id": "denovo10488", "metadata": {"taxonomy": ["Bacteria", "__Proteobacteria", "__Alphaproteobacteria", "__Rhizobiales"]}},{"id": "denovo10492", "metadata": {"taxonomy": ["Bacteria", "__TM6", "__c", "__o", "__f", "__g"]}},{"id": "denovo10493", "metadata": {"taxonomy": ["Bacteria", "__Bacteroidetes", "__Sphingobacteriia", "__Sphingobacteriales", "__Saprospiraceae", "__g"]}},{"id": "denovo10495", "metadata": {"taxonomy": ["Bacteria", "__Cyanobacteria", "__Cyanobacteria", "__SubsectionI", "__FamilyI", "__g"]}},{"id": "denovo10502", "metadata": {"taxonomy": ["Bacteria", "__Proteobacteria", "__Gammaproteobacteria", "__Thiotrichales", "__Piscirickettsiaceae", "__g"]}},{"id": "denovo10515", "metadata": {"taxonomy": ["Bacteria", "__Proteobacteria", "__Alphaproteobacteria", "__Rhodospirillales", "__Rhodospirillaceae", "__Defluviicoccus"]}},{"id": "denovo10517", "metadata": {"taxonomy": ["Bacteria", "__Proteobacteria", "__Alphaproteobacteria", "__Rhodobacterales", "__Rhodobacteraceae", "__Roseobacter_clade_OCT_lineage"]}},{"id": "denovo10520", "metadata": {"taxonomy": ["Bacteria", "__Proteobacteria", "__Gammaproteobacteria", "__Pseudomonadales", "__Moraxellaceae", "__Acinetobacter"]}},{"id": "denovo10523", "metadata": {"taxonomy": ["Bacteria", "__Proteobacteria", "__Alphaproteobacteria", "__Rhodobacterales", "__Rhodobacteraceae", "__Tropicimonas"]}},{"id": "denovo10524", "metadata": {"taxonomy": ["Bacteria", "__Proteobacteria", "__Deltaproteobacteria", "__Desulfobacterales", "__Nitrospinaceae", "__Candidatus_Entotheonella"]}},{"id": "denovo10528", "metadata": {"taxonomy": ["Bacteria", "__Bacteroidetes", "__Sphingobacteriia", "__Sphingobacteriales", "__Saprospiraceae", "__g"]}},{"id": "denovo10529", "metadata": {"taxonomy": ["Bacteria", "__Proteobacteria", "__Alphaproteobacteria", "__S26-47", "__f", "__g"]}},{"id": "denovo10531", "metadata": {"taxonomy": ["Bacteria", "__Acidobacteria", "__Acidobacteria", "__DA023", "__f", "__g"]}}, {"id": "denovo10534", "metadata": {"taxonomy": ["Bacteria", "__Proteobacteria", "__Alphaproteobacteria", "__Rhodospirillales", "__Rhodospirillaceae", "__g"]}},{"id": "denovo10535", "metadata": {"taxonomy": ["Bacteria", "__Proteobacteria", "__Gammaproteobacteria", "__Oceanospirillales", "__SAR86_clade", "__g"]}},{"id": "denovo10536",

"metadata": {"taxonomy": ["Bacteria", "__Proteobacteria",
"__Deltaproteobacteria", "__Sh765B-TzT-29", "__f", "__g"]}},{"id":
"denovo10537", "metadata": {"taxonomy": ["Bacteria",
"__Bacteroidetes", "__Flavobacteria", "__Flavobacteriales",
"__Flavobacteriaceae"]}},{"id": "denovo10543", "metadata":
{"taxonomy": ["Bacteria", "__Proteobacteria", "__Alphaproteobacteria",
"__OCS116_clade", "__f", "__g"]}},{"id": "denovo10548", "metadata":
{"taxonomy": ["Bacteria", "__Lentisphaerae", "__Lentisphaeria",
"__Victivallales", "__Victivallaceae", "__g"]}},{"id": "denovo10550",
"metadata": {"taxonomy": ["Bacteria", "__Proteobacteria",
"__Gammaproteobacteria", "__Alteromonadales", "__Colwelliaceae",
"__Thalassomonas"]}},{"id": "denovo10553", "metadata": {"taxonomy":
["Bacteria", "__Proteobacteria", "__Gammaproteobacteria",
"__Pseudomonadales", "__Moraxellaceae", "__Acinetobacter"]}},{"id":
"denovo10555", "metadata": {"taxonomy": ["Bacteria",
"__Proteobacteria"]}},{"id": "denovo10562", "metadata": {"taxonomy":
["Bacteria", "__Actinobacteria", "__Actinobacteria",
"__Micrococcales", "__Micrococcaceae", "__Kocuria"]}},{"id":
"denovo10563", "metadata": {"taxonomy": ["Bacteria", "__Chlamydiae",
"__Chlamydiae", "__Chlamydiales", "__Simkaniaceae",
"__Candidatus_Fritschea"]}},{"id": "denovo10564", "metadata":
{"taxonomy": ["Bacteria", "__Bacteroidetes", "__Flavobacteria",
"__Flavobacteriales", "__Cryomorphaceae", "__Crocinitomix"]}},{"id":
"denovo10570", "metadata": {"taxonomy": ["Bacteria",
"__Proteobacteria", "__Alphaproteobacteria", "__Rhodobacterales",
"__Rhodobacteraceae"]}},{"id": "denovo10571", "metadata": {"taxonomy":
["Bacteria", "__Proteobacteria", "__Alphaproteobacteria",
"__Rhodospirillales", "__Rhodospirillaceae", "__Defluviicoccus"]}},
{"id": "denovo10572", "metadata": {"taxonomy": ["Bacteria",
"__Bacteroidetes", "__Flavobacteria", "__Flavobacteriales",
"__Flavobacteriaceae", "__NS5_marine_group"]}},{"id": "denovo10573",
"metadata": {"taxonomy": ["Bacteria", "__Proteobacteria",
"__Gammaproteobacteria", "__Oceanospirillales",
"__Oceanospirillaceae"]}},{"id": "denovo10580", "metadata":
{"taxonomy": ["Bacteria", "__Proteobacteria", "__Gammaproteobacteria",
"__Pseudomonadales", "__Moraxellaceae", "__Acinetobacter"]}},{"id":
"denovo10585", "metadata": {"taxonomy": ["Bacteria",
"__Proteobacteria", "__Gammaproteobacteria", "__Aeromonadales",
"__Aeromonadaceae", "__Aeromonas"]}},{"id": "denovo10588", "metadata":
{"taxonomy": ["Bacteria", "__Proteobacteria", "__Gammaproteobacteria",
"__Alteromonadales", "__Alteromonadaceae", "__BD1-7_clade"]}},{"id":
"denovo10590", "metadata": {"taxonomy": ["Bacteria",
"__Gemmatimonadetes", "__Gemmatimonadetes",
"__BD2-11_terrestrial_group", "__f", "__g"]}},{"id": "denovo10593",
"metadata": {"taxonomy": ["Bacteria", "__Proteobacteria",
"__Gammaproteobacteria"]}},{"id": "denovo10597", "metadata":
{"taxonomy": ["Bacteria", "__Bacteroidetes", "__Flavobacteria",
"__Flavobacteriales", "__Flavobacteriaceae", "__NS5_marine_group"]}},
{"id": "denovo10598", "metadata": {"taxonomy": ["Bacteria",
"__Cyanobacteria", "__Cyanobacteria"]}},{"id": "denovo10601",

"metadata": {"taxonomy": ["Bacteria", "__Proteobacteria", "__Gammaproteobacteria", "__KI89A_clade", "__f", "__g"]}},{"id": "denovo10609", "metadata": {"taxonomy": ["Bacteria", "__Proteobacteria", "__Deltaproteobacteria", "__Sh765B-TzT-29", "__f", "__g"]}},{"id": "denovo10611", "metadata": {"taxonomy": ["Bacteria", "__Proteobacteria", "__Gammaproteobacteria", "__Alteromonadales", "__Alteromonadaceae", "__BD2-7"]}},{"id": "denovo10613", "metadata": {"taxonomy": ["Bacteria", "__Planctomycetes", "__Phycisphaerae", "__Phycisphaerales", "__Phycisphaeraceae", "__CL500-3"]}},{"id": "denovo10616", "metadata": {"taxonomy": ["Bacteria", "__Firmicutes", "__Clostridia", "__Clostridiales", "__Clostridiaceae", "__Clostridium"]}},{"id": "denovo10622", "metadata": {"taxonomy": ["Bacteria", "__Nitrospirae", "__Nitrospira", "__Nitrospirales", "__Nitrospiraceae", "__Nitrospira"]}},{"id": "denovo10623", "metadata": {"taxonomy": ["Bacteria", "__Firmicutes", "__Clostridia", "__Clostridiales", "__Christensenellaceae", "__Christensenella"]}},{"id": "denovo10625", "metadata": {"taxonomy": ["Bacteria", "__Proteobacteria", "__Gammaproteobacteria", "__Pseudomonadales", "__Moraxellaceae", "__Acinetobacter"]}},{"id": "denovo10627", "metadata": {"taxonomy": ["Bacteria", "__Proteobacteria", "__Alphaproteobacteria", "__Sphingomonadales", "__SWB04", "__g"]}},{"id": "denovo10632", "metadata": {"taxonomy": ["Bacteria", "__Proteobacteria", "__Alphaproteobacteria", "__Rhodospirillales", "__Rhodospirillaceae", "__Defluviicoccus"]}},{"id": "denovo10635", "metadata": {"taxonomy": ["Bacteria", "__Proteobacteria", "__Gammaproteobacteria", "__Alteromonadales", "__Alteromonadaceae", "__C1-B045"]}},{"id": "denovo10639", "metadata": {"taxonomy": ["Bacteria", "__Proteobacteria", "__Gammaproteobacteria"]}},{"id": "denovo10640", "metadata": {"taxonomy": ["Bacteria", "__Verrucomicrobia", "__Opitutae", "__Opitutales", "__Opitutaceae", "__Alterococcus"]}},{"id": "denovo10643", "metadata": {"taxonomy": ["Bacteria", "__Acidobacteria", "__RB25", "__o", "__f", "__g"]}},{"id": "denovo10644", "metadata": {"taxonomy": ["Bacteria", "__Proteobacteria", "__Alphaproteobacteria", "__Rhodobacterales", "__Rhodobacteraceae"]}},{"id": "denovo10648", "metadata": {"taxonomy": ["Bacteria", "__Proteobacteria", "__Deltaproteobacteria", "__Myxococcales", "__Haliangiaceae", "__Haliangium"]}},{"id": "denovo10649", "metadata": {"taxonomy": ["Bacteria", "__Proteobacteria", "__Alphaproteobacteria", "__Rhodospirillales", "__Rhodospirillaceae", "__g"]}},{"id": "denovo10651", "metadata": {"taxonomy": ["Bacteria", "__Proteobacteria", "__Gammaproteobacteria", "__Xanthomonadales", "__Sinobacteraceae", "__JTB255_marine_benthic_group"]}},{"id": "denovo10653", "metadata": {"taxonomy": ["Bacteria", "__Proteobacteria", "__Gammaproteobacteria", "__KI89A_clade", "__f", "__g"]}},{"id": "denovo10655", "metadata": {"taxonomy": ["Archaea", "__Euryarchaeota", "__Thermoplasmata", "__Thermoplasmatales", "__Marine_Group_II", "__g"]}},{"id": "denovo10659", "metadata": {"taxonomy": ["Bacteria", "__Chlorobi", "__Ignavibacteria", "__Ignavibacteriales", "__IheB3-7", "__g"]}},{"id": "denovo10660", "metadata": {"taxonomy": ["Bacteria",

"__Proteobacteria", "__Alphaproteobacteria", "__Rhodospirillales", "__Rhodospirillaceae", "__OM75_clade"]}},{"id": "denovo10661", "metadata": {"taxonomy": ["Bacteria", "__Acidobacteria", "__Holophagae", "__Sva0725", "__f", "__g"]}},{"id": "denovo10663", "metadata": {"taxonomy": ["Bacteria", "__Proteobacteria", "__Alphaproteobacteria", "__Rhodobacterales", "__Rhodobacteraceae", "__Ruegeria"]}},{"id": "denovo10665", "metadata": {"taxonomy": ["Bacteria", "__Proteobacteria", "__Alphaproteobacteria", "__Rhodospirillales", "__Rhodospirillaceae"]}},{"id": "denovo10667", "metadata": {"taxonomy": ["Archaea", "__Euryarchaeota", "__Halobacteria", "__Halobacteriales", "__Deep_Sea_Hydrothermal_Vent_Gp_6(DHVEG-6)", "__g"]}},{"id": "denovo10668", "metadata": {"taxonomy": ["Bacteria", "__Bacteroidetes", "__Cytophagia", "__Order_II_Incertae_Sedis", "__Rhodothermaceae", "__g"]}},{"id": "denovo10669", "metadata": {"taxonomy": ["Bacteria", "__Proteobacteria", "__Deltaproteobacteria", "__Myxococcales", "__0319-6G20", "__g"]}},{"id": "denovo10674", "metadata": {"taxonomy": ["Bacteria", "__Proteobacteria", "__Gammaproteobacteria"]}},{"id": "denovo10678", "metadata": {"taxonomy": ["Bacteria", "__Proteobacteria", "__Gammaproteobacteria", "__KI89A_clade", "__f", "__g"]}},{"id": "denovo10679", "metadata": {"taxonomy": ["Bacteria", "__Actinobacteria", "__Acidimicrobiia", "__Acidimicrobiales", "__uncultured", "__g"]}},{"id": "denovo10681", "metadata": {"taxonomy": ["Bacteria", "__Proteobacteria", "__Gammaproteobacteria", "__Legionellales", "__Coxiellaceae", "__Coxiella"]}},{"id": "denovo10682", "metadata": {"taxonomy": ["Bacteria", "__Proteobacteria", "__Deltaproteobacteria"]}},{"id": "denovo10684", "metadata": {"taxonomy": ["Bacteria", "__Cyanobacteria", "__Cyanobacteria", "__SubsectionI", "__FamilyI", "__Acaryochloris"]}},{"id": "denovo10686", "metadata": {"taxonomy": ["Bacteria", "__Proteobacteria", "__Alphaproteobacteria", "__Sphingomonadales", "__Sphingomonadaceae", "__Sphingomonas"]}},{"id": "denovo10687", "metadata": {"taxonomy": ["Bacteria", "__Proteobacteria", "__Alphaproteobacteria", "__Rhizobiales", "__Brucellaceae", "__g"]}},{"id": "denovo10689", "metadata": {"taxonomy": ["Bacteria", "__Chloroflexi", "__Anaerolineae", "__Anaerolineales", "__Anaerolineaceae", "__g"]}},{"id": "denovo10690", "metadata": {"taxonomy": ["Bacteria", "__Proteobacteria", "__Alphaproteobacteria", "__Rhodobacterales", "__Rhodobacteraceae"]}},{"id": "denovo10693", "metadata": {"taxonomy": ["Bacteria", "__Cyanobacteria", "__Cyanobacteria", "__SubsectionII", "__FamilyII", "__Pleurocapsa"]}},{"id": "denovo10694", "metadata": {"taxonomy": ["Bacteria", "__Bacteroidetes", "__Sphingobacteriia", "__Sphingobacteriales", "__Saprospiraceae", "__g"]}},{"id": "denovo10698", "metadata": {"taxonomy": ["Bacteria", "__Proteobacteria", "__Alphaproteobacteria", "__Rhodospirillales", "__Rhodospirillaceae", "__Defluviicoccus"]}},{"id": "denovo10699", "metadata": {"taxonomy": ["Bacteria", "__Proteobacteria", "__Alphaproteobacteria", "__Rhodobacterales", "__Rhodobacteraceae", "__Rhodovulum"]}},{"id": "denovo10709", "metadata": {"taxonomy":

["Bacteria", "__Proteobacteria", "__Gammaproteobacteria"]}},{"id": "denovo10715", "metadata": {"taxonomy": ["Bacteria", "__Proteobacteria", "__Gammaproteobacteria", "__Xanthomonadales", "__Sinobacteraceae", "__JTB255_marine_benthic_group"]}},{"id": "denovo10716", "metadata": {"taxonomy": ["Bacteria", "__Proteobacteria", "__Deltaproteobacteria", "__SAR324_clade(Marine_group_B)", "__f", "__g"]}},{"id": "denovo10717", "metadata": {"taxonomy": ["Bacteria", "__Proteobacteria", "__Gammaproteobacteria", "__Oceanospirillales", "__Oceanospirillaceae", "__Marinobacterium"]}},{"id": "denovo10718", "metadata": {"taxonomy": ["Bacteria", "__Proteobacteria", "__Gammaproteobacteria", "__Pseudomonadales", "__Moraxellaceae", "__Acinetobacter"]}},{"id": "denovo10719", "metadata": {"taxonomy": ["Bacteria", "__Proteobacteria", "__Gammaproteobacteria", "__Run-SP154", "__f", "__g"]}},{"id": "denovo10720", "metadata": {"taxonomy": ["Bacteria", "__Proteobacteria", "__Alphaproteobacteria", "__DB1-14", "__f", "__g"]}},{"id": "denovo10726", "metadata": {"taxonomy": ["Bacteria", "__Proteobacteria", "__Gammaproteobacteria", "__Alteromonadales", "__Alteromonadaceae", "__Melitea"]}},{"id": "denovo10727", "metadata": {"taxonomy": ["Bacteria", "__Proteobacteria", "__Alphaproteobacteria", "__Rhodobacterales", "__Rhodobacteraceae"]}},{"id": "denovo10730", "metadata": {"taxonomy": ["Bacteria", "__Nitrospirae", "__Nitrospira", "__Nitrospirales", "__OPB95", "__g"]}},{"id": "denovo10731", "metadata": {"taxonomy": ["Bacteria", "__Cyanobacteria", "__Cyanobacteria", "__SubsectionI", "__FamilyI"]}},{"id": "denovo10733", "metadata": {"taxonomy": ["Bacteria", "__Proteobacteria", "__Gammaproteobacteria"]}},{"id": "denovo10739", "metadata": {"taxonomy": ["Bacteria", "__Actinobacteria", "__Acidimicrobiia", "__Acidimicrobiales", "__Sva0996_marine_group", "__g"]}},{"id": "denovo10740", "metadata": {"taxonomy": ["Bacteria", "__Proteobacteria", "__Gammaproteobacteria", "__Alteromonadales", "__Alteromonadaceae", "__Haliea"]}},{"id": "denovo10745", "metadata": {"taxonomy": ["Bacteria", "__Actinobacteria", "__Acidimicrobiia", "__Acidimicrobiales", "__Sva0996_marine_group", "__g"]}},{"id": "denovo10747", "metadata": {"taxonomy": ["Bacteria", "__Chloroflexi", "__Caldilineae", "__Caldilineales", "__Caldilineaceae", "__Caldilinea"]}},{"id": "denovo10751", "metadata": {"taxonomy": ["Bacteria", "__Proteobacteria", "__Gammaproteobacteria", "__Alteromonadales", "__Alteromonadaceae", "__Eionea"]}},{"id": "denovo10752", "metadata": {"taxonomy": ["Archaea", "__Thaumarchaeota", "__Soil_Crenarchaeotic_Group(SCG)", "__o", "__f", "__g"]}},{"id": "denovo10753", "metadata": {"taxonomy": ["Bacteria", "__Proteobacteria", "__Alphaproteobacteria"]}},{"id": "denovo10755", "metadata": {"taxonomy": ["Bacteria", "__Proteobacteria", "__Gammaproteobacteria", "__Chromatiales", "__Chromatiaceae", "__Nitrosococcus"]}},{"id": "denovo10757", "metadata": {"taxonomy": ["Bacteria", "__Deferribacteres", "__Deferribacteres", "__Deferribacterales", "__SAR406_clade(Marine_group_A)", "__g"]}},
{"id": "denovo10762", "metadata": {"taxonomy": ["Bacteria",

"__Proteobacteria", "__Gammaproteobacteria", "__Vibrionales", "__Vibrionaceae", "__Vibrio"]}},{"id": "denovo10764", "metadata": {"taxonomy": ["Bacteria", "__Proteobacteria", "__Gammaproteobacteria"]}},{"id": "denovo10765", "metadata": {"taxonomy": ["Bacteria", "__Firmicutes", "__Clostridia", "__Clostridiales", "__Kazan-2B-17", "__g"]}},{"id": "denovo10767", "metadata": {"taxonomy": ["Bacteria", "__Proteobacteria", "__Deltaproteobacteria", "__Desulfobacterales", "__Desulfobulbaceae"]}},{"id": "denovo10773", "metadata": {"taxonomy": ["Bacteria", "__Proteobacteria", "__Alphaproteobacteria", "__Rhodospirillales", "__Rhodospirillaceae", "__Pelagibius"]}},{"id": "denovo10774", "metadata": {"taxonomy": ["Bacteria", "__Proteobacteria", "__Deltaproteobacteria", "__Bdellovibrionales", "__Bdellovibrionaceae", "__OM27_clade"]}},{"id": "denovo10776", "metadata": {"taxonomy": ["Bacteria", "__Proteobacteria", "__Alphaproteobacteria", "__Rhodobacterales", "__Rhodobacteraceae", "__g"]}},{"id": "denovo10777", "metadata": {"taxonomy": ["Archaea", "__Euryarchaeota", "__Halobacteria", "__Halobacteriales", "__Deep_Sea_Hydrothermal_Vent_Gp_6(DHVEG-6)", "__Candidatus_Parvarchaeum"]}},{"id": "denovo10778", "metadata": {"taxonomy": ["Bacteria", "__Proteobacteria", "__Alphaproteobacteria", "__Rickettsiales"]}},{"id": "denovo10780", "metadata": {"taxonomy": ["Bacteria", "__Acidobacteria", "__RB25", "__o", "__f", "__g"]}},{"id": "denovo10781", "metadata": {"taxonomy": ["Bacteria", "__Proteobacteria", "__Alphaproteobacteria", "__Caulobacterales", "__Hyphomonadaceae", "__Hyphomonas"]}},{"id": "denovo10782", "metadata": {"taxonomy": ["Bacteria", "__Proteobacteria", "__Gammaproteobacteria", "__Vibrionales", "__Vibrionaceae", "__Enterovibrio"]}},{"id": "denovo10783", "metadata": {"taxonomy": ["Bacteria", "__Proteobacteria", "__Gammaproteobacteria", "__Alteromonadales", "__Alteromonadaceae", "__Teredinibacter"]}},{"id": "denovo10784", "metadata": {"taxonomy": ["Bacteria", "__Proteobacteria", "__Elev-16S-509", "__o", "__f", "__g"]}},{"id": "denovo10785", "metadata": {"taxonomy": ["Bacteria", "__Bacteroidetes", "__Flavobacteria", "__Flavobacteriales", "__Flavobacteriaceae", "__Polaribacter"]}},{"id": "denovo10788", "metadata": {"taxonomy": ["Bacteria", "__Actinobacteria", "__Acidimicrobiia", "__Acidimicrobiales", "__uncultured", "__g"]}},{"id": "denovo10790", "metadata": {"taxonomy": ["Bacteria", "__Chloroflexi", "__SAR202_clade", "__o", "__f", "__g"]}},{"id": "denovo10792", "metadata": {"taxonomy": ["Bacteria", "__Proteobacteria", "__Alphaproteobacteria", "__Rhodobacterales", "__Rhodobacteraceae", "__Pseudovibrio"]}},{"id": "denovo10796", "metadata": {"taxonomy": ["Bacteria", "__Chloroflexi", "__Anaerolineae", "__Anaerolineales", "__Anaerolineaceae", "__g"]}},{"id": "denovo10797", "metadata": {"taxonomy": ["Bacteria", "__Chloroflexi", "__JG30-KF-CM66", "__o", "__f", "__g"]}},{"id": "denovo10798", "metadata": {"taxonomy": ["Bacteria", "__Proteobacteria", "__Gammaproteobacteria", "__Oceanospirillales", "__MBAE14", "__g"]}},{"id": "denovo10802", "metadata": {"taxonomy":

["Bacteria", "__Proteobacteria", "__Alphaproteobacteria", "__Rhodobacterales", "__Rhodobacteraceae", "__Dinoroseobacter"]}}, {"id": "denovo10806", "metadata": {"taxonomy": ["Bacteria", "__Proteobacteria", "__Betaproteobacteria", "__Burkholderiales", "__Burkholderiaceae", "__Ralstonia"]}},{"id": "denovo10808", "metadata": {"taxonomy": ["Bacteria", "__Acidobacteria", "__Holophagae", "__NKB17", "__f", "__g"]}},{"id": "denovo10811", "metadata": {"taxonomy": ["Bacteria", "__Proteobacteria", "__Alphaproteobacteria", "__Rhodospirillales", "__Rhodospirillaceae", "__Defluviicoccus"]}},{"id": "denovo10812", "metadata": {"taxonomy": ["Bacteria", "__Proteobacteria", "__Alphaproteobacteria", "__Caulobacterales", "__Caulobacteraceae", "__Brevundimonas"]}},{"id": "denovo10818", "metadata": {"taxonomy": ["Bacteria", "__Proteobacteria", "__Alphaproteobacteria", "__Rhizobiales", "__Rhodobiaceae", "__Rhodobium"]}},{"id": "denovo10819", "metadata": {"taxonomy": ["Bacteria", "__Proteobacteria", "__Alphaproteobacteria"]}},{"id": "denovo10822", "metadata": {"taxonomy": ["Bacteria", "__Proteobacteria", "__Gammaproteobacteria", "__Pseudomonadales", "__Moraxellaceae", "__Acinetobacter"]}},{"id": "denovo10824", "metadata": {"taxonomy": ["Bacteria", "__Proteobacteria", "__Gammaproteobacteria", "__Alteromonadales", "__Alteromonadaceae", "__g"]}},{"id": "denovo10826", "metadata": {"taxonomy": ["Bacteria", "__Bacteroidetes", "__Sphingobacteriia", "__Sphingobacteriales", "__Saprospiraceae", "__g"]}},{"id": "denovo10827", "metadata": {"taxonomy": ["Bacteria", "__Proteobacteria", "__Gammaproteobacteria", "__HOC36", "__f", "__g"]}},{"id": "denovo10835", "metadata": {"taxonomy": ["Bacteria", "__Chloroflexi", "__KD4-96", "__o", "__f", "__g"]}},{"id": "denovo10838", "metadata": {"taxonomy": ["Bacteria", "__Proteobacteria", "__Alphaproteobacteria", "__Rhodobacterales", "__Rhodobacteraceae", "__Roseobacter"]}},{"id": "denovo10839", "metadata": {"taxonomy": ["Bacteria", "__Cyanobacteria"]}},{"id": "denovo10840", "metadata": {"taxonomy": ["Bacteria", "__Proteobacteria", "__Alphaproteobacteria", "__Rhodobacterales", "__Rhodobacteraceae"]}},{"id": "denovo10843", "metadata": {"taxonomy": ["Bacteria", "__Actinobacteria", "__Acidimicrobiia", "__Acidimicrobiales", "__Candidatus_Microthrix", "__g"]}},{"id": "denovo10846", "metadata": {"taxonomy": ["Bacteria", "__Firmicutes"]}},{"id": "denovo10849", "metadata": {"taxonomy": ["Archaea", "__Euryarchaeota", "__Thermoplasmata", "__Thermoplasmatales", "__Marine_Group_III", "__g"]}},{"id": "denovo10852", "metadata": {"taxonomy": ["Bacteria", "__Proteobacteria", "__Betaproteobacteria", "__Nitrosomonadales", "__Nitrosomonadaceae", "__Nitrosospira"]}},{"id": "denovo10853", "metadata": {"taxonomy": ["Bacteria", "__Proteobacteria", "__Gammaproteobacteria", "__Alteromonadales", "__Alteromonadaceae", "__Candidatus_Endobugula"]}},{"id": "denovo10855", "metadata": {"taxonomy": ["Bacteria", "__Proteobacteria", "__Gammaproteobacteria", "__Oceanospirillales", "__JL-ETNP-Y6", "__g"]}},{"id": "denovo10856", "metadata": {"taxonomy": ["Bacteria", "__Proteobacteria",

"__Deltaproteobacteria", "__Bdellovibrionales", "__Bdellovibrionaceae", "__Bdellovibrio"]}},{"id": "denovo10858", "metadata": {"taxonomy": ["Bacteria", "__Planctomycetes", "__Phycisphaerae", "__Phycisphaerales", "__Phycisphaeraceae", "__Phycisphaera"]}},{"id": "denovo10860", "metadata": {"taxonomy": ["Bacteria", "__Proteobacteria", "__Gammaproteobacteria", "__Xanthomonadales", "__Sinobacteraceae", "__JTB255_marine_benthic_group"]}},{"id": "denovo10862", "metadata": {"taxonomy": ["Bacteria", "__Candidate_division_WS3", "__c", "__o", "__f", "__g"]}},{"id": "denovo10867", "metadata": {"taxonomy": ["Bacteria", "__Proteobacteria", "__Alphaproteobacteria", "__Rhizobiales", "__Brucellaceae", "__Brucella"]}},{"id": "denovo10869", "metadata": {"taxonomy": ["Bacteria", "__Proteobacteria", "__Deltaproteobacteria", "__Syntrophobacterales", "__Syntrophaceae", "__g"]}},{"id": "denovo10881", "metadata": {"taxonomy": ["Bacteria", "__Proteobacteria", "__Gammaproteobacteria", "__Oceanospirillales", "__Alcanivoracaceae", "__Kangiella"]}},{"id": "denovo10884", "metadata": {"taxonomy": ["Bacteria", "__Proteobacteria", "__Deltaproteobacteria", "__Desulfobacterales", "__Desulfobacteraceae", "__Desulfobacula"]}},{"id": "denovo10895", "metadata": {"taxonomy": ["Bacteria", "__Bacteroidetes", "__Sphingobacteriia", "__Sphingobacteriales", "__Saprospiraceae", "__g"]}},{"id": "denovo10900", "metadata": {"taxonomy": ["Bacteria", "__Bacteroidetes", "__Sphingobacteriia", "__Sphingobacteriales", "__Saprospiraceae", "__g"]}},{"id": "denovo10911", "metadata": {"taxonomy": ["Bacteria", "__Bacteroidetes", "__Flavobacteria", "__Flavobacteriales", "__Flavobacteriaceae", "__Maritimimonas"]}},{"id": "denovo10912", "metadata": {"taxonomy": ["Bacteria", "__Proteobacteria", "__Gammaproteobacteria", "__Chromatiales", "__Ectothiorhodospiraceae"]}},{"id": "denovo10915", "metadata": {"taxonomy": ["Bacteria", "__Cyanobacteria", "__Cyanobacteria", "__SubsectionI", "__FamilyI", "__g"]}},{"id": "denovo10918", "metadata": {"taxonomy": ["Bacteria", "__Proteobacteria", "__Gammaproteobacteria", "__Enterobacteriales", "__Enterobacteriaceae"]}},{"id": "denovo10920", "metadata": {"taxonomy": ["Bacteria", "__Cyanobacteria", "__Cyanobacteria", "__SubsectionIII", "__FamilyI", "__Phormidium"]}},{"id": "denovo10927", "metadata": {"taxonomy": ["Bacteria", "__Proteobacteria", "__Betaproteobacteria", "__Burkholderiales", "__Burkholderiaceae", "__Limnobacter"]}},{"id": "denovo10929", "metadata": {"taxonomy": ["Archaea", "__Euryarchaeota", "__Halobacteria", "__Halobacteriales", "__Deep_Sea_Hydrothermal_Vent_Gp_6(DHVEG-6)", "__g"]}},{"id": "denovo10933", "metadata": {"taxonomy": ["Bacteria", "__Proteobacteria", "__Gammaproteobacteria", "__Legionellales", "__Coxiellaceae", "__Coxiella"]}},{"id": "denovo10935", "metadata": {"taxonomy": ["Bacteria", "__Proteobacteria", "__Gammaproteobacteria", "__Alteromonadales", "__Alteromonadaceae", "__Haliea"]}},{"id": "denovo10937", "metadata": {"taxonomy": ["Bacteria", "__Proteobacteria", "__Gammaproteobacteria", "__Oceanospirillales",

"__Oceanospirillaceae", "__Neptunomonas"]}},{"id": "denovo10943", "metadata": {"taxonomy": ["Bacteria", "__Planctomycetes", "__Phycisphaerae", "__Phycisphaerales", "__Phycisphaeraceae", "__Phycisphaera"]}},{"id": "denovo10945", "metadata": {"taxonomy": ["Bacteria", "__Proteobacteria", "__Deltaproteobacteria", "__Sh765B-TzT-29", "__f", "__g"]}},{"id": "denovo10946", "metadata": {"taxonomy": ["Bacteria", "__Cyanobacteria", "__Cyanobacteria", "__SubsectionIV", "__FamilyII", "__Calothrix"]}},{"id": "denovo10947", "metadata": {"taxonomy": ["Bacteria", "__Proteobacteria", "__Deltaproteobacteria", "__Myxococcales"]}},{"id": "denovo10948", "metadata": {"taxonomy": ["Bacteria", "__Proteobacteria", "__Alphaproteobacteria", "__Rhodobacterales", "__Rhodobacteraceae"]}},{"id": "denovo10951", "metadata": {"taxonomy": ["Bacteria", "__Proteobacteria", "__SPOTSOCT00m83", "__o", "__f", "__g"]}},{"id": "denovo10953", "metadata": {"taxonomy": ["Bacteria", "__Proteobacteria", "__Gammaproteobacteria", "__HOC36", "__f", "__g"]}},{"id": "denovo10954", "metadata": {"taxonomy": ["Bacteria", "__Acidobacteria", "__RB25", "__o", "__f", "__g"]}},{"id": "denovo10955", "metadata": {"taxonomy": ["Bacteria", "__Bacteroidetes", "__Flavobacteria", "__Flavobacteriales", "__Flavobacteriaceae"]}},{"id": "denovo10957", "metadata": {"taxonomy": ["Bacteria", "__Proteobacteria", "__SPOTSOCT00m83", "__o", "__f", "__g"]}},{"id": "denovo10958", "metadata": {"taxonomy": ["Bacteria", "__Proteobacteria", "__Gammaproteobacteria", "__Legionellales", "__Coxiellaceae", "__Coxiella"]}},{"id": "denovo10959", "metadata": {"taxonomy": ["Bacteria", "__Bacteroidetes", "__Cytophagia", "__Cytophagales", "__Flammeovirgaceae", "__Candidatus_Amoebophilus"]}},{"id": "denovo10963", "metadata": {"taxonomy": ["Bacteria", "__Bacteroidetes", "__Cytophagia", "__Cytophagales", "__Flammeovirgaceae", "__Reichenbachiella"]}},{"id": "denovo10968", "metadata": {"taxonomy": ["Bacteria", "__Proteobacteria", "__Gammaproteobacteria", "__Alteromonadales", "__Alteromonadaceae"]}},{"id": "denovo10978", "metadata": {"taxonomy": ["Bacteria", "__Candidate_division_WS3", "__c", "__o", "__f", "__g"]}},{"id": "denovo10979", "metadata": {"taxonomy": ["Bacteria", "__Proteobacteria", "__Gammaproteobacteria", "__Order_Incertae_Sedis", "__Family_Incertae_Sedis", "__Marinicella"]}},{"id": "denovo10981", "metadata": {"taxonomy": ["Bacteria", "__Proteobacteria", "__Deltaproteobacteria", "__Sh765B-TzT-29", "__f", "__g"]}},{"id": "denovo10982", "metadata": {"taxonomy": ["Bacteria", "__Proteobacteria", "__Deltaproteobacteria", "__Desulfovibrionales", "__Desulfovibrionaceae", "__Desulfovibrio"]}},{"id": "denovo10986", "metadata": {"taxonomy": ["Bacteria", "__Proteobacteria", "__Gammaproteobacteria", "__Alteromonadales", "__Alteromonadaceae", "__Alteromonas"]}},{"id": "denovo10991", "metadata": {"taxonomy": ["Bacteria", "__Proteobacteria", "__Alphaproteobacteria", "__Rhizobiales", "__Brucellaceae"]}},{"id": "denovo10993", "metadata": {"taxonomy": ["Bacteria", "__Gemmatimonadetes", "__Gemmatimonadetes", "__BD2-11_terrestrial_group", "__f", "__g"]}},{"id": "denovo10998",

"metadata": {"taxonomy": ["Bacteria", "__Cyanobacteria",
"__Cyanobacteria", "__SubsectionIII", "__FamilyI"]}},{"id":
"denovo11001", "metadata": {"taxonomy": ["Bacteria",
"__Candidate_division_TM7", "__c", "__o", "__f", "__g"]}},{"id":
"denovo11005", "metadata": {"taxonomy": ["Bacteria",
"__Proteobacteria", "__Deltaproteobacteria"]}},{"id": "denovo11007",
"metadata": {"taxonomy": ["Bacteria", "__Planctomycetes",
"__Planctomycetacia", "__Planctomycetales", "__Planctomycetaceae",
"__Pir4_lineage"]}},{"id": "denovo11011", "metadata": {"taxonomy":
["Bacteria", "__Bacteroidetes", "__Flavobacteria",
"__Flavobacteriales", "__Flavobacteriaceae"]}},{"id": "denovo11013",
"metadata": {"taxonomy": ["Bacteria", "__Proteobacteria",
"__Gammaproteobacteria", "__Alteromonadales", "__Alteromonadaceae",
"__OM60(NOR5)_clade"]}},{"id": "denovo11018", "metadata": {"taxonomy":
["Bacteria", "__Cyanobacteria", "__Cyanobacteria", "__SubsectionI",
"__FamilyI", "__g"]}},{"id": "denovo11020", "metadata": {"taxonomy":
["Bacteria", "__Proteobacteria", "__Alphaproteobacteria",
"__Rhodobacterales", "__Rhodobacteraceae"]}},{"id": "denovo11025",
"metadata": {"taxonomy": ["Bacteria", "__Proteobacteria",
"__Alphaproteobacteria", "__Rhodobacterales", "__Rhodobacteraceae",
"__Pseudoruegeria"]}},{"id": "denovo11026", "metadata": {"taxonomy":
["Bacteria", "__Proteobacteria", "__Alphaproteobacteria",
"__Kordiimonadales", "__Kordiimonadaceae", "__Kordiimonas"]}},{"id":
"denovo11031", "metadata": {"taxonomy": ["Bacteria",
"__Proteobacteria", "__Alphaproteobacteria", "__Rhodobacterales",
"__Rhodobacteraceae", "__Pseudovibrio"]}},{"id": "denovo11032",
"metadata": {"taxonomy": ["Bacteria", "__Proteobacteria",
"__Deltaproteobacteria", "__Bdellovibrionales",
"__Bdellovibrionaceae", "__Bdellovibrio"]}},{"id": "denovo11033",
"metadata": {"taxonomy": ["Bacteria", "__Cyanobacteria",
"__Cyanobacteria", "__SubsectionIV", "__FamilyII", "__Calothrix"]}},
{"id": "denovo11035", "metadata": {"taxonomy": ["Bacteria",
"__Proteobacteria", "__Alphaproteobacteria", "__Parvularculales",
"__Parvularculaceae", "__Parvularcula"]}},{"id": "denovo11036",
"metadata": {"taxonomy": ["Bacteria", "__Acidobacteria",
"__Acidobacteria", "__BPC015", "__f", "__g"]}},{"id": "denovo11038",
"metadata": {"taxonomy": ["Bacteria", "__Bacteroidetes",
"__Sphingobacteriia", "__Sphingobacteriales", "__Saprospiraceae",
"__g"]}},{"id": "denovo11039", "metadata": {"taxonomy": ["Bacteria",
"__Chloroflexi", "__Caldilineae", "__Caldilineales",
"__Caldilineaceae", "__Caldilinea"]}},{"id": "denovo11040",
"metadata": {"taxonomy": ["Bacteria", "__Proteobacteria",
"__Alphaproteobacteria", "__Sphingomonadales",
"__Erythrobacteraceae"]}},{"id": "denovo11041", "metadata":
{"taxonomy": ["Bacteria", "__Proteobacteria", "__Gammaproteobacteria",
"__Oceanospirillales", "__OM182_clade", "__g"]}},{"id": "denovo11042",
"metadata": {"taxonomy": ["Bacteria", "__Cyanobacteria",
"__Cyanobacteria", "__SubsectionI", "__FamilyI", "__g"]}},{"id":
"denovo11043", "metadata": {"taxonomy": ["Bacteria",
"__Bacteroidetes", "__Flavobacteria", "__Flavobacteriales",

"__Flavobacteriaceae", "__Galbibacter"]}},{"id": "denovo11044",
"metadata": {"taxonomy": ["Archaea", "__Thaumarchaeota",
"__Marine_Group_I", "__o", "__f", "__g"]}},{"id": "denovo11047",
"metadata": {"taxonomy": ["Bacteria", "__Lentisphaerae",
"__Lentisphaeria", "__Victivallales", "__Victivallaceae", "__g"]}},
{"id": "denovo11053", "metadata": {"taxonomy": ["Bacteria",
"__Proteobacteria", "__Alphaproteobacteria", "__Sphingomonadales",
"__Family_Incertae_Sedis", "__Kiloniella"]}},{"id": "denovo11055",
"metadata": {"taxonomy": ["Bacteria", "__Bacteroidetes",
"__Cytophagia", "__Cytophagales", "__Flammeovirgaceae",
"__Ekhidna"]}},{"id": "denovo11059", "metadata": {"taxonomy":
["Bacteria", "__Proteobacteria", "__Gammaproteobacteria",
"__Oceanospirillales", "__Oceanospirillaceae", "__Pseudospirillum"]}},
{"id": "denovo11066", "metadata": {"taxonomy": ["Bacteria",
"__Bacteroidetes", "__Flavobacteria", "__Flavobacteriales",
"__Flavobacteriaceae", "__NS4_marine_group"]}},{"id": "denovo11068",
"metadata": {"taxonomy": ["Bacteria"]}},{"id": "denovo11077",
"metadata": {"taxonomy": ["Bacteria", "__Firmicutes", "__Clostridia",
"__Clostridiales", "__Family_XI_Incertae_Sedis", "__Anaerococcus"]}},
{"id": "denovo11081", "metadata": {"taxonomy": ["Bacteria",
"__Proteobacteria", "__Alphaproteobacteria", "__Rhodobacterales",
"__Rhodobacteraceae", "__Labrenzia"]}},{"id": "denovo11089",
"metadata": {"taxonomy": ["Bacteria", "__Bacteroidetes",
"__Cytophagia", "__Cytophagales", "__Flammeovirgaceae"]}},{"id":
"denovo11090", "metadata": {"taxonomy": ["Bacteria",
"__Bacteroidetes", "__Flavobacteria", "__Flavobacteriales",
"__Flavobacteriaceae", "__NS5_marine_group"]}},{"id": "denovo11093",
"metadata": {"taxonomy": ["Bacteria", "__Proteobacteria",
"__Gammaproteobacteria", "__KI89A_clade", "__f", "__g"]}},{"id":
"denovo11094", "metadata": {"taxonomy": ["Bacteria",
"__Proteobacteria", "__Gammaproteobacteria", "__Oceanospirillales",
"__Oceanospirillaceae", "__Pseudospirillum"]}},{"id": "denovo11107",
"metadata": {"taxonomy": ["Bacteria", "__Proteobacteria",
"__Gammaproteobacteria", "__Order_Incertae_Sedis",
"__Family_Incertae_Sedis", "__Arenicella"]}},{"id": "denovo11108",
"metadata": {"taxonomy": ["Bacteria", "__Bacteroidetes",
"__Cytophagia", "__Cytophagales", "__Flammeovirgaceae", "__g"]}},
{"id": "denovo11110", "metadata": {"taxonomy": ["Bacteria",
"__Proteobacteria", "__Gammaproteobacteria", "__Alteromonadales",
"__Alteromonadaceae", "__Haliea"]}},{"id": "denovo11113", "metadata":
{"taxonomy": ["Bacteria", "__Proteobacteria", "__Gammaproteobacteria",
"__Pseudomonadales", "__Moraxellaceae", "__Psychrobacter"]}},{"id":
"denovo11114", "metadata": {"taxonomy": ["Bacteria",
"__Proteobacteria", "__Deltaproteobacteria", "__Desulfuromonadales",
"__GR-WP33-58", "__g"]}},{"id": "denovo11116", "metadata":
{"taxonomy": ["Bacteria", "__Proteobacteria", "__Gammaproteobacteria",
"__Xanthomonadales", "__Sinobacteraceae",
"__JTB255_marine_benthic_group"]}},{"id": "denovo11117", "metadata":
{"taxonomy": ["Bacteria", "__Proteobacteria", "__Deltaproteobacteria",
"__Sh765B-TzT-29", "__f", "__g"]}},{"id": "denovo11120", "metadata":

{"taxonomy": ["Bacteria", "__Firmicutes", "__Bacilli",
"__Lactobacillales", "__Carnobacteriaceae", "__Granulicatella"]}},
{"id": "denovo11121", "metadata": {"taxonomy": ["Bacteria",
"__Proteobacteria", "__Alphaproteobacteria", "__Rickettsiales"]}},
{"id": "denovo11124", "metadata": {"taxonomy": ["Bacteria",
"__Deferribacteres", "__Deferribacteres", "__Deferribacterales",
"__Family_Incertae_Sedis", "__Caldithrix"]}},{"id": "denovo11126",
"metadata": {"taxonomy": ["Bacteria", "__Proteobacteria",
"__Alphaproteobacteria", "__Rickettsiales", "__SM2D12", "__g"]}},
{"id": "denovo11127", "metadata": {"taxonomy": ["Bacteria",
"__Bacteroidetes"]}},{"id": "denovo11129", "metadata": {"taxonomy":
["Bacteria", "__Proteobacteria", "__Gammaproteobacteria",
"__OXIC-003", "__f", "__g"]}},{"id": "denovo11134", "metadata":
{"taxonomy": ["Bacteria"]}},{"id": "denovo11137", "metadata":
{"taxonomy": ["Bacteria", "__Proteobacteria", "__Gammaproteobacteria",
"__Xanthomonadales", "__Sinobacteraceae",
"__JTB255_marine_benthic_group"]}},{"id": "denovo11139", "metadata":
{"taxonomy": ["Bacteria", "__Proteobacteria", "__Alphaproteobacteria",
"__Rhodobacterales", "__Rhodobacteraceae"]}},{"id": "denovo11148",
"metadata": {"taxonomy": ["Bacteria", "__Proteobacteria"]}},{"id":
"denovo11152", "metadata": {"taxonomy": ["Bacteria",
"__Actinobacteria", "__Acidimicrobiia", "__Acidimicrobiales",
"__OCS155_marine_group", "__g"]}},{"id": "denovo11154", "metadata":
{"taxonomy": ["Bacteria", "__Acidobacteria", "__Holophagae", "__TK85",
"__f", "__g"]}},{"id": "denovo11157", "metadata": {"taxonomy":
["Bacteria", "__Proteobacteria", "__Gammaproteobacteria"]}},{"id":
"denovo11158", "metadata": {"taxonomy": ["Bacteria",
"__Gemmatimonadetes", "__Gemmatimonadetes", "__Kazan-1B-37", "__f",
"__g"]}},{"id": "denovo11161", "metadata": {"taxonomy": ["Bacteria",
"__Proteobacteria", "__Deltaproteobacteria", "__Desulfobacterales",
"__Desulfobulbaceae"]}},{"id": "denovo11162", "metadata": {"taxonomy":
["Bacteria", "__Proteobacteria", "__Gammaproteobacteria",
"__BD7-8_marine_group", "__f", "__g"]}},{"id": "denovo11165",
"metadata": {"taxonomy": ["Bacteria", "__Proteobacteria",
"__Gammaproteobacteria", "__Alteromonadales", "__Alteromonadaceae"]}},
{"id": "denovo11166", "metadata": {"taxonomy": ["Bacteria",
"__Proteobacteria", "__Alphaproteobacteria", "__DB1-14", "__f",
"__g"]}},{"id": "denovo11168", "metadata": {"taxonomy": ["Bacteria",
"__Proteobacteria", "__Alphaproteobacteria", "__Rhodobacterales",
"__Rhodobacteraceae", "__Roseobacter_clade_CHAB-I-5_lineage"]}},{"id":
"denovo11177", "metadata": {"taxonomy": ["Bacteria",
"__Proteobacteria", "__Gammaproteobacteria"]}},{"id": "denovo11187",
"metadata": {"taxonomy": ["Bacteria", "__Proteobacteria",
"__Alphaproteobacteria", "__Rhodospirillales", "__Rhodospirillaceae",
"__Defluviicoccus"]}},{"id": "denovo11192", "metadata": {"taxonomy":
["Bacteria", "__Proteobacteria", "__Deltaproteobacteria",
"__Bdellovibrionales", "__Bdellovibrionaceae", "__Bdellovibrio"]}},
{"id": "denovo11197", "metadata": {"taxonomy": ["Bacteria",
"__Chlorobi", "__Chlorobia", "__Chlorobiales", "__Chlorobiaceae",
"__Prosthecochloris"]}},{"id": "denovo11199", "metadata": {"taxonomy":

["Bacteria", "__Bacteroidetes", "__Sphingobacteriia", "__Sphingobacteriales", "__Saprospiraceae", "__g"]}},{"id": "denovo11202", "metadata": {"taxonomy": ["Bacteria", "__Bacteroidetes", "__Flavobacteria", "__Flavobacteriales", "__Flavobacteriaceae", "__Polaribacter"]}},{"id": "denovo11203", "metadata": {"taxonomy": ["Bacteria", "__Firmicutes", "__Clostridia", "__Clostridiales", "__Lachnospiraceae"]}},{"id": "denovo11205", "metadata": {"taxonomy": ["Bacteria", "__Proteobacteria", "__Alphaproteobacteria", "__Rhodobacterales", "__Rhodobacteraceae", "__Pseudoruegeria"]}},{"id": "denovo11208", "metadata": {"taxonomy": ["Bacteria", "__Proteobacteria", "__Alphaproteobacteria", "__Rhodobacterales", "__Rhodobacteraceae", "__Tropicimonas"]}},{"id": "denovo11209", "metadata": {"taxonomy": ["Bacteria", "__Proteobacteria", "__Alphaproteobacteria", "__Rhizobiales", "__Hyphomicrobiaceae", "__Devosia"]}},{"id": "denovo11211", "metadata": {"taxonomy": ["Bacteria", "__Proteobacteria", "__Gammaproteobacteria", "__Alteromonadales", "__Alteromonadaceae", "__C1-B045"]}},{"id": "denovo11212", "metadata": {"taxonomy": ["Bacteria", "__Proteobacteria", "__Deltaproteobacteria", "__Myxococcales", "__Sorangiineae", "__g"]}},{"id": "denovo11218", "metadata": {"taxonomy": ["Bacteria", "__Proteobacteria", "__Gammaproteobacteria", "__Chromatiales", "__Granulosicoccaceae", "__Granulosicoccus"]}},{"id": "denovo11219", "metadata": {"taxonomy": ["Bacteria", "__Proteobacteria", "__Alphaproteobacteria", "__SAR11_clade", "__Deep_1", "__g"]}},{"id": "denovo11220", "metadata": {"taxonomy": ["Bacteria", "__Proteobacteria", "__Gammaproteobacteria"]}},{"id": "denovo11223", "metadata": {"taxonomy": ["Bacteria", "__Proteobacteria", "__Gammaproteobacteria", "__Chromatiales", "__Chromatiaceae"]}},{"id": "denovo11227", "metadata": {"taxonomy": ["Bacteria", "__Proteobacteria", "__SC3-20", "__o", "__f", "__g"]}},{"id": "denovo11236", "metadata": {"taxonomy": ["Bacteria", "__Cyanobacteria", "__Cyanobacteria", "__SubsectionI", "__FamilyI"]}},{"id": "denovo11237", "metadata": {"taxonomy": ["Bacteria", "__Proteobacteria", "__Alphaproteobacteria", "__Rhodobacterales", "__Rhodobacteraceae"]}},{"id": "denovo11238", "metadata": {"taxonomy": ["Bacteria", "__Proteobacteria", "__Alphaproteobacteria", "__Sphingomonadales", "__Erythrobacteraceae", "__Erythrobacter"]}},{"id": "denovo11240", "metadata": {"taxonomy": ["Bacteria", "__Proteobacteria", "__Gammaproteobacteria", "__Enterobacteriales", "__Enterobacteriaceae"]}},{"id": "denovo11242", "metadata": {"taxonomy": ["Bacteria", "__Planctomycetes", "__Phycisphaerae", "__MSBL9", "__f", "__g"]}},{"id": "denovo11245", "metadata": {"taxonomy": ["Bacteria", "__Acidobacteria", "__Holophagae", "__CA002", "__f", "__g"]}},{"id": "denovo11249", "metadata": {"taxonomy": ["Bacteria", "__Acidobacteria", "__Acidobacteria", "__AT-s3-28", "__f", "__g"]}},{"id": "denovo11250", "metadata": {"taxonomy": ["Bacteria", "__Proteobacteria", "__Gammaproteobacteria", "__Alteromonadales", "__Alteromonadaceae", "__g"]}},{"id": "denovo11259", "metadata": {"taxonomy": ["Bacteria", "__Proteobacteria", "__Gammaproteobacteria"]}},{"id": "denovo11262",

"metadata": {"taxonomy": ["Bacteria", "__Cyanobacteria",
"__Cyanobacteria", "__SubsectionIII", "__FamilyI",
"__Halomicronema"]}},{"id": "denovo11263", "metadata": {"taxonomy":
["Bacteria", "__Proteobacteria", "__Gammaproteobacteria",
"__Enterobacteriales", "__Enterobacteriaceae"]}},{"id": "denovo11264",
"metadata": {"taxonomy": ["Bacteria", "__Verrucomicrobia",
"__Verrucomicrobiae", "__Verrucomicrobiales", "__Verrucomicrobiaceae",
"__Persicirhabdus"]}},{"id": "denovo11265", "metadata": {"taxonomy":
["Bacteria", "__Cyanobacteria", "__Cyanobacteria", "__SubsectionIII",
"__FamilyI", "__Halomicronema"]}},{"id": "denovo11266", "metadata":
{"taxonomy": ["Bacteria", "__Proteobacteria", "__Gammaproteobacteria",
"__Alteromonadales", "__Alteromonadaceae", "__BD2-7"]}},{"id":
"denovo11267", "metadata": {"taxonomy": ["Bacteria",
"__Proteobacteria", "__Gammaproteobacteria", "__BD7-8_marine_group",
"__f", "__g"]}},{"id": "denovo11268", "metadata": {"taxonomy":
["Bacteria", "__Bacteroidetes", "__Flavobacteria",
"__Flavobacteriales", "__Flavobacteriaceae", "__NS5_marine_group"]}},
{"id": "denovo11271", "metadata": {"taxonomy": ["Bacteria",
"__Proteobacteria", "__Alphaproteobacteria", "__Rhodobacterales",
"__Rhodobacteraceae"]}},{"id": "denovo11272", "metadata": {"taxonomy":
["Bacteria", "__Bacteroidetes", "__Sphingobacteriia",
"__Sphingobacteriales", "__Saprospiraceae", "__g"]}},{"id":
"denovo11273", "metadata": {"taxonomy": ["Bacteria",
"__Proteobacteria", "__Deltaproteobacteria", "__Myxococcales",
"__Nannocystaceae", "__g"]}},{"id": "denovo11274", "metadata":
{"taxonomy": ["Bacteria", "__Proteobacteria", "__Alphaproteobacteria",
"__Rhizobiales", "__Phyllobacteriaceae", "__g"]}},{"id":
"denovo11276", "metadata": {"taxonomy": ["Bacteria",
"__Proteobacteria", "__Gammaproteobacteria", "__Chromatiales"]}},
{"id": "denovo11283", "metadata": {"taxonomy": ["Bacteria",
"__Proteobacteria", "__Alphaproteobacteria", "__Sphingomonadales",
"__Erythrobacteraceae", "__Erythrobacter"]}},{"id": "denovo11287",
"metadata": {"taxonomy": ["Bacteria", "__Proteobacteria",
"__Alphaproteobacteria", "__Rhodobacterales", "__Rhodobacteraceae"]}},
{"id": "denovo11290", "metadata": {"taxonomy": ["Bacteria",
"__Proteobacteria", "__Alphaproteobacteria", "__Rhodobacterales",
"__Rhodobacteraceae", "__Roseobacter_clade_OCT_lineage"]}},{"id":
"denovo11293", "metadata": {"taxonomy": ["Bacteria",
"__Proteobacteria", "__Gammaproteobacteria", "__Alteromonadales",
"__Alteromonadaceae", "__Alteromonas"]}},{"id": "denovo11300",
"metadata": {"taxonomy": ["Bacteria", "__Proteobacteria",
"__Gammaproteobacteria", "__Xanthomonadales", "__Sinobacteraceae",
"__JTB255_marine_benthic_group"]}},{"id": "denovo11301", "metadata":
{"taxonomy": ["Bacteria", "__Lentisphaerae", "__Lentisphaeria",
"__Victivallales", "__Victivallaceae", "__g"]}},{"id": "denovo11302",
"metadata": {"taxonomy": ["Bacteria", "__Proteobacteria",
"__Alphaproteobacteria", "__Rhodobacterales", "__Rhodobacteraceae",
"__g"]}},{"id": "denovo11307", "metadata": {"taxonomy": ["Bacteria",
"__Proteobacteria", "__Deltaproteobacteria", "__Sh765B-TzT-29", "__f",
"__g"]}},{"id": "denovo11308", "metadata": {"taxonomy": ["Bacteria",

"__Firmicutes", "__Clostridia", "__Clostridiales",
"__Ruminococcaceae", "__Ruminococcus"]}},{"id": "denovo11309",
"metadata": {"taxonomy": ["Bacteria", "__Proteobacteria",
"__Deltaproteobacteria", "__Desulfobacterales", "__Nitrospinaceae",
"__Nitrospina"]}},{"id": "denovo11312", "metadata": {"taxonomy":
["Bacteria", "__Bacteroidetes", "__Flavobacteria",
"__Flavobacteriales", "__Flavobacteriaceae", "__Algibacter"]}},{"id":
"denovo11316", "metadata": {"taxonomy": ["Bacteria",
"__Proteobacteria", "__Gammaproteobacteria", "__Xanthomonadales",
"__Sinobacteraceae", "__JTB255_marine_benthic_group"]}},{"id":
"denovo11325", "metadata": {"taxonomy": ["Bacteria",
"__Proteobacteria", "__Alphaproteobacteria"]}},{"id": "denovo11327",
"metadata": {"taxonomy": ["Bacteria", "__Planctomycetes",
"__Phycisphaerae", "__Phycisphaerales", "__Phycisphaeraceae",
"__Phycisphaera"]}},{"id": "denovo11329", "metadata": {"taxonomy":
["Bacteria", "__Proteobacteria", "__Deltaproteobacteria",
"__SAR324_clade(Marine_group_B)", "__f", "__g"]}},{"id":
"denovo11330", "metadata": {"taxonomy": ["Bacteria",
"__Proteobacteria", "__Gammaproteobacteria"]}},{"id": "denovo11332",
"metadata": {"taxonomy": ["Bacteria", "__Proteobacteria", "__TA18",
"__o", "__f", "__g"]}},{"id": "denovo11336", "metadata": {"taxonomy":
["Bacteria", "__Bacteroidetes", "__Sphingobacteriia",
"__Sphingobacteriales", "__Saprospiraceae", "__g"]}},{"id":
"denovo11337", "metadata": {"taxonomy": ["Bacteria",
"__Proteobacteria", "__Alphaproteobacteria", "__Rhodobacterales",
"__Rhodobacteraceae", "__Ruegeria"]}},{"id": "denovo11338",
"metadata": {"taxonomy": ["Bacteria", "__Bacteroidetes",
"__Flavobacteria", "__Flavobacteriales", "__Flavobacteriaceae"]}},
{"id": "denovo11339", "metadata": {"taxonomy": ["Bacteria",
"__Chloroflexi", "__SAR202_clade", "__o", "__f", "__g"]}},{"id":
"denovo11348", "metadata": {"taxonomy": ["Bacteria", "__Firmicutes",
"__Bacilli", "__Bacillales", "__Pasteuriaceae", "__Pasteuria"]}},
{"id": "denovo11354", "metadata": {"taxonomy": ["Bacteria",
"__Proteobacteria", "__Alphaproteobacteria", "__Rhodospirillales",
"__Rhodospirillaceae", "__g"]}},{"id": "denovo11358", "metadata":
{"taxonomy": ["Bacteria", "__Proteobacteria", "__Deltaproteobacteria",
"__Desulfobacterales", "__Nitrospinaceae", "__Nitrospina"]}},{"id":
"denovo11362", "metadata": {"taxonomy": ["Bacteria",
"__Acidobacteria", "__Holophagae", "__CA002", "__f", "__g"]}},{"id":
"denovo11363", "metadata": {"taxonomy": ["Bacteria",
"__Proteobacteria", "__Alphaproteobacteria"]}},{"id": "denovo11367",
"metadata": {"taxonomy": ["Bacteria", "__Proteobacteria", "__TA18",
"__o", "__f", "__g"]}},{"id": "denovo11368", "metadata": {"taxonomy":
["Bacteria", "__Proteobacteria", "__Alphaproteobacteria",
"__Rhodospirillales", "__Rhodospirillaceae", "__Nisaea"]}},{"id":
"denovo11379", "metadata": {"taxonomy": ["Bacteria",
"__Proteobacteria", "__Alphaproteobacteria"]}},{"id": "denovo11382",
"metadata": {"taxonomy": ["Bacteria", "__Acidobacteria",
"__Acidobacteria", "__PAUC26f", "__f", "__g"]}},{"id": "denovo11383",
"metadata": {"taxonomy": ["Bacteria", "__Proteobacteria",

"__Deltaproteobacteria", "__Desulfuromonadales"]}},{"id": "denovo11388", "metadata": {"taxonomy": ["Bacteria", "__Proteobacteria", "__Gammaproteobacteria", "__Pseudomonadales", "__Moraxellaceae", "__Acinetobacter"]}},{"id": "denovo11390", "metadata": {"taxonomy": ["Bacteria", "__Proteobacteria", "__Deltaproteobacteria", "__Bdellovibrionales", "__Bdellovibrionaceae", "__Bdellovibrio"]}},{"id": "denovo11400", "metadata": {"taxonomy": ["Bacteria", "__Proteobacteria", "__Gammaproteobacteria", "__Order_Incertae_Sedis", "__Family_Incertae_Sedis", "__Marinicella"]}},{"id": "denovo11404", "metadata": {"taxonomy": ["Bacteria", "__Candidate_division_OD1", "__c", "__o", "__f", "__g"]}},{"id": "denovo11405", "metadata": {"taxonomy": ["Bacteria", "__Proteobacteria", "__Gammaproteobacteria", "__KI89A_clade", "__f", "__g"]}},{"id": "denovo11407", "metadata": {"taxonomy": ["Bacteria", "__Proteobacteria", "__Alphaproteobacteria", "__Rhodobacterales", "__Rhodobacteraceae"]}},{"id": "denovo11409", "metadata": {"taxonomy": ["Bacteria", "__Bacteroidetes", "__Flavobacteria", "__Flavobacteriales", "__Flavobacteriaceae", "__NS4_marine_group"]}},{"id": "denovo11412", "metadata": {"taxonomy": ["Bacteria", "__Proteobacteria", "__Gammaproteobacteria", "__Alteromonadales", "__Alteromonadaceae", "__Haliea"]}},{"id": "denovo11416", "metadata": {"taxonomy": ["Bacteria", "__Proteobacteria", "__Gammaproteobacteria", "__Vibrionales", "__Vibrionaceae", "__Grimontia"]}},{"id": "denovo11417", "metadata": {"taxonomy": ["Bacteria", "__TM6", "__c", "__o", "__f", "__g"]}},{"id": "denovo11423", "metadata": {"taxonomy": ["Bacteria", "__Proteobacteria", "__Alphaproteobacteria", "__Rhizobiales", "__Phyllobacteriaceae"]}},{"id": "denovo11429", "metadata": {"taxonomy": ["Bacteria", "__Proteobacteria", "__Alphaproteobacteria", "__Rhodobacterales", "__Rhodobacteraceae", "__Pseudovibrio"]}},{"id": "denovo11433", "metadata": {"taxonomy": ["Bacteria", "__Proteobacteria", "__Alphaproteobacteria", "__Rhodospirillales", "__Rhodospirillaceae", "__Thalassospira"]}},{"id": "denovo11435", "metadata": {"taxonomy": ["Bacteria", "__Proteobacteria", "__Alphaproteobacteria", "__Rickettsiales", "__Holosporaceae", "__Holospora"]}},{"id": "denovo11436", "metadata": {"taxonomy": ["Bacteria", "__Proteobacteria", "__Deltaproteobacteria", "__Sh765B-TzT-29", "__f", "__g"]}},{"id": "denovo11437", "metadata": {"taxonomy": ["Bacteria", "__Acidobacteria", "__Holophagae", "__Acanthopleuribacterales", "__Acanthopleuribacteraceae", "__Acanthopleuribacter"]}},{"id": "denovo11438", "metadata": {"taxonomy": ["Bacteria", "__Proteobacteria", "__Alphaproteobacteria", "__Rhodobacterales", "__Rhodobacteraceae", "__Wenxinia"]}},{"id": "denovo11439", "metadata": {"taxonomy": ["Bacteria", "__Proteobacteria", "__Deltaproteobacteria", "__Myxococcales", "__Haliangiaceae", "__Haliangium"]}},{"id": "denovo11443", "metadata": {"taxonomy": ["Bacteria", "__Proteobacteria", "__Gammaproteobacteria", "__Pseudomonadales", "__Moraxellaceae", "__Acinetobacter"]}},{"id": "denovo11445", "metadata": {"taxonomy": ["Bacteria", "__Acidobacteria", "__Holophagae", "__Acanthopleuribacterales",

"__Acanthopleuribacteraceae", "__Acanthopleuribacter"]}},{"id": "denovo11447", "metadata": {"taxonomy": ["Bacteria", "__Proteobacteria", "__Alphaproteobacteria", "__SB1-18", "__f", "__g"]}},{"id": "denovo11454", "metadata": {"taxonomy": ["Bacteria", "__TM6", "__c", "__o", "__f", "__g"]}},{"id": "denovo11457", "metadata": {"taxonomy": ["Bacteria", "__Bacteroidetes", "__Flavobacteria", "__Flavobacteriales", "__Flavobacteriaceae", "__Formosa"]}},{"id": "denovo11462", "metadata": {"taxonomy": ["Bacteria", "__Proteobacteria", "__Gammaproteobacteria", "__Order_Incertae_Sedis", "__Family_Incertae_Sedis", "__Marinicella"]}},{"id": "denovo11463", "metadata": {"taxonomy": ["Bacteria", "__Bacteroidetes", "__Sphingobacteriia", "__Sphingobacteriales", "__Saprospiraceae", "__g"]}},{"id": "denovo11467", "metadata": {"taxonomy": ["Bacteria", "__Proteobacteria", "__Deltaproteobacteria", "__Bdellovibrionales", "__Bacteriovoracaceae", "__Peredibacter"]}},{"id": "denovo11472", "metadata": {"taxonomy": ["Bacteria", "__Proteobacteria", "__Alphaproteobacteria", "__Rhodobacterales", "__Rhodobacteraceae", "__Pseudovibrio"]}},{"id": "denovo11475", "metadata": {"taxonomy": ["Bacteria", "__Proteobacteria", "__Alphaproteobacteria", "__Parvularculales", "__Parvularculaceae", "__Parvularcula"]}},{"id": "denovo11476", "metadata": {"taxonomy": ["Bacteria", "__Proteobacteria", "__Gammaproteobacteria", "__Alteromonadales", "__Alteromonadaceae", "__Haliea"]}},{"id": "denovo11477", "metadata": {"taxonomy": ["Bacteria", "__Proteobacteria", "__Alphaproteobacteria", "__Parvularculales", "__Parvularculaceae", "__Parvularcula"]}},{"id": "denovo11478", "metadata": {"taxonomy": ["Bacteria", "__Cyanobacteria", "__Cyanobacteria", "__SubsectionIII", "__FamilyI", "__g"]}},{"id": "denovo11481", "metadata": {"taxonomy": ["Bacteria", "__Proteobacteria", "__Gammaproteobacteria", "__Alteromonadales", "__Alteromonadaceae", "__g"]}},{"id": "denovo11489", "metadata": {"taxonomy": ["Bacteria", "__Proteobacteria", "__Deltaproteobacteria", "__Sva0485", "__f", "__g"]}},{"id": "denovo11492", "metadata": {"taxonomy": ["Bacteria", "__Proteobacteria", "__Deltaproteobacteria", "__Myxococcales", "__Nannocystineae", "__g"]}},{"id": "denovo11493", "metadata": {"taxonomy": ["Bacteria", "__Planctomycetes", "__Phycisphaerae", "__Phycisphaerales", "__Phycisphaeraceae", "__Phycisphaera"]}},{"id": "denovo11494", "metadata": {"taxonomy": ["Bacteria", "__Proteobacteria", "__Alphaproteobacteria", "__Rhodobacterales", "__Rhodobacteraceae"]}},{"id": "denovo11496", "metadata": {"taxonomy": ["Bacteria", "__Proteobacteria", "__Deltaproteobacteria", "__Desulfobacterales", "__Desulfobulbaceae", "__Desulfobulbus"]}},{"id": "denovo11497", "metadata": {"taxonomy": ["Bacteria", "__Chloroflexi", "__Caldilineae", "__Caldilineales", "__Caldilineaceae", "__Caldilinea"]}},{"id": "denovo11498", "metadata": {"taxonomy": ["Bacteria", "__Proteobacteria", "__Deltaproteobacteria", "__GR-WP33-30", "__f", "__g"]}},{"id": "denovo11500", "metadata": {"taxonomy": ["Bacteria", "__Proteobacteria", "__Alphaproteobacteria", "__SAR11_clade", "__Surface_1", "__Candidatus_Pelagibacter"]}},{"id": "denovo11502",

"metadata": {"taxonomy": ["Bacteria", "__Proteobacteria", "__Gammaproteobacteria", "__Order_Incertae_Sedis", "__Family_Incertae_Sedis", "__Marinicella"]}},{"id": "denovo11504", "metadata": {"taxonomy": ["Bacteria", "__Proteobacteria", "__Alphaproteobacteria", "__Rhodobacterales", "__Rhodobacteraceae", "__Ruegeria"]}},{"id": "denovo11506", "metadata": {"taxonomy": ["Bacteria", "__Proteobacteria", "__Gammaproteobacteria", "__KI89A_clade", "__f", "__g"]}},{"id": "denovo11507", "metadata": {"taxonomy": ["Archaea", "__Thaumarchaeota", "__Marine_Group_I", "__o", "__f", "__g"]}},{"id": "denovo11512", "metadata": {"taxonomy": ["Bacteria", "__Proteobacteria", "__TA18", "__o", "__f", "__g"]}},{"id": "denovo11516", "metadata": {"taxonomy": ["Bacteria", "__Proteobacteria", "__Alphaproteobacteria", "__Rhodospirillales", "__Rhodospirillaceae", "__g"]}},{"id": "denovo11518", "metadata": {"taxonomy": ["Bacteria", "__Proteobacteria", "__Gammaproteobacteria", "__KI89A_clade", "__f", "__g"]}},{"id": "denovo11519", "metadata": {"taxonomy": ["Bacteria", "__Bacteroidetes", "__Flavobacteria", "__Flavobacteriales", "__Cryomorphaceae", "__Crocinitomix"]}},{"id": "denovo11520", "metadata": {"taxonomy": ["Bacteria", "__Proteobacteria", "__Alphaproteobacteria", "__Rhodospirillales", "__Rhodospirillaceae", "__Pelagibius"]}},{"id": "denovo11521", "metadata": {"taxonomy": ["Bacteria", "__Proteobacteria", "__Gammaproteobacteria", "__Order_Incertae_Sedis", "__Family_Incertae_Sedis", "__Sedimenticola"]}},{"id": "denovo11527", "metadata": {"taxonomy": ["Bacteria", "__Firmicutes", "__Clostridia", "__Clostridiales", "__Family_XI_Incertae_Sedis", "__Finegoldia"]}},{"id": "denovo11532", "metadata": {"taxonomy": ["Bacteria", "__Proteobacteria", "__Deltaproteobacteria", "__Desulfobacterales", "__Desulfobulbaceae", "__Desulfobulbus"]}},{"id": "denovo11534", "metadata": {"taxonomy": ["Bacteria", "__Proteobacteria", "__Alphaproteobacteria", "__Rhodobacterales", "__Rhodobacteraceae", "__Tropicimonas"]}},{"id": "denovo11537", "metadata": {"taxonomy": ["Bacteria", "__Proteobacteria", "__Deltaproteobacteria", "__Bdellovibrionales", "__Bacteriovoracaceae", "__Peredibacter"]}},{"id": "denovo11538", "metadata": {"taxonomy": ["Bacteria", "__Proteobacteria", "__Alphaproteobacteria", "__Caulobacterales", "__Hyphomonadaceae", "__Hellea"]}},{"id": "denovo11539", "metadata": {"taxonomy": ["Bacteria", "__Proteobacteria", "__Gammaproteobacteria", "__Oceanospirillales"]}},{"id": "denovo11540", "metadata": {"taxonomy": ["Bacteria", "__Proteobacteria", "__Alphaproteobacteria", "__Rhodospirillales", "__Rhodospirillaceae", "__Pelagibius"]}},{"id": "denovo11542", "metadata": {"taxonomy": ["Bacteria", "__Proteobacteria", "__Deltaproteobacteria", "__Myxococcales", "__Sorangiineae", "__Sandaracinaceae"]}},{"id": "denovo11545", "metadata": {"taxonomy": ["Bacteria", "__Acidobacteria", "__RB25", "__o", "__f", "__g"]}},{"id": "denovo11546", "metadata": {"taxonomy": ["Bacteria", "__Proteobacteria", "__Gammaproteobacteria", "__Alteromonadales", "__Alteromonadaceae", "__Haliea"]}},{"id": "denovo11548", "metadata": {"taxonomy": ["Bacteria", "__Proteobacteria", "__Gammaproteobacteria", "__Alteromonadales",

"__Alteromonadaceae", "__Teredinibacter"]}},{"id": "denovo11550",
"metadata": {"taxonomy": ["Bacteria", "__Cyanobacteria",
"__Cyanobacteria", "__SubsectionIII", "__FamilyI", "__Phormidium"]}},
{"id": "denovo11552", "metadata": {"taxonomy": ["Bacteria",
"__Proteobacteria", "__Gammaproteobacteria", "__Oceanospirillales",
"__MBAE14", "__g"]}},{"id": "denovo11559", "metadata": {"taxonomy":
["Bacteria", "__Proteobacteria", "__Alphaproteobacteria",
"__Rhodospirillales", "__Rhodospirillaceae", "__g"]}},{"id":
"denovo11560", "metadata": {"taxonomy": ["Bacteria",
"__Proteobacteria", "__JTB23", "__o", "__f", "__g"]}},{"id":
"denovo11565", "metadata": {"taxonomy": ["Bacteria",
"__Proteobacteria", "__Gammaproteobacteria", "__Alteromonadales",
"__Alteromonadaceae", "__BD1-7_clade"]}},{"id": "denovo11566",
"metadata": {"taxonomy": ["Bacteria", "__Proteobacteria",
"__Alphaproteobacteria", "__Rhodobacterales", "__Rhodobacteraceae"]}},
{"id": "denovo11571", "metadata": {"taxonomy": ["Bacteria",
"__Proteobacteria", "__Gammaproteobacteria", "__Pseudomonadales",
"__Moraxellaceae", "__Acinetobacter"]}},{"id": "denovo11573",
"metadata": {"taxonomy": ["Bacteria", "__Cyanobacteria",
"__Cyanobacteria", "__SubsectionI", "__FamilyI", "__Acaryochloris"]}},
{"id": "denovo11575", "metadata": {"taxonomy": ["Bacteria",
"__Proteobacteria", "__Gammaproteobacteria", "__Pseudomonadales",
"__Moraxellaceae"]}},{"id": "denovo11576", "metadata": {"taxonomy":
["Bacteria", "__Proteobacteria", "__Alphaproteobacteria",
"__Rhodobacterales", "__Rhodobacteraceae"]}},{"id": "denovo11579",
"metadata": {"taxonomy": ["Bacteria", "__Proteobacteria",
"__Alphaproteobacteria", "__Rhodospirillales", "__Rhodospirillaceae",
"__Thalassospira"]}},{"id": "denovo11584", "metadata": {"taxonomy":
["Bacteria", "__Proteobacteria", "__Gammaproteobacteria",
"__Alteromonadales", "__Alteromonadaceae", "__g"]}},{"id":
"denovo11587", "metadata": {"taxonomy": ["Bacteria",
"__Proteobacteria", "__Gammaproteobacteria", "__Alteromonadales",
"__Alteromonadaceae", "__OM60(NOR5)_clade"]}},{"id": "denovo11588",
"metadata": {"taxonomy": ["Bacteria", "__Bacteroidetes",
"__Flavobacteria", "__Flavobacteriales", "__Flavobacteriaceae"]}},
{"id": "denovo11594", "metadata": {"taxonomy": ["Bacteria",
"__Bacteroidetes", "__Sphingobacteriia", "__Sphingobacteriales",
"__Saprospiraceae", "__g"]}},{"id": "denovo11597", "metadata":
{"taxonomy": ["Bacteria", "__Bacteroidetes", "__Flavobacteria",
"__Flavobacteriales", "__Cryomorphaceae", "__Owenweeksia"]}},{"id":
"denovo11603", "metadata": {"taxonomy": ["Bacteria",
"__Bacteroidetes", "__Flavobacteria", "__Flavobacteriales",
"__Cryomorphaceae", "__Fluviicola"]}},{"id": "denovo11606",
"metadata": {"taxonomy": ["Bacteria", "__Proteobacteria",
"__Alphaproteobacteria", "__Parvularculales", "__Parvularculaceae",
"__Parvularcula"]}},{"id": "denovo11610", "metadata": {"taxonomy":
["Bacteria", "__Proteobacteria", "__Alphaproteobacteria",
"__Rhodospirillales", "__Rhodospirillaceae", "__Pelagibius"]}},{"id":
"denovo11611", "metadata": {"taxonomy": ["Bacteria",
"__Proteobacteria", "__Deltaproteobacteria", "__Myxococcales",

"__Cystobacterineae", "__g"]}},{"id": "denovo11613", "metadata":
{"taxonomy": ["Bacteria", "__Cyanobacteria", "__Cyanobacteria",
"__SubsectionIII", "__FamilyI"]}},{"id": "denovo11617", "metadata":
{"taxonomy": ["Bacteria", "__TM6", "__c", "__o", "__f", "__g"]}},
{"id": "denovo11618", "metadata": {"taxonomy": ["Bacteria",
"__Acidobacteria", "__Acidobacteria", "__DA023", "__f", "__g"]}},
{"id": "denovo11621", "metadata": {"taxonomy": ["Bacteria",
"__Proteobacteria", "__Alphaproteobacteria", "__Rhodobacterales",
"__Rhodobacteraceae", "__Pseudovibrio"]}},{"id": "denovo11624",
"metadata": {"taxonomy": ["Bacteria", "__Acidobacteria",
"__Holophagae", "__Acanthopleuribacterales",
"__Acanthopleuribacteraceae", "__Acanthopleuribacter"]}},{"id":
"denovo11627", "metadata": {"taxonomy": ["Bacteria",
"__Proteobacteria", "__Deltaproteobacteria", "__Desulfovibrionales",
"__Desulfovibrionaceae", "__Desulfovibrio"]}},{"id": "denovo11628",
"metadata": {"taxonomy": ["Bacteria", "__Proteobacteria",
"__Deltaproteobacteria", "__Myxococcales", "__Sorangiineae", "__g"]}},
{"id": "denovo11638", "metadata": {"taxonomy": ["Archaea",
"__Thaumarchaeota", "__Marine_Group_I", "__Candidatus_Nitrosopumilus",
"__f", "__g"]}},{"id": "denovo11645", "metadata": {"taxonomy":
["Bacteria", "__Planctomycetes", "__Phycisphaerae",
"__Phycisphaerales", "__Phycisphaeraceae",
"__Urania-1B-19_marine_sediment_group"]}},{"id": "denovo11652",
"metadata": {"taxonomy": ["Bacteria", "__Acidobacteria",
"__Holophagae", "__CA002", "__f", "__g"]}},{"id": "denovo11656",
"metadata": {"taxonomy": ["Bacteria", "__Bacteroidetes",
"__Cytophagia", "__Cytophagales", "__Flammeovirgaceae"]}},{"id":
"denovo11658", "metadata": {"taxonomy": ["Bacteria",
"__Proteobacteria", "__Deltaproteobacteria", "__Desulfobacterales",
"__Desulfobacteraceae"]}},{"id": "denovo11659", "metadata":
{"taxonomy": ["Bacteria", "__Proteobacteria", "__Gammaproteobacteria",
"__Enterobacteriales", "__Enterobacteriaceae", "__Escherichia-
Shigella"]}},{"id": "denovo11660", "metadata": {"taxonomy":
["Bacteria", "__Proteobacteria", "__Alphaproteobacteria",
"__Parvularculales", "__Parvularculaceae", "__Parvularcula"]}},{"id":
"denovo11663", "metadata": {"taxonomy": ["Bacteria",
"__Proteobacteria", "__Alphaproteobacteria", "__Rhizobiales",
"__Phyllobacteriaceae", "__Ahrensia"]}},{"id": "denovo11664",
"metadata": {"taxonomy": ["Bacteria", "__Proteobacteria",
"__Deltaproteobacteria", "__Desulfobacterales", "__Nitrospinaceae",
"__Candidatus_Entotheonella"]}},{"id": "denovo11670", "metadata":
{"taxonomy": ["Bacteria", "__Bacteroidetes", "__Sphingobacteriia",
"__Sphingobacteriales", "__Saprospiraceae", "__g"]}},{"id":
"denovo11672", "metadata": {"taxonomy": ["Bacteria",
"__Cyanobacteria", "__Cyanobacteria", "__o", "__f", "__g"]}},{"id":
"denovo11675", "metadata": {"taxonomy": ["Bacteria",
"__Proteobacteria", "__Alphaproteobacteria", "__Rhodospirillales",
"__Rhodospirillaceae"]}},{"id": "denovo11677", "metadata":
{"taxonomy": ["Bacteria", "__Bacteroidetes", "__Flavobacteria",
"__Flavobacteriales", "__Flavobacteriaceae", "__Psychroserpens"]}},

{"id": "denovo11682", "metadata": {"taxonomy": ["Bacteria", "__Proteobacteria", "__Alphaproteobacteria", "__Rhizobiales", "__Rhodobiaceae", "__Anderseniella"]}},{"id": "denovo11684", "metadata": {"taxonomy": ["Bacteria", "__Proteobacteria", "__Alphaproteobacteria", "__Rhodobacterales", "__Rhodobacteraceae", "__Marinovum"]}},{"id": "denovo11688", "metadata": {"taxonomy": ["Bacteria", "__Proteobacteria", "__Alphaproteobacteria", "__Rhodospirillales"]}},{"id": "denovo11690", "metadata": {"taxonomy": ["Bacteria", "__Chloroflexi", "__Thermomicrobia", "__JG30-KF-CM45", "__f", "__g"]}},{"id": "denovo11692", "metadata": {"taxonomy": ["Bacteria", "__Proteobacteria", "__Deltaproteobacteria", "__Desulfurellales", "__Desulfurellaceae", "__g"]}},{"id": "denovo11694", "metadata": {"taxonomy": ["Bacteria", "__Proteobacteria", "__Gammaproteobacteria"]}},{"id": "denovo11696", "metadata": {"taxonomy": ["Bacteria", "__Actinobacteria", "__Acidimicrobiia", "__Acidimicrobiales", "__Candidatus_Microthrix", "__g"]}},{"id": "denovo11700", "metadata": {"taxonomy": ["Bacteria", "__Proteobacteria", "__Alphaproteobacteria", "__Adriatic90", "__f", "__g"]}},{"id": "denovo11703", "metadata": {"taxonomy": ["Bacteria", "__Proteobacteria", "__Alphaproteobacteria", "__Caulobacterales", "__Hyphomonadaceae", "__g"]}},{"id": "denovo11704", "metadata": {"taxonomy": ["Bacteria", "__Proteobacteria", "__Alphaproteobacteria", "__Rhodobacterales", "__Rhodobacteraceae", "__Tateyamaria"]}},{"id": "denovo11706", "metadata": {"taxonomy": ["Bacteria", "__Proteobacteria", "__Gammaproteobacteria"]}},{"id": "denovo11707", "metadata": {"taxonomy": ["Bacteria", "__Proteobacteria", "__Gammaproteobacteria", "__Order_Incertae_Sedis", "__Family_Incertae_Sedis", "__Marinicella"]}},{"id": "denovo11710", "metadata": {"taxonomy": ["Bacteria", "__Bacteroidetes", "__Flavobacteria", "__Flavobacteriales", "__Flavobacteriaceae", "__Marixanthomonas"]}},{"id": "denovo11713", "metadata": {"taxonomy": ["Bacteria", "__Bacteroidetes", "__Sphingobacteriia", "__Sphingobacteriales", "__Saprospiraceae", "__g"]}},{"id": "denovo11721", "metadata": {"taxonomy": ["Bacteria", "__Proteobacteria", "__Gammaproteobacteria", "__Oceanospirillales"]}},{"id": "denovo11731", "metadata": {"taxonomy": ["Bacteria", "__Proteobacteria", "__Deltaproteobacteria", "__Myxococcales", "__0319-6G20", "__g"]}},{"id": "denovo11733", "metadata": {"taxonomy": ["Bacteria", "__Proteobacteria", "__Gammaproteobacteria", "__Oceanospirillales"]}},{"id": "denovo11735", "metadata": {"taxonomy": ["Bacteria", "__Cyanobacteria", "__Cyanobacteria", "__SubsectionIII", "__FamilyI", "__Leptolyngbya"]}},{"id": "denovo11736", "metadata": {"taxonomy": ["Bacteria", "__Planctomycetes", "__Phycisphaerae", "__Phycisphaerales", "__08D2Z94_hypersaline_microbial_mat_group", "__g"]}},{"id": "denovo11737", "metadata": {"taxonomy": ["Bacteria", "__Chlorobi", "__Chlorobia", "__Chlorobiales", "__OPB56", "__g"]}},{"id": "denovo11741", "metadata": {"taxonomy": ["Bacteria", "__Chloroflexi", "__KD4-96", "__o", "__f", "__g"]}},{"id": "denovo11743", "metadata": {"taxonomy": ["Bacteria", "__Proteobacteria", "__Gammaproteobacteria",

"__Alteromonadales", "__Alteromonadaceae"]}},{"id": "denovo11745", "metadata": {"taxonomy": ["Bacteria", "__Proteobacteria", "__Gammaproteobacteria"]}},{"id": "denovo11746", "metadata": {"taxonomy": ["Bacteria", "__Proteobacteria", "__Gammaproteobacteria", "__Alteromonadales", "__Alteromonadaceae", "__Candidatus_Endobugula"]}},{"id": "denovo11747", "metadata": {"taxonomy": ["Bacteria", "__Proteobacteria", "__Gammaproteobacteria", "__Alteromonadales", "__Alteromonadaceae", "__BD1-7_clade"]}},{"id": "denovo11749", "metadata": {"taxonomy": ["Bacteria", "__Bacteroidetes", "__Sphingobacteriia", "__Sphingobacteriales", "__Saprospiraceae", "__g"]}},{"id": "denovo11752", "metadata": {"taxonomy": ["Bacteria", "__Bacteroidetes", "__Sphingobacteriia", "__Sphingobacteriales", "__Saprospiraceae", "__g"]}},{"id": "denovo11755", "metadata": {"taxonomy": ["Bacteria", "__Proteobacteria", "__Alphaproteobacteria", "__Rhodospirillales", "__Rhodospirillaceae", "__g"]}},{"id": "denovo11756", "metadata": {"taxonomy": ["Bacteria", "__Proteobacteria", "__Alphaproteobacteria", "__Rhodobacterales", "__Rhodobacteraceae", "__Pseudovibrio"]}},{"id": "denovo11758", "metadata": {"taxonomy": ["Bacteria", "__Proteobacteria", "__Deltaproteobacteria", "__Desulfobacterales", "__Desulfobulbaceae", "__g"]}},{"id": "denovo11761", "metadata": {"taxonomy": ["Bacteria", "__Cyanobacteria", "__Cyanobacteria", "__SubsectionI", "__FamilyI", "__Prochlorococcus"]}},{"id": "denovo11762", "metadata": {"taxonomy": ["Bacteria", "__Proteobacteria", "__Gammaproteobacteria", "__Oceanospirillales", "__Hahellaceae", "__Endozoicomonas"]}},{"id": "denovo11765", "metadata": {"taxonomy": ["Bacteria", "__Candidate_division_BRC1", "__c", "__o", "__f", "__g"]}},{"id": "denovo11766", "metadata": {"taxonomy": ["Bacteria", "__Proteobacteria", "__Alphaproteobacteria", "__Rhodospirillales", "__Rhodospirillaceae", "__Defluviicoccus"]}},{"id": "denovo11769", "metadata": {"taxonomy": ["Bacteria", "__Proteobacteria", "__Gammaproteobacteria", "__HOC36", "__f", "__g"]}},{"id": "denovo11770", "metadata": {"taxonomy": ["Bacteria", "__Chlorobi", "__Ignavibacteria", "__Ignavibacteriales", "__IheB3-7", "__g"]}},{"id": "denovo11771", "metadata": {"taxonomy": ["Bacteria", "__Proteobacteria", "__Gammaproteobacteria", "__Order_Incertae_Sedis", "__Family_Incertae_Sedis", "__Marinicella"]}},{"id": "denovo11775", "metadata": {"taxonomy": ["Bacteria", "__Proteobacteria", "__Alphaproteobacteria", "__Caulobacterales", "__Caulobacteraceae", "__g"]}},{"id": "denovo11776", "metadata": {"taxonomy": ["Bacteria", "__Bacteroidetes", "__Cytophagia", "__Cytophagales", "__Flammeovirgaceae", "__g"]}},{"id": "denovo11778", "metadata": {"taxonomy": ["Bacteria", "__Proteobacteria", "__Gammaproteobacteria", "__Xanthomonadales", "__Sinobacteraceae", "__JTB255_marine_benthic_group"]}},{"id": "denovo11782", "metadata": {"taxonomy": ["Bacteria", "__Proteobacteria", "__Alphaproteobacteria", "__Caulobacterales", "__Hyphomonadaceae", "__Ponticaulis"]}},{"id": "denovo11783", "metadata": {"taxonomy": ["Bacteria", "__Proteobacteria", "__Alphaproteobacteria", "__Rhizobiales"]}},{"id": "denovo11786", "metadata": {"taxonomy": ["Bacteria",

"__Proteobacteria", "__Alphaproteobacteria", "__Rhodobacterales", "__Rhodobacteraceae"]}},{"id": "denovo11787", "metadata": {"taxonomy": ["Bacteria", "__Proteobacteria", "__Alphaproteobacteria", "__E6aD10", "__f", "__g"]}},{"id": "denovo11789", "metadata": {"taxonomy": ["Bacteria", "__Acidobacteria", "__RB25", "__o", "__f", "__g"]}},{"id": "denovo11791", "metadata": {"taxonomy": ["Bacteria", "__Proteobacteria", "__Gammaproteobacteria"]}},{"id": "denovo11792", "metadata": {"taxonomy": ["Bacteria", "__Proteobacteria", "__Gammaproteobacteria", "__Alteromonadales", "__Alteromonadaceae", "__Candidatus_Endobugula"]}},{"id": "denovo11794", "metadata": {"taxonomy": ["Bacteria", "__Bacteroidetes", "__Sphingobacteriia", "__Sphingobacteriales", "__Saprospiraceae", "__g"]}},{"id": "denovo11796", "metadata": {"taxonomy": ["Bacteria", "__Bacteroidetes", "__Flavobacteria", "__Flavobacteriales", "__Cryomorphaceae", "__Owenweeksia"]}},{"id": "denovo11797", "metadata": {"taxonomy": ["Bacteria", "__Proteobacteria", "__Alphaproteobacteria", "__Rhodobacterales", "__Rhodobacteraceae"]}},{"id": "denovo11798", "metadata": {"taxonomy": ["Bacteria", "__Proteobacteria", "__Gammaproteobacteria", "__Xanthomonadales", "__Sinobacteraceae", "__JTB255_marine_benthic_group"]}},{"id": "denovo11799", "metadata": {"taxonomy": ["Bacteria", "__Proteobacteria", "__Alphaproteobacteria", "__Rhodospirillales", "__Rhodospirillaceae", "__g"]}},{"id": "denovo11802", "metadata": {"taxonomy": ["Bacteria", "__Proteobacteria", "__Alphaproteobacteria", "__Sphingomonadales", "__Erythrobacteraceae", "__Porphyrobacter"]}},{"id": "denovo11811", "metadata": {"taxonomy": ["Bacteria", "__Proteobacteria", "__Gammaproteobacteria"]}},{"id": "denovo11816", "metadata": {"taxonomy": ["Bacteria", "__Candidate_division_BRC1", "__c", "__o", "__f", "__g"]}},{"id": "denovo11817", "metadata": {"taxonomy": ["Bacteria", "__Proteobacteria", "__Alphaproteobacteria", "__Rickettsiales", "__SAR116_clade", "__g"]}},{"id": "denovo11824", "metadata": {"taxonomy": ["Bacteria", "__Proteobacteria", "__Deltaproteobacteria", "__SAR324_clade(Marine_group_B)", "__f", "__g"]}},{"id": "denovo11832", "metadata": {"taxonomy": ["Bacteria", "__Proteobacteria", "__Gammaproteobacteria"]}},{"id": "denovo11836", "metadata": {"taxonomy": ["Bacteria", "__Proteobacteria", "__Epsilonproteobacteria", "__Campylobacterales", "__Campylobacteraceae", "__Arcobacter"]}},{"id": "denovo11838", "metadata": {"taxonomy": ["Bacteria", "__Proteobacteria", "__Alphaproteobacteria", "__Rhodospirillales", "__Rhodospirillaceae"]}},{"id": "denovo11844", "metadata": {"taxonomy": ["Bacteria", "__Cyanobacteria", "__Cyanobacteria", "__SubsectionIII", "__FamilyI", "__Spirulina"]}},{"id": "denovo11845", "metadata": {"taxonomy": ["Bacteria", "__Spirochaetes", "__Spirochaetes", "__Spirochaetales", "__Spirochaetaceae", "__Spirochaeta"]}},{"id": "denovo11847", "metadata": {"taxonomy": ["Bacteria", "__Actinobacteria", "__Acidimicrobiia", "__Acidimicrobiales", "__Sva0996_marine_group", "__g"]}},{"id": "denovo11848", "metadata": {"taxonomy": ["Bacteria", "__Proteobacteria", "__Gammaproteobacteria", "__Pseudomonadales",

"__Moraxellaceae", "__Acinetobacter"]}},{"id": "denovo11849",
"metadata": {"taxonomy": ["Bacteria", "__Bacteroidetes",
"__Flavobacteria", "__Flavobacteriales", "__NS9_marine_group",
"__g"]}},{"id": "denovo11858", "metadata": {"taxonomy": ["Bacteria",
"__Gemmatimonadetes", "__Gemmatimonadetes",
"__PAUC43f_marine_benthic_group", "__f", "__g"]}},{"id":
"denovo11859", "metadata": {"taxonomy": ["Bacteria",
"__Proteobacteria", "__Alphaproteobacteria", "__Rhizobiales",
"__Hyphomicrobiaceae", "__Filomicrobium"]}},{"id": "denovo11863",
"metadata": {"taxonomy": ["Bacteria", "__Proteobacteria",
"__Deltaproteobacteria", "__Sh765B-TzT-29", "__f", "__g"]}},{"id":
"denovo11865", "metadata": {"taxonomy": ["Bacteria",
"__Bacteroidetes", "__Flavobacteria", "__Flavobacteriales",
"__Flavobacteriaceae", "__Leptobacterium"]}},{"id": "denovo11868",
"metadata": {"taxonomy": ["Bacteria", "__Proteobacteria"]}},{"id":
"denovo11870", "metadata": {"taxonomy": ["Bacteria",
"__Cyanobacteria", "__Cyanobacteria", "__SubsectionIII",
"__FamilyI"]}},{"id": "denovo11876", "metadata": {"taxonomy":
["Bacteria", "__Cyanobacteria", "__Cyanobacteria", "__SubsectionIII",
"__FamilyI", "__Phormidium"]}},{"id": "denovo11879", "metadata":
{"taxonomy": ["Bacteria", "__Proteobacteria", "__Gammaproteobacteria",
"__Alteromonadales", "__Alteromonadaceae", "__Haliea"]}},{"id":
"denovo11880", "metadata": {"taxonomy": ["Bacteria",
"__Proteobacteria", "__TA18", "__o", "__f", "__g"]}},{"id":
"denovo11885", "metadata": {"taxonomy": ["Bacteria",
"__Proteobacteria", "__Gammaproteobacteria", "__KI89A_clade", "__f",
"__g"]}},{"id": "denovo11889", "metadata": {"taxonomy": ["Bacteria",
"__Proteobacteria", "__Deltaproteobacteria", "__Desulfurellales",
"__Desulfurellaceae", "__g"]}},{"id": "denovo11891", "metadata":
{"taxonomy": ["Bacteria", "__Acidobacteria", "__Holophagae",
"__Sva0725", "__f", "__g"]}},{"id": "denovo11893", "metadata":
{"taxonomy": ["Bacteria", "__Firmicutes", "__Clostridia",
"__Clostridiales"]}},{"id": "denovo11894", "metadata": {"taxonomy":
["Bacteria"]}},{"id": "denovo11896", "metadata": {"taxonomy":
["Bacteria", "__Proteobacteria", "__Gammaproteobacteria",
"__Legionellales", "__Coxiellaceae", "__Coxiella"]}},{"id":
"denovo11902", "metadata": {"taxonomy": ["Bacteria",
"__Proteobacteria", "__Gammaproteobacteria", "__Pseudomonadales",
"__Moraxellaceae", "__Acinetobacter"]}},{"id": "denovo11906",
"metadata": {"taxonomy": ["Bacteria", "__Cyanobacteria",
"__Cyanobacteria", "__SubsectionIV", "__FamilyII", "__Rivularia"]}},
{"id": "denovo11907", "metadata": {"taxonomy": ["Bacteria",
"__Proteobacteria", "__Deltaproteobacteria", "__Desulfobacterales",
"__Desulfobulbaceae", "__Desulforhopalus"]}},{"id": "denovo11909",
"metadata": {"taxonomy": ["Bacteria", "__Planctomycetes",
"__Planctomycetacia", "__Planctomycetales", "__Planctomycetaceae",
"__Rhodopirellula"]}},{"id": "denovo11910", "metadata": {"taxonomy":
["Bacteria", "__Bacteroidetes", "__Cytophagia", "__Cytophagales",
"__Flammeovirgaceae", "__Candidatus_Amoebophilus"]}},{"id":
"denovo11913", "metadata": {"taxonomy": ["Bacteria",

"__Proteobacteria", "__Alphaproteobacteria", "__Rhodobacterales", "__Rhodobacteraceae", "__Pseudovibrio"]}},{"id": "denovo11916", "metadata": {"taxonomy": ["Bacteria", "__Proteobacteria", "__Deltaproteobacteria"]}},{"id": "denovo11917", "metadata": {"taxonomy": ["Bacteria", "__Proteobacteria", "__Alphaproteobacteria", "__Rhodospirillales", "__Rhodospirillaceae", "__g"]}},{"id": "denovo11922", "metadata": {"taxonomy": ["Bacteria", "__Proteobacteria", "__Gammaproteobacteria"]}},{"id": "denovo11927", "metadata": {"taxonomy": ["Bacteria", "__Proteobacteria", "__Alphaproteobacteria", "__Rhodobacterales", "__Rhodobacteraceae"]}},{"id": "denovo11929", "metadata": {"taxonomy": ["Bacteria", "__Firmicutes", "__Clostridia", "__Clostridiales"]}},{"id": "denovo11931", "metadata": {"taxonomy": ["Bacteria", "__Proteobacteria", "__Gammaproteobacteria", "__Alteromonadales", "__Alteromonadaceae", "__g"]}},{"id": "denovo11935", "metadata": {"taxonomy": ["Bacteria", "__Proteobacteria", "__Alphaproteobacteria"]}},{"id": "denovo11937", "metadata": {"taxonomy": ["Bacteria", "__Proteobacteria", "__Alphaproteobacteria", "__Rhodobacterales", "__Rhodobacteraceae", "__Pacificibacter"]}},{"id": "denovo11939", "metadata": {"taxonomy": ["Bacteria", "__Proteobacteria", "__Gammaproteobacteria", "__Alteromonadales", "__Alteromonadaceae", "__Haliea"]}},{"id": "denovo11947", "metadata": {"taxonomy": ["Bacteria", "__Proteobacteria", "__Alphaproteobacteria", "__Rhodospirillales", "__Rhodospirillaceae", "__Pelagibius"]}},{"id": "denovo11948", "metadata": {"taxonomy": ["Bacteria", "__Proteobacteria", "__Gammaproteobacteria"]}},{"id": "denovo11949", "metadata": {"taxonomy": ["Bacteria", "__Proteobacteria", "__Gammaproteobacteria", "__KI89A_clade", "__f", "__g"]}},{"id": "denovo11950", "metadata": {"taxonomy": ["Bacteria", "__Spirochaetes", "__Spirochaetes", "__Spirochaetales", "__Spirochaetaceae", "__Spirochaeta"]}},{"id": "denovo11951", "metadata": {"taxonomy": ["Bacteria", "__Cyanobacteria", "__Cyanobacteria", "__SubsectionI", "__FamilyI", "__Synechococcus"]}},{"id": "denovo11956", "metadata": {"taxonomy": ["Bacteria", "__Proteobacteria", "__Gammaproteobacteria", "__Oceanospirillales", "__OM182_clade", "__g"]}},{"id": "denovo11961", "metadata": {"taxonomy": ["Bacteria", "__Proteobacteria", "__Deltaproteobacteria", "__Desulfobacterales", "__Nitrospinaceae", "__Nitrospina"]}},{"id": "denovo11965", "metadata": {"taxonomy": ["Bacteria", "__Proteobacteria", "__Gammaproteobacteria", "__Alteromonadales", "__Alteromonadaceae", "__BD2-7"]}},{"id": "denovo11967", "metadata": {"taxonomy": ["Bacteria", "__Proteobacteria", "__Deltaproteobacteria", "__Bdellovibrionales", "__Bacteriovoracaceae", "__Bacteriovorax"]}},{"id": "denovo11968", "metadata": {"taxonomy": ["Bacteria", "__Bacteroidetes", "__Flavobacteria", "__Flavobacteriales", "__Flavobacteriaceae", "__Polaribacter"]}},{"id": "denovo11969", "metadata": {"taxonomy": ["Bacteria", "__Proteobacteria", "__Alphaproteobacteria", "__Rhodobacterales", "__Rhodobacteraceae"]}},{"id": "denovo11972", "metadata": {"taxonomy": ["Bacteria", "__Gemmatimonadetes", "__Gemmatimonadetes", "__BD2-11_terrestrial_group", "__f", "__g"]}},

{"id": "denovo11973", "metadata": {"taxonomy": ["Bacteria", "__Proteobacteria", "__Gammaproteobacteria", "__Alteromonadales", "__Alteromonadaceae", "__OM60(NOR5)_clade"]}},{"id": "denovo11974", "metadata": {"taxonomy": ["Bacteria", "__Proteobacteria", "__Alphaproteobacteria", "__Rhodospirillales", "__Rhodospirillaceae", "__Defluviicoccus"]}},{"id": "denovo11975", "metadata": {"taxonomy": ["Bacteria", "__Firmicutes", "__Clostridia", "__Halanaerobiales", "__Halobacteroidaceae", "__g"]}},{"id": "denovo11976", "metadata": {"taxonomy": ["Bacteria", "__Actinobacteria", "__Acidimicrobiia", "__Acidimicrobiales", "__Sva0996_marine_group", "__g"]}},{"id": "denovo11977", "metadata": {"taxonomy": ["Bacteria", "__Planctomycetes", "__Planctomycetacia", "__Planctomycetales", "__Planctomycetaceae", "__Rhodopirellula"]}},{"id": "denovo11979", "metadata": {"taxonomy": ["Bacteria", "__Proteobacteria", "__Gammaproteobacteria", "__Legionellales", "__Coxiellaceae", "__Coxiella"]}},{"id": "denovo11981", "metadata": {"taxonomy": ["Bacteria", "__Proteobacteria", "__Alphaproteobacteria", "__Rhodobacterales", "__Rhodobacteraceae", "__Labrenzia"]}},{"id": "denovo11983", "metadata": {"taxonomy": ["Bacteria", "__Bacteroidetes", "__Sphingobacteriia", "__Sphingobacteriales", "__Saprospiraceae", "__g"]}},{"id": "denovo11986", "metadata": {"taxonomy": ["Bacteria", "__Proteobacteria", "__Deltaproteobacteria", "__SAR324_clade(Marine_group_B)", "__f", "__g"]}},{"id": "denovo11990", "metadata": {"taxonomy": ["Bacteria", "__Firmicutes", "__Clostridia", "__Clostridiales", "__Lachnospiraceae", "__Pseudobutyrivibrio"]}},{"id": "denovo11991", "metadata": {"taxonomy": ["Archaea", "__Euryarchaeota", "__Thermoplasmata", "__Thermoplasmatales", "__Marine_Group_II", "__g"]}},{"id": "denovo11992", "metadata": {"taxonomy": ["Bacteria", "__Proteobacteria", "__Gammaproteobacteria", "__Vibrionales", "__Vibrionaceae", "__Aliivibrio"]}},{"id": "denovo11997", "metadata": {"taxonomy": ["Bacteria", "__Proteobacteria", "__AEGEAN-245", "__o", "__f", "__g"]}},{"id": "denovo11998", "metadata": {"taxonomy": ["Bacteria", "__Proteobacteria", "__Alphaproteobacteria", "__SAR11_clade", "__Surface_1"]}},{"id": "denovo12001", "metadata": {"taxonomy": ["Bacteria", "__Bacteroidetes", "__Sphingobacteriia", "__Sphingobacteriales", "__WCHB1-69", "__g"]}},{"id": "denovo12003", "metadata": {"taxonomy": ["Bacteria", "__Proteobacteria", "__Gammaproteobacteria", "__Enterobacteriales", "__Enterobacteriaceae", "__Brenneria"]}},{"id": "denovo12013", "metadata": {"taxonomy": ["Bacteria", "__Chloroflexi", "__Caldilineae", "__Caldilineales", "__Caldilineaceae", "__Caldilinea"]}},{"id": "denovo12019", "metadata": {"taxonomy": ["Bacteria", "__Bacteroidetes", "__Flavobacteria", "__Flavobacteriales", "__Flavobacteriaceae", "__g"]}},{"id": "denovo12023", "metadata": {"taxonomy": ["Bacteria", "__Candidate_division_WS3", "__c", "__o", "__f", "__g"]}},{"id": "denovo12024", "metadata": {"taxonomy": ["Bacteria", "__Proteobacteria", "__Alphaproteobacteria"]}},{"id": "denovo12025", "metadata": {"taxonomy": ["Bacteria", "__Bacteroidetes", "__BD2-2",

"__o", "__f", "__g"]}},{"id": "denovo12026", "metadata": {"taxonomy": ["Bacteria", "__Verrucomicrobia", "__OPB35_soil_group", "__o", "__f", "__g"]}},{"id": "denovo12027", "metadata": {"taxonomy": ["Bacteria", "__Proteobacteria", "__Alphaproteobacteria", "__Rhodospirillales", "__Rhodospirillaceae", "__Rhodovibrio"]}},{"id": "denovo12032", "metadata": {"taxonomy": ["Bacteria", "__Proteobacteria", "__Gammaproteobacteria", "__Legionellales", "__Coxiellaceae", "__Aquicella"]}},{"id": "denovo12033", "metadata": {"taxonomy": ["Bacteria", "__Kazan-3B-28", "__c", "__o", "__f", "__g"]}},{"id": "denovo12034", "metadata": {"taxonomy": ["Bacteria", "__Firmicutes", "__Clostridia", "__Clostridiales", "__Clostridiaceae", "__Clostridium"]}},{"id": "denovo12038", "metadata": {"taxonomy": ["Bacteria"]}},{"id": "denovo12039", "metadata": {"taxonomy": ["Bacteria", "__Proteobacteria", "__Alphaproteobacteria", "__Caulobacterales", "__Hyphomonadaceae", "__Hyphomonas"]}},{"id": "denovo12042", "metadata": {"taxonomy": ["Bacteria", "__Chloroflexi", "__Caldilineae", "__Caldilineales", "__Caldilineaceae", "__Caldilinea"]}},{"id": "denovo12048", "metadata": {"taxonomy": ["Bacteria", "__Proteobacteria", "__Gammaproteobacteria", "__Alteromonadales", "__Pseudoalteromonadaceae", "__Pseudoalteromonas"]}},{"id": "denovo12058", "metadata": {"taxonomy": ["Bacteria", "__Deferribacteres", "__Deferribacteres", "__Deferribacterales", "__Family_Incertae_Sedis", "__Caldithrix"]}},{"id": "denovo12059", "metadata": {"taxonomy": ["Archaea", "__Euryarchaeota", "__Halobacteria", "__Halobacteriales", "__Deep_Sea_Hydrothermal_Vent_Gp_6(DHVEG-6)", "__g"]}},{"id": "denovo12060", "metadata": {"taxonomy": ["Bacteria", "__Nitrospirae", "__Nitrospira", "__Nitrospirales", "__Nitrospiraceae", "__Nitrospira"]}},{"id": "denovo12061", "metadata": {"taxonomy": ["Bacteria", "__Acidobacteria", "__Acidobacteria", "__PAUC26f", "__f", "__g"]}},{"id": "denovo12062", "metadata": {"taxonomy": ["Bacteria", "__Bacteroidetes", "__Cytophagia", "__Cytophagales", "__Flammeovirgaceae", "__Candidatus_Amoebophilus"]}},{"id": "denovo12064", "metadata": {"taxonomy": ["Bacteria", "__Proteobacteria"]}},{"id": "denovo12071", "metadata": {"taxonomy": ["Bacteria", "__Proteobacteria", "__Deltaproteobacteria", "__Bdellovibrionales", "__Bacteriovoracaceae", "__Deferrisoma"]}},{"id": "denovo12075", "metadata": {"taxonomy": ["Bacteria", "__Proteobacteria", "__Gammaproteobacteria", "__Legionellales", "__Coxiellaceae", "__Coxiella"]}},{"id": "denovo12078", "metadata": {"taxonomy": ["Bacteria", "__Proteobacteria", "__Gammaproteobacteria"]}},{"id": "denovo12079", "metadata": {"taxonomy": ["Bacteria", "__Chloroflexi", "__Caldilineae", "__Caldilineales", "__Caldilineaceae", "__g"]}},{"id": "denovo12083", "metadata": {"taxonomy": ["Bacteria", "__Bacteroidetes", "__Flavobacteria", "__Flavobacteriales", "__Cryomorphaceae", "__NS10_marine_group"]}},{"id": "denovo12085", "metadata": {"taxonomy": ["Bacteria", "__Proteobacteria", "__Alphaproteobacteria", "__Rickettsiales", "__SAR116_clade", "__g"]}},{"id": "denovo12086", "metadata": {"taxonomy": ["Bacteria", "__Proteobacteria",

"__Alphaproteobacteria", "__Rhodobacterales", "__Rhodobacteraceae", "__Roseobacter_clade_OCT_lineage"]}},{"id": "denovo12094", "metadata": {"taxonomy": ["Bacteria", "__Bacteroidetes"]}},{"id": "denovo12095", "metadata": {"taxonomy": ["Bacteria", "__Bacteroidetes", "__Flavobacteria", "__Flavobacteriales", "__Cryomorphaceae"]}},{"id": "denovo12096", "metadata": {"taxonomy": ["Bacteria", "__Bacteroidetes", "__Flavobacteria", "__Flavobacteriales", "__Flavobacteriaceae", "__Leptobacterium"]}},{"id": "denovo12097", "metadata": {"taxonomy": ["Bacteria", "__Proteobacteria", "__Deltaproteobacteria", "__Bdellovibrionales", "__Bacteriovoracaceae", "__Peredibacter"]}},{"id": "denovo12098", "metadata": {"taxonomy": ["Bacteria", "__Proteobacteria", "__Alphaproteobacteria", "__Rickettsiales", "__LWSR-14", "__g"]}},{"id": "denovo12107", "metadata": {"taxonomy": ["Bacteria", "__Proteobacteria", "__Alphaproteobacteria", "__Rhodospirillales", "__MSB-1E8", "__g"]}},{"id": "denovo12112", "metadata": {"taxonomy": ["Bacteria", "__Bacteroidetes", "__ML602M-17", "__o", "__f", "__g"]}},{"id": "denovo12113", "metadata": {"taxonomy": ["Bacteria", "__Bacteroidetes", "__Flavobacteria", "__Flavobacteriales", "__Flavobacteriaceae"]}},{"id": "denovo12116", "metadata": {"taxonomy": ["Bacteria", "__Actinobacteria", "__Actinobacteria", "__PeM15", "__f", "__g"]}},{"id": "denovo12117", "metadata": {"taxonomy": ["Bacteria", "__Proteobacteria", "__Alphaproteobacteria"]}},{"id": "denovo12118", "metadata": {"taxonomy": ["Bacteria", "__Bacteroidetes", "__Flavobacteria", "__Flavobacteriales", "__Flavobacteriaceae", "__Gilvibacter"]}},{"id": "denovo12127", "metadata": {"taxonomy": ["Bacteria", "__Bacteroidetes", "__Flavobacteria", "__Flavobacteriales", "__Flavobacteriaceae", "__NS4_marine_group"]}},{"id": "denovo12128", "metadata": {"taxonomy": ["Bacteria", "__Bacteroidetes", "__Cytophagia", "__Cytophagales", "__Cyclobacteriaceae", "__g"]}},{"id": "denovo12129", "metadata": {"taxonomy": ["Bacteria", "__Proteobacteria"]}},{"id": "denovo12130", "metadata": {"taxonomy": ["Bacteria", "__Proteobacteria", "__Deltaproteobacteria", "__Bdellovibrionales", "__Bacteriovoracaceae", "__Peredibacter"]}},{"id": "denovo12132", "metadata": {"taxonomy": ["Bacteria", "__Proteobacteria", "__Gammaproteobacteria", "__Vibrionales", "__Vibrionaceae", "__Photobacterium"]}},{"id": "denovo12135", "metadata": {"taxonomy": ["Bacteria", "__Proteobacteria", "__Alphaproteobacteria", "__Rhizobiales"]}},{"id": "denovo12137", "metadata": {"taxonomy": ["Bacteria", "__Proteobacteria", "__Gammaproteobacteria", "__Alteromonadales", "__Alteromonadaceae", "__OM60(NOR5)_clade"]}},{"id": "denovo12140", "metadata": {"taxonomy": ["Bacteria", "__Proteobacteria", "__Deltaproteobacteria", "__Bdellovibrionales", "__Bacteriovoracaceae", "__Peredibacter"]}},{"id": "denovo12142", "metadata": {"taxonomy": ["Bacteria", "__Proteobacteria", "__Gammaproteobacteria", "__Alteromonadales", "__Psychromonadaceae", "__Psychromonas"]}},{"id": "denovo12143", "metadata": {"taxonomy": ["Bacteria", "__Proteobacteria", "__Alphaproteobacteria", "__Sphingomonadales", "__Sphingomonadaceae",

"__Sphingomonas"]}},{"id": "denovo12148", "metadata": {"taxonomy": ["Bacteria", "__Proteobacteria", "__Gammaproteobacteria", "__Oceanospirillales", "__Oceanospirillaceae", "__Oceanobacter"]}}, {"id": "denovo12149", "metadata": {"taxonomy": ["Bacteria", "__Proteobacteria", "__Alphaproteobacteria", "__Rhodobacterales", "__Rhodobacteraceae"]}},{"id": "denovo12152", "metadata": {"taxonomy": ["Bacteria", "__Proteobacteria", "__Gammaproteobacteria", "__Alteromonadales", "__Alteromonadaceae"]}},{"id": "denovo12154", "metadata": {"taxonomy": ["Bacteria", "__Firmicutes", "__Clostridia", "__Clostridiales"]}},{"id": "denovo12159", "metadata": {"taxonomy": ["Bacteria", "__Bacteroidetes", "__Cytophagia", "__Cytophagales", "__Flammeovirgaceae"]}},{"id": "denovo12160", "metadata": {"taxonomy": ["Bacteria", "__Proteobacteria", "__Deltaproteobacteria", "__Bdellovibrionales", "__Bdellovibrionaceae", "__OM27_clade"]}}, {"id": "denovo12164", "metadata": {"taxonomy": ["Bacteria", "__Bacteroidetes", "__Cytophagia", "__Cytophagales", "__Flammeovirgaceae", "__g"]}},{"id": "denovo12173", "metadata": {"taxonomy": ["Bacteria", "__Proteobacteria", "__Deltaproteobacteria", "__Myxococcales", "__0319-6G20", "__g"]}},{"id": "denovo12174", "metadata": {"taxonomy": ["Bacteria", "__Proteobacteria", "__Gammaproteobacteria", "__Alteromonadales", "__Alteromonadaceae"]}}, {"id": "denovo12175", "metadata": {"taxonomy": ["Bacteria", "__Bacteroidetes", "__Flavobacteria", "__Flavobacteriales", "__Cryomorphaceae", "__Fluviicola"]}},{"id": "denovo12178", "metadata": {"taxonomy": ["Bacteria", "__Planctomycetes", "__Phycisphaerae", "__Phycisphaerales", "__Phycisphaeraceae", "__Phycisphaera"]}},{"id": "denovo12181", "metadata": {"taxonomy": ["Bacteria", "__Spirochaetes", "__Spirochaetes", "__Spirochaetales", "__Leptospiraceae", "__g"]}},{"id": "denovo12185", "metadata": {"taxonomy": ["Bacteria", "__Proteobacteria", "__Deltaproteobacteria", "__Sh765B-TzT-29", "__f", "__g"]}},{"id": "denovo12186", "metadata": {"taxonomy": ["Bacteria", "__Chloroflexi", "__SAR202_clade", "__o", "__f", "__g"]}},{"id": "denovo12188", "metadata": {"taxonomy": ["Bacteria", "__Firmicutes", "__Clostridia", "__Halanaerobiales", "__64K2", "__g"]}},{"id": "denovo12191", "metadata": {"taxonomy": ["Bacteria", "__Proteobacteria", "__Gammaproteobacteria", "__Pseudomonadales", "__Moraxellaceae", "__Acinetobacter"]}},{"id": "denovo12192", "metadata": {"taxonomy": ["Bacteria", "__Proteobacteria", "__Alphaproteobacteria", "__Rhodobacterales", "__Rhodobacteraceae", "__Loktanella"]}},{"id": "denovo12194", "metadata": {"taxonomy": ["Bacteria", "__Proteobacteria", "__Gammaproteobacteria", "__o", "__f", "__g"]}},{"id": "denovo12195", "metadata": {"taxonomy": ["Bacteria", "__Proteobacteria", "__Gammaproteobacteria", "__Order_Incertae_Sedis", "__Family_Incertae_Sedis", "__Arenicella"]}},{"id": "denovo12197", "metadata": {"taxonomy": ["Bacteria", "__Bacteroidetes"]}},{"id": "denovo12202", "metadata": {"taxonomy": ["Bacteria", "__Proteobacteria", "__Deltaproteobacteria", "__Desulfobacterales", "__Desulfobulbaceae", "__Desulfobulbus"]}},{"id": "denovo12205", "metadata": {"taxonomy": ["Bacteria", "__Proteobacteria",

"__Gammaproteobacteria", "__Xanthomonadales", "__Sinobacteraceae", "__JTB255_marine_benthic_group"]}},{"id": "denovo12207", "metadata": {"taxonomy": ["Bacteria", "__Deinococcus-Thermus", "__Deinococci", "__Deinococcales", "__Trueperaceae", "__Truepera"]}},{"id": "denovo12208", "metadata": {"taxonomy": ["Bacteria", "__Proteobacteria", "__Gammaproteobacteria", "__Xanthomonadales", "__Sinobacteraceae"]}},{"id": "denovo12214", "metadata": {"taxonomy": ["Bacteria", "__Bacteroidetes", "__Flavobacteria", "__Flavobacteriales", "__Flavobacteriaceae", "__Tenacibaculum"]}},{"id": "denovo12215", "metadata": {"taxonomy": ["Bacteria", "__Proteobacteria", "__Deltaproteobacteria", "__Bdellovibrionales", "__Bacteriovoracaceae", "__Peredibacter"]}},{"id": "denovo12218", "metadata": {"taxonomy": ["Bacteria", "__Proteobacteria", "__Gammaproteobacteria", "__Pseudomonadales", "__Moraxellaceae", "__Acinetobacter"]}},{"id": "denovo12219", "metadata": {"taxonomy": ["Bacteria", "__Proteobacteria", "__Alphaproteobacteria", "__Sphingomonadales", "__Sphingomonadaceae", "__Sphingopyxis"]}},{"id": "denovo12220", "metadata": {"taxonomy": ["Bacteria", "__Proteobacteria", "__Gammaproteobacteria", "__Pseudomonadales", "__Moraxellaceae", "__Acinetobacter"]}},{"id": "denovo12222", "metadata": {"taxonomy": ["Bacteria", "__Proteobacteria", "__Deltaproteobacteria", "__Myxococcales", "__Sorangiineae", "__Sandaracinaceae"]}},{"id": "denovo12223", "metadata": {"taxonomy": ["Bacteria", "__Acidobacteria", "__Acidobacteria", "__PAUC26f", "__f", "__g"]}},{"id": "denovo12226", "metadata": {"taxonomy": ["Bacteria", "__Proteobacteria", "__Gammaproteobacteria", "__Alteromonadales", "__Alteromonadaceae"]}},{"id": "denovo12228", "metadata": {"taxonomy": ["Bacteria", "__Proteobacteria", "__Alphaproteobacteria", "__Kordiimonadales", "__Kordiimonadaceae", "__Kordiimonas"]}},{"id": "denovo12229", "metadata": {"taxonomy": ["Bacteria", "__Candidate_division_BRC1", "__c", "__o", "__f", "__g"]}},{"id": "denovo12232", "metadata": {"taxonomy": ["Bacteria", "__Proteobacteria", "__Alphaproteobacteria", "__Rhizobiales", "__Bradyrhizobiaceae", "__Bosea"]}},{"id": "denovo12236", "metadata": {"taxonomy": ["Bacteria", "__Firmicutes", "__Clostridia", "__Halanaerobiales", "__Halobacteroidaceae", "__g"]}},{"id": "denovo12239", "metadata": {"taxonomy": ["Bacteria", "__Proteobacteria", "__Gammaproteobacteria", "__Oceanospirillales", "__Hahellaceae", "__Endozoicomonas"]}},{"id": "denovo12240", "metadata": {"taxonomy": ["Bacteria", "__Acidobacteria", "__Acidobacteria", "__Candidatus_Chloracidobacterium", "__f", "__g"]}},{"id": "denovo12243", "metadata": {"taxonomy": ["Bacteria", "__Proteobacteria", "__Gammaproteobacteria", "__Chromatiales", "__Granulosicoccaceae", "__Granulosicoccus"]}},{"id": "denovo12252", "metadata": {"taxonomy": ["Bacteria", "__BD1-5", "__c", "__o", "__f", "__g"]}},{"id": "denovo12253", "metadata": {"taxonomy": ["Bacteria", "__Proteobacteria", "__Alphaproteobacteria"]}},{"id": "denovo12254", "metadata": {"taxonomy": ["Bacteria", "__Bacteroidetes", "__Sphingobacteriia", "__Sphingobacteriales", "__Saprospiraceae", "__g"]}},{"id": "denovo12256", "metadata": {"taxonomy": ["Bacteria",

"__Proteobacteria", "__Alphaproteobacteria", "__Sneathiellales", "__Sneathiellaceae", "__Sneathiella"]}},{"id": "denovo12257", "metadata": {"taxonomy": ["Bacteria", "__Proteobacteria", "__Alphaproteobacteria", "__Rhodobacterales", "__Rhodobacteraceae"]}}, {"id": "denovo12259", "metadata": {"taxonomy": ["Bacteria", "__Proteobacteria", "__Alphaproteobacteria", "__Rhodobacterales", "__Rhodobacteraceae", "__Roseibacterium"]}},{"id": "denovo12264", "metadata": {"taxonomy": ["Bacteria", "__Bacteroidetes", "__Sphingobacteriia", "__Sphingobacteriales", "__Saprospiraceae", "__g"]}},{"id": "denovo12266", "metadata": {"taxonomy": ["Bacteria", "__Bacteroidetes", "__BD2-2", "__o", "__f", "__g"]}},{"id": "denovo12268", "metadata": {"taxonomy": ["Bacteria", "__Bacteroidetes", "__Sphingobacteriia", "__Sphingobacteriales", "__Saprospiraceae", "__g"]}},{"id": "denovo12269", "metadata": {"taxonomy": ["Bacteria", "__Deferribacteres", "__Deferribacteres", "__Deferribacterales", "__SAR406_clade(Marine_group_A)", "__g"]}}, {"id": "denovo12270", "metadata": {"taxonomy": ["Bacteria", "__Proteobacteria", "__Gammaproteobacteria", "__Alteromonadales", "__Alteromonadaceae", "__BD1-7_clade"]}},{"id": "denovo12273", "metadata": {"taxonomy": ["Bacteria", "__Proteobacteria", "__Gammaproteobacteria", "__Chromatiales"]}},{"id": "denovo12275", "metadata": {"taxonomy": ["Bacteria", "__Bacteroidetes", "__Cytophagia", "__Cytophagales", "__Flammeovirgaceae", "__g"]}}, {"id": "denovo12283", "metadata": {"taxonomy": ["Bacteria", "__Cyanobacteria", "__ML635J-21", "__o", "__f", "__g"]}},{"id": "denovo12297", "metadata": {"taxonomy": ["Bacteria", "__Actinobacteria", "__Actinobacteria", "__Corynebacteriales", "__Dietziaceae", "__Dietzia"]}},{"id": "denovo12299", "metadata": {"taxonomy": ["Bacteria", "__Proteobacteria", "__Deltaproteobacteria", "__Desulfobacterales", "__Nitrospinaceae", "__Candidatus_Entotheonella"]}},{"id": "denovo12301", "metadata": {"taxonomy": ["Bacteria", "__Proteobacteria", "__Gammaproteobacteria", "__Oceanospirillales", "__Oceanospirillaceae", "__Pseudospirillum"]}}, {"id": "denovo12304", "metadata": {"taxonomy": ["Bacteria", "__Bacteroidetes", "__Flavobacteria", "__Flavobacteriales", "__Cryomorphaceae", "__Owenweeksia"]}},{"id": "denovo12307", "metadata": {"taxonomy": ["Bacteria", "__Proteobacteria", "__Alphaproteobacteria", "__Rhodospirillales", "__Rhodospirillaceae", "__Defluviicoccus"]}},{"id": "denovo12308", "metadata": {"taxonomy": ["Bacteria", "__Proteobacteria", "__AEGEAN-245", "__o", "__f", "__g"]}},{"id": "denovo12309", "metadata": {"taxonomy": ["Bacteria", "__Proteobacteria", "__Gammaproteobacteria", "__Chromatiales", "__Chromatiaceae", "__Nitrosococcus"]}},{"id": "denovo12318", "metadata": {"taxonomy": ["Bacteria", "__Proteobacteria", "__Alphaproteobacteria", "__Rhodospirillales", "__Rhodospirillaceae", "__g"]}},{"id": "denovo12321", "metadata": {"taxonomy": ["Bacteria", "__Acidobacteria", "__RB25", "__o", "__f", "__g"]}},{"id": "denovo12322", "metadata": {"taxonomy": ["Bacteria", "__Bacteroidetes", "__Flavobacteria", "__Flavobacteriales", "__Flavobacteriaceae"]}},{"id": "denovo12324", "metadata":

{"taxonomy": ["Bacteria", "__Bacteroidetes", "__Bacteroidia", "__Bacteroidales", "__Marinilabiaceae", "__Marinifilum"]}},{"id": "denovo12328", "metadata": {"taxonomy": ["Bacteria", "__Proteobacteria", "__Alphaproteobacteria", "__Rhizobiales", "__Hyphomicrobiaceae", "__Hyphomicrobium"]}},{"id": "denovo12331", "metadata": {"taxonomy": ["Bacteria", "__Planctomycetes", "__Phycisphaerae", "__mle1-8", "__f", "__g"]}},{"id": "denovo12337", "metadata": {"taxonomy": ["Bacteria", "__Proteobacteria", "__Gammaproteobacteria", "__Oceanospirillales", "__Hahellaceae", "__Endozoicomonas"]}},{"id": "denovo12341", "metadata": {"taxonomy": ["Bacteria", "__Proteobacteria", "__Alphaproteobacteria", "__Rhodospirillales", "__Rhodospirillaceae"]}},{"id": "denovo12343", "metadata": {"taxonomy": ["Bacteria", "__Bacteroidetes", "__Cytophagia", "__Cytophagales", "__Flammeovirgaceae", "__g"]}},{"id": "denovo12344", "metadata": {"taxonomy": ["Bacteria", "__Proteobacteria", "__Alphaproteobacteria", "__Rhizobiales", "__Bradyrhizobiaceae", "__Bosea"]}},{"id": "denovo12346", "metadata": {"taxonomy": ["Bacteria", "__Proteobacteria", "__Alphaproteobacteria", "__Rhodobacterales", "__Rhodobacteraceae"]}},{"id": "denovo12347", "metadata": {"taxonomy": ["Bacteria", "__Proteobacteria", "__Gammaproteobacteria", "__Xanthomonadales", "__Sinobacteraceae", "__JTB255_marine_benthic_group"]}},{"id": "denovo12349", "metadata": {"taxonomy": ["Bacteria", "__Proteobacteria", "__Deltaproteobacteria", "__Myxococcales", "__Haliangiaceae", "__Haliangium"]}},{"id": "denovo12360", "metadata": {"taxonomy": ["Bacteria", "__Proteobacteria", "__Alphaproteobacteria", "__Rhodobacterales", "__Rhodobacteraceae"]}},{"id": "denovo12361", "metadata": {"taxonomy": ["Bacteria", "__Proteobacteria", "__Alphaproteobacteria", "__Rhodobacterales", "__Rhodobacteraceae"]}},{"id": "denovo12362", "metadata": {"taxonomy": ["Bacteria", "__Proteobacteria", "__Gammaproteobacteria", "__KI89A_clade", "__f", "__g"]}},{"id": "denovo12363", "metadata": {"taxonomy": ["Bacteria", "__Proteobacteria", "__Deltaproteobacteria", "__Desulfarculales", "__Desulfarculaceae", "__g"]}},{"id": "denovo12370", "metadata": {"taxonomy": ["Bacteria", "__Cyanobacteria", "__Cyanobacteria", "__SubsectionIII", "__FamilyI"]}},{"id": "denovo12374", "metadata": {"taxonomy": ["Bacteria", "__Proteobacteria", "__Alphaproteobacteria", "__Rickettsiales", "__Rickettsiaceae"]}},{"id": "denovo12376", "metadata": {"taxonomy": ["Bacteria", "__Proteobacteria", "__Gammaproteobacteria", "__Legionellales", "__Legionellaceae", "__g"]}},{"id": "denovo12382", "metadata": {"taxonomy": ["Bacteria", "__Proteobacteria", "__JTB23", "__o", "__f", "__g"]}},{"id": "denovo12387", "metadata": {"taxonomy": ["Bacteria", "__Proteobacteria", "__Alphaproteobacteria", "__Rhodospirillales", "__Rhodospirillaceae", "__g"]}},{"id": "denovo12388", "metadata": {"taxonomy": ["Bacteria", "__Bacteroidetes", "__BD2-2", "__o", "__f", "__g"]}},{"id": "denovo12389", "metadata": {"taxonomy": ["Bacteria", "__Proteobacteria", "__Deltaproteobacteria", "__SAR324_clade(Marine_group_B)", "__f", "__g"]}},{"id": "denovo12390", "metadata": {"taxonomy": ["Bacteria",

"__Proteobacteria", "__Gammaproteobacteria", "__Vibrionales", "__Vibrionaceae", "__Grimontia"]}},{"id": "denovo12391", "metadata": {"taxonomy": ["Bacteria", "__Proteobacteria", "__Alphaproteobacteria", "__Rhizobiales", "__Hyphomicrobiaceae", "__Devosia"]}},{"id": "denovo12393", "metadata": {"taxonomy": ["Bacteria", "__Proteobacteria", "__Alphaproteobacteria", "__Rhodobacterales", "__Rhodobacteraceae", "__Pseudovibrio"]}},{"id": "denovo12394", "metadata": {"taxonomy": ["Bacteria", "__Proteobacteria", "__Gammaproteobacteria", "__Vibrionales", "__Vibrionaceae", "__Aliivibrio"]}},{"id": "denovo12399", "metadata": {"taxonomy": ["Bacteria", "__Proteobacteria", "__Deltaproteobacteria", "__Sh765B-TzT-29", "__f", "__g"]}},{"id": "denovo12401", "metadata": {"taxonomy": ["Bacteria", "__Bacteroidetes", "__Cytophagia", "__Cytophagales", "__Flammeovirgaceae", "__g"]}},{"id": "denovo12403", "metadata": {"taxonomy": ["Bacteria", "__Verrucomicrobia", "__OPB35_soil_group", "__o", "__f", "__g"]}},{"id": "denovo12406", "metadata": {"taxonomy": ["Bacteria", "__Proteobacteria", "__Gammaproteobacteria", "__Order_Incertae_Sedis", "__Family_Incertae_Sedis", "__Arenicella"]}},{"id": "denovo12408", "metadata": {"taxonomy": ["Bacteria", "__Proteobacteria", "__Deltaproteobacteria", "__GR-WP33-30", "__f", "__g"]}},{"id": "denovo12409", "metadata": {"taxonomy": ["Bacteria", "__Proteobacteria", "__Alphaproteobacteria", "__Rhodobacterales", "__Rhodobacteraceae", "__Rhodobacter"]}},{"id": "denovo12410", "metadata": {"taxonomy": ["Bacteria", "__Planctomycetes", "__Planctomycetacia", "__Planctomycetales", "__Planctomycetaceae"]}},{"id": "denovo12411", "metadata": {"taxonomy": ["Bacteria", "__Proteobacteria", "__Alphaproteobacteria", "__Rhizobiales", "__Hyphomicrobiaceae", "__Devosia"]}},{"id": "denovo12414", "metadata": {"taxonomy": ["Bacteria", "__Candidate_division_WS3", "__c", "__o", "__f", "__g"]}},{"id": "denovo12420", "metadata": {"taxonomy": ["Bacteria", "__Proteobacteria", "__Alphaproteobacteria", "__Rickettsiales", "__Rickettsiaceae"]}},{"id": "denovo12421", "metadata": {"taxonomy": ["Bacteria", "__Proteobacteria", "__Gammaproteobacteria", "__Xanthomonadales", "__Sinobacteraceae", "__JTB255_marine_benthic_group"]}},{"id": "denovo12426", "metadata": {"taxonomy": ["Bacteria", "__Actinobacteria", "__Acidimicrobiia", "__Acidimicrobiales", "__TM214", "__g"]}},{"id": "denovo12428", "metadata": {"taxonomy": ["Bacteria", "__Cyanobacteria", "__Cyanobacteria", "__SubsectionIII", "__FamilyI", "__Prochlorothrix"]}},{"id": "denovo12431", "metadata": {"taxonomy": ["Bacteria", "__Bacteroidetes", "__Bacteroidia", "__Bacteroidales", "__Marinilabiaceae", "__Marinifilum"]}},{"id": "denovo12433", "metadata": {"taxonomy": ["Bacteria", "__Proteobacteria", "__Deltaproteobacteria", "__Desulfobacterales", "__Desulfobacteraceae", "__g"]}},{"id": "denovo12435", "metadata": {"taxonomy": ["Bacteria", "__Proteobacteria", "__Gammaproteobacteria", "__Methylococcales", "__Methylococcaceae", "__Methylomonas"]}},{"id": "denovo12436", "metadata": {"taxonomy": ["Bacteria", "__Proteobacteria", "__Gammaproteobacteria", "__Oceanospirillales"]}},

{"id": "denovo12438", "metadata": {"taxonomy": ["Bacteria", "__Bacteroidetes", "__Flavobacteria", "__Flavobacteriales", "__NS9_marine_group", "__g"]}},{"id": "denovo12446", "metadata": {"taxonomy": ["Bacteria", "__Cyanobacteria", "__Cyanobacteria", "__SubsectionI", "__FamilyI", "__Synechococcus"]}},{"id": "denovo12449", "metadata": {"taxonomy": ["Bacteria", "__Proteobacteria", "__TA18", "__o", "__f", "__g"]}},{"id": "denovo12450", "metadata": {"taxonomy": ["Bacteria", "__Proteobacteria", "__Gammaproteobacteria", "__KI89A_clade", "__f", "__g"]}},{"id": "denovo12453", "metadata": {"taxonomy": ["Bacteria", "__Proteobacteria", "__Alphaproteobacteria", "__Rhodospirillales", "__Rhodospirillaceae"]}},{"id": "denovo12457", "metadata": {"taxonomy": ["Bacteria", "__Lentisphaerae", "__Lentisphaeria", "__Victivallales", "__Victivallaceae", "__g"]}},{"id": "denovo12463", "metadata": {"taxonomy": ["Bacteria", "__Actinobacteria", "__Acidimicrobiia", "__Acidimicrobiales", "__Sva0996_marine_group", "__g"]}},{"id": "denovo12465", "metadata": {"taxonomy": ["Bacteria", "__Proteobacteria", "__Gammaproteobacteria", "__Chromatiales", "__Chromatiaceae", "__Nitrosococcus"]}},{"id": "denovo12467", "metadata": {"taxonomy": ["Bacteria", "__Bacteroidetes", "__Cytophagia", "__Cytophagales", "__Flammeovirgaceae", "__Candidatus_Amoebophilus"]}},{"id": "denovo12468", "metadata": {"taxonomy": ["Bacteria", "__Firmicutes", "__Clostridia", "__Clostridiales", "__Lachnospiraceae"]}},{"id": "denovo12471", "metadata": {"taxonomy": ["Bacteria", "__Proteobacteria", "__Gammaproteobacteria", "__Order_Incertae_Sedis", "__Family_Incertae_Sedis", "__Arenicella"]}},{"id": "denovo12482", "metadata": {"taxonomy": ["Bacteria", "__Acidobacteria", "__Holophagae", "__CA002", "__f", "__g"]}},{"id": "denovo12484", "metadata": {"taxonomy": ["Bacteria", "__Proteobacteria", "__Gammaproteobacteria", "__Oceanospirillales", "__E12-CR02-full", "__g"]}},{"id": "denovo12486", "metadata": {"taxonomy": ["Bacteria", "__Proteobacteria", "__Gammaproteobacteria", "__KI89A_clade", "__f", "__g"]}},{"id": "denovo12488", "metadata": {"taxonomy": ["Bacteria", "__Proteobacteria", "__Gammaproteobacteria", "__Oceanospirillales"]}},{"id": "denovo12499", "metadata": {"taxonomy": ["Bacteria", "__Chloroflexi", "__JG30-KF-CM66", "__o", "__f", "__g"]}},{"id": "denovo12500", "metadata": {"taxonomy": ["Bacteria", "__Firmicutes", "__Bacilli", "__Lactobacillales", "__Lactobacillaceae", "__Lactobacillus"]}},{"id": "denovo12503", "metadata": {"taxonomy": ["Bacteria", "__Proteobacteria", "__Gammaproteobacteria"]}},{"id": "denovo12504", "metadata": {"taxonomy": ["Bacteria", "__Actinobacteria", "__Actinobacteria", "__Micrococcales", "__Micrococcaceae", "__Micrococcus"]}},{"id": "denovo12510", "metadata": {"taxonomy": ["Bacteria", "__Proteobacteria", "__JTB23", "__o", "__f", "__g"]}},{"id": "denovo12511", "metadata": {"taxonomy": ["Bacteria", "__Planctomycetes", "__Planctomycetacia", "__Planctomycetales", "__Planctomycetaceae", "__Rhodopirellula"]}},{"id": "denovo12512", "metadata": {"taxonomy": ["Bacteria", "__Proteobacteria", "__Deltaproteobacteria", "__Desulfuromonadales",

"__GR-WP33-58", "__g"]}},{"id": "denovo12519", "metadata": {"taxonomy": ["Bacteria", "__Proteobacteria", "__Deltaproteobacteria", "__Myxococcales", "__Sorangiineae", "__Sandaracinaceae"]}},{"id": "denovo12522", "metadata": {"taxonomy": ["Bacteria", "__Chloroflexi", "__Caldilineae", "__Caldilineales", "__Caldilineaceae", "__Caldilinea"]}},{"id": "denovo12526", "metadata": {"taxonomy": ["Bacteria", "__Cyanobacteria", "__Cyanobacteria", "__SubsectionI", "__FamilyI", "__Synechococcus"]}},{"id": "denovo12527", "metadata": {"taxonomy": ["Bacteria", "__Proteobacteria", "__Betaproteobacteria", "__Burkholderiales", "__Oxalobacteraceae", "__Massilia"]}},{"id": "denovo12529", "metadata": {"taxonomy": ["Bacteria", "__Proteobacteria", "__Betaproteobacteria", "__Hydrogenophilales", "__Hydrogenophilaceae", "__Thiobacillus"]}},{"id": "denovo12534", "metadata": {"taxonomy": ["Bacteria", "__Proteobacteria", "__Alphaproteobacteria", "__Rhodobacterales", "__Rhodobacteraceae", "__Tropicimonas"]}},{"id": "denovo12535", "metadata": {"taxonomy": ["Archaea", "__Euryarchaeota", "__Halobacteria", "__Halobacteriales", "__Deep_Sea_Hydrothermal_Vent_Gp_6(DHVEG-6)", "__Candidatus_Parvarchaeum"]}},{"id": "denovo12536", "metadata": {"taxonomy": ["Archaea", "__Euryarchaeota", "__Thermoplasmata", "__Thermoplasmatales", "__Marine_Group_II", "__g"]}},{"id": "denovo12538", "metadata": {"taxonomy": ["Bacteria", "__Bacteroidetes", "__Flavobacteria", "__Flavobacteriales", "__Flavobacteriaceae"]}},{"id": "denovo12539", "metadata": {"taxonomy": ["Bacteria", "__Proteobacteria", "__Gammaproteobacteria", "__Oceanospirillales", "__Oleiphilaceae", "__Oleiphilus"]}},{"id": "denovo12540", "metadata": {"taxonomy": ["Bacteria", "__Acidobacteria", "__Holophagae", "__CA002", "__f", "__g"]}},{"id": "denovo12543", "metadata": {"taxonomy": ["Bacteria", "__Proteobacteria", "__Gammaproteobacteria", "__Alteromonadales", "__Alteromonadaceae", "__Candidatus_Endobugula"]}},{"id": "denovo12545", "metadata": {"taxonomy": ["Bacteria", "__Proteobacteria", "__Deltaproteobacteria", "__Desulfobacterales", "__Desulfobulbaceae", "__Desulforhopalus"]}},{"id": "denovo12546", "metadata": {"taxonomy": ["Bacteria", "__Proteobacteria", "__Alphaproteobacteria"]}},{"id": "denovo12549", "metadata": {"taxonomy": ["Bacteria", "__Proteobacteria", "__Deltaproteobacteria", "__Desulfobacterales", "__Desulfobulbaceae", "__Desulfobulbus"]}}, {"id": "denovo12550", "metadata": {"taxonomy": ["Bacteria", "__Proteobacteria", "__Alphaproteobacteria", "__Kordiimonadales", "__Kordiimonadaceae", "__Kordiimonas"]}},{"id": "denovo12552", "metadata": {"taxonomy": ["Bacteria", "__Proteobacteria", "__Deltaproteobacteria", "__Myxococcales", "__Haliangiaceae", "__Haliangium"]}},{"id": "denovo12573", "metadata": {"taxonomy": ["Bacteria", "__Proteobacteria", "__Gammaproteobacteria"]}},{"id": "denovo12578", "metadata": {"taxonomy": ["Bacteria", "__Proteobacteria", "__Gammaproteobacteria", "__Thiotrichales", "__Piscirickettsiaceae", "__Piscirickettsia"]}},{"id": "denovo12579", "metadata": {"taxonomy": ["Bacteria", "__Firmicutes", "__Bacilli", "__Bacillales", "__Bacillaceae", "__Anoxybacillus"]}},{"id":

"denovo12580", "metadata": {"taxonomy": ["Bacteria", "__Proteobacteria", "__Gammaproteobacteria", "__Oceanospirillales", "__MBAE14", "__g"]}},{"id": "denovo12582", "metadata": {"taxonomy": ["Bacteria", "__Proteobacteria", "__Alphaproteobacteria", "__Rhodobacterales", "__Rhodobacteraceae", "__Ruegeria"]}},{"id": "denovo12583", "metadata": {"taxonomy": ["Bacteria", "__Proteobacteria", "__Deltaproteobacteria", "__Desulfobacterales", "__Desulfobacteraceae"]}},{"id": "denovo12584", "metadata": {"taxonomy": ["Bacteria", "__Proteobacteria", "__Deltaproteobacteria", "__Sh765B-TzT-29", "__f", "__g"]}},{"id": "denovo12585", "metadata": {"taxonomy": ["Bacteria", "__Bacteroidetes", "__Cytophagia", "__Cytophagales", "__Flammeovirgaceae", "__Candidatus_Amoebophilus"]}},{"id": "denovo12590", "metadata": {"taxonomy": ["Bacteria", "__Bacteroidetes", "__Sphingobacteriia", "__Sphingobacteriales", "__Saprospiraceae", "__g"]}},{"id": "denovo12591", "metadata": {"taxonomy": ["Bacteria", "__Cyanobacteria", "__Cyanobacteria", "__SubsectionIII", "__FamilyI"]}},{"id": "denovo12595", "metadata": {"taxonomy": ["Bacteria", "__Proteobacteria", "__Deltaproteobacteria", "__Desulfobacterales", "__Nitrospinaceae", "__g"]}},{"id": "denovo12596", "metadata": {"taxonomy": ["Bacteria", "__Verrucomicrobia", "__Opitutae", "__Puniceicoccales", "__Puniceicoccaceae", "__Cerasicoccus"]}},{"id": "denovo12600", "metadata": {"taxonomy": ["Bacteria", "__Proteobacteria", "__Gammaproteobacteria", "__Alteromonadales", "__Alteromonadaceae"]}},{"id": "denovo12602", "metadata": {"taxonomy": ["Bacteria", "__Proteobacteria", "__Gammaproteobacteria", "__Oceanospirillales", "__Oceanospirillaceae", "__Pseudospirillum"]}},{"id": "denovo12605", "metadata": {"taxonomy": ["Bacteria", "__Proteobacteria", "__Alphaproteobacteria", "__Rhodospirillales", "__Rhodospirillaceae"]}},{"id": "denovo12607", "metadata": {"taxonomy": ["Bacteria", "__Bacteroidetes", "__Flavobacteria", "__Flavobacteriales", "__Cryomorphaceae", "__Owenweeksia"]}},{"id": "denovo12610", "metadata": {"taxonomy": ["Bacteria", "__Proteobacteria", "__Alphaproteobacteria", "__Rhodobacterales", "__Rhodobacteraceae", "__Ruegeria"]}},{"id": "denovo12612", "metadata": {"taxonomy": ["Bacteria", "__Firmicutes", "__Clostridia", "__Clostridiales", "__Clostridiaceae", "__Clostridium"]}},{"id": "denovo12613", "metadata": {"taxonomy": ["Bacteria", "__Bacteroidetes", "__Cytophagia", "__Cytophagales", "__Flammeovirgaceae", "__Ekhidna"]}},{"id": "denovo12615", "metadata": {"taxonomy": ["Bacteria", "__Proteobacteria", "__Alphaproteobacteria", "__Rhizobiales"]}},{"id": "denovo12617", "metadata": {"taxonomy": ["Bacteria", "__Proteobacteria", "__Deltaproteobacteria", "__Desulfovibrionales", "__Desulfovibrionaceae", "__Desulfovibrio"]}},{"id": "denovo12618", "metadata": {"taxonomy": ["Bacteria", "__Proteobacteria", "__Gammaproteobacteria", "__Alteromonadales", "__Alteromonadaceae", "__Dasania"]}},{"id": "denovo12622", "metadata": {"taxonomy": ["Bacteria", "__Cyanobacteria", "__Cyanobacteria", "__SubsectionIII", "__FamilyI", "__Phormidium"]}},{"id":

"denovo12627", "metadata": {"taxonomy": ["Bacteria", "__Planctomycetes", "__Planctomycetacia", "__Planctomycetales", "__Planctomycetaceae", "__Rhodopirellula"]}},{"id": "denovo12629", "metadata": {"taxonomy": ["Bacteria", "__Proteobacteria", "__Gammaproteobacteria"]}},{"id": "denovo12634", "metadata": {"taxonomy": ["Bacteria", "__Verrucomicrobia", "__Verrucomicrobiae", "__Verrucomicrobiales", "__Rubritaleaceae", "__Rubritalea"]}},{"id": "denovo12635", "metadata": {"taxonomy": ["Bacteria", "__Proteobacteria", "__Betaproteobacteria", "__Methylophilales", "__Methylophilaceae", "__Methylophilus"]}},{"id": "denovo12637", "metadata": {"taxonomy": ["Bacteria", "__Proteobacteria", "__Alphaproteobacteria", "__OCS116_clade", "__f", "__g"]}},{"id": "denovo12639", "metadata": {"taxonomy": ["Bacteria", "__Proteobacteria"]}},{"id": "denovo12640", "metadata": {"taxonomy": ["Bacteria", "__Proteobacteria", "__Alphaproteobacteria", "__SAR11_clade", "__Surface_1", "__g"]}},{"id": "denovo12644", "metadata": {"taxonomy": ["Bacteria", "__Bacteroidetes", "__Flavobacteria", "__Flavobacteriales", "__Flavobacteriaceae", "__NS2b_marine_group"]}},{"id": "denovo12655", "metadata": {"taxonomy": ["Bacteria", "__Deferribacteres", "__Deferribacteres", "__Deferribacterales", "__Family_Incertae_Sedis", "__Caldithrix"]}},{"id": "denovo12656", "metadata": {"taxonomy": ["Bacteria", "__Proteobacteria", "__Gammaproteobacteria", "__Alteromonadales", "__Alteromonadaceae"]}},{"id": "denovo12665", "metadata": {"taxonomy": ["Bacteria", "__Proteobacteria", "__Gammaproteobacteria", "__Alteromonadales", "__Shewanellaceae", "__Shewanella"]}},{"id": "denovo12669", "metadata": {"taxonomy": ["Bacteria", "__Proteobacteria", "__Gammaproteobacteria", "__Xanthomonadales", "__Sinobacteraceae", "__JTB255_marine_benthic_group"]}},{"id": "denovo12671", "metadata": {"taxonomy": ["Bacteria", "__Proteobacteria", "__Deltaproteobacteria", "__Sh765B-TzT-29", "__f", "__g"]}},{"id": "denovo12679", "metadata": {"taxonomy": ["Bacteria", "__Proteobacteria", "__Alphaproteobacteria", "__Rhodospirillales", "__Rhodospirillaceae"]}},{"id": "denovo12680", "metadata": {"taxonomy": ["Bacteria", "__Planctomycetes", "__Phycisphaerae", "__Phycisphaerales", "__Phycisphaeraceae", "__Urania-1B-19_marine_sediment_group"]}},{"id": "denovo12684", "metadata": {"taxonomy": ["Bacteria", "__Proteobacteria", "__Alphaproteobacteria", "__Parvularculales", "__Parvularculaceae", "__Parvularcula"]}},{"id": "denovo12692", "metadata": {"taxonomy": ["Bacteria", "__Planctomycetes", "__BD7-11", "__o", "__f", "__g"]}},{"id": "denovo12698", "metadata": {"taxonomy": ["Bacteria", "__Proteobacteria", "__Deltaproteobacteria", "__Desulfobacterales", "__Desulfobacteraceae", "__Sva0081_sediment_group"]}},{"id": "denovo12702", "metadata": {"taxonomy": ["Bacteria", "__Proteobacteria", "__Gammaproteobacteria", "__Alteromonadales", "__Alteromonadaceae"]}},{"id": "denovo12706", "metadata": {"taxonomy": ["Bacteria", "__Cyanobacteria", "__Cyanobacteria", "__SubsectionI", "__FamilyI", "__Acaryochloris"]}},{"id": "denovo12722", "metadata": {"taxonomy": ["Bacteria", "__Bacteroidetes", "__Sphingobacteriia",

"__Sphingobacteriales", "__Saprospiraceae", "__g"]}},{"id": "denovo12725", "metadata": {"taxonomy": ["Bacteria", "__Proteobacteria", "__Alphaproteobacteria", "__Rhodobacterales", "__Rhodobacteraceae"]}},{"id": "denovo12726", "metadata": {"taxonomy": ["Bacteria", "__Planctomycetes", "__Planctomycetacia", "__Planctomycetales", "__Planctomycetaceae", "__Pir4_lineage"]}}, {"id": "denovo12727", "metadata": {"taxonomy": ["Bacteria", "__Proteobacteria", "__Gammaproteobacteria", "__Pseudomonadales", "__Moraxellaceae", "__Acinetobacter"]}},{"id": "denovo12729", "metadata": {"taxonomy": ["Bacteria", "__Proteobacteria", "__Gammaproteobacteria"]}},{"id": "denovo12731", "metadata": {"taxonomy": ["Bacteria", "__Proteobacteria", "__Alphaproteobacteria", "__Rhodobacterales", "__Rhodobacteraceae"]}},{"id": "denovo12733", "metadata": {"taxonomy": ["Bacteria", "__Proteobacteria", "__Gammaproteobacteria", "__Alteromonadales", "__Alteromonadaceae", "__OM60(NOR5)_clade"]}},{"id": "denovo12734", "metadata": {"taxonomy": ["Bacteria", "__Acidobacteria", "__Holophagae", "__Acanthopleuribacterales", "__Acanthopleuribacteraceae", "__Acanthopleuribacter"]}},{"id": "denovo12736", "metadata": {"taxonomy": ["Bacteria", "__Armatimonadetes", "__c", "__o", "__f", "__g"]}},{"id": "denovo12738", "metadata": {"taxonomy": ["Bacteria", "__Bacteroidetes", "__Sphingobacteriia", "__Sphingobacteriales", "__Saprospiraceae", "__g"]}},{"id": "denovo12739", "metadata": {"taxonomy": ["Bacteria", "__Proteobacteria", "__Alphaproteobacteria", "__Rhodobacterales", "__Rhodobacteraceae"]}},{"id": "denovo12740", "metadata": {"taxonomy": ["Bacteria", "__Bacteroidetes", "__Flavobacteria", "__Flavobacteriales", "__Cryomorphaceae", "__Lishizhenia"]}},{"id": "denovo12741", "metadata": {"taxonomy": ["Bacteria", "__Proteobacteria", "__Gammaproteobacteria", "__NKB5", "__f", "__g"]}},{"id": "denovo12742", "metadata": {"taxonomy": ["Bacteria", "__Proteobacteria", "__Deltaproteobacteria", "__GR-WP33-30", "__f", "__g"]}},{"id": "denovo12743", "metadata": {"taxonomy": ["Bacteria", "__Proteobacteria", "__Gammaproteobacteria"]}},{"id": "denovo12746", "metadata": {"taxonomy": ["Bacteria", "__Proteobacteria", "__Gammaproteobacteria", "__KI89A_clade", "__f", "__g"]}},{"id": "denovo12751", "metadata": {"taxonomy": ["Bacteria", "__Proteobacteria", "__Gammaproteobacteria"]}},{"id": "denovo12757", "metadata": {"taxonomy": ["Bacteria", "__Proteobacteria", "__Gammaproteobacteria", "__Chromatiales", "__Granulosicoccaceae", "__Granulosicoccus"]}}, {"id": "denovo12758", "metadata": {"taxonomy": ["Bacteria", "__Proteobacteria", "__Gammaproteobacteria", "__NKB5", "__f", "__g"]}},{"id": "denovo12763", "metadata": {"taxonomy": ["Bacteria", "__Proteobacteria", "__Deltaproteobacteria", "__SAR324_clade(Marine_group_B)", "__f", "__g"]}},{"id": "denovo12767", "metadata": {"taxonomy": ["Bacteria", "__Actinobacteria", "__Acidimicrobiia", "__Acidimicrobiales", "__OCS155_marine_group", "__g"]}},{"id": "denovo12768", "metadata": {"taxonomy": ["Bacteria", "__Bacteroidetes", "__Cytophagia", "__Cytophagales", "__Flammeovirgaceae", "__Flexithrix"]}},{"id":

"denovo12770", "metadata": {"taxonomy": ["Bacteria", "__Proteobacteria", "__Gammaproteobacteria", "__Alteromonadales"]}}, {"id": "denovo12776", "metadata": {"taxonomy": ["Bacteria", "__Proteobacteria", "__Gammaproteobacteria", "__Pseudomonadales", "__Moraxellaceae", "__Acinetobacter"]}},{"id": "denovo12782", "metadata": {"taxonomy": ["Bacteria", "__Proteobacteria", "__Gammaproteobacteria", "__Thiotrichales", "__Thiotrichaceae", "__Thiothrix"]}},{"id": "denovo12787", "metadata": {"taxonomy": ["Bacteria", "__Proteobacteria", "__Deltaproteobacteria", "__Desulfuromonadales", "__Desulfuromonadaceae", "__Desulfuromusa"]}}, {"id": "denovo12789", "metadata": {"taxonomy": ["Bacteria", "__Proteobacteria", "__Alphaproteobacteria", "__Rhizobiales", "__Rhodobiaceae", "__Afifella"]}},{"id": "denovo12790", "metadata": {"taxonomy": ["Bacteria", "__Proteobacteria", "__Alphaproteobacteria", "__Rhizobiales"]}},{"id": "denovo12791", "metadata": {"taxonomy": ["Bacteria", "__Proteobacteria", "__Alphaproteobacteria", "__Sphingomonadales", "__Sphingomonadaceae", "__Sphingomonas"]}}, {"id": "denovo12792", "metadata": {"taxonomy": ["Bacteria", "__Bacteroidetes", "__Cytophagia", "__Cytophagales", "__Flammeovirgaceae"]}},{"id": "denovo12793", "metadata": {"taxonomy": ["Bacteria", "__Proteobacteria", "__Alphaproteobacteria", "__Rhodospirillales", "__Rhodospirillaceae", "__g"]}},{"id": "denovo12794", "metadata": {"taxonomy": ["Bacteria", "__Proteobacteria", "__Deltaproteobacteria", "__Desulfobacterales", "__Desulfobacteraceae", "__g"]}},{"id": "denovo12797", "metadata": {"taxonomy": ["Bacteria", "__Cyanobacteria", "__Cyanobacteria"]}}, {"id": "denovo12803", "metadata": {"taxonomy": ["Bacteria", "__Proteobacteria", "__Alphaproteobacteria", "__OCS116_clade", "__f", "__g"]}},{"id": "denovo12806", "metadata": {"taxonomy": ["Bacteria", "__Actinobacteria", "__Actinobacteria", "__Micrococcales", "__Microbacteriaceae"]}},{"id": "denovo12809", "metadata": {"taxonomy": ["Bacteria", "__Proteobacteria", "__Deltaproteobacteria", "__Myxococcales", "__Nannocystaceae", "__Enhygromyxa"]}},{"id": "denovo12810", "metadata": {"taxonomy": ["Bacteria", "__Candidate_division_BRC1", "__c", "__o", "__f", "__g"]}},{"id": "denovo12811", "metadata": {"taxonomy": ["Bacteria", "__Proteobacteria", "__Gammaproteobacteria", "__Alteromonadales", "__Alteromonadaceae", "__OM60(NOR5)_clade"]}},{"id": "denovo12813", "metadata": {"taxonomy": ["Bacteria", "__Bacteroidetes", "__Flavobacteria", "__Flavobacteriales", "__Cryomorphaceae"]}},{"id": "denovo12816", "metadata": {"taxonomy": ["Bacteria"]}},{"id": "denovo12817", "metadata": {"taxonomy": ["Bacteria", "__Proteobacteria", "__Gammaproteobacteria", "__B38", "__f", "__g"]}}, {"id": "denovo12818", "metadata": {"taxonomy": ["Bacteria", "__Proteobacteria", "__Gammaproteobacteria", "__Alteromonadales", "__Alteromonadaceae", "__Umboniibacter"]}},{"id": "denovo12819", "metadata": {"taxonomy": ["Bacteria", "__Proteobacteria", "__Alphaproteobacteria", "__Rhodospirillales"]}},{"id": "denovo12820", "metadata": {"taxonomy": ["Bacteria", "__Proteobacteria", "__Gammaproteobacteria"]}},{"id": "denovo12826", "metadata":

{"taxonomy": ["Bacteria", "__Bacteroidetes", "__Flavobacteria", "__Flavobacteriales", "__Flavobacteriaceae", "__Leptobacterium"]}}, {"id": "denovo12829", "metadata": {"taxonomy": ["Bacteria", "__Proteobacteria", "__Deltaproteobacteria", "__Myxococcales", "__Haliangiaceae", "__Haliangium"]}},{"id": "denovo12836", "metadata": {"taxonomy": ["Bacteria", "__Chloroflexi", "__Anaerolineae", "__Anaerolineales", "__Anaerolineaceae", "__g"]}},{"id": "denovo12837", "metadata": {"taxonomy": ["Bacteria", "__Proteobacteria", "__Deltaproteobacteria", "__Myxococcales", "__Cystobacterineae", "__g"]}},{"id": "denovo12840", "metadata": {"taxonomy": ["Bacteria", "__Proteobacteria", "__Alphaproteobacteria", "__Rhodobacterales", "__Rhodobacteraceae", "__Pseudovibrio"]}},{"id": "denovo12844", "metadata": {"taxonomy": ["Bacteria", "__Firmicutes", "__Clostridia", "__Clostridiales", "__Family_XII_Incertae_Sedis", "__Fusibacter"]}},{"id": "denovo12846", "metadata": {"taxonomy": ["Bacteria", "__Proteobacteria", "__Alphaproteobacteria", "__Rhodospirillales", "__Rhodospirillaceae", "__g"]}},{"id": "denovo12852", "metadata": {"taxonomy": ["Bacteria", "__Spirochaetes", "__Spirochaetes", "__Kazan-3B-09", "__f", "__g"]}},{"id": "denovo12853", "metadata": {"taxonomy": ["Bacteria", "__Cyanobacteria", "__Cyanobacteria", "__SubsectionI", "__FamilyI", "__Synechococcus"]}},{"id": "denovo12855", "metadata": {"taxonomy": ["Bacteria", "__Proteobacteria", "__Alphaproteobacteria", "__Rhodobacterales", "__Rhodobacteraceae"]}},{"id": "denovo12856", "metadata": {"taxonomy": ["Bacteria", "__Proteobacteria", "__Alphaproteobacteria", "__Rhodobacterales", "__Rhodobacteraceae", "__Ruegeria"]}},{"id": "denovo12858", "metadata": {"taxonomy": ["Bacteria", "__Proteobacteria", "__Gammaproteobacteria", "__Order_Incertae_Sedis", "__Family_Incertae_Sedis", "__Marinicella"]}},{"id": "denovo12859", "metadata": {"taxonomy": ["Bacteria", "__Proteobacteria", "__Gammaproteobacteria", "__Alteromonadales", "__Alteromonadaceae", "__Dasania"]}},{"id": "denovo12860", "metadata": {"taxonomy": ["Bacteria", "__Proteobacteria", "__Alphaproteobacteria", "__DB1-14", "__f", "__g"]}},{"id": "denovo12863", "metadata": {"taxonomy": ["Archaea", "__Thaumarchaeota", "__Marine_Group_I", "__Candidatus_Nitrosopumilus", "__f", "__g"]}},{"id": "denovo12865", "metadata": {"taxonomy": ["Bacteria", "__Proteobacteria", "__Alphaproteobacteria", "__Rhizobiales", "__Phyllobacteriaceae", "__g"]}},{"id": "denovo12872", "metadata": {"taxonomy": ["Bacteria", "__Bacteroidetes", "__Cytophagia", "__Order_II_Incertae_Sedis", "__Rhodothermaceae", "__g"]}},{"id": "denovo12873", "metadata": {"taxonomy": ["Bacteria", "__Bacteroidetes", "__Sphingobacteriia", "__Sphingobacteriales", "__Chitinophagaceae", "__Hydrotalea"]}},{"id": "denovo12874", "metadata": {"taxonomy": ["Bacteria", "__Proteobacteria", "__Gammaproteobacteria", "__Aeromonadales", "__Aeromonadaceae", "__Aeromonas"]}},{"id": "denovo12875", "metadata": {"taxonomy": ["Bacteria", "__Proteobacteria", "__Deltaproteobacteria", "__Myxococcales", "__0319-6G20", "__g"]}},{"id": "denovo12877", "metadata": {"taxonomy": ["Bacteria", "__Proteobacteria",

"__Gammaproteobacteria", "__Order_Incertae_Sedis",
"__Family_Incertae_Sedis", "__Sedimenticola"]}},{"id": "denovo12878",
"metadata": {"taxonomy": ["Bacteria", "__Proteobacteria",
"__Deltaproteobacteria", "__Bdellovibrionales",
"__Bdellovibrionaceae", "__OM27_clade"]}},{"id": "denovo12879",
"metadata": {"taxonomy": ["Bacteria", "__Proteobacteria",
"__Gammaproteobacteria", "__Oceanospirillales", "__SS1-B-06-26",
"__g"]}},{"id": "denovo12885", "metadata": {"taxonomy": ["Bacteria",
"__Proteobacteria", "__Gammaproteobacteria", "__Alteromonadales"]}},
{"id": "denovo12887", "metadata": {"taxonomy": ["Bacteria",
"__Cyanobacteria", "__Cyanobacteria", "__SubsectionI", "__FamilyI",
"__Synechococcus"]}},{"id": "denovo12888", "metadata": {"taxonomy":
["Bacteria", "__Proteobacteria", "__Gammaproteobacteria",
"__Oceanospirillales"]}},{"id": "denovo12889", "metadata":
{"taxonomy": ["Bacteria", "__Proteobacteria", "__Betaproteobacteria",
"__Burkholderiales", "__Comamonadaceae", "__Delftia"]}},{"id":
"denovo12895", "metadata": {"taxonomy": ["Bacteria",
"__Planctomycetes", "__Phycisphaerae", "__Phycisphaerales",
"__Phycisphaeraceae", "__Phycisphaera"]}},{"id": "denovo12901",
"metadata": {"taxonomy": ["Bacteria", "__Proteobacteria",
"__Alphaproteobacteria", "__Rhizobiales", "__Phyllobacteriaceae"]}},
{"id": "denovo12910", "metadata": {"taxonomy": ["Bacteria",
"__Proteobacteria", "__Deltaproteobacteria", "__Desulfovibrionales",
"__Desulfovibrionaceae", "__Desulfovibrio"]}},{"id": "denovo12922",
"metadata": {"taxonomy": ["Bacteria", "__Bacteroidetes",
"__Cytophagia", "__Cytophagales", "__Flammeovirgaceae",
"__Persicobacter"]}},{"id": "denovo12926", "metadata": {"taxonomy":
["Bacteria", "__Proteobacteria", "__Gammaproteobacteria",
"__Oceanospirillales", "__SAR86_clade", "__g"]}},{"id": "denovo12930",
"metadata": {"taxonomy": ["Bacteria", "__Proteobacteria",
"__Gammaproteobacteria", "__Xanthomonadales", "__Sinobacteraceae",
"__JTB255_marine_benthic_group"]}},{"id": "denovo12931", "metadata":
{"taxonomy": ["Bacteria", "__Bacteroidetes", "__Cytophagia",
"__Cytophagales", "__Flammeovirgaceae", "__g"]}},{"id": "denovo12932",
"metadata": {"taxonomy": ["Bacteria", "__Proteobacteria",
"__Gammaproteobacteria"]}},{"id": "denovo12934", "metadata":
{"taxonomy": ["Bacteria", "__Actinobacteria", "__Acidimicrobiia",
"__Acidimicrobiales", "__Sva0996_marine_group", "__g"]}},{"id":
"denovo12938", "metadata": {"taxonomy": ["Bacteria",
"__Bacteroidetes", "__Sphingobacteriia", "__Sphingobacteriales",
"__Chitinophagaceae", "__g"]}},{"id": "denovo12939", "metadata":
{"taxonomy": ["Bacteria", "__Proteobacteria", "__TA18", "__o", "__f",
"__g"]}},{"id": "denovo12942", "metadata": {"taxonomy": ["Bacteria",
"__Firmicutes", "__Clostridia", "__Clostridiales",
"__Clostridiaceae"]}},{"id": "denovo12951", "metadata": {"taxonomy":
["Bacteria", "__Planctomycetes", "__Phycisphaerae",
"__Phycisphaerales", "__Phycisphaeraceae", "__Phycisphaera"]}},{"id":
"denovo12952", "metadata": {"taxonomy": ["Bacteria",
"__Bacteroidetes", "__Flavobacteria", "__Flavobacteriales",
"__Flavobacteriaceae", "__Tenacibaculum"]}},{"id": "denovo12957",

"metadata": {"taxonomy": ["Bacteria", "__Proteobacteria", "__Gammaproteobacteria", "__Alteromonadales", "__Psychromonadaceae", "__Psychromonas"]}},{"id": "denovo12959", "metadata": {"taxonomy": ["Bacteria", "__Planctomycetes", "__OM190", "__o", "__f", "__g"]}}, {"id": "denovo12960", "metadata": {"taxonomy": ["Bacteria", "__Proteobacteria", "__Gammaproteobacteria", "__Pseudomonadales", "__Moraxellaceae", "__Acinetobacter"]}},{"id": "denovo12964", "metadata": {"taxonomy": ["Bacteria", "__Proteobacteria", "__Alphaproteobacteria", "__Rhodobacterales", "__Rhodobacteraceae"]}}, {"id": "denovo12966", "metadata": {"taxonomy": ["Bacteria", "__Proteobacteria", "__Deltaproteobacteria", "__Sh765B-TzT-29", "__f", "__g"]}},{"id": "denovo12970", "metadata": {"taxonomy": ["Bacteria", "__Proteobacteria", "__Deltaproteobacteria", "__Desulfobacterales", "__Nitrospinaceae", "__Nitrospina"]}},{"id": "denovo12978", "metadata": {"taxonomy": ["Bacteria", "__Bacteroidetes", "__Flavobacteria", "__Flavobacteriales", "__Cryomorphaceae", "__Fluviicola"]}},{"id": "denovo12980", "metadata": {"taxonomy": ["Bacteria", "__Proteobacteria", "__Deltaproteobacteria", "__Myxococcales", "__0319-6G20", "__g"]}},{"id": "denovo12981", "metadata": {"taxonomy": ["Bacteria", "__Proteobacteria", "__Alphaproteobacteria", "__Caulobacterales", "__Hyphomonadaceae", "__Maricaulis"]}},{"id": "denovo12983", "metadata": {"taxonomy": ["Bacteria", "__Proteobacteria"]}},{"id": "denovo12986", "metadata": {"taxonomy": ["Bacteria", "__Proteobacteria", "__Gammaproteobacteria", "__Alteromonadales", "__Alteromonadaceae", "__BD2-7"]}},{"id": "denovo12988", "metadata": {"taxonomy": ["Bacteria", "__Proteobacteria", "__Alphaproteobacteria", "__Rhodospirillales", "__Rhodospirillaceae", "__Tistlia"]}},{"id": "denovo12989", "metadata": {"taxonomy": ["Bacteria", "__Bacteroidetes", "__Flavobacteria", "__Flavobacteriales", "__Flavobacteriaceae", "__NS5_marine_group"]}},{"id": "denovo12991", "metadata": {"taxonomy": ["Bacteria", "__Proteobacteria", "__Gammaproteobacteria", "__Alteromonadales", "__Pseudoalteromonadaceae", "__Algicola"]}}, {"id": "denovo12994", "metadata": {"taxonomy": ["Bacteria", "__Proteobacteria", "__Gammaproteobacteria"]}},{"id": "denovo12995", "metadata": {"taxonomy": ["Bacteria", "__Proteobacteria", "__Gammaproteobacteria", "__KI89A_clade", "__f", "__g"]}},{"id": "denovo12998", "metadata": {"taxonomy": ["Archaea", "__Thaumarchaeota", "__Marine_Group_I", "__Candidatus_Nitrosopumilus", "__f", "__g"]}},{"id": "denovo13001", "metadata": {"taxonomy": ["Bacteria", "__Proteobacteria", "__Alphaproteobacteria", "__Rhodospirillales", "__Rhodospirillaceae"]}},{"id": "denovo13002", "metadata": {"taxonomy": ["Bacteria", "__Proteobacteria", "__Alphaproteobacteria", "__Rickettsiales", "__SAR116_clade", "__g"]}},{"id": "denovo13003", "metadata": {"taxonomy": ["Bacteria", "__Proteobacteria", "__Alphaproteobacteria", "__Rhodobacterales", "__Rhodobacteraceae"]}},{"id": "denovo13004", "metadata": {"taxonomy": ["Bacteria", "__Armatimonadetes", "__c", "__o", "__f", "__g"]}},{"id": "denovo13005", "metadata": {"taxonomy": ["Bacteria", "__Proteobacteria", "__Alphaproteobacteria", "__Rhodobacterales",

"__Rhodobacteraceae"]}},{"id": "denovo13008", "metadata": {"taxonomy": ["Bacteria", "__Firmicutes", "__Clostridia", "__Clostridiales", "__Veillonellaceae", "__Megasphaera"]}},{"id": "denovo13011", "metadata": {"taxonomy": ["Archaea", "__Thaumarchaeota", "__Marine_Group_I", "__Candidatus_Nitrosopumilus", "__f", "__g"]}}, {"id": "denovo13015", "metadata": {"taxonomy": ["Bacteria", "__Proteobacteria", "__Gammaproteobacteria", "__Alteromonadales", "__Alteromonadaceae", "__OM60(NOR5)_clade"]}},{"id": "denovo13016", "metadata": {"taxonomy": ["Bacteria", "__Proteobacteria", "__Gammaproteobacteria", "__Chromatiales"]}},{"id": "denovo13023", "metadata": {"taxonomy": ["Bacteria", "__Proteobacteria", "__Alphaproteobacteria", "__Rhizobiales", "__Phyllobacteriaceae", "__Hoeflea"]}},{"id": "denovo13029", "metadata": {"taxonomy": ["Bacteria", "__Actinobacteria", "__Acidimicrobiia", "__Acidimicrobiales", "__OCS155_marine_group", "__g"]}},{"id": "denovo13033", "metadata": {"taxonomy": ["Bacteria", "__Proteobacteria", "__Alphaproteobacteria", "__Rhodobacterales", "__Rhodobacteraceae"]}},{"id": "denovo13043", "metadata": {"taxonomy": ["Bacteria", "__Proteobacteria", "__Gammaproteobacteria", "__Alteromonadales", "__Psychromonadaceae", "__Psychromonas"]}},{"id": "denovo13044", "metadata": {"taxonomy": ["Bacteria", "__Proteobacteria", "__Deltaproteobacteria", "__SAR324_clade(Marine_group_B)", "__f", "__g"]}},{"id": "denovo13046", "metadata": {"taxonomy": ["Bacteria", "__Cyanobacteria", "__Cyanobacteria", "__SubsectionIII", "__FamilyI", "__Phormidium"]}},{"id": "denovo13047", "metadata": {"taxonomy": ["Bacteria", "__Proteobacteria", "__Alphaproteobacteria", "__Rhodobacterales", "__Rhodobacteraceae"]}},{"id": "denovo13049", "metadata": {"taxonomy": ["Bacteria", "__Proteobacteria", "__Gammaproteobacteria", "__Xanthomonadales", "__Sinobacteraceae", "__JTB255_marine_benthic_group"]}},{"id": "denovo13052", "metadata": {"taxonomy": ["Bacteria", "__Proteobacteria", "__Gammaproteobacteria"]}},{"id": "denovo13053", "metadata": {"taxonomy": ["Bacteria", "__Proteobacteria", "__Alphaproteobacteria", "__Rhodospirillales"]}},{"id": "denovo13054", "metadata": {"taxonomy": ["Bacteria", "__Firmicutes", "__Clostridia", "__Clostridiales"]}}, {"id": "denovo13057", "metadata": {"taxonomy": ["Bacteria", "__Firmicutes", "__Clostridia", "__Clostridiales"]}},{"id": "denovo13058", "metadata": {"taxonomy": ["Bacteria", "__Candidate_division_BRC1", "__c", "__o", "__f", "__g"]}},{"id": "denovo13061", "metadata": {"taxonomy": ["Bacteria", "__Proteobacteria", "__Alphaproteobacteria", "__Rhodobacterales", "__Rhodobacteraceae", "__Rhodothalassium"]}},{"id": "denovo13063", "metadata": {"taxonomy": ["Bacteria", "__Proteobacteria", "__Gammaproteobacteria", "__Pseudomonadales", "__Moraxellaceae", "__Acinetobacter"]}},{"id": "denovo13067", "metadata": {"taxonomy": ["Bacteria", "__Bacteroidetes", "__Cytophagia", "__Cytophagales", "__Flammeovirgaceae", "__g"]}},{"id": "denovo13070", "metadata": {"taxonomy": ["Bacteria", "__Proteobacteria", "__Alphaproteobacteria", "__Rhizobiales", "__Hyphomicrobiaceae", "__Filomicrobium"]}},{"id":
[truncated: 421,509 more chars]
