# Supplementary material for: Correlations Between Prokaryotic Microbes and Stress-Resistant Algae in Different Corals Subjected to Environmental Stress in Hong Kong
Source: Front Microbiol. 2020 Apr 23;11:686. doi: 10.3389/fmicb.2020.00686 (PMC7191007; doi:10.3389/fmicb.2020.00686)
Supplement: Supplementary file 22 [file Image_8.PDF]

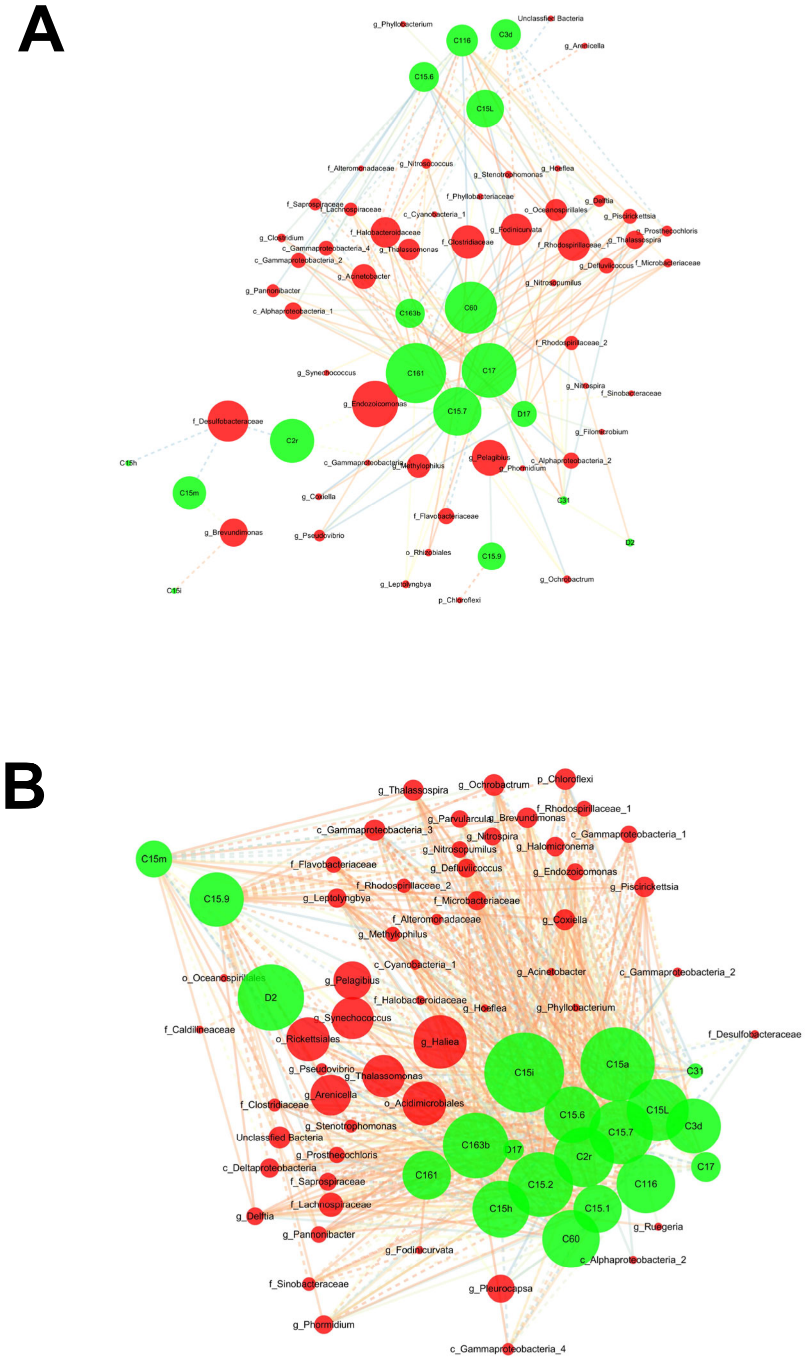
A
g_Phyllobacterium
Unclassified Bacteria
C116
C3d
g_Arenicella
C15.6
C15L
f_Alteromonadaceae
g_Nitrosococcus
g_Stenotrophomonas
g_Hoeflea
f_Phyllobacteriaceae
f_Saprospiraceae
f_Lachnospiraceae
c_Cyanobacteria_1
o_Oceanospirillales
g_Delftia
g_Piscirickettsia
g_Prosthecochloris
f_Halobacteroidaceae
g_Fodinicurvata
g_Thalassospira
g_Clostridium
c_Gammaproteobacteria_4
g_Thalassomonas
f_Clostridiaceae
f_Rhodospirillaceae_1
c_Gammaproteobacteria_2
g_Defluviicoccus
f_Microbacteriaceae
g_Acinetobacter
g_Nitrosopumilus
g_Pannonibacter
c_Alphaproteobacteria_1
C163b
C60
f_Rhodospirillaceae_2
g_Synechococcus
C161
C17
g_Nitrospira
f_Sinobacteraceae
g_Endozoicomonas
C15.7
D17
f_Desulfobacteraceae
C2r
g_Filomicrobium
C15h
c_Gammaproteobacteria
g_Methylophilus
g_Pelagibius
c_Alphaproteobacteria_2
g_Phormidium
C15m
g_Coxiella
C31
f_Flavobacteriaceae
g_Brevundimonas
g_Pseudovibrio
D2
o_Rhizobiales
C15.9
C15i
g_Leptolyngbya
g_Ochrobactrum
p_Chloroflexi
B
g_Thalassospira
g_Ochrobactrum
p_Chloroflexi
f_Rhodospirillaceae_1
g_Parvularcula
g_Brevundimonas
c_Gammaproteobacteria_3
g_Nitrospira
c_Gammaproteobacteria_1
g_Nitrosopumilus
g_Halomicronema
C15m
f_Flavobacteriaceae
g_Defluviicoccus
g_Endozoicomonas
f_Rhodospirillaceae_2
g_Piscirickettsia
C15.9
g_Leptolyngbya
f_Microbacteriaceae
f_Alteromonadaceae
g_Coxiella
g_Methylophilus
c_Cyanobacteria_1
o_Oceanospirillales
g_Pelagibius
g_Acinetobacter
c_Gammaproteobacteria_2
D2
f_Halobacteroidaceae
g_Hoeflea
g_Phyllobacterium
g_Synechococcus
f_Desulfobacteraceae
f_Caldilineaceae
o_Rickettsiales
g_Haliea
C15a
g_Pseudovibrio
C15i
C31
g_Thalassomonas
g_Arenicella
o_Acidimicrobiales
C15L
f_Clostridiaceae
C15.6
C3d
g_Stenotrophomonas
C15.7
Unclassfied Bacteria
C163b
D17
g_Prosthecochloris
C2r
C17
c_Deltaproteobacteria
C161
f_Saprospiraceae
C15.2
C116
f_Lachnospiraceae
C15h
C15.1
g_Delftia
g_Ruegeria
g_Pannonibacter
C60
c_Alphaproteobacteria_2
g_Fodinicurvata
f_Sinobacteraceae
g_Pleurocapsa
g_Phormidium
c_Gammaproteobacteria_4
